# Altered transcriptional regulatory proteins in glioblastoma and YBX1 as a potential regulator of tumor invasion

**DOI:** 10.1038/s41598-019-47360-9

**Published:** 2019-07-29

**Authors:** Manoj Kumar Gupta, Ravindra Varma Polisetty, Rakesh Sharma, Raksha A. Ganesh, Harsha Gowda, Aniruddh K. Purohit, Praveen Ankathi, Komal Prasad, Kiran Mariswamappa, Akhila Lakshmikantha, Megha S. Uppin, Challa Sundaram, Poonam Gautam, Ravi Sirdeshmukh

**Affiliations:** 1 Institute of Bioinformatics, International Tech Park, Bangalore, 560066 India; 2 Manipal Academy of Higher Education, Madhav Nagar, Manipal, 576104 India; 3 Department of Biochemistry, Sri Venkateswara College, University of Delhi, New Delhi, 110021 India; 4 Neuro-Oncology, Mazumdar Shaw Center for Translational Research, Bangalore, 560099 India; 5 Nizam’s Institute of Medical Sciences, Hyderabad, 500082 India; 6 Mazumdar Shaw Medical Center, Narayana Health, Bangalore, 560099 India; 7 ICMR-National Institute of Pathology, Safdarjung Hospital Campus, New Delhi, 110029 India

**Keywords:** Proteomics, CNS cancer

## Abstract

We have studied differentially regulated nuclear proteome of the clinical tissue specimens of glioblastoma (GBM, WHO Grade IV) and lower grades of gliomas (Grade II and III) using high resolution mass spectrometry- based quantitative proteomics approach. The results showed altered expression of many regulatory proteins from the nucleus such as DNA binding proteins, transcription and post transcriptional processing factors and also included enrichment of nuclear proteins that are targets of granzyme signaling – an immune surveillance pathway. Protein - protein interaction network analysis using integrated proteomics and transcriptomics data of transcription factors and proteins for cell invasion process (drawn from another GBM dataset) revealed YBX1, a ubiquitous RNA and DNA-binding protein and a transcription factor, as a key interactor of major cell invasion-associated proteins from GBM. To verify the regulatory link between them, the co-expression of YBX1 and six of the interacting proteins (EGFR, MAPK1, CD44, SOX2, TNC and MMP13) involved in cell invasion network was examined by immunohistochemistry on tissue micro arrays. Our analysis suggests YBX1 as a potential regulator of these key molecules involved in tumor invasion and thus as a promising target for development of new therapeutic strategies for GBM.

## Introduction

Glioblastoma multiforme (GBM) belongs to a group of tumors, namely, gliomas which are predominant primary brain tumors^1^. Gliomas are clinically and histologically classified into four different grades (I-IV) as per WHO guidelines. Grade I (pilocytic astrocytoma) and II (diffused astrocytoma) are relatively slow growing and are considered as low-grade gliomas. Grade III (anaplastic astrocytoma) and Grade IV (Glioblastoma multiforme, GBM) are associated with poor survival and are considered as high-grade gliomas. GBM is the most aggressive form of all and is characterized by extreme proliferation, invasiveness, vascularization and necrosis^1^. GBM exhibits further heterogeneity with additional molecular subclasses - Neural, Pro-neural, Classical and Mesenchymal^2^ suggesting complexity of the disease. As per WHO guidelines (WHO 2016), they are now evaluated on the basis of both histological and molecular features for the purpose of clinical diagnosis and outcome prediction^3^.

The nucleus of the cells is a prime area of focus in the determination of malignancy. The signaling pathways involved in malignant transformation and progression are regulated by nuclear proteins and their post-translational modifications (PTMs). Earlier work on altered nuclear proteins from other tumors, revealed the role of oxidative stress response pathway, proteins involved in chromosome integrity, RNA processing or mRNA metabolism^4,5^ making them a promising source as therapeutic targets or biomarkers for clinical applications^6^. Sub-cellular fractionation of tissue for proteomic profiling is a useful strategy to reduce complexity of the sample to access important regulatory proteins such as the nuclear proteins that may be in low abundance. We have therefore applied this strategy and earlier studied altered levels of proteins from microsomal fraction^7–9^ and the present study represents differentially expressed proteins from the nuclear fraction of the clinical specimens of glioblastoma (WHO-Grade IV) and lower grades of astrocytomas (WHO-Grade II and III) along with temporal lobe epilepsy tissues as controls using iTRAQ-based LC-MS/MS approach and high-resolution mass spectrometry.

## Materials and Methods

### Sample collection

Tumor tissue specimens and controls (temporal lobe epilepsy surgery cases) were collected from Nizam’s Institute of Medical Sciences (NIMS), Hyderabad, India, at the time of surgery and snap frozen in liquid nitrogen for proteomic analysis. All the samples were collected with informed consent from the patients and approval from “Ethics Committee, Nizam’s Institute of Medical Sciences, Hyderabad”. All the experiments were performed as per the recommended guidelines and regulations. One part of the tumor or control tissue was used for histopathology studies. The histopathological evaluation of the surgical biopsies received in the pathology lab of the hospital was performed in terms of classical histo-morphological features and grading of the diffuse gliomas according to WHO classification of central nervous system (CNS) tumors 2007. Immunohistochemistry (IHC) for glial fibrillary acidic protein, vimentin, S100-B and Ki-67 was done with all the biopsies. We did not apply any other molecular criteria such as IDH1 mutation profile or GBM sub-typing (as per WHO 2016 guideline^3^) for classifying these tumors. The glioblastoma specimens were found to be largely tumor tissue including necrosis whereas the tumor content was approximately 80% in grade III and 60–70% in Grade II tumors. This difference largely refers to distinction of actual tumor boundary from the surrounding normal brain tissues while the tumor is being resected. Glioblastoma tumor is soft and can be removed maximally. This distinction is difficult in Grade II and grade III tumors owing to single cell infiltration in the adjacent brain, affecting the tumor content in the specimen received. Recently several computational methods have emerged to predict/infer tumor purity based on copy number variations, gene expression data or methylation data^10–12^. In the absence of genomic or transcriptomic data for our samples we could not directly apply these methods for determination of the tumor purity. However, we examined the stromal or immune cells specific gene signature profiles, derived from TCGA data^11,12^ to our proteomics dataset and infer insignificant contribution of the stromal/immune cell component in the protein analysis results **(**Supplementary Table S1**)**.

Out of over 100 surgical biopsies collected, 45 were astrocytomas, 22 of them grouped as glioblastomas, 13 as anaplastic astrocytoma and 10 as diffuse astrocytoma. The frozen tissue was stored at −80 °C until used for proteomic analysis. We selected six age-matched samples of either sex from each group of Grade II (20–50 years), Grade III (30–50 years) and Glioblastoma (WHO Grade IV; 50–60 years) tumors, for the present study. Gliomas do not generally have defined surgical margins. For this reason, brain tissue obtained from epilepsy surgeries is generally used as the controls for the experiments. Three samples used as controls in this study were from temporal lobe epilepsy surgeries from individuals (20–30 years). Multiple sections from the temporal neocortex were studied, both morphologically and immunohistochemically. The temporal cortex that was used as control did not show any abnormalities by light microscopy. Further, IHC with antibodies directed against phosphorylated neurofilament and synaptophysin proteins did not reveal any abnormality in the cortex^7^.

IHC-based validations were performed using commercial tissue microarrays (US BioMax; or tissue microarrays prepared in-house, as specified under the respective experiments shown in the results section. For in-house tissue microarrays (TMAs) preparation, formalin-fixed paraffin-embedded (FFPE) tissue samples from GBM cases and controls (epilepsy cases) were drawn from the archives of the Mazumdar Shaw Medical Center (MSMC), Bangalore, after obtaining clearance from “Narayana Health Medical Ethics committee”, governing MSMC, Bangalore. Details of the TMA preparation and samples used are described under Immunohistochemistry section.

### Isolation of nuclear fraction and extraction of nuclear proteins

For enrichment of nuclear proteins, the same tissue biopsies as used in our previous studies^7–9^ from the three grades of glioma patients (WHO grade II, II and IV; separate pools of 6 biopsies of each grade) or control subjects (n = 3) were used for subcellular fractionation as described by Cox *et al*. group^13^. This protocol uses differential centrifugation in density gradients to isolate nuclear, cytosolic, mitochondrial and mixed microsomal fractions. Briefly, pooled tissue samples (1.0 g) were homogenized with a Dounce homogenizer (PISCO, Kolkata, India) in 5 ml of 250 STMDPS buffer [250 mM sucrose, 50 mM Tris-HCl (pH 7.4), 5 mM MgCl_2_, 1 mM DTT, 25 μg/ml spermine, and 1 mM PMSF (Sigma Aldrich, St Louis, MO, USA)]. The homogenate was centrifuged at 800 × g for 15 min at 4 °C. The pellet was then washed with 5 ml of 250 STMDPS buffer and centrifuged at 800 × g for 15 min at 4 °C. The pellet was resuspended in 1 ml of 250 STMDPS buffer. The nuclei were visualized by placing 10 µl of homogenate and 2 μl of 4,6-diamidino-2-phenylindole (DAPI; 10 mg/ml in H_2_O stock solution), on a microscope slide using fluorescent microscopy. The re-suspension was further diluted with 250 STMDPS buffer and filtered through several layers of cheese cloth. The filtrate was centrifuged at 800 × g for 15 min at 4 °C and pellet was resuspended in 2 M STMDPS buffer and layered onto the cushion (4 ml of 2 M STMDPS buffer in a ultracentrifuge tube) followed by ultracentrifugation at 80,000 × g for 35 minute at 4 °C. Supernatant was then removed carefully without disturbing the pellet, which was used as the nuclei fraction. The nuclei pellet was resuspended in 250 STMDPS buffer and visualized under fluorescent microscopy.

The nuclei pellet was resuspended in 200 μl of NE buffer [20 mM HEPES (pH 7.9), 1.5 mM MgCl_2_, 0.5 M NaCl, 0.2 mM EDTA and 20% glycerol] and incubated for 30 min with gentle rocking at 4 °C. The nuclei were mechanically disrupted with 10 passages through 18 guage needle followed by centrifugation at 9,000 × g for 30 min at 4 °C and the supernatant (S1) collected. The pellet was now re-suspended in 200 µl of NET buffer [1% Triton-X-100, 1 mM DTT, 1 mM PMSF in NE Buffer] and incubated for 30 min with gentle rocking at 4 °C, and centrifuged at 9,000 × g for 30 min at 4 °C and the supernatant (S2) was collected separately. The supernatant ‘S1’ contains nuclear proteins that are soluble and not tightly bound to DNA, while Supernatant ‘S2’ contains proteins that are tightly bound to DNA, mostly histones. Therefore the study was performed with supernatant ‘S1’ proteins which may contain only some residual histones which otherwise would interfere with the analysis. Protein concentration was estimated using Bradford method and the preparation stored at −80 °C until proteomic analysis to identify differentially expressed nuclear proteins.

### Proteomic analysis

#### Trypsin digestion and iTRAQ labeling

Nuclear proteins from the pooled control or tumor samples (WHO grade II, II or IV) were digested with trypsin and the peptides were labeled with iTRAQ reagents according to the manufacturer’s instructions (iTRAQ Reagents Multiplex kit; Applied Biosystems/MDS Sciex, Foster City, CA). Briefly, 20 µg of nuclear proteins from each grade of the tumor or the control were vacuum-dried and resuspended in 20 µl of dissolution buffer and 1 µl of denaturant. Proteins were reduced, alkylated and trypsinized (with 1 µg modified sequencing grade trypsin; Promega, Madison, WI, USA) for 16 h, at 37 °C. Trypsin digested samples were labeled with four different iTRAQ reagents dissolved in 70 µl of ethanol at room temperature for 1.5 h^7^. Labeling tag details are as follows: control sample with 114, grade II sample with 115, grade III sample with 116 while grade IV (GBM) with 117 tags. Reactions were quenched with 10 mM glycine. All the four labeled samples were pooled, desalted using C18 microtips, vacuum dried and stored at −80 °C until mass spectrometric analysis.

#### LC-MS/MS analysis

Nanoflow electrospray ionization tandem mass spectrometric analyses of peptide samples were carried out using LTQ - Orbitrap Velos (Thermo Scientific, Bremen, Germany) coupled with Proxeon Easy nLC system (Thermo Scientific, Bremen, Germany). The chromatographic capillary columns used were packed with Magic C18 AQ (particle size 5 μm, pore size 100 Å; Michrom Bioresources, Auburn, CA, USA) reversed phase material in 100% ACN at a pressure of 1000 psi. Peptide samples were enriched using a C18 Trap column and separated on an analytical column (75 μm × 10 cm) at a flow rate of 350 nl/min using a linear gradient of 7–30% acetonitrile (ACN) over 65 min^9^. Mass spectrometric analysis was carried out in a data dependent manner with full scans acquired using the Orbitrap mass analyzer at a mass resolution of 60,000 at m/z 400. From each MS scan, twenty most intense precursor ions were selected for MS/MS fragmentation and detected at a mass resolution of 15,000 at m/z 400. The fragmentation was carried out using higher-energy collision dissociation (HCD) with 40% normalized collision energy. The ions selected for fragmentation were excluded for 30 sec^8^. The automatic gain control for full FT-MS was set to 1 million ions and for FT-MS/MS was set to 0.1 million ions with a maximum time of accumulation of 500 ms. For accurate mass measurements, the lock mass option was enabled. The entire procedure was repeated to generate a replicate run.

### Bioinformatics analysis

#### Identification and quantification of proteins

Protein identifications and quantifications of differentially expressed proteins were carried out as follows. The MS data was analyzed using Proteome Discoverer (Thermo Fisher Scientific, Version 1.4). MS/MS search was carried out using SEQUEST search engine against the NCBI RefSeq database version 70. Search parameters included trypsin as an enzyme with 1 missed cleavage allowed; precursor and fragment mass tolerance were set to 20 ppm and 0.1 Da, respectively; Methionine oxidation was set as a dynamic modification while methylthio modification at cysteine and iTRAQ modification at N-terminus of the peptide were set as static modifications. The FDR was calculated by enabling the peptide sequence analysis using a decoy database. High confidence peptide identifications were obtained by setting a target FDR threshold of 1% at the peptide level^8^. Both for identification of peptides/proteins and their quantification, signal to noise ratio applied was 1.5 or more and this is within the acceptable standards for the instrumentation platform used. Only peptides detected and identified in both normal and tumor specimens are included for quantitative analysis. Relative quantitation of proteins was determined based on the ratios of relative intensities of the reporter ions from tumor and normal released during MS/MS fragmentation of the each peptide. Appropriate quality control filters at the level of peptides/peptide spectral matches (PSMs) and then at the protein level was applied for the iTRAQ data.

- First, from each replicate run, only peptide/PSMs that are unique for a protein were included for fold change calculation and further selected only those proteins which were identified in both the runs.
- We then selected proteins with 1.3 fold change cut off and curated them further for 1.3 fold change even at the level of their respective peptide/PSMs. Then final protein fold change values were recalculated based on the fold change values of the curated peptides/PSMs and percentage variability determined.
- We finally selected proteins with minimum 1.5 fold change based on ≥2 unique peptides/PSMs having less than 40% coefficient of variation (CV). This list was used for further analysis.

#### Protein annotations, pathway and network analysis

Gene Ontology annotation of the differentially expressed proteins identified was carried out based on Human Protein Reference Database (HPRD, http://www.hprd.org)^14^ and UniProt Knowledge Database (UniProt, http://www.uniprot.org). The molecular functions and pathways were generated through the use of IPA^15^ (QIAGEN Inc., https://www.qiagenbioinformatics.com/products/ingenuity-pathway-analysis) tool.

For the network analysis, differentially expressed nuclear proteins from GBM as well as those from the microsomal fraction^7^ that are associated with the ‘transcription regulation’ function were considered. The combined dataset was further examined for concordance with expression at the transcript level (using Oncomine and TCGA resource). These combined concordant entities were used and functional interaction network was constructed using STRING (version 10.0)^16^ web resource (https://string-db.org). The confidence of the network was assessed by *p*-values observed in the STRING output. We then examined all the entities present in the functional network to map to regulatory miRNAs and target cascades. For this, we selected experimentally validated miRNA-target pair mode in miRWalk and used it as a reference to pair up the network entities with respective miRNA regulators together with inverse correlation in expression observed in GBM datasets^17^.

### Immunohistochemistry (IHC)

IHC was performed on formalin-fixed paraffin-embedded tissue section microarrays (TMAs) for selected proteins. As mentioned above under “Sample collection”, either commercial TMAs or in-house TMAs were used for IHC-based analysis. Commercial tissue microarrays (US BioMax) consisted of 4 control subjects, 13 diffuse astrocytomas (Grade II), 11 anaplastic astrocytomas (Grade III), and 27 glioblastoma (Grade IV) tissue samples. In-house TMAs were prepared as follows. TMA blocks were constructed using the FFPE blocks and included 18 GBM cases and 4 epilepsy controls. The TMA consisted of 44 cores of 2 mm diameter accounting for technical replicate of each sample, and Hematoxylin and Eosin (H & E) - stained sections of the blocks were used to define tumor regions. Further, 3-micron sections were cut from the TMA block for carrying out IHC. In brief, deparaffinization was performed in xylene solution followed by dehydration in ethanol and rehydration of the tissue with water. Antigen retrieval was performed by immersing the slide in antigen retrieval buffer (10 mM sodium citrate, 0.05% Tween 20, pH 6.0) at 95 °C for 5 min. Endogenous peroxidases were blocked with 0.03% hydrogen peroxide, and nonspecific binding was blocked with 2% bovine serum albumin (BSA) in Tris-buffered saline with 0.1% Triton X-100 (TBST, pH 7.6) for 30 min. Sections were then incubated for 1.5 h at room temperature with primary antibodies - HMGB2 (Abcam, ab11973, 1:200 dilution), PARP1 (Santacruz, sc-8007, 1:100 dilution), NUCKS1 (Abcam, ab84710, 1:100 dilution), SMARCA5 (Atlas Antibodies, HPA008751, 1:200 dilution), NF1B (Abcam, ab186738, 1:150 dilution), PTBP1 (Abcam, ab5642, 1:100 dilution), YBX1 (Abcam, ab12148, 1:500 dilution), EGFR (Atlas antibodies, HPA018530, 1:100 dilution), MAPK1 (Abcam, ab32081, 1:100 dilution), CD44 (BioGenex, AM310-5M, Ready to use), SOX2 (Invitrogen, PA-1-16968, 1:150 dilution), TNC (Cloud-clone corp, MAB975Hu22, 1:100 dilution) and MMP13 (Abcam, ab39012, 1:100 dilution) followed by peroxidase-labeled polymer conjugate to anti-goat, anti-mouse or anti-rabbit immunoglobulins compatible with the primary antibody for 1 h. After washing, sections were incubated for 5–20 min. with the 3, 3 - diaminobenzidine chromogen (DAB) system to develop the stain. Sections were counter stained with Mayer’s hematoxylin for about 30–60 sec. Finally, the sections were dehydrated through 95% ethanol, 100% ethanol and cleared them in xylene. IHC images were acquired and analyzed by two neuropathologists. The scoring details are provided in the results.

## Results

**Figure 1:**
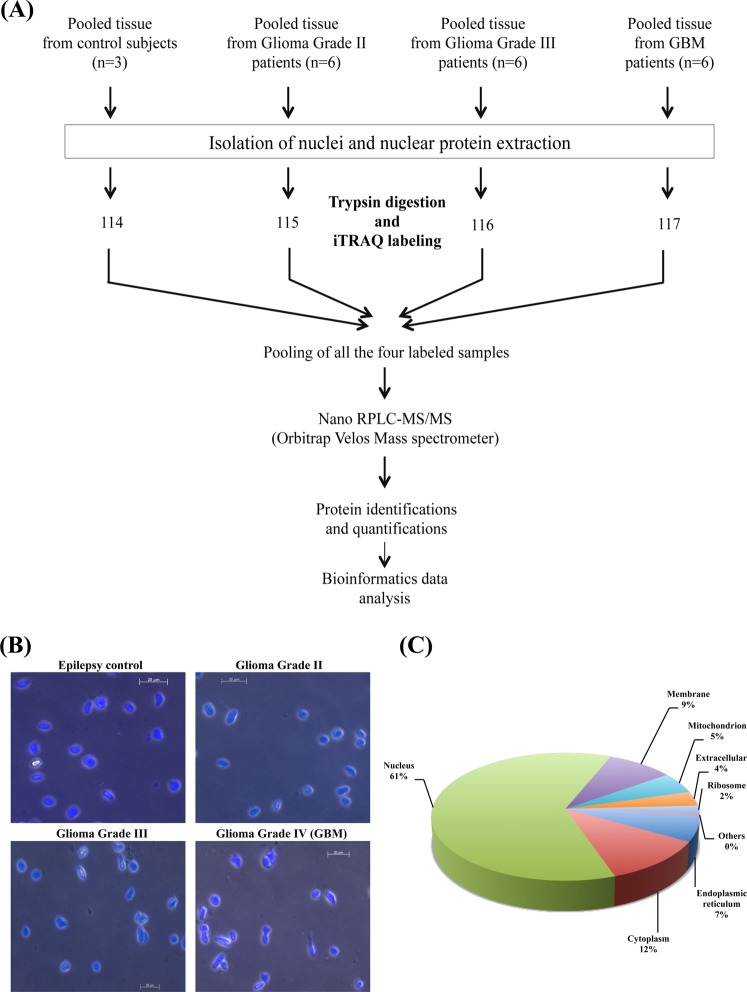
(**A**) Workflow for the analysis of differentially expressed nuclear proteins of different grades of astrocytoma. (**B**) Nuclear preparation from the clinical specimens stained with DAPI and observed under fluorescent phase contrast microscope. (**C**) Sub cellular classification of differentially expressed proteins carried out using Human Protein Reference Database. The classification shows the enrichment of nuclear proteins in the preparation.

In this study, we have performed sub-cellular fractionation and isolated nuclear fractions from the clinical tissue specimens of Glioblastoma and lower grade gliomas along with the control samples. Differentially expressed proteins were identified from these fractions using iTRAQ and LC-MS/MS analysis on LTQ Orbitrap Velos mass spectrometer. The overall workflow of the study is shown in Fig. 1A. Fluorescence microscopy analysis showed nuclei stained with DAPI with insignificant contamination of cells in the nuclear fractions isolated from different grades of gliomas and control tissue samples. We observed changes in the nuclear shape to elongated form and these changes were greater in Grade III and IV in comparison to Grade II and control samples **(**Fig. 1B**)**. Changes in nuclear size, shape and other properties have been earlier reported to be related to cell invasion and migration^18,19^.

Nuclear proteins are generally in low abundance than cytoplasmic or membrane proteins. The protocol used (or other protocols) for subcellular fractionation provides enrichment of the proteins localized in the respective fraction. Total purity of any subcellular fraction is difficult to be achieved. Although it is not possible to claim that the protocol yields all nuclear proteins, the protein profile identified revealed enrichment of nuclear proteins. More than 60% of the proteins identified have been known for their established nuclear function and included several low abundant proteins such as transcription or splicing factors.

### Differentially expressed proteins

From the replicate runs of nLC-MS/MS analysis of iTRAQ labeled tryptic digests of proteins from different grades of tumor tissues along with control, 5,623 peptides were identified which mapped to 743 proteins. Overall, we identified a total of 244 differentially expressed proteins (≥1.5 fold change and at least 2 PSMs) most of them (207 proteins) also with ≥2 unique peptides **(**Supplementary Table S2**)**. The distribution of differentially expressed proteins across grades along with their peptides is given in Table 1. Subcellular classification of the differentially expressed proteins from each grade using Gene Ontology information from HPRD and UniProtKB revealed about 65% of the proteins to have nuclear localization (Fig. 1C**)**. Supplementary Tables S3–S5 provides the list of these proteins along with their peptide information, quantity levels with percentage variability from grade II, grade III and GBM, respectively.

**Table 1:** Distribution of the differentially expressed (DE) nuclear proteins (n = 244) and their peptides across different grades of astrocytomas.

Sample	Identification of differentially expressed (DE) nuclear proteins				
	No. of DE proteins	Proteins identified with 4 or more unique peptides	Proteins identified with 3 unique peptides	Proteins identified with 2 unique peptides	Proteins identified with 1 unique peptide (with ≥2 PSMs)
Glioma Grade II	162 (78 up and 84 down)	72	33	31	26
Glioma Grade III	131 (67 up and 64 down)	52	21	34	24
GBM (Glioma Grade IV)	147 (70 up and 77 down)	68	24	34	21

Out of 743 total proteins identified in the analysis, 244 were found to be DE and used for further analysis. Majority of the DE proteins were identified with ≥2 unique peptides as shown in the Table. A small number of proteins identified with single unique peptide, which are included in the Table, were all represented by at least ≥2 PSMs.

Further, we compared the proteomic data with transcriptome data for different grades of gliomas from public domain resources and relevant literature^20–23^ (https://www.oncomine.org; http://csbi.ltdk.helsinki.fi/anduril/tcga-gbm/index.html). Of the total of 244 differentially expressed nuclear proteins, 168 matched with mRNA expression data from the respective grades **(**Supplementary Table S2**)**. Thus more than 65% of the entities showed concordance in expression trends at both mRNA and protein levels, adding further confidence to the data. The remaining proteins either did not have matching differential transcripts or not found to be concordant.

### Altered processes and enriched pathways

**Figure 2:**
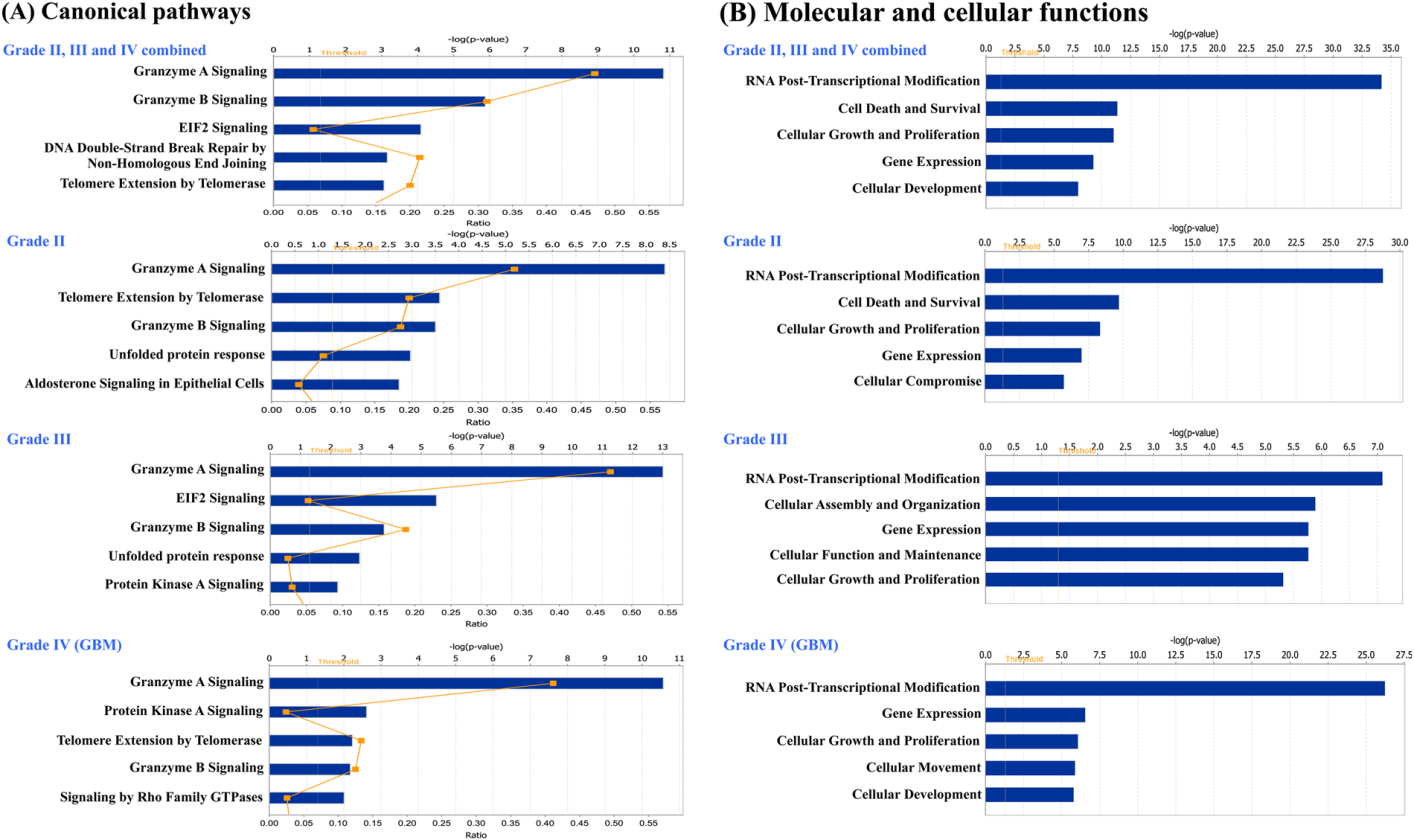
Ingenuity Pathway Analysis of differentially expressed nuclear proteins. The molecular functions and pathways were generated through the use of IPA^15^ (QIAGEN Inc., https://www.qiagenbioinformatics.com/products/ingenuity-pathway-analysis). Top five significant Canonical pathways (**A**) and Molecular and cellular functions (**B**) identified from analysis of non-redundant list of differentially expressed nuclear proteins (n = 244) from all grades combined and differentially expressed nuclear proteins grade-wise (Grade II- 162, Grade III-131 and Grade IV- 147 proteins).

To understand cellular pathways and processes enriched in our dataset, we subjected the total non-redundant list of differentially expressed proteins from all grades (n = 244) to Ingenuity Pathway Analysis (IPA) and identified enriched canonical pathways, molecular and cellular functions, and networks **(**Fig. 2A**)**. Granzyme A and B Signaling, was found to be the top pathway identified along with others such as EIF2 Signaling, DNA Double-Strand Break Repair Non-Homologous End Joining, and Telomere Extension. Several proteins belonging to ‘granzyme signaling’ including histones, high mobility group protein B2 (HMGB2), DNA-dependent protein kinase catalytic subunit isoform 2 (PRKDC), nuclear mitotic apparatus protein 1 isoform 2 (NUMA1), lamin-B1 isoform 1 (LMNB1), lamin-B2 (LMNB2) and poly [ADP-ribose] polymerase 1 (PARP1), were identified with altered levels.

Transcriptional and Post-Transcriptional Modification, Cell Death and Survival, Cellular Growth and Proliferation, Gene Expression and Cellular Development were the top 5 important molecular and cellular functions **(**Fig. 2B; Supplementary Table S2**)**. The protein IDs and p-values associated with canonical pathways, molecular and cellular functions, and networks are shown in Supplementary Table S6A–C. IPA analysis of the differentially expressed proteins from each of the three glioma grades (Grade II- 162 proteins, Grade III- 131 proteins, Grade IV- 147 proteins) separately also mapped to the same pathways, molecular and cellular functions **(**Fig. 2**)**, and networks (Supplementary Table S7**)**, except for the differences in ranking. Further, comparison of differentially expressed proteins in different grades revealed 53 of them being common and concurs in their expression trend across the grades **(**Supplementary Table S8). They are suggestive of the some of the common events and molecules underlying gliomagenesis regardless of the grade and type of the tumor and merit deeper exploration.

**Figure 3:**
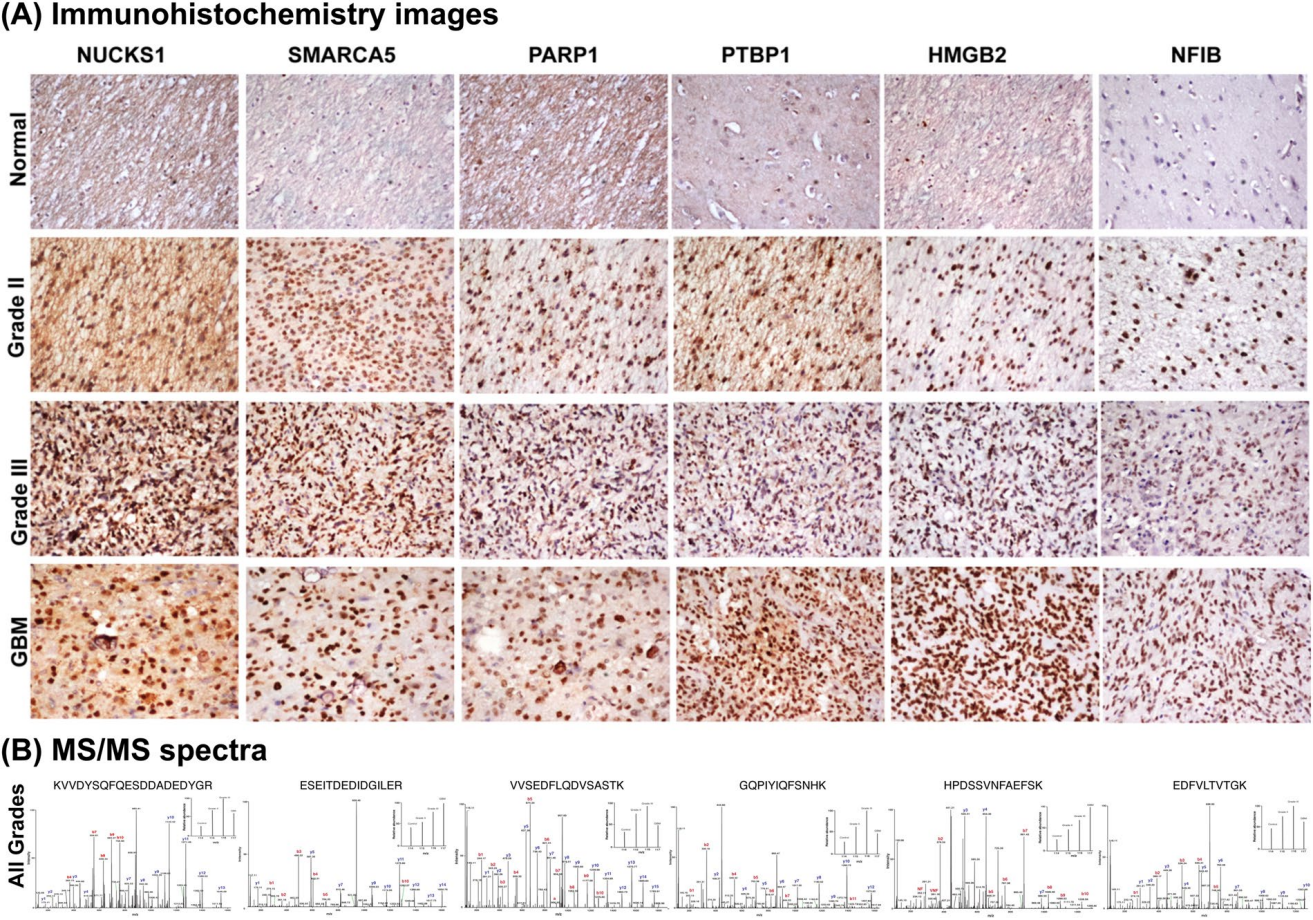
Immunohistochemistry on tissue microarrays of different grades of astrocytomas (**A**) and MS/MS spectra of representative peptides (**B**) for six selected proteins - NUCKS1, SMARCA5, PARP1, PTBP1, HMGB2 and NFIB. IHC analysis of the proteins tested using commercially available tissue microarrays (US BioMax), confirmed overexpression of these proteins in multiple tumor specimens. The details of tissue microarrays used and IHC procedure are described in the Methods and the staining scoring details are shown in Supplementary Table S9. MS/MS spectra acquisition is described under Methods.

The expression levels of functionally important proteins were further verified by IHC on TMAs consisting of multiple specimens from all grades. We selected six protein candidates - HMGB2, PARP1, nuclear ubiquitous casein and cyclin-dependent kinase substrate 1 (NUCKS1), SWI/SNF-related matrix-associated actin-dependent regulator of chromatin subfamily A member 5 (SMARCA5), nuclear factor 1 B-type isoform 3 (NF1B) and polypyrimidine tract-binding protein 1 isoform c (PTBP1) [or heterogeneous nuclear ribonucleoprotein A1 isoform a (HNRNPI)] on the basis of the extent of over expression in different grades, their functional and regulatory significance and/or its novelty as a differential protein. All of them are involved in transcriptional or post-transcriptional regulation while HMGB2 and PARP1 are also members of granzyme signaling. The IHC scoring results for each of the six proteins is shown in the Supplementary Table S9. The results not only confirm the observation made through iTRAQ analysis but also represent their expression trend in multiple tumor specimens. Representative IHC images and MS/MS spectra with the corresponding reporter ions for peptides are shown in Fig. 3A,B, respectively.

### Protein-protein interaction networks and functions

**Figure 4:**
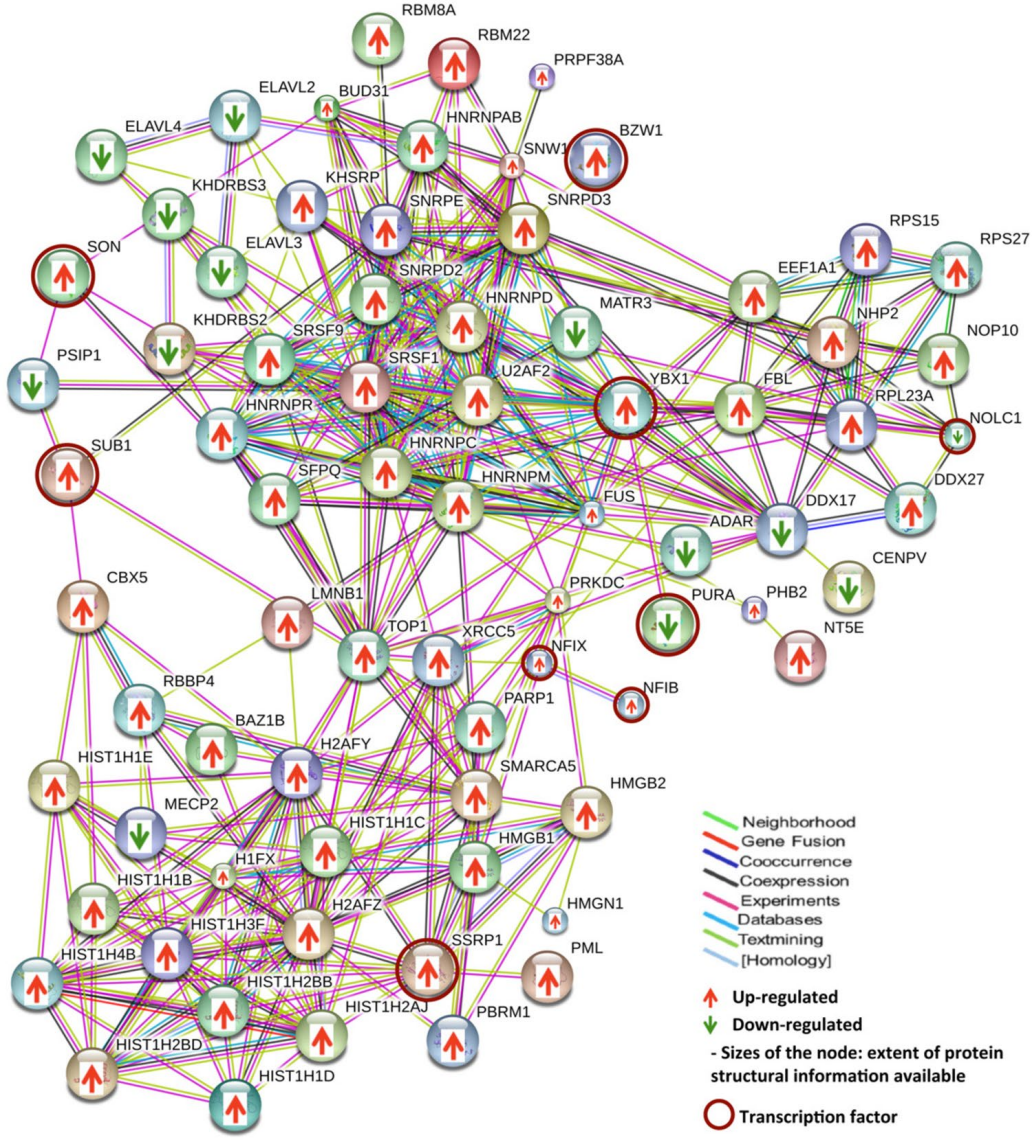
Protein-protein interaction network of proteins associated with transcription process. The interaction network was built with the transcription regulatory proteins including transcription factors and other DNA/RNA-binding proteins involved in transcription and post-transcription process, identified in GBM as shown in Supplementary Table 2. The network was constructed using STRING v10 web resource tool^16^ (https://string-db.org) as described in the Methods. Up or down regulation trend is represented by arrows.

**Figure 5:**
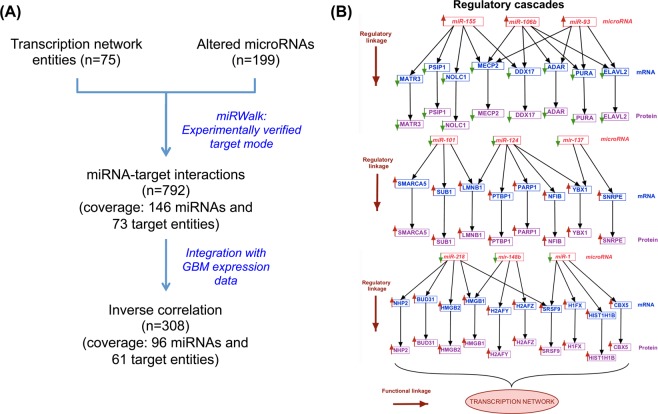
Integration of transcription network proteins with their corresponding transcripts and miRNA regulators to construct 2 - Dimensional molecular map with regulatory cascades in relation to function. (**A**) Pipeline for mapping transcription network proteins and transcripts (as shown in Fig. 4) to their putative miRNA regulators in GBM. (**B**) Representative regulatory cascades mapped using the workflow. The Figure represents 2D map analysis of the key miRNAs and their targets identified in GBM with inverse relationship in their expression. Up or down regulation trend of all the entities is represented by arrows. A complete list of these cascades is provided in the Supplementary Table S10.

With several altered transcription regulatory proteins (n = 131) observed in the dataset **(**Supplementary Table S2**)**, we carried out functional network analysis for these proteins to understand the interactions among them. We attempted this specifically for GBM and included proteins with transcription regulatory function identified in both microsomal^7^ and nuclear protein analysis (n = 51 and n = 90, respectively). To incorporate multilayer gene expression changes observed, we further selected only those proteins which were also supported with concordant differential expression of their corresponding transcripts (using information derived from the Public domain TCGA data). A total of 83 of these proteins annotated to transcription regulatory function, were found to be overlapping and concordant in their expression trend at the transcript level. These entities were used to construct the protein-protein interaction network using STRING tool. The STRING output captured 348 interactions (*p*-*value* = *2*.*17e*-*15*; Fig. 4) involving 75 of 83 entities, which included hnRNPs, high mobility group (HMG) proteins and some of the transcription factors highlighted as hub groups. Further extension of multi-omics integration to build regulatory cascades between differentially altered miRNAs and the mRNA/protein target entities are important in the context of tumor functions^17^. For this purpose, we mapped each one of these entities to experimentally validated miRNA regulators using miRWalk database, and screened them further for their respective inverse expression trend observed in the experimental data on GBM (TCGA transcriptomics and our proteomics data). We identified 61 miRNAs (more than 80%) to conform to this criterion. A schematic representation of the analysis pipeline and the results are shown in Fig. 5A and the details in Supplementary Table S10. Figure 5B shows a 2-Dimensional map consisting of the representative regulatory cascades that include protein members of the network, their corresponding transcripts and regulatory miRNA–with inverse trend in expression. MicroRNAs miR-155, miR-106b and miR-93 are reported to be overexpressed and are known to target many GBM-downregulated proteins/genes present in the network such as PC4 and SFRS1-interacting protein isoform 2 (PSIP1), nucleolar and coiled-body phosphoprotein 1 isoform 2 (NOLC1), methyl-CpG-binding protein 2 isoform 2 (MECP2), double-stranded RNA-specific adenosine deaminase isoform d (ADAR), transcriptional activator protein Pur-alpha (PURA), ELAV-like protein 2 isoform A (ELAVL2). On the other hand miRNAs miR-101, miR-124, miR-137, miR-218, miR-148b and miR-1 that are down regulated in GBM are known to target some of the GBM-overexpressed transcription factors such as Nuclease-sensitive element-binding protein 1 (YBX1 or YB1), activated RNA polymerase II transcriptional coactivator p15 (SUB1) and NF1B and other transcriptional regulators like HMGB1/2, PTBP1, PARP1, SMARCA5, LMNB1, protein BUD31 homolog (BUD31) present in the network. The oncogenic or tumor suppressor characteristics of these miRNAs have been well documented in the literature^24^. Many of the miRNAs represented in the figure show regulatory connection with more than one target in the network thus adding further significance to their regulatory role. This approach to consolidate and visualize the functional entities in regulatory perspective – the 2 Dimensional Molecular map, has been described by us earlier^17^.

**Figure 6:**
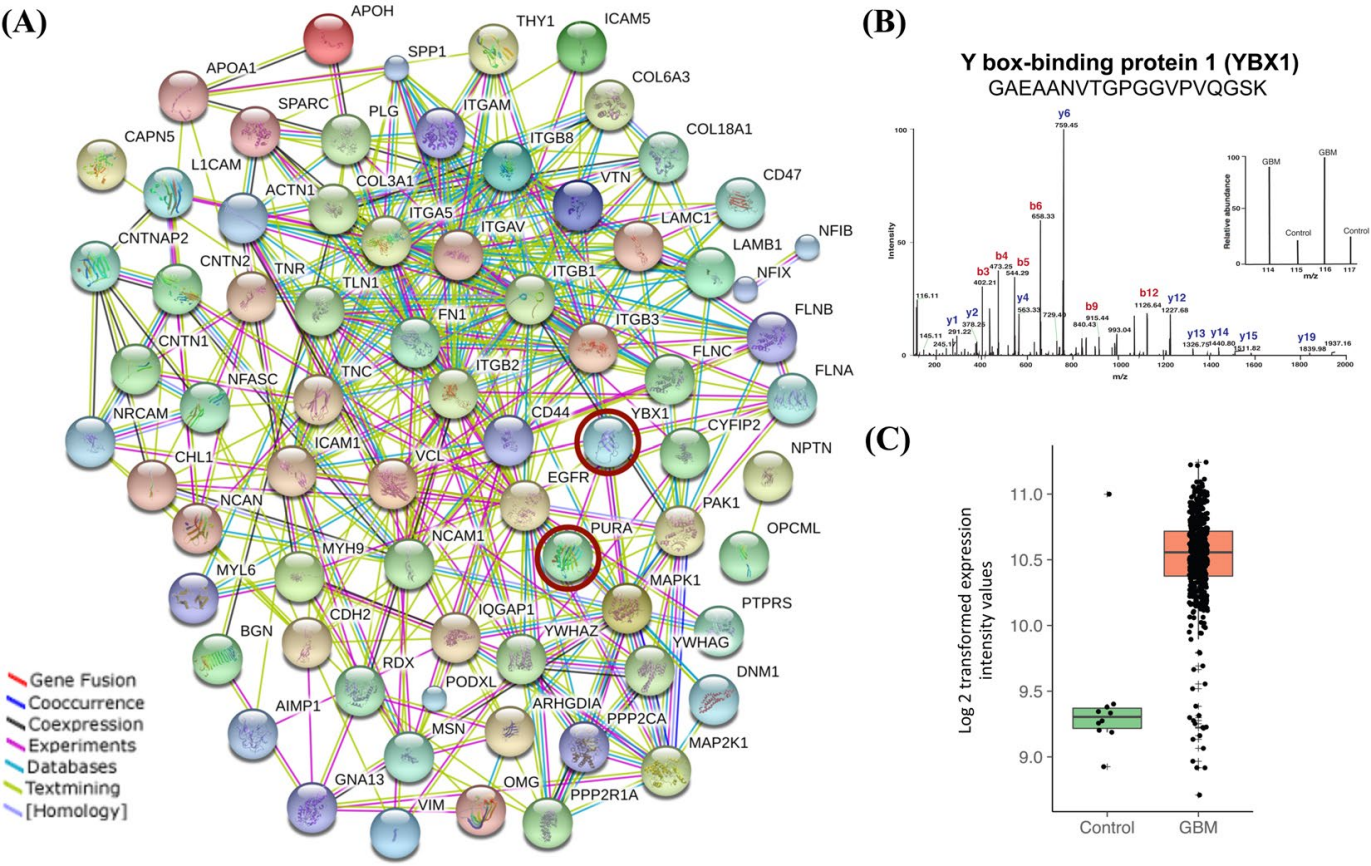
(**A**) Integrated protein-protein interaction network with transcription factors and proteins involved in the invasion process. The protein-protein interaction network was constructed with the transcription factors and invasion process entities, through the use of STRING v10 web resource tool^16^ (https://string-db.org). Two transcription factors YBX1 and PURA (circled with brown color) were captured in the interactions between the two - molecular functions. (**B**) Differential expression of YBX1 protein. This sub-panel showing the representative MS/MS spectra of YBX1 peptide with iTRAQ reporter ions and the peptide sequence identified in GBM as compared to the control (from the data from ref.^7^). (**C**) Scatter plot showing expression of YBX1 mRNA in GBM and control sample using public domain data (TCGA mRNA expression data^20^). Overexpression of YBX1 with 2.4 fold change was observed with p value < 0.001.

**Figure 7:**
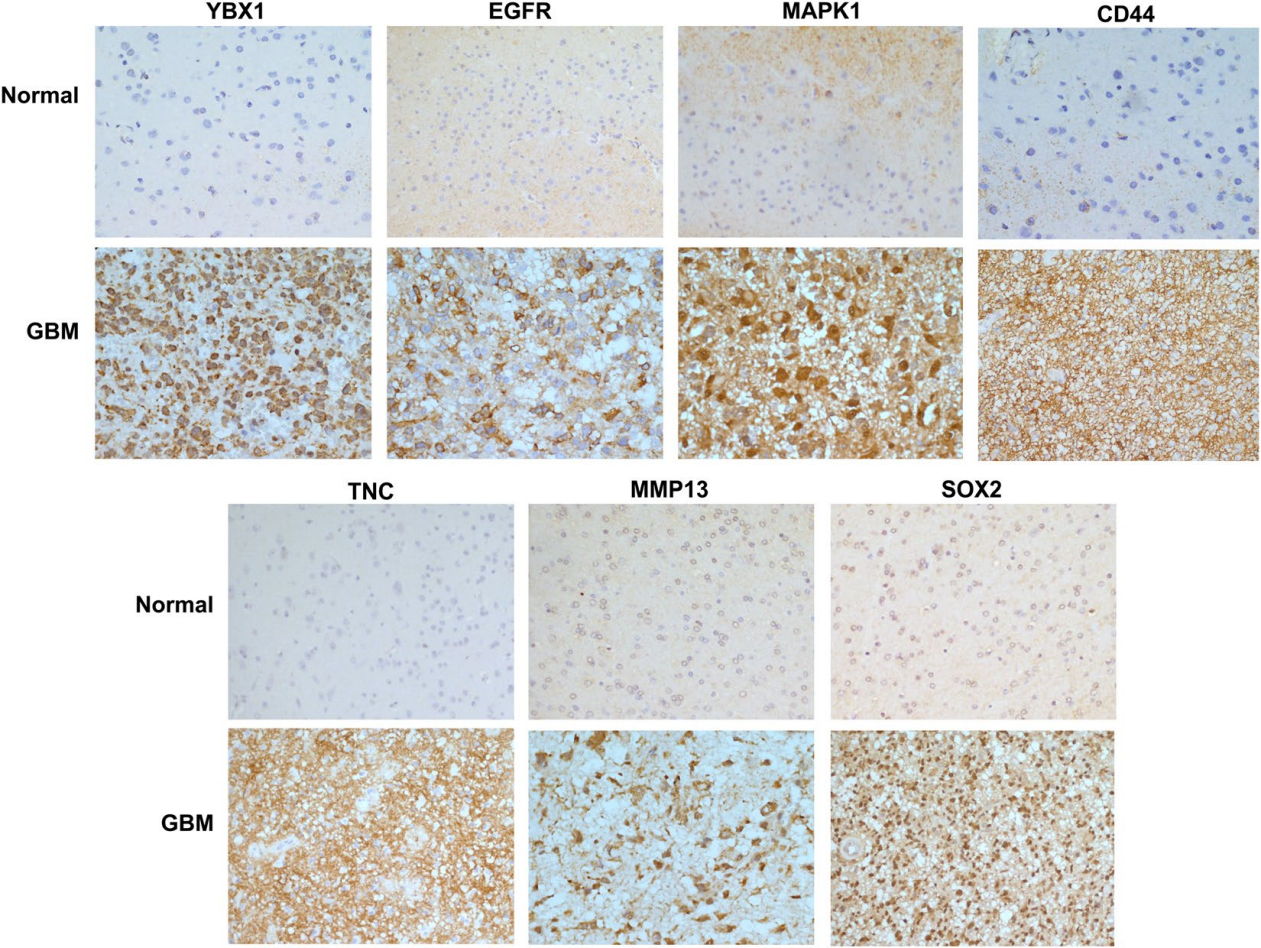
Immunohistochemistry on tissue microarrays of GBM for YBX1 and its interacting proteins - EGFR, MAPK1, CD44, TNC, MMP13 and SOX2. IHC analysis of the proteins tested using in-house prepared tissue microarrays, confirmed overexpression of YBX1 and its interacting proteins in multiple tumor specimens. The details of tissue microarrays used and IHC procedure are described in the Methods and the staining scoring details are shown in Supplementary Table S11.

Interestingly, many of the key transcription factors such as YBX1, NF1B, nuclear factor 1X -type isoform 3 (NF1X), SUB1, PURA, basic leucine zipper and W2 domain-containing protein 1 isoform 4 (BZW1) and FACT complex subunit SSRP1 (SSRP1) present in the network were found to be overexpressed in GBM and known to interact with various other tumor associated genes such as HMGB1, HMGB2, hnRNPs, DNA-dependent protein kinase catalytic subunit isoform 2 (PRKDC), RNA-binding protein FUS isoform 3 (FUS), rRNA 2′-O-methyltransferase fibrillarin (FBL), X-ray repair cross-complementing protein 5 (XRCC5), LMNB1, PSIP1, chromobox protein homolog 5 (CBX5), some of them implicated in tumor cell invasion. We therefore examined connections between the transcription factors with cell invasion network entities^17^. For this, we selected all the transcription factors i.e. proteins specifically binding to transcription regulatory sequences [n = 8; YBX1, NF1B, NF1X, SUB1, PURA, BZW1, protein SON isoform E (SON) and SSRP1] that were captured in the transcription network and looked for their interaction with the invasion related entities. This interaction network analysis generated a merged network **(**Fig. 6A**)**. Out of eight transcription factors used, the transcription factor YBX1 was found to be interacting with the key hub molecules like epidermal growth factor receptor isoform b (EGFR), mitogen-activated protein kinase 1 (MAPK1), integrin beta chain, beta 3 (ITGB3), and these in turn interacting further with several other molecules involved in tumor cell invasion. In our analysis, although YBX1 protein was not detected in the nuclear fraction it was distinctly detected as a differentially expressed protein in the microsomal fraction^7^ that included some of the nuclear proteins [This may be attributed to technical limitation of accessing proteins in mass spectrometric analysis of proteins/peptides without pre fractionation]. The levels of YBX1 were found to be significantly higher in GBM as compared to the control, i.e. 7.1 fold higher with 2 unique peptides and 8 PSMs^7^. Figure 6B shows a representative MS/MS spectrum of YBX1 peptide with iTRAQ reporter ion. The transcriptomic data for GBM from TCGA resource also supports overexpression of YBX1 – 2.4 fold change with significant *p*-*value* (≤0.001) **(**Fig. 6C**)**. Interestingly, YBX1 levels were also higher in the microsomal fractions of lower grade gliomas (6.8 fold change in Grade II and 3.8 fold change in Grade III) with 4 and 3 unique peptides and 10, 8 PSMs, respectively^8,9^. To substantiate the validity of some of the key linkages of YBX1 in GBM, we hypothesized to find concordance between the expression of YBX1 and its interacting proteins, if YBX1 was to be regulating their expression. With this hypothesis, we verified the expression of YBX1 and six of the interacting proteins (EGFR, MAPK1, CD44, tenascin C (TNC), matrix metalloproteinase 13 (MMP13) and SOX2) involved in cell invasion network by IHC on Tissue microarrays using clinical specimens of GBM. Activated EGFR and MAPK1 are involved in the Epithelial to mesenchymal transition by promoting transcription factors such as zinc finger protein SNAI1, and Twist-related protein 1 **(**TWIST1) in GBM, which in turn induce expression of cell invasion associated genes/proteins such as MMPs, Secreted protein acidic and rich in cysteine (SPARC)^25^. The interaction of CD44, a cell surface adhesion receptor, and matrix metalloproteinase 9 (MMP9) results in secretion of active MMP9 leading to migration and invasion of prostate cancer cells^26^. Sex determining region Y (SRY)-box 2 (SOX2) is a marker of cancer stem-like cells (CSCs) and is associated with the proneural molecular subtype of GBM. Ectopic Sox2 expression is reported to induce invasion and migration of glioma cells, and was further confirmed by knockdown experiments^27^. SOX2 knockdown decreased SRPK1 expression, which further led to the downregulation of PI3K and AKT expression levels^28^, further contributing to the metastasis and invasion phenotype^29^. TNC, an ECM protein, mediates cell invasion through MMP-12 and MMP-13, the key modulators of tumor invasion and metastasis, in glioma^30^ and breast cancer^31^. We observed positive expression of YBX1 in tumor tissues in the IHC analysis and interestingly, all of the interacting proteins selected showed concordance with YBX1 expression and showed positive expression in the tumor tissues as compared to the control **(**Fig. 7, Supplementary Table S11**)**. Thus we would like to infer that YBX1 has a role in regulating the expression of the key players involved in the invasion process in GBM.

## Discussion

The aim of this study has been to identify differentially expressed nuclear proteins that may impact gene expression changes relevant to tumor development and progression. We found 131 significantly altered transcription and post transcriptional- regulatory proteins which include a number of important DNA-binding proteins such as transcription factors (specifically binding to transcription regulatory sequences), high mobility group proteins (HMGs) and RNA-binding proteins such as hnRNPs, as described in Supplementary Table S12. Mapping of all the differentially expressed nuclear proteins to pathways and molecular functions revealed two important observations: 1. Altered expression of several DNA binding proteins and hnRNPs with their roles in transcription or post-transcriptional regulation and 2. Enrichment of granzyme signaling pathway.

### Transcription regulatory proteins

We observed a number of transcription regulatory proteins with altered expression in the study as listed in Supplementary Table S2. The results confirm the differential expression of many transcriptional regulatory proteins that have been already implicated in the pathogenesis of gliomas (HMG proteins -HMGA1, B1 and B2) as well as revealing some novel proteins (NUCKS1 and SON) discussed here. Overexpression of NUCKS1, having sequence homology to HMGA1 and role in DNA damage repair, has been reported in breast cancers^32^. Our analysis now reports altered expression of NUCKS1 in gliomas for the first time. SON is a splicing factor regulating genes involved in cell cycle, mitosis and other related processes^33^ and observed to be overexpressed in GBM. Similarly, protein level overexpression of HMGN proteins (HMGN1 and HMGN4) having nucleosome binding domain, is also being reported for the first time. HMGN1 is known to affect the structure and function of chromatin and plays an important role in repair of damaged DNA^34^. It promotes ADP-ribosylation of PARP1, a multifunctional nuclear enzyme reported to recognize and repair DNA lesions and to promote chromatin-remodeling^34^. PARP1 is also regarded as an important drug target. We observed overexpression of HMGN1 and PARP1 (also verified in TMAs) in GBM. We have identified another important transcription regulatory protein SMARCA5, a member of SWI/SNF family of proteins having helicase and ATPase activity involved in chromatin remodeling. It is reported to be overexpressed in advanced grades in comparison to lower grades of glioma and its overexpression correlates with increased cell proliferation, invasion and chemo resistance in gliomas^35^. Consistent with this report, we observed its overexpression in Grade IV tumors. Put together, we infer changes in many transcription determinants that may lead to downstream expression changes in other proteins governing cellular and molecular processes.

HNRNPs are a major group of RNA–binding proteins that have roles in splicing and other post-transcriptional processes and translation. They have been observed to be downregulated in all different grades of gliomas suggesting compromised post-transcriptional processing leading to altered expression of gene products and functional consequences^36^. A key multi-functional RNA binding protein, PTBP1 has been reported to be involved in tumorigenesis through aberrant alternate splicing of several genes involved in cell proliferation (*FGFR1*, *FGFR2*), invasion (*CSRC*), motility (*ACTN*, *FBN*), apoptosis (*FAS*, *CASP2*), and multi-drug resistance (*ABCC1*)^37^. It is also reported to be overexpressed in glioma cell lines and observed to promote cell proliferation and migration and inhibit cell adhesion^38^. We observe its overexpression in Grade II and III. Thus the deregulated expression of several members involved in post-transcriptional splicing process suggests the possibility of presence of splicing defects or alternatively spliced protein variants which may be explored using RNASeq and proteogenomic approaches.

### Altered Granzyme signaling and immunotherapeutic potential for GBM

Granzymes A and B, stored in the cytotoxic granules of cytotoxic T lymphocytes (CTL) and natural killer (NK) cells, are the most abundant granzymes of the pathway. They are delivered to the target cancerous cell through perforin and induce cell death^39^. Granzymes themselves could not be detected, presumably because of their low abundance in the target cells/tissue. However, the proteins targeted and cleaved by granzyme-mediated activation of caspase 3, are upregulated in the tumor tissues. These include members of histone family, other proteins such as HMGB2, LMNB1/B2, PARP1, NUMA1 and PRKDC. Upregulation of LMNB2, PRKDC and NUMA1 was detected in the microsomal fraction^7^ presumably due to their transport out of the nucleus. Over-expressions of HMGB2 and PARP1 have been verified by tissue microarray (TMA)-based immunohistochemistry (IHC) analysis. Consistent with this, the levels of miRNAs (miR-218 and miR-124) targeting HMGB2, PARP1 as well as miR-27a, targeting granzyme B and perforin, have been found to show inverse trend in their expression^40^. We thus speculate down regulation of granzyme signaling that may possibly induce anti-apoptotic events leading to cell survival, although not proven. Targeted cytotoxic killing of EGFRvIII mutant cancer cells in GBM by regulatory T lymphocytes using bi-specific antibodies has been reported to be a promising approach^41^ and may involve granzyme-mediated processes. The inference that granzyme activity may be down regulated in GBM opens up new possibilities of exploring this pathway of immune surveillance further. For example, it would be interesting to investigate and test the regulatory, cytotoxic T cell mediated killing of the tumor cells, using 3D cell culture models that include co-cultured tumor and activated T lymphocytes.

### Multi-omics data integration and protein - protein interaction network of transcription regulatory proteins

Transcription and post-transcriptional regulation is one of the complex process in the cells involving cis and trans elements, the latter being specific transcription factors^42^ and other DNA binding, chromatin modeling as well as RNA binding proteins. The interplay among these various factors could be a dynamic process changing from one functional context to the other. Through bioinformatics analysis, we have attempted to explore the regulatory and functional connectivity among these proteins. Multi-omics data integration and the 2-Dimensional molecular map for the protein-protein interaction network discussed in the results section (Figs 4, 5) reveal regulatory relationships in functional context among the proteins identified in the datasets. As shown and discussed under Fig. 5, most of the entities in the network were found to be linked in the regulatory dimension with their putative miRNA regulators observed with inverse trend in expression in GBM. Altered expression of hnRNPs, high mobility group (HMG) proteins and some of the transcription factors (TFs) were observed as ‘hub groups’. Of particular attention are SSRP1, a transcription factor, interacting with several histones and YBX1, another transcription factor and RNA binding protein, interacting with number of hnRNPs implying their regulatory impact on transcription and post-transcriptional events in GBM. HMG protein family members, involved in chromatin remodeling also form important hubs in the network and conform to the regulatory cascades discussed. Similarly, we could link regulatory miRNAs for other key proteins in the transcription network such as H1FX, LMNB1, SMARCA5, PARP1, HMGA1, HMGB2, PTBP1, NF1B, YBX1, PURA and BUD31.

### Identification of YBX1 as a putative regulator of cell invasion

To investigate the possibility of identifying transcription factor that may be potentially involved in the regulation of an important tumor-related process like cell invasion, we further extended the network analysis by merging transcription factors (n = 8) identified in GBM expression data with altered molecular entities involved in invasion^17^. Interestingly, in this merged interaction network (Fig. 6A), out of eight transcription factors used, we observed two transcription factor proteins - YBX1 and PURA interacting with the major hub molecules in the invasion network such as integrins, MAPK1 and EGFR. Of the two, YBX1 exhibited more direct interactions and may be of special significance.

YBX1 is a DNA and RNA binding protein implicated in regulation of transcription, pre-mRNA splicing, as well as stability and translation of mRNAs^43^. In brain, it is essential for normal development and is part of neural stem cell network. As discussed under the ‘Results’ section, YBX1 is overexpressed in GBM and other grades of gliomas. Further, several studies reported that the increased levels of YBX1 protein is connected to tumor progression as well as poor prognosis in glioma and other cancers^44,45^. Although it is not clear how exactly the expression of YBX1 itself is regulated, it is interesting to note the observation that miR-137 and miR-29b reported to regulate the expression of YBX1^46,47^, are found to be significantly downregulated in glioma. Mechanistically, when phosphorylated, YBX1 gets localized to nucleus and activates oncogenes such as EGFR, PI3KCA, cyclin A and B1, MMP2 and in turn affects proliferation and invasion process in colorectal cancer^48^. YBX1 is also reported to regulate the expression of MAPK1, involved in cell proliferation and invasion, and CD44 and CD49f (or integrin alpha 6), the genes linked to cancer stem cells^49^. YBX1 is also known to play a role in epithelial to mesenchymal transition by directly activating zinc finger protein, SNAI1 and other transcription factors implicated in activation of mesenchymal cell associated genes in breast cancer^50^. Recently, YBX1 gene silencing has been shown to affect the migratory and invasive potential of breast cancer *in*-*vitro* and involves CORO1C – a protein with WD repeats important for multi-molecular regulatory complexes^51^.

In view of the role of YBX1 in tumor-related processes and in the regulation of some of the key invasion-associated gene expression, we studied a plausible regulatory link between YBX1 expression and expression of key genes involved in the cell invasion network as explained under results, the hypothesis being concordance between the expression of the two in case of a regulatory connection. The IHC analysis using TMAs of GBM tissue specimens, for YBX1 and its interacting proteins (EGFR, MAPK1, CD44, TNC, MMP13 and SOX2) with documented role in cell invasion revealed distinct concordance between their expression, supporting a regulatory link between YBX1 and these proteins (Fig. 7). Consistently, several other studies also support the role of YBX1 as a regulator of these proteins. YBX1 has been reported to enhance EGFR transcription by directly binding to its promoter in breast cancer cells and chordoma cells^52,53^. It is reported to regulate protein expression of p-EGFR, p-AKT and its downstream target genes that influence cell apoptosis, cell cycle transition and cell invasion^53^. Silencing of YBX1 using siRNA decreased the expression of MAPK1 and phosphorylated MAPK1^54^ involved in several tumor related processes including invasion. YBX1 is also reported to bind to CD44 and CD49f promoter and enhance CD44 expression that promotes cancer cell growth and drug resistance in breast cancer^55^. TNC, an extracellular matrix protein, is reported to be overexpressed in high-grade gliomas^56^ and has a role in the upregulation of MMP13 in breast cancer^31^. YBX1 silencing is reported to significantly reduce MMP13 expression in melanoma cells^57^. YBX1 siRNA knockdown in GBM cell line and GBM patient derived primary brain tumor-initiating cells (BTIC) led to reduced expression of SOX2^58^. In the mice models for GBM, inhibition of YBX1 reduces GBM cell invasion and growth as well as enhances temozolomide sensitivity^59^. Put together, all these studies are in support of the key role of YBX1 in regulating key players in the processes such as tumor growth and invasion and thus in GBM biology in general. Our analysis arrives at this same inference independently through a different approach, i.e. the integrated bioinformatics analysis of the altered molecular profiles of GBM, and complements the leads revealed from the earlier experimental results elsewhere. The data together, underscores the need for further exploration of YBX1 as a potential therapeutic target^60^.

## Conclusions

Our study presents a resource of nearly 250 differentially altered nuclear proteins containing proteins involved in chromatin structure, transcriptional and post- transcriptional regulation of gene expression in gliomas. The dataset includes identification of novel candidates in the context of glioma biology. The highly suggestive, down regulation of granzyme signaling, an immuno-surveillance mechanism leads to the possibility of developing and exploring strategies for targeted induction of granzyme mediated cytotoxicity towards malignant cells. The novel bioinformatics strategy applied to protein-protein interaction network analysis integrates the data in two dimensions - functional and regulatory. This 2-Dimensional molecular map (2D map) not only consolidated the high-throughput molecular findings but also led to identification of putative miRNAs with key functional and clinical importance in GBM biology. The merged 2D map for transcription factors and cell invasion related entities reveals YBX1 to be potentially regulating cell invasion process – a conclusion independently drawn through bioinformatics approach and supports multiple earlier findings suggesting a key role of the protein in GBM pathophysiology.

## Supplementary information

Supplementary Tables S1, S2, S3, S4, S5, S6, S7, S8, S9, S10, S11 and S12

## Data Availability

The mass spectrometry based proteomics raw data have been deposited to The PRIDE PRoteomics IDEntifications (PRIDE) database and are available via ProteomeXchange with identifier PXD014111.
