# Supplementary material for: Altered transcriptional regulatory proteins in glioblastoma and YBX1 as a potential regulator of tumor invasion
Source: Sci Rep. 2019 Jul 29;9:10986. doi: 10.1038/s41598-019-47360-9 (PMC6662741; doi:10.1038/s41598-019-47360-9)
Supplement: Supplementary file 1 — Supplementary Tables S1, S2, S3, S4, S5, S6, S7, S8, S9, S10, S11 and S12 [file 41598_2019_47360_MOESM1_ESM.pdf]

Altered transcriptional regulatory proteins in glioblastoma and YBX1 as a potential regulator of tumor invasion

Manoj Kumar Gupta[1,2], Ravindra Varma Polisetty[3], Rakesh Sharma[1], Raksha A Ganesh[4], Harsha Gowda[1], Aniruddh K Purohit[5], Praveen Ankathi[5], Komal Prasad[6], Kiran Mariswamappa[6], Akhila Lakshmikantha[6], Megha S Uppin[5], Challa Sundaram[5], Poonam Gautam[7#], Ravi Sirdeshmukh[1,2,4#]

[1]Institute of Bioinformatics, International Tech Park, Bangalore-560066, India; [2]Manipal Academy of Higher Education, Madhav Nagar, Manipal-576104, India; [3]Department of Biochemistry, Sri Venkateswara College, University of Delhi, New Delhi-110021, India; [4]Neuro-Oncology, Mazumdar Shaw Center for Translational Research, Bangalore-560099, India; [5]Nizam's Institute of Medical Sciences, Hyderabad-500082, India; [6]Mazumdar Shaw Medical Center, Narayana Health, Bangalore-560099, India; [7]ICMR-National Institute of Pathology, Safdarjung Hospital Campus, New Delhi-110029, India

Running title: Glioma Nuclear Proteome Analysis

# Corresponding authors

Dr. Ravi Sirdeshmukh

Institute of Bioinformatics, International Tech Park,

Bangalore-560066, India

Phone: 0091-9885090963

E-mail: ravisirdeshmukh@gmail.com; ravi@ibioinformatics.org

AND

Dr. Poonam Gautam

ICMR-National Institute of Pathology,

Safdarjung Hospital Campus,

New Delhi-110029, India

Phone: 0091-8800845323

E-mail: gautam.poonam@gmail.com; poonamgautam.nip@gov.in

SUPPLEMENTARY FILE LEGENDS

Supplementary Table S1:

Number of stromal/immune cell signature proteins present in the proteomics datasets. The stromal and immune cell signatures determined through the application of the ESTIMATE tool to TCGA gene expression data for GBM and other cancers, were taken from the study of *Yoshihara, et al., 2013* (Ref. 11). We used the same set of pooled clinical specimens of different grades of gliomas to isolate membrane and nuclear sub-cellular fractions and profiled proteins in those fractions by mass spectrometry [membrane proteins (Ref. 7-9) or nuclear proteins (present study)]. The membrane and nuclear proteins identified by us from different grades of glioma specimens were separately compared for their overlap with the stromal/immune cell signature genes. A comparison was carried out between these overlapping proteins identified in each grade and the total proteins identified in the respective grades. The comparison was done at protein level, the peptide level as well as at the level of peptide mass spectral signals for the peptides (PSMs). The numbers in the Table correspond to total proteins identified in a sample, total number of peptides for all proteins identified and total number of mass spectrum matches/signals (PSMs) identified for all peptides of all proteins. The numbers given for the nuclear protein dataset for different grades are identical because all those samples were run in one multiplexed mass spectrometry run and the protein identifications are applicable to each of the grades (see Materials and Method section).

Supplementary Table S2:

Non-redundant list of differentially expressed proteins (DEPs) identified in different grades of astrocytomas. The list contains a total of 244 non-redundant DEPs derived from 162 DEPs identified from Grade II, 131 DEPs from Grade III and 147 DEPs from Grade IV GBM. Majority of them (n=207) were identified with ≥2 unique peptides and ≥1.5 fold change. The number of peptide/PSMs information, molecular function, biological process and cellular localization for each of the proteins identified are shown. The grade-wise quantitation details of DEPs in each grade (Gr II, III and IV) is shown in **Supplementary Tables S3, S4 and S5**,

respectively. Data on mRNA level expression from literature (as mentioned in the Results section) is shown in Columns I, M and Q and supports many protein members being concordant in their expression with corresponding mRNAs. DEPs with transcription regulatory function have been marked in column V.

Supplementary Table S3:

Differentially expressed proteins in Grade II astrocytoma. A total of 162 nuclear proteins (≥1.5 fold change) were identified after applying quality control filters as detailed in Methods section. The Table describes the total number of proteins and peptides identified in replicate mass spectrometry runs along with their quantity values.

Supplementary Table S4:

Differentially expressed proteins in Grade III astrocytoma. A total of 131 nuclear proteins (≥1.5 fold change) were identified after applying quality control filters as detailed in Methods section. The Table describes the total number of proteins and peptides identified in replicate mass spectrometry runs along with their quantity values.

Supplementary Table S5:

Differentially expressed proteins in Grade IV astrocytoma (GBM). A total of 147 nuclear proteins (≥1.5 fold change) were identified after applying quality control filters as detailed in Methods section. The Table describes the total number of proteins and peptides identified in replicate mass spectrometry runs along with their quantity values.

Supplementary Table S6:

Ingenuity Pathway Analysis of the non-redundant list of differentially expressed nuclear proteins (n=244) identified in different grades of astrocytomas. The proteins associated with major canonical pathways (A), molecular and cellular functions (B) and networks and processes (C), are listed with their p-value. The Table shows only top 5 canonical pathways, molecular and cellular functions, and networks.

In (C), proteins present in the dataset mapping to respective networks are shown in bold.

Supplementary Table S7:

Top 5 significant Networks and processes identified in nuclear protein analysis of different grades of astrocytoma when analyzed separately using Ingenuity Pathway Analysis.

Supplementary Table S8:

Proteins identified to be differentially expressed in all the grades studied - Grade II, III and IV. A total of 53 proteins identified were found common to all the grades. This protein subset has been derived from **Supplementary Table S2.**

Supplementary Table S9:

Immunohistochemistry on tissue microarray for six differentially expressed proteins, NUCKS1, SMARCA5, PARP1, PTBP1, HMGB2, and NFIB, identified by quantitative mass spectrometric analysis. Immunohistochemistry was performed using commercially available tissue microarrays (US BioMax) consisting of 4 control subjects, 13 diffuse astrocytomas (Grade II), 11 anaplastic astrocytomas (Grade III), and 27 glioblastoma (Grade IV) patients samples. Scoring criteria used were based on both staining intensities and distributions. Scores 0, +1, +2 and +3 indicate negative, low, medium and strong staining, respectively. The distribution of staining of cancer cells was scored as 0 (less than 5% of cells staining), 1+ (5–30% of cell staining), 2+ (31–60% of cells staining) and 3+ (greater than 60% of cells staining). TMA slides were evaluated independently by two neuropathologists.

Supplementary Table S10:

List of 61 concordant mRNA and protein target entities present in the transcription interaction network and their microRNA regulators. The miRNA interaction were identified, as per pipeline shown in **Figure 5A** and described in the Methods, for mRNA and protein entities present in the transcription network as shown in **Figure 4**. Each of the miRNA-target interaction was supported by at least one PubMed ID.

Supplementary Table S11:

Immunohistochemistry on tissue microarrays of GBM for YBX1 and its interacting proteins, EGFR, MAPK1, CD44, TNC, MMP13 and SOX2 identified by protein-protein network analysis. Immunohistochemistry was performed using in-house prepared tissue microarrays consisting of 4 control subjects and 18 glioblastoma (Grade IV) patient samples. Scoring criteria used were based on both staining intensities and distributions. TMA slides were evaluated independently by two neuropathologists. The distribution of staining of cancer cells was scored as 0 (less than 5% of cells staining), 1+ (5–30% of cell staining), 2+ (31–60% of cells staining) and 3+ (greater than 60% of cells staining). Scores 0, +1, +2 and +3 indicate negative, low, medium and strong staining respectively.

Supplementary Table S12:

List of selected differentially expressed transcription regulatory proteins identified in the study and their role in tumorigenesis.

Gupta et al., 2019. Altered transcriptional regulatory proteins in glioblastoma and YBX1 as a potential regulator of tumor invasion

Supplementary Table 1: Number of stromal/immune cell signature proteins present in the proteomics datasets. The stromal and immune cell signatures determined through the application of the ESTIMATE tool to TCGA gene expression data for GBM and other cancers, were taken from the study of *Yoshihara, et al., 2013* (Ref. 11). We used the same set of pooled clinical specimens of different grades of gliomas to isolate membrane and nuclear sub-cellular fractions and profiled proteins in those fractions by mass spectrometry [membrane proteins (Ref. 7-9) or nuclear proteins (present study)]. The membrane and nuclear proteins identified by us from different grades of glioma specimens were separately compared for their overlap with the stromal/immune cell signature genes. A comparison was carried out between these overlapping proteins identified in each grade and the total proteins identified in the respective grades. The comparison was done at protein level, the peptide level as well as at the level of peptide mass spectral signals for the peptides (PSMs). The numbers in the Table correspond to total proteins identified in a sample, total number of peptides for all proteins identified and total number of mass spectrum matches/signals (PSMs) identified for all peptides of all proteins. The numbers given for the nuclear protein dataset for different grades are identical because all those samples were run in one multiplexed mass spectrometry run and the protein identifications are applicable to each of the grades (see Materials and Method section).

**Protein Level**			
**Specimen - dataset**	**Total proteins**	**Stromal Cell Signature proteins**	**Immune Cell Signature proteins**
GBM - Nuclear	743	1	3
GrIII - Nuclear			
GrII - Nuclear			
GBM - Membrane	1834	12	16
GrIII - Membrane	2642	9	19
GrII - Membrane	2803	6	17
**Peptide Level**			
**Specimen - dataset**	**Total peptides**	**Stromal Cell Signature peptides**	**Immune Cell Signature peptides**
GBM - Nuclear	2409	1	4
GrIII - Nuclear			
GrII - Nuclear			
GBM - Membrane	7596	34	84
GrIII - Membrane	9393	20	45
GrII - Membrane	9352	20	48
**PSM Level**			
**Specimen - dataset**	**Total PSMs**	**Stromal Cell Signature PSMs**	**Immune Cell Signature PSMs**
GBM - Nuclear	5623	19	5
GrIII - Nuclear			
GrII - Nuclear			
GBM - Membrane	21408	44	213
GrIII - Membrane	22991	31	69
GrII - Membrane	23688	35	105

Supplementary Table S2: Non-redundant list of differentially expressed proteins (DEPs) identified in different grades of astrocytomas. The list contains a total of 244 non-redundant DEPs derived from 162 DEPs identified from Grade II, 131 DEPs from Grade III and 147 DEPs from Grade IV GBM. Majority of them (n=207) were identified with ≥2 unique peptides and ≥1.5 fold change. The number of peptide/PSMs information, molecular function, biological process and cellular localization for each of the proteins identified are shown. The grade-wise quantitation details of DEPs in each grade (Gr II, III and IV) is shown in **Supplementary Tables S3, S4 and S5**, respectively. Data on mRNA level expression from literature (as mentioned in the Results section) is shown in Columns I, M and Q and supports many protein members being concordant in their expression with corresponding mRNAs. DEPs with transcription regulatory function have been marked in column V.

Accession	NP accession	Gene Symbol	Gene id	Protein name	Grade II Σ# Unique Peptides	Grade II Σ# PSMs	Grade II protein fold change [115/114]	Grade II transcript fold change	Grade III Σ# Unique Peptides	Grade III Σ# PSMs	Grade III protein fold change [116/114]	Grade III transcript fold change	Grade IV Σ# Unique Peptides	Grade IV Σ# PSMs	Grade IV protein fold change [117/114]	Grade IV transcript fold change	Molecular class	Biological process	Molecular function	Final Localization	Transcription regulatory proteins
109689718	NP_001035937.1	ABHD12	26090	monoacylglycerol lipase ABHD12 isoform a	2	2	0.486		2	3	0.622	1.32	2	3	0.654		Integral membrane protein	phosphatidylserine catabolic process	carboxylesterase activity	Membrane	
316659409	NP_001186883.1	ACTG1	71	actin, cytoplasmic 2	3	7	2.430	1.29					2	5	1.867	1.38	Structural protein	Cell growth and/or maintenance	Structural constituent of cytoskeleton	Cytoplasm	
70167113	NP_001020278.1	ADAR	103	double-stranded RNA-specific adenosine deaminase isoform d	8	18	0.448		5	12	0.560		11	23	0.509	0.77	Enzyme: Deaminase	Regulation of nucleobase, nucleoside, nucleotide and nucleic acid metabolism	Deaminase activity	Nucleus	+
4502027	NP_000468.1	ALB	213	serum albumin preproprotein	11	18	3.103		11	16	1.887		11	18	2.684		Transport/cargo protein	Transport	Transporter activity	Extracellular	
238776833	NP_005773.3	ALYREF	10189	THO complex subunit 4					3	9	1.554	1.50					-	-	-	Nucleus	
4502101	NP_000691.1	ANXA1	301	annexin A1									4	5	3.894	7.58	Calcium binding protein	Cell communication,Signal transduction	Calcium ion binding	Membrane	
50845386	NP_001002857.1	ANXA2	302	annexin A2 isoform 2									1	2	3.360	6.12	Calcium binding protein	Cell communication,Signal transduction	Calcium ion binding	Nucleus	
4502107	NP_001145.1	ANXA5	308	annexin A5	7	10	2.695	2.42	7	10	2.263	2.34	6	8	2.963	3.60	Calcium binding protein	Cell communication,Signal transduction	Calcium ion binding	Cytoplasm	
302129652	NP_001180473.1	ANXA6	309	annexin A6 isoform 2	6	8	2.613		6	10	1.828		7	10	2.405	0.58	Calcium binding protein	Cell communication,Signal transduction	Calcium ion binding	Endoplasmic reticulum	
4557321	NP_000030.1	APOA1	335	apolipoprotein A-I preproprotein	5	10	1.961						6	11	2.036		Transport/cargo protein	Transport	Transporter activity	Extracellular	
4557325	NP_000032.1	APOE	348	apolipoprotein E isoform b precursor	5	5	2.183	2.73									Transport/cargo protein	Transport	Transporter activity	Extracellular	
50428938	NP_004308.2	ASNA1	439	ATPase ASNA1									1	2	0.667	1.29	ATPase	Metabolism,Energy pathways	ATPase activity	Cytoplasm	
4502277	NP_001668.1	ATP1B1	481	sodium/potassium-transporting ATPase subunit beta-1					1	2	0.674	0.57					ATPase	Transport	ATPase activity	Membrane	
4757810	NP_004037.1	ATP5A1	498	ATP synthase subunit alpha, mitochondrial isoform a precursor					5	9	0.560						Transport/cargo protein	Metabolism,Energy pathways	Transporter activity	Mitochondrion	
32189394	NP_001677.2	ATP5B	506	ATP synthase subunit beta, mitochondrial precursor	5	6	1.503	0.64	5	7	0.607	0.73					Transport/cargo protein	Metabolism,Energy pathways	Transporter activity	Mitochondrion	
30795231	NP_006308.3	BASP1	10409	brain acid soluble protein 1									4	5	0.564	0.23	Transcription regulatory protein	Regulation of nucleobase, nucleoside, nucleotide and nucleic acid metabolism	Transcription regulator activity	Nucleus	+
14670392	NP_115784.1	BAZ1B	9031	tyrosine-protein kinase BAZ1B									8	11	1.819	2.27	Transcription regulatory protein	Regulation of nucleobase, nucleoside, nucleotide and nucleic acid metabolism,Regulation of gene expression, epigenetic	Transcription regulator activity	Nucleus	+
213511012	NP_001132913.1	BCAP31	10134	B-cell receptor-associated protein 31 isoform b									2	4	1.532		Transport/cargo protein	Transport	Transporter activity	Endoplasmic reticulum	
32171175	NP_003901.2	BUD31	8896	protein BUD31 homolog									2	2	2.025	1.57	Transcription regulatory protein	Regulation of nucleobase, nucleoside, nucleotide and nucleic acid metabolism	Transcription regulator activity	Nucleus	+
25952118	NP_741960.1	CAMK2A	815	calcium/calmodulin-dependent protein kinase type II subunit alpha isoform 2	1	2	0.459	0.18	1	2	0.357	0.25	1	2	0.294	0.05	Serine/threonine kinase	Cell communication,Signal transduction	Protein serine/threonine kinase activity	Others	
6912292	NP_036249.1	CBX5	23468	chromobox protein homolog 5	3	4	0.650						2	4	1.510	1.78	DNA binding protein	Regulation of nucleobase, nucleoside, nucleotide and nucleic acid metabolism	DNA binding	Nucleus	+
10835071	NP_004346.1	CD74	972	HLA class II histocompatibility antigen gamma chain isoform b									2	2	1.843	2.75	MHC complex protein	Immune response	MHC class I receptor activity,MHC class II receptor activity	Membrane	
4502693	NP_001760.1	CD9	928	CD9 antigen	1	2	1.604										Unclassified	Immune response	Molecular function unknown	Membrane	
11067747	NP_001244.1	CDC5L	988	cell division cycle 5-like protein					4	6	0.668						Cell cycle control protein	Cell communication,Signal transduction	Protein binding	Nucleus	
56118234	NP_859067.2	CENPV	201161	centromere protein V	4	5	0.397		3	4	0.597		2	4	0.582	0.56	DNA binding protein	Regulation of nucleobase, nucleoside, nucleotide and nucleic acid metabolism	DNA binding	Nucleus	+
32455256	NP_859530.1	CERS2	29956	ceramide synthase 2	2	3	0.593										Integral membrane protein	Regulation of cell growth	Molecular function unknown	Nucleus	
664806003	NP_001287744.1	CIRBP	1153	cold-inducible RNA-binding protein isoform 2	2	4	0.381		2	3	0.434		1	2	0.157		RNA binding protein	Cell communication,Signal transduction	RNA binding	Nucleus	+
21536286	NP_001814.2	CKB	1152	creatine kinase B-type	3	4	1.867	0.54	3	4	1.610		3	3	0.665	0.48	Enzyme: Phosphotransferase	Metabolism,Energy pathways	Catalytic activity	Cytoplasm	
66346730	NP_001015001.1	CKMT1A	548596	creatine kinase U-type, mitochondrial precursor	5	7	0.503	0.30	6	7	0.444	0.20	5	7	0.458	0.50	Enzyme: Phosphotransferase	Energy pathways	Transferase activity	Mitochondrion	
8923900	NP_061156.1	CMAS	55907	N-acylneuraminate cytidylyltransferase	3	6	0.481	0.39	4	7	0.647	0.47	4	7	0.535	0.30	Enzyme: Ligase	Metabolism,Energy pathways	Ligase activity	Nucleus	
94721261	NP_149124.3	CNP	1267	2',3'-cyclic-nucleotide 3'-phosphodiesterase	2	4	1.498	0.38					1	3	0.641	0.55	Enzyme: Phosphodiesterase	Metabolism,Energy pathways	Phosphoric diester hydrolase activity	Cytoplasm	
366039949	NP_001242992.1	CNTN1	1272	contactin-1 isoform 3 precursor									1	2	0.432	0.16	Adhesion molecule	Cell growth and/or maintenance	Cell adhesion molecule activity	Membrane	
4503057	NP_001876.1	CRYAB	1410	alpha-crystallin B chain	4	6	2.292	1.59									Heat shock protein	Protein metabolism	Heat shock protein activity	Nucleus	
4503149	NP_001902.1	CTSG	1511	cathepsin G preproprotein									3	4	3.675	1.43	Serine protease	Protein metabolism	Serine-type peptidase activity	Extracellular	
4503183	NP_001905.1	CYB5A	1528	cytochrome b5 isoform 2	3	4	1.899	1.35	2	3	1.887		2	3	1.871	1.76	Enzyme: Oxidoreductase	Metabolism,Energy pathways	Oxidoreductase activity	Mitochondrion	
193794826	NP_001123291.1	CYB5R3	1727	NADH-cytochrome b5 reductase 3 isoform 2	1	3	1.561		1	3	1.551						Enzyme: Reductase	Metabolism,Energy pathways	Catalytic activity	Cytoplasm	
38201710	NP_006377.2	DDX17	10521	probable ATP-dependent RNA helicase DDX17 isoform 1	15	23	0.417	1.52	14	22	0.567	2.39	15	25	0.535	0.69	ATPase,RNA helicase	Regulation of nucleobase, nucleoside, nucleotide and nucleic acid metabolism	ATPase activity	Nucleus	+
379317177	NP_001243839.1	DDX21	9188	nucleolar RNA helicase 2 isoform 2	5	8	1.599		5	8	1.788						ATPase	Metabolism,Energy pathways	ATPase activity	Nucleus	
224593278	NP_060365.7	DDX27	55661	probable ATP-dependent RNA helicase DDX27									3	3	1.728	1.35	RNA helicase	Regulation of nucleobase, nucleoside, nucleotide and nucleic acid metabolism	Helicase activity	Nucleus	+
41327773	NP_055644.2	DDX46	9879	probable ATP-dependent RNA helicase DDX46 isoform 2	3	4	0.651		3	4	0.659						RNA helicase	Regulation of nucleobase, nucleoside, nucleotide and nucleic acid metabolism	Helicase activity	Nucleus	+
4758138	NP_004387.1	DDX5	1655	probable ATP-dependent RNA helicase DDX5	8	12	0.450		5	9	0.597		8	13	0.552	2.06	RNA binding protein	Regulation of nucleobase, nucleoside, nucleotide and nucleic acid metabolism	RNA binding	Nucleus	+
197927454	NP_001128181.1	DEK	7913	protein DEK isoform 2	4	11	1.724		6	16	1.889	1.41					DNA binding protein	Regulation of nucleobase, nucleoside, nucleotide and nucleic acid metabolism	DNA binding	Nucleus	+
100913206	NP_001348.2	DHX9	1660	ATP-dependent RNA helicase A									11	23	0.612	1.50	Transcription factor	Regulation of nucleobase, nucleoside, nucleotide and nucleic acid metabolism	Transcription factor activity	Nucleus	+
4503337	NP_001354.1	DKC1	1736	H/ACA ribonucleoprotein complex subunit 4 isoform 1					6	8	1.789	2.71					RNA binding protein	Regulation of nucleobase, nucleoside, nucleotide and nucleic acid metabolism	RNA binding	Nucleus	+
38201714	NP_001410.2	ELAVL1	1994	ELAV-like protein 1	9	18	0.429	2.19					7	11	0.174	5.49	RNA binding protein	Regulation of nucleobase, nucleoside, nucleotide and nucleic acid metabolism	RNA binding	Nucleus	+
49355765	NP_115657.2	ELAVL3	1995	ELAV-like protein 3 isoform 2	3	5	0.581	0.21	2	4	0.630	0.55	1	2	0.127	0.28	RNA binding protein	Regulation of nucleobase, nucleoside, nucleotide and nucleic acid metabolism	RNA binding	Nucleus	+
221316767	NP_001138246.1	ELAVL4	1996	ELAV-like protein 4 isoform 2	1	2	0.427	0.53	1	2	0.450	0.54	1	2	0.418	0.15	RNA binding protein	Regulation of nucleobase, nucleoside, nucleotide and nucleic acid metabolism	RNA binding	Nucleus	+
4503571	NP_001419.1	ENO1	2023	alpha-enolase isoform 1	3	4	2.610		3	4	1.929						Enzyme: Hydratase	Metabolism,Energy pathways	Catalytic activity	Cytoplasm	
6005721	NP_009106.1	ERLIN2	11160	erlin-2 isoform 1					3	6	0.625	1.34	3	6	0.552	1.39	Unclassified	Biological_process unknown	Molecular function unknown	Endoplasmic reticulum	

344925834	NP_001230710.1	FAM213A	84293	redox-regulatory protein FAM213A isoform 2 precursor					4	8	2.140						-	oxidation-reduction process	antioxidant activity	Cytoplasm	
12056465	NP_001427.2	FBL	2091	rRNA 2'-O-methyltransferase fibrillarin	9	16	1.638						6	11	1.662	1.78	Ribonucleoprotein	Regulation of nucleobase, nucleoside, nucleotide and nucleic acid metabolism	Ribonucleoprotein	Nucleolus	+
283135173	NP_001164408.1	FUS	2521	RNA-binding protein FUS isoform 3									3	5	1.486	1.60	RNA binding protein	RNA localization	Nucleocytoplasmic transporter activity	Nucleus	+
378404908	NP_001243728.1	GAPDH	2597	glyceraldehyde-3-phosphate dehydrogenase isoform 2	2	4	2.460		2	4	1.875						Enzyme: Dehydrogenase	Metabolism,Energy pathways	Catalytic activity	Cytoplasm	
9506713	NP_061856.1	GAR1	54433	H/ACA ribonucleoprotein complex subunit 1					3	6	1.616	1.49					Enzyme: Reductase	Regulation of nucleobase, nucleoside, nucleotide and nucleic acid metabolism	Ribonucleoprotein	Nucleolus	+
4503979	NP_002046.1	GFAP	2670	glial fibrillary acidic protein isoform 1	17	25	3.054	4.91	16	23	2.076	5.25	14	21	1.940	1.40	Structural protein	Cell growth and/or maintenance	Structural molecule activity	Cytoplasm	
4885281	NP_005262.1	GLUD1	2746	glutamate dehydrogenase 1, mitochondrial precursor	4	5	2.417										Enzyme: Dehydrogenase	Metabolism,Energy pathways	Catalytic activity	Mitochondrion	
40556376	NP_115958.2	GLYR1	84656	putative oxidoreductase GLYR1					2	3	1.507						Enzyme: Dehydrogenase	Metabolism,Energy pathways	Catalytic activity	Nucleus	
10567816	NP_066268.1	GNAO1	2775	guanine nucleotide-binding protein G(o) subunit alpha isoform a	3	3	1.563	0.46					4	5	0.626	0.16	G protein	Cell communication,Signal transduction	GTPase activity	Membrane	
54114974	NP_444292.1	GNG2	54331	guanine nucleotide-binding protein G(I)/G(S)/G(O) subunit gamma-2 precursor									2	3	0.425	0.40	G protein	Cell communication,Signal transduction	GTPase activity	Membrane	
387598076	NP_001248376.1	GPM6A	2823	neuronal membrane glycoprotein M6-a isoform 2	3	3	1.759	0.77					2	2	0.484	0.45	Integral membrane protein	Neurogenesis	Ion channel activity	Membrane	
4885371	NP_005309.1	H1F0	3005	histone H1.0	4	30	1.713		5	35	2.249						DNA binding protein	Regulation of nucleobase, nucleoside, nucleotide and nucleic acid metabolism	DNA binding	Nucleus	+
5174449	NP_006017.1	H1FX	8971	histone H1x	4	13	1.653		4	17	2.438		4	16	1.889	1.55	DNA binding protein	Regulation of nucleobase, nucleoside, nucleotide and nucleic acid metabolism	DNA binding	Nucleus	+
4504253	NP_002096.1	H2AFX	3014	histone H2AX	2	2	0.431										DNA binding protein	DNA repair	DNA binding	Nucleus	+
20336746	NP_613075.1	H2AFY	9555	core histone macro-H2A.1 isoform 1									7	11	3.786	1.49	DNA binding protein	Regulation of nucleobase, nucleoside, nucleotide and nucleic acid metabolism	DNA binding	Nucleus	+
4504255	NP_002097.1	H2AFZ	3015	histone H2A.Z	2	6	1.998		2	5	1.561		2	6	2.701	1.84	DNA binding protein	Regulation of nucleobase, nucleoside, nucleotide and nucleic acid metabolism	DNA binding	Nucleus	+
117168248	NP_057479.2	HACD3	51495	very-long-chain (3R)-3-hydroxyacyl-CoA dehydratase 3	2	2	2.053										-	-	-	Endoplasmic reticulum	
4504347	NP_000549.1	HBA1	3039	hemoglobin subunit alpha	3	14	2.181	1.65									Transport/cargo protein	Transport	Transporter activity	Cytoplasm	
4504349	NP_000509.1	HBB	3043	hemoglobin subunit beta	11	39	2.147	1.53									Transport/cargo protein	Transport	Transporter activity	Extracellular	
4885373	NP_005316.1	HIST1H1A	3024	histone H1.1					3	4	3.136		3	3	2.124		DNA binding protein	Regulation of nucleobase, nucleoside, nucleotide and nucleic acid metabolism	DNA binding	Nucleus	+
4885381	NP_005313.1	HIST1H1B	3009	histone H1.5	7	27	2.815	0.73	8	27	2.788		7	18	4.726	13.30	DNA binding protein	Regulation of nucleobase, nucleoside, nucleotide and nucleic acid metabolism	DNA binding	Nucleus	+
4885375	NP_005310.1	HIST1H1C	3006	histone H1.2	3	9	3.478	1.44	3	10	4.044	2.06	3	10	3.483	2.65	DNA binding protein	Regulation of nucleobase, nucleoside, nucleotide and nucleic acid metabolism	DNA binding	Nucleus	+
4885377	NP_005311.1	HIST1H1D	3007	histone H1.3	1	2	2.183		2	4	4.208		2	4	7.321	3.60	DNA binding protein	Regulation of nucleobase, nucleoside, nucleotide and nucleic acid metabolism	DNA binding	Nucleus	+
4885379	NP_005312.1	HIST1H1E	3008	histone H1.4					2	7	2.002	1.81	2	8	1.811	1.89	DNA binding protein	Regulation of nucleobase, nucleoside, nucleotide and nucleic acid metabolism	DNA binding	Nucleus	+
10800144	NP_066544.1	HIST1H2AJ	8331	histone cluster 1, H2aj	1	6	3.829		1	5	2.115		1	7	4.403	1.38	DNA binding protein	Regulation of nucleobase, nucleoside, nucleotide and nucleic acid metabolism	DNA binding	Nucleus	+
10800140	NP_066406.1	HIST1H2BB	3018	histone H2B type 1-B	2	3	3.463		2	3	1.755		2	3	2.666	2.59	DNA binding protein	Regulation of nucleobase, nucleoside, nucleotide and nucleic acid metabolism	DNA binding	Nucleus	+
10800138	NP_066407.1	HIST1H2BD	3017	histone H2B type 1-D	2	5	2.654		1	4	1.553		2	5	2.499	1.39	DNA binding protein	Chromosome organization and biogenesis (sensu Eukarya)	DNA binding	Nucleus	+
4504313	NP_003535.1	HIST1H4B	8366	histone H4	6	19	3.717		6	25	2.142	1.33	6	27	3.327	3.79	DNA binding protein	Regulation of nucleobase, nucleoside, nucleotide and nucleic acid metabolism	DNA binding	Nucleus	+
751368126	NP_001291434.1	HMG20A	10363	high mobility group protein 20A isoform b	2	2	0.404		2	2	0.546						Transcription regulatory protein	Regulation of nucleobase, nucleoside, nucleotide and nucleic acid metabolism	Transcription regulator activity	Nucleus	+
22208967	NP_665906.1	HMGA1	3159	high mobility group protein HMG-I/HMG-Y isoform a					3	13	2.201						DNA binding protein	Regulation of nucleobase, nucleoside, nucleotide and nucleic acid metabolism	DNA binding	Nucleus	+
4504425	NP_002119.1	HMGB1	3146	high mobility group protein B1	5	16	3.679	1.41	5	16	2.634	1.52	5	16	3.346	1.56	DNA binding protein	Regulation of nucleobase, nucleoside, nucleotide and nucleic acid metabolism	DNA binding	Nucleus	+
194688133	NP_001124160.1	HMGB2	3148	high mobility group protein B2	5	8	1.959	1.64	5	7	2.729	2.35	5	7	3.890	3.31	Transcription regulatory protein	Regulation of nucleobase, nucleoside, nucleotide and nucleic acid metabolism	Transcription regulator activity	Nucleus	+
48255933	NP_004956.5	HMGN1	3150	non-histone chromosomal protein HMG-14									4	9	1.940	1.48	Transcription regulatory protein	Regulation of nucleobase, nucleoside, nucleotide and nucleic acid metabolism	Transcription regulator activity	Nucleus	+
10835240	NP_006344.1	HMGN4	10473	high mobility group nucleosome-binding domain-containing protein 4					1	2	1.757	1.28					Transcription regulatory protein	Regulation of nucleobase, nucleoside, nucleotide and nucleic acid metabolism	Transcription regulator activity	Nucleus	+
5803036	NP_006796.1	HNRNPA0	10949	heterogeneous nuclear ribonucleoprotein A0	4	7	0.549						4	7	0.209	1.39	Ribonucleoprotein	Regulation of nucleobase, nucleoside, nucleotide and nucleic acid metabolism	RNA binding	Nucleus	+
4504445	NP_002127.1	HNRNPA1	3178	heterogeneous nuclear ribonucleoprotein A1 isoform a	7	16	0.287						7	16	0.165	1.52	RNA binding protein	Regulation of nucleobase, nucleoside, nucleotide and nucleic acid metabolism	RNA binding	Nucleus	+
14043072	NP_112533.1	HNRNPA2B1	3181	heterogeneous nuclear ribonucleoproteins A2/B1 isoform B1	14	36	0.226		11	28	0.649	1.37	12	29	0.173	1.39	RNA binding protein,Ribonucleoprotein	Regulation of nucleobase, nucleoside, nucleotide and nucleic acid metabolism	RNA binding,Transcription factor binding	Nucleus	+
34740329	NP_919223.1	HNRNPA3	220988	heterogeneous nuclear ribonucleoprotein A3	10	29	0.278		10	31	0.613		10	26	0.148		Ribonucleoprotein	Regulation of nucleobase, nucleoside, nucleotide and nucleic acid metabolism	RNA binding	Nucleus	+
55956921	NP_004490.2	HNRNPAB	3182	heterogeneous nuclear ribonucleoprotein A/B isoform b	4	11	0.353						3	9	0.146	2.03	Ribonucleoprotein	Regulation of nucleobase, nucleoside, nucleotide and nucleic acid metabolism	MRNA binding	Nucleolus	+
117190174	NP_004491.2	HNRNPC	3183	heterogeneous nuclear ribonucleoproteins C1/C2 isoform b	12	35	0.452	1.30					13	40	0.383	2.98	RNA binding protein	Regulation of nucleobase, nucleoside, nucleotide and nucleic acid metabolism	RNA binding	Nucleus	+

51477708	NP_001003810.1	HNRNPD	3184	heterogeneous nuclear ribonucleoprotein D0 isoform d	4	11	0.218	1.62					4	10	0.132	1.59	RNA binding protein	Regulation of nucleobase, nucleoside, nucleotide and nucleic acid metabolism	MRNA binding	Nucleus	+
332801090	NP_001193929.1	HNRNPDL	9987	heterogeneous nuclear ribonucleoprotein D-like isoform b	9	24	0.315						8	17	0.196		Ribonucleoprotein	Regulation of nucleobase, nucleoside, nucleotide and nucleic acid metabolism	RNA binding	Nucleus	+
14141159	NP_067676.2	HNRNPH3	3189	heterogeneous nuclear ribonucleoprotein H3 isoform b	4	8	0.504	1.41					4	7	0.267	1.35	Ribonucleoprotein	Regulation of nucleobase, nucleoside, nucleotide and nucleic acid metabolism	Ribonucleoprotein	Nucleus	+
14165435	NP_112552.1	HNRNPK	3190	heterogeneous nuclear ribonucleoprotein K isoform b	15	37	0.382						14	28	0.225	1.39	Ribonucleoprotein	Regulation of nucleobase, nucleoside, nucleotide and nucleic acid metabolism	Ribonucleoprotein	Nucleus	+
52632383	NP_001524.2	HNRNPL	3191	heterogeneous nuclear ribonucleoprotein L isoform a	11	26	0.345						9	17	0.195	1.46	Ribonucleoprotein	Regulation of nucleobase, nucleoside, nucleotide and nucleic acid metabolism	RNA binding	Nucleus	+
157412270	NP_112480.2	HNRNPM	4670	heterogeneous nuclear ribonucleoprotein M isoform b	8	13	0.504	1.34	9	15	0.548	1.42	6	9	0.674	1.72	Ribonucleoprotein	Regulation of nucleobase, nucleoside, nucleotide and nucleic acid metabolism	Ribonucleoprotein	Nucleus	+
5031755	NP_005817.1	HNRNPR	10236	heterogeneous nuclear ribonucleoprotein R isoform 2	16	50	0.392						16	41	0.216	1.61	RNA binding protein	Regulation of nucleobase, nucleoside, nucleotide and nucleic acid metabolism	RNA binding	Nucleus	+
14141161	NP_004492.2	HNRNPU	3192	heterogeneous nuclear ribonucleoprotein U isoform b	8	20	0.393	1.39					8	17	0.209	1.76	Ribonucleoprotein	Regulation of nucleobase, nucleoside, nucleotide and nucleic acid metabolism	RNA binding	Nucleus	+
118601081	NP_001073027.1	HNRNPUL2	221092	heterogeneous nuclear ribonucleoprotein U-like protein 2	15	23	0.408						14	23	0.244	0.64	RNA binding protein	Regulation of nucleobase, nucleoside, nucleotide and nucleic acid metabolism	RNA binding	Nucleus	+
56676330	NP_057371.2	HP1BP3	50809	heterochromatin protein 1-binding protein 3	13	33	1.652		14	41	2.025	1.84					DNA binding protein	Regulation of nucleobase, nucleoside, nucleotide and nucleic acid metabolism	DNA binding	Nucleus	+
13676857	NP_068814.2	HSPA2	3306	heat shock-related 70 kDa protein 2	2	2	0.322		2	2	0.341		2	2	0.449	0.53	Heat shock protein	Protein metabolism	Heat shock protein activity	Nucleus	
16507237	NP_005338.1	HSPA5	3309	78 kDa glucose-regulated protein precursor	14	22	0.640		16	24	0.605						Chaperone	Protein metabolism	Chaperone activity	Endoplasmic reticulum	
34419635	NP_002146.2	HSPA6	3310	heat shock 70 kDa protein 6	1	2	0.656	1.95	1	2	0.553	1.27					Heat shock protein	Protein metabolism	Heat shock protein activity	Nucleus	
31542947	NP_002147.2	HSPD1	3329	60 kDa heat shock protein, mitochondrial	3	4	1.850	1.42					2	3	1.491	1.41	Heat shock protein	Apoptosis,Protein metabolism,Signal transduction,Protein folding,Regulation of immune response	Heat shock protein activity	Mitochondrion	
4504523	NP_002148.1	HSPE1	3336	10 kDa heat shock protein, mitochondrial	1	2	2.034										Heat shock protein	Protein metabolism	Heat shock protein activity	Mitochondrion	
392513662	NP_001254738.1	ILF2	3608	interleukin enhancer-binding factor 2 isoform 2	5	11	0.505						7	15	0.326	1.33	Transcription factor	Regulation of nucleobase, nucleoside, nucleotide and nucleic acid metabolism	Transcription factor activity	Nucleus	+
212549553	NP_060090.2	ILF3	3609	interleukin enhancer-binding factor 3 isoform d	21	53	0.493	1.62					21	60	0.377	1.44	RNA binding protein,Transcription factor	Regulation of nucleobase, nucleoside, nucleotide and nucleic acid metabolism,Regulation of gene expression, epigenetic	Transcription factor activity,Transcription regulator activity	Nucleus	+
5730027	NP_006550.1	KHDRBS1	10657	KH domain-containing, RNA-binding, signal transduction-associated protein 1 isoform 1	4	10	0.282		4	12	0.468		4	12	0.277		RNA binding protein	Regulation of nucleobase, nucleoside, nucleotide and nucleic acid metabolism	RNA binding	Nucleus	+
189217895	NP_689901.2	KHDRBS2	202559	KH domain-containing, RNA-binding, signal transduction-associated protein 2	2	3	0.282	0.52	2	3	0.294	0.57	2	3	0.185	0.11	RNA binding protein	Regulation of nucleobase, nucleoside, nucleotide and nucleic acid metabolism	RNA binding	Nucleus	+
5730073	NP_006549.1	KHDRBS3	10656	KH domain-containing, RNA-binding, signal transduction-associated protein 3	6	11	0.316	0.65	6	11	0.657		5	8	0.200	0.67	RNA binding protein	Regulation of nucleobase, nucleoside, nucleotide and nucleic acid metabolism	RNA binding	Nucleus	+
119395750	NP_006112.3	KRT1	3848	keratin, type II cytoskeletal 1					4	6	2.249	0.74	4	6	1.936		Structural protein	Cell growth and/or maintenance	Structural molecule activity	Membrane	
264681410	NP_001161086.1	LEMD3	23592	inner nuclear membrane protein Man1 isoform 2	3	5	0.486	0.66					3	4	0.655	0.64	Integral membrane protein	Immune response	Nucleotide binding	Nucleus	
27436946	NP_733821.1	LMNA	4000	lamin isoform A					1	2	0.436	1.55					Structural protein	Cell growth and/or maintenance	Structural molecule activity	Nucleus	
5031877	NP_005564.1	LMNB1	4001	lamin-B1 isoform 1	21	45	1.846	2.56	22	42	1.943	3.59	24	50	3.019	3.58	Structural protein	Cell growth and/or maintenance	Structural molecule activity	Nucleus	+
388240801	NP_116126.3	LMNB2	84823	lamin-B2	23	46	0.596										Structural protein	Cell growth and/or maintenance	Structural molecule activity	Nucleus	
33946291	NP_079106.3	LPCAT1	79888	lysophosphatidylcholine acyltransferase 1	2	3	1.538	1.32	2	3	2.322	1.39					Enzyme: Acyltransferase	Cell organization and biogenesis	Acyltransferase activity	Endoplasmic reticulum	
312433998	NP_001186078.1	LTF	4057	lactotransferrin isoform 2									7	11	4.622	12.60	Transport/cargo protein	Transport	Transporter activity	Nucleus	
116812577	NP_057103.2	LUC7L2	51631	putative RNA-binding protein Luc7-like 2 isoform 1									4	4	1.724	1.83	Unclassified	Biological_process unknown	Molecular function unknown	Nucleolus	
153070260	NP_002347.5	MARCKS	4082	myristoylated alanine-rich C-kinase substrate	3	4	3.951	1.65	3	5	2.987	2.09					Cytoskeletal protein	Cell growth and/or maintenance	Structural constituent of cytoskeleton	Membrane	
62750354	NP_954659.1	MATR3	9782	matrin-3 isoform a	13	35	0.400		11	29	0.644		14	38	0.375	0.73	RNA binding protein	Regulation of nucleobase, nucleoside, nucleotide and nucleic acid metabolism	RNA binding	Nucleus	+
68509932	NP_001020263.1	MBP	4155	myelin basic protein isoform 4	5	31	0.373		5	30	0.404		5	32	0.400	0.20	Structural protein	Immune response,Neurogenesis	Structural constituent of myelin sheath	Cytoplasm	
538919685	NP_001269332.1	MDH2	4191	malate dehydrogenase, mitochondrial isoform 2 precursor									2	2	0.528		Enzyme: Dehydrogenase	Metabolism,Energy pathways	Catalytic activity	Mitochondrion	
160707950	NP_001104262.1	MECP2	4204	methyl-CpG-binding protein 2 isoform 2	16	40	0.359		14	35	0.608		16	39	0.456	0.77	DNA binding protein	Regulation of nucleobase, nucleoside, nucleotide and nucleic acid metabolism	DNA binding	Nucleus	+
89145417	NP_054752.3	METTL7A	25840	methyltransferase-like protein 7A precursor	2	3	2.118	1.87	1	2	1.764	1.91					Enzyme: Methyltransferase	Metabolism,Energy pathways	Methyltransferase activity	Nucleus	
50726968	NP_005917.2	MFAP1	4236	microfibrillar-associated protein 1									2	4	1.521		Extracellular matrix protein	Cell growth and/or maintenance	Extracellular matrix structural constituent	Extracellular	
390125204	NP_001254527.1	MGST1	4257	microsomal glutathione S-transferase 1 isoform c	1	2	1.702	3.10					2	3	1.965	1.41	Enzyme: Glutathione transferase	Xenobiotic metabolism,Metabolism,Energy pathways	Glutathione transferase activity	Endoplasmic reticulum	
747165394	NP_001290556.1	MLEC	9761	malectin isoform 2									1	2	1.722	1.43	Unclassified	Biological_process unknown	Molecular function unknown	Membrane	
225903430	NP_001139630.1	MOGS	7841	mannosyl-oligosaccharide glucosidase isoform 2					4	9	2.091						Enzyme: Glucosidase	Metabolism,Energy pathways	Glucosidase activity	Endoplasmic reticulum	
4557759	NP_000241.1	MPO	4353	myeloperoxidase precursor									5	6	4.077		Enzyme: Oxidoreductase	Metabolism,Energy pathways	Oxidoreductase activity	Extracellular	
25121993	NP_733839.1	MSI2	124540	RNA-binding protein Musashi homolog 2 isoform b					1	2	2.525	2.32	1	2	0.318	1.66	RNA binding protein	Regulation of nucleobase, nucleoside, nucleotide and nucleic acid metabolism	RNA binding	Cytoplasm	+
223555917	NP_848927.2	MTDH	92140	protein LYRIC	3	5	0.651		2	4	0.646		3	6	0.645	1.87	Unclassified	Biological_process unknown	Molecular function unknown	Endoplasmic reticulum	
667743700	NP_001288139.1	MYEF2	50804	myelin expression factor 2 isoform b	7	13	0.345		6	9	0.589	1.39	7	13	0.303		Transcription regulatory protein	Regulation of nucleobase, nucleoside, nucleotide and nucleic acid metabolism	Transcription regulator activity	Nucleus	+
5453740	NP_006462.1	MYL12A	10627	myosin regulatory light chain 12A isoform 1									1	2	1.663	2.25	Calcium binding protein	Cell communication,Signal transduction	Calcium ion binding	Cytoplasm	
68051721	NP_065843.3	NCEH1	57552	neutral cholesterol ester hydrolase 1 isoform b	3	7	0.500	0.39	3	7	0.528	0.34	3	7	0.590	0.30	Unclassified	Lipid degradation, Lipid metabolism	carboxylic ester hydrolase activity	Membrane	
55956788	NP_005372.2	NCL	4691	nucleolin					17	27	1.489		14	29	0.614	1.53	RNA binding protein	Regulation of nucleobase, nucleoside, nucleotide and nucleic acid metabolism	RNA binding	Nucleolus	+
93004092	NP_005587.2	NFIB	4781	nuclear factor 1 B-type isoform 3	3	4	1.762	2.16	3	4	1.823	2.31					Transcription factor	Regulation of nucleobase, nucleoside, nucleotide and nucleic acid metabolism	Transcription factor activity	Nucleus	+

402534522	NP_001257973.1	NFIX	4784	nuclear factor 1 X-type isoform 3	1	2	2.655	1.60	1	2	1.906	2.21	1	2	1.590	1.56	Transcription factor	Regulation of nucleobase, nucleoside, nucleotide and nucleic acid metabolism	Transcription factor activity	Nucleus	+
8923444	NP_060308.1	NHP2	55651	H/ACA ribonucleoprotein complex subunit 2 isoform a					4	6	1.812		3	5	1.510	1.30	Ribonucleoprotein	Ribosome biogenesis and assembly	RNA binding	Nucleolus	+
148596949	NP_004732.2	NOLC1	9221	nucleolar and coiled-body phosphoprotein 1 isoform 2					4	5	0.651		6	7	0.583	0.71	Transcription factor	Regulation of nucleobase, nucleoside, nucleotide and nucleic acid metabolism	Transcription factor activity	Nucleolus	+
8923942	NP_061118.1	NOP10	55505	H/ACA ribonucleoprotein complex subunit 3					1	3	1.819		1	3	1.774	1.63	Ribonucleoprotein	Regulation of nucleobase, nucleoside, nucleotide and nucleic acid metabolism	Ribonucleoprotein	Nucleolus	+
385198063	NP_001245239.1	NOP2	4839	probable 28S rRNA (cytosine(4447)-C(5))-methyltransferase isoform 4	3	3	1.558						3	3	2.666	1.73	Unclassified	Cell proliferation,Regulation of cell cycle	Molecular function unknown	Nucleolus	
56118310	NP_073568.2	NUCKS1	64710	nuclear ubiquitous casein and cyclin-dependent kinase substrate 1	2	2	2.337		3	3	3.108	1.34					DNA binding protein	Regulation of nucleobase, nucleoside, nucleotide and nucleic acid metabolism	DNA binding	Nucleus	+
557440899	NP_001273490.1	NUMA1	4926	nuclear mitotic apparatus protein 1 isoform 2					24	32	0.642	1.30					Structural protein	Cell growth and/or maintenance	Structural molecule activity	Nucleus	+
4758876	NP_004634.1	PABPN1	8106	polyadenylate-binding protein 2	3	4	0.488		3	3	0.617		3	4	0.567		RNA binding protein	Regulation of nucleobase, nucleoside, nucleotide and nucleic acid metabolism	RNA binding	Nucleus	+
156523968	NP_001609.2	PARP1	142	poly [ADP-ribose] polymerase 1	13	23	1.690		18	31	1.788						Enzyme: Ribosyltransferase	Protein metabolism	Catalytic activity	Nucleus	+
93102371	NP_060783.3	PBRM1	55193	protein polybromo-1					2	3	1.743	1.49	2	3	1.938	1.59	Regulatory/other subunit	Regulation of development	Chromatin binding	Nucleus	+
222352151	NP_006187.2	PCBP1	5093	poly(rC)-binding protein 1									2	3	1.643		RNA binding protein	Regulation of nucleobase, nucleoside, nucleotide and nucleic acid metabolism	RNA binding	Nucleus	+
193083114	NP_001122386.1	PCBP2	5094	poly(rC)-binding protein 2 isoform g	2	4	0.613	1.34									RNA binding protein	Regulation of nucleobase, nucleoside, nucleotide and nucleic acid metabolism	RNA binding,DNA binding	Nucleus	+
503774453	NP_055204.3	PELP1	27043	proline-, glutamic acid- and leucine-rich protein 1 isoform 1									2	2	1.652	0.66	Transcription regulatory protein	Regulation of nucleobase, nucleoside, nucleotide and nucleic acid metabolism	Transcription regulator activity	Nucleus	+
5729875	NP_006658.1	PGRMC1	10857	membrane-associated progesterone receptor component 1 isoform 1	3	6	0.644	0.42									Cell surface receptor	Cell communication,Signal transduction	Receptor activity	Membrane	
291621647	NP_006311.2	PGRMC2	10424	membrane-associated progesterone receptor component 2					3	5	1.598						Integral membrane protein	Cell communication,Signal transduction	Steroid hormone receptor activity	Membrane	
4505881	NP_000292.1	PLG	5340	plasminogen isoform 1 precursor	3	3	1.617						3	3	4.338		Protease	Protein metabolism	Peptidase activity	Extracellular	
192449447	NP_001122306.1	PLP1	5354	myelin proteolipid protein isoform 1					3	4	0.633	0.67					Structural protein	Cell growth and/or maintenance	Structural molecule activity	Membrane	
15451775	NP_150249.1	PML	5371	protein PML isoform 7					1	2	1.723	1.34	1	2	2.423	1.49	Transcription regulatory protein	Regulation of gene expression, epigenetic	Transporter activity,Transcription regulator activity	Nucleus	+
66529396	NP_001018171.1	PON2	5445	serum paraoxonase/arylesterase 2 isoform 2	4	10	3.150	2.03	4	10	4.699	2.36	4	11	2.579	1.84	Enzyme: Esterase	Metabolism,Energy pathways,Lipid metabolism	Hydrolase activity,Antioxidant activity	Membrane	
10863927	NP_066953.1	PPIA	5478	peptidyl-prolyl cis-trans isomerase A isoform 1	3	5	2.766		3	5	1.950	1.39					Enzyme: Isomerase	Peptide metabolism,Protein folding	Isomerase activity	Cytoplasm	
4758950	NP_000933.1	PPIB	5479	peptidyl-prolyl cis-trans isomerase B precursor	3	6	1.627	1.84					3	5	2.399	2.79	Chaperone	Protein metabolism	Chaperone activity	Endoplasmic reticulum	
42560244	NP_004783.2	PPIG	9360	peptidyl-prolyl cis-trans isomerase G	3	5	0.610	1.32									Enzyme: Isomerase	Metabolism,Energy pathways	Isomerase activity	Nucleus	
4506003	NP_002699.1	PPP1CA	5499	serine/threonine-protein phosphatase PP1-alpha catalytic subunit isoform 1					1	2	0.668						Serine/threonine phosphatase	Cell proliferation	Protein serine/threonine phosphatase activity	Cytoplasm	
32455260	NP_857634.1	PRDX5	25824	peroxiredoxin-5, mitochondrial isoform b precursor	1	3	1.997										Enzyme: Oxidoreductase	Metabolism,Energy pathways	Oxidoreductase activity	Mitochondrion	
126032350	NP_001075109.1	PRKDC	5591	DNA-dependent protein kinase catalytic subunit isoform 2									12	12	0.642	1.34	Serine/threonine kinase,DNA repair protein	Cell communication,Signal transduction	DNA repair protein,Protein serine/threonine kinase activity	Nucleus	+
24762236	NP_116253.2	PRPF38A	84950	pre-mRNA-splicing factor 38A									2	3	1.813	1.49	Unclassified	mRNA processing, mRNA splicing	protein binding	Nucleus	+
91208426	NP_006436.3	PRPF8	10594	pre-mRNA-processing-splicing factor 8	11	15	1.555										RNA binding protein	Regulation of nucleobase, nucleoside, nucleotide and nucleic acid metabolism	RNA binding	Nucleus	+
11386147	NP_002769.1	PSAP	5660	prosaposin isoform a preproprotein					2	3	0.641						Integral membrane protein	Cell communication,Signal transduction	Lipid binding	Extracellular	
19923653	NP_150091.2	PSIP1	11168	PC4 and SFRS1-interacting protein isoform 2	11	31	0.524		11	30	0.559	1.44	11	29	0.594	0.57	Transcription regulatory protein	Regulation of nucleobase, nucleoside, nucleotide and nucleic acid metabolism	Transcription regulator activity	Nucleus	+
109240550	NP_001035879.1	PSPC1	55269	paraspeckle component 1	8	13	0.261		8	14	0.315		8	14	0.423		RNA binding protein	Regulation of nucleobase, nucleoside, nucleotide and nucleic acid metabolism	RNA binding	Nucleus	+
14165466	NP_114368.1	PTBP1	5725	polypyrimidine tract-binding protein 1 isoform c	5	6	1.582	1.79	6	8	2.022	2.41					Ribonucleoprotein	Regulation of nucleobase, nucleoside, nucleotide and nucleic acid metabolism	Ribonucleoprotein	Nucleus	+
665821291	NP_001287919.1	PTBP2	58155	polypyrimidine tract-binding protein 2 isoform 7	1	2	1.633	0.63	1	2	3.041	0.69					RNA binding protein	Regulation of nucleobase, nucleoside, nucleotide and nucleic acid metabolism	RNA binding	Nucleus	+
5032007	NP_005850.1	PURA	5813	transcriptional activator protein Pur-alpha	1	2	0.331	0.74	2	3	0.594		2	2	0.147	0.62	Transcription factor	Regulation of nucleobase, nucleoside, nucleotide and nucleic acid metabolism	Transcription factor activity	Nucleus	+
5032027	NP_005601.1	RBBP4	5928	histone-binding protein RBBP4 isoform a	3	4	0.624	1.47	3	5	0.628	1.81					Transcription regulatory protein	Regulation of nucleobase, nucleoside, nucleotide and nucleic acid metabolism	Transcription regulator activity	Nucleus	+
8922328	NP_060517.1	RBM22	55696	pre-mRNA-splicing factor RBM22									1	2	1.493	1.45	Unclassified	mRNA processing, mRNA splicing, Transport	Metal-binding, RNA-binding	Nucleus	+
5032031	NP_005769.1	RBM5	10181	RNA-binding protein 5	1	2	0.629										RNA binding protein	Regulation of nucleobase, nucleoside, nucleotide and nucleic acid metabolism	RNA binding	Nucleus	+
4826972	NP_005096.1	RBM8A	9939	RNA-binding protein 8A									2	4	2.238	1.81	RNA binding protein	Regulation of nucleobase, nucleoside, nucleotide and nucleic acid metabolism	RNA binding	Nucleus	+
56699409	NP_002130.2	RBMX	27316	RNA-binding motif protein, X chromosome isoform 1	3	13	0.519	1.66					3	13	0.341	1.75	RNA binding protein	Regulation of nucleobase, nucleoside, nucleotide and nucleic acid metabolism	RNA binding	Nucleus	+
21361809	NP_062556.2	RBMXL1	494115	RNA binding motif protein, X-linked-like-1	3	6	0.595						3	6	0.346		-	mRNA processing, mRNA splicing	RNA binding	Nucleus	+
395132436	NP_001257419.1	RPL18	6141	60S ribosomal protein L18 isoform 2					2	2	0.620	1.32					Ribosomal subunit	Protein metabolism	Structural constituent of ribosome	Ribosome	
4506613	NP_000974.1	RPL22	6146	60S ribosomal protein L22 proprotein	1	2	0.597	1.52	1	2	0.611						Ribosomal subunit	Protein metabolism	Structural constituent of ribosome	Ribosome	
153252132	NP_001093163.1	RPL31	6160	60S ribosomal protein L31 isoform 3	2	3	0.532										Ribosomal subunit	Protein metabolism	Structural constituent of ribosome	Ribosome	
4506661	NP_000963.1	RPL7A	6130	60S ribosomal protein L7a	1	2	0.543	1.49	2	3	0.642	1.42					Ribosomal subunit	Protein metabolism	Structural constituent of ribosome	Cytoplasm	
4506671	NP_000995.1	RPLP2	6181	60S acidic ribosomal protein P2	1	2	0.633	2.03	2	3	0.673	1.70	1	2	0.575	1.87	Ribosomal subunit	Protein metabolism	Structural constituent of ribosome	Cytoplasm	
4506675	NP_002941.1	RPN1	6184	dolichyl-diphosphooligosaccharide--protein glycosyltransferase subunit 1 precursor					8	14	1.594						Ubiquitin proteasome system protein	Protein metabolism	Ubiquitin-specific protease activity	Nucleus	
5032051	NP_005608.1	RPS14	6208	40S ribosomal protein S14					2	3	0.577	1.77					Ribosomal subunit	Protein metabolism	Structural constituent of ribosome	Ribosome	
4506695	NP_001013.1	RPS19	6223	40S ribosomal protein S19	2	2	0.581	2.33	2	2	0.501	1.96					Ribosomal subunit	Protein metabolism	Structural constituent of ribosome	Nucleolus	
4506707	NP_001019.1	RPS25	6230	40S ribosomal protein S25					2	3	0.647						Ribosomal subunit	Protein metabolism	Structural constituent of ribosome	Ribosome	
386869506	NP_001247436.1	RPS3	6188	40S ribosomal protein S3 isoform 3					1	2	0.615	1.54					Ribosomal subunit	Protein metabolism	Structural constituent of ribosome	Cytoplasm	

118498359	NP_056474.2	RSL1D1	26156	ribosomal L1 domain-containing protein 1									3	6	1.876	1.57	Unclassified	Biological_process unknown	Molecular function unknown	Nucleolus	
24431933	NP_722550.1	RTN4	57142	reticulon-4 isoform B	1	2	2.322						1	2	2.053	0.36	Integral membrane protein	Regulation of cell growth	Protein binding	Endoplasmic reticulum	
55956779	NP_001007101.1	SCP2	6342	non-specific lipid-transfer protein isoform 4 proprotein	1	2	2.902	1.57									Membrane transport protein	Transport,Lipid metabolism	Auxiliary transport protein activity,Sterol transporter activity	Cytoplasm	
380837121	NP_004883.3	SEC22B	9554	vesicle-trafficking protein SEC22b precursor					2	3	0.555						Integral membrane protein	Cell communication,Signal transduction	Transporter activity	Endoplasmic reticulum	
4826998	NP_005057.1	SFPQ	6421	splicing factor, proline- and glutamine-rich	15	35	0.588						13	28	0.638	1.39	RNA binding protein	Regulation of nucleobase, nucleoside, nucleotide and nucleic acid metabolism	RNA binding	Nucleus	+
584277111	NP_001276868.1	SLC1A3	6507	excitatory amino acid transporter 1 isoform 4	2	5	5.262	1.29	2	6	2.238	2.19					Transport/cargo protein	Transport	Transporter activity	Membrane	
48255898	NP_620614.2	SMARCA2	6595	probable global transcription activator SNF2L2 isoform b	4	5	0.640		3	4	0.595						Transcription factor	Regulation of nucleobase, nucleoside, nucleotide and nucleic acid metabolism	Transcription factor activity	Nucleus	+
325651836	NP_003592.3	SMARCA5	8467	SWI/SNF-related matrix-associated actin-dependent regulator of chromatin subfamily A member 5									3	4	1.955	2.09	DNA binding protein	Regulation of nucleobase, nucleoside, nucleotide and nucleic acid metabolism	DNA binding	Nucleus	+
283806604	NP_001164554.1	SMPD4	55627	sphingomyelin phosphodiesterase 4 isoform 3	2	3	1.719						2	3	1.766		Integral membrane protein	Biological_process unknown	Molecular function unknown	Endoplasmic reticulum	
29568103	NP_003080.2	SNRNP70	6625	U1 small nuclear ribonucleoprotein 70 kDa isoform 1	7	11	0.631						6	9	0.638	1.29	RNA binding protein,Ribonucleoprotein	Regulation of nucleobase, nucleoside, nucleotide and nucleic acid metabolism	RNA binding,Ribonucleoprotein	Nucleus	+
4759156	NP_004587.1	SNRPA	6626	U1 small nuclear ribonucleoprotein A	2	3	0.569	1.30									RNA binding protein	Regulation of nucleobase, nucleoside, nucleotide and nucleic acid metabolism	RNA binding	Nucleus	+
4507127	NP_003084.1	SNRPC	6631	U1 small nuclear ribonucleoprotein C	3	3	0.627						2	2	0.618	1.45	Ribonucleoprotein	Regulation of nucleobase, nucleoside, nucleotide and nucleic acid metabolism	RNA binding	Nucleus	+
4759160	NP_004166.1	SNRPD3	6634	small nuclear ribonucleoprotein Sm D3	2	10	0.602	1.32	2	8	0.667	1.68					RNA binding protein	Regulation of nucleobase, nucleoside, nucleotide and nucleic acid metabolism	RNA binding	Nucleus	+
4507129	NP_003085.1	SNRPE	6635	small nuclear ribonucleoprotein E isoform 1					1	3	1.505	1.50					Ribonucleoprotein	Regulation of nucleobase, nucleoside, nucleotide and nucleic acid metabolism	Ribonucleoprotein	Nucleus	+
6912676	NP_036377.1	SNW1	22938	SNW domain-containing protein 1									1	2	1.619		Transcription regulatory protein	Regulation of nucleobase, nucleoside, nucleotide and nucleic acid metabolism	Transcription regulator activity	Nucleus	+
609876591	NP_001278340.1	SON	6651	protein SON isoform E									4	5	1.559	1.34	Transcription factor	Regulation of nucleobase, nucleoside, nucleotide and nucleic acid metabolism	Transcription factor activity	Nucleus	+
149999611	NP_003125.3	SRP14	6727	signal recognition particle 14 kDa protein					2	3	1.662		2	3	1.745		RNA binding protein	Protein metabolism	RNA binding	Cytoplasm	+
118572613	NP_057417.3	SRRM2	23524	serine/arginine repetitive matrix protein 2									7	8	0.550		RNA binding protein	Regulation of nucleobase, nucleoside, nucleotide and nucleic acid metabolism	RNA binding	Nucleus	+
5902076	NP_008855.1	SRSF1	6426	serine/arginine-rich splicing factor 1 isoform 1	11	37	0.667										RNA binding protein	Protein metabolism	RNA binding	Nucleus	+
86991438	NP_001034554.1	SRSF5	6430	serine/arginine-rich splicing factor 5	3	7	0.623										RNA binding protein	Regulation of nucleobase, nucleoside, nucleotide and nucleic acid metabolism	RNA binding	Nucleus	+
306482694	NP_001182375.1	SRSF7	6432	serine/arginine-rich splicing factor 7 isoform 2	3	6	0.644										RNA binding protein	Regulation of nucleobase, nucleoside, nucleotide and nucleic acid metabolism	RNA binding	Nucleus	+
4506903	NP_003760.1	SRSF9	8683	serine/arginine-rich splicing factor 9									9	13	1.719	1.40	RNA binding protein	Protein metabolism	RNA binding	Nucleus	+
4507241	NP_003137.1	SSRP1	6749	FACT complex subunit SSRP1	4	4	1.532										Transcription factor	Regulation of nucleobase, nucleoside, nucleotide and nucleic acid metabolism	Transcription factor activity	Nucleus	+
73760415	NP_001027392.1	STXBP1	6812	syntaxin-binding protein 1 isoform b	2	4	1.726	0.35					3	5	0.453	0.15	Transport/cargo protein	Neurotransmitter transport	Transporter activity	Membrane	
217330646	NP_006704.3	SUB1	10923	activated RNA polymerase II transcriptional coactivator p15	2	4	2.200		2	3	1.505		2	4	1.642	1.55	Transcription factor	Regulation of nucleobase, nucleoside, nucleotide and nucleic acid metabolism	Transcription factor activity	Nucleus	+
224282117	NP_055699.2	SUGP2	10147	SURP and G-patch domain-containing protein 2									3	3	0.638	1.60	RNA binding protein	Protein metabolism	RNA binding	Nucleus	+
31742498	NP_056189.1	SUN2	25777	SUN domain-containing protein 2 isoform b	3	6	1.568		4	7	1.523						Unclassified	Cell organization and biogenesis	Molecular function unknown	Membrane	
228008295	NP_001153147.1	SYNCRIP	10492	heterogeneous nuclear ribonucleoprotein Q isoform 4	9	15	0.383	1.95					9	15	0.261	1.66	RNA binding protein	Regulation of nucleobase, nucleoside, nucleotide and nucleic acid metabolism	RNA binding	Cytoplasm	+
631790805	NP_001278830.1	SYT1	6857	synaptotagmin-1 isoform 2					2	2	0.548	0.16	3	4	0.514	0.04	Calcium binding protein	Cell communication,Signal transduction	Calcium ion binding	Cytoplasm	
4507401	NP_003192.1	TFAM	7019	transcription factor A, mitochondrial isoform 1 precursor	6	8	1.735		5	6	1.860	1.32					Transcription factor	Regulation of nucleobase, nucleoside, nucleotide and nucleic acid metabolism	Transcription factor activity	Mitochondrion	+
125656165	NP_001075019.1	THOC2	57187	THO complex subunit 2	4	4	1.487	2.67	4	5	1.596	3.11					Transcription factor	Regulation of nucleobase, nucleoside, nucleotide and nucleic acid metabolism	Transcription factor activity	Nucleus	+
167234419	NP_005110.2	THRAP3	9967	thyroid hormone receptor-associated protein 3									4	9	0.582		Transcription regulatory protein	Regulation of nucleobase, nucleoside, nucleotide and nucleic acid metabolism	Transcription regulator activity	Nucleus	+
39725636	NP_059980.2	TMED9	54732	transmembrane emp24 domain-containing protein 9 precursor					1	3	0.662						Unclassified	Biological_process unknown	Molecular function unknown	Endoplasmic reticulum	
134152683	NP_001077059.1	TMEM214	54867	transmembrane protein 214 isoform 2	1	2	0.525										Unclassified	apoptotic process	Molecular function unknown	Endoplasmic reticulum	
13236587	NP_077310.1	TMEM43	79188	transmembrane protein 43	1	2	2.260		1	2	1.757						Integral membrane protein	Biological_process unknown	Molecular function unknown	Membrane	
808175976	NP_001294904.1	TMPO	7112	thymopoietin isoform delta	1	3	1.501										Peptide hormone	Regulation of cell cycle,Nuclear organization and biogenesis	Molecular function unknown	Mitochondrion	
151101292	NP_110382.3	TMX1	81542	thioredoxin-related transmembrane protein 1 precursor	2	3	2.917	1.59	2	3	2.209	1.91	2	3	1.511	2.83	Enzyme: Oxidoreductase	Metabolism,Energy pathways	Oxidoreductase activity	Endoplasmic reticulum	
11225260	NP_003277.1	TOP1	7150	DNA topoisomerase 1					6	8	1.682	1.40	7	9	1.934	1.60	Enzyme: Topoisomerase	Regulation of nucleobase, nucleoside, nucleotide and nucleic acid metabolism	DNA topoisomerase activity	Nucleus	+
19913408	NP_001059.2	TOP2B	7155	DNA topoisomerase 2-beta	17	24	1.546										Enzyme: Topoisomerase	Regulation of nucleobase, nucleoside, nucleotide and nucleic acid metabolism	DNA topoisomerase activity	Nucleus	+
39753957	NP_056417.2	TOR1AIP1	26092	torsin-1A-interacting protein 1 isoform 2	1	2	0.595	1.63	1	2	0.475	2.06					Unclassified	Biological_process unknown	Molecular function unknown	Nucleus	
5032189	NP_005648.1	TP53BP1	7158	tumor suppressor p53-binding protein 1 isoform 3	6	7	0.648										Transcription regulatory protein	Regulation of nucleobase, nucleoside, nucleotide and nucleic acid metabolism	Transcription regulator activity	Nucleus	+
114155142	NP_003283.2	TPR	7175	nucleoprotein TPR	9	10	0.661	1.88									Unclassified	Regulation of nucleobase, nucleoside, nucleotide and nucleic acid metabolism	Molecular function unknown	Nucleus	+
544346329	NP_001269688.1	TRA2A	29896	transformer-2 protein homolog alpha isoform 3	2	4	0.536	1.71									RNA binding protein	Regulation of nucleobase, nucleoside, nucleotide and nucleic acid metabolism	RNA binding	Nucleus	+
440309857	NP_001258978.1	TRAP1	10131	heat shock protein 75 kDa, mitochondrial isoform 2	1	2	1.791	1.44									Heat shock protein	Protein metabolism	Heat shock protein activity	Mitochondrion	

5032179	NP_005753.1	TRIM28	10155	transcription intermediary factor 1-beta					3	3	0.523	1.28					Transcription regulatory protein	Regulation of nucleobase, nucleoside, nucleotide and nucleic acid metabolism	Transcription regulator activity	Nucleus	+
733606247	NP_001290046.1	TUBA1C	84790	tubulin alpha-1C chain isoform b									4	7	0.556	6.50	Cytoskeletal protein	Cell growth and/or maintenance	Structural constituent of cytoskeleton	Cytoplasm	
29788768	NP_821080.1	TUBB2B	347733	tubulin beta-2B chain					2	5	0.537	1.47	2	4	0.404	1.74	Cytoskeletal protein	Cell growth and/or maintenance	Structural constituent of cytoskeleton	Cytoplasm	
60279268	NP_001012496.1	U2AF2	11338	splicing factor U2AF 65 kDa subunit isoform b									3	6	1.662	1.41	RNA binding protein	RNA metabolism	Spliceosomal catalysis	Nucleus	+
122937227	NP_001073884.1	U2SURP	23350	U2 snRNP-associated SURP motif-containing protein	2	2	1.489	1.88									RNA binding protein	RNA processing	RNA-binding	Nucleus	+
601984520	NP_066289.3	UBC	7316	polyubiquitin-C	1	2	2.545										Ubiquitin proteasome system protein	Protein metabolism	Ubiquitin-specific protease activity	Cytoplasm	
4759302	NP_004729.1	VAPB	9217	vesicle-associated membrane protein-associated protein B/C isoform 1	2	3	0.657	1.57	2	3	0.442	1.56	1	2	0.505	0.68	Membrane transport protein	Transport	Auxiliary transport protein activity	Membrane	
6005942	NP_009057.1	VCP	7415	transitional endoplasmic reticulum ATPase	2	2	0.657										ATPase	Metabolism,Energy pathways	ATPase activity	Nucleolus	
4507879	NP_003365.1	VDAC1	7416	voltage-dependent anion-selective channel protein 1	1	2	1.851		2	3	0.626						Voltage gated channel	Transport	Voltage-gated ion channel activity	Mitochondrion	
62414289	NP_003371.2	VIM	7431	vimentin	9	14	2.007	2.40					9	14	3.373	5.10	Cytoskeletal protein	Cell growth and/or maintenance	Structural constituent of cytoskeleton	Cytoplasm	
63025214	NP_001017980.1	VMA21	203547	vacuolar ATPase assembly integral membrane protein VMA21					1	2	0.558		1	2	0.549	1.33	Unclassified	vacuolar proton-transporting V-type ATPase complex assembly	Molecular function unknown	Cytoplasm	
7706501	NP_057396.1	WBP11	51729	WW domain-binding protein 11					1	2	1.952						RNA binding protein	Regulation of nucleobase, nucleoside, nucleotide and nucleic acid metabolism	RNA binding	Nucleus	+
10863945	NP_066964.1	XRCC5	7520	X-ray repair cross-complementing protein 5	4	7	0.661	1.72									DNA binding protein	Regulation of nucleobase, nucleoside, nucleotide and nucleic acid metabolism	DNA repair protein	Nucleus	+
34101286	NP_057191.2	ZFR	51663	zinc finger RNA-binding protein	3	3	0.401		4	4	0.671	1.43	4	4	0.592	0.74	Transcription regulatory protein	Regulation of nucleobase, nucleoside, nucleotide and nucleic acid metabolism	Transcription regulator activity	Nucleus	+
33946297	NP_892021.1	ZNF326	284695	DBIRD complex subunit ZNF326 isoform 1	2	3	0.512						2	3	0.438		DNA binding protein	Regulation of nucleobase, nucleoside, nucleotide and nucleic acid metabolism	DNA binding	Nucleolus	+
404501455	NP_001258218.1	ZNF512	84450	zinc finger protein 512 isoform d	5	6	1.634		5	7	1.570						Transcription regulatory protein	Regulation of nucleobase, nucleoside, nucleotide and nucleic acid metabolism	Transcription regulator activity	Nucleus	+

Supplementary Table S3: Differentially expressed proteins in Grade II astrocytoma. A total of 162 nuclear proteins (≥1.5 fold change) were identified after applying quality control filters as detailed in Methods section. The Table describes the total number of proteins and peptides identified in replicate mass spectrometry runs along with their quantity values.

Protein Accession	RefSeq id	Gene Symbol	Gene id	Protein name	ΣCoverage	Σ# Proteins	Σ# Unique Peptides	Σ# PSMs	115/114	115/114 Count	115/114 Variability [%]	Maximum Score	# AAs	MW [kDa]	calc. pI																			
Protein Group Accessions	A2	B2	Sequence	PSM Ambiguity	# Proteins	# Protein Groups	Modifications	Activation Type	ΔScore	ΔCn	Rank	Search Engine Rank	QuanResultID	Quan Info	Quan Usage	115/114	114	115	Peptides Matched	XCorr	Probability	# Missed Cleavages	Isolation Interference [%]	Ion Inject Time [ms]	Charge	m/z [Da]	MH+ [Da]	ΔM [ppm]	RT [min]	First Scan	Last Scan	MS Order	Ions Matched	Spectrum File
4502027	NP_000468.1	ALB	213	serum albumin preproprotein [Homo sapiens]	23.32	1	11	18	3.103	7;11	27.9	46.84	609	69.3	6.28																			
4502027		High	fQNALLVR	Unambiguous	1	1	N-Term(iTRAQ4plex)	HCD	1.0000	0.0000	1	1	10037	Unique	Used	3.405	4.349E3	1.614E4	360	3.27	0.00	0	37	300	2	552.83624	1104.66521	0.52	45.50	4492	4492	MS2	14/14	Glioma_Nuclear_prep_GradeII_repeat_4plex.raw
4502027		High	tcVADESAENcDk	Unambiguous	1	1	N-Term(iTRAQ4plex); C2(Methylthio); C11(Methylthio); K13(iTRAQ4plex)	HCD	1.0000	0.0000	1	1	9004	Unique	Used	3.404	1.109E3	4.114E3	16	4.91	0.00	0	18	300	2	882.86334	1764.71941	2.41	33.75	3386	3386	MS2	23/24	Glioma_Nuclear_prep_GradeII_repeat_4plex.raw
4502027		High	ylcENQDSISSk	Unambiguous	1	1	N-Term(iTRAQ4plex); C3(Methylthio); K12(iTRAQ4plex)	HCD	1.0000	0.0000	1	1	9609	Unique	Used	4.518	2.815E3	1.386E4	62	5.08	0.00	0	19	300	2	860.90955	1720.81182	-0.37	40.50	4033	4033	MS2	19/22	Glioma_Nuclear_prep_GradeII_repeat_4plex.raw
4502027	High		ylcENQDSISSk	Unambiguous	1	1	N-Term(iTRAQ4plex); C3(Methylthio); K12(iTRAQ4plex)	HCD	1.0000	0.0000	1	1	9141	Unique	Used	4.702	5.508E4	2.842E5	35	4.24	0.00	0	3	10	2	860.89545	1720.78362	-16.76	34.59	3694	3694	MS2	20/22	Glioma_Nuclear_prep_GradeII_4plex.raw
4502027		High	nEcFLQHk	Unambiguous	1	1	N-Term(iTRAQ4plex); C3(Methylthio); K8(iTRAQ4plex)	HCD	1.0000	0.0000	1	1	8637	Unique	Used	3.030	1.902E3	6.281E3	149	3.53	0.00	0	39	210	3	451.56073	1352.66764	-1.27	30.01	2997	2997	MS2	19/28	Glioma_Nuclear_prep_GradeII_repeat_4plex.raw
4502027	High		eTYGEMADccAk	Unambiguous	1	1	N-Term(iTRAQ4plex); C9(Methylthio); C10(Methylthio); K12(iTRAQ4plex)	HCD	1.0000	0.0000	1	1	10220	Unique	Used	2.306	2.593E4	6.563E4	6	4.36	0.00	0	43	47	2	850.82495	1700.64263	-16.17	42.49	4869	4869	MS2	19/22	Glioma_Nuclear_prep_GradeII_4plex.raw
4502027		High	lVNEVTEFAk	Unambiguous	1	1	N-Term(iTRAQ4plex); K10(iTRAQ4plex)	HCD	1.0000	0.0000	1	1	10924	Unique	Used	2.111	3.621E2	8.330E2	298	3.39	0.00	0	53	300	3	479.94406	1437.81763	-1.09	56.22	5449	5449	MS2	16/36	Glioma_Nuclear_prep_GradeII_repeat_4plex.raw
4502027		High	lVNEVTEFAk	Unambiguous	1	1	N-Term(iTRAQ4plex); K10(iTRAQ4plex)	HCD	1.0000	0.0000	1	1	10914	Unique	Used	4.244	3.308E3	1.530E4	278	4.44	0.00	0	24	300	2	719.41309	1437.81890	-0.21	56.11	5439	5439	MS2	18/18	Glioma_Nuclear_prep_GradeII_repeat_4plex.raw
4502027	High		lVNEVTEFAk	Unambiguous	1	1	N-Term(iTRAQ4plex); K10(iTRAQ4plex)	HCD	1.0000	0.0000	1	1	10132	Unique	Used	4.555	1.621E5	8.104E5	237	4.01	0.00	0	33	4	2	719.40125	1437.79521	-16.68	41.84	4774	4774	MS2	17/18	Glioma_Nuclear_prep_GradeII_4plex.raw
4502027	High		aVMDDFAAFVEk	Unambiguous	1	1	N-Term(iTRAQ4plex); K12(iTRAQ4plex)	HCD	1.0000	0.0000	1	1	11832	Unique	Used	3.175	3.708E3	1.292E4	200	4.20	0.00	0	32	31	3	544.28186	1630.83103	-4.87	55.48	6629	6629	MS2	19/44	Glioma_Nuclear_prep_GradeII_4plex.raw
4502027	High		vFDEFkPLVEEPQNLIk	Unambiguous	1	1	N-Term(iTRAQ4plex); K6(iTRAQ4plex); K17(iTRAQ4plex)	HCD	1.0000	0.0000	1	1	11833	Unique	Used	3.463	3.837E3	1.458E4	72	6.38	0.00	0	29	29	3	826.46887	2477.39206	-3.87	55.48	6630	6630	MS2	28/64	Glioma_Nuclear_prep_GradeII_4plex.raw
4502027		High	aEFAEVSk	Unambiguous	1	1	N-Term(iTRAQ4plex); K8(iTRAQ4plex)	HCD	1.0000	0.0000	1	1	8298	Unique	Used	2.297	2.163E4	5.414E4	284	3.19	0.00	0	60	196	2	584.82880	1168.65032	4.34	26.68	2640	2640	MS2	14/14	Glioma_Nuclear_prep_GradeII_repeat_4plex.raw
4502027		High	qTALVELVk	Unambiguous	1	1	N-Term(iTRAQ4plex); K9(iTRAQ4plex)	HCD	1.0000	0.0000	1	1	10214	Unique	Used	2.494	8.136E3	2.212E4	177	3.86	0.00	0	39	286	2	644.90710	1288.80693	-0.75	47.63	4683	4683	MS2	15/16	Glioma_Nuclear_prep_GradeII_repeat_4plex.raw
4502027		High	sLHTLFGDk	Unambiguous	1	1	N-Term(iTRAQ4plex); K9(iTRAQ4plex)	HCD	1.0000	0.0000	1	1	9348	Unique	Used	2.824	5.666E3	1.744E4	354	3.69	0.00	0	27	270	2	653.37341	1305.73955	-0.76	37.59	3750	3750	MS2	16/16	Glioma_Nuclear_prep_GradeII_repeat_4plex.raw
4502027		High	sLHTLFGDk	Unambiguous	1	1	N-Term(iTRAQ4plex); K9(iTRAQ4plex)	HCD	1.0000	0.0000	1	1	9326	Unique	Used	4.133	1.514E3	6.819E3	323	3.86	0.00	0	22	147	3	435.91788	1305.73908	-1.12	37.37	3727	3727	MS2	16/32	Glioma_Nuclear_prep_GradeII_repeat_4plex.raw
4502027	High		sLHTLFGDk	Unambiguous	1	1	N-Term(iTRAQ4plex); K9(iTRAQ4plex)	HCD	1.0000	0.0000	1	1	8652	Unique	Used	2.316	6.819E4	1.733E5	279	3.39	0.00	0	35	21	2	653.36340	1305.71953	-16.09	31.02	3161	3161	MS2	15/16	Glioma_Nuclear_prep_GradeII_4plex.raw
4502027	High		qTALVELVk	Unambiguous	1	1	N-Term(iTRAQ4plex); K9(iTRAQ4plex)	HCD	1.0000	0.0000	1	1	9521	Unique	Used	2.344	1.484E5	3.817E5	196	3.89	0.00	0	36	7	2	644.89648	1288.78569	-17.23	37.40	4110	4110	MS2	15/16	Glioma_Nuclear_prep_GradeII_4plex.raw
4502027		High	aVmDDFAAFVEk	Unambiguous	1	1	N-Term(iTRAQ4plex); M3(Oxidation); K12(iTRAQ4plex)	HCD	1.0000	0.0000	1	1	11406	Unique	Used	2.398	1.763E3	4.609E3	172	4.54	0.00	0	49	300	2	823.92010	1646.83293	-0.58	62.89	6020	6020	MS2	19/22	Glioma_Nuclear_prep_GradeII_repeat_4plex.raw
4502107	NP_001145.1	ANXA5	308	annexin A5 [Homo sapiens]	22.19	1	7	10	2.695	7;3	26.8	30.13	320	35.9	5.05																			
4502107		High	gTVTDFPGFDER	Unambiguous	1	1	N-Term(iTRAQ4plex)	HCD	1.0000	0.0000	1	1	10351	Unique	Used	2.831	3.187E3	9.836E3	135	4.34	0.00	0	25	241	2	742.86212	1484.71697	2.10	49.27	4830	4830	MS2	19/22	Glioma_Nuclear_prep_GradeII_repeat_4plex.raw
4502107	High		sEIDLFNIR	Unambiguous	1	1	N-Term(iTRAQ4plex)	HCD	1.0000	0.0000	1	1	10747	Unique	Used	2.169	5.126E4	1.220E5	310	3.45	0.00	0	52	28	2	625.83740	1250.66753	-14.90	46.51	5446	5446	MS2	15/16	Glioma_Nuclear_prep_GradeII_4plex.raw
4502107	High		gTVTDFPGFDER	Unambiguous	1	1	N-Term(iTRAQ4plex)	HCD	1.0000	0.0000	1	1	9737	Unique	Used	2.559	2.265E4	6.359E4	89	3.56	0.00	0	32	14	2	742.84894	1484.69060	-15.66	38.95	4344	4344	MS2	17/22	Glioma_Nuclear_prep_GradeII_4plex.raw
4502107	High		gLGTDEESILTLLTSR	Unambiguous	1	1	N-Term(iTRAQ4plex)	HCD	1.0000	0.0000	1	1	12885	Unique	Used	3.211	1.605E3	5.656E3	136	6.42	0.00	0	6	300	2	925.00800	1849.00871	2.79	67.74	7826	7826	MS2	26/30	Glioma_Nuclear_prep_GradeII_4plex.raw
4502107	High		gLGTDEESILTLLTSR	Unambiguous	1	1	N-Term(iTRAQ4plex)	HCD	1.0000	0.0000	1	1	12926	Unique	Used	3.386	1.040E3	3.865E3	153	6.32	0.00	0	22	300	2	925.00751	1849.00774	2.26	68.44	7881	7881	MS2	27/30	Glioma_Nuclear_prep_GradeII_4plex.raw
4502107	High		nFATSLYSMIk	Unambiguous	1	1	N-Term(iTRAQ4plex); K11(iTRAQ4plex)	HCD	1.0000	0.0000	1	1	11722	Unique	Used	1.732	1.910E4	3.630E4	304	4.39	0.00	0	63	111	2	781.92511	1562.84294	-3.94	54.52	6509	6509	MS2	18/20	Glioma_Nuclear_prep_GradeII_4plex.raw
4502107		High	dLLDDLk	Unambiguous	1	1	N-Term(iTRAQ4plex); K7(iTRAQ4plex)	HCD	1.0000	0.0000	1	1	10781	Unique	Used	3.433	2.903E3	1.086E4	330	3.08	0.00	0	39	300	2	560.32770	1119.64812	-1.68	54.43	5292	5292	MS2	12/12	Glioma_Nuclear_prep_GradeII_repeat_4plex.raw
4502107		High	lYDAYELk	Unambiguous	1	1	N-Term(iTRAQ4plex); K8(iTRAQ4plex)	HCD	1.0000	0.0000	1	1	10177	Unique	Used	2.853	4.678E3	1.454E4	292	3.34	0.00	0	32	300	2	651.86285	1302.71843	0.03	47.20	4644	4644	MS2	14/14	Glioma_Nuclear_prep_GradeII_repeat_4plex.raw
4502107	High		lYDAYELk	Unambiguous	1	1	N-Term(iTRAQ4plex); K8(iTRAQ4plex)	HCD	1.0000	0.0000	1	1	9546	Unique	Used	1.717	1.249E5	2.353E5	214	2.93	0.00	0	47	13	2	651.85236	1302.69744	-16.08	37.58	4137	4137	MS2	14/14	Glioma_Nuclear_prep_GradeII_4plex.raw
4502107	High		qEISAAFk	Unambiguous	1	1	N-Term(iTRAQ4plex); K8(iTRAQ4plex)	HCD	1.0000	0.0000	1	1	8223	Unique	Used	1.749	5.580E4	1.071E5	345	3.05	0.00	0	52	15	2	591.33167	1181.65605	-17.62	27.93	2694	2694	MS2	14/14	Glioma_Nuclear_prep_GradeII_4plex.raw
4502693	NP_001760.1	CD9	928	CD9 antigen [Homo sapiens]	4.39	1	1	2	1.604	1;1	16.1	9.33	228	25.4	7.15																			
4502693		High	kDVLETFTVk	Unambiguous	1	1	N-Term(iTRAQ4plex); K1(iTRAQ4plex); K10(iTRAQ4plex)	HCD	1.0000	0.0000	1	1	10133	Unique	Used	1.421	2.251E3	3.486E3	125	3.73	0.00	1	52	300	2	806.48920	1611.97112	1.80	46.68	4597	4597	MS2	18/18	Glioma_Nuclear_prep_GradeII_repeat_4plex.raw
4502693	High		kDVLETFTVk	Unambiguous	1	1	N-Term(iTRAQ4plex); K1(iTRAQ4plex); K10(iTRAQ4plex)	HCD	1.0000	0.0000	1	1	9360	Unique	Used	1.786	6.895E4	1.351E5	182	3.79	0.00	1	16	19	2	806.47284	1611.93840	-18.49	36.21	3934	3934	MS2	18/18	Glioma_Nuclear_prep_GradeII_4plex.raw
4503057	NP_001876.1	CRYAB	1410	alpha-crystallin B chain [Homo sapiens]	38.29	1	4	6	2.292	3;3	29.5	14.91	175	20.1	7.33																			
4503057		High	rPFFPFHSPSR	Unambiguous	1	1	N-Term(iTRAQ4plex)	HCD	1.0000	0.0000	1	1	9352	Unique	Used	1.390	1.450E2	2.197E2	209	3.87	0.00	0	12	220	4	380.45761	1518.80861	-0.05	37.64	3754	3754	MS2	18/60	Glioma_Nuclear_prep_GradeII_repeat_4plex.raw
4503057		High	vLGDVIEVHGk	Unambiguous	1	1	N-Term(iTRAQ4plex); K11(iTRAQ4plex)	HCD	1.0000	0.0000	1	1	9669	Unique	Used	2.680	2.890E3	8.441E3	187	4.38	0.00	0	20	185	3	485.29190	1453.86115	-0.40	41.18	4097	4097	MS2	26/40	Glioma_Nuclear_prep_GradeII_repeat_4plex.raw
4503057	High		vLGDVIEVHGk	Unambiguous	1	1	N-Term(iTRAQ4plex); K11(iTRAQ4plex)	HCD	1.0000	0.0000	1	1	8996	Unique	Used	1.939	1.276E5	2.716E5	241	4.16	0.00	0	34	10	2	727.42114	1453.83501	-18.39	33.49	3534	3534	MS2	19/20	Glioma_Nuclear_prep_GradeII_4plex.raw
4503057	High		vLGDVIEVHGk	Unambiguous	1	1	N-Term(iTRAQ4plex); K11(iTRAQ4plex)	HCD	1.0000	0.0000	1	1	8979	Unique	Used	2.645	2.612E4	7.582E4	166	3.52	0.00	0	21	6	3	485.28308	1453.83469	-18.60	33.37	3516	3516	MS2	25/40	Glioma_Nuclear_prep_GradeII_4plex.raw
4503057	High		eEkPAVTAAPk	Unambiguous	1	1	N-Term(iTRAQ4plex); K3(iTRAQ4plex); K11(iTRAQ4plex)	HCD	1.0000	0.0000	1	1	7359	Unique	Used	1.568	4.322E4	7.438E4	138	3.46	0.00	0	40	36	2	786.96252	1572.91777	-9.15	21.80	1758	1758	MS2	17/20	Glioma_Nuclear_prep_GradeII_4plex.raw
4503057		High	hFSPEELk	Unambiguous	1	1	N-Term(iTRAQ4plex); K8(iTRAQ4plex)	HCD	1.0000	0.0000	1	1	8425	Unique	Used	2.930	4.685E3	1.496E4	222	3.67	0.00	0	50	154	3	425.56900	1274.69245	-4.62	27.87	2773	2773	MS2	16/28	Glioma_Nuclear_prep_GradeII_repeat_4plex.raw
4503183	NP_001905.1	CYB5A	1528	cytochrome b5 isoform 2 [Homo sapiens]	43.88	3	3	4	1.899	1;3	35.4	15.63	98	11.3	5.14																			
4503183		High	fLEEHPGGEEVLR	Unambiguous	3	1	N-Term(iTRAQ4plex)	HCD	1.0000	0.0000	1	1	9640	Unique	Used	1.955	1.087E3	2.317E3	174	3.34	0.00	0	63	300	3	552.62006	1655.84562	-3.27	40.85	4066	4066	MS2	14/48	Glioma_Nuclear_prep_GradeII_repeat_4plex.raw
4503183		High	eQAGGDATENFEDVGHSTDAR	Unambiguous	3	1	N-Term(iTRAQ4plex)	HCD	1.0000	0.0000	1	1	8515	Unique	Used	3.033	2.595E2	8.579E2	28	8.40	0.00	0	2	190	3	784.01379	2350.02683	-1.39	28.78	2869	2869	MS2	39/80	Glioma_Nuclear_prep_GradeII_repeat_4plex.raw
4503183		High	yYTLEEIQk	Unambiguous	3	1	N-Term(iTRAQ4plex); K9(iTRAQ4plex)	HCD	1.0000	0.0000	1	1	10273	Unique	Used	1.842	4.587E3	9.211E3	286	3.89	0.00	0	45	300	2	737.90649	1474.80571	1.72	48.31	4745	4745	MS2	16/16	Glioma_Nuclear_prep_GradeII_repeat_4plex.raw
4503183	High		yYTLEEIQk	Unambiguous	3	1	N-Term(iTRAQ4plex); K9(iTRAQ4plex)	HCD	1.0000	0.0000	1	1	9609	Unique	Used	1.313	1.294E5	1.863E5	242	3.83	0.00	0	47	20	2	737.89368	1474.78008	-15.66	38.03	4205	4205	MS2	15/16	Glioma_Nuclear_prep_GradeII_4plex.raw
4503571	NP_001419.1	ENO1	2023	alpha-enolase isoform 1 [Homo sapiens]	7.83	2	3	4	2.610	2;2	20.2	9.21	434	47.1	7.39																			
4503571		High	yISPDQLADLYk	Unambiguous	2	1	N-Term(iTRAQ4plex); K12(iTRAQ4plex)	HCD	1.0000	0.0000	1	1	11864	Unique	Used	2.927	1.183E3	3.774E3	249	4.36	0.00	0	26	300	2	857.46912	1713.93096	0.45	69.67	6590	6590	MS2	19/22	Glioma_Nuclear_prep_GradeII_repeat_4plex.raw
4503571	High		yISPDQLADLYk	Unambiguous	2	1	N-Term(iTRAQ4plex); K12(iTRAQ4plex)	HCD	1.0000	0.0000	1	1	11079	Unique	Used	3.386	1.078E4	4.007E4	259	4.77	0.00	0	57	58	2	857.46625	1713.92522	-2.90	49.07	5807	5807	MS2	18/22	Glioma_Nuclear_prep_GradeII_4plex.raw
4503571	High		gNPTVEVDLFTSk	Unambiguous	1	1	N-Term(iTRAQ4plex); K13(iTRAQ4plex)	HCD	1.0000	0.0000	1	1	10169	Unique	Used	2.240	2.966E4	7.289E4	238	4.43	0.00	0	22	46	2	847.95081	1694.89433	-15.37	42.12	4814	4814	MS2	22/24	Glioma_Nuclear_prep_GradeII_4plex.raw
4503571		High	tIAPALVSk	Unambiguous	1	1	N-Term(iTRAQ4plex); K9(iTRAQ4plex)	HCD	1.0000	0.0000	1	1	8945	Unique	Used	2.293	1.060E4	2.650E4	173	3.45	0.00	0	54	213	2	594.38025	1187.75322	-5.89	33.06	3321	3321	MS2	14/16	Glioma_Nuclear_prep_GradeII_repeat_4plex.raw
4503979	NP_002046.1	GFAP	2670	glial fibrillary acidic protein isoform 1 [Homo sapiens]	38.66	3	17	25	3.054	10;15	28.5	54.61	432	49.8	5.52																			
4503979		High	qEADEATLAR	Unambiguous	3	1	N-Term(iTRAQ4plex)	HCD	1.0000	0.0000	1	1	7325	Unique	Used	1.552	9.848E3	1.666E4	168	3.09	0.00	0	16	167	2	624.31885	1247.63042	-3.56	17.62	1611	1611	MS2	13/18	Glioma_Nuclear_prep_GradeII_repeat_4plex.raw
4503979		High	lLEGEENR	Unambiguous	3	1	N-Term(iTRAQ4plex)	HCD	1.0000	0.0000	1	1	7815	Unique	Used	2.003	1.811E4	3.954E4	230	2.90	0.00	0	43	180	2	552.29462	1103.58196	0.54	22.00	2131	2131	MS2	12/14	Glioma_Nuclear_prep_GradeII_repeat_4plex.raw
4503979		High	lQDETNLR	Unambiguous	3	1	N-Term(iTRAQ4plex)	HCD	1.0000	0.0000	1	1	7925	Unique	Used	2.404	2.565E4	6.722E4	221	3.10	0.00	0	26	71	2	566.80737	1132.60747	-0.38	23.05	2247	2247	MS2	14/14	Glioma_Nuclear_prep_GradeII_repeat_4plex.raw
4503979		High	aLAAELNQLR	Unambiguous	3	1	N-Term(iTRAQ4plex)	HCD	1.0000	0.0000	1	1	10676	Unique	Used	2.611	5.326E3	1.515E4	241	3.67	0.00	0	25	300	2	621.86816	1242.72905	0.28	53.15	5178	5178	MS2	17/18	Glioma_Nuclear_prep_GradeII_repeat_4plex.raw
4503979		High	fADLTDAAAR	Unambiguous	3	1	N-Term(iTRAQ4plex)	HCD	1.0000	0.0000	1	1	9402	Unique	Used	2.657	4.014E4	1.162E5	273	3.53	0.00	0	16	48	2	597.81573	1194.62419	0.52	38.17	3807	3807	MS2	17/18	Glioma_Nuclear_prep_GradeII_repeat_4plex.raw
4503979		High	qEADEATLAR	Unambiguous	3	1	N-Term(iTRAQ4plex)	HCD	1.0000	0.0000	1	1	7280	Unique	Used	2.951	8.225E3	2.645E4	130	3.47	0.00	0	35	284	2	624.31091	1247.61455	-16.28	17.20	1564	1564	MS2	17/18	Glioma_Nuclear_prep_GradeII_repeat_4plex.raw
4503979		High	dNLAQDLATVR	Unambiguous	3	1	N-Term(iTRAQ4plex)	HCD	1.0000	0.0000	1	1	10376	Unique	Used	3.361	4.927E3	1.805E4	329	4.13	0.00	0	23	300	2	680.37109	1359.73491	0.00	49.55	4856	4856	MS2	19/20	Glioma_Nuclear_prep_GradeII_repeat_4plex.raw
4503979		High	lADVYQAELR	Unambiguous	3	1	N-Term(iTRAQ4plex)	HCD	1.0000	0.0000	1	1	10231	Unique	Used	3.646	5.526E3	2.196E4	271	3.61	0.00	0	16	300	2	661.36517	1321.72307	-0.15	47.82	4701	4701	MS2	17/18	Glioma_Nuclear_prep_GradeII_repeat_4plex.raw
4503979		High	lEAENNLAAYR	Unambiguous	3	1	N-Term(iTRAQ4plex)	HCD	1.0000	0.0000	1	1	9361	Unique	Used	4.348	8.523E3	4.039E4	203	4.18	0.00	0	13	146	2	704.37256	1407.73784	2.09	37.74	3764	3764	MS2	19/20	Glioma_Nuclear_prep_GradeII_repeat_4plex.raw
4503979	High		lTIPVQTFSNLQIR	Unambiguous	3	1	N-Term(iTRAQ4plex)	HCD	1.0000	0.0000	1	1	11641	Unique	Used	1.544	1.211E4	2.051E4	110	4.07	0.00	0	29	157	2	887.52228	1774.03728	1.63	53.77	6421	6421	MS2	16/26	Glioma_Nuclear_prep_GradeII_4plex.raw
4503979	High		aLAAELNQLR	Unambiguous	3	1	N-Term(iTRAQ4plex)	HCD	1.0000	0.0000	1	1	9896	Unique	Used	2.132	1.054E5	2.465E5	249	3.35	0.00	0	33	11	2	621.85791	1242.70854	-16.22	40.11	4517	4517	MS2	17/18	Glioma_Nuclear_prep_GradeII_4plex.raw
4503979	High		eAASYQEALAR	Unambiguous	3	1	N-Term(iTRAQ4plex)	HCD	1.0000	0.0000	1	1	8189	Unique	Used	2.773	2.555E5	7.774E5	174	3.91	0.00	0	32	4	2	676.83807	1352.66887	-17.62	27.70	2658	2658	MS2	17/20	Glioma_Nuclear_prep_GradeII_4plex.raw
4503979	High		lADVYQAELR	Unambiguous	3	1	N-Term(iTRAQ4plex)	HCD	1.0000	0.0000	1	1	9628	Unique	Used	3.169	1.128E5	3.924E5	268	3.40	0.00	0	22	10	2	661.35413	1321.70098	-16.87	38.17	4226	4226	MS2	17/18	Glioma_Nuclear_prep_GradeII_4plex.raw
4503979	High		lEAENNLAAYR	Unambiguous	3	1	N-Term(iTRAQ4plex)	HCD	1.0000	0.0000	1	1	8902	Unique	Used	3.463	1.493E5	5.673E5	196	3.41	0.00	0	39	12	2	704.35925	1407.71123	-16.82	32.81	3432	3432	MS2	17/20	Glioma_Nuclear_prep_GradeII_4plex.raw
4503979	High		dNLAQDLATVR	Unambiguous	3	1	N-Term(iTRAQ4plex)	HCD	1.0000	0.0000	1	1	9713	Unique	Used	4.121	1.272E5	5.753E5	276	3.91	0.00	0	22	6	2	680.36011	1359.71294	-16.16	38.78	4318	4318	MS2	18/20	Glioma_Nuclear_prep_GradeII_4plex.raw
4503979		High	qLQSLTcDLESLR	Unambiguous	3	1	N-Term(iTRAQ4plex); C7(Methylthio)	HCD	1.0000	0.0000	1	1	12342	Unique	Used	3.148	6.196E2	2.126E3	169	3.56	0.00	0	18	300	2	848.43103	1695.85478	1.25	80.48	7420	7420	MS2	16/24	Glioma_Nuclear_prep_GradeII_repeat_4plex.raw
4503979	High		qLQSLTcDLESLR	Unambiguous	3	1	N-Term(iTRAQ4plex); C7(Methylthio)	HCD	1.0000	0.0000	1	1	11894	Unique	Used	4.061	2.868E4	1.278E5	204	4.74	0.00	0	6	50	2	848.43439	1695.86150	5.21	56.00	6696	6696	MS2	20/24	Glioma_Nuclear_prep_GradeII_4plex.raw
4503979	High		kIESLEEEIR	Unambiguous	3	1	N-Term(iTRAQ4plex); K1(iTRAQ4plex)	HCD	1.0000	0.0000	1	1	9124	Unique	Used	3.955	8.576E4	3.722E5	230	3.36	0.00	1	9	6	2	767.42664	1533.84599	-17.41	34.47	3676	3676	MS2	18/18	Glioma_Nuclear_prep_GradeII_4plex.raw
4503979		High	hLQEYQDLLNVk	Unambiguous	3	1	N-Term(iTRAQ4plex); K12(iTRAQ4plex)	HCD	1.0000	0.0000	1	1	10576	Unique	Used	2.600	2.188E3	6.198E3	197	5.37	0.00	0	38	300	2	894.49872	1787.99016	0.40	51.94	5070	5070	MS2	22/22	Glioma_Nuclear_prep_GradeII_repeat_4plex.raw
4503979	High		hLQEYQDLLNVk	Unambiguous	3	1	N-Term(iTRAQ4plex); K12(iTRAQ4plex)	HCD	1.0000	0.0000	1	1	9750	Unique	Used	3.680	6.902E4	2.787E5	192	5.26	0.00	0	13	15	2	894.48425	1787.96123	-15.78	39.04	4358	4358	MS2	22/22	Glioma_Nuclear_prep_GradeII_4plex.raw
4503979		High	fASYIEk	Unambiguous	3	1	N-Term(iTRAQ4plex); K7(iTRAQ4plex)	HCD	1.0000	0.0000	1	1	9591	Unique	Used	4.889	2.108E4	1.123E5	158	2.97	0.00	0	19	66	2	573.32288	1145.63848	-5.26	40.31	4014	4014	MS2	12/12	Glioma_Nuclear_prep_GradeII_repeat_4plex.raw
4503979		High	sVSEGHLk	Unambiguous	1	1	N-Term(iTRAQ4plex); K8(iTRAQ4plex)	HCD	1.0000	0.0000	1	1	6864	Unique	Used	2.401	1.054E4	2.759E4	249	2.97	0.00	0	21	93	2	572.82861	1144.64995	-5.69	13.56	1124	1124	MS2	14/14	Glioma_Nuclear_prep_GradeII_repeat_4plex.raw
4503979		High	lEEEGQSLk	Unambiguous	3	1	N-Term(iTRAQ4plex); K9(iTRAQ4plex)	HCD	1.0000	0.0000	1	1	8263	Unique	Used	2.103	1.043E4	2.390E4	310	3.06	0.00	0	51	232	2	660.86633	1320.72539	0.33	26.32	2603	2603	MS2	16/16	Glioma_Nuclear_prep_GradeII_repeat_4plex.raw
4503979		High	lEEEGQSLkDEMAR	Unambiguous	3	1	N-Term(iTRAQ4plex); K9(iTRAQ4plex)	HCD	1.0000	0.0000	1	1	10102	Unique	Used	3.054	8.163E2	2.717E3	175	5.00	0.00	1	4	300	2	961.99164	1922.97600	1.43	46.32	4564	4564	MS2	24/26	Glioma_Nuclear_prep_GradeII_repeat_4plex.raw
4503979	High		lEEEGQSLkDEMAR	Unambiguous	3	1	N-Term(iTRAQ4plex); K9(iTRAQ4plex)	HCD	1.0000	0.0000	1	1	9427	Unique	Used	3.101	1.981E4	6.741E4	119	4.96	0.00	1	20	28	2	961.97290	1922.93852	-18.06	36.70	4007	4007	MS2	23/26	Glioma_Nuclear_prep_GradeII_4plex.raw
4504253	NP_002096.1	H2AFX	3014	histone H2AX [Homo sapiens]	33.57	4	2	2	0.431	1;1	15.6	17.14	143	15.1	10.74																			
4504253		High	kTSATVGPk	Unambiguous	1	1	N-Term(iTRAQ4plex); K1(iTRAQ4plex); K9(iTRAQ4plex)	HCD	1.0000	0.0000	1	1	6660	Unique	Used	0.479	1.622E4	8.470E3	132	4.90	0.00	1	52	71	3	440.94336	1320.81552	-4.25	11.75	910	910	MS2	18/32	Glioma_Nuclear_prep_GradeII_repeat_4plex.raw
4504253	High		lLGGVTIAQGGVLPNIQAVLLPk	Unambiguous	3	1	N-Term(iTRAQ4plex); K23(iTRAQ4plex)	HCD	1.0000	0.0000	1	1	13006	Unique	Used	0.384	2.726E2	1.149E2	5	6.52	0.00	0	14	300	3	853.86865	2559.59140	2.85	69.98	8000	8000	MS2	27/88	Glioma_Nuclear_prep_GradeII_4plex.raw
4504255	NP_002097.1	H2AFZ	3015	histone H2A.Z [Homo sapiens]	25.78	6	2	6	1.998	3;3	26.9	24.17	128	13.5	10.58																			
4504255		High	gDEELDSLIk	Unambiguous	5	1	N-Term(iTRAQ4plex); K10(iTRAQ4plex)	HCD	1.0000	0.0000	1	1	10297	Unique	Used	1.861	5.113E3	1.037E4	260	4.50	0.00	0	19	300	2	703.88446	1406.76164	-0.07	48.63	4772	4772	MS2	18/18	Glioma_Nuclear_prep_GradeII_repeat_4plex.raw
4504255	High		gDEELDSLIk	Unambiguous	5	1	N-Term(iTRAQ4plex); K10(iTRAQ4plex)	HCD	1.0000	0.0000	1	1	9666	Unique	Used	1.345	1.493E5	2.204E5	236	3.83	0.00	0	34	15	2	703.87286	1406.73845	-16.56	38.44	4267	4267	MS2	18/18	Glioma_Nuclear_prep_GradeII_4plex.raw
4504255		High	aTIAGGGVIPHIHk	Unambiguous	4	1	N-Term(iTRAQ4plex); K14(iTRAQ4plex)	HCD	1.0000	0.0000	1	1	9057	Unique	Used	1.914	2.108E3	4.397E3	108	5.89	0.00	0	35	300	2	830.00323	1658.99919	2.84	34.39	3443	3443	MS2	25/26	Glioma_Nuclear_prep_GradeII_repeat_4plex.raw
4504255		High	aTIAGGGVIPHIHk	Unambiguous	4	1	N-Term(iTRAQ4plex); K14(iTRAQ4plex)	HCD	1.0000	0.0000	1	1	9038	Unique	Used	2.082	4.776E2	1.084E3	127	5.07	0.00	0	54	300	4	415.50314	1658.99074	-2.26	34.17	3423	3423	MS2	29/78	Glioma_Nuclear_prep_GradeII_repeat_4plex.raw
4504255	High		aTIAGGGVIPHIHk	Unambiguous	4	1	N-Term(iTRAQ4plex); K14(iTRAQ4plex)	HCD	1.0000	0.0000	1	1	8448	Unique	Used	2.446	4.939E4	1.326E5	165	5.62	0.00	0	4	8	2	829.98712	1658.96697	-16.59	29.53	2938	2938	MS2	25/26	Glioma_Nuclear_prep_GradeII_4plex.raw
4504255	High		aTIAGGGVIPHIHk	Unambiguous	4	1	N-Term(iTRAQ4plex); K14(iTRAQ4plex)	HCD	1.0000	0.0000	1	1	8440	Unique	Used	3.008	2.192E4	7.234E4	177	4.27	0.00	0	8	1	4	415.49701	1658.96621	-17.05	29.48	2930	2930	MS2	31/78	Glioma_Nuclear_prep_GradeII_4plex.raw
4504313	NP_003535.1	HIST1H4B	8366	histone H4 [Homo sapiens]	51.46	1	6	19	3.717	8;11	38.8	68.81	103	11.4	11.36																			
4504313		High	iSGLIYEETR	Unambiguous	1	1	N-Term(iTRAQ4plex)	HCD	1.0000	0.0000	1	1	10478	Unique	Used	3.717	4.199E3	1.701E4	366	3.71	0.00	0	41	300	2	662.86560	1324.72392	0.75	50.76	4964	4964	MS2	17/18	Glioma_Nuclear_prep_GradeII_repeat_4plex.raw
4504313		High	vFLENVIR	Unambiguous	1	1	N-Term(iTRAQ4plex)	HCD	1.0000	0.0000	1	1	11126	Unique	Used	5.509	2.166E3	1.301E4	340	2.84	0.00	0	29	300	2	567.34338	1133.67949	-0.41	58.82	5676	5676	MS2	13/14	Glioma_Nuclear_prep_GradeII_repeat_4plex.raw
4504313		High	iSGLIYEETR	Unambiguous	1	1	N-Term(iTRAQ4plex)	HCD	1.0000	0.0000	1	1	10555	Unique	Used	5.843	1.120E4	7.133E4	274	3.66	0.00	0	8	64	2	662.86560	1324.72392	0.75	51.70	5048	5048	MS2	17/18	Glioma_Nuclear_prep_GradeII_repeat_4plex.raw
4504313	High		iSGLIYEETR	Unambiguous	1	1	N-Term(iTRAQ4plex)	HCD	1.0000	0.0000	1	1	9793	Unique	Used	3.348	2.742E5	1.007E6	286	3.66	0.00	0	23	4	2	662.85443	1324.70159	-16.12	39.35	4405	4405	MS2	17/18	Glioma_Nuclear_prep_GradeII_4plex.raw
4504313	High		kTVTAMDVVYALk	Unambiguous	1	1	N-Term(iTRAQ4plex); K1(iTRAQ4plex); K13(iTRAQ4plex)	HCD	1.0000	0.0000	1	1	10700	Unique	Used	2.559	4.159E4	1.168E5	147	5.63	0.00	1	28	22	2	936.04224	1871.07720	-14.15	46.16	5395	5395	MS2	23/24	Glioma_Nuclear_prep_GradeII_4plex.raw
4504313		High	dAVTYTEHAk	Unambiguous	1	1	N-Term(iTRAQ4plex); K10(iTRAQ4plex)	HCD	1.0000	0.0000	1	1	7329	Unique	Used	6.968	1.667E4	1.266E5	223	4.68	0.00	0	8	31	2	711.87164	1422.73601	-7.54	17.66	1616	1616	MS2	18/18	Glioma_Nuclear_prep_GradeII_repeat_4plex.raw
4504313		High	dAVTYTEHAk	Unambiguous	1	1	N-Term(iTRAQ4plex); K10(iTRAQ4plex)	HCD	1.0000	0.0000	1	1	7327	Unique	Used	7.309	2.058E4	1.639E5	211	4.29	0.00	0	9	5	3	474.91681	1422.73587	-7.64	17.65	1614	1614	MS2	24/36	Glioma_Nuclear_prep_GradeII_repeat_4plex.raw
4504313	High		dAVTYTEHAk	Unambiguous	1	1	N-Term(iTRAQ4plex); K10(iTRAQ4plex)	HCD	1.0000	0.0000	1	1	7400	Unique	Used	5.495	4.602E4	2.775E5	229	4.71	0.00	0	11	11	2	711.87225	1422.73723	-6.68	22.11	1803	1803	MS2	18/18	Glioma_Nuclear_prep_GradeII_4plex.raw
4504313		High	tVTAMDVVYALk	Unambiguous	1	1	N-Term(iTRAQ4plex); K12(iTRAQ4plex)	HCD	1.0000	0.0000	1	1	12257	Unique	Used	2.408	1.625E3	4.264E3	232	5.16	0.00	0	40	300	2	799.95746	1598.90764	0.63	77.75	7221	7221	MS2	21/22	Glioma_Nuclear_prep_GradeII_repeat_4plex.raw
4504313	High		tVTAMDVVYALk	Unambiguous	1	1	N-Term(iTRAQ4plex); K12(iTRAQ4plex)	HCD	1.0000	0.0000	1	1	11685	Unique	Used	4.172	3.890E4	1.781E5	234	6.00	0.00	0	22	53	2	799.95740	1598.90752	0.55	54.19	6469	6469	MS2	21/22	Glioma_Nuclear_prep_GradeII_4plex.raw
4504313	High		tVTAMDVVYALk	Unambiguous	1	1	N-Term(iTRAQ4plex); K12(iTRAQ4plex)	HCD	1.0000	0.0000	1	1	11694	Unique	Used	5.236	6.707E3	3.853E4	251	4.10	0.00	0	30	9	3	533.63824	1598.90018	-4.04	54.27	6479	6479	MS2	19/44	Glioma_Nuclear_prep_GradeII_4plex.raw
4504313		High	dNIQGITkPAIR	Unambiguous	1	1	N-Term(iTRAQ4plex); K8(iTRAQ4plex)	HCD	1.0000	0.0000	1	1	8757	Unique	Used	2.379	3.192E3	8.275E3	119	3.90	0.00	0	25	296	2	807.47198	1613.93669	-13.06	31.13	3123	3123	MS2	16/22	Glioma_Nuclear_prep_GradeII_repeat_4plex.raw
4504313	High		dNIQGITkPAIR	Unambiguous	1	1	N-Term(iTRAQ4plex); K8(iTRAQ4plex)	HCD	1.0000	0.0000	1	1	8312	Unique	Used	2.503	8.531E4	2.343E5	205	3.85	0.00	0	21	7	2	807.46869	1613.93010	-17.14	28.57	2791	2791	MS2	17/22	Glioma_Nuclear_prep_GradeII_4plex.raw
4504313		High	tVTAmDVVYALk	Unambiguous	1	1	N-Term(iTRAQ4plex); M5(Oxidation); K12(iTRAQ4plex)	HCD	1.0000	0.0000	1	1	11439	Unique	Used	2.179	7.399E2	1.757E3	220	4.84	0.00	0	11	217	3	538.97186	1614.90104	-0.32	63.34	6059	6059	MS2	21/44	Glioma_Nuclear_prep_GradeII_repeat_4plex.raw
4504313		High	tVTAmDVVYALk	Unambiguous	1	1	N-Term(iTRAQ4plex); M5(Oxidation); K12(iTRAQ4plex)	HCD	1.0000	0.0000	1	1	11361	Unique	Used	2.751	2.945E2	8.831E2	216	4.25	0.00	0	22	300	3	538.97205	1614.90158	0.02	62.27	5967	5967	MS2	20/44	Glioma_Nuclear_prep_GradeII_repeat_4plex.raw
4504313		High	tVTAmDVVYALk	Unambiguous	1	1	N-Term(iTRAQ4plex); M5(Oxidation); K12(iTRAQ4plex)	HCD	1.0000	0.0000	1	1	11356	Unique	Used	3.485	1.371E3	5.208E3	210	4.84	0.00	0	18	300	2	807.95477	1614.90227	0.44	62.20	5961	5961	MS2	22/22	Glioma_Nuclear_prep_GradeII_repeat_4plex.raw
4504313		High	tVTAmDVVYALk	Unambiguous	1	1	N-Term(iTRAQ4plex); M5(Oxidation); K12(iTRAQ4plex)	HCD	1.0000	0.0000	1	1	11434	Unique	Used	4.975	2.367E3	1.283E4	210	5.11	0.00	0	23	300	2	807.95465	1614.90203	0.29	63.27	6053	6053	MS2	20/22	Glioma_Nuclear_prep_GradeII_repeat_4plex.raw
4504313	High		tVTAmDVVYALk	Unambiguous	1	1	N-Term(iTRAQ4plex); M5(Oxidation); K12(iTRAQ4plex)	HCD	1.0000	0.0000	1	1	10515	Unique	Used	3.019	4.122E4	1.365E5	223	4.93	0.00	0	32	32	2	807.94171	1614.87615	-15.73	44.76	5193	5193	MS2	20/22	Glioma_Nuclear_prep_GradeII_4plex.raw
4504313	High		tVTAmDVVYALk	Unambiguous	1	1	N-Term(iTRAQ4plex); M5(Oxidation); K12(iTRAQ4plex)	HCD	1.0000	0.0000	1	1	10606	Unique	Used	4.790	6.613E4	3.476E5	226	5.01	0.00	0	15	9	2	807.94202	1614.87676	-15.35	45.44	5292	5292	MS2	20/22	Glioma_Nuclear_prep_GradeII_4plex.raw
4504347	NP_000549.1	HBA1	3039	hemoglobin subunit alpha [Homo sapiens]	48.59	1	3	14	2.181	6;8	18.9	35.91	142	15.2	8.68																			
4504347		High	vGAHAGEYGAEALER	Unambiguous	1	1	N-Term(iTRAQ4plex)	HCD	1.0000	0.0000	1	1	8510	Unique	Used	1.473	5.521E3	8.866E3	136	4.11	0.00	0	21	50	3	558.61511	1673.83078	-3.38	28.74	2864	2864	MS2	27/56	Glioma_Nuclear_prep_GradeII_repeat_4plex.raw
4504347		High	vGAHAGEYGAEALER	Unambiguous	1	1	N-Term(iTRAQ4plex)	HCD	1.0000	0.0000	1	1	8514	Unique	Used	2.064	5.559E3	1.251E4	157	5.88	0.00	0	4	196	2	837.42035	1673.83342	-1.80	28.77	2868	2868	MS2	23/28	Glioma_Nuclear_prep_GradeII_repeat_4plex.raw
4504347	High		vGAHAGEYGAEALER	Unambiguous	1	1	N-Term(iTRAQ4plex)	HCD	1.0000	0.0000	1	1	8201	Unique	Used	1.593	1.166E5	2.038E5	105	4.57	0.00	0	14	1	3	558.60712	1673.80680	-17.71	27.78	2671	2671	MS2	28/56	Glioma_Nuclear_prep_GradeII_4plex.raw
4504347	High		vGAHAGEYGAEALER	Unambiguous	1	1	N-Term(iTRAQ4plex)	HCD	1.0000	0.0000	1	1	8196	Unique	Used	2.373	1.215E5	3.164E5	127	5.51	0.00	0	5	10	2	837.40851	1673.80974	-15.95	27.74	2665	2665	MS2	21/28	Glioma_Nuclear_prep_GradeII_4plex.raw
4504347		High	tYFPHFDLSHGSAQVk	Unambiguous	1	1	N-Term(iTRAQ4plex); K16(iTRAQ4plex)	HCD	1.0000	0.0000	1	1	10556	Unique	Used	2.052	2.312E3	5.171E3	159	6.14	0.00	0	23	129	3	708.03796	2122.09934	1.56	51.71	5049	5049	MS2	35/60	Glioma_Nuclear_prep_GradeII_repeat_4plex.raw
4504347		High	tYFPHFDLSHGSAQVk	Unambiguous	1	1	N-Term(iTRAQ4plex); K16(iTRAQ4plex)	HCD	1.0000	0.0000	1	1	10563	Unique	Used	2.332	1.408E3	3.579E3	196	5.70	0.00	0	0	300	2	1061.55090	2122.09453	-0.71	51.78	5056	5056	MS2	22/30	Glioma_Nuclear_prep_GradeII_repeat_4plex.raw
4504347		High	tYFPHFDLSHGSAQVk	Unambiguous	1	1	N-Term(iTRAQ4plex); K16(iTRAQ4plex)	HCD	1.0000	0.0000	1	1	10526	Unique	Used	3.052	4.027E2	1.340E3	162	4.23	0.00	0	21	165	4	531.27979	2122.09731	0.60	51.36	5017	5017	MS2	28/90	Glioma_Nuclear_prep_GradeII_repeat_4plex.raw
4504347	High		tYFPHFDLSHGSAQVk	Unambiguous	1	1	N-Term(iTRAQ4plex); K16(iTRAQ4plex)	HCD	1.0000	0.0000	1	1	9642	Unique	Used	2.472	5.814E4	1.577E5	153	5.08	0.00	0	35	13	2	1061.53308	2122.05889	-17.51	38.27	4241	4241	MS2	21/30	Glioma_Nuclear_prep_GradeII_4plex.raw
4504347	High		tYFPHFDLSHGSAQVk	Unambiguous	1	1	N-Term(iTRAQ4plex); K16(iTRAQ4plex)	HCD	1.0000	0.0000	1	1	9639	Unique	Used	2.518	7.596E4	2.099E5	144	5.55	0.00	0	16	3	3	708.02551	2122.06198	-16.05	38.25	4238	4238	MS2	33/60	Glioma_Nuclear_prep_GradeII_4plex.raw
4504347		High	mFLSFPTTk	Unambiguous	1	1	N-Term(iTRAQ4plex); K9(iTRAQ4plex)	HCD	1.0000	0.0000	1	1	11478	Unique	Used	1.979	4.412E3	9.514E3	266	3.28	0.00	0	40	300	2	680.38226	1359.75725	-0.92	63.86	6104	6104	MS2	13/16	Glioma_Nuclear_prep_GradeII_repeat_4plex.raw
4504347		High	mFLSFPTTk	Unambiguous	1	1	N-Term(iTRAQ4plex); K9(iTRAQ4plex)	HCD	1.0000	0.0000	1	1	11550	Unique	Used	2.326	1.411E4	3.577E4	233	3.39	0.00	0	7	165	2	680.38300	1359.75871	0.15	64.83	6188	6188	MS2	13/16	Glioma_Nuclear_prep_GradeII_repeat_4plex.raw
4504347	High		mFLSFPTTk	Unambiguous	1	1	N-Term(iTRAQ4plex); K9(iTRAQ4plex)	HCD	1.0000	0.0000	1	1	10674	Unique	Used	1.829	2.048E5	4.111E5	260	3.51	0.00	0	32	11	2	680.37280	1359.73833	-14.84	45.96	5367	5367	MS2	13/16	Glioma_Nuclear_prep_GradeII_4plex.raw
4504347	High		mFLSFPTTk	Unambiguous	1	1	N-Term(iTRAQ4plex); K9(iTRAQ4plex)	HCD	1.0000	0.0000	1	1	10572	Unique	Used	2.298	1.951E5	4.921E5	282	3.52	0.00	0	15	11	2	680.37183	1359.73638	-16.28	45.18	5255	5255	MS2	13/16	Glioma_Nuclear_prep_GradeII_4plex.raw
4504347		High	mFLSFPTTk	Unambiguous	1	1	N-Term(iTRAQ4plex); M1(Oxidation); K9(iTRAQ4plex)	HCD	1.0000	0.0000	1	1	10672	Unique	Used	1.902	6.719E3	1.393E4	243	3.17	0.00	0	19	300	2	688.38025	1375.75322	-0.15	53.10	5174	5174	MS2	14/16	Glioma_Nuclear_prep_GradeII_repeat_4plex.raw
4504349	NP_000509.1	HBB	3043	hemoglobin subunit beta [Homo sapiens]	78.91	5	11	39	2.147	23;16	36.1	128.94	147	16.0	7.28																			
4504349		High	vNVDEVGGEALGR	Unambiguous	1	1	N-Term(iTRAQ4plex)	HCD	1.0000	0.0000	1	1	9606	Unique	Used	2.448	1.202E4	3.208E4	191	4.48	0.00	0	10	123	2	729.88538	1458.76348	-2.39	40.48	4030	4030	MS2	21/24	Glioma_Nuclear_prep_GradeII_repeat_4plex.raw
4504349		High	lLVVYPWTQR	Unambiguous	5	1	N-Term(iTRAQ4plex)	HCD	1.0000	0.0000	1	1	12058	Unique	Used	2.626	2.204E3	6.307E3	238	3.47	0.00	0	12	300	2	709.91760	1418.82793	0.18	73.09	6865	6865	MS2	14/18	Glioma_Nuclear_prep_GradeII_repeat_4plex.raw
4504349		High	lHVDPENFR	Unambiguous	2	1	N-Term(iTRAQ4plex)	HCD	1.0000	0.0000	1	1	8992	Unique	Used	2.668	1.325E4	3.854E4	218	3.12	0.00	0	46	243	2	635.83667	1270.66606	-0.02	33.62	3373	3373	MS2	11/16	Glioma_Nuclear_prep_GradeII_repeat_4plex.raw
4504349		High	lLVVYPWTQR	Unambiguous	5	1	N-Term(iTRAQ4plex)	HCD	1.0000	0.0000	1	1	12092	Unique	Used	2.720	7.761E3	2.301E4	186	3.61	0.00	0	6	213	2	709.91809	1418.82891	0.87	74.10	6939	6939	MS2	14/18	Glioma_Nuclear_prep_GradeII_repeat_4plex.raw
4504349		High	lHVDPENFR	Unambiguous	2	1	N-Term(iTRAQ4plex)	HCD	1.0000	0.0000	1	1	8962	Unique	Used	4.170	2.788E3	1.267E4	125	3.64	0.00	0	46	256	3	424.22406	1270.65763	-6.66	33.25	3339	3339	MS2	17/32	Glioma_Nuclear_prep_GradeII_repeat_4plex.raw
4504349		High	lHVDPENFR	Unambiguous	2	1	N-Term(iTRAQ4plex)	HCD	1.0000	0.0000	1	1	9053	Unique	Used	4.415	1.910E3	9.192E3	170	4.00	0.00	0	43	230	3	424.22690	1270.66614	0.04	34.35	3439	3439	MS2	18/32	Glioma_Nuclear_prep_GradeII_repeat_4plex.raw
4504349	High		lLVVYPWTQR	Unambiguous	5	1	N-Term(iTRAQ4plex)	HCD	1.0000	0.0000	1	1	11299	Unique	Used	1.979	2.105E4	4.571E4	265	3.53	0.00	0	37	107	2	709.91589	1418.82451	-2.23	50.85	6047	6047	MS2	14/18	Glioma_Nuclear_prep_GradeII_4plex.raw
4504349	High		lLVVYPWTQR	Unambiguous	5	1	N-Term(iTRAQ4plex)	HCD	1.0000	0.0000	1	1	11448	Unique	Used	2.083	2.343E4	5.355E4	251	3.14	0.00	0	65	120	2	709.91809	1418.82891	0.87	52.17	6211	6211	MS2	12/18	Glioma_Nuclear_prep_GradeII_4plex.raw
4504349	High		vNVDEVGGEALGR	Unambiguous	1	1	N-Term(iTRAQ4plex)	HCD	1.0000	0.0000	1	1	9183	Unique	Used	2.156	3.617E5	8.559E5	130	4.02	0.00	0	6	3	2	729.87524	1458.74321	-16.28	34.90	3740	3740	MS2	22/24	Glioma_Nuclear_prep_GradeII_4plex.raw
4504349	High		lLVVYPWTQR	Unambiguous	5	1	N-Term(iTRAQ4plex)	HCD	1.0000	0.0000	1	1	11373	Unique	Used	2.728	3.515E5	1.052E6	171	3.59	0.00	0	5	5	2	709.91785	1418.82842	0.52	51.52	6129	6129	MS2	14/18	Glioma_Nuclear_prep_GradeII_4plex.raw
4504349		High	gTFATLSELHcDk	Unambiguous	1	1	N-Term(iTRAQ4plex); C11(Methylthio); K13(iTRAQ4plex)	HCD	1.0000	0.0000	1	1	11095	Unique	Used	2.385	1.707E3	4.437E3	169	5.32	0.00	0	36	300	2	878.43884	1755.87041	3.17	58.38	5639	5639	MS2	22/24	Glioma_Nuclear_prep_GradeII_repeat_4plex.raw
4504349	High		gTFATLSELHcDk	Unambiguous	1	1	N-Term(iTRAQ4plex); C11(Methylthio); K13(iTRAQ4plex)	HCD	1.0000	0.0000	1	1	10126	Unique	Used	2.856	7.892E4	2.474E5	111	5.17	0.00	0	18	14	2	878.41998	1755.83269	-18.32	41.79	4767	4767	MS2	22/24	Glioma_Nuclear_prep_GradeII_4plex.raw
4504349	High		gTFATLSELHcDk	Unambiguous	1	1	N-Term(iTRAQ4plex); C11(Methylthio); K13(iTRAQ4plex)	HCD	1.0000	0.0000	1	1	10113	Unique	Used	3.773	1.863E4	7.713E4	80	4.66	0.00	0	27	6	3	585.94910	1755.83274	-18.29	41.69	4753	4753	MS2	28/48	Glioma_Nuclear_prep_GradeII_4plex.raw
4504349	High		kVLGAFSDGLAHLDNLk	Unambiguous	2	1	N-Term(iTRAQ4plex); K1(iTRAQ4plex); K17(iTRAQ4plex)	HCD	1.0000	0.0000	1	1	10799	Unique	Used	1.995	3.261E4	7.141E4	99	4.30	0.00	1	15	5	3	744.09119	2230.25901	-14.81	46.92	5503	5503	MS2	31/64	Glioma_Nuclear_prep_GradeII_4plex.raw
4504349		High	vVAGVANALAHk	Unambiguous	2	1	N-Term(iTRAQ4plex); K12(iTRAQ4plex)	HCD	1.0000	0.0000	1	1	10055	Unique	Used	1.495	4.545E3	7.404E3	95	6.52	0.00	0	21	171	3	479.96448	1437.87888	0.57	45.75	4513	4513	MS2	30/44	Glioma_Nuclear_prep_GradeII_repeat_4plex.raw
4504349		High	vVAGVANALAHk	Unambiguous	2	1	N-Term(iTRAQ4plex); K12(iTRAQ4plex)	HCD	1.0000	0.0000	1	1	9978	Unique	Used	1.734	1.909E3	3.608E3	84	6.45	0.00	0	46	300	3	479.96323	1437.87513	-2.04	44.79	4428	4428	MS2	30/44	Glioma_Nuclear_prep_GradeII_repeat_4plex.raw
4504349		High	vVAGVANALAHkYH	Unambiguous	2	1	N-Term(iTRAQ4plex); K12(iTRAQ4plex)	HCD	1.0000	0.0000	1	1	10466	Unique	Used	1.864	8.348E2	1.696E3	114	4.58	0.00	1	51	300	3	580.00403	1737.99753	-1.59	50.61	4951	4951	MS2	26/52	Glioma_Nuclear_prep_GradeII_repeat_4plex.raw
4504349		High	vVAGVANALAHk	Unambiguous	2	1	N-Term(iTRAQ4plex); K12(iTRAQ4plex)	HCD	1.0000	0.0000	1	1	9979	Unique	Used	2.147	6.627E3	1.551E4	145	5.42	0.00	0	53	300	2	719.44244	1437.87761	-0.31	44.80	4429	4429	MS2	22/22	Glioma_Nuclear_prep_GradeII_repeat_4plex.raw
4504349	High		vVAGVANALAHk	Unambiguous	2	1	N-Term(iTRAQ4plex); K12(iTRAQ4plex)	HCD	1.0000	0.0000	1	1	9300	Unique	Used	1.727	6.460E4	1.224E5	204	5.40	0.00	0	11	2	3	479.95554	1437.85205	-18.08	35.76	3868	3868	MS2	33/44	Glioma_Nuclear_prep_GradeII_4plex.raw
4504349	High		vVAGVANALAHk	Unambiguous	2	1	N-Term(iTRAQ4plex); K12(iTRAQ4plex)	HCD	1.0000	0.0000	1	1	9201	Unique	Used	1.842	1.059E5	2.142E5	234	5.08	0.00	0	45	13	2	719.43079	1437.85430	-16.53	35.01	3759	3759	MS2	21/22	Glioma_Nuclear_prep_GradeII_4plex.raw
4504349	High		vVAGVANALAHk	Unambiguous	2	1	N-Term(iTRAQ4plex); K12(iTRAQ4plex)	HCD	1.0000	0.0000	1	1	9287	Unique	Used	1.929	5.036E4	1.066E5	215	3.69	0.00	0	15	3	2	719.43097	1437.85466	-16.27	35.66	3854	3854	MS2	21/22	Glioma_Nuclear_prep_GradeII_4plex.raw

4504349	High		vVAGVANALAHk	Unambiguous	2	1	N-Term(iTRAQ4plex); K12(iTRAQ4plex)	HCD	1.0000	0.0000	1	1	9209	Unique	Used	1.993	8.616E4	1.884E5	112	5.90	0.00	0	42	3	3	479.95535	1437.85151	-18.47	35.08	3768	3768	MS2	30/44	Glioma_Nuclear_prep_Gradeall_4plex.raw
4504349	High		eFTPPVQAAYQk	Unambiguous	1	1	N-Term(iTRAQ4plex); K12(iTRAQ4plex)	HCD	1.0000	0.0000	1	1	9054	Unique	Used	5.606	3.378E4	2.078E5	233	4.02	0.00	0	11	27	2	833.94110	1666.87493	-17.63	33.93	3598	3598	MS2	19/22	Glioma_Nuclear_prep_Gradeall_4plex.raw
4504349		High	vLGAFSDGLAHLDNLk	Unambiguous	2	1	N-Term(iTRAQ4plex); K16(iTRAQ4plex)	HCD	1.0000	0.0000	1	1	12347	Unique	Used	2.745	3.269E2	9.779E2	196	4.98	0.00	0	21	300	3	653.36987	1958.09507	0.03	80.58	7428	7428	MS2	29/60	Glioma_Nuclear_prep_Gradeall_repeat_4plex.raw
4504349	High		vLGAFSDGLAHLDNLk	Unambiguous	2	1	N-Term(iTRAQ4plex); K16(iTRAQ4plex)	HCD	1.0000	0.0000	1	1	12007	Unique	Used	2.175	3.035E3	7.243E3	193	5.78	0.00	0	33	174	3	653.37067	1958.09745	1.25	57.05	6820	6820	MS2	29/60	Glioma_Nuclear_prep_Gradeall_4plex.raw
4504349	High		vLGAFSDGLAHLDNLk	Unambiguous	2	1	N-Term(iTRAQ4plex); K16(iTRAQ4plex)	HCD	1.0000	0.0000	1	1	11787	Unique	Used	2.800	7.153E3	2.198E4	184	5.85	0.00	0	44	41	3	653.36731	1958.08738	-3.90	55.09	6580	6580	MS2	24/60	Glioma_Nuclear_prep_Gradeall_4plex.raw
4504349	High		vLGAFSDGLAHLDNLk	Unambiguous	2	1	N-Term(iTRAQ4plex); K16(iTRAQ4plex)	HCD	1.0000	0.0000	1	1	11903	Unique	Used	2.989	2.554E4	8.377E4	190	6.98	0.00	0	2	44	2	979.54877	1958.09026	-2.43	56.07	6706	6706	MS2	29/30	Glioma_Nuclear_prep_Gradeall_4plex.raw
4504349	High		vLGAFSDGLAHLDNLk	Unambiguous	2	1	N-Term(iTRAQ4plex); K16(iTRAQ4plex)	HCD	1.0000	0.0000	1	1	11866	Unique	Used	3.425	9.026E4	3.392E5	196	5.20	0.00	0	11	4	3	653.36902	1958.09250	-1.28	55.77	6666	6666	MS2	32/60	Glioma_Nuclear_prep_Gradeall_4plex.raw
4504349	High		vLGAFSDGLAHLDNLk	Unambiguous	2	1	N-Term(iTRAQ4plex); K16(iTRAQ4plex)	HCD	1.0000	0.0000	1	1	11821	Unique	Used	3.561	2.071E4	8.094E4	193	7.39	0.00	0	36	53	2	979.54779	1958.08830	-3.42	55.39	6617	6617	MS2	29/30	Glioma_Nuclear_prep_Gradeall_4plex.raw
4504349		High	fFESFGDLSTPDAVMGNPk	Unambiguous	1	1	N-Term(iTRAQ4plex); K19(iTRAQ4plex)	HCD	1.0000	0.0000	1	1	12335	Unique	Used	1.728	6.707E1	1.263E2	139	4.46	0.00	0	17	300	3	783.05615	2347.15390	0.83	80.20	7401	7401	MS2	28/72	Glioma_Nuclear_prep_Gradeall_repeat_4plex.raw
4504349	High		fFESFGDLSTPDAVMGNPk	Unambiguous	1	1	N-Term(iTRAQ4plex); K19(iTRAQ4plex)	HCD	1.0000	0.0000	1	1	11925	Unique	Used	1.299	3.420E3	4.876E3	144	6.59	0.00	0	31	50	3	783.05853	2347.16104	3.87	56.27	6730	6730	MS2	25/72	Glioma_Nuclear_prep_Gradeall_4plex.raw
4504349	High		fFESFGDLSTPDAVMGNPk	Unambiguous	1	1	N-Term(iTRAQ4plex); K19(iTRAQ4plex)	HCD	1.0000	0.0000	1	1	12001	Unique	Used	1.565	1.209E4	2.077E4	141	6.12	0.00	0	8	7	3	783.05591	2347.15317	0.52	57.01	6814	6814	MS2	27/72	Glioma_Nuclear_prep_Gradeall_4plex.raw
4504349	High		fFESFGDLSTPDAVMGNPk	Unambiguous	1	1	N-Term(iTRAQ4plex); K19(iTRAQ4plex)	HCD	1.0000	0.0000	1	1	11938	Unique	Used	2.088	2.003E4	4.590E4	132	7.58	0.00	0	0	69	2	1174.08020	2347.15312	0.50	56.38	6744	6744	MS2	29/36	Glioma_Nuclear_prep_Gradeall_4plex.raw
4504349		High	sAVTALWGk	Unambiguous	1	1	N-Term(iTRAQ4plex); K9(iTRAQ4plex)	HCD	1.0000	0.0000	1	1	10139	Unique	Used	2.393	1.028E4	2.681E4	220	3.95	0.00	0	39	167	2	610.86560	1220.72392	-0.20	46.76	4604	4604	MS2	16/16	Glioma_Nuclear_prep_Gradeall_repeat_4plex.raw
4504349	High		sAVTALWGk	Unambiguous	1	1	N-Term(iTRAQ4plex); K9(iTRAQ4plex)	HCD	1.0000	0.0000	1	1	9485	Unique	Used	1.988	2.148E5	4.687E5	300	3.69	0.00	0	38	6	2	610.85516	1220.70305	-17.30	37.15	4071	4071	MS2	16/16	Glioma_Nuclear_prep_Gradeall_4plex.raw
4504349		High	fFESFGDLSTPDAVmGNPk	Unambiguous	1	1	N-Term(iTRAQ4plex); M15(Oxidation); K19(iTRAQ4plex)	HCD	1.0000	0.0000	1	1	11987	Unique	Used	1.758	2.332E2	4.469E2	127	4.64	0.00	0	23	300	3	788.38824	2363.15018	1.40	71.78	6761	6761	MS2	28/72	Glioma_Nuclear_prep_Gradeall_repeat_4plex.raw
4504349		High	fFESFGDLSTPDAVmGNPk	Unambiguous	1	1	N-Term(iTRAQ4plex); M15(Oxidation); K19(iTRAQ4plex)	HCD	1.0000	0.0000	1	1	12005	Unique	Used	2.146	5.771E2	1.350E3	156	5.93	0.00	0	0	300	2	1182.07642	2363.14556	-0.56	72.12	6788	6788	MS2	25/36	Glioma_Nuclear_prep_Gradeall_repeat_4plex.raw
4504349	High		fFESFGDLSTPDAVmGNPk	Unambiguous	1	1	N-Term(iTRAQ4plex); M15(Oxidation); K19(iTRAQ4plex)	HCD	1.0000	0.0000	1	1	11304	Unique	Used	1.715	1.227E4	2.309E4	135	5.40	0.00	0	7	8	3	788.38440	2363.13864	-3.48	50.91	6053	6053	MS2	26/72	Glioma_Nuclear_prep_Gradeall_4plex.raw
4504349	High		fFESFGDLSTPDAVmGNPk	Unambiguous	1	1	N-Term(iTRAQ4plex); M15(Oxidation); K19(iTRAQ4plex)	HCD	1.0000	0.0000	1	1	11294	Unique	Used	1.984	2.313E4	5.036E4	140	7.11	0.00	0	0	38	2	1182.07251	2363.13774	-3.86	50.82	6042	6042	MS2	23/36	Glioma_Nuclear_prep_Gradeall_4plex.raw
4504425	NP_002119.1	HMGB1	3146	high mobility group protein B1 [Homo sapiens]	27.44	1	5	16	3.679	6;10	35.9	64.64	215	24.9	5.74																			
4504425		High	kHPDASVNFSEFSk	Unambiguous	1	1	N-Term(iTRAQ4plex); K1(iTRAQ4plex); K14(iTRAQ4plex)	HCD	1.0000	0.0000	1	1	9095	Unique	Used	2.755	5.103E2	1.533E3	178	4.06	0.00	1	24	68	4	507.02374	2025.07314	-1.71	34.82	3484	3484	MS2	15/78	Glioma_Nuclear_prep_Gradeall_repeat_4plex.raw
4504425		High	kHPDASVNFSEFSk	Unambiguous	1	1	N-Term(iTRAQ4plex); K1(iTRAQ4plex); K14(iTRAQ4plex)	HCD	1.0000	0.0000	1	1	9087	Unique	Used	4.546	1.344E3	6.657E3	97	4.85	0.00	1	0	300	2	1013.04340	2025.07952	1.44	34.72	3475	3475	MS2	22/26	Glioma_Nuclear_prep_Gradeall_repeat_4plex.raw
4504425		High	kHPDASVNFSEFSk	Unambiguous	1	1	N-Term(iTRAQ4plex); K1(iTRAQ4plex); K14(iTRAQ4plex)	HCD	1.0000	0.0000	1	1	9078	Unique	Used	4.698	1.265E3	6.477E3	119	5.57	0.00	1	14	70	3	675.69849	2025.08091	2.13	34.63	3466	3466	MS2	24/52	Glioma_Nuclear_prep_Gradeall_repeat_4plex.raw
4504425	High		kHPDASVNFSEFSk	Unambiguous	1	1	N-Term(iTRAQ4plex); K1(iTRAQ4plex); K14(iTRAQ4plex)	HCD	1.0000	0.0000	1	1	8459	Unique	Used	3.761	2.296E4	9.473E4	140	4.04	0.00	1	7	12	2	1013.02338	2025.03948	-18.33	29.60	2950	2950	MS2	20/26	Glioma_Nuclear_prep_Gradeall_4plex.raw
4504425		High	hPDASVNFSEFSk	Unambiguous	1	1	N-Term(iTRAQ4plex); K13(iTRAQ4plex)	HCD	1.0000	0.0000	1	1	9650	Unique	Used	2.349	1.394E3	3.570E3	165	6.29	0.00	0	9	138	3	584.96356	1752.87613	-1.96	40.97	4077	4077	MS2	24/48	Glioma_Nuclear_prep_Gradeall_repeat_4plex.raw
4504425		High	gEHPGLSIGDVAk	Unambiguous	1	1	N-Term(iTRAQ4plex); K13(iTRAQ4plex)	HCD	1.0000	0.0000	1	1	8723	Unique	Used	2.591	1.720E3	4.856E3	230	6.04	0.00	0	39	242	3	523.29498	1567.87040	1.34	30.84	3087	3087	MS2	21/48	Glioma_Nuclear_prep_Gradeall_repeat_4plex.raw
4504425		High	gEHPGLSIGDVAk	Unambiguous	1	1	N-Term(iTRAQ4plex); K13(iTRAQ4plex)	HCD	1.0000	0.0000	1	1	8745	Unique	Used	3.933	2.713E3	1.163E4	242	5.47	0.00	0	10	53	3	523.28613	1567.84385	-15.59	31.02	3111	3111	MS2	24/48	Glioma_Nuclear_prep_Gradeall_repeat_4plex.raw
4504425		High	gEHPGLSIGDVAk	Unambiguous	1	1	N-Term(iTRAQ4plex); K13(iTRAQ4plex)	HCD	1.0000	0.0000	1	1	8755	Unique	Used	3.961	1.566E3	6.762E3	204	3.13	0.00	0	6	257	2	784.42700	1567.84673	-13.75	31.11	3121	3121	MS2	20/24	Glioma_Nuclear_prep_Gradeall_repeat_4plex.raw
4504425		High	hPDASVNFSEFSk	Unambiguous	1	1	N-Term(iTRAQ4plex); K13(iTRAQ4plex)	HCD	1.0000	0.0000	1	1	9659	Unique	Used	4.127	1.863E3	8.377E3	147	4.49	0.00	0	10	300	2	876.94238	1752.87749	-1.19	41.07	4086	4086	MS2	19/24	Glioma_Nuclear_prep_Gradeall_repeat_4plex.raw
4504425	High		hPDASVNFSEFSk	Unambiguous	1	1	N-Term(iTRAQ4plex); K13(iTRAQ4plex)	HCD	1.0000	0.0000	1	1	9015	Unique	Used	1.509	1.521E4	2.520E4	125	5.25	0.00	0	19	9	3	584.95447	1752.84885	-17.52	33.63	3555	3555	MS2	24/48	Glioma_Nuclear_prep_Gradeall_4plex.raw
4504425	High		gEHPGLSIGDVAk	Unambiguous	1	1	N-Term(iTRAQ4plex); K13(iTRAQ4plex)	HCD	1.0000	0.0000	1	1	8284	Unique	Used	3.083	3.025E4	1.023E5	254	3.76	0.00	0	56	21	2	784.42365	1567.84001	-18.03	28.36	2760	2760	MS2	18/24	Glioma_Nuclear_prep_Gradeall_4plex.raw
4504425	High		hPDASVNFSEFSk	Unambiguous	1	1	N-Term(iTRAQ4plex); K13(iTRAQ4plex)	HCD	1.0000	0.0000	1	1	9006	Unique	Used	3.819	2.967E4	1.243E5	120	4.44	0.00	0	0	14	2	876.92950	1752.85173	-15.88	33.56	3545	3545	MS2	17/24	Glioma_Nuclear_prep_Gradeall_4plex.raw
4504425		High	ikGEHPGLSIGDVAk	Unambiguous	1	1	N-Term(iTRAQ4plex); K2(iTRAQ4plex); K15(iTRAQ4plex)	HCD	1.0000	0.0000	1	1	8902	Unique	Used	2.704	1.443E3	4.252E3	105	3.50	0.00	1	14	45	3	651.71893	1953.14225	-3.66	32.67	3276	3276	MS2	25/56	Glioma_Nuclear_prep_Gradeall_repeat_4plex.raw
4504425	High		ikGEHPGLSIGDVAk	Unambiguous	1	1	N-Term(iTRAQ4plex); K2(iTRAQ4plex); K15(iTRAQ4plex)	HCD	1.0000	0.0000	1	1	8373	Unique	Used	6.880	2.034E4	1.536E5	164	4.51	0.00	1	5	7	2	977.06030	1953.11333	-18.46	29.00	2857	2857	MS2	22/28	Glioma_Nuclear_prep_Gradeall_4plex.raw
4504425		High	tYIPPkGETk	Unambiguous	1	1	N-Term(iTRAQ4plex); K6(iTRAQ4plex); K10(iTRAQ4plex)	HCD	1.0000	0.0000	1	1	7564	Unique	Used	3.596	1.325E3	5.195E3	156	2.96	0.00	1	10	167	2	783.46857	1565.92986	2.25	19.64	1862	1862	MS2	15/18	Glioma_Nuclear_prep_Gradeall_repeat_4plex.raw
4504425	High		tYIPPkGETk	Unambiguous	1	1	N-Term(iTRAQ4plex); K6(iTRAQ4plex); K10(iTRAQ4plex)	HCD	1.0000	0.0000	1	1	7511	Unique	Used	2.201	6.612E4	1.597E5	143	3.29	0.00	1	18	8	2	783.46265	1565.91802	-5.31	22.90	1923	1923	MS2	14/18	Glioma_Nuclear_prep_Gradeall_4plex.raw
4504445	NP_002127.1	HNRNPA1	3178	heterogeneous nuclear ribonucleoprotein A1 isoform a [Homo sapiens]	36.56	3	7	16	0.287	8;8	30.2	49.91	320	34.2	9.23																			
4504445		High	nQGGYGGSSSSSSYGSGR	Unambiguous	2	1	N-Term(iTRAQ4plex)	HCD	1.0000	0.0000	1	1	7004	Unique	Used	0.111	2.648E4	3.211E3	13	6.00	0.00	0	4	192	2	919.90302	1838.79875	-1.88	14.83	1272	1272	MS2	25/34	Glioma_Nuclear_prep_Gradeall_repeat_4plex.raw
4504445		High	iEVIEIMTDR	Unambiguous	3	1	N-Term(iTRAQ4plex)	HCD	1.0000	0.0000	1	1	11990	Unique	Used	0.323	1.852E4	6.519E3	337	3.73	0.00	0	34	300	2	681.87610	1362.74492	2.15	71.84	6766	6766	MS2	14/18	Glioma_Nuclear_prep_Gradeall_repeat_4plex.raw
4504445	High		iEVIEIMTDR	Unambiguous	3	1	N-Term(iTRAQ4plex)	HCD	1.0000	0.0000	1	1	11291	Unique	Used	0.283	2.192E5	6.803E4	329	3.99	0.00	0	22	27	2	681.87372	1362.74016	-1.34	50.80	6039	6039	MS2	15/18	Glioma_Nuclear_prep_Gradeall_4plex.raw
4504445	High		iEVIEIMTDR	Unambiguous	3	1	N-Term(iTRAQ4plex)	HCD	1.0000	0.0000	1	1	11205	Unique	Used	0.329	2.436E5	8.793E4	304	4.05	0.00	0	33	21	2	681.87054	1362.73381	-6.00	50.06	5945	5945	MS2	15/18	Glioma_Nuclear_prep_Gradeall_4plex.raw
4504445	High		iFIGGLSFETTDESLR	Unambiguous	3	1	N-Term(iTRAQ4plex)	HCD	1.0000	0.0000	1	1	12107	Unique	Used	0.353	4.547E4	1.763E4	231	5.11	0.00	0	21	184	2	965.01422	1929.02117	6.49	58.09	6930	6930	MS2	23/30	Glioma_Nuclear_prep_Gradeall_4plex.raw
4504445		High	eDSQRPGAHLTVk	Unambiguous	3	1	N-Term(iTRAQ4plex); K13(iTRAQ4plex)	HCD	1.0000	0.0000	1	1	7203	Unique	Used	0.266	4.880E3	1.415E3	200	3.25	0.00	0	9	168	2	863.46692	1725.92656	-12.80	16.55	1483	1483	MS2	16/24	Glioma_Nuclear_prep_Gradeall_repeat_4plex.raw
4504445	High		eDSQRPGAHLTVk	Unambiguous	3	1	N-Term(iTRAQ4plex); K13(iTRAQ4plex)	HCD	1.0000	0.0000	1	1	7328	Unique	Used	0.290	6.510E4	2.075E4	219	4.05	0.00	0	12	22	2	863.47632	1725.94536	-1.91	21.57	1724	1724	MS2	18/24	Glioma_Nuclear_prep_Gradeall_4plex.raw
4504445		High	sSGPYGGGGQYFAkPR	Unambiguous	2	1	N-Term(iTRAQ4plex); K14(iTRAQ4plex)	HCD	1.0000	0.0000	1	1	8618	Unique	Used	0.133	7.967E3	1.151E3	93	5.65	0.00	0	13	300	2	958.99927	1916.99126	2.86	29.82	2977	2977	MS2	28/30	Glioma_Nuclear_prep_Gradeall_repeat_4plex.raw
4504445		High	sSGPYGGGGQYFAkPR	Unambiguous	2	1	N-Term(iTRAQ4plex); K14(iTRAQ4plex)	HCD	1.0000	0.0000	1	1	8587	Unique	Used	0.254	7.323E3	2.027E3	130	5.02	0.00	0	21	119	3	639.66583	1916.98294	-1.47	29.50	2945	2945	MS2	32/60	Glioma_Nuclear_prep_Gradeall_repeat_4plex.raw
4504445	High		sSGPYGGGGQYFAkPR	Unambiguous	2	1	N-Term(iTRAQ4plex); K14(iTRAQ4plex)	HCD	1.0000	0.0000	1	1	8202	Unique	Used	0.251	1.545E5	4.261E4	88	4.95	0.00	0	32	3	3	639.65662	1916.95530	-15.90	27.79	2672	2672	MS2	30/60	Glioma_Nuclear_prep_Gradeall_4plex.raw
4504445		High	gFAFVTFDDHDSVDk	Unambiguous	3	1	N-Term(iTRAQ4plex); K15(iTRAQ4plex)	HCD	1.0000	0.0000	1	1	11213	Unique	Used	0.316	4.258E3	1.467E3	124	5.89	0.00	0	3	300	2	994.48755	1987.96782	1.90	59.98	5777	5777	MS2	23/28	Glioma_Nuclear_prep_Gradeall_repeat_4plex.raw
4504445		High	gFAFVTFDDHDSVDk	Unambiguous	3	1	N-Term(iTRAQ4plex); K15(iTRAQ4plex)	HCD	1.0000	0.0000	1	1	11172	Unique	Used	0.411	1.557E3	6.968E2	110	5.40	0.00	0	14	300	3	663.32794	1987.96927	2.63	59.44	5730	5730	MS2	29/56	Glioma_Nuclear_prep_Gradeall_repeat_4plex.raw
4504445	High		gFAFVTFDDHDSVDk	Unambiguous	3	1	N-Term(iTRAQ4plex); K15(iTRAQ4plex)	HCD	1.0000	0.0000	1	1	10260	Unique	Used	0.310	1.672E5	5.688E4	92	5.16	0.00	0	5	12	2	994.47174	1987.93620	-14.01	42.79	4913	4913	MS2	25/28	Glioma_Nuclear_prep_Gradeall_4plex.raw
4504445	High		gFAFVTFDDHDSVDk	Unambiguous	3	1	N-Term(iTRAQ4plex); K15(iTRAQ4plex)	HCD	1.0000	0.0000	1	1	10245	Unique	Used	0.455	7.780E4	3.882E4	92	4.09	0.00	0	15	6	3	663.31683	1987.93595	-14.14	42.69	4897	4897	MS2	27/56	Glioma_Nuclear_prep_Gradeall_4plex.raw
4504445		High	dYFEQYGk	Unambiguous	3	1	N-Term(iTRAQ4plex); K8(iTRAQ4plex)	HCD	1.0000	0.0000	1	1	9472	Unique	Used	0.235	7.780E4	1.995E4	103	3.18	0.00	0	50	140	2	669.33630	1337.66533	2.79	38.93	3882	3882	MS2	13/14	Glioma_Nuclear_prep_Gradeall_repeat_4plex.raw
4504445	High		dYFEQYGk	Unambiguous	3	1	N-Term(iTRAQ4plex); K8(iTRAQ4plex)	HCD	1.0000	0.0000	1	1	9044	Unique	Used	0.276	7.061E5	2.141E5	87	3.15	0.00	0	31	7	2	669.32495	1337.64263	-14.18	33.85	3587	3587	MS2	13/14	Glioma_Nuclear_prep_Gradeall_4plex.raw
4504523	NP_002148.1	HSPE1	3336	10 kDa heat shock protein, mitochondrial [Homo sapiens]	9.80	1	1	2	2.034	1;1	8.3	3.13	102	10.9	8.92																			
4504523		High	vLLPEYGGTk	Unambiguous	1	1	N-Term(iTRAQ4plex); K10(iTRAQ4plex)	HCD	1.0000	0.0000	1	1	9802	Unique	Used	2.154	9.558E3	2.244E4	253	3.13	0.00	0	43	300	2	682.90552	1364.80376	0.70	42.69	4237	4237	MS2	13/18	Glioma_Nuclear_prep_Gradeall_repeat_4plex.raw
4504523	High		vLLPEYGGTk	Unambiguous	1	1	N-Term(iTRAQ4plex); K10(iTRAQ4plex)	HCD	1.0000	0.0000	1	1	9200	Unique	Used	1.914	9.052E4	1.901E5	293	3.01	0.00	0	45	12	2	682.89288	1364.77849	-17.82	35.01	3758	3758	MS2	13/18	Glioma_Nuclear_prep_Gradeall_4plex.raw
4505881	NP_000292.1	PLG	5340	plasminogen isoform 1 precursor [Homo sapiens]	4.32	1	3	3	1.617	2;1	22.4	8.13	810	90.5	7.24																			
4505881	High		fSPATHPSEGLEENYcR	Unambiguous	1	1	N-Term(iTRAQ4plex); C16(Methylthio)	HCD	1.0000	0.0000	1	1	9462	Unique	Used	2.055	1.297E4	2.924E4	25	5.11	0.00	0	23	6	3	709.64056	2126.90714	-15.10	36.98	4046	4046	MS2	27/64	Glioma_Nuclear_prep_Gradeall_4plex.raw
4505881	High		wELcDIPR	Unambiguous	1	1	N-Term(iTRAQ4plex); C4(Methylthio)	HCD	1.0000	0.0000	1	1	11389	Unique	Used	1.617	2.041E4	3.621E4	105	3.02	0.00	0	40	67	2	611.29657	1221.58586	-1.52	51.64	6146	6146	MS2	13/14	Glioma_Nuclear_prep_Gradeall_4plex.raw
4505881		High	lSSPAVITDk	Unambiguous	1	1	N-Term(iTRAQ4plex); K10(iTRAQ4plex)	HCD	1.0000	0.0000	1	1	8963	Unique	Used	1.314	6.561E3	9.397E3	299	3.70	0.00	0	45	300	2	659.89063	1318.77397	-6.15	33.26	3340	3340	MS2	16/18	Glioma_Nuclear_prep_Gradeall_repeat_4plex.raw
4506613	NP_000974.1	RPL22	6146	60S ribosomal protein L22 proprotein [Homo sapiens]	10.16	1	1	2	0.597	1;1	10.4	4.38	128	14.8	9.19																			
4506613		High	aGNLGGGVVTIER	Unambiguous	1	1	N-Term(iTRAQ4plex)	HCD	1.0000	0.0000	1	1	9206	Unique	Used	0.641	8.597E3	6.007E3	238	4.38	0.00	0	39	300	2	693.89624	1386.78520	2.16	36.03	3600	3600	MS2	23/24	Glioma_Nuclear_prep_Gradeall_repeat_4plex.raw
4506613	High		aGNLGGGVVTIER	Unambiguous	1	1	N-Term(iTRAQ4plex)	HCD	1.0000	0.0000	1	1	8780	Unique	Used	0.553	7.088E4	4.302E4	238	4.12	0.00	0	23	23	2	693.88306	1386.75884	-16.86	31.94	3300	3300	MS2	21/24	Glioma_Nuclear_prep_Gradeall_4plex.raw
4506661	NP_000963.1	RPL7A	6130	60S ribosomal protein L7a [Homo sapiens]	7.14	1	1	2	0.543	1;1	19.8	7.14	266	30.0	10.61																			
4506661		High	nFGIGQDIQPk	Unambiguous	1	1	N-Term(iTRAQ4plex); K11(iTRAQ4plex)	HCD	1.0000	0.0000	1	1	9540	Unique	Used	0.619	6.314E3	4.260E3	296	3.98	0.00	0	23	300	2	752.91888	1504.83049	-3.82	39.73	3958	3958	MS2	19/20	Glioma_Nuclear_prep_Gradeall_repeat_4plex.raw
4506661	High		nFGIGQDIQPk	Unambiguous	1	1	N-Term(iTRAQ4plex); K11(iTRAQ4plex)	HCD	1.0000	0.0000	1	1	9019	Unique	Used	0.467	1.151E5	5.902E4	312	3.76	0.00	0	39	28	2	752.91046	1504.81365	-15.02	33.65	3559	3559	MS2	19/20	Glioma_Nuclear_prep_Gradeall_4plex.raw
4506671	NP_000995.1	RPLP2	6181	60S acidic ribosomal protein P2 [Homo sapiens]	26.96	1	1	2	0.633	1;1	14.9	15.37	115	11.7	4.54																			
4506671		High	nIEDVIAQGIGk	Unambiguous	1	1	N-Term(iTRAQ4plex); K12(iTRAQ4plex)	HCD	1.0000	0.0000	1	1	11701	Unique	Used	0.700	3.187E3	2.430E3	221	4.36	0.00	0	48	300	2	772.94995	1544.89263	2.55	66.99	6370	6370	MS2	21/22	Glioma_Nuclear_prep_Gradeall_repeat_4plex.raw
4506671	High		nIEDVIAQGIGk	Unambiguous	1	1	N-Term(iTRAQ4plex); K12(iTRAQ4plex)	HCD	1.0000	0.0000	1	1	10887	Unique	Used	0.566	3.787E4	2.354E4	219	4.35	0.00	0	58	65	2	772.93610	1544.86492	-15.39	47.58	5598	5598	MS2	20/22	Glioma_Nuclear_prep_Gradeall_4plex.raw
4506695	NP_001013.1	RPS19	6223	40S ribosomal protein S19 [Homo sapiens]	11.03	1	2	2	0.581	1;1	20.2	3.69	145	16.1	10.32																			
4506695		High	aLAAFLk	Unambiguous	1	1	N-Term(iTRAQ4plex); K7(iTRAQ4plex)	HCD	1.0000	0.0000	1	1	10765	Unique	Used	0.498	1.520E4	8.254E3	166	3.18	0.00	0	61	300	2	511.33707	1021.66686	1.96	54.23	5274	5274	MS2	12/12	Glioma_Nuclear_prep_Gradeall_repeat_4plex.raw
4506695	High		vLQALEGLk	Unambiguous	1	1	N-Term(iTRAQ4plex); K9(iTRAQ4plex)	HCD	1.0000	0.0000	1	1	10941	Unique	Used	0.665	2.107E5	1.537E5	157	3.69	0.00	0	66	19	2	629.89539	1258.78349	-11.00	48.00	5657	5657	MS2	16/16	Glioma_Nuclear_prep_Gradeall_4plex.raw
4507127	NP_003084.1	SNRPC	6631	U1 small nuclear ribonucleoprotein C [Homo sapiens]	24.53	1	3	3	0.627	2;1	18.9	12.59	159	17.4	9.67																			
4507127	High		fYcDYcDTYLTHDSPSVR	Unambiguous	1	1	N-Term(iTRAQ4plex); C3(Methylthio); C6(Methylthio)	HCD	1.0000	0.0000	1	1	12204	Unique	Used	0.652	2.388E3	1.708E3	9	6.94	0.00	0	36	174	3	807.66772	2420.98862	4.52	59.03	7036	7036	MS2	28/68	Glioma_Nuclear_prep_Gradeall_4plex.raw
4507127	High		wMEEQAQSLIDk	Unambiguous	1	1	N-Term(iTRAQ4plex); K12(iTRAQ4plex)	HCD	1.0000	0.0000	1	1	10641	Unique	Used	0.627	8.240E4	5.672E4	164	5.66	0.00	0	24	36	2	883.44366	1765.88005	-13.20	45.70	5330	5330	MS2	22/22	Glioma_Nuclear_prep_Gradeall_4plex.raw
4507127		High	tTAAFQQGk	Unambiguous	1	1	N-Term(iTRAQ4plex); K9(iTRAQ4plex)	HCD	1.0000	0.0000	1	1	7315	Unique	Used	0.452	6.970E4	3.435E4	237	3.76	0.00	0	11	58	2	620.34906	1239.69084	-2.21	17.55	1601	1601	MS2	16/16	Glioma_Nuclear_prep_Gradeall_repeat_4plex.raw
4507241	NP_003137.1	SSRP1	6749	FACT complex subunit SSRP1 [Homo sapiens]	9.87	1	4	4	1.532	3;1	3.9	25.82	709	81.0	6.87																			
4507241		High	aSSGLLYPLER	Unambiguous	1	1	N-Term(iTRAQ4plex)	HCD	1.0000	0.0000	1	1	10573	Unique	Used	1.648	4.414E3	7.928E3	327	3.59	0.00	0	59	300	2	675.38013	1349.75298	-1.18	51.91	5067	5067	MS2	15/20	Glioma_Nuclear_prep_Gradeall_repeat_4plex.raw
4507241	High		iYPTFLHLHGk	Unambiguous	1	1	N-Term(iTRAQ4plex); K11(iTRAQ4plex)	HCD	1.0000	0.0000	1	1	9490	Unique	Used	1.520	2.569E4	4.284E4	204	3.77	0.00	0	14	7	4	404.23383	1613.91347	-16.83	37.18	4076	4076	MS2	22/60	Glioma_Nuclear_prep_Gradeall_4plex.raw
4507241	High		sDHPGISITDLSk	Unambiguous	1	1	N-Term(iTRAQ4plex); K13(iTRAQ4plex)	HCD	1.0000	0.0000	1	1	8827	Unique	Used	1.537	3.249E4	5.479E4	206	4.17	0.00	0	6	24	2	829.44000	1657.87273	-16.43	32.28	3351	3351	MS2	18/24	Glioma_Nuclear_prep_Gradeall_4plex.raw
4507241	High		fYVPPTQEDGVDPVEAFAQNVLSk	Unambiguous	1	1	N-Term(iTRAQ4plex); K24(iTRAQ4plex)	HCD	1.0000	0.0000	1	1	12930	Unique	Used	1.527	6.864E2	1.150E3	134	7.95	0.00	0	5	189	3	980.17456	2938.50913	0.46	68.50	7886	7886	MS2	29/92	Glioma_Nuclear_prep_Gradeall_4plex.raw
4507401	NP_003192.1	TFAM	7019	transcription factor A, mitochondrial isoform 1 precursor [Homo sapiens]	19.92	2	6	8	1.735	3;5	21.5	19.34	246	29.1	9.72																			
4507401		High	sAYNVYVAER	Unambiguous	1	1	N-Term(iTRAQ4plex)	HCD	1.0000	0.0000	1	1	8751	Unique	Used	1.722	1.176E4	2.208E4	121	3.30	0.00	0	8	166	2	658.33234	1315.65740	-14.37	31.07	3117	3117	MS2	17/18	Glioma_Nuclear_prep_Gradeall_repeat_4plex.raw
4507401	High		eQLTPSQIMSLEk	Unambiguous	2	1	N-Term(iTRAQ4plex); K13(iTRAQ4plex)	HCD	1.0000	0.0000	1	1	10147	Unique	Used	2.295	2.882E4	7.259E4	241	4.40	0.00	0	10	32	2	896.47809	1791.94890	-15.41	41.95	4790	4790	MS2	19/24	Glioma_Nuclear_prep_Gradeall_4plex.raw
4507401		High	fkEQLTPSQIMSLEk	Unambiguous	2	1	N-Term(iTRAQ4plex); K2(iTRAQ4plex); K15(iTRAQ4plex)	HCD	1.0000	0.0000	1	1	11081	Unique	Used	1.367	4.918E2	7.325E2	114	6.15	0.00	1	33	300	3	737.75311	2211.24479	1.27	58.20	5623	5623	MS2	23/56	Glioma_Nuclear_prep_Gradeall_repeat_4plex.raw
4507401		High	eQLPIFk	Unambiguous	2	1	N-Term(iTRAQ4plex); K7(iTRAQ4plex)	HCD	1.0000	0.0000	1	1	10190	Unique	Used	1.967	1.584E4	3.397E4	236	3.08	0.00	0	14	181	2	581.85742	1162.70757	0.10	47.36	4658	4658	MS2	12/12	Glioma_Nuclear_prep_Gradeall_repeat_4plex.raw
4507401		High	eLTLLGkPk	Unambiguous	1	1	N-Term(iTRAQ4plex); K7(iTRAQ4plex); K9(iTRAQ4plex)	HCD	1.0000	0.0000	1	1	9088	Unique	Used	1.304	5.491E3	7.803E3	27	3.15	0.00	0	69	300	2	715.96936	1430.93144	0.52	34.73	3476	3476	MS2	14/16	Glioma_Nuclear_prep_Gradeall_repeat_4plex.raw
4507401	High		eLTLLGkPk	Unambiguous	1	1	N-Term(iTRAQ4plex); K7(iTRAQ4plex); K9(iTRAQ4plex)	HCD	1.0000	0.0000	1	1	8473	Unique	Used	1.996	9.285E4	2.034E5	58	2.90	0.00	0	30	13	2	715.95557	1430.90386	-18.76	29.70	2965	2965	MS2	15/16	Glioma_Nuclear_prep_Gradeall_4plex.raw
4507401		High	eLYIQHAk	Unambiguous	2	1	N-Term(iTRAQ4plex); K8(iTRAQ4plex)	HCD	1.0000	0.0000	1	1	7820	Unique	Used	1.296	1.023E4	1.445E4	332	3.66	0.00	0	53	300	2	645.37793	1289.74858	2.31	22.04	2136	2136	MS2	14/14	Glioma_Nuclear_prep_Gradeall_repeat_4plex.raw
4507401	High		eLYIQHAk	Unambiguous	2	1	N-Term(iTRAQ4plex); K8(iTRAQ4plex)	HCD	1.0000	0.0000	1	1	7645	Unique	Used	1.749	9.887E4	1.897E5	332	3.40	0.00	0	20	15	2	645.37421	1289.74114	-3.47	23.85	2068	2068	MS2	14/14	Glioma_Nuclear_prep_Gradeall_4plex.raw
4507879	NP_003365.1	VDAC1	7416	voltage-dependent anion-selective channel protein 1 [Homo sapiens]	8.13	1	1	2	1.851	1;1	6.7	8.89	283	30.8	8.54																			
4507879		High	lTLSALLDGk	Unambiguous	1	1	N-Term(iTRAQ4plex); K10(iTRAQ4plex)	HCD	1.0000	0.0000	1	1	12193	Unique	Used	1.763	2.321E3	4.460E3	223	3.96	0.00	0	40	300	2	659.91144	1318.81560	-2.17	75.94	7086	7086	MS2	17/18	Glioma_Nuclear_prep_Gradeall_repeat_4plex.raw
4507879	High		lTLSALLDGk	Unambiguous	1	1	N-Term(iTRAQ4plex); K10(iTRAQ4plex)	HCD	1.0000	0.0000	1	1	11509	Unique	Used	1.939	5.850E4	1.245E5	179	4.38	0.00	0	50	43	2	659.90796	1318.80864	-7.45	52.68	6278	6278	MS2	17/18	Glioma_Nuclear_prep_Gradeall_4plex.raw
4557321	NP_000030.1	APOA1	335	apolipoprotein A-I preproprotein [Homo sapiens]	24.72	1	5	10	1.961	6;4	25.3	31.76	267	30.8	5.76																			
4557321		High	lSPLGEEMR	Unambiguous	1	1	N-Term(iTRAQ4plex)	HCD	1.0000	0.0000	1	1	9384	Unique	Used	1.622	1.429E4	2.526E4	251	3.48	0.00	0	23	227	2	588.31262	1175.61797	-2.69	37.98	3788	3788	MS2	13/16	Glioma_Nuclear_prep_Gradeall_repeat_4plex.raw
4557321		High	vQPYLDDFQk	Unambiguous	1	1	N-Term(iTRAQ4plex); K10(iTRAQ4plex)	HCD	1.0000	0.0000	1	1	10395	Unique	Used	1.985	6.077E3	1.314E4	253	4.13	0.00	0	31	300	2	770.91699	1540.82671	1.11	49.77	4876	4876	MS2	15/18	Glioma_Nuclear_prep_Gradeall_repeat_4plex.raw
4557321	High		vQPYLDDFQk	Unambiguous	1	1	N-Term(iTRAQ4plex); K10(iTRAQ4plex)	HCD	1.0000	0.0000	1	1	9704	Unique	Used	1.930	6.022E4	1.275E5	207	3.58	0.00	0	28	26	2	770.90442	1540.80156	-15.21	38.71	4308	4308	MS2	15/18	Glioma_Nuclear_prep_Gradeall_4plex.raw
4557321		High	qGLLPVLESFk	Unambiguous	1	1	N-Term(iTRAQ4plex); K11(iTRAQ4plex)	HCD	1.0000	0.0000	1	1	12399	Unique	Used	3.231	4.076E2	1.435E3	123	2.89	0.00	0	3	300	2	759.96045	1518.91362	0.12	83.45	7626	7626	MS2	14/20	Glioma_Nuclear_prep_Gradeall_repeat_4plex.raw
4557321	High		qGLLPVLESFk	Unambiguous	1	1	N-Term(iTRAQ4plex); K11(iTRAQ4plex)	HCD	1.0000	0.0000	1	1	12051	Unique	Used	2.167	3.230E3	7.683E3	128	4.65	0.00	0	21	84	3	506.97488	1518.91010	-2.20	57.49	6868	6868	MS2	21/40	Glioma_Nuclear_prep_Gradeall_4plex.raw
4557321	High		qGLLPVLESFk	Unambiguous	1	1	N-Term(iTRAQ4plex); K11(iTRAQ4plex)	HCD	1.0000	0.0000	1	1	12061	Unique	Used	2.767	2.444E4	7.420E4	127	4.11	0.00	0	6	54	2	759.95996	1518.91265	-0.52	57.59	6879	6879	MS2	18/20	Glioma_Nuclear_prep_Gradeall_4plex.raw
4557321	High		vSFLSALEEYTk	Unambiguous	1	1	N-Term(iTRAQ4plex); K12(iTRAQ4plex)	HCD	1.0000	0.0000	1	1	12533	Unique	Used	1.575	5.378E2	9.292E2	214	4.29	0.00	0	29	204	3	558.97760	1674.91825	-0.63	62.80	7404	7404	MS2	16/44	Glioma_Nuclear_prep_Gradeall_4plex.raw
4557321	High		vSFLSALEEYTk	Unambiguous	1	1	N-Term(iTRAQ4plex); K12(iTRAQ4plex)	HCD	1.0000	0.0000	1	1	12537	Unique	Used	2.702	8.540E3	2.532E4	221	5.74	0.00	0	19	237	2	837.96204	1674.91680	-1.49	62.86	7409	7409	MS2	20/22	Glioma_Nuclear_prep_Gradeall_4plex.raw
4557321		High	dYVSQFEGSALGk	Unambiguous	1	1	N-Term(iTRAQ4plex); K13(iTRAQ4plex)	HCD	1.0000	0.0000	1	1	10947	Unique	Used	1.780	1.954E3	3.792E3	181	5.05	0.00	0	37	300	2	844.94214	1688.87700	2.12	56.51	5474	5474	MS2	22/24	Glioma_Nuclear_prep_Gradeall_repeat_4plex.raw
4557321	High		dYVSQFEGSALGk	Unambiguous	1	1	N-Term(iTRAQ4plex); K13(iTRAQ4plex)	HCD	1.0000	0.0000	1	1	10261	Unique	Used	1.937	7.440E4	1.581E5	170	5.00	0.00	0	16	14	2	844.92902	1688.85076	-13.42	42.80	4914	4914	MS2	22/24	Glioma_Nuclear_prep_Gradeall_4plex.raw
4557325	NP_000032.1	APOE	348	apolipoprotein E isoform b precursor [Homo sapiens]	20.82	2	5	5	2.183	1;4	18.6	19.77	317	36.1	5.73																			
4557325		High	lAVYQAGAR	Unambiguous	2	1	N-Term(iTRAQ4plex)	HCD	1.0000	0.0000	1	1	8260	Unique	Used	1.797	2.641E4	5.171E4	367	2.87	0.00	0	25	135	2	546.81750	1092.62773	-0.47	26.29	2600	2600	MS2	14/16	Glioma_Nuclear_prep_Gradeall_repeat_4plex.raw
4557325		High	aATVGSLAGQPLQER	Unambiguous	2	1	N-Term(iTRAQ4plex)	HCD	1.0000	0.0000	1	1	9275	Unique	Used	2.183	1.604E3	3.816E3	211	4.93	0.00	0	5	300	2	821.45648	1641.90569	0.95	36.80	3673	3673	MS2	22/28	Glioma_Nuclear_prep_Gradeall_repeat_4plex.raw
4557325		High	sELEEQLTPVAEETR	Unambiguous	2	1	N-Term(iTRAQ4plex)	HCD	1.0000	0.0000	1	1	10167	Unique	Used	2.552	1.093E3	3.039E3	215	4.94	0.00	0	17	300	2	937.97705	1874.94682	0.20	47.07	4633	4633	MS2	23/28	Glioma_Nuclear_prep_Gradeall_repeat_4plex.raw
4557325	High		sWFEPLVEDMQR	Unambiguous	2	1	N-Term(iTRAQ4plex)	HCD	1.0000	0.0000	1	1	12203	Unique	Used	2.571	8.386E3	2.366E4	144	4.27	0.00	0	45	267	2	840.91602	1680.82475	4.44	59.02	7035	7035	MS2	17/22	Glioma_Nuclear_prep_Gradeall_4plex.raw
4557325		High	gEVQAmLGQSTEELR	Unambiguous	2	1	N-Term(iTRAQ4plex); M6(Oxidation)	HCD	1.0000	0.0000	1	1	9314	Unique	Used	1.723	1.381E3	2.594E3	184	3.58	0.00	0	20	300	2	904.45416	1807.90105	1.84	37.25	3714	3714	MS2	18/28	Glioma_Nuclear_prep_Gradeall_repeat_4plex.raw
4758138	NP_004387.1	DDX5	1655	probable ATP-dependent RNA helicase DDX5 [Homo sapiens]	25.08	6	8	12	0.450	5;7	35.7	57.08	614	69.1	8.92																			
4758138		High	lLQLVEDR	Unambiguous	1	1	N-Term(iTRAQ4plex)	HCD	1.0000	0.0000	1	1	11231	Unique	Used	0.242	3.162E4	8.336E3	268	3.30	0.00	0	5	152	2	565.33978	1129.67229	2.22	60.20	5797	5797	MS2	14/14	Glioma_Nuclear_prep_Gradeall_repeat_4plex.raw
4758138		High	nFYQEHPDLAR	Unambiguous	1	1	N-Term(iTRAQ4plex)	HCD	1.0000	0.0000	1	1	8607	Unique	Used	0.483	1.197E4	6.305E3	175	4.21	0.00	0	37	108	3	511.92468	1533.75949	1.83	29.72	2966	2966	MS2	21/40	Glioma_Nuclear_prep_Gradeall_repeat_4plex.raw
4758138		High	qVSDLISVLR	Unambiguous	1	1	N-Term(iTRAQ4plex)	HCD	1.0000	0.0000	1	1	11777	Unique	Used	0.494	8.649E3	4.655E3	196	2.90	0.00	0	40	300	2	637.38483	1273.76238	2.13	68.17	6469	6469	MS2	14/18	Glioma_Nuclear_prep_Gradeall_repeat_4plex.raw
4758138		High	tTYLVLDEADR	Unambiguous	1	1	N-Term(iTRAQ4plex)	HCD	1.0000	0.0000	1	1	10144	Unique	Used	0.552	1.267E4	7.620E3	260	4.32	0.00	0	27	300	2	720.38013	1439.75298	2.15	46.81	4609	4609	MS2	18/20	Glioma_Nuclear_prep_Gradeall_repeat_4plex.raw
4758138		High	fVINYDYPNSSEDYIHR	Unambiguous	1	1	N-Term(iTRAQ4plex)	HCD	1.0000	0.0000	1	1	11084	Unique	Used	0.638	4.716E2	3.277E2	105	4.40	0.00	0	55	300	3	759.36365	2276.07639	1.02	58.23	5626	5626	MS2	19/64	Glioma_Nuclear_prep_Gradeall_repeat_4plex.raw
4758138	High		lLQLVEDR	Unambiguous	1	1	N-Term(iTRAQ4plex)	HCD	1.0000	0.0000	1	1	10349	Unique	Used	0.237	7.002E5	1.823E5	196	3.14	0.00	0	7	4	2	565.33069	1129.65410	-13.88	43.45	5010	5010	MS2	14/14	Glioma_Nuclear_prep_Gradeall_4plex.raw
4758138	High		tTYLVLDEADR	Unambiguous	1	1	N-Term(iTRAQ4plex)	HCD	1.0000	0.0000	1	1	9593	Unique	Used	0.627	3.629E5	2.497E5	169	2.98	0.00	0	23	7	2	720.36810	1439.72893	-14.55	37.92	4188	4188	MS2	16/20	Glioma_Nuclear_prep_Gradeall_4plex.raw
4758138	High		eLAQQVQQVAAEYcR	Unambiguous	1	1	N-Term(iTRAQ4plex); C14(Methylthio)	HCD	1.0000	0.0000	1	1	12581	Unique	Used	0.295	2.013E4	6.526E3	162	6.33	0.00	0	34	150	2	963.46948	1925.93169	-0.70	63.42	7459	7459	MS2	24/28	Glioma_Nuclear_prep_Gradeall_4plex.raw
4758138	High		eLAQQVQQVAAEYcR	Unambiguous	1	1	N-Term(iTRAQ4plex); C14(Methylthio)	HCD	1.0000	0.0000	1	1	12530	Unique	Used	0.417	1.088E4	4.982E3	172	6.13	0.00	0	13	300	2	963.47015	1925.93303	-0.01	62.75	7400	7400	MS2	24/28	Glioma_Nuclear_prep_Gradeall_4plex.raw
4758138		High	lIDFLEcGk	Unambiguous	1	1	N-Term(iTRAQ4plex); C7(Methylthio); K9(iTRAQ4plex)	HCD	1.0000	0.0000	1	1	12428	Unique	Used	0.352	3.346E3	1.283E3	197	3.36	0.00	0	42	300	2	686.36798	1371.72869	2.32	85.65	7774	7774	MS2	14/16	Glioma_Nuclear_prep_Gradeall_repeat_4plex.raw
4758138	High		lIDFLEcGk	Unambiguous	1	1	N-Term(iTRAQ4plex); C7(Methylthio); K9(iTRAQ4plex)	HCD	1.0000	0.0000	1	1	12296	Unique	Used	0.294	1.923E5	6.196E4	202	3.75	0.00	0	27	32	2	686.36743	1371.72759	1.52	60.00	7138	7138	MS2	14/16	Glioma_Nuclear_prep_Gradeall_4plex.raw
4758138		High	tIVFVETk	Unambiguous	1	1	N-Term(iTRAQ4plex); K8(iTRAQ4plex)	HCD	1.0000	0.0000	1	1	9624	Unique	Used	0.664	1.566E4	1.133E4	205	3.53	0.00	0	62	300	2	612.87482	1224.74236	-1.53	40.67	4049	4049	MS2	13/14	Glioma_Nuclear_prep_Gradeall_repeat_4plex.raw
4758876	NP_004634.1	PABPN1	8106	polyadenylate-binding protein 2 [Homo sapiens]	9.15	4	3	4	0.488	1;3	29.1	11.38	306	32.7	5.06																			
4758876		High	tSLALDESLFR	Unambiguous	2	1	N-Term(iTRAQ4plex)	HCD	1.0000	0.0000	1	1	11514	Unique	Used	0.418	7.059E3	3.217E3	344	4.21	0.00	0	14	300	2	698.38568	1395.76409	2.88	64.32	6144	6144	MS2	17/20	Glioma_Nuclear_prep_Gradeall_repeat_4plex.raw
4758876		High	vTILcDk	Unambiguous	4	1	N-Term(iTRAQ4plex); C5(Methylthio); K7(iTRAQ4plex)	HCD	1.0000	0.0000	1	1	10299	Unique	Used	0.399	1.551E4	6.741E3	284	2.96	0.00	0	53	300	2	563.31512	1125.62297	-1.84	48.65	4774	4774	MS2	12/12	Glioma_Nuclear_prep_Gradeall_repeat_4plex.raw
4758876		High	gFAYIEFSDk	Unambiguous	2	1	N-Term(iTRAQ4plex); K10(iTRAQ4plex)	HCD	1.0000	0.0000	1	1	11155	Unique	Used	0.730	3.317E3	2.640E3	220	4.21	0.00	0	53	300	2	732.88672	1464.76616	3.29	59.19	5709	5709	MS2	18/18	Glioma_Nuclear_prep_Gradeall_repeat_4plex.raw
4758876	High		gFAYIEFSDk	Unambiguous	2	1	N-Term(iTRAQ4plex); K10(iTRAQ4plex)	HCD	1.0000	0.0000	1	1	10297	Unique	Used	0.559	1.643E5	1.007E5	159	3.75	0.00	0	41	20	2	732.87317	1464.73906	-15.21	43.06	4953	4953	MS2	17/18	Glioma_Nuclear_prep_Gradeall_4plex.raw
4758950	NP_000933.1	PPIB	5479	peptidyl-prolyl cis-trans isomerase B precursor [Homo sapiens]	14.81	1	3	6	1.627	3;3	35.5	12.70	216	23.7	9.41																			
4758950		High	tVDNFVALATGEk	Unambiguous	1	1	N-Term(iTRAQ4plex); K13(iTRAQ4plex)	HCD	1.0000	0.0000	1	1	11678	Unique	Used	1.410	1.584E3	2.435E3	306	4.89	0.00	0	59	300	2	826.95856	1652.90984	0.01	66.67	6343	6343	MS2	21/24	Glioma_Nuclear_prep_Gradeall_repeat_4plex.raw
4758950	High		tVDNFVALATGEk	Unambiguous	1	1	N-Term(iTRAQ4plex); K13(iTRAQ4plex)	HCD	1.0000	0.0000	1	1	10866	Unique	Used	1.682	3.351E4	6.185E4	247	4.81	0.00	0	33	44	2	826.94623	1652.88518	-14.91	47.41	5575	5575	MS2	22/24	Glioma_Nuclear_prep_Gradeall_4plex.raw
4758950		High	iEVEkPFAIAk	Unambiguous	1	1	N-Term(iTRAQ4plex); K5(iTRAQ4plex); K11(iTRAQ4plex)	HCD	1.0000	0.0000	1	1	10284	Unique	Used	1.501	1.960E3	3.206E3	57	4.33	0.00	0	50	300	2	839.02039	1677.03349	1.39	48.45	4757	4757	MS2	19/20	Glioma_Nuclear_prep_Gradeall_repeat_4plex.raw
4758950	High		iEVEkPFAIAk	Unambiguous	1	1	N-Term(iTRAQ4plex); K5(iTRAQ4plex); K11(iTRAQ4plex)	HCD	1.0000	0.0000	1	1	9516	Unique	Used	1.573	6.192E4	1.069E5	133	4.79	0.00	0	12	15	2	839.00519	1677.00310	-16.73	37.36	4104	4104	MS2	19/20	Glioma_Nuclear_prep_Gradeall_4plex.raw

4758950		High	vIFGLFGk	Unambiguous	1	1	N-Term(iTRAQ4plex); K8(iTRAQ4plex)	HCD	1.0000	0.0000	1	1	12214	Unique	Used	3.106	1.785E3	6.042E3	143	3.10	0.00	0	59	300	2	584.86835	1168.72942	-3.30	76.51	7129	7129	MS2	13/14	Glioma_Nuclear_prep_Gradeall_repeat_4plex.raw
4758950	High		vIFGLFGk	Unambiguous	1	1	N-Term(iTRAQ4plex); K8(iTRAQ4plex)	HCD	1.0000	0.0000	1	1	11570	Unique	Used	2.604	4.998E4	1.428E5	132	3.09	0.00	0	40	33	2	584.86908	1168.73088	-2.05	53.17	6344	6344	MS2	13/14	Glioma_Nuclear_prep_Gradeall_4plex.raw
4759156	NP_004587.1	SNRPA	6626	U1 small nuclear ribonucleoprotein A [Homo sapiens]	13.48	1	2	3	0.569	2;1	16.9	13.17	282	31.3	9.83																			
4759156		High	eVSSATNALR	Unambiguous	1	1	N-Term(iTRAQ4plex)	HCD	1.0000	0.0000	1	1	7810	Unique	Used	0.569	7.221E4	4.481E4	281	3.17	0.00	0	8	46	2	596.32782	1191.64836	2.80	21.96	2126	2126	MS2	18/18	Glioma_Nuclear_prep_Gradeall_repeat_4plex.raw
4759156	High		eVSSATNALR	Unambiguous	1	1	N-Term(iTRAQ4plex)	HCD	1.0000	0.0000	1	1	7701	Unique	Used	0.751	4.215E5	3.473E5	274	3.44	0.00	0	11	5	2	596.32599	1191.64470	-0.27	24.25	2129	2129	MS2	18/18	Glioma_Nuclear_prep_Gradeall_4plex.raw
4759156	High		sMQGFPFYDkPMR	Unambiguous	1	1	N-Term(iTRAQ4plex); K10(iTRAQ4plex)	HCD	1.0000	0.0000	1	1	9808	Unique	Used	0.564	5.122E4	3.170E4	102	3.98	0.00	0	26	9	3	631.30994	1891.91526	-15.08	39.46	4421	4421	MS2	23/48	Glioma_Nuclear_prep_Gradeall_4plex.raw
4759160	NP_004166.1	SNRPD3	6634	small nuclear ribonucleoprotein Sm D3 [Homo sapiens]	31.75	1	2	10	0.602	6;4	13.7	29.83	126	13.9	10.32																			
4759160		High	vAQLEQVYIR	Unambiguous	1	1	N-Term(iTRAQ4plex)	HCD	1.0000	0.0000	1	1	10475	Unique	Used	0.600	1.551E4	1.015E4	276	4.14	0.00	0	38	300	2	681.89813	1362.78899	2.04	50.72	4961	4961	MS2	17/18	Glioma_Nuclear_prep_Gradeall_repeat_4plex.raw
4759160		High	vAQLEQVYIR	Unambiguous	1	1	N-Term(iTRAQ4plex)	HCD	1.0000	0.0000	1	1	10544	Unique	Used	0.604	1.236E4	8.132E3	347	3.28	0.00	0	55	300	2	681.89709	1362.78691	0.52	51.57	5036	5036	MS2	15/18	Glioma_Nuclear_prep_Gradeall_repeat_4plex.raw
4759160	High		vAQLEQVYIR	Unambiguous	1	1	N-Term(iTRAQ4plex)	HCD	1.0000	0.0000	1	1	9758	Unique	Used	0.752	2.980E5	2.458E5	349	3.89	0.00	0	28	8	2	681.88660	1362.76592	-14.89	39.10	4367	4367	MS2	17/18	Glioma_Nuclear_prep_Gradeall_4plex.raw
4759160		High	fLILPDMLk	Unambiguous	1	1	N-Term(iTRAQ4plex); K9(iTRAQ4plex)	HCD	1.0000	0.0000	1	1	12441	Unique	Used	0.492	1.611E3	8.633E2	204	3.17	0.00	0	17	300	2	689.42499	1377.84270	0.61	86.40	7826	7826	MS2	14/16	Glioma_Nuclear_prep_Gradeall_repeat_4plex.raw
4759160	High		fLILPDMLk	Unambiguous	1	1	N-Term(iTRAQ4plex); K9(iTRAQ4plex)	HCD	1.0000	0.0000	1	1	12304	Unique	Used	0.511	2.770E5	1.552E5	192	3.40	0.00	0	7	16	2	689.42462	1377.84197	0.08	60.09	7147	7147	MS2	15/16	Glioma_Nuclear_prep_Gradeall_4plex.raw
4759160	High		fLILPDMLk	Unambiguous	1	1	N-Term(iTRAQ4plex); K9(iTRAQ4plex)	HCD	1.0000	0.0000	1	1	12365	Unique	Used	0.549	2.899E4	1.747E4	200	3.58	0.00	0	29	166	2	689.42529	1377.84331	1.05	60.76	7214	7214	MS2	15/16	Glioma_Nuclear_prep_Gradeall_4plex.raw
4759160	High		fLILPDMLk	Unambiguous	1	1	N-Term(iTRAQ4plex); K9(iTRAQ4plex)	HCD	1.0000	0.0000	1	1	12279	Unique	Used	0.580	5.722E4	3.643E4	193	3.43	0.00	0	50	85	2	689.42456	1377.84184	-0.01	59.82	7119	7119	MS2	15/16	Glioma_Nuclear_prep_Gradeall_4plex.raw
4759160		High	fLILPDmLk	Unambiguous	1	1	N-Term(iTRAQ4plex); M7(Oxidation); K9(iTRAQ4plex)	HCD	1.0000	0.0000	1	1	12006	Unique	Used	0.655	3.635E3	2.596E3	212	2.94	0.00	0	29	300	2	697.42291	1393.83855	1.27	72.14	6790	6790	MS2	14/16	Glioma_Nuclear_prep_Gradeall_repeat_4plex.raw
4759160	High		fLILPDmLk	Unambiguous	1	1	N-Term(iTRAQ4plex); M7(Oxidation); K9(iTRAQ4plex)	HCD	1.0000	0.0000	1	1	11251	Unique	Used	0.659	6.841E4	4.947E4	210	2.99	0.00	0	52	63	2	697.42224	1393.83721	0.31	50.45	5995	5995	MS2	13/16	Glioma_Nuclear_prep_Gradeall_4plex.raw
4759160	High		fLILPDmLk	Unambiguous	1	1	N-Term(iTRAQ4plex); M7(Oxidation); K9(iTRAQ4plex)	HCD	1.0000	0.0000	1	1	11196	Unique	Used	0.710	7.140E4	5.562E4	257	3.06	0.00	0	27	36	2	697.41687	1393.82646	-7.40	49.99	5935	5935	MS2	15/16	Glioma_Nuclear_prep_Gradeall_4plex.raw
4759302	NP_004729.1	VAPB	9217	vesicle-associated membrane protein-associated protein B/C isoform 1 [Homo sapiens]	15.64	2	2	3	0.657	2;1	4.0	13.73	243	27.2	7.30																			
4759302		High	sLSSSLDDTEVk	Unambiguous	1	1	N-Term(iTRAQ4plex); K12(iTRAQ4plex)	HCD	1.0000	0.0000	1	1	9072	Unique	Used	0.625	1.130E4	7.693E3	257	5.03	0.00	0	20	275	2	784.91711	1568.82695	0.74	34.55	3459	3459	MS2	22/22	Glioma_Nuclear_prep_Gradeall_repeat_4plex.raw
4759302	High		sLSSSLDDTEVk	Unambiguous	1	1	N-Term(iTRAQ4plex); K12(iTRAQ4plex)	HCD	1.0000	0.0000	1	1	8685	Unique	Used	0.657	1.566E5	1.129E5	212	4.97	0.00	0	39	19	2	784.90491	1568.80254	-14.83	31.26	3197	3197	MS2	22/22	Glioma_Nuclear_prep_Gradeall_4plex.raw
4759302	High		vEQVLSLEPQHELk	Unambiguous	2	1	N-Term(iTRAQ4plex); K14(iTRAQ4plex)	HCD	1.0000	0.0000	1	1	9664	Unique	Used	0.676	8.615E4	6.389E4	144	3.98	0.00	0	52	9	3	646.36121	1937.06906	-13.21	38.43	4265	4265	MS2	24/52	Glioma_Nuclear_prep_Gradeall_4plex.raw
4826998	NP_005057.1	SFPQ	6421	splicing factor, proline- and glutamine-rich [Homo sapiens]	27.72	1	15	35	0.588	21;14	20.3	134.65	707	76.1	9.44																			
4826998		High	fATHAAALSVR	Unambiguous	1	1	N-Term(iTRAQ4plex)	HCD	1.0000	0.0000	1	1	8434	Unique	Used	0.439	4.043E4	1.932E4	271	3.69	0.00	0	11	132	2	644.37061	1287.73393	3.81	27.97	2783	2783	MS2	18/20	Glioma_Nuclear_prep_Gradeall_repeat_4plex.raw
4826998		High	fGQGGAGPVGGQGPR	Unambiguous	1	1	N-Term(iTRAQ4plex)	HCD	1.0000	0.0000	1	1	8034	Unique	Used	0.502	4.335E4	2.372E4	167	4.90	0.00	0	9	66	2	743.38483	1485.76238	-3.77	24.12	2362	2362	MS2	25/28	Glioma_Nuclear_prep_Gradeall_repeat_4plex.raw
4826998		High	fAQHGTFEYEYSQR	Unambiguous	1	1	N-Term(iTRAQ4plex)	HCD	1.0000	0.0000	1	1	9290	Unique	Used	0.588	4.027E3	2.581E3	83	4.89	0.00	0	13	300	2	953.94720	1906.88713	1.60	36.97	3689	3689	MS2	20/26	Glioma_Nuclear_prep_Gradeall_repeat_4plex.raw
4826998		High	fATHAAALSVR	Unambiguous	1	1	N-Term(iTRAQ4plex)	HCD	1.0000	0.0000	1	1	8424	Unique	Used	0.651	8.088E3	5.735E3	253	4.07	0.00	0	39	133	3	429.91443	1287.72873	-0.23	27.86	2772	2772	MS2	21/40	Glioma_Nuclear_prep_Gradeall_repeat_4plex.raw
4826998		High	fAQHGTFEYEYSQR	Unambiguous	1	1	N-Term(iTRAQ4plex)	HCD	1.0000	0.0000	1	1	9282	Unique	Used	0.670	3.606E3	2.634E3	51	5.60	0.00	0	22	146	3	636.30115	1906.88889	2.52	36.89	3681	3681	MS2	22/52	Glioma_Nuclear_prep_Gradeall_repeat_4plex.raw
4826998	High		nLSPYVSNELLEEAFSQFGPIER	Unambiguous	1	1	N-Term(iTRAQ4plex)	HCD	1.0000	0.0000	1	1	13108	Unique	Used	0.351	1.036E3	3.986E2	107	5.80	0.00	0	0	300	2	1392.20593	2783.40459	1.31	73.67	8257	8257	MS2	27/44	Glioma_Nuclear_prep_Gradeall_4plex.raw
4826998	High		fATHAAALSVR	Unambiguous	1	1	N-Term(iTRAQ4plex)	HCD	1.0000	0.0000	1	1	8079	Unique	Used	0.448	2.339E5	1.149E5	283	3.72	0.00	0	19	13	2	644.35724	1287.70720	-16.96	26.91	2538	2538	MS2	18/20	Glioma_Nuclear_prep_Gradeall_4plex.raw
4826998	High		fGQGGAGPVGGQGPR	Unambiguous	1	1	N-Term(iTRAQ4plex)	HCD	1.0000	0.0000	1	1	7871	Unique	Used	0.473	4.284E5	2.222E5	199	4.82	0.00	0	8	8	2	743.38818	1485.76909	0.75	25.45	2313	2313	MS2	24/28	Glioma_Nuclear_prep_Gradeall_4plex.raw
4826998	High		fAQHGTFEYEYSQR	Unambiguous	1	1	N-Term(iTRAQ4plex)	HCD	1.0000	0.0000	1	1	8719	Unique	Used	0.553	1.067E5	6.475E4	49	4.89	0.00	0	23	16	2	953.93140	1906.85552	-14.98	31.51	3234	3234	MS2	20/26	Glioma_Nuclear_prep_Gradeall_4plex.raw
4826998	High		qHHPPYHQQHHQGPPPGGPGGR	Unambiguous	1	1	N-Term(iTRAQ4plex)	HCD	1.0000	0.0000	1	1	6874	Unique	Used	0.555	3.727E2	2.268E2	78	5.12	0.00	0	8	300	3	849.75104	2547.23856	0.54	17.08	1226	1226	MS2	23/84	Glioma_Nuclear_prep_Gradeall_4plex.raw
4826998	High		rEEEMMIR	Unambiguous	1	1	N-Term(iTRAQ4plex)	HCD	1.0000	0.0000	1	1	7550	Unique	Used	0.720	7.660E4	6.049E4	179	2.99	0.00	1	34	17	2	619.31152	1237.61577	0.61	23.17	1965	1965	MS2	12/14	Glioma_Nuclear_prep_Gradeall_4plex.raw
4826998		High	yGEPGEVFINk	Unambiguous	1	1	N-Term(iTRAQ4plex); K11(iTRAQ4plex)	HCD	1.0000	0.0000	1	1	10080	Unique	Used	0.661	8.100E3	5.835E3	227	3.89	0.00	0	39	300	2	770.91559	1540.82390	-0.72	46.07	4541	4541	MS2	18/20	Glioma_Nuclear_prep_Gradeall_repeat_4plex.raw
4826998		High	yGEPGEVFINk	Unambiguous	1	1	N-Term(iTRAQ4plex); K11(iTRAQ4plex)	HCD	1.0000	0.0000	1	1	10181	Unique	Used	0.696	4.896E3	3.713E3	306	3.81	0.00	0	25	300	2	770.91669	1540.82610	0.71	47.24	4648	4648	MS2	16/20	Glioma_Nuclear_prep_Gradeall_repeat_4plex.raw
4826998		High	mEELHNQEMQk	Unambiguous	1	1	N-Term(iTRAQ4plex); K11(iTRAQ4plex)	HCD	1.0000	0.0000	1	1	7957	Unique	Used	0.722	5.737E3	4.514E3	83	4.27	0.00	0	23	115	3	568.94672	1704.82560	-1.88	23.39	2281	2281	MS2	21/40	Glioma_Nuclear_prep_Gradeall_repeat_4plex.raw
4826998		High	mEELHNQEMQk	Unambiguous	1	1	N-Term(iTRAQ4plex); K11(iTRAQ4plex)	HCD	1.0000	0.0000	1	1	7970	Unique	Used	0.773	2.671E3	2.251E3	98	3.32	0.00	0	0	300	2	852.91644	1704.82561	-1.88	23.52	2294	2294	MS2	20/20	Glioma_Nuclear_prep_Gradeall_repeat_4plex.raw
4826998	High		mEELHNQEMQk	Unambiguous	1	1	N-Term(iTRAQ4plex); K11(iTRAQ4plex)	HCD	1.0000	0.0000	1	1	7766	Unique	Used	0.602	1.186E5	7.838E4	89	5.15	0.00	0	33	13	2	852.91797	1704.82866	-0.09	24.70	2199	2199	MS2	19/20	Glioma_Nuclear_prep_Gradeall_4plex.raw
4826998	High		yGEPGEVFINk	Unambiguous	1	1	N-Term(iTRAQ4plex); K11(iTRAQ4plex)	HCD	1.0000	0.0000	1	1	9473	Unique	Used	0.607	4.329E5	2.885E5	189	3.45	0.00	0	15	6	2	770.90540	1540.80351	-13.95	37.06	4058	4058	MS2	20/20	Glioma_Nuclear_prep_Gradeall_4plex.raw
4826998	High		rMEELHNQEMQk	Unambiguous	1	1	N-Term(iTRAQ4plex); K12(iTRAQ4plex)	HCD	1.0000	0.0000	1	1	7440	Unique	Used	0.541	7.512E4	4.461E4	147	4.90	0.00	1	7	16	2	930.96503	1860.92278	-3.84	22.39	1846	1846	MS2	19/22	Glioma_Nuclear_prep_Gradeall_4plex.raw
4826998		High	lFVGNLPADITEDEFkR	Unambiguous	1	1	N-Term(iTRAQ4plex); K16(iTRAQ4plex)	HCD	1.0000	0.0000	1	1	12096	Unique	Used	0.474	1.178E3	6.091E2	149	6.05	0.00	1	18	300	3	751.41180	2252.22086	1.89	74.16	6944	6944	MS2	30/64	Glioma_Nuclear_prep_Gradeall_repeat_4plex.raw
4826998		High	lFVGNLPADITEDEFk	Unambiguous	1	1	N-Term(iTRAQ4plex); K16(iTRAQ4plex)	HCD	1.0000	0.0000	1	1	12384	Unique	Used	0.749	1.535E3	1.253E3	194	5.50	0.00	0	2	300	2	1048.56165	2096.11601	0.26	82.30	7548	7548	MS2	22/30	Glioma_Nuclear_prep_Gradeall_repeat_4plex.raw
4826998	High		lFVGNLPADITEDEFkR	Unambiguous	1	1	N-Term(iTRAQ4plex); K16(iTRAQ4plex)	HCD	1.0000	0.0000	1	1	11326	Unique	Used	0.399	4.720E4	2.068E4	143	6.25	0.00	1	11	42	2	1126.61157	2252.21587	-0.32	51.09	6077	6077	MS2	22/32	Glioma_Nuclear_prep_Gradeall_4plex.raw
4826998	High		lFVGNLPADITEDEFkR	Unambiguous	1	1	N-Term(iTRAQ4plex); K16(iTRAQ4plex)	HCD	1.0000	0.0000	1	1	11363	Unique	Used	0.444	4.003E4	1.948E4	154	6.01	0.00	1	12	12	3	751.40985	2252.21500	-0.71	51.44	6118	6118	MS2	29/64	Glioma_Nuclear_prep_Gradeall_4plex.raw
4826998	High		lFVGNLPADITEDEFkR	Unambiguous	1	1	N-Term(iTRAQ4plex); K16(iTRAQ4plex)	HCD	1.0000	0.0000	1	1	11272	Unique	Used	0.444	3.200E4	1.561E4	146	5.63	0.00	1	10	19	3	751.41168	2252.22049	1.73	50.63	6018	6018	MS2	28/64	Glioma_Nuclear_prep_Gradeall_4plex.raw
4826998	High		lFVGNLPADITEDEFk	Unambiguous	1	1	N-Term(iTRAQ4plex); K16(iTRAQ4plex)	HCD	1.0000	0.0000	1	1	12060	Unique	Used	0.600	1.242E5	8.179E4	204	6.23	0.00	0	5	30	2	1048.56287	2096.11846	1.42	57.59	6878	6878	MS2	22/30	Glioma_Nuclear_prep_Gradeall_4plex.raw
4826998	High		lFVGNLPADITEDEFk	Unambiguous	1	1	N-Term(iTRAQ4plex); K16(iTRAQ4plex)	HCD	1.0000	0.0000	1	1	12127	Unique	Used	0.608	5.699E4	3.802E4	192	5.69	0.00	0	4	50	2	1048.56824	2096.12920	6.55	58.28	6952	6952	MS2	22/30	Glioma_Nuclear_prep_Gradeall_4plex.raw
4826998	High		lFVGNLPADITEDEFk	Unambiguous	1	1	N-Term(iTRAQ4plex); K16(iTRAQ4plex)	HCD	1.0000	0.0000	1	1	12126	Unique	Used	0.761	1.333E4	1.112E4	194	5.23	0.00	0	6	22	3	699.38196	2096.13132	7.56	58.28	6951	6951	MS2	26/60	Glioma_Nuclear_prep_Gradeall_4plex.raw
4826998		High	mGGGGAMNMGDPYGSGGQk	Unambiguous	1	1	N-Term(iTRAQ4plex); K19(iTRAQ4plex)	HCD	1.0000	0.0000	1	1	9048	Unique	Used	0.405	1.930E3	8.524E2	43	4.69	0.00	0	10	300	2	1030.46448	2059.92168	-1.08	34.28	3433	3433	MS2	24/36	Glioma_Nuclear_prep_Gradeall_repeat_4plex.raw
4826998	High		mGGGGAMNMGDPYGSGGQk	Unambiguous	1	1	N-Term(iTRAQ4plex); K19(iTRAQ4plex)	HCD	1.0000	0.0000	1	1	8718	Unique	Used	0.447	1.180E5	5.785E4	19	6.86	0.00	0	0	14	2	1030.44849	2059.88970	-16.60	31.50	3233	3233	MS2	24/36	Glioma_Nuclear_prep_Gradeall_4plex.raw
4826998	High		dkLESEMEDAYHEHQANLLR	Unambiguous	1	1	N-Term(iTRAQ4plex); K2(iTRAQ4plex)	HCD	1.0000	0.0000	1	1	9924	Unique	Used	0.643	9.345E3	6.590E3	53	3.63	0.00	1	5	9	3	906.10065	2716.28739	-13.47	40.30	4547	4547	MS2	29/76	Glioma_Nuclear_prep_Gradeall_4plex.raw
4826998		High	eEYEGPNk	Unambiguous	1	1	N-Term(iTRAQ4plex); K8(iTRAQ4plex)	HCD	1.0000	0.0000	1	1	7194	Unique	Used	0.577	6.112E4	3.846E4	108	3.00	0.00	0	14	51	2	627.30585	1253.60442	-16.60	16.49	1474	1474	MS2	13/14	Glioma_Nuclear_prep_Gradeall_repeat_4plex.raw
4826998		High	iSDSEGFk	Unambiguous	1	1	N-Term(iTRAQ4plex); K8(iTRAQ4plex)	HCD	1.0000	0.0000	1	1	8169	Unique	Used	0.681	9.243E4	6.865E4	181	3.06	0.00	0	17	58	2	585.81378	1170.62029	-3.61	25.41	2504	2504	MS2	14/14	Glioma_Nuclear_prep_Gradeall_repeat_4plex.raw
4826998	High		rMEELHNQEmQk	Unambiguous	1	1	N-Term(iTRAQ4plex); M10(Oxidation); K12(iTRAQ4plex)	HCD	1.0000	0.0000	1	1	7191	Unique	Used	0.564	2.587E3	1.602E3	88	4.09	0.00	1	25	35	4	469.98114	1876.90273	-11.78	20.48	1573	1573	MS2	18/66	Glioma_Nuclear_prep_Gradeall_4plex.raw
4826998	High		rMEELHNQEmQk	Unambiguous	1	1	N-Term(iTRAQ4plex); M10(Oxidation); K12(iTRAQ4plex)	HCD	1.0000	0.0000	1	1	7204	Unique	Used	0.704	5.195E3	4.013E3	141	3.84	0.00	1	17	13	4	469.98645	1876.92397	-0.46	20.58	1587	1587	MS2	20/66	Glioma_Nuclear_prep_Gradeall_4plex.raw
4826998	High		rMEELHNQEmQk	Unambiguous	1	1	N-Term(iTRAQ4plex); M10(Oxidation); K12(iTRAQ4plex)	HCD	1.0000	0.0000	1	1	7189	Unique	Used	0.747	5.961E3	4.887E3	82	3.92	0.00	1	16	29	3	626.30682	1876.90592	-10.08	20.47	1571	1571	MS2	20/44	Glioma_Nuclear_prep_Gradeall_4plex.raw
4826998	High		mEELHNQEmQk	Unambiguous	1	1	N-Term(iTRAQ4plex); M9(Oxidation); K11(iTRAQ4plex)	HCD	1.0000	0.0000	1	1	7385	Unique	Used	0.530	2.791E4	1.624E4	91	3.35	0.00	0	8	38	2	860.91339	1720.81951	-2.45	21.98	1786	1786	MS2	17/20	Glioma_Nuclear_prep_Gradeall_4plex.raw
4885281	NP_005262.1	GLUD1	2746	glutamate dehydrogenase 1, mitochondrial precursor [Homo sapiens]	9.86	2	4	5	2.417	1;4	32.5	15.27	558	61.4	7.80																			
4885281		High	hGGTIPIVPTAEFQDR	Unambiguous	1	1	N-Term(iTRAQ4plex)	HCD	1.0000	0.0000	1	1	10222	Unique	Used	1.296	7.798E2	1.101E3	190	3.86	0.00	0	39	300	3	628.00293	1881.99424	0.12	47.71	4691	4691	MS2	19/60	Glioma_Nuclear_prep_Gradeall_repeat_4plex.raw
4885281		High	hGGTIPIVPTAEFQDR	Unambiguous	1	1	N-Term(iTRAQ4plex)	HCD	1.0000	0.0000	1	1	10229	Unique	Used	3.212	7.776E2	2.723E3	279	3.80	0.00	0	7	300	2	941.50061	1881.99394	-0.03	47.79	4698	4698	MS2	15/30	Glioma_Nuclear_prep_Gradeall_repeat_4plex.raw
4885281	High		gFIGPGIDVPAPDMSTGER	Unambiguous	1	1	N-Term(iTRAQ4plex)	HCD	1.0000	0.0000	1	1	10770	Unique	Used	2.450	9.304E3	2.502E4	138	5.32	0.00	0	29	62	2	1030.49817	2059.98906	-16.97	46.69	5471	5471	MS2	24/36	Glioma_Nuclear_prep_Gradeall_4plex.raw
4885281		High	cAVVDVPFGGAk	Unambiguous	2	1	N-Term(iTRAQ4plex); C1(Methylthio); K12(iTRAQ4plex)	HCD	1.0000	0.0000	1	1	11258	Unique	Used	1.799	2.339E3	4.586E3	256	4.25	0.00	0	46	300	2	748.89655	1496.78581	0.93	60.54	5827	5827	MS2	17/22	Glioma_Nuclear_prep_Gradeall_repeat_4plex.raw
4885281		High	gASIVEDk	Unambiguous	2	1	N-Term(iTRAQ4plex); K8(iTRAQ4plex)	HCD	1.0000	0.0000	1	1	7565	Unique	Used	2.417	1.963E4	5.169E4	282	3.36	0.00	0	51	166	2	553.81757	1106.62786	-1.57	19.64	1863	1863	MS2	14/14	Glioma_Nuclear_prep_Gradeall_repeat_4plex.raw
4885371	NP_005309.1	H1F0	3005	histone H1.0 [Homo sapiens]	24.23	1	4	30	1.713	12;18	19.6	89.21	194	20.9	10.84																			
4885371		High	gVGASGSFR	Unambiguous	1	1	N-Term(iTRAQ4plex)	HCD	1.0000	0.0000	1	1	7307	Unique	Used	2.038	1.445E5	3.210E5	186	3.36	0.00	0	6	10	2	491.26401	981.52074	-2.77	17.49	1593	1593	MS2	15/16	Glioma_Nuclear_prep_Gradeall_repeat_4plex.raw
4885371		High	gVGASGSFR	Unambiguous	1	1	N-Term(iTRAQ4plex)	HCD	1.0000	0.0000	1	1	7233	Unique	Used	2.142	6.077E4	1.419E5	162	3.18	0.00	0	20	32	2	491.25702	981.50676	-17.01	16.83	1515	1515	MS2	15/16	Glioma_Nuclear_prep_Gradeall_repeat_4plex.raw
4885371	High		gVGASGSFR	Unambiguous	1	1	N-Term(iTRAQ4plex)	HCD	1.0000	0.0000	1	1	7353	Unique	Used	2.112	6.485E5	1.503E6	153	3.36	0.00	0	13	2	2	491.26276	981.51824	-5.32	21.76	1752	1752	MS2	15/16	Glioma_Nuclear_prep_Gradeall_4plex.raw
4885371		High	vGENADSQlk	Unambiguous	1	1	N-Term(iTRAQ4plex); K10(iTRAQ4plex)	HCD	1.0000	0.0000	1	1	7521	Unique	Used	1.312	1.496E4	2.139E4	246	3.90	0.00	0	34	103	2	674.86218	1348.71709	-10.39	19.28	1817	1817	MS2	17/18	Glioma_Nuclear_prep_Gradeall_repeat_4plex.raw
4885371		High	vGENADSQlk	Unambiguous	1	1	N-Term(iTRAQ4plex); K10(iTRAQ4plex)	HCD	1.0000	0.0000	1	1	7447	Unique	Used	1.447	1.664E5	2.624E5	270	4.01	0.00	0	6	8	2	674.86908	1348.73088	-0.17	18.66	1740	1740	MS2	18/18	Glioma_Nuclear_prep_Gradeall_repeat_4plex.raw
4885371		High	vGENADSQlk	Unambiguous	1	1	N-Term(iTRAQ4plex); K10(iTRAQ4plex)	HCD	1.0000	0.0000	1	1	7387	Unique	Used	1.785	1.113E5	2.166E5	203	3.35	0.00	0	4	12	2	674.85858	1348.70989	-15.73	18.14	1677	1677	MS2	18/18	Glioma_Nuclear_prep_Gradeall_repeat_4plex.raw
4885371	High		vGENADSQlk	Unambiguous	1	1	N-Term(iTRAQ4plex); K10(iTRAQ4plex)	HCD	1.0000	0.0000	1	1	7435	Unique	Used	1.728	3.857E5	7.314E5	229	4.30	0.00	0	6	3	2	674.86560	1348.72392	-5.33	22.36	1841	1841	MS2	18/18	Glioma_Nuclear_prep_Gradeall_4plex.raw
4885371		High	ySDMIVAAIQAEk	Unambiguous	1	1	N-Term(iTRAQ4plex); K13(iTRAQ4plex)	HCD	1.0000	0.0000	1	1	12155	Unique	Used	1.336	4.268E2	6.215E2	213	4.56	0.00	0	33	300	3	576.31421	1726.92807	-0.44	75.18	7026	7026	MS2	24/48	Glioma_Nuclear_prep_Gradeall_repeat_4plex.raw
4885371		High	ySDMIVAAIQAEk	Unambiguous	1	1	N-Term(iTRAQ4plex); K13(iTRAQ4plex)	HCD	1.0000	0.0000	1	1	12204	Unique	Used	1.471	1.056E3	1.693E3	251	5.24	0.00	0	6	104	3	576.31494	1726.93027	0.83	76.17	7104	7104	MS2	24/48	Glioma_Nuclear_prep_Gradeall_repeat_4plex.raw
4885371		High	ySDMIVAAIQAEk	Unambiguous	1	1	N-Term(iTRAQ4plex); K13(iTRAQ4plex)	HCD	1.0000	0.0000	1	1	12162	Unique	Used	1.610	9.800E2	1.720E3	255	5.03	0.00	0	15	300	2	863.96899	1726.93071	1.09	75.29	7035	7035	MS2	21/24	Glioma_Nuclear_prep_Gradeall_repeat_4plex.raw
4885371		High	ySDMIVAAIQAEk	Unambiguous	1	1	N-Term(iTRAQ4plex); K13(iTRAQ4plex)	HCD	1.0000	0.0000	1	1	12239	Unique	Used	1.698	2.588E2	4.789E2	243	5.17	0.00	0	10	300	3	576.31451	1726.92899	0.09	77.18	7180	7180	MS2	24/48	Glioma_Nuclear_prep_Gradeall_repeat_4plex.raw
4885371		High	ySDMIVAAIQAEk	Unambiguous	1	1	N-Term(iTRAQ4plex); K13(iTRAQ4plex)	HCD	1.0000	0.0000	1	1	12206	Unique	Used	1.950	3.854E3	8.189E3	223	5.57	0.00	0	7	300	2	863.96924	1726.93120	1.37	76.28	7112	7112	MS2	22/24	Glioma_Nuclear_prep_Gradeall_repeat_4plex.raw
4885371		High	ySDMIVAAIQAEk	Unambiguous	1	1	N-Term(iTRAQ4plex); K13(iTRAQ4plex)	HCD	1.0000	0.0000	1	1	12270	Unique	Used	2.577	6.670E1	1.873E2	233	4.08	0.00	0	13	300	3	576.31390	1726.92716	-0.97	78.20	7254	7254	MS2	22/48	Glioma_Nuclear_prep_Gradeall_repeat_4plex.raw
4885371	High		ySDMIVAAIQAEk	Unambiguous	1	1	N-Term(iTRAQ4plex); K13(iTRAQ4plex)	HCD	1.0000	0.0000	1	1	11727	Unique	Used	1.316	9.232E4	1.333E5	217	5.65	0.00	0	36	20	2	863.96637	1726.92546	-1.95	54.58	6515	6515	MS2	21/24	Glioma_Nuclear_prep_Gradeall_4plex.raw
4885371	High		ySDMIVAAIQAEk	Unambiguous	1	1	N-Term(iTRAQ4plex); K13(iTRAQ4plex)	HCD	1.0000	0.0000	1	1	11919	Unique	Used	1.371	9.433E3	1.419E4	273	5.32	0.00	0	35	262	2	863.97235	1726.93743	4.97	56.20	6723	6723	MS2	21/24	Glioma_Nuclear_prep_Gradeall_4plex.raw
4885371	High		ySDMIVAAIQAEk	Unambiguous	1	1	N-Term(iTRAQ4plex); K13(iTRAQ4plex)	HCD	1.0000	0.0000	1	1	11814	Unique	Used	1.386	2.014E4	3.064E4	259	5.56	0.00	0	42	90	2	863.96844	1726.92961	0.45	55.32	6609	6609	MS2	21/24	Glioma_Nuclear_prep_Gradeall_4plex.raw
4885371	High		ySDMIVAAIQAEk	Unambiguous	1	1	N-Term(iTRAQ4plex); K13(iTRAQ4plex)	HCD	1.0000	0.0000	1	1	11569	Unique	Used	1.609	6.296E4	1.112E5	231	5.64	0.00	0	32	34	2	863.96802	1726.92876	-0.05	53.17	6343	6343	MS2	21/24	Glioma_Nuclear_prep_Gradeall_4plex.raw
4885371	High		ySDMIVAAIQAEk	Unambiguous	1	1	N-Term(iTRAQ4plex); K13(iTRAQ4plex)	HCD	1.0000	0.0000	1	1	11651	Unique	Used	1.832	8.794E5	1.768E6	229	5.69	0.00	0	7	2	2	863.96979	1726.93230	2.00	53.87	6432	6432	MS2	21/24	Glioma_Nuclear_prep_Gradeall_4plex.raw
4885371	High		ySDMIVAAIQAEk	Unambiguous	1	1	N-Term(iTRAQ4plex); K13(iTRAQ4plex)	HCD	1.0000	0.0000	1	1	11650	Unique	Used	2.130	1.123E5	2.624E5	240	5.58	0.00	0	7	1	3	576.31567	1726.93247	2.10	53.87	6431	6431	MS2	24/48	Glioma_Nuclear_prep_Gradeall_4plex.raw
4885371	High		ySDMIVAAIQAEk	Unambiguous	1	1	N-Term(iTRAQ4plex); K13(iTRAQ4plex)	HCD	1.0000	0.0000	1	1	11726	Unique	Used	2.215	8.059E3	1.959E4	235	5.14	0.00	0	7	7	3	576.31329	1726.92533	-2.03	54.57	6514	6514	MS2	24/48	Glioma_Nuclear_prep_Gradeall_4plex.raw
4885371		High	lVTTGVLk	Unambiguous	1	1	N-Term(iTRAQ4plex); K8(iTRAQ4plex)	HCD	1.0000	0.0000	1	1	9196	Unique	Used	1.458	2.412E3	3.834E3	70	3.47	0.00	0	26	253	3	373.58450	1118.73896	0.19	35.92	3590	3590	MS2	14/28	Glioma_Nuclear_prep_Gradeall_repeat_4plex.raw
4885371		High	lVTTGVLk	Unambiguous	1	1	N-Term(iTRAQ4plex); K8(iTRAQ4plex)	HCD	1.0000	0.0000	1	1	9263	Unique	Used	1.835	2.502E5	5.004E5	126	3.02	0.00	0	7	10	2	559.87469	1118.74211	3.01	36.67	3661	3661	MS2	14/14	Glioma_Nuclear_prep_Gradeall_repeat_4plex.raw
4885371		High	lVTTGVLk	Unambiguous	1	1	N-Term(iTRAQ4plex); K8(iTRAQ4plex)	HCD	1.0000	0.0000	1	1	9171	Unique	Used	2.143	5.275E4	1.232E5	102	3.14	0.00	0	10	34	2	559.87085	1118.73442	-3.87	35.66	3564	3564	MS2	14/14	Glioma_Nuclear_prep_Gradeall_repeat_4plex.raw
4885371		High	ySDmIVAAIQAEk	Unambiguous	1	1	N-Term(iTRAQ4plex); M4(Oxidation); K13(iTRAQ4plex)	HCD	1.0000	0.0000	1	1	11481	Unique	Used	1.489	2.813E2	4.564E2	212	4.29	0.00	0	45	300	3	581.64587	1742.92307	-0.39	63.89	6107	6107	MS2	24/48	Glioma_Nuclear_prep_Gradeall_repeat_4plex.raw
4885371		High	ySDmIVAAIQAEk	Unambiguous	1	1	N-Term(iTRAQ4plex); M4(Oxidation); K13(iTRAQ4plex)	HCD	1.0000	0.0000	1	1	11405	Unique	Used	1.566	3.890E3	6.640E3	217	4.88	0.00	0	5	254	2	871.96661	1742.92595	1.26	62.87	6019	6019	MS2	20/24	Glioma_Nuclear_prep_Gradeall_repeat_4plex.raw
4885371		High	ySDmIVAAIQAEk	Unambiguous	1	1	N-Term(iTRAQ4plex); M4(Oxidation); K13(iTRAQ4plex)	HCD	1.0000	0.0000	1	1	11329	Unique	Used	1.873	2.250E3	4.592E3	209	5.23	0.00	0	11	300	2	871.96704	1742.92681	1.75	61.83	5929	5929	MS2	21/24	Glioma_Nuclear_prep_Gradeall_repeat_4plex.raw
4885371		High	ySDmIVAAIQAEk	Unambiguous	1	1	N-Term(iTRAQ4plex); M4(Oxidation); K13(iTRAQ4plex)	HCD	1.0000	0.0000	1	1	11394	Unique	Used	2.387	1.369E3	3.561E3	229	5.08	0.00	0	5	54	3	581.64703	1742.92655	1.60	62.71	6005	6005	MS2	25/48	Glioma_Nuclear_prep_Gradeall_repeat_4plex.raw
4885371	High		ySDmIVAAIQAEk	Unambiguous	1	1	N-Term(iTRAQ4plex); M4(Oxidation); K13(iTRAQ4plex)	HCD	1.0000	0.0000	1	1	10662	Unique	Used	1.421	1.136E5	1.772E5	187	4.31	0.00	0	16	9	2	871.95343	1742.89958	-13.87	45.87	5354	5354	MS2	20/24	Glioma_Nuclear_prep_Gradeall_4plex.raw
4885371	High		ySDmIVAAIQAEk	Unambiguous	1	1	N-Term(iTRAQ4plex); M4(Oxidation); K13(iTRAQ4plex)	HCD	1.0000	0.0000	1	1	10652	Unique	Used	1.533	1.744E4	2.934E4	212	3.68	0.00	0	11	3	3	581.63721	1742.89707	-15.31	45.80	5343	5343	MS2	25/48	Glioma_Nuclear_prep_Gradeall_4plex.raw
4885371	High		ySDmIVAAIQAEk	Unambiguous	1	1	N-Term(iTRAQ4plex); M4(Oxidation); K13(iTRAQ4plex)	HCD	1.0000	0.0000	1	1	10569	Unique	Used	1.735	2.705E5	5.149E5	197	4.54	0.00	0	7	4	2	871.95294	1742.89861	-14.43	45.16	5252	5252	MS2	22/24	Glioma_Nuclear_prep_Gradeall_4plex.raw
4885375	NP_005310.1	HIST1H1C	3006	histone H1.2 [Homo sapiens]	36.62	2	3	9	3.478	4;5	34.2	188.38	213	21.4	10.93																			
4885375		High	kAAGGATPk	Unambiguous	1	1	N-Term(iTRAQ4plex); K1(iTRAQ4plex); K9(iTRAQ4plex)	HCD	1.0000	0.0000	1	1	6415	Unique	Used	1.380	3.698E3	5.560E3	188	4.45	0.00	1	28	58	3	411.59473	1232.76963	0.74	9.38	651	651	MS2	27/32	Glioma_Nuclear_prep_Gradeall_repeat_4plex.raw
4885375		High	kPAAATVTk	Unambiguous	1	1	N-Term(iTRAQ4plex); K1(iTRAQ4plex); K9(iTRAQ4plex)	HCD	1.0000	0.0000	1	1	7032	Unique	Used	1.505	6.980E3	1.145E4	124	4.50	0.00	0	56	112	3	440.28391	1318.83716	-3.58	15.08	1301	1301	MS2	22/32	Glioma_Nuclear_prep_Gradeall_repeat_4plex.raw
4885375		High	kPAAATVTk	Unambiguous	1	1	N-Term(iTRAQ4plex); K1(iTRAQ4plex); K9(iTRAQ4plex)	HCD	1.0000	0.0000	1	1	6860	Unique	Used	3.383	1.191E4	4.391E4	124	4.62	0.00	0	39	39	3	440.28333	1318.83542	-4.90	13.53	1120	1120	MS2	23/32	Glioma_Nuclear_prep_Gradeall_repeat_4plex.raw
4885375		High	kPAAATVTk	Unambiguous	1	1	N-Term(iTRAQ4plex); K1(iTRAQ4plex); K9(iTRAQ4plex)	HCD	1.0000	0.0000	1	1	6868	Unique	Used	4.225	3.128E4	1.440E5	115	2.94	0.00	0	34	157	2	659.92169	1318.83611	-4.38	13.59	1128	1128	MS2	14/16	Glioma_Nuclear_prep_Gradeall_repeat_4plex.raw
4885375	High		kPAAATVTk	Unambiguous	1	1	N-Term(iTRAQ4plex); K1(iTRAQ4plex); K9(iTRAQ4plex)	HCD	1.0000	0.0000	1	1	7198	Unique	Used	3.309	5.368E4	1.949E5	117	4.61	0.00	0	11	3	3	440.28479	1318.83982	-1.56	20.55	1581	1581	MS2	23/32	Glioma_Nuclear_prep_Gradeall_4plex.raw
4885375	High		kPAAATVTk	Unambiguous	1	1	N-Term(iTRAQ4plex); K1(iTRAQ4plex); K9(iTRAQ4plex)	HCD	1.0000	0.0000	1	1	7200	Unique	Used	3.478	9.019E4	3.442E5	111	2.95	0.00	0	10	7	2	659.92352	1318.83977	-1.60	20.56	1583	1583	MS2	15/16	Glioma_Nuclear_prep_Gradeall_4plex.raw
4885375	High		kPAAATVTk	Unambiguous	1	1	N-Term(iTRAQ4plex); K1(iTRAQ4plex); K9(iTRAQ4plex)	HCD	1.0000	0.0000	1	1	7163	Unique	Used	4.032	4.943E4	2.187E5	157	3.11	0.00	0	15	31	2	659.91516	1318.82305	-14.28	20.27	1543	1543	MS2	14/16	Glioma_Nuclear_prep_Gradeall_4plex.raw
4885375	High		kPAAATVTk	Unambiguous	1	1	N-Term(iTRAQ4plex); K1(iTRAQ4plex); K9(iTRAQ4plex)	HCD	1.0000	0.0000	1	1	7161	Unique	Used	4.557	1.717E4	8.585E4	170	4.43	0.00	0	14	10	3	440.27896	1318.82233	-14.82	20.25	1541	1541	MS2	23/32	Glioma_Nuclear_prep_Gradeall_4plex.raw
4885375		High	aAGGATPk	Unambiguous	1	1	N-Term(iTRAQ4plex); K8(iTRAQ4plex)	HCD	1.0000	0.0000	1	1	6453	Unique	Used	3.824	1.625E4	6.774E4	228	2.93	0.00	0	7	91	2	480.78970	960.57213	0.46	9.77	691	691	MS2	13/14	Glioma_Nuclear_prep_Gradeall_repeat_4plex.raw
4885377	NP_005311.1	HIST1H1D	3007	histone H1.3 [Homo sapiens]	30.77	2	1	2	2.183	1;1	11.9	176.89	221	22.3	11.02																			
4885377		High	kVAGAATPk	Unambiguous	1	1	N-Term(iTRAQ4plex); K1(iTRAQ4plex); K9(iTRAQ4plex)	HCD	1.0000	0.0000	1	1	6656	Unique	Used	1.999	4.418E3	9.624E3	103	4.72	0.00	1	26	42	3	425.60880	1274.81183	-3.01	11.72	906	906	MS2	22/32	Glioma_Nuclear_prep_Gradeall_repeat_4plex.raw
4885377	High		kVAGAATPk	Unambiguous	1	1	N-Term(iTRAQ4plex); K1(iTRAQ4plex); K9(iTRAQ4plex)	HCD	1.0000	0.0000	1	1	7019	Unique	Used	2.367	3.655E3	9.494E3	109	4.53	0.00	1	23	41	3	425.61038	1274.81659	0.72	18.75	1385	1385	MS2	23/32	Glioma_Nuclear_prep_Gradeall_4plex.raw
4885381	NP_005313.1	HIST1H1B	3009	histone H1.5 [Homo sapiens]	37.61	1	7	27	2.815	11;16	39.2	153.21	226	22.6	10.92																			
4885381		High	kALAAGGYDVEk	Unambiguous	1	1	N-Term(iTRAQ4plex); K1(iTRAQ4plex); K12(iTRAQ4plex)	HCD	1.0000	0.0000	1	1	8183	Unique	Used	1.540	6.946E3	1.166E4	214	5.96	0.00	1	12	19	3	551.98767	1653.94846	-3.13	25.56	2519	2519	MS2	28/44	Glioma_Nuclear_prep_Gradeall_repeat_4plex.raw
4885381		High	kALAAGGYDVEk	Unambiguous	1	1	N-Term(iTRAQ4plex); K1(iTRAQ4plex); K12(iTRAQ4plex)	HCD	1.0000	0.0000	1	1	8201	Unique	Used	1.704	4.562E3	8.471E3	198	4.31	0.00	1	15	57	2	827.48254	1653.95781	2.53	25.75	2538	2538	MS2	22/22	Glioma_Nuclear_prep_Gradeall_repeat_4plex.raw
4885381	High		kALAAGGYDVEk	Unambiguous	1	1	N-Term(iTRAQ4plex); K1(iTRAQ4plex); K12(iTRAQ4plex)	HCD	1.0000	0.0000	1	1	7929	Unique	Used	2.950	2.082E4	6.740E4	183	4.31	0.00	1	3	5	2	827.48077	1653.95427	0.39	25.86	2376	2376	MS2	22/22	Glioma_Nuclear_prep_Gradeall_4plex.raw
4885381		High	kATGPPVSELITk	Unambiguous	1	1	N-Term(iTRAQ4plex); K1(iTRAQ4plex); K13(iTRAQ4plex)	HCD	1.0000	0.0000	1	1	9147	Unique	Used	1.925	1.221E3	2.563E3	76	4.67	0.00	1	79	300	2	887.04419	1773.08110	-2.00	35.39	3538	3538	MS2	20/24	Glioma_Nuclear_prep_Gradeall_repeat_4plex.raw
4885381		High	kATGPPVSELITk	Unambiguous	1	1	N-Term(iTRAQ4plex); K1(iTRAQ4plex); K13(iTRAQ4plex)	HCD	1.0000	0.0000	1	1	8610	Unique	Used	1.995	1.495E3	3.251E3	49	4.61	0.00	1	43	140	3	591.70020	1773.08603	0.78	29.74	2969	2969	MS2	18/48	Glioma_Nuclear_prep_Gradeall_repeat_4plex.raw
4885381		High	kATGPPVSELITk	Unambiguous	1	1	N-Term(iTRAQ4plex); K1(iTRAQ4plex); K13(iTRAQ4plex)	HCD	1.0000	0.0000	1	1	9230	Unique	Used	2.815	3.850E2	1.181E3	52	5.17	0.00	1	6	89	4	444.02740	1773.08779	1.77	36.29	3626	3626	MS2	23/72	Glioma_Nuclear_prep_Gradeall_repeat_4plex.raw
4885381	High		kATGPPVSELITk	Unambiguous	1	1	N-Term(iTRAQ4plex); K1(iTRAQ4plex); K13(iTRAQ4plex)	HCD	1.0000	0.0000	1	1	8513	Unique	Used	2.469	1.712E4	4.638E4	117	5.41	0.00	1	7	2	3	591.68878	1773.05179	-18.53	30.00	3009	3009	MS2	22/48	Glioma_Nuclear_prep_Gradeall_4plex.raw
4885381	High		kATGPPVSELITk	Unambiguous	1	1	N-Term(iTRAQ4plex); K1(iTRAQ4plex); K13(iTRAQ4plex)	HCD	1.0000	0.0000	1	1	8681	Unique	Used	4.033	2.414E4	1.068E5	95	4.56	0.00	1	4	8	2	887.03210	1773.05693	-15.63	31.24	3193	3193	MS2	21/24	Glioma_Nuclear_prep_Gradeall_4plex.raw
4885381	High		kATGPPVSELITk	Unambiguous	1	1	N-Term(iTRAQ4plex); K1(iTRAQ4plex); K13(iTRAQ4plex)	HCD	1.0000	0.0000	1	1	8497	Unique	Used	5.368	1.368E4	8.061E4	103	4.40	0.00	1	8	15	2	887.03076	1773.05425	-17.15	29.87	2991	2991	MS2	21/24	Glioma_Nuclear_prep_Gradeall_4plex.raw
4885381		High	kPAAAGVk	Unambiguous	1	1	N-Term(iTRAQ4plex); K1(iTRAQ4plex); K8(iTRAQ4plex)	HCD	1.0000	0.0000	1	1	6827	Unique	Used	1.669	7.043E3	1.281E4	89	3.43	0.00	0	61	88	3	391.92816	1173.76993	1.65	13.25	1085	1085	MS2	18/28	Glioma_Nuclear_prep_Gradeall_repeat_4plex.raw
4885381	High		kPAAAGVk	Unambiguous	1	1	N-Term(iTRAQ4plex); K1(iTRAQ4plex); K8(iTRAQ4plex)	HCD	1.0000	0.0000	1	1	7109	Unique	Used	2.982	2.360E3	7.721E3	83	3.43	0.00	0	43	108	3	391.92831	1173.77039	2.04	19.74	1484	1484	MS2	20/28	Glioma_Nuclear_prep_Gradeall_4plex.raw
4885381		High	kAAGAGAAk	Unambiguous	1	1	N-Term(iTRAQ4plex); K1(iTRAQ4plex); K9(iTRAQ4plex)	HCD	1.0000	0.0000	1	1	6394	Unique	Used	3.347	9.348E2	3.410E3	114	4.06	0.00	1	21	85	3	392.91916	1176.74292	0.35	9.13	628	628	MS2	24/32	Glioma_Nuclear_prep_Gradeall_repeat_4plex.raw
4885381		High	kAAGAGAAk	Unambiguous	1	1	N-Term(iTRAQ4plex); K1(iTRAQ4plex); K9(iTRAQ4plex)	HCD	1.0000	0.0000	1	1	6396	Unique	Used	5.223	6.962E2	3.963E3	145	3.33	0.00	1	10	261	2	588.87482	1176.74236	-0.13	9.15	630	630	MS2	16/16	Glioma_Nuclear_prep_Gradeall_repeat_4plex.raw
4885381	High		kAAGAGAAk	Unambiguous	1	1	N-Term(iTRAQ4plex); K1(iTRAQ4plex); K9(iTRAQ4plex)	HCD	1.0000	0.0000	1	1	6725	Unique	Used	3.010	1.563E2	5.163E2	139	3.45	0.00	1	41	300	3	392.91870	1176.74155	-0.82	14.68	1023	1023	MS2	22/32	Glioma_Nuclear_prep_Gradeall_4plex.raw
4885381		High	aLAAGGYDVEk	Unambiguous	1	1	N-Term(iTRAQ4plex); K11(iTRAQ4plex)	HCD	1.0000	0.0000	1	1	8842	Unique	Used	3.836	6.042E3	2.526E4	236	4.34	0.00	0	20	120	2	691.38281	1381.75835	1.27	32.08	3213	3213	MS2	20/20	Glioma_Nuclear_prep_Gradeall_repeat_4plex.raw
4885381		High	aTGPPVSELITk	Unambiguous	1	1	N-Term(iTRAQ4plex); K12(iTRAQ4plex)	HCD	1.0000	0.0000	1	1	9162	Unique	Used	1.932	1.940E3	4.086E3	149	3.49	0.00	0	18	300	2	750.94934	1500.89141	2.52	35.55	3554	3554	MS2	15/22	Glioma_Nuclear_prep_Gradeall_repeat_4plex.raw
4885381		High	aTGPPVSELITk	Unambiguous	1	1	N-Term(iTRAQ4plex); K12(iTRAQ4plex)	HCD	1.0000	0.0000	1	1	9903	Unique	Used	2.102	2.716E3	6.223E3	192	3.74	0.00	0	89	300	2	750.94684	1500.88640	-0.81	43.82	4344	4344	MS2	16/22	Glioma_Nuclear_prep_Gradeall_repeat_4plex.raw
4885381		High	aTGPPVSELITk	Unambiguous	1	1	N-Term(iTRAQ4plex); K12(iTRAQ4plex)	HCD	1.0000	0.0000	1	1	10193	Unique	Used	2.480	3.088E3	8.348E3	155	4.77	0.00	0	26	300	2	750.94843	1500.88957	1.30	47.39	4661	4661	MS2	21/22	Glioma_Nuclear_prep_Gradeall_repeat_4plex.raw
4885381		High	aTGPPVSELITk	Unambiguous	1	1	N-Term(iTRAQ4plex); K12(iTRAQ4plex)	HCD	1.0000	0.0000	1	1	10106	Unique	Used	2.603	1.172E3	3.324E3	155	4.07	0.00	0	22	61	3	500.96735	1500.88749	-0.09	46.38	4569	4569	MS2	20/44	Glioma_Nuclear_prep_Gradeall_repeat_4plex.raw
4885381		High	aTGPPVSELITk	Unambiguous	1	1	N-Term(iTRAQ4plex); K12(iTRAQ4plex)	HCD	1.0000	0.0000	1	1	9925	Unique	Used	3.303	2.579E3	9.284E3	164	4.28	0.00	0	39	300	2	750.94489	1500.88249	-3.41	44.11	4369	4369	MS2	20/22	Glioma_Nuclear_prep_Gradeall_repeat_4plex.raw
4885381	High		aTGPPVSELITk	Unambiguous	1	1	N-Term(iTRAQ4plex); K12(iTRAQ4plex)	HCD	1.0000	0.0000	1	1	9410	Unique	Used	1.960	1.020E5	2.194E5	227	4.24	0.00	0	10	1	3	500.95880	1500.86185	-17.17	36.58	3989	3989	MS2	23/44	Glioma_Nuclear_prep_Gradeall_4plex.raw

4885381	High		aTGPPVSELITk	Unambiguous	1	1	N-Term(iTRAQ4plex); K12(iTRAQ4plex)	HCD	1.0000	0.0000	1	1	8539	Unique	Used	3.635	2.127E4	8.483E4	193	2.92	0.00	0	9	10	2	750.93536	1500.86345	-16.10	30.18	3037	3037	MS2	17/22	Glioma_Nuclear_prep_Gradeall_4plex.raw
4885381	High		aTGPPVSELITk	Unambiguous	1	1	N-Term(iTRAQ4plex); K12(iTRAQ4plex)	HCD	1.0000	0.0000	1	1	9290	Unique	Used	4.909	3.807E3	2.051E4	190	3.09	0.00	0	15	13	2	750.93335	1500.85942	-18.79	35.68	3857	3857	MS2	17/22	Glioma_Nuclear_prep_Gradeall_4plex.raw
4885381		High	nGLSLAALk	Unambiguous	1	1	N-Term(iTRAQ4plex); K9(iTRAQ4plex)	HCD	1.0000	0.0000	1	1	10215	Unique	Used	2.886	5.890E2	1.853E3	155	3.59	0.00	0	12	206	3	392.25165	1174.74039	0.49	47.64	4684	4684	MS2	18/32	Glioma_Nuclear_prep_Gradeall_repeat_4plex.raw
4885381		High	nGLSLAALk	Unambiguous	1	1	N-Term(iTRAQ4plex); K9(iTRAQ4plex)	HCD	1.0000	0.0000	1	1	10210	Unique	Used	4.317	7.155E3	3.367E4	162	3.76	0.00	0	5	41	2	587.87408	1174.74089	0.92	47.59	4679	4679	MS2	16/16	Glioma_Nuclear_prep_Gradeall_repeat_4plex.raw
4885381	High		nGLSLAALk	Unambiguous	1	1	N-Term(iTRAQ4plex); K9(iTRAQ4plex)	HCD	1.0000	0.0000	1	1	9484	Unique	Used	1.494	1.279E5	2.096E5	254	3.79	0.00	0	24	5	2	587.86414	1174.72099	-16.02	37.14	4070	4070	MS2	16/16	Glioma_Nuclear_prep_Gradeall_4plex.raw
4885381	High		nGLSLAALk	Unambiguous	1	1	N-Term(iTRAQ4plex); K9(iTRAQ4plex)	HCD	1.0000	0.0000	1	1	9579	Unique	Used	1.677	1.246E5	2.293E5	256	3.47	0.00	0	18	4	2	587.86420	1174.72112	-15.92	37.83	4173	4173	MS2	16/16	Glioma_Nuclear_prep_Gradeall_4plex.raw
5031755	NP_005817.1	HNRNPR	10236	heterogeneous nuclear ribonucleoprotein R isoform 2 [Homo sapiens]	36.33	7	16	50	0.392	28;22	35.1	179.51	633	70.9	8.13																			
5031755		High	sTAYEDYYYHPPPR	Unambiguous	7	1	N-Term(iTRAQ4plex)	HCD	1.0000	0.0000	1	1	9089	Unique	Used	0.306	6.727E3	2.241E3	51	6.05	0.00	0	0	300	2	951.94531	1902.88335	2.86	34.74	3477	3477	MS2	23/26	Glioma_Nuclear_prep_Gradeall_repeat_4plex.raw
5031755		High	sTAYEDYYYHPPPR	Unambiguous	7	1	N-Term(iTRAQ4plex)	HCD	1.0000	0.0000	1	1	9186	Unique	Used	0.381	1.932E3	8.018E2	49	5.56	0.00	0	10	300	2	951.93958	1902.87187	-3.17	35.81	3579	3579	MS2	23/26	Glioma_Nuclear_prep_Gradeall_repeat_4plex.raw
5031755		High	sTAYEDYYYHPPPR	Unambiguous	7	1	N-Term(iTRAQ4plex)	HCD	1.0000	0.0000	1	1	9079	Unique	Used	0.421	5.850E3	2.685E3	55	4.86	0.00	0	14	133	3	634.96637	1902.88456	3.50	34.64	3467	3467	MS2	26/52	Glioma_Nuclear_prep_Gradeall_repeat_4plex.raw
5031755		High	lMMDPLSGQNR	Unambiguous	6	1	N-Term(iTRAQ4plex)	HCD	1.0000	0.0000	1	1	10386	Unique	Used	0.424	2.170E4	1.002E4	188	3.74	0.00	0	16	258	2	703.35767	1405.70806	2.25	49.68	4867	4867	MS2	15/20	Glioma_Nuclear_prep_Gradeall_repeat_4plex.raw
5031755		High	sTAYEDYYYHPPPR	Unambiguous	7	1	N-Term(iTRAQ4plex)	HCD	1.0000	0.0000	1	1	9174	Unique	Used	0.473	3.866E3	1.991E3	44	4.63	0.00	0	12	115	3	634.96265	1902.87339	-2.37	35.68	3567	3567	MS2	27/52	Glioma_Nuclear_prep_Gradeall_repeat_4plex.raw
5031755		High	dYAFVHFEDR	Unambiguous	7	1	N-Term(iTRAQ4plex)	HCD	1.0000	0.0000	1	1	10110	Unique	Used	0.723	3.822E3	3.011E3	99	4.08	0.00	0	29	182	3	481.56610	1442.68375	1.12	46.41	4573	4573	MS2	21/36	Glioma_Nuclear_prep_Gradeall_repeat_4plex.raw
5031755		High	dYAFVHFEDR	Unambiguous	7	1	N-Term(iTRAQ4plex)	HCD	1.0000	0.0000	1	1	10117	Unique	Used	0.731	6.729E3	5.361E3	103	3.88	0.00	0	47	300	2	721.84625	1442.68523	2.14	46.48	4580	4580	MS2	16/18	Glioma_Nuclear_prep_Gradeall_repeat_4plex.raw
5031755	High		sTAYEDYYYHPPPR	Unambiguous	7	1	N-Term(iTRAQ4plex)	HCD	1.0000	0.0000	1	1	8609	Unique	Used	0.242	4.182E4	1.111E4	35	3.70	0.00	0	26	6	3	634.95398	1902.84739	-16.04	30.72	3115	3115	MS2	27/52	Glioma_Nuclear_prep_Gradeall_4plex.raw
5031755	High		sTAYEDYYYHPPPR	Unambiguous	7	1	N-Term(iTRAQ4plex)	HCD	1.0000	0.0000	1	1	8614	Unique	Used	0.280	1.003E5	3.085E4	33	5.67	0.00	0	0	21	2	951.92761	1902.84795	-15.74	30.75	3120	3120	MS2	23/26	Glioma_Nuclear_prep_Gradeall_4plex.raw
5031755	High		lMMDPLSGQNR	Unambiguous	6	1	N-Term(iTRAQ4plex)	HCD	1.0000	0.0000	1	1	9757	Unique	Used	0.434	8.422E5	4.011E5	121	2.97	0.00	0	15	4	2	703.34570	1405.68413	-14.77	39.10	4366	4366	MS2	15/20	Glioma_Nuclear_prep_Gradeall_4plex.raw
5031755	High		lcDSYEIRPGk	Unambiguous	6	1	N-Term(iTRAQ4plex); C2(Methylthio); K11(iTRAQ4plex)	HCD	1.0000	0.0000	1	1	9251	Unique	Used	0.377	8.473E4	3.507E4	88	3.41	0.00	0	21	31	2	807.90234	1614.79741	-15.38	35.37	3813	3813	MS2	16/20	Glioma_Nuclear_prep_Gradeall_4plex.raw
5031755		High	gYAFITFcGk	Unambiguous	6	1	N-Term(iTRAQ4plex); C8(Methylthio); K10(iTRAQ4plex)	HCD	1.0000	0.0000	1	1	12320	Unique	Used	0.428	3.241E3	1.511E3	180	3.65	0.00	0	28	300	2	720.86768	1440.72807	1.57	79.57	7356	7356	MS2	16/18	Glioma_Nuclear_prep_Gradeall_repeat_4plex.raw
5031755	High		gYAFITFcGk	Unambiguous	6	1	N-Term(iTRAQ4plex); C8(Methylthio); K10(iTRAQ4plex)	HCD	1.0000	0.0000	1	1	11751	Unique	Used	0.369	1.620E5	6.558E4	181	4.02	0.00	0	53	34	2	720.86652	1440.72576	-0.04	54.80	6541	6541	MS2	15/18	Glioma_Nuclear_prep_Gradeall_4plex.raw
5031755	High		gYAFITFcGk	Unambiguous	6	1	N-Term(iTRAQ4plex); C8(Methylthio); K10(iTRAQ4plex)	HCD	1.0000	0.0000	1	1	11831	Unique	Used	0.402	9.465E4	4.173E4	186	4.23	0.00	0	39	41	2	720.86713	1440.72698	0.80	55.47	6628	6628	MS2	15/18	Glioma_Nuclear_prep_Gradeall_4plex.raw
5031755		High	kYGGPPPDSVYSGVQPGIGTEVFVGk	Unambiguous	4	1	N-Term(iTRAQ4plex); K1(iTRAQ4plex); K26(iTRAQ4plex)	HCD	1.0000	0.0000	1	1	11224	Unique	Used	0.439	1.267E2	6.068E1	71	5.39	0.00	1	33	300	4	767.66901	3067.65420	2.49	60.11	5789	5789	MS2	21/150	Glioma_Nuclear_prep_Gradeall_repeat_4plex.raw
5031755		High	eSDLSHVQNk	Unambiguous	3	1	N-Term(iTRAQ4plex); K10(iTRAQ4plex)	HCD	1.0000	0.0000	1	1	7141	Unique	Used	0.504	7.755E3	4.260E3	193	4.80	0.00	0	39	214	2	722.87561	1444.74394	-13.51	16.06	1418	1418	MS2	17/18	Glioma_Nuclear_prep_Gradeall_repeat_4plex.raw
5031755		High	eSDLSHVQNk	Unambiguous	3	1	N-Term(iTRAQ4plex); K10(iTRAQ4plex)	HCD	1.0000	0.0000	1	1	7137	Unique	Used	0.612	9.583E3	6.390E3	159	3.49	0.00	0	29	84	3	482.25342	1444.74570	-12.29	16.03	1414	1414	MS2	18/36	Glioma_Nuclear_prep_Gradeall_repeat_4plex.raw
5031755		High	dLYEDELVPLFEk	Unambiguous	6	1	N-Term(iTRAQ4plex); K13(iTRAQ4plex)	HCD	1.0000	0.0000	1	1	12390	Unique	Used	0.304	1.683E3	5.583E2	179	4.97	0.00	0	5	300	2	949.50635	1898.00542	0.86	82.86	7586	7586	MS2	20/24	Glioma_Nuclear_prep_Gradeall_repeat_4plex.raw
5031755		High	nLATTVTEEILEk	Unambiguous	7	1	N-Term(iTRAQ4plex); K13(iTRAQ4plex)	HCD	1.0000	0.0000	1	1	12341	Unique	Used	0.334	2.261E3	8.220E2	220	4.93	0.00	0	13	300	2	874.99988	1748.99248	2.30	80.47	7419	7419	MS2	23/24	Glioma_Nuclear_prep_Gradeall_repeat_4plex.raw
5031755		High	dLYEDELVPLFEk	Unambiguous	6	1	N-Term(iTRAQ4plex); K13(iTRAQ4plex)	HCD	1.0000	0.0000	1	1	12407	Unique	Used	0.382	1.007E3	4.199E2	174	4.80	0.00	0	11	300	2	949.50726	1898.00725	1.83	83.87	7655	7655	MS2	19/24	Glioma_Nuclear_prep_Gradeall_repeat_4plex.raw
5031755		High	nLATTVTEEILEk	Unambiguous	7	1	N-Term(iTRAQ4plex); K13(iTRAQ4plex)	HCD	1.0000	0.0000	1	1	12338	Unique	Used	0.406	1.990E2	8.802E1	174	4.06	0.00	0	22	300	3	583.66815	1748.98990	0.83	80.35	7411	7411	MS2	24/48	Glioma_Nuclear_prep_Gradeall_repeat_4plex.raw
5031755		High	nLATTVTEEILEk	Unambiguous	7	1	N-Term(iTRAQ4plex); K13(iTRAQ4plex)	HCD	1.0000	0.0000	1	1	12369	Unique	Used	0.709	1.898E2	1.467E2	212	4.08	0.00	0	27	300	3	583.66895	1748.99228	2.19	81.41	7487	7487	MS2	23/48	Glioma_Nuclear_prep_Gradeall_repeat_4plex.raw
5031755	High		nLATTVTEEILEk	Unambiguous	7	1	N-Term(iTRAQ4plex); K13(iTRAQ4plex)	HCD	1.0000	0.0000	1	1	11945	Unique	Used	0.164	2.406E4	4.330E3	219	5.21	0.00	0	8	10	3	583.66882	1748.99192	1.98	56.45	6752	6752	MS2	26/48	Glioma_Nuclear_prep_Gradeall_4plex.raw
5031755	High		nLATTVTEEILEk	Unambiguous	7	1	N-Term(iTRAQ4plex); K13(iTRAQ4plex)	HCD	1.0000	0.0000	1	1	11946	Unique	Used	0.283	2.132E5	6.609E4	221	5.72	0.00	0	10	19	2	875.00085	1748.99443	3.42	56.46	6753	6753	MS2	23/24	Glioma_Nuclear_prep_Gradeall_4plex.raw
5031755	High		dLYEDELVPLFEk	Unambiguous	6	1	N-Term(iTRAQ4plex); K13(iTRAQ4plex)	HCD	1.0000	0.0000	1	1	12192	Unique	Used	0.314	2.451E5	8.449E4	168	5.45	0.00	0	6	15	2	949.51166	1898.01604	6.46	58.91	7023	7023	MS2	20/24	Glioma_Nuclear_prep_Gradeall_4plex.raw
5031755	High		nLATTVTEEILEk	Unambiguous	7	1	N-Term(iTRAQ4plex); K13(iTRAQ4plex)	HCD	1.0000	0.0000	1	1	11873	Unique	Used	0.320	8.651E4	3.035E4	220	5.81	0.00	0	20	85	2	874.99957	1748.99187	1.95	55.82	6673	6673	MS2	22/24	Glioma_Nuclear_prep_Gradeall_4plex.raw
5031755	High		dLYEDELVPLFEk	Unambiguous	6	1	N-Term(iTRAQ4plex); K13(iTRAQ4plex)	HCD	1.0000	0.0000	1	1	12114	Unique	Used	0.324	1.345E5	4.785E4	176	5.44	0.00	0	6	17	2	949.50867	1898.01006	3.31	58.17	6938	6938	MS2	20/24	Glioma_Nuclear_prep_Gradeall_4plex.raw
5031755	High		dLYEDELVPLFEk	Unambiguous	6	1	N-Term(iTRAQ4plex); K13(iTRAQ4plex)	HCD	1.0000	0.0000	1	1	12264	Unique	Used	0.501	1.111E4	6.109E3	222	5.31	0.00	0	53	300	2	949.50476	1898.00224	-0.81	59.64	7102	7102	MS2	19/24	Glioma_Nuclear_prep_Gradeall_4plex.raw
5031755	High		dLYEDELVPLFEk	Unambiguous	6	1	N-Term(iTRAQ4plex); K13(iTRAQ4plex)	HCD	1.0000	0.0000	1	1	12108	Unique	Used	0.583	3.750E3	2.399E3	153	3.68	0.00	0	37	146	3	633.34503	1898.02054	8.83	58.10	6931	6931	MS2	17/48	Glioma_Nuclear_prep_Gradeall_4plex.raw
5031755	High		eIEGEEIEIVLAkPPDk	Unambiguous	7	1	N-Term(iTRAQ4plex); K13(iTRAQ4plex); K17(iTRAQ4plex)	HCD	1.0000	0.0000	1	1	10389	Unique	Used	0.448	1.848E4	9.091E3	97	5.65	0.00	0	0	42	2	1171.14905	2341.29082	-13.64	43.75	5053	5053	MS2	24/32	Glioma_Nuclear_prep_Gradeall_4plex.raw
5031755	High		eFNEEGALSVLQQFk	Unambiguous	3	1	N-Term(iTRAQ4plex); K15(iTRAQ4plex)	HCD	1.0000	0.0000	1	1	12354	Unique	Used	0.312	4.249E4	1.453E4	187	7.26	0.00	0	4	72	2	1014.03790	2027.06853	-0.16	60.65	7202	7202	MS2	26/28	Glioma_Nuclear_prep_Gradeall_4plex.raw
5031755	High		eFNEEGALSVLQQFk	Unambiguous	3	1	N-Term(iTRAQ4plex); K15(iTRAQ4plex)	HCD	1.0000	0.0000	1	1	12337	Unique	Used	0.760	3.018E3	2.518E3	201	6.16	0.00	0	22	93	3	676.36096	2027.06833	-0.25	60.44	7183	7183	MS2	25/56	Glioma_Nuclear_prep_Gradeall_4plex.raw
5031755		High	vTEGLVDVILYHQPDDk	Unambiguous	5	1	N-Term(iTRAQ4plex); K17(iTRAQ4plex)	HCD	1.0000	0.0000	1	1	12178	Unique	Used	0.323	8.220E2	2.893E2	143	6.75	0.00	0	28	300	3	743.73944	2229.20377	1.43	75.68	7065	7065	MS2	32/64	Glioma_Nuclear_prep_Gradeall_repeat_4plex.raw
5031755	High		vTEGLVDVILYHQPDDk	Unambiguous	5	1	N-Term(iTRAQ4plex); K17(iTRAQ4plex)	HCD	1.0000	0.0000	1	1	11486	Unique	Used	0.243	1.000E5	2.670E4	151	6.85	0.00	0	7	4	3	743.73688	2229.19608	-2.02	52.49	6253	6253	MS2	34/64	Glioma_Nuclear_prep_Gradeall_4plex.raw
5031755	High		vTEGLVDVILYHQPDDk	Unambiguous	5	1	N-Term(iTRAQ4plex); K17(iTRAQ4plex)	HCD	1.0000	0.0000	1	1	11470	Unique	Used	0.320	3.424E4	1.202E4	181	7.93	0.00	0	0	39	2	1115.10193	2229.19658	-1.80	52.35	6235	6235	MS2	25/32	Glioma_Nuclear_prep_Gradeall_4plex.raw
5031755	High		vTEGLVDVILYHQPDDk	Unambiguous	5	1	N-Term(iTRAQ4plex); K17(iTRAQ4plex)	HCD	1.0000	0.0000	1	1	11404	Unique	Used	0.361	2.986E4	1.182E4	160	7.49	0.00	0	17	19	3	743.73749	2229.19791	-1.20	51.79	6163	6163	MS2	34/64	Glioma_Nuclear_prep_Gradeall_4plex.raw
5031755	High		vTEGLVDVILYHQPDDk	Unambiguous	5	1	N-Term(iTRAQ4plex); K17(iTRAQ4plex)	HCD	1.0000	0.0000	1	1	11437	Unique	Used	0.617	1.389E3	9.401E2	155	4.99	0.00	0	20	37	4	558.05426	2229.19521	-2.41	52.07	6199	6199	MS2	27/96	Glioma_Nuclear_prep_Gradeall_4plex.raw
5031755	High		vTEGLVDVILYHQPDDk	Unambiguous	5	1	N-Term(iTRAQ4plex); K17(iTRAQ4plex)	HCD	1.0000	0.0000	1	1	11539	Unique	Used	0.659	8.711E2	6.302E2	141	3.44	0.00	0	39	99	4	558.05420	2229.19497	-2.52	52.91	6310	6310	MS2	20/96	Glioma_Nuclear_prep_Gradeall_4plex.raw
5031755	High		lkDYAFVHFEDR	Unambiguous	7	1	N-Term(iTRAQ4plex); K2(iTRAQ4plex)	HCD	1.0000	0.0000	1	1	9635	Unique	Used	0.333	1.626E4	5.943E3	201	3.62	0.00	1	33	9	4	457.73895	1827.93398	-16.01	38.21	4233	4233	MS2	24/66	Glioma_Nuclear_prep_Gradeall_4plex.raw
5031755		High	tkENILEEFSk	Unambiguous	7	1	N-Term(iTRAQ4plex); K2(iTRAQ4plex); K11(iTRAQ4plex)	HCD	1.0000	0.0000	1	1	10508	Unique	Used	0.309	6.980E3	2.351E3	172	4.76	0.00	1	29	300	2	885.50598	1770.00469	2.10	51.14	4997	4997	MS2	19/20	Glioma_Nuclear_prep_Gradeall_repeat_4plex.raw
5031755		High	tkENILEEFSk	Unambiguous	7	1	N-Term(iTRAQ4plex); K2(iTRAQ4plex); K11(iTRAQ4plex)	HCD	1.0000	0.0000	1	1	10524	Unique	Used	0.693	3.909E3	2.953E3	134	4.36	0.00	1	37	94	3	590.67352	1770.00602	2.85	51.35	5015	5015	MS2	17/40	Glioma_Nuclear_prep_Gradeall_repeat_4plex.raw
5031755	High		tkENILEEFSk	Unambiguous	7	1	N-Term(iTRAQ4plex); K2(iTRAQ4plex); K11(iTRAQ4plex)	HCD	1.0000	0.0000	1	1	9624	Unique	Used	0.310	1.937E5	6.586E4	188	3.72	0.00	1	34	22	2	885.49170	1769.97612	-14.04	38.13	4221	4221	MS2	17/20	Glioma_Nuclear_prep_Gradeall_4plex.raw
5031755		High	yGGPPPDSVYSGVQPGIGTEVFVGk	Unambiguous	4	1	N-Term(iTRAQ4plex); K25(iTRAQ4plex)	HCD	1.0000	0.0000	1	1	11737	Unique	Used	0.312	4.245E2	1.444E2	90	6.68	0.00	0	11	300	3	932.49182	2795.46091	4.08	67.49	6413	6413	MS2	28/96	Glioma_Nuclear_prep_Gradeall_repeat_4plex.raw
5031755	High		yGGPPPDSVYSGVQPGIGTEVFVGk	Unambiguous	4	1	N-Term(iTRAQ4plex); K25(iTRAQ4plex)	HCD	1.0000	0.0000	1	1	10967	Unique	Used	0.318	5.614E4	1.962E4	104	5.70	0.00	0	0	17	2	1398.20752	2795.40776	-14.94	48.20	5685	5685	MS2	25/48	Glioma_Nuclear_prep_Gradeall_4plex.raw
5031755	High		yGGPPPDSVYSGVQPGIGTEVFVGk	Unambiguous	4	1	N-Term(iTRAQ4plex); K25(iTRAQ4plex)	HCD	1.0000	0.0000	1	1	11069	Unique	Used	0.382	5.830E3	2.443E3	93	5.70	0.00	0	31	89	2	1398.23169	2795.45610	2.36	48.99	5796	5796	MS2	27/48	Glioma_Nuclear_prep_Gradeall_4plex.raw
5031755	High		yGGPPPDSVYSGVQPGIGTEVFVGk	Unambiguous	4	1	N-Term(iTRAQ4plex); K25(iTRAQ4plex)	HCD	1.0000	0.0000	1	1	10964	Unique	Used	0.685	1.409E4	1.059E4	100	6.44	0.00	0	5	6	3	932.47693	2795.41623	-11.91	48.18	5682	5682	MS2	27/96	Glioma_Nuclear_prep_Gradeall_4plex.raw
5031755	High		yGGPPPDSVYSGVQPGIGTEVFVGk	Unambiguous	4	1	N-Term(iTRAQ4plex); K25(iTRAQ4plex)	HCD	1.0000	0.0000	1	1	11003	Unique	Used	0.706	1.683E4	1.304E4	101	6.91	0.00	0	6	5	3	932.48889	2795.45212	0.93	48.48	5724	5724	MS2	27/96	Glioma_Nuclear_prep_Gradeall_4plex.raw
5031755		High	eNILEEFSk	Unambiguous	7	1	N-Term(iTRAQ4plex); K9(iTRAQ4plex)	HCD	1.0000	0.0000	1	1	11241	Unique	Used	0.476	9.874E3	5.124E3	250	3.58	0.00	0	16	300	2	698.88177	1396.75627	0.01	60.32	5808	5808	MS2	15/16	Glioma_Nuclear_prep_Gradeall_repeat_4plex.raw
5031755		High	eNILEEFSk	Unambiguous	7	1	N-Term(iTRAQ4plex); K9(iTRAQ4plex)	HCD	1.0000	0.0000	1	1	11304	Unique	Used	0.614	9.181E3	6.144E3	241	3.34	0.00	0	59	300	2	698.88385	1396.76042	2.98	61.33	5890	5890	MS2	14/16	Glioma_Nuclear_prep_Gradeall_repeat_4plex.raw
5031755	High		eNILEEFSk	Unambiguous	7	1	N-Term(iTRAQ4plex); K9(iTRAQ4plex)	HCD	1.0000	0.0000	1	1	10358	Unique	Used	0.480	3.454E5	1.821E5	216	3.54	0.00	0	21	7	2	698.87219	1396.73711	-13.71	43.53	5020	5020	MS2	15/16	Glioma_Nuclear_prep_Gradeall_4plex.raw
5031877	NP_005564.1	LMNB1	4001	lamin-B1 isoform 1 [Homo sapiens]	47.10	2	21	45	1.846	22;23	27.2	150.40	586	66.4	5.16																			
5031877		High	sLETENSALQLQVTER	Unambiguous	1	1	N-Term(iTRAQ4plex)	HCD	1.0000	0.0000	1	1	10256	Unique	Used	1.394	7.656E2	1.163E3	190	5.60	0.00	0	33	300	3	654.68036	1962.02652	0.22	48.11	4727	4727	MS2	24/60	Glioma_Nuclear_prep_Gradeall_repeat_4plex.raw
5031877		High	aGGPTTPLSPTR	Unambiguous	1	1	N-Term(iTRAQ4plex)	HCD	1.0000	0.0000	1	1	7770	Unique	Used	1.467	5.931E4	9.482E4	156	4.30	0.00	0	9	19	2	649.86041	1298.71355	-3.84	21.62	2084	2084	MS2	16/22	Glioma_Nuclear_prep_Gradeall_repeat_4plex.raw
5031877		High	aLYETELADAR	Unambiguous	1	1	N-Term(iTRAQ4plex)	HCD	1.0000	0.0000	1	1	9841	Unique	Used	1.706	7.880E3	1.465E4	270	4.16	0.00	0	33	219	2	698.36694	1395.72661	2.10	43.10	4278	4278	MS2	19/20	Glioma_Nuclear_prep_Gradeall_repeat_4plex.raw
5031877		High	lSSEMNTSTVNSAR	Unambiguous	2	1	N-Term(iTRAQ4plex)	HCD	1.0000	0.0000	1	1	8362	Unique	Used	1.763	2.185E3	4.197E3	159	4.10	0.00	0	7	225	2	820.91040	1640.81352	6.37	27.28	2707	2707	MS2	20/26	Glioma_Nuclear_prep_Gradeall_repeat_4plex.raw
5031877		High	sMYEEEINETR	Unambiguous	1	1	N-Term(iTRAQ4plex)	HCD	1.0000	0.0000	1	1	8861	Unique	Used	1.947	6.725E3	1.427E4	72	4.49	0.00	0	23	207	2	772.85272	1544.69817	-2.45	32.26	3233	3233	MS2	18/20	Glioma_Nuclear_prep_Gradeall_repeat_4plex.raw
5031877		High	lAQALHEMR	Unambiguous	2	1	N-Term(iTRAQ4plex)	HCD	1.0000	0.0000	1	1	8958	Unique	Used	1.966	2.881E3	6.174E3	210	3.80	0.00	0	43	142	3	404.89056	1212.65714	-5.65	33.21	3335	3335	MS2	20/32	Glioma_Nuclear_prep_Gradeall_repeat_4plex.raw
5031877		High	aLYETELADAR	Unambiguous	1	1	N-Term(iTRAQ4plex)	HCD	1.0000	0.0000	1	1	9776	Unique	Used	2.175	2.368E4	5.614E4	267	3.99	0.00	0	7	53	2	698.36560	1395.72392	0.18	42.40	4210	4210	MS2	18/20	Glioma_Nuclear_prep_Gradeall_repeat_4plex.raw
5031877		High	sLETENSALQLQVTER	Unambiguous	1	1	N-Term(iTRAQ4plex)	HCD	1.0000	0.0000	1	1	10245	Unique	Used	2.214	1.092E3	2.635E3	222	6.48	0.00	0	0	300	2	981.51550	1962.02373	-1.20	47.98	4715	4715	MS2	27/30	Glioma_Nuclear_prep_Gradeall_repeat_4plex.raw
5031877	High		lALDMEISAYR	Unambiguous	2	1	N-Term(iTRAQ4plex)	HCD	1.0000	0.0000	1	1	10802	Unique	Used	1.373	3.083E4	4.647E4	232	3.61	0.00	0	53	52	2	713.37000	1425.73271	-14.14	46.93	5506	5506	MS2	18/20	Glioma_Nuclear_prep_Gradeall_4plex.raw
5031877	High		aLYETELADAR	Unambiguous	1	1	N-Term(iTRAQ4plex)	HCD	1.0000	0.0000	1	1	9261	Unique	Used	1.481	4.630E4	7.525E4	183	3.34	0.00	0	42	12	2	698.35425	1395.70122	-16.09	35.46	3825	3825	MS2	18/20	Glioma_Nuclear_prep_Gradeall_4plex.raw
5031877	High		lSSEMNTSTVNSAR	Unambiguous	2	1	N-Term(iTRAQ4plex)	HCD	1.0000	0.0000	1	1	8149	Unique	Used	1.695	3.550E4	6.602E4	105	4.16	0.00	0	9	18	2	820.89117	1640.77507	-17.07	27.41	2614	2614	MS2	20/26	Glioma_Nuclear_prep_Gradeall_4plex.raw
5031877	High		lAQALHEMR	Unambiguous	2	1	N-Term(iTRAQ4plex)	HCD	1.0000	0.0000	1	1	8433	Unique	Used	2.193	2.723E4	6.551E4	169	3.59	0.00	0	25	6	3	404.88565	1212.64240	-17.81	29.43	2922	2922	MS2	20/32	Glioma_Nuclear_prep_Gradeall_4plex.raw
5031877	High		sLETENSALQLQVTER	Unambiguous	1	1	N-Term(iTRAQ4plex)	HCD	1.0000	0.0000	1	1	9637	Unique	Used	2.212	3.481E4	8.447E4	202	6.26	0.00	0	3	14	2	981.50037	1961.99346	-16.63	38.22	4235	4235	MS2	26/30	Glioma_Nuclear_prep_Gradeall_4plex.raw
5031877	High		tTIPEEEEEEEEAAGVVVEEELFHQQGTPR	Unambiguous	2	1	N-Term(iTRAQ4plex)	HCD	1.0000	0.0000	1	1	12492	Unique	Used	2.414	1.143E3	3.028E3	46	10.53	0.00	0	0	69	3	1185.89844	3555.68076	2.11	62.23	7355	7355	MS2	36/116	Glioma_Nuclear_prep_Gradeall_4plex.raw
5031877	High		tTIPEEEEEEEEAAGVVVEEELFHQQGTPR	Unambiguous	2	1	N-Term(iTRAQ4plex)	HCD	1.0000	0.0000	1	1	12461	Unique	Used	3.862	6.996E1	2.965E2	82	8.33	0.00	0	39	131	4	889.67609	3555.68252	2.60	61.84	7320	7320	MS2	30/174	Glioma_Nuclear_prep_Gradeall_4plex.raw
5031877		High	cQSLTEDLEFR	Unambiguous	1	1	N-Term(iTRAQ4plex); C1(Methylthio)	HCD	1.0000	0.0000	1	1	11746	Unique	Used	1.908	1.245E3	2.590E3	77	4.05	0.00	0	22	300	2	765.85699	1530.70671	1.16	67.66	6427	6427	MS2	20/20	Glioma_Nuclear_prep_Gradeall_repeat_4plex.raw
5031877	High		cQSLTEDLEFR	Unambiguous	1	1	N-Term(iTRAQ4plex); C1(Methylthio)	HCD	1.0000	0.0000	1	1	10932	Unique	Used	1.549	4.986E4	8.475E4	50	4.51	0.00	0	18	27	2	765.84552	1530.68376	-13.83	47.93	5647	5647	MS2	19/20	Glioma_Nuclear_prep_Gradeall_4plex.raw
5031877		High	kQLADETLLk	Unambiguous	1	1	N-Term(iTRAQ4plex); K1(iTRAQ4plex); K10(iTRAQ4plex)	HCD	1.0000	0.0000	1	1	8767	Unique	Used	1.360	2.628E3	3.895E3	149	3.65	0.00	1	36	180	3	530.98889	1590.95212	-16.96	31.25	3134	3134	MS2	18/36	Glioma_Nuclear_prep_Gradeall_repeat_4plex.raw
5031877		High	kQLADETLLk	Unambiguous	1	1	N-Term(iTRAQ4plex); K1(iTRAQ4plex); K10(iTRAQ4plex)	HCD	1.0000	0.0000	1	1	8779	Unique	Used	1.588	1.615E3	2.794E3	120	2.99	0.00	1	41	300	2	795.98175	1590.95622	-14.39	31.38	3146	3146	MS2	18/18	Glioma_Nuclear_prep_Gradeall_repeat_4plex.raw
5031877	High		kIGDTSVSYk	Unambiguous	2	1	N-Term(iTRAQ4plex); K1(iTRAQ4plex); K10(iTRAQ4plex)	HCD	1.0000	0.0000	1	1	7649	Unique	Used	1.508	2.163E4	3.579E4	143	3.39	0.00	1	56	15	3	510.63409	1529.88773	-1.45	23.87	2072	2072	MS2	14/36	Glioma_Nuclear_prep_Gradeall_4plex.raw
5031877	High		kQLADETLLk	Unambiguous	1	1	N-Term(iTRAQ4plex); K1(iTRAQ4plex); K10(iTRAQ4plex)	HCD	1.0000	0.0000	1	1	8290	Unique	Used	2.217	3.310E4	8.051E4	127	3.89	0.00	1	34	21	2	795.98059	1590.95391	-15.84	28.41	2767	2767	MS2	18/18	Glioma_Nuclear_prep_Gradeall_4plex.raw
5031877		High	iQELEDLLAk	Unambiguous	2	1	N-Term(iTRAQ4plex); K10(iTRAQ4plex)	HCD	1.0000	0.0000	1	1	12337	Unique	Used	2.130	2.613E3	6.065E3	231	4.55	0.00	0	6	300	2	730.43488	1459.86247	0.96	80.31	7408	7408	MS2	18/18	Glioma_Nuclear_prep_Gradeall_repeat_4plex.raw
5031877	High		eELEQTYHAk	Unambiguous	2	1	N-Term(iTRAQ4plex); K10(iTRAQ4plex)	HCD	1.0000	0.0000	1	1	7549	Unique	Used	1.487	1.577E4	2.574E4	163	3.59	0.00	0	50	12	3	512.60211	1535.79178	-1.72	23.16	1964	1964	MS2	21/36	Glioma_Nuclear_prep_Gradeall_4plex.raw
5031877	High		iQELEDLLAk	Unambiguous	2	1	N-Term(iTRAQ4plex); K10(iTRAQ4plex)	HCD	1.0000	0.0000	1	1	11890	Unique	Used	1.590	1.367E5	2.385E5	214	4.19	0.00	0	18	14	2	730.43774	1459.86821	4.89	55.97	6692	6692	MS2	18/18	Glioma_Nuclear_prep_Gradeall_4plex.raw
5031877	High		iQELEDLLAk	Unambiguous	2	1	N-Term(iTRAQ4plex); K10(iTRAQ4plex)	HCD	1.0000	0.0000	1	1	11807	Unique	Used	1.922	1.154E5	2.434E5	236	4.39	0.00	0	17	15	2	730.43365	1459.86003	-0.71	55.28	6602	6602	MS2	18/18	Glioma_Nuclear_prep_Gradeall_4plex.raw
5031877		High	dAALATALGDk	Unambiguous	1	1	N-Term(iTRAQ4plex); K11(iTRAQ4plex)	HCD	1.0000	0.0000	1	1	9595	Unique	Used	1.455	9.541E3	1.513E4	310	4.45	0.00	0	28	231	2	667.37866	1333.75005	-4.92	40.34	4018	4018	MS2	19/20	Glioma_Nuclear_prep_Gradeall_repeat_4plex.raw
5031877	High		dAALATALGDk	Unambiguous	1	1	N-Term(iTRAQ4plex); K11(iTRAQ4plex)	HCD	1.0000	0.0000	1	1	9082	Unique	Used	2.074	1.726E5	3.929E5	299	4.23	0.00	0	15	5	2	667.37073	1333.73418	-16.82	34.16	3630	3630	MS2	19/20	Glioma_Nuclear_prep_Gradeall_4plex.raw
5031877		High	aEHDQLLLNYAk	Unambiguous	1	1	N-Term(iTRAQ4plex); K12(iTRAQ4plex)	HCD	1.0000	0.0000	1	1	9489	Unique	Used	2.124	1.862E3	4.310E3	193	5.39	0.00	0	15	300	2	851.97540	1702.94353	4.02	39.13	3900	3900	MS2	22/22	Glioma_Nuclear_prep_Gradeall_repeat_4plex.raw
5031877	High		aEHDQLLLNYAk	Unambiguous	1	1	N-Term(iTRAQ4plex); K12(iTRAQ4plex)	HCD	1.0000	0.0000	1	1	8846	Unique	Used	2.602	8.294E4	2.368E5	163	5.04	0.00	0	10	8	2	851.95844	1702.90959	-15.90	32.43	3372	3372	MS2	22/22	Glioma_Nuclear_prep_Gradeall_4plex.raw
5031877		High	iESLSSQLSNLQk	Unambiguous	2	1	N-Term(iTRAQ4plex); K13(iTRAQ4plex)	HCD	1.0000	0.0000	1	1	11003	Unique	Used	1.592	2.566E3	4.452E3	264	5.91	0.00	0	14	300	2	867.99768	1734.98808	2.34	57.18	5535	5535	MS2	24/24	Glioma_Nuclear_prep_Gradeall_repeat_4plex.raw
5031877	High		iESLSSQLSNLQk	Unambiguous	2	1	N-Term(iTRAQ4plex); K13(iTRAQ4plex)	HCD	1.0000	0.0000	1	1	10191	Unique	Used	1.671	6.032E4	1.106E5	254	5.44	0.00	0	19	20	2	867.98315	1734.95903	-14.41	42.28	4838	4838	MS2	24/24	Glioma_Nuclear_prep_Gradeall_4plex.raw
5031877	High		dQMQQQLNDYEQLLDVk	Unambiguous	2	1	N-Term(iTRAQ4plex); K17(iTRAQ4plex)	HCD	1.0000	0.0000	1	1	11974	Unique	Used	1.846	2.322E3	4.702E3	158	7.74	0.00	0	18	68	3	799.40289	2396.19413	-2.73	56.73	6784	6784	MS2	28/64	Glioma_Nuclear_prep_Gradeall_4plex.raw
5031877	High		dQMQQQLNDYEQLLDVk	Unambiguous	2	1	N-Term(iTRAQ4plex); K17(iTRAQ4plex)	HCD	1.0000	0.0000	1	1	11983	Unique	Used	2.756	5.272E3	1.595E4	151	7.48	0.00	0	0	62	2	1198.59827	2396.18926	-4.76	56.82	6794	6794	MS2	25/32	Glioma_Nuclear_prep_Gradeall_4plex.raw
5031877		High	lYkEELEQTYHAk	Unambiguous	2	1	N-Term(iTRAQ4plex); K3(iTRAQ4plex); K13(iTRAQ4plex)	HCD	1.0000	0.0000	1	1	9412	Unique	Used	1.917	6.182E2	1.291E3	139	5.05	0.00	1	20	170	3	695.38544	2084.14176	1.40	38.25	3817	3817	MS2	27/48	Glioma_Nuclear_prep_Gradeall_repeat_4plex.raw
5031877	High		lYkEELEQTYHAk	Unambiguous	2	1	N-Term(iTRAQ4plex); K3(iTRAQ4plex); K13(iTRAQ4plex)	HCD	1.0000	0.0000	1	1	8735	Unique	Used	2.175	2.175E4	5.191E4	161	5.40	0.00	1	0	13	2	1042.55481	2084.10234	-17.51	31.62	3251	3251	MS2	22/24	Glioma_Nuclear_prep_Gradeall_4plex.raw
5031877	High		lYkEELEQTYHAk	Unambiguous	2	1	N-Term(iTRAQ4plex); K3(iTRAQ4plex); K13(iTRAQ4plex)	HCD	1.0000	0.0000	1	1	8728	Unique	Used	3.673	1.296E4	5.223E4	134	4.60	0.00	1	13	3	3	695.37292	2084.10422	-16.61	31.58	3244	3244	MS2	30/48	Glioma_Nuclear_prep_Gradeall_4plex.raw
5031877		High	lAVYIDk	Unambiguous	1	1	N-Term(iTRAQ4plex); K7(iTRAQ4plex)	HCD	1.0000	0.0000	1	1	9757	Unique	Used	2.633	1.724E4	4.947E4	213	2.84	0.00	0	12	112	2	555.34491	1109.68254	1.49	42.18	4190	4190	MS2	12/12	Glioma_Nuclear_prep_Gradeall_repeat_4plex.raw
5031877		High	eYEAALNSk	Unambiguous	1	1	N-Term(iTRAQ4plex); K9(iTRAQ4plex)	HCD	1.0000	0.0000	1	1	8290	Unique	Used	1.774	3.564E4	6.890E4	165	3.44	0.00	0	8	39	2	656.85645	1312.70561	5.25	26.61	2632	2632	MS2	16/16	Glioma_Nuclear_prep_Gradeall_repeat_4plex.raw
5031877		High	qLADETLLk	Unambiguous	1	1	N-Term(iTRAQ4plex); K9(iTRAQ4plex)	HCD	1.0000	0.0000	1	1	9505	Unique	Used	1.816	1.486E4	2.941E4	253	3.69	0.00	0	40	80	2	659.89038	1318.77349	-6.52	39.32	3919	3919	MS2	16/16	Glioma_Nuclear_prep_Gradeall_repeat_4plex.raw
5031877	High		qLADETLLk	Unambiguous	1	1	N-Term(iTRAQ4plex); K9(iTRAQ4plex)	HCD	1.0000	0.0000	1	1	8941	Unique	Used	1.763	9.957E4	1.926E5	277	3.45	0.00	0	48	13	2	659.88373	1318.76018	-16.60	33.10	3475	3475	MS2	16/16	Glioma_Nuclear_prep_Gradeall_4plex.raw
5031877		High	smYEEEINETR	Unambiguous	1	1	N-Term(iTRAQ4plex); M2(Oxidation)	HCD	1.0000	0.0000	1	1	8279	Unique	Used	2.123	5.123E3	1.186E4	52	3.20	0.00	0	29	258	2	780.85693	1560.70659	6.23	26.48	2620	2620	MS2	16/20	Glioma_Nuclear_prep_Gradeall_repeat_4plex.raw
5031877		High	dQmQQQLNDYEQLLDVk	Unambiguous	2	1	N-Term(iTRAQ4plex); M3(Oxidation); K17(iTRAQ4plex)	HCD	1.0000	0.0000	1	1	11794	Unique	Used	1.387	1.335E2	2.018E2	132	6.36	0.00	0	19	300	3	804.73755	2412.19809	1.04	68.47	6493	6493	MS2	27/64	Glioma_Nuclear_prep_Gradeall_repeat_4plex.raw
5031877	High		dQmQQQLNDYEQLLDVk	Unambiguous	2	1	N-Term(iTRAQ4plex); M3(Oxidation); K17(iTRAQ4plex)	HCD	1.0000	0.0000	1	1	11082	Unique	Used	2.255	5.479E3	1.356E4	111	5.92	0.00	0	3	41	2	1206.59436	2412.18144	-5.86	49.09	5810	5810	MS2	25/32	Glioma_Nuclear_prep_Gradeall_4plex.raw
5031877		High	lSSEmNTSTVNSAR	Unambiguous	2	1	N-Term(iTRAQ4plex); M5(Oxidation)	HCD	1.0000	0.0000	1	1	7323	Unique	Used	1.814	5.104E3	1.009E4	115	4.88	0.00	0	25	146	2	828.90106	1656.79485	-1.90	17.60	1609	1609	MS2	20/26	Glioma_Nuclear_prep_Gradeall_repeat_4plex.raw
5031877		High	lAQALHEmR	Unambiguous	2	1	N-Term(iTRAQ4plex); M8(Oxidation)	HCD	1.0000	0.0000	1	1	7740	Unique	Used	1.531	4.500E3	7.509E3	213	3.80	0.00	0	24	112	3	410.22388	1228.65708	-1.49	21.32	2052	2052	MS2	18/32	Glioma_Nuclear_prep_Gradeall_repeat_4plex.raw
5032007	NP_005850.1	PURA	5813	transcriptional activator protein Pur-alpha [Homo sapiens]	9.94	1	1	2	0.331	1;1	26.9	11.23	322	34.9	6.44																			
5032007		High	fFFDVGSNk	Unambiguous	1	1	N-Term(iTRAQ4plex); K9(iTRAQ4plex)	HCD	1.0000	0.0000	1	1	11289	Unique	Used	0.268	1.124E4	3.281E3	310	3.38	0.00	0	60	300	2	674.86194	1348.71660	1.93	61.05	5868	5868	MS2	15/16	Glioma_Nuclear_prep_Gradeall_repeat_4plex.raw
5032007	High		fFFDVGSNk	Unambiguous	1	1	N-Term(iTRAQ4plex); K9(iTRAQ4plex)	HCD	1.0000	0.0000	1	1	10419	Unique	Used	0.394	9.786E4	4.226E4	293	3.73	0.00	0	88	46	2	674.85797	1348.70867	-3.95	43.98	5086	5086	MS2	14/16	Glioma_Nuclear_prep_Gradeall_4plex.raw
5032027	NP_005601.1	RBBP4	5928	histone-binding protein RBBP4 isoform a [Homo sapiens]	13.65	5	3	4	0.624	2;2	5.8	18.43	425	47.6	4.89																			
5032027		High	eAAFDDAVEER	Unambiguous	1	1	N-Term(iTRAQ4plex)	HCD	1.0000	0.0000	1	1	8859	Unique	Used	0.651	2.659E4	1.887E4	80	4.12	0.00	0	22	144	2	698.32764	1395.64800	-2.10	32.25	3231	3231	MS2	19/20	Glioma_Nuclear_prep_Gradeall_repeat_4plex.raw
5032027	High		eAAFDDAVEER	Unambiguous	1	1	N-Term(iTRAQ4plex)	HCD	1.0000	0.0000	1	1	8519	Unique	Used	0.571	1.966E5	1.231E5	56	4.02	0.00	0	13	16	2	698.31805	1395.62883	-15.83	30.03	3015	3015	MS2	17/20	Glioma_Nuclear_prep_Gradeall_4plex.raw
5032027	High		tPSSDVLVFDYTk	Unambiguous	5	1	N-Term(iTRAQ4plex); K13(iTRAQ4plex)	HCD	1.0000	0.0000	1	1	10329	Unique	Used	0.610	2.108E5	1.411E5	227	5.26	0.00	0	19	12	2	880.45966	1759.91203	-13.43	43.29	4988	4988	MS2	20/24	Glioma_Nuclear_prep_Gradeall_4plex.raw
5032027		High	vINEEYk	Unambiguous	4	1	N-Term(iTRAQ4plex); K7(iTRAQ4plex)	HCD	1.0000	0.0000	1	1	7986	Unique	Used	0.638	3.878E4	2.697E4	275	3.02	0.00	0	22	133	2	591.83569	1182.66411	2.73	23.67	2311	2311	MS2	12/12	Glioma_Nuclear_prep_Gradeall_repeat_4plex.raw
5032031	NP_005769.1	RBM5	10181	RNA-binding protein 5 [Homo sapiens]	2.94	1	1	2	0.629	1;1	1.2	8.76	815	92.1	6.28																			
5032031		High	lSEQELEALELR	Unambiguous	1	1	N-Term(iTRAQ4plex)	HCD	1.0000	0.0000	1	1	11586	Unique	Used	0.624	3.206E3	2.180E3	258	4.65	0.00	0	29	300	2	787.43384	1573.86040	3.15	65.40	6234	6234	MS2	21/22	Glioma_Nuclear_prep_Gradeall_repeat_4plex.raw
5032031	High		lSEQELEALELR	Unambiguous	1	1	N-Term(iTRAQ4plex)	HCD	1.0000	0.0000	1	1	10792	Unique	Used	0.635	8.754E4	6.100E4	192	4.43	0.00	0	22	20	2	787.42029	1573.83330	-14.06	46.86	5495	5495	MS2	21/22	Glioma_Nuclear_prep_Gradeall_4plex.raw
5032189	NP_005648.1	TP53BP1	7158	tumor suppressor p53-binding protein 1 isoform 3 [Homo sapiens]	6.19	3	6	7	0.648	3;4	16.6	30.35	1972	213.4	4.70																			
5032189		High	tSGTEPADFALPSSR	Unambiguous	3	1	N-Term(iTRAQ4plex)	HCD	1.0000	0.0000	1	1	9603	Unique	Used	0.630	4.773E3	3.275E3	182	5.06	0.00	0	9	300	2	840.41925	1679.83122	-2.70	40.43	4026	4026	MS2	22/28	Glioma_Nuclear_prep_Gradeall_repeat_4plex.raw
5032189		High	vITDVYYVDGTEVER	Unambiguous	3	1	N-Term(iTRAQ4plex)	HCD	1.0000	0.0000	1	1	10746	Unique	Used	0.682	1.325E3	9.857E2	168	4.46	0.00	0	23	300	2	951.48492	1901.96257	0.65	53.99	5253	5253	MS2	19/28	Glioma_Nuclear_prep_Gradeall_repeat_4plex.raw
5032189	High		nYLLPAGYSLEEQR	Unambiguous	3	1	N-Term(iTRAQ4plex)	HCD	1.0000	0.0000	1	1	10411	Unique	Used	0.649	4.026E4	2.867E4	147	3.66	0.00	0	19	51	2	898.95654	1796.90581	-13.45	43.92	5077	5077	MS2	16/26	Glioma_Nuclear_prep_Gradeall_4plex.raw
5032189		High	vDVScEPLEGVEk	Unambiguous	3	1	N-Term(iTRAQ4plex); C5(Methylthio); K13(iTRAQ4plex)	HCD	1.0000	0.0000	1	1	11228	Unique	Used	0.397	3.493E3	1.513E3	175	4.87	0.00	0	34	300	2	869.43945	1737.87163	4.28	60.16	5793	5793	MS2	21/24	Glioma_Nuclear_prep_Gradeall_repeat_4plex.raw
5032189	High		vDVScEPLEGVEk	Unambiguous	3	1	N-Term(iTRAQ4plex); C5(Methylthio); K13(iTRAQ4plex)	HCD	1.0000	0.0000	1	1	10426	Unique	Used	0.576	4.003E4	2.531E4	121	4.44	0.00	0	34	37	2	869.42426	1737.84123	-13.21	44.04	5094	5094	MS2	23/24	Glioma_Nuclear_prep_Gradeall_4plex.raw
5032189	High		aADISLDNLVEGk	Unambiguous	3	1	N-Term(iTRAQ4plex); K13(iTRAQ4plex)	HCD	1.0000	0.0000	1	1	10439	Unique	Used	0.648	5.212E4	3.709E4	226	5.23	0.00	0	48	40	2	816.94550	1632.88371	-12.87	44.15	5109	5109	MS2	23/24	Glioma_Nuclear_prep_Gradeall_4plex.raw
5032189		High	eTAVPGPLGIEDISPNLSPDDk	Unambiguous	3	1	N-Term(iTRAQ4plex); K22(iTRAQ4plex)	HCD	1.0000	0.0000	1	1	11798	Unique	Used	0.691	4.499E2	3.387E2	146	5.55	0.00	0	18	300	3	851.45221	2552.34208	3.36	68.59	6502	6502	MS2	27/84	Glioma_Nuclear_prep_Gradeall_repeat_4plex.raw
5174449	NP_006017.1	H1FX	8971	histone H1x [Homo sapiens]	23.00	1	4	13	1.653	6;7	12.2	54.20	213	22.5	10.76																			
5174449		High	ySQLVVETIR	Unambiguous	1	1	N-Term(iTRAQ4plex)	HCD	1.0000	0.0000	1	1	10740	Unique	Used	1.609	5.680E3	9.960E3	323	3.88	0.00	0	54	300	2	676.38989	1351.77251	1.70	53.92	5247	5247	MS2	16/18	Glioma_Nuclear_prep_Gradeall_repeat_4plex.raw
5174449		High	ySQLVVETIR	Unambiguous	1	1	N-Term(iTRAQ4plex)	HCD	1.0000	0.0000	1	1	10814	Unique	Used	1.688	2.466E4	4.538E4	204	3.68	0.00	0	5	47	2	676.38959	1351.77190	1.25	54.86	5329	5329	MS2	17/18	Glioma_Nuclear_prep_Gradeall_repeat_4plex.raw
5174449	High		ySQLVVETIR	Unambiguous	1	1	N-Term(iTRAQ4plex)	HCD	1.0000	0.0000	1	1	9962	Unique	Used	1.535	1.144E5	1.927E5	259	3.27	0.00	0	22	14	2	676.37793	1351.74858	-16.00	40.58	4588	4588	MS2	17/18	Glioma_Nuclear_prep_Gradeall_4plex.raw
5174449	High		ySQLVVETIR	Unambiguous	1	1	N-Term(iTRAQ4plex)	HCD	1.0000	0.0000	1	1	10050	Unique	Used	1.665	1.970E5	3.599E5	283	3.30	0.00	0	7	4	2	676.37854	1351.74980	-15.10	41.22	4684	4684	MS2	17/18	Glioma_Nuclear_prep_Gradeall_4plex.raw
5174449		High	aGGSAALSPSk	Unambiguous	1	1	N-Term(iTRAQ4plex); K11(iTRAQ4plex)	HCD	1.0000	0.0000	1	1	7153	Unique	Used	1.760	6.225E4	1.194E5	296	4.29	0.00	0	5	16	2	617.35303	1233.69878	-4.37	16.19	1431	1431	MS2	19/20	Glioma_Nuclear_prep_Gradeall_repeat_4plex.raw
5174449	High		aGGSAALSPSk	Unambiguous	1	1	N-Term(iTRAQ4plex); K11(iTRAQ4plex)	HCD	1.0000	0.0000	1	1	7309	Unique	Used	1.356	6.617E4	9.846E4	314	4.01	0.00	0	21	17	2	617.35223	1233.69719	-5.65	21.44	1704	1704	MS2	17/20	Glioma_Nuclear_prep_Gradeall_4plex.raw
5174449		High	aLVQNDTLLQVk	Unambiguous	1	1	N-Term(iTRAQ4plex); K12(iTRAQ4plex)	HCD	1.0000	0.0000	1	1	10472	Unique	Used	1.413	3.706E3	5.706E3	129	5.67	0.00	0	13	300	2	815.49384	1629.98039	1.57	50.69	4958	4958	MS2	22/22	Glioma_Nuclear_prep_Gradeall_repeat_4plex.raw
5174449		High	aLVQNDTLLQVk	Unambiguous	1	1	N-Term(iTRAQ4plex); K12(iTRAQ4plex)	HCD	1.0000	0.0000	1	1	10398	Unique	Used	1.427	1.227E3	1.909E3	128	5.34	0.00	0	16	300	3	543.99811	1629.97977	1.19	49.80	4879	4879	MS2	22/44	Glioma_Nuclear_prep_Gradeall_repeat_4plex.raw
5174449		High	aLVQNDTLLQVk	Unambiguous	1	1	N-Term(iTRAQ4plex); K12(iTRAQ4plex)	HCD	1.0000	0.0000	1	1	10400	Unique	Used	1.468	5.268E3	8.432E3	128	5.95	0.00	0	38	300	2	815.49310	1629.97893	0.68	49.83	4881	4881	MS2	22/22	Glioma_Nuclear_prep_Gradeall_repeat_4plex.raw
5174449	High		aLVQNDTLLQVk	Unambiguous	1	1	N-Term(iTRAQ4plex); K12(iTRAQ4plex)	HCD	1.0000	0.0000	1	1	9786	Unique	Used	1.653	6.782E4	1.230E5	161	4.65	0.00	0	27	11	2	815.48041	1629.95354	-14.90	39.30	4397	4397	MS2	22/22	Glioma_Nuclear_prep_Gradeall_4plex.raw
5174449	High		aLVQNDTLLQVk	Unambiguous	1	1	N-Term(iTRAQ4plex); K12(iTRAQ4plex)	HCD	1.0000	0.0000	1	1	9686	Unique	Used	2.085	1.088E5	2.489E5	154	4.85	0.00	0	8	5	2	815.47968	1629.95207	-15.80	38.59	4289	4289	MS2	22/22	Glioma_Nuclear_prep_Gradeall_4plex.raw
5174449		High	gAPAAATAPAPTAHk	Unambiguous	1	1	N-Term(iTRAQ4plex); K15(iTRAQ4plex)	HCD	1.0000	0.0000	1	1	7090	Unique	Used	1.840	5.384E3	1.080E4	239	5.27	0.00	0	10	86	2	810.44672	1619.88616	-15.23	15.56	1363	1363	MS2	24/28	Glioma_Nuclear_prep_Gradeall_repeat_4plex.raw
5174449	High		gAPAAATAPAPTAHk	Unambiguous	1	1	N-Term(iTRAQ4plex); K15(iTRAQ4plex)	HCD	1.0000	0.0000	1	1	7287	Unique	Used	1.706	2.202E4	4.122E4	243	6.22	0.00	0	0	44	2	810.45483	1619.90239	-5.21	21.25	1679	1679	MS2	24/28	Glioma_Nuclear_prep_Gradeall_4plex.raw
5729875	NP_006658.1	PGRMC1	10857	membrane-associated progesterone receptor component 1 isoform 1 [Homo sapiens]	21.03	2	3	6	0.644	1;5	9.7	32.38	195	21.7	4.70																			
5729875		High	fYGPEGPYGVFAGR	Unambiguous	1	1	N-Term(iTRAQ4plex)	HCD	1.0000	0.0000	1	1	11573	Unique	Used	0.581	4.593E3	2.908E3	193	4.04	0.00	0	13	300	2	830.91711	1660.82695	1.74	65.20	6218	6218	MS2	21/26	Glioma_Nuclear_prep_Gradeall_repeat_4plex.raw
5729875		High	fDGVQDPR	Unambiguous	2	1	N-Term(iTRAQ4plex)	HCD	1.0000	0.0000	1	1	8018	Unique	Used	0.684	4.101E4	3.056E4	152	2.89	0.00	0	36	95	2	539.27631	1077.54534	0.70	23.96	2345	2345	MS2	11/14	Glioma_Nuclear_prep_Gradeall_repeat_4plex.raw
5729875		High	fYGPEGPYGVFAGR	Unambiguous	1	1	N-Term(iTRAQ4plex)	HCD	1.0000	0.0000	1	1	11590	Unique	Used	0.697	4.895E2	3.717E2	145	4.87	0.00	0	46	300	3	554.28082	1660.82792	2.32	65.44	6238	6238	MS2	27/52	Glioma_Nuclear_prep_Gradeall_repeat_4plex.raw
5729875		High	kFYGPEGPYGVFAGR	Unambiguous	1	1	N-Term(iTRAQ4plex); K1(iTRAQ4plex)	HCD	1.0000	0.0000	1	1	10731	Unique	Used	0.571	2.882E3	1.795E3	111	4.37	0.00	1	7	300	2	967.01465	1933.02202	0.48	53.81	5237	5237	MS2	21/28	Glioma_Nuclear_prep_Gradeall_repeat_4plex.raw
5729875		High	kFYGPEGPYGVFAGR	Unambiguous	1	1	N-Term(iTRAQ4plex); K1(iTRAQ4plex)	HCD	1.0000	0.0000	1	1	10711	Unique	Used	0.709	1.388E3	1.072E3	140	5.71	0.00	1	17	274	3	645.01337	1933.02555	2.30	53.57	5216	5216	MS2	26/56	Glioma_Nuclear_prep_Gradeall_repeat_4plex.raw
5729875	High		kFYGPEGPYGVFAGR	Unambiguous	1	1	N-Term(iTRAQ4plex); K1(iTRAQ4plex)	HCD	1.0000	0.0000	1	1	9838	Unique	Used	0.604	9.732E4	6.455E4	135	4.15	0.00	1	0	16	2	966.99896	1932.99065	-15.75	39.66	4453	4453	MS2	21/28	Glioma_Nuclear_prep_Gradeall_4plex.raw

5730027	NP_006550.1	KHDRBS1	10657	KH domain-containing, RNA-binding, signal transduction-associated protein 1 isoform 1 [Homo sapiens]	16.70	6	4	10	0.282	4;6	39.1	41.67	443	48.2	8.66																			
5730027		High	sGSMDPSGAHPSVR	Unambiguous	2	1	N-Term(iTRAQ4plex)	HCD	1.0000	0.0000	1	1	7122	Unique	Used	0.246	1.219E4	3.264E3	70	4.04	0.00	0	13	213	2	764.85870	1528.71013	-12.69	15.88	1398	1398	MS2	18/26	Glioma_Nuclear_prep_Gradeall_repeat_4plex.raw
5730027		High	kDDEENYLDLFSHk	Unambiguous	2	1	N-Term(iTRAQ4plex); K1(iTRAQ4plex); K14(iTRAQ4plex)	HCD	1.0000	0.0000	1	1	10766	Unique	Used	0.343	1.142E3	4.276E2	146	6.55	0.00	1	30	300	3	729.04706	2185.12662	5.88	54.24	5275	5275	MS2	33/52	Glioma_Nuclear_prep_Gradeall_repeat_4plex.raw
5730027		High	kDDEENYLDLFSHk	Unambiguous	2	1	N-Term(iTRAQ4plex); K1(iTRAQ4plex); K14(iTRAQ4plex)	HCD	1.0000	0.0000	1	1	10779	Unique	Used	0.454	4.847E2	2.399E2	137	3.37	0.00	1	16	123	4	547.03625	2185.12319	4.31	54.41	5290	5290	MS2	30/78	Glioma_Nuclear_prep_Gradeall_repeat_4plex.raw
5730027	High		kDDEENYLDLFSHk	Unambiguous	2	1	N-Term(iTRAQ4plex); K1(iTRAQ4plex); K14(iTRAQ4plex)	HCD	1.0000	0.0000	1	1	9796	Unique	Used	0.301	7.273E4	2.401E4	125	6.32	0.00	1	26	5	3	729.03448	2185.08890	-11.38	39.37	4408	4408	MS2	28/52	Glioma_Nuclear_prep_Gradeall_4plex.raw
5730027		High	iLGPQGNTIk	Unambiguous	1	1	N-Term(iTRAQ4plex); K10(iTRAQ4plex)	HCD	1.0000	0.0000	1	1	8708	Unique	Used	0.163	1.250E5	2.217E4	86	3.14	0.00	0	28	62	2	664.91547	1328.82366	7.23	30.73	3072	3072	MS2	17/18	Glioma_Nuclear_prep_Gradeall_repeat_4plex.raw
5730027		High	iLGPQGNTIk	Unambiguous	1	1	N-Term(iTRAQ4plex); K10(iTRAQ4plex)	HCD	1.0000	0.0000	1	1	8739	Unique	Used	0.170	9.978E4	1.848E4	153	3.94	0.00	0	16	80	2	664.90356	1328.79985	-10.68	30.97	3104	3104	MS2	17/18	Glioma_Nuclear_prep_Gradeall_repeat_4plex.raw
5730027	High		dSLDPSFTHAMQLLTAEIEk	Unambiguous	2	1	N-Term(iTRAQ4plex); K20(iTRAQ4plex)	HCD	1.0000	0.0000	1	1	12908	Unique	Used	0.171	9.833E2	1.841E2	131	6.33	0.00	0	16	300	3	845.44263	2534.31333	3.22	68.12	7856	7856	MS2	27/76	Glioma_Nuclear_prep_Gradeall_4plex.raw
5730027	High		dSLDPSFTHAMQLLTAEIEk	Unambiguous	2	1	N-Term(iTRAQ4plex); K20(iTRAQ4plex)	HCD	1.0000	0.0000	1	1	12863	Unique	Used	0.292	1.083E3	3.472E2	150	6.33	0.00	0	13	300	3	845.44232	2534.31241	2.86	67.38	7797	7797	MS2	28/76	Glioma_Nuclear_prep_Gradeall_4plex.raw
5730027	High		dSLDPSFTHAMQLLTAEIEk	Unambiguous	2	1	N-Term(iTRAQ4plex); K20(iTRAQ4plex)	HCD	1.0000	0.0000	1	1	12853	Unique	Used	0.486	1.196E2	6.376E1	152	3.68	0.00	0	37	300	4	634.33398	2534.31411	3.53	67.22	7784	7784	MS2	20/114	Glioma_Nuclear_prep_Gradeall_4plex.raw
5730027		High	sGSmDPSGAHPSVR	Unambiguous	2	1	N-Term(iTRAQ4plex); M4(Oxidation)	HCD	1.0000	0.0000	1	1	6668	Unique	Used	0.272	4.580E3	1.360E3	78	5.02	0.00	0	47	188	3	515.57904	1544.72257	-1.21	11.81	918	918	MS2	21/52	Glioma_Nuclear_prep_Gradeall_repeat_4plex.raw
5730073	NP_006549.1	KHDRBS3	10656	KH domain-containing, RNA-binding, signal transduction-associated protein 3 [Homo sapiens]	20.81	5	6	11	0.316	6;5	38.5	32.90	346	38.8	7.61																			
5730073		High	fLIPDYNDEIR	Unambiguous	1	1	N-Term(iTRAQ4plex)	HCD	1.0000	0.0000	1	1	11565	Unique	Used	0.230	5.741E3	1.441E3	276	3.23	0.00	0	15	300	2	769.90417	1538.80107	2.53	65.08	6208	6208	MS2	14/20	Glioma_Nuclear_prep_Gradeall_repeat_4plex.raw
5730073		High	fLIPDYNDEIR	Unambiguous	1	1	N-Term(iTRAQ4plex)	HCD	1.0000	0.0000	1	1	11640	Unique	Used	0.251	1.473E4	4.033E3	274	3.53	0.00	0	29	300	2	769.90393	1538.80058	2.22	66.11	6296	6296	MS2	13/20	Glioma_Nuclear_prep_Gradeall_repeat_4plex.raw
5730073	High		fLIPDYNDEIR	Unambiguous	1	1	N-Term(iTRAQ4plex)	HCD	1.0000	0.0000	1	1	10790	Unique	Used	0.192	6.674E5	1.409E5	239	3.19	0.00	0	17	10	2	769.89221	1538.77715	-13.01	46.85	5493	5493	MS2	15/20	Glioma_Nuclear_prep_Gradeall_4plex.raw
5730073	High		dSLDPSFTHALR	Unambiguous	1	1	N-Term(iTRAQ4plex)	HCD	1.0000	0.0000	1	1	9043	Unique	Used	0.530	7.496E4	4.359E4	84	4.28	0.00	0	13	5	3	501.58771	1502.74857	-15.61	33.84	3586	3586	MS2	27/44	Glioma_Nuclear_prep_Gradeall_4plex.raw
5730073		High	yIDVVINk	Unambiguous	1	1	N-Term(iTRAQ4plex); K8(iTRAQ4plex)	HCD	1.0000	0.0000	1	1	10407	Unique	Used	0.313	3.301E4	1.127E4	179	3.54	0.00	0	28	300	2	626.38336	1251.75945	3.46	49.92	4889	4889	MS2	13/14	Glioma_Nuclear_prep_Gradeall_repeat_4plex.raw
5730073		High	IVNQEIEk	Unambiguous	1	1	N-Term(iTRAQ4plex); K8(iTRAQ4plex)	HCD	1.0000	0.0000	1	1	8373	Unique	Used	0.354	9.220E4	3.560E4	274	3.32	0.00	0	40	79	2	630.87683	1260.74639	4.90	27.40	2719	2719	MS2	14/14	Glioma_Nuclear_prep_Gradeall_repeat_4plex.raw
5730073		High	IVNQEIEk	Unambiguous	1	1	N-Term(iTRAQ4plex); K8(iTRAQ4plex)	HCD	1.0000	0.0000	1	1	8411	Unique	Used	0.644	9.523E4	6.682E4	268	2.86	0.00	0	65	51	2	630.87531	1260.74333	2.48	27.76	2759	2759	MS2	14/14	Glioma_Nuclear_prep_Gradeall_repeat_4plex.raw
5730073	High		IQEETLTk	Unambiguous	1	1	N-Term(iTRAQ4plex); K8(iTRAQ4plex)	HCD	1.0000	0.0000	1	1	8026	Unique	Used	0.263	5.245E5	1.516E5	291	3.18	0.00	0	28	10	2	625.35840	1249.70952	-11.76	26.54	2481	2481	MS2	14/14	Glioma_Nuclear_prep_Gradeall_4plex.raw
5730073	High		yIDVVINk	Unambiguous	1	1	N-Term(iTRAQ4plex); K8(iTRAQ4plex)	HCD	1.0000	0.0000	1	1	9725	Unique	Used	0.316	5.592E5	1.936E5	259	3.07	0.00	0	52	8	2	626.37323	1251.73918	-12.73	38.87	4331	4331	MS2	13/14	Glioma_Nuclear_prep_Gradeall_4plex.raw
5730073	High		IVNQEIEk	Unambiguous	1	1	N-Term(iTRAQ4plex); K8(iTRAQ4plex)	HCD	1.0000	0.0000	1	1	8105	Unique	Used	0.443	1.172E6	5.694E5	213	2.99	0.00	0	53	4	2	630.86597	1260.72466	-12.33	27.11	2567	2567	MS2	14/14	Glioma_Nuclear_prep_Gradeall_4plex.raw
5730073	High		mGHALEEIk	Unambiguous	1	1	N-Term(iTRAQ4plex); K9(iTRAQ4plex)	HCD	1.0000	0.0000	1	1	8232	Unique	Used	0.360	5.378E5	2.127E5	182	3.57	0.00	0	52	10	2	658.35742	1315.70757	-15.74	28.00	2704	2704	MS2	14/16	Glioma_Nuclear_prep_Gradeall_4plex.raw
5803036	NP_006796.1	HNRNPA0	10949	heterogeneous nuclear ribonucleoprotein A0 [Homo sapiens]	18.36	1	4	7	0.549	3;4	26.0	18.26	305	30.8	9.29																			
5803036		High	eDIYSGGGGGGSR	Unambiguous	1	1	N-Term(iTRAQ4plex)	HCD	1.0000	0.0000	1	1	7258	Unique	Used	0.424	4.434E4	2.047E4	41	3.37	0.00	0	5	72	2	678.30884	1355.61040	-15.08	17.01	1541	1541	MS2	17/24	Glioma_Nuclear_prep_Gradeall_repeat_4plex.raw
5803036		High	fHPIQGHR	Unambiguous	1	1	N-Term(iTRAQ4plex)	HCD	1.0000	0.0000	1	1	6846	Unique	Used	0.445	4.656E4	2.256E4	275	3.70	0.00	0	37	65	3	379.21201	1135.62146	-2.37	13.42	1105	1105	MS2	18/28	Glioma_Nuclear_prep_Gradeall_repeat_4plex.raw
5803036		High	gFGFVYFQNHDAADk	Unambiguous	1	1	N-Term(iTRAQ4plex); K15(iTRAQ4plex)	HCD	1.0000	0.0000	1	1	10964	Unique	Used	0.558	2.134E3	1.298E3	137	5.50	0.00	0	18	240	3	668.66827	2003.99027	2.41	56.71	5493	5493	MS2	30/56	Glioma_Nuclear_prep_Gradeall_repeat_4plex.raw
5803036	High		gFGFVYFQNHDAADk	Unambiguous	1	1	N-Term(iTRAQ4plex); K15(iTRAQ4plex)	HCD	1.0000	0.0000	1	1	10008	Unique	Used	0.549	7.088E4	4.267E4	102	5.95	0.00	0	11	25	2	1002.48206	2003.95683	-14.27	40.91	4638	4638	MS2	25/28	Glioma_Nuclear_prep_Gradeall_4plex.raw
5803036		High	gDVAEGDLIEHFSQFGTVEk	Unambiguous	1	1	N-Term(iTRAQ4plex); K20(iTRAQ4plex)	HCD	1.0000	0.0000	1	1	12029	Unique	Used	0.747	6.084E2	4.951E2	132	5.70	0.00	0	24	300	3	822.75385	2466.24698	3.16	72.53	6821	6821	MS2	30/76	Glioma_Nuclear_prep_Gradeall_repeat_4plex.raw
5803036	High		gDVAEGDLIEHFSQFGTVEk	Unambiguous	1	1	N-Term(iTRAQ4plex); K20(iTRAQ4plex)	HCD	1.0000	0.0000	1	1	11259	Unique	Used	0.392	3.102E4	1.335E4	138	6.47	0.00	0	24	14	3	822.74890	2466.23215	-2.85	50.52	6004	6004	MS2	30/76	Glioma_Nuclear_prep_Gradeall_4plex.raw
5803036	High		gDVAEGDLIEHFSQFGTVEk	Unambiguous	1	1	N-Term(iTRAQ4plex); K20(iTRAQ4plex)	HCD	1.0000	0.0000	1	1	11382	Unique	Used	0.728	6.256E3	4.999E3	139	5.76	0.00	0	25	62	3	822.75409	2466.24771	3.46	51.58	6138	6138	MS2	29/76	Glioma_Nuclear_prep_Gradeall_4plex.raw
5902076	NP_008855.1	SRSF1	6426	serine/arginine-rich splicing factor 1 isoform 1 [Homo sapiens]	44.76	2	11	37	0.667	16;21	10.8	150.16	248	27.7	10.36																			
5902076		High	sHEGETAYIR	Unambiguous	1	1	N-Term(iTRAQ4plex)	HCD	1.0000	0.0000	1	1	7023	Unique	Used	0.547	3.399E4	2.028E4	110	5.41	0.00	0	29	41	3	436.21979	1306.64481	-4.60	15.01	1292	1292	MS2	21/36	Glioma_Nuclear_prep_Gradeall_repeat_4plex.raw
5902076		High	iYVGNLPPDIR	Unambiguous	2	1	N-Term(iTRAQ4plex)	HCD	1.0000	0.0000	1	1	11036	Unique	Used	0.563	1.391E4	8.542E3	207	4.27	0.00	0	9	300	2	700.90540	1400.80351	1.18	57.63	5573	5573	MS2	17/20	Glioma_Nuclear_prep_Gradeall_repeat_4plex.raw
5902076		High	iYVGNLPPDIR	Unambiguous	2	1	N-Term(iTRAQ4plex)	HCD	1.0000	0.0000	1	1	10859	Unique	Used	0.585	3.731E4	2.381E4	195	4.20	0.00	0	8	78	2	700.90631	1400.80535	2.49	55.42	5378	5378	MS2	17/20	Glioma_Nuclear_prep_Gradeall_repeat_4plex.raw
5902076		High	sHEGETAYIR	Unambiguous	1	1	N-Term(iTRAQ4plex)	HCD	1.0000	0.0000	1	1	7022	Unique	Used	0.594	8.150E4	5.276E4	136	3.66	0.00	0	15	60	2	653.82593	1306.64458	-4.78	15.01	1291	1291	MS2	17/18	Glioma_Nuclear_prep_Gradeall_repeat_4plex.raw
5902076		High	iYVGNLPPDIR	Unambiguous	2	1	N-Term(iTRAQ4plex)	HCD	1.0000	0.0000	1	1	10952	Unique	Used	0.608	2.663E4	1.765E4	256	4.21	0.00	0	16	131	2	700.90564	1400.80400	1.53	56.57	5480	5480	MS2	17/20	Glioma_Nuclear_prep_Gradeall_repeat_4plex.raw
5902076		High	iYVGNLPPDIR	Unambiguous	2	1	N-Term(iTRAQ4plex)	HCD	1.0000	0.0000	1	1	11114	Unique	Used	0.609	9.629E3	6.396E3	209	4.25	0.00	0	13	300	2	700.90588	1400.80449	1.88	58.63	5661	5661	MS2	17/20	Glioma_Nuclear_prep_Gradeall_repeat_4plex.raw
5902076		High	dGTGVVEFVR	Unambiguous	2	1	N-Term(iTRAQ4plex)	HCD	1.0000	0.0000	1	1	9525	Unique	Used	0.645	1.515E5	1.065E5	254	3.64	0.00	0	4	11	2	611.82751	1222.64775	-5.82	39.57	3942	3942	MS2	18/18	Glioma_Nuclear_prep_Gradeall_repeat_4plex.raw
5902076		High	gGPPFAFVEFEDPR	Unambiguous	2	1	N-Term(iTRAQ4plex)	HCD	1.0000	0.0000	1	1	12201	Unique	Used	0.667	5.715E2	4.156E2	120	5.14	0.00	0	6	212	3	570.28748	1708.84787	1.55	76.10	7099	7099	MS2	24/52	Glioma_Nuclear_prep_Gradeall_repeat_4plex.raw
5902076		High	gGPPFAFVEFEDPR	Unambiguous	2	1	N-Term(iTRAQ4plex)	HCD	1.0000	0.0000	1	1	12215	Unique	Used	0.706	2.177E4	1.675E4	104	5.15	0.00	0	3	91	2	854.92896	1708.85063	3.17	76.53	7131	7131	MS2	24/26	Glioma_Nuclear_prep_Gradeall_repeat_4plex.raw
5902076		High	iYVGNLPPDIR	Unambiguous	2	1	N-Term(iTRAQ4plex)	HCD	1.0000	0.0000	1	1	10780	Unique	Used	0.725	1.547E4	1.222E4	216	4.10	0.00	0	32	256	2	700.90601	1400.80474	2.05	54.42	5291	5291	MS2	17/20	Glioma_Nuclear_prep_Gradeall_repeat_4plex.raw
5902076		High	dAEDAVYGR	Unambiguous	2	1	N-Term(iTRAQ4plex)	HCD	1.0000	0.0000	1	1	7566	Unique	Used	0.751	1.320E5	1.080E5	77	3.26	0.00	0	3	19	2	570.27417	1139.54106	-3.43	19.67	1865	1865	MS2	15/16	Glioma_Nuclear_prep_Gradeall_repeat_4plex.raw
5902076		High	gGPPFAFVEFEDPR	Unambiguous	2	1	N-Term(iTRAQ4plex)	HCD	1.0000	0.0000	1	1	12249	Unique	Used	0.764	1.783E3	1.484E3	143	4.66	0.00	0	14	300	2	854.92834	1708.84941	2.45	77.54	7206	7206	MS2	23/26	Glioma_Nuclear_prep_Gradeall_repeat_4plex.raw
5902076		High	gGPPFAFVEFEDPR	Unambiguous	2	1	N-Term(iTRAQ4plex)	HCD	1.0000	0.0000	1	1	12236	Unique	Used	0.765	2.422E2	2.020E2	109	4.38	0.00	0	5	224	3	570.28735	1708.84751	1.34	77.12	7175	7175	MS2	23/52	Glioma_Nuclear_prep_Gradeall_repeat_4plex.raw
5902076	High		gGPPFAFVEFEDPR	Unambiguous	2	1	N-Term(iTRAQ4plex)	HCD	1.0000	0.0000	1	1	11535	Unique	Used	0.557	8.705E3	5.322E3	89	4.02	0.00	0	18	24	3	570.28485	1708.84000	-3.06	52.88	6306	6306	MS2	22/52	Glioma_Nuclear_prep_Gradeall_4plex.raw
5902076	High		iYVGNLPPDIR	Unambiguous	2	1	N-Term(iTRAQ4plex)	HCD	1.0000	0.0000	1	1	10350	Unique	Used	0.588	1.598E5	1.031E5	279	3.19	0.00	0	29	10	2	700.89465	1400.78203	-14.16	43.46	5011	5011	MS2	16/20	Glioma_Nuclear_prep_Gradeall_4plex.raw
5902076	High		dGTGVVEFVR	Unambiguous	2	1	N-Term(iTRAQ4plex)	HCD	1.0000	0.0000	1	1	9102	Unique	Used	0.621	6.803E5	4.637E5	244	3.32	0.00	0	7	2	2	611.82104	1222.63481	-16.41	34.31	3652	3652	MS2	18/18	Glioma_Nuclear_prep_Gradeall_4plex.raw
5902076	High		sHEGETAYIR	Unambiguous	1	1	N-Term(iTRAQ4plex)	HCD	1.0000	0.0000	1	1	7267	Unique	Used	0.661	1.447E5	1.049E5	113	3.20	0.00	0	27	14	2	653.82654	1306.64580	-3.85	21.12	1658	1658	MS2	17/18	Glioma_Nuclear_prep_Gradeall_4plex.raw
5902076	High		iYVGNLPPDIR	Unambiguous	2	1	N-Term(iTRAQ4plex)	HCD	1.0000	0.0000	1	1	10163	Unique	Used	0.675	3.972E5	2.942E5	257	3.83	0.00	0	16	6	2	700.89459	1400.78191	-14.25	42.08	4808	4808	MS2	17/20	Glioma_Nuclear_prep_Gradeall_4plex.raw
5902076	High		dGTGVVEFVR	Unambiguous	2	1	N-Term(iTRAQ4plex)	HCD	1.0000	0.0000	1	1	9002	Unique	Used	0.687	2.234E5	1.685E5	265	3.22	0.00	0	25	10	2	611.82129	1222.63530	-16.01	33.54	3541	3541	MS2	18/18	Glioma_Nuclear_prep_Gradeall_4plex.raw
5902076	High		iYVGNLPPDIR	Unambiguous	2	1	N-Term(iTRAQ4plex)	HCD	1.0000	0.0000	1	1	10074	Unique	Used	0.687	7.311E5	5.514E5	257	3.89	0.00	0	13	3	2	700.89423	1400.78118	-14.77	41.39	4710	4710	MS2	17/20	Glioma_Nuclear_prep_Gradeall_4plex.raw
5902076	High		iYVGNLPPDIR	Unambiguous	2	1	N-Term(iTRAQ4plex)	HCD	1.0000	0.0000	1	1	10444	Unique	Used	0.696	2.043E5	1.561E5	245	3.55	0.00	0	25	11	2	700.89478	1400.78227	-13.99	44.20	5115	5115	MS2	17/20	Glioma_Nuclear_prep_Gradeall_4plex.raw
5902076	High		iYVGNLPPDIR	Unambiguous	2	1	N-Term(iTRAQ4plex)	HCD	1.0000	0.0000	1	1	10259	Unique	Used	0.713	2.497E5	1.953E5	271	3.91	0.00	0	24	7	2	700.89478	1400.78227	-13.99	42.79	4912	4912	MS2	17/20	Glioma_Nuclear_prep_Gradeall_4plex.raw
5902076	High		dGYDYDGYR	Unambiguous	2	1	N-Term(iTRAQ4plex)	HCD	1.0000	0.0000	1	1	8158	Unique	Used	0.742	2.416E5	1.967E5	7	3.27	0.00	0	17	6	2	634.26056	1267.51384	-16.51	27.48	2624	2624	MS2	15/16	Glioma_Nuclear_prep_Gradeall_4plex.raw
5902076	High		gGPPFAFVEFEDPR	Unambiguous	2	1	N-Term(iTRAQ4plex)	HCD	1.0000	0.0000	1	1	11590	Unique	Used	0.764	6.701E5	5.620E5	111	4.85	0.00	0	5	2	2	854.92719	1708.84709	1.09	53.34	6366	6366	MS2	22/26	Glioma_Nuclear_prep_Gradeall_4plex.raw
5902076	High		eAGDVcYADVYR	Unambiguous	2	1	N-Term(iTRAQ4plex); C6(Methylthio)	HCD	1.0000	0.0000	1	1	9800	Unique	Used	0.727	1.158E5	9.236E4	15	3.53	0.00	0	35	15	2	775.82941	1550.65154	-14.24	39.40	4412	4412	MS2	21/22	Glioma_Nuclear_prep_Gradeall_4plex.raw
5902076		High	kEDMTYAVR	Unambiguous	1	1	N-Term(iTRAQ4plex); K1(iTRAQ4plex)	HCD	1.0000	0.0000	1	1	7622	Unique	Used	0.634	2.859E4	1.976E4	189	3.32	0.00	1	8	65	2	700.87500	1400.74272	-1.37	20.13	1924	1924	MS2	16/16	Glioma_Nuclear_prep_Gradeall_repeat_4plex.raw
5902076	High		kEDMTYAVR	Unambiguous	1	1	N-Term(iTRAQ4plex); K1(iTRAQ4plex)	HCD	1.0000	0.0000	1	1	7531	Unique	Used	0.685	1.147E5	8.617E4	179	3.55	0.00	1	14	2	3	467.58603	1400.74353	-0.79	23.04	1945	1945	MS2	21/32	Glioma_Nuclear_prep_Gradeall_4plex.raw
5902076		High	vVVSGLPPSGSWQDLk	Unambiguous	2	1	N-Term(iTRAQ4plex); K16(iTRAQ4plex)	HCD	1.0000	0.0000	1	1	11551	Unique	Used	0.550	2.784E3	1.668E3	112	4.18	0.00	0	32	300	2	979.05426	1957.10124	0.76	64.85	6189	6189	MS2	20/30	Glioma_Nuclear_prep_Gradeall_repeat_4plex.raw
5902076		High	vVVSGLPPSGSWQDLk	Unambiguous	2	1	N-Term(iTRAQ4plex); K16(iTRAQ4plex)	HCD	1.0000	0.0000	1	1	11624	Unique	Used	0.574	1.587E3	9.928E2	150	5.02	0.00	0	9	300	2	979.05414	1957.10100	0.63	65.89	6277	6277	MS2	20/30	Glioma_Nuclear_prep_Gradeall_repeat_4plex.raw
5902076		High	vVVSGLPPSGSWQDLk	Unambiguous	2	1	N-Term(iTRAQ4plex); K16(iTRAQ4plex)	HCD	1.0000	0.0000	1	1	11465	Unique	Used	0.668	7.341E2	5.343E2	161	4.11	0.00	0	37	300	3	653.03857	1957.10117	0.72	63.70	6090	6090	MS2	19/60	Glioma_Nuclear_prep_Gradeall_repeat_4plex.raw
5902076	High		vVVSGLPPSGSWQDLk	Unambiguous	2	1	N-Term(iTRAQ4plex); K16(iTRAQ4plex)	HCD	1.0000	0.0000	1	1	10745	Unique	Used	0.584	1.014E5	6.506E4	168	3.62	0.00	0	36	21	2	979.03931	1957.07134	-14.52	46.50	5444	5444	MS2	21/30	Glioma_Nuclear_prep_Gradeall_4plex.raw
5902076	High		vVVSGLPPSGSWQDLk	Unambiguous	2	1	N-Term(iTRAQ4plex); K16(iTRAQ4plex)	HCD	1.0000	0.0000	1	1	10654	Unique	Used	0.743	1.057E5	8.619E4	178	3.84	0.00	0	15	10	2	979.03931	1957.07134	-14.52	45.81	5345	5345	MS2	21/30	Glioma_Nuclear_prep_Gradeall_4plex.raw
5902076		High	tkDIEDVFYk	Unambiguous	2	1	N-Term(iTRAQ4plex); K2(iTRAQ4plex); K10(iTRAQ4plex)	HCD	1.0000	0.0000	1	1	10010	Unique	Used	0.576	9.652E3	6.055E3	230	4.04	0.00	1	51	300	2	845.47626	1689.94524	1.69	45.16	4462	4462	MS2	16/18	Glioma_Nuclear_prep_Gradeall_repeat_4plex.raw
5902076		High	tkDIEDVFYk	Unambiguous	2	1	N-Term(iTRAQ4plex); K2(iTRAQ4plex); K10(iTRAQ4plex)	HCD	1.0000	0.0000	1	1	10005	Unique	Used	0.750	2.195E3	1.794E3	210	4.43	0.00	1	17	169	3	563.98633	1689.94443	1.21	45.11	4457	4457	MS2	21/36	Glioma_Nuclear_prep_Gradeall_repeat_4plex.raw
5902076	High		tkDIEDVFYk	Unambiguous	2	1	N-Term(iTRAQ4plex); K2(iTRAQ4plex); K10(iTRAQ4plex)	HCD	1.0000	0.0000	1	1	9320	Unique	Used	0.579	1.828E5	1.162E5	197	3.94	0.00	1	13	8	2	845.46161	1689.91594	-15.65	35.93	3891	3891	MS2	17/18	Glioma_Nuclear_prep_Gradeall_4plex.raw
5902076		High	dIEDVFYk	Unambiguous	2	1	N-Term(iTRAQ4plex); K8(iTRAQ4plex)	HCD	1.0000	0.0000	1	1	10719	Unique	Used	0.644	2.826E4	1.985E4	157	3.70	0.00	0	7	162	2	658.85358	1316.69988	1.68	53.67	5225	5225	MS2	14/14	Glioma_Nuclear_prep_Gradeall_repeat_4plex.raw
5902076		High	dIEDVFYk	Unambiguous	2	1	N-Term(iTRAQ4plex); K8(iTRAQ4plex)	HCD	1.0000	0.0000	1	1	10634	Unique	Used	0.740	1.993E4	1.607E4	164	3.56	0.00	0	40	287	2	658.85406	1316.70085	2.42	52.64	5133	5133	MS2	14/14	Glioma_Nuclear_prep_Gradeall_repeat_4plex.raw
6005942	NP_009057.1	VCP	7415	transitional endoplasmic reticulum ATPase [Homo sapiens]	5.21	1	2	2	0.657	1;1	18.1	13.26	806	89.3	5.26																			
6005942		High	gDDLSTAILk	Unambiguous	1	1	N-Term(iTRAQ4plex); K10(iTRAQ4plex)	HCD	1.0000	0.0000	1	1	9674	Unique	Used	0.741	9.390E3	7.585E3	305	4.36	0.00	0	43	300	2	660.88556	1320.76384	1.89	41.23	4102	4102	MS2	17/18	Glioma_Nuclear_prep_Gradeall_repeat_4plex.raw
6005942	High		nAPAIIFIDELDAIAPk	Unambiguous	1	1	N-Term(iTRAQ4plex); K17(iTRAQ4plex)	HCD	1.0000	0.0000	1	1	13106	Unique	Used	0.573	2.228E2	1.401E2	81	4.51	0.00	0	19	300	3	700.40466	2099.19944	0.13	73.56	8250	8250	MS2	28/64	Glioma_Nuclear_prep_Gradeall_4plex.raw
6912292	NP_036249.1	CBX5	23468	chromobox protein homolog 5 [Homo sapiens]	16.75	1	3	4	0.650	3;1	17.1	18.47	191	22.2	5.86																			
6912292	High		nLDcPELISEFMk	Unambiguous	1	1	N-Term(iTRAQ4plex); C4(Methylthio); K13(iTRAQ4plex)	HCD	1.0000	0.0000	1	1	13068	Unique	Used	0.571	2.754E3	1.726E3	149	5.02	0.00	0	32	300	2	936.96429	1872.92131	3.45	71.58	8117	8117	MS2	20/24	Glioma_Nuclear_prep_Gradeall_4plex.raw
6912292	High		nLDcPELISEFmk	Unambiguous	1	1	N-Term(iTRAQ4plex); C4(Methylthio); M12(Oxidation); K13(iTRAQ4plex)	HCD	1.0000	0.0000	1	1	12376	Unique	Used	0.728	7.901E3	6.311E3	125	4.66	0.00	0	41	300	2	944.96069	1888.91411	2.30	60.87	7225	7225	MS2	18/24	Glioma_Nuclear_prep_Gradeall_4plex.raw
6912292		High	sNFSNSADDIk	Unambiguous	1	1	N-Term(iTRAQ4plex); K11(iTRAQ4plex)	HCD	1.0000	0.0000	1	1	8118	Unique	Used	0.546	1.234E4	7.345E3	125	3.93	0.00	0	39	202	2	743.36945	1485.73161	-7.26	24.91	2450	2450	MS2	20/20	Glioma_Nuclear_prep_Gradeall_repeat_4plex.raw
6912292	High		gQVEYLLk	Unambiguous	1	1	N-Term(iTRAQ4plex); K8(iTRAQ4plex)	HCD	1.0000	0.0000	1	1	9000	Unique	Used	0.771	2.145E5	1.815E5	273	3.32	0.00	0	28	10	2	619.36328	1237.71929	-16.31	33.53	3539	3539	MS2	14/14	Glioma_Nuclear_prep_Gradeall_4plex.raw
8923900	NP_061156.1	CMAS	55907	N-acylneuraminate cytidylyltransferase [Homo sapiens]	12.67	1	3	6	0.481	3;3	21.5	19.63	434	48.3	7.93																			
8923900		High	vGLSGAPADAcSTAQk	Unambiguous	1	1	N-Term(iTRAQ4plex); C11(Methylthio); K16(iTRAQ4plex)	HCD	1.0000	0.0000	1	1	9709	Unique	Used	0.579	5.222E3	3.294E3	188	5.89	0.00	0	7	300	2	905.46283	1809.91838	5.85	41.63	4139	4139	MS2	23/30	Glioma_Nuclear_prep_Gradeall_repeat_4plex.raw
8923900	High		vGLSGAPADAcSTAQk	Unambiguous	1	1	N-Term(iTRAQ4plex); C11(Methylthio); K16(iTRAQ4plex)	HCD	1.0000	0.0000	1	1	9221	Unique	Used	0.446	1.659E5	8.113E4	97	5.45	0.00	0	6	11	2	905.44586	1809.88445	-12.90	35.16	3781	3781	MS2	26/30	Glioma_Nuclear_prep_Gradeall_4plex.raw
8923900		High	eVTEPLNLNPAk	Unambiguous	1	1	N-Term(iTRAQ4plex); K12(iTRAQ4plex)	HCD	1.0000	0.0000	1	1	9671	Unique	Used	0.489	8.276E3	4.412E3	226	4.95	0.00	0	34	300	2	806.96393	1612.92058	3.52	41.20	4099	4099	MS2	20/22	Glioma_Nuclear_prep_Gradeall_repeat_4plex.raw
8923900	High		eVTEPLNLNPAk	Unambiguous	1	1	N-Term(iTRAQ4plex); K12(iTRAQ4plex)	HCD	1.0000	0.0000	1	1	9120	Unique	Used	0.473	1.315E5	6.824E4	239	4.08	0.00	0	20	20	2	806.94867	1612.89006	-15.40	34.43	3671	3671	MS2	19/22	Glioma_Nuclear_prep_Gradeall_4plex.raw
8923900		High	gVEkPPHLAALILAR	Unambiguous	1	1	N-Term(iTRAQ4plex); K4(iTRAQ4plex)	HCD	1.0000	0.0000	1	1	10834	Unique	Used	0.305	4.342E2	1.441E2	21	4.63	0.00	0	77	300	4	469.04745	1873.16799	2.86	55.12	5351	5351	MS2	28/84	Glioma_Nuclear_prep_Gradeall_repeat_4plex.raw
8923900	High		gVEkPPHLAALILAR	Unambiguous	1	1	N-Term(iTRAQ4plex); K4(iTRAQ4plex)	HCD	1.0000	0.0000	1	1	9767	Unique	Used	0.586	6.679E4	4.293E4	42	3.88	0.00	0	23	25	2	937.07251	1873.13774	-13.29	39.16	4376	4376	MS2	17/28	Glioma_Nuclear_prep_Gradeall_4plex.raw
10567816	NP_066268.1	GNAO1	2775	guanine nucleotide-binding protein G(o) subunit alpha isoform a [Homo sapiens]	13.56	15	3	3	1.563	2;1	6.0	11.97	354	40.0	5.53																			
10567816	High		iGAADYQPTEQDILR	Unambiguous	2	1	N-Term(iTRAQ4plex)	HCD	1.0000	0.0000	1	1	9898	Unique	Used	1.678	9.391E4	1.730E5	151	4.41	0.00	0	3	13	2	917.46368	1833.92009	-14.33	40.12	4519	4519	MS2	21/28	Glioma_Nuclear_prep_Gradeall_4plex.raw
10567816	High		ILLLGAGESGk	Unambiguous	15	1	N-Term(iTRAQ4plex); K11(iTRAQ4plex)	HCD	1.0000	0.0000	1	1	9904	Unique	Used	1.490	1.203E5	1.967E5	260	3.65	0.00	0	43	16	2	673.40656	1345.80583	-17.49	40.16	4525	4525	MS2	17/20	Glioma_Nuclear_prep_Gradeall_4plex.raw
10567816		High	iIHEDGFSGEDVk	Unambiguous	2	1	N-Term(iTRAQ4plex); K13(iTRAQ4plex)	HCD	1.0000	0.0000	1	1	9029	Unique	Used	1.563	1.644E3	2.801E3	189	4.55	0.00	0	10	300	2	867.45264	1733.89800	1.79	34.06	3413	3413	MS2	21/24	Glioma_Nuclear_prep_Gradeall_repeat_4plex.raw
10800138	NP_066407.1	HIST1H2BD	3017	histone H2B type 1-D [Homo sapiens]	42.06	11	2	5	2.654	2;3	30.1	69.78	126	13.9	10.32																			
10800138		High	kESYSVYVYk	Unambiguous	10	1	N-Term(iTRAQ4plex); K1(iTRAQ4plex); K10(iTRAQ4plex)	HCD	1.0000	0.0000	1	1	8878	Unique	Used	2.021	6.454E3	1.422E4	250	3.57	0.00	1	8	133	2	849.47577	1697.94426	-1.87	32.44	3251	3251	MS2	18/18	Glioma_Nuclear_prep_Gradeall_repeat_4plex.raw
10800138		High	eSYSVYVYk	Unambiguous	10	1	N-Term(iTRAQ4plex); K9(iTRAQ4plex)	HCD	1.0000	0.0000	1	1	9638	Unique	Used	2.654	4.043E3	1.170E4	222	4.22	0.00	0	29	300	2	713.37665	1425.74602	-3.07	40.83	4064	4064	MS2	15/16	Glioma_Nuclear_prep_Gradeall_repeat_4plex.raw
10800138		High	eSYSVYVYk	Unambiguous	10	1	N-Term(iTRAQ4plex); K9(iTRAQ4plex)	HCD	1.0000	0.0000	1	1	9542	Unique	Used	4.349	8.585E3	4.070E4	183	4.08	0.00	0	27	90	2	713.37048	1425.73369	-11.72	39.77	3961	3961	MS2	16/16	Glioma_Nuclear_prep_Gradeall_repeat_4plex.raw
10800138	High		eSYSVYVYk	Unambiguous	10	1	N-Term(iTRAQ4plex); K9(iTRAQ4plex)	HCD	1.0000	0.0000	1	1	9093	Unique	Used	2.564	8.509E4	2.394E5	173	3.72	0.00	0	21	9	2	713.36658	1425.72588	-17.20	34.24	3642	3642	MS2	16/16	Glioma_Nuclear_prep_Gradeall_4plex.raw
10800138	High		eSYSVYVYk	Unambiguous	10	1	N-Term(iTRAQ4plex); K9(iTRAQ4plex)	HCD	1.0000	0.0000	1	1	9009	Unique	Used	3.411	5.070E4	1.898E5	182	4.10	0.00	0	20	23	2	713.36597	1425.72466	-18.05	33.58	3548	3548	MS2	16/16	Glioma_Nuclear_prep_Gradeall_4plex.raw
10800140	NP_066406.1	HIST1H2BB	3018	histone H2B type 1-B [Homo sapiens]	42.06	6	2	3	3.463	2;1	25.4	69.41	126	13.9	10.32																			
10800140	High		kESYSIYVYk	Unambiguous	5	1	N-Term(iTRAQ4plex); K1(iTRAQ4plex); K10(iTRAQ4plex)	HCD	1.0000	0.0000	1	1	8667	Unique	Used	3.463	4.290E4	1.630E5	170	3.34	0.00	1	26	15	2	856.47137	1711.93547	-16.13	31.14	3178	3178	MS2	18/18	Glioma_Nuclear_prep_Gradeall_4plex.raw
10800140		High	eSYSIYVYk	Unambiguous	5	1	N-Term(iTRAQ4plex); K9(iTRAQ4plex)	HCD	1.0000	0.0000	1	1	10054	Unique	Used	4.793	9.556E3	4.992E4	255	4.37	0.00	0	48	226	2	720.38745	1439.76763	1.10	45.74	4512	4512	MS2	15/16	Glioma_Nuclear_prep_Gradeall_repeat_4plex.raw
10800140	High		eSYSIYVYk	Unambiguous	5	1	N-Term(iTRAQ4plex); K9(iTRAQ4plex)	HCD	1.0000	0.0000	1	1	9411	Unique	Used	2.956	1.247E5	4.044E5	219	4.11	0.00	0	26	7	2	720.37354	1439.73979	-18.23	36.59	3990	3990	MS2	15/16	Glioma_Nuclear_prep_Gradeall_4plex.raw
10800144	NP_066544.1	HIST1H2AJ	8331	histone cluster 1, H2aj [Homo sapiens]	27.34	11	1	6	3.829	5;1	22.5	65.65	128	13.9	10.89																			
10800144		High	vTIAQGGVLPNIQAVLLPk	Unambiguous	10	1	N-Term(iTRAQ4plex); K19(iTRAQ4plex)	HCD	1.0000	0.0000	1	1	12459	Unique	Used	3.797	6.621E1	2.740E2	25	5.33	0.00	0	6	129	3	740.46295	2219.37430	0.57	88.80	7985	7985	MS2	26/72	Glioma_Nuclear_prep_Gradeall_repeat_4plex.raw
10800144	High		vTIAQGGVLPNIQAVLLPk	Unambiguous	10	1	N-Term(iTRAQ4plex); K19(iTRAQ4plex)	HCD	1.0000	0.0000	1	1	12555	Unique	Used	2.403	6.047E2	1.594E3	23	6.34	0.00	0	32	147	3	740.46149	2219.36991	-1.41	63.09	7429	7429	MS2	25/72	Glioma_Nuclear_prep_Gradeall_4plex.raw
10800144	High		vTIAQGGVLPNIQAVLLPk	Unambiguous	10	1	N-Term(iTRAQ4plex); K19(iTRAQ4plex)	HCD	1.0000	0.0000	1	1	12498	Unique	Used	3.213	2.104E3	7.420E3	20	5.94	0.00	0	6	14	3	740.46484	2219.37998	3.13	62.33	7363	7363	MS2	28/72	Glioma_Nuclear_prep_Gradeall_4plex.raw
10800144	High		vTIAQGGVLPNIQAVLLPk	Unambiguous	10	1	N-Term(iTRAQ4plex); K19(iTRAQ4plex)	HCD	1.0000	0.0000	1	1	12514	Unique	Used	3.862	2.084E3	8.831E3	21	7.78	0.00	0	3	185	2	1110.18896	2219.37065	-1.07	62.53	7381	7381	MS2	24/36	Glioma_Nuclear_prep_Gradeall_4plex.raw
10800144	High		vTIAQGGVLPNIQAVLLPk	Unambiguous	10	1	N-Term(iTRAQ4plex); K19(iTRAQ4plex)	HCD	1.0000	0.0000	1	1	12404	Unique	Used	4.530	2.046E3	1.017E4	25	7.87	0.00	0	3	249	2	1110.18555	2219.36382	-4.15	61.18	7256	7256	MS2	23/36	Glioma_Nuclear_prep_Gradeall_4plex.raw
10800144	High		vTIAQGGVLPNIQAVLLPk	Unambiguous	10	1	N-Term(iTRAQ4plex); K19(iTRAQ4plex)	HCD	1.0000	0.0000	1	1	12457	Unique	Used	4.663	2.333E4	1.194E5	23	8.00	0.00	0	2	13	2	1110.18958	2219.37187	-0.52	61.81	7316	7316	MS2	27/36	Glioma_Nuclear_prep_Gradeall_4plex.raw
10863927	NP_066953.1	PPIA	5478	peptidyl-prolyl cis-trans isomerase A isoform 1 [Homo sapiens]	19.39	3	3	5	2.766	3;2	12.3	10.67	165	18.0	7.81																			
10863927	High		iIPGFMcQGGDFTR	Unambiguous	2	1	N-Term(iTRAQ4plex); C7(Methylthio)	HCD	1.0000	0.0000	1	1	12014	Unique	Used	2.937	2.883E4	9.292E4	108	3.40	0.00	0	57	132	2	866.41138	1731.81548	0.97	57.13	6828	6828	MS2	12/26	Glioma_Nuclear_prep_Gradeall_4plex.raw
10863927		High	fEDENFILk	Unambiguous	2	1	N-Term(iTRAQ4plex); K9(iTRAQ4plex)	HCD	1.0000	0.0000	1	1	11065	Unique	Used	2.620	4.923E3	1.406E4	223	3.46	0.00	0	36	300	2	721.89392	1442.78057	2.47	58.00	5606	5606	MS2	16/16	Glioma_Nuclear_prep_Gradeall_repeat_4plex.raw
10863927		High	vSFELFADk	Unambiguous	1	1	N-Term(iTRAQ4plex); K9(iTRAQ4plex)	HCD	1.0000	0.0000	1	1	11661	Unique	Used	2.966	3.151E3	1.018E4	232	3.97	0.00	0	26	300	2	672.37524	1343.74321	-1.31	66.45	6324	6324	MS2	16/16	Glioma_Nuclear_prep_Gradeall_repeat_4plex.raw
10863927	High		vSFELFADk	Unambiguous	1	1	N-Term(iTRAQ4plex); K9(iTRAQ4plex)	HCD	1.0000	0.0000	1	1	10769	Unique	Used	2.154	5.468E4	1.293E5	246	4.03	0.00	0	52	38	2	672.36462	1343.72197	-17.11	46.68	5470	5470	MS2	16/16	Glioma_Nuclear_prep_Gradeall_4plex.raw
10863927	High		fEDENFILk	Unambiguous	2	1	N-Term(iTRAQ4plex); K9(iTRAQ4plex)	HCD	1.0000	0.0000	1	1	10234	Unique	Used	2.766	1.200E5	3.642E5	163	3.25	0.00	0	33	13	2	721.88068	1442.75408	-15.89	42.61	4885	4885	MS2	16/16	Glioma_Nuclear_prep_Gradeall_4plex.raw
10863945	NP_066964.1	XRCC5	7520	X-ray repair cross-complementing protein 5 [Homo sapiens]	11.75	1	4	7	0.661	4;3	13.5	34.25	732	82.7	5.81																			
10863945	High		IFQcLLHR	Unambiguous	1	1	N-Term(iTRAQ4plex); C4(Methylthio)	HCD	1.0000	0.0000	1	1	10819	Unique	Used	0.514	9.207E3	5.190E3	192	4.30	0.00	0	0	45	3	407.21744	1219.63776	-15.00	47.06	5524	5524	MS2	17/28	Glioma_Nuclear_prep_Gradeall_4plex.raw
10863945		High	tDTLEDLFPTTk	Unambiguous	1	1	N-Term(iTRAQ4plex); K12(iTRAQ4plex)	HCD	1.0000	0.0000	1	1	11597	Unique	Used	0.661	3.393E3	2.444E3	243	4.63	0.00	0	28	300	2	834.95221	1668.89714	2.19	65.53	6246	6246	MS2	19/22	Glioma_Nuclear_prep_Gradeall_repeat_4plex.raw
10863945	High		tDTLEDLFPTTk	Unambiguous	1	1	N-Term(iTRAQ4plex); K12(iTRAQ4plex)	HCD	1.0000	0.0000	1	1	10810	Unique	Used	0.609	9.140E4	6.111E4	210	5.26	0.00	0	25	19	2	834.93903	1668.87078	-13.61	47.00	5515	5515	MS2	19/22	Glioma_Nuclear_prep_Gradeall_4plex.raw
10863945	High		yAPTEAQLNAVDALIDSMSLAk	Unambiguous	1	1	N-Term(iTRAQ4plex); K22(iTRAQ4plex)	HCD	1.0000	0.0000	1	1	13098	Unique	Used	0.661	9.053E1	6.568E1	151	6.39	0.00	0	24	300	3	870.46484	2609.37998	2.45	73.10	8219	8219	MS2	32/84	Glioma_Nuclear_prep_Gradeall_4plex.raw
10863945		High	tLFPLIEAk	Unambiguous	1	1	N-Term(iTRAQ4plex); K9(iTRAQ4plex)	HCD	1.0000	0.0000	1	1	11872	Unique	Used	0.697	4.175E3	3.172E3	180	3.45	0.00	0	39	300	2	660.41364	1319.81999	1.71	69.79	6600	6600	MS2	15/16	Glioma_Nuclear_prep_Gradeall_repeat_4plex.raw
10863945		High	tLFPLIEAk	Unambiguous	1	1	N-Term(iTRAQ4plex); K9(iTRAQ4plex)	HCD	1.0000	0.0000	1	1	11809	Unique	Used	0.772	3.877E3	3.263E3	186	3.57	0.00	0	45	300	2	660.41266	1319.81804	0.23	68.76	6516	6516	MS2	15/16	Glioma_Nuclear_prep_Gradeall_repeat_4plex.raw
10863945	High		tLFPLIEAk	Unambiguous	1	1	N-Term(iTRAQ4plex); K9(iTRAQ4plex)	HCD	1.0000	0.0000	1	1	10912	Unique	Used	0.766	4.645E4	3.906E4	206	3.32	0.00	0	44	53	2	660.40247	1319.79766	-15.22	47.77	5625	5625	MS2	15/16	Glioma_Nuclear_prep_Gradeall_4plex.raw
12056465	NP_001427.2	FBL	2091	rRNA 2'-O-methyltransferase fibrillarin [Homo sapiens]	31.15	1	9	16	1.638	6;10	11.9	38.70	321	33.8	10.18																			
12056465		High	tNIIPVIEDAR	Unambiguous	1	1	N-Term(iTRAQ4plex)	HCD	1.0000	0.0000	1	1	10723	Unique	Used	1.749	6.268E3	1.195E4	285	3.02	0.00	0	26	300	2	692.89966	1384.79204	0.24	53.72	5229	5229	MS2	17/20	Glioma_Nuclear_prep_Gradeall_repeat_4plex.raw
12056465	High		iVALNAHTFLR	Unambiguous	1	1	N-Term(iTRAQ4plex)	HCD	1.0000	0.0000	1	1	9890	Unique	Used	1.417	1.375E4	2.137E4	110	3.78	0.00	0	25	15	3	466.94162	1398.81031	-16.82	40.06	4510	4510	MS2	24/40	Glioma_Nuclear_prep_Gradeall_4plex.raw
12056465	High		tNIIPVIEDAR	Unambiguous	1	1	N-Term(iTRAQ4plex)	HCD	1.0000	0.0000	1	1	9963	Unique	Used	1.702	9.594E4	1.792E5	303	3.03	0.00	0	33	14	2	692.88831	1384.76933	-16.15	40.58	4589	4589	MS2	17/20	Glioma_Nuclear_prep_Gradeall_4plex.raw
12056465		High	hEGVFIcR	Unambiguous	1	1	N-Term(iTRAQ4plex); C7(Methylthio)	HCD	1.0000	0.0000	1	1	9155	Unique	Used	1.572	4.972E3	8.519E3	116	3.64	0.00	0	18	211	3	384.19284	1150.56397	1.86	35.48	3547	3547	MS2	16/28	Glioma_Nuclear_prep_Gradeall_repeat_4plex.raw
12056465		High	nLVPGESVYGEk	Unambiguous	1	1	N-Term(iTRAQ4plex); K12(iTRAQ4plex)	HCD	1.0000	0.0000	1	1	9248	Unique	Used	1.530	1.021E4	1.703E4	269	3.51	0.00	0	23	198	2	790.43115	1579.85503	-1.27	36.50	3645	3645	MS2	14/22	Glioma_Nuclear_prep_Gradeall_repeat_4plex.raw
12056465		High	nLVPGESVYGEk	Unambiguous	1	1	N-Term(iTRAQ4plex); K12(iTRAQ4plex)	HCD	1.0000	0.0000	1	1	9264	Unique	Used	1.644	2.627E3	4.709E3	221	3.84	0.00	0	14	132	3	527.29169	1579.86051	2.20	36.68	3662	3662	MS2	16/44	Glioma_Nuclear_prep_Gradeall_repeat_4plex.raw
12056465	High		nLVPGESVYGEk	Unambiguous	1	1	N-Term(iTRAQ4plex); K12(iTRAQ4plex)	HCD	1.0000	0.0000	1	1	8809	Unique	Used	1.445	1.518E5	2.408E5	208	3.00	0.00	0	8	9	2	790.41895	1579.83061	-16.73	32.16	3332	3332	MS2	15/22	Glioma_Nuclear_prep_Gradeall_4plex.raw
12056465		High	dHAVVVGVYRPPPk	Unambiguous	1	1	N-Term(iTRAQ4plex); K14(iTRAQ4plex)	HCD	1.0000	0.0000	1	1	8268	Unique	Used	1.576	2.683E3	4.609E3	136	5.33	0.00	0	14	300	2	911.53418	1822.06108	1.79	26.37	2608	2608	MS2	21/26	Glioma_Nuclear_prep_Gradeall_repeat_4plex.raw
12056465		High	dHAVVVGVYRPPPk	Unambiguous	1	1	N-Term(iTRAQ4plex); K14(iTRAQ4plex)	HCD	1.0000	0.0000	1	1	8259	Unique	Used	1.760	3.230E3	6.195E3	103	3.51	0.00	0	27	110	3	608.02539	1822.06162	2.09	26.28	2599	2599	MS2	23/52	Glioma_Nuclear_prep_Gradeall_repeat_4plex.raw
12056465	High		dHAVVVGVYRPPPk	Unambiguous	1	1	N-Term(iTRAQ4plex); K14(iTRAQ4plex)	HCD	1.0000	0.0000	1	1	7928	Unique	Used	2.198	6.724E4	1.622E5	124	3.74	0.00	0	18	2	3	608.02338	1822.05558	-1.23	25.86	2375	2375	MS2	25/52	Glioma_Nuclear_prep_Gradeall_4plex.raw
12056465	High		IAAAILGGVDQIHIkPGAk	Unambiguous	1	1	N-Term(iTRAQ4plex); K15(iTRAQ4plex); K19(iTRAQ4plex)	HCD	1.0000	0.0000	1	1	10893	Unique	Used	1.473	5.211E3	8.425E3	21	4.98	0.00	0	29	7	4	576.85016	2304.37880	-14.77	47.64	5605	5605	MS2	35/108	Glioma_Nuclear_prep_Gradeall_4plex.raw
12056465		High	gkEDALVTk	Unambiguous	1	1	N-Term(iTRAQ4plex); K2(iTRAQ4plex); K9(iTRAQ4plex)	HCD	1.0000	0.0000	1	1	7353	Unique	Used	1.743	1.376E4	2.615E4	218	2.90	0.00	1	12	113	2	696.92523	1392.84319	0.65	17.87	1641	1641	MS2	15/16	Glioma_Nuclear_prep_Gradeall_repeat_4plex.raw
12056465		High	gkEDALVTk	Unambiguous	1	1	N-Term(iTRAQ4plex); K2(iTRAQ4plex); K9(iTRAQ4plex)	HCD	1.0000	0.0000	1	1	7374	Unique	Used	1.803	1.733E4	3.404E4	285	2.89	0.00	1	12	41	2	696.91254	1392.81780	-17.58	18.04	1663	1663	MS2	15/16	Glioma_Nuclear_prep_Gradeall_repeat_4plex.raw
12056465		High	dLINLAk	Unambiguous	1	1	N-Term(iTRAQ4plex); K7(iTRAQ4plex)	HCD	1.0000	0.0000	1	1	9685	Unique	Used	1.632	4.911E4	8.735E4	265	3.26	0.00	0	30	94	2	537.84229	1074.67729	1.07	41.36	4114	4114	MS2	12/12	Glioma_Nuclear_prep_Gradeall_repeat_4plex.raw
12056465		High	vSISEGDDk	Unambiguous	1	1	N-Term(iTRAQ4plex); K9(iTRAQ4plex)	HCD	1.0000	0.0000	1	1	7666	Unique	Used	1.890	3.497E4	7.204E4	243	3.64	0.00	0	27	70	2	619.32074	1237.63420	-13.94	20.60	1974	1974	MS2	16/16	Glioma_Nuclear_prep_Gradeall_repeat_4plex.raw
12056465	High		vSISEGDDk	Unambiguous	1	1	N-Term(iTRAQ4plex); K9(iTRAQ4plex)	HCD	1.0000	0.0000	1	1	7597	Unique	Used	1.499	1.882E5	3.095E5	264	3.16	0.00	0	20	12	2	619.32806	1237.64885	-2.11	23.51	2016	2016	MS2	15/16	Glioma_Nuclear_prep_Gradeall_4plex.raw
13236587	NP_077310.1	TMEM43	79188	transmembrane protein 43 [Homo sapiens]	2.50	1	1	2	2.260	1;1	11.9	3.64	400	44.8	8.13																			
13236587		High	fFLSSGLIDk	Unambiguous	1	1	N-Term(iTRAQ4plex); K10(iTRAQ4plex)	HCD	1.0000	0.0000	1	1	11832	Unique	Used	2.449	1.974E3	5.271E3	234	3.62	0.00	0	27	300	2	707.91193	1414.81658	-1.34	69.14	6547	6547	MS2	18/18	Glioma_Nuclear_prep_Gradeall_repeat_4plex.raw
13236587	High		fFLSSGLIDk	Unambiguous	1	1	N-Term(iTRAQ4plex); K10(iTRAQ4plex)	HCD	1.0000	0.0000	1	1	11040	Unique	Used	2.070	4.297E4	9.762E4	252	3.64	0.00	0	37	42	2	707.91046	1414.81365	-3.41	48.75	5764	5764	MS2	18/18	Glioma_Nuclear_prep_Gradeall_4plex.raw
13676857	NP_068814.2	HSPA2	3306	heat shock-related 70 kDa protein 2 [Homo sapiens]	13.77	1	2	2	0.322	1;1	6.0	27.89	639	70.0	5.74																			

13676857	High		cQEVINWLDR	Unambiguous	1	1	N-Term(iTRAQ4plex); C1(Methylthio)	HCD	1.0000	0.0000	1	1	12170	Unique	Used	0.336	2.892E4	1.067E4	132	3.67	0.00	0	24	241	2	733.35657	1465.70586	0.67	58.66	6998	6998	MS2	16/18	Glioma_Nuclear_prep_Gradeall_4plex.raw
13676857		High	vcNPIISk	Unambiguous	1	1	N-Term(iTRAQ4plex); C2(Methylthio); K8(iTRAQ4plex)	HCD	1.0000	0.0000	1	1	9805	Unique	Used	0.309	1.984E4	6.672E3	357	3.28	0.00	0	69	300	2	604.34686	1207.68645	6.88	42.72	4240	4240	MS2	13/14	Glioma_Nuclear_prep_Gradeall_repeat_4plex.raw
14043072	NP_112533.1	HNRNPA2B1	3181	heterogeneous nuclear ribonucleoproteins A2/B1 isoform B1 [Homo sapiens]	46.74	2	14	36	0.226	14;22	39.5	112.17	353	37.4	8.95																			
14043072		High	nMGGPYGGGNYGPGGSGGSGGYGGR	Unambiguous	2	1	N-Term(iTRAQ4plex)	HCD	1.0000	0.0000	1	1	8851	Unique	Used	0.305	2.137E3	7.109E2	22	8.92	0.00	0	14	179	3	778.67645	2334.01480	3.12	32.16	3222	3222	MS2	28/96	Glioma_Nuclear_prep_Gradeall_repeat_4plex.raw
14043072		High	nMGGPYGGGNYGPGGSGGSGGYGGR	Unambiguous	2	1	N-Term(iTRAQ4plex)	HCD	1.0000	0.0000	1	1	8860	Unique	Used	0.055	1.876E4	1.126E3	17	7.78	0.00	0	0	115	2	1167.50415	2334.00102	-2.78	32.25	3232	3232	MS2	33/48	Glioma_Nuclear_prep_Gradeall_repeat_4plex.raw
14043072		High	iDTIEIITDR	Unambiguous	2	1	N-Term(iTRAQ4plex)	HCD	1.0000	0.0000	1	1	11150	Unique	Used	0.144	1.558E5	2.447E4	221	4.00	0.00	0	7	30	2	666.88007	1332.75286	2.77	59.14	5704	5704	MS2	17/18	Glioma_Nuclear_prep_Gradeall_repeat_4plex.raw
14043072		High	gGGGNFGPGPGSNFR	Unambiguous	2	1	N-Term(iTRAQ4plex)	HCD	1.0000	0.0000	1	1	8567	Unique	Used	0.189	1.939E4	4.001E3	110	5.29	0.00	0	25	123	2	761.37262	1521.73796	4.19	29.30	2924	2924	MS2	25/28	Glioma_Nuclear_prep_Gradeall_repeat_4plex.raw
14043072		High	tLETVPLER	Unambiguous	1	1	N-Term(iTRAQ4plex)	HCD	1.0000	0.0000	1	1	9110	Unique	Used	0.202	1.234E5	2.724E4	205	3.04	0.00	0	17	100	2	601.35022	1201.69316	1.87	34.98	3500	3500	MS2	12/16	Glioma_Nuclear_prep_Gradeall_repeat_4plex.raw
14043072		High	gGNFGFGDSR	Unambiguous	2	1	N-Term(iTRAQ4plex)	HCD	1.0000	0.0000	1	1	8426	Unique	Used	0.267	4.806E4	1.397E4	92	3.51	0.00	0	42	231	2	579.27667	1157.54607	0.36	27.88	2774	2774	MS2	17/18	Glioma_Nuclear_prep_Gradeall_repeat_4plex.raw
14043072		High	iDTIEIITDR	Unambiguous	2	1	N-Term(iTRAQ4plex)	HCD	1.0000	0.0000	1	1	11075	Unique	Used	0.336	1.142E4	4.183E3	307	3.88	0.00	0	33	300	2	666.87964	1332.75200	2.13	58.13	5617	5617	MS2	16/18	Glioma_Nuclear_prep_Gradeall_repeat_4plex.raw
14043072		High	gGNFGFGDSR	Unambiguous	2	1	N-Term(iTRAQ4plex)	HCD	1.0000	0.0000	1	1	8537	Unique	Used	0.455	1.854E4	9.194E3	66	3.47	0.00	0	27	181	2	579.27179	1157.53630	-8.08	28.99	2892	2892	MS2	17/18	Glioma_Nuclear_prep_Gradeall_repeat_4plex.raw
14043072	High		nMGGPYGGGNYGPGGSGGSGGYGGR	Unambiguous	2	1	N-Term(iTRAQ4plex)	HCD	1.0000	0.0000	1	1	8580	Unique	Used	0.103	1.091E5	1.239E4	8	6.76	0.00	0	0	17	2	1167.48657	2333.96587	-17.85	30.46	3081	3081	MS2	35/48	Glioma_Nuclear_prep_Gradeall_4plex.raw
14043072	High		iDTIEIITDR	Unambiguous	2	1	N-Term(iTRAQ4plex)	HCD	1.0000	0.0000	1	1	10357	Unique	Used	0.136	9.457E6	1.412E6	198	3.74	0.00	0	7	1	2	666.86932	1332.73137	-13.35	43.52	5019	5019	MS2	17/18	Glioma_Nuclear_prep_Gradeall_4plex.raw
14043072	High		lFIGGLSFETTEESLR	Unambiguous	2	1	N-Term(iTRAQ4plex)	HCD	1.0000	0.0000	1	1	12251	Unique	Used	0.206	6.302E4	1.425E4	208	5.65	0.00	0	15	116	2	972.01471	1943.02214	-1.11	59.51	7088	7088	MS2	22/30	Glioma_Nuclear_prep_Gradeall_4plex.raw
14043072	High		gGGGNFGPGPGSNFR	Unambiguous	2	1	N-Term(iTRAQ4plex)	HCD	1.0000	0.0000	1	1	8224	Unique	Used	0.269	2.390E5	7.052E4	63	5.36	0.00	0	14	15	2	761.35645	1521.70561	-17.07	27.93	2695	2695	MS2	22/28	Glioma_Nuclear_prep_Gradeall_4plex.raw
14043072	High		lFIGGLSFETTEESLR	Unambiguous	2	1	N-Term(iTRAQ4plex)	HCD	1.0000	0.0000	1	1	12382	Unique	Used	0.290	2.532E4	8.068E3	228	5.43	0.00	0	19	293	2	972.01550	1943.02373	-0.30	60.94	7232	7232	MS2	23/30	Glioma_Nuclear_prep_Gradeall_4plex.raw
14043072	High		lFIGGLSFETTEESLR	Unambiguous	2	1	N-Term(iTRAQ4plex)	HCD	1.0000	0.0000	1	1	12314	Unique	Used	0.392	1.086E5	4.668E4	203	5.62	0.00	0	31	64	2	972.01392	1943.02056	-1.93	60.20	7158	7158	MS2	22/30	Glioma_Nuclear_prep_Gradeall_4plex.raw
14043072	High		qEMQEVQSSR	Unambiguous	2	1	N-Term(iTRAQ4plex)	HCD	1.0000	0.0000	1	1	7285	Unique	Used	0.422	5.571E4	2.577E4	90	3.35	0.00	0	7	45	2	683.32916	1365.65105	-2.85	21.24	1677	1677	MS2	16/18	Glioma_Nuclear_prep_Gradeall_4plex.raw
14043072		High	lTDcVVMR	Unambiguous	2	1	N-Term(iTRAQ4plex); C4(Methylthio)	HCD	1.0000	0.0000	1	1	10860	Unique	Used	0.200	2.349E4	5.124E3	146	3.01	0.00	0	18	288	2	563.78192	1126.55657	2.30	55.43	5379	5379	MS2	13/14	Glioma_Nuclear_prep_Gradeall_repeat_4plex.raw
14043072		High	lTDcVVMR	Unambiguous	2	1	N-Term(iTRAQ4plex); C4(Methylthio)	HCD	1.0000	0.0000	1	1	10778	Unique	Used	0.218	1.356E4	3.224E3	157	2.86	0.00	0	40	300	2	563.78253	1126.55779	3.38	54.39	5288	5288	MS2	12/14	Glioma_Nuclear_prep_Gradeall_repeat_4plex.raw
14043072	High		kLFIGGLSFETTEESLR	Unambiguous	2	1	N-Term(iTRAQ4plex); K1(iTRAQ4plex)	HCD	1.0000	0.0000	1	1	11185	Unique	Used	0.383	1.127E4	4.733E3	105	6.65	0.00	1	28	29	3	739.07678	2215.21579	-2.50	49.91	5923	5923	MS2	27/64	Glioma_Nuclear_prep_Gradeall_4plex.raw
14043072		High	kLFVGGIk	Unambiguous	2	1	N-Term(iTRAQ4plex); K1(iTRAQ4plex); K8(iTRAQ4plex)	HCD	1.0000	0.0000	1	1	8942	Unique	Used	0.236	5.273E4	1.354E4	26	3.03	0.00	1	55	142	2	647.43115	1293.85503	-5.30	33.03	3318	3318	MS2	14/14	Glioma_Nuclear_prep_Gradeall_repeat_4plex.raw
14043072		High	kLFVGGIk	Unambiguous	2	1	N-Term(iTRAQ4plex); K1(iTRAQ4plex); K8(iTRAQ4plex)	HCD	1.0000	0.0000	1	1	8925	Unique	Used	0.289	1.519E4	4.787E3	22	3.38	0.00	1	79	157	3	431.96130	1293.86936	5.77	32.86	3300	3300	MS2	20/28	Glioma_Nuclear_prep_Gradeall_repeat_4plex.raw
14043072		High	kLFVGGIk	Unambiguous	2	1	N-Term(iTRAQ4plex); K1(iTRAQ4plex); K8(iTRAQ4plex)	HCD	1.0000	0.0000	1	1	8749	Unique	Used	0.301	1.310E4	4.296E3	27	3.46	0.00	1	66	115	3	431.95746	1293.85782	-3.14	31.06	3115	3115	MS2	19/28	Glioma_Nuclear_prep_Gradeall_repeat_4plex.raw
14043072		High	kLFVGGIk	Unambiguous	2	1	N-Term(iTRAQ4plex); K1(iTRAQ4plex); K8(iTRAQ4plex)	HCD	1.0000	0.0000	1	1	9099	Unique	Used	0.421	4.237E3	1.945E3	21	3.61	0.00	1	75	240	3	431.95746	1293.85782	-3.14	34.85	3488	3488	MS2	19/28	Glioma_Nuclear_prep_Gradeall_repeat_4plex.raw
14043072	High		kLFVGGIk	Unambiguous	2	1	N-Term(iTRAQ4plex); K1(iTRAQ4plex); K8(iTRAQ4plex)	HCD	1.0000	0.0000	1	1	8346	Unique	Used	0.165	2.189E5	3.959E4	26	3.63	0.00	1	59	3	3	431.95651	1293.85498	-5.34	28.81	2828	2828	MS2	19/28	Glioma_Nuclear_prep_Gradeall_4plex.raw
14043072		High	gFGFVTFDDHDPVDk	Unambiguous	2	1	N-Term(iTRAQ4plex); K15(iTRAQ4plex)	HCD	1.0000	0.0000	1	1	11303	Unique	Used	0.184	4.639E3	9.304E2	106	5.29	0.00	0	4	300	2	992.48987	1983.97246	1.67	61.32	5889	5889	MS2	25/28	Glioma_Nuclear_prep_Gradeall_repeat_4plex.raw
14043072		High	gFGFVTFDDHDPVDk	Unambiguous	2	1	N-Term(iTRAQ4plex); K15(iTRAQ4plex)	HCD	1.0000	0.0000	1	1	11238	Unique	Used	0.210	1.030E4	2.357E3	104	4.58	0.00	0	8	96	3	661.99634	1983.97446	2.68	60.29	5805	5805	MS2	27/56	Glioma_Nuclear_prep_Gradeall_repeat_4plex.raw
14043072		High	gFGFVTFDDHDPVDk	Unambiguous	2	1	N-Term(iTRAQ4plex); K15(iTRAQ4plex)	HCD	1.0000	0.0000	1	1	11302	Unique	Used	0.211	6.564E3	1.506E3	99	5.13	0.00	0	8	139	3	661.99646	1983.97483	2.87	61.30	5888	5888	MS2	29/56	Glioma_Nuclear_prep_Gradeall_repeat_4plex.raw
14043072		High	gFGFVTFDDHDPVDk	Unambiguous	2	1	N-Term(iTRAQ4plex); K15(iTRAQ4plex)	HCD	1.0000	0.0000	1	1	11240	Unique	Used	0.274	5.874E3	1.756E3	102	5.04	0.00	0	3	300	2	992.48981	1983.97234	1.61	60.31	5807	5807	MS2	25/28	Glioma_Nuclear_prep_Gradeall_repeat_4plex.raw
14043072	High		gFGFVTFDDHDPVDk	Unambiguous	2	1	N-Term(iTRAQ4plex); K15(iTRAQ4plex)	HCD	1.0000	0.0000	1	1	10340	Unique	Used	0.200	8.865E5	1.947E5	71	4.36	0.00	0	8	1	3	661.98633	1983.94443	-12.45	43.38	5000	5000	MS2	29/56	Glioma_Nuclear_prep_Gradeall_4plex.raw
14043072	High		gFGFVTFDDHDPVDk	Unambiguous	2	1	N-Term(iTRAQ4plex); K15(iTRAQ4plex)	HCD	1.0000	0.0000	1	1	10320	Unique	Used	0.204	1.858E5	4.153E4	71	5.53	0.00	0	21	17	2	992.47443	1983.94158	-13.89	43.23	4978	4978	MS2	25/28	Glioma_Nuclear_prep_Gradeall_4plex.raw
14043072	High		gFGFVTFDDHDPVDk	Unambiguous	2	1	N-Term(iTRAQ4plex); K15(iTRAQ4plex)	HCD	1.0000	0.0000	1	1	10414	Unique	Used	0.298	1.287E5	4.212E4	73	5.45	0.00	0	15	15	2	992.47351	1983.93974	-14.82	43.95	5081	5081	MS2	25/28	Glioma_Nuclear_prep_Gradeall_4plex.raw
14043072	High		eESGkPGAHVTVk	Unambiguous	2	1	N-Term(iTRAQ4plex); K5(iTRAQ4plex); K13(iTRAQ4plex)	HCD	1.0000	0.0000	1	1	7223	Unique	Used	0.298	3.951E4	1.291E4	151	4.24	0.00	0	10	67	2	886.00848	1771.00969	1.26	20.73	1608	1608	MS2	18/24	Glioma_Nuclear_prep_Gradeall_4plex.raw
14043072		High	dYFEEYGk	Unambiguous	2	1	N-Term(iTRAQ4plex); K8(iTRAQ4plex)	HCD	1.0000	0.0000	1	1	9635	Unique	Used	0.213	7.409E4	1.720E4	88	3.61	0.00	0	36	157	2	669.82617	1338.64507	-0.41	40.80	4061	4061	MS2	13/14	Glioma_Nuclear_prep_Gradeall_repeat_4plex.raw
14043072		High	nYYEQWGk	Unambiguous	2	1	N-Term(iTRAQ4plex); K8(iTRAQ4plex)	HCD	1.0000	0.0000	1	1	9311	Unique	Used	0.438	2.090E4	9.988E3	145	3.05	0.00	0	71	300	2	688.34686	1375.68645	-1.47	37.21	3711	3711	MS2	12/14	Glioma_Nuclear_prep_Gradeall_repeat_4plex.raw
14043072	High		dYFEEYGk	Unambiguous	2	1	N-Term(iTRAQ4plex); K8(iTRAQ4plex)	HCD	1.0000	0.0000	1	1	9232	Unique	Used	0.163	3.931E6	7.016E5	50	2.92	0.00	0	9	1	2	669.81757	1338.62786	-13.27	35.24	3793	3793	MS2	14/14	Glioma_Nuclear_prep_Gradeall_4plex.raw
14043072		High	nmGGPYGGGNYGPGGSGGSGGYGGR	Unambiguous	2	1	N-Term(iTRAQ4plex); M2(Oxidation)	HCD	1.0000	0.0000	1	1	8283	Unique	Used	0.100	6.744E3	7.322E2	21	5.99	0.00	0	0	288	2	1175.51099	2350.01470	5.22	26.52	2624	2624	MS2	29/48	Glioma_Nuclear_prep_Gradeall_repeat_4plex.raw
14043072		High	nmGGPYGGGNYGPGGSGGSGGYGGR	Unambiguous	2	1	N-Term(iTRAQ4plex); M2(Oxidation)	HCD	1.0000	0.0000	1	1	8294	Unique	Used	0.234	1.213E3	3.095E2	20	5.39	0.00	0	11	102	3	784.01093	2350.01822	6.72	26.65	2636	2636	MS2	29/96	Glioma_Nuclear_prep_Gradeall_repeat_4plex.raw
14141159	NP_067676.2	HNRNPH3	3189	heterogeneous nuclear ribonucleoprotein H3 isoform b [Homo sapiens]	17.82	2	4	8	0.504	6;2	30.3	42.66	331	35.2	6.87																			
14141159	High		aTENDIANFFSPLNPIR	Unambiguous	2	1	N-Term(iTRAQ4plex)	HCD	1.0000	0.0000	1	1	12595	Unique	Used	0.311	4.382E4	1.496E4	175	6.96	0.00	0	2	54	2	1032.03723	2063.06719	-0.35	63.66	7479	7479	MS2	25/32	Glioma_Nuclear_prep_Gradeall_4plex.raw
14141159	High		aTENDIANFFSPLNPIR	Unambiguous	2	1	N-Term(iTRAQ4plex)	HCD	1.0000	0.0000	1	1	12546	Unique	Used	0.401	1.154E4	5.075E3	179	6.80	0.00	0	23	300	2	1032.03882	2063.07036	1.19	62.98	7419	7419	MS2	25/32	Glioma_Nuclear_prep_Gradeall_4plex.raw
14141159	High		aTENDIANFFSPLNPIR	Unambiguous	2	1	N-Term(iTRAQ4plex)	HCD	1.0000	0.0000	1	1	12605	Unique	Used	0.731	1.101E3	8.838E2	163	6.44	0.00	0	5	110	3	688.36151	2063.06998	1.01	63.78	7490	7490	MS2	27/64	Glioma_Nuclear_prep_Gradeall_4plex.raw
14141159		High	sTGEAFVQFASk	Unambiguous	2	1	N-Term(iTRAQ4plex); K12(iTRAQ4plex)	HCD	1.0000	0.0000	1	1	10260	Unique	Used	0.397	8.339E3	3.605E3	166	4.37	0.00	0	17	300	2	780.42078	1559.83428	2.21	48.15	4731	4731	MS2	21/22	Glioma_Nuclear_prep_Gradeall_repeat_4plex.raw
14141159		High	sTGEAFVQFASk	Unambiguous	2	1	N-Term(iTRAQ4plex); K12(iTRAQ4plex)	HCD	1.0000	0.0000	1	1	10270	Unique	Used	0.740	1.264E3	1.019E3	214	4.12	0.00	0	37	300	3	520.61572	1559.83261	1.15	48.27	4742	4742	MS2	19/44	Glioma_Nuclear_prep_Gradeall_repeat_4plex.raw
14141159	High		sTGEAFVQFASk	Unambiguous	2	1	N-Term(iTRAQ4plex); K12(iTRAQ4plex)	HCD	1.0000	0.0000	1	1	9584	Unique	Used	0.468	1.957E5	1.005E5	152	4.44	0.00	0	18	14	2	780.40826	1559.80925	-13.83	37.85	4178	4178	MS2	21/22	Glioma_Nuclear_prep_Gradeall_4plex.raw
14141159	High		aTGEADVEFVTHEDAVAAMSk	Unambiguous	2	1	N-Term(iTRAQ4plex); K21(iTRAQ4plex)	HCD	1.0000	0.0000	1	1	10133	Unique	Used	0.541	1.961E4	1.163E4	60	7.47	0.00	0	20	9	3	822.72858	2466.17118	-14.20	41.85	4775	4775	MS2	33/80	Glioma_Nuclear_prep_Gradeall_4plex.raw
14141159	High		eIAENALGk	Unambiguous	2	1	N-Term(iTRAQ4plex); K9(iTRAQ4plex)	HCD	1.0000	0.0000	1	1	8101	Unique	Used	0.614	2.074E5	1.398E5	288	3.80	0.00	0	58	13	2	616.84814	1232.68901	-16.15	27.07	2562	2562	MS2	16/16	Glioma_Nuclear_prep_Gradeall_4plex.raw
14141161	NP_004492.2	HNRNPU	3192	heterogeneous nuclear ribonucleoprotein U isoform b [Homo sapiens]	12.66	2	8	20	0.393	8;12	37.7	68.48	806	88.9	5.78																			
14141161	High		ekPYFPIPEEYTFIQNVPLEDR	Unambiguous	2	1	N-Term(iTRAQ4plex); K2(iTRAQ4plex)	HCD	1.0000	0.0000	1	1	12150	Unique	Used	0.185	6.575E3	1.334E3	108	8.72	0.00	0	6	19	3	1004.86108	3012.56870	2.96	58.49	6977	6977	MS2	27/84	Glioma_Nuclear_prep_Gradeall_4plex.raw
14141161		High	sSGPTSLFAVTVAPPGAR	Unambiguous	2	1	N-Term(iTRAQ4plex)	HCD	1.0000	0.0000	1	1	11487	Unique	Used	0.155	5.456E3	9.191E2	139	6.48	0.00	0	3	300	2	930.01379	1859.02031	3.17	63.98	6114	6114	MS2	27/34	Glioma_Nuclear_prep_Gradeall_repeat_4plex.raw
14141161		High	nFILDQTNVSAAAQR	Unambiguous	2	1	N-Term(iTRAQ4plex)	HCD	1.0000	0.0000	1	1	10328	Unique	Used	0.372	5.771E3	2.337E3	239	4.88	0.00	0	4	300	2	896.47925	1791.95122	2.33	49.00	4805	4805	MS2	22/28	Glioma_Nuclear_prep_Gradeall_repeat_4plex.raw
14141161		High	fIEIAAR	Unambiguous	2	1	N-Term(iTRAQ4plex)	HCD	1.0000	0.0000	1	1	9627	Unique	Used	0.444	2.488E4	1.205E4	333	2.95	0.00	0	28	300	2	482.29086	963.57445	0.03	40.70	4052	4052	MS2	11/12	Glioma_Nuclear_prep_Gradeall_repeat_4plex.raw
14141161		High	sSGPTSLFAVTVAPPGAR	Unambiguous	2	1	N-Term(iTRAQ4plex)	HCD	1.0000	0.0000	1	1	11410	Unique	Used	0.465	3.250E3	1.648E3	151	5.63	0.00	0	17	300	2	930.01428	1859.02129	3.70	62.95	6025	6025	MS2	26/34	Glioma_Nuclear_prep_Gradeall_repeat_4plex.raw
14141161		High	sSGPTSLFAVTVAPPGAR	Unambiguous	2	1	N-Term(iTRAQ4plex)	HCD	1.0000	0.0000	1	1	11409	Unique	Used	0.703	3.110E2	2.384E2	190	4.14	0.00	0	41	300	3	620.34412	1859.01780	1.82	62.93	6024	6024	MS2	18/68	Glioma_Nuclear_prep_Gradeall_repeat_4plex.raw
14141161	High		sSGPTSLFAVTVAPPGAR	Unambiguous	2	1	N-Term(iTRAQ4plex)	HCD	1.0000	0.0000	1	1	10605	Unique	Used	0.291	4.785E5	1.527E5	157	6.61	0.00	0	13	4	2	929.99884	1858.99040	-12.92	45.43	5291	5291	MS2	26/34	Glioma_Nuclear_prep_Gradeall_4plex.raw
14141161	High		nFILDQTNVSAAAQR	Unambiguous	2	1	N-Term(iTRAQ4plex)	HCD	1.0000	0.0000	1	1	9707	Unique	Used	0.384	1.063E5	4.480E4	262	4.77	0.00	0	33	33	2	896.46423	1791.92119	-14.43	38.73	4311	4311	MS2	21/28	Glioma_Nuclear_prep_Gradeall_4plex.raw
14141161		High	gYFEYIEENk	Unambiguous	2	1	N-Term(iTRAQ4plex); K10(iTRAQ4plex)	HCD	1.0000	0.0000	1	1	11212	Unique	Used	0.318	7.422E3	2.573E3	148	4.66	0.00	0	14	300	2	790.39941	1579.79155	2.08	59.96	5776	5776	MS2	18/18	Glioma_Nuclear_prep_Gradeall_repeat_4plex.raw
14141161		High	gYFEYIEENk	Unambiguous	2	1	N-Term(iTRAQ4plex); K10(iTRAQ4plex)	HCD	1.0000	0.0000	1	1	11134	Unique	Used	0.495	6.084E3	3.284E3	168	4.56	0.00	0	44	300	2	790.39966	1579.79204	2.39	58.93	5686	5686	MS2	17/18	Glioma_Nuclear_prep_Gradeall_repeat_4plex.raw
14141161	High		gYFEYIEENk	Unambiguous	2	1	N-Term(iTRAQ4plex); K10(iTRAQ4plex)	HCD	1.0000	0.0000	1	1	10317	Unique	Used	0.404	4.219E5	1.869E5	103	4.52	0.00	0	18	7	2	790.38715	1579.76702	-13.45	43.21	4975	4975	MS2	18/18	Glioma_Nuclear_prep_Gradeall_4plex.raw
14141161		High	yNILGTNTIMDk	Unambiguous	2	1	N-Term(iTRAQ4plex); K12(iTRAQ4plex)	HCD	1.0000	0.0000	1	1	11064	Unique	Used	0.418	3.862E3	1.760E3	258	4.10	0.00	0	83	300	2	835.96014	1670.91301	6.23	57.98	5604	5604	MS2	20/22	Glioma_Nuclear_prep_Gradeall_repeat_4plex.raw
14141161		High	yNILGTNTIMDk	Unambiguous	2	1	N-Term(iTRAQ4plex); K12(iTRAQ4plex)	HCD	1.0000	0.0000	1	1	11149	Unique	Used	0.475	3.341E3	1.730E3	265	3.97	0.00	0	48	300	2	835.95837	1670.90947	4.11	59.12	5702	5702	MS2	18/22	Glioma_Nuclear_prep_Gradeall_repeat_4plex.raw
14141161	High		yNILGTNTIMDk	Unambiguous	2	1	N-Term(iTRAQ4plex); K12(iTRAQ4plex)	HCD	1.0000	0.0000	1	1	10258	Unique	Used	0.321	6.393E5	2.252E5	211	4.39	0.00	0	16	5	2	835.94427	1670.88127	-12.77	42.78	4911	4911	MS2	20/22	Glioma_Nuclear_prep_Gradeall_4plex.raw
14141161		High	vSELkEELk	Unambiguous	2	1	N-Term(iTRAQ4plex); K5(iTRAQ4plex); K9(iTRAQ4plex)	HCD	1.0000	0.0000	1	1	9145	Unique	Used	0.184	1.333E4	2.676E3	189	3.76	0.00	1	31	300	2	753.95679	1506.90630	-2.70	35.36	3536	3536	MS2	16/16	Glioma_Nuclear_prep_Gradeall_repeat_4plex.raw
14141161	High		vSELkEELk	Unambiguous	2	1	N-Term(iTRAQ4plex); K5(iTRAQ4plex); K9(iTRAQ4plex)	HCD	1.0000	0.0000	1	1	8560	Unique	Used	0.109	3.665E5	4.399E4	225	3.58	0.00	1	9	7	2	753.94598	1506.88469	-17.03	30.33	3060	3060	MS2	16/16	Glioma_Nuclear_prep_Gradeall_4plex.raw
14141161		High	dLPEHAVLk	Unambiguous	2	1	N-Term(iTRAQ4plex); K9(iTRAQ4plex)	HCD	1.0000	0.0000	1	1	8227	Unique	Used	0.417	2.185E4	9.928E3	195	3.75	0.00	0	31	298	2	655.39233	1309.77739	4.23	25.99	2565	2565	MS2	15/16	Glioma_Nuclear_prep_Gradeall_repeat_4plex.raw
14141161	High		dLPEHAVLk	Unambiguous	2	1	N-Term(iTRAQ4plex); K9(iTRAQ4plex)	HCD	1.0000	0.0000	1	1	7934	Unique	Used	0.322	4.843E5	1.713E5	242	3.94	0.00	0	18	8	2	655.38977	1309.77226	0.32	25.89	2381	2381	MS2	16/16	Glioma_Nuclear_prep_Gradeall_4plex.raw
14141161		High	yNILGTNTImDk	Unambiguous	2	1	N-Term(iTRAQ4plex); M10(Oxidation); K12(iTRAQ4plex)	HCD	1.0000	0.0000	1	1	10183	Unique	Used	0.419	5.736E3	2.617E3	223	4.31	0.00	0	10	300	2	843.95459	1686.90190	2.60	47.27	4650	4650	MS2	20/22	Glioma_Nuclear_prep_Gradeall_repeat_4plex.raw
14141161	High		yNILGTNTImDk	Unambiguous	2	1	N-Term(iTRAQ4plex); M10(Oxidation); K12(iTRAQ4plex)	HCD	1.0000	0.0000	1	1	9557	Unique	Used	0.403	2.004E5	8.852E4	195	4.09	0.00	0	18	13	2	843.94183	1686.87639	-12.53	37.66	4149	4149	MS2	21/22	Glioma_Nuclear_prep_Gradeall_4plex.raw
14165435	NP_112552.1	HNRNPK	3190	heterogeneous nuclear ribonucleoprotein K isoform b [Homo sapiens]	41.90	2	15	37	0.382	20;17	40.0	115.96	463	50.9	5.54																			
14165435	High		iITITGTQDQIQNAQYLLQNSVk	Unambiguous	2	1	N-Term(iTRAQ4plex); K23(iTRAQ4plex)	HCD	1.0000	0.0000	1	1	12261	Unique	Used	0.548	2.961E4	1.780E4	105	9.52	0.00	0	2	8	3	959.86871	2877.59159	-0.31	59.62	7099	7099	MS2	31/88	Glioma_Nuclear_prep_Gradeall_4plex.raw
14165435		High	tDYNASVSVPDSSGPER	Unambiguous	2	1	N-Term(iTRAQ4plex)	HCD	1.0000	0.0000	1	1	8677	Unique	Used	0.185	2.077E4	4.189E3	91	5.35	0.00	0	8	198	2	962.95660	1924.90593	2.80	30.43	3039	3039	MS2	23/32	Glioma_Nuclear_prep_Gradeall_repeat_4plex.raw
14165435		High	nLPLPPPPPPR	Unambiguous	2	1	N-Term(iTRAQ4plex)	HCD	1.0000	0.0000	1	1	9451	Unique	Used	0.212	5.110E4	1.180E4	172	3.32	0.00	0	30	160	2	669.90662	1338.80596	3.34	38.68	3859	3859	MS2	14/20	Glioma_Nuclear_prep_Gradeall_repeat_4plex.raw
14165435		High	iDEPLEGSEDR	Unambiguous	2	1	N-Term(iTRAQ4plex)	HCD	1.0000	0.0000	1	1	8457	Unique	Used	0.247	5.372E4	1.448E4	109	3.75	0.00	0	25	142	2	702.34180	1403.67632	-0.58	28.18	2807	2807	MS2	18/20	Glioma_Nuclear_prep_Gradeall_repeat_4plex.raw
14165435		High	nLPLPPPPPPR	Unambiguous	2	1	N-Term(iTRAQ4plex)	HCD	1.0000	0.0000	1	1	9625	Unique	Used	0.279	3.070E4	9.325E3	191	3.03	0.00	0	44	300	2	669.90399	1338.80071	-0.58	40.68	4050	4050	MS2	13/20	Glioma_Nuclear_prep_Gradeall_repeat_4plex.raw
14165435		High	gGDLMAYDR	Unambiguous	2	1	N-Term(iTRAQ4plex)	HCD	1.0000	0.0000	1	1	8570	Unique	Used	0.400	3.691E4	1.609E4	99	3.31	0.00	0	26	133	2	571.27716	1141.54704	3.65	29.32	2927	2927	MS2	15/16	Glioma_Nuclear_prep_Gradeall_repeat_4plex.raw
14165435		High	nLPLPPPPPPR	Unambiguous	2	1	N-Term(iTRAQ4plex)	HCD	1.0000	0.0000	1	1	9354	Unique	Used	0.440	2.149E4	1.030E4	194	3.35	0.00	0	52	300	2	669.90564	1338.80400	1.88	37.66	3756	3756	MS2	14/20	Glioma_Nuclear_prep_Gradeall_repeat_4plex.raw
14165435		High	nLPLPPPPPPR	Unambiguous	2	1	N-Term(iTRAQ4plex)	HCD	1.0000	0.0000	1	1	9485	Unique	Used	0.626	2.959E3	2.021E3	142	3.85	0.00	0	46	300	3	446.94080	1338.80783	4.74	39.09	3896	3896	MS2	14/40	Glioma_Nuclear_prep_Gradeall_repeat_4plex.raw
14165435	High		tDYNASVSVPDSSGPER	Unambiguous	2	1	N-Term(iTRAQ4plex)	HCD	1.0000	0.0000	1	1	8503	Unique	Used	0.166	1.836E5	3.354E4	59	4.94	0.00	0	0	8	2	962.93762	1924.86797	-16.92	29.92	2998	2998	MS2	24/32	Glioma_Nuclear_prep_Gradeall_4plex.raw
14165435	High		nLPLPPPPPPR	Unambiguous	2	1	N-Term(iTRAQ4plex)	HCD	1.0000	0.0000	1	1	8955	Unique	Used	0.255	7.780E5	2.176E5	243	2.99	0.00	0	9	4	2	669.89429	1338.78130	-15.08	33.20	3490	3490	MS2	14/20	Glioma_Nuclear_prep_Gradeall_4plex.raw
14165435	High		nLPLPPPPPPR	Unambiguous	2	1	N-Term(iTRAQ4plex)	HCD	1.0000	0.0000	1	1	8857	Unique	Used	0.293	5.933E5	1.910E5	233	3.05	0.00	0	13	6	2	669.89417	1338.78105	-15.26	32.51	3384	3384	MS2	14/20	Glioma_Nuclear_prep_Gradeall_4plex.raw
14165435	High		iDEPLEGSEDR	Unambiguous	2	1	N-Term(iTRAQ4plex)	HCD	1.0000	0.0000	1	1	8263	Unique	Used	0.323	5.240E5	1.860E5	80	3.78	0.00	0	24	7	2	702.33099	1403.65471	-15.98	28.22	2738	2738	MS2	16/20	Glioma_Nuclear_prep_Gradeall_4plex.raw
14165435		High	lFQEccPHSTDR	Unambiguous	2	1	N-Term(iTRAQ4plex); C5(Methylthio); C6(Methylthio)	HCD	1.0000	0.0000	1	1	11383	Unique	Used	0.240	1.695E3	4.442E2	18	4.10	0.00	0	59	300	3	557.90173	1671.69065	2.27	62.55	5991	5991	MS2	22/44	Glioma_Nuclear_prep_Gradeall_repeat_4plex.raw
14165435		High	gSDFDcELR	Unambiguous	2	1	N-Term(iTRAQ4plex); C6(Methylthio)	HCD	1.0000	0.0000	1	1	10007	Unique	Used	0.355	2.043E4	7.912E3	13	3.42	0.00	0	34	300	2	616.26532	1231.52336	2.39	45.13	4459	4459	MS2	16/16	Glioma_Nuclear_prep_Gradeall_repeat_4plex.raw
14165435	High		iILDLISESPIk	Unambiguous	2	1	N-Term(iTRAQ4plex); K12(iTRAQ4plex)	HCD	1.0000	0.0000	1	1	12718	Unique	Used	0.382	6.980E4	2.929E4	70	5.22	0.00	0	6	81	2	815.01025	1629.01323	3.37	65.23	7618	7618	MS2	20/22	Glioma_Nuclear_prep_Gradeall_4plex.raw
14165435	High		iILDLISESPIk	Unambiguous	2	1	N-Term(iTRAQ4plex); K12(iTRAQ4plex)	HCD	1.0000	0.0000	1	1	12772	Unique	Used	0.408	1.296E4	5.798E3	66	5.23	0.00	0	17	300	2	815.01111	1629.01494	4.42	65.94	7680	7680	MS2	20/22	Glioma_Nuclear_prep_Gradeall_4plex.raw
14165435	High		iILDLISESPIk	Unambiguous	2	1	N-Term(iTRAQ4plex); K12(iTRAQ4plex)	HCD	1.0000	0.0000	1	1	12728	Unique	Used	0.449	1.085E4	5.349E3	63	4.88	0.00	0	5	28	3	543.67554	1629.01206	2.65	65.36	7629	7629	MS2	19/44	Glioma_Nuclear_prep_Gradeall_4plex.raw
14165435	High		iILDLISESPIk	Unambiguous	2	1	N-Term(iTRAQ4plex); K12(iTRAQ4plex)	HCD	1.0000	0.0000	1	1	12672	Unique	Used	0.455	1.521E3	7.590E2	71	4.61	0.00	0	38	176	3	543.67419	1629.00803	0.18	64.65	7566	7566	MS2	18/44	Glioma_Nuclear_prep_Gradeall_4plex.raw
14165435	High		iILDLISESPIk	Unambiguous	2	1	N-Term(iTRAQ4plex); K12(iTRAQ4plex)	HCD	1.0000	0.0000	1	1	12664	Unique	Used	0.549	1.014E4	6.113E3	70	5.26	0.00	0	29	300	2	815.00928	1629.01128	2.17	64.56	7557	7557	MS2	20/22	Glioma_Nuclear_prep_Gradeall_4plex.raw
14165435		High	rPAEDMEEEQAFk	Unambiguous	2	1	N-Term(iTRAQ4plex); K13(iTRAQ4plex)	HCD	1.0000	0.0000	1	1	8459	Unique	Used	0.540	9.290E3	5.471E3	83	3.68	0.00	0	49	231	2	934.46021	1867.91313	1.72	28.19	2809	2809	MS2	17/24	Glioma_Nuclear_prep_Gradeall_repeat_4plex.raw
14165435	High		rPAEDMEEEQAFk	Unambiguous	2	1	N-Term(iTRAQ4plex); K13(iTRAQ4plex)	HCD	1.0000	0.0000	1	1	8133	Unique	Used	0.419	2.044E5	9.390E4	47	4.44	0.00	0	35	10	2	934.44543	1867.88359	-14.09	27.30	2597	2597	MS2	16/24	Glioma_Nuclear_prep_Gradeall_4plex.raw
14165435		High	lLIHQSLAGGIIGVk	Unambiguous	2	1	N-Term(iTRAQ4plex); K15(iTRAQ4plex)	HCD	1.0000	0.0000	1	1	11369	Unique	Used	0.710	4.899E2	3.790E2	17	5.25	0.00	0	32	300	3	603.05334	1807.14548	2.58	62.36	5975	5975	MS2	26/56	Glioma_Nuclear_prep_Gradeall_repeat_4plex.raw
14165435	High		lLIHQSLAGGIIGVk	Unambiguous	2	1	N-Term(iTRAQ4plex); K15(iTRAQ4plex)	HCD	1.0000	0.0000	1	1	10294	Unique	Used	0.346	2.049E5	7.769E4	38	5.11	0.00	0	51	16	2	904.06311	1807.11894	-12.11	43.04	4950	4950	MS2	22/28	Glioma_Nuclear_prep_Gradeall_4plex.raw
14165435	High		lLIHQSLAGGIIGVk	Unambiguous	2	1	N-Term(iTRAQ4plex); K15(iTRAQ4plex)	HCD	1.0000	0.0000	1	1	10282	Unique	Used	0.635	3.115E4	2.169E4	38	4.77	0.00	0	43	7	3	603.04309	1807.11472	-14.45	42.95	4937	4937	MS2	25/56	Glioma_Nuclear_prep_Gradeall_4plex.raw
14165435	High		iLSISADIETIGEILk	Unambiguous	2	1	N-Term(iTRAQ4plex); K16(iTRAQ4plex)	HCD	1.0000	0.0000	1	1	13145	Unique	Used	0.531	3.072E2	1.792E2	55	3.45	0.00	0	35	300	3	668.40332	2003.19541	3.74	78.31	8559	8559	MS2	20/60	Glioma_Nuclear_prep_Gradeall_4plex.raw
14165435		High	gSYGDLGGPIITTQVTIPk	Unambiguous	2	1	N-Term(iTRAQ4plex); K19(iTRAQ4plex)	HCD	1.0000	0.0000	1	1	11600	Unique	Used	0.215	2.141E3	5.023E2	91	6.58	0.00	0	4	300	2	1103.12415	2205.24101	1.83	65.57	6249	6249	MS2	29/36	Glioma_Nuclear_prep_Gradeall_repeat_4plex.raw
14165435	High		gSYGDLGGPIITTQVTIPk	Unambiguous	2	1	N-Term(iTRAQ4plex); K19(iTRAQ4plex)	HCD	1.0000	0.0000	1	1	10788	Unique	Used	0.199	1.601E5	3.499E4	125	6.80	0.00	0	1	6	2	1103.10657	2205.20586	-14.11	46.83	5491	5491	MS2	27/36	Glioma_Nuclear_prep_Gradeall_4plex.raw
14165435	High		iITITGTQDQIQNAQYLLQNSVk	Unambiguous	2	1	N-Term(iTRAQ4plex); K23(iTRAQ4plex)	HCD	1.0000	0.0000	1	1	12270	Unique	Used	0.124	1.667E4	2.264E3	108	7.93	0.00	0	5	69	2	1439.29895	2877.59062	-0.64	59.72	7109	7109	MS2	28/44	Glioma_Nuclear_prep_Gradeall_4plex.raw
14165435	High		iITITGTQDQIQNAQYLLQNSVk	Unambiguous	2	1	N-Term(iTRAQ4plex); K23(iTRAQ4plex)	HCD	1.0000	0.0000	1	1	12243	Unique	Used	0.156	1.401E3	2.399E2	89	8.16	0.00	0	49	116	4	720.15308	2877.59047	-0.69	59.43	7079	7079	MS2	32/132	Glioma_Nuclear_prep_Gradeall_4plex.raw
14165435	High		vVLIGGkPDR	Unambiguous	2	1	N-Term(iTRAQ4plex); K7(iTRAQ4plex)	HCD	1.0000	0.0000	1	1	7996	Unique	Used	0.443	5.256E5	2.555E5	103	3.28	0.00	0	31	6	2	671.41730	1341.82732	-13.70	26.33	2448	2448	MS2	15/18	Glioma_Nuclear_prep_Gradeall_4plex.raw
14165435		High	eNTQTTIk	Unambiguous	2	1	N-Term(iTRAQ4plex); K8(iTRAQ4plex)	HCD	1.0000	0.0000	1	1	6878	Unique	Used	0.236	7.185E4	1.848E4	307	3.13	0.00	0	22	58	2	611.84772	1222.68816	0.00	13.68	1139	1139	MS2	14/14	Glioma_Nuclear_prep_Gradeall_repeat_4plex.raw
14165435		High	dLAGSIIGk	Unambiguous	2	1	N-Term(iTRAQ4plex); K9(iTRAQ4plex)	HCD	1.0000	0.0000	1	1	9578	Unique	Used	0.393	3.618E4	1.549E4	299	3.51	0.00	0	49	177	2	581.35773	1161.70818	-0.01	40.17	4000	4000	MS2	15/16	Glioma_Nuclear_prep_Gradeall_repeat_4plex.raw
14165435		High	dLAGSIIGk	Unambiguous	2	1	N-Term(iTRAQ4plex); K9(iTRAQ4plex)	HCD	1.0000	0.0000	1	1	9695	Unique	Used	0.628	1.288E4	8.817E3	329	3.44	0.00	0	59	300	2	581.35870	1161.71013	1.67	41.46	4124	4124	MS2	15/16	Glioma_Nuclear_prep_Gradeall_repeat_4plex.raw
14165435	High		dLAGSIIGk	Unambiguous	2	1	N-Term(iTRAQ4plex); K9(iTRAQ4plex)	HCD	1.0000	0.0000	1	1	9116	Unique	Used	0.400	3.074E5	1.350E5	347	3.47	0.00	0	44	12	2	581.34711	1161.68694	-18.29	34.41	3667	3667	MS2	15/16	Glioma_Nuclear_prep_Gradeall_4plex.raw
14165435		High	rPAEDmEEEQAFk	Unambiguous	2	1	N-Term(iTRAQ4plex); M6(Oxidation); K13(iTRAQ4plex)	HCD	1.0000	0.0000	1	1	7736	Unique	Used	0.339	3.938E3	1.453E3	104	5.84	0.00	0	16	77	3	628.64093	1883.90824	1.81	21.29	2048	2048	MS2	20/48	Glioma_Nuclear_prep_Gradeall_repeat_4plex.raw
14165435		High	rPAEDmEEEQAFk	Unambiguous	2	1	N-Term(iTRAQ4plex); M6(Oxidation); K13(iTRAQ4plex)	HCD	1.0000	0.0000	1	1	7766	Unique	Used	0.352	2.370E3	9.082E2	83	3.16	0.00	0	8	300	2	942.45447	1883.90166	-1.68	21.56	2079	2079	MS2	15/24	Glioma_Nuclear_prep_Gradeall_repeat_4plex.raw
14165435	High		rPAEDmEEEQAFk	Unambiguous	2	1	N-Term(iTRAQ4plex); M6(Oxidation); K13(iTRAQ4plex)	HCD	1.0000	0.0000	1	1	7642	Unique	Used	0.505	1.693E4	9.372E3	86	4.44	0.00	0	20	8	3	628.64032	1883.90641	0.84	23.83	2065	2065	MS2	22/48	Glioma_Nuclear_prep_Gradeall_4plex.raw
14165466	NP_114368.1	PTBP1	5725	polypyrimidine tract-binding protein 1 isoform c [Homo sapiens]	17.51	9	5	6	1.582	2;4	21.1	31.83	531	57.2	9.17																			
14165466	High		kLPIDVTEGEVISLGLPFGk	Unambiguous	3	1	N-Term(iTRAQ4plex); K1(iTRAQ4plex); K20(iTRAQ4plex)	HCD	1.0000	0.0000	1	1	12528	Unique	Used	1.354	5.589E2	8.306E2	27	9.13	0.00	1	13	300	3	848.83893	2544.50223	0.33	62.73	7398	7398	MS2	27/76	Glioma_Nuclear_prep_Gradeall_4plex.raw
14165466		High	hQNVQLPR	Unambiguous	3	1	N-Term(iTRAQ4plex)	HCD	1.0000	0.0000	1	1	7121	Unique	Used	1.466	4.102E3	6.554E3	310	3.74	0.00	0	23	104	3	379.21384	1135.62696	-16.14	15.87	1397	1397	MS2	17/28	Glioma_Nuclear_prep_Gradeall_repeat_4plex.raw
14165466	High		iAIPGLAGAGNSVLLVSNLNPER	Unambiguous	3	1	N-Term(iTRAQ4plex)	HCD	1.0000	0.0000	1	1	12569	Unique	Used	2.274	2.203E3	5.498E3	61	6.82	0.00	0	0	300	2	1210.18982	2419.37236	-2.76	63.24	7444	7444	MS2	27/44	Glioma_Nuclear_prep_Gradeall_4plex.raw
14165466		High	eGQEDQGLTk	Unambiguous	9	1	N-Term(iTRAQ4plex); K10(iTRAQ4plex)	HCD	1.0000	0.0000	1	1	7170	Unique	Used	1.407	6.247E3	9.581E3	179	2.96	0.00	0	17	140	2	696.86163	1392.71599	-3.55	16.30	1448	1448	MS2	15/18	Glioma_Nuclear_prep_Gradeall_repeat_4plex.raw
14165466		High	gQPIYIQFSNHk	Unambiguous	3	1	N-Term(iTRAQ4plex); K12(iTRAQ4plex)	HCD	1.0000	0.0000	1	1	9572	Unique	Used	1.699	2.785E3	5.156E3	260	4.39	0.00	0	0	300	2	860.46863	1719.92998	-7.04	40.09	3993	3993	MS2	19/22	Glioma_Nuclear_prep_Gradeall_repeat_4plex.raw
14165466		High	gQPIYIQFSNHk	Unambiguous	3	1	N-Term(iTRAQ4plex); K12(iTRAQ4plex)	HCD	1.0000	0.0000	1	1	9543	Unique	Used	1.914	1.747E3	3.644E3	158	6.23	0.00	0	35	170	3	573.97870	1719.92154	-11.94	39.78	3962	3962	MS2	26/44	Glioma_Nuclear_prep_Gradeall_repeat_4plex.raw
16507237	NP_005338.1	HSPA5	3309	78 kDa glucose-regulated protein precursor [Homo sapiens]	38.07	1	14	22	0.640	13;9	17.9	82.15	654	72.3	5.16																			
16507237		High	vTHAVVTVPAYFNDAQR	Unambiguous	1	1	N-Term(iTRAQ4plex)	HCD	1.0000	0.0000	1	1	10433	Unique	Used	0.582	1.339E3	8.494E2	260	4.42	0.00	0	8	300	2	1016.54010	2032.07292	-0.19	50.21	4916	4916	MS2	19/32	Glioma_Nuclear_prep_Gradeall_repeat_4plex.raw
16507237		High	iTPSYVAFTPEGER	Unambiguous	1	1	N-Term(iTRAQ4plex)	HCD	1.0000	0.0000	1	1	10625	Unique	Used	0.615	5.877E3	3.940E3	243	4.43	0.00	0	37	300	2	855.94690	1710.88652	2.66	52.53	5123	5123	MS2	19/26	Glioma_Nuclear_prep_Gradeall_repeat_4plex.raw
16507237		High	iTITNDQNR	Unambiguous	1	1	N-Term(iTRAQ4plex)	HCD	1.0000	0.0000	1	1	7699	Unique	Used	0.639	2.962E4	2.062E4	252	3.29	0.00	0	35	198	2	609.82666	1218.64604	-8.10	20.92	2009	2009	MS2	14/16	Glioma_Nuclear_prep_Gradeall_repeat_4plex.raw
16507237		High	iTPSYVAFTPEGER	Unambiguous	1	1	N-Term(iTRAQ4plex)	HCD	1.0000	0.0000	1	1	10640	Unique	Used	0.769	7.245E2	6.074E2	185	4.82	0.00	0	26	300	3	570.96729	1710.88730	3.11	52.71	5139	5139	MS2	19/52	Glioma_Nuclear_prep_Gradeall_repeat_4plex.raw
16507237	High		iTPSYVAFTPEGER	Unambiguous	1	1	N-Term(iTRAQ4plex)	HCD	1.0000	0.0000	1	1	9942	Unique	Used	0.551	5.099E5	3.085E5	141	4.12	0.00	0	10	5	2	855.93298	1710.85869	-13.61	40.44	4567	4567	MS2	17/26	Glioma_Nuclear_prep_Gradeall_4plex.raw
16507237	High		kSDIDEIVLVGGSTR	Unambiguous	1	1	N-Term(iTRAQ4plex); K1(iTRAQ4plex)	HCD	1.0000	0.0000	1	1	9541	Unique	Used	0.707	6.014E4	4.668E4	172	4.26	0.00	1	0	29	2	939.01959	1877.03191	-14.05	37.54	4131	4131	MS2	21/28	Glioma_Nuclear_prep_Gradeall_4plex.raw
16507237		High	nELESYAYSLk	Unambiguous	1	1	N-Term(iTRAQ4plex); K11(iTRAQ4plex)	HCD	1.0000	0.0000	1	1	11147	Unique	Used	0.502	6.326E3	3.459E3	221	4.94	0.00	0	17	300	2	802.92560	1604.84392	1.80	59.09	5700	5700	MS2	19/20	Glioma_Nuclear_prep_Gradeall_repeat_4plex.raw
16507237	High		nELESYAYSLk	Unambiguous	1	1	N-Term(iTRAQ4plex); K11(iTRAQ4plex)	HCD	1.0000	0.0000	1	1	10296	Unique	Used	0.551	1.599E5	9.670E4	194	4.36	0.00	0	46	20	2	802.91260	1604.81792	-14.40	43.05	4952	4952	MS2	19/20	Glioma_Nuclear_prep_Gradeall_4plex.raw
16507237		High	eLEEIVQPIISk	Unambiguous	1	1	N-Term(iTRAQ4plex); K12(iTRAQ4plex)	HCD	1.0000	0.0000	1	1	11879	Unique	Used	0.672	2.342E3	1.715E3	149	5.04	0.00	0	18	300	2	843.50269	1685.99809	3.13	69.88	6608	6608	MS2	21/22	Glioma_Nuclear_prep_Gradeall_repeat_4plex.raw
16507237	High		eLEEIVQPIISk	Unambiguous	1	1	N-Term(iTRAQ4plex); K12(iTRAQ4plex)	HCD	1.0000	0.0000	1	1	11087	Unique	Used	0.720	6.900E4	5.455E4	155	5.67	0.00	0	35	33	2	843.49994	1685.99260	-0.13	49.14	5816	5816	MS2	19/22	Glioma_Nuclear_prep_Gradeall_4plex.raw
16507237	High		eLEEIVQPIISk	Unambiguous	1	1	N-Term(iTRAQ4plex); K12(iTRAQ4plex)	HCD	1.0000	0.0000	1	1	11088	Unique	Used	0.770	6.743E3	5.700E3	136	3.59	0.00	0	48	22	3	562.66681	1685.98587	-4.12	49.15	5817	5817	MS2	13/44	Glioma_Nuclear_prep_Gradeall_4plex.raw
16507237	High		tFAPEEISAMVLTk	Unambiguous	1	1	N-Term(iTRAQ4plex); K14(iTRAQ4plex)	HCD	1.0000	0.0000	1	1	11766	Unique	Used	0.451	9.449E4	4.673E4	247	5.03	0.00	0	45	50	2	913.00800	1825.00871	3.67	54.92	6557	6557	MS2	24/26	Glioma_Nuclear_prep_Gradeall_4plex.raw
16507237	High		tFAPEEISAMVLTk	Unambiguous	1	1	N-Term(iTRAQ4plex); K14(iTRAQ4plex)	HCD	1.0000	0.0000	1	1	11752	Unique	Used	0.549	8.735E3	5.258E3	240	5.34	0.00	0	16	32	3	609.00439	1824.99863	-1.86	54.81	6542	6542	MS2	24/52	Glioma_Nuclear_prep_Gradeall_4plex.raw
16507237	High		nQLTSNPENTVFDAk	Unambiguous	1	1	N-Term(iTRAQ4plex); K15(iTRAQ4plex)	HCD	1.0000	0.0000	1	1	9344	Unique	Used	0.542	1.617E5	9.624E4	136	5.34	0.00	0	9	10	2	983.49347	1965.97966	-16.47	36.10	3917	3917	MS2	23/28	Glioma_Nuclear_prep_Gradeall_4plex.raw
16507237	High		nQLTSNPENTVFDAk	Unambiguous	1	1	N-Term(iTRAQ4plex); K15(iTRAQ4plex)	HCD	1.0000	0.0000	1	1	9325	Unique	Used	0.729	9.077E4	7.263E4	162	5.00	0.00	0	34	25	2	983.50903	1966.01079	-0.64	35.96	3896	3896	MS2	19/28	Glioma_Nuclear_prep_Gradeall_4plex.raw
16507237		High	iEWLESHQDADIEDFk	Unambiguous	1	1	N-Term(iTRAQ4plex); K16(iTRAQ4plex)	HCD	1.0000	0.0000	1	1	11766	Unique	Used	0.375	3.688E2	1.505E2	105	4.55	0.00	0	34	300	3	755.04388	2263.11710	2.18	67.99	6454	6454	MS2	24/60	Glioma_Nuclear_prep_Gradeall_repeat_4plex.raw
16507237	High		iEWLESHQDADIEDFk	Unambiguous	1	1	N-Term(iTRAQ4plex); K16(iTRAQ4plex)	HCD	1.0000	0.0000	1	1	10875	Unique	Used	0.750	1.505E4	1.238E4	75	5.38	0.00	0	40	22	3	755.03278	2263.08377	-12.55	47.48	5585	5585	MS2	30/60	Glioma_Nuclear_prep_Gradeall_4plex.raw
16507237	High		sQIFSTASDNQPTVTIk	Unambiguous	1	1	N-Term(iTRAQ4plex); K17(iTRAQ4plex)	HCD	1.0000	0.0000	1	1	9560	Unique	Used	0.659	7.345E4	5.311E4	203	5.63	0.00	0	10	16	2	1063.05640	2125.10552	-15.27	37.68	4152	4152	MS2	26/32	Glioma_Nuclear_prep_Gradeall_4plex.raw
16507237	High		tkPYIQVDIGGGQTk	Unambiguous	1	1	N-Term(iTRAQ4plex); K2(iTRAQ4plex); K15(iTRAQ4plex)	HCD	1.0000	0.0000	1	1	8697	Unique	Used	0.497	5.435E4	2.966E4	107	5.00	0.00	0	0	20	2	1019.07404	2037.14079	-14.58	31.35	3210	3210	MS2	23/28	Glioma_Nuclear_prep_Gradeall_4plex.raw
16507237		High	vLEDSDLk	Unambiguous	1	1	N-Term(iTRAQ4plex); K8(iTRAQ4plex)	HCD	1.0000	0.0000	1	1	8616	Unique	Used	0.699	1.920E4	1.464E4	315	3.04	0.00	0	47	294	2	603.84644	1206.68559	2.96	29.80	2975	2975	MS2	14/14	Glioma_Nuclear_prep_Gradeall_repeat_4plex.raw
16507237	High		dAGTIAGLNVmR	Unambiguous	1	1	N-Term(iTRAQ4plex); M11(Oxidation)	HCD	1.0000	0.0000	1	1	8769	Unique	Used	0.755	9.998E4	8.284E4	223	3.59	0.00	0	9	14	2	689.35663	1377.70598	-15.79	31.86	3288	3288	MS2	17/22	Glioma_Nuclear_prep_Gradeall_4plex.raw
16507237		High	fEELNmDLFR	Unambiguous	1	1	N-Term(iTRAQ4plex); M6(Oxidation)	HCD	1.0000	0.0000	1	1	11669	Unique	Used	0.642	5.569E3	3.894E3	135	2.97	0.00	0	35	300	2	737.36353	1473.71977	2.22	66.56	6333	6333	MS2	15/18	Glioma_Nuclear_prep_Gradeall_repeat_4plex.raw
19913408	NP_001059.2	TOP2B	7155	DNA topoisomerase 2-beta [Homo sapiens]	16.84	2	17	24	1.546	17;7	33.1	98.48	1621	182.5	8.06																			
19913408		High	sLAVSGLGVIGR	Unambiguous	1	1	N-Term(iTRAQ4plex)	HCD	1.0000	0.0000	1	1	10775	Unique	Used	1.771	6.110E3	1.179E4	211	3.35	0.00	0	45	300	2	636.89185	1272.77641	0.59	54.35	5285	5285	MS2	21/22	Glioma_Nuclear_prep_Gradeall_repeat_4plex.raw
19913408	High		dLIQMLVQR	Unambiguous	1	1	N-Term(iTRAQ4plex)	HCD	1.0000	0.0000	1	1	11627	Unique	Used	2.304	1.701E4	4.301E4	286	3.54	0.00	0	26	65	2	630.36456	1259.72185	-3.50	53.64	6406	6406	MS2	15/16	Glioma_Nuclear_prep_Gradeall_4plex.raw
19913408	High		yIFTMLSTLAR	Unambiguous	1	1	N-Term(iTRAQ4plex)	HCD	1.0000	0.0000	1	1	13037	Unique	Used	3.344	4.033E3	1.480E4	272	3.79	0.00	0	21	300	2	730.40845	1459.80962	-0.25	70.62	8048	8048	MS2	17/20	Glioma_Nuclear_prep_Gradeall_4plex.raw
19913408	High		kLQLEETMPSPYGR	Unambiguous	1	1	N-Term(iTRAQ4plex); K1(iTRAQ4plex)	HCD	1.0000	0.0000	1	1	9309	Unique	Used	1.355	1.150E4	1.710E4	148	4.83	0.00	1	19	10	3	646.34094	1937.00827	-16.65	35.83	3878	3878	MS2	21/52	Glioma_Nuclear_prep_Gradeall_4plex.raw
19913408	High		kVLFTcFk	Unambiguous	2	1	N-Term(iTRAQ4plex); K1(iTRAQ4plex); C6(Methylthio); K8(iTRAQ4plex)	HCD	1.0000	0.0000	1	1	10160	Unique	Used	1.305	6.400E3	9.163E3	284	3.73	0.00	1	55	58	3	488.61307	1463.82465	-15.88	42.04	4804	4804	MS2	19/28	Glioma_Nuclear_prep_Gradeall_4plex.raw
19913408		High	eDVLAGMSGk	Unambiguous	1	1	N-Term(iTRAQ4plex); K10(iTRAQ4plex)	HCD	1.0000	0.0000	1	1	9180	Unique	Used	1.328	8.090E3	1.171E4	278	3.80	0.00	0	51	300	2	647.84564	1294.68401	-5.84	35.74	3573	3573	MS2	16/18	Glioma_Nuclear_prep_Gradeall_repeat_4plex.raw
19913408		High	eVTFVPGLYk	Unambiguous	2	1	N-Term(iTRAQ4plex); K10(iTRAQ4plex)	HCD	1.0000	0.0000	1	1	11015	Unique	Used	1.728	3.123E3	5.880E3	242	3.85	0.00	0	41	300	2	720.92151	1440.83574	1.13	57.34	5548	5548	MS2	14/18	Glioma_Nuclear_prep_Gradeall_repeat_4plex.raw

19913408	High		eDVLAGMSGk	Unambiguous	1	1	N-Term(iTRAQ4plex); K10(iTRAQ4plex)	HCD	1.0000	0.0000	1	1	8765	Unique	Used	1.436	1.155E5	1.819E5	226	3.07	0.00	0	20	12	2	647.83752	1294.66777	-18.38	31.83	3284	3284	MS2	17/18	Glioma_Nuclear_prep_Gradeall_4plex.raw
19913408	High		eVTFVPGLYk	Unambiguous	2	1	N-Term(iTRAQ4plex); K10(iTRAQ4plex)	HCD	1.0000	0.0000	1	1	10173	Unique	Used	2.217	8.444E4	2.055E5	222	3.60	0.00	0	32	17	2	720.90955	1440.81182	-15.47	42.16	4819	4819	MS2	14/18	Glioma_Nuclear_prep_Gradeall_4plex.raw
19913408		High	lLFPAVDDNLLk	Unambiguous	1	1	N-Term(iTRAQ4plex); K12(iTRAQ4plex)	HCD	1.0000	0.0000	1	1	12351	Unique	Used	2.444	3.027E2	8.063E2	170	3.89	0.00	0	30	300	3	549.32916	1645.97293	-2.33	80.67	7435	7435	MS2	19/44	Glioma_Nuclear_prep_Gradeall_repeat_4plex.raw
19913408	High		lLFPAVDDNLLk	Unambiguous	1	1	N-Term(iTRAQ4plex); K12(iTRAQ4plex)	HCD	1.0000	0.0000	1	1	11979	Unique	Used	1.785	1.362E4	2.668E4	192	4.56	0.00	0	56	191	2	823.48901	1645.97075	-3.66	56.77	6789	6789	MS2	16/22	Glioma_Nuclear_prep_Gradeall_4plex.raw
19913408	High		eDLAAFVEELDk	Unambiguous	1	1	N-Term(iTRAQ4plex); K12(iTRAQ4plex)	HCD	1.0000	0.0000	1	1	12273	Unique	Used	1.999	9.396E3	2.061E4	240	4.85	0.00	0	36	199	2	833.94110	1666.87493	-1.76	59.74	7112	7112	MS2	21/22	Glioma_Nuclear_prep_Gradeall_4plex.raw
19913408	High		eDLAAFVEELDkVESQER	Unambiguous	1	1	N-Term(iTRAQ4plex); K12(iTRAQ4plex)	HCD	1.0000	0.0000	1	1	12579	Unique	Used	2.111	1.416E3	3.280E3	127	3.84	0.00	1	16	209	3	799.07642	2395.21469	-3.55	63.38	7456	7456	MS2	31/68	Glioma_Nuclear_prep_Gradeall_4plex.raw
19913408	High		lLFPAVDDNLLk	Unambiguous	1	1	N-Term(iTRAQ4plex); K12(iTRAQ4plex)	HCD	1.0000	0.0000	1	1	11896	Unique	Used	2.220	2.425E3	5.907E3	130	3.80	0.00	0	29	50	3	549.33307	1645.98465	4.78	56.01	6698	6698	MS2	23/44	Glioma_Nuclear_prep_Gradeall_4plex.raw
19913408	High		lLFPAVDDNLLk	Unambiguous	1	1	N-Term(iTRAQ4plex); K12(iTRAQ4plex)	HCD	1.0000	0.0000	1	1	11883	Unique	Used	3.447	2.930E4	1.108E5	171	4.61	0.00	0	33	65	2	823.49188	1645.97649	-0.18	55.90	6684	6684	MS2	18/22	Glioma_Nuclear_prep_Gradeall_4plex.raw
19913408		High	iFDEILVNAADNk	Unambiguous	2	1	N-Term(iTRAQ4plex); K13(iTRAQ4plex)	HCD	1.0000	0.0000	1	1	12279	Unique	Used	1.453	2.580E2	4.087E2	171	3.60	0.00	0	27	300	3	583.99164	1749.96036	-1.27	78.51	7276	7276	MS2	22/48	Glioma_Nuclear_prep_Gradeall_repeat_4plex.raw
19913408	High		iFDEILVNAADNk	Unambiguous	2	1	N-Term(iTRAQ4plex); K13(iTRAQ4plex)	HCD	1.0000	0.0000	1	1	11730	Unique	Used	1.297	5.382E3	7.660E3	191	4.18	0.00	0	46	63	3	583.99036	1749.95652	-3.47	54.60	6518	6518	MS2	21/48	Glioma_Nuclear_prep_Gradeall_4plex.raw
19913408	High		iFDEILVNAADNk	Unambiguous	2	1	N-Term(iTRAQ4plex); K13(iTRAQ4plex)	HCD	1.0000	0.0000	1	1	11754	Unique	Used	1.349	2.844E4	4.209E4	232	5.11	0.00	0	34	78	2	875.48364	1749.96001	-1.47	54.82	6544	6544	MS2	22/24	Glioma_Nuclear_prep_Gradeall_4plex.raw
19913408	High		gFQQISFVNSIATTk	Unambiguous	1	1	N-Term(iTRAQ4plex); K15(iTRAQ4plex)	HCD	1.0000	0.0000	1	1	10856	Unique	Used	1.417	7.826E3	1.217E4	187	3.39	0.00	0	55	44	3	643.68536	1929.04154	-13.94	47.33	5564	5564	MS2	18/56	Glioma_Nuclear_prep_Gradeall_4plex.raw
19913408		High	fDSNEEDSASVFSPSFGLk	Unambiguous	1	1	N-Term(iTRAQ4plex); K19(iTRAQ4plex)	HCD	1.0000	0.0000	1	1	11885	Unique	Used	1.464	1.989E2	3.176E2	71	4.99	0.00	0	24	300	3	784.38263	2351.13333	2.17	69.96	6615	6615	MS2	26/72	Glioma_Nuclear_prep_Gradeall_repeat_4plex.raw
19913408	High		vYVPALIFGQLLTSSNYDDDEk	Unambiguous	1	1	N-Term(iTRAQ4plex); K22(iTRAQ4plex)	HCD	1.0000	0.0000	1	1	13124	Unique	Used	1.445	8.346E1	1.323E2	110	5.31	0.00	0	14	300	3	925.81769	2775.43851	1.92	75.16	8355	8355	MS2	29/84	Glioma_Nuclear_prep_Gradeall_4plex.raw
19913408	High		lDkDIVALMTR	Unambiguous	1	1	N-Term(iTRAQ4plex); K3(iTRAQ4plex)	HCD	1.0000	0.0000	1	1	10884	Unique	Used	1.511	3.555E4	5.893E4	179	3.40	0.00	1	39	35	2	781.95184	1562.89641	-13.74	47.56	5595	5595	MS2	18/20	Glioma_Nuclear_prep_Gradeall_4plex.raw
19913408		High	gIPVVEHk	Unambiguous	2	1	N-Term(iTRAQ4plex); K8(iTRAQ4plex)	HCD	1.0000	0.0000	1	1	7759	Unique	Used	1.582	9.452E3	1.629E4	237	3.67	0.00	0	48	73	3	389.57397	1166.70737	-5.34	21.50	2072	2072	MS2	18/28	Glioma_Nuclear_prep_Gradeall_repeat_4plex.raw
19913408	High		iIPEITAMk	Unambiguous	1	1	N-Term(iTRAQ4plex); K9(iTRAQ4plex)	HCD	1.0000	0.0000	1	1	10307	Unique	Used	1.353	1.691E5	2.512E5	281	3.43	0.00	0	40	13	2	652.38861	1303.76995	-15.24	43.14	4964	4964	MS2	12/16	Glioma_Nuclear_prep_Gradeall_4plex.raw
19923653	NP_150091.2	PSIP1	11168	PC4 and SFRS1-interacting protein isoform 2 [Homo sapiens]	34.34	2	11	31	0.524	14;17	20.1	96.58	530	60.1	9.13																			
19923653		High	qPcPSESDIITEEDk	Unambiguous	2	1	N-Term(iTRAQ4plex); C3(Methylthio); K15(iTRAQ4plex)	HCD	1.0000	0.0000	1	1	10067	Unique	Used	0.509	6.944E3	3.850E3	73	5.42	0.00	0	11	300	2	1012.97614	2024.94499	2.69	45.89	4526	4526	MS2	21/28	Glioma_Nuclear_prep_Gradeall_repeat_4plex.raw
19923653		High	qPcPSESDIITEEDk	Unambiguous	2	1	N-Term(iTRAQ4plex); C3(Methylthio); K15(iTRAQ4plex)	HCD	1.0000	0.0000	1	1	10079	Unique	Used	0.612	2.752E3	1.837E3	84	5.16	0.00	0	13	83	3	675.65344	2024.94577	3.07	46.06	4540	4540	MS2	22/56	Glioma_Nuclear_prep_Gradeall_repeat_4plex.raw
19923653	High		qPcPSESDIITEEDk	Unambiguous	2	1	N-Term(iTRAQ4plex); C3(Methylthio); K15(iTRAQ4plex)	HCD	1.0000	0.0000	1	1	9522	Unique	Used	0.462	3.341E5	1.696E5	41	5.71	0.00	0	3	8	2	1012.95758	2024.90788	-15.64	37.41	4111	4111	MS2	21/28	Glioma_Nuclear_prep_Gradeall_4plex.raw
19923653	High		qPcPSESDIITEEDk	Unambiguous	2	1	N-Term(iTRAQ4plex); C3(Methylthio); K15(iTRAQ4plex)	HCD	1.0000	0.0000	1	1	9531	Unique	Used	0.727	1.424E4	1.136E4	45	3.73	0.00	0	50	18	3	675.64038	2024.90659	-16.28	37.46	4120	4120	MS2	18/56	Glioma_Nuclear_prep_Gradeall_4plex.raw
19923653		High	kGFNEGLWEIDNNPk	Unambiguous	2	1	N-Term(iTRAQ4plex); K1(iTRAQ4plex); K15(iTRAQ4plex)	HCD	1.0000	0.0000	1	1	10427	Unique	Used	0.657	1.020E3	7.304E2	182	6.24	0.00	1	41	300	3	731.72705	2193.16660	0.05	50.15	4910	4910	MS2	23/56	Glioma_Nuclear_prep_Gradeall_repeat_4plex.raw
19923653	High		kGFNEGLWEIDNNPk	Unambiguous	2	1	N-Term(iTRAQ4plex); K1(iTRAQ4plex); K15(iTRAQ4plex)	HCD	1.0000	0.0000	1	1	9600	Unique	Used	0.600	3.151E4	2.075E4	185	5.98	0.00	1	40	10	3	731.71692	2193.13620	-13.81	37.96	4195	4195	MS2	23/56	Glioma_Nuclear_prep_Gradeall_4plex.raw
19923653		High	vDEVPDGAVkPPTNk	Unambiguous	2	1	N-Term(iTRAQ4plex); K10(iTRAQ4plex); K15(iTRAQ4plex)	HCD	1.0000	0.0000	1	1	8485	Unique	Used	0.320	3.014E4	1.051E4	103	3.71	0.00	0	9	16	3	666.71460	1998.12925	3.01	28.47	2837	2837	MS2	22/56	Glioma_Nuclear_prep_Gradeall_repeat_4plex.raw
19923653		High	vDEVPDGAVkPPTNk	Unambiguous	2	1	N-Term(iTRAQ4plex); K10(iTRAQ4plex); K15(iTRAQ4plex)	HCD	1.0000	0.0000	1	1	8500	Unique	Used	0.493	1.295E3	6.958E2	127	3.58	0.00	0	51	132	4	500.28598	1998.12209	-0.58	28.62	2853	2853	MS2	17/84	Glioma_Nuclear_prep_Gradeall_repeat_4plex.raw
19923653		High	vDEVPDGAVkPPTNk	Unambiguous	2	1	N-Term(iTRAQ4plex); K10(iTRAQ4plex); K15(iTRAQ4plex)	HCD	1.0000	0.0000	1	1	8522	Unique	Used	0.670	9.947E2	7.267E2	85	2.98	0.00	0	0	300	2	999.56824	1998.12920	2.98	28.85	2876	2876	MS2	15/28	Glioma_Nuclear_prep_Gradeall_repeat_4plex.raw
19923653	High		vDEVPDGAVkPPTNk	Unambiguous	2	1	N-Term(iTRAQ4plex); K10(iTRAQ4plex); K15(iTRAQ4plex)	HCD	1.0000	0.0000	1	1	8171	Unique	Used	0.403	2.172E5	9.595E4	127	4.72	0.00	0	13	7	2	999.54926	1998.09123	-16.02	27.57	2638	2638	MS2	20/28	Glioma_Nuclear_prep_Gradeall_4plex.raw
19923653	High		vDEVPDGAVkPPTNk	Unambiguous	2	1	N-Term(iTRAQ4plex); K10(iTRAQ4plex); K15(iTRAQ4plex)	HCD	1.0000	0.0000	1	1	8215	Unique	Used	0.411	1.335E5	6.015E4	138	3.77	0.00	0	11	2	3	666.70203	1998.09153	-15.87	27.88	2686	2686	MS2	22/56	Glioma_Nuclear_prep_Gradeall_4plex.raw
19923653		High	qSNASSDVEVEEk	Unambiguous	2	1	N-Term(iTRAQ4plex); K13(iTRAQ4plex)	HCD	1.0000	0.0000	1	1	7421	Unique	Used	0.349	2.451E4	9.325E3	105	5.63	0.00	0	11	174	2	855.41418	1709.82109	-12.96	18.40	1712	1712	MS2	24/24	Glioma_Nuclear_prep_Gradeall_repeat_4plex.raw
19923653	High		qSNASSDVEVEEk	Unambiguous	2	1	N-Term(iTRAQ4plex); K13(iTRAQ4plex)	HCD	1.0000	0.0000	1	1	7450	Unique	Used	0.308	3.407E5	1.150E5	143	5.84	0.00	0	6	10	2	855.42322	1709.83916	-2.39	22.46	1857	1857	MS2	24/24	Glioma_Nuclear_prep_Gradeall_4plex.raw
19923653		High	gFNEGLWEIDNNPk	Unambiguous	2	1	N-Term(iTRAQ4plex); K14(iTRAQ4plex)	HCD	1.0000	0.0000	1	1	11247	Unique	Used	0.623	2.317E3	1.574E3	162	5.84	0.00	0	13	300	2	960.98914	1920.97099	0.80	60.40	5815	5815	MS2	24/26	Glioma_Nuclear_prep_Gradeall_repeat_4plex.raw
19923653	High		gFNEGLWEIDNNPk	Unambiguous	2	1	N-Term(iTRAQ4plex); K14(iTRAQ4plex)	HCD	1.0000	0.0000	1	1	10397	Unique	Used	0.639	6.251E4	4.381E4	130	6.17	0.00	0	0	33	2	960.97516	1920.94304	-13.76	43.82	5062	5062	MS2	24/26	Glioma_Nuclear_prep_Gradeall_4plex.raw
19923653	High		lPIFFFGTHETAFLGPk	Unambiguous	2	1	N-Term(iTRAQ4plex); K17(iTRAQ4plex)	HCD	1.0000	0.0000	1	1	12794	Unique	Used	0.545	2.720E3	1.628E3	101	6.70	0.00	0	17	300	3	737.41638	2210.23459	4.21	66.28	7707	7707	MS2	27/64	Glioma_Nuclear_prep_Gradeall_4plex.raw
19923653	High		lPIFFFGTHETAFLGPk	Unambiguous	2	1	N-Term(iTRAQ4plex); K17(iTRAQ4plex)	HCD	1.0000	0.0000	1	1	12744	Unique	Used	0.622	1.987E3	1.356E3	109	5.89	0.00	0	45	300	3	737.41449	2210.22892	1.64	65.55	7646	7646	MS2	27/64	Glioma_Nuclear_prep_Gradeall_4plex.raw
19923653		High	dFkPGDLIFAk	Unambiguous	2	1	N-Term(iTRAQ4plex); K3(iTRAQ4plex); K11(iTRAQ4plex)	HCD	1.0000	0.0000	1	1	11010	Unique	Used	0.524	3.784E3	2.159E3	120	4.27	0.00	0	27	266	3	561.66833	1682.99045	3.71	57.28	5543	5543	MS2	23/40	Glioma_Nuclear_prep_Gradeall_repeat_4plex.raw
19923653		High	dFkPGDLIFAk	Unambiguous	2	1	N-Term(iTRAQ4plex); K3(iTRAQ4plex); K11(iTRAQ4plex)	HCD	1.0000	0.0000	1	1	11014	Unique	Used	0.531	5.977E3	3.461E3	143	3.67	0.00	0	58	300	2	841.99731	1682.98735	1.87	57.33	5547	5547	MS2	18/20	Glioma_Nuclear_prep_Gradeall_repeat_4plex.raw
19923653		High	dFkPGDLIFAk	Unambiguous	2	1	N-Term(iTRAQ4plex); K3(iTRAQ4plex); K11(iTRAQ4plex)	HCD	1.0000	0.0000	1	1	11098	Unique	Used	0.545	2.432E3	1.445E3	117	3.20	0.00	0	84	300	2	842.00507	1683.00286	11.08	58.42	5642	5642	MS2	17/20	Glioma_Nuclear_prep_Gradeall_repeat_4plex.raw
19923653	High		dFkPGDLIFAk	Unambiguous	2	1	N-Term(iTRAQ4plex); K3(iTRAQ4plex); K11(iTRAQ4plex)	HCD	1.0000	0.0000	1	1	10051	Unique	Used	0.516	1.509E5	8.541E4	159	4.07	0.00	0	25	3	3	561.65826	1682.96024	-14.24	41.23	4685	4685	MS2	23/40	Glioma_Nuclear_prep_Gradeall_4plex.raw
19923653	High		dFkPGDLIFAk	Unambiguous	2	1	N-Term(iTRAQ4plex); K3(iTRAQ4plex); K11(iTRAQ4plex)	HCD	1.0000	0.0000	1	1	10055	Unique	Used	0.518	2.617E5	1.488E5	190	3.72	0.00	0	37	13	2	841.98444	1682.96160	-13.44	41.25	4689	4689	MS2	17/20	Glioma_Nuclear_prep_Gradeall_4plex.raw
19923653		High	aSNEDVTk	Unambiguous	2	1	N-Term(iTRAQ4plex); K8(iTRAQ4plex)	HCD	1.0000	0.0000	1	1	6685	Unique	Used	0.509	2.345E4	1.301E4	239	3.01	0.00	0	49	143	2	576.30902	1151.61077	-3.39	11.97	936	936	MS2	14/14	Glioma_Nuclear_prep_Gradeall_repeat_4plex.raw
19923653		High	aVDITTPk	Unambiguous	2	1	N-Term(iTRAQ4plex); K8(iTRAQ4plex)	HCD	1.0000	0.0000	1	1	8022	Unique	Used	0.607	5.094E4	3.371E4	268	3.27	0.00	0	38	120	2	566.84460	1132.68193	0.27	23.99	2349	2349	MS2	13/14	Glioma_Nuclear_prep_Gradeall_repeat_4plex.raw
19923653	High		aSNEDVTk	Unambiguous	2	1	N-Term(iTRAQ4plex); K8(iTRAQ4plex)	HCD	1.0000	0.0000	1	1	6991	Unique	Used	0.517	6.414E3	3.638E3	226	3.18	0.00	0	82	300	2	576.31494	1151.62261	6.89	18.42	1354	1354	MS2	14/14	Glioma_Nuclear_prep_Gradeall_4plex.raw
19923653		High	fSSQQAATk	Unambiguous	2	1	N-Term(iTRAQ4plex); K9(iTRAQ4plex)	HCD	1.0000	0.0000	1	1	7156	Unique	Used	0.384	2.347E5	9.831E4	166	3.89	0.00	0	8	20	2	628.34619	1255.68511	-2.70	16.21	1434	1434	MS2	16/16	Glioma_Nuclear_prep_Gradeall_repeat_4plex.raw
19923653		High	fSSQQAATk	Unambiguous	2	1	N-Term(iTRAQ4plex); K9(iTRAQ4plex)	HCD	1.0000	0.0000	1	1	7126	Unique	Used	0.506	4.529E4	2.497E4	201	3.83	0.00	0	64	214	2	628.33734	1255.66741	-16.80	15.91	1402	1402	MS2	16/16	Glioma_Nuclear_prep_Gradeall_repeat_4plex.raw
19923653		High	dIFPYSENk	Unambiguous	2	1	N-Term(iTRAQ4plex); K9(iTRAQ4plex)	HCD	1.0000	0.0000	1	1	9619	Unique	Used	0.555	2.990E4	1.808E4	197	2.90	0.00	0	17	138	2	700.86725	1400.72722	-2.00	40.62	4044	4044	MS2	14/16	Glioma_Nuclear_prep_Gradeall_repeat_4plex.raw
19923653		High	dIFPYSENk	Unambiguous	2	1	N-Term(iTRAQ4plex); K9(iTRAQ4plex)	HCD	1.0000	0.0000	1	1	9529	Unique	Used	0.621	3.274E4	2.217E4	164	3.13	0.00	0	37	138	2	700.86395	1400.72063	-6.71	39.60	3946	3946	MS2	13/16	Glioma_Nuclear_prep_Gradeall_repeat_4plex.raw
19923653	High		dIFPYSENk	Unambiguous	2	1	N-Term(iTRAQ4plex); K9(iTRAQ4plex)	HCD	1.0000	0.0000	1	1	9091	Unique	Used	0.430	1.061E6	5.013E5	127	3.13	0.00	0	3	2	2	700.85840	1400.70952	-14.64	34.23	3640	3640	MS2	14/16	Glioma_Nuclear_prep_Gradeall_4plex.raw
19923653	High		dIFPYSENk	Unambiguous	2	1	N-Term(iTRAQ4plex); K9(iTRAQ4plex)	HCD	1.0000	0.0000	1	1	9008	Unique	Used	0.639	1.893E5	1.327E5	133	3.32	0.00	0	52	18	2	700.85547	1400.70366	-18.82	33.57	3547	3547	MS2	13/16	Glioma_Nuclear_prep_Gradeall_4plex.raw
21361809	NP_062556.2	RBMXL1	494115	RNA binding motif protein, X-linked-like-1 [Homo sapiens]	22.05	2	3	6	0.595	4;2	22.8	64.29	390	42.1	9.89																			
21361809	High		vEQATkPSFER	Unambiguous	1	1	N-Term(iTRAQ4plex); K6(iTRAQ4plex)	HCD	1.0000	0.0000	1	1	7721	Unique	Used	0.507	1.161E5	6.465E4	255	3.76	0.00	0	5	16	2	790.43921	1579.87114	1.81	24.38	2150	2150	MS2	16/20	Glioma_Nuclear_prep_Gradeall_4plex.raw
21361809		High	iVEVLLIk	Unambiguous	1	1	N-Term(iTRAQ4plex); K8(iTRAQ4plex)	HCD	1.0000	0.0000	1	1	11880	Unique	Used	0.463	6.957E3	3.514E3	23	3.34	0.00	0	51	300	2	607.92145	1214.83562	2.44	69.89	6609	6609	MS2	14/14	Glioma_Nuclear_prep_Gradeall_repeat_4plex.raw
21361809		High	aLETVFGk	Unambiguous	1	1	N-Term(iTRAQ4plex); K8(iTRAQ4plex)	HCD	1.0000	0.0000	1	1	10036	Unique	Used	0.729	1.388E4	1.103E4	290	3.27	0.00	0	49	300	2	576.84735	1152.68743	0.61	45.48	4491	4491	MS2	13/14	Glioma_Nuclear_prep_Gradeall_repeat_4plex.raw
21361809	High		iVEVLLIk	Unambiguous	1	1	N-Term(iTRAQ4plex); K8(iTRAQ4plex)	HCD	1.0000	0.0000	1	1	11004	Unique	Used	0.439	4.983E5	2.402E5	22	3.51	0.00	0	27	8	2	607.92010	1214.83293	0.23	48.48	5725	5725	MS2	14/14	Glioma_Nuclear_prep_Gradeall_4plex.raw
21361809	High		aLETVFGk	Unambiguous	1	1	N-Term(iTRAQ4plex); K8(iTRAQ4plex)	HCD	1.0000	0.0000	1	1	9444	Unique	Used	0.683	3.841E5	2.879E5	336	3.13	0.00	0	48	6	2	576.83807	1152.66887	-15.48	36.84	4026	4026	MS2	13/14	Glioma_Nuclear_prep_Gradeall_4plex.raw
21361809	High		iVEVLLIk	Unambiguous	1	1	N-Term(iTRAQ4plex); K8(iTRAQ4plex)	HCD	1.0000	0.0000	1	1	10978	Unique	Used	0.720	3.305E5	2.612E5	43	3.34	0.00	0	54	13	2	607.91217	1214.81706	-12.84	48.29	5697	5697	MS2	13/14	Glioma_Nuclear_prep_Gradeall_4plex.raw
21536286	NP_001814.2	CKB	1152	creatine kinase B-type [Homo sapiens]	9.71	1	3	4	1.867	2;2	26.3	9.63	381	42.6	5.59																			
21536286		High	vLTPELYAELR	Unambiguous	1	1	N-Term(iTRAQ4plex)	HCD	1.0000	0.0000	1	1	11665	Unique	Used	1.498	5.587E3	9.119E3	296	3.86	0.00	0	17	300	2	724.41827	1447.82927	1.05	66.51	6329	6329	MS2	16/20	Glioma_Nuclear_prep_Gradeall_repeat_4plex.raw
21536286		High	dLFDPIIEDR	Unambiguous	1	1	N-Term(iTRAQ4plex)	HCD	1.0000	0.0000	1	1	11553	Unique	Used	1.685	2.631E3	4.830E3	231	3.45	0.00	0	48	300	2	688.86298	1376.71868	0.58	64.87	6191	6191	MS2	17/18	Glioma_Nuclear_prep_Gradeall_repeat_4plex.raw
21536286	High		lAVEALSSLDGDLAGR	Unambiguous	1	1	N-Term(iTRAQ4plex)	HCD	1.0000	0.0000	1	1	12038	Unique	Used	2.049	8.479E2	1.906E3	249	4.04	0.00	0	26	77	3	577.65063	1730.93735	-1.87	57.36	6854	6854	MS2	23/60	Glioma_Nuclear_prep_Gradeall_4plex.raw
21536286	High		lAVEALSSLDGDLAGR	Unambiguous	1	1	N-Term(iTRAQ4plex)	HCD	1.0000	0.0000	1	1	12047	Unique	Used	2.677	2.841E4	8.345E4	272	5.59	0.00	0	29	85	2	865.97430	1730.94133	0.43	57.46	6864	6864	MS2	23/30	Glioma_Nuclear_prep_Gradeall_4plex.raw
24431933	NP_722550.1	RTN4	57142	reticulon-4 isoform B [Homo sapiens]	3.49	3	1	2	2.322	1;1	12.2	4.28	373	40.3	4.78																			
24431933		High	gPLPAAPPVAPER	Unambiguous	3	1	N-Term(iTRAQ4plex)	HCD	1.0000	0.0000	1	1	9132	Unique	Used	2.121	5.291E3	1.223E4	240	4.28	0.00	0	13	250	2	708.40796	1415.80864	-2.93	35.23	3523	3523	MS2	18/24	Glioma_Nuclear_prep_Gradeall_repeat_4plex.raw
24431933	High		gPLPAAPPVAPER	Unambiguous	3	1	N-Term(iTRAQ4plex)	HCD	1.0000	0.0000	1	1	8708	Unique	Used	2.523	9.415E4	2.607E5	296	3.84	0.00	0	8	13	2	708.39825	1415.78923	-16.64	31.43	3222	3222	MS2	15/24	Glioma_Nuclear_prep_Gradeall_4plex.raw
25952118	NP_741960.1	CAMK2A	815	calcium/calmodulin-dependent protein kinase type II subunit alpha isoform 2 [Homo sapiens]	2.51	2	1	2	0.459	1;1	26.5	3.85	478	54.1	7.08																			
25952118		High	iTQYLDAGGIPR	Unambiguous	2	1	N-Term(iTRAQ4plex)	HCD	1.0000	0.0000	1	1	11026	Unique	Used	0.373	9.949E3	4.041E3	321	3.85	0.00	0	42	300	2	724.40668	1447.80608	2.41	57.49	5561	5561	MS2	16/22	Glioma_Nuclear_prep_Gradeall_repeat_4plex.raw
25952118	High		iTQYLDAGGIPR	Unambiguous	2	1	N-Term(iTRAQ4plex)	HCD	1.0000	0.0000	1	1	10237	Unique	Used	0.545	1.314E5	7.854E4	304	3.66	0.00	0	41	23	2	724.39508	1447.78288	-13.61	42.62	4888	4888	MS2	18/22	Glioma_Nuclear_prep_Gradeall_4plex.raw
29568103	NP_003080.2	SNRNP70	6625	U1 small nuclear ribonucleoprotein 70 kDa isoform 1 [Homo sapiens]	13.73	2	7	11	0.631	5;6	12.2	29.88	437	51.5	9.94																			
29568103		High	gYAFIEYEHER	Unambiguous	2	1	N-Term(iTRAQ4plex)	HCD	1.0000	0.0000	1	1	9528	Unique	Used	0.631	4.169E3	2.869E3	86	4.61	0.00	0	26	91	3	519.91632	1557.73441	-7.09	39.59	3945	3945	MS2	22/40	Glioma_Nuclear_prep_Gradeall_repeat_4plex.raw
29568103		High	lGGGLGGTR	Unambiguous	2	1	N-Term(iTRAQ4plex)	HCD	1.0000	0.0000	1	1	7691	Unique	Used	0.647	8.028E4	5.664E4	248	3.13	0.00	0	18	70	2	466.27176	931.53624	-8.54	20.86	2001	2001	MS2	13/16	Glioma_Nuclear_prep_Gradeall_repeat_4plex.raw
29568103	High		gYAFIEYEHER	Unambiguous	2	1	N-Term(iTRAQ4plex)	HCD	1.0000	0.0000	1	1	8886	Unique	Used	0.679	1.154E5	8.600E4	93	3.68	0.00	0	0	15	2	779.36499	1557.72270	-14.60	32.71	3415	3415	MS2	19/20	Glioma_Nuclear_prep_Gradeall_4plex.raw
29568103		High	rEFEVYGPIk	Unambiguous	1	1	N-Term(iTRAQ4plex); K10(iTRAQ4plex)	HCD	1.0000	0.0000	1	1	9351	Unique	Used	0.732	3.650E3	2.912E3	225	3.51	0.00	1	64	231	3	509.29288	1525.86408	1.54	37.63	3753	3753	MS2	16/36	Glioma_Nuclear_prep_Gradeall_repeat_4plex.raw
29568103	High		rQQEVETELk	Unambiguous	2	1	N-Term(iTRAQ4plex); K10(iTRAQ4plex)	HCD	1.0000	0.0000	1	1	7462	Unique	Used	0.452	1.033E5	5.125E4	258	3.25	0.00	1	10	12	2	774.43152	1547.85576	-4.80	22.55	1870	1870	MS2	16/18	Glioma_Nuclear_prep_Gradeall_4plex.raw
29568103		High	dPIPYLPPLEk	Unambiguous	2	1	N-Term(iTRAQ4plex); K11(iTRAQ4plex)	HCD	1.0000	0.0000	1	1	11721	Unique	Used	0.588	8.963E3	5.740E3	172	3.54	0.00	0	10	300	2	785.46234	1569.91741	2.74	67.25	6393	6393	MS2	16/20	Glioma_Nuclear_prep_Gradeall_repeat_4plex.raw
29568103	High		dPIPYLPPLEk	Unambiguous	2	1	N-Term(iTRAQ4plex); K11(iTRAQ4plex)	HCD	1.0000	0.0000	1	1	10834	Unique	Used	0.607	5.415E5	3.606E5	214	3.51	0.00	0	17	5	2	785.45032	1569.89336	-12.57	47.18	5541	5541	MS2	16/20	Glioma_Nuclear_prep_Gradeall_4plex.raw
29568103		High	qQEVETELk	Unambiguous	2	1	N-Term(iTRAQ4plex); K9(iTRAQ4plex)	HCD	1.0000	0.0000	1	1	8002	Unique	Used	0.662	2.519E4	1.816E4	281	4.07	0.00	0	22	118	2	696.38556	1391.76384	1.27	23.81	2328	2328	MS2	16/16	Glioma_Nuclear_prep_Gradeall_repeat_4plex.raw
29568103		High	eFEVYGPIk	Unambiguous	2	1	N-Term(iTRAQ4plex); K9(iTRAQ4plex)	HCD	1.0000	0.0000	1	1	10252	Unique	Used	0.704	1.472E4	1.128E4	252	4.18	0.00	0	19	300	2	685.38513	1369.76299	1.74	48.06	4723	4723	MS2	15/16	Glioma_Nuclear_prep_Gradeall_repeat_4plex.raw
29568103	High		qQEVETELk	Unambiguous	2	1	N-Term(iTRAQ4plex); K9(iTRAQ4plex)	HCD	1.0000	0.0000	1	1	7800	Unique	Used	0.559	3.472E5	2.129E5	247	4.13	0.00	0	13	11	2	696.38458	1391.76189	-0.13	24.94	2236	2236	MS2	16/16	Glioma_Nuclear_prep_Gradeall_4plex.raw
29568103	High		eFEVYGPIk	Unambiguous	2	1	N-Term(iTRAQ4plex); K9(iTRAQ4plex)	HCD	1.0000	0.0000	1	1	9581	Unique	Used	0.618	4.351E5	2.948E5	231	3.53	0.00	0	30	7	2	685.37366	1369.74004	-15.02	37.84	4175	4175	MS2	14/16	Glioma_Nuclear_prep_Gradeall_4plex.raw
31542947	NP_002147.2	HSPD1	3329	60 kDa heat shock protein, mitochondrial [Homo sapiens]	8.20	1	3	4	1.850	3;1	15.8	15.79	573	61.0	5.87																			
31542947	High		tLNDELEIIEGMk	Unambiguous	1	1	N-Term(iTRAQ4plex); K13(iTRAQ4plex)	HCD	1.0000	0.0000	1	1	12147	Unique	Used	1.843	1.211E4	2.450E4	223	5.66	0.00	0	34	145	2	896.98364	1792.96001	-0.30	58.45	6973	6973	MS2	22/24	Glioma_Nuclear_prep_Gradeall_4plex.raw
31542947	High		iQEIIEQLDVTTSEYEk	Unambiguous	1	1	N-Term(iTRAQ4plex); K17(iTRAQ4plex)	HCD	1.0000	0.0000	1	1	12820	Unique	Used	1.326	3.755E2	5.465E2	175	7.21	0.00	0	14	300	3	776.08209	2326.23172	2.09	66.62	7737	7737	MS2	28/64	Glioma_Nuclear_prep_Gradeall_4plex.raw
31542947		High	lSDGVAVLk	Unambiguous	1	1	N-Term(iTRAQ4plex); K9(iTRAQ4plex)	HCD	1.0000	0.0000	1	1	9569	Unique	Used	1.857	7.937E3	1.606E4	271	3.41	0.00	0	17	273	2	595.36792	1189.72856	-9.18	40.06	3990	3990	MS2	16/16	Glioma_Nuclear_prep_Gradeall_repeat_4plex.raw
31542947	High		lSDGVAVLk	Unambiguous	1	1	N-Term(iTRAQ4plex); K9(iTRAQ4plex)	HCD	1.0000	0.0000	1	1	9027	Unique	Used	1.917	7.304E4	1.537E5	333	2.92	0.00	0	40	21	2	595.36261	1189.71794	-18.11	33.71	3568	3568	MS2	16/16	Glioma_Nuclear_prep_Gradeall_4plex.raw
31742498	NP_056189.1	SUN2	25777	SUN domain-containing protein 2 isoform b [Homo sapiens]	14.37	2	3	6	1.568	3;3	6.6	33.52	717	80.3	6.73																			
31742498		High	iGLADYALESGGASVISTR	Unambiguous	2	1	N-Term(iTRAQ4plex)	HCD	1.0000	0.0000	1	1	11907	Unique	Used	1.597	1.524E2	2.653E2	157	4.98	0.00	0	23	300	3	675.36572	2024.08261	2.21	70.31	6644	6644	MS2	24/72	Glioma_Nuclear_prep_Gradeall_repeat_4plex.raw
31742498		High	iGLADYALESGGASVISTR	Unambiguous	2	1	N-Term(iTRAQ4plex)	HCD	1.0000	0.0000	1	1	11915	Unique	Used	1.753	5.796E2	1.107E3	162	5.24	0.00	0	13	300	2	1012.54291	2024.07854	0.20	70.45	6655	6655	MS2	24/36	Glioma_Nuclear_prep_Gradeall_repeat_4plex.raw
31742498	High		iGLADYALESGGASVISTR	Unambiguous	2	1	N-Term(iTRAQ4plex)	HCD	1.0000	0.0000	1	1	11194	Unique	Used	1.538	3.837E4	6.477E4	164	6.84	0.00	0	0	24	2	1012.53455	2024.06182	-8.06	49.98	5933	5933	MS2	26/36	Glioma_Nuclear_prep_Gradeall_4plex.raw
31742498	High		iGLADYALESGGASVISTR	Unambiguous	2	1	N-Term(iTRAQ4plex)	HCD	1.0000	0.0000	1	1	11176	Unique	Used	1.540	3.132E4	5.294E4	160	6.48	0.00	0	9	41	2	1012.54407	2024.08086	1.35	49.84	5913	5913	MS2	26/36	Glioma_Nuclear_prep_Gradeall_4plex.raw
31742498		High	aEHQQDSEDLFk	Unambiguous	2	1	N-Term(iTRAQ4plex); K12(iTRAQ4plex)	HCD	1.0000	0.0000	1	1	8080	Unique	Used	1.441	3.730E3	5.859E3	112	6.01	0.00	0	31	84	3	578.95532	1734.85141	-1.35	24.55	2410	2410	MS2	22/44	Glioma_Nuclear_prep_Gradeall_repeat_4plex.raw
31742498	High		lEDQLAGLQQELAALALk	Unambiguous	2	1	N-Term(iTRAQ4plex); K18(iTRAQ4plex)	HCD	1.0000	0.0000	1	1	13114	Unique	Used	1.622	8.454E1	1.505E2	82	5.21	0.00	0	9	300	3	738.09894	2212.28226	1.38	74.05	8282	8282	MS2	28/68	Glioma_Nuclear_prep_Gradeall_4plex.raw
32189394	NP_001677.2	ATP5B	506	ATP synthase subunit beta, mitochondrial precursor [Homo sapiens]	16.07	1	5	6	1.503	4;2	24.8	27.75	529	56.5	5.40																			
32189394		High	iMNVIGEPIDER	Unambiguous	1	1	N-Term(iTRAQ4plex)	HCD	1.0000	0.0000	1	1	10974	Unique	Used	1.359	4.377E3	6.483E3	296	3.94	0.00	0	54	300	2	765.41028	1529.81328	1.18	56.83	5503	5503	MS2	16/22	Glioma_Nuclear_prep_Gradeall_repeat_4plex.raw
32189394		High	aIAELGIYPAVDPLDSTSR	Unambiguous	1	1	N-Term(iTRAQ4plex)	HCD	1.0000	0.0000	1	1	12223	Unique	Used	2.261	4.447E2	1.096E3	113	4.86	0.00	0	0	300	3	1066.57019	2132.13310	-1.20	76.67	7142	7142	MS2	20/36	Glioma_Nuclear_prep_Gradeall_repeat_4plex.raw
32189394	High		lVLEVAQHLGESTVR	Unambiguous	1	1	N-Term(iTRAQ4plex)	HCD	1.0000	0.0000	1	1	11956	Unique	Used	1.354	1.562E4	2.320E4	164	4.32	0.00	0	34	24	3	599.00189	1794.99112	-15.80	48.11	5873	5873	MS2	27/56	Glioma_Nuclear_prep_Gradeall_4plex.raw
32189394	High		iMDPNIVGSEHYDVAR	Unambiguous	1	1	N-Term(iTRAQ4plex)	HCD	1.0000	0.0000	1	1	9509	Unique	Used	1.420	3.512E4	5.472E4	89	5.39	0.00	0	43	6	3	653.98517	1959.94095	-15.61	37.32	4097	4097	MS2	30/60	Glioma_Nuclear_prep_Gradeall_4plex.raw
32189394	High		aIAELGIYPAVDPLDSTSR	Unambiguous	1	1	N-Term(iTRAQ4plex)	HCD	1.0000	0.0000	1	1	11664	Unique	Used	1.587	1.280E4	2.358E4	134	5.65	0.00	0	7	101	2	1066.56995	2132.13282	-1.43	53.99	6446	6446	MS2	21/36	Glioma_Nuclear_prep_Gradeall_4plex.raw
32189394	High		fTQAGSEVSALLGR	Unambiguous	1	1	N-Term(iTRAQ4plex)	HCD	1.0000	0.0000	1	1	11077	Unique	Used	2.190	6.003E4	8.659E4	282	4.81	0.00	0	37	52	2	790.42651	1579.84575	-6.55	49.06	5805	5805	MS2	23/26	Glioma_Nuclear_prep_Gradeall_4plex.raw
32455256	NP_859530.1	CERS2	29956	ceramide synthase 2 [Homo sapiens]	6.58	1	2	3	0.593	1;2	14.0	9.07	380	44.8	8.98																			
32455256		High	qVEVELLSR	Unambiguous	1	1	N-Term(iTRAQ4plex)	HCD	1.0000	0.0000	1	1	9899	Unique	Used	0.700	8.764E3	6.690E3	261	3.43	0.00	0	54	300	2	608.85577	1216.70427	2.02	43.76	4339	4339	MS2	15/16	Glioma_Nuclear_prep_Gradeall_repeat_4plex.raw
32455256		High	aPPNATLEHFYLTSGk	Unambiguous	1	1	N-Term(iTRAQ4plex); K16(iTRAQ4plex)	HCD	1.0000	0.0000	1	1	10571	Unique	Used	0.593	2.187E3	1.414E3	128	5.64	0.00	0	38	300	3	678.70294	2034.09427	2.15	51.88	5065	5065	MS2	21/60	Glioma_Nuclear_prep_Gradeall_repeat_4plex.raw
32455256	High		aPPNATLEHFYLTSGk	Unambiguous	1	1	N-Term(iTRAQ4plex); K16(iTRAQ4plex)	HCD	1.0000	0.0000	1	1	9679	Unique	Used	0.532	5.934E4	3.465E4	146	5.33	0.00	0	20	7	3	678.69202	2034.06150	-13.96	38.53	4281	4281	MS2	22/60	Glioma_Nuclear_prep_Gradeall_4plex.raw
32455260	NP_857634.1	PRDX5	25824	peroxiredoxin-5, mitochondrial isoform b precursor [Homo sapiens]	4.71	2	1	3	1.997	2;1	17.0	7.15	170	17.4	8.76																			
32455260		High	vNLAELFk	Unambiguous	2	1	N-Term(iTRAQ4plex); K8(iTRAQ4plex)	HCD	1.0000	0.0000	1	1	12069	Unique	Used	2.630	2.045E3	5.862E3	260	3.24	0.00	0	61	300	2	611.37433	1221.74138	-2.61	73.58	6899	6899	MS2	14/14	Glioma_Nuclear_prep_Gradeall_repeat_4plex.raw
32455260	High		vNLAELFk	Unambiguous	2	1	N-Term(iTRAQ4plex); K8(iTRAQ4plex)	HCD	1.0000	0.0000	1	1	11257	Unique	Used	1.969	1.697E4	3.666E4	171	3.60	0.00	0	[illegible]	134	2	611.38361	1221.75994	12.58	50.49	6001	6001	MS2	14/14	Glioma_Nuclear_prep_Gradeall_4plex.raw
32455260	High		vNLAELFk	Unambiguous	2	1	N-Term(iTRAQ4plex); K8(iTRAQ4plex)	HCD	1.0000	0.0000	1	1	11264	Unique	Used	1.997	1.653E4	3.623E4	278	3.55	0.00	0	64	106	2	611.37079	1221.73430	-8.40	50.55	6009	6009	MS2	14/14	Glioma_Nuclear_prep_Gradeall_4plex.raw
33946291	NP_079106.3	LPCAT1	79888	lysophosphatidylcholine acyltransferase 1 [Homo sapiens]	3.75	1	2	3	1.538	2;1	7.3	7.58	534	59.1	6.02																			
33946291		High	lTFADFHR	Unambiguous	1	1	N-Term(iTRAQ4plex)	HCD	1.0000	0.0000	1	1	9962	Unique	Used	1.386	8.278E2	1.250E3	280	3.40	0.00	0	49	300	3	384.20914	1150.61286	0.23	44.58	4410	4410	MS2	16/28	Glioma_Nuclear_prep_Gradeall_repeat_4plex.raw
33946291	High		lPADTcLLEFAR	Unambiguous	1	1	N-Term(iTRAQ4plex); C6(Methylthio)	HCD	1.0000	0.0000	1	1	12526	Unique	Used	1.538	3.616E3	6.101E3	255	3.51	0.00	0	38	300	2	769.89539	1538.78349	0.45	62.69	7395	7395	MS2	16/22	Glioma_Nuclear_prep_Gradeall_4plex.raw
33946291	High		lPADTcLLEFAR	Unambiguous	1	1	N-Term(iTRAQ4plex); C6(Methylthio)	HCD	1.0000	0.0000	1	1	12474	Unique	Used	1.601	6.537E3	1.148E4	253	4.07	0.00	0	37	300	2	769.89532	1538.78337	0.37	61.99	7334	7334	MS2	15/22	Glioma_Nuclear_prep_Gradeall_4plex.raw
33946297	NP_892021.1	ZNF326	284695	DBIRD complex subunit ZNF326 isoform 1 [Homo sapiens]	4.47	1	2	3	0.612	1;2	30.9	11.92	582	65.6	5.15																			
33946297		High	fGPYESYDSR	Unambiguous	1	1	N-Term(iTRAQ4plex)	HCD	1.0000	0.0000	1	1	9179	Unique	Used	0.512	1.672E4	9.326E3	67	3.08	0.00	0	8	300	2	682.81464	1364.62200	-1.42	35.73	3572	3572	MS2	12/18	Glioma_Nuclear_prep_Gradeall_repeat_4plex.raw
33946297		High	eSVLTATSILNNPIVk	Unambiguous	1	1	N-Term(iTRAQ4plex); K16(iTRAQ4plex)	HCD	1.0000	0.0000	1	1	12038	Unique	Used	0.734	2.860E2	2.290E2	94	5.01	0.00	0	32	300	3	663.06213	1987.17185	2.02	72.71	6835	6835	MS2	24/60	Glioma_Nuclear_prep_Gradeall_repeat_4plex.raw
33946297	High		eSVLTATSILNNPIVk	Unambiguous	1	1	N-Term(iTRAQ4plex); K16(iTRAQ4plex)	HCD	1.0000	0.0000	1	1	11311	Unique	Used	0.401	2.289E4	1.008E4	99	7.53	0.00	0	13	108	2	994.04872	1987.16216	-2.85	50.95	6060	6060	MS2	24/30	Glioma_Nuclear_prep_Gradeall_4plex.raw
34101286	NP_057191.2	ZFR	51663	zinc finger RNA-binding protein [Homo sapiens]	6.24	1	3	3	0.401	2;1	25.4	11.14	1074	116.9	9.04																			
34101286		High	cAVSEAAILNScVEPk	Unambiguous	1	1	N-Term(iTRAQ4plex); C1(Methylthio); C12(Methylthio); K16(iTRAQ4plex)	HCD	1.0000	0.0000	1	1	12974	Unique	Used	0.558	3.486E2	2.136E2	115	4.20	0.00	0	30	300	3	709.69281	2127.06388	3.64	69.42	7956	7956	MS2	21/64	Glioma_Nuclear_prep_Gradeall_repeat_4plex.raw
34101286	High		vSAVPTNMAAk	Unambiguous	1	1	N-Term(iTRAQ4plex); K11(iTRAQ4plex)	HCD	1.0000	0.0000	1	1	8206	Unique	Used	0.401	1.760E5	7.741E4	325	3.48	0.00	0	29	13	2	688.88409	1376.76091	-14.63	27.82	2676	2676	MS2	15/20	Glioma_Nuclear_prep_Gradeall_4plex.raw
34101286		High	sTPVTSAVQIPEVk	Unambiguous	1	1	N-Term(iTRAQ4plex); K14(iTRAQ4plex)	HCD	1.0000	0.0000	1	1	10225	Unique	Used	0.345	4.569E3	1.718E3	173	4.77	0.00	0	41	300	2	872.51343	1744.01958	5.76	47.75	4694	4694	MS2	20/26	Glioma_Nuclear_prep_Gradeall_repeat_4plex.raw
34419635	NP_002146.2	HSPA8	3310	heat shock 70 kDa protein 8 isoform 2 [Homo sapiens]	10.26	1	1	2	0.656	1;1	1.2	15.15	643	71.0	6.14																			
34419635		High	vEILANDQGNR	Unambiguous	1	1	N-Term(iTRAQ4plex)	HCD	1.0000	0.0000	1	1	8255	Unique	Used	0.662	2.148E4	1.549E4	223	3.40	0.00	0	[illegible]	130	2	686.87016	1372.73308	2.12	26.25	2595	2595	MS2	16/20	Glioma_Nuclear_prep_Gradeall_repeat_4plex.raw
34419635	High		vEILANDQGNR	Unambiguous	1	1	N-Term(iTRAQ4plex)	HCD	1.0000	0.0000	1	1	8048	Unique	Used	0.650	1.865E5	1.331E5	224	3.15	0.00	0	14	11	2	686.85797	1372.70867	-15.66	26.70	2505	2505	MS2	16/20	Glioma_Nuclear_prep_Gradeall_4plex.raw
34740329	NP_919223.1	HNRNPA3	220988	heterogeneous nuclear ribonucleoprotein A3 [Homo sapiens]	31.22	1	10	29	0.275	16;13	38.7	97.89	378	39.6	9.01																			
34740329		High	sSGSPYGGGYGSGGGSGGYGSR	Unambiguous	1	1	N-Term(iTRAQ4plex)	HCD	1.0000	0.0000	1	1	7948	Unique	Used	0.128	1.593E4	2.223E3	31	7.21	0.00	0	0	300	2	1027.94800	2054.88872	-1.65	23.27	2271	2271	MS2	31/42	Glioma_Nuclear_prep_Gradeall_repeat_4plex.raw
34740329		High	eDTEEYNLR	Unambiguous	1	1	N-Term(iTRAQ4plex)	HCD	1.0000	0.0000	1	1	7928	Unique	Used	0.260	7.413E4	2.104E4	107	3.74	0.00	0	10	128	2	656.81165	1312.61601	1.71	23.07	2250	2250	MS2	16/16	Glioma_Nuclear_prep_Gradeall_repeat_4plex.raw
34740329		High	iETIEVMEDR	Unambiguous	1	1	N-Term(iTRAQ4plex)	HCD	1.0000	0.0000	1	1	10250	Unique	Used	0.262	4.834E3	1.381E4	231	4.21	0.00	0	38	193	2	689.85655	1378.70403	2.55	48.04	4721	4721	MS2	15/18	Glioma_Nuclear_prep_Gradeall_repeat_4plex.raw
34740329		High	sSGSPYGGGYGSGGGSGGYGSR	Unambiguous	1	1	N-Term(iTRAQ4plex)	HCD	1.0000	0.0000	1	1	7479	Unique	Used	0.436	1.283E3	6.102E2	29	7.13	0.00	0	26	212	3	685.63562	2054.88231	0.10	18.91	1773	1773	MS2	27/84	Glioma_Nuclear_prep_Gradeall_repeat_4plex.raw
34740329	High		sSGSPYGGGYGSGGGSGGYGSR	Unambiguous	1	1	N-Term(iTRAQ4plex)	HCD	1.0000	0.0000	1	1	7861	Unique	Used	0.150	1.219E5	2.003E4	33	6.75	0.00	0	0	14	2	1027.95142	2054.89556	1.68	25.37	2302	2302	MS2	33/42	Glioma_Nuclear_prep_Gradeall_4plex.raw
34740329	High		eDTEEYNLR	Unambiguous	1	1	N-Term(iTRAQ4plex)	HCD	1.0000	0.0000	1	1	7866	Unique	Used	0.278	8.174E5	2.496E5	109	3.55	0.00	0	7	5	2	656.81184	1312.61479	0.78	24.83	2218	2218	MS2	16/16	Glioma_Nuclear_prep_Gradeall_4plex.raw
34740329	High		iETIEVMEDR	Unambiguous	1	1	N-Term(iTRAQ4plex)	HCD	1.0000	0.0000	1	1	9697	Unique	Used	0.281	1.246E5	3.840E5	242	3.85	0.00	0	54	5	2	689.86011	1378.71294	9.01	38.67	4301	4301	MS2	14/18	Glioma_Nuclear_prep_Gradeall_4plex.raw
34740329		High	iFGGGLSFETTDDSLR	Unambiguous	1	1	N-Term(iTRAQ4plex)	HCD	1.0000	0.0000	1	1	12193	Unique	Used	0.208	3.424E3	7.794E2	236	6.25	0.00	0	13	31	2	958.00537	1915.00347	3.46	56.91	7024	7024	MS2	23/30	Glioma_Nuclear_prep_Gradeall_repeat_4plex.raw
34740329	High		iETIEVMEDR	Unambiguous	1	1	N-Term(iTRAQ4plex)	HCD	1.0000	0.0000	1	1	9630	Unique	Used	0.307	4.990E5	1.517E5	173	3.76	0.00	0	5	3	2	689.84446	1378.68169	-13.66	38.18	4228	4228	MS2	18/18	Glioma_Nuclear_prep_Gradeall_4plex.raw
34740329	High		iFGGGLSFETTDDSLR	Unambiguous	1	1	N-Term(iTRAQ4plex)	HCD	1.0000	0.0000	1	1	12115	Unique	Used	0.310	1.394E5	4.733E4	235	5.40	0.00	0	21	5	2	958.00504	1914.99980	3.55	58.18	6939	6939	MS2	23/30	Glioma_Nuclear_prep_Gradeall_4plex.raw
34740329	High		wGTLTDcVVMR	Unambiguous	1	1	N-Term(iTRAQ4plex); C7(Methylthio)	HCD	1.0000	0.0000	1	1	11830	Unique	Used	0.223	3.722E5	9.127E4	89	4.21	0.00	0	10	23	2	735.85382	1470.70036	-1.41	55.46	6627	6627	MS2	19/20	Glioma_Nuclear_prep_Gradeall_4plex.raw
34740329	High		wGTLTDcVVMR	Unambiguous	1	1	N-Term(iTRAQ4plex); C7(Methylthio)	HCD	1.0000	0.0000	1	1	11758	Unique	Used	0.269	8.888E4	2.618E4	103	3.96	0.00	0	34	98	2	735.85516	1470.70305	0.41	54.85	6548	6548	MS2	19/20	Glioma_Nuclear_prep_Gradeall_4plex.raw
34740329		High	wGTLTDcVVmR	Unambiguous	1	1	N-Term(iTRAQ4plex); C7(Methylthio); M10(Oxidation)	HCD	1.0000	0.0000	1	1	11779	Unique	Used	0.574	5.166E2	3.231E2	92	3.72	0.00	0	25	300	3	496.23758	1486.69818	0.55	68.20	6471	6471	MS2	17/40	Glioma_Nuclear_prep_Gradeall_repeat_4plex.raw

34740329	High		kLFIGGLSFETTDDSLR	Unambiguous	1	1	N-Term(iTRAQ4plex); K1(iTRAQ4plex)	HCD	1.0000	0.0000	1	1	11117	Unique	Used	0.194	6.051E4	1.288E4	154	5.46	0.00	1	5	47	2	1094.09705	2187.18682	-1.47	49.36	5848	5848	MS2	25/32	Glioma_Nuclear_prep_Gradeall_4plex.raw
34740329	High		kLFIGGLSFETTDDSLR	Unambiguous	1	1	N-Term(iTRAQ4plex); K1(iTRAQ4plex)	HCD	1.0000	0.0000	1	1	11062	Unique	Used	0.314	1.834E4	6.315E3	136	6.96	0.00	1	28	18	3	729.73425	2187.18821	-0.84	48.94	5789	5789	MS2	27/64	Glioma_Nuclear_prep_Gradeall_4plex.raw
34740329		High	gFAFVTFDDHDTVDk	Unambiguous	1	1	N-Term(iTRAQ4plex); K15(iTRAQ4plex)	HCD	1.0000	0.0000	1	1	11232	Unique	Used	0.233	6.908E3	1.752E3	108	4.99	0.00	0	11	116	3	667.99921	2001.98307	1.68	60.21	5798	5798	MS2	30/56	Glioma_Nuclear_prep_Gradeall_repeat_4plex.raw
34740329		High	gFAFVTFDDHDTVDk	Unambiguous	1	1	N-Term(iTRAQ4plex); K15(iTRAQ4plex)	HCD	1.0000	0.0000	1	1	11299	Unique	Used	0.270	4.554E3	1.338E3	117	4.90	0.00	0	13	209	3	667.99994	2001.98526	2.78	61.24	5883	5883	MS2	29/56	Glioma_Nuclear_prep_Gradeall_repeat_4plex.raw
34740329		High	gFAFVTFDDHDTVDk	Unambiguous	1	1	N-Term(iTRAQ4plex); K15(iTRAQ4plex)	HCD	1.0000	0.0000	1	1	11239	Unique	Used	0.288	5.979E3	1.880E3	121	6.09	0.00	0	10	300	2	1001.49506	2001.98284	1.57	60.30	5806	5806	MS2	24/28	Glioma_Nuclear_prep_Gradeall_repeat_4plex.raw
34740329		High	gFAFVTFDDHDTVDk	Unambiguous	1	1	N-Term(iTRAQ4plex); K15(iTRAQ4plex)	HCD	1.0000	0.0000	1	1	11364	Unique	Used	0.408	1.279E3	5.683E2	106	4.97	0.00	0	30	300	3	668.00067	2001.98746	3.88	62.31	5970	5970	MS2	27/56	Glioma_Nuclear_prep_Gradeall_repeat_4plex.raw
34740329		High	gFAFVTFDDHDTVDk	Unambiguous	1	1	N-Term(iTRAQ4plex); K15(iTRAQ4plex)	HCD	1.0000	0.0000	1	1	11448	Unique	Used	0.549	8.937E2	5.344E2	105	3.81	0.00	0	54	300	3	667.99841	2001.98069	0.49	63.47	6070	6070	MS2	25/56	Glioma_Nuclear_prep_Gradeall_repeat_4plex.raw
34740329	High		gFAFVTFDDHDTVDk	Unambiguous	1	1	N-Term(iTRAQ4plex); K15(iTRAQ4plex)	HCD	1.0000	0.0000	1	1	10293	Unique	Used	0.268	2.238E5	6.575E4	85	5.64	0.00	0	4	9	2	1001.47919	2001.95110	-14.29	43.03	4949	4949	MS2	24/28	Glioma_Nuclear_prep_Gradeall_4plex.raw
34740329	High		gFAFVTFDDHDTVDk	Unambiguous	1	1	N-Term(iTRAQ4plex); K15(iTRAQ4plex)	HCD	1.0000	0.0000	1	1	10395	Unique	Used	0.344	9.822E4	3.704E4	85	5.74	0.00	0	21	16	2	1001.47864	2001.95000	-14.83	43.80	5060	5060	MS2	24/28	Glioma_Nuclear_prep_Gradeall_4plex.raw
34740329	High		gFAFVTFDDHDTVDk	Unambiguous	1	1	N-Term(iTRAQ4plex); K15(iTRAQ4plex)	HCD	1.0000	0.0000	1	1	10443	Unique	Used	0.353	6.117E4	2.369E4	87	4.94	0.00	0	29	7	3	667.98877	2001.95176	-13.96	44.19	5114	5114	MS2	31/56	Glioma_Nuclear_prep_Gradeall_4plex.raw
34740329	High		gFAFVTFDDHDTVDk	Unambiguous	1	1	N-Term(iTRAQ4plex); K15(iTRAQ4plex)	HCD	1.0000	0.0000	1	1	10283	Unique	Used	0.438	6.342E4	3.048E4	78	4.61	0.00	0	31	8	3	667.98853	2001.95102	-14.32	42.96	4938	4938	MS2	28/56	Glioma_Nuclear_prep_Gradeall_4plex.raw
34740329		High	eDSVkPGAHLTVk	Unambiguous	1	1	N-Term(iTRAQ4plex); K5(iTRAQ4plex); K13(iTRAQ4plex)	HCD	1.0000	0.0000	1	1	7708	Unique	Used	0.191	1.616E4	3.357E3	126	4.91	0.00	0	19	300	2	907.02472	1813.04216	-6.76	21.01	2018	2018	MS2	17/24	Glioma_Nuclear_prep_Gradeall_repeat_4plex.raw
34740329		High	iFVGGIkEDTEEYNLR	Unambiguous	1	1	N-Term(iTRAQ4plex); K7(iTRAQ4plex)	HCD	1.0000	0.0000	1	1	10950	Unique	Used	0.140	4.178E3	6.358E2	121	5.80	0.00	1	5	300	2	1086.08557	2171.16387	2.37	56.54	5477	5477	MS2	25/30	Glioma_Nuclear_prep_Gradeall_repeat_4plex.raw
34740329		High	iFVGGIkEDTEEYNLR	Unambiguous	1	1	N-Term(iTRAQ4plex); K7(iTRAQ4plex)	HCD	1.0000	0.0000	1	1	10937	Unique	Used	0.185	4.754E3	9.567E2	134	3.50	0.00	1	14	263	3	724.39355	2171.16611	3.40	56.39	5464	5464	MS2	25/60	Glioma_Nuclear_prep_Gradeall_repeat_4plex.raw
34740329	High		iFVGGIkEDTEEYNLR	Unambiguous	1	1	N-Term(iTRAQ4plex); K7(iTRAQ4plex)	HCD	1.0000	0.0000	1	1	10057	Unique	Used	0.130	1.893E5	2.705E4	152	5.26	0.00	1	2	12	2	1086.06665	2171.12602	-15.06	41.26	4691	4691	MS2	24/30	Glioma_Nuclear_prep_Gradeall_4plex.raw
34740329	High		gEEGHDPkEPEQLR	Unambiguous	1	1	N-Term(iTRAQ4plex); K8(iTRAQ4plex)	HCD	1.0000	0.0000	1	1	7282	Unique	Used	0.280	2.799E4	8.607E3	98	3.88	0.00	1	4	28	2	954.98309	1908.95891	-3.42	21.22	1674	1674	MS2	18/26	Glioma_Nuclear_prep_Gradeall_4plex.raw
38201710	NP_006377.2	DDX17	10521	probable ATP-dependent RNA helicase DDX17 isoform 1 [Homo sapiens]	32.24	7	15	23	0.417	9;14	23.5	87.65	729	80.2	8.27																			
38201710		High	lMQLVDHR	Unambiguous	2	1	N-Term(iTRAQ4plex)	HCD	1.0000	0.0000	1	1	9388	Unique	Used	0.380	6.504E3	2.691E3	181	3.84	0.00	0	17	239	3	385.88513	1155.64084	-1.46	38.02	3792	3792	MS2	16/28	Glioma_Nuclear_prep_Gradeall_repeat_4plex.raw
38201710		High	lTPYEVDELR	Unambiguous	2	1	N-Term(iTRAQ4plex)	HCD	1.0000	0.0000	1	1	10338	Unique	Used	0.386	1.968E4	8.278E3	289	3.96	0.00	0	56	300	2	689.87238	1378.73747	2.88	49.11	4816	4816	MS2	15/18	Glioma_Nuclear_prep_Gradeall_repeat_4plex.raw
38201710		High	nFYVEHPEVAR	Unambiguous	2	1	N-Term(iTRAQ4plex)	HCD	1.0000	0.0000	1	1	8590	Unique	Used	0.445	1.139E4	5.529E3	150	4.12	0.00	0	43	123	3	502.25986	1504.76502	-1.01	29.53	2948	2948	MS2	21/40	Glioma_Nuclear_prep_Gradeall_repeat_4plex.raw
38201710		High	lTPYEVDELRR	Unambiguous	2	1	N-Term(iTRAQ4plex)	HCD	1.0000	0.0000	1	1	9645	Unique	Used	0.463	3.352E3	1.691E3	237	3.51	0.00	1	31	300	3	512.28143	1534.82975	-3.18	40.91	4071	4071	MS2	15/40	Glioma_Nuclear_prep_Gradeall_repeat_4plex.raw
38201710		High	sSQSSSQQFSGIGR	Unambiguous	2	1	N-Term(iTRAQ4plex)	HCD	1.0000	0.0000	1	1	7908	Unique	Used	0.483	1.000E4	5.268E3	119	4.84	0.00	0	0	293	2	800.39044	1599.77361	-6.73	22.89	2229	2229	MS2	23/26	Glioma_Nuclear_prep_Gradeall_repeat_4plex.raw
38201710		High	lTPYEVDELR	Unambiguous	2	1	N-Term(iTRAQ4plex)	HCD	1.0000	0.0000	1	1	10430	Unique	Used	0.493	6.631E3	3.561E3	298	3.23	0.00	0	49	300	2	689.87115	1378.73503	1.11	50.18	4913	4913	MS2	13/18	Glioma_Nuclear_prep_Gradeall_repeat_4plex.raw
38201710		High	dWVLNEFR	Unambiguous	2	1	N-Term(iTRAQ4plex)	HCD	1.0000	0.0000	1	1	11588	Unique	Used	0.590	5.811E3	3.736E3	279	3.17	0.00	0	48	300	2	611.82257	1222.63786	3.38	65.42	6236	6236	MS2	13/14	Glioma_Nuclear_prep_Gradeall_repeat_4plex.raw
38201710	High		lTPYEVDELR	Unambiguous	2	1	N-Term(iTRAQ4plex)	HCD	1.0000	0.0000	1	1	9710	Unique	Used	0.344	1.314E6	4.963E5	243	4.00	0.00	0	9	4	2	689.86035	1378.71343	-14.56	38.76	4315	4315	MS2	16/18	Glioma_Nuclear_prep_Gradeall_4plex.raw
38201710	High		aPLPDLYPFGTMR	Unambiguous	2	1	N-Term(iTRAQ4plex)	HCD	1.0000	0.0000	1	1	11594	Unique	Used	0.369	1.812E5	7.347E4	310	3.18	0.00	0	33	27	2	811.43036	1621.85344	0.31	53.36	6370	6370	MS2	12/24	Glioma_Nuclear_prep_Gradeall_4plex.raw
38201710	High		nFYVEHPEVAR	Unambiguous	2	1	N-Term(iTRAQ4plex)	HCD	1.0000	0.0000	1	1	8209	Unique	Used	0.409	1.608E5	7.216E4	142	2.89	0.00	0	13	13	2	752.87524	1504.74321	-15.50	27.83	2679	2679	MS2	15/20	Glioma_Nuclear_prep_Gradeall_4plex.raw
38201710		High	cTYLVLDEADR	Unambiguous	2	1	N-Term(iTRAQ4plex); C1(Methylthio)	HCD	1.0000	0.0000	1	1	11402	Unique	Used	0.413	4.999E3	2.251E3	123	4.09	0.00	0	34	300	2	744.35461	1487.70195	1.91	62.83	6015	6015	MS2	17/20	Glioma_Nuclear_prep_Gradeall_repeat_4plex.raw
38201710		High	dMVGIAQTGSGk	Unambiguous	2	1	N-Term(iTRAQ4plex); K12(iTRAQ4plex)	HCD	1.0000	0.0000	1	1	8387	Unique	Used	0.617	1.432E4	9.631E3	253	4.15	0.00	0	66	300	2	726.39417	1451.78105	3.00	27.53	2733	2733	MS2	18/22	Glioma_Nuclear_prep_Gradeall_repeat_4plex.raw
38201710	High		vLEEANQAINPk	Unambiguous	2	1	N-Term(iTRAQ4plex); K12(iTRAQ4plex)	HCD	1.0000	0.0000	1	1	9071	Unique	Used	0.342	1.492E5	5.604E4	234	4.87	0.00	0	11	15	2	807.44653	1613.88579	-15.09	34.06	3617	3617	MS2	21/22	Glioma_Nuclear_prep_Gradeall_4plex.raw
38201710	High		dMVGIAQTGSGk	Unambiguous	2	1	N-Term(iTRAQ4plex); K12(iTRAQ4plex)	HCD	1.0000	0.0000	1	1	8169	Unique	Used	0.513	2.863E5	1.613E5	206	3.94	0.00	0	5	7	2	726.38080	1451.75432	-15.41	27.56	2636	2636	MS2	19/22	Glioma_Nuclear_prep_Gradeall_4plex.raw
38201710		High	gTAYTFFTPGNLk	Unambiguous	2	1	N-Term(iTRAQ4plex); K13(iTRAQ4plex)	HCD	1.0000	0.0000	1	1	11411	Unique	Used	0.537	2.965E3	1.736E3	235	4.82	0.00	0	13	300	2	852.96509	1704.92290	1.72	62.96	6026	6026	MS2	20/24	Glioma_Nuclear_prep_Gradeall_repeat_4plex.raw
38201710	High		gTAYTFFTPGNLk	Unambiguous	2	1	N-Term(iTRAQ4plex); K13(iTRAQ4plex)	HCD	1.0000	0.0000	1	1	10533	Unique	Used	0.699	1.946E5	1.492E5	205	5.06	0.00	0	14	9	2	852.95264	1704.89800	-12.89	44.90	5213	5213	MS2	21/24	Glioma_Nuclear_prep_Gradeall_4plex.raw
38201710		High	eLAQQVQQVADDYGk	Unambiguous	2	1	N-Term(iTRAQ4plex); K15(iTRAQ4plex)	HCD	1.0000	0.0000	1	1	11222	Unique	Used	0.399	2.980E3	1.297E3	193	6.35	0.00	0	20	300	2	990.51953	1980.03179	2.06	60.09	5787	5787	MS2	26/28	Glioma_Nuclear_prep_Gradeall_repeat_4plex.raw
38201710	High		eLAQQVQQVADDYGk	Unambiguous	2	1	N-Term(iTRAQ4plex); K15(iTRAQ4plex)	HCD	1.0000	0.0000	1	1	10415	Unique	Used	0.417	1.088E5	4.975E4	163	6.27	0.00	0	6	20	2	990.50336	1979.99944	-14.27	43.96	5082	5082	MS2	26/28	Glioma_Nuclear_prep_Gradeall_4plex.raw
38201710		High	tIIFVETk	Unambiguous	2	1	N-Term(iTRAQ4plex); K8(iTRAQ4plex)	HCD	1.0000	0.0000	1	1	10028	Unique	Used	0.590	1.548E4	9.956E3	176	3.54	0.00	0	59	300	2	619.88477	1238.76225	1.92	45.38	4482	4482	MS2	13/14	Glioma_Nuclear_prep_Gradeall_repeat_4plex.raw
38201710		High	wDLSELPk	Unambiguous	2	1	N-Term(iTRAQ4plex); K8(iTRAQ4plex)	HCD	1.0000	0.0000	1	1	11023	Unique	Used	0.659	5.558E3	3.993E3	271	3.01	0.00	0	60	300	2	638.36493	1275.72258	3.01	57.44	5557	5557	MS2	13/14	Glioma_Nuclear_prep_Gradeall_repeat_4plex.raw
38201710		High	lIDFLESGk	Unambiguous	2	1	N-Term(iTRAQ4plex); K9(iTRAQ4plex)	HCD	1.0000	0.0000	1	1	12023	Unique	Used	0.350	1.722E4	6.562E3	281	3.49	0.00	0	16	300	2	655.38495	1309.76262	1.53	72.44	6813	6813	MS2	15/16	Glioma_Nuclear_prep_Gradeall_repeat_4plex.raw
38201710	High		lIDFLESGk	Unambiguous	2	1	N-Term(iTRAQ4plex); K9(iTRAQ4plex)	HCD	1.0000	0.0000	1	1	11339	Unique	Used	0.337	1.553E5	5.743E4	297	3.56	0.00	0	35	30	2	655.38434	1309.76140	0.60	51.22	6092	6092	MS2	15/16	Glioma_Nuclear_prep_Gradeall_4plex.raw
38201710	High		lIDFLESGk	Unambiguous	2	1	N-Term(iTRAQ4plex); K9(iTRAQ4plex)	HCD	1.0000	0.0000	1	1	11261	Unique	Used	0.375	1.603E5	6.603E4	297	3.80	0.00	0	21	39	2	655.38251	1309.75774	-2.20	50.53	6006	6006	MS2	15/16	Glioma_Nuclear_prep_Gradeall_4plex.raw
38201714	NP_001410.2	ELAVL1	1994	ELAV-like protein 1 [Homo sapiens]	31.29	1	9	18	0.429	8;10	24.3	43.19	326	36.1	9.17																			
38201714		High	vLVDQTTGLSR	Unambiguous	1	1	N-Term(iTRAQ4plex)	HCD	1.0000	0.0000	1	1	8901	Unique	Used	0.314	8.129E4	2.779E4	253	3.62	0.00	0	20	69	2	666.88263	1332.75798	-1.81	32.67	3275	3275	MS2	17/20	Glioma_Nuclear_prep_Gradeall_repeat_4plex.raw
38201714		High	nVALLSQLYHSPAR	Unambiguous	1	1	N-Term(iTRAQ4plex)	HCD	1.0000	0.0000	1	1	10843	Unique	Used	0.336	4.569E3	1.675E3	178	5.09	0.00	0	20	300	2	856.98328	1712.95928	1.64	55.21	5360	5360	MS2	22/26	Glioma_Nuclear_prep_Gradeall_repeat_4plex.raw
38201714		High	dVEDMFSR	Unambiguous	1	1	N-Term(iTRAQ4plex)	HCD	1.0000	0.0000	1	1	9892	Unique	Used	0.516	1.339E4	7.526E3	64	3.09	0.00	0	35	300	2	571.76843	1142.52959	2.36	43.68	4332	4332	MS2	13/14	Glioma_Nuclear_prep_Gradeall_repeat_4plex.raw
38201714		High	nVALLSQLYHSPAR	Unambiguous	1	1	N-Term(iTRAQ4plex)	HCD	1.0000	0.0000	1	1	10826	Unique	Used	0.523	1.983E3	1.130E3	158	6.35	0.00	0	33	300	3	571.65839	1712.96061	2.42	55.01	5342	5342	MS2	30/52	Glioma_Nuclear_prep_Gradeall_repeat_4plex.raw
38201714	High		tNLIVNYLPQNMTQDELR	Unambiguous	1	1	N-Term(iTRAQ4plex)	HCD	1.0000	0.0000	1	1	11654	Unique	Used	0.262	2.441E4	7.025E3	137	5.74	0.00	0	8	56	2	1153.59998	2306.19267	-0.22	53.89	6435	6435	MS2	23/34	Glioma_Nuclear_prep_Gradeall_4plex.raw
38201714	High		nVALLSQLYHSPAR	Unambiguous	1	1	N-Term(iTRAQ4plex)	HCD	1.0000	0.0000	1	1	9882	Unique	Used	0.381	4.772E4	1.994E4	136	5.61	0.00	0	14	4	3	571.64819	1712.93003	-15.43	40.01	4502	4502	MS2	32/52	Glioma_Nuclear_prep_Gradeall_4plex.raw
38201714	High		nVALLSQLYHSPAR	Unambiguous	1	1	N-Term(iTRAQ4plex)	HCD	1.0000	0.0000	1	1	9911	Unique	Used	0.438	3.439E5	1.652E5	212	4.54	0.00	0	37	12	2	856.97070	1712.93413	-13.04	40.21	4533	4533	MS2	22/26	Glioma_Nuclear_prep_Gradeall_4plex.raw
38201714	High		dANLYISGLPR	Unambiguous	1	1	N-Term(iTRAQ4plex)	HCD	1.0000	0.0000	1	1	9821	Unique	Used	0.492	3.605E5	1.948E5	277	3.91	0.00	0	26	8	2	681.86853	1362.72978	-14.71	39.55	4435	4435	MS2	17/20	Glioma_Nuclear_prep_Gradeall_4plex.raw
38201714	High		fGGPVHHQAQR	Unambiguous	1	1	N-Term(iTRAQ4plex)	HCD	1.0000	0.0000	1	1	7048	Unique	Used	0.571	6.662E3	4.174E3	283	3.59	0.00	0	34	106	3	459.91394	1377.72727	1.16	19.05	1416	1416	MS2	22/40	Glioma_Nuclear_prep_Gradeall_4plex.raw
38201714		High	sLFSSIGEVESAk	Unambiguous	1	1	N-Term(iTRAQ4plex); K13(iTRAQ4plex)	HCD	1.0000	0.0000	1	1	11486	Unique	Used	0.371	8.261E3	3.341E3	217	5.59	0.00	0	12	300	2	821.45276	1641.89824	2.69	63.96	6113	6113	MS2	22/24	Glioma_Nuclear_prep_Gradeall_repeat_4plex.raw
38201714		High	sLFSSIGEVESAk	Unambiguous	1	1	N-Term(iTRAQ4plex); K13(iTRAQ4plex)	HCD	1.0000	0.0000	1	1	11485	Unique	Used	0.494	1.372E3	7.384E2	225	5.16	0.00	0	9	281	3	547.97009	1641.89573	1.16	63.95	6112	6112	MS2	22/48	Glioma_Nuclear_prep_Gradeall_repeat_4plex.raw
38201714	High		sLFSSIGEVESAk	Unambiguous	1	1	N-Term(iTRAQ4plex); K13(iTRAQ4plex)	HCD	1.0000	0.0000	1	1	10617	Unique	Used	0.420	3.200E5	1.474E5	217	5.19	0.00	0	8	8	2	821.43945	1641.87163	-13.52	45.52	5304	5304	MS2	23/24	Glioma_Nuclear_prep_Gradeall_4plex.raw
38201714	High		sLFSSIGEVESAk	Unambiguous	1	1	N-Term(iTRAQ4plex); K13(iTRAQ4plex)	HCD	1.0000	0.0000	1	1	10628	Unique	Used	0.465	2.051E4	1.047E4	225	3.53	0.00	0	12	6	3	547.96161	1641.87027	-14.35	45.60	5316	5316	MS2	22/48	Glioma_Nuclear_prep_Gradeall_4plex.raw
38201714		High	iLQVSFk	Unambiguous	1	1	N-Term(iTRAQ4plex); K7(iTRAQ4plex)	HCD	1.0000	0.0000	1	1	10482	Unique	Used	0.479	2.330E4	1.216E4	148	2.97	0.00	0	52	299	2	561.86072	1122.71416	1.45	50.82	4969	4969	MS2	11/12	Glioma_Nuclear_prep_Gradeall_repeat_4plex.raw
38201714		High	fAANPNQNk	Unambiguous	1	1	N-Term(iTRAQ4plex); K9(iTRAQ4plex)	HCD	1.0000	0.0000	1	1	7239	Unique	Used	0.311	6.692E4	2.269E4	198	3.33	0.00	0	19	71	2	646.34338	1291.67949	-15.67	16.86	1521	1521	MS2	16/16	Glioma_Nuclear_prep_Gradeall_repeat_4plex.raw
38201714		High	tNLIVNYLPQNmTQDELR	Unambiguous	1	1	N-Term(iTRAQ4plex); M12(Oxidation)	HCD	1.0000	0.0000	1	1	11625	Unique	Used	0.257	6.121E2	1.713E2	130	3.80	0.00	0	4	300	2	1161.59644	2322.18559	-1.07	65.90	6278	6278	MS2	21/34	Glioma_Nuclear_prep_Gradeall_repeat_4plex.raw
38201714		High	tNLIVNYLPQNmTQDELR	Unambiguous	1	1	N-Term(iTRAQ4plex); M12(Oxidation)	HCD	1.0000	0.0000	1	1	11614	Unique	Used	0.448	3.056E2	1.493E2	147	4.18	0.00	0	13	300	3	774.73633	2322.19443	2.73	65.77	6266	6266	MS2	24/68	Glioma_Nuclear_prep_Gradeall_repeat_4plex.raw
38201714	High		tNLIVNYLPQNmTQDELR	Unambiguous	1	1	N-Term(iTRAQ4plex); M12(Oxidation)	HCD	1.0000	0.0000	1	1	10837	Unique	Used	0.266	3.644E4	1.066E4	115	4.38	0.00	0	17	23	2	1161.58154	2322.15581	-13.90	47.20	5544	5544	MS2	19/34	Glioma_Nuclear_prep_Gradeall_4plex.raw
39753957	NP_056417.2	TOR1AIP1	26092	torsin-1A-interacting protein 1 isoform 2 [Homo sapiens]	19.38	1	1	2	0.595	1;1	22.5	61.97	583	66.2	8.18																			
39753957		High	sIQEAPVSEDLVIR	Unambiguous	1	1	N-Term(iTRAQ4plex)	HCD	1.0000	0.0000	1	1	10550	Unique	Used	0.500	5.780E3	3.153E3	231	4.63	0.00	0	10	300	2	850.47345	1699.93962	2.86	51.64	5042	5042	MS2	21/26	Glioma_Nuclear_prep_Gradeall_repeat_4plex.raw
39753957	High		sIQEAPVSEDLVIR	Unambiguous	1	1	N-Term(iTRAQ4plex)	HCD	1.0000	0.0000	1	1	9876	Unique	Used	0.690	1.142E5	8.646E4	259	3.97	0.00	0	51	25	2	850.45850	1699.90972	-14.73	39.96	4495	4495	MS2	17/26	Glioma_Nuclear_prep_Gradeall_4plex.raw
41327773	NP_055644.2	DDX46	9879	probable ATP-dependent RNA helicase DDX46 isoform 2 [Homo sapiens]	4.07	2	3	4	0.651	3;1	5.0	11.45	1031	117.3	9.29																			
41327773		High	fDETEQALANER	Unambiguous	2	1	N-Term(iTRAQ4plex)	HCD	1.0000	0.0000	1	1	9487	Unique	Used	0.627	7.379E3	5.040E3	141	4.42	0.00	0	33	300	2	783.88232	1566.75737	3.62	39.11	3898	3898	MS2	18/22	Glioma_Nuclear_prep_Gradeall_repeat_4plex.raw
41327773	High		vTYVVLDEADR	Unambiguous	2	1	N-Term(iTRAQ4plex)	HCD	1.0000	0.0000	1	1	9625	Unique	Used	0.648	1.278E5	9.089E4	210	3.03	0.00	0	40	24	2	712.36920	1423.73113	-16.75	38.14	4222	4222	MS2	14/20	Glioma_Nuclear_prep_Gradeall_4plex.raw
41327773	High		tIAFLLPMFR	Unambiguous	2	1	N-Term(iTRAQ4plex)	HCD	1.0000	0.0000	1	1	13035	Unique	Used	0.655	5.469E3	3.930E3	288	3.33	0.00	0	53	300	2	676.89948	1352.79167	2.61	70.58	8045	8045	MS2	13/18	Glioma_Nuclear_prep_Gradeall_repeat_4plex.raw
41327773	High		tIAFLLPmFR	Unambiguous	2	1	N-Term(iTRAQ4plex); M8(Oxidation)	HCD	1.0000	0.0000	1	1	12426	Unique	Used	0.704	1.204E4	9.311E3	239	3.05	0.00	0	62	300	2	684.89594	1368.78459	1.12	61.43	7280	7280	MS2	14/18	Glioma_Nuclear_prep_Gradeall_repeat_4plex.raw
42560244	NP_004783.2	PPIG	9360	peptidyl-prolyl cis-trans isomerase G [Homo sapiens]	5.70	1	3	5	0.610	2;3	16.6	11.86	754	88.6	10.29																			
42560244		High	eFLLSMANR	Unambiguous	1	1	N-Term(iTRAQ4plex)	HCD	1.0000	0.0000	1	1	10965	Unique	Used	0.610	9.828E3	6.534E3	264	3.22	0.00	0	36	300	2	612.83038	1224.65349	0.59	56.73	5494	5494	MS2	14/16	Glioma_Nuclear_prep_Gradeall_repeat_4plex.raw
42560244		High	iLScGELIPk	Unambiguous	1	1	N-Term(iTRAQ4plex); C4(Methylthio); K10(iTRAQ4plex)	HCD	1.0000	0.0000	1	1	12182	Unique	Used	0.491	2.386E3	1.277E3	256	3.48	0.00	0	33	300	2	703.90356	1406.79985	0.61	75.74	7070	7070	MS2	16/18	Glioma_Nuclear_prep_Gradeall_repeat_4plex.raw
42560244	High		iLScGELIPk	Unambiguous	1	1	N-Term(iTRAQ4plex); C4(Methylthio); K10(iTRAQ4plex)	HCD	1.0000	0.0000	1	1	11572	Unique	Used	0.566	5.446E4	3.384E4	257	3.85	0.00	0	38	50	2	703.90344	1406.79961	0.43	53.19	6346	6346	MS2	17/18	Glioma_Nuclear_prep_Gradeall_4plex.raw
42560244	High		iLScGELIPk	Unambiguous	1	1	N-Term(iTRAQ4plex); C4(Methylthio); K10(iTRAQ4plex)	HCD	1.0000	0.0000	1	1	11483	Unique	Used	0.751	1.912E4	1.575E4	259	3.72	0.00	0	48	131	2	703.90167	1406.79607	-2.08	52.45	6249	6249	MS2	16/18	Glioma_Nuclear_prep_Gradeall_4plex.raw
42560244		High	tDAASkPFAEVR	Unambiguous	1	1	N-Term(iTRAQ4plex); K6(iTRAQ4plex)	HCD	1.0000	0.0000	1	1	8181	Unique	Used	0.700	2.809E3	2.144E3	249	3.66	0.00	0	33	300	2	790.43408	1579.86089	-4.68	25.52	2516	2516	MS2	16/22	Glioma_Nuclear_prep_Gradeall_repeat_4plex.raw
48255898	NP_620614.2	SMARCA2	6595	probable global transcription activator SNF2L2 isoform b [Homo sapiens]	6.62	6	4	5	0.640	3;2	12.6	22.76	1572	179.2	7.27																			
48255898	High		iLLDPNSEEVSEk	Unambiguous	3	1	N-Term(iTRAQ4plex); K13(iTRAQ4plex)	HCD	1.0000	0.0000	1	1	9428	Unique	Used	0.640	8.536E4	5.993E4	261	4.21	0.00	0	44	33	2	880.96527	1760.92327	-16.36	36.71	4008	4008	MS2	17/24	Glioma_Nuclear_prep_Gradeall_4plex.raw
48255898	High		qMNAIIDTVINYk	Unambiguous	6	1	N-Term(iTRAQ4plex); K13(iTRAQ4plex)	HCD	1.0000	0.0000	1	1	12205	Unique	Used	0.675	1.228E4	9.097E3	220	5.70	0.00	0	42	298	2	906.00891	1811.01055	7.16	59.04	7037	7037	MS2	21/24	Glioma_Nuclear_prep_Gradeall_4plex.raw
48255898		High	vLFGPEAPk	Unambiguous	3	1	N-Term(iTRAQ4plex); K9(iTRAQ4plex)	HCD	1.0000	0.0000	1	1	9600	Unique	Used	0.502	1.426E4	7.800E3	250	3.44	0.00	0	80	300	2	623.37854	1245.74980	4.20	40.40	4023	4023	MS2	14/16	Glioma_Nuclear_prep_Gradeall_repeat_4plex.raw
48255898	High		vLFGPEAPk	Unambiguous	3	1	N-Term(iTRAQ4plex); K9(iTRAQ4plex)	HCD	1.0000	0.0000	1	1	8970	Unique	Used	0.576	1.184E5	7.482E4	272	3.08	0.00	0	13	19	2	623.36536	1245.72344	-16.97	33.30	3506	3506	MS2	14/16	Glioma_Nuclear_prep_Gradeall_4plex.raw
48255898		High	aIEDGNLEEmEEEVR	Unambiguous	5	1	N-Term(iTRAQ4plex); M10(Oxidation)	HCD	1.0000	0.0000	1	1	9299	Unique	Used	0.690	1.928E3	1.449E3	40	3.96	0.00	0	15	300	2	961.94476	1922.88225	2.69	37.07	3698	3698	MS2	20/28	Glioma_Nuclear_prep_Gradeall_repeat_4plex.raw
49355765	NP_115657.2	ELAVL3	1995	ELAV-like protein 3 isoform 2 [Homo sapiens]	26.39	2	3	5	0.581	4;1	37.8	29.87	360	38.8	9.28																			
49355765	High		tGQALLTHLYQSSAR	Unambiguous	2	1	N-Term(iTRAQ4plex)	HCD	1.0000	0.0000	1	1	9724	Unique	Used	0.638	5.413E4	3.790E4	123	3.69	0.00	0	38	6	3	597.31818	1789.93998	-15.52	38.86	4330	4330	MS2	19/56	Glioma_Nuclear_prep_Gradeall_4plex.raw
49355765		High	sLFGSIGDIESck	Unambiguous	2	1	N-Term(iTRAQ4plex); C12(Methylthio); K13(iTRAQ4plex)	HCD	1.0000	0.0000	1	1	12321	Unique	Used	0.290	3.147E3	9.947E2	149	4.31	0.00	0	30	300	2	845.43024	1689.85320	6.00	79.59	7357	7357	MS2	20/24	Glioma_Nuclear_prep_Gradeall_repeat_4plex.raw
49355765	High		sLFGSIGDIESck	Unambiguous	2	1	N-Term(iTRAQ4plex); C12(Methylthio); K13(iTRAQ4plex)	HCD	1.0000	0.0000	1	1	11822	Unique	Used	0.272	1.094E5	3.260E4	184	5.77	0.00	0	20	74	2	845.42706	1689.84685	2.24	55.39	6618	6618	MS2	22/24	Glioma_Nuclear_prep_Gradeall_4plex.raw
49355765	High		sLFGSIGDIESck	Unambiguous	2	1	N-Term(iTRAQ4plex); C12(Methylthio); K13(iTRAQ4plex)	HCD	1.0000	0.0000	1	1	11844	Unique	Used	0.581	4.944E3	3.153E3	164	4.18	0.00	0	36	57	3	563.95416	1689.84793	2.89	55.58	6642	6642	MS2	17/48	Glioma_Nuclear_prep_Gradeall_4plex.raw
49355765	High		tNLIVNYLPQNMTQDEFk	Unambiguous	2	1	N-Term(iTRAQ4plex); K18(iTRAQ4plex)	HCD	1.0000	0.0000	1	1	11798	Unique	Used	0.602	5.400E3	3.565E3	170	5.36	0.00	0	11	33	3	819.42944	2456.27378	0.14	55.19	6592	6592	MS2	22/68	Glioma_Nuclear_prep_Gradeall_4plex.raw
51477708	NP_001003810.1	HNRNPD	3184	heterogeneous nuclear ribonucleoprotein D0 isoform d [Homo sapiens]	20.56	4	4	11	0.218	8;3	37.9	66.61	287	30.7	8.41																			
51477708	High		gFcFITFk	Unambiguous	4	1	N-Term(iTRAQ4plex); C3(Methylthio); K8(iTRAQ4plex)	HCD	1.0000	0.0000	1	1	12229	Unique	Used	0.221	4.933E4	1.198E4	254	3.12	0.00	0	44	178	2	648.84271	1296.67815	4.49	59.28	7064	7064	MS2	13/14	Glioma_Nuclear_prep_Gradeall_4plex.raw
51477708		High	fGEVVDcTLk	Unambiguous	4	1	N-Term(iTRAQ4plex); C7(Methylthio); K10(iTRAQ4plex)	HCD	1.0000	0.0000	1	1	11621	Unique	Used	0.176	1.641E4	3.141E3	188	3.85	0.00	0	38	300	2	722.87689	1444.74651	3.20	65.85	6274	6274	MS2	18/18	Glioma_Nuclear_prep_Gradeall_repeat_4plex.raw
51477708		High	fGEVVDcTLk	Unambiguous	4	1	N-Term(iTRAQ4plex); C7(Methylthio); K10(iTRAQ4plex)	HCD	1.0000	0.0000	1	1	11453	Unique	Used	0.218	1.508E4	3.589E3	165	3.81	0.00	0	49	300	2	722.87622	1444.74516	2.27	63.54	6076	6076	MS2	18/18	Glioma_Nuclear_prep_Gradeall_repeat_4plex.raw
51477708	High		fGEVVDcTLk	Unambiguous	4	1	N-Term(iTRAQ4plex); C7(Methylthio); K10(iTRAQ4plex)	HCD	1.0000	0.0000	1	1	10663	Unique	Used	0.199	5.436E5	1.188E5	134	3.66	0.00	0	43	9	2	722.86627	1444.72527	-11.50	45.88	5355	5355	MS2	18/18	Glioma_Nuclear_prep_Gradeall_4plex.raw
51477708	High		fGEVVDcTLk	Unambiguous	4	1	N-Term(iTRAQ4plex); C7(Methylthio); K10(iTRAQ4plex)	HCD	1.0000	0.0000	1	1	10571	Unique	Used	0.225	4.928E5	1.218E5	128	3.79	0.00	0	32	11	2	722.86511	1444.72295	-13.10	45.18	5254	5254	MS2	18/18	Glioma_Nuclear_prep_Gradeall_4plex.raw
51477708	High		iFVGGLSPDTPEEk	Unambiguous	4	1	N-Term(iTRAQ4plex); K14(iTRAQ4plex)	HCD	1.0000	0.0000	1	1	9910	Unique	Used	0.188	2.086E5	4.314E4	244	4.79	0.00	0	11	11	2	888.97406	1776.94084	-12.05	40.21	4532	4532	MS2	22/26	Glioma_Nuclear_prep_Gradeall_4plex.raw
51477708	High		iFVGGLSPDTPEEk	Unambiguous	4	1	N-Term(iTRAQ4plex); K14(iTRAQ4plex)	HCD	1.0000	0.0000	1	1	9811	Unique	Used	0.222	2.280E5	5.559E4	233	5.37	0.00	0	6	21	2	888.97565	1776.94402	-10.26	39.48	4424	4424	MS2	21/26	Glioma_Nuclear_prep_Gradeall_4plex.raw
51477708	High		eYFGGFGEVESIELPMDNk	Unambiguous	4	1	N-Term(iTRAQ4plex); K19(iTRAQ4plex)	HCD	1.0000	0.0000	1	1	12210	Unique	Used	0.127	3.193E4	4.449E3	113	7.91	0.00	0	2	89	2	1225.10352	2449.19975	6.58	59.09	7043	7043	MS2	27/36	Glioma_Nuclear_prep_Gradeall_4plex.raw
51477708	High		eYFGGFGEVESIELPMDNk	Unambiguous	4	1	N-Term(iTRAQ4plex); K19(iTRAQ4plex)	HCD	1.0000	0.0000	1	1	12275	Unique	Used	0.171	9.045E3	1.695E3	91	7.50	0.00	0	17	190	2	1225.09485	2449.18242	-0.50	59.76	7114	7114	MS2	27/36	Glioma_Nuclear_prep_Gradeall_4plex.raw
51477708	High		eYFGGFGEVESIELPMDNk	Unambiguous	4	1	N-Term(iTRAQ4plex); K19(iTRAQ4plex)	HCD	1.0000	0.0000	1	1	12217	Unique	Used	0.441	1.126E4	5.446E3	95	6.58	0.00	0	6	13	3	817.06836	2449.19052	2.81	59.18	7051	7051	MS2	27/72	Glioma_Nuclear_prep_Gradeall_4plex.raw
51477708		High	eYFGGFGEVESIELPmDNk	Unambiguous	4	1	N-Term(iTRAQ4plex); M16(Oxidation); K19(iTRAQ4plex)	HCD	1.0000	0.0000	1	1	12222	Unique	Used	0.336	1.831E2	6.705E1	99	5.54	0.00	0	12	300	3	822.39935	2465.18351	2.01	76.66	7141	7141	MS2	26/72	Glioma_Nuclear_prep_Gradeall_repeat_4plex.raw
52632383	NP_001524.2	HNRNPL	3191	heterogeneous nuclear ribonucleoprotein L isoform a [Homo sapiens]	23.43	2	11	26	0.345	13;13	38.4	71.74	589	64.1	8.22																			
52632383		High	mGPPVGGHR	Unambiguous	2	1	N-Term(iTRAQ4plex)	HCD	1.0000	0.0000	1	1	6938	Unique	Used	0.167	6.233E4	1.135E4	228	3.62	0.00	0	38	56	3	351.19040	1051.55664	-2.05	14.23	1202	1202	MS2	16/32	Glioma_Nuclear_prep_Gradeall_repeat_4plex.raw
52632383		High	yYGGSSEGGR	Unambiguous	1	1	N-Term(iTRAQ4plex)	HCD	1.0000	0.0000	1	1	7031	Unique	Used	0.243	7.232E4	1.912E4	49	2.85	0.00	0	33	165	2	573.76556	1146.52385	-5.05	15.07	1300	1300	MS2	17/18	Glioma_Nuclear_prep_Gradeall_repeat_4plex.raw
52632383		High	tPASPVVHIR	Unambiguous	1	1	N-Term(iTRAQ4plex)	HCD	1.0000	0.0000	1	1	7922	Unique	Used	0.457	2.526E4	1.257E4	236	5.17	0.00	0	25	41	3	407.57953	1220.72403	0.67	23.03	2244	2244	MS2	22/36	Glioma_Nuclear_prep_Gradeall_repeat_4plex.raw
52632383	High		tPASPVVHIR	Unambiguous	1	1	N-Term(iTRAQ4plex)	HCD	1.0000	0.0000	1	1	7723	Unique	Used	0.538	1.544E5	9.111E4	222	4.94	0.00	0	20	3	3	407.57907	1220.72266	-0.45	24.40	2153	2153	MS2	20/36	Glioma_Nuclear_prep_Gradeall_4plex.raw
52632383	High		tPASPVVHIR	Unambiguous	1	1	N-Term(iTRAQ4plex)	HCD	1.0000	0.0000	1	1	7727	Unique	Used	0.693	4.049E5	3.078E5	247	3.06	0.00	0	37	7	2	610.86566	1220.72405	0.68	24.43	2157	2157	MS2	12/18	Glioma_Nuclear_prep_Gradeall_4plex.raw
52632383	High		qPAIMPGQSYGLEDGScSYk	Unambiguous	2	1	N-Term(iTRAQ4plex); C17(Methylthio); K20(iTRAQ4plex)	HCD	1.0000	0.0000	1	1	10331	Unique	Used	0.185	1.001E5	2.037E4	39	4.61	0.00	0	3	13	2	1233.05444	2465.10161	-15.17	43.31	4990	4990	MS2	21/38	Glioma_Nuclear_prep_Gradeall_4plex.raw
52632383		High	lcFSTAQHAS	Unambiguous	2	1	N-Term(iTRAQ4plex); C2(Methylthio)	HCD	1.0000	0.0000	1	1	9440	Unique	Used	0.476	1.310E4	6.794E3	58	3.24	0.00	0	44	300	2	627.79163	1254.57598	2.55	38.55	3847	3847	MS2	12/18	Glioma_Nuclear_prep_Gradeall_repeat_4plex.raw
52632383		High	lcFSTAQHAS	Unambiguous	2	1	N-Term(iTRAQ4plex); C2(Methylthio)	HCD	1.0000	0.0000	1	1	9527	Unique	Used	0.481	2.332E4	1.223E4	47	2.94	0.00	0	18	105	2	627.78662	1254.56597	-5.43	39.58	3944	3944	MS2	11/18	Glioma_Nuclear_prep_Gradeall_repeat_4plex.raw
52632383		High	sSSGLLEWESk	Unambiguous	2	1	N-Term(iTRAQ4plex); K11(iTRAQ4plex)	HCD	1.0000	0.0000	1	1	10232	Unique	Used	0.349	1.507E4	5.733E3	218	4.74	0.00	0	31	300	2	755.90564	1510.80400	3.19	47.83	4702	4702	MS2	19/20	Glioma_Nuclear_prep_Gradeall_repeat_4plex.raw
52632383		High	nPNGPYPYTLk	Unambiguous	2	1	N-Term(iTRAQ4plex); K11(iTRAQ4plex)	HCD	1.0000	0.0000	1	1	9233	Unique	Used	0.367	1.175E4	4.700E3	312	2.92	0.00	0	48	300	2	776.43750	1551.86772	17.24	36.32	3629	3629	MS2	12/20	Glioma_Nuclear_prep_Gradeall_repeat_4plex.raw
52632383		High	sSSGLLEWESk	Unambiguous	2	1	N-Term(iTRAQ4plex); K11(iTRAQ4plex)	HCD	1.0000	0.0000	1	1	10242	Unique	Used	0.626	1.948E3	1.330E3	227	3.93	0.00	0	55	300	3	504.27209	1510.80173	1.68	47.94	4712	4712	MS2	12/40	Glioma_Nuclear_prep_Gradeall_repeat_4plex.raw
52632383	High		nPNGPYPYTLk	Unambiguous	2	1	N-Term(iTRAQ4plex); K11(iTRAQ4plex)	HCD	1.0000	0.0000	1	1	8747	Unique	Used	0.157	3.541E5	6.116E4	247	3.82	0.00	0	13	10	2	776.41351	1551.81975	-13.67	31.70	3264	3264	MS2	16/20	Glioma_Nuclear_prep_Gradeall_4plex.raw
52632383	High		sSSGLLEWESk	Unambiguous	2	1	N-Term(iTRAQ4plex); K11(iTRAQ4plex)	HCD	1.0000	0.0000	1	1	9558	Unique	Used	0.449	2.406E5	1.185E5	176	4.49	0.00	0	37	15	2	755.89264	1510.77800	-14.02	37.67	4150	4150	MS2	20/20	Glioma_Nuclear_prep_Gradeall_4plex.raw
52632383		High	aITHLNNNFMFGQk	Unambiguous	2	1	N-Term(iTRAQ4plex); K14(iTRAQ4plex)	HCD	1.0000	0.0000	1	1	10394	Unique	Used	0.318	2.340E3	8.099E2	185	6.43	0.00	0	27	300	3	641.67810	1923.01975	2.49	49.76	4875	4875	MS2	23/52	Glioma_Nuclear_prep_Gradeall_repeat_4plex.raw
52632383	High		aITHLNNNFMFGQk	Unambiguous	2	1	N-Term(iTRAQ4plex); K14(iTRAQ4plex)	HCD	1.0000	0.0000	1	1	9576	Unique	Used	0.243	1.414E5	3.770E4	193	6.04	0.00	0	0	17	2	961.99634	1922.98540	-15.38	37.79	4169	4169	MS2	22/26	Glioma_Nuclear_prep_Gradeall_4plex.raw
52632383	High		aITHLNNNFMFGQk	Unambiguous	2	1	N-Term(iTRAQ4plex); K14(iTRAQ4plex)	HCD	1.0000	0.0000	1	1	9580	Unique	Used	0.299	1.433E5	4.702E4	165	5.71	0.00	0	16	3	3	641.66718	1922.98697	-14.56	37.83	4174	4174	MS2	25/52	Glioma_Nuclear_prep_Gradeall_4plex.raw
52632383	High		sDALETLGFLNHYQMk	Unambiguous	2	1	N-Term(iTRAQ4plex); K16(iTRAQ4plex)	HCD	1.0000	0.0000	1	1	11224	Unique	Used	0.204	5.722E4	1.282E4	193	7.33	0.00	0	10	49	2	1078.05920	2155.11113	0.68	50.23	5966	5966	MS2	26/30	Glioma_Nuclear_prep_Gradeall_4plex.raw
52632383	High		sDALETLGFLNHYQMk	Unambiguous	2	1	N-Term(iTRAQ4plex); K16(iTRAQ4plex)	HCD	1.0000	0.0000	1	1	11181	Unique	Used	0.341	4.449E4	1.667E4	114	6.40	0.00	0	23	16	3	719.04059	2155.10721	-1.13	49.89	5919	5919	MS2	34/60	Glioma_Nuclear_prep_Gradeall_4plex.raw
52632383	High		sDALETLGFLNHYQMk	Unambiguous	2	1	N-Term(iTRAQ4plex); K16(iTRAQ4plex)	HCD	1.0000	0.0000	1	1	11282	Unique	Used	0.369	2.215E4	8.978E3	110	6.96	0.00	0	13	26	3	719.04144	2155.10978	0.05	50.71	6029	6029	MS2	34/60	Glioma_Nuclear_prep_Gradeall_4plex.raw
52632383	High		skPGAAMVEMADGYAVDR	Unambiguous	2	1	N-Term(iTRAQ4plex); K2(iTRAQ4plex)	HCD	1.0000	0.0000	1	1	9688	Unique	Used	0.383	3.432E4	1.442E4	99	4.99	0.00	0	14	6	3	719.35187	2156.04105	-14.33	38.60	4291	4291	MS2	29/68	Glioma_Nuclear_prep_Gradeall_4plex.raw
52632383		High	skPGAAmVEmADGYAVDR	Unambiguous	2	1	N-Term(iTRAQ4plex); K2(iTRAQ4plex); M7(Oxidation); M10(Oxidation)	HCD	1.0000	0.0000	1	1	8458	Unique	Used	0.399	1.785E3	7.766E2	112	5.17	0.00	0	11	139	3	730.02649	2188.06491	1.44	28.19	2808	2808	MS2	28/68	Glioma_Nuclear_prep_Gradeall_repeat_4plex.raw
52632383		High	fSTPEQAAk	Unambiguous	2	1	N-Term(iTRAQ4plex); K9(iTRAQ4plex)	HCD	1.0000	0.0000	1	1	7828	Unique	Used	0.301	8.876E4	2.911E4	305	3.08	0.00	0	29	83	2	633.84991	1266.69255	-0.56	22.14	2145	2145	MS2	14/16	Glioma_Nuclear_prep_Gradeall_repeat_4plex.raw
52632383	High		fSTPEQAAk	Unambiguous	2	1	N-Term(iTRAQ4plex); K9(iTRAQ4plex)	HCD	1.0000	0.0000	1	1	7711	Unique	Used	0.231	2.800E6	7.093E5	292	3.13	0.00	0	13	2	2	633.85144	1266.69560	1.85	24.32	2140	2140	MS2	15/16	Glioma_Nuclear_prep_Gradeall_4plex.raw
52632383		High	aITHLNNNFmFGQk	Unambiguous	2	1	N-Term(iTRAQ4plex); M10(Oxidation); K14(iTRAQ4plex)	HCD	1.0000	0.0000	1	1	9826	Unique	Used	0.401	2.284E3	9.978E2	191	5.68	0.00	0	16	268	3	647.00995	1939.01529	2.79	42.94	4262	4262	MS2	24/52	Glioma_Nuclear_prep_Gradeall_repeat_4plex.raw
52632383		High	sDALETLGFLNHYQmk	Unambiguous	2	1	N-Term(iTRAQ4plex); M15(Oxidation); K16(iTRAQ4plex)	HCD	1.0000	0.0000	1	1	11702	Unique	Used	0.262	2.139E3	6.114E2	129	5.56	0.00	0	16	274	3	724.37488	2171.11008	2.54	67.01	6372	6372	MS2	29/60	Glioma_Nuclear_prep_Gradeall_repeat_4plex.raw
52632383	High		sDALETLGFLNHYQmk	Unambiguous	2	1	N-Term(iTRAQ4plex); M15(Oxidation); K16(iTRAQ4plex)	HCD	1.0000	0.0000	1	1	10738	Unique	Used	0.283	3.103E4	9.620E3	164	5.75	0.00	0	0	42	2	1086.04004	2171.07280	-14.63	46.44	5436	5436	MS2	22/30	Glioma_Nuclear_prep_Gradeall_4plex.raw
55956779	NP_001007101.1	SCP2	6342	non-specific lipid-transfer protein isoform 4 proprotein [Homo sapiens]	5.71	6	1	2	2.902	1;1	8.9	3.10	140	15.1	8.92																			
55956779		High	iGGIFAFk	Unambiguous	6	1	N-Term(iTRAQ4plex); K8(iTRAQ4plex)	HCD	1.0000	0.0000	1	1	11655	Unique	Used	3.084	2.272E3	7.638E3	172	3.10	0.00	0	63	300	2	570.85223	1140.69719	-4.20	66.36	6316	6316	MS2	14/14	Glioma_Nuclear_prep_Gradeall_repeat_4plex.raw
55956779	High		iGGIFAFk	Unambiguous	6	1	N-Term(iTRAQ4plex); K8(iTRAQ4plex)	HCD	1.0000	0.0000	1	1	10736	Unique	Used	2.720	3.895E4	1.163E5	202	3.03	0.00	0	58	32	2	570.84528	1140.68328	-16.40	46.42	5434	5434	MS2	13/14	Glioma_Nuclear_prep_Gradeall_4plex.raw
55956921	NP_004490.2	HNRNPAB	3182	heterogeneous nuclear ribonucleoprotein A/B isoform b [Homo sapiens]	13.68	2	4	11	0.353	5;6	30.8	23.07	285	30.6	7.91																			
55956921		High	kFHTVSGSk	Unambiguous	2	1	N-Term(iTRAQ4plex); K1(iTRAQ4plex); K9(iTRAQ4plex)	HCD	1.0000	0.0000	1	1	6785	Unique	Used	0.486	3.054E3	1.618E3	261	3.59	0.00	1	71	112	4	356.46594	1422.84194	-0.70	12.88	1041	1041	MS2	17/48	Glioma_Nuclear_prep_Gradeall_repeat_4plex.raw
55956921		High	iFVGGLNPEATEEk	Unambiguous	2	1	N-Term(iTRAQ4plex); K14(iTRAQ4plex)	HCD	1.0000	0.0000	1	1	10631	Unique	Used	0.266	2.652E4	7.679E3	220	5.94	0.00	0	6	166	2	896.49432	1791.98137	4.58	52.61	5130	5130	MS2	22/26	Glioma_Nuclear_prep_Gradeall_repeat_4plex.raw
55956921		High	iFVGGLNPEATEEk	Unambiguous	2	1	N-Term(iTRAQ4plex); K14(iTRAQ4plex)	HCD	1.0000	0.0000	1	1	10618	Unique	Used	0.693	3.043E3	2.297E3	169	3.74	0.00	0	34	160	3	597.99707	1791.97666	1.95	52.46	5116	5116	MS2	16/52	Glioma_Nuclear_prep_Gradeall_repeat_4plex.raw
55956921	High		iFVGGLNPEATEEk	Unambiguous	2	1	N-Term(iTRAQ4plex); K14(iTRAQ4plex)	HCD	1.0000	0.0000	1	1	9873	Unique	Used	0.340	3.947E5	1.471E5	229	5.23	0.00	0	13	9	2	896.47772	1791.94817	-13.95	39.94	4492	4492	MS2	21/26	Glioma_Nuclear_prep_Gradeall_4plex.raw
55956921		High	gFGFILFk	Unambiguous	2	1	N-Term(iTRAQ4plex); K8(iTRAQ4plex)	HCD	1.0000	0.0000	1	1	12414	Unique	Used	0.331	3.643E3	1.316E3	210	3.42	0.00	0	23	300	2	608.87067	1216.73406	0.64	84.38	7689	7689	MS2	14/14	Glioma_Nuclear_prep_Gradeall_repeat_4plex.raw
55956921		High	gFGFILFk	Unambiguous	2	1	N-Term(iTRAQ4plex); K8(iTRAQ4plex)	HCD	1.0000	0.0000	1	1	12397	Unique	Used	0.353	3.325E3	1.280E3	200	3.21	0.00	0	39	300	2	608.87091	1216.73454	1.04	83.35	7619	7619	MS2	14/14	Glioma_Nuclear_prep_Gradeall_repeat_4plex.raw
55956921		High	gFVFITFk	Unambiguous	2	1	N-Term(iTRAQ4plex); K8(iTRAQ4plex)	HCD	1.0000	0.0000	1	1	12237	Unique	Used	0.573	6.477E3	4.045E3	293	3.18	0.00	0	25	300	2	623.87701	1246.74675	2.34	77.14	7176	7176	MS2	13/14	Glioma_Nuclear_prep_Gradeall_repeat_4plex.raw
55956921	High		gFGFILFk	Unambiguous	2	1	N-Term(iTRAQ4plex); K8(iTRAQ4plex)	HCD	1.0000	0.0000	1	1	12095	Unique	Used	0.331	9.805E4	3.564E4	163	3.48	0.00	0	26	48	2	608.87067	1216.73406	0.64	57.97	6917	6917	MS2	14/14	Glioma_Nuclear_prep_Gradeall_4plex.raw
55956921	High		gFGFILFk	Unambiguous	2	1	N-Term(iTRAQ4plex); K8(iTRAQ4plex)	HCD	1.0000	0.0000	1	1	12045	Unique	Used	0.335	6.527E3	2.400E3	140	3.39	0.00	0	34	240	3	406.24963	1216.73435	0.88	57.43	6861	6861	MS2	13/28	Glioma_Nuclear_prep_Gradeall_4plex.raw
55956921	High		gFGFILFk	Unambiguous	2	1	N-Term(iTRAQ4plex); K8(iTRAQ4plex)	HCD	1.0000	0.0000	1	1	12023	Unique	Used	0.395	8.669E4	3.757E4	159	3.39	0.00	0	28	46	2	608.87036	1216.73345	0.14	57.22	6838	6838	MS2	14/14	Glioma_Nuclear_prep_Gradeall_4plex.raw

55956921	High		gFVFITFk	Unambiguous	2	1	N-Term(iTRAQ4plex); K8(iTRAQ4plex)	HCD	1.0000	0.0000	1	1	11532	Unique	Used	0.412	3.146E5	1.423E5	241	3.26	0.00	0	21	15	2	623.87445	1246.74162	-1.77	52.86	6303	6303	MS2	13/14	Glioma_Nuclear_prep_Gradeall_4plex.raw
56118234	NP_859067.2	CENPV	201161	centromere protein V [Homo sapiens]	23.16	1	4	5	0.397	3;2	36.5	19.48	272	29.7	9.73																			
56118234	High		sGASAAPAASAAAALAPSATR	Unambiguous	1	1	N-Term(iTRAQ4plex)	HCD	1.0000	0.0000	1	1	9064	Unique	Used	0.476	2.927E4	1.530E4	176	6.60	0.00	0	35	31	2	957.49518	1913.98308	-17.32	34.01	3609	3609	MS2	30/40	Glioma_Nuclear_prep_Gradeall_4plex.raw
56118234		High	cGVQSFYTPR	Unambiguous	1	1	N-Term(iTRAQ4plex); C1(Methylthio)	HCD	1.0000	0.0000	1	1	10325	Unique	Used	0.365	1.193E4	4.742E3	70	3.58	0.00	0	46	300	2	674.32123	1347.63518	3.38	48.97	4802	4802	MS2	17/18	Glioma_Nuclear_prep_Gradeall_repeat_4plex.raw
56118234	High		cGVQSFYTPR	Unambiguous	1	1	N-Term(iTRAQ4plex); C1(Methylthio)	HCD	1.0000	0.0000	1	1	9657	Unique	Used	0.397	1.615E5	7.039E4	55	2.91	0.00	0	37	17	2	674.30853	1347.60979	-15.46	38.37	4257	4257	MS2	17/18	Glioma_Nuclear_prep_Gradeall_4plex.raw
56118234		High	sNPGGFGIAPHcLDEGTVR	Unambiguous	1	1	N-Term(iTRAQ4plex); C12(Methylthio)	HCD	1.0000	0.0000	1	1	10907	Unique	Used	0.162	1.235E3	2.176E2	66	5.32	0.00	0	37	300	3	706.34021	2117.00608	1.66	56.02	5431	5431	MS2	30/72	Glioma_Nuclear_prep_Gradeall_repeat_4plex.raw
56118234	High		lLLDTFEYQGLVk	Unambiguous	1	1	N-Term(iTRAQ4plex); K13(iTRAQ4plex)	HCD	1.0000	0.0000	1	1	12177	Unique	Used	0.528	1.568E4	9.080E3	132	5.71	0.00	0	65	300	2	914.03717	1827.06706	8.98	58.74	7006	7006	MS2	23/24	Glioma_Nuclear_prep_Gradeall_4plex.raw
56118310	NP_073568.2	NUCKS1	64710	nuclear ubiquitous casein and cyclin-dependent kinase substrate 1 [Homo sapiens]	11.93	1	2	2	2.337	1;1	14.1	16.88	243	27.3	5.08																			
56118310		High	kVVDYSQFQESDDADEDYGR	Unambiguous	1	1	N-Term(iTRAQ4plex); K1(iTRAQ4plex)	HCD	1.0000	0.0000	1	1	9579	Unique	Used	2.570	2.538E2	7.108E2	37	7.25	0.00	1	5	131	3	885.40448	2654.19889	-4.08	40.18	4001	4001	MS2	33/76	Glioma_Nuclear_prep_Gradeall_repeat_4plex.raw
56118310	High		aTVTPSPVk	Unambiguous	1	1	N-Term(iTRAQ4plex); K9(iTRAQ4plex)	HCD	1.0000	0.0000	1	1	7476	Unique	Used	2.104	5.643E4	1.303E5	225	3.34	0.00	0	35	20	2	594.36053	1187.71379	-8.45	22.65	1885	1885	MS2	13/16	Glioma_Nuclear_prep_Gradeall_4plex.raw
56676330	NP_057371.2	HP1BP3	50809	heterochromatin protein 1-binding protein 3 [Homo sapiens]	26.76	1	13	33	1.652	14;19	25.3	111.59	553	61.2	9.67																			
56676330		High	tIPSWATLSASQLAR	Unambiguous	1	1	N-Term(iTRAQ4plex)	HCD	1.0000	0.0000	1	1	11935	Unique	Used	1.315	1.366E2	1.957E2	194	3.76	0.00	0	38	300	3	582.66101	1745.96848	1.01	70.82	6685	6685	MS2	21/56	Glioma_Nuclear_prep_Gradeall_repeat_4plex.raw
56676330		High	tIPSWATLSASQLAR	Unambiguous	1	1	N-Term(iTRAQ4plex)	HCD	1.0000	0.0000	1	1	11933	Unique	Used	1.382	1.183E3	1.782E3	126	3.63	0.00	0	32	300	2	873.48883	1745.97038	2.10	70.80	6683	6683	MS2	20/28	Glioma_Nuclear_prep_Gradeall_repeat_4plex.raw
56676330		High	tIPSWATLSASQLAR	Unambiguous	1	1	N-Term(iTRAQ4plex)	HCD	1.0000	0.0000	1	1	11988	Unique	Used	1.413	2.784E3	4.287E3	119	4.14	0.00	0	8	300	2	873.48877	1745.97026	2.03	71.80	6763	6763	MS2	21/28	Glioma_Nuclear_prep_Gradeall_repeat_4plex.raw
56676330		High	tIPSWATLSASQLAR	Unambiguous	1	1	N-Term(iTRAQ4plex)	HCD	1.0000	0.0000	1	1	11989	Unique	Used	1.441	1.974E2	3.099E2	201	5.10	0.00	0	6	300	3	582.66077	1745.96775	0.59	71.82	6764	6764	MS2	26/56	Glioma_Nuclear_prep_Gradeall_repeat_4plex.raw
56676330		High	sGASVVAIR	Unambiguous	1	1	N-Term(iTRAQ4plex)	HCD	1.0000	0.0000	1	1	7791	Unique	Used	2.680	1.197E5	3.497E5	218	3.05	0.00	0	14	15	2	502.30493	1003.60259	0.88	21.79	2106	2106	MS2	16/16	Glioma_Nuclear_prep_Gradeall_repeat_4plex.raw
56676330	High		tIPSWATLSASQLAR	Unambiguous	1	1	N-Term(iTRAQ4plex)	HCD	1.0000	0.0000	1	1	11166	Unique	Used	1.369	2.540E5	3.818E5	143	4.16	0.00	0	27	6	2	873.48871	1745.97014	1.96	49.78	5903	5903	MS2	22/28	Glioma_Nuclear_prep_Gradeall_4plex.raw
56676330	High		tIPSWATLSASQLAR	Unambiguous	1	1	N-Term(iTRAQ4plex)	HCD	1.0000	0.0000	1	1	11190	Unique	Used	1.465	3.604E5	5.794E5	137	4.44	0.00	0	9	4	2	873.48132	1745.95537	-6.50	49.95	5929	5929	MS2	22/28	Glioma_Nuclear_prep_Gradeall_4plex.raw
56676330	High		tcSTTALk	Unambiguous	1	1	N-Term(iTRAQ4plex); C2(Methylthio); K8(iTRAQ4plex)	HCD	1.0000	0.0000	1	1	7877	Unique	Used	1.495	3.841E5	6.302E5	138	3.19	0.00	0	29	4	2	579.80774	1158.60820	-1.65	25.49	2320	2320	MS2	14/14	Glioma_Nuclear_prep_Gradeall_4plex.raw
56676330		High	kYVSQYYPk	Unambiguous	1	1	N-Term(iTRAQ4plex); K1(iTRAQ4plex); K9(iTRAQ4plex)	HCD	1.0000	0.0000	1	1	8352	Unique	Used	1.552	1.427E4	2.414E4	246	3.30	0.00	1	34	111	2	804.46576	1607.92424	5.29	27.20	2697	2697	MS2	14/16	Glioma_Nuclear_prep_Gradeall_repeat_4plex.raw
56676330	High		kYVSQYYPk	Unambiguous	1	1	N-Term(iTRAQ4plex); K1(iTRAQ4plex); K9(iTRAQ4plex)	HCD	1.0000	0.0000	1	1	8028	Unique	Used	1.566	1.539E5	2.645E5	226	2.95	0.00	1	11	11	2	804.44836	1607.88945	-16.34	26.55	2483	2483	MS2	14/16	Glioma_Nuclear_prep_Gradeall_4plex.raw
56676330		High	mDAILTEAlk	Unambiguous	1	1	N-Term(iTRAQ4plex); K10(iTRAQ4plex)	HCD	1.0000	0.0000	1	1	11357	Unique	Used	1.698	2.004E3	3.708E3	279	3.94	0.00	0	55	300	2	696.90436	1392.80144	0.23	62.22	5962	5962	MS2	18/18	Glioma_Nuclear_prep_Gradeall_repeat_4plex.raw
56676330		High	mDAILTEAlk	Unambiguous	1	1	N-Term(iTRAQ4plex); K10(iTRAQ4plex)	HCD	1.0000	0.0000	1	1	11432	Unique	Used	2.581	7.307E3	2.056E4	251	4.30	0.00	0	8	137	2	696.90472	1392.80217	0.76	63.25	6051	6051	MS2	18/18	Glioma_Nuclear_prep_Gradeall_repeat_4plex.raw
56676330	High		mDAILTEAlk	Unambiguous	1	1	N-Term(iTRAQ4plex); K10(iTRAQ4plex)	HCD	1.0000	0.0000	1	1	10627	Unique	Used	2.038	1.471E5	3.290E5	276	3.77	0.00	0	16	7	2	696.89380	1392.78032	-14.93	45.59	5315	5315	MS2	18/18	Glioma_Nuclear_prep_Gradeall_4plex.raw
56676330	High		mDAILTEAlk	Unambiguous	1	1	N-Term(iTRAQ4plex); K10(iTRAQ4plex)	HCD	1.0000	0.0000	1	1	10539	Unique	Used	2.390	6.108E4	1.602E5	298	3.97	0.00	0	38	20	2	696.89337	1392.77947	-15.54	44.94	5219	5219	MS2	18/18	Glioma_Nuclear_prep_Gradeall_4plex.raw
56676330		High	sSAVDPEPQVk	Unambiguous	1	1	N-Term(iTRAQ4plex); K11(iTRAQ4plex)	HCD	1.0000	0.0000	1	1	7607	Unique	Used	1.447	1.102E4	1.739E4	195	4.09	0.00	0	7	139	2	722.89386	1444.78044	-5.66	19.99	1908	1908	MS2	19/20	Glioma_Nuclear_prep_Gradeall_repeat_4plex.raw
56676330		High	gASGSFVVVQk	Unambiguous	1	1	N-Term(iTRAQ4plex); K11(iTRAQ4plex)	HCD	1.0000	0.0000	1	1	8783	Unique	Used	2.409	1.485E4	3.899E4	296	4.32	0.00	0	16	91	2	683.89032	1366.77336	-14.60	31.44	3151	3151	MS2	20/20	Glioma_Nuclear_prep_Gradeall_repeat_4plex.raw
56676330		High	gASGSFVVVQk	Unambiguous	1	1	N-Term(iTRAQ4plex); K11(iTRAQ4plex)	HCD	1.0000	0.0000	1	1	8837	Unique	Used	2.629	7.913E4	2.267E5	254	4.55	0.00	0	8	16	2	683.90131	1366.79534	1.48	32.05	3208	3208	MS2	20/20	Glioma_Nuclear_prep_Gradeall_repeat_4plex.raw
56676330		High	gASGSFVVVQk	Unambiguous	1	1	N-Term(iTRAQ4plex); K11(iTRAQ4plex)	HCD	1.0000	0.0000	1	1	8838	Unique	Used	2.813	4.989E3	1.530E4	251	4.00	0.00	0	6	30	3	456.26984	1366.79496	1.20	32.05	3209	3209	MS2	18/40	Glioma_Nuclear_prep_Gradeall_repeat_4plex.raw
56676330	High		sSAVDPEPQVk	Unambiguous	1	1	N-Term(iTRAQ4plex); K11(iTRAQ4plex)	HCD	1.0000	0.0000	1	1	7567	Unique	Used	1.431	1.771E5	2.780E5	191	4.17	0.00	0	12	6	2	722.89648	1444.78569	-2.03	23.30	1984	1984	MS2	19/20	Glioma_Nuclear_prep_Gradeall_4plex.raw
56676330	High		gASGSFVVVQk	Unambiguous	1	1	N-Term(iTRAQ4plex); K11(iTRAQ4plex)	HCD	1.0000	0.0000	1	1	8383	Unique	Used	2.400	4.358E4	1.148E5	311	4.41	0.00	0	20	10	2	683.88916	1366.77104	-16.30	29.07	2868	2868	MS2	20/20	Glioma_Nuclear_prep_Gradeall_4plex.raw
56676330		High	aLPLIVGAQLIHADk	Unambiguous	1	1	N-Term(iTRAQ4plex); K15(iTRAQ4plex)	HCD	1.0000	0.0000	1	1	12247	Unique	Used	1.712	5.121E2	9.557E2	37	7.57	0.00	0	26	300	3	616.38354	1847.13608	0.18	77.41	7197	7197	MS2	39/56	Glioma_Nuclear_prep_Gradeall_repeat_4plex.raw
56676330		High	aLPLIVGAQLIHADk	Unambiguous	1	1	N-Term(iTRAQ4plex); K15(iTRAQ4plex)	HCD	1.0000	0.0000	1	1	12209	Unique	Used	1.802	1.646E3	3.232E3	34	7.28	0.00	0	8	259	3	616.38416	1847.13791	1.17	76.37	7119	7119	MS2	39/56	Glioma_Nuclear_prep_Gradeall_repeat_4plex.raw
56676330	High		aLPLIVGAQLIHADk	Unambiguous	1	1	N-Term(iTRAQ4plex); K15(iTRAQ4plex)	HCD	1.0000	0.0000	1	1	11303	Unique	Used	1.366	6.843E4	1.026E5	45	7.29	0.00	0	16	7	3	616.38104	1847.12857	-3.88	50.90	6052	6052	MS2	37/56	Glioma_Nuclear_prep_Gradeall_4plex.raw
56676330	High		aLPLIVGAQLIHADk	Unambiguous	1	1	N-Term(iTRAQ4plex); K15(iTRAQ4plex)	HCD	1.0000	0.0000	1	1	11386	Unique	Used	1.459	1.494E4	2.392E4	38	7.12	0.00	0	47	25	3	616.38300	1847.13443	-0.71	51.62	6143	6143	MS2	39/56	Glioma_Nuclear_prep_Gradeall_4plex.raw
56676330	High		aLPLIVGAQLIHADk	Unambiguous	1	1	N-Term(iTRAQ4plex); K15(iTRAQ4plex)	HCD	1.0000	0.0000	1	1	11284	Unique	Used	1.461	1.953E4	3.132E4	37	6.37	0.00	0	31	97	2	924.07147	1847.13567	-0.04	50.73	6031	6031	MS2	26/28	Glioma_Nuclear_prep_Gradeall_4plex.raw
56676330	High		yVLENHPGTNSNYQMHLLk	Unambiguous	1	1	N-Term(iTRAQ4plex); K19(iTRAQ4plex)	HCD	1.0000	0.0000	1	1	9334	Unique	Used	2.251	1.513E4	3.736E4	135	6.53	0.00	0	4	8	3	849.42743	2546.26773	-15.20	36.03	3906	3906	MS2	29/72	Glioma_Nuclear_prep_Gradeall_4plex.raw
56676330		High	yVSQYYPk	Unambiguous	1	1	N-Term(iTRAQ4plex); K8(iTRAQ4plex)	HCD	1.0000	0.0000	1	1	9046	Unique	Used	1.619	1.107E4	1.953E4	265	3.40	0.00	0	54	300	2	668.36646	1335.72563	5.19	34.26	3431	3431	MS2	13/14	Glioma_Nuclear_prep_Gradeall_repeat_4plex.raw
56676330		High	gEPENEEkEENk	Unambiguous	1	1	N-Term(iTRAQ4plex); K8(iTRAQ4plex); K12(iTRAQ4plex)	HCD	1.0000	0.0000	1	1	6726	Unique	Used	1.391	1.838E3	2.786E3	129	4.00	0.00	1	5	299	2	932.47113	1863.93498	2.85	12.34	979	979	MS2	21/22	Glioma_Nuclear_prep_Gradeall_repeat_4plex.raw
56676330		High	gQLEQITGk	Unambiguous	1	1	N-Term(iTRAQ4plex); K9(iTRAQ4plex)	HCD	1.0000	0.0000	1	1	8525	Unique	Used	1.652	1.006E5	1.811E5	249	3.69	0.00	0	8	15	2	631.36591	1261.72453	-8.66	28.90	2880	2880	MS2	16/16	Glioma_Nuclear_prep_Gradeall_repeat_4plex.raw
56676330		High	gASGTFQLk	Unambiguous	1	1	N-Term(iTRAQ4plex); K9(iTRAQ4plex)	HCD	1.0000	0.0000	1	1	8486	Unique	Used	1.727	4.733E4	8.911E4	233	3.76	0.00	0	15	47	2	598.84753	1196.68779	0.01	28.47	2838	2838	MS2	16/16	Glioma_Nuclear_prep_Gradeall_repeat_4plex.raw
56676330	High		gQLEQITGk	Unambiguous	1	1	N-Term(iTRAQ4plex); K9(iTRAQ4plex)	HCD	1.0000	0.0000	1	1	8214	Unique	Used	1.859	4.041E5	8.243E5	225	3.11	0.00	0	9	3	2	631.36017	1261.71306	-17.75	27.88	2685	2685	MS2	16/16	Glioma_Nuclear_prep_Gradeall_4plex.raw
56676330		High	mDAILTEAlk	Unambiguous	1	1	N-Term(iTRAQ4plex); M1(Oxidation); K10(iTRAQ4plex)	HCD	1.0000	0.0000	1	1	9909	Unique	Used	2.265	4.733E3	1.169E4	254	4.40	0.00	0	21	300	2	704.90326	1408.79924	2.28	43.90	4351	4351	MS2	18/18	Glioma_Nuclear_prep_Gradeall_repeat_4plex.raw
56676330	High		mDAILTEAlk	Unambiguous	1	1	N-Term(iTRAQ4plex); M1(Oxidation); K10(iTRAQ4plex)	HCD	1.0000	0.0000	1	1	9353	Unique	Used	1.819	1.492E5	2.978E5	253	3.79	0.00	0	17	6	2	704.89014	1408.77300	-16.35	36.17	3927	3927	MS2	16/18	Glioma_Nuclear_prep_Gradeall_4plex.raw
56699409	NP_002130.2	RBMX	27316	RNA-binding motif protein, X chromosome isoform 1 [Homo sapiens]	22.51	4	3	13	0.519	5;8	16.6	86.27	391	42.3	10.05																			
56699409		High	vEQATkPSFESGR	Unambiguous	1	1	N-Term(iTRAQ4plex); K6(iTRAQ4plex)	HCD	1.0000	0.0000	1	1	7861	Unique	Used	0.410	4.195E4	1.875E4	172	4.13	0.00	0	21	48	2	862.46301	1723.91875	-1.76	22.48	2180	2180	MS2	19/24	Glioma_Nuclear_prep_Gradeall_repeat_4plex.raw
56699409	High		vEQATkPSFESGR	Unambiguous	1	1	N-Term(iTRAQ4plex); K6(iTRAQ4plex)	HCD	1.0000	0.0000	1	1	7714	Unique	Used	0.437	3.331E5	1.596E5	149	4.58	0.00	0	8	5	2	862.46582	1723.92436	1.50	24.34	2143	2143	MS2	19/24	Glioma_Nuclear_prep_Gradeall_4plex.raw
56699409		High	iVEVLLMk	Unambiguous	3	1	N-Term(iTRAQ4plex); K8(iTRAQ4plex)	HCD	1.0000	0.0000	1	1	12048	Unique	Used	0.385	9.314E3	3.905E3	93	3.63	0.00	0	54	300	2	616.89868	1232.79009	0.81	72.88	6848	6848	MS2	13/14	Glioma_Nuclear_prep_Gradeall_repeat_4plex.raw
56699409		High	iVEVLLMk	Unambiguous	3	1	N-Term(iTRAQ4plex); K8(iTRAQ4plex)	HCD	1.0000	0.0000	1	1	12079	Unique	Used	0.448	1.210E4	5.909E3	92	3.60	0.00	0	30	300	2	616.89844	1232.78960	0.41	73.88	6921	6921	MS2	13/14	Glioma_Nuclear_prep_Gradeall_repeat_4plex.raw
56699409		High	iVEVLLMk	Unambiguous	3	1	N-Term(iTRAQ4plex); K8(iTRAQ4plex)	HCD	1.0000	0.0000	1	1	12144	Unique	Used	0.535	4.155E3	2.425E3	102	3.27	0.00	0	63	300	2	616.89777	1232.78826	-0.67	75.02	7013	7013	MS2	13/14	Glioma_Nuclear_prep_Gradeall_repeat_4plex.raw
56699409		High	aLEAVFGk	Unambiguous	2	1	N-Term(iTRAQ4plex); K8(iTRAQ4plex)	HCD	1.0000	0.0000	1	1	10349	Unique	Used	0.575	2.616E4	1.639E4	212	3.63	0.00	0	18	191	2	561.84314	1122.67900	2.53	49.25	4828	4828	MS2	14/14	Glioma_Nuclear_prep_Gradeall_repeat_4plex.raw
56699409	High		aLEAVFGk	Unambiguous	2	1	N-Term(iTRAQ4plex); K8(iTRAQ4plex)	HCD	1.0000	0.0000	1	1	9651	Unique	Used	0.516	3.596E5	2.036E5	267	3.12	0.00	0	60	6	2	561.83270	1122.65813	-16.06	38.34	4251	4251	MS2	13/14	Glioma_Nuclear_prep_Gradeall_4plex.raw
56699409	High		iVEVLLMk	Unambiguous	3	1	N-Term(iTRAQ4plex); K8(iTRAQ4plex)	HCD	1.0000	0.0000	1	1	11178	Unique	Used	0.519	4.873E5	2.775E5	94	3.60	0.00	0	22	10	2	616.89667	1232.78606	-2.46	49.86	5916	5916	MS2	13/14	Glioma_Nuclear_prep_Gradeall_4plex.raw
56699409		High	iVEVLLmk	Unambiguous	3	1	N-Term(iTRAQ4plex); M7(Oxidation); K8(iTRAQ4plex)	HCD	1.0000	0.0000	1	1	11572	Unique	Used	0.478	2.819E4	1.469E4	191	3.46	0.00	0	12	142	2	624.89746	1248.78765	2.92	65.18	6217	6217	MS2	13/14	Glioma_Nuclear_prep_Gradeall_repeat_4plex.raw
56699409		High	iVEVLLmk	Unambiguous	3	1	N-Term(iTRAQ4plex); M7(Oxidation); K8(iTRAQ4plex)	HCD	1.0000	0.0000	1	1	11504	Unique	Used	0.605	7.695E3	5.072E3	197	3.54	0.00	0	50	300	2	624.89648	1248.78569	1.35	64.19	6133	6133	MS2	13/14	Glioma_Nuclear_prep_Gradeall_repeat_4plex.raw
56699409		High	iVEVLLmk	Unambiguous	3	1	N-Term(iTRAQ4plex); M7(Oxidation); K8(iTRAQ4plex)	HCD	1.0000	0.0000	1	1	11700	Unique	Used	0.656	4.190E3	2.994E3	191	3.33	0.00	0	47	300	2	624.89703	1248.78679	2.23	66.98	6369	6369	MS2	13/14	Glioma_Nuclear_prep_Gradeall_repeat_4plex.raw
56699409	High		iVEVLLmk	Unambiguous	3	1	N-Term(iTRAQ4plex); M7(Oxidation); K8(iTRAQ4plex)	HCD	1.0000	0.0000	1	1	10685	Unique	Used	0.520	8.019E5	4.575E5	232	3.01	0.00	0	43	4	2	624.88745	1248.76763	-13.11	46.05	5379	5379	MS2	13/14	Glioma_Nuclear_prep_Gradeall_4plex.raw
56699409	High		iVEVLLmk	Unambiguous	3	1	N-Term(iTRAQ4plex); M7(Oxidation); K8(iTRAQ4plex)	HCD	1.0000	0.0000	1	1	10598	Unique	Used	0.644	1.304E5	9.210E4	265	2.99	0.00	0	45	21	2	624.88641	1248.76555	-14.78	45.37	5283	5283	MS2	13/14	Glioma_Nuclear_prep_Gradeall_4plex.raw
62414289	NP_003371.2	VIM	7431	vimentin [Homo sapiens]	29.40	2	9	14	2.007	10;4	28.4	44.10	466	53.6	5.12																			
62414289		High	fADLSEAANR	Unambiguous	1	1	N-Term(iTRAQ4plex)	HCD	1.0000	0.0000	1	1	9085	Unique	Used	1.820	4.985E3	9.887E3	246	3.36	0.00	0	43	300	2	619.31927	1237.63127	1.53	34.70	3473	3473	MS2	16/18	Glioma_Nuclear_prep_Gradeall_repeat_4plex.raw
62414289		High	vELQELNDR	Unambiguous	2	1	N-Term(iTRAQ4plex)	HCD	1.0000	0.0000	1	1	9214	Unique	Used	2.188	5.458E3	1.301E4	250	3.56	0.00	0	17	232	2	630.33990	1259.67253	1.02	36.11	3609	3609	MS2	15/16	Glioma_Nuclear_prep_Gradeall_repeat_4plex.raw
62414289		High	eEAENTLQSFR	Unambiguous	1	1	N-Term(iTRAQ4plex)	HCD	1.0000	0.0000	1	1	9221	Unique	Used	2.312	2.889E3	7.281E3	150	4.36	0.00	0	0	300	2	734.36353	1467.71977	0.08	36.19	3616	3616	MS2	19/20	Glioma_Nuclear_prep_Gradeall_repeat_4plex.raw
62414289	High		eEAENTLQSFR	Unambiguous	1	1	N-Term(iTRAQ4plex)	HCD	1.0000	0.0000	1	1	8800	Unique	Used	1.962	3.024E4	6.512E4	112	3.57	0.00	0	28	33	2	734.35138	1467.69548	-16.47	32.09	3322	3322	MS2	18/20	Glioma_Nuclear_prep_Gradeall_4plex.raw
62414289	High		fADLSEAANR	Unambiguous	1	1	N-Term(iTRAQ4plex)	HCD	1.0000	0.0000	1	1	8713	Unique	Used	2.006	7.018E4	1.545E5	158	3.06	0.00	0	35	23	2	619.30750	1237.60771	-17.51	31.46	3227	3227	MS2	17/18	Glioma_Nuclear_prep_Gradeall_4plex.raw
62414289	High		iSLPLPNFSSLNLR	Unambiguous	1	1	N-Term(iTRAQ4plex)	HCD	1.0000	0.0000	1	1	12623	Unique	Used	3.249	3.376E3	1.203E4	169	4.19	0.00	0	39	300	2	858.00201	1714.99675	-0.31	64.03	7511	7511	MS2	17/26	Glioma_Nuclear_prep_Gradeall_4plex.raw
62414289	High		qVQSLTcEVDALk	Unambiguous	1	1	N-Term(iTRAQ4plex); C7(Methylthio); K13(iTRAQ4plex)	HCD	1.0000	0.0000	1	1	10588	Unique	Used	2.007	1.914E4	4.216E4	207	5.54	0.00	0	23	60	2	884.45343	1767.89958	-12.89	45.29	5272	5272	MS2	23/24	Glioma_Nuclear_prep_Gradeall_4plex.raw
62414289	High		kVESLQEEIAFLk	Unambiguous	1	1	N-Term(iTRAQ4plex); K1(iTRAQ4plex); K13(iTRAQ4plex)	HCD	1.0000	0.0000	1	1	10707	Unique	Used	2.435	1.878E4	5.019E4	123	5.65	0.00	1	23	38	2	983.56842	1966.12956	-14.74	46.20	5402	5402	MS2	23/24	Glioma_Nuclear_prep_Gradeall_4plex.raw
62414289	High		kVESLQEEIAFLk	Unambiguous	1	1	N-Term(iTRAQ4plex); K1(iTRAQ4plex); K13(iTRAQ4plex)	HCD	1.0000	0.0000	1	1	10677	Unique	Used	2.526	4.392E3	1.217E4	111	5.38	0.00	1	31	24	3	656.04803	1966.12955	-14.75	45.98	5370	5370	MS2	24/48	Glioma_Nuclear_prep_Gradeall_4plex.raw
62414289	High		iLLAELEQLk	Unambiguous	1	1	N-Term(iTRAQ4plex); K10(iTRAQ4plex)	HCD	1.0000	0.0000	1	1	12402	Unique	Used	1.708	1.546E4	2.897E4	74	4.12	0.00	0	49	270	2	729.46106	1457.91484	-2.29	61.16	7254	7254	MS2	17/18	Glioma_Nuclear_prep_Gradeall_4plex.raw
62414289	High		iLLAELEQLk	Unambiguous	1	1	N-Term(iTRAQ4plex); K10(iTRAQ4plex)	HCD	1.0000	0.0000	1	1	12401	Unique	Used	3.206	1.100E3	3.868E3	64	4.13	0.00	0	21	149	3	486.64212	1457.91181	-4.38	61.15	7253	7253	MS2	21/36	Glioma_Nuclear_prep_Gradeall_4plex.raw
62414289		High	eTNLDSLPLVDTHSk	Unambiguous	1	1	N-Term(iTRAQ4plex); K15(iTRAQ4plex)	HCD	1.0000	0.0000	1	1	10060	Unique	Used	1.424	5.572E2	8.645E2	151	5.40	0.00	0	26	300	3	653.02161	1957.05027	1.11	45.80	4518	4518	MS2	26/56	Glioma_Nuclear_prep_Gradeall_repeat_4plex.raw
62414289	High		eTNLDSLPLVDTHSk	Unambiguous	1	1	N-Term(iTRAQ4plex); K15(iTRAQ4plex)	HCD	1.0000	0.0000	1	1	9400	Unique	Used	1.326	1.542E4	2.243E4	145	5.51	0.00	0	18	7	3	653.01001	1957.01548	-16.67	36.51	3978	3978	MS2	27/56	Glioma_Nuclear_prep_Gradeall_4plex.raw
62414289	High		fANYIDk	Unambiguous	1	1	N-Term(iTRAQ4plex); K7(iTRAQ4plex)	HCD	1.0000	0.0000	1	1	8924	Unique	Used	1.419	1.108E5	1.725E5	193	2.97	0.00	0	48	14	2	579.81281	1158.61833	-18.48	32.97	3456	3456	MS2	12/12	Glioma_Nuclear_prep_Gradeall_4plex.raw
62750354	NP_954659.1	MATR3	9782	matrin-3 isoform a [Homo sapiens]	23.49	3	13	35	0.400	20;15	35.3	126.57	847	94.6	6.25																			
62750354		High	tEEGPTLSYGR	Unambiguous	1	1	N-Term(iTRAQ4plex)	HCD	1.0000	0.0000	1	1	8234	Unique	Used	0.256	4.768E4	1.331E4	153	3.36	0.00	0	12	103	2	677.34473	1353.68218	4.04	26.07	2573	2573	MS2	16/20	Glioma_Nuclear_prep_Gradeall_repeat_4plex.raw
62750354		High	gDADQASNILASFGLSAR	Unambiguous	1	1	N-Term(iTRAQ4plex)	HCD	1.0000	0.0000	1	1	12393	Unique	Used	0.392	9.668E2	4.127E2	144	4.95	0.00	0	12	300	2	968.99695	1936.98662	1.06	83.02	7597	7597	MS2	26/34	Glioma_Nuclear_prep_Gradeall_repeat_4plex.raw
62750354		High	gPLPLSSQHR	Unambiguous	1	1	N-Term(iTRAQ4plex)	HCD	1.0000	0.0000	1	1	7537	Unique	Used	0.399	3.377E4	1.469E4	330	3.40	0.00	0	21	146	2	618.34619	1235.68511	-10.21	19.41	1834	1834	MS2	15/18	Glioma_Nuclear_prep_Gradeall_repeat_4plex.raw
62750354		High	gPSLNPVLDYDHGSR	Unambiguous	1	1	N-Term(iTRAQ4plex)	HCD	1.0000	0.0000	1	1	9729	Unique	Used	0.454	7.678E3	3.798E3	110	4.99	0.00	0	38	300	2	885.95282	1770.89836	5.18	41.85	4160	4160	MS2	20/28	Glioma_Nuclear_prep_Gradeall_repeat_4plex.raw
62750354		High	rTEEGPTLSYGR	Unambiguous	1	1	N-Term(iTRAQ4plex)	HCD	1.0000	0.0000	1	1	7705	Unique	Used	0.495	3.286E3	1.773E3	175	3.32	0.00	1	0	300	2	755.38690	1509.76653	-7.49	20.98	2015	2015	MS2	17/22	Glioma_Nuclear_prep_Gradeall_repeat_4plex.raw
62750354		High	vVHIMDFQR	Unambiguous	3	1	N-Term(iTRAQ4plex)	HCD	1.0000	0.0000	1	1	9705	Unique	Used	0.518	4.724E3	2.666E3	158	3.93	0.00	0	20	269	3	430.23706	1288.69663	1.04	41.59	4135	4135	MS2	19/32	Glioma_Nuclear_prep_Gradeall_repeat_4plex.raw
62750354		High	gPLPLSSQHR	Unambiguous	1	1	N-Term(iTRAQ4plex)	HCD	1.0000	0.0000	1	1	7557	Unique	Used	0.602	1.169E4	7.674E3	212	4.36	0.00	0	29	67	3	412.57187	1235.70105	2.69	19.58	1855	1855	MS2	19/36	Glioma_Nuclear_prep_Gradeall_repeat_4plex.raw
62750354		High	gPSLNPVLDYDHGSR	Unambiguous	1	1	N-Term(iTRAQ4plex)	HCD	1.0000	0.0000	1	1	9722	Unique	Used	0.686	4.466E3	3.338E3	101	5.51	0.00	0	33	157	3	590.96924	1770.89316	2.25	41.77	4153	4153	MS2	23/56	Glioma_Nuclear_prep_Gradeall_repeat_4plex.raw
62750354	High		gDADQASNILASFGLSAR	Unambiguous	1	1	N-Term(iTRAQ4plex)	HCD	1.0000	0.0000	1	1	12207	Unique	Used	0.300	6.112E4	2.015E4	149	6.31	0.00	0	6	35	2	969.00220	1936.99712	6.48	59.07	7040	7040	MS2	27/34	Glioma_Nuclear_prep_Gradeall_4plex.raw
62750354	High		tEEGPTLSYGR	Unambiguous	1	1	N-Term(iTRAQ4plex)	HCD	1.0000	0.0000	1	1	8049	Unique	Used	0.302	2.585E5	8.576E4	114	3.26	0.00	0	27	12	2	677.33185	1353.65642	-14.99	26.71	2506	2506	MS2	17/20	Glioma_Nuclear_prep_Gradeall_4plex.raw
62750354	High		gDADQASNILASFGLSAR	Unambiguous	1	1	N-Term(iTRAQ4plex)	HCD	1.0000	0.0000	1	1	12139	Unique	Used	0.348	5.401E4	2.061E4	156	6.49	0.00	0	17	40	2	968.99481	1936.98235	-1.15	58.39	6965	6965	MS2	27/34	Glioma_Nuclear_prep_Gradeall_4plex.raw
62750354	High		gPSLNPVLDYDHGSR	Unambiguous	1	1	N-Term(iTRAQ4plex)	HCD	1.0000	0.0000	1	1	9117	Unique	Used	0.528	1.315E5	7.618E4	92	4.47	0.00	0	11	17	2	885.93506	1770.86284	-14.88	34.42	3668	3668	MS2	22/28	Glioma_Nuclear_prep_Gradeall_4plex.raw
62750354	High		gPSLNPVLDYDHGSR	Unambiguous	1	1	N-Term(iTRAQ4plex)	HCD	1.0000	0.0000	1	1	9104	Unique	Used	0.540	2.877E4	1.704E4	89	4.64	0.00	0	12	7	3	590.95776	1770.85874	-17.19	34.32	3654	3654	MS2	25/56	Glioma_Nuclear_prep_Gradeall_4plex.raw
62750354	High		gDADQASNILASFGLSAR	Unambiguous	1	1	N-Term(iTRAQ4plex)	HCD	1.0000	0.0000	1	1	12151	Unique	Used	0.543	8.645E3	5.149E3	169	5.64	0.00	0	32	34	3	646.33496	1936.99033	2.97	58.49	6978	6978	MS2	25/68	Glioma_Nuclear_prep_Gradeall_4plex.raw
62750354		High	lcSLFYTNEEVAk	Unambiguous	3	1	N-Term(iTRAQ4plex); C2(Methylthio); K13(iTRAQ4plex)	HCD	1.0000	0.0000	1	1	12360	Unique	Used	0.230	2.324E3	5.824E2	153	4.83	0.00	0	4	300	2	925.97003	1850.93279	3.07	80.88	7451	7451	MS2	22/24	Glioma_Nuclear_prep_Gradeall_repeat_4plex.raw
62750354		High	lcSLFYTNEEVAk	Unambiguous	3	1	N-Term(iTRAQ4plex); C2(Methylthio); K13(iTRAQ4plex)	HCD	1.0000	0.0000	1	1	12355	Unique	Used	0.744	2.720E2	2.207E2	136	3.54	0.00	0	17	300	3	617.64844	1850.93076	1.97	80.78	7443	7443	MS2	19/48	Glioma_Nuclear_prep_Gradeall_repeat_4plex.raw
62750354	High		lcSLFYTNEEVAk	Unambiguous	3	1	N-Term(iTRAQ4plex); C2(Methylthio); K13(iTRAQ4plex)	HCD	1.0000	0.0000	1	1	11923	Unique	Used	0.297	1.616E4	5.273E3	165	4.62	0.00	0	22	15	3	617.65088	1850.93808	5.93	56.25	6728	6728	MS2	21/48	Glioma_Nuclear_prep_Gradeall_4plex.raw
62750354	High		lcSLFYTNEEVAk	Unambiguous	3	1	N-Term(iTRAQ4plex); C2(Methylthio); K13(iTRAQ4plex)	HCD	1.0000	0.0000	1	1	11880	Unique	Used	0.310	8.423E4	2.866E4	207	5.10	0.00	0	9	38	2	925.97003	1850.93279	3.07	55.89	6681	6681	MS2	21/24	Glioma_Nuclear_prep_Gradeall_4plex.raw
62750354	High		lcSLFYTNEEVAk	Unambiguous	3	1	N-Term(iTRAQ4plex); C2(Methylthio); K13(iTRAQ4plex)	HCD	1.0000	0.0000	1	1	11962	Unique	Used	0.331	4.576E4	1.663E4	163	5.69	0.00	0	26	106	2	925.96802	1850.92876	0.89	56.63	6771	6771	MS2	21/24	Glioma_Nuclear_prep_Gradeall_4plex.raw
62750354	High		nTHcSSLPHYQk	Unambiguous	3	1	N-Term(iTRAQ4plex); C4(Methylthio); K12(iTRAQ4plex)	HCD	1.0000	0.0000	1	1	7725	Unique	Used	0.212	5.466E4	1.274E4	66	3.50	0.00	0	43	3	4	437.96671	1748.84499	-0.05	24.42	2155	2155	MS2	25/66	Glioma_Nuclear_prep_Gradeall_4plex.raw
62750354	High		gYPHLcSlcDLPVHSNk	Unambiguous	1	1	N-Term(iTRAQ4plex); C6(Methylthio); C9(Methylthio); K17(iTRAQ4plex)	HCD	1.0000	0.0000	1	1	11152	Unique	Used	0.548	3.220E3	1.935E3	52	4.93	0.00	0	48	21	4	566.52124	2263.06313	-4.57	49.65	5887	5887	MS2	31/96	Glioma_Nuclear_prep_Gradeall_4plex.raw
62750354	High		iTPENLPQILLQLk	Unambiguous	1	1	N-Term(iTRAQ4plex); K14(iTRAQ4plex)	HCD	1.0000	0.0000	1	1	13024	Unique	Used	0.345	6.607E3	2.501E3	28	5.05	0.00	0	18	300	2	954.59485	1908.18242	2.70	70.38	8030	8030	MS2	22/26	Glioma_Nuclear_prep_Gradeall_4plex.raw
62750354	High		iTPENLPQILLQLk	Unambiguous	1	1	N-Term(iTRAQ4plex); K14(iTRAQ4plex)	HCD	1.0000	0.0000	1	1	13021	Unique	Used	0.347	3.361E3	1.281E3	28	5.65	0.00	0	8	65	3	636.73273	1908.18363	3.33	70.30	8024	8024	MS2	24/52	Glioma_Nuclear_prep_Gradeall_4plex.raw
62750354	High		iTPENLPQILLQLk	Unambiguous	1	1	N-Term(iTRAQ4plex); K14(iTRAQ4plex)	HCD	1.0000	0.0000	1	1	12989	Unique	Used	0.348	1.076E4	4.109E3	29	5.56	0.00	0	23	300	2	954.59357	1908.17986	1.36	69.71	7978	7978	MS2	21/26	Glioma_Nuclear_prep_Gradeall_4plex.raw
62750354	High		iTPENLPQILLQLk	Unambiguous	1	1	N-Term(iTRAQ4plex); K14(iTRAQ4plex)	HCD	1.0000	0.0000	1	1	12983	Unique	Used	0.505	9.530E2	5.277E2	27	5.62	0.00	0	48	300	3	636.73187	1908.18106	1.99	69.63	7971	7971	MS2	24/52	Glioma_Nuclear_prep_Gradeall_4plex.raw
62750354		High	gAPPSSNIEDFHGLLPk	Unambiguous	1	1	N-Term(iTRAQ4plex); K17(iTRAQ4plex)	HCD	1.0000	0.0000	1	1	10918	Unique	Used	0.395	2.030E3	8.731E2	177	5.56	0.00	0	0	300	2	1034.06226	2067.11724	2.83	56.16	5443	5443	MS2	23/32	Glioma_Nuclear_prep_Gradeall_repeat_4plex.raw
62750354		High	gAPPSSNIEDFHGLLPk	Unambiguous	1	1	N-Term(iTRAQ4plex); K17(iTRAQ4plex)	HCD	1.0000	0.0000	1	1	10852	Unique	Used	0.479	1.148E3	5.992E2	133	4.50	0.00	0	43	300	3	689.70953	2067.11405	1.29	55.33	5370	5370	MS2	23/64	Glioma_Nuclear_prep_Gradeall_repeat_4plex.raw
62750354		High	gAPPSSNIEDFHGLLPk	Unambiguous	1	1	N-Term(iTRAQ4plex); K17(iTRAQ4plex)	HCD	1.0000	0.0000	1	1	10954	Unique	Used	0.594	2.821E3	1.826E3	136	4.68	0.00	0	6	119	3	689.71002	2067.11551	2.00	56.59	5482	5482	MS2	28/64	Glioma_Nuclear_prep_Gradeall_repeat_4plex.raw
62750354	High		gAPPSSNIEDFHGLLPk	Unambiguous	1	1	N-Term(iTRAQ4plex); K17(iTRAQ4plex)	HCD	1.0000	0.0000	1	1	9982	Unique	Used	0.513	6.898E4	3.884E4	179	4.63	0.00	0	23	20	2	1034.04333	2067.07939	-15.48	40.72	4610	4610	MS2	24/32	Glioma_Nuclear_prep_Gradeall_4plex.raw
62750354	High		gAPPSSNIEDFHGLLPk	Unambiguous	1	1	N-Term(iTRAQ4plex); K17(iTRAQ4plex)	HCD	1.0000	0.0000	1	1	10052	Unique	Used	0.584	6.404E4	4.106E4	114	4.09	0.00	0	7	4	3	689.69934	2067.08347	-13.51	41.23	4686	4686	MS2	26/64	Glioma_Nuclear_prep_Gradeall_4plex.raw
62750354		High	iGPYQPNVPVGIDYVIPk	Unambiguous	3	1	N-Term(iTRAQ4plex); K18(iTRAQ4plex)	HCD	1.0000	0.0000	1	1	12161	Unique	Used	0.097	7.368E2	7.779E1	71	4.17	0.00	0	2	300	2	1129.14612	2257.28496	0.63	75.27	7034	7034	MS2	18/34	Glioma_Nuclear_prep_Gradeall_repeat_4plex.raw
62750354		High	iGPYQPNVPVGIDYVIPk	Unambiguous	3	1	N-Term(iTRAQ4plex); K18(iTRAQ4plex)	HCD	1.0000	0.0000	1	1	12154	Unique	Used	0.768	2.474E2	2.072E2	106	5.07	0.00	0	26	271	3	753.10120	2257.28904	2.44	75.16	7025	7025	MS2	20/68	Glioma_Nuclear_prep_Gradeall_repeat_4plex.raw
62750354	High		iGPYQPNVPVGIDYVIPk	Unambiguous	3	1	N-Term(iTRAQ4plex); K18(iTRAQ4plex)	HCD	1.0000	0.0000	1	1	11508	Unique	Used	0.283	6.943E4	2.155E4	91	5.95	0.00	0	3	17	2	1129.14001	2257.27275	-4.77	52.67	6277	6277	MS2	22/34	Glioma_Nuclear_prep_Gradeall_4plex.raw
62750354	High		iGPYQPNVPVGIDYVIPk	Unambiguous	3	1	N-Term(iTRAQ4plex); K18(iTRAQ4plex)	HCD	1.0000	0.0000	1	1	11498	Unique	Used	0.400	1.026E4	4.505E3	104	4.44	0.00	0	27	13	3	753.09662	2257.27530	-3.64	52.59	6266	6266	MS2	19/68	Glioma_Nuclear_prep_Gradeall_4plex.raw
62750354	High		vIHLSNLPHSGYSDSAVLk	Unambiguous	3	1	N-Term(iTRAQ4plex); K19(iTRAQ4plex)	HCD	1.0000	0.0000	1	1	9461	Unique	Used	0.512	3.877E4	2.180E4	143	6.44	0.00	0	13	4	3	775.75354	2325.24607	-14.83	36.97	4045	4045	MS2	36/72	Glioma_Nuclear_prep_Gradeall_4plex.raw
66346730	NP_001015001.1	CKMT1A	548596	creatine kinase U-type, mitochondrial precursor [Homo sapiens]	18.71	2	5	7	0.503	4;3	22.3	25.84	417	47.0	8.34																			
66346730		High	lYPPSAEYPDLR	Unambiguous	1	1	N-Term(iTRAQ4plex)	HCD	1.0000	0.0000	1	1	10599	Unique	Used	0.454	1.135E4	5.613E3	259	3.14	0.00	0	28	300	2	782.91235	1564.81743	2.95	52.21	5095	5095	MS2	15/22	Glioma_Nuclear_prep_Gradeall_repeat_4plex.raw
66346730	High		gTGGVDTAATGGVFDISNLDR	Unambiguous	1	1	N-Term(iTRAQ4plex)	HCD	1.0000	0.0000	1	1	10864	Unique	Used	0.462	4.993E4	2.529E4	139	7.51	0.00	0	12	33	2	1084.02380	2167.04033	-15.94	47.40	5573	5573	MS2	31/40	Glioma_Nuclear_prep_Gradeall_4plex.raw
66346730	High		gTGGVDTAATGGVFDISNLDR	Unambiguous	1	1	N-Term(iTRAQ4plex)	HCD	1.0000	0.0000	1	1	10872	Unique	Used	0.503	2.446E4	1.349E4	116	5.41	0.00	0	38	18	3	723.02075	2167.04770	-12.53	47.47	5582	5582	MS2	25/80	Glioma_Nuclear_prep_Gradeall_4plex.raw
66346730	High		lGYILTcPSNLGTGLR	Unambiguous	2	1	N-Term(iTRAQ4plex); C7(Methylthio)	HCD	1.0000	0.0000	1	1	12355	Unique	Used	0.462	4.118E4	2.086E4	217	4.74	0.00	0	14	137	2	934.49872	1867.99016	0.56	60.65	7203	7203	MS2	21/30	Glioma_Nuclear_prep_Gradeall_4plex.raw
66346730		High	vVVDALSGLk	Unambiguous	1	1	N-Term(iTRAQ4plex); K10(iTRAQ4plex)	HCD	1.0000	0.0000	1	1	11107	Unique	Used	0.706	1.065E4	8.198E3	128	4.30	0.00	0	47	300	2	644.90942	1288.81157	2.84	58.53	5652	5652	MS2	18/18	Glioma_Nuclear_prep_Gradeall_repeat_4plex.raw
66346730	High		vVVDALSGLk	Unambiguous	1	1	N-Term(iTRAQ4plex); K10(iTRAQ4plex)	HCD	1.0000	0.0000	1	1	10306	Unique	Used	0.753	5.423E5	4.483E5	193	3.86	0.00	0	50	8	2	644.90204	1288.79680	-8.62	43.13	4963	4963	MS2	16/18	Glioma_Nuclear_prep_Gradeall_4plex.raw
66346730		High	iPTPVIHTk	Unambiguous	1	1	N-Term(iTRAQ4plex); K9(iTRAQ4plex)	HCD	1.0000	0.0000	1	1	8696	Unique	Used	0.577	1.360E4	8.556E3	88	4.00	0.00	0	60	116	3	431.94684	1293.82596	9.78	30.60	3059	3059	MS2	17/32	Glioma_Nuclear_prep_Gradeall_repeat_4plex.raw
66529396	NP_001018171.1	PON2	5445	serum paraoxonase/arylesterase 2 isoform 2 [Homo sapiens]	14.91	2	4	10	3.150	4;6	23.7	26.20	342	38.0	5.72																			
66529396		High	lFVYDPNNPPSSEVLR	Unambiguous	2	1	N-Term(iTRAQ4plex)	HCD	1.0000	0.0000	1	1	11606	Unique	Used	1.899	3.480E2	7.202E2	204	4.08	0.00	0	31	300	3	664.35138	1991.03958	2.04	65.65	6256	6256	MS2	19/60	Glioma_Nuclear_prep_Gradeall_repeat_4plex.raw
66529396		High	lFVYDPNNPPSSEVLR	Unambiguous	2	1	N-Term(iTRAQ4plex)	HCD	1.0000	0.0000	1	1	11599	Unique	Used	3.021	9.547E2	3.144E3	198	4.47	0.00	0	11	300	2	996.02185	1991.03642	0.45	65.55	6248	6248	MS2	21/30	Glioma_Nuclear_prep_Gradeall_repeat_4plex.raw
66529396		High	lLIGTLYHR	Unambiguous	2	1	N-Term(iTRAQ4plex)	HCD	1.0000	0.0000	1	1	10463	Unique	Used	3.874	3.547E2	1.497E3	139	3.73	0.00	0	34	300	3	410.58746	1229.74784	-0.69	50.58	4948	4948	MS2	17/32	Glioma_Nuclear_prep_Gradeall_repeat_4plex.raw
66529396		High	lFVYDPNNPPSSEVLR	Unambiguous	2	1	N-Term(iTRAQ4plex)	HCD	1.0000	0.0000	1	1	11667	Unique	Used	4.133	6.836E2	3.079E3	165	4.42	0.00	0	5	300	2	996.02203	1991.03679	0.64	66.54	6331	6331	MS2	18/30	Glioma_Nuclear_prep_Gradeall_repeat_4plex.raw
66529396	High		lFVYDPNNPPSSEVLR	Unambiguous	2	1	N-Term(iTRAQ4plex)	HCD	1.0000	0.0000	1	1	10930	Unique	Used	3.061	2.667E4	8.960E4	139	4.53	0.00	0	2	18	2	996.00604	1991.00481	-15.43	47.92	5645	5645	MS2	20/30	Glioma_Nuclear_prep_Gradeall_4plex.raw
66529396	High		lFVYDPNNPPSSEVLR	Unambiguous	2	1	N-Term(iTRAQ4plex)	HCD	1.0000	0.0000	1	1	10842	Unique	Used	3.238	2.236E4	7.947E4	140	4.53	0.00	0	9	29	2	996.00720	1991.00713	-14.26	47.23	5549	5549	MS2	21/30	Glioma_Nuclear_prep_Gradeall_4plex.raw
66529396	High		lLIGTLYHR	Unambiguous	2	1	N-Term(iTRAQ4plex)	HCD	1.0000	0.0000	1	1	9636	Unique	Used	4.146	7.050E3	3.208E4	201	3.78	0.00	0	8	13	3	410.58063	1229.72733	-17.37	38.22	4234	4234	MS2	20/32	Glioma_Nuclear_prep_Gradeall_4plex.raw
66529396		High	eVESVDLPHcHLlk	Unambiguous	2	1	N-Term(iTRAQ4plex); C10(Methylthio); K14(iTRAQ4plex)	HCD	1.0000	0.0000	1	1	10863	Unique	Used	3.518	1.264E2	4.848E2	210	4.82	0.00	0	17	300	4	489.01044	1953.01992	1.15	55.46	5382	5382	MS2	33/78	Glioma_Nuclear_prep_Gradeall_repeat_4plex.raw
66529396		High	fEEAENSLLHLk	Unambiguous	2	1	N-Term(iTRAQ4plex); K12(iTRAQ4plex)	HCD	1.0000	0.0000	1	1	11397	Unique	Used	2.909	2.751E2	8.724E2	195	4.67	0.00	0	37	300	3	573.31671	1717.93558	-0.45	62.74	6008	6008	MS2	27/44	Glioma_Nuclear_prep_Gradeall_repeat_4plex.raw
66529396	High		fEEAENSLLHLk	Unambiguous	2	1	N-Term(iTRAQ4plex); K12(iTRAQ4plex)	HCD	1.0000	0.0000	1	1	10404	Unique	Used	2.182	1.325E4	3.173E4	241	4.43	0.00	0	22	8	3	573.30786	1717.90903	-15.91	43.87	5070	5070	MS2	26/44	Glioma_Nuclear_prep_Gradeall_4plex.raw
68051721	NP_065843.3	NCEH1	57552	neutral cholesterol ester hydrolase 1 isoform b [Homo sapiens]	9.09	3	3	7	0.500	5;2	32.3	26.34	440	49.0	7.93																			
68051721		High	iVQELPQLLDAR	Unambiguous	3	1	N-Term(iTRAQ4plex)	HCD	1.0000	0.0000	1	1	12148	Unique	Used	0.353	4.070E3	1.566E3	196	3.73	0.00	0	21	300	2	769.95703	1538.90679	2.90	75.08	7018	7018	MS2	18/22	Glioma_Nuclear_prep_Gradeall_repeat_4plex.raw
68051721		High	vTDTDFDGVEVR	Unambiguous	2	1	N-Term(iTRAQ4plex)	HCD	1.0000	0.0000	1	1	9845	Unique	Used	0.700	7.866E3	5.999E3	192	4.39	0.00	0	33	300	2	748.87231	1496.73735	1.58	43.15	4282	4282	MS2	18/22	Glioma_Nuclear_prep_Gradeall_repeat_4plex.raw
68051721	High		iVQELPQLLDAR	Unambiguous	3	1	N-Term(iTRAQ4plex)	HCD	1.0000	0.0000	1	1	11561	Unique	Used	0.372	4.267E4	1.741E4	223	3.76	0.00	0	33	82	2	769.95557	1538.90386	1.00	53.10	6334	6334	MS2	17/22	Glioma_Nuclear_prep_Gradeall_4plex.raw
68051721	High		iVQELPQLLDAR	Unambiguous	3	1	N-Term(iTRAQ4plex)	HCD	1.0000	0.0000	1	1	11473	Unique	Used	0.380	4.849E4	2.021E4	226	3.56	0.00	0	47	94	2	769.95526	1538.90325	0.60	52.37	6238	6238	MS2	18/22	Glioma_Nuclear_prep_Gradeall_4plex.raw
68051721	High		sAPLIADQAVLQLLPk	Unambiguous	3	1	N-Term(iTRAQ4plex); K16(iTRAQ4plex)	HCD	1.0000	0.0000	1	1	12439	Unique	Used	0.500	1.079E4	5.925E3	39	6.73	0.00	0	29	300	2	983.10126	1965.19524	-1.79	61.58	7295	7295	MS2	26/30	Glioma_Nuclear_prep_Gradeall_4plex.raw
68051721	High		sAPLIADQAVLQLLPk	Unambiguous	3	1	N-Term(iTRAQ4plex); K16(iTRAQ4plex)	HCD	1.0000	0.0000	1	1	12470	Unique	Used	0.576	2.071E3	1.309E3	28	6.04	0.00	0	25	145	3	655.73907	1965.20267	2.00	61.95	7330	7330	MS2	27/60	Glioma_Nuclear_prep_Gradeall_4plex.raw
68051721	High		sAPLIADQAVLQLLPk	Unambiguous	3	1	N-Term(iTRAQ4plex); K16(iTRAQ4plex)	HCD	1.0000	0.0000	1	1	12413	Unique	Used	0.775	1.879E3	1.598E3	28	6.26	0.00	0	49	183	3	655.73926	1965.20322	2.28	61.28	7266	7266	MS2	25/60	Glioma_Nuclear_prep_Gradeall_4plex.raw
68509932	NP_001020263.1	MBP	4155	myelin basic protein isoform 4 [Homo sapiens]	33.75	6	5	31	0.373	14;17	35.6	78.27	160	17.3	11.14																			
68509932		High	yLATASTMDHAR	Unambiguous	6	1	N-Term(iTRAQ4plex)	HCD	1.0000	0.0000	1	1	8568	Unique	Used	0.226	4.927E4	1.211E4	196	4.34	0.00	0	19	109	2	740.87335	1480.73943	3.99	29.31	2925	2925	MS2	18/22	Glioma_Nuclear_prep_Gradeall_repeat_4plex.raw
68509932		High	yLATASTMDHAR	Unambiguous	6	1	N-Term(iTRAQ4plex)	HCD	1.0000	0.0000	1	1	8534	Unique	Used	0.263	1.797E4	5.152E3	115	6.36	0.00	0	42	92	3	494.24536	1480.72153	-8.10	28.96	2889	2889	MS2	23/44	Glioma_Nuclear_prep_Gradeall_repeat_4plex.raw
68509932		High	dTGILDSIGR	Unambiguous	6	1	N-Term(iTRAQ4plex)	HCD	1.0000	0.0000	1	1	10053	Unique	Used	0.332	1.013E5	3.660E4	255	3.59	0.00	0	5	67	2	595.83032	1190.65337	3.01	45.73	4511	4511	MS2	17/18	Glioma_Nuclear_prep_Gradeall_repeat_4plex.raw
68509932		High	dTGILDSIGR	Unambiguous	6	1	N-Term(iTRAQ4plex)	HCD	1.0000	0.0000	1	1	9972	Unique	Used	0.399	2.672E4	1.162E4	286	3.70	0.00	0	10	244	2	595.83038	1190.65349	3.11	44.72	4422	4422	MS2	17/18	Glioma_Nuclear_prep_Gradeall_repeat_4plex.raw
68509932		High	yLATASTMDHAR	Unambiguous	6	1	N-Term(iTRAQ4plex)	HCD	1.0000	0.0000	1	1	8222	Unique	Used	0.548	5.407E3	3.228E3	143	4.95	0.00	0	37	130	3	494.25058	1480.73719	2.47	25.95	2560	2560	MS2	21/44	Glioma_Nuclear_prep_Gradeall_repeat_4plex.raw
68509932	High		yLATASTMDHAR	Unambiguous	6	1	N-Term(iTRAQ4plex)	HCD	1.0000	0.0000	1	1	8180	Unique	Used	0.188	1.649E5	3.401E4	93	5.92	0.00	0	24	4	3	494.24185	1480.71100	-15.21	27.64	2648	2648	MS2	22/44	Glioma_Nuclear_prep_Gradeall_4plex.raw
68509932	High		dTGILDSIGR	Unambiguous	6	1	N-Term(iTRAQ4plex)	HCD	1.0000	0.0000	1	1	9578	Unique	Used	0.374	1.103E6	4.522E5	185	3.07	0.00	0	10	2	2	595.81958	1190.63188	-15.03	37.82	4172	4172	MS2	17/18	Glioma_Nuclear_prep_Gradeall_4plex.raw
68509932	High		dTGILDSIGR	Unambiguous	6	1	N-Term(iTRAQ4plex)	HCD	1.0000	0.0000	1	1	9489	Unique	Used	0.439	2.808E5	1.353E5	220	3.25	0.00	0	45	10	2	595.82001	1190.63274	-14.32	37.17	4075	4075	MS2	15/18	Glioma_Nuclear_prep_Gradeall_4plex.raw
68509932		High	tAHYGSLPQk	Unambiguous	5	1	N-Term(iTRAQ4plex); K10(iTRAQ4plex)	HCD	1.0000	0.0000	1	1	7617	Unique	Used	0.290	1.129E5	3.565E4	224	3.74	0.00	0	35	39	2	695.39362	1389.77995	5.09	20.10	1919	1919	MS2	17/18	Glioma_Nuclear_prep_Gradeall_repeat_4plex.raw
68509932		High	tAHYGSLPQk	Unambiguous	5	1	N-Term(iTRAQ4plex); K10(iTRAQ4plex)	HCD	1.0000	0.0000	1	1	7584	Unique	Used	0.456	1.686E4	8.387E3	256	4.06	0.00	0	44	92	3	463.92877	1389.77176	-0.81	19.79	1883	1883	MS2	18/36	Glioma_Nuclear_prep_Gradeall_repeat_4plex.raw

68509932		High	tAHYGSLPQk	Unambiguous	5	1	N-Term(iTRAQ4plex); K10(iTRAQ4plex)	HCD	1.0000	0.0000	1	1	7673	Unique	Used	0.462	9.796E3	4.928E3	232	3.91	0.00	0	36	86	3	463.92545	1389.76178	-7.99	20.67	1982	1982	MS2	18/36	Glioma_Nuclear_prep_Gradeall_repeat_4plex.raw
68509932		High	gVDAQGTLSk	Unambiguous	5	1	N-Term(iTRAQ4plex); K10(iTRAQ4plex)	HCD	1.0000	0.0000	1	1	7568	Unique	Used	0.508	1.320E5	7.304E4	255	3.65	0.00	0	10	28	2	632.35974	1263.71221	-2.00	19.68	1867	1867	MS2	18/18	Glioma_Nuclear_prep_Gradeall_repeat_4plex.raw
68509932		High	gVDAQGTLSk	Unambiguous	5	1	N-Term(iTRAQ4plex); K10(iTRAQ4plex)	HCD	1.0000	0.0000	1	1	7694	Unique	Used	0.545	3.445E4	2.048E4	279	3.76	0.00	0	17	118	2	632.35486	1263.70244	-9.72	20.88	2004	2004	MS2	18/18	Glioma_Nuclear_prep_Gradeall_repeat_4plex.raw
68509932	High		tAHYGSLPQk	Unambiguous	5	1	N-Term(iTRAQ4plex); K10(iTRAQ4plex)	HCD	1.0000	0.0000	1	1	7516	Unique	Used	0.267	5.515E5	1.615E5	265	4.15	0.00	0	23	12	2	695.38824	1389.76921	-2.64	22.92	1928	1928	MS2	17/18	Glioma_Nuclear_prep_Gradeall_4plex.raw
68509932	High		gVDAQGTLSk	Unambiguous	5	1	N-Term(iTRAQ4plex); K10(iTRAQ4plex)	HCD	1.0000	0.0000	1	1	7535	Unique	Used	0.424	6.797E5	3.163E5	273	4.06	0.00	0	27	7	2	632.36218	1263.71709	1.87	23.07	1949	1949	MS2	18/18	Glioma_Nuclear_prep_Gradeall_4plex.raw
68509932	High		tAHYGSLPQk	Unambiguous	5	1	N-Term(iTRAQ4plex); K10(iTRAQ4plex)	HCD	1.0000	0.0000	1	1	7499	Unique	Used	0.481	5.979E4	3.155E4	296	3.58	0.00	0	49	8	3	463.92868	1389.77149	-1.01	22.81	1910	1910	MS2	18/36	Glioma_Nuclear_prep_Gradeall_4plex.raw
68509932	High		gVDAQGTLSk	Unambiguous	5	1	N-Term(iTRAQ4plex); K10(iTRAQ4plex)	HCD	1.0000	0.0000	1	1	7667	Unique	Used	0.758	2.235E5	1.858E5	279	3.70	0.00	0	20	10	2	632.36041	1263.71355	-0.93	24.01	2092	2092	MS2	18/18	Glioma_Nuclear_prep_Gradeall_4plex.raw
68509932		High	tQDENPVVHFFk	Unambiguous	5	1	N-Term(iTRAQ4plex); K12(iTRAQ4plex)	HCD	1.0000	0.0000	1	1	10591	Unique	Used	0.230	3.971E4	9.975E3	123	4.12	0.00	0	8	23	3	583.64740	1748.92765	3.78	52.13	5087	5087	MS2	26/44	Glioma_Nuclear_prep_Gradeall_repeat_4plex.raw
68509932		High	tQDENPVVHFFk	Unambiguous	5	1	N-Term(iTRAQ4plex); K12(iTRAQ4plex)	HCD	1.0000	0.0000	1	1	10592	Unique	Used	0.248	3.918E4	1.059E4	204	5.38	0.00	0	8	115	2	874.96832	1748.92937	4.76	52.14	5088	5088	MS2	21/22	Glioma_Nuclear_prep_Gradeall_repeat_4plex.raw
68509932		High	tQDENPVVHFFk	Unambiguous	5	1	N-Term(iTRAQ4plex); K12(iTRAQ4plex)	HCD	1.0000	0.0000	1	1	10686	Unique	Used	0.297	3.147E3	1.019E3	137	4.09	0.00	0	22	272	3	583.64502	1748.92051	-0.30	53.27	5189	5189	MS2	25/44	Glioma_Nuclear_prep_Gradeall_repeat_4plex.raw
68509932		High	tQDENPVVHFFk	Unambiguous	5	1	N-Term(iTRAQ4plex); K12(iTRAQ4plex)	HCD	1.0000	0.0000	1	1	10574	Unique	Used	0.331	1.023E3	3.692E2	170	3.65	0.00	0	27	300	4	437.98669	1748.92495	2.24	51.92	5068	5068	MS2	20/66	Glioma_Nuclear_prep_Gradeall_repeat_4plex.raw
68509932		High	tQDENPVVHFFk	Unambiguous	5	1	N-Term(iTRAQ4plex); K12(iTRAQ4plex)	HCD	1.0000	0.0000	1	1	10515	Unique	Used	0.392	7.042E3	3.007E3	206	5.28	0.00	0	29	300	2	874.96686	1748.92644	3.09	51.22	5004	5004	MS2	21/22	Glioma_Nuclear_prep_Gradeall_repeat_4plex.raw
68509932		High	tQDENPVVHFFk	Unambiguous	5	1	N-Term(iTRAQ4plex); K12(iTRAQ4plex)	HCD	1.0000	0.0000	1	1	10505	Unique	Used	0.394	2.800E3	1.203E3	139	4.13	0.00	0	35	300	3	583.64563	1748.92234	0.74	51.11	4994	4994	MS2	25/44	Glioma_Nuclear_prep_Gradeall_repeat_4plex.raw
68509932	High		tQDENPVVHFFk	Unambiguous	5	1	N-Term(iTRAQ4plex); K12(iTRAQ4plex)	HCD	1.0000	0.0000	1	1	9745	Unique	Used	0.249	5.883E5	1.609E5	176	5.16	0.00	0	6	5	2	874.95319	1748.89910	-12.55	39.01	4353	4353	MS2	21/22	Glioma_Nuclear_prep_Gradeall_4plex.raw
68509932	High		tQDENPVVHFFk	Unambiguous	5	1	N-Term(iTRAQ4plex); K12(iTRAQ4plex)	HCD	1.0000	0.0000	1	1	9721	Unique	Used	0.255	8.362E5	2.341E5	147	4.66	0.00	0	8	1	3	583.63715	1748.89688	-13.81	38.84	4327	4327	MS2	27/44	Glioma_Nuclear_prep_Gradeall_4plex.raw
68509932	High		tQDENPVVHFFk	Unambiguous	5	1	N-Term(iTRAQ4plex); K12(iTRAQ4plex)	HCD	1.0000	0.0000	1	1	9653	Unique	Used	0.306	2.330E5	7.837E4	179	5.33	0.00	0	13	14	2	874.95319	1748.89910	-12.55	38.35	4253	4253	MS2	22/22	Glioma_Nuclear_prep_Gradeall_4plex.raw
68509932	High		tQDENPVVHFFk	Unambiguous	5	1	N-Term(iTRAQ4plex); K12(iTRAQ4plex)	HCD	1.0000	0.0000	1	1	9632	Unique	Used	0.572	4.076E4	2.559E4	126	4.92	0.00	0	43	9	3	583.63696	1748.89634	-14.12	38.19	4230	4230	MS2	23/44	Glioma_Nuclear_prep_Gradeall_4plex.raw
68509932	High		tQDENPVVHFFk	Unambiguous	5	1	N-Term(iTRAQ4plex); K12(iTRAQ4plex)	HCD	1.0000	0.0000	1	1	9822	Unique	Used	0.651	3.997E4	2.855E4	111	4.44	0.00	0	42	5	3	583.63727	1748.89725	-13.60	39.56	4436	4436	MS2	24/44	Glioma_Nuclear_prep_Gradeall_4plex.raw
68509932		High	yLATASTmDHAR	Unambiguous	6	1	N-Term(iTRAQ4plex); M8(Oxidation)	HCD	1.0000	0.0000	1	1	7571	Unique	Used	0.320	3.503E4	1.220E4	118	6.36	0.00	0	20	28	3	499.57956	1496.72412	-2.88	19.70	1870	1870	MS2	23/44	Glioma_Nuclear_prep_Gradeall_repeat_4plex.raw
68509932	High		yLATASTmDHAR	Unambiguous	6	1	N-Term(iTRAQ4plex); M8(Oxidation)	HCD	1.0000	0.0000	1	1	7510	Unique	Used	0.262	1.515E5	4.354E4	114	6.01	0.00	0	33	5	3	499.57874	1496.72165	-4.53	22.89	1922	1922	MS2	23/44	Glioma_Nuclear_prep_Gradeall_4plex.raw
68509932	High		yLATASTmDHAR	Unambiguous	6	1	N-Term(iTRAQ4plex); M8(Oxidation)	HCD	1.0000	0.0000	1	1	7520	Unique	Used	0.373	3.547E5	1.453E5	181	2.91	0.00	0	20	9	2	748.86938	1496.73149	2.04	22.96	1933	1933	MS2	13/22	Glioma_Nuclear_prep_Gradeall_4plex.raw
70167113	NP_001020278.1	ADAR	103	double-stranded RNA-specific adenosine deaminase isoform d [Homo sapiens]	17.29	4	8	18	0.448	10;8	39.4	71.76	931	103.6	8.66																			
70167113		High	yLNTNPVGGLLEYAR	Unambiguous	4	1	N-Term(iTRAQ4plex)	HCD	1.0000	0.0000	1	1	12293	Unique	Used	0.193	2.782E3	5.845E2	242	4.22	0.00	0	10	300	2	912.49359	1823.97991	1.45	78.85	7302	7302	MS2	19/28	Glioma_Nuclear_prep_Gradeall_repeat_4plex.raw
70167113		High	qEAADAALR	Unambiguous	4	1	N-Term(iTRAQ4plex)	HCD	1.0000	0.0000	1	1	7197	Unique	Used	0.467	4.466E4	2.271E4	166	3.40	0.00	0	23	78	2	544.78552	1088.56377	-16.48	16.51	1477	1477	MS2	13/16	Glioma_Nuclear_prep_Gradeall_repeat_4plex.raw
70167113		High	dINAVLIDMER	Unambiguous	4	1	N-Term(iTRAQ4plex)	HCD	1.0000	0.0000	1	1	11835	Unique	Used	0.472	3.302E3	1.698E3	289	3.75	0.00	0	34	300	2	716.88452	1432.76177	2.14	69.19	6551	6551	MS2	17/20	Glioma_Nuclear_prep_Gradeall_repeat_4plex.raw
70167113	High		yLNTNPVGGLLEYAR	Unambiguous	4	1	N-Term(iTRAQ4plex)	HCD	1.0000	0.0000	1	1	11796	Unique	Used	0.288	3.810E5	1.205E5	294	4.90	0.00	0	14	11	2	912.49103	1823.97478	-1.36	55.17	6590	6590	MS2	20/28	Glioma_Nuclear_prep_Gradeall_4plex.raw
70167113	High		dINAVLIDMER	Unambiguous	4	1	N-Term(iTRAQ4plex)	HCD	1.0000	0.0000	1	1	11121	Unique	Used	0.577	1.557E5	9.857E4	264	4.44	0.00	0	40	22	2	716.88391	1432.76055	1.29	49.40	5853	5853	MS2	16/20	Glioma_Nuclear_prep_Gradeall_4plex.raw
70167113	High		sPVTTLLEcMHk	Unambiguous	4	1	N-Term(iTRAQ4plex); C9(Methylthio); K12(iTRAQ4plex)	HCD	1.0000	0.0000	1	1	11858	Unique	Used	0.231	1.636E4	4.150E3	156	6.19	0.00	0	41	55	3	564.96161	1692.87027	-1.36	55.70	6657	6657	MS2	34/44	Glioma_Nuclear_prep_Gradeall_4plex.raw
70167113	High		sPVTTLLEcMHk	Unambiguous	4	1	N-Term(iTRAQ4plex); C9(Methylthio); K12(iTRAQ4plex)	HCD	1.0000	0.0000	1	1	11933	Unique	Used	0.307	5.632E4	1.896E4	162	7.01	0.00	0	10	14	3	564.96387	1692.87705	2.64	56.35	6739	6739	MS2	34/44	Glioma_Nuclear_prep_Gradeall_4plex.raw
70167113		High	aMTILLEEAk	Unambiguous	4	1	N-Term(iTRAQ4plex); K10(iTRAQ4plex)	HCD	1.0000	0.0000	1	1	11392	Unique	Used	0.724	3.642E3	2.875E3	234	4.34	0.00	0	37	300	2	703.91516	1406.82305	4.46	62.68	6002	6002	MS2	18/18	Glioma_Nuclear_prep_Gradeall_repeat_4plex.raw
70167113	High		aMTILLEEAk	Unambiguous	4	1	N-Term(iTRAQ4plex); K10(iTRAQ4plex)	HCD	1.0000	0.0000	1	1	10521	Unique	Used	0.649	2.526E5	1.798E5	269	4.15	0.00	0	28	8	2	703.90125	1406.79521	-15.32	44.81	5200	5200	MS2	18/18	Glioma_Nuclear_prep_Gradeall_4plex.raw
70167113		High	lVDQSGPPHEPk	Unambiguous	4	1	N-Term(iTRAQ4plex); K12(iTRAQ4plex)	HCD	1.0000	0.0000	1	1	7692	Unique	Used	0.324	1.784E4	6.305E3	188	4.06	0.00	0	16	58	3	531.29028	1591.85630	-7.53	20.87	2002	2002	MS2	24/44	Glioma_Nuclear_prep_Gradeall_repeat_4plex.raw
70167113	High		lVDQSGPPHEPk	Unambiguous	4	1	N-Term(iTRAQ4plex); K12(iTRAQ4plex)	HCD	1.0000	0.0000	1	1	7608	Unique	Used	0.325	7.913E4	2.822E4	213	4.20	0.00	0	35	8	3	531.29547	1591.87186	2.25	23.59	2028	2028	MS2	22/44	Glioma_Nuclear_prep_Gradeall_4plex.raw
70167113	High		lVDQSGPPHEPk	Unambiguous	4	1	N-Term(iTRAQ4plex); K12(iTRAQ4plex)	HCD	1.0000	0.0000	1	1	7633	Unique	Used	0.370	1.043E5	4.233E4	239	4.76	0.00	0	6	18	2	796.43866	1591.87004	1.11	23.77	2055	2055	MS2	20/22	Glioma_Nuclear_prep_Gradeall_4plex.raw
70167113		High	tAESQTPTPSATSFFSGk	Unambiguous	4	1	N-Term(iTRAQ4plex); K18(iTRAQ4plex)	HCD	1.0000	0.0000	1	1	10336	Unique	Used	0.662	9.643E2	6.956E2	137	6.73	0.00	0	16	269	3	711.36450	2132.07895	1.83	49.09	4814	4814	MS2	22/68	Glioma_Nuclear_prep_Gradeall_repeat_4plex.raw
70167113	High		tAESQTPTPSATSFFSGk	Unambiguous	4	1	N-Term(iTRAQ4plex); K18(iTRAQ4plex)	HCD	1.0000	0.0000	1	1	9667	Unique	Used	0.247	7.980E4	2.161E4	128	6.40	0.00	0	0	18	2	1066.52258	2132.03789	-17.43	38.45	4268	4268	MS2	22/34	Glioma_Nuclear_prep_Gradeall_4plex.raw
70167113		High	nIFLLFk	Unambiguous	4	1	N-Term(iTRAQ4plex); K7(iTRAQ4plex)	HCD	1.0000	0.0000	1	1	12446	Unique	Used	0.700	1.581E3	1.206E3	152	3.04	0.00	0	39	300	2	591.87726	1182.74724	-1.42	86.94	7862	7862	MS2	11/12	Glioma_Nuclear_prep_Gradeall_repeat_4plex.raw
70167113	High		nIFLLFk	Unambiguous	4	1	N-Term(iTRAQ4plex); K7(iTRAQ4plex)	HCD	1.0000	0.0000	1	1	12321	Unique	Used	0.687	1.661E4	1.252E4	129	3.23	0.00	0	50	177	2	591.87830	1182.74932	0.34	60.25	7165	7165	MS2	11/12	Glioma_Nuclear_prep_Gradeall_4plex.raw
70167113		High	dINAVLIDmER	Unambiguous	4	1	N-Term(iTRAQ4plex); M9(Oxidation)	HCD	1.0000	0.0000	1	1	10126	Unique	Used	0.430	1.006E4	4.716E3	229	4.01	0.00	0	18	300	2	724.88239	1448.75749	2.67	46.60	4590	4590	MS2	18/20	Glioma_Nuclear_prep_Gradeall_repeat_4plex.raw
70167113	High		dINAVLIDmER	Unambiguous	4	1	N-Term(iTRAQ4plex); M9(Oxidation)	HCD	1.0000	0.0000	1	1	9597	Unique	Used	0.580	1.612E5	1.026E5	183	3.20	0.00	0	23	14	2	724.86945	1448.73161	-15.19	37.94	4192	4192	MS2	17/20	Glioma_Nuclear_prep_Gradeall_4plex.raw
73760415	NP_001027392.1	STXBP1	6812	syntaxin-binding protein 1 isoform b [Homo sapiens]	5.56	2	2	4	1.726	3;1	15.9	13.57	594	67.5	6.96																			
73760415	High		lAEQIATLcATLk	Unambiguous	2	1	N-Term(iTRAQ4plex); C9(Methylthio); K13(iTRAQ4plex)	HCD	1.0000	0.0000	1	1	13123	Unique	Used	1.851	1.187E3	2.411E3	217	4.35	0.00	0	25	300	2	854.98480	1708.96233	2.52	75.14	8353	8353	MS2	21/24	Glioma_Nuclear_prep_Gradeall_4plex.raw
73760415	High		lAEQIATLcATLk	Unambiguous	2	1	N-Term(iTRAQ4plex); C9(Methylthio); K13(iTRAQ4plex)	HCD	1.0000	0.0000	1	1	13122	Unique	Used	2.154	8.250E2	1.950E3	205	4.60	0.00	0	0	300	2	854.98358	1708.95989	1.09	75.06	8348	8348	MS2	21/24	Glioma_Nuclear_prep_Gradeall_4plex.raw
73760415		High	dNALLAQLIQDk	Unambiguous	2	1	N-Term(iTRAQ4plex); K12(iTRAQ4plex)	HCD	1.0000	0.0000	1	1	12175	Unique	Used	1.529	1.945E2	3.241E2	206	3.93	0.00	0	37	300	3	543.98492	1629.94022	-0.75	75.60	7059	7059	MS2	20/44	Glioma_Nuclear_prep_Gradeall_repeat_4plex.raw
73760415	High		dNALLAQLIQDk	Unambiguous	2	1	N-Term(iTRAQ4plex); K12(iTRAQ4plex)	HCD	1.0000	0.0000	1	1	11529	Unique	Used	1.602	5.010E3	8.807E3	209	4.62	0.00	0	15	52	3	543.98163	1629.93033	-6.82	52.83	6299	6299	MS2	21/44	Glioma_Nuclear_prep_Gradeall_4plex.raw
86991438	NP_001034554.1	SRSF5	6430	serine/arginine-rich splicing factor 5 [Homo sapiens]	17.28	1	3	7	0.623	4;3	11.9	25.08	272	31.2	11.59																			
86991438		High	gFGFVEFEDPR	Unambiguous	1	1	N-Term(iTRAQ4plex)	HCD	1.0000	0.0000	1	1	11672	Unique	Used	0.524	6.648E3	3.797E3	115	4.16	0.00	0	14	300	2	722.35632	1443.70537	1.94	66.60	6337	6337	MS2	17/20	Glioma_Nuclear_prep_Gradeall_repeat_4plex.raw
86991438	High		gFGFVEFEDPR	Unambiguous	1	1	N-Term(iTRAQ4plex)	HCD	1.0000	0.0000	1	1	10882	Unique	Used	0.603	1.396E5	9.239E4	100	4.02	0.00	0	24	11	2	722.34497	1443.68266	-13.78	47.54	5593	5593	MS2	17/20	Glioma_Nuclear_prep_Gradeall_4plex.raw
86991438		High	dADDAVYELDGk	Unambiguous	1	1	N-Term(iTRAQ4plex); K12(iTRAQ4plex)	HCD	1.0000	0.0000	1	1	9607	Unique	Used	0.623	1.195E4	8.117E3	101	4.99	0.00	0	9	182	2	799.89288	1598.77849	-0.23	40.49	4031	4031	MS2	21/22	Glioma_Nuclear_prep_Gradeall_repeat_4plex.raw
86991438		High	dADDAVYELDGk	Unambiguous	1	1	N-Term(iTRAQ4plex); K12(iTRAQ4plex)	HCD	1.0000	0.0000	1	1	9068	Unique	Used	0.747	1.409E4	1.148E4	108	5.23	0.00	0	21	118	2	799.89410	1598.78093	1.30	34.52	3455	3455	MS2	21/22	Glioma_Nuclear_prep_Gradeall_repeat_4plex.raw
86991438	High		dADDAVYELDGk	Unambiguous	1	1	N-Term(iTRAQ4plex); K12(iTRAQ4plex)	HCD	1.0000	0.0000	1	1	9229	Unique	Used	0.600	6.713E4	4.419E4	76	4.72	0.00	0	0	35	2	799.87927	1598.75127	-17.26	35.21	3789	3789	MS2	21/22	Glioma_Nuclear_prep_Gradeall_4plex.raw
86991438	High		INEGVVEFASYGDLk	Unambiguous	1	1	N-Term(iTRAQ4plex); K15(iTRAQ4plex)	HCD	1.0000	0.0000	1	1	11086	Unique	Used	0.637	1.415E5	9.885E4	238	6.26	0.00	0	12	13	2	965.01074	1929.01421	-3.43	49.13	5815	5815	MS2	24/28	Glioma_Nuclear_prep_Gradeall_4plex.raw
86991438	High		INEGVVEFASYGDLk	Unambiguous	1	1	N-Term(iTRAQ4plex); K15(iTRAQ4plex)	HCD	1.0000	0.0000	1	1	11085	Unique	Used	0.719	1.262E4	9.957E3	213	4.46	0.00	0	28	10	3	643.67651	1929.01499	-3.03	49.13	5814	5814	MS2	26/56	Glioma_Nuclear_prep_Gradeall_4plex.raw
89145417	NP_054752.3	METTL7A	25840	methyltransferase-like protein 7A precursor [Homo sapiens]	11.07	1	2	3	2.118	1;2	24.4	9.23	244	28.3	8.38																			
89145417		High	vTcIDPNPNFEk	Unambiguous	1	1	N-Term(iTRAQ4plex); C3(Methylthio); K12(iTRAQ4plex)	HCD	1.0000	0.0000	1	1	10569	Unique	Used	2.238	2.213E3	5.397E3	128	4.45	0.00	0	48	300	2	855.92700	1710.84673	1.95	51.85	5062	5062	MS2	17/22	Glioma_Nuclear_prep_Gradeall_repeat_4plex.raw
89145417	High		vTcIDPNPNFEk	Unambiguous	1	1	N-Term(iTRAQ4plex); C3(Methylthio); K12(iTRAQ4plex)	HCD	1.0000	0.0000	1	1	9854	Unique	Used	2.118	4.660E4	1.083E5	85	4.61	0.00	0	11	25	2	855.91235	1710.81743	-15.17	39.79	4471	4471	MS2	17/22	Glioma_Nuclear_prep_Gradeall_4plex.raw
89145417		High	eLFSNLQEFAGPSGk	Unambiguous	1	1	N-Term(iTRAQ4plex); K15(iTRAQ4plex)	HCD	1.0000	0.0000	1	1	11812	Unique	Used	1.377	2.593E2	3.892E2	161	4.61	0.00	0	36	300	3	638.00671	1912.00559	0.04	68.81	6520	6520	MS2	26/56	Glioma_Nuclear_prep_Gradeall_repeat_4plex.raw
91208426	NP_006436.3	PRPF8	10594	pre-mRNA-processing-splicing factor 8 [Homo sapiens]	8.27	1	11	15	1.555	8;7	19.8	61.40	2335	273.4	8.84																			
91208426		High	eELGLIEQAYDNPHEALSR	Unambiguous	1	1	N-Term(iTRAQ4plex)	HCD	1.0000	0.0000	1	1	11418	Unique	Used	1.722	2.953E2	5.541E2	102	8.38	0.00	0	14	300	3	776.72583	2328.16294	1.73	63.05	6034	6034	MS2	42/72	Glioma_Nuclear_prep_Gradeall_repeat_4plex.raw
91208426		High	tAEEVAALIR	Unambiguous	1	1	N-Term(iTRAQ4plex)	HCD	1.0000	0.0000	1	1	10734	Unique	Used	1.311	1.048E4	1.498E4	232	3.92	0.00	0	13	300	2	608.85541	1216.70354	1.41	53.85	5241	5241	MS2	17/18	Glioma_Nuclear_prep_Gradeall_repeat_4plex.raw
91208426	High		eELGLIEQAYDNPHEALSR	Unambiguous	1	1	N-Term(iTRAQ4plex)	HCD	1.0000	0.0000	1	1	10504	Unique	Used	1.395	1.566E4	2.397E4	69	6.72	0.00	0	8	7	3	776.71332	2328.12540	-14.39	44.68	5181	5181	MS2	39/72	Glioma_Nuclear_prep_Gradeall_4plex.raw
91208426	High		vPGLPTPIENMILR	Unambiguous	1	1	N-Term(iTRAQ4plex)	HCD	1.0000	0.0000	1	1	12493	Unique	Used	2.052	5.139E3	1.157E4	177	3.68	0.00	0	29	300	2	847.49506	1693.98284	2.14	62.24	7356	7356	MS2	14/26	Glioma_Nuclear_prep_Gradeall_4plex.raw
91208426	High		vPGLPTPIENMILR	Unambiguous	1	1	N-Term(iTRAQ4plex)	HCD	1.0000	0.0000	1	1	12436	Unique	Used	2.458	9.182E3	2.476E4	124	3.33	0.00	0	30	222	2	847.49286	1693.97844	-0.46	61.55	7292	7292	MS2	14/26	Glioma_Nuclear_prep_Gradeall_4plex.raw
91208426	High		gSELQLPFQAcLk	Unambiguous	1	1	N-Term(iTRAQ4plex); C11(Methylthio); K13(iTRAQ4plex)	HCD	1.0000	0.0000	1	1	12334	Unique	Used	1.555	5.726E3	9.773E3	263	4.82	0.00	0	30	300	2	884.47253	1767.93779	0.09	60.40	7179	7179	MS2	19/24	Glioma_Nuclear_prep_Gradeall_4plex.raw
91208426	High		fSPIPFPPLSYk	Unambiguous	1	1	N-Term(iTRAQ4plex); K12(iTRAQ4plex)	HCD	1.0000	0.0000	1	1	11988	Unique	Used	1.910	1.378E4	2.887E4	252	4.36	0.00	0	48	187	2	840.98376	1680.96025	-0.07	56.86	6799	6799	MS2	17/22	Glioma_Nuclear_prep_Gradeall_4plex.raw
91208426		High	gPGNPVPGPLAPLPDYMSEEk	Unambiguous	1	1	N-Term(iTRAQ4plex); K21(iTRAQ4plex)	HCD	1.0000	0.0000	1	1	11677	Unique	Used	1.320	1.538E2	2.213E2	120	4.28	0.00	0	34	300	3	818.42731	2453.26737	1.95	66.66	6342	6342	MS2	30/80	Glioma_Nuclear_prep_Gradeall_repeat_4plex.raw
91208426	High		sQEPLPDDDEEFELPEFVEPFLk	Unambiguous	1	1	N-Term(iTRAQ4plex); K23(iTRAQ4plex)	HCD	1.0000	0.0000	1	1	13062	Unique	Used	1.577	1.515E2	2.622E2	105	6.26	0.00	0	2	220	3	1013.16815	3037.48990	2.94	71.33	8100	8100	MS2	27/88	Glioma_Nuclear_prep_Gradeall_4plex.raw
91208426		High	fGFVDAQk	Unambiguous	1	1	N-Term(iTRAQ4plex); K8(iTRAQ4plex)	HCD	1.0000	0.0000	1	1	9594	Unique	Used	1.416	1.780E4	2.748E4	168	2.92	0.00	0	43	178	2	600.33405	1199.66081	-4.59	40.33	4017	4017	MS2	14/14	Glioma_Nuclear_prep_Gradeall_repeat_4plex.raw
91208426		High	aLDIPLVk	Unambiguous	1	1	N-Term(iTRAQ4plex); K8(iTRAQ4plex)	HCD	1.0000	0.0000	1	1	10730	Unique	Used	1.953	3.372E3	7.178E3	48	3.09	0.00	0	48	300	2	578.88031	1156.75334	-0.92	53.80	5236	5236	MS2	14/14	Glioma_Nuclear_prep_Gradeall_repeat_4plex.raw
91208426	High		aLDIPLVk	Unambiguous	1	1	N-Term(iTRAQ4plex); K8(iTRAQ4plex)	HCD	1.0000	0.0000	1	1	9955	Unique	Used	1.552	1.451E5	2.470E5	85	2.99	0.00	0	46	6	2	578.87067	1156.73406	-17.59	40.54	4581	4581	MS2	14/14	Glioma_Nuclear_prep_Gradeall_4plex.raw
91208426		High	sLPVEEQPk	Unambiguous	1	1	N-Term(iTRAQ4plex); K9(iTRAQ4plex)	HCD	1.0000	0.0000	1	1	8020	Unique	Used	1.438	2.780E4	4.357E4	290	3.47	0.00	0	48	117	2	657.87866	1314.75005	-0.56	23.98	2347	2347	MS2	14/16	Glioma_Nuclear_prep_Gradeall_repeat_4plex.raw
91208426		High	dLILADYGk	Unambiguous	1	1	N-Term(iTRAQ4plex); K9(iTRAQ4plex)	HCD	1.0000	0.0000	1	1	10341	Unique	Used	1.975	6.846E3	1.473E4	364	3.55	0.00	0	49	300	2	648.37463	1295.74199	-2.29	49.15	4819	4819	MS2	14/16	Glioma_Nuclear_prep_Gradeall_repeat_4plex.raw
91208426	High		dLILADYGk	Unambiguous	1	1	N-Term(iTRAQ4plex); K9(iTRAQ4plex)	HCD	1.0000	0.0000	1	1	9720	Unique	Used	1.441	8.186E4	1.294E5	315	3.32	0.00	0	48	18	2	648.36432	1295.72136	-18.21	38.82	4325	4325	MS2	14/16	Glioma_Nuclear_prep_Gradeall_4plex.raw
93004092	NP_005587.2	NFIB	4781	nuclear factor 1 B-type isoform 3 [Homo sapiens]	10.48	12	3	4	1.762	1;3	7.9	14.26	420	47.4	8.87																			
93004092		High	sGVFNVSELVR	Unambiguous	3	1	N-Term(iTRAQ4plex)	HCD	1.0000	0.0000	1	1	11422	Unique	Used	1.788	3.554E3	6.927E3	369	3.86	0.00	0	39	300	2	675.87885	1350.75041	0.43	63.11	6039	6039	MS2	18/20	Glioma_Nuclear_prep_Gradeall_repeat_4plex.raw
93004092	High		sGVFNVSELVR	Unambiguous	3	1	N-Term(iTRAQ4plex)	HCD	1.0000	0.0000	1	1	10536	Unique	Used	1.577	1.011E5	1.749E5	342	4.01	0.00	0	31	20	2	675.86859	1350.72991	-14.75	44.92	5216	5216	MS2	19/20	Glioma_Nuclear_prep_Gradeall_4plex.raw
93004092		High	eDFVLTVTGk	Unambiguous	7	1	N-Term(iTRAQ4plex); K10(iTRAQ4plex)	HCD	1.0000	0.0000	1	1	10757	Unique	Used	1.735	3.534E3	6.685E3	278	4.02	0.00	0	44	300	2	698.90155	1396.79582	2.27	54.12	5265	5265	MS2	15/18	Glioma_Nuclear_prep_Gradeall_repeat_4plex.raw
93004092		High	nPPGYLEDSFVk	Unambiguous	3	1	N-Term(iTRAQ4plex); K12(iTRAQ4plex)	HCD	1.0000	0.0000	1	1	10487	Unique	Used	1.909	2.013E3	4.189E3	240	3.09	0.00	0	46	300	2	827.43994	1653.87261	-0.05	50.87	4974	4974	MS2	12/22	Glioma_Nuclear_prep_Gradeall_repeat_4plex.raw
94721261	NP_149124.3	CNP	1267	2',3'-cyclic-nucleotide 3'-phosphodiesterase [Homo sapiens]	5.23	1	2	4	1.498	1;3	7.5	13.51	421	47.5	9.07																			
94721261		High	aGQVFLEELGNHk	Unambiguous	1	1	N-Term(iTRAQ4plex); K13(iTRAQ4plex)	HCD	1.0000	0.0000	1	1	10926	Unique	Used	1.670	9.341E2	1.700E3	163	6.49	0.00	0	22	300	3	577.32056	1729.94712	-0.28	56.25	5452	5452	MS2	32/48	Glioma_Nuclear_prep_Gradeall_repeat_4plex.raw
94721261		High	mDLVTYFGk	Unambiguous	1	1	N-Term(iTRAQ4plex); K9(iTRAQ4plex)	HCD	1.0000	0.0000	1	1	11706	Unique	Used	1.409	5.670E3	8.709E3	318	3.65	0.00	0	58	300	2	681.37317	1361.73906	0.95	67.06	6376	6376	MS2	14/16	Glioma_Nuclear_prep_Gradeall_repeat_4plex.raw
94721261	High		mDLVTYFGk	Unambiguous	1	1	N-Term(iTRAQ4plex); K9(iTRAQ4plex)	HCD	1.0000	0.0000	1	1	10892	Unique	Used	1.461	3.994E5	6.406E5	260	3.76	0.00	0	32	9	2	681.36230	1361.71733	-15.01	47.63	5604	5604	MS2	15/16	Glioma_Nuclear_prep_Gradeall_4plex.raw
94721261		High	mDLVTYFGk	Unambiguous	1	1	N-Term(iTRAQ4plex); M1(Oxidation); K9(iTRAQ4plex)	HCD	1.0000	0.0000	1	1	10627	Unique	Used	1.534	5.496E3	9.190E3	296	3.37	0.00	0	75	300	2	689.36896	1377.73064	-1.48	52.55	5125	5125	MS2	14/16	Glioma_Nuclear_prep_Gradeall_repeat_4plex.raw
109240550	NP_001035879.1	PSPC1	55269	paraspeckle component 1 [Homo sapiens]	20.65	1	8	13	0.261	6;7	29.3	38.59	523	58.7	6.67																			
109240550	High		nLSPVVSNELLEQAFSQFGPVEk	Unambiguous	1	1	N-Term(iTRAQ4plex); K23(iTRAQ4plex)	HCD	1.0000	0.0000	1	1	12958	Unique	Used	0.232	1.029E3	2.623E2	109	9.06	0.00	0	13	235	3	940.84186	2820.51102	3.10	68.97	7924	7924	MS2	34/88	Glioma_Nuclear_prep_Gradeall_4plex.raw
109240550		High	fAQPGTFEFEYASR	Unambiguous	1	1	N-Term(iTRAQ4plex)	HCD	1.0000	0.0000	1	1	11306	Unique	Used	0.155	7.395E3	1.252E3	134	4.26	0.00	0	12	300	2	897.43707	1793.86687	2.95	61.38	5894	5894	MS2	20/26	Glioma_Nuclear_prep_Gradeall_repeat_4plex.raw
109240550		High	yGEPSEVFINR	Unambiguous	1	1	N-Term(iTRAQ4plex)	HCD	1.0000	0.0000	1	1	10006	Unique	Used	0.257	2.175E4	6.103E3	220	3.67	0.00	0	41	300	2	727.87573	1454.74419	3.12	45.12	4458	4458	MS2	15/20	Glioma_Nuclear_prep_Gradeall_repeat_4plex.raw
109240550	High		fAQPGTFEFEYASR	Unambiguous	1	1	N-Term(iTRAQ4plex)	HCD	1.0000	0.0000	1	1	10496	Unique	Used	0.233	2.908E5	7.442E4	86	4.34	0.00	0	28	27	2	897.42389	1793.84050	-11.75	44.61	5172	5172	MS2	17/26	Glioma_Nuclear_prep_Gradeall_4plex.raw
109240550		High	fATHGAALTVk	Unambiguous	1	1	N-Term(iTRAQ4plex); K11(iTRAQ4plex)	HCD	1.0000	0.0000	1	1	8429	Unique	Used	0.345	1.898E4	7.134E3	264	3.63	0.00	0	53	300	2	702.41937	1403.83147	4.65	27.91	2777	2777	MS2	18/20	Glioma_Nuclear_prep_Gradeall_repeat_4plex.raw
109240550	High		fATHGAALTVk	Unambiguous	1	1	N-Term(iTRAQ4plex); K11(iTRAQ4plex)	HCD	1.0000	0.0000	1	1	8055	Unique	Used	0.293	2.097E5	6.734E4	235	4.02	0.00	0	10	23	2	702.40753	1403.80779	-12.22	26.74	2512	2512	MS2	20/20	Glioma_Nuclear_prep_Gradeall_4plex.raw
109240550		High	IFVGNLPTDITEEDFkR	Unambiguous	1	1	N-Term(iTRAQ4plex); K16(iTRAQ4plex)	HCD	1.0000	0.0000	1	1	12030	Unique	Used	0.141	1.603E3	2.461E2	162	4.55	0.00	1	26	300	3	761.41620	2282.23404	3.02	72.55	6822	6822	MS2	27/64	Glioma_Nuclear_prep_Gradeall_repeat_4plex.raw
109240550		High	IFVGNLPTDITEEDFk	Unambiguous	1	1	N-Term(iTRAQ4plex); K16(iTRAQ4plex)	HCD	1.0000	0.0000	1	1	12356	Unique	Used	0.360	2.917E2	1.143E2	173	4.54	0.00	0	17	300	3	709.38214	2126.13187	2.74	80.79	7444	7444	MS2	25/60	Glioma_Nuclear_prep_Gradeall_repeat_4plex.raw
109240550	High		IFVGNLPTDITEEDFk	Unambiguous	1	1	N-Term(iTRAQ4plex); K16(iTRAQ4plex)	HCD	1.0000	0.0000	1	1	11992	Unique	Used	0.184	7.421E4	1.495E4	218	6.23	0.00	0	11	67	2	1063.56628	2126.12529	-0.35	56.91	6804	6804	MS2	22/30	Glioma_Nuclear_prep_Gradeall_4plex.raw
109240550	High		IFVGNLPTDITEEDFk	Unambiguous	1	1	N-Term(iTRAQ4plex); K16(iTRAQ4plex)	HCD	1.0000	0.0000	1	1	11981	Unique	Used	0.292	1.169E4	3.750E3	172	5.57	0.00	0	19	21	3	709.38043	2126.12674	0.33	56.81	6792	6792	MS2	23/60	Glioma_Nuclear_prep_Gradeall_4plex.raw
109240550		High	gFVEFAAkPPAR	Unambiguous	1	1	N-Term(iTRAQ4plex); K8(iTRAQ4plex)	HCD	1.0000	0.0000	1	1	9360	Unique	Used	0.401	1.371E4	5.986E3	166	3.77	0.00	0	25	104	3	526.64221	1577.91208	4.94	37.74	3763	3763	MS2	25/44	Glioma_Nuclear_prep_Gradeall_repeat_4plex.raw
109240550		High	aELDGTILk	Unambiguous	1	1	N-Term(iTRAQ4plex); K9(iTRAQ4plex)	HCD	1.0000	0.0000	1	1	9383	Unique	Used	0.261	4.272E4	1.216E4	258	3.70	0.00	0	32	207	2	624.37762	1247.74797	2.41	37.97	3787	3787	MS2	16/16	Glioma_Nuclear_prep_Gradeall_repeat_4plex.raw
109240550	High		aELDGTILk	Unambiguous	1	1	N-Term(iTRAQ4plex); K9(iTRAQ4plex)	HCD	1.0000	0.0000	1	1	8908	Unique	Used	0.282	4.397E5	1.363E5	266	3.10	0.00	0	37	10	2	624.36835	1247.72942	-12.46	32.87	3439	3439	MS2	16/16	Glioma_Nuclear_prep_Gradeall_4plex.raw
109689718	NP_001035937.1	ABHD12	26090	monoacylglycerol lipase ABHD12 isoform a [Homo sapiens]	9.55	2	2	2	0.486	1;1	26.0	13.25	398	45.1	8.65																			
109689718		High	vQFVPFHSDLGYR	Unambiguous	2	1	N-Term(iTRAQ4plex)	HCD	1.0000	0.0000	1	1	11073	Unique	Used	0.397	1.496E3	6.474E2	206	3.96	0.00	0	32	300	3	570.30334	1708.89548	1.57	58.09	5614	5614	MS2	21/48	Glioma_Nuclear_prep_Gradeall_repeat_4plex.raw
109689718	High		eTPPDALILESPFTNIR	Unambiguous	2	1	N-Term(iTRAQ4plex)	HCD	1.0000	0.0000	1	1	12214	Unique	Used	0.576	1.594E4	1.008E4	246	5.44	0.00	0	48	300	2	1029.06226	2057.11724	6.62	59.13	7047	7047	MS2	18/32	Glioma_Nuclear_prep_Gradeall_4plex.raw
114155142	NP_003283.2	TPR	7175	nucleoprotein TPR [Homo sapiens]	6.94	1	9	10	0.661	6;4	12.9	39.51	2363	267.1	5.02																			
114155142		High	iQQLTEEIGR	Unambiguous	1	1	N-Term(iTRAQ4plex)	HCD	1.0000	0.0000	1	1	10290	Unique	Used	0.700	6.664E3	5.084E3	294	3.73	0.00	0	41	300	2	665.87695	1330.74663	1.42	48.53	4764	4764	MS2	16/18	Glioma_Nuclear_prep_Gradeall_repeat_4plex.raw
114155142		High	sQEQILEILR	Unambiguous	1	1	N-Term(iTRAQ4plex)	HCD	1.0000	0.0000	1	1	11477	Unique	Used	0.741	4.655E3	3.757E3	196	4.09	0.00	0	50	300	2	686.90118	1372.79509	2.47	63.85	6103	6103	MS2	17/18	Glioma_Nuclear_prep_Gradeall_repeat_4plex.raw
114155142	High		lESALTELEQLR	Unambiguous	1	1	N-Term(iTRAQ4plex)	HCD	1.0000	0.0000	1	1	12242	Unique	Used	0.544	2.381E4	1.421E4	214	4.20	0.00	0	43	205	2	773.43506	1545.86284	1.50	59.42	7078	7078	MS2	21/22	Glioma_Nuclear_prep_Gradeall_4plex.raw
114155142	High		nLQEQTVQLQSELSR	Unambiguous	1	1	N-Term(iTRAQ4plex)	HCD	1.0000	0.0000	1	1	10010	Unique	Used	0.549	4.082E4	2.459E4	175	5.07	0.00	0	0	54	2	958.99701	1916.98674	-15.18	40.92	4640	4640	MS2	22/28	Glioma_Nuclear_prep_Gradeall_4plex.raw
114155142	High		gQNLLLTNLQTIQGILER	Unambiguous	1	1	N-Term(iTRAQ4plex)	HCD	1.0000	0.0000	1	1	13129	Unique	Used	0.552	1.080E2	6.545E1	64	6.08	0.00	0	12	300	3	723.42450	2168.25895	3.20	75.82	8397	8397	MS2	26/68	Glioma_Nuclear_prep_Gradeall_4plex.raw
114155142	High		sQEQILEILR	Unambiguous	1	1	N-Term(iTRAQ4plex)	HCD	1.0000	0.0000	1	1	10599	Unique	Used	0.728	9.622E4	7.691E4	253	3.12	0.00	0	54	27	2	686.89056	1372.77385	-13.00	45.38	5284	5284	MS2	17/18	Glioma_Nuclear_prep_Gradeall_4plex.raw
114155142	High		qLQEIFENYk	Unambiguous	1	1	N-Term(iTRAQ4plex); K10(iTRAQ4plex)	HCD	1.0000	0.0000	1	1	10679	Unique	Used	0.614	4.290E4	2.889E4	238	3.53	0.00	0	56	46	2	800.42529	1599.84331	-11.75	45.99	5372	5372	MS2	17/18	Glioma_Nuclear_prep_Gradeall_4plex.raw
114155142	High		lDELQASDVSVk	Unambiguous	1	1	N-Term(iTRAQ4plex); K12(iTRAQ4plex)	HCD	1.0000	0.0000	1	1	9348	Unique	Used	0.659	7.068E4	5.110E4	236	5.10	0.00	0	6	23	2	796.43158	1591.85588	-14.01	36.13	3921	3921	MS2	21/22	Glioma_Nuclear_prep_Gradeall_4plex.raw
114155142		High	eLENANDLLSATk	Unambiguous	1	1	N-Term(iTRAQ4plex); K13(iTRAQ4plex)	HCD	1.0000	0.0000	1	1	10360	Unique	Used	0.664	2.902E3	2.099E3	229	5.20	0.00	0	27	300	2	853.46735	1705.92742	3.70	49.37	4839	4839	MS2	21/24	Glioma_Nuclear_prep_Gradeall_repeat_4plex.raw
114155142		High	eTLNQAETk	Unambiguous	1	1	N-Term(iTRAQ4plex); K9(iTRAQ4plex)	HCD	1.0000	0.0000	1	1	7279	Unique	Used	0.768	1.577E4	1.321E4	237	3.07	0.00	0	39	211	2	661.35260	1321.69792	-16.85	17.19	1563	1563	MS2	15/16	Glioma_Nuclear_prep_Gradeall_repeat_4plex.raw
117168248	NP_057479.2	HACD3	51495	very-long-chain (3R)-3-hydroxyacyl-CoA dehydratase 3 [Homo sapiens]	14.92	1	2	2	2.053	1;1	25.6	15.80	362	43.1	8.94																			
117168248	High		vELSDVQNPAISITENVLHFk	Unambiguous	1	1	N-Term(iTRAQ4plex); K21(iTRAQ4plex)	HCD	1.0000	0.0000	1	1	12798	Unique	Used	2.764	2.346E2	7.117E2	80	8.27	0.00	0	33	300	3	881.15302	2641.44449	0.18	66.32	7711	7711	MS2	45/80	Glioma_Nuclear_prep_Gradeall_4plex.raw
117168248		High	qVNITVQk	Unambiguous	1	1	N-Term(iTRAQ4plex); K8(iTRAQ4plex)	HCD	1.0000	0.0000	1	1	8242	Unique	Used	1.341	1.643E4	2.401E4	223	3.05	0.00	0	49	159	2	609.37653	1217.74578	0.13	26.13	2581	2581	MS2	12/14	Glioma_Nuclear_prep_Gradeall_repeat_4plex.raw
117190174	NP_004491.2	HNRNPC	3183	heterogeneous nuclear ribonucleoproteins C1/C2 isoform b [Homo sapiens]	41.64	5	12	35	0.452	19;16	28.7	113.18	293	32.3	5.08																			
117190174		High	gFAFVQYVNER	Unambiguous	2	1	N-Term(iTRAQ4plex)	HCD	1.0000	0.0000	1	1	11385	Unique	Used	0.514	1.285E4	7.199E3	249	4.27	0.00	0	26	300	2	737.38544	1473.76360	1.95	62.58	5994	5994	MS2	16/20	Glioma_Nuclear_prep_Gradeall_repeat_4plex.raw
117190174		High	aAVAGEDGR	Unambiguous	5	1	N-Term(iTRAQ4plex)	HCD	1.0000	0.0000	1	1	6592	Unique	Used	0.771	2.529E4	2.125E4	107	3.22	0.00	0	42	223	2	495.25595	989.50463	-8.76	11.09	838	838	MS2	15/16	Glioma_Nuclear_prep_Gradeall_repeat_4plex.raw
117190174	High		sAAEMYGSSFDLDYDFQR	Unambiguous	1	1	N-Term(iTRAQ4plex)	HCD	1.0000	0.0000	1	1	11390	Unique	Used	0.489	2.470E4	1.327E4	36	6.58	0.00	0	14	64	2	1123.49646	2245.98564	1.22	51.65	6147	6147	MS2	27/34	Glioma_Nuclear_prep_Gradeall_4plex.raw
117190174	High		gFAFVQYVNER	Unambiguous	2	1	N-Term(iTRAQ4plex)	HCD	1.0000	0.0000	1	1	10522	Unique	Used	0.493	4.202E5	2.273E5	178	4.35	0.00	0	37	13	2	737.37433	1473.74138	-13.13	44.82	5201	5201	MS2	17/20	Glioma_Nuclear_prep_Gradeall_4plex.raw
117190174		High	kSDVEAIFSk	Unambiguous	5	1	N-Term(iTRAQ4plex); K1(iTRAQ4plex); K10(iTRAQ4plex)	HCD	1.0000	0.0000	1	1	9622	Unique	Used	0.513	3.319E3	1.856E3	190	4.11	0.00	1	81	253	3	519.30322	1555.89511	-6.75	40.65	4047	4047	MS2	22/36	Glioma_Nuclear_prep_Gradeall_repeat_4plex.raw
117190174	High		kSDVEAIFSk	Unambiguous	5	1	N-Term(iTRAQ4plex); K1(iTRAQ4plex); K10(iTRAQ4plex)	HCD	1.0000	0.0000	1	1	8995	Unique	Used	0.289	3.177E5	1.006E5	226	4.15	0.00	1	54	9	2	778.44763	1555.88799	-11.33	33.48	3533	3533	MS2	15/18	Glioma_Nuclear_prep_Gradeall_4plex.raw
117190174	High		kSDVEAIFSk	Unambiguous	5	1	N-Term(iTRAQ4plex); K1(iTRAQ4plex); K10(iTRAQ4plex)	HCD	1.0000	0.0000	1	1	8991	Unique	Used	0.435	4.812E4	2.300E4	210	3.44	0.00	1	54	5	3	519.30243	1555.89273	-8.28	33.46	3529	3529	MS2	17/36	Glioma_Nuclear_prep_Gradeall_4plex.raw
117190174		High	vDSLLENLEk	Unambiguous	5	1	N-Term(iTRAQ4plex); K10(iTRAQ4plex)	HCD	1.0000	0.0000	1	1	11521	Unique	Used	0.513	1.355E4	7.571E3	270	4.79	0.00	0	19	286	2	724.41724	1447.82720	1.74	64.41	6152	6152	MS2	18/18	Glioma_Nuclear_prep_Gradeall_repeat_4plex.raw
117190174		High	vDSLLENLEk	Unambiguous	5	1	N-Term(iTRAQ4plex); K10(iTRAQ4plex)	HCD	1.0000	0.0000	1	1	11591	Unique	Used	0.532	1.434E4	8.315E3	288	4.16	0.00	0	52	300	2	724.41766	1447.82805	2.33	65.46	6240	6240	MS2	17/18	Glioma_Nuclear_prep_Gradeall_repeat_4plex.raw
117190174		High	vDSLLENLEk	Unambiguous	5	1	N-Term(iTRAQ4plex); K10(iTRAQ4plex)	HCD	1.0000	0.0000	1	1	11580	Unique	Used	0.584	9.491E2	6.038E2	304	3.72	0.00	0	34	300	3	483.28049	1447.82691	1.54	65.31	6227	6227	MS2	16/36	Glioma_Nuclear_prep_Gradeall_repeat_4plex.raw
117190174	High		vDSLLENLEk	Unambiguous	5	1	N-Term(iTRAQ4plex); K10(iTRAQ4plex)	HCD	1.0000	0.0000	1	1	10709	Unique	Used	0.434	9.726E5	4.630E5	259	4.32	0.00	0	43	4	2	724.40656	1447.80583	-13.02	46.22	5405	5405	MS2	18/18	Glioma_Nuclear_prep_Gradeall_4plex.raw
117190174	High		vDSLLENLEk	Unambiguous	5	1	N-Term(iTRAQ4plex); K10(iTRAQ4plex)	HCD	1.0000	0.0000	1	1	10798	Unique	Used	0.756	6.780E5	5.626E5	335	3.55	0.00	0	21	5	2	724.40710	1447.80693	-12.26	46.91	5502	5502	MS2	16/18	Glioma_Nuclear_prep_Gradeall_4plex.raw
117190174		High	sEEEQSSSSVk	Unambiguous	2	1	N-Term(iTRAQ4plex); K11(iTRAQ4plex)	HCD	1.0000	0.0000	1	1	6651	Unique	Used	0.452	6.120E3	3.012E3	92	3.90	0.00	0	8	300	2	742.86237	1484.71745	-9.72	11.65	900	900	MS2	20/20	Glioma_Nuclear_prep_Gradeall_repeat_4plex.raw
117190174		High	vFIGNLNTLVVk	Unambiguous	5	1	N-Term(iTRAQ4plex); K12(iTRAQ4plex)	HCD	1.0000	0.0000	1	1	11991	Unique	Used	0.186	1.636E4	3.309E3	79	5.66	0.00	0	18	300	2	803.00543	1605.00359	3.58	71.86	6767	6767	MS2	22/22	Glioma_Nuclear_prep_Gradeall_repeat_4plex.raw
117190174		High	vFIGNLNTLVVk	Unambiguous	5	1	N-Term(iTRAQ4plex); K12(iTRAQ4plex)	HCD	1.0000	0.0000	1	1	11992	Unique	Used	0.415	6.608E2	2.987E2	88	4.02	0.00	0	19	300	3	535.67163	1605.00034	1.56	71.87	6768	6768	MS2	17/44	Glioma_Nuclear_prep_Gradeall_repeat_4plex.raw
117190174	High		vFIGNLNTLVVk	Unambiguous	5	1	N-Term(iTRAQ4plex); K12(iTRAQ4plex)	HCD	1.0000	0.0000	1	1	11149	Unique	Used	0.275	1.430E5	4.318E4	93	5.62	0.00	0	43	33	2	802.99988	1604.99248	-3.34	49.63	5884	5884	MS2	22/22	Glioma_Nuclear_prep_Gradeall_4plex.raw
117190174	High		mIAGQVLDINLAAEPk	Unambiguous	2	1	N-Term(iTRAQ4plex); K16(iTRAQ4plex)	HCD	1.0000	0.0000	1	1	11260	Unique	Used	0.448	6.350E4	3.119E4	107	6.98	0.00	0	14	32	2	986.05878	1971.11028	-4.32	50.52	6005	6005	MS2	29/30	Glioma_Nuclear_prep_Gradeall_4plex.raw
117190174	High		mIAGQVLDINLAAEPk	Unambiguous	2	1	N-Term(iTRAQ4plex); K16(iTRAQ4plex)	HCD	1.0000	0.0000	1	1	11246	Unique	Used	0.453	1.893E4	9.404E3	127	4.90	0.00	0	9	9	3	657.71191	1971.12119	1.21	50.41	5990	5990	MS2	26/60	Glioma_Nuclear_prep_Gradeall_4plex.raw
117190174	High		mIAGQVLDINLAAEPk	Unambiguous	2	1	N-Term(iTRAQ4plex); K16(iTRAQ4plex)	HCD	1.0000	0.0000	1	1	11168	Unique	Used	0.485	1.293E5	6.888E4	102	6.63	0.00	0	30	21	2	986.06342	1971.11955	0.38	49.79	5905	5905	MS2	29/30	Glioma_Nuclear_prep_Gradeall_4plex.raw
117190174	High		mIAGQVLDINLAAEPk	Unambiguous	2	1	N-Term(iTRAQ4plex); K16(iTRAQ4plex)	HCD	1.0000	0.0000	1	1	11370	Unique	Used	0.637	2.133E4	1.491E4	136	6.24	0.00	0	44	109	2	986.05975	1971.11223	-3.33	51.48	6125	6125	MS2	28/30	Glioma_Nuclear_prep_Gradeall_4plex.raw
117190174		High	lkGDDLQAIk	Unambiguous	5	1	N-Term(iTRAQ4plex); K2(iTRAQ4plex); K10(iTRAQ4plex)	HCD	1.0000	0.0000	1	1	8727	Unique	Used	0.431	3.807E4	1.788E4	163	3.59	0.00	1	11	13	3	511.64398	1532.91739	-12.95	30.89	3092	3092	MS2	18/36	Glioma_Nuclear_prep_Gradeall_repeat_4plex.raw
117190174		High	lkGDDLQAIk	Unambiguous	5	1	N-Term(iTRAQ4plex); K2(iTRAQ4plex); K10(iTRAQ4plex)	HCD	1.0000	0.0000	1	1	8734	Unique	Used	0.447	4.442E4	2.163E4	178	3.31	0.00	1	13	51	2	766.96307	1532.91887	-11.99	30.93	3099	3099	MS2	17/18	Glioma_Nuclear_prep_Gradeall_repeat_4plex.raw
117190174	High		lkGDDLQAIk	Unambiguous	5	1	N-Term(iTRAQ4plex); K2(iTRAQ4plex); K10(iTRAQ4plex)	HCD	1.0000	0.0000	1	1	8273	Unique	Used	0.324	4.969E5	1.765E5	182	3.87	0.00	1	13	1	3	511.64252	1532.91300	-15.82	28.29	2749	2749	MS2	20/36	Glioma_Nuclear_prep_Gradeall_4plex.raw
117190174	High		lkGDDLQAIk	Unambiguous	5	1	N-Term(iTRAQ4plex); K2(iTRAQ4plex); K10(iTRAQ4plex)	HCD	1.0000	0.0000	1	1	8264	Unique	Used	0.484	3.822E5	2.030E5	193	3.27	0.00	1	29	8	2	766.95898	1532.91069	-17.33	28.23	2739	2739	MS2	17/18	Glioma_Nuclear_prep_Gradeall_4plex.raw
117190174		High	qkVDSLLENLEk	Unambiguous	5	1	N-Term(iTRAQ4plex); K2(iTRAQ4plex); K12(iTRAQ4plex)	HCD	1.0000	0.0000	1	1	10897	Unique	Used	0.315	4.747E3	1.631E3	100	5.09	0.00	1	29	300	2	924.54694	1848.08660	3.41	55.90	5420	5420	MS2	22/22	Glioma_Nuclear_prep_Gradeall_repeat_4plex.raw

117190174		High	qkVDSLLENLEk	Unambiguous	5	1	N-Term(iTRAQ4plex); K2(iTRAQ4plex); K12(iTRAQ4plex)	HCD	1.0000	0.0000	1	1	10879	Unique	Used		0.387	2.118E3	8.934E2	98	5.02	0.00	1	50	300	3	616.70001	1848.08548	2.81	55.67	5400	5400	MS2	17/44	Glioma_Nuclear_prep_Gradeall_repeat_4plex.raw
117190174	High		qkVDSLLENLEk	Unambiguous	5	1	N-Term(iTRAQ4plex); K2(iTRAQ4plex); K12(iTRAQ4plex)	HCD	1.0000	0.0000	1	1	9946	Unique	Used		0.266	2.833E5	8.286E4	157	4.74	0.00	1	26	11	2	924.53107	1848.05486	-13.76	40.47	4571	4571	MS2	22/22	Glioma_Nuclear_prep_Gradeall_4plex.raw
117190174	High		qkVDSLLENLEk	Unambiguous	5	1	N-Term(iTRAQ4plex); K2(iTRAQ4plex); K12(iTRAQ4plex)	HCD	1.0000	0.0000	1	1	9943	Unique	Used		0.302	7.636E4	2.526E4	155	4.45	0.00	1	37	4	3	616.68970	1848.05454	-13.93	40.45	4568	4568	MS2	18/44	Glioma_Nuclear_prep_Gradeall_4plex.raw
117190174		High	sDVEAIFSk	Unambiguous	5	1	N-Term(iTRAQ4plex); K9(iTRAQ4plex)	HCD	1.0000	0.0000	1	1	10143	Unique	Used		0.324	2.030E4	7.174E3	226	3.16	0.00	0	43	300	2	642.36163	1283.71599	5.77	46.80	4608	4608	MS2	15/16	Glioma_Nuclear_prep_Gradeall_repeat_4plex.raw
117190174	High		sDVEAIFSk	Unambiguous	5	1	N-Term(iTRAQ4plex); K9(iTRAQ4plex)	HCD	1.0000	0.0000	1	1	9533	Unique	Used		0.279	4.734E5	1.451E5	231	3.67	0.00	0	43	8	2	642.35071	1283.69414	-11.25	37.49	4123	4123	MS2	15/16	Glioma_Nuclear_prep_Gradeall_4plex.raw
117190174	High		gDDQLELIkDDEk	Unambiguous	2	1	N-Term(iTRAQ4plex); K9(iTRAQ4plex); K13(iTRAQ4plex)	HCD	1.0000	0.0000	1	1	8874	Unique	Used		0.519	6.100E4	3.473E4	159	4.57	0.00	1	0	14	2	975.51056	1950.01384	-13.02	32.62	3402	3402	MS2	21/24	Glioma_Nuclear_prep_Gradeall_4plex.raw
117190174		High	mIAGQVLDINLAAEPk	Unambiguous	2	1	N-Term(iTRAQ4plex); M1(Oxidation); K16(iTRAQ4plex)	HCD	1.0000	0.0000	1	1	11376	Unique	Used		0.375	9.809E2	4.012E2	142	4.64	0.00	0	45	300	3	663.04395	1987.11728	1.80	62.47	5984	5984	MS2	23/60	Glioma_Nuclear_prep_Gradeall_repeat_4plex.raw
117190174		High	mIAGQVLDINLAAEPk	Unambiguous	2	1	N-Term(iTRAQ4plex); M1(Oxidation); K16(iTRAQ4plex)	HCD	1.0000	0.0000	1	1	11389	Unique	Used		0.508	2.519E3	1.395E3	149	6.14	0.00	0	20	300	2	994.06183	1987.11638	1.34	62.64	5999	5999	MS2	25/30	Glioma_Nuclear_prep_Gradeall_repeat_4plex.raw
117190174	High		mIAGQVLDINLAAEPk	Unambiguous	2	1	N-Term(iTRAQ4plex); M1(Oxidation); K16(iTRAQ4plex)	HCD	1.0000	0.0000	1	1	10573	Unique	Used		0.474	1.322E5	6.883E4	192	5.83	0.00	0	7	13	2	994.04657	1987.08586	-14.02	45.19	5256	5256	MS2	25/30	Glioma_Nuclear_prep_Gradeall_4plex.raw
117190174	High		sAAEmYGSSFDLDYDFQR	Unambiguous	1	1	N-Term(iTRAQ4plex); M5(Oxidation)	HCD	1.0000	0.0000	1	1	10879	Unique	Used		0.479	4.129E4	2.170E4	16	5.78	0.00	0	33	49	2	1131.47522	2261.94316	-15.32	47.51	5589	5589	MS2	22/34	Glioma_Nuclear_prep_Gradeall_4plex.raw
118601081	NP_001073027.1	HNRNPUL2	221092	heterogeneous nuclear ribonucleoprotein U-like protein 2 [Homo sapiens]	23.96	1	15	23	0.408	14;9	25.6	76.08	747	85.1	4.91																				
118601081	High		IQEALDAEMLEDEAGGGGAGPGGAck	Unambiguous	1	1	N-Term(iTRAQ4plex); C25(Methylthio); K26(iTRAQ4plex)	HCD	1.0000	0.0000	1	1	11353	Unique	Used		0.381	8.650E3	3.614E3	64	9.04	0.00	0	15	29	3	927.43158	2780.28019	0.75	51.35	6107	6107	MS2	39/100	Glioma_Nuclear_prep_Gradeall_4plex.raw
118601081		High	nYYGYQGYR	Unambiguous	1	1	N-Term(iTRAQ4plex)	HCD	1.0000	0.0000	1	1	8674	Unique	Used		0.311	3.760E4	1.273E4	89	2.94	0.00	0	23	211	2	664.31488	1327.62248	2.81	30.40	3036	3036	MS2	13/16	Glioma_Nuclear_prep_Gradeall_repeat_4plex.raw
118601081	High		eEPFFPPPEEFVFIHAVPVEER	Unambiguous	1	1	N-Term(iTRAQ4plex)	HCD	1.0000	0.0000	1	1	12730	Unique	Used		0.325	2.730E3	9.735E2	125	7.95	0.00	0	5	258	3	929.14105	2785.40860	3.26	65.38	7631	7631	MS2	28/84	Glioma_Nuclear_prep_Gradeall_4plex.raw
118601081	High		yNVLGAETVLNQMR	Unambiguous	1	1	N-Term(iTRAQ4plex)	HCD	1.0000	0.0000	1	1	11296	Unique	Used		0.334	9.903E4	3.626E4	239	5.17	0.00	0	24	51	2	876.46490	1751.92253	-0.34	50.83	6044	6044	MS2	22/26	Glioma_Nuclear_prep_Gradeall_4plex.raw
118601081	High		eEPFFPPPEEFVFIHAVPVEER	Unambiguous	1	1	N-Term(iTRAQ4plex)	HCD	1.0000	0.0000	1	1	12782	Unique	Used		0.441	1.261E3	6.105E2	125	7.71	0.00	0	9	300	3	929.14111	2785.40879	3.33	66.11	7693	7693	MS2	30/84	Glioma_Nuclear_prep_Gradeall_4plex.raw
118601081	High		cDYMDEVTYGELEk	Unambiguous	1	1	N-Term(iTRAQ4plex); C1(Methylthio); K14(iTRAQ4plex)	HCD	1.0000	0.0000	1	1	11032	Unique	Used		0.305	4.841E4	1.619E4	30	6.28	0.00	0	23	61	2	1014.94659	2028.88591	0.78	48.68	5755	5755	MS2	22/26	Glioma_Nuclear_prep_Gradeall_4plex.raw
118601081	High		cDYMDEVTYGELEkEEAQPIVTk	Unambiguous	1	1	N-Term(iTRAQ4plex); C1(Methylthio); K14(iTRAQ4plex); K23(iTRAQ4plex)	HCD	1.0000	0.0000	1	1	11240	Unique	Used		0.638	7.133E1	4.997E1	84	6.62	0.00	1	21	20	4	792.88647	3168.52407	2.80	50.36	5983	5983	MS2	30/132	Glioma_Nuclear_prep_Gradeall_4plex.raw
118601081		High	dLLVQQASQcLSk	Unambiguous	1	1	N-Term(iTRAQ4plex); C10(Methylthio); K13(iTRAQ4plex)	HCD	1.0000	0.0000	1	1	11979	Unique	Used		0.674	6.196E2	4.553E2	241	4.92	0.00	0	19	300	3	589.65210	1766.94175	1.92	71.65	6751	6751	MS2	23/48	Glioma_Nuclear_prep_Gradeall_repeat_4plex.raw
118601081	High		dLLVQQASQcLSk	Unambiguous	1	1	N-Term(iTRAQ4plex); C10(Methylthio); K13(iTRAQ4plex)	HCD	1.0000	0.0000	1	1	11239	Unique	Used		0.442	1.114E5	5.409E4	242	5.68	0.00	0	20	33	2	883.97406	1766.94084	1.41	50.36	5982	5982	MS2	20/24	Glioma_Nuclear_prep_Gradeall_4plex.raw
118601081	High		dLLVQQASQcLSk	Unambiguous	1	1	N-Term(iTRAQ4plex); C10(Methylthio); K13(iTRAQ4plex)	HCD	1.0000	0.0000	1	1	11236	Unique	Used		0.503	1.750E4	9.659E3	196	6.28	0.00	0	30	15	3	589.65155	1766.94010	0.99	50.34	5979	5979	MS2	22/48	Glioma_Nuclear_prep_Gradeall_4plex.raw
118601081	High		ncVVELNFGQk	Unambiguous	1	1	N-Term(iTRAQ4plex); C2(Methylthio); K11(iTRAQ4plex)	HCD	1.0000	0.0000	1	1	10517	Unique	Used		0.572	1.243E5	7.798E4	200	4.16	0.00	0	68	45	2	792.89911	1584.79094	-13.09	44.77	5195	5195	MS2	16/20	Glioma_Nuclear_prep_Gradeall_4plex.raw
118601081		High	eGcTEVSLLR	Unambiguous	1	1	N-Term(iTRAQ4plex); C3(Methylthio)	HCD	1.0000	0.0000	1	1	10444	Unique	Used		0.586	1.101E4	7.029E3	147	3.64	0.00	0	36	300	2	648.82544	1296.64360	2.10	50.35	4928	4928	MS2	17/18	Glioma_Nuclear_prep_Gradeall_repeat_4plex.raw
118601081		High	aLLPHVLck	Unambiguous	1	1	N-Term(iTRAQ4plex); C8(Methylthio); K9(iTRAQ4plex)	HCD	1.0000	0.0000	1	1	10773	Unique	Used		0.490	9.844E3	5.261E3	218	3.05	0.00	0	33	300	2	664.39722	1327.78716	2.92	54.33	5283	5283	MS2	15/16	Glioma_Nuclear_prep_Gradeall_repeat_4plex.raw
118601081		High	yGGQPLFSEk	Unambiguous	1	1	N-Term(iTRAQ4plex); K10(iTRAQ4plex)	HCD	1.0000	0.0000	1	1	9483	Unique	Used		0.408	3.905E4	1.738E4	237	3.57	0.00	0	22	147	2	707.38641	1413.76555	2.75	39.07	3894	3894	MS2	17/18	Glioma_Nuclear_prep_Gradeall_repeat_4plex.raw
118601081		High	gLEEPEMDPk	Unambiguous	1	1	N-Term(iTRAQ4plex); K10(iTRAQ4plex)	HCD	1.0000	0.0000	1	1	8632	Unique	Used		0.432	1.517E4	7.141E3	166	3.48	0.00	0	32	245	2	716.86780	1432.72832	3.53	29.96	2992	2992	MS2	17/18	Glioma_Nuclear_prep_Gradeall_repeat_4plex.raw
118601081	High		yGGQPLFSEk	Unambiguous	1	1	N-Term(iTRAQ4plex); K10(iTRAQ4plex)	HCD	1.0000	0.0000	1	1	8931	Unique	Used		0.364	4.446E5	1.774E5	196	2.93	0.00	0	25	6	2	707.37408	1413.74089	-14.70	33.03	3464	3464	MS2	17/18	Glioma_Nuclear_prep_Gradeall_4plex.raw
118601081	High		gLEEPEMDPk	Unambiguous	1	1	N-Term(iTRAQ4plex); K10(iTRAQ4plex)	HCD	1.0000	0.0000	1	1	8325	Unique	Used		0.407	2.645E5	1.182E5	106	3.42	0.00	0	13	7	2	716.85431	1432.70134	-15.30	28.66	2805	2805	MS2	17/18	Glioma_Nuclear_prep_Gradeall_4plex.raw
118601081	High		sGDETPGSEVPGDk	Unambiguous	1	1	N-Term(iTRAQ4plex); K14(iTRAQ4plex)	HCD	1.0000	0.0000	1	1	7500	Unique	Used		0.284	1.575E5	4.911E4	103	4.29	0.00	0	0	10	2	831.90533	1662.80339	-1.66	22.82	1911	1911	MS2	21/26	Glioma_Nuclear_prep_Gradeall_4plex.raw
118601081		High	aNFSLPEk	Unambiguous	1	1	N-Term(iTRAQ4plex); K8(iTRAQ4plex)	HCD	1.0000	0.0000	1	1	9336	Unique	Used		0.489	3.260E4	1.736E4	335	3.23	0.00	0	65	300	2	597.34332	1193.67937	2.09	37.47	3737	3737	MS2	13/14	Glioma_Nuclear_prep_Gradeall_repeat_4plex.raw
118601081	High		aNFSLPEk	Unambiguous	1	1	N-Term(iTRAQ4plex); K8(iTRAQ4plex)	HCD	1.0000	0.0000	1	1	8797	Unique	Used		0.481	2.792E5	1.473E5	329	2.89	0.00	0	43	15	2	597.33081	1193.65434	-18.88	32.07	3319	3319	MS2	13/14	Glioma_Nuclear_prep_Gradeall_4plex.raw
118601081		High	eEAQPIVTk	Unambiguous	1	1	N-Term(iTRAQ4plex); K9(iTRAQ4plex)	HCD	1.0000	0.0000	1	1	7637	Unique	Used		0.385	2.652E4	1.112E4	223	4.09	0.00	0	35	131	2	651.87506	1302.74285	-6.09	20.28	1941	1941	MS2	16/16	Glioma_Nuclear_prep_Gradeall_repeat_4plex.raw
118601081		High	yNVLGAETVLNQmR	Unambiguous	1	1	N-Term(iTRAQ4plex); M13(Oxidation)	HCD	1.0000	0.0000	1	1	10985	Unique	Used		0.291	8.292E3	2.630E3	222	3.89	0.00	0	8	300	2	884.46552	1767.92375	3.23	56.96	5515	5515	MS2	20/26	Glioma_Nuclear_prep_Gradeall_repeat_4plex.raw
118601081	High		yNVLGAETVLNQmR	Unambiguous	1	1	N-Term(iTRAQ4plex); M13(Oxidation)	HCD	1.0000	0.0000	1	1	10189	Unique	Used		0.363	2.023E5	8.050E4	189	3.66	0.00	0	10	16	2	884.45190	1767.89653	-12.17	42.27	4836	4836	MS2	19/26	Glioma_Nuclear_prep_Gradeall_repeat_4plex.raw
122937227	NP_001073884.1	U2SURP	23350	U2 snRNP-associated SURP motif-containing protein [Homo sapiens]	2.53	1	2	2	1.489	1;1	14.1	3.59	1029	118.2	8.47																				
122937227	High		nLLALIHR	Unambiguous	1	1	N-Term(iTRAQ4plex)	HCD	1.0000	0.0000	1	1	10012	Unique	Used		1.637	6.679E3	1.200E4	108	3.59	0.00	0	47	35	3	365.23114	1093.67887	-15.91	40.93	4642	4642	MS2	19/28	Glioma_Nuclear_prep_Gradeall_4plex.raw
122937227		High	sLDDDLDGVPLDATEDSk	Unambiguous	1	1	N-Term(iTRAQ4plex); K18(iTRAQ4plex)	HCD	1.0000	0.0000	1	1	10896	Unique	Used		1.340	3.131E2	4.574E2	127	3.39	0.00	0	19	300	3	731.69464	2193.06937	2.01	55.89	5419	5419	MS2	19/68	Glioma_Nuclear_prep_Gradeall_repeat_4plex.raw
125656165	NP_001075019.1	THOC2	57187	THO complex subunit 2 [Homo sapiens]	3.58	1	4	4	1.487	2;2	10.8	13.23	1593	182.7	8.44																				
125656165		High	vLNLGQALER	Unambiguous	1	1	N-Term(iTRAQ4plex)	HCD	1.0000	0.0000	1	1	10796	Unique	Used		1.402	3.197E3	4.884E3	239	2.94	0.00	0	58	300	2	628.87592	1256.74455	0.16	54.61	5308	5308	MS2	15/18	Glioma_Nuclear_prep_Gradeall_repeat_4plex.raw
125656165	High		IDPETLESLGLIk	Unambiguous	1	1	N-Term(iTRAQ4plex); K13(iTRAQ4plex)	HCD	1.0000	0.0000	1	1	12091	Unique	Used		1.557	8.896E3	1.520E4	149	5.13	0.00	0	39	300	2	858.50769	1716.00810	2.75	57.92	6912	6912	MS2	22/24	Glioma_Nuclear_prep_Gradeall_4plex.raw
125656165		High	sFDLLILk	Unambiguous	1	1	N-Term(iTRAQ4plex); K8(iTRAQ4plex)	HCD	1.0000	0.0000	1	1	12167	Unique	Used		1.417	2.026E3	3.128E3	129	3.23	0.00	0	45	300	2	618.89301	1236.77873	-1.52	75.40	7044	7044	MS2	14/14	Glioma_Nuclear_prep_Gradeall_repeat_4plex.raw
125656165	High		IGLLEALLk	Unambiguous	1	1	N-Term(iTRAQ4plex); K9(iTRAQ4plex)	HCD	1.0000	0.0000	1	1	12931	Unique	Used		1.760	4.312E3	8.326E3	41	3.75	0.00	0	46	300	2	629.42255	1257.83782	-0.53	68.51	7887	7887	MS2	16/16	Glioma_Nuclear_prep_Gradeall_4plex.raw
134152683	NP_001077059.1	TMEM214	54867	transmembrane protein 214 isoform 2 [Homo sapiens]	6.21	2	1	2	0.525	1;1	5.6	14.68	644	72.0	9.26																				
134152683		High	sLSPFAITYLDR	Unambiguous	2	1	N-Term(iTRAQ4plex)	HCD	1.0000	0.0000	1	1	12111	Unique	Used		0.504	2.287E3	1.255E3	275	3.81	0.00	0	42	300	2	763.92157	1526.83586	1.51	74.43	6965	6965	MS2	18/22	Glioma_Nuclear_prep_Gradeall_repeat_4plex.raw
134152683	High		sLSPFAITYLDR	Unambiguous	2	1	N-Term(iTRAQ4plex)	HCD	1.0000	0.0000	1	1	11411	Unique	Used		0.545	3.297E4	1.973E4	286	4.45	0.00	0	47	124	2	763.91827	1526.82927	-2.81	51.83	6170	6170	MS2	18/22	Glioma_Nuclear_prep_Gradeall_4plex.raw
151101292	NP_110382.3	TMX1	81542	thioredoxin-related transmembrane protein 1 precursor [Homo sapiens]	7.86	1	2	3	2.917	2;1	28.7	11.00	280	31.8	4.98																				
151101292	High		fIITALPTIYHck	Unambiguous	1	1	N-Term(iTRAQ4plex); C12(Methylthio); K13(iTRAQ4plex)	HCD	1.0000	0.0000	1	1	11820	Unique	Used		3.961	6.536E3	2.841E4	208	7.00	0.00	0	23	31	3	618.67883	1854.02195	-2.20	55.38	6616	6616	MS2	28/48	Glioma_Nuclear_prep_Gradeall_4plex.raw
151101292		High	dFINFISDk	Unambiguous	1	1	N-Term(iTRAQ4plex); K9(iTRAQ4plex)	HCD	1.0000	0.0000	1	1	11690	Unique	Used		2.230	1.767E3	4.295E3	278	3.88	0.00	0	45	300	2	693.87970	1386.75212	0.97	66.84	6357	6357	MS2	16/16	Glioma_Nuclear_prep_Gradeall_repeat_4plex.raw
151101292	High		dFINFISDk	Unambiguous	1	1	N-Term(iTRAQ4plex); K9(iTRAQ4plex)	HCD	1.0000	0.0000	1	1	10895	Unique	Used		2.917	6.505E4	2.082E5	230	4.00	0.00	0	38	20	2	693.86829	1386.72930	-15.49	47.65	5607	5607	MS2	16/16	Glioma_Nuclear_prep_Gradeall_4plex.raw
153070260	NP_002347.5	MARCKS	4082	myristoylated alanine-rich C-kinase substrate [Homo sapiens]	15.06	1	3	4	3.951	1;3	32.2	16.50	332	31.5	4.45																				
153070260		High	aEDGATPSPSNETPk	Unambiguous	1	1	N-Term(iTRAQ4plex); K15(iTRAQ4plex)	HCD	1.0000	0.0000	1	1	7285	Unique	Used		3.662	5.456E2	2.178E3	78	3.96	0.00	0	41	300	2	894.93042	1788.85356	-17.83	17.25	1569	1569	MS2	19/28	Glioma_Nuclear_prep_Gradeall_repeat_4plex.raw
153070260		High	eAGEGGEAEAPAAEGGk	Unambiguous	1	1	N-Term(iTRAQ4plex); K17(iTRAQ4plex)	HCD	1.0000	0.0000	1	1	7209	Unique	Used		4.241	3.143E2	1.453E3	54	4.08	0.00	0	10	300	2	909.42712	1817.84697	-15.78	16.61	1489	1489	MS2	28/32	Glioma_Nuclear_prep_Gradeall_repeat_4plex.raw
153070260		High	gEPAAAAAPEAGASPVEk	Unambiguous	1	1	N-Term(iTRAQ4plex); K18(iTRAQ4plex)	HCD	1.0000	0.0000	1	1	8212	Unique	Used		2.792	1.111E3	3.381E3	166	4.99	0.00	0	0	300	2	956.00629	1911.00530	-0.51	25.84	2549	2549	MS2	19/34	Glioma_Nuclear_prep_Gradeall_repeat_4plex.raw
153070260	High		gEPAAAAAPEAGASPVEk	Unambiguous	1	1	N-Term(iTRAQ4plex); K18(iTRAQ4plex)	HCD	1.0000	0.0000	1	1	8059	Unique	Used		5.970	1.434E4	9.392E4	169	5.71	0.00	0	11	14	2	955.98950	1910.97173	-18.08	26.78	2517	2517	MS2	26/34	Glioma_Nuclear_prep_Gradeall_4plex.raw
153252132	NP_001093163.1	RPL31	6160	60S ribosomal protein L31 isoform 3 [Homo sapiens]	18.18	3	2	3	0.532	2;1	9.0	13.71	121	14.0	10.80																				
153252132	High		IYTLVTYVPVTTFk	Unambiguous	3	1	N-Term(iTRAQ4plex); K14(iTRAQ4plex)	HCD	1.0000	0.0000	1	1	12318	Unique	Used		0.503	2.060E4	1.138E4	126	4.99	0.00	0	22	146	2	967.06573	1933.12419	-2.43	60.23	7162	7162	MS2	21/26	Glioma_Nuclear_prep_Gradeall_4plex.raw
153252132	High		IYTLVTYVPVTTFk	Unambiguous	3	1	N-Term(iTRAQ4plex); K14(iTRAQ4plex)	HCD	1.0000	0.0000	1	1	12306	Unique	Used		0.532	2.575E3	1.502E3	107	3.87	0.00	0	23	108	3	645.04718	1933.12699	-0.98	60.10	7149	7149	MS2	19/52	Glioma_Nuclear_prep_Gradeall_4plex.raw
153252132		High	eYTINIHk	Unambiguous	3	1	N-Term(iTRAQ4plex); K8(iTRAQ4plex)	HCD	1.0000	0.0000	1	1	8633	Unique	Used		0.599	4.234E3	2.765E3	314	3.42	0.00	0	36	196	3	435.91925	1305.74320	2.05	29.97	2993	2993	MS2	17/28	Glioma_Nuclear_prep_Gradeall_repeat_4plex.raw
156523968	NP_001609.2	PARP1	142	poly [ADP-ribose] polymerase 1 [Homo sapiens]	21.60	1	13	23	1.690	11;12	22.9	90.89	1014	113.0	8.88																				
156523968		High	tTNFAGILSQGLR	Unambiguous	1	1	N-Term(iTRAQ4plex)	HCD	1.0000	0.0000	1	1	11840	Unique	Used		1.530	1.729E3	2.883E3	247	4.59	0.00	0	32	300	2	761.42950	1521.85173	0.73	69.28	6558	6558	MS2	21/24	Glioma_Nuclear_prep_Gradeall_repeat_4plex.raw
156523968		High	aEPVEVVAPR	Unambiguous	1	1	N-Term(iTRAQ4plex)	HCD	1.0000	0.0000	1	1	8392	Unique	Used		1.734	4.176E4	7.893E4	151	3.24	0.00	0	7	51	2	605.84784	1210.68840	-2.36	27.59	2739	2739	MS2	17/18	Glioma_Nuclear_prep_Gradeall_repeat_4plex.raw
156523968		High	qQVPSGESAILDR	Unambiguous	1	1	N-Term(iTRAQ4plex)	HCD	1.0000	0.0000	1	1	8978	Unique	Used		1.943	3.693E3	7.820E3	260	4.07	0.00	0	22	300	2	772.41547	1543.82366	2.55	33.45	3357	3357	MS2	18/24	Glioma_Nuclear_prep_Gradeall_repeat_4plex.raw
156523968	High		tTNFAGILSQGLR	Unambiguous	1	1	N-Term(iTRAQ4plex)	HCD	1.0000	0.0000	1	1	11015	Unique	Used		1.701	7.952E4	1.484E5	234	5.01	0.00	0	17	17	2	761.42804	1521.84880	-1.19	48.56	5737	5737	MS2	20/24	Glioma_Nuclear_prep_Gradeall_4plex.raw
156523968	High		qQVPSGESAILDR	Unambiguous	1	1	N-Term(iTRAQ4plex)	HCD	1.0000	0.0000	1	1	8621	Unique	Used		1.764	7.909E4	1.531E5	240	3.11	0.00	0	9	15	2	772.40070	1543.79411	-16.58	30.81	3128	3128	MS2	17/24	Glioma_Nuclear_prep_Gradeall_4plex.raw
156523968		High	kGDEVDGVDEVAk	Unambiguous	1	1	N-Term(iTRAQ4plex); K1(iTRAQ4plex); K13(iTRAQ4plex)	HCD	1.0000	0.0000	1	1	7911	Unique	Used		1.366	2.108E3	3.137E3	222	4.74	0.00	1	38	300	2	896.97998	1792.95268	-7.06	22.92	2232	2232	MS2	20/24	Glioma_Nuclear_prep_Gradeall_repeat_4plex.raw
156523968		High	kGDEVDGVDEVAk	Unambiguous	1	1	N-Term(iTRAQ4plex); K1(iTRAQ4plex); K13(iTRAQ4plex)	HCD	1.0000	0.0000	1	1	7887	Unique	Used		1.680	2.163E3	3.959E3	201	6.18	0.00	1	29	77	3	598.32269	1792.95353	-6.59	22.70	2207	2207	MS2	28/48	Glioma_Nuclear_prep_Gradeall_repeat_4plex.raw
156523968	High		kGDEVDGVDEVAk	Unambiguous	1	1	N-Term(iTRAQ4plex); K1(iTRAQ4plex); K13(iTRAQ4plex)	HCD	1.0000	0.0000	1	1	7765	Unique	Used		1.384	7.422E4	1.127E5	178	5.02	0.00	1	11	11	2	896.98718	1792.96709	0.98	24.69	2198	2198	MS2	21/24	Glioma_Nuclear_prep_Gradeall_4plex.raw
156523968	High		kGDEVDGVDEVAk	Unambiguous	1	1	N-Term(iTRAQ4plex); K1(iTRAQ4plex); K13(iTRAQ4plex)	HCD	1.0000	0.0000	1	1	7761	Unique	Used		3.041	3.940E4	1.315E5	164	4.46	0.00	1	48	3	3	598.32654	1792.96506	-0.16	24.67	2194	2194	MS2	26/48	Glioma_Nuclear_prep_Gradeall_4plex.raw
156523968		High	kPPLLNNADSVQAk	Unambiguous	1	1	N-Term(iTRAQ4plex); K1(iTRAQ4plex); K14(iTRAQ4plex)	HCD	1.0000	0.0000	1	1	8670	Unique	Used		1.372	2.771E3	4.144E3	70	6.99	0.00	0	29	101	3	643.04919	1927.13303	-0.36	30.37	3032	3032	MS2	24/52	Glioma_Nuclear_prep_Gradeall_repeat_4plex.raw
156523968	High		kPPLLNNADSVQAk	Unambiguous	1	1	N-Term(iTRAQ4plex); K1(iTRAQ4plex); K14(iTRAQ4plex)	HCD	1.0000	0.0000	1	1	8255	Unique	Used		1.690	2.848E4	5.280E4	118	4.21	0.00	0	20	6	3	643.03717	1927.09696	-19.08	28.16	2729	2729	MS2	24/52	Glioma_Nuclear_prep_Gradeall_4plex.raw
156523968		High	gFSLLATEDk	Unambiguous	1	1	N-Term(iTRAQ4plex); K10(iTRAQ4plex)	HCD	1.0000	0.0000	1	1	10304	Unique	Used		1.674	1.028E4	1.874E4	226	4.35	0.00	0	17	220	2	684.88501	1368.76274	1.02	48.72	4780	4780	MS2	18/18	Glioma_Nuclear_prep_Gradeall_repeat_4plex.raw
156523968		High	gIYFADMVSk	Unambiguous	1	1	N-Term(iTRAQ4plex); K10(iTRAQ4plex)	HCD	1.0000	0.0000	1	1	11087	Unique	Used		1.683	3.374E3	6.190E3	269	3.98	0.00	0	62	300	2	709.88293	1418.75859	-0.45	58.28	5630	5630	MS2	17/18	Glioma_Nuclear_prep_Gradeall_repeat_4plex.raw
156523968	High		gFSLLATEDk	Unambiguous	1	1	N-Term(iTRAQ4plex); K10(iTRAQ4plex)	HCD	1.0000	0.0000	1	1	9615	Unique	Used		1.479	3.126E5	5.074E5	232	3.52	0.00	0	20	4	2	684.87335	1368.73943	-16.01	38.08	4212	4212	MS2	18/18	Glioma_Nuclear_prep_Gradeall_4plex.raw
156523968	High		gIYFADMVSk	Unambiguous	1	1	N-Term(iTRAQ4plex); K10(iTRAQ4plex)	HCD	1.0000	0.0000	1	1	10262	Unique	Used		1.900	8.083E4	1.685E5	220	3.09	0.00	0	35	15	2	709.87207	1418.73686	-15.77	42.80	4915	4915	MS2	17/18	Glioma_Nuclear_prep_Gradeall_4plex.raw
156523968		High	tLGDFAAEYAk	Unambiguous	1	1	N-Term(iTRAQ4plex); K11(iTRAQ4plex)	HCD	1.0000	0.0000	1	1	10762	Unique	Used		1.555	8.247E3	1.398E4	208	4.48	0.00	0	20	300	2	737.39630	1473.78533	1.71	54.19	5271	5271	MS2	20/20	Glioma_Nuclear_prep_Gradeall_repeat_4plex.raw
156523968	High		tLGDFAAEYAk	Unambiguous	1	1	N-Term(iTRAQ4plex); K11(iTRAQ4plex)	HCD	1.0000	0.0000	1	1	10028	Unique	Used		1.380	2.123E5	3.215E5	198	4.32	0.00	0	20	11	2	737.38245	1473.75762	-17.09	41.06	4660	4660	MS2	20/20	Glioma_Nuclear_prep_Gradeall_4plex.raw
156523968	High		fYTLIPHDFGMk	Unambiguous	1	1	N-Term(iTRAQ4plex); K12(iTRAQ4plex)	HCD	1.0000	0.0000	1	1	11156	Unique	Used		1.795	9.336E3	1.839E4	158	3.94	0.00	0	77	31	3	586.31543	1756.93174	-1.01	49.67	5891	5891	MS2	24/44	Glioma_Nuclear_prep_Gradeall_4plex.raw
156523968		High	vVSEDFLQDVSASTk	Unambiguous	1	1	N-Term(iTRAQ4plex); K15(iTRAQ4plex)	HCD	1.0000	0.0000	1	1	11518	Unique	Used		2.371	1.050E3	2.714E3	186	6.33	0.00	0	7	300	2	957.01007	1913.01286	1.15	64.36	6148	6148	MS2	25/28	Glioma_Nuclear_prep_Gradeall_repeat_4plex.raw
156523968	High		vVSEDFLQDVSASTk	Unambiguous	1	1	N-Term(iTRAQ4plex); K15(iTRAQ4plex)	HCD	1.0000	0.0000	1	1	10754	Unique	Used		2.173	8.154E4	1.944E5	166	6.57	0.00	0	10	11	2	956.99445	1912.98161	-15.18	46.57	5454	5454	MS2	24/28	Glioma_Nuclear_prep_Gradeall_4plex.raw
156523968		High	eLLIFNk	Unambiguous	1	1	N-Term(iTRAQ4plex); K7(iTRAQ4plex)	HCD	1.0000	0.0000	1	1	10911	Unique	Used		2.325	6.297E3	1.596E4	265	3.11	0.00	0	41	300	2	582.86475	1164.72222	-0.76	56.08	5436	5436	MS2	12/12	Glioma_Nuclear_prep_Gradeall_repeat_4plex.raw
156523968		High	ITVNPGTk	Unambiguous	1	1	N-Term(iTRAQ4plex); K8(iTRAQ4plex)	HCD	1.0000	0.0000	1	1	7846	Unique	Used		1.285	2.322E4	3.252E4	313	3.21	0.00	0	51	119	2	559.34167	1117.67607	-5.26	22.32	2164	2164	MS2	11/14	Glioma_Nuclear_prep_Gradeall_repeat_4plex.raw
156523968	High		mIFDVESMk	Unambiguous	1	1	N-Term(iTRAQ4plex); K9(iTRAQ4plex)	HCD	1.0000	0.0000	1	1	10629	Unique	Used		1.714	1.268E5	2.386E5	125	3.51	0.00	0	18	11	2	694.35297	1387.69866	-15.69	45.61	5317	5317	MS2	15/16	Glioma_Nuclear_prep_Gradeall_4plex.raw
157412270	NP_112480.2	HNRNPM	4670	heterogeneous nuclear ribonucleoprotein M isoform b [Homo sapiens]	20.26	3	8	13	0.504	7;6	20.7	41.50	691	73.6	8.82																				
157412270		High	gNFGGSFAGSFGGAGGHAPGVAR	Unambiguous	3	1	N-Term(iTRAQ4plex)	HCD	1.0000	0.0000	1	1	10150	Unique	Used		0.509	1.550E3	8.593E2	146	8.75	0.00	0	28	296	3	727.02515	2179.06089	2.66	46.87	4615	4615	MS2	39/88	Glioma_Nuclear_prep_Gradeall_repeat_4plex.raw
157412270		High	fESPEVAER	Unambiguous	3	1	N-Term(iTRAQ4plex)	HCD	1.0000	0.0000	1	1	8338	Unique	Used		0.375	3.179E4	1.300E4	178	3.22	0.00	0	44	182	2	604.31018	1207.61308	4.55	27.06	2682	2682	MS2	15/16	Glioma_Nuclear_prep_Gradeall_repeat_4plex.raw
157412270	High		mGPAMGPALGAGIER	Unambiguous	3	1	N-Term(iTRAQ4plex)	HCD	1.0000	0.0000	1	1	9606	Unique	Used		0.404	2.417E5	1.072E5	154	4.33	0.00	0	31	14	2	786.40015	1571.79302	-14.33	38.01	4202	4202	MS2	23/28	Glioma_Nuclear_prep_Gradeall_4plex.raw
157412270	High		mGPLGLDHMASSIER	Unambiguous	3	1	N-Term(iTRAQ4plex)	HCD	1.0000	0.0000	1	1	9975	Unique	Used		0.555	9.449E4	5.753E4	124	4.76	0.00	0	5	25	2	879.43085	1757.85442	-14.32	40.67	4602	4602	MS2	21/28	Glioma_Nuclear_prep_Gradeall_4plex.raw
157412270	High		mGPLGLDHMASSIER	Unambiguous	3	1	N-Term(iTRAQ4plex)	HCD	1.0000	0.0000	1	1	9957	Unique	Used		0.561	5.280E4	3.249E4	121	3.58	0.00	0	25	7	3	586.62225	1757.85221	-15.57	40.55	4583	4583	MS2	24/56	Glioma_Nuclear_prep_Gradeall_4plex.raw
157412270	High		gNFGGSFAGSFGGAGGHAPGVAR	Unambiguous	3	1	N-Term(iTRAQ4plex)	HCD	1.0000	0.0000	1	1	9412	Unique	Used		0.650	1.547E4	1.103E4	95	5.47	0.00	0	25	8	3	727.01166	2179.02042	-15.91	36.60	3991	3991	MS2	39/88	Glioma_Nuclear_prep_Gradeall_4plex.raw
157412270		High	iNEILSNALk	Unambiguous	3	1	N-Term(iTRAQ4plex); K10(iTRAQ4plex)	HCD	1.0000	0.0000	1	1	11475	Unique	Used		0.354	6.644E3	2.561E3	158	4.06	0.00	0	21	300	2	701.93066	1402.85405	2.30	63.83	6101	6101	MS2	18/18	Glioma_Nuclear_prep_Gradeall_repeat_4plex.raw
157412270		High	iNEILSNALk	Unambiguous	3	1	N-Term(iTRAQ4plex); K10(iTRAQ4plex)	HCD	1.0000	0.0000	1	1	11490	Unique	Used		0.494	4.458E2	2.400E2	137	3.62	0.00	0	21	300	3	468.29068	1402.85749	4.75	64.01	6117	6117	MS2	20/36	Glioma_Nuclear_prep_Gradeall_repeat_4plex.raw
157412270	High		iNEILSNALk	Unambiguous	3	1	N-Term(iTRAQ4plex); K10(iTRAQ4plex)	HCD	1.0000	0.0000	1	1	10597	Unique	Used		0.432	2.310E5	1.096E5	209	4.20	0.00	0	49	20	2	701.91925	1402.83122	-13.97	45.37	5282	5282	MS2	17/18	Glioma_Nuclear_prep_Gradeall_4plex.raw
157412270		High	aFITNIPFDVk	Unambiguous	2	1	N-Term(iTRAQ4plex); K11(iTRAQ4plex)	HCD	1.0000	0.0000	1	1	12031	Unique	Used		0.690	2.738E3	2.059E3	196	4.22	0.00	0	37	300	2	776.95386	1552.90044	1.71	72.57	6824	6824	MS2	17/20	Glioma_Nuclear_prep_Gradeall_repeat_4plex.raw
157412270	High		aFITNIPFDVk	Unambiguous	2	1	N-Term(iTRAQ4plex); K11(iTRAQ4plex)	HCD	1.0000	0.0000	1	1	11249	Unique	Used		0.504	3.509E4	1.940E4	214	4.46	0.00	0	35	49	2	776.95209	1552.89690	-0.57	50.43	5993	5993	MS2	17/20	Glioma_Nuclear_prep_Gradeall_4plex.raw
157412270		High	fEPYANPTk	Unambiguous	2	1	N-Term(iTRAQ4plex); K9(iTRAQ4plex)	HCD	1.0000	0.0000	1	1	8693	Unique	Used		0.522	4.249E4	2.415E4	238	3.44	0.00	0	16	147	2	677.87012	1354.73296	6.21	30.58	3056	3056	MS2	15/16	Glioma_Nuclear_prep_Gradeall_repeat_4plex.raw
157412270	High		nLPFDFTWk	Unambiguous	3	1	N-Term(iTRAQ4plex); K9(iTRAQ4plex)	HCD	1.0000	0.0000	1	1	11755	Unique	Used		0.397	7.123E4	3.104E4	277	3.87	0.00	0	44	73	2	728.39569	1455.78411	-2.33	54.83	6545	6545	MS2	13/16	Glioma_Nuclear_prep_Gradeall_4plex.raw
160707950	NP_001104262.1	MECP2	4204	methyl-CpG-binding protein 2 isoform 2 [Homo sapiens]	37.15	2	16	40	0.359	17;23	30.9	113.42	498	53.3	9.88																				
160707950		High	vGDTSLDPNDFDFTVTGR	Unambiguous	2	1	N-Term(iTRAQ4plex)	HCD	1.0000	0.0000	1	1	11776	Unique	Used		0.273	1.938E3	5.770E2	69	5.17	0.00	0	0	300	2	1050.50513	2100.00298	1.28	68.15	6467	6467	MS2	24/34	Glioma_Nuclear_prep_Gradeall_repeat_4plex.raw
160707950		High	vGDTSLDPNDFDFTVTGR	Unambiguous	2	1	N-Term(iTRAQ4plex)	HCD	1.0000	0.0000	1	1	11790	Unique	Used		0.458	5.034E2	2.513E2	97	5.01	0.00	0	31	292	3	700.67334	2100.00547	2.47	68.41	6488	6488	MS2	21/68	Glioma_Nuclear_prep_Gradeall_repeat_4plex.raw
160707950	High		vGDTSLDPNDFDFTVTGR	Unambiguous	2	1	N-Term(iTRAQ4plex)	HCD	1.0000	0.0000	1	1	11109	Unique	Used		0.202	1.150E5	2.550E4	66	6.00	0.00	0	6	20	2	1050.49988	2099.99248	-3.72	49.31	5840	5840	MS2	24/34	Glioma_Nuclear_prep_Gradeall_4plex.raw
160707950	High		vGDTSLDPNDFDFTVTGR	Unambiguous	2	1	N-Term(iTRAQ4plex)	HCD	1.0000	0.0000	1	1	11212	Unique	Used		0.413	1.967E4	8.923E3	73	5.92	0.00	0	36	94	2	1050.49500	2099.98271	-8.37	50.11	5952	5952	MS2	23/34	Glioma_Nuclear_prep_Gradeall_4plex.raw
160707950		High	gGSLESDGcPk	Unambiguous	2	1	N-Term(iTRAQ4plex); C9(Methylthio); K11(iTRAQ4plex)	HCD	1.0000	0.0000	1	1	8376	Unique	Used		0.333	1.846E4	6.707E3	87	4.00	0.00	0	28	209	2	692.33191	1383.65654	5.66	27.42	2722	2722	MS2	18/20	Glioma_Nuclear_prep_Gradeall_repeat_4plex.raw
160707950		High	gGSLESDGcPk	Unambiguous	2	1	N-Term(iTRAQ4plex); C9(Methylthio); K11(iTRAQ4plex)	HCD	1.0000	0.0000	1	1	8269	Unique	Used		0.498	8.687E3	4.717E3	102	3.57	0.00	0	42	300	2	692.33301	1383.65874	7.25	26.38	2609	2609	MS2	16/20	Glioma_Nuclear_prep_Gradeall_repeat_4plex.raw
160707950	High		gGSLESDGcPk	Unambiguous	2	1	N-Term(iTRAQ4plex); C9(Methylthio); K11(iTRAQ4plex)	HCD	1.0000	0.0000	1	1	8082	Unique	Used		0.311	3.124E5	1.066E5	50	4.34	0.00	0	37	11	2	692.31897	1383.63066	-13.04	26.94	2542	2542	MS2	19/20	Glioma_Nuclear_prep_Gradeall_4plex.raw
160707950	High		kAEADPQAIPk	Unambiguous	1	1	N-Term(iTRAQ4plex); K1(iTRAQ4plex); K11(iTRAQ4plex)	HCD	1.0000	0.0000	1	1	7425	Unique	Used		0.330	3.530E4	1.277E4	128	4.69	0.00	1	49	8	3	533.98468	1599.93949	-2.24	22.28	1830	1830	MS2	18/40	Glioma_Nuclear_prep_Gradeall_4plex.raw
160707950	High		kAEADPQAIPk	Unambiguous	1	1	N-Term(iTRAQ4plex); K1(iTRAQ4plex); K11(iTRAQ4plex)	HCD	1.0000	0.0000	1	1	7449	Unique	Used		0.338	1.861E5	6.910E4	139	3.60	0.00	1	9	7	2	800.47180	1599.93633	-4.21	22.46	1856	1856	MS2	17/20	Glioma_Nuclear_prep_Gradeall_4plex.raw
160707950		High	kPGSVVAAAAAAEAk	Unambiguous	2	1	N-Term(iTRAQ4plex); K1(iTRAQ4plex); K14(iTRAQ4plex)	HCD	1.0000	0.0000	1	1	8821	Unique	Used		0.320	1.792E4	6.242E3	90	5.70	0.00	0	25	300	2	851.51697	1702.02666	2.50	31.86	3191	3191	MS2	24/26	Glioma_Nuclear_prep_Gradeall_repeat_4plex.raw
160707950		High	kPGSVVAAAAAAEAk	Unambiguous	2	1	N-Term(iTRAQ4plex); K1(iTRAQ4plex); K14(iTRAQ4plex)	HCD	1.0000	0.0000	1	1	8801	Unique	Used		0.425	4.692E3	2.174E3	136	6.03	0.00	0	35	93	3	568.00439	1701.99863	-13.97	31.64	3170	3170	MS2	27/52	Glioma_Nuclear_prep_Gradeall_repeat_4plex.raw
160707950		High	kPGSVVAAAAAAEAk	Unambiguous	2	1	N-Term(iTRAQ4plex); K1(iTRAQ4plex); K14(iTRAQ4plex)	HCD	1.0000	0.0000	1	1	8824	Unique	Used		0.445	1.092E3	5.292E2	104	4.45	0.00	0	52	243	4	426.26117	1702.02285	0.26	31.89	3194	3194	MS2	22/78	Glioma_Nuclear_prep_Gradeall_repeat_4plex.raw
160707950		High	sEDQDLQGLk	Unambiguous	2	1	N-Term(iTRAQ4plex); K10(iTRAQ4plex)	HCD	1.0000	0.0000	1	1	8391	Unique	Used		0.219	8.212E4	1.956E4	180	4.20	0.00	0	4	52	2	710.87982	1420.75237	0.09	27.59	2738	2738	MS2	18/18	Glioma_Nuclear_prep_Gradeall_repeat_4plex.raw
160707950		High	aATSEGVQVk	Unambiguous	2	1	N-Term(iTRAQ4plex); K10(iTRAQ4plex)	HCD	1.0000	0.0000	1	1	7214	Unique	Used		0.360	7.557E4	2.963E4	244	3.07	0.00	0	11	44	2	639.35919	1277.71111	-15.08	16.66	1495	1495	MS2	18/18	Glioma_Nuclear_prep_Gradeall_repeat_4plex.raw
160707950		High	sVQETVLPIk	Unambiguous	2	1	N-Term(iTRAQ4plex); K10(iTRAQ4plex)	HCD	1.0000	0.0000	1	1	9777	Unique	Used		0.403	4.892E4	2.149E4	139	4.10	0.00	0	41	87	2	701.43262	1401.85796	1.70	42.41	4211	4211	MS2	16/18	Glioma_Nuclear_prep_Gradeall_repeat_4plex.raw
160707950		High	aATSEGVQVk	Unambiguous	2	1	N-Term(iTRAQ4plex); K10(iTRAQ4plex)	HCD	1.0000	0.0000	1	1	7225	Unique	Used		0.582	9.618E3	6.099E3	265	3.59	0.00	0	56	129	3	426.57532	1277.71140	-14.85	16.74	1506	1506	MS2	16/36	Glioma_Nuclear_prep_Gradeall_repeat_4plex.raw
160707950		High	aEADPQAIPk	Unambiguous	2	1	N-Term(iTRAQ4plex); K10(iTRAQ4plex)	HCD	1.0000	0.0000	1	1	7783	Unique	Used		0.614	1.381E4	9.238E3	268	3.28	0.00	0	23	155	2	664.37604	1327.74480	-0.93	21.71	2097	2097	MS2	15/18	Glioma_Nuclear_prep_Gradeall_repeat_4plex.raw
160707950	High		sEDQDLQGLk	Unambiguous	2	1	N-Term(iTRAQ4plex); K10(iTRAQ4plex)	HCD	1.0000	0.0000	1	1	8155	Unique	Used		0.256	8.328E5	2.335E5	144	4.25	0.00	0	10	4	2	710.86884	1420.73039	-15.37	27.46	2621	2621	MS2	18/18	Glioma_Nuclear_prep_Gradeall_4plex.raw
160707950	High		sVQETVLPIk	Unambiguous	2	1	N-Term(iTRAQ4plex); K10(iTRAQ4plex)	HCD	1.0000	0.0000	1	1	9182	Unique	Used		0.375	1.023E6	4.213E5	159	3.27	0.00	0	29	3	2	701.42194	1401.83660	-13.54	34.89	3739	3739	MS2	16/18	Glioma_Nuclear_prep_Gradeall_4plex.raw
160707950	High		mPFQTSPGGk	Unambiguous	2	1	N-Term(iTRAQ4plex); K10(iTRAQ4plex)	HCD	1.0000	0.0000	1	1	8372	Unique	Used		0.477	4.182E5	2.189E5	208	2.95	0.00	0	39	7	2	669.35095	1337.69463	-13.46	28.99	2856	2856	MS2	15/18	Glioma_Nuclear_prep_Gradeall_4plex.raw
160707950	High		aEADPQAIPk	Unambiguous	2	1	N-Term(iTRAQ4plex); K10(iTRAQ4plex)	HCD	1.0000	0.0000	1	1	7684	Unique	Used		0.553	1.020E5	6.188E4	263	3.18	0.00	0	23	15	2	664.37653	1327.74578	-0.20	24.12	2110	2110	MS2	16/18	Glioma_Nuclear_prep_Gradeall_4plex.raw
160707950		High	yDVYLINPQGk	Unambiguous	2	1	N-Term(iTRAQ4plex); K11(iTRAQ4plex)	HCD	1.0000	0.0000	1	1	10685	Unique	Used		0.587	3.599E3	2.301E3	185	3.99	0.00	0	23	166	3	533.30054	1597.88706	2.64	53.26	5188	5188	MS2	16/40	Glioma_Nuclear_prep_Gradeall_repeat_4plex.raw
160707950		High	tQPAVATAATAAEk	Unambiguous	2	1	N-Term(iTRAQ4plex); K14(iTRAQ4plex)	HCD	1.0000	0.0000	1	1	8136	Unique	Used		0.270	2.403E4	7.073E3	217	5.33	0.00	0	52	300	2	809.45740	1617.90752	1.51	25.09	2469	2469	MS2	21/26	Glioma_Nuclear_prep_Gradeall_repeat_4plex.raw
160707950		High	tQPAVATAATAAEk	Unambiguous	2	1	N-Term(iTRAQ4plex); K14(iTRAQ4plex)	HCD	1.0000	0.0000	1	1	8152	Unique	Used		0.555	8.912E3	5.388E3	218	5.96	0.00	0	25	51	3	539.97394	1617.90726	1.36	25.26	2486	2486	MS2	23/52	Glioma_Nuclear_prep_Gradeall_repeat_4plex.raw
160707950	High		tQPAVATAATAAEk	Unambiguous	2	1	N-Term(iTRAQ4plex); K14(iTRAQ4plex)	HCD	1.0000	0.0000	1	1	7939	Unique	Used		0.200	4.780E5	1.049E5	230	5.06	0.00	0	6	8	2	809.45905	1617.91081	3.55	25.94	2387	2387	MS2	22/26	Glioma_Nuclear_prep_Gradeall_4plex.raw
160707950		High	aEGGGATTSTQVMVIk	Unambiguous	2	1	N-Term(iTRAQ4plex); K16(iTRAQ4plex)	HCD	1.0000	0.0000	1	1	8944	Unique	Used		0.340	1.074E4	3.977E3	203	7.20	0.00	0	0	196	2	919.50018	1837.99309	-0.09	33.05	3320	3320	MS2	29/30	Glioma_Nuclear_prep_Gradeall_repeat_4plex.raw
160707950		High	aEGGGATTSTQVMVIk	Unambiguous	2	1	N-Term(iTRAQ4plex); K16(iTRAQ4plex)	HCD	1.0000	0.0000	1	1	9043	Unique	Used		0.427	2.536E3	1.181E3	211	6.58	0.00	0	13	300	2	919.50397	1838.00066	4.03	34.23	3428	3428	MS2	30/30	Glioma_Nuclear_prep_Gradeall_repeat_4plex.raw
160707950		High	aEGGGATTSTQVMVIk	Unambiguous	2	1	N-Term(iTRAQ4plex); K16(iTRAQ4plex)	HCD	1.0000	0.0000	1	1	8923	Unique	Used		0.553	3.153E3	1.899E3	204	6.33	0.00	0	37	117	3	613.33319	1837.98502	-4.48	32.85	3298	3298	MS2	23/60	Glioma_Nuclear_prep_Gradeall_repeat_4plex.raw
160707950	High		aEGGGATTSTQVMVIk	Unambiguous	2	1	N-Term(iTRAQ4plex); K16(iTRAQ4plex)	HCD	1.0000	0.0000	1	1	8509	Unique	Used		0.327	1.704E4	6.109E3	182	5.18	0.00	0	8	22	2	919.48737	1837.96745	-14.03	29.96	3004	3004	MS2	29/30	Glioma_Nuclear_prep_Gradeall_4plex.raw
160707950		High	hEPVQPSAHHSAEPAEAGk	Unambiguous	2	1	N-Term(iTRAQ4plex); K19(iTRAQ4plex)	HCD	1.0000	0.0000	1	1	6752	Unique	Used		0.209	4.121E3	9.380E2	89	4.17	0.00	0	14	131	3	756.38641	2267.14469	1.72	12.58	1006	1006	MS2	21/72	Glioma_Nuclear_prep_Gradeall_repeat_4plex.raw

160707950		High	hEPVQPSAHHSAEPAEAGk	Unambiguous	2	1	N-Term(iTRAQ4plex); K19(iTRAQ4plex)	HCD	1.0000	0.0000	1	1	6741	Unique	Used	0.285	4.840E3	1.503E3	150	5.02	0.00	0		30	4	567.53339	2267.11172	-12.82	12.50	995	995	MS2	25/108	Glioma_Nuclear_prep_Gradeall_repeat_4plex.raw
160707950	High		hEPVQPSAHHSAEPAEAGk	Unambiguous	2	1	N-Term(iTRAQ4plex); K19(iTRAQ4plex)	HCD	1.0000	0.0000	1	1	7101	Unique	Used	0.241	6.197E3	1.637E3	161	5.16	0.00	0	15	22	4	567.54199	2267.14614	2.36	19.65	1475	1475	MS2	26/108	Glioma_Nuclear_prep_Gradeall_4plex.raw
160707950	High		hEPVQPSAHHSAEPAEAGk	Unambiguous	2	1	N-Term(iTRAQ4plex); K19(iTRAQ4plex)	HCD	1.0000	0.0000	1	1	7092	Unique	Used	0.260	6.821E3	1.946E3	94	3.71	0.00	0	21	300	3	756.38770	2267.14853	3.42	19.53	1464	1464	MS2	22/72	Glioma_Nuclear_prep_Gradeall_4plex.raw
160707950		High	eVVkPLLVSTLGEk	Unambiguous	2	1	N-Term(iTRAQ4plex); K4(iTRAQ4plex); K14(iTRAQ4plex)	HCD	1.0000	0.0000	1	1	10872	Unique	Used	0.364	2.029E3	8.055E2	10	4.12	0.00	0	42	300	3	648.74677	1944.22574	7.79	55.58	5392	5392	MS2	24/52	Glioma_Nuclear_prep_Gradeall_repeat_4plex.raw
160707950	High		eVVkPLLVSTLGEk	Unambiguous	2	1	N-Term(iTRAQ4plex); K4(iTRAQ4plex); K14(iTRAQ4plex)	HCD	1.0000	0.0000	1	1	9940	Unique	Used	0.262	1.421E5	4.090E4	32	4.75	0.00	0	12	25	2	972.60138	1944.19548	-7.77	40.42	4564	4564	MS2	21/26	Glioma_Nuclear_prep_Gradeall_4plex.raw
160707950		High	eTVSIEVk	Unambiguous	2	1	N-Term(iTRAQ4plex); K8(iTRAQ4plex)	HCD	1.0000	0.0000	1	1	8585	Unique	Used	0.406	9.364E4	4.143E4	221	3.57	0.00	0	15	58	2	596.85474	1192.70220	-0.47	29.48	2943	2943	MS2	14/14	Glioma_Nuclear_prep_Gradeall_repeat_4plex.raw
160707950	High		eTVSIEVk	Unambiguous	2	1	N-Term(iTRAQ4plex); K8(iTRAQ4plex)	HCD	1.0000	0.0000	1	1	8217	Unique	Used	0.519	5.163E5	2.942E5	296	3.38	0.00	0	43	7	2	596.84741	1192.68755	-12.75	27.89	2688	2688	MS2	14/14	Glioma_Nuclear_prep_Gradeall_4plex.raw
160707950		High	vELIAYFEk	Unambiguous	2	1	N-Term(iTRAQ4plex); K9(iTRAQ4plex)	HCD	1.0000	0.0000	1	1	12124	Unique	Used	0.358	4.559E3	1.780E3	205	3.67	0.00	0	55	300	2	700.40961	1399.81194	3.13	74.67	6984	6984	MS2	15/16	Glioma_Nuclear_prep_Gradeall_repeat_4plex.raw
160707950	High		vELIAYFEk	Unambiguous	2	1	N-Term(iTRAQ4plex); K9(iTRAQ4plex)	HCD	1.0000	0.0000	1	1	11464	Unique	Used	0.318	3.273E5	1.141E5	217	3.98	0.00	0	23	15	2	700.40765	1399.80803	0.34	52.31	6229	6229	MS2	16/16	Glioma_Nuclear_prep_Gradeall_4plex.raw
160707950	High		vELIAYFEk	Unambiguous	2	1	N-Term(iTRAQ4plex); K9(iTRAQ4plex)	HCD	1.0000	0.0000	1	1	11387	Unique	Used	0.424	7.945E4	3.701E4	225	4.24	0.00	0	42	51	2	700.40808	1399.80889	0.95	51.63	6144	6144	MS2	15/16	Glioma_Nuclear_prep_Gradeall_4plex.raw
189217895	NP_689901.2	KHDRBS2	202559	KH domain-containing, RNA-binding, signal transduction-associated protein 2 [Homo sapiens]	10.03	5	2	3	0.282	2;1	13.6	13.20	349	38.9	6.29																			
189217895		High	fLVPDYNDEIR	Unambiguous	1	1	N-Term(iTRAQ4plex)	HCD	1.0000	0.0000	1	1	11164	Unique	Used	0.231	1.118E4	2.814E3	280	3.24	0.00	0	38	300	2	762.89685	1524.78642	3.21	59.32	5720	5720	MS2	13/20	Glioma_Nuclear_prep_Gradeall_repeat_4plex.raw
189217895	High		fLVPDYNDEIR	Unambiguous	1	1	N-Term(iTRAQ4plex)	HCD	1.0000	0.0000	1	1	10388	Unique	Used	0.303	1.484E5	4.926E4	194	3.02	0.00	0	49	33	2	762.88330	1524.75932	-14.56	43.75	5052	5052	MS2	12/20	Glioma_Nuclear_prep_Gradeall_4plex.raw
189217895	High		ILAEEIEk	Unambiguous	1	1	N-Term(iTRAQ4plex); K8(iTRAQ4plex)	HCD	1.0000	0.0000	1	1	9223	Unique	Used	0.282	3.521E5	1.091E5	292	3.08	0.00	0	35	12	2	616.86536	1232.72344	-8.62	35.17	3783	3783	MS2	14/14	Glioma_Nuclear_prep_Gradeall_4plex.raw
193083114	NP_001122386.1	PCBP2	5094	poly(rC)-binding protein 2 isoform g [Homo sapiens]	14.47	9	2	4	0.613	1;3	27.7	21.73	318	33.5	8.24																			
193083114		High	IVVPASQcGSLIGk	Unambiguous	9	1	N-Term(iTRAQ4plex); C8(Methylthio); K14(iTRAQ4plex)	HCD	1.0000	0.0000	1	1	11932	Unique	Used	0.358	2.581E3	1.007E3	189	3.91	0.00	0	40	300	2	853.48627	1705.96526	4.04	70.76	6680	6680	MS2	17/26	Glioma_Nuclear_prep_Gradeall_4plex.raw
193083114		High	iITLAGPTNAIFk	Unambiguous	7	1	N-Term(iTRAQ4plex); K13(iTRAQ4plex)	HCD	1.0000	0.0000	1	1	11968	Unique	Used	0.563	2.713E3	1.666E3	127	3.73	0.00	0	55	300	2	824.00958	1647.01189	2.11	71.46	6735	6735	MS2	17/24	Glioma_Nuclear_prep_Gradeall_4plex.raw
193083114		High	iITLAGPTNAIFk	Unambiguous	7	1	N-Term(iTRAQ4plex); K13(iTRAQ4plex)	HCD	1.0000	0.0000	1	1	11964	Unique	Used	0.663	5.201E2	3.757E2	118	3.50	0.00	0	43	300	3	549.67340	1647.00565	-1.67	71.39	6730	6730	MS2	21/48	Glioma_Nuclear_prep_Gradeall_repeat_4plex.raw
193083114	High		iITLAGPTNAIFk	Unambiguous	7	1	N-Term(iTRAQ4plex); K13(iTRAQ4plex)	HCD	1.0000	0.0000	1	1	11154	Unique	Used	0.722	4.218E4	3.341E4	128	4.67	0.00	0	56	47	2	824.00616	1647.00505	-2.04	49.66	5889	5889	MS2	16/24	Glioma_Nuclear_prep_Gradeall_4plex.raw
193794826	NP_001123291.1	CYB5R3	1727	NADH-cytochrome b5 reductase 3 isoform 2 [Homo sapiens]	4.68	3	1	3	1.561	1;2	2.5	9.52	278	31.6	7.58																			
193794826		High	sTPAITLESPDIk	Unambiguous	3	1	N-Term(iTRAQ4plex); K13(iTRAQ4plex)	HCD	1.0000	0.0000	1	1	10141	Unique	Used	1.508	9.521E2	1.565E3	200	4.44	0.00	0	29	168	3	553.98572	1659.94260	1.10	46.78	4606	4606	MS2	17/48	Glioma_Nuclear_prep_Gradeall_repeat_4plex.raw
193794826		High	sTPAITLESPDIk	Unambiguous	3	1	N-Term(iTRAQ4plex); K13(iTRAQ4plex)	HCD	1.0000	0.0000	1	1	10137	Unique	Used	1.561	4.187E3	7.123E3	215	5.08	0.00	0	40	300	2	830.47577	1659.94426	2.10	46.72	4601	4601	MS2	20/24	Glioma_Nuclear_prep_Gradeall_repeat_4plex.raw
193794826	High		sTPAITLESPDIk	Unambiguous	3	1	N-Term(iTRAQ4plex); K13(iTRAQ4plex)	HCD	1.0000	0.0000	1	1	9500	Unique	Used	1.583	9.147E4	1.589E5	227	4.44	0.00	0	21	15	2	830.46063	1659.91399	-16.14	37.25	4087	4087	MS2	21/24	Glioma_Nuclear_prep_Gradeall_4plex.raw
194688133	NP_001124160.1	HMGB2	3148	high mobility group protein B2 [Homo sapiens]	26.32	1	5	8	1.959	3;5	30.3	34.59	209	24.0	7.81																			
194688133		High	kHPDSSVNFAEFSk	Unambiguous	1	1	N-Term(iTRAQ4plex); K1(iTRAQ4plex); K14(iTRAQ4plex)	HCD	1.0000	0.0000	1	1	8993	Unique	Used	1.773	8.297E2	1.604E3	145	6.39	0.00	1	30	259	3	675.69824	2025.08017	1.76	33.63	3374	3374	MS2	25/52	Glioma_Nuclear_prep_Gradeall_repeat_4plex.raw
194688133		High	sEHPGLSIGDTAk	Unambiguous	1	1	N-Term(iTRAQ4plex); K13(iTRAQ4plex)	HCD	1.0000	0.0000	1	1	7978	Unique	Used	1.690	1.162E4	2.141E4	169	5.30	0.00	0	15	25	3	533.95837	1599.86057	1.54	23.61	2303	2303	MS2	28/48	Glioma_Nuclear_prep_Gradeall_repeat_4plex.raw
194688133		High	hPDSSVNFAEFSk	Unambiguous	1	1	N-Term(iTRAQ4plex); K13(iTRAQ4plex)	HCD	1.0000	0.0000	1	1	9557	Unique	Used	1.906	7.852E2	1.631E3	140	5.39	0.00	0	13	215	3	584.95868	1752.86148	-10.32	39.93	3977	3977	MS2	24/48	Glioma_Nuclear_prep_Gradeall_repeat_4plex.raw
194688133		High	sEHPGLSIGDTAk	Unambiguous	1	1	N-Term(iTRAQ4plex); K13(iTRAQ4plex)	HCD	1.0000	0.0000	1	1	7985	Unique	Used	3.539	3.496E3	1.349E4	175	4.06	0.00	0	5	122	2	800.43475	1599.86223	2.58	23.66	2310	2310	MS2	20/24	Glioma_Nuclear_prep_Gradeall_repeat_4plex.raw
194688133	High		sEHPGLSIGDTAk	Unambiguous	1	1	N-Term(iTRAQ4plex); K13(iTRAQ4plex)	HCD	1.0000	0.0000	1	1	7771	Unique	Used	2.013	9.432E4	2.083E5	175	4.43	0.00	0	11	1	3	533.95703	1599.85654	-0.98	24.74	2205	2205	MS2	28/48	Glioma_Nuclear_prep_Gradeall_4plex.raw
194688133	High		sEHPGLSIGDTAk	Unambiguous	1	1	N-Term(iTRAQ4plex); K13(iTRAQ4plex)	HCD	1.0000	0.0000	1	1	7762	Unique	Used	2.915	6.605E4	2.113E5	182	4.13	0.00	0	8	6	2	800.43286	1599.85845	0.21	24.68	2195	2195	MS2	22/24	Glioma_Nuclear_prep_Gradeall_4plex.raw
194688133		High	dkQPYEQk	Unambiguous	1	1	N-Term(iTRAQ4plex); K2(iTRAQ4plex); K8(iTRAQ4plex)	HCD	1.0000	0.0000	1	1	6805	Unique	Used	2.789	2.649E3	8.053E3	254	3.13	0.00	1	9	121	2	734.41266	1467.81804	0.87	13.06	1062	1062	MS2	13/14	Glioma_Nuclear_prep_Gradeall_repeat_4plex.raw
194688133	High		skFEDMAk	Unambiguous	1	1	N-Term(iTRAQ4plex); K2(iTRAQ4plex); K8(iTRAQ4plex)	HCD	1.0000	0.0000	1	1	7730	Unique	Used	1.716	8.216E4	1.547E5	245	3.03	0.00	1	14	13	2	694.38489	1387.76250	0.65	24.45	2160	2160	MS2	14/14	Glioma_Nuclear_prep_Gradeall_4plex.raw
197927454	NP_001128181.1	DEK	7913	protein DEK isoform 2 [Homo sapiens]	20.82	2	4	11	1.724	4;7	20.5	48.25	341	38.7	8.15																			
197927454		High	vYENYPTYDLTER	Unambiguous	2	1	N-Term(iTRAQ4plex)	HCD	1.0000	0.0000	1	1	10274	Unique	Used	2.187	2.736E3	6.520E3	139	4.64	0.00	0	8	300	2	903.94019	1806.87309	3.55	48.32	4746	4746	MS2	19/24	Glioma_Nuclear_prep_Gradeall_repeat_4plex.raw
197927454	High		vYENYPTYDLTER	Unambiguous	2	1	N-Term(iTRAQ4plex)	HCD	1.0000	0.0000	1	1	9689	Unique	Used	2.078	5.379E4	1.227E5	84	4.58	0.00	0	10	15	2	903.92401	1806.84075	-14.36	38.61	4292	4292	MS2	19/24	Glioma_Nuclear_prep_Gradeall_4plex.raw
197927454		High	slcEVLDLER	Unambiguous	2	1	N-Term(iTRAQ4plex); C3(Methylthio)	HCD	1.0000	0.0000	1	1	12416	Unique	Used	1.526	1.219E3	2.028E3	161	3.37	0.00	0	14	300	2	683.84576	1366.68425	1.10	84.60	7704	7704	MS2	17/18	Glioma_Nuclear_prep_Gradeall_repeat_4plex.raw
197927454	High		slcEVLDLER	Unambiguous	2	1	N-Term(iTRAQ4plex); C3(Methylthio)	HCD	1.0000	0.0000	1	1	12161	Unique	Used	1.409	1.197E5	1.852E5	160	3.77	0.00	0	18	17	2	683.84369	1366.68010	-1.93	58.59	6989	6989	MS2	16/18	Glioma_Nuclear_prep_Gradeall_4plex.raw
197927454	High		slcEVLDLER	Unambiguous	2	1	N-Term(iTRAQ4plex); C3(Methylthio)	HCD	1.0000	0.0000	1	1	12226	Unique	Used	1.490	1.348E5	2.205E5	162	3.92	0.00	0	11	18	2	683.84637	1366.68547	2.00	59.26	7061	7061	MS2	17/18	Glioma_Nuclear_prep_Gradeall_4plex.raw
197927454		High	nVGQFSGFPFEk	Unambiguous	2	1	N-Term(iTRAQ4plex); K12(iTRAQ4plex)	HCD	1.0000	0.0000	1	1	11435	Unique	Used	1.334	5.966E3	8.674E3	207	4.62	0.00	0	9	300	2	822.93604	1644.86479	1.41	63.28	6054	6054	MS2	21/22	Glioma_Nuclear_prep_Gradeall_repeat_4plex.raw
197927454		High	nVGQFSGFPFEk	Unambiguous	2	1	N-Term(iTRAQ4plex); K12(iTRAQ4plex)	HCD	1.0000	0.0000	1	1	11433	Unique	Used	1.351	9.352E2	1.377E3	156	4.24	0.00	0	10	190	3	548.95923	1644.86313	0.40	63.26	6052	6052	MS2	22/44	Glioma_Nuclear_prep_Gradeall_repeat_4plex.raw
197927454		High	ILASANLEEVTMk	Unambiguous	2	1	N-Term(iTRAQ4plex); K13(iTRAQ4plex)	HCD	1.0000	0.0000	1	1	11343	Unique	Used	2.045	1.456E3	3.246E3	221	5.61	0.00	0	25	300	2	853.98492	1706.96257	1.42	62.03	5946	5946	MS2	22/24	Glioma_Nuclear_prep_Gradeall_repeat_4plex.raw
197927454	High		ILASANLEEVTMk	Unambiguous	2	1	N-Term(iTRAQ4plex); K13(iTRAQ4plex)	HCD	1.0000	0.0000	1	1	10485	Unique	Used	2.327	4.376E4	1.117E5	256	5.37	0.00	0	41	28	2	853.97198	1706.93669	-13.74	44.52	5160	5160	MS2	23/24	Glioma_Nuclear_prep_Gradeall_4plex.raw
197927454		High	ILASANLEEVTmk	Unambiguous	2	1	N-Term(iTRAQ4plex); M12(Oxidation); K13(iTRAQ4plex)	HCD	1.0000	0.0000	1	1	10865	Unique	Used	1.724	2.969E3	5.578E3	230	5.33	0.00	0	34	300	2	861.98334	1722.95940	2.52	55.48	5384	5384	MS2	22/24	Glioma_Nuclear_prep_Gradeall_repeat_4plex.raw
197927454		High	ILASANLEEVTmk	Unambiguous	2	1	N-Term(iTRAQ4plex); M12(Oxidation); K13(iTRAQ4plex)	HCD	1.0000	0.0000	1	1	10861	Unique	Used	1.755	6.661E2	1.274E3	176	4.04	0.00	0	30	300	3	574.99023	1722.95615	0.63	55.44	5380	5380	MS2	20/48	Glioma_Nuclear_prep_Gradeall_repeat_4plex.raw
212549553	NP_060090.2	ILF3	3609	interleukin enhancer-binding factor 3 isoform d [Homo sapiens]	33.18	5	21	53	0.493	23;30	23.3	170.51	898	95.7	8.81																			
212549553	High		hSSVYPTQEELEAVQNMVSHTER	Unambiguous	5	1	N-Term(iTRAQ4plex)	HCD	1.0000	0.0000	1	1	10965	Unique	Used	0.465	4.501E4	2.296E4	52	9.60	0.00	0	6	9	3	939.10748	2815.30790	-12.76	48.19	5683	5683	MS2	34/88	Glioma_Nuclear_prep_Gradeall_4plex.raw
212549553		High	fNYSGSGGR	Unambiguous	2	1	N-Term(iTRAQ4plex)	HCD	1.0000	0.0000	1	1	7572	Unique	Used	0.355	1.397E5	5.404E4	71	3.39	0.00	0	23	56	2	544.76404	1088.52080	-3.09	19.71	1871	1871	MS2	16/16	Glioma_Nuclear_prep_Gradeall_repeat_4plex.raw
212549553		High	eDITQSAQHALR	Unambiguous	5	1	N-Term(iTRAQ4plex)	HCD	1.0000	0.0000	1	1	8262	Unique	Used	0.452	1.549E4	7.631E3	205	4.40	0.00	0	2	159	2	756.90045	1512.79363	3.23	26.31	2602	2602	MS2	22/22	Glioma_Nuclear_prep_Gradeall_repeat_4plex.raw
212549553		High	eATDAIGHLDR	Unambiguous	5	1	N-Term(iTRAQ4plex)	HCD	1.0000	0.0000	1	1	8168	Unique	Used	0.455	5.406E4	2.680E4	205	4.34	0.00	0	9	59	2	671.34570	1341.68413	-2.86	25.41	2503	2503	MS2	19/20	Glioma_Nuclear_prep_Gradeall_repeat_4plex.raw
212549553		High	vLAGETLSVNDPPDVLDR	Unambiguous	5	1	N-Term(iTRAQ4plex)	HCD	1.0000	0.0000	1	1	11358	Unique	Used	0.476	1.528E3	7.919E2	139	5.89	0.00	0	4	300	2	1027.54944	2054.09160	1.41	62.23	5963	5963	MS2	28/34	Glioma_Nuclear_prep_Gradeall_repeat_4plex.raw
212549553		High	aEPPQAMNALMR	Unambiguous	5	1	N-Term(iTRAQ4plex)	HCD	1.0000	0.0000	1	1	9830	Unique	Used	0.632	8.078E3	5.560E3	216	3.76	0.00	0	20	300	2	736.88013	1472.75298	3.99	42.98	4266	4266	MS2	16/22	Glioma_Nuclear_prep_Gradeall_repeat_4plex.raw
212549553		High	eATDAIGHLDR	Unambiguous	5	1	N-Term(iTRAQ4plex)	HCD	1.0000	0.0000	1	1	8153	Unique	Used	0.641	1.657E4	1.159E4	183	4.94	0.00	0	19	49	3	447.90054	1341.68708	-0.66	25.26	2487	2487	MS2	26/40	Glioma_Nuclear_prep_Gradeall_repeat_4plex.raw
212549553		High	vLQDMGLPTGAEGR	Unambiguous	5	1	N-Term(iTRAQ4plex)	HCD	1.0000	0.0000	1	1	10609	Unique	Used	0.657	5.850E3	4.192E3	226	4.45	0.00	0	17	300	2	794.41907	1587.83086	1.68	52.34	5106	5106	MS2	18/26	Glioma_Nuclear_prep_Gradeall_repeat_4plex.raw
212549553		High	aEPPQAMNALMR	Unambiguous	5	1	N-Term(iTRAQ4plex)	HCD	1.0000	0.0000	1	1	9711	Unique	Used	0.670	7.182E3	5.246E3	215	3.65	0.00	0	31	300	2	736.87958	1472.75188	3.24	41.65	4141	4141	MS2	16/22	Glioma_Nuclear_prep_Gradeall_repeat_4plex.raw
212549553		High	sIGTANRPMGAGEALR	Unambiguous	5	1	N-Term(iTRAQ4plex)	HCD	1.0000	0.0000	1	1	8246	Unique	Used	0.676	3.537E3	2.605E3	169	2.86	0.00	0	20	210	2	872.96930	1744.93132	3.87	26.17	2585	2585	MS2	14/30	Glioma_Nuclear_prep_Gradeall_repeat_4plex.raw
212549553		High	eDITQSAQHALR	Unambiguous	5	1	N-Term(iTRAQ4plex)	HCD	1.0000	0.0000	1	1	8253	Unique	Used	0.695	1.311E4	9.920E3	175	5.88	0.00	0	18	44	3	504.93570	1512.79255	2.52	26.24	2593	2593	MS2	28/44	Glioma_Nuclear_prep_Gradeall_repeat_4plex.raw
212549553	High		vLAGETLSVNDPPDVLDR	Unambiguous	5	1	N-Term(iTRAQ4plex)	HCD	1.0000	0.0000	1	1	10644	Unique	Used	0.397	1.965E5	8.572E4	137	6.68	0.00	0	1	9	2	1027.53357	2054.05986	-14.05	45.73	5334	5334	MS2	28/34	Glioma_Nuclear_prep_Gradeall_4plex.raw
212549553	High		eDITQSAQHALR	Unambiguous	5	1	N-Term(iTRAQ4plex)	HCD	1.0000	0.0000	1	1	8011	Unique	Used	0.450	2.610E5	1.290E5	191	3.78	0.00	0	6	6	2	756.88623	1512.76518	-15.57	26.44	2465	2465	MS2	21/22	Glioma_Nuclear_prep_Gradeall_4plex.raw
212549553	High		eATDAIGHLDR	Unambiguous	5	1	N-Term(iTRAQ4plex)	HCD	1.0000	0.0000	1	1	7918	Unique	Used	0.480	3.376E4	1.777E4	188	3.93	0.00	0	42	9	3	447.90045	1341.68680	-0.87	25.78	2364	2364	MS2	22/40	Glioma_Nuclear_prep_Gradeall_4plex.raw
212549553	High		eATDAIGHLDR	Unambiguous	5	1	N-Term(iTRAQ4plex)	HCD	1.0000	0.0000	1	1	7930	Unique	Used	0.483	4.207E5	2.230E5	211	3.93	0.00	0	9	6	2	671.34766	1341.68804	0.05	25.87	2377	2377	MS2	19/20	Glioma_Nuclear_prep_Gradeall_4plex.raw
212549553	High		eDITQSAQHALR	Unambiguous	5	1	N-Term(iTRAQ4plex)	HCD	1.0000	0.0000	1	1	8001	Unique	Used	0.484	6.333E4	3.363E4	153	4.74	0.00	0	26	4	3	504.92676	1512.76572	-15.21	26.37	2454	2454	MS2	25/44	Glioma_Nuclear_prep_Gradeall_4plex.raw
212549553	High		vLQDMGLPTGAEGR	Unambiguous	5	1	N-Term(iTRAQ4plex)	HCD	1.0000	0.0000	1	1	9971	Unique	Used	0.610	1.956E5	1.310E5	125	4.15	0.00	0	7	11	2	794.40601	1587.80474	-14.77	40.64	4598	4598	MS2	19/26	Glioma_Nuclear_prep_Gradeall_4plex.raw
212549553		High	cLAALASLR	Unambiguous	5	1	N-Term(iTRAQ4plex); C1(Methylthio)	HCD	1.0000	0.0000	1	1	12184	Unique	Used	0.364	6.202E3	2.462E3	328	2.85	0.00	0	19	300	2	554.31134	1107.61540	1.69	75.78	7073	7073	MS2	15/16	Glioma_Nuclear_prep_Gradeall_repeat_4plex.raw
212549553		High	cLAALASLR	Unambiguous	5	1	N-Term(iTRAQ4plex); C1(Methylthio)	HCD	1.0000	0.0000	1	1	12127	Unique	Used	0.397	1.168E4	5.048E3	234	3.28	0.00	0	16	300	2	554.31134	1107.61540	1.69	74.77	6992	6992	MS2	15/16	Glioma_Nuclear_prep_Gradeall_repeat_4plex.raw
212549553	High		cLAALASLR	Unambiguous	5	1	N-Term(iTRAQ4plex); C1(Methylthio)	HCD	1.0000	0.0000	1	1	11426	Unique	Used	0.351	7.701E5	2.970E5	255	3.12	0.00	0	4	5	2	554.30896	1107.61064	-2.61	51.98	6187	6187	MS2	15/16	Glioma_Nuclear_prep_Gradeall_4plex.raw
212549553	High		cLAALASLR	Unambiguous	5	1	N-Term(iTRAQ4plex); C1(Methylthio)	HCD	1.0000	0.0000	1	1	11343	Unique	Used	0.493	4.179E4	2.261E4	305	3.28	0.00	0	40	84	2	554.31012	1107.61296	-0.52	51.25	6096	6096	MS2	15/16	Glioma_Nuclear_prep_Gradeall_4plex.raw
212549553		High	aYAALAALEk	Unambiguous	5	1	N-Term(iTRAQ4plex); K10(iTRAQ4plex)	HCD	1.0000	0.0000	1	1	10894	Unique	Used	0.430	1.747E4	8.191E3	219	4.46	0.00	0	27	262	2	654.89368	1308.78008	2.66	55.86	5417	5417	MS2	18/18	Glioma_Nuclear_prep_Gradeall_repeat_4plex.raw
212549553		High	vLGMDPLPSk	Unambiguous	5	1	N-Term(iTRAQ4plex); K10(iTRAQ4plex)	HCD	1.0000	0.0000	1	1	10372	Unique	Used	0.498	1.554E4	8.436E3	221	3.93	0.00	0	28	300	2	672.89520	1344.78313	2.34	49.51	4852	4852	MS2	16/18	Glioma_Nuclear_prep_Gradeall_repeat_4plex.raw
212549553		High	aYAALAALEk	Unambiguous	5	1	N-Term(iTRAQ4plex); K10(iTRAQ4plex)	HCD	1.0000	0.0000	1	1	10895	Unique	Used	0.629	9.878E2	6.768E2	232	3.45	0.00	0	11	300	3	436.93115	1308.77890	1.76	55.88	5418	5418	MS2	18/36	Glioma_Nuclear_prep_Gradeall_repeat_4plex.raw
212549553		High	aYAALAALEk	Unambiguous	5	1	N-Term(iTRAQ4plex); K10(iTRAQ4plex)	HCD	1.0000	0.0000	1	1	10812	Unique	Used	0.646	7.179E2	5.056E2	233	3.44	0.00	0	46	300	3	436.93073	1308.77762	0.78	54.83	5326	5326	MS2	18/36	Glioma_Nuclear_prep_Gradeall_repeat_4plex.raw
212549553		High	aYAALAALEk	Unambiguous	5	1	N-Term(iTRAQ4plex); K10(iTRAQ4plex)	HCD	1.0000	0.0000	1	1	10808	Unique	Used	0.701	7.640E3	5.834E3	255	4.15	0.00	0	52	300	2	654.89301	1308.77873	1.63	54.78	5322	5322	MS2	17/18	Glioma_Nuclear_prep_Gradeall_repeat_4plex.raw
212549553	High		vLGMDPLPSk	Unambiguous	5	1	N-Term(iTRAQ4plex); K10(iTRAQ4plex)	HCD	1.0000	0.0000	1	1	9699	Unique	Used	0.512	4.141E5	2.326E5	236	3.67	0.00	0	42	7	2	672.88361	1344.75994	-14.91	38.68	4303	4303	MS2	16/18	Glioma_Nuclear_prep_Gradeall_4plex.raw
212549553	High		aYAALAALEk	Unambiguous	5	1	N-Term(iTRAQ4plex); K10(iTRAQ4plex)	HCD	1.0000	0.0000	1	1	9977	Unique	Used	0.543	2.345E5	1.398E5	270	4.55	0.00	0	76	20	2	654.88074	1308.75420	-17.11	40.68	4604	4604	MS2	18/18	Glioma_Nuclear_prep_Gradeall_4plex.raw
212549553	High		aYAALAALEk	Unambiguous	5	1	N-Term(iTRAQ4plex); K10(iTRAQ4plex)	HCD	1.0000	0.0000	1	1	10065	Unique	Used	0.656	2.393E5	1.723E5	311	4.09	0.00	0	33	9	2	654.88257	1308.75786	-14.32	41.33	4700	4700	MS2	17/18	Glioma_Nuclear_prep_Gradeall_4plex.raw
212549553	High		rPMEEDGEEk	Unambiguous	5	1	N-Term(iTRAQ4plex); K10(iTRAQ4plex)	HCD	1.0000	0.0000	1	1	7102	Unique	Used	0.715	3.440E3	2.699E3	80	3.72	0.00	0	65	197	3	503.24728	1507.72730	-1.89	19.66	1476	1476	MS2	16/36	Glioma_Nuclear_prep_Gradeall_4plex.raw
212549553		High	aVSDWIDEQEk	Unambiguous	5	1	N-Term(iTRAQ4plex); K11(iTRAQ4plex)	HCD	1.0000	0.0000	1	1	10174	Unique	Used	0.535	9.491E3	5.533E3	183	4.38	0.00	0	12	259	2	804.41382	1607.82036	2.97	47.17	4641	4641	MS2	20/20	Glioma_Nuclear_prep_Gradeall_repeat_4plex.raw
212549553	High		aVSDWIDEQEk	Unambiguous	5	1	N-Term(iTRAQ4plex); K11(iTRAQ4plex)	HCD	1.0000	0.0000	1	1	9656	Unique	Used	0.631	9.726E4	6.734E4	150	3.94	0.00	0	36	16	2	804.39960	1607.79192	-14.72	38.37	4256	4256	MS2	20/20	Glioma_Nuclear_prep_Gradeall_4plex.raw
212549553		High	IFPDTPLALDANk	Unambiguous	5	1	N-Term(iTRAQ4plex); K13(iTRAQ4plex)	HCD	1.0000	0.0000	1	1	11770	Unique	Used	0.385	9.853E3	4.136E3	199	4.95	0.00	0	10	300	2	851.98755	1702.96782	3.50	68.06	6459	6459	MS2	20/24	Glioma_Nuclear_prep_Gradeall_repeat_4plex.raw
212549553		High	IFPDTPLALDANk	Unambiguous	5	1	N-Term(iTRAQ4plex); K13(iTRAQ4plex)	HCD	1.0000	0.0000	1	1	11703	Unique	Used	0.489	8.630E3	4.597E3	201	4.70	0.00	0	31	300	2	851.98718	1702.96709	3.07	67.03	6373	6373	MS2	20/24	Glioma_Nuclear_prep_Gradeall_repeat_4plex.raw
212549553	High		IFPDTPLALDANk	Unambiguous	5	1	N-Term(iTRAQ4plex); K13(iTRAQ4plex)	HCD	1.0000	0.0000	1	1	10894	Unique	Used	0.457	3.705E5	1.856E5	212	4.92	0.00	0	26	14	2	851.97418	1702.94109	-12.20	47.64	5606	5606	MS2	20/24	Glioma_Nuclear_prep_Gradeall_4plex.raw
212549553		High	IFPDTPLALDANkk	Unambiguous	5	1	N-Term(iTRAQ4plex); K13(iTRAQ4plex); K14(iTRAQ4plex)	HCD	1.0000	0.0000	1	1	11019	Unique	Used	0.429	2.182E3	1.019E3	95	4.68	0.00	1	44	300	3	659.05853	1975.16104	1.09	57.40	5553	5553	MS2	26/52	Glioma_Nuclear_prep_Gradeall_repeat_4plex.raw
212549553	High		IFPDTPLALDANkk	Unambiguous	5	1	N-Term(iTRAQ4plex); K13(iTRAQ4plex); K14(iTRAQ4plex)	HCD	1.0000	0.0000	1	1	10092	Unique	Used	0.343	5.148E4	1.936E4	124	3.56	0.00	1	54	14	3	659.04742	1975.12772	-15.78	41.51	4729	4729	MS2	28/52	Glioma_Nuclear_prep_Gradeall_4plex.raw
212549553		High	ekPTTALLDk	Unambiguous	5	1	N-Term(iTRAQ4plex); K2(iTRAQ4plex); K10(iTRAQ4plex)	HCD	1.0000	0.0000	1	1	8501	Unique	Used	0.730	8.018E3	6.376E3	132	3.09	0.00	0	27	271	2	774.47339	1547.93950	1.67	28.62	2854	2854	MS2	16/18	Glioma_Nuclear_prep_Gradeall_repeat_4plex.raw
212549553	High		ekPTTALLDk	Unambiguous	5	1	N-Term(iTRAQ4plex); K2(iTRAQ4plex); K10(iTRAQ4plex)	HCD	1.0000	0.0000	1	1	8124	Unique	Used	0.719	2.235E5	1.763E5	191	3.35	0.00	0	29	8	2	774.45801	1547.90874	-18.20	27.24	2587	2587	MS2	17/18	Glioma_Nuclear_prep_Gradeall_4plex.raw
212549553	High		nENPVDYTVQIPPSTTYAITPMk	Unambiguous	5	1	N-Term(iTRAQ4plex); K23(iTRAQ4plex)	HCD	1.0000	0.0000	1	1	11083	Unique	Used	0.224	7.472E3	1.837E3	105	5.68	0.00	0	0	45	2	1434.23572	2867.46416	-3.43	49.10	5811	5811	MS2	25/44	Glioma_Nuclear_prep_Gradeall_4plex.raw
212549553	High		INQLkPGLQYk	Unambiguous	5	1	N-Term(iTRAQ4plex); K5(iTRAQ4plex); K11(iTRAQ4plex)	HCD	1.0000	0.0000	1	1	8705	Unique	Used	0.461	3.212E5	1.627E5	118	4.35	0.00	0	26	9	2	867.52228	1734.03728	-15.31	31.41	3219	3219	MS2	17/20	Glioma_Nuclear_prep_Gradeall_4plex.raw
212549553		High	fQGAGSNk	Unambiguous	5	1	N-Term(iTRAQ4plex); K8(iTRAQ4plex)	HCD	1.0000	0.0000	1	1	6896	Unique	Used	0.433	9.085E4	4.289E4	207	2.85	0.00	0	41	69	2	548.80084	1096.59441	-4.15	13.84	1158	1158	MS2	14/14	Glioma_Nuclear_prep_Gradeall_repeat_4plex.raw
212549553		High	qQGPILTk	Unambiguous	2	1	N-Term(iTRAQ4plex); K8(iTRAQ4plex)	HCD	1.0000	0.0000	1	1	7755	Unique	Used	0.539	6.015E4	3.535E4	170	2.84	0.00	0	31	67	2	586.86450	1172.72173	-2.07	21.47	2068	2068	MS2	13/14	Glioma_Nuclear_prep_Gradeall_repeat_4plex.raw
212549553		High	IAAFGQLHk	Unambiguous	5	1	N-Term(iTRAQ4plex); K9(iTRAQ4plex)	HCD	1.0000	0.0000	1	1	9450	Unique	Used	0.493	3.786E4	2.036E4	185	3.40	0.00	0	38	140	2	636.88879	1272.77031	2.84	38.67	3858	3858	MS2	16/16	Glioma_Nuclear_prep_Gradeall_repeat_4plex.raw
212549553		High	IAAFGQLHk	Unambiguous	5	1	N-Term(iTRAQ4plex); K9(iTRAQ4plex)	HCD	1.0000	0.0000	1	1	9407	Unique	Used	0.502	1.191E4	6.512E3	161	4.53	0.00	0	20	101	3	424.92758	1272.76819	1.17	38.21	3812	3812	MS2	22/32	Glioma_Nuclear_prep_Gradeall_repeat_4plex.raw
212549553		High	IAAFGQLHk	Unambiguous	5	1	N-Term(iTRAQ4plex); K9(iTRAQ4plex)	HCD	1.0000	0.0000	1	1	9497	Unique	Used	0.519	1.099E4	6.221E3	172	4.25	0.00	0	25	61	3	424.92358	1272.75620	-8.25	39.22	3909	3909	MS2	21/32	Glioma_Nuclear_prep_Gradeall_repeat_4plex.raw
212549553	High		IAAFGQLHk	Unambiguous	5	1	N-Term(iTRAQ4plex); K9(iTRAQ4plex)	HCD	1.0000	0.0000	1	1	8715	Unique	Used	0.348	5.752E5	2.195E5	195	4.13	0.00	0	10	1	3	424.92010	1272.74576	-16.45	31.48	3230	3230	MS2	23/32	Glioma_Nuclear_prep_Gradeall_4plex.raw
212549553	High		IAAFGQLHk	Unambiguous	5	1	N-Term(iTRAQ4plex); K9(iTRAQ4plex)	HCD	1.0000	0.0000	1	1	8726	Unique	Used	0.443	1.181E6	5.735E5	200	3.47	0.00	0	42	3	2	636.87769	1272.74809	-14.62	31.56	3242	3242	MS2	16/16	Glioma_Nuclear_prep_Gradeall_4plex.raw
212549553		High	hSSVYPTQEELEAVQNmVSHTER	Unambiguous	5	1	N-Term(iTRAQ4plex); M17(Oxidation)	HCD	1.0000	0.0000	1	1	10866	Unique	Used	0.416	4.835E2	2.190E2	80	5.23	0.00	0	11	300	3	944.45221	2831.34208	1.18	55.49	5385	5385	MS2	22/88	Glioma_Nuclear_prep_Gradeall_repeat_4plex.raw
212549553		High	vLGmDPLPSk	Unambiguous	5	1	N-Term(iTRAQ4plex); M4(Oxidation); K10(iTRAQ4plex)	HCD	1.0000	0.0000	1	1	9342	Unique	Used	0.531	3.350E4	1.940E4	265	3.72	0.00	0	20	101	2	680.89307	1360.77886	2.91	37.54	3744	3744	MS2	15/18	Glioma_Nuclear_prep_Gradeall_repeat_4plex.raw
212549553	High		vLGmDPLPSk	Unambiguous	5	1	N-Term(iTRAQ4plex); M4(Oxidation); K10(iTRAQ4plex)	HCD	1.0000	0.0000	1	1	8876	Unique	Used	0.529	2.482E5	1.441E5	262	3.85	0.00	0	36	18	2	680.87933	1360.75139	-17.28	32.63	3404	3404	MS2	15/18	Glioma_Nuclear_prep_Gradeall_4plex.raw
212549553		High	vLQDmGLPTGAEGR	Unambiguous	5	1	N-Term(iTRAQ4plex); M5(Oxidation)	HCD	1.0000	0.0000	1	1	9108	Unique	Used	0.671	6.699E3	4.897E3	261	4.07	0.00	0	14	300	2	802.41620	1603.82512	1.25	34.94	3497	3497	MS2	19/26	Glioma_Nuclear_prep_Gradeall_repeat_4plex.raw
212549553	High		vLQDmGLPTGAEGR	Unambiguous	5	1	N-Term(iTRAQ4plex); M5(Oxidation)	HCD	1.0000	0.0000	1	1	8795	Unique	Used	0.729	4.299E4	3.437E4	170	3.59	0.00	0	34	43	2	802.40308	1603.79888	-15.11	32.04	3316	3316	MS2	19/26	Glioma_Nuclear_prep_Gradeall_4plex.raw
217330646	NP_006704.3	SUB1	10923	activated RNA polymerase II transcriptional coactivator p15 [Homo sapiens]	18.90	1	2	4	2.200	2;2	8.4	8.73	127	14.4	9.60																			
217330646		High	eQISDIDDAVR	Unambiguous	1	1	N-Term(iTRAQ4plex)	HCD	1.0000	0.0000	1	1	9152	Unique	Used	2.172	1.048E4	2.481E4	196	4.45	0.00	0	11	237	2	702.85931	1404.71135	1.84	35.45	3544	3544	MS2	19/20	Glioma_Nuclear_prep_Gradeall_repeat_4plex.raw
217330646	High		eQISDIDDAVR	Unambiguous	1	1	N-Term(iTRAQ4plex)	HCD	1.0000	0.0000	1	1	8813	Unique	Used	2.534	8.036E4	2.235E5	135	3.55	0.00	0	4	16	2	702.84607	1404.68486	-17.02	32.18	3336	3336	MS2	20/20	Glioma_Nuclear_prep_Gradeall_4plex.raw
217330646		High	gISLNPEQWSQLk	Unambiguous	1	1	N-Term(iTRAQ4plex); K13(iTRAQ4plex)	HCD	1.0000	0.0000	1	1	11892	Unique	Used	2.227	2.211E2	5.368E2	201	4.02	0.00	0	25	300	3	596.66736	1787.98752	-1.08	70.08	6625	6625	MS2	21/48	Glioma_Nuclear_prep_Gradeall_repeat_4plex.raw
217330646	High		gISLNPEQWSQLk	Unambiguous	1	1	N-Term(iTRAQ4plex); K13(iTRAQ4plex)	HCD	1.0000	0.0000	1	1	11102	Unique	Used	2.107	2.670E4	6.173E4	203	5.18	0.00	0	20	34	2	894.49408	1787.98088	-4.80	49.25	5832	5832	MS2	22/24	Glioma_Nuclear_prep_Gradeall_4plex.raw
221316767	NP_001138246.1	ELAVL4	1996	ELAV-like protein 4 isoform 2 [Homo sapiens]	24.04	6	1	2	0.427	1;1	15.3	23.79	366	40.4	9.39																			
221316767		High	sSQALLSQLYQSPNR	Unambiguous	6	1	N-Term(iTRAQ4plex)	HCD	1.0000	0.0000	1	1	10892	Unique	Used	0.381	2.419E3	1.005E3	278	4.28	0.00	0	63	300	2	918.49414	1835.98100	4.23	55.83	5414	5414	MS2	20/28	Glioma_Nuclear_prep_Gradeall_repeat_4plex.raw
221316767	High		sSQALLSQLYQSPNR	Unambiguous	6	1	N-Term(iTRAQ4plex)	HCD	1.0000	0.0000	1	1	10150	Unique	Used	0.474	3.118E4	1.621E4	268	3.99	0.00	0	0	43	2	918.48303	1835.95879	-7.87	41.96	4793	4793	MS2	17/28	Glioma_Nuclear_prep_Gradeall_4plex.raw
223555917	NP_848927.2	MTDH	92140	protein LYRIC [Homo sapiens]	7.04	1	3	5	0.651	3;2	6.4	13.12	582	63.8	9.32																			
223555917		High	tVEVAEGEAVR	Unambiguous	1	1	N-Term(iTRAQ4plex)	HCD	1.0000	0.0000	1	1	8093	Unique	Used	0.653	3.422E4	2.434E4	256	3.09	0.00	0	42	143	2	652.35114	1303.69499	-1.90	24.68	2424	2424	MS2	18/20	Glioma_Nuclear_prep_Gradeall_repeat_4plex.raw
223555917	High		tVEVAEGEAVR	Unambiguous	1	1	N-Term(iTRAQ4plex)	HCD	1.0000	0.0000	1	1	7923	Unique	Used	0.597	1.041E5	6.818E4	250	3.13	0.00	0	51	27	2	652.35291	1303.69853	0.81	25.81	2369	2369	MS2	18/20	Glioma_Nuclear_prep_Gradeall_4plex.raw
223555917		High	tELGLDLGLEPk	Unambiguous	1	1	N-Term(iTRAQ4plex); K12(iTRAQ4plex)	HCD	1.0000	0.0000	1	1	11316	Unique	Used	0.572	3.087E3	1.923E3	183	4.76	0.00	0	38	300	2	786.96039	1572.91350	3.02	61.57	5909	5909	MS2	21/22	Glioma_Nuclear_prep_Gradeall_repeat_4plex.raw
223555917	High		tELGLDLGLEPk	Unambiguous	1	1	N-Term(iTRAQ4plex); K12(iTRAQ4plex)	HCD	1.0000	0.0000	1	1	10495	Unique	Used	0.662	8.597E4	6.248E4	224	5.08	0.00	0	30	24	2	786.94739	1572.88750	-13.52	44.61	5171	5171	MS2	21/22	Glioma_Nuclear_prep_Gradeall_4plex.raw
223555917	High		eEAAAVPAAAPDDLALLk	Unambiguous	1	1	N-Term(iTRAQ4plex); K18(iTRAQ4plex)	HCD	1.0000	0.0000	1	1	10623	Unique	Used	0.651	1.343E4	9.595E3	215	4.27	0.00	0	26	52	2	1027.05896	2053.11064	-15.30	45.56	5310	5310	MS2	17/34	Glioma_Nuclear_prep_Gradeall_4plex.raw
228008295	NP_001153147.1	SYNCRIP	10492	heterogeneous nuclear ribonucleoprotein Q isoform 4 [Homo sapiens]	30.78	7	9	15	0.383	12;3	33.2	106.20	588	65.6	8.60																			
228008295	High		IMMDPLTGLNR	Unambiguous	7	1	N-Term(iTRAQ4plex)	HCD	1.0000	0.0000	1	1	10909	Unique	Used	0.386	1.941E5	8.210E4	199	3.24	0.00	0	34	23	2	702.86682	1404.72637	-14.00	47.75	5622	5622	MS2	16/20	Glioma_Nuclear_prep_Gradeall_4plex.raw
228008295	High		IDEIYVAGLVAHSDLDER	Unambiguous	5	1	N-Term(iTRAQ4plex)	HCD	1.0000	0.0000	1	1	11593	Unique	Used	0.443	1.831E4	8.902E3	166	7.84	0.00	0	15	13	3	720.37469	2159.10953	-0.29	53.36	6369	6369	MS2	40/68	Glioma_Nuclear_prep_Gradeall_4plex.raw
228008295	High		gYAFVTFcTk	Unambiguous	7	1	N-Term(iTRAQ4plex); C8(Methylthio); K10(iTRAQ4plex)	HCD	1.0000	0.0000	1	1	11560	Unique	Used	0.658	5.427E4	3.920E4	177	4.03	0.00	0	69	77	2	735.87152	1470.73577	-0.42	53.09	6333	6333	MS2	15/18	Glioma_Nuclear_prep_Gradeall_4plex.raw
228008295		High	nLANTVTEEILEk	Unambiguous	7	1	N-Term(iTRAQ4plex); K13(iTRAQ4plex)	HCD	1.0000	0.0000	1	1	12013	Unique	Used	0.246	2.906E3	7.800E2	211	5.69	0.00	0	34	300	2	881.49847	1761.98967	3.38	72.25	6799	6799	MS2	24/24	Glioma_Nuclear_prep_Gradeall_repeat_4plex.raw
228008295	High		dLFEDELVPLFEk	Unambiguous	7	1	N-Term(iTRAQ4plex); K13(iTRAQ4plex)	HCD	1.0000	0.0000	1	1	12852	Unique	Used	0.202	3.935E3	8.719E2	196	3.46	0.00	0	7	49	3	628.01019	1882.01603	3.80	67.21	7783	7783	MS2	19/48	Glioma_Nuclear_prep_Gradeall_4plex.raw
228008295	High		dLFEDELVPLFEk	Unambiguous	7	1	N-Term(iTRAQ4plex); K13(iTRAQ4plex)	HCD	1.0000	0.0000	1	1	12851	Unique	Used	0.259	7.689E4	2.184E4	203	5.71	0.00	0	8	61	2	941.51093	1882.01457	3.03	67.20	7782	7782	MS2	19/24	Glioma_Nuclear_prep_Gradeall_4plex.raw
228008295	High		nLANTVTEEILEk	Unambiguous	7	1	N-Term(iTRAQ4plex); K13(iTRAQ4plex)	HCD	1.0000	0.0000	1	1	11247	Unique	Used	0.289	9.206E4	2.922E4	210	5.95	0.00	0	20	44	2	881.49792	1761.98857	2.76	50.42	5991	5991	MS2	24/24	Glioma_Nuclear_prep_Gradeall_4plex.raw
228008295	High		dLFEDELVPLFEk	Unambiguous	7	1	N-Term(iTRAQ4plex); K13(iTRAQ4plex)	HCD	1.0000	0.0000	1	1	12816	Unique	Used	0.406	6.014E3	2.680E3	209	5.82	0.00	0	8	300	2	941.50970	1882.01213	1.73	66.56	7732	7732	MS2	20/24	Glioma_Nuclear_prep_Gradeall_4plex.raw
228008295	High		eFNEDGALAVLQQFk	Unambiguous	5	1	N-Term(iTRAQ4plex); K15(iTRAQ4plex)	HCD	1.0000	0.0000	1	1	12405	Unique	Used	0.379	1.690E4	7.024E3	199	7.53	0.00	0	40	300	2	999.03064	1997.05400	-2.15	61.19	7257	7257	MS2	24/28	Glioma_Nuclear_prep_Gradeall_4plex.raw
228008295	High		eFNEDGALAVLQQFk	Unambiguous	5	1	N-Term(iTRAQ4plex); K15(iTRAQ4plex)	HCD	1.0000	0.0000	1	1	12399	Unique	Used	0.652	2.643E3	1.892E3	200	7.04	0.00	0	40	288	3	666.35730	1997.05735	-0.47	61.11	7250	7250	MS2	26/56	Glioma_Nuclear_prep_Gradeall_4plex.raw
228008295		High	vTEGLTDVILYHQPDDk	Unambiguous	7	1	N-Term(iTRAQ4plex); K17(iTRAQ4plex)	HCD	1.0000	0.0000	1	1	11260	Unique	Used	0.495	9.184E2	4.950E2	170	6.83	0.00	0	21	300	3	744.39984	2231.18497	2.30	60.58	5830	5830	MS2	34/64	Glioma_Nuclear_prep_Gradeall_repeat_4plex.raw
228008295	High		vTEGLTDVILYHQPDDk	Unambiguous	7	1	N-Term(iTRAQ4plex); K17(iTRAQ4plex)	HCD	1.0000	0.0000	1	1	10341	Unique	Used	0.383	5.304E4	2.229E4	148	6.68	0.00	0	13	6	3	744.38763	2231.14835	-14.12	43.39	5001	5001	MS2	34/64	Glioma_Nuclear_prep_Gradeall_4plex.raw
228008295	High		yGGPPPDSVYSGQQPSVGTEIFVGk	Unambiguous	6	1	N-Term(iTRAQ4plex); K25(iTRAQ4plex)	HCD	1.0000	0.0000	1	1	10671	Unique	Used	0.344	7.319E3	2.766E3	94	5.60	0.00	0	4	24	3	952.14148	2854.40989	-14.14	45.93	5363	5363	MS2	29/96	Glioma_Nuclear_prep_Gradeall_4plex.raw
228008295		High	eQILEEFSk	Unambiguous	7	1	N-Term(iTRAQ4plex); K9(iTRAQ4plex)	HCD	1.0000	0.0000	1	1	10673	Unique	Used	0.381	1.419E4	5.888E3	298	4.03	0.00	0	24	300	2	705.89209	1410.77690	3.54	53.11	5175	5175	MS2	16/16	Glioma_Nuclear_prep_Gradeall_repeat_4plex.raw
228008295	High		eQILEEFSk	Unambiguous	7	1	N-Term(iTRAQ4plex); K9(iTRAQ4plex)	HCD	1.0000	0.0000	1	1	9923	Unique	Used	0.479	2.914E5	1.532E5	269	3.78	0.00	0	28	11	2	705.87970	1410.75212	-14.02	40.30	4546	4546	MS2	16/16	Glioma_Nuclear_prep_Gradeall_4plex.raw
264681410	NP_001161086.1	LEMD3	23592	inner nuclear membrane protein Man1 isoform 2 [Homo sapiens]	3.85	2	3	5	0.486	3;2	19.6	14.00	910	99.8	7.78																			
264681410	High		iGGADFLVWR	Unambiguous	2	1	N-Term(iTRAQ4plex)	HCD	1.0000	0.0000	1	1	11505	Unique	Used	0.578	3.771E4	2.391E4	270	3.44	0.00	0	43	77	2	639.35510	1277.70293	-7.36	52.64	6273	6273	MS2	16/18	Glioma_Nuclear_prep_Gradeall_4plex.raw
264681410		High	tLSVQEAAAYLk	Unambiguous	2	1	N-Term(iTRAQ4plex); K12(iTRAQ4plex)	HCD	1.0000	0.0000	1	1	11387	Unique	Used	0.486	4.582E3	2.427E3	225	5.41	0.00	0	36	300	2	791.46039	1581.91350	2.80	62.61	5996	5996	MS2	21/22	Glioma_Nuclear_prep_Gradeall_repeat_4plex.raw
264681410	High		tLSVQEAAAYLk	Unambiguous	2	1	N-Term(iTRAQ4plex); K12(iTRAQ4plex)	HCD	1.0000	0.0000	1	1	10562	Unique	Used	0.663	1.053E5	7.662E4	256	4.81	0.00	0	55	26	2	791.44751	1581.88774	-13.48	45.10	5244	5244	MS2	21/22	Glioma_Nuclear_prep_Gradeall_4plex.raw
264681410		High	ISEPEEELLQQFk	Unambiguous	2	1	N-Term(iTRAQ4plex); K13(iTRAQ4plex)	HCD	1.0000	0.0000	1	1	12240	Unique	Used	0.415	3.276E2	1.483E2	202	4.52	0.00	0	19	300	3	626.67609	1878.01371	2.01	77.20	7181	7181	MS2	24/48	Glioma_Nuclear_prep_Gradeall_repeat_4plex.raw
264681410	High		ISEPEEELLQQFk	Unambiguous	2	1	N-Term(iTRAQ4plex); K13(iTRAQ4plex)	HCD	1.0000	0.0000	1	1	11644	Unique	Used	0.448	3.373E4	1.659E4	220	5.75	0.00	0	33	142	2	939.51208	1878.01689	3.71	53.80	6424	6424	MS2	22/24	Glioma_Nuclear_prep_Gradeall_4plex.raw
283806604	NP_001164554.1	SMPD4	55627	sphingomyelin phosphodiesterase 4 isoform 3 [Homo sapiens]	6.81	3	2	3	1.719	2;1	27.6	11.83	764	86.1	8.65																			
283806604		High	gEQLFLEPELVIPHR	Unambiguous	3	1	N-Term(iTRAQ4plex)	HCD	1.0000	0.0000	1	1	12084	Unique	Used	1.673	1.224E2	2.231E2	201	4.05	0.00	0	36	300	3	641.02783	1921.06894	1.30	73.97	6928	6928	MS2	20/56	Glioma_Nuclear_prep_Gradeall_repeat_4plex.raw
283806604	High		gEQLFLEPELVIPHR	Unambiguous	3	1	N-Term(iTRAQ4plex)	HCD	1.0000	0.0000	1	1	11277	Unique	Used	2.660	3.319E3	9.689E3	198	4.52	0.00	0	52	54	3	641.02673	1921.06565	-0.42	50.66	6023	6023	MS2	21/56	Glioma_Nuclear_prep_Gradeall_4plex.raw
283806604	High		gLPDcIVGEDGLILTPLGR	Unambiguous	3	1	N-Term(iTRAQ4plex); C5(Methylthio)	HCD	1.0000	0.0000	1	1	12815	Unique	Used	1.719	2.623E2	4.949E2	166	4.00	0.00	0	12	300	3	733.72546	2199.16184	-0.75	66.55	7731	7731	MS2	20/72	Glioma_Nuclear_prep_Gradeall_4plex.raw
302129652	NP_001180473.1	ANXA6	309	annexin A6 isoform 2 [Homo sapiens]	14.35	2	6	8	2.613	6;2	38.4	45.45	641	72.4	5.60																			
302129652		High	eAILDIITSR	Unambiguous	2	1	N-Term(iTRAQ4plex)	HCD	1.0000	0.0000	1	1	11938	Unique	Used	3.441	1.128E3	4.230E3	324	3.33	0.00	0	47	300	2	637.87555	1274.74382	0.11	70.87	6689	6689	MS2	16/18	Glioma_Nuclear_prep_Gradeall_repeat_4plex.raw
302129652	High		gLGTDEDTIIDIITHR	Unambiguous	2	1	N-Term(iTRAQ4plex)	HCD	1.0000	0.0000	1	1	12331	Unique	Used	1.407	9.266E2	1.430E3	169	6.66	0.00	0	36	226	3	638.34045	1913.00681	-1.52	60.37	7176	7176	MS2	27/60	Glioma_Nuclear_prep_Gradeall_4plex.raw
302129652	High		sEIDLLNIR	Unambiguous	2	1	N-Term(iTRAQ4plex)	HCD	1.0000	0.0000	1	1	10337	Unique	Used	1.644	3.066E4	5.531E4	274	3.00	0.00	0	63	56	2	608.84528	1216.68328	-15.24	43.35	4996	4996	MS2	15/16	Glioma_Nuclear_prep_Gradeall_4plex.raw

302129652	High		gLGTDEDTIIDIITHR	Unambiguous	2	1	N-Term(iTRAQ4plex)	HCD	1.0000	0.0000	1	1	12389	Unique	Used	2.576	1.186E3	3.352E3	148	7.08	0.00	0	9	66	3	638.34082	1913.00791	-0.94	61.03	7240	7240	MS2	29/60	Glioma_Nuclear_prep_Gradeall_4plex.raw
302129652	High		aLIEILATR	Unambiguous	2	1	N-Term(iTRAQ4plex)	HCD	1.0000	0.0000	1	1	10974	Unique	Used	2.923	1.131E4	3.628E4	186	3.36	0.00	0	57	97	2	572.35565	1143.70403	-15.56	48.24	5692	5692	MS2	15/16	Glioma_Nuclear_prep_Gradeall_4plex.raw
302129652	High		aLIEILATR	Unambiguous	2	1	N-Term(iTRAQ4plex)	HCD	1.0000	0.0000	1	1	11018	Unique	Used	4.542	2.733E4	1.362E5	137	3.28	0.00	0	20	18	2	572.36298	1143.71868	-2.75	48.58	5740	5740	MS2	15/16	Glioma_Nuclear_prep_Gradeall_4plex.raw
302129652	High		lIVGLMRPPAYcDAk	Unambiguous	2	1	N-Term(iTRAQ4plex); C12(Methylthio); K15(iTRAQ4plex)	HCD	1.0000	0.0000	1	1	11160	Unique	Used	2.650	6.246E3	1.816E4	161	3.43	0.00	0	28	54	3	661.02423	1981.05814	-4.78	49.72	5896	5896	MS2	16/56	Glioma_Nuclear_prep_Gradeall_4plex.raw
302129652		High	vFQEFIk	Unambiguous	2	1	N-Term(iTRAQ4plex); K7(iTRAQ4plex)	HCD	1.0000	0.0000	1	1	10878	Unique	Used	2.007	3.929E3	8.594E3	253	2.83	0.00	0	69	300	2	599.85590	1198.70452	-2.45	55.64	5398	5398	MS2	12/12	Glioma_Nuclear_prep_Gradeall_repeat_4plex.raw
306482694	NP_001182375.1	SRSF7	6432	serine/arginine-rich splicing factor 7 isoform 2 [Homo sapiens]	19.47	2	3	6	0.644	2;4	7.6	42.64	226	26.0	11.77																			
306482694		High	aFSYYGPLR	Unambiguous	2	1	N-Term(iTRAQ4plex)	HCD	1.0000	0.0000	1	1	10405	Unique	Used	0.628	2.283E4	1.563E4	227	3.19	0.00	0	11	234	2	609.32690	1217.64653	2.45	49.89	4887	4887	MS2	14/16	Glioma_Nuclear_prep_Gradeall_repeat_4plex.raw
306482694	High		aFSYYGPLR	Unambiguous	2	1	N-Term(iTRAQ4plex)	HCD	1.0000	0.0000	1	1	9638	Unique	Used	0.691	1.360E6	1.031E6	153	3.21	0.00	0	13	2	2	609.31641	1217.62554	-14.79	38.24	4237	4237	MS2	14/16	Glioma_Nuclear_prep_Gradeall_4plex.raw
306482694		High	gHYAYDcHR	Unambiguous	2	1	N-Term(iTRAQ4plex); C7(Methylthio)	HCD	1.0000	0.0000	1	1	7218	Unique	Used	0.661	3.133E4	2.256E4	7	3.48	0.00	0	8	76	2	656.26703	1311.52678	-16.13	16.69	1499	1499	MS2	16/16	Glioma_Nuclear_prep_Gradeall_repeat_4plex.raw
306482694		High	vYVGNLGTGAGk	Unambiguous	2	1	N-Term(iTRAQ4plex); K12(iTRAQ4plex)	HCD	1.0000	0.0000	1	1	9041	Unique	Used	0.622	5.910E3	4.007E3	244	4.77	0.00	0	43	300	2	712.41272	1423.81816	2.38	34.21	3426	3426	MS2	19/22	Glioma_Nuclear_prep_Gradeall_repeat_4plex.raw
306482694		High	vYVGNLGTGAGk	Unambiguous	2	1	N-Term(iTRAQ4plex); K12(iTRAQ4plex)	HCD	1.0000	0.0000	1	1	8922	Unique	Used	0.704	3.866E4	2.968E4	240	5.16	0.00	0	44	188	2	712.40796	1423.80864	-4.31	32.84	3297	3297	MS2	18/22	Glioma_Nuclear_prep_Gradeall_repeat_4plex.raw
306482694	High		vYVGNLGTGAGk	Unambiguous	2	1	N-Term(iTRAQ4plex); K12(iTRAQ4plex)	HCD	1.0000	0.0000	1	1	8516	Unique	Used	0.572	4.771E5	2.994E5	244	4.51	0.00	0	20	7	2	712.39966	1423.79204	-15.97	30.01	3012	3012	MS2	21/22	Glioma_Nuclear_prep_Gradeall_4plex.raw
316659409	NP_001186883.1	ACTG1	71	actin, cytoplasmic 2 [Homo sapiens]	36.27	8	3	7	2.430	4;3	16.7	59.70	375	41.8	5.48																			
316659409		High	gYSFTTTAER	Unambiguous	2	1	N-Term(iTRAQ4plex)	HCD	1.0000	0.0000	1	1	8397	Unique	Used	2.655	1.884E4	5.452E4	120	4.01	0.00	0	22	132	2	638.81586	1276.62444	-3.59	27.63	2744	2744	MS2	16/18	Glioma_Nuclear_prep_Gradeall_repeat_4plex.raw
316659409	High		gYSFTTTAER	Unambiguous	2	1	N-Term(iTRAQ4plex)	HCD	1.0000	0.0000	1	1	8118	Unique	Used	1.957	2.572E5	5.525E5	83	3.54	0.00	0	25	5	2	638.80688	1276.60649	-17.65	27.20	2581	2581	MS2	15/18	Glioma_Nuclear_prep_Gradeall_4plex.raw
316659409	High		tTGIVMDSGDGVTHTVPIYEGYALPHAILR	Unambiguous	2	1	N-Term(iTRAQ4plex)	HCD	1.0000	0.0000	1	1	11280	Unique	Used	2.218	8.101E2	1.971E3	77	8.19	0.00	0	0	75	3	1109.90894	3327.71225	-1.28	50.68	6026	6026	MS2	31/116	Glioma_Nuclear_prep_Gradeall_4plex.raw
316659409		High	vAPEEHPVLLTEAPLNPk	Unambiguous	2	1	N-Term(iTRAQ4plex); K18(iTRAQ4plex)	HCD	1.0000	0.0000	1	1	10387	Unique	Used	1.720	9.502E2	1.782E3	113	7.07	0.00	0	12	200	3	748.09558	2242.27219	1.59	49.69	4868	4868	MS2	31/68	Glioma_Nuclear_prep_Gradeall_repeat_4plex.raw
316659409		High	vAPEEHPVLLTEAPLNPk	Unambiguous	2	1	N-Term(iTRAQ4plex); K18(iTRAQ4plex)	HCD	1.0000	0.0000	1	1	10474	Unique	Used	2.652	5.160E2	1.492E3	86	6.98	0.00	0	11	300	3	748.09558	2242.27219	1.59	50.71	4960	4960	MS2	30/68	Glioma_Nuclear_prep_Gradeall_repeat_4plex.raw
316659409	High		vAPEEHPVLLTEAPLNPk	Unambiguous	2	1	N-Term(iTRAQ4plex); K18(iTRAQ4plex)	HCD	1.0000	0.0000	1	1	9604	Unique	Used	2.430	4.212E4	1.123E5	96	5.42	0.00	0	0	8	2	1121.61926	2242.23125	-16.67	38.00	4200	4200	MS2	22/34	Glioma_Nuclear_prep_Gradeall_4plex.raw
316659409	High		vAPEEHPVLLTEAPLNPk	Unambiguous	2	1	N-Term(iTRAQ4plex); K18(iTRAQ4plex)	HCD	1.0000	0.0000	1	1	9591	Unique	Used	2.753	2.845E4	8.595E4	115	5.98	0.00	0	6	3	3	748.08301	2242.23447	-15.23	37.91	4186	4186	MS2	30/68	Glioma_Nuclear_prep_Gradeall_4plex.raw
332801090	NP_001193929.1	HNRNPDL	9987	heterogeneous nuclear ribonucleoprotein D-like isoform b [Homo sapiens]	28.10	2	9	24	0.315	12;12	30.9	88.24	363	40.0	9.96																			
332801090		High	qLAPLLPSLAPSSAR	Unambiguous	2	1	N-Term(iTRAQ4plex)	HCD	1.0000	0.0000	1	1	11542	Unique	Used	0.330	3.116E3	1.119E3	154	3.49	0.00	0	31	300	2	832.99683	1664.98638	2.84	64.70	6177	6177	MS2	15/28	Glioma_Nuclear_prep_Gradeall_repeat_4plex.raw
332801090		High	dLTEYLSR	Unambiguous	2	1	N-Term(iTRAQ4plex)	HCD	1.0000	0.0000	1	1	10178	Unique	Used	0.496	1.041E4	5.629E3	264	2.84	0.00	0	46	300	2	570.80573	1140.60417	2.13	47.21	4645	4645	MS2	13/14	Glioma_Nuclear_prep_Gradeall_repeat_4plex.raw
332801090	High		qLAPLLPSLAPSSAR	Unambiguous	2	1	N-Term(iTRAQ4plex)	HCD	1.0000	0.0000	1	1	10721	Unique	Used	0.410	1.045E5	4.704E4	212	3.47	0.00	0	28	27	2	832.98395	1664.96062	-12.63	46.31	5418	5418	MS2	17/28	Glioma_Nuclear_prep_Gradeall_4plex.raw
332801090		High	gFcFITYTDEEPVk	Unambiguous	2	1	N-Term(iTRAQ4plex); C3(Methylthio); K14(iTRAQ4plex)	HCD	1.0000	0.0000	1	1	12298	Unique	Used	0.224	1.708E3	4.168E2	109	4.55	0.00	0	3	300	2	991.97919	1982.95110	1.44	78.97	7311	7311	MS2	22/26	Glioma_Nuclear_prep_Gradeall_repeat_4plex.raw
332801090		High	gFcFITYTDEEPVk	Unambiguous	2	1	N-Term(iTRAQ4plex); C3(Methylthio); K14(iTRAQ4plex)	HCD	1.0000	0.0000	1	1	12294	Unique	Used	0.271	2.417E2	7.129E1	119	4.33	0.00	0	40	300	3	661.65570	1982.95255	2.17	78.86	7303	7303	MS2	22/52	Glioma_Nuclear_prep_Gradeall_repeat_4plex.raw
332801090	High		gFcFITYTDEEPVk	Unambiguous	2	1	N-Term(iTRAQ4plex); C3(Methylthio); K14(iTRAQ4plex)	HCD	1.0000	0.0000	1	1	11811	Unique	Used	0.266	6.233E4	1.820E4	105	5.49	0.00	0	15	48	2	991.97717	1982.94707	-0.59	55.30	6606	6606	MS2	23/26	Glioma_Nuclear_prep_Gradeall_4plex.raw
332801090	High		gFcFITYTDEEPVk	Unambiguous	2	1	N-Term(iTRAQ4plex); C3(Methylthio); K14(iTRAQ4plex)	HCD	1.0000	0.0000	1	1	11735	Unique	Used	0.442	2.610E4	1.267E4	105	5.50	0.00	0	44	204	2	991.97693	1982.94658	-0.84	54.64	6523	6523	MS2	20/26	Glioma_Nuclear_prep_Gradeall_4plex.raw
332801090		High	gFcFITYTDEEPVkk	Unambiguous	2	1	N-Term(iTRAQ4plex); C3(Methylthio); K14(iTRAQ4plex); K15(iTRAQ4plex)	HCD	1.0000	0.0000	1	1	11783	Unique	Used	0.312	1.061E3	3.611E2	114	5.19	0.00	1	10	300	3	752.38953	2255.15403	3.88	68.26	6476	6476	MS2	31/56	Glioma_Nuclear_prep_Gradeall_repeat_4plex.raw
332801090	High		gFcFITYTDEEPVkk	Unambiguous	2	1	N-Term(iTRAQ4plex); C3(Methylthio); K14(iTRAQ4plex); K15(iTRAQ4plex)	HCD	1.0000	0.0000	1	1	10731	Unique	Used	0.186	9.524E4	1.942E4	88	4.89	0.00	1	12	4	3	752.37750	2255.11795	-12.12	46.39	5429	5429	MS2	30/56	Glioma_Nuclear_prep_Gradeall_4plex.raw
332801090		High	kDLTEYLSR	Unambiguous	2	1	N-Term(iTRAQ4plex); K1(iTRAQ4plex)	HCD	1.0000	0.0000	1	1	9333	Unique	Used	0.318	1.740E4	6.026E3	203	3.11	0.00	1	35	300	2	706.90540	1412.80351	3.35	37.44	3734	3734	MS2	15/16	Glioma_Nuclear_prep_Gradeall_repeat_4plex.raw
332801090		High	kDLTEYLSR	Unambiguous	2	1	N-Term(iTRAQ4plex); K1(iTRAQ4plex)	HCD	1.0000	0.0000	1	1	9324	Unique	Used	0.473	4.108E3	2.117E3	184	3.65	0.00	1	34	127	3	471.60529	1412.80130	1.79	37.36	3725	3725	MS2	18/32	Glioma_Nuclear_prep_Gradeall_repeat_4plex.raw
332801090		High	mFIGGLSWDTSk	Unambiguous	2	1	N-Term(iTRAQ4plex); K12(iTRAQ4plex)	HCD	1.0000	0.0000	1	1	11944	Unique	Used	0.299	3.861E3	1.260E3	202	3.93	0.00	0	34	300	2	815.43347	1629.85967	2.90	71.03	6701	6701	MS2	19/22	Glioma_Nuclear_prep_Gradeall_repeat_4plex.raw
332801090	High		mFIGGLSWDTSk	Unambiguous	2	1	N-Term(iTRAQ4plex); K12(iTRAQ4plex)	HCD	1.0000	0.0000	1	1	11143	Unique	Used	0.281	1.042E5	3.210E4	209	4.66	0.00	0	34	40	2	815.43390	1629.86052	3.42	49.57	5877	5877	MS2	20/22	Glioma_Nuclear_prep_Gradeall_4plex.raw
332801090		High	vFVGGLSPDTSEEQIk	Unambiguous	2	1	N-Term(iTRAQ4plex); K16(iTRAQ4plex)	HCD	1.0000	0.0000	1	1	10587	Unique	Used	0.324	7.332E3	2.588E3	214	6.24	0.00	0	0	300	2	997.53955	1994.07182	1.66	52.08	5082	5082	MS2	25/30	Glioma_Nuclear_prep_Gradeall_repeat_4plex.raw
332801090		High	vFVGGLSPDTSEEQIk	Unambiguous	2	1	N-Term(iTRAQ4plex); K16(iTRAQ4plex)	HCD	1.0000	0.0000	1	1	10583	Unique	Used	0.632	1.283E3	8.837E2	222	6.87	0.00	0	16	300	3	665.36316	1994.07492	3.21	52.03	5078	5078	MS2	24/60	Glioma_Nuclear_prep_Gradeall_repeat_4plex.raw
332801090	High		vFVGGLSPDTSEEQIk	Unambiguous	2	1	N-Term(iTRAQ4plex); K16(iTRAQ4plex)	HCD	1.0000	0.0000	1	1	9906	Unique	Used	0.258	8.145E5	2.305E5	199	5.94	0.00	0	7	4	2	997.52344	1994.03960	-14.50	40.18	4528	4528	MS2	24/30	Glioma_Nuclear_prep_Gradeall_4plex.raw
332801090	High		eYFGAFGEIENIELPMDTk	Unambiguous	2	1	N-Term(iTRAQ4plex); K19(iTRAQ4plex)	HCD	1.0000	0.0000	1	1	12673	Unique	Used	0.255	8.617E3	2.411E3	89	8.05	0.00	0	3	283	2	1246.11792	2491.22856	-0.82	64.66	7567	7567	MS2	28/36	Glioma_Nuclear_prep_Gradeall_4plex.raw
332801090	High		eYFGAFGEIENIELPMDTk	Unambiguous	2	1	N-Term(iTRAQ4plex); K19(iTRAQ4plex)	HCD	1.0000	0.0000	1	1	12675	Unique	Used	0.326	2.285E3	8.161E2	99	7.33	0.00	0	13	67	3	831.08154	2491.23008	-0.21	64.70	7570	7570	MS2	29/72	Glioma_Nuclear_prep_Gradeall_4plex.raw
332801090	High		yHQIGSGk	Unambiguous	2	1	N-Term(iTRAQ4plex); K8(iTRAQ4plex)	HCD	1.0000	0.0000	1	1	7151	Unique	Used	0.254	5.367E4	1.499E4	361	3.61	0.00	0	41	50	3	393.22375	1177.65671	-0.06	20.16	1530	1530	MS2	15/28	Glioma_Nuclear_prep_Gradeall_4plex.raw
332801090		High	mFIGGLSWDTSk	Unambiguous	2	1	N-Term(iTRAQ4plex); M1(Oxidation); K12(iTRAQ4plex)	HCD	1.0000	0.0000	1	1	11174	Unique	Used	0.333	4.852E3	1.764E3	197	3.97	0.00	0	25	300	2	823.43127	1645.85527	3.29	59.46	5732	5732	MS2	20/22	Glioma_Nuclear_prep_Gradeall_repeat_4plex.raw
332801090		High	mFIGGLSWDTSk	Unambiguous	2	1	N-Term(iTRAQ4plex); M1(Oxidation); K12(iTRAQ4plex)	HCD	1.0000	0.0000	1	1	11183	Unique	Used	0.412	1.075E3	4.822E2	205	3.79	0.00	0	15	300	3	549.28864	1645.85135	0.91	59.58	5743	5743	MS2	19/44	Glioma_Nuclear_prep_Gradeall_repeat_4plex.raw
332801090	High		mFIGGLSWDTSk	Unambiguous	2	1	N-Term(iTRAQ4plex); M1(Oxidation); K12(iTRAQ4plex)	HCD	1.0000	0.0000	1	1	10370	Unique	Used	0.291	1.272E5	4.056E4	150	3.98	0.00	0	51	29	2	823.41852	1645.82976	-12.21	43.62	5033	5033	MS2	19/22	Glioma_Nuclear_prep_Gradeall_4plex.raw
332801090	High		eYFGAFGEIENIELPmDTk	Unambiguous	2	1	N-Term(iTRAQ4plex); M16(Oxidation); K19(iTRAQ4plex)	HCD	1.0000	0.0000	1	1	12276	Unique	Used	0.276	6.125E3	1.855E3	124	6.96	0.00	0	5	209	2	1254.11523	2507.22319	-0.93	59.77	7115	7115	MS2	25/36	Glioma_Nuclear_prep_Gradeall_4plex.raw
332801090	High		eYFGAFGEIENIELPmDTk	Unambiguous	2	1	N-Term(iTRAQ4plex); M16(Oxidation); K19(iTRAQ4plex)	HCD	1.0000	0.0000	1	1	12269	Unique	Used	0.492	3.180E3	1.718E3	144	6.98	0.00	0	16	54	3	836.41437	2507.22855	1.21	59.71	7108	7108	MS2	30/72	Glioma_Nuclear_prep_Gradeall_4plex.raw
378404908	NP_001243728.1	GAPDH	2597	glyceraldehyde-3-phosphate dehydrogenase isoform 2 [Homo sapiens]	9.90	2	2	4	2.460	2;2	8.1	9.94	293	31.5	7.61																			
378404908		High	vPTANVSVVDLTcR	Unambiguous	2	1	N-Term(iTRAQ4plex); C13(Methylthio)	HCD	1.0000	0.0000	1	1	11641	Unique	Used	2.349	3.330E3	8.527E3	216	4.67	0.00	0	22	300	2	832.43585	1663.86443	0.95	66.12	6297	6297	MS2	17/26	Glioma_Nuclear_prep_Gradeall_repeat_4plex.raw
378404908	High		vPTANVSVVDLTcR	Unambiguous	2	1	N-Term(iTRAQ4plex); C13(Methylthio)	HCD	1.0000	0.0000	1	1	10846	Unique	Used	2.817	8.852E4	2.736E5	110	4.43	0.00	0	14	12	2	832.42334	1663.83940	-14.09	47.27	5554	5554	MS2	17/26	Glioma_Nuclear_prep_Gradeall_4plex.raw
378404908		High	gALQNIIPASTGAAk	Unambiguous	2	1	N-Term(iTRAQ4plex); K15(iTRAQ4plex)	HCD	1.0000	0.0000	1	1	10127	Unique	Used	2.492	2.585E3	7.019E3	142	5.27	0.00	0	37	300	2	850.50165	1699.99602	0.86	46.61	4591	4591	MS2	19/28	Glioma_Nuclear_prep_Gradeall_repeat_4plex.raw
378404908	High		gALQNIIPASTGAAk	Unambiguous	2	1	N-Term(iTRAQ4plex); K15(iTRAQ4plex)	HCD	1.0000	0.0000	1	1	9476	Unique	Used	2.428	5.659E4	1.508E5	195	4.56	0.00	0	60	19	2	850.48730	1699.96733	-16.01	37.08	4061	4061	MS2	19/28	Glioma_Nuclear_prep_Gradeall_4plex.raw
379317177	NP_001243839.1	DDX21	9188	nucleolar RNA helicase 2 isoform 2 [Homo sapiens]	11.75	2	5	8	1.599	7;1	17.8	31.54	715	79.6	9.38																			
379317177		High	aPQVLVLAPTR	Unambiguous	2	1	N-Term(iTRAQ4plex)	HCD	1.0000	0.0000	1	1	10112	Unique	Used	1.585	8.087E3	1.397E4	165	4.06	0.00	0	51	286	2	654.90997	1308.81267	0.48	46.43	4575	4575	MS2	16/20	Glioma_Nuclear_prep_Gradeall_repeat_4plex.raw
379317177	High		aPQVLVLAPTR	Unambiguous	2	1	N-Term(iTRAQ4plex)	HCD	1.0000	0.0000	1	1	9479	Unique	Used	1.614	4.427E4	7.842E4	204	3.70	0.00	0	58	31	2	654.89917	1308.79106	-16.03	37.09	4064	4064	MS2	15/20	Glioma_Nuclear_prep_Gradeall_4plex.raw
379317177	High		gAVEALAAALAHISGATSVDQR	Unambiguous	2	1	N-Term(iTRAQ4plex)	HCD	1.0000	0.0000	1	1	12975	Unique	Used	1.756	1.700E2	3.276E2	109	5.36	0.00	0	80	300	3	751.40997	2252.21537	1.66	69.44	7957	7957	MS2	38/84	Glioma_Nuclear_prep_Gradeall_4plex.raw
379317177	High		gVTFLFPIQAk	Unambiguous	2	1	N-Term(iTRAQ4plex); K11(iTRAQ4plex)	HCD	1.0000	0.0000	1	1	11480	Unique	Used	1.385	3.050E3	4.635E3	169	4.11	0.00	0	24	56	3	503.64020	1508.90604	-1.27	52.43	6246	6246	MS2	19/40	Glioma_Nuclear_prep_Gradeall_4plex.raw
379317177	High		tFSFAIPLIEk	Unambiguous	2	1	N-Term(iTRAQ4plex); K11(iTRAQ4plex)	HCD	1.0000	0.0000	1	1	12174	Unique	Used	1.542	1.637E4	2.769E4	126	3.85	0.00	0	30	143	2	777.46649	1553.92571	4.84	58.71	7003	7003	MS2	16/20	Glioma_Nuclear_prep_Gradeall_4plex.raw
379317177	High		tFSFAIPLIEk	Unambiguous	2	1	N-Term(iTRAQ4plex); K11(iTRAQ4plex)	HCD	1.0000	0.0000	1	1	12244	Unique	Used	1.567	6.587E3	1.133E4	175	4.36	0.00	0	32	262	2	777.46283	1553.91838	0.13	59.43	7080	7080	MS2	16/20	Glioma_Nuclear_prep_Gradeall_4plex.raw
379317177	High		gVTFLFPIQAk	Unambiguous	2	1	N-Term(iTRAQ4plex); K11(iTRAQ4plex)	HCD	1.0000	0.0000	1	1	11459	Unique	Used	2.234	9.840E3	2.413E4	166	5.08	0.00	0	49	90	2	754.95648	1508.90569	-1.50	52.26	6223	6223	MS2	16/20	Glioma_Nuclear_prep_Gradeall_4plex.raw
379317177	High		lLDSVPPTAISHFk	Unambiguous	2	1	N-Term(iTRAQ4plex); K14(iTRAQ4plex)	HCD	1.0000	0.0000	1	1	10239	Unique	Used	2.168	7.144E3	1.700E4	203	5.06	0.00	0	38	23	3	605.01099	1813.01841	-15.36	42.64	4890	4890	MS2	29/52	Glioma_Nuclear_prep_Gradeall_4plex.raw
385198063	NP_001245239.1	NOP2	4839	probable 28S rRNA (cytosine(4447)-C(5))-methyltransferase isoform 4 [Homo sapiens]	5.25	4	3	3	1.558	2;1	17.4	8.63	628	69.6	6.58																			
385198063		High	dLAQALINR	Unambiguous	4	1	N-Term(iTRAQ4plex)	HCD	1.0000	0.0000	1	1	10202	Unique	Used	1.436	4.765E3	7.459E3	216	3.18	0.00	0	65	300	2	579.34186	1157.67644	0.44	47.49	4670	4670	MS2	14/16	Glioma_Nuclear_prep_Gradeall_repeat_4plex.raw
385198063	High		iQDIVGILR	Unambiguous	4	1	N-Term(iTRAQ4plex)	HCD	1.0000	0.0000	1	1	11325	Unique	Used	1.986	3.484E4	7.594E4	74	3.20	0.00	0	42	34	2	585.86975	1170.73223	-0.43	51.09	6076	6076	MS2	14/16	Glioma_Nuclear_prep_Gradeall_4plex.raw
385198063	High		vLLDAPcSGTGVISk	Unambiguous	4	1	N-Term(iTRAQ4plex); C7(Methylthio); K15(iTRAQ4plex)	HCD	1.0000	0.0000	1	1	10784	Unique	Used	1.558	2.962E4	5.066E4	274	5.43	0.00	0	24	48	2	897.47778	1793.94829	-14.56	46.79	5486	5486	MS2	21/28	Glioma_Nuclear_prep_Gradeall_4plex.raw
387598076	NP_001248376.1	GPM6A	2823	neuronal membrane glycoprotein M6-a isoform 2 [Homo sapiens]	14.40	4	3	3	1.759	2;1	14.4	7.37	243	26.8	7.36																			
387598076		High	icTVSENFLR	Unambiguous	4	1	N-Term(iTRAQ4plex); C2(Methylthio)	HCD	1.0000	0.0000	1	1	11891	Unique	Used	1.759	3.248E3	6.226E3	140	3.94	0.00	0	38	300	2	686.34784	1371.68840	0.18	70.07	6624	6624	MS2	17/18	Glioma_Nuclear_prep_Gradeall_repeat_4plex.raw
387598076	High		qFGIVTIGEEk	Unambiguous	4	1	N-Term(iTRAQ4plex); K11(iTRAQ4plex)	HCD	1.0000	0.0000	1	1	9814	Unique	Used	2.066	4.748E4	1.076E5	299	3.57	0.00	0	28	29	2	754.91913	1508.83098	-16.79	39.49	4427	4427	MS2	20/20	Glioma_Nuclear_prep_Gradeall_4plex.raw
387598076	High		skEEQELHDIHSTR	Unambiguous	4	1	N-Term(iTRAQ4plex); K2(iTRAQ4plex)	HCD	1.0000	0.0000	1	1	7417	Unique	Used	1.552	9.489E3	1.617E4	111	3.81	0.00	1	10	7	3	666.34418	1997.01798	-5.56	22.22	1821	1821	MS2	21/52	Glioma_Nuclear_prep_Gradeall_4plex.raw
388240801	NP_116126.3	LMNB2	84823	lamin-B2 [Homo sapiens]	43.39	1	23	46	0.596	24;22	19.8	155.69	620	69.9	5.59																			
388240801		High	aTSSSSGSLSATGR	Unambiguous	1	1	N-Term(iTRAQ4plex)	HCD	1.0000	0.0000	1	1	6883	Unique	Used	0.288	1.567E4	4.910E3	124	4.44	0.00	0	11	120	2	706.85699	1412.70671	-2.20	13.71	1144	1144	MS2	23/26	Glioma_Nuclear_prep_Gradeall_repeat_4plex.raw
388240801		High	mAQALEELR	Unambiguous	1	1	N-Term(iTRAQ4plex)	HCD	1.0000	0.0000	1	1	10580	Unique	Used	0.404	3.104E4	1.367E4	296	3.15	0.00	0	40	195	2	602.82794	1204.64861	0.77	52.00	5075	5075	MS2	15/16	Glioma_Nuclear_prep_Gradeall_repeat_4plex.raw
388240801		High	lAHYIDR	Unambiguous	1	1	N-Term(iTRAQ4plex)	HCD	1.0000	0.0000	1	1	8056	Unique	Used	0.526	1.225E5	7.016E4	297	2.84	0.00	0	41	50	2	516.29199	1031.57671	1.21	24.32	2385	2385	MS2	12/12	Glioma_Nuclear_prep_Gradeall_repeat_4plex.raw
388240801		High	aLELENDR	Unambiguous	1	1	N-Term(iTRAQ4plex)	HCD	1.0000	0.0000	1	1	8111	Unique	Used	0.592	7.013E4	4.526E4	200	3.19	0.00	0	11	60	2	552.28949	1103.57170	-8.75	24.86	2443	2443	MS2	14/14	Glioma_Nuclear_prep_Gradeall_repeat_4plex.raw
388240801		High	gLESDVAELR	Unambiguous	1	1	N-Term(iTRAQ4plex)	HCD	1.0000	0.0000	1	1	9856	Unique	Used	0.607	6.885E4	4.554E4	218	3.96	0.00	0	11	69	2	616.83484	1232.66240	1.66	43.28	4294	4294	MS2	17/18	Glioma_Nuclear_prep_Gradeall_repeat_4plex.raw
388240801		High	eLEEAMAGER	Unambiguous	1	1	N-Term(iTRAQ4plex)	HCD	1.0000	0.0000	1	1	8850	Unique	Used	0.669	3.033E4	2.210E4	105	3.26	0.00	0	50	267	2	639.81079	1278.61431	2.03	32.15	3221	3221	MS2	15/18	Glioma_Nuclear_prep_Gradeall_repeat_4plex.raw
388240801		High	sVFEEEVR	Unambiguous	1	1	N-Term(iTRAQ4plex)	HCD	1.0000	0.0000	1	1	8857	Unique	Used	0.672	4.984E4	3.648E4	208	3.56	0.00	0	22	84	2	569.79492	1138.58257	-3.12	32.23	3229	3229	MS2	14/14	Glioma_Nuclear_prep_Gradeall_repeat_4plex.raw
388240801		High	eGELTVAQGR	Unambiguous	1	1	N-Term(iTRAQ4plex)	HCD	1.0000	0.0000	1	1	7739	Unique	Used	0.674	1.925E4	1.413E4	215	3.12	0.00	0	28	122	2	602.32574	1203.64421	-0.68	21.31	2051	2051	MS2	15/18	Glioma_Nuclear_prep_Gradeall_repeat_4plex.raw
388240801		High	tVLVNADGEEVAMR	Unambiguous	1	1	N-Term(iTRAQ4plex)	HCD	1.0000	0.0000	1	1	9662	Unique	Used	0.722	5.226E3	4.111E3	247	4.38	0.00	0	20	300	2	824.42841	1647.84953	0.13	41.10	4089	4089	MS2	20/26	Glioma_Nuclear_prep_Gradeall_repeat_4plex.raw
388240801		High	dLESLFHR	Unambiguous	1	1	N-Term(iTRAQ4plex)	HCD	1.0000	0.0000	1	1	10542	Unique	Used	0.750	2.861E3	2.338E3	225	3.81	0.00	0	18	300	3	387.54495	1160.62030	1.92	51.55	5034	5034	MS2	17/28	Glioma_Nuclear_prep_Gradeall_repeat_4plex.raw
388240801	High		aTSSSSGSLSATGR	Unambiguous	1	1	N-Term(iTRAQ4plex)	HCD	1.0000	0.0000	1	1	7153	Unique	Used	0.328	1.958E4	7.050E3	101	4.43	0.00	0	37	127	2	706.85718	1412.70708	-1.94	20.18	1532	1532	MS2	23/26	Glioma_Nuclear_prep_Gradeall_4plex.raw
388240801	High		eGELTVAQGR	Unambiguous	1	1	N-Term(iTRAQ4plex)	HCD	1.0000	0.0000	1	1	7602	Unique	Used	0.583	1.879E5	1.201E5	187	3.08	0.00	0	28	19	2	602.32593	1203.64458	-0.38	23.54	2021	2021	MS2	17/18	Glioma_Nuclear_prep_Gradeall_4plex.raw
388240801	High		eLEEAMAGER	Unambiguous	1	1	N-Term(iTRAQ4plex)	HCD	1.0000	0.0000	1	1	8515	Unique	Used	0.596	3.905E5	2.556E5	55	3.46	0.00	0	33	8	2	639.79865	1278.59001	-16.96	30.01	3011	3011	MS2	16/18	Glioma_Nuclear_prep_Gradeall_4plex.raw
388240801	High		lALDMEINAYR	Unambiguous	1	1	N-Term(iTRAQ4plex)	HCD	1.0000	0.0000	1	1	10675	Unique	Used	0.624	1.228E5	8.403E4	248	3.20	0.00	0	45	19	2	726.87561	1452.74394	-13.65	45.97	5368	5368	MS2	19/20	Glioma_Nuclear_prep_Gradeall_4plex.raw
388240801	High		sVFEEEVR	Unambiguous	1	1	N-Term(iTRAQ4plex)	HCD	1.0000	0.0000	1	1	8434	Unique	Used	0.653	1.946E5	1.395E5	151	2.99	0.00	0	27	8	2	569.78760	1138.56792	-15.98	29.43	2923	2923	MS2	14/14	Glioma_Nuclear_prep_Gradeall_4plex.raw
388240801	High		gLESDVAELR	Unambiguous	1	1	N-Term(iTRAQ4plex)	HCD	1.0000	0.0000	1	1	9314	Unique	Used	0.751	1.404E5	1.157E5	172	3.26	0.00	0	11	8	2	616.82391	1232.64055	-16.07	35.88	3884	3884	MS2	17/18	Glioma_Nuclear_prep_Gradeall_4plex.raw
388240801	High		aLYESELADAR	Unambiguous	1	1	N-Term(iTRAQ4plex)	HCD	1.0000	0.0000	1	1	9075	Unique	Used	0.751	1.130E5	9.320E4	172	3.65	0.00	0	30	19	2	691.34723	1381.68718	-15.09	34.10	3622	3622	MS2	19/20	Glioma_Nuclear_prep_Gradeall_4plex.raw
388240801	High		dLESLFHR	Unambiguous	1	1	N-Term(iTRAQ4plex)	HCD	1.0000	0.0000	1	1	9764	Unique	Used	0.777	3.878E4	3.308E4	183	3.72	0.00	0	28	13	3	387.53751	1160.59797	-17.33	39.14	4373	4373	MS2	15/28	Glioma_Nuclear_prep_Gradeall_4plex.raw
388240801		High	cQSLQEELDFR	Unambiguous	1	1	N-Term(iTRAQ4plex); C1(Methylthio)	HCD	1.0000	0.0000	1	1	11556	Unique	Used	0.476	4.495E3	2.331E3	98	4.23	0.00	0	25	300	2	779.36395	1557.72063	3.08	64.93	6196	6196	MS2	18/20	Glioma_Nuclear_prep_Gradeall_repeat_4plex.raw
388240801		High	cQSLQEELDFR	Unambiguous	1	1	N-Term(iTRAQ4plex); C1(Methylthio)	HCD	1.0000	0.0000	1	1	11568	Unique	Used	0.522	7.572E3	4.308E3	98	4.70	0.00	0	17	300	2	779.36316	1557.71904	2.06	65.13	6212	6212	MS2	19/20	Glioma_Nuclear_prep_Gradeall_repeat_4plex.raw
388240801	High		cQSLQEELDFR	Unambiguous	1	1	N-Term(iTRAQ4plex); C1(Methylthio)	HCD	1.0000	0.0000	1	1	10749	Unique	Used	0.600	7.071E4	4.657E4	54	4.34	0.00	0	43	35	2	779.35150	1557.69573	-12.91	46.52	5448	5448	MS2	19/20	Glioma_Nuclear_prep_Gradeall_4plex.raw
388240801	High		cQSLQEELDFR	Unambiguous	1	1	N-Term(iTRAQ4plex); C1(Methylthio)	HCD	1.0000	0.0000	1	1	10838	Unique	Used	0.667	9.191E4	6.726E4	54	3.44	0.00	0	53	30	2	779.35126	1557.69524	-13.22	47.21	5545	5545	MS2	18/20	Glioma_Nuclear_prep_Gradeall_4plex.raw
388240801		High	lELEQTYQAk	Unambiguous	1	1	N-Term(iTRAQ4plex); K10(iTRAQ4plex)	HCD	1.0000	0.0000	1	1	9379	Unique	Used	0.673	4.592E4	3.368E4	265	4.25	0.00	0	8	71	2	755.92084	1510.83440	-0.77	37.93	3783	3783	MS2	18/18	Glioma_Nuclear_prep_Gradeall_repeat_4plex.raw
388240801	High		lELEQTYQAk	Unambiguous	1	1	N-Term(iTRAQ4plex); K10(iTRAQ4plex)	HCD	1.0000	0.0000	1	1	8893	Unique	Used	0.622	5.925E5	4.044E5	242	4.42	0.00	0	10	4	2	755.90991	1510.81255	-15.23	32.76	3423	3423	MS2	18/18	Glioma_Nuclear_prep_Gradeall_4plex.raw
388240801	High		qVLEGEEIAYk	Unambiguous	1	1	N-Term(iTRAQ4plex); K11(iTRAQ4plex)	HCD	1.0000	0.0000	1	1	9074	Unique	Used	0.702	1.457E5	1.123E5	215	4.76	0.00	0	5	16	2	783.92224	1566.83721	-15.69	34.10	3621	3621	MS2	20/20	Glioma_Nuclear_prep_Gradeall_4plex.raw
388240801		High	sEVELAAALSDkR	Unambiguous	1	1	N-Term(iTRAQ4plex); K12(iTRAQ4plex)	HCD	1.0000	0.0000	1	1	9913	Unique	Used	0.478	2.593E3	1.350E3	161	5.21	0.00	1	36	300	3	559.65283	1676.94394	1.05	43.96	4356	4356	MS2	29/48	Glioma_Nuclear_prep_Gradeall_repeat_4plex.raw
388240801		High	sEVELAAALSDk	Unambiguous	1	1	N-Term(iTRAQ4plex); K12(iTRAQ4plex)	HCD	1.0000	0.0000	1	1	10499	Unique	Used	0.749	5.164E3	4.216E3	263	5.19	0.00	0	22	300	2	760.92517	1520.84307	1.31	51.02	4987	4987	MS2	21/22	Glioma_Nuclear_prep_Gradeall_repeat_4plex.raw
388240801	High		sEVELAAALSDk	Unambiguous	1	1	N-Term(iTRAQ4plex); K12(iTRAQ4plex)	HCD	1.0000	0.0000	1	1	9805	Unique	Used	0.615	5.620E5	3.792E5	255	4.32	0.00	0	9	3	2	760.91394	1520.82060	-13.46	39.44	4418	4418	MS2	21/22	Glioma_Nuclear_prep_Gradeall_4plex.raw
388240801		High	lESLSYQLSGLQk	Unambiguous	1	1	N-Term(iTRAQ4plex); K13(iTRAQ4plex)	HCD	1.0000	0.0000	1	1	11417	Unique	Used	0.474	4.226E3	2.181E3	194	5.63	0.00	0	12	300	2	877.50244	1753.99761	2.14	63.04	6033	6033	MS2	24/24	Glioma_Nuclear_prep_Gradeall_repeat_4plex.raw
388240801	High		lESLSYQLSGLQk	Unambiguous	1	1	N-Term(iTRAQ4plex); K13(iTRAQ4plex)	HCD	1.0000	0.0000	1	1	10583	Unique	Used	0.482	1.148E5	6.069E4	217	5.49	0.00	0	27	24	2	877.48853	1753.96977	-13.73	45.26	5267	5267	MS2	23/24	Glioma_Nuclear_prep_Gradeall_4plex.raw
388240801	High		lESLSYQLSGLQk	Unambiguous	1	1	N-Term(iTRAQ4plex); K13(iTRAQ4plex)	HCD	1.0000	0.0000	1	1	10593	Unique	Used	0.531	1.985E4	1.158E4	226	4.39	0.00	0	13	5	3	585.32813	1753.96982	-13.70	45.34	5278	5278	MS2	24/48	Glioma_Nuclear_prep_Gradeall_4plex.raw
388240801	High		dVMQQQLAEYQELLDVk	Unambiguous	1	1	N-Term(iTRAQ4plex); K17(iTRAQ4plex)	HCD	1.0000	0.0000	1	1	12448	Unique	Used	0.400	7.715E3	3.383E3	170	7.82	0.00	0	5	20	3	780.07959	2338.22422	1.65	61.71	7306	7306	MS2	31/64	Glioma_Nuclear_prep_Gradeall_4plex.raw
388240801	High		dVMQQQLAEYQELLDVk	Unambiguous	1	1	N-Term(iTRAQ4plex); K17(iTRAQ4plex)	HCD	1.0000	0.0000	1	1	12419	Unique	Used	0.553	2.632E4	1.597E4	193	7.04	0.00	0	1	40	2	1169.61194	2338.21660	-1.60	61.36	7273	7273	MS2	25/32	Glioma_Nuclear_prep_Gradeall_4plex.raw
388240801	High		vkDLESLFHR	Unambiguous	1	1	N-Term(iTRAQ4plex); K2(iTRAQ4plex)	HCD	1.0000	0.0000	1	1	9714	Unique	Used	0.560	4.567E4	2.807E4	196	3.95	0.00	1	41	7	3	511.29208	1531.86170	-14.25	38.79	4319	4319	MS2	18/36	Glioma_Nuclear_prep_Gradeall_4plex.raw
388240801	High		iSEkEEVTTR	Unambiguous	1	1	N-Term(iTRAQ4plex); K4(iTRAQ4plex)	HCD	1.0000	0.0000	1	1	7423	Unique	Used	0.426	3.581E5	1.673E5	137	3.53	0.00	1	6	3	2	740.41370	1479.82012	-3.80	22.27	1828	1828	MS2	15/18	Glioma_Nuclear_prep_Gradeall_4plex.raw
388240801		High	qQEYDFk	Unambiguous	1	1	N-Term(iTRAQ4plex); K7(iTRAQ4plex)	HCD	1.0000	0.0000	1	1	8110	Unique	Used	0.595	8.473E4	5.498E4	106	3.30	0.00	0	19	55	2	623.31659	1245.62590	-7.61	24.86	2442	2442	MS2	12/12	Glioma_Nuclear_prep_Gradeall_repeat_4plex.raw
388240801		High	qQEYDFk	Unambiguous	1	1	N-Term(iTRAQ4plex); K7(iTRAQ4plex)	HCD	1.0000	0.0000	1	1	8069	Unique	Used	0.693	2.457E4	1.857E4	191	3.05	0.00	0	36	300	2	623.32330	1245.63933	3.17	24.44	2398	2398	MS2	12/12	Glioma_Nuclear_prep_Gradeall_repeat_4plex.raw
388240801		High	aELDEVNk	Unambiguous	1	1	N-Term(iTRAQ4plex); K8(iTRAQ4plex)	HCD	1.0000	0.0000	1	1	7829	Unique	Used	0.636	4.671E4	3.238E4	247	3.63	0.00	0	32	104	2	603.33191	1205.65654	-4.22	22.14	2146	2146	MS2	14/14	Glioma_Nuclear_prep_Gradeall_repeat_4plex.raw
388240801	High		aELDEVNk	Unambiguous	1	1	N-Term(iTRAQ4plex); K8(iTRAQ4plex)	HCD	1.0000	0.0000	1	1	7713	Unique	Used	0.611	7.820E5	5.240E5	251	3.56	0.00	0	17	4	2	603.33405	1205.66081	-0.67	24.34	2142	2142	MS2	14/14	Glioma_Nuclear_prep_Gradeall_4plex.raw
388240801		High	tVLVNADGEEVAmR	Unambiguous	1	1	N-Term(iTRAQ4plex); M13(Oxidation)	HCD	1.0000	0.0000	1	1	8785	Unique	Used	0.588	7.156E3	4.587E3	100	3.50	0.00	0	4	182	2	832.41486	1663.82244	-13.10	31.45	3153	3153	MS2	17/26	Glioma_Nuclear_prep_Gradeall_repeat_4plex.raw
388240801	High		tVLVNADGEEVAmR	Unambiguous	1	1	N-Term(iTRAQ4plex); M13(Oxidation)	HCD	1.0000	0.0000	1	1	8485	Unique	Used	0.384	1.542E5	6.499E4	132	3.98	0.00	0	25	12	2	832.41321	1663.81914	-15.09	29.79	2978	2978	MS2	18/26	Glioma_Nuclear_prep_Gradeall_4plex.raw
388240801	High		dVmQQQLAEYQELLDVk	Unambiguous	1	1	N-Term(iTRAQ4plex); M3(Oxidation); K17(iTRAQ4plex)	HCD	1.0000	0.0000	1	1	11376	Unique	Used	0.541	8.016E3	4.758E3	159	7.67	0.00	0	11	29	3	785.41107	2354.21866	1.44	51.54	6132	6132	MS2	25/64	Glioma_Nuclear_prep_Gradeall_4plex.raw
388240801		High	lALDmEINAYR	Unambiguous	1	1	N-Term(iTRAQ4plex); M5(Oxidation)	HCD	1.0000	0.0000	1	1	10701	Unique	Used	0.541	9.519E3	5.615E3	208	3.42	0.00	0	11	300	2	734.88464	1468.76201	2.26	53.44	5205	5205	MS2	19/20	Glioma_Nuclear_prep_Gradeall_repeat_4plex.raw
388240801	High		lALDmEINAYR	Unambiguous	1	1	N-Term(iTRAQ4plex); M5(Oxidation)	HCD	1.0000	0.0000	1	1	10003	Unique	Used	0.606	2.023E5	1.346E5	189	3.27	0.00	0	20	10	2	734.87195	1468.73662	-15.03	40.88	4633	4633	MS2	17/20	Glioma_Nuclear_prep_Gradeall_4plex.raw
388240801		High	eLEEAmAGER	Unambiguous	1	1	N-Term(iTRAQ4plex); M6(Oxidation)	HCD	1.0000	0.0000	1	1	7451	Unique	Used	0.580	7.083E4	4.477E4	68	3.11	0.00	0	7	40	2	647.80682	1294.60637	-0.19	18.69	1744	1744	MS2	15/18	Glioma_Nuclear_prep_Gradeall_repeat_4plex.raw
388240801	High		eLEEAmAGER	Unambiguous	1	1	N-Term(iTRAQ4plex); M6(Oxidation)	HCD	1.0000	0.0000	1	1	7451	Unique	Used	0.493	2.087E5	1.129E5	70	2.90	0.00	0	22	15	2	647.80396	1294.60063	-4.62	22.47	1858	1858	MS2	14/18	Glioma_Nuclear_prep_Gradeall_4plex.raw
390125204	NP_001254527.1	MGST1	4257	microsomal glutathione S-transferase 1 isoform c [Homo sapiens]	16.09	3	1	2	1.702	1;1	27.0	10.87	87	10.0	9.14																			
390125204		High	vFANPEDcVAFGk	Unambiguous	3	1	N-Term(iTRAQ4plex); C8(Methylthio); K13(iTRAQ4plex)	HCD	1.0000	0.0000	1	1	11901	Unique	Used	2.027	6.542E2	1.445E3	152	4.48	0.00	0	18	300	2	865.92938	1730.85149	1.73	70.23	6637	6637	MS2	19/24	Glioma_Nuclear_prep_Gradeall_repeat_4plex.raw
390125204	High		vFANPEDcVAFGk	Unambiguous	3	1	N-Term(iTRAQ4plex); C8(Methylthio); K13(iTRAQ4plex)	HCD	1.0000	0.0000	1	1	11118	Unique	Used	1.377	1.803E4	2.724E4	157	5.34	0.00	0	39	74	2	865.92578	1730.84429	-2.43	49.36	5849	5849	MS2	19/24	Glioma_Nuclear_prep_Gradeall_4plex.raw
392513662	NP_001254738.1	ILF2	3608	interleukin enhancer-binding factor 2 isoform 2 [Homo sapiens]	26.14	2	5	11	0.505	4;7	30.9	49.89	352	38.9	4.94																			
392513662		High	vLQSALAAIR	Unambiguous	2	1	N-Term(iTRAQ4plex)	HCD	1.0000	0.0000	1	1	11601	Unique	Used	0.471	1.796E4	9.219E3	133	3.29	0.00	0	10	239	2	593.37604	1185.74480	0.99	65.59	6251	6251	MS2	17/18	Glioma_Nuclear_prep_Gradeall_repeat_4plex.raw
392513662		High	iLITTVPPNLR	Unambiguous	2	1	N-Term(iTRAQ4plex)	HCD	1.0000	0.0000	1	1	11106	Unique	Used	0.530	8.953E3	5.170E3	99	3.21	0.00	0	50	300	2	690.93884	1380.87041	0.62	58.52	5651	5651	MS2	12/20	Glioma_Nuclear_prep_Gradeall_repeat_4plex.raw
392513662	High		vLQSALAAIR	Unambiguous	2	1	N-Term(iTRAQ4plex)	HCD	1.0000	0.0000	1	1	10687	Unique	Used	0.747	5.090E5	4.170E5	155	3.30	0.00	0	36	8	2	593.36700	1185.72673	-14.25	46.06	5381	5381	MS2	17/18	Glioma_Nuclear_prep_Gradeall_4plex.raw
392513662	High		kLDPELHLDIk	Unambiguous	2	1	N-Term(iTRAQ4plex); K1(iTRAQ4plex); K11(iTRAQ4plex)	HCD	1.0000	0.0000	1	1	9098	Unique	Used	0.711	8.099E4	6.314E4	119	3.32	0.00	1	40	25	2	877.02209	1753.03691	-12.27	34.27	3647	3647	MS2	18/20	Glioma_Nuclear_prep_Gradeall_4plex.raw
392513662		High	vkPAPDETSFSEALLk	Unambiguous	2	1	N-Term(iTRAQ4plex); K2(iTRAQ4plex); K16(iTRAQ4plex)	HCD	1.0000	0.0000	1	1	10557	Unique	Used	0.287	1.237E4	3.867E3	98	4.98	0.00	0	3	161	2	1082.61719	2164.22710	2.07	51.72	5050	5050	MS2	22/30	Glioma_Nuclear_prep_Gradeall_repeat_4plex.raw
392513662		High	vkPAPDETSFSEALLk	Unambiguous	2	1	N-Term(iTRAQ4plex); K2(iTRAQ4plex); K16(iTRAQ4plex)	HCD	1.0000	0.0000	1	1	10595	Unique	Used	0.375	3.811E2	1.558E2	109	5.26	0.00	0	21	245	4	541.81506	2164.23842	7.30	52.17	5091	5091	MS2	23/90	Glioma_Nuclear_prep_Gradeall_repeat_4plex.raw
392513662		High	vkPAPDETSFSEALLk	Unambiguous	2	1	N-Term(iTRAQ4plex); K2(iTRAQ4plex); K16(iTRAQ4plex)	HCD	1.0000	0.0000	1	1	10554	Unique	Used	0.393	1.096E4	4.699E3	103	7.53	0.00	0	6	18	3	722.08154	2164.23008	3.44	51.70	5047	5047	MS2	28/60	Glioma_Nuclear_prep_Gradeall_repeat_4plex.raw
392513662	High		vkPAPDETSFSEALLk	Unambiguous	2	1	N-Term(iTRAQ4plex); K2(iTRAQ4plex); K16(iTRAQ4plex)	HCD	1.0000	0.0000	1	1	9785	Unique	Used	0.723	2.672E4	2.121E4	153	7.04	0.00	0	15	6	3	722.06934	2164.19345	-13.48	39.29	4396	4396	MS2	29/60	Glioma_Nuclear_prep_Gradeall_4plex.raw
392513662		High	nQDLAPNSAEQASILSLVTk	Unambiguous	2	1	N-Term(iTRAQ4plex); K20(iTRAQ4plex)	HCD	1.0000	0.0000	1	1	12185	Unique	Used	0.505	2.613E2	1.437E2	137	6.18	0.00	0	7	300	3	796.44135	2387.30948	3.09	75.79	7074	7074	MS2	30/76	Glioma_Nuclear_prep_Gradeall_repeat_4plex.raw
392513662		High	nQDLAPNSAEQASILSLVTk	Unambiguous	2	1	N-Term(iTRAQ4plex); K20(iTRAQ4plex)	HCD	1.0000	0.0000	1	1	12125	Unique	Used	0.709	1.547E2	1.195E2	135	4.80	0.00	0	19	300	3	796.44043	2387.30674	1.94	74.73	6989	6989	MS2	28/76	Glioma_Nuclear_prep_Gradeall_repeat_4plex.raw
392513662	High		nQDLAPNSAEQASILSLVTk	Unambiguous	2	1	N-Term(iTRAQ4plex); K20(iTRAQ4plex)	HCD	1.0000	0.0000	1	1	11550	Unique	Used	0.407	1.935E4	8.635E3	108	6.34	0.00	0	12	60	2	1194.15479	2387.30229	0.08	53.01	6322	6322	MS2	31/38	Glioma_Nuclear_prep_Gradeall_4plex.raw
402534522	NP_001257973.1	NFIX	4784	nuclear factor 1 X-type isoform 3 [Homo sapiens]	4.85	8	1	2	2.655	1;1	16.7	7.29	433	47.9	8.53																			
402534522		High	eDFVLTITGk	Unambiguous	3	1	N-Term(iTRAQ4plex); K10(iTRAQ4plex)	HCD	1.0000	0.0000	1	1	11337	Unique	Used	2.968	1.592E3	5.148E3	300	4.00	0.00	0	39	300	2	705.90833	1410.80937	0.76	61.94	5938	5938	MS2	16/18	Glioma_Nuclear_prep_Gradeall_repeat_4plex.raw
402534522	High		eDFVLTITGk	Unambiguous	3	1	N-Term(iTRAQ4plex); K10(iTRAQ4plex)	HCD	1.0000	0.0000	1	1	10494	Unique	Used	2.342	7.093E4	1.823E5	281	4.01	0.00	0	33	21	2	705.89703	1410.78679	-15.24	44.60	5170	5170	MS2	15/18	Glioma_Nuclear_prep_Gradeall_4plex.raw
404501455	NP_001258218.1	ZNF512	84450	zinc finger protein 512 isoform d [Homo sapiens]	18.61	4	5	6	1.634	2;4	10.7	22.27	462	53.1	9.60																			
404501455		High	vLQDLVPDDR	Unambiguous	4	1	N-Term(iTRAQ4plex)	HCD	1.0000	0.0000	1	1	10548	Unique	Used	1.425	5.955E3	9.251E3	311	3.85	0.00	0	48	300	2	657.36371	1313.72014	1.47	51.61	5040	5040	MS2	15/18	Glioma_Nuclear_prep_Gradeall_repeat_4plex.raw
404501455		High	aGDEIDEPSER	Unambiguous	4	1	N-Term(iTRAQ4plex)	HCD	1.0000	0.0000	1	1	7399	Unique	Used	1.721	8.177E3	1.534E4	43	3.36	0.00	0	29	103	2	681.30762	1361.60796	-16.32	18.22	1689	1689	MS2	15/20	Glioma_Nuclear_prep_Gradeall_repeat_4plex.raw
404501455		High	qQPGIELPETELSLR	Unambiguous	4	1	N-Term(iTRAQ4plex)	HCD	1.0000	0.0000	1	1	11400	Unique	Used	1.836	1.434E3	2.869E3	185	4.33	0.00	0	11	300	2	927.50989	1854.01250	1.90	62.79	6012	6012	MS2	18/28	Glioma_Nuclear_prep_Gradeall_repeat_4plex.raw
404501455	High		aGDEIDEPSER	Unambiguous	4	1	N-Term(iTRAQ4plex)	HCD	1.0000	0.0000	1	1	7431	Unique	Used	1.432	3.694E4	5.806E4	60	3.79	0.00	0	41	31	2	681.31476	1361.62224	-5.83	22.32	1836	1836	MS2	14/20	Glioma_Nuclear_prep_Gradeall_4plex.raw
404501455		High	sEHGPISFFPESGQPEcLk	Unambiguous	4	1	N-Term(iTRAQ4plex); C17(Methylthio); K19(iTRAQ4plex)	HCD	1.0000	0.0000	1	1	11713	Unique	Used	1.745	2.509E2	4.772E2	107	7.08	0.00	0	17	300	3	808.39331	2423.16538	1.62	67.15	6384	6384	MS2	29/72	Glioma_Nuclear_prep_Gradeall_repeat_4plex.raw
404501455	High		ePEQEPVPAQFQk	Unambiguous	4	1	N-Term(iTRAQ4plex); K13(iTRAQ4plex)	HCD	1.0000	0.0000	1	1	8701	Unique	Used	1.547	3.272E4	5.554E4	175	4.09	0.00	0	17	31	2	907.96735	1814.92742	-13.96	31.37	3214	3214	MS2	17/24	Glioma_Nuclear_prep_Gradeall_4plex.raw

440309857	NP_001258978.1	TRAP1	10131	heat shock protein 75 kDa, mitochondrial isoform 2 [Homo sapiens]	2.15	2	1	2	1.791	1;1	0.6	4.70	651	74.2	7.87																			
440309857		High	gVVDSEDIPLNLSR	Unambiguous	2	1	N-Term(iTRAQ4plex)	HCD	1.0000	0.0000	1	1	10809	Unique	Used	1.799	1.558E3	3.055E3	259	4.70	0.00	0	28	300	2	829.44891	1657.89055	1.66	54.79	5323	5323	MS2	23/26	Glioma_Nuclear_prep_Gradeall_repeat_4plex.raw
440309857	High		gVVDSEDIPLNLSR	Unambiguous	2	1	N-Term(iTRAQ4plex)	HCD	1.0000	0.0000	1	1	10128	Unique	Used	1.783	5.360E4	1.049E5	228	4.66	0.00	0	8	20	2	829.43384	1657.86040	-16.53	41.80	4769	4769	MS2	23/26	Glioma_Nuclear_prep_Gradeall_4plex.raw
544346329	NP_001269688.1	TRA2A	29896	transformer-2 protein homolog alpha isoform 3 [Homo sapiens]	12.78	3	2	4	0.536	2;2	23.5	8.63	180	20.6	10.17																			
544346329		High	yGPLSGVNVVYDQR	Unambiguous	3	1	N-Term(iTRAQ4plex)	HCD	1.0000	0.0000	1	1	10977	Unique	Used	0.435	3.785E3	1.795E3	305	5.04	0.00	0	12	300	2	855.95227	1710.89726	2.38	56.86	5506	5506	MS2	23/26	Glioma_Nuclear_prep_Gradeall_repeat_4plex.raw
544346329		High	gFAFVYFER	Unambiguous	3	1	N-Term(iTRAQ4plex)	HCD	1.0000	0.0000	1	1	12109	Unique	Used	0.545	2.946E3	1.751E3	252	3.16	0.00	0	24	300	2	640.33337	1279.65947	0.20	74.36	6960	6960	MS2	14/16	Glioma_Nuclear_prep_Gradeall_repeat_4plex.raw
544346329	High		yGPLSGVNVVYDQR	Unambiguous	3	1	N-Term(iTRAQ4plex)	HCD	1.0000	0.0000	1	1	10217	Unique	Used	0.528	9.095E4	5.266E4	214	4.86	0.00	0	28	31	2	855.93823	1710.86919	-14.03	42.47	4866	4866	MS2	22/26	Glioma_Nuclear_prep_Gradeall_4plex.raw
544346329	High		gFAFVYFER	Unambiguous	3	1	N-Term(iTRAQ4plex)	HCD	1.0000	0.0000	1	1	11379	Unique	Used	0.750	3.296E4	2.711E4	253	3.77	0.00	0	44	76	2	640.33313	1279.65898	-0.18	51.56	6135	6135	MS2	15/16	Glioma_Nuclear_prep_Gradeall_4plex.raw
584277111	NP_001276868.1	SLC1A3	6507	excitatory amino acid transporter 1 isoform 4 [Homo sapiens]	5.04	6	2	5	5.262	4;1	35.4	22.01	496	54.2	7.44																			
584277111		High	vTAADAFLDLIR	Unambiguous	4	1	N-Term(iTRAQ4plex)	HCD	1.0000	0.0000	1	1	12473	Unique	Used	6.070	3.394E2	2.245E3	232	4.15	0.00	0	13	300	2	724.91437	1448.82146	-1.07	92.52	8228	8228	MS2	19/22	Glioma_Nuclear_prep_Gradeall_repeat_4plex.raw
584277111	High		vTAADAFLDLIR	Unambiguous	4	1	N-Term(iTRAQ4plex)	HCD	1.0000	0.0000	1	1	12674	Unique	Used	3.106	3.554E3	1.211E4	332	4.55	0.00	0	67	300	2	724.91498	1448.82268	-0.23	64.67	7568	7568	MS2	20/22	Glioma_Nuclear_prep_Gradeall_4plex.raw
584277111	High		vTAADAFLDLIR	Unambiguous	4	1	N-Term(iTRAQ4plex)	HCD	1.0000	0.0000	1	1	12777	Unique	Used	5.262	4.526E3	2.613E4	239	5.11	0.00	0	31	300	2	724.91602	1448.82475	1.20	66.03	7687	7687	MS2	21/22	Glioma_Nuclear_prep_Gradeall_4plex.raw
584277111	High		vTAADAFLDLIR	Unambiguous	4	1	N-Term(iTRAQ4plex)	HCD	1.0000	0.0000	1	1	12729	Unique	Used	7.067	1.416E4	1.098E5	229	4.91	0.00	0	9	82	2	724.91608	1448.82488	1.29	65.37	7630	7630	MS2	21/22	Glioma_Nuclear_prep_Gradeall_4plex.raw
584277111	High		nMFPPNLVEAcFk	Unambiguous	5	1	N-Term(iTRAQ4plex); C11(Methylthio); K13(iTRAQ4plex)	HCD	1.0000	0.0000	1	1	12920	Unique	Used	3.206	1.912E3	6.725E3	170	3.79	0.00	0	59	300	2	922.46204	1843.91680	1.08	68.32	7872	7872	MS2	14/24	Glioma_Nuclear_prep_Gradeall_4plex.raw
601984520	NP_066289.3	UBC	7316	polyubiquitin-C [Homo sapiens]	21.02	4	1	2	2.545	1;1	21.6	5.36	685	77.0	7.66																			
601984520		High	tITLEVEPSDTIENVk	Unambiguous	4	1	N-Term(iTRAQ4plex); K16(iTRAQ4plex)	HCD	1.0000	0.0000	1	1	11045	Unique	Used	2.933	4.716E2	1.508E3	204	5.36	0.00	0	11	300	2	1038.56897	2076.13066	-0.41	57.74	5583	5583	MS2	21/30	Glioma_Nuclear_prep_Gradeall_repeat_4plex.raw
601984520	High		tITLEVEPSDTIENVk	Unambiguous	4	1	N-Term(iTRAQ4plex); K16(iTRAQ4plex)	HCD	1.0000	0.0000	1	1	10273	Unique	Used	2.158	3.241E4	7.673E4	216	5.33	0.00	0	9	17	2	1038.55212	2076.09697	-16.64	42.88	4927	4927	MS2	19/30	Glioma_Nuclear_prep_Gradeall_4plex.raw
664806003	NP_001287744.1	CIRBP	1153	cold-inducible RNA-binding protein isoform 2 [Homo sapiens]	14.88	3	2	4	0.381	3;1	38.0	14.01	168	18.1	9.64																			
664806003	High		yGQISEVVVVk	Unambiguous	3	1	N-Term(iTRAQ4plex); K11(iTRAQ4plex)	HCD	1.0000	0.0000	1	1	9702	Unique	Used	0.456	1.640E5	8.215E4	240	3.96	0.00	0	52	22	2	754.94128	1508.87529	-11.53	38.70	4306	4306	MS2	19/20	Glioma_Nuclear_prep_Gradeall_4plex.raw
664806003		High	gFGFVTFENIDDAk	Unambiguous	3	1	N-Term(iTRAQ4plex); K14(iTRAQ4plex)	HCD	1.0000	0.0000	1	1	12168	Unique	Used	0.306	2.554E3	8.532E2	184	5.21	0.00	0	35	300	2	924.47766	1847.94805	3.35	75.42	7045	7045	MS2	23/26	Glioma_Nuclear_prep_Gradeall_repeat_4plex.raw
664806003	High		gFGFVTFENIDDAk	Unambiguous	3	1	N-Term(iTRAQ4plex); K14(iTRAQ4plex)	HCD	1.0000	0.0000	1	1	11512	Unique	Used	0.282	1.036E5	3.211E4	166	6.40	0.00	0	38	63	2	924.47278	1847.93828	-1.94	52.70	6281	6281	MS2	25/26	Glioma_Nuclear_prep_Gradeall_4plex.raw
664806003	High		gFGFVTFENIDDAk	Unambiguous	3	1	N-Term(iTRAQ4plex); K14(iTRAQ4plex)	HCD	1.0000	0.0000	1	1	11511	Unique	Used	0.626	7.884E3	5.416E3	175	3.65	0.00	0	38	40	3	616.65179	1847.94083	-0.56	52.69	6280	6280	MS2	20/52	Glioma_Nuclear_prep_Gradeall_4plex.raw
665821291	NP_001287919.1	PTBP2	58155	polypyrimidine tract-binding protein 2 isoform 7 [Homo sapiens]	1.67	7	1	2	1.633	1;1	2.5	3.35	480	52.3	8.97																			
665821291		High	vTNILMLk	Unambiguous	7	1	N-Term(iTRAQ4plex); K8(iTRAQ4plex)	HCD	1.0000	0.0000	1	1	11335	Unique	Used	1.604	3.795E3	6.633E3	160	3.35	0.00	0	49	300	2	610.38867	1219.77007	1.14	61.91	5936	5936	MS2	14/14	Glioma_Nuclear_prep_Gradeall_repeat_4plex.raw
665821291	High		vTNILMLk	Unambiguous	7	1	N-Term(iTRAQ4plex); K8(iTRAQ4plex)	HCD	1.0000	0.0000	1	1	10434	Unique	Used	1.661	3.029E5	5.523E5	241	3.16	0.00	0	33	4	2	610.37848	1219.74968	-15.57	44.13	5104	5104	MS2	14/14	Glioma_Nuclear_prep_Gradeall_4plex.raw
667743700	NP_001288139.1	MYEF2	50804	myelin expression factor 2 isoform b [Homo sapiens]	15.10	2	7	13	0.345	5;8	32.6	31.17	576	61.9	8.75																			
667743700		High	aEVPGATGGDSPHLQPAEPPGEPR	Unambiguous	2	1	N-Term(iTRAQ4plex)	HCD	1.0000	0.0000	1	1	9006	Unique	Used	0.187	2.173E3	4.418E2	69	5.23	0.00	0	10	161	3	837.42126	2510.24924	3.95	33.79	3389	3389	MS2	26/92	Glioma_Nuclear_prep_Gradeall_repeat_4plex.raw
667743700		High	mGPGIGAILER	Unambiguous	2	1	N-Term(iTRAQ4plex)	HCD	1.0000	0.0000	1	1	11068	Unique	Used	0.221	1.640E4	3.945E3	262	3.24	0.00	0	28	300	2	629.36108	1257.71489	3.38	58.04	5609	5609	MS2	19/20	Glioma_Nuclear_prep_Gradeall_repeat_4plex.raw
667743700	High		aEVPGATGGDSPHLQPAEPPGEPR	Unambiguous	2	1	N-Term(iTRAQ4plex)	HCD	1.0000	0.0000	1	1	8613	Unique	Used	0.255	1.440E4	4.036E3	56	3.98	0.00	0	0	18	3	837.40521	2510.20108	-15.23	30.74	3119	3119	MS2	26/92	Glioma_Nuclear_prep_Gradeall_4plex.raw
667743700	High		mGPGIGAILER	Unambiguous	2	1	N-Term(iTRAQ4plex)	HCD	1.0000	0.0000	1	1	10201	Unique	Used	0.281	2.237E5	6.889E4	231	3.27	0.00	0	28	19	2	629.35065	1257.69402	-13.21	42.36	4849	4849	MS2	19/20	Glioma_Nuclear_prep_Gradeall_4plex.raw
667743700	High		gFLSGPMGSGMR	Unambiguous	2	1	N-Term(iTRAQ4plex)	HCD	1.0000	0.0000	1	1	9599	Unique	Used	0.345	3.068E5	1.160E5	118	3.99	0.00	0	31	14	2	670.82251	1340.63774	-14.53	37.96	4194	4194	MS2	15/22	Glioma_Nuclear_prep_Gradeall_4plex.raw
667743700		High	gcGVVEFk	Unambiguous	2	1	N-Term(iTRAQ4plex); C2(Methylthio); K8(iTRAQ4plex)	HCD	1.0000	0.0000	1	1	10083	Unique	Used	0.399	1.235E4	5.366E3	143	2.96	0.00	0	63	300	2	586.80756	1172.60784	2.72	46.10	4544	4544	MS2	14/14	Glioma_Nuclear_prep_Gradeall_repeat_4plex.raw
667743700		High	rEPHPAEAEk	Unambiguous	2	1	N-Term(iTRAQ4plex); K10(iTRAQ4plex)	HCD	1.0000	0.0000	1	1	6509	Unique	Used	0.350	3.502E3	1.335E3	262	4.07	0.00	1	55	162	4	363.70331	1451.79140	4.72	10.34	750	750	MS2	17/54	Glioma_Nuclear_prep_Gradeall_repeat_4plex.raw
667743700		High	vGEVTYVELFk	Unambiguous	2	1	N-Term(iTRAQ4plex); K11(iTRAQ4plex)	HCD	1.0000	0.0000	1	1	11894	Unique	Used	0.250	4.970E3	1.356E3	211	4.57	0.00	0	27	300	2	786.45331	1571.89934	4.44	70.12	6628	6628	MS2	18/20	Glioma_Nuclear_prep_Gradeall_repeat_4plex.raw
667743700		High	vGEVTYVELFk	Unambiguous	2	1	N-Term(iTRAQ4plex); K11(iTRAQ4plex)	HCD	1.0000	0.0000	1	1	11899	Unique	Used	0.486	2.662E2	1.410E2	212	3.47	0.00	0	18	300	3	524.63702	1571.89652	2.65	70.21	6635	6635	MS2	18/40	Glioma_Nuclear_prep_Gradeall_repeat_4plex.raw
667743700		High	vFISNIPYDMk	Unambiguous	2	1	N-Term(iTRAQ4plex); K11(iTRAQ4plex)	HCD	1.0000	0.0000	1	1	11513	Unique	Used	0.492	9.226E3	4.947E3	271	4.49	0.00	0	50	300	2	807.94672	1614.88616	3.55	64.31	6143	6143	MS2	17/20	Glioma_Nuclear_prep_Gradeall_repeat_4plex.raw
667743700	High		vGEVTYVELFk	Unambiguous	2	1	N-Term(iTRAQ4plex); K11(iTRAQ4plex)	HCD	1.0000	0.0000	1	1	11084	Unique	Used	0.217	3.818E5	9.096E4	207	5.31	0.00	0	28	13	2	786.44916	1571.89104	-0.84	49.12	5813	5813	MS2	19/20	Glioma_Nuclear_prep_Gradeall_4plex.raw
667743700	High		vFISNIPYDMk	Unambiguous	2	1	N-Term(iTRAQ4plex); K11(iTRAQ4plex)	HCD	1.0000	0.0000	1	1	10698	Unique	Used	0.434	3.627E5	1.729E5	257	4.34	0.00	0	53	12	2	807.93475	1614.86223	-11.26	46.14	5393	5393	MS2	16/20	Glioma_Nuclear_prep_Gradeall_4plex.raw
667743700		High	vFISNIPYDmk	Unambiguous	2	1	N-Term(iTRAQ4plex); M10(Oxidation); K11(iTRAQ4plex)	HCD	1.0000	0.0000	1	1	10726	Unique	Used	0.468	9.737E3	4.963E3	186	3.14	0.00	0	58	300	2	815.94543	1630.88359	5.06	53.75	5232	5232	MS2	13/20	Glioma_Nuclear_prep_Gradeall_repeat_4plex.raw
751368126	NP_001291434.1	HMG20A	10363	high mobility group protein 20A isoform b [Homo sapiens]	11.11	2	2	2	0.404	1;1	39.9	6.92	234	27.7	6.92																			
751368126		High	lEVDVIQER	Unambiguous	2	1	N-Term(iTRAQ4plex)	HCD	1.0000	0.0000	1	1	9869	Unique	Used	0.524	9.984E3	5.700E3	302	3.34	0.00	0	61	300	2	622.85181	1244.69634	-0.32	43.40	4307	4307	MS2	13/16	Glioma_Nuclear_prep_Gradeall_repeat_4plex.raw
751368126	High		sVFDIPIFTEEFLNHSk	Unambiguous	2	1	N-Term(iTRAQ4plex); K17(iTRAQ4plex)	HCD	1.0000	0.0000	1	1	13023	Unique	Used	0.284	6.054E2	1.890E2	139	6.92	0.00	0	34	300	3	771.08203	2311.23154	4.42	70.34	8027	8027	MS2	34/64	Glioma_Nuclear_prep_Gradeall_4plex.raw
808175976	NP_001294904.1	TMPO	7112	thymopoietin isoform delta [Homo sapiens]	38.65	3	1	3	1.501	2;1	8.3	62.90	414	46.3	9.03																			
808175976		High	hASPILPITEFSDIPR	Unambiguous	2	1	N-Term(iTRAQ4plex)	HCD	1.0000	0.0000	1	1	11909	Unique	Used	1.558	2.377E2	4.035E2	232	5.20	0.00	0	14	300	3	646.35944	1937.06375	1.24	70.34	6647	6647	MS2	27/60	Glioma_Nuclear_prep_Gradeall_repeat_4plex.raw
808175976	High		hASPILPITEFSDIPR	Unambiguous	2	1	N-Term(iTRAQ4plex)	HCD	1.0000	0.0000	1	1	10900	Unique	Used	1.325	1.001E4	1.455E4	213	4.68	0.00	0	41	22	3	646.34961	1937.03427	-13.98	47.68	5612	5612	MS2	23/60	Glioma_Nuclear_prep_Gradeall_4plex.raw
808175976	High		hASPILPITEFSDIPR	Unambiguous	2	1	N-Term(iTRAQ4plex)	HCD	1.0000	0.0000	1	1	10915	Unique	Used	1.501	1.914E4	3.152E4	301	4.26	0.00	0	24	71	2	969.01941	1937.03154	-15.39	47.79	5628	5628	MS2	17/30	Glioma_Nuclear_prep_Gradeall_4plex.raw

Supplementary Table S4: Differentially expressed proteins in Grade III astrocytoma. A total of 131 nuclear proteins (≥1.5 fold change) were identified after applying quality control filters as detailed in Methods section. The Table describes the total number of proteins and peptides identified in replicate mass spectrometry runs along with their quantity values.

Protein Accession	RefSeq id	Gene Symbol	Gene id	Protein name	ΣCoverage	Σ# Proteins	Σ# Unique Peptides	Σ# PSMs	116/114	116/114 Count	116/114 Variability [%]	Maximum Score	# AAs	MW [kDa]	calc. pI																			
Protein Group Accessions	A2	B2	Sequence	PSM Ambiguity	# Proteins	# Protein Groups	Modifications	Activation Type	ΔScore	ΔCn	Rank	Search Engine Rank	QuanResultID	Quan Info	Quan Usage	116/114	114	116	Peptides Matched	XCorr	Probability	# Missed Cleavages	Isolation Interference [%]	Ion Inject Time [ms]	Charge	m/z [Da]	MH+ [Da]	ΔM [ppm]	RT [min]	First Scan	Last Scan	MS Order	Ions Matched	Spectrum File
4502027	NP_000468.1	ALB	213	serum albumin preproprotein [Homo sapiens]	23.32	1	11	16	1.887	5;11	16.9	46.84	609	69.3	6.28																			
4502027		High	fQNALLVR	Unambiguous	1	1	N-Term(iTRAQ4plex)	HCD	1.0000	0.0000	1	1	10037	Unique	Used	1.978	4.349E3	1.041E4	360	3.27	0.00	0	37	300	2	552.83624	1104.66521	0.52	45.50	4492	4492	MS2	14/14	Glioma_Nuclear_prep_Gradeall_repeat_4plex.raw
4502027		High	tcVADESAENcDk	Unambiguous	1	1	N-Term(iTRAQ4plex); C2(Methylthio); C11(Methylthio); K13(iTRAQ4plex)	HCD	1.0000	0.0000	1	1	9004	Unique	Used	1.746	1.109E3	2.342E3	16	4.91	0.00	0	18	300	2	882.86334	1764.71941	2.41	33.75	3386	3386	MS2	23/24	Glioma_Nuclear_prep_Gradeall_repeat_4plex.raw
4502027		High	ylcENQDSISSk	Unambiguous	1	1	N-Term(iTRAQ4plex); C3(Methylthio); K12(iTRAQ4plex)	HCD	1.0000	0.0000	1	1	9609	Unique	Used	2.149	2.815E3	7.318E3	62	5.08	0.00	0	19	300	2	860.90955	1720.81182	-0.37	40.50	4033	4033	MS2	19/22	Glioma_Nuclear_prep_Gradeall_repeat_4plex.raw
4502027	High		ylcENQDSISSk	Unambiguous	1	1	N-Term(iTRAQ4plex); C3(Methylthio); K12(iTRAQ4plex)	HCD	1.0000	0.0000	1	1	9141	Unique	Used	2.046	5.508E4	1.375E5	35	4.24	0.00	0	3	10	2	860.89545	1720.78362	-16.76	34.59	3694	3694	MS2	20/22	Glioma_Nuclear_prep_Gradeall_4plex.raw
4502027		High	nEcFLQHk	Unambiguous	1	1	N-Term(iTRAQ4plex); C3(Methylthio); K8(iTRAQ4plex)	HCD	1.0000	0.0000	1	1	8637	Unique	Used	1.499	1.902E3	3.449E3	149	3.53	0.00	0	39	210	3	451.56073	1352.66764	-1.27	30.01	2997	2997	MS2	19/28	Glioma_Nuclear_prep_Gradeall_repeat_4plex.raw
4502027	High		eTYGEMADccAk	Unambiguous	1	1	N-Term(iTRAQ4plex); C9(Methylthio); C10(Methylthio); K12(iTRAQ4plex)	HCD	1.0000	0.0000	1	1	10220	Unique	Used	1.385	2.593E4	4.381E4	6	4.36	0.00	0	43	47	2	850.82495	1700.64263	-16.17	42.49	4869	4869	MS2	19/22	Glioma_Nuclear_prep_Gradeall_4plex.raw
4502027		High	lVNEVTEFAk	Unambiguous	1	1	N-Term(iTRAQ4plex); K10(iTRAQ4plex)	HCD	1.0000	0.0000	1	1	10914	Unique	Used	1.920	3.308E3	7.684E3	278	4.44	0.00	0	24	300	2	719.41309	1437.81890	-0.21	56.11	5439	5439	MS2	18/18	Glioma_Nuclear_prep_Gradeall_repeat_4plex.raw
4502027		High	lVNEVTEFAk	Unambiguous	1	1	N-Term(iTRAQ4plex); K10(iTRAQ4plex)	HCD	1.0000	0.0000	1	1	10924	Unique	Used	1.500	3.621E2	6.570E2	298	3.39	0.00	0	53	300	3	479.94406	1437.81763	-1.09	56.22	5449	5449	MS2	16/36	Glioma_Nuclear_prep_Gradeall_repeat_4plex.raw
4502027	High		lVNEVTEFAk	Unambiguous	1	1	N-Term(iTRAQ4plex); K10(iTRAQ4plex)	HCD	1.0000	0.0000	1	1	10132	Unique	Used	2.232	1.621E5	4.416E5	237	4.01	0.00	0	33	4	2	719.40125	1437.79521	-16.68	41.84	4774	4774	MS2	17/18	Glioma_Nuclear_prep_Gradeall_4plex.raw
4502027	High		aVMDDFAAFVEk	Unambiguous	1	1	N-Term(iTRAQ4plex); K12(iTRAQ4plex)	HCD	1.0000	0.0000	1	1	11832	Unique	Used	1.735	3.708E3	7.847E3	200	4.20	0.00	0	32	31	3	544.28186	1630.83103	-4.87	55.48	6629	6629	MS2	19/44	Glioma_Nuclear_prep_Gradeall_4plex.raw
4502027	High		vFDEFkPLVEEPQNLIk	Unambiguous	1	1	N-Term(iTRAQ4plex); K6(iTRAQ4plex); K17(iTRAQ4plex)	HCD	1.0000	0.0000	1	1	11833	Unique	Used	2.016	3.837E3	9.440E3	72	6.38	0.00	0	29	29	3	826.46887	2477.39206	-3.87	55.48	6630	6630	MS2	28/64	Glioma_Nuclear_prep_Gradeall_4plex.raw
4502027		High	aEFAEVSk	Unambiguous	1	1	N-Term(iTRAQ4plex); K8(iTRAQ4plex)	HCD	1.0000	0.0000	1	1	8298	Unique	Used	1.854	2.163E4	4.850E4	284	3.19	0.00	0	60	196	2	584.82880	1168.65032	4.34	26.68	2640	2640	MS2	14/14	Glioma_Nuclear_prep_Gradeall_repeat_4plex.raw
4502027		High	sLHTLFGDk	Unambiguous	1	1	N-Term(iTRAQ4plex); K9(iTRAQ4plex)	HCD	1.0000	0.0000	1	1	9326	Unique	Used	2.114	1.514E3	3.872E3	323	3.86	0.00	0	22	147	3	435.91788	1305.73908	-1.12	37.37	3727	3727	MS2	16/32	Glioma_Nuclear_prep_Gradeall_repeat_4plex.raw
4502027		High	qTALVELVk	Unambiguous	1	1	N-Term(iTRAQ4plex); K9(iTRAQ4plex)	HCD	1.0000	0.0000	1	1	10214	Unique	Used	1.490	8.136E3	1.467E4	177	3.86	0.00	0	39	286	2	644.90710	1288.80693	-0.75	47.63	4683	4683	MS2	15/16	Glioma_Nuclear_prep_Gradeall_repeat_4plex.raw
4502027		High	sLHTLFGDk	Unambiguous	1	1	N-Term(iTRAQ4plex); K9(iTRAQ4plex)	HCD	1.0000	0.0000	1	1	9348	Unique	Used	1.480	5.666E3	1.014E4	354	3.69	0.00	0	27	270	2	653.37341	1305.73955	-0.76	37.59	3750	3750	MS2	16/16	Glioma_Nuclear_prep_Gradeall_repeat_4plex.raw
4502027		High	aVmDDFAAFVEk	Unambiguous	1	1	N-Term(iTRAQ4plex); M3(Oxidation); K12(iTRAQ4plex)	HCD	1.0000	0.0000	1	1	11406	Unique	Used	2.418	1.763E3	5.157E3	172	4.54	0.00	0	49	300	2	823.92010	1646.83293	-0.58	62.89	6020	6020	MS2	19/22	Glioma_Nuclear_prep_Gradeall_repeat_4plex.raw
4502107	NP_001145.1	ANXA5	308	annexin A5 [Homo sapiens]	22.19	1	7	10	2.263	7;3	20.6	30.13	320	35.9	5.05																			
4502107		High	gTVTDFPGFDER	Unambiguous	1	1	N-Term(iTRAQ4plex)	HCD	1.0000	0.0000	1	1	10351	Unique	Used	2.305	3.187E3	8.888E3	135	4.34	0.00	0	25	241	2	742.86212	1484.71697	2.10	49.27	4830	4830	MS2	19/22	Glioma_Nuclear_prep_Gradeall_repeat_4plex.raw
4502107	High		gLGTDEESILTLLTSR	Unambiguous	1	1	N-Term(iTRAQ4plex)	HCD	1.0000	0.0000	1	1	12926	Unique	Used	2.682	1.040E3	3.403E3	153	6.32	0.00	0	22	300	2	925.00751	1849.00774	2.26	68.44	7881	7881	MS2	27/30	Glioma_Nuclear_prep_Gradeall_4plex.raw
4502107	High		gLGTDEESILTLLTSR	Unambiguous	1	1	N-Term(iTRAQ4plex)	HCD	1.0000	0.0000	1	1	12885	Unique	Used	2.499	1.605E3	4.895E3	136	6.42	0.00	0	6	300	2	925.00800	1849.00871	2.79	67.74	7826	7826	MS2	26/30	Glioma_Nuclear_prep_Gradeall_4plex.raw
4502107	High		sEIDLFNIR	Unambiguous	1	1	N-Term(iTRAQ4plex)	HCD	1.0000	0.0000	1	1	10747	Unique	Used	2.221	5.126E4	1.389E5	310	3.45	0.00	0	52	28	2	625.83740	1250.66753	-14.90	46.51	5446	5446	MS2	15/16	Glioma_Nuclear_prep_Gradeall_4plex.raw
4502107	High		gTVTDFPGFDER	Unambiguous	1	1	N-Term(iTRAQ4plex)	HCD	1.0000	0.0000	1	1	9737	Unique	Used	2.070	2.265E4	5.719E4	89	3.56	0.00	0	32	14	2	742.84894	1484.69060	-15.66	38.95	4344	4344	MS2	17/22	Glioma_Nuclear_prep_Gradeall_4plex.raw
4502107	High		nFATSLYSMIk	Unambiguous	1	1	N-Term(iTRAQ4plex); K11(iTRAQ4plex)	HCD	1.0000	0.0000	1	1	11722	Unique	Used	1.664	1.910E4	3.877E4	304	4.39	0.00	0	63	111	2	781.92511	1562.84294	-3.94	54.52	6509	6509	MS2	18/20	Glioma_Nuclear_prep_Gradeall_4plex.raw
4502107		High	dLLDDLk	Unambiguous	1	1	N-Term(iTRAQ4plex); K7(iTRAQ4plex)	HCD	1.0000	0.0000	1	1	10781	Unique	Used	2.799	2.903E3	9.831E3	330	3.08	0.00	0	39	300	2	560.32770	1119.64812	-1.68	54.43	5292	5292	MS2	12/12	Glioma_Nuclear_prep_Gradeall_repeat_4plex.raw
4502107		High	lYDAYELk	Unambiguous	1	1	N-Term(iTRAQ4plex); K8(iTRAQ4plex)	HCD	1.0000	0.0000	1	1	10177	Unique	Used	2.348	4.678E3	1.329E4	292	3.34	0.00	0	32	300	2	651.86285	1302.71843	0.03	47.20	4644	4644	MS2	14/14	Glioma_Nuclear_prep_Gradeall_repeat_4plex.raw
4502107	High		qEISAAFk	Unambiguous	1	1	N-Term(iTRAQ4plex); K8(iTRAQ4plex)	HCD	1.0000	0.0000	1	1	8223	Unique	Used	1.674	5.580E4	1.140E5	345	3.05	0.00	0	52	15	2	591.33167	1181.65605	-17.62	27.93	2694	2694	MS2	14/14	Glioma_Nuclear_prep_Gradeall_4plex.raw
4502107	High		lYDAYELk	Unambiguous	1	1	N-Term(iTRAQ4plex); K8(iTRAQ4plex)	HCD	1.0000	0.0000	1	1	9546	Unique	Used	1.484	1.249E5	2.262E5	214	2.93	0.00	0	47	13	2	651.85236	1302.69744	-16.08	37.58	4137	4137	MS2	14/14	Glioma_Nuclear_prep_Gradeall_4plex.raw
4502277	NP_001668.1	ATP1B1	481	sodium/potassium-transporting ATPase subunit beta-1 [Homo sapiens]	8.25	1	1	2	0.674	1;1	1.6	11.53	303	35.0	8.53																			
4502277		High	sYEAYVLNIVR	Unambiguous	1	1	N-Term(iTRAQ4plex)	HCD	1.0000	0.0000	1	1	12183	Unique	Used	0.666	2.223E3	1.792E3	259	3.94	0.00	0	23	300	2	735.90863	1470.80998	1.81	75.75	7071	7071	MS2	18/20	Glioma_Nuclear_prep_Gradeall_repeat_4plex.raw
4502277	High		sYEAYVLNIVR	Unambiguous	1	1	N-Term(iTRAQ4plex)	HCD	1.0000	0.0000	1	1	11536	Unique	Used	0.682	6.974E4	5.799E4	270	3.29	0.00	0	58	50	2	735.90485	1470.80242	-3.34	52.89	6307	6307	MS2	16/20	Glioma_Nuclear_prep_Gradeall_4plex.raw
4503183	NP_001905.1	CYB5A	1528	cytochrome b5 isoform 2 [Homo sapiens]	43.88	3	2	3	1.887	1;2	23.8	15.63	98	11.3	5.14																			
4503183		High	fLEEHPGGEEVLR	Unambiguous	3	1	N-Term(iTRAQ4plex)	HCD	1.0000	0.0000	1	1	9640	Unique	Used	2.417	1.087E3	3.180E3	174	3.34	0.00	0	63	300	3	552.62006	1655.84562	-3.27	40.85	4066	4066	MS2	14/48	Glioma_Nuclear_prep_Gradeall_repeat_4plex.raw
4503183	High		yYTLEEIQk	Unambiguous	3	1	N-Term(iTRAQ4plex); K9(iTRAQ4plex)	HCD	1.0000	0.0000	1	1	9609	Unique	Used	1.499	1.294E5	2.366E5	242	3.83	0.00	0	47	20	2	737.89368	1474.78008	-15.66	38.03	4205	4205	MS2	15/16	Glioma_Nuclear_prep_Gradeall_4plex.raw
4503183		High	yYTLEEIQk	Unambiguous	3	1	N-Term(iTRAQ4plex); K9(iTRAQ4plex)	HCD	1.0000	0.0000	1	1	10273	Unique	Used	1.887	4.587E3	1.047E4	286	3.89	0.00	0	45	300	2	737.90649	1474.80571	1.72	48.31	4745	4745	MS2	16/16	Glioma_Nuclear_prep_Gradeall_repeat_4plex.raw
4503337	NP_001354.1	DKC1	1736	H/ACA ribonucleoprotein complex subunit 4 isoform 1 [Homo sapiens]	17.90	3	6	8	1.789	3;5	13.0	24.69	514	57.6	9.42																			
4503337		High	lHNAIEGGTQLSR	Unambiguous	3	1	N-Term(iTRAQ4plex)	HCD	1.0000	0.0000	1	1	8142	Unique	Used	1.644	4.229E3	8.411E3	257	3.25	0.00	0	25	300	2	770.42120	1539.83513	-0.58	25.16	2475	2475	MS2	16/24	Glioma_Nuclear_prep_Gradeall_repeat_4plex.raw
4503337		High	tTHYTPLAcGSNPLk	Unambiguous	3	1	N-Term(iTRAQ4plex); C9(Methylthio); K15(iTRAQ4plex)	HCD	1.0000	0.0000	1	1	10147	Unique	Used	1.347	1.251E3	2.039E3	164	6.32	0.00	0	25	276	3	646.33356	1936.98612	-0.12	46.84	4612	4612	MS2	28/56	Glioma_Nuclear_prep_Gradeall_repeat_4plex.raw
4503337	High		tTHYTPLAcGSNPLk	Unambiguous	3	1	N-Term(iTRAQ4plex); C9(Methylthio); K15(iTRAQ4plex)	HCD	1.0000	0.0000	1	1	9416	Unique	Used	1.435	1.006E4	1.760E4	127	5.86	0.00	0	20	11	3	646.32294	1936.95426	-16.57	36.62	3995	3995	MS2	28/56	Glioma_Nuclear_prep_Gradeall_4plex.raw
4503337	High		kEVVAEVVk	Unambiguous	2	1	N-Term(iTRAQ4plex); K1(iTRAQ4plex); K9(iTRAQ4plex)	HCD	1.0000	0.0000	1	1	7832	Unique	Used	1.746	9.903E4	2.109E5	124	3.29	0.00	1	23	13	2	716.95770	1432.90813	-1.29	25.18	2271	2271	MS2	16/16	Glioma_Nuclear_prep_Gradeall_4plex.raw
4503337		High	aPQVVAEAAk	Unambiguous	2	1	N-Term(iTRAQ4plex); K10(iTRAQ4plex)	HCD	1.0000	0.0000	1	1	8125	Unique	Used	1.879	1.385E4	3.149E4	251	4.08	0.00	0	42	300	2	636.37524	1271.74321	-10.22	24.98	2457	2457	MS2	16/18	Glioma_Nuclear_prep_Gradeall_repeat_4plex.raw
4503337	High		aPQVVAEAAk	Unambiguous	2	1	N-Term(iTRAQ4plex); K10(iTRAQ4plex)	HCD	1.0000	0.0000	1	1	7903	Unique	Used	1.916	1.436E5	3.358E5	171	4.06	0.00	0	21	9	2	636.38202	1271.75676	0.44	25.68	2348	2348	MS2	16/18	Glioma_Nuclear_prep_Gradeall_4plex.raw
4503337		High	qEYVDYSESAk	Unambiguous	1	1	N-Term(iTRAQ4plex); K11(iTRAQ4plex)	HCD	1.0000	0.0000	1	1	8324	Unique	Used	1.934	5.191E3	1.215E4	116	3.59	0.00	0	12	271	2	803.89911	1606.79094	4.38	26.92	2667	2667	MS2	18/20	Glioma_Nuclear_prep_Gradeall_repeat_4plex.raw
4503337		High	lDTSQWPLLLk	Unambiguous	3	1	N-Term(iTRAQ4plex); K11(iTRAQ4plex)	HCD	1.0000	0.0000	1	1	12332	Unique	Used	1.833	8.563E2	1.899E3	165	3.76	0.00	0	16	300	2	801.47955	1601.95183	0.80	79.98	7386	7386	MS2	18/20	Glioma_Nuclear_prep_Gradeall_repeat_4plex.raw
4503571	NP_001419.1	ENO1	2023	alpha-enolase isoform 1 [Homo sapiens]	7.83	2	3	4	1.929	2;2	4.6	9.21	434	47.1	7.39																			
4503571		High	yISPDQLADLYk	Unambiguous	2	1	N-Term(iTRAQ4plex); K12(iTRAQ4plex)	HCD	1.0000	0.0000	1	1	11864	Unique	Used	1.933	1.183E3	2.767E3	249	4.36	0.00	0	26	300	2	857.46912	1713.93096	0.45	69.67	6590	6590	MS2	19/22	Glioma_Nuclear_prep_Gradeall_repeat_4plex.raw
4503571	High		yISPDQLADLYk	Unambiguous	2	1	N-Term(iTRAQ4plex); K12(iTRAQ4plex)	HCD	1.0000	0.0000	1	1	11079	Unique	Used	1.925	1.078E4	2.533E4	259	4.77	0.00	0	57	58	2	857.46625	1713.92522	-2.90	49.07	5807	5807	MS2	18/22	Glioma_Nuclear_prep_Gradeall_4plex.raw
4503571	High		gNPTVEVDLFTSk	Unambiguous	1	1	N-Term(iTRAQ4plex); K13(iTRAQ4plex)	HCD	1.0000	0.0000	1	1	10169	Unique	Used	1.771	2.966E4	6.407E4	238	4.43	0.00	0	22	46	2	847.95081	1694.89433	-15.37	42.12	4814	4814	MS2	22/24	Glioma_Nuclear_prep_Gradeall_4plex.raw
4503571		High	tIAPALVSk	Unambiguous	1	1	N-Term(iTRAQ4plex); K9(iTRAQ4plex)	HCD	1.0000	0.0000	1	1	8945	Unique	Used	1.966	1.060E4	2.522E4	173	3.45	0.00	0	54	213	2	594.38025	1187.75322	-5.89	33.06	3321	3321	MS2	14/16	Glioma_Nuclear_prep_Gradeall_repeat_4plex.raw
4503979	NP_002046.1	GFAP	2670	glial fibrillary acidic protein isoform 1 [Homo sapiens]	38.66	3	16	23	2.076	9;14	19.9	54.61	432	49.8	5.52																			
4503979		High	lEAENNLAAYR	Unambiguous	3	1	N-Term(iTRAQ4plex)	HCD	1.0000	0.0000	1	1	9361	Unique	Used	2.592	8.523E3	2.673E4	203	4.18	0.00	0	13	146	2	704.37256	1407.73784	2.09	37.74	3764	3764	MS2	19/20	Glioma_Nuclear_prep_Gradeall_repeat_4plex.raw
4503979		High	lADVYQAELR	Unambiguous	3	1	N-Term(iTRAQ4plex)	HCD	1.0000	0.0000	1	1	10231	Unique	Used	2.174	5.526E3	1.454E4	271	3.61	0.00	0	16	300	2	661.36517	1321.72307	-0.15	47.82	4701	4701	MS2	17/18	Glioma_Nuclear_prep_Gradeall_repeat_4plex.raw
4503979		High	fADLTDAAAR	Unambiguous	3	1	N-Term(iTRAQ4plex)	HCD	1.0000	0.0000	1	1	9402	Unique	Used	2.076	4.014E4	1.008E5	273	3.53	0.00	0	16	48	2	597.81573	1194.62419	0.52	38.17	3807	3807	MS2	17/18	Glioma_Nuclear_prep_Gradeall_repeat_4plex.raw
4503979		High	qEADEATLAR	Unambiguous	3	1	N-Term(iTRAQ4plex)	HCD	1.0000	0.0000	1	1	7280	Unique	Used	2.009	8.225E3	1.999E4	130	3.47	0.00	0	35	284	2	624.31091	1247.61455	-16.28	17.20	1564	1564	MS2	17/18	Glioma_Nuclear_prep_Gradeall_repeat_4plex.raw
4503979		High	dNLAQDLATVR	Unambiguous	3	1	N-Term(iTRAQ4plex)	HCD	1.0000	0.0000	1	1	10376	Unique	Used	1.942	4.927E3	1.158E4	329	4.13	0.00	0	23	300	2	680.37109	1359.73491	0.00	49.55	4856	4856	MS2	19/20	Glioma_Nuclear_prep_Gradeall_repeat_4plex.raw
4503979		High	aLAAELNQLR	Unambiguous	3	1	N-Term(iTRAQ4plex)	HCD	1.0000	0.0000	1	1	10676	Unique	Used	1.904	5.326E3	1.227E4	241	3.67	0.00	0	25	300	2	621.86816	1242.72905	0.28	53.15	5178	5178	MS2	17/18	Glioma_Nuclear_prep_Gradeall_repeat_4plex.raw
4503979		High	lLEGEENR	Unambiguous	3	1	N-Term(iTRAQ4plex)	HCD	1.0000	0.0000	1	1	7815	Unique	Used	1.758	1.811E4	3.851E4	230	2.90	0.00	0	43	180	2	552.29462	1103.58196	0.54	22.00	2131	2131	MS2	12/14	Glioma_Nuclear_prep_Gradeall_repeat_4plex.raw
4503979		High	lQDETNLR	Unambiguous	3	1	N-Term(iTRAQ4plex)	HCD	1.0000	0.0000	1	1	7925	Unique	Used	1.605	2.565E4	4.980E4	221	3.10	0.00	0	26	71	2	566.80737	1132.60747	-0.38	23.05	2247	2247	MS2	14/14	Glioma_Nuclear_prep_Gradeall_repeat_4plex.raw
4503979	High		lEAENNLAAYR	Unambiguous	3	1	N-Term(iTRAQ4plex)	HCD	1.0000	0.0000	1	1	8902	Unique	Used	2.575	1.493E5	4.690E5	196	3.41	0.00	0	39	12	2	704.35925	1407.71123	-16.82	32.81	3432	3432	MS2	17/20	Glioma_Nuclear_prep_Gradeall_4plex.raw
4503979	High		dNLAQDLATVR	Unambiguous	3	1	N-Term(iTRAQ4plex)	HCD	1.0000	0.0000	1	1	9713	Unique	Used	2.460	1.272E5	3.818E5	276	3.91	0.00	0	22	6	2	680.36011	1359.71294	-16.16	38.78	4318	4318	MS2	18/20	Glioma_Nuclear_prep_Gradeall_4plex.raw
4503979	High		eAASYQEALAR	Unambiguous	3	1	N-Term(iTRAQ4plex)	HCD	1.0000	0.0000	1	1	8189	Unique	Used	2.294	2.555E5	7.149E5	174	3.91	0.00	0	32	4	2	676.83807	1352.66887	-17.62	27.70	2658	2658	MS2	17/20	Glioma_Nuclear_prep_Gradeall_4plex.raw
4503979	High		lADVYQAELR	Unambiguous	3	1	N-Term(iTRAQ4plex)	HCD	1.0000	0.0000	1	1	9628	Unique	Used	1.862	1.128E5	2.563E5	268	3.40	0.00	0	22	10	2	661.35413	1321.70098	-16.87	38.17	4226	4226	MS2	17/18	Glioma_Nuclear_prep_Gradeall_4plex.raw
4503979	High		aLAAELNQLR	Unambiguous	3	1	N-Term(iTRAQ4plex)	HCD	1.0000	0.0000	1	1	9896	Unique	Used	1.564	1.054E5	2.010E5	249	3.35	0.00	0	33	11	2	621.85791	1242.70854	-16.22	40.11	4517	4517	MS2	17/18	Glioma_Nuclear_prep_Gradeall_4plex.raw
4503979		High	qLQSLTcDLESLR	Unambiguous	3	1	N-Term(iTRAQ4plex); C7(Methylthio)	HCD	1.0000	0.0000	1	1	12342	Unique	Used	1.897	6.196E2	1.422E3	169	3.56	0.00	0	18	300	2	848.43103	1695.85478	1.25	80.48	7420	7420	MS2	16/24	Glioma_Nuclear_prep_Gradeall_repeat_4plex.raw
4503979	High		qLQSLTcDLESLR	Unambiguous	3	1	N-Term(iTRAQ4plex); C7(Methylthio)	HCD	1.0000	0.0000	1	1	11894	Unique	Used	2.590	2.868E4	9.062E4	204	4.74	0.00	0	6	50	2	848.43439	1695.86150	5.21	56.00	6696	6696	MS2	20/24	Glioma_Nuclear_prep_Gradeall_4plex.raw
4503979	High		kIESLEEEIR	Unambiguous	3	1	N-Term(iTRAQ4plex); K1(iTRAQ4plex)	HCD	1.0000	0.0000	1	1	9124	Unique	Used	2.269	8.576E4	2.374E5	230	3.36	0.00	1	9	6	2	767.42664	1533.84599	-17.41	34.47	3676	3676	MS2	18/18	Glioma_Nuclear_prep_Gradeall_4plex.raw
4503979		High	hLQEYQDLLNVk	Unambiguous	3	1	N-Term(iTRAQ4plex); K12(iTRAQ4plex)	HCD	1.0000	0.0000	1	1	10576	Unique	Used	1.835	2.188E3	4.857E3	197	5.37	0.00	0	38	300	2	894.49872	1787.99016	0.40	51.94	5070	5070	MS2	22/22	Glioma_Nuclear_prep_Gradeall_repeat_4plex.raw
4503979	High		hLQEYQDLLNVk	Unambiguous	3	1	N-Term(iTRAQ4plex); K12(iTRAQ4plex)	HCD	1.0000	0.0000	1	1	9750	Unique	Used	2.627	6.902E4	2.212E5	192	5.26	0.00	0	13	15	2	894.48425	1787.96123	-15.78	39.04	4358	4358	MS2	22/22	Glioma_Nuclear_prep_Gradeall_4plex.raw
4503979		High	fASYIEk	Unambiguous	3	1	N-Term(iTRAQ4plex); K7(iTRAQ4plex)	HCD	1.0000	0.0000	1	1	9591	Unique	Used	3.322	2.108E4	8.473E4	158	2.97	0.00	0	19	66	2	573.32288	1145.63848	-5.26	40.31	4014	4014	MS2	12/12	Glioma_Nuclear_prep_Gradeall_repeat_4plex.raw
4503979		High	sVSEGHLk	Unambiguous	1	1	N-Term(iTRAQ4plex); K8(iTRAQ4plex)	HCD	1.0000	0.0000	1	1	6864	Unique	Used	1.752	1.054E4	2.235E4	249	2.97	0.00	0	21	93	2	572.82861	1144.64995	-5.69	13.56	1124	1124	MS2	14/14	Glioma_Nuclear_prep_Gradeall_repeat_4plex.raw
4503979		High	lEEEGQSLkDEMAR	Unambiguous	3	1	N-Term(iTRAQ4plex); K9(iTRAQ4plex)	HCD	1.0000	0.0000	1	1	10102	Unique	Used	2.441	8.163E2	2.410E3	175	5.00	0.00	1	4	300	2	961.99164	1922.97600	1.43	46.32	4564	4564	MS2	24/26	Glioma_Nuclear_prep_Gradeall_repeat_4plex.raw
4503979		High	lEEEGQSLk	Unambiguous	3	1	N-Term(iTRAQ4plex); K9(iTRAQ4plex)	HCD	1.0000	0.0000	1	1	8263	Unique	Used	1.606	1.043E4	2.025E4	310	3.06	0.00	0	51	232	2	660.86633	1320.72539	0.33	26.32	2603	2603	MS2	16/16	Glioma_Nuclear_prep_Gradeall_repeat_4plex.raw
4503979	High		lEEEGQSLkDEMAR	Unambiguous	3	1	N-Term(iTRAQ4plex); K9(iTRAQ4plex)	HCD	1.0000	0.0000	1	1	9427	Unique	Used	2.253	1.981E4	5.445E4	119	4.96	0.00	1	20	28	2	961.97290	1922.93852	-18.06	36.70	4007	4007	MS2	23/26	Glioma_Nuclear_prep_Gradeall_4plex.raw
4504255	NP_002097.1	H2AFZ	3015	histone H2A.Z [Homo sapiens]	25.78	6	2	5	1.561	2;3	8.1	24.17	128	13.5	10.58																			
4504255		High	gDEELDSLIk	Unambiguous	5	1	N-Term(iTRAQ4plex); K10(iTRAQ4plex)	HCD	1.0000	0.0000	1	1	10297	Unique	Used	1.407	5.113E3	8.700E3	260	4.50	0.00	0	19	300	2	703.88446	1406.76164	-0.07	48.63	4772	4772	MS2	18/18	Glioma_Nuclear_prep_Gradeall_repeat_4plex.raw
4504255		High	aTIAGGGVIPHIHk	Unambiguous	4	1	N-Term(iTRAQ4plex); K14(iTRAQ4plex)	HCD	1.0000	0.0000	1	1	9057	Unique	Used	1.583	2.108E3	4.038E3	108	5.89	0.00	0	35	300	2	830.00323	1658.99919	2.84	34.39	3443	3443	MS2	25/26	Glioma_Nuclear_prep_Gradeall_repeat_4plex.raw
4504255		High	aTIAGGGVIPHIHk	Unambiguous	4	1	N-Term(iTRAQ4plex); K14(iTRAQ4plex)	HCD	1.0000	0.0000	1	1	9038	Unique	Used	1.561	4.776E2	9.022E2	127	5.07	0.00	0	54	300	4	415.50314	1658.99074	-2.26	34.17	3423	3423	MS2	29/78	Glioma_Nuclear_prep_Gradeall_repeat_4plex.raw
4504255	High		aTIAGGGVIPHIHk	Unambiguous	4	1	N-Term(iTRAQ4plex); K14(iTRAQ4plex)	HCD	1.0000	0.0000	1	1	8448	Unique	Used	1.756	4.939E4	1.058E5	165	5.62	0.00	0	4	8	2	829.98712	1658.96697	-16.59	29.53	2938	2938	MS2	25/26	Glioma_Nuclear_prep_Gradeall_4plex.raw
4504255	High		aTIAGGGVIPHIHk	Unambiguous	4	1	N-Term(iTRAQ4plex); K14(iTRAQ4plex)	HCD	1.0000	0.0000	1	1	8440	Unique	Used	1.522	2.192E4	4.070E4	177	4.27	0.00	0	8	1	4	415.49701	1658.96621	-17.05	29.48	2930	2930	MS2	31/78	Glioma_Nuclear_prep_Gradeall_4plex.raw
4504313	NP_003535.1	HIST1H4B	8366	histone H4 [Homo sapiens]	51.46	1	6	25	2.142	12;13	38.5	68.81	103	11.4	11.36																			
4504313		High	vFLENVIR	Unambiguous	1	1	N-Term(iTRAQ4plex)	HCD	1.0000	0.0000	1	1	11209	Unique	Used	3.382	1.534E4	6.276E4	271	3.03	0.00	0	6	71	2	567.34393	1133.68059	0.56	59.91	5772	5772	MS2	14/14	Glioma_Nuclear_prep_Gradeall_repeat_4plex.raw
4504313		High	vFLENVIR	Unambiguous	1	1	N-Term(iTRAQ4plex)	HCD	1.0000	0.0000	1	1	11126	Unique	Used	2.506	2.166E3	6.568E3	340	2.84	0.00	0	29	300	2	567.34338	1133.67949	-0.41	58.82	5676	5676	MS2	13/14	Glioma_Nuclear_prep_Gradeall_repeat_4plex.raw
4504313		High	iSGLIYEETR	Unambiguous	1	1	N-Term(iTRAQ4plex)	HCD	1.0000	0.0000	1	1	10555	Unique	Used	2.413	1.120E4	3.270E4	274	3.66	0.00	0	8	64	2	662.86560	1324.72392	0.75	51.70	5048	5048	MS2	17/18	Glioma_Nuclear_prep_Gradeall_repeat_4plex.raw
4504313		High	iSGLIYEETR	Unambiguous	1	1	N-Term(iTRAQ4plex)	HCD	1.0000	0.0000	1	1	10478	Unique	Used	1.798	4.199E3	9.132E3	366	3.71	0.00	0	41	300	2	662.86560	1324.72392	0.75	50.76	4964	4964	MS2	17/18	Glioma_Nuclear_prep_Gradeall_repeat_4plex.raw
4504313	High		vFLENVIR	Unambiguous	1	1	N-Term(iTRAQ4plex)	HCD	1.0000	0.0000	1	1	10257	Unique	Used	2.994	1.636E5	5.974E5	322	3.25	0.00	0	11	4	2	567.33478	1133.66228	-15.59	42.77	4910	4910	MS2	13/14	Glioma_Nuclear_prep_Gradeall_4plex.raw
4504313	High		iSGLIYEETR	Unambiguous	1	1	N-Term(iTRAQ4plex)	HCD	1.0000	0.0000	1	1	9793	Unique	Used	1.582	2.742E5	5.290E5	286	3.66	0.00	0	23	4	2	662.85443	1324.70159	-16.12	39.35	4405	4405	MS2	17/18	Glioma_Nuclear_prep_Gradeall_4plex.raw
4504313	High		kTVTAMDVVYALk	Unambiguous	1	1	N-Term(iTRAQ4plex); K1(iTRAQ4plex); K13(iTRAQ4plex)	HCD	1.0000	0.0000	1	1	10700	Unique	Used	1.682	4.159E4	8.534E4	147	5.63	0.00	1	28	22	2	936.04224	1871.07720	-14.15	46.16	5395	5395	MS2	23/24	Glioma_Nuclear_prep_Gradeall_4plex.raw
4504313		High	dAVTYTEHAk	Unambiguous	1	1	N-Term(iTRAQ4plex); K10(iTRAQ4plex)	HCD	1.0000	0.0000	1	1	7327	Unique	Used	3.185	2.058E4	7.931E4	211	4.29	0.00	0	9	5	3	474.91681	1422.73587	-7.64	17.65	1614	1614	MS2	24/36	Glioma_Nuclear_prep_Gradeall_repeat_4plex.raw
4504313		High	dAVTYTEHAk	Unambiguous	1	1	N-Term(iTRAQ4plex); K10(iTRAQ4plex)	HCD	1.0000	0.0000	1	1	7329	Unique	Used	2.834	1.667E4	5.715E4	223	4.68	0.00	0	8	31	2	711.87164	1422.73601	-7.54	17.66	1616	1616	MS2	18/18	Glioma_Nuclear_prep_Gradeall_repeat_4plex.raw
4504313	High		dAVTYTEHAk	Unambiguous	1	1	N-Term(iTRAQ4plex); K10(iTRAQ4plex)	HCD	1.0000	0.0000	1	1	7389	Unique	Used	4.042	2.899E4	1.430E5	210	4.12	0.00	0	16	3	3	474.91730	1422.73734	-6.61	22.02	1791	1791	MS2	24/36	Glioma_Nuclear_prep_Gradeall_4plex.raw
4504313	High		dAVTYTEHAk	Unambiguous	1	1	N-Term(iTRAQ4plex); K10(iTRAQ4plex)	HCD	1.0000	0.0000	1	1	7400	Unique	Used	2.439	4.602E4	1.370E5	229	4.71	0.00	0	11	11	2	711.87225	1422.73723	-6.68	22.11	1803	1803	MS2	18/18	Glioma_Nuclear_prep_Gradeall_4plex.raw
4504313		High	tVTAMDVVYALk	Unambiguous	1	1	N-Term(iTRAQ4plex); K12(iTRAQ4plex)	HCD	1.0000	0.0000	1	1	12291	Unique	Used	3.760	1.728E2	7.861E2	230	4.16	0.00	0	9	179	3	533.64001	1598.90549	-0.72	78.81	7299	7299	MS2	22/44	Glioma_Nuclear_prep_Gradeall_repeat_4plex.raw
4504313		High	tVTAMDVVYALk	Unambiguous	1	1	N-Term(iTRAQ4plex); K12(iTRAQ4plex)	HCD	1.0000	0.0000	1	1	12289	Unique	Used	2.820	1.634E3	5.575E3	233	5.90	0.00	0	6	300	2	799.95709	1598.90691	0.17	78.76	7295	7295	MS2	21/22	Glioma_Nuclear_prep_Gradeall_repeat_4plex.raw
4504313	High		tVTAMDVVYALk	Unambiguous	1	1	N-Term(iTRAQ4plex); K12(iTRAQ4plex)	HCD	1.0000	0.0000	1	1	11775	Unique	Used	4.833	3.969E3	2.341E4	239	4.23	0.00	0	9	7	3	533.63898	1598.90238	-2.66	55.00	6567	6567	MS2	23/44	Glioma_Nuclear_prep_Gradeall_4plex.raw
4504313	High		tVTAMDVVYALk	Unambiguous	1	1	N-Term(iTRAQ4plex); K12(iTRAQ4plex)	HCD	1.0000	0.0000	1	1	11762	Unique	Used	3.081	8.596E4	3.232E5	230	5.78	0.00	0	8	6	2	799.95691	1598.90654	-0.06	54.89	6553	6553	MS2	21/22	Glioma_Nuclear_prep_Gradeall_4plex.raw
4504313	High		tVTAMDVVYALk	Unambiguous	1	1	N-Term(iTRAQ4plex); K12(iTRAQ4plex)	HCD	1.0000	0.0000	1	1	11694	Unique	Used	2.142	6.707E3	1.753E4	251	4.10	0.00	0	30	9	3	533.63824	1598.90018	-4.04	54.27	6479	6479	MS2	19/44	Glioma_Nuclear_prep_Gradeall_4plex.raw
4504313	High		tVTAMDVVYALk	Unambiguous	1	1	N-Term(iTRAQ4plex); K12(iTRAQ4plex)	HCD	1.0000	0.0000	1	1	11685	Unique	Used	1.982	3.890E4	9.407E4	234	6.00	0.00	0	22	53	2	799.95740	1598.90752	0.55	54.19	6469	6469	MS2	21/22	Glioma_Nuclear_prep_Gradeall_4plex.raw
4504313		High	dNIQGITkPAIR	Unambiguous	1	1	N-Term(iTRAQ4plex); K8(iTRAQ4plex)	HCD	1.0000	0.0000	1	1	8757	Unique	Used	1.292	3.192E3	4.988E3	119	3.90	0.00	0	25	296	2	807.47198	1613.93669	-13.06	31.13	3123	3123	MS2	16/22	Glioma_Nuclear_prep_Gradeall_repeat_4plex.raw
4504313	High		dNIQGITkPAIR	Unambiguous	1	1	N-Term(iTRAQ4plex); K8(iTRAQ4plex)	HCD	1.0000	0.0000	1	1	8312	Unique	Used	1.461	8.531E4	1.521E5	205	3.85	0.00	0	21	7	2	807.46869	1613.93010	-17.14	28.57	2791	2791	MS2	17/22	Glioma_Nuclear_prep_Gradeall_4plex.raw
4504313		High	tVTAmDVVYALk	Unambiguous	1	1	N-Term(iTRAQ4plex); M5(Oxidation); K12(iTRAQ4plex)	HCD	1.0000	0.0000	1	1	11434	Unique	Used	2.053	2.367E3	5.878E3	210	5.11	0.00	0	23	300	2	807.95465	1614.90203	0.29	63.27	6053	6053	MS2	20/22	Glioma_Nuclear_prep_Gradeall_repeat_4plex.raw
4504313		High	tVTAmDVVYALk	Unambiguous	1	1	N-Term(iTRAQ4plex); M5(Oxidation); K12(iTRAQ4plex)	HCD	1.0000	0.0000	1	1	11356	Unique	Used	1.764	1.371E3	2.925E3	210	4.84	0.00	0	18	300	2	807.95477	1614.90227	0.44	62.20	5961	5961	MS2	22/22	Glioma_Nuclear_prep_Gradeall_repeat_4plex.raw
4504313		High	tVTAmDVVYALk	Unambiguous	1	1	N-Term(iTRAQ4plex); M5(Oxidation); K12(iTRAQ4plex)	HCD	1.0000	0.0000	1	1	11439	Unique	Used	1.615	7.399E2	1.446E3	220	4.84	0.00	0	11	217	3	538.97186	1614.90104	-0.32	63.34	6059	6059	MS2	21/44	Glioma_Nuclear_prep_Gradeall_repeat_4plex.raw
4504313		High	tVTAmDVVYALk	Unambiguous	1	1	N-Term(iTRAQ4plex); M5(Oxidation); K12(iTRAQ4plex)	HCD	1.0000	0.0000	1	1	11361	Unique	Used	1.331	2.945E2	4.741E2	216	4.25	0.00	0	22	300	3	538.97205	1614.90158	0.02	62.27	5967	5967	MS2	20/44	Glioma_Nuclear_prep_Gradeall_repeat_4plex.raw
4504313	High		tVTAmDVVYALk	Unambiguous	1	1	N-Term(iTRAQ4plex); M5(Oxidation); K12(iTRAQ4plex)	HCD	1.0000	0.0000	1	1	10606	Unique	Used	1.856	6.613E4	1.497E5	226	5.01	0.00	0	15	9	2	807.94202	1614.87676	-15.35	45.44	5292	5292	MS2	20/22	Glioma_Nuclear_prep_Gradeall_4plex.raw
4504313	High		tVTAmDVVYALk	Unambiguous	1	1	N-Term(iTRAQ4plex); M5(Oxidation); K12(iTRAQ4plex)	HCD	1.0000	0.0000	1	1	10515	Unique	Used	1.423	4.122E4	7.156E4	223	4.93	0.00	0	32	32	2	807.94171	1614.87615	-15.73	44.76	5193	5193	MS2	20/22	Glioma_Nuclear_prep_Gradeall_4plex.raw
4504425	NP_002119.1	HMGB1	3146	high mobility group protein B1 [Homo sapiens]	27.44	1	5	16	2.634	6;10	38.2	64.64	215	24.9	5.74																			
4504425		High	kHPDASVNFSEFSk	Unambiguous	1	1	N-Term(iTRAQ4plex); K1(iTRAQ4plex); K14(iTRAQ4plex)	HCD	1.0000	0.0000	1	1	9078	Unique	Used	4.731	1.265E3	7.240E3	119	5.57	0.00	1	14	70	3	675.69849	2025.08091	2.13	34.63	3466	3466	MS2	24/52	Glioma_Nuclear_prep_Gradeall_repeat_4plex.raw
4504425		High	kHPDASVNFSEFSk	Unambiguous	1	1	N-Term(iTRAQ4plex); K1(iTRAQ4plex); K14(iTRAQ4plex)	HCD	1.0000	0.0000	1	1	9087	Unique	Used	3.594	1.344E3	5.842E3	97	4.85	0.00	1	0	300	2	1013.04340	2025.07952	1.44	34.72	3475	3475	MS2	22/26	Glioma_Nuclear_prep_Gradeall_repeat_4plex.raw
4504425		High	kHPDASVNFSEFSk	Unambiguous	1	1	N-Term(iTRAQ4plex); K1(iTRAQ4plex); K14(iTRAQ4plex)	HCD	1.0000	0.0000	1	1	9095	Unique	Used	2.118	5.103E2	1.308E3	178	4.06	0.00	1	24	68	4	507.02374	2025.07314	-1.71	34.82	3484	3484	MS2	15/78	Glioma_Nuclear_prep_Gradeall_repeat_4plex.raw
4504425	High		kHPDASVNFSEFSk	Unambiguous	1	1	N-Term(iTRAQ4plex); K1(iTRAQ4plex); K14(iTRAQ4plex)	HCD	1.0000	0.0000	1	1	8459	Unique	Used	2.953	2.296E4	8.271E4	140	4.04	0.00	1	7	12	2	1013.02338	2025.03948	-18.33	29.60	2950	2950	MS2	20/26	Glioma_Nuclear_prep_Gradeall_4plex.raw
4504425		High	hPDASVNFSEFSk	Unambiguous	1	1	N-Term(iTRAQ4plex); K13(iTRAQ4plex)	HCD	1.0000	0.0000	1	1	9659	Unique	Used	3.064	1.863E3	6.903E3	147	4.49	0.00	0	10	300	2	876.94238	1752.87749	-1.19	41.07	4086	4086	MS2	19/24	Glioma_Nuclear_prep_Gradeall_repeat_4plex.raw
4504425		High	gEHPGLSIGDVAk	Unambiguous	1	1	N-Term(iTRAQ4plex); K13(iTRAQ4plex)	HCD	1.0000	0.0000	1	1	8745	Unique	Used	2.825	2.713E3	9.271E3	242	5.47	0.00	0	10	53	3	523.28613	1567.84385	-15.59	31.02	3111	3111	MS2	24/48	Glioma_Nuclear_prep_Gradeall_repeat_4plex.raw
4504425		High	gEHPGLSIGDVAk	Unambiguous	1	1	N-Term(iTRAQ4plex); K13(iTRAQ4plex)	HCD	1.0000	0.0000	1	1	8755	Unique	Used	2.506	1.566E3	4.749E3	204	3.13	0.00	0	6	257	2	784.42700	1567.84673	-13.75	31.11	3121	3121	MS2	20/24	Glioma_Nuclear_prep_Gradeall_repeat_4plex.raw
4504425		High	gEHPGLSIGDVAk	Unambiguous	1	1	N-Term(iTRAQ4plex); K13(iTRAQ4plex)	HCD	1.0000	0.0000	1	1	8723	Unique	Used	1.919	1.720E3	3.992E3	230	6.04	0.00	0	39	242	3	523.29498	1567.87040	1.34	30.84	3087	3087	MS2	21/48	Glioma_Nuclear_prep_Gradeall_repeat_4plex.raw
4504425		High	hPDASVNFSEFSk	Unambiguous	1	1	N-Term(iTRAQ4plex); K13(iTRAQ4plex)	HCD	1.0000	0.0000	1	1	9650	Unique	Used	1.668	1.394E3	2.814E3	165	6.29	0.00	0	9	138	3	584.96356	1752.87613	-1.96	40.97	4077	4077	MS2	24/48	Glioma_Nuclear_prep_Gradeall_repeat_4plex.raw
4504425	High		hPDASVNFSEFSk	Unambiguous	1	1	N-Term(iTRAQ4plex); K13(iTRAQ4plex)	HCD	1.0000	0.0000	1	1	9006	Unique	Used	2.922	2.967E4	1.058E5	120	4.44	0.00	0	0	14	2	876.92950	1752.85173	-15.88	33.56	3545	3545	MS2	17/24	Glioma_Nuclear_prep_Gradeall_4plex.raw
4504425	High		gEHPGLSIGDVAk	Unambiguous	1	1	N-Term(iTRAQ4plex); K13(iTRAQ4plex)	HCD	1.0000	0.0000	1	1	8284	Unique	Used	1.884	3.025E4	6.951E4	254	3.76	0.00	0	56	21	2	784.42365	1567.84001	-18.03	28.36	2760	2760	MS2	18/24	Glioma_Nuclear_prep_Gradeall_4plex.raw
4504425	High		hPDASVNFSEFSk	Unambiguous	1	1	N-Term(iTRAQ4plex); K13(iTRAQ4plex)	HCD	1.0000	0.0000	1	1	9015	Unique	Used	1.620	1.521E4	3.006E4	125	5.25	0.00	0	19	9	3	584.95447	1752.84885	-17.52	33.63	3555	3555	MS2	24/48	Glioma_Nuclear_prep_Gradeall_4plex.raw
4504425		High	ikGEHPGLSIGDVAk	Unambiguous	1	1	N-Term(iTRAQ4plex); K2(iTRAQ4plex); K15(iTRAQ4plex)	HCD	1.0000	0.0000	1	1	8902	Unique	Used	2.517	1.443E3	4.394E3	105	3.50	0.00	1	14	45	3	651.71893	1953.14225	-3.66	32.67	3276	3276	MS2	25/56	Glioma_Nuclear_prep_Gradeall_repeat_4plex.raw
4504425	High		ikGEHPGLSIGDVAk	Unambiguous	1	1	N-Term(iTRAQ4plex); K2(iTRAQ4plex); K15(iTRAQ4plex)	HCD	1.0000	0.0000	1	1	8373	Unique	Used	5.114	2.034E4	1.269E5	164	4.51	0.00	1	5	7	2	977.06030	1953.11333	-18.46	29.00	2857	2857	MS2	22/28	Glioma_Nuclear_prep_Gradeall_4plex.raw
4504425		High	tYIPPkGETk	Unambiguous	1	1	N-Term(iTRAQ4plex); K6(iTRAQ4plex); K10(iTRAQ4plex)	HCD	1.0000	0.0000	1	1	7564	Unique	Used	2.751	1.325E3	4.410E3	156	2.96	0.00	1	10	167	2	783.46857	1565.92986	2.25	19.64	1862	1862	MS2	15/18	Glioma_Nuclear_prep_Gradeall_repeat_4plex.raw
4504425	High		tYIPPkGETk	Unambiguous	1	1	N-Term(iTRAQ4plex); K6(iTRAQ4plex); K10(iTRAQ4plex)	HCD	1.0000	0.0000	1	1	7511	Unique	Used	1.525	6.612E4	1.230E5	143	3.29	0.00	1	18	8	2	783.46265	1565.91802	-5.31	22.90	1923	1923	MS2	14/18	Glioma_Nuclear_prep_Gradeall_4plex.raw
4506003	NP_002699.1	PPP1CA	5499	serine/threonine-protein phosphatase PP1-alpha catalytic subunit isoform 1 [Homo sapiens]	15.45	4	1	2	0.668	1;1	1.0	19.74	330	37.5	6.33																			
4506003		High	lNLDSIIGR	Unambiguous	3	1	N-Term(iTRAQ4plex)	HCD	1.0000	0.0000	1	1	11583	Unique	Used	0.673	4.316E3	3.514E3	221	3.21	0.00	0	55	300	2	572.84534	1144.68340	2.37	65.34	6230	6230	MS2	14/16	Glioma_Nuclear_prep_Gradeall_repeat_4plex.raw
4506003	High		lNLDSIIGR	Unambiguous	3	1	N-Term(iTRAQ4plex)	HCD	1.0000	0.0000	1	1	10796	Unique	Used	0.664	6.844E4	5.543E4	270	2.98	0.00	0	51	29	2	572.83490	1144.66252	-15.87	46.88	5499	5499	MS2	15/16	Glioma_Nuclear_prep_Gradeall_4plex.raw

4506613	NP_000974.1	RPL22	6146	60S ribosomal protein L22 proprotein [Homo sapiens]	10.16	1	1	2	0.611	1;1	14.2	4.38	128	14.8	9.19																			
4506613		High	aGNLGGGVVTIER	Unambiguous	1	1	N-Term(iTRAQ4plex)	HCD	1.0000	0.0000	1	1	9206	Unique	Used	0.672	8.597E3	6.991E3	238	4.38	0.00	0	39	300	2	693.89624	1386.78520	2.16	36.03	3600	3600	MS2	23/24	Glioma_Nuclear_prep_Gradeall_repeat_4plex.raw
4506613	High		aGNLGGGVVTIER	Unambiguous	1	1	N-Term(iTRAQ4plex)	HCD	1.0000	0.0000	1	1	8780	Unique	Used	0.549	7.088E4	4.749E4	238	4.12	0.00	0	23	23	2	693.88306	1386.75884	-16.86	31.94	3300	3300	MS2	21/24	Glioma_Nuclear_prep_Gradeall_4plex.raw
4506661	NP_000963.1	RPL7A	6130	60S ribosomal protein L7a [Homo sapiens]	7.14	1	2	3	0.642	2;1	8.6	7.14	266	30.0	10.61																			
4506661		High	nFGIGQDIQPk	Unambiguous	1	1	N-Term(iTRAQ4plex); K11(iTRAQ4plex)	HCD	1.0000	0.0000	1	1	9540	Unique	Used	0.591	6.314E3	4.517E3	296	3.98	0.00	0	23	300	2	752.91888	1504.83049	-3.82	39.73	3958	3958	MS2	19/20	Glioma_Nuclear_prep_Gradeall_repeat_4plex.raw
4506661	High		nFGIGQDIQPk	Unambiguous	1	1	N-Term(iTRAQ4plex); K11(iTRAQ4plex)	HCD	1.0000	0.0000	1	1	9019	Unique	Used	0.642	1.151E5	9.018E4	312	3.76	0.00	0	39	28	2	752.91046	1504.81365	-15.02	33.65	3559	3559	MS2	19/20	Glioma_Nuclear_prep_Gradeall_4plex.raw
4506661	High		vVNPLFEk	Unambiguous	1	1	N-Term(iTRAQ4plex); K8(iTRAQ4plex)	HCD	1.0000	0.0000	1	1	9454	Unique	Used	0.702	1.099E5	9.418E4	380	3.37	0.00	0	88	29	2	617.36597	1233.72466	-16.14	36.91	4037	4037	MS2	13/14	Glioma_Nuclear_prep_Gradeall_4plex.raw
4506671	NP_000995.1	RPLP2	6181	60S acidic ribosomal protein P2 [Homo sapiens]	26.96	1	2	3	0.673	2;1	17.9	15.37	115	11.7	4.54																			
4506671		High	nIEDVIAQGIGk	Unambiguous	1	1	N-Term(iTRAQ4plex); K12(iTRAQ4plex)	HCD	1.0000	0.0000	1	1	11701	Unique	Used	0.673	3.187E3	2.596E3	221	4.36	0.00	0	48	300	2	772.94995	1544.89263	2.55	66.99	6370	6370	MS2	21/22	Glioma_Nuclear_prep_Gradeall_repeat_4plex.raw
4506671	High		nIEDVIAQGIGk	Unambiguous	1	1	N-Term(iTRAQ4plex); K12(iTRAQ4plex)	HCD	1.0000	0.0000	1	1	10887	Unique	Used	0.516	3.787E4	2.385E4	219	4.35	0.00	0	58	65	2	772.93610	1544.86492	-15.39	47.58	5598	5598	MS2	20/22	Glioma_Nuclear_prep_Gradeall_4plex.raw
4506671	High		yVASYLLAALGGNSSPSAk	Unambiguous	1	1	N-Term(iTRAQ4plex); K19(iTRAQ4plex)	HCD	1.0000	0.0000	1	1	12649	Unique	Used	0.742	1.266E3	1.146E3	155	6.31	0.00	0	30	157	3	719.73340	2157.18564	2.87	64.38	7541	7541	MS2	28/72	Glioma_Nuclear_prep_Gradeall_4plex.raw
4506675	NP_002941.1	RPN1	6184	dolichyl-diphosphooligosaccharide--protein glycosyltransferase subunit 1 precursor [Homo sapiens]	20.43	1	8	14	1.594	7;7	13.8	47.77	607	68.5	6.38																			
4506675		High	tILPAAAQDVYYR	Unambiguous	1	1	N-Term(iTRAQ4plex)	HCD	1.0000	0.0000	1	1	10851	Unique	Used	1.746	2.766E3	5.842E3	242	3.87	0.00	0	42	300	2	812.94507	1624.88286	0.80	55.32	5369	5369	MS2	16/24	Glioma_Nuclear_prep_Gradeall_repeat_4plex.raw
4506675		High	sEDLLDYGPFR	Unambiguous	1	1	N-Term(iTRAQ4plex)	HCD	1.0000	0.0000	1	1	11756	Unique	Used	1.691	3.209E3	6.564E3	139	3.86	0.00	0	28	300	2	728.36627	1455.72527	1.09	67.82	6440	6440	MS2	17/20	Glioma_Nuclear_prep_Gradeall_repeat_4plex.raw
4506675		High	gEDEEENNLEVR	Unambiguous	1	1	N-Term(iTRAQ4plex)	HCD	1.0000	0.0000	1	1	7848	Unique	Used	1.578	4.452E3	8.501E3	66	4.83	0.00	0	6	217	2	788.86206	1576.71684	-2.51	22.33	2166	2166	MS2	22/22	Glioma_Nuclear_prep_Gradeall_repeat_4plex.raw
4506675		High	nIEIDSPYEISR	Unambiguous	1	1	N-Term(iTRAQ4plex)	HCD	1.0000	0.0000	1	1	10211	Unique	Used	1.510	9.207E3	1.682E4	213	4.12	0.00	0	7	193	2	790.40924	1579.81120	1.73	47.60	4680	4680	MS2	19/22	Glioma_Nuclear_prep_Gradeall_repeat_4plex.raw
4506675		High	vTAEVVLAHLGGGSTSR	Unambiguous	1	1	N-Term(iTRAQ4plex)	HCD	1.0000	0.0000	1	1	11132	Unique	Used	1.491	5.540E2	9.993E2	186	5.51	0.00	0	34	300	3	600.00220	1797.99204	-1.09	58.89	5683	5683	MS2	26/64	Glioma_Nuclear_prep_Gradeall_repeat_4plex.raw
4506675	High		vTAEVVLAHLGGGSTSR	Unambiguous	1	1	N-Term(iTRAQ4plex)	HCD	1.0000	0.0000	1	1	10142	Unique	Used	2.249	7.694E3	2.112E4	188	3.80	0.00	0	19	10	3	599.99390	1797.96714	-14.94	41.92	4785	4785	MS2	25/64	Glioma_Nuclear_prep_Gradeall_4plex.raw
4506675	High		aTSFLLALEPELEAR	Unambiguous	1	1	N-Term(iTRAQ4plex)	HCD	1.0000	0.0000	1	1	12697	Unique	Used	1.694	1.545E4	3.193E4	216	6.25	0.00	0	6	80	2	902.50476	1804.00224	2.71	64.98	7595	7595	MS2	22/28	Glioma_Nuclear_prep_Gradeall_4plex.raw
4506675	High		nIEIDSPYEISR	Unambiguous	1	1	N-Term(iTRAQ4plex)	HCD	1.0000	0.0000	1	1	9641	Unique	Used	1.555	1.187E5	2.253E5	151	3.17	0.00	0	11	11	2	790.39581	1579.78435	-15.26	38.26	4240	4240	MS2	18/22	Glioma_Nuclear_prep_Gradeall_4plex.raw
4506675	High		sEDLLDYGPFR	Unambiguous	1	1	N-Term(iTRAQ4plex)	HCD	1.0000	0.0000	1	1	10945	Unique	Used	1.521	2.873E4	5.331E4	110	3.40	0.00	0	44	55	2	728.35559	1455.70391	-13.58	48.02	5661	5661	MS2	17/20	Glioma_Nuclear_prep_Gradeall_4plex.raw
4506675	High		sEDLLDYGPFR	Unambiguous	1	1	N-Term(iTRAQ4plex)	HCD	1.0000	0.0000	1	1	11020	Unique	Used	1.416	6.370E4	1.101E5	148	3.33	0.00	0	23	18	2	728.36469	1455.72209	-1.09	48.59	5742	5742	MS2	17/20	Glioma_Nuclear_prep_Gradeall_4plex.raw
4506675	High		tILPAAAQDVYYR	Unambiguous	1	1	N-Term(iTRAQ4plex)	HCD	1.0000	0.0000	1	1	10105	Unique	Used	1.307	3.862E4	6.158E4	259	3.53	0.00	0	41	32	2	812.93207	1624.85686	-15.20	41.62	4744	4744	MS2	17/24	Glioma_Nuclear_prep_Gradeall_4plex.raw
4506675		High	dVPAYSQDTFk	Unambiguous	1	1	N-Term(iTRAQ4plex); K11(iTRAQ4plex)	HCD	1.0000	0.0000	1	1	9169	Unique	Used	1.830	4.610E3	1.021E4	193	3.68	0.00	0	36	300	2	779.90497	1558.80266	2.23	35.63	3561	3561	MS2	19/20	Glioma_Nuclear_prep_Gradeall_repeat_4plex.raw
4506675	High		dVPAYSQDTFk	Unambiguous	1	1	N-Term(iTRAQ4plex); K11(iTRAQ4plex)	HCD	1.0000	0.0000	1	1	8789	Unique	Used	1.755	7.039E4	1.507E5	133	3.79	0.00	0	5	16	2	779.89026	1558.77324	-16.64	32.01	3310	3310	MS2	17/20	Glioma_Nuclear_prep_Gradeall_4plex.raw
4506675		High	lAHLGVQVk	Unambiguous	1	1	N-Term(iTRAQ4plex); K9(iTRAQ4plex)	HCD	1.0000	0.0000	1	1	8640	Unique	Used	1.610	2.613E3	5.089E3	58	4.78	0.00	0	41	295	3	418.27118	1252.79898	0.79	30.05	3000	3000	MS2	20/32	Glioma_Nuclear_prep_Gradeall_repeat_4plex.raw
4506695	NP_001013.1	RPS19	6223	40S ribosomal protein S19 [Homo sapiens]	11.03	1	2	2	0.501	1;1	15.2	3.69	145	16.1	10.32																			
4506695		High	aLAAFLk	Unambiguous	1	1	N-Term(iTRAQ4plex); K7(iTRAQ4plex)	HCD	1.0000	0.0000	1	1	10765	Unique	Used	0.447	1.520E4	8.224E3	166	3.18	0.00	0	61	300	2	511.33707	1021.66686	1.96	54.23	5274	5274	MS2	12/12	Glioma_Nuclear_prep_Gradeall_repeat_4plex.raw
4506695	High		vLQALEGLk	Unambiguous	1	1	N-Term(iTRAQ4plex); K9(iTRAQ4plex)	HCD	1.0000	0.0000	1	1	10941	Unique	Used	0.555	2.107E5	1.427E5	157	3.69	0.00	0	66	19	2	629.89539	1258.78349	-11.00	48.00	5657	5657	MS2	16/16	Glioma_Nuclear_prep_Gradeall_4plex.raw
4506707	NP_001019.1	RPS25	6230	40S ribosomal protein S25 [Homo sapiens]	15.20	1	2	3	0.647	1;2	10.3	6.86	125	13.7	10.11																			
4506707		High	lITPAVVSER	Unambiguous	1	1	N-Term(iTRAQ4plex)	HCD	1.0000	0.0000	1	1	9740	Unique	Used	0.614	1.887E4	1.402E4	164	2.95	0.00	0	47	300	2	614.87451	1228.74175	2.88	41.99	4172	4172	MS2	12/18	Glioma_Nuclear_prep_Gradeall_repeat_4plex.raw
4506707		High	aALQELLSk	Unambiguous	1	1	N-Term(iTRAQ4plex); K9(iTRAQ4plex)	HCD	1.0000	0.0000	1	1	11025	Unique	Used	0.647	7.029E3	5.506E3	289	3.91	0.00	0	41	300	2	630.89148	1260.77568	-0.73	57.48	5560	5560	MS2	16/16	Glioma_Nuclear_prep_Gradeall_repeat_4plex.raw
4506707	High		aALQELLSk	Unambiguous	1	1	N-Term(iTRAQ4plex); K9(iTRAQ4plex)	HCD	1.0000	0.0000	1	1	10155	Unique	Used	0.747	1.134E5	1.034E5	287	3.47	0.00	0	51	21	2	630.88306	1260.75884	-14.09	42.01	4799	4799	MS2	16/16	Glioma_Nuclear_prep_Gradeall_4plex.raw
4507129	NP_003085.1	SNRPE	6635	small nuclear ribonucleoprotein E isoform 1 [Homo sapiens]	11.96	1	1	3	1.505	2;1	8.1	10.77	92	10.8	9.44																			
4507129	High		vMVQPINLIFR	Unambiguous	1	1	N-Term(iTRAQ4plex)	HCD	1.0000	0.0000	1	1	12152	Unique	Used	1.505	8.093E4	1.486E5	160	3.69	0.00	0	18	59	2	737.44202	1473.87676	2.36	58.50	6979	6979	MS2	16/20	Glioma_Nuclear_prep_Gradeall_4plex.raw
4507129		High	vmVQPINLIFR	Unambiguous	1	1	N-Term(iTRAQ4plex); M2(Oxidation)	HCD	1.0000	0.0000	1	1	12212	Unique	Used	1.315	4.851E3	7.721E3	222	3.38	0.00	0	22	300	2	745.43866	1489.87004	1.24	76.49	7127	7127	MS2	16/20	Glioma_Nuclear_prep_Gradeall_repeat_4plex.raw
4507129	High		vmVQPINLIFR	Unambiguous	1	1	N-Term(iTRAQ4plex); M2(Oxidation)	HCD	1.0000	0.0000	1	1	11544	Unique	Used	1.531	1.073E5	2.005E5	218	3.53	0.00	0	25	16	2	745.43939	1489.87151	2.23	52.97	6316	6316	MS2	16/20	Glioma_Nuclear_prep_Gradeall_4plex.raw
4507401	NP_003192.1	TFAM	7019	transcription factor A, mitochondrial isoform 1 precursor [Homo sapiens]	19.92	2	5	6	1.860	3;3	20.1	19.34	246	29.1	9.72																			
4507401		High	sAYNVYVAER	Unambiguous	1	1	N-Term(iTRAQ4plex)	HCD	1.0000	0.0000	1	1	8751	Unique	Used	1.838	1.176E4	2.615E4	121	3.30	0.00	0	8	166	2	658.33234	1315.65740	-14.37	31.07	3117	3117	MS2	17/18	Glioma_Nuclear_prep_Gradeall_repeat_4plex.raw
4507401	High		eQLTPSQIMSLEk	Unambiguous	2	1	N-Term(iTRAQ4plex); K13(iTRAQ4plex)	HCD	1.0000	0.0000	1	1	10147	Unique	Used	1.881	2.882E4	6.615E4	241	4.40	0.00	0	10	32	2	896.47809	1791.94890	-15.41	41.95	4790	4790	MS2	19/24	Glioma_Nuclear_prep_Gradeall_4plex.raw
4507401		High	eQLPIFk	Unambiguous	2	1	N-Term(iTRAQ4plex); K7(iTRAQ4plex)	HCD	1.0000	0.0000	1	1	10190	Unique	Used	2.481	1.584E4	4.757E4	236	3.08	0.00	0	14	181	2	581.85742	1162.70757	0.10	47.36	4658	4658	MS2	12/12	Glioma_Nuclear_prep_Gradeall_repeat_4plex.raw
4507401	High		eLTLLGkPk	Unambiguous	1	1	N-Term(iTRAQ4plex); K7(iTRAQ4plex); K9(iTRAQ4plex)	HCD	1.0000	0.0000	1	1	8473	Unique	Used	1.891	9.285E4	2.142E5	58	2.90	0.00	0	30	13	2	715.95557	1430.90386	-18.76	29.70	2965	2965	MS2	15/16	Glioma_Nuclear_prep_Gradeall_4plex.raw
4507401		High	eLYIQHAk	Unambiguous	2	1	N-Term(iTRAQ4plex); K8(iTRAQ4plex)	HCD	1.0000	0.0000	1	1	7820	Unique	Used	1.337	1.023E4	1.656E4	332	3.66	0.00	0	53	300	2	645.37793	1289.74858	2.31	22.04	2136	2136	MS2	14/14	Glioma_Nuclear_prep_Gradeall_repeat_4plex.raw
4507401	High		eLYIQHAk	Unambiguous	2	1	N-Term(iTRAQ4plex); K8(iTRAQ4plex)	HCD	1.0000	0.0000	1	1	7645	Unique	Used	1.676	9.887E4	2.022E5	332	3.40	0.00	0	20	15	2	645.37421	1289.74114	-3.47	23.85	2068	2068	MS2	14/14	Glioma_Nuclear_prep_Gradeall_4plex.raw
4507879	NP_003365.1	VDAC1	7416	voltage-dependent anion-selective channel protein 1 [Homo sapiens]	8.13	1	2	3	0.626	2;1	4.6	8.89	283	30.8	8.54																			
4507879		High	lTLSALLDGk	Unambiguous	1	1	N-Term(iTRAQ4plex); K10(iTRAQ4plex)	HCD	1.0000	0.0000	1	1	12193	Unique	Used	0.626	2.321E3	1.758E3	223	3.96	0.00	0	40	300	2	659.91144	1318.81560	-2.17	75.94	7086	7086	MS2	17/18	Glioma_Nuclear_prep_Gradeall_repeat_4plex.raw
4507879	High		lTLSALLDGk	Unambiguous	1	1	N-Term(iTRAQ4plex); K10(iTRAQ4plex)	HCD	1.0000	0.0000	1	1	11509	Unique	Used	0.671	5.850E4	4.787E4	179	4.38	0.00	0	50	43	2	659.90796	1318.80864	-7.45	52.68	6278	6278	MS2	17/18	Glioma_Nuclear_prep_Gradeall_4plex.raw
4507879	High		lTFDSSFSPNTGk	Unambiguous	1	1	N-Term(iTRAQ4plex); K13(iTRAQ4plex)	HCD	1.0000	0.0000	1	1	9792	Unique	Used	0.615	5.811E4	4.361E4	214	4.50	0.00	0	57	35	2	844.93134	1688.85539	-10.67	39.33	4403	4403	MS2	20/24	Glioma_Nuclear_prep_Gradeall_4plex.raw
4757810	NP_004037.1	ATP5A1	498	ATP synthase subunit alpha, mitochondrial isoform a precursor [Homo sapiens]	16.09	3	5	9	0.560	3;6	13.7	33.17	553	59.7	9.13																			
4757810		High	iLGADTSVDLEETGR	Unambiguous	3	1	N-Term(iTRAQ4plex)	HCD	1.0000	0.0000	1	1	10382	Unique	Used	0.512	3.047E3	1.886E3	257	4.90	0.00	0	31	300	2	860.44965	1719.89202	2.22	49.62	4862	4862	MS2	24/28	Glioma_Nuclear_prep_Gradeall_repeat_4plex.raw
4757810		High	tGAIVDVPVGEELLGR	Unambiguous	2	1	N-Term(iTRAQ4plex)	HCD	1.0000	0.0000	1	1	11910	Unique	Used	0.512	1.438E3	8.912E2	143	4.98	0.00	0	5	300	2	885.00183	1768.99639	2.13	70.36	6648	6648	MS2	23/30	Glioma_Nuclear_prep_Gradeall_repeat_4plex.raw
4757810		High	aVDSLVPIGR	Unambiguous	3	1	N-Term(iTRAQ4plex)	HCD	1.0000	0.0000	1	1	9924	Unique	Used	0.682	1.901E4	1.568E4	179	3.23	0.00	0	54	300	2	585.85199	1170.69670	0.31	44.10	4368	4368	MS2	13/18	Glioma_Nuclear_prep_Gradeall_repeat_4plex.raw
4757810		High	tGTAEMSSILEER	Unambiguous	2	1	N-Term(iTRAQ4plex)	HCD	1.0000	0.0000	1	1	10467	Unique	Used	0.701	2.474E3	2.099E3	162	4.01	0.00	0	43	300	2	784.39264	1567.77800	1.61	50.62	4952	4952	MS2	20/24	Glioma_Nuclear_prep_Gradeall_repeat_4plex.raw
4757810	High		tGAIVDVPVGEELLGR	Unambiguous	2	1	N-Term(iTRAQ4plex)	HCD	1.0000	0.0000	1	1	11153	Unique	Used	0.560	5.434E4	3.713E4	194	5.25	0.00	0	50	40	2	884.99713	1768.98699	-3.19	49.66	5888	5888	MS2	19/30	Glioma_Nuclear_prep_Gradeall_4plex.raw
4757810	High		iLGADTSVDLEETGR	Unambiguous	3	1	N-Term(iTRAQ4plex)	HCD	1.0000	0.0000	1	1	9858	Unique	Used	0.615	3.701E4	2.779E4	188	4.59	0.00	0	33	49	2	860.43420	1719.86113	-15.74	39.82	4475	4475	MS2	22/28	Glioma_Nuclear_prep_Gradeall_4plex.raw
4757810		High	vVDALGNAIDGk	Unambiguous	2	1	N-Term(iTRAQ4plex); K12(iTRAQ4plex)	HCD	1.0000	0.0000	1	1	10614	Unique	Used	0.591	6.393E3	4.570E3	291	4.34	0.00	0	61	300	2	730.42120	1459.83513	-0.55	52.40	5111	5111	MS2	21/22	Glioma_Nuclear_prep_Gradeall_repeat_4plex.raw
4757810	High		vVDALGNAIDGk	Unambiguous	2	1	N-Term(iTRAQ4plex); K12(iTRAQ4plex)	HCD	1.0000	0.0000	1	1	9958	Unique	Used	0.513	2.756E5	1.726E5	218	4.91	0.00	0	45	13	2	730.40875	1459.81023	-17.60	40.55	4584	4584	MS2	21/22	Glioma_Nuclear_prep_Gradeall_4plex.raw
4757810		High	tGTAEmSSILEER	Unambiguous	2	1	N-Term(iTRAQ4plex); M6(Oxidation)	HCD	1.0000	0.0000	1	1	8543	Unique	Used	0.484	6.237E3	3.653E3	101	3.80	0.00	0	0	300	2	792.38135	1583.75542	-9.45	29.05	2898	2898	MS2	20/24	Glioma_Nuclear_prep_Gradeall_repeat_4plex.raw
4758138	NP_004387.1	DDX5	1655	probable ATP-dependent RNA helicase DDX5 [Homo sapiens]	25.08	6	5	9	0.597	5;4	15.2	57.08	614	69.1	8.92																			
4758138		High	lLQLVEDR	Unambiguous	1	1	N-Term(iTRAQ4plex)	HCD	1.0000	0.0000	1	1	11231	Unique	Used	0.543	3.162E4	2.078E4	268	3.30	0.00	0	5	152	2	565.33978	1129.67229	2.22	60.20	5797	5797	MS2	14/14	Glioma_Nuclear_prep_Gradeall_repeat_4plex.raw
4758138		High	tTYLVLDEADR	Unambiguous	1	1	N-Term(iTRAQ4plex)	HCD	1.0000	0.0000	1	1	10144	Unique	Used	0.704	1.267E4	1.079E4	260	4.32	0.00	0	27	300	2	720.38013	1439.75298	2.15	46.81	4609	4609	MS2	18/20	Glioma_Nuclear_prep_Gradeall_repeat_4plex.raw
4758138		High	qVSDLISVLR	Unambiguous	1	1	N-Term(iTRAQ4plex)	HCD	1.0000	0.0000	1	1	11777	Unique	Used	0.775	8.649E3	8.108E3	196	2.90	0.00	0	40	300	2	637.38483	1273.76238	2.13	68.17	6469	6469	MS2	14/18	Glioma_Nuclear_prep_Gradeall_repeat_4plex.raw
4758138	High		lLQLVEDR	Unambiguous	1	1	N-Term(iTRAQ4plex)	HCD	1.0000	0.0000	1	1	10349	Unique	Used	0.530	7.002E5	4.526E5	196	3.14	0.00	0	7	4	2	565.33069	1129.65410	-13.88	43.45	5010	5010	MS2	14/14	Glioma_Nuclear_prep_Gradeall_4plex.raw
4758138	High		tTYLVLDEADR	Unambiguous	1	1	N-Term(iTRAQ4plex)	HCD	1.0000	0.0000	1	1	9593	Unique	Used	0.746	3.629E5	3.303E5	169	2.98	0.00	0	23	7	2	720.36810	1439.72893	-14.55	37.92	4188	4188	MS2	16/20	Glioma_Nuclear_prep_Gradeall_4plex.raw
4758138	High		eLAQQVQQVAAEYcR	Unambiguous	1	1	N-Term(iTRAQ4plex); C14(Methylthio)	HCD	1.0000	0.0000	1	1	12581	Unique	Used	0.527	2.013E4	1.295E4	162	6.33	0.00	0	34	150	2	963.46948	1925.93169	-0.70	63.42	7459	7459	MS2	24/28	Glioma_Nuclear_prep_Gradeall_4plex.raw
4758138	High		eLAQQVQQVAAEYcR	Unambiguous	1	1	N-Term(iTRAQ4plex); C14(Methylthio)	HCD	1.0000	0.0000	1	1	12530	Unique	Used	0.594	1.088E4	7.886E3	172	6.13	0.00	0	13	300	2	963.47015	1925.93303	-0.01	62.75	7400	7400	MS2	24/28	Glioma_Nuclear_prep_Gradeall_4plex.raw
4758138		High	lIDFLEcGk	Unambiguous	1	1	N-Term(iTRAQ4plex); C7(Methylthio); K9(iTRAQ4plex)	HCD	1.0000	0.0000	1	1	12428	Unique	Used	0.699	3.346E3	2.829E3	197	3.36	0.00	0	42	300	2	686.36798	1371.72869	2.32	85.65	7774	7774	MS2	14/16	Glioma_Nuclear_prep_Gradeall_repeat_4plex.raw
4758138	High		lIDFLEcGk	Unambiguous	1	1	N-Term(iTRAQ4plex); C7(Methylthio); K9(iTRAQ4plex)	HCD	1.0000	0.0000	1	1	12296	Unique	Used	0.597	1.923E5	1.401E5	202	3.75	0.00	0	27	32	2	686.36743	1371.72759	1.52	60.00	7138	7138	MS2	14/16	Glioma_Nuclear_prep_Gradeall_4plex.raw
4758876	NP_004634.1	PABPN1	8106	polyadenylate-binding protein 2 [Homo sapiens]	9.15	4	3	3	0.617	1;2	3.8	11.38	306	32.7	5.06																			
4758876		High	tSLALDESLFR	Unambiguous	2	1	N-Term(iTRAQ4plex)	HCD	1.0000	0.0000	1	1	11514	Unique	Used	0.603	7.059E3	5.147E3	344	4.21	0.00	0	14	300	2	698.38568	1395.76409	2.88	64.32	6144	6144	MS2	17/20	Glioma_Nuclear_prep_Gradeall_repeat_4plex.raw
4758876		High	vTILcDk	Unambiguous	4	1	N-Term(iTRAQ4plex); C5(Methylthio); K7(iTRAQ4plex)	HCD	1.0000	0.0000	1	1	10299	Unique	Used	0.648	1.551E4	1.217E4	284	2.96	0.00	0	53	300	2	563.31512	1125.62297	-1.84	48.65	4774	4774	MS2	12/12	Glioma_Nuclear_prep_Gradeall_repeat_4plex.raw
4758876	High		gFAYIEFSDk	Unambiguous	2	1	N-Term(iTRAQ4plex); K10(iTRAQ4plex)	HCD	1.0000	0.0000	1	1	10297	Unique	Used	0.617	1.643E5	1.237E5	159	3.75	0.00	0	41	20	2	732.87317	1464.73906	-15.21	43.06	4953	4953	MS2	17/18	Glioma_Nuclear_prep_Gradeall_4plex.raw
4759160	NP_004166.1	SNRPD3	6634	small nuclear ribonucleoprotein Sm D3 [Homo sapiens]	31.75	1	2	8	0.667	6;2	9.2	29.83	126	13.9	10.32																			
4759160	High		vAQLEQVYIR	Unambiguous	1	1	N-Term(iTRAQ4plex)	HCD	1.0000	0.0000	1	1	9758	Unique	Used	0.777	2.980E5	2.825E5	349	3.89	0.00	0	28	8	2	681.88660	1362.76592	-14.89	39.10	4367	4367	MS2	17/18	Glioma_Nuclear_prep_Gradeall_4plex.raw
4759160		High	fLILPDMLk	Unambiguous	1	1	N-Term(iTRAQ4plex); K9(iTRAQ4plex)	HCD	1.0000	0.0000	1	1	12441	Unique	Used	0.638	1.611E3	1.244E3	204	3.17	0.00	0	17	300	2	689.42499	1377.84270	0.61	86.40	7826	7826	MS2	14/16	Glioma_Nuclear_prep_Gradeall_repeat_4plex.raw
4759160	High		fLILPDMLk	Unambiguous	1	1	N-Term(iTRAQ4plex); K9(iTRAQ4plex)	HCD	1.0000	0.0000	1	1	12304	Unique	Used	0.587	2.770E5	1.985E5	192	3.40	0.00	0	7	16	2	689.42462	1377.84197	0.08	60.09	7147	7147	MS2	15/16	Glioma_Nuclear_prep_Gradeall_4plex.raw
4759160	High		fLILPDMLk	Unambiguous	1	1	N-Term(iTRAQ4plex); K9(iTRAQ4plex)	HCD	1.0000	0.0000	1	1	12365	Unique	Used	0.628	2.899E4	2.222E4	200	3.58	0.00	0	29	166	2	689.42529	1377.84331	1.05	60.76	7214	7214	MS2	15/16	Glioma_Nuclear_prep_Gradeall_4plex.raw
4759160	High		fLILPDMLk	Unambiguous	1	1	N-Term(iTRAQ4plex); K9(iTRAQ4plex)	HCD	1.0000	0.0000	1	1	12279	Unique	Used	0.689	5.722E4	4.811E4	193	3.43	0.00	0	50	85	2	689.42456	1377.84184	-0.01	59.82	7119	7119	MS2	15/16	Glioma_Nuclear_prep_Gradeall_4plex.raw
4759160		High	fLILPDmLk	Unambiguous	1	1	N-Term(iTRAQ4plex); M7(Oxidation); K9(iTRAQ4plex)	HCD	1.0000	0.0000	1	1	12006	Unique	Used	0.743	3.635E3	3.267E3	212	2.94	0.00	0	29	300	2	697.42291	1393.83855	1.27	72.14	6790	6790	MS2	14/16	Glioma_Nuclear_prep_Gradeall_repeat_4plex.raw
4759160	High		fLILPDmLk	Unambiguous	1	1	N-Term(iTRAQ4plex); M7(Oxidation); K9(iTRAQ4plex)	HCD	1.0000	0.0000	1	1	11251	Unique	Used	0.656	6.841E4	5.475E4	210	2.99	0.00	0	52	63	2	697.42224	1393.83721	0.31	50.45	5995	5995	MS2	13/16	Glioma_Nuclear_prep_Gradeall_4plex.raw
4759160	High		fLILPDmLk	Unambiguous	1	1	N-Term(iTRAQ4plex); M7(Oxidation); K9(iTRAQ4plex)	HCD	1.0000	0.0000	1	1	11196	Unique	Used	0.677	7.140E4	5.899E4	257	3.06	0.00	0	27	36	2	697.41687	1393.82646	-7.40	49.99	5935	5935	MS2	15/16	Glioma_Nuclear_prep_Gradeall_4plex.raw
4759302	NP_004729.1	VAPB	9217	vesicle-associated membrane protein-associated protein B/C isoform 1 [Homo sapiens]	15.64	2	2	3	0.442	2;1	21.5	13.73	243	27.2	7.30																			
4759302		High	sLSSSLDDTEVk	Unambiguous	1	1	N-Term(iTRAQ4plex); K12(iTRAQ4plex)	HCD	1.0000	0.0000	1	1	9072	Unique	Used	0.434	1.130E4	5.928E3	257	5.03	0.00	0	20	275	2	784.91711	1568.82695	0.74	34.55	3459	3459	MS2	22/22	Glioma_Nuclear_prep_Gradeall_repeat_4plex.raw
4759302	High		sLSSSLDDTEVk	Unambiguous	1	1	N-Term(iTRAQ4plex); K12(iTRAQ4plex)	HCD	1.0000	0.0000	1	1	8685	Unique	Used	0.442	1.566E5	8.451E4	212	4.97	0.00	0	39	19	2	784.90491	1568.80254	-14.83	31.26	3197	3197	MS2	22/22	Glioma_Nuclear_prep_Gradeall_4plex.raw
4759302	High		vEQVLSLEPQHELk	Unambiguous	2	1	N-Term(iTRAQ4plex); K14(iTRAQ4plex)	HCD	1.0000	0.0000	1	1	9664	Unique	Used	0.624	8.615E4	6.555E4	144	3.98	0.00	0	52	9	3	646.36121	1937.06906	-13.21	38.43	4265	4265	MS2	24/52	Glioma_Nuclear_prep_Gradeall_4plex.raw
4885371	NP_005309.1	H1F0	3005	histone H1.0 [Homo sapiens]	24.23	1	5	35	2.249	17;18	27.3	89.21	194	20.9	10.84																			
4885371		High	gVGASGSFR	Unambiguous	1	1	N-Term(iTRAQ4plex)	HCD	1.0000	0.0000	1	1	7233	Unique	Used	2.519	6.077E4	1.852E5	162	3.18	0.00	0	20	32	2	491.25702	981.50676	-17.01	16.83	1515	1515	MS2	15/16	Glioma_Nuclear_prep_Gradeall_repeat_4plex.raw
4885371		High	gVGASGSFR	Unambiguous	1	1	N-Term(iTRAQ4plex)	HCD	1.0000	0.0000	1	1	7307	Unique	Used	2.417	1.445E5	4.226E5	186	3.36	0.00	0	6	10	2	491.26401	981.52074	-2.77	17.49	1593	1593	MS2	15/16	Glioma_Nuclear_prep_Gradeall_repeat_4plex.raw
4885371	High		gVGASGSFR	Unambiguous	1	1	N-Term(iTRAQ4plex)	HCD	1.0000	0.0000	1	1	7353	Unique	Used	2.700	6.485E5	2.136E6	153	3.36	0.00	0	13	2	2	491.26276	981.51824	-5.32	21.76	1752	1752	MS2	15/16	Glioma_Nuclear_prep_Gradeall_4plex.raw
4885371		High	kLAATPk	Unambiguous	1	1	N-Term(iTRAQ4plex); K1(iTRAQ4plex); K7(iTRAQ4plex)	HCD	1.0000	0.0000	1	1	6815	Unique	Used	2.294	6.820E4	1.893E5	52	3.82	0.00	1	19	6	3	387.59683	1160.77594	2.77	13.17	1073	1073	MS2	17/24	Glioma_Nuclear_prep_Gradeall_repeat_4plex.raw
4885371	High		kLAATPk	Unambiguous	1	1	N-Term(iTRAQ4plex); K1(iTRAQ4plex); K7(iTRAQ4plex)	HCD	1.0000	0.0000	1	1	7137	Unique	Used	1.957	2.821E4	6.733E4	63	3.87	0.00	1	35	15	3	387.59497	1160.77036	-2.04	20.05	1515	1515	MS2	17/24	Glioma_Nuclear_prep_Gradeall_4plex.raw
4885371		High	vGENADSQIk	Unambiguous	1	1	N-Term(iTRAQ4plex); K10(iTRAQ4plex)	HCD	1.0000	0.0000	1	1	7387	Unique	Used	2.822	1.113E5	3.801E5	203	3.35	0.00	0	4	12	2	674.85858	1348.70989	-15.73	18.14	1677	1677	MS2	18/18	Glioma_Nuclear_prep_Gradeall_repeat_4plex.raw
4885371		High	vGENADSQIk	Unambiguous	1	1	N-Term(iTRAQ4plex); K10(iTRAQ4plex)	HCD	1.0000	0.0000	1	1	7447	Unique	Used	2.405	1.664E5	4.841E5	270	4.01	0.00	0	6	8	2	674.86908	1348.73088	-0.17	18.66	1740	1740	MS2	18/18	Glioma_Nuclear_prep_Gradeall_repeat_4plex.raw
4885371		High	vGENADSQIk	Unambiguous	1	1	N-Term(iTRAQ4plex); K10(iTRAQ4plex)	HCD	1.0000	0.0000	1	1	7521	Unique	Used	1.703	1.496E4	3.081E4	246	3.90	0.00	0	34	103	2	674.86218	1348.71709	-10.39	19.28	1817	1817	MS2	17/18	Glioma_Nuclear_prep_Gradeall_repeat_4plex.raw
4885371	High		vGENADSQIk	Unambiguous	1	1	N-Term(iTRAQ4plex); K10(iTRAQ4plex)	HCD	1.0000	0.0000	1	1	7435	Unique	Used	2.627	3.857E5	1.236E6	229	4.30	0.00	0	6	3	2	674.86560	1348.72392	-5.33	22.36	1841	1841	MS2	18/18	Glioma_Nuclear_prep_Gradeall_4plex.raw
4885371		High	ySDMIVAAIQAEk	Unambiguous	1	1	N-Term(iTRAQ4plex); K13(iTRAQ4plex)	HCD	1.0000	0.0000	1	1	12204	Unique	Used	3.028	1.056E3	3.869E3	251	5.24	0.00	0	6	104	3	576.31494	1726.93027	0.83	76.17	7104	7104	MS2	24/48	Glioma_Nuclear_prep_Gradeall_repeat_4plex.raw
4885371		High	ySDMIVAAIQAEk	Unambiguous	1	1	N-Term(iTRAQ4plex); K13(iTRAQ4plex)	HCD	1.0000	0.0000	1	1	12270	Unique	Used	3.005	6.670E1	2.425E2	233	4.08	0.00	0	13	300	3	576.31390	1726.92716	-0.97	78.20	7254	7254	MS2	22/48	Glioma_Nuclear_prep_Gradeall_repeat_4plex.raw
4885371		High	ySDMIVAAIQAEk	Unambiguous	1	1	N-Term(iTRAQ4plex); K13(iTRAQ4plex)	HCD	1.0000	0.0000	1	1	12206	Unique	Used	2.632	3.854E3	1.227E4	223	5.57	0.00	0	7	300	2	863.96924	1726.93120	1.37	76.28	7112	7112	MS2	22/24	Glioma_Nuclear_prep_Gradeall_repeat_4plex.raw
4885371		High	ySDMIVAAIQAEk	Unambiguous	1	1	N-Term(iTRAQ4plex); K13(iTRAQ4plex)	HCD	1.0000	0.0000	1	1	12162	Unique	Used	2.203	9.800E2	2.611E3	255	5.03	0.00	0	15	300	2	863.96899	1726.93071	1.09	75.29	7035	7035	MS2	21/24	Glioma_Nuclear_prep_Gradeall_repeat_4plex.raw
4885371		High	ySDMIVAAIQAEk	Unambiguous	1	1	N-Term(iTRAQ4plex); K13(iTRAQ4plex)	HCD	1.0000	0.0000	1	1	12239	Unique	Used	1.951	2.588E2	6.107E2	243	5.17	0.00	0	10	300	3	576.31451	1726.92899	0.09	77.18	7180	7180	MS2	24/48	Glioma_Nuclear_prep_Gradeall_repeat_4plex.raw
4885371	High		ySDMIVAAIQAEk	Unambiguous	1	1	N-Term(iTRAQ4plex); K13(iTRAQ4plex)	HCD	1.0000	0.0000	1	1	11650	Unique	Used	3.826	1.123E5	5.240E5	240	5.58	0.00	0	7	1	3	576.31567	1726.93247	2.10	53.87	6431	6431	MS2	24/48	Glioma_Nuclear_prep_Gradeall_4plex.raw
4885371	High		ySDMIVAAIQAEk	Unambiguous	1	1	N-Term(iTRAQ4plex); K13(iTRAQ4plex)	HCD	1.0000	0.0000	1	1	11726	Unique	Used	3.804	8.059E3	3.740E4	235	5.14	0.00	0	7	7	3	576.31329	1726.92533	-2.03	54.57	6514	6514	MS2	24/48	Glioma_Nuclear_prep_Gradeall_4plex.raw
4885371	High		ySDMIVAAIQAEk	Unambiguous	1	1	N-Term(iTRAQ4plex); K13(iTRAQ4plex)	HCD	1.0000	0.0000	1	1	11651	Unique	Used	2.732	8.794E5	2.931E6	229	5.69	0.00	0	7	2	2	863.96979	1726.93230	2.00	53.87	6432	6432	MS2	21/24	Glioma_Nuclear_prep_Gradeall_4plex.raw
4885371	High		ySDMIVAAIQAEk	Unambiguous	1	1	N-Term(iTRAQ4plex); K13(iTRAQ4plex)	HCD	1.0000	0.0000	1	1	11569	Unique	Used	2.249	6.296E4	1.728E5	231	5.64	0.00	0	32	34	2	863.96802	1726.92876	-0.05	53.17	6343	6343	MS2	21/24	Glioma_Nuclear_prep_Gradeall_4plex.raw
4885371	High		ySDMIVAAIQAEk	Unambiguous	1	1	N-Term(iTRAQ4plex); K13(iTRAQ4plex)	HCD	1.0000	0.0000	1	1	11567	Unique	Used	2.161	7.483E3	1.973E4	232	5.24	0.00	0	26	14	3	576.31366	1726.92643	-1.40	53.16	6341	6341	MS2	24/48	Glioma_Nuclear_prep_Gradeall_4plex.raw
4885371	High		ySDMIVAAIQAEk	Unambiguous	1	1	N-Term(iTRAQ4plex); K13(iTRAQ4plex)	HCD	1.0000	0.0000	1	1	11727	Unique	Used	1.917	9.232E4	2.159E5	217	5.65	0.00	0	36	20	2	863.96637	1726.92546	-1.95	54.58	6515	6515	MS2	21/24	Glioma_Nuclear_prep_Gradeall_4plex.raw
4885371	High		ySDMIVAAIQAEk	Unambiguous	1	1	N-Term(iTRAQ4plex); K13(iTRAQ4plex)	HCD	1.0000	0.0000	1	1	11810	Unique	Used	1.907	3.457E3	8.044E3	228	5.04	0.00	0	11	30	3	576.31305	1726.92459	-2.46	55.29	6605	6605	MS2	24/48	Glioma_Nuclear_prep_Gradeall_4plex.raw
4885371	High		ySDMIVAAIQAEk	Unambiguous	1	1	N-Term(iTRAQ4plex); K13(iTRAQ4plex)	HCD	1.0000	0.0000	1	1	11814	Unique	Used	1.864	2.014E4	4.580E4	259	5.56	0.00	0	42	90	2	863.96844	1726.92961	0.45	55.32	6609	6609	MS2	21/24	Glioma_Nuclear_prep_Gradeall_4plex.raw
4885371	High		ySDMIVAAIQAEk	Unambiguous	1	1	N-Term(iTRAQ4plex); K13(iTRAQ4plex)	HCD	1.0000	0.0000	1	1	11919	Unique	Used	1.578	9.433E3	1.816E4	273	5.32	0.00	0	35	262	2	863.97235	1726.93743	4.97	56.20	6723	6723	MS2	21/24	Glioma_Nuclear_prep_Gradeall_4plex.raw
4885371	High		ySDMIVAAIQAEk	Unambiguous	1	1	N-Term(iTRAQ4plex); K13(iTRAQ4plex)	HCD	1.0000	0.0000	1	1	12303	Unique	Used	1.344	7.469E2	1.225E3	213	4.43	0.00	0	59	300	3	576.31287	1726.92405	-2.77	60.06	7145	7145	MS2	21/48	Glioma_Nuclear_prep_Gradeall_4plex.raw
4885371		High	lVTTGVLk	Unambiguous	1	1	N-Term(iTRAQ4plex); K8(iTRAQ4plex)	HCD	1.0000	0.0000	1	1	9171	Unique	Used	3.366	5.275E4	2.148E5	102	3.14	0.00	0	10	34	2	559.87085	1118.73442	-3.87	35.66	3564	3564	MS2	14/14	Glioma_Nuclear_prep_Gradeall_repeat_4plex.raw
4885371		High	lVTTGVLk	Unambiguous	1	1	N-Term(iTRAQ4plex); K8(iTRAQ4plex)	HCD	1.0000	0.0000	1	1	9263	Unique	Used	2.730	2.502E5	8.265E5	126	3.02	0.00	0	7	10	2	559.87469	1118.74211	3.01	36.67	3661	3661	MS2	14/14	Glioma_Nuclear_prep_Gradeall_repeat_4plex.raw
4885371		High	lVTTGVLk	Unambiguous	1	1	N-Term(iTRAQ4plex); K8(iTRAQ4plex)	HCD	1.0000	0.0000	1	1	9196	Unique	Used	2.163	2.412E3	6.312E3	70	3.47	0.00	0	26	253	3	373.58450	1118.73896	0.19	35.92	3590	3590	MS2	14/28	Glioma_Nuclear_prep_Gradeall_repeat_4plex.raw
4885371		High	ySDmIVAAIQAEk	Unambiguous	1	1	N-Term(iTRAQ4plex); M4(Oxidation); K13(iTRAQ4plex)	HCD	1.0000	0.0000	1	1	11394	Unique	Used	3.858	1.369E3	6.389E3	229	5.08	0.00	0	5	54	3	581.64703	1742.92655	1.60	62.71	6005	6005	MS2	25/48	Glioma_Nuclear_prep_Gradeall_repeat_4plex.raw
4885371		High	ySDmIVAAIQAEk	Unambiguous	1	1	N-Term(iTRAQ4plex); M4(Oxidation); K13(iTRAQ4plex)	HCD	1.0000	0.0000	1	1	11329	Unique	Used	2.608	2.250E3	7.099E3	209	5.23	0.00	0	11	300	2	871.96704	1742.92681	1.75	61.83	5929	5929	MS2	21/24	Glioma_Nuclear_prep_Gradeall_repeat_4plex.raw
4885371		High	ySDmIVAAIQAEk	Unambiguous	1	1	N-Term(iTRAQ4plex); M4(Oxidation); K13(iTRAQ4plex)	HCD	1.0000	0.0000	1	1	11405	Unique	Used	2.026	3.890E3	9.534E3	217	4.88	0.00	0	5	254	2	871.96661	1742.92595	1.26	62.87	6019	6019	MS2	20/24	Glioma_Nuclear_prep_Gradeall_repeat_4plex.raw
4885371		High	ySDmIVAAIQAEk	Unambiguous	1	1	N-Term(iTRAQ4plex); M4(Oxidation); K13(iTRAQ4plex)	HCD	1.0000	0.0000	1	1	11767	Unique	Used	1.917	2.891E2	6.705E2	167	4.06	0.00	0	29	300	3	581.64435	1742.91849	-3.02	68.01	6455	6455	MS2	21/48	Glioma_Nuclear_prep_Gradeall_repeat_4plex.raw
4885371	High		ySDmIVAAIQAEk	Unambiguous	1	1	N-Term(iTRAQ4plex); M4(Oxidation); K13(iTRAQ4plex)	HCD	1.0000	0.0000	1	1	10569	Unique	Used	2.162	2.705E5	7.134E5	197	4.54	0.00	0	7	4	2	871.95294	1742.89861	-14.43	45.16	5252	5252	MS2	22/24	Glioma_Nuclear_prep_Gradeall_4plex.raw
4885371	High		ySDmIVAAIQAEk	Unambiguous	1	1	N-Term(iTRAQ4plex); M4(Oxidation); K13(iTRAQ4plex)	HCD	1.0000	0.0000	1	1	10662	Unique	Used	1.852	1.136E5	2.567E5	187	4.31	0.00	0	16	9	2	871.95343	1742.89958	-13.87	45.87	5354	5354	MS2	20/24	Glioma_Nuclear_prep_Gradeall_4plex.raw
4885371	High		ySDmIVAAIQAEk	Unambiguous	1	1	N-Term(iTRAQ4plex); M4(Oxidation); K13(iTRAQ4plex)	HCD	1.0000	0.0000	1	1	10652	Unique	Used	1.579	1.744E4	3.358E4	212	3.68	0.00	0	11	3	3	581.63721	1742.89707	-15.31	45.80	5343	5343	MS2	25/48	Glioma_Nuclear_prep_Gradeall_4plex.raw
4885371	High		ySDmIVAAIQAEk	Unambiguous	1	1	N-Term(iTRAQ4plex); M4(Oxidation); K13(iTRAQ4plex)	HCD	1.0000	0.0000	1	1	10801	Unique	Used	1.438	4.538E4	7.963E4	247	4.38	0.00	0	42	43	2	871.95477	1742.90227	-12.33	46.93	5505	5505	MS2	19/24	Glioma_Nuclear_prep_Gradeall_4plex.raw
4885373	NP_005316.1	HIST1H1A	3024	histone H1.1 [Homo sapiens]	32.09	2	3	4	3.136	2;2	38.4	69.48	215	21.8	10.99																			
4885373		High	kPAGPSVSELIVQAASSSk	Unambiguous	1	1	N-Term(iTRAQ4plex); K1(iTRAQ4plex); K19(iTRAQ4plex)	HCD	1.0000	0.0000	1	1	11305	Unique	Used	1.669	2.629E2	5.308E2	68	5.94	0.00	0	29	300	3	763.44598	2288.32340	2.07	61.35	5892	5892	MS2	27/72	Glioma_Nuclear_prep_Gradeall_repeat_4plex.raw
4885373		High	kkPAGPSVSELIVQAASSSk	Unambiguous	1	1	N-Term(iTRAQ4plex); K1(iTRAQ4plex); K2(iTRAQ4plex); K20(iTRAQ4plex)	HCD	1.0000	0.0000	1	1	10833	Unique	Used	3.528	2.391E2	1.020E3	13	7.14	0.00	1	11	220	3	854.17883	2560.52195	2.45	55.11	5350	5350	MS2	24/76	Glioma_Nuclear_prep_Gradeall_repeat_4plex.raw
4885373	High		kkPAGPSVSELIVQAASSSk	Unambiguous	1	1	N-Term(iTRAQ4plex); K1(iTRAQ4plex); K2(iTRAQ4plex); K20(iTRAQ4plex)	HCD	1.0000	0.0000	1	1	9711	Unique	Used	2.744	1.501E4	5.026E4	35	5.83	0.00	1	11	3	3	854.16431	2560.47837	-14.57	38.77	4316	4316	MS2	24/76	Glioma_Nuclear_prep_Gradeall_4plex.raw
4885373	High		aSSVETkPGASk	Unambiguous	1	1	N-Term(iTRAQ4plex); K7(iTRAQ4plex); K12(iTRAQ4plex)	HCD	1.0000	0.0000	1	1	7156	Unique	Used	4.482	2.178E3	1.191E4	190	4.30	0.00	0	12	168	2	797.46027	1593.91326	-2.50	20.20	1535	1535	MS2	18/22	Glioma_Nuclear_prep_Gradeall_4plex.raw

4885375	NP_005310.1	HIST1H1C	3006	histone H1.2 [Homo sapiens]	36.62	2	3	10	4.044	5;5	39.9	188.38	213	21.4	10.93																			
4885375		High	kPAAATVTk	Unambiguous	1	1	N-Term(iTRAQ4plex); K1(iTRAQ4plex); K9(iTRAQ4plex)	HCD	1.0000	0.0000	1	1	6868	Unique	Used	5.269	3.128E4	1.994E5	115	2.94	0.00	0	34	157	2	659.92169	1318.83611	-4.38	13.59	1128	1128	MS2	14/16	Glioma_Nuclear_prep_Gradeall_repeat_4plex.raw
4885375		High	kPAAATVTk	Unambiguous	1	1	N-Term(iTRAQ4plex); K1(iTRAQ4plex); K9(iTRAQ4plex)	HCD	1.0000	0.0000	1	1	6860	Unique	Used	4.294	1.191E4	6.187E4	124	4.62	0.00	0	39	39	3	440.28333	1318.83542	-4.90	13.53	1120	1120	MS2	23/32	Glioma_Nuclear_prep_Gradeall_repeat_4plex.raw
4885375		High	kAAGGATPk	Unambiguous	1	1	N-Term(iTRAQ4plex); K1(iTRAQ4plex); K9(iTRAQ4plex)	HCD	1.0000	0.0000	1	1	6415	Unique	Used	2.869	3.698E3	1.283E4	188	4.45	0.00	1	28	58	3	411.59473	1232.76963	0.74	9.38	651	651	MS2	27/32	Glioma_Nuclear_prep_Gradeall_repeat_4plex.raw
4885375		High	kPAAATVTk	Unambiguous	1	1	N-Term(iTRAQ4plex); K1(iTRAQ4plex); K9(iTRAQ4plex)	HCD	1.0000	0.0000	1	1	7032	Unique	Used	1.451	6.980E3	1.225E4	124	4.50	0.00	0	56	112	3	440.28391	1318.83716	-3.58	15.08	1301	1301	MS2	22/32	Glioma_Nuclear_prep_Gradeall_repeat_4plex.raw
4885375	High		kPAAATVTk	Unambiguous	1	1	N-Term(iTRAQ4plex); K1(iTRAQ4plex); K9(iTRAQ4plex)	HCD	1.0000	0.0000	1	1	7161	Unique	Used	5.985	1.717E4	1.254E5	170	4.43	0.00	0	14	10	3	440.27896	1318.82233	-14.82	20.25	1541	1541	MS2	23/32	Glioma_Nuclear_prep_Gradeall_4plex.raw
4885375	High		kPAAATVTk	Unambiguous	1	1	N-Term(iTRAQ4plex); K1(iTRAQ4plex); K9(iTRAQ4plex)	HCD	1.0000	0.0000	1	1	7163	Unique	Used	5.224	4.943E4	3.151E5	157	3.11	0.00	0	15	31	2	659.91516	1318.82305	-14.28	20.27	1543	1543	MS2	14/16	Glioma_Nuclear_prep_Gradeall_4plex.raw
4885375	High		kPAAATVTk	Unambiguous	1	1	N-Term(iTRAQ4plex); K1(iTRAQ4plex); K9(iTRAQ4plex)	HCD	1.0000	0.0000	1	1	7200	Unique	Used	3.945	9.019E4	4.340E5	111	2.95	0.00	0	10	7	2	659.92352	1318.83977	-1.60	20.56	1583	1583	MS2	15/16	Glioma_Nuclear_prep_Gradeall_4plex.raw
4885375	High		kPAAATVTk	Unambiguous	1	1	N-Term(iTRAQ4plex); K1(iTRAQ4plex); K9(iTRAQ4plex)	HCD	1.0000	0.0000	1	1	7198	Unique	Used	3.162	5.368E4	2.071E5	117	4.61	0.00	0	11	3	3	440.28479	1318.83982	-1.56	20.55	1581	1581	MS2	23/32	Glioma_Nuclear_prep_Gradeall_4plex.raw
4885375	High		kAAGGATPk	Unambiguous	1	1	N-Term(iTRAQ4plex); K1(iTRAQ4plex); K9(iTRAQ4plex)	HCD	1.0000	0.0000	1	1	6736	Unique	Used	1.655	7.379E2	1.490E3	149	3.53	0.00	1	54	300	3	411.59396	1232.76734	-1.12	15.20	1061	1061	MS2	26/32	Glioma_Nuclear_prep_Gradeall_4plex.raw
4885375		High	aAGGATPk	Unambiguous	1	1	N-Term(iTRAQ4plex); K8(iTRAQ4plex)	HCD	1.0000	0.0000	1	1	6453	Unique	Used	4.144	1.625E4	8.149E4	228	2.93	0.00	0	7	91	2	480.78970	960.57213	0.46	9.77	691	691	MS2	13/14	Glioma_Nuclear_prep_Gradeall_repeat_4plex.raw
4885377	NP_005311.1	HIST1H1D	3007	histone H1.3 [Homo sapiens]	30.77	2	2	4	4.208	1;3	21.8	176.89	221	22.3	11.02																			
4885377		High	kVAGAATPk	Unambiguous	1	1	N-Term(iTRAQ4plex); K1(iTRAQ4plex); K9(iTRAQ4plex)	HCD	1.0000	0.0000	1	1	6679	Unique	Used	5.594	1.524E4	1.032E5	104	2.89	0.00	1	5	26	2	637.90979	1274.81230	-2.64	11.93	930	930	MS2	15/16	Glioma_Nuclear_prep_Gradeall_repeat_4plex.raw
4885377		High	kPATAAGTk	Unambiguous	1	1	N-Term(iTRAQ4plex); K1(iTRAQ4plex); K9(iTRAQ4plex)	HCD	1.0000	0.0000	1	1	6539	Unique	Used	4.300	1.041E4	5.415E4	186	3.49	0.00	0	10	14	3	426.27023	1276.79615	0.95	10.61	782	782	MS2	19/32	Glioma_Nuclear_prep_Gradeall_repeat_4plex.raw
4885377		High	kVAGAATPk	Unambiguous	1	1	N-Term(iTRAQ4plex); K1(iTRAQ4plex); K9(iTRAQ4plex)	HCD	1.0000	0.0000	1	1	6656	Unique	Used	4.116	4.418E3	2.200E4	103	4.72	0.00	1	26	42	3	425.60880	1274.81183	-3.01	11.72	906	906	MS2	22/32	Glioma_Nuclear_prep_Gradeall_repeat_4plex.raw
4885377	High		kVAGAATPk	Unambiguous	1	1	N-Term(iTRAQ4plex); K1(iTRAQ4plex); K9(iTRAQ4plex)	HCD	1.0000	0.0000	1	1	7019	Unique	Used	3.311	3.655E3	1.476E4	109	4.53	0.00	1	23	41	3	425.61038	1274.81659	0.72	18.75	1385	1385	MS2	23/32	Glioma_Nuclear_prep_Gradeall_4plex.raw
4885379	NP_005312.1	HIST1H1E	3008	histone H1.4 [Homo sapiens]	35.62	2	2	7	2.002	3;4	17.9	184.31	219	21.9	11.03																			
4885379		High	kPAAAAGAk	Unambiguous	1	1	N-Term(iTRAQ4plex); K1(iTRAQ4plex); K9(iTRAQ4plex)	HCD	1.0000	0.0000	1	1	6558	Unique	Used	2.630	1.387E5	4.414E5	157	2.95	0.00	0	4	18	2	608.88013	1216.75298	-17.12	10.76	802	802	MS2	15/16	Glioma_Nuclear_prep_Gradeall_repeat_4plex.raw
4885379		High	kPAAAAGAk	Unambiguous	1	1	N-Term(iTRAQ4plex); K1(iTRAQ4plex); K9(iTRAQ4plex)	HCD	1.0000	0.0000	1	1	6556	Unique	Used	2.522	8.903E4	2.717E5	173	4.13	0.00	0	11	7	3	406.25598	1216.75339	-16.78	10.75	800	800	MS2	22/32	Glioma_Nuclear_prep_Gradeall_repeat_4plex.raw
4885379		High	kPAAAAGAk	Unambiguous	1	1	N-Term(iTRAQ4plex); K1(iTRAQ4plex); K9(iTRAQ4plex)	HCD	1.0000	0.0000	1	1	6604	Unique	Used	1.835	5.772E4	1.281E5	150	4.11	0.00	0	8	11	3	406.26361	1216.77628	2.03	11.23	851	851	MS2	24/32	Glioma_Nuclear_prep_Gradeall_repeat_4plex.raw
4885379		High	kATGAATPk	Unambiguous	1	1	N-Term(iTRAQ4plex); K1(iTRAQ4plex); K9(iTRAQ4plex)	HCD	1.0000	0.0000	1	1	6471	Unique	Used	1.780	7.034E3	1.515E4	169	4.18	0.00	1	63	164	3	426.26987	1276.79505	0.09	9.95	710	710	MS2	20/32	Glioma_Nuclear_prep_Gradeall_repeat_4plex.raw
4885379	High		kPAAAAGAk	Unambiguous	1	1	N-Term(iTRAQ4plex); K1(iTRAQ4plex); K9(iTRAQ4plex)	HCD	1.0000	0.0000	1	1	6900	Unique	Used	2.240	3.815E3	1.042E4	156	3.96	0.00	0	44	157	3	406.26218	1216.77198	-1.51	17.38	1255	1255	MS2	21/32	Glioma_Nuclear_prep_Gradeall_4plex.raw
4885379	High		kPAAAAGAk	Unambiguous	1	1	N-Term(iTRAQ4plex); K1(iTRAQ4plex); K9(iTRAQ4plex)	HCD	1.0000	0.0000	1	1	6967	Unique	Used	2.002	1.002E4	2.449E4	141	2.90	0.00	0	20	154	2	608.89026	1216.77324	-0.47	18.14	1328	1328	MS2	15/16	Glioma_Nuclear_prep_Gradeall_4plex.raw
4885379	High		kPAAAAGAk	Unambiguous	1	1	N-Term(iTRAQ4plex); K1(iTRAQ4plex); K9(iTRAQ4plex)	HCD	1.0000	0.0000	1	1	6966	Unique	Used	1.671	5.317E3	1.084E4	155	4.10	0.00	0	24	91	3	406.26263	1216.77335	-0.38	18.14	1327	1327	MS2	23/32	Glioma_Nuclear_prep_Gradeall_4plex.raw
4885381	NP_005313.1	HIST1H1B	3009	histone H1.5 [Homo sapiens]	37.61	1	8	27	2.788	10;17	39.6	153.21	226	22.6	10.92																			
4885381		High	kALAAGGYDVEk	Unambiguous	1	1	N-Term(iTRAQ4plex); K1(iTRAQ4plex); K12(iTRAQ4plex)	HCD	1.0000	0.0000	1	1	8201	Unique	Used	3.949	4.562E3	2.179E4	198	4.31	0.00	1	15	57	2	827.48254	1653.95781	2.53	25.75	2538	2538	MS2	22/22	Glioma_Nuclear_prep_Gradeall_repeat_4plex.raw
4885381		High	kALAAGGYDVEk	Unambiguous	1	1	N-Term(iTRAQ4plex); K1(iTRAQ4plex); K12(iTRAQ4plex)	HCD	1.0000	0.0000	1	1	8183	Unique	Used	1.792	6.946E3	1.506E4	214	5.96	0.00	1	12	19	3	551.98767	1653.94846	-3.13	25.56	2519	2519	MS2	28/44	Glioma_Nuclear_prep_Gradeall_repeat_4plex.raw
4885381		High	kATGPPVSELITk	Unambiguous	1	1	N-Term(iTRAQ4plex); K1(iTRAQ4plex); K13(iTRAQ4plex)	HCD	1.0000	0.0000	1	1	9230	Unique	Used	4.538	3.850E2	2.113E3	52	5.17	0.00	1	6	89	4	444.02740	1773.08779	1.77	36.29	3626	3626	MS2	23/72	Glioma_Nuclear_prep_Gradeall_repeat_4plex.raw
4885381		High	kATGPPVSELITk	Unambiguous	1	1	N-Term(iTRAQ4plex); K1(iTRAQ4plex); K13(iTRAQ4plex)	HCD	1.0000	0.0000	1	1	9147	Unique	Used	2.769	1.221E3	4.092E3	76	4.67	0.00	1	79	300	2	887.04419	1773.08110	-2.00	35.39	3538	3538	MS2	20/24	Glioma_Nuclear_prep_Gradeall_repeat_4plex.raw
4885381		High	kATGPPVSELITk	Unambiguous	1	1	N-Term(iTRAQ4plex); K1(iTRAQ4plex); K13(iTRAQ4plex)	HCD	1.0000	0.0000	1	1	8610	Unique	Used	1.959	1.495E3	3.544E3	49	4.61	0.00	1	43	140	3	591.70020	1773.08603	0.78	29.74	2969	2969	MS2	18/48	Glioma_Nuclear_prep_Gradeall_repeat_4plex.raw
4885381		High	kATGPPVSELITk	Unambiguous	1	1	N-Term(iTRAQ4plex); K1(iTRAQ4plex); K13(iTRAQ4plex)	HCD	1.0000	0.0000	1	1	9341	Unique	Used	1.838	2.721E3	6.051E3	48	5.31	0.00	1	24	63	3	591.69971	1773.08457	-0.05	37.54	3743	3743	MS2	24/48	Glioma_Nuclear_prep_Gradeall_repeat_4plex.raw
4885381		High	kATGPPVSELITk	Unambiguous	1	1	N-Term(iTRAQ4plex); K1(iTRAQ4plex); K13(iTRAQ4plex)	HCD	1.0000	0.0000	1	1	9154	Unique	Used	1.475	2.811E3	5.016E3	53	6.09	0.00	1	31	191	3	591.69934	1773.08347	-0.67	35.47	3546	3546	MS2	22/48	Glioma_Nuclear_prep_Gradeall_repeat_4plex.raw
4885381	High		kATGPPVSELITk	Unambiguous	1	1	N-Term(iTRAQ4plex); K1(iTRAQ4plex); K13(iTRAQ4plex)	HCD	1.0000	0.0000	1	1	8681	Unique	Used	5.441	2.414E4	1.602E5	95	4.56	0.00	1	4	8	2	887.03210	1773.05693	-15.63	31.24	3193	3193	MS2	21/24	Glioma_Nuclear_prep_Gradeall_4plex.raw
4885381	High		kATGPPVSELITk	Unambiguous	1	1	N-Term(iTRAQ4plex); K1(iTRAQ4plex); K13(iTRAQ4plex)	HCD	1.0000	0.0000	1	1	8513	Unique	Used	5.285	1.712E4	1.104E5	117	5.41	0.00	1	7	2	3	591.68878	1773.05179	-18.53	30.00	3009	3009	MS2	22/48	Glioma_Nuclear_prep_Gradeall_4plex.raw
4885381		High	kPAAAGVk	Unambiguous	1	1	N-Term(iTRAQ4plex); K1(iTRAQ4plex); K8(iTRAQ4plex)	HCD	1.0000	0.0000	1	1	6827	Unique	Used	2.433	7.043E3	2.073E4	89	3.43	0.00	0	61	88	3	391.92816	1173.76993	1.65	13.25	1085	1085	MS2	18/28	Glioma_Nuclear_prep_Gradeall_repeat_4plex.raw
4885381	High		kPAAAGVk	Unambiguous	1	1	N-Term(iTRAQ4plex); K1(iTRAQ4plex); K8(iTRAQ4plex)	HCD	1.0000	0.0000	1	1	7109	Unique	Used	3.956	2.360E3	1.139E4	83	3.43	0.00	0	43	108	3	391.92831	1173.77039	2.04	19.74	1484	1484	MS2	20/28	Glioma_Nuclear_prep_Gradeall_4plex.raw
4885381	High		kPAGATPk	Unambiguous	1	1	N-Term(iTRAQ4plex); K1(iTRAQ4plex); K8(iTRAQ4plex)	HCD	1.0000	0.0000	1	1	6987	Unique	Used	1.570	1.597E3	3.058E3	95	3.65	0.00	0	79	300	3	401.26010	1201.76575	2.37	18.38	1350	1350	MS2	16/28	Glioma_Nuclear_prep_Gradeall_4plex.raw
4885381		High	kAAGAGAAk	Unambiguous	1	1	N-Term(iTRAQ4plex); K1(iTRAQ4plex); K9(iTRAQ4plex)	HCD	1.0000	0.0000	1	1	6394	Unique	Used	5.388	9.348E2	6.093E3	114	4.06	0.00	1	21	85	3	392.91916	1176.74292	0.35	9.13	628	628	MS2	24/32	Glioma_Nuclear_prep_Gradeall_repeat_4plex.raw
4885381	High		kAAGAGAAk	Unambiguous	1	1	N-Term(iTRAQ4plex); K1(iTRAQ4plex); K9(iTRAQ4plex)	HCD	1.0000	0.0000	1	1	6725	Unique	Used	4.779	1.563E2	9.115E2	139	3.45	0.00	1	41	300	3	392.91870	1176.74155	-0.82	14.68	1023	1023	MS2	22/32	Glioma_Nuclear_prep_Gradeall_4plex.raw
4885381		High	aLAAGGYDVEk	Unambiguous	1	1	N-Term(iTRAQ4plex); K11(iTRAQ4plex)	HCD	1.0000	0.0000	1	1	8842	Unique	Used	4.554	6.042E3	3.329E4	236	4.34	0.00	0	20	120	2	691.38281	1381.75835	1.27	32.08	3213	3213	MS2	20/20	Glioma_Nuclear_prep_Gradeall_repeat_4plex.raw
4885381		High	aTGPPVSELITk	Unambiguous	1	1	N-Term(iTRAQ4plex); K12(iTRAQ4plex)	HCD	1.0000	0.0000	1	1	9925	Unique	Used	4.305	2.579E3	1.343E4	164	4.28	0.00	0	39	300	2	750.94489	1500.88249	-3.41	44.11	4369	4369	MS2	20/22	Glioma_Nuclear_prep_Gradeall_repeat_4plex.raw
4885381		High	aTGPPVSELITk	Unambiguous	1	1	N-Term(iTRAQ4plex); K12(iTRAQ4plex)	HCD	1.0000	0.0000	1	1	9903	Unique	Used	2.788	2.716E3	9.160E3	192	3.74	0.00	0	89	300	2	750.94684	1500.88640	-0.81	43.82	4344	4344	MS2	16/22	Glioma_Nuclear_prep_Gradeall_repeat_4plex.raw
4885381		High	aTGPPVSELITk	Unambiguous	1	1	N-Term(iTRAQ4plex); K12(iTRAQ4plex)	HCD	1.0000	0.0000	1	1	10106	Unique	Used	2.756	1.172E3	3.907E3	155	4.07	0.00	0	22	61	3	500.96735	1500.88749	-0.09	46.38	4569	4569	MS2	20/44	Glioma_Nuclear_prep_Gradeall_repeat_4plex.raw
4885381		High	aTGPPVSELITk	Unambiguous	1	1	N-Term(iTRAQ4plex); K12(iTRAQ4plex)	HCD	1.0000	0.0000	1	1	10193	Unique	Used	2.531	3.088E3	9.455E3	155	4.77	0.00	0	26	300	2	750.94843	1500.88957	1.30	47.39	4661	4661	MS2	21/22	Glioma_Nuclear_prep_Gradeall_repeat_4plex.raw
4885381		High	aTGPPVSELITk	Unambiguous	1	1	N-Term(iTRAQ4plex); K12(iTRAQ4plex)	HCD	1.0000	0.0000	1	1	9162	Unique	Used	2.240	1.940E3	5.258E3	149	3.49	0.00	0	18	300	2	750.94934	1500.89141	2.52	35.55	3554	3554	MS2	15/22	Glioma_Nuclear_prep_Gradeall_repeat_4plex.raw
4885381	High		aTGPPVSELITk	Unambiguous	1	1	N-Term(iTRAQ4plex); K12(iTRAQ4plex)	HCD	1.0000	0.0000	1	1	9290	Unique	Used	5.415	3.807E3	2.515E4	190	3.09	0.00	0	15	13	2	750.93335	1500.85942	-18.79	35.68	3857	3857	MS2	17/22	Glioma_Nuclear_prep_Gradeall_4plex.raw
4885381	High		aTGPPVSELITk	Unambiguous	1	1	N-Term(iTRAQ4plex); K12(iTRAQ4plex)	HCD	1.0000	0.0000	1	1	8539	Unique	Used	3.317	2.127E4	8.607E4	193	2.92	0.00	0	9	10	2	750.93536	1500.86345	-16.10	30.18	3037	3037	MS2	17/22	Glioma_Nuclear_prep_Gradeall_4plex.raw
4885381	High		aTGPPVSELITk	Unambiguous	1	1	N-Term(iTRAQ4plex); K12(iTRAQ4plex)	HCD	1.0000	0.0000	1	1	9410	Unique	Used	2.811	1.020E5	3.500E5	227	4.24	0.00	0	10	1	3	500.95880	1500.86185	-17.17	36.58	3989	3989	MS2	23/44	Glioma_Nuclear_prep_Gradeall_4plex.raw
4885381		High	nGLSLAALk	Unambiguous	1	1	N-Term(iTRAQ4plex); K9(iTRAQ4plex)	HCD	1.0000	0.0000	1	1	10215	Unique	Used	3.810	5.890E2	2.715E3	155	3.59	0.00	0	12	206	3	392.25165	1174.74039	0.49	47.64	4684	4684	MS2	18/32	Glioma_Nuclear_prep_Gradeall_repeat_4plex.raw
4885381		High	nGLSLAALk	Unambiguous	1	1	N-Term(iTRAQ4plex); K9(iTRAQ4plex)	HCD	1.0000	0.0000	1	1	10308	Unique	Used	2.034	4.280E3	1.053E4	183	4.14	0.00	0	33	300	2	587.87335	1174.73943	-0.33	48.76	4784	4784	MS2	16/16	Glioma_Nuclear_prep_Gradeall_repeat_4plex.raw
4885381	High		nGLSLAALk	Unambiguous	1	1	N-Term(iTRAQ4plex); K9(iTRAQ4plex)	HCD	1.0000	0.0000	1	1	9484	Unique	Used	2.666	1.279E5	4.159E5	254	3.79	0.00	0	24	5	2	587.86414	1174.72099	-16.02	37.14	4070	4070	MS2	16/16	Glioma_Nuclear_prep_Gradeall_4plex.raw
4885381	High		nGLSLAALk	Unambiguous	1	1	N-Term(iTRAQ4plex); K9(iTRAQ4plex)	HCD	1.0000	0.0000	1	1	9579	Unique	Used	2.234	1.246E5	3.396E5	256	3.47	0.00	0	18	4	2	587.86420	1174.72112	-15.92	37.83	4173	4173	MS2	16/16	Glioma_Nuclear_prep_Gradeall_4plex.raw
5031877	NP_005564.1	LMNB1	4001	lamin-B1 isoform 1 [Homo sapiens]	47.10	2	22	42	1.943	22;20	25.6	150.40	586	66.4	5.16																			
5031877	High		tTIPEEEEEEEEAAG VVVEEELFHQQGTP R	Unambiguous	2	1	N-Term(iTRAQ4plex)	HCD	1.0000	0.0000	1	1	12461	Unique	Used	2.297	6.996E1	1.961E2	82	8.33	0.00	0	39	131	4	889.67609	3555.68252	2.60	61.84	7320	7320	MS2	30/174	Glioma_Nuclear_prep_Gradeall_4plex.raw
5031877		High	sLETENSALQLQVTE R	Unambiguous	1	1	N-Term(iTRAQ4plex)	HCD	1.0000	0.0000	1	1	10245	Unique	Used	2.320	1.092E3	3.066E3	222	6.48	0.00	0	0	300	2	981.51550	1962.02373	-1.20	47.98	4715	4715	MS2	27/30	Glioma_Nuclear_prep_Gradeall_repeat_4plex.raw
5031877		High	aLYETELADAR	Unambiguous	1	1	N-Term(iTRAQ4plex)	HCD	1.0000	0.0000	1	1	9776	Unique	Used	1.931	2.368E4	5.532E4	267	3.99	0.00	0	7	53	2	698.36560	1395.72392	0.18	42.40	4210	4210	MS2	18/20	Glioma_Nuclear_prep_Gradeall_repeat_4plex.raw
5031877		High	ISSEMNTSTVNSAR	Unambiguous	2	1	N-Term(iTRAQ4plex)	HCD	1.0000	0.0000	1	1	8362	Unique	Used	1.802	2.185E3	4.762E3	159	4.10	0.00	0	7	225	2	820.91040	1640.81352	6.37	27.28	2707	2707	MS2	20/26	Glioma_Nuclear_prep_Gradeall_repeat_4plex.raw
5031877		High	IAQALHEMR	Unambiguous	2	1	N-Term(iTRAQ4plex)	HCD	1.0000	0.0000	1	1	8958	Unique	Used	1.797	2.881E3	6.263E3	210	3.80	0.00	0	43	142	3	404.89056	1212.65714	-5.65	33.21	3335	3335	MS2	20/32	Glioma_Nuclear_prep_Gradeall_repeat_4plex.raw
5031877		High	sMYEEEINETR	Unambiguous	1	1	N-Term(iTRAQ4plex)	HCD	1.0000	0.0000	1	1	8861	Unique	Used	1.732	6.725E3	1.409E4	72	4.49	0.00	0	23	207	2	772.85272	1544.69817	-2.45	32.26	3233	3233	MS2	18/20	Glioma_Nuclear_prep_Gradeall_repeat_4plex.raw
5031877		High	aLYETELADAR	Unambiguous	1	1	N-Term(iTRAQ4plex)	HCD	1.0000	0.0000	1	1	9841	Unique	Used	1.527	7.880E3	1.456E4	270	4.16	0.00	0	33	219	2	698.36694	1395.72661	2.10	43.10	4278	4278	MS2	19/20	Glioma_Nuclear_prep_Gradeall_repeat_4plex.raw
5031877		High	aGGPTTPLSPTR	Unambiguous	1	1	N-Term(iTRAQ4plex)	HCD	1.0000	0.0000	1	1	7770	Unique	Used	1.484	5.931E4	1.065E5	156	4.30	0.00	0	9	19	2	649.86041	1298.71355	-3.84	21.62	2084	2084	MS2	16/22	Glioma_Nuclear_prep_Gradeall_repeat_4plex.raw
5031877	High		IAQALHEMR	Unambiguous	2	1	N-Term(iTRAQ4plex)	HCD	1.0000	0.0000	1	1	8433	Unique	Used	2.489	2.723E4	8.268E4	169	3.59	0.00	0	25	6	3	404.88565	1212.64240	-17.81	29.43	2922	2922	MS2	20/32	Glioma_Nuclear_prep_Gradeall_4plex.raw
5031877	High		sLETENSALQLQVTE R	Unambiguous	1	1	N-Term(iTRAQ4plex)	HCD	1.0000	0.0000	1	1	9637	Unique	Used	2.450	3.481E4	1.041E5	202	6.26	0.00	0	3	14	2	981.50037	1961.99346	-16.63	38.22	4235	4235	MS2	26/30	Glioma_Nuclear_prep_Gradeall_4plex.raw
5031877	High		ISSEMNTSTVNSAR	Unambiguous	2	1	N-Term(iTRAQ4plex)	HCD	1.0000	0.0000	1	1	8149	Unique	Used	1.984	3.550E4	8.593E4	105	4.16	0.00	0	9	18	2	820.89117	1640.77507	-17.07	27.41	2614	2614	MS2	20/26	Glioma_Nuclear_prep_Gradeall_4plex.raw
5031877	High		IALDMEISAYR	Unambiguous	2	1	N-Term(iTRAQ4plex)	HCD	1.0000	0.0000	1	1	10802	Unique	Used	1.400	3.083E4	5.266E4	232	3.61	0.00	0	53	52	2	713.37000	1425.73271	-14.14	46.93	5506	5506	MS2	18/20	Glioma_Nuclear_prep_Gradeall_4plex.raw
5031877	High		aLYETELADAR	Unambiguous	1	1	N-Term(iTRAQ4plex)	HCD	1.0000	0.0000	1	1	9261	Unique	Used	1.320	4.630E4	7.459E4	183	3.34	0.00	0	42	12	2	698.35425	1395.70122	-16.09	35.46	3825	3825	MS2	18/20	Glioma_Nuclear_prep_Gradeall_4plex.raw
5031877		High	cQSLTEDLEFR	Unambiguous	1	1	N-Term(iTRAQ4plex); C1(Methylthio)	HCD	1.0000	0.0000	1	1	11746	Unique	Used	1.955	1.245E3	2.945E3	77	4.05	0.00	0	22	300	2	765.85699	1530.70671	1.16	67.66	6427	6427	MS2	20/20	Glioma_Nuclear_prep_Gradeall_repeat_4plex.raw
5031877	High		cQSLTEDLEFR	Unambiguous	1	1	N-Term(iTRAQ4plex); C1(Methylthio)	HCD	1.0000	0.0000	1	1	10932	Unique	Used	1.613	4.986E4	9.809E4	50	4.51	0.00	0	18	27	2	765.84552	1530.68376	-13.83	47.93	5647	5647	MS2	19/20	Glioma_Nuclear_prep_Gradeall_4plex.raw
5031877		High	kQLADETLLk	Unambiguous	1	1	N-Term(iTRAQ4plex); K1(iTRAQ4plex); K10(iTRAQ4plex)	HCD	1.0000	0.0000	1	1	8779	Unique	Used	1.855	1.615E3	3.624E3	120	2.99	0.00	1	41	300	2	795.98175	1590.95622	-14.39	31.38	3146	3146	MS2	18/18	Glioma_Nuclear_prep_Gradeall_repeat_4plex.raw
5031877	High		kQLADETLLk	Unambiguous	1	1	N-Term(iTRAQ4plex); K1(iTRAQ4plex); K10(iTRAQ4plex)	HCD	1.0000	0.0000	1	1	8290	Unique	Used	2.072	3.310E4	8.366E4	127	3.89	0.00	1	34	21	2	795.98059	1590.95391	-15.84	28.41	2767	2767	MS2	18/18	Glioma_Nuclear_prep_Gradeall_4plex.raw
5031877		High	iQELEDLLAk	Unambiguous	2	1	N-Term(iTRAQ4plex); K10(iTRAQ4plex)	HCD	1.0000	0.0000	1	1	12337	Unique	Used	2.348	2.613E3	7.424E3	231	4.55	0.00	0	6	300	2	730.43488	1459.86247	0.96	80.31	7408	7408	MS2	18/18	Glioma_Nuclear_prep_Gradeall_repeat_4plex.raw
5031877	High		iQELEDLLAk	Unambiguous	2	1	N-Term(iTRAQ4plex); K10(iTRAQ4plex)	HCD	1.0000	0.0000	1	1	11807	Unique	Used	2.113	1.154E5	2.974E5	236	4.39	0.00	0	17	15	2	730.43365	1459.86003	-0.71	55.28	6602	6602	MS2	18/18	Glioma_Nuclear_prep_Gradeall_4plex.raw
5031877	High		eELEQTYHAk	Unambiguous	2	1	N-Term(iTRAQ4plex); K10(iTRAQ4plex)	HCD	1.0000	0.0000	1	1	7549	Unique	Used	1.593	1.577E4	3.066E4	163	3.59	0.00	0	50	12	3	512.60211	1535.79178	-1.72	23.16	1964	1964	MS2	21/36	Glioma_Nuclear_prep_Gradeall_4plex.raw
5031877	High		iQELEDLLAk	Unambiguous	2	1	N-Term(iTRAQ4plex); K10(iTRAQ4plex)	HCD	1.0000	0.0000	1	1	11890	Unique	Used	1.565	1.367E5	2.611E5	214	4.19	0.00	0	18	14	2	730.43774	1459.86821	4.89	55.97	6692	6692	MS2	18/18	Glioma_Nuclear_prep_Gradeall_4plex.raw
5031877		High	dAALATALGDk	Unambiguous	1	1	N-Term(iTRAQ4plex); K11(iTRAQ4plex)	HCD	1.0000	0.0000	1	1	9595	Unique	Used	1.368	9.541E3	1.579E4	310	4.45	0.00	0	28	231	2	667.37866	1333.75005	-4.92	40.34	4018	4018	MS2	19/20	Glioma_Nuclear_prep_Gradeall_repeat_4plex.raw
5031877	High		dAALATALGDk	Unambiguous	1	1	N-Term(iTRAQ4plex); K11(iTRAQ4plex)	HCD	1.0000	0.0000	1	1	9082	Unique	Used	1.734	1.726E5	3.652E5	299	4.23	0.00	0	15	5	2	667.37073	1333.73418	-16.82	34.16	3630	3630	MS2	19/20	Glioma_Nuclear_prep_Gradeall_4plex.raw
5031877	High		dAALATALGDkk	Unambiguous	1	1	N-Term(iTRAQ4plex); K11(iTRAQ4plex); K12(iTRAQ4plex)	HCD	1.0000	0.0000	1	1	8481	Unique	Used	1.575	3.144E4	6.039E4	210	4.33	0.00	1	7	13	2	803.46765	1605.92803	-15.95	29.76	2974	2974	MS2	20/22	Glioma_Nuclear_prep_Gradeall_4plex.raw
5031877		High	aEHDQLLLNYAk	Unambiguous	1	1	N-Term(iTRAQ4plex); K12(iTRAQ4plex)	HCD	1.0000	0.0000	1	1	9489	Unique	Used	2.205	1.862E3	4.966E3	193	5.39	0.00	0	15	300	2	851.97540	1702.94353	4.02	39.13	3900	3900	MS2	22/22	Glioma_Nuclear_prep_Gradeall_repeat_4plex.raw
5031877	High		aEHDQLLLNYAk	Unambiguous	1	1	N-Term(iTRAQ4plex); K12(iTRAQ4plex)	HCD	1.0000	0.0000	1	1	8846	Unique	Used	1.993	8.294E4	2.017E5	163	5.04	0.00	0	10	8	2	851.95844	1702.90959	-15.90	32.43	3372	3372	MS2	22/22	Glioma_Nuclear_prep_Gradeall_4plex.raw
5031877		High	iESLSSQLSNLQk	Unambiguous	2	1	N-Term(iTRAQ4plex); K13(iTRAQ4plex)	HCD	1.0000	0.0000	1	1	11003	Unique	Used	1.800	2.566E3	5.587E3	264	5.91	0.00	0	14	300	2	867.99768	1734.98808	2.34	57.18	5535	5535	MS2	24/24	Glioma_Nuclear_prep_Gradeall_repeat_4plex.raw
5031877	High		iESLSSQLSNLQk	Unambiguous	2	1	N-Term(iTRAQ4plex); K13(iTRAQ4plex)	HCD	1.0000	0.0000	1	1	10191	Unique	Used	1.931	6.032E4	1.421E5	254	5.44	0.00	0	19	20	2	867.98315	1734.95903	-14.41	42.28	4838	4838	MS2	24/24	Glioma_Nuclear_prep_Gradeall_4plex.raw
5031877	High		dQIAQLEASLAAAk	Unambiguous	1	1	N-Term(iTRAQ4plex); K14(iTRAQ4plex)	HCD	1.0000	0.0000	1	1	11440	Unique	Used	2.134	3.167E3	8.244E3	180	3.64	0.00	0	38	81	3	572.99371	1716.96659	-4.02	52.09	6202	6202	MS2	23/52	Glioma_Nuclear_prep_Gradeall_4plex.raw
5031877	High		dQMQQQLNDYEQLL DVk	Unambiguous	2	1	N-Term(iTRAQ4plex); K17(iTRAQ4plex)	HCD	1.0000	0.0000	1	1	11983	Unique	Used	3.205	5.272E3	2.062E4	151	7.48	0.00	0	0	62	2	1198.59827	2396.18926	-4.76	56.82	6794	6794	MS2	25/32	Glioma_Nuclear_prep_Gradeall_4plex.raw
5031877	High		dQMQQQLNDYEQLL DVk	Unambiguous	2	1	N-Term(iTRAQ4plex); K17(iTRAQ4plex)	HCD	1.0000	0.0000	1	1	11974	Unique	Used	1.526	2.322E3	4.322E3	158	7.74	0.00	0	18	68	3	799.40289	2396.19413	-2.73	56.73	6784	6784	MS2	28/64	Glioma_Nuclear_prep_Gradeall_4plex.raw
5031877		High	lYkEELEQTYHAk	Unambiguous	2	1	N-Term(iTRAQ4plex); K3(iTRAQ4plex); K13(iTRAQ4plex)	HCD	1.0000	0.0000	1	1	9412	Unique	Used	2.466	6.182E2	1.844E3	139	5.05	0.00	1	20	170	3	695.38544	2084.14176	1.40	38.25	3817	3817	MS2	27/48	Glioma_Nuclear_prep_Gradeall_repeat_4plex.raw
5031877	High		lYkEELEQTYHAk	Unambiguous	2	1	N-Term(iTRAQ4plex); K3(iTRAQ4plex); K13(iTRAQ4plex)	HCD	1.0000	0.0000	1	1	8728	Unique	Used	3.778	1.296E4	5.974E4	134	4.60	0.00	1	13	3	3	695.37292	2084.10422	-16.61	31.58	3244	3244	MS2	30/48	Glioma_Nuclear_prep_Gradeall_4plex.raw
5031877	High		lYkEELEQTYHAk	Unambiguous	2	1	N-Term(iTRAQ4plex); K3(iTRAQ4plex); K13(iTRAQ4plex)	HCD	1.0000	0.0000	1	1	8735	Unique	Used	2.665	2.175E4	7.071E4	161	5.40	0.00	1	0	13	2	1042.55481	2084.10234	-17.51	31.62	3251	3251	MS2	22/24	Glioma_Nuclear_prep_Gradeall_4plex.raw
5031877		High	lAVYIDk	Unambiguous	1	1	N-Term(iTRAQ4plex); K7(iTRAQ4plex)	HCD	1.0000	0.0000	1	1	9757	Unique	Used	2.868	1.724E4	5.982E4	213	2.84	0.00	0	12	112	2	555.34491	1109.68254	1.49	42.18	4190	4190	MS2	12/12	Glioma_Nuclear_prep_Gradeall_repeat_4plex.raw
5031877		High	qLADETLLk	Unambiguous	1	1	N-Term(iTRAQ4plex); K9(iTRAQ4plex)	HCD	1.0000	0.0000	1	1	9505	Unique	Used	2.065	1.486E4	3.712E4	253	3.69	0.00	0	40	80	2	659.89038	1318.77349	-6.52	39.32	3919	3919	MS2	16/16	Glioma_Nuclear_prep_Gradeall_repeat_4plex.raw
5031877		High	eYEAALNSk	Unambiguous	1	1	N-Term(iTRAQ4plex); K9(iTRAQ4plex)	HCD	1.0000	0.0000	1	1	8290	Unique	Used	1.886	3.564E4	8.134E4	165	3.44	0.00	0	8	39	2	656.85645	1312.70561	5.25	26.61	2632	2632	MS2	16/16	Glioma_Nuclear_prep_Gradeall_repeat_4plex.raw
5031877	High		qLADETLLk	Unambiguous	1	1	N-Term(iTRAQ4plex); K9(iTRAQ4plex)	HCD	1.0000	0.0000	1	1	8941	Unique	Used	1.465	9.957E4	1.780E5	277	3.45	0.00	0	48	13	2	659.88373	1318.76018	-16.60	33.10	3475	3475	MS2	16/16	Glioma_Nuclear_prep_Gradeall_4plex.raw
5031877		High	smYEEEINETR	Unambiguous	1	1	N-Term(iTRAQ4plex); M2(Oxidation)	HCD	1.0000	0.0000	1	1	8279	Unique	Used	2.264	5.123E3	1.403E4	52	3.20	0.00	0	29	258	2	780.85693	1560.70659	6.23	26.48	2620	2620	MS2	16/20	Glioma_Nuclear_prep_Gradeall_repeat_4plex.raw
5031877		High	dQmQQQLNDYEQLL DVk	Unambiguous	2	1	N-Term(iTRAQ4plex); M3(Oxidation); K17(iTRAQ4plex)	HCD	1.0000	0.0000	1	1	11794	Unique	Used	1.332	1.335E2	2.151E2	132	6.36	0.00	0	19	300	3	804.73755	2412.19809	1.04	68.47	6493	6493	MS2	27/64	Glioma_Nuclear_prep_Gradeall_repeat_4plex.raw
5031877	High		dQmQQQLNDYEQLL DVk	Unambiguous	2	1	N-Term(iTRAQ4plex); M3(Oxidation); K17(iTRAQ4plex)	HCD	1.0000	0.0000	1	1	11082	Unique	Used	2.461	5.479E3	1.645E4	111	5.92	0.00	0	3	41	2	1206.59436	2412.18144	-5.86	49.09	5810	5810	MS2	25/32	Glioma_Nuclear_prep_Gradeall_4plex.raw
5031877		High	ISSEmNTSTVNSAR	Unambiguous	2	1	N-Term(iTRAQ4plex); M5(Oxidation)	HCD	1.0000	0.0000	1	1	7323	Unique	Used	2.271	5.104E3	1.403E4	115	4.88	0.00	0	25	146	2	828.90106	1656.79485	-1.90	17.60	1609	1609	MS2	20/26	Glioma_Nuclear_prep_Gradeall_repeat_4plex.raw
5032007	NP_005850.1	PURA	5813	transcriptional activator protein Pur-alpha [Homo sapiens]	9.94	1	2	3	0.594	2;1	34.9	11.23	322	34.9	6.44																			
5032007	High		gPGLGSTQGQTIALP AQGLIEFR	Unambiguous	1	1	N-Term(iTRAQ4plex)	HCD	1.0000	0.0000	1	1	11841	Unique	Used	0.298	2.891E4	1.050E4	89	7.50	0.00	0	0	94	2	1228.17200	2455.33672	-2.42	55.54	6638	6638	MS2	30/44	Glioma_Nuclear_prep_Gradeall_4plex.raw
5032007		High	fFFDVGSNk	Unambiguous	1	1	N-Term(iTRAQ4plex); K9(iTRAQ4plex)	HCD	1.0000	0.0000	1	1	11289	Unique	Used	0.604	1.124E4	8.209E3	310	3.38	0.00	0	60	300	2	674.86194	1348.71660	1.93	61.05	5868	5868	MS2	15/16	Glioma_Nuclear_prep_Gradeall_repeat_4plex.raw
5032007	High		fFFDVGSNk	Unambiguous	1	1	N-Term(iTRAQ4plex); K9(iTRAQ4plex)	HCD	1.0000	0.0000	1	1	10419	Unique	Used	0.594	9.786E4	7.088E4	293	3.73	0.00	0	88	46	2	674.85797	1348.70867	-3.95	43.98	5086	5086	MS2	14/16	Glioma_Nuclear_prep_Gradeall_4plex.raw
5032027	NP_005601.1	RBBP4	5928	histone-binding protein RBBP4 isoform a [Homo sapiens]	13.65	5	3	5	0.628	2;3	4.5	18.43	425	47.6	4.89																			
5032027		High	eAAFDDAVEER	Unambiguous	1	1	N-Term(iTRAQ4plex)	HCD	1.0000	0.0000	1	1	8859	Unique	Used	0.636	2.659E4	2.045E4	80	4.12	0.00	0	22	144	2	698.32764	1395.64800	-2.10	32.25	3231	3231	MS2	19/20	Glioma_Nuclear_prep_Gradeall_repeat_4plex.raw
5032027	High		eAAFDDAVEER	Unambiguous	1	1	N-Term(iTRAQ4plex)	HCD	1.0000	0.0000	1	1	8519	Unique	Used	0.643	1.966E5	1.543E5	56	4.02	0.00	0	13	16	2	698.31805	1395.62883	-15.83	30.03	3015	3015	MS2	17/20	Glioma_Nuclear_prep_Gradeall_4plex.raw
5032027		High	tPSSDVLVFDYTk	Unambiguous	5	1	N-Term(iTRAQ4plex); K13(iTRAQ4plex)	HCD	1.0000	0.0000	1	1	11152	Unique	Used	0.603	5.016E3	3.657E3	224	5.36	0.00	0	27	300	2	880.47412	1759.94097	3.00	59.15	5706	5706	MS2	19/24	Glioma_Nuclear_prep_Gradeall_repeat_4plex.raw
5032027	High		tPSSDVLVFDYTk	Unambiguous	5	1	N-Term(iTRAQ4plex); K13(iTRAQ4plex)	HCD	1.0000	0.0000	1	1	10329	Unique	Used	0.575	2.108E5	1.480E5	227	5.26	0.00	0	19	12	2	880.45966	1759.91203	-13.43	43.29	4988	4988	MS2	20/24	Glioma_Nuclear_prep_Gradeall_4plex.raw
5032027		High	vINEEYk	Unambiguous	4	1	N-Term(iTRAQ4plex); K7(iTRAQ4plex)	HCD	1.0000	0.0000	1	1	7986	Unique	Used	0.628	3.878E4	2.947E4	275	3.02	0.00	0	22	133	2	591.83569	1182.66411	2.73	23.67	2311	2311	MS2	12/12	Glioma_Nuclear_prep_Gradeall_repeat_4plex.raw
5032051	NP_005608.1	RPS14	6208	40S ribosomal protein S14 [Homo sapiens]	15.23	1	2	3	0.577	2;1	5.2	7.78	151	16.3	10.05																			
5032051		High	iEDVTPIPSDSTR	Unambiguous	1	1	N-Term(iTRAQ4plex)	HCD	1.0000	0.0000	1	1	9335	Unique	Used	0.629	6.770E3	5.149E3	181	4.07	0.00	0	27	300	2	787.41467	1573.82207	1.92	37.46	3736	3736	MS2	18/24	Glioma_Nuclear_prep_Gradeall_repeat_4plex.raw
5032051	High		iEDVTPIPSDSTR	Unambiguous	1	1	N-Term(iTRAQ4plex)	HCD	1.0000	0.0000	1	1	9087	Unique	Used	0.574	5.802E4	4.066E4	160	4.33	0.00	0	5	24	2	787.40082	1573.79436	-15.69	34.19	3635	3635	MS2	19/24	Glioma_Nuclear_prep_Gradeall_4plex.raw
5032051	High		eLGITALHIk	Unambiguous	1	1	N-Term(iTRAQ4plex); K10(iTRAQ4plex)	HCD	1.0000	0.0000	1	1	9513	Unique	Used	0.577	1.550E4	1.090E4	148	3.45	0.00	0	29	12	3	461.61694	1382.83628	-17.88	37.34	4101	4101	MS2	21/36	Glioma_Nuclear_prep_Gradeall_4plex.raw
5032179	NP_005753.1	TRIM28	10155	transcription intermediary factor 1-beta [Homo sapiens]	5.39	1	3	3	0.523	1;2	19.1	7.72	835	88.5	5.77																			
5032179		High	lSPPYSSPQEFAQDV GR	Unambiguous	1	1	N-Term(iTRAQ4plex)	HCD	1.0000	0.0000	1	1	11251	Unique	Used	0.431	1.840E3	9.594E2	142	3.61	0.00	0	30	300	2	1011.50629	2022.00530	0.17	60.45	5819	5819	MS2	18/32	Glioma_Nuclear_prep_Gradeall_repeat_4plex.raw
5032179		High	aDVQSIIGLQR	Unambiguous	1	1	N-Term(iTRAQ4plex)	HCD	1.0000	0.0000	1	1	10497	Unique	Used	0.633	5.335E3	4.083E3	289	4.12	0.00	0	56	300	2	672.39343	1343.77959	2.38	51.00	4985	4985	MS2	17/20	Glioma_Nuclear_prep_Gradeall_repeat_4plex.raw
5032179	High		vFPGSTTEDYNLIVIE R	Unambiguous	1	1	N-Term(iTRAQ4plex)	HCD	1.0000	0.0000	1	1	11401	Unique	Used	0.523	9.743E3	6.222E3	241	3.59	0.00	0	34	227	2	1049.04834	2097.08940	-4.35	51.75	6159	6159	MS2	13/32	Glioma_Nuclear_prep_Gradeall_4plex.raw
5174449	NP_006017.1	H1FX	8971	histone H1x [Homo sapiens]	23.00	1	4	17	2.438	7;10	23.6	54.20	213	22.5	10.76																			
5174449		High	ySQLVVETIR	Unambiguous	1	1	N-Term(iTRAQ4plex)	HCD	1.0000	0.0000	1	1	10814	Unique	Used	3.217	2.466E4	9.600E4	204	3.68	0.00	0	5	47	2	676.38959	1351.77190	1.25	54.86	5329	5329	MS2	17/18	Glioma_Nuclear_prep_Gradeall_repeat_4plex.raw
5174449		High	ySQLVVETIR	Unambiguous	1	1	N-Term(iTRAQ4plex)	HCD	1.0000	0.0000	1	1	10740	Unique	Used	2.838	5.680E3	1.950E4	323	3.88	0.00	0	54	300	2	676.38989	1351.77251	1.70	53.92	5247	5247	MS2	16/18	Glioma_Nuclear_prep_Gradeall_repeat_4plex.raw
5174449	High		ySQLVVETIR	Unambiguous	1	1	N-Term(iTRAQ4plex)	HCD	1.0000	0.0000	1	1	10050	Unique	Used	2.950	1.970E5	7.088E5	283	3.30	0.00	0	7	4	2	676.37854	1351.74980	-15.10	41.22	4684	4684	MS2	17/18	Glioma_Nuclear_prep_Gradeall_4plex.raw
5174449	High		ySQLVVETIR	Unambiguous	1	1	N-Term(iTRAQ4plex)	HCD	1.0000	0.0000	1	1	9962	Unique	Used	2.422	1.144E5	3.381E5	259	3.27	0.00	0	22	14	2	676.37793	1351.74858	-16.00	40.58	4588	4588	MS2	17/18	Glioma_Nuclear_prep_Gradeall_4plex.raw
5174449		High	aGGSAALSPSk	Unambiguous	1	1	N-Term(iTRAQ4plex); K11(iTRAQ4plex)	HCD	1.0000	0.0000	1	1	7153	Unique	Used	3.175	6.225E4	2.391E5	296	4.29	0.00	0	5	16	2	617.35303	1233.69878	-4.37	16.19	1431	1431	MS2	19/20	Glioma_Nuclear_prep_Gradeall_repeat_4plex.raw
5174449		High	aGGSAALSPSk	Unambiguous	1	1	N-Term(iTRAQ4plex); K11(iTRAQ4plex)	HCD	1.0000	0.0000	1	1	7119	Unique	Used	2.202	2.112E4	5.627E4	266	4.09	0.00	0	11	79	2	617.34595	1233.68462	-15.85	15.86	1395	1395	MS2	19/20	Glioma_Nuclear_prep_Gradeall_repeat_4plex.raw
5174449	High		aGGSAALSPSk	Unambiguous	1	1	N-Term(iTRAQ4plex); K11(iTRAQ4plex)	HCD	1.0000	0.0000	1	1	7309	Unique	Used	2.157	6.617E4	1.741E5	314	4.01	0.00	0	21	17	2	617.35223	1233.69719	-5.65	21.44	1704	1704	MS2	17/20	Glioma_Nuclear_prep_Gradeall_4plex.raw
5174449		High	aLVQNDTLLQVk	Unambiguous	1	1	N-Term(iTRAQ4plex); K12(iTRAQ4plex)	HCD	1.0000	0.0000	1	1	10400	Unique	Used	3.084	5.268E3	1.966E4	128	5.95	0.00	0	38	300	2	815.49310	1629.97893	0.68	49.83	4881	4881	MS2	22/22	Glioma_Nuclear_prep_Gradeall_repeat_4plex.raw

5174449		High	aLVQNDTLLQVk	Unambiguous	1	1	N-Term(iTRAQ4plex); K12(iTRAQ4plex)	HCD	1.0000	0.0000	1	1	10472	Unique	Used	2.438	3.706E3	1.093E4	129	5.67	0.00	0	13	300	2	815.49384	1629.98039	1.57	50.69	4958	4958	MS2	22/22	Glioma_Nuclear_prep_Gradeall_repeat_4plex.raw
5174449		High	aLVQNDTLLQVk	Unambiguous	1	1	N-Term(iTRAQ4plex); K12(iTRAQ4plex)	HCD	1.0000	0.0000	1	1	10398	Unique	Used	2.215	1.227E3	3.289E3	128	5.34	0.00	0	16	300	3	543.99811	1629.97977	1.19	49.80	4879	4879	MS2	22/44	Glioma_Nuclear_prep_Gradeall_repeat_4plex.raw
5174449		High	aLVQNDTLLQVk	Unambiguous	1	1	N-Term(iTRAQ4plex); K12(iTRAQ4plex)	HCD	1.0000	0.0000	1	1	10473	Unique	Used	1.329	1.199E3	1.929E3	132	5.03	0.00	0	25	300	3	543.99750	1629.97794	0.07	50.70	4959	4959	MS2	22/44	Glioma_Nuclear_prep_Gradeall_repeat_4plex.raw
5174449	High		aLVQNDTLLQVk	Unambiguous	1	1	N-Term(iTRAQ4plex); K12(iTRAQ4plex)	HCD	1.0000	0.0000	1	1	9686	Unique	Used	3.763	1.088E5	4.996E5	154	4.85	0.00	0	8	5	2	815.47968	1629.95207	-15.80	38.59	4289	4289	MS2	22/22	Glioma_Nuclear_prep_Gradeall_4plex.raw
5174449	High		aLVQNDTLLQVk	Unambiguous	1	1	N-Term(iTRAQ4plex); K12(iTRAQ4plex)	HCD	1.0000	0.0000	1	1	9786	Unique	Used	2.379	6.782E4	1.969E5	161	4.65	0.00	0	27	11	2	815.48041	1629.95354	-14.90	39.30	4397	4397	MS2	22/22	Glioma_Nuclear_prep_Gradeall_4plex.raw
5174449		High	gAPAAATAPAPTAHk	Unambiguous	1	1	N-Term(iTRAQ4plex); K15(iTRAQ4plex)	HCD	1.0000	0.0000	1	1	7090	Unique	Used	3.432	5.384E3	2.236E4	239	5.27	0.00	0	10	86	2	810.44672	1619.88616	-15.23	15.56	1363	1363	MS2	24/28	Glioma_Nuclear_prep_Gradeall_repeat_4plex.raw
5174449		High	gAPAAATAPAPTAHk	Unambiguous	1	1	N-Term(iTRAQ4plex); K15(iTRAQ4plex)	HCD	1.0000	0.0000	1	1	7082	Unique	Used	1.964	4.256E3	1.011E4	174	5.95	0.00	0	24	83	3	540.63165	1619.88041	-18.78	15.48	1354	1354	MS2	23/56	Glioma_Nuclear_prep_Gradeall_repeat_4plex.raw
5174449	High		gAPAAATAPAPTAHk	Unambiguous	1	1	N-Term(iTRAQ4plex); K15(iTRAQ4plex)	HCD	1.0000	0.0000	1	1	7287	Unique	Used	3.131	2.202E4	8.413E4	243	6.22	0.00	0	0	44	2	810.45483	1619.90239	-5.21	21.25	1679	1679	MS2	24/28	Glioma_Nuclear_prep_Gradeall_4plex.raw
5174449	High		gAPAAATAPAPTAHk	Unambiguous	1	1	N-Term(iTRAQ4plex); K15(iTRAQ4plex)	HCD	1.0000	0.0000	1	1	7279	Unique	Used	2.211	2.407E4	6.494E4	162	5.99	0.00	0	16	5	3	540.63879	1619.90183	-5.56	21.21	1671	1671	MS2	27/56	Glioma_Nuclear_prep_Gradeall_4plex.raw
5730027	NP_006550.1	KHDRBS1	10657	KH domain-containing, RNA-binding, signal transduction-associated protein 1 isoform 1 [Homo sapiens]	16.70	6	4	12	0.468	6;6	37.8	41.67	443	48.2	8.66																			
5730027		High	sGSMDPSGAHPSVR	Unambiguous	2	1	N-Term(iTRAQ4plex)	HCD	1.0000	0.0000	1	1	7122	Unique	Used	0.441	1.219E4	6.497E3	70	4.04	0.00	0	13	213	2	764.85870	1528.71013	-12.69	15.88	1398	1398	MS2	18/26	Glioma_Nuclear_prep_Gradeall_repeat_4plex.raw
5730027	High		sGSMDPSGAHPSVR	Unambiguous	2	1	N-Term(iTRAQ4plex)	HCD	1.0000	0.0000	1	1	7298	Unique	Used	0.652	2.630E4	2.094E4	109	3.12	0.00	0	12	76	2	764.86591	1528.72453	-3.26	21.34	1691	1691	MS2	15/26	Glioma_Nuclear_prep_Gradeall_4plex.raw
5730027		High	kDDEENYLDLFSHk	Unambiguous	2	1	N-Term(iTRAQ4plex); K1(iTRAQ4plex); K14(iTRAQ4plex)	HCD	1.0000	0.0000	1	1	10779	Unique	Used	0.504	4.847E2	2.956E2	137	3.37	0.00	1	16	123	4	547.03625	2185.12319	4.31	54.41	5290	5290	MS2	30/78	Glioma_Nuclear_prep_Gradeall_repeat_4plex.raw
5730027		High	kDDEENYLDLFSHk	Unambiguous	2	1	N-Term(iTRAQ4plex); K1(iTRAQ4plex); K14(iTRAQ4plex)	HCD	1.0000	0.0000	1	1	10766	Unique	Used	0.594	1.142E3	8.213E2	146	6.55	0.00	1	30	300	3	729.04706	2185.12662	5.88	54.24	5275	5275	MS2	33/52	Glioma_Nuclear_prep_Gradeall_repeat_4plex.raw
5730027	High		kDDEENYLDLFSHk	Unambiguous	2	1	N-Term(iTRAQ4plex); K1(iTRAQ4plex); K14(iTRAQ4plex)	HCD	1.0000	0.0000	1	1	9841	Unique	Used	0.010	7.465E3	6.689E1	119	3.54	0.00	1	46	7	4	547.02454	2185.07631	-17.14	39.70	4457	4457	MS2	26/78	Glioma_Nuclear_prep_Gradeall_4plex.raw
5730027	High		kDDEENYLDLFSHk	Unambiguous	2	1	N-Term(iTRAQ4plex); K1(iTRAQ4plex); K14(iTRAQ4plex)	HCD	1.0000	0.0000	1	1	9796	Unique	Used	0.640	7.273E4	5.681E4	125	6.32	0.00	1	26	5	3	729.03448	2185.08890	-11.38	39.37	4408	4408	MS2	28/52	Glioma_Nuclear_prep_Gradeall_4plex.raw
5730027		High	iLGPQGNTIk	Unambiguous	1	1	N-Term(iTRAQ4plex); K10(iTRAQ4plex)	HCD	1.0000	0.0000	1	1	8739	Unique	Used	0.429	9.978E4	5.184E4	153	3.94	0.00	0	16	80	2	664.90356	1328.79985	-10.68	30.97	3104	3104	MS2	17/18	Glioma_Nuclear_prep_Gradeall_repeat_4plex.raw
5730027		High	iLGPQGNTIk	Unambiguous	1	1	N-Term(iTRAQ4plex); K10(iTRAQ4plex)	HCD	1.0000	0.0000	1	1	8708	Unique	Used	0.468	1.250E5	7.082E4	86	3.14	0.00	0	28	62	2	664.91547	1328.82366	7.23	30.73	3072	3072	MS2	17/18	Glioma_Nuclear_prep_Gradeall_repeat_4plex.raw
5730027	High		iLGPQGNTIk	Unambiguous	1	1	N-Term(iTRAQ4plex); K10(iTRAQ4plex)	HCD	1.0000	0.0000	1	1	8322	Unique	Used	0.370	1.482E6	6.700E5	147	3.64	0.00	0	13	3	2	664.90179	1328.79631	-13.35	28.64	2802	2802	MS2	17/18	Glioma_Nuclear_prep_Gradeall_4plex.raw
5730027	High		dSLDPSFTHAMQLLTAEIEk	Unambiguous	2	1	N-Term(iTRAQ4plex); K20(iTRAQ4plex)	HCD	1.0000	0.0000	1	1	12863	Unique	Used	0.408	1.083E3	5.390E2	150	6.33	0.00	0	13	300	3	845.44232	2534.31241	2.86	67.38	7797	7797	MS2	28/76	Glioma_Nuclear_prep_Gradeall_4plex.raw
5730027	High		dSLDPSFTHAMQLLTAEIEk	Unambiguous	2	1	N-Term(iTRAQ4plex); K20(iTRAQ4plex)	HCD	1.0000	0.0000	1	1	12908	Unique	Used	0.469	9.833E2	5.621E2	131	6.33	0.00	0	16	300	3	845.44263	2534.31333	3.22	68.12	7856	7856	MS2	27/76	Glioma_Nuclear_prep_Gradeall_4plex.raw
5730027		High	sGSmDPSGAHPSVR	Unambiguous	2	1	N-Term(iTRAQ4plex); M4(Oxidation)	HCD	1.0000	0.0000	1	1	6668	Unique	Used	0.681	4.580E3	3.771E3	78	5.02	0.00	0	47	188	3	515.57904	1544.72257	-1.21	11.81	918	918	MS2	21/52	Glioma_Nuclear_prep_Gradeall_repeat_4plex.raw
5730073	NP_006549.1	KHDRBS3	10656	KH domain-containing, RNA-binding, signal transduction-associated protein 3 [Homo sapiens]	20.81	5	6	11	0.657	6;5	9.9	32.90	346	38.8	7.61																			
5730073		High	fLIPDYNDEIR	Unambiguous	1	1	N-Term(iTRAQ4plex)	HCD	1.0000	0.0000	1	1	11565	Unique	Used	0.562	5.741E3	3.903E3	276	3.23	0.00	0	15	300	2	769.90417	1538.80107	2.53	65.08	6208	6208	MS2	14/20	Glioma_Nuclear_prep_Gradeall_repeat_4plex.raw
5730073		High	fLIPDYNDEIR	Unambiguous	1	1	N-Term(iTRAQ4plex)	HCD	1.0000	0.0000	1	1	11640	Unique	Used	0.596	1.473E4	1.062E4	274	3.53	0.00	0	29	300	2	769.90393	1538.80058	2.22	66.11	6296	6296	MS2	13/20	Glioma_Nuclear_prep_Gradeall_repeat_4plex.raw
5730073	High		fLIPDYNDEIR	Unambiguous	1	1	N-Term(iTRAQ4plex)	HCD	1.0000	0.0000	1	1	10790	Unique	Used	0.609	6.674E5	4.960E5	239	3.19	0.00	0	17	10	2	769.89221	1538.77715	-13.01	46.85	5493	5493	MS2	15/20	Glioma_Nuclear_prep_Gradeall_4plex.raw
5730073	High		dSLDPSFTHALR	Unambiguous	1	1	N-Term(iTRAQ4plex)	HCD	1.0000	0.0000	1	1	9043	Unique	Used	0.657	7.496E4	6.007E4	84	4.28	0.00	0	13	5	3	501.58771	1502.74857	-15.61	33.84	3586	3586	MS2	27/44	Glioma_Nuclear_prep_Gradeall_4plex.raw
5730073		High	yIDVVINk	Unambiguous	1	1	N-Term(iTRAQ4plex); K8(iTRAQ4plex)	HCD	1.0000	0.0000	1	1	10407	Unique	Used	0.714	3.301E4	2.853E4	179	3.54	0.00	0	28	300	2	626.38336	1251.75945	3.46	49.92	4889	4889	MS2	13/14	Glioma_Nuclear_prep_Gradeall_repeat_4plex.raw
5730073		High	lVNQEIEk	Unambiguous	1	1	N-Term(iTRAQ4plex); K8(iTRAQ4plex)	HCD	1.0000	0.0000	1	1	8373	Unique	Used	0.750	9.220E4	8.364E4	274	3.32	0.00	0	40	79	2	630.87683	1260.74639	4.90	27.40	2719	2719	MS2	14/14	Glioma_Nuclear_prep_Gradeall_repeat_4plex.raw
5730073	High		lQEETLTk	Unambiguous	1	1	N-Term(iTRAQ4plex); K8(iTRAQ4plex)	HCD	1.0000	0.0000	1	1	8026	Unique	Used	0.626	5.245E5	4.009E5	291	3.18	0.00	0	28	10	2	625.35840	1249.70952	-11.76	26.54	2481	2481	MS2	14/14	Glioma_Nuclear_prep_Gradeall_4plex.raw
5730073	High		yIDVVINk	Unambiguous	1	1	N-Term(iTRAQ4plex); K8(iTRAQ4plex)	HCD	1.0000	0.0000	1	1	9725	Unique	Used	0.645	5.592E5	4.397E5	259	3.07	0.00	0	52	8	2	626.37323	1251.73918	-12.73	38.87	4331	4331	MS2	13/14	Glioma_Nuclear_prep_Gradeall_4plex.raw
5730073	High		lVNQEIEk	Unambiguous	1	1	N-Term(iTRAQ4plex); K8(iTRAQ4plex)	HCD	1.0000	0.0000	1	1	8105	Unique	Used	0.778	1.172E6	1.112E6	213	2.99	0.00	0	53	4	2	630.86597	1260.72466	-12.33	27.11	2567	2567	MS2	14/14	Glioma_Nuclear_prep_Gradeall_4plex.raw
5730073		High	mGHALEEIk	Unambiguous	1	1	N-Term(iTRAQ4plex); K9(iTRAQ4plex)	HCD	1.0000	0.0000	1	1	8694	Unique	Used	0.669	6.709E3	5.428E3	273	3.38	0.00	0	59	120	3	439.24963	1315.73435	4.61	30.59	3057	3057	MS2	17/32	Glioma_Nuclear_prep_Gradeall_repeat_4plex.raw
5730073	High		mGHALEEIk	Unambiguous	1	1	N-Term(iTRAQ4plex); K9(iTRAQ4plex)	HCD	1.0000	0.0000	1	1	8232	Unique	Used	0.679	5.378E5	4.453E5	182	3.57	0.00	0	52	10	2	658.35742	1315.70757	-15.74	28.00	2704	2704	MS2	14/16	Glioma_Nuclear_prep_Gradeall_4plex.raw
6005721	NP_009106.1	ERLIN2	11160	erlin-2 isoform 1 [Homo sapiens]	11.50	2	3	6	0.625	3;3	5.6	16.03	339	37.8	5.62																			
6005721		High	iSEIEDAAFLAR	Unambiguous	2	1	N-Term(iTRAQ4plex)	HCD	1.0000	0.0000	1	1	11715	Unique	Used	0.642	5.555E3	4.316E3	243	4.17	0.00	0	33	300	2	739.90289	1478.79851	0.89	67.17	6386	6386	MS2	20/22	Glioma_Nuclear_prep_Gradeall_repeat_4plex.raw
6005721	High		iSEIEDAAFLAR	Unambiguous	2	1	N-Term(iTRAQ4plex)	HCD	1.0000	0.0000	1	1	10917	Unique	Used	0.650	2.278E5	1.805E5	211	4.13	0.00	0	21	15	2	739.89276	1478.77825	-12.81	47.82	5631	5631	MS2	20/22	Glioma_Nuclear_prep_Gradeall_4plex.raw
6005721		High	vAQVAEITYGQk	Unambiguous	1	1	N-Term(iTRAQ4plex); K12(iTRAQ4plex)	HCD	1.0000	0.0000	1	1	9545	Unique	Used	0.558	7.362E3	4.969E3	231	4.54	0.00	0	19	300	2	797.94678	1594.88628	-11.31	39.79	3964	3964	MS2	18/22	Glioma_Nuclear_prep_Gradeall_repeat_4plex.raw
6005721		High	vAQVAEITYGQk	Unambiguous	1	1	N-Term(iTRAQ4plex); K12(iTRAQ4plex)	HCD	1.0000	0.0000	1	1	9521	Unique	Used	0.640	2.818E3	2.181E3	236	4.16	0.00	0	53	271	3	532.30322	1594.89511	-5.77	39.51	3937	3937	MS2	16/44	Glioma_Nuclear_prep_Gradeall_repeat_4plex.raw
6005721	High		vAQVAEITYGQk	Unambiguous	1	1	N-Term(iTRAQ4plex); K12(iTRAQ4plex)	HCD	1.0000	0.0000	1	1	9030	Unique	Used	0.603	6.093E4	4.482E4	243	4.94	0.00	0	9	48	2	797.94379	1594.88030	-15.06	33.73	3571	3571	MS2	18/22	Glioma_Nuclear_prep_Gradeall_4plex.raw
6005721	High		iSFGLEDEPLETATk	Unambiguous	1	1	N-Term(iTRAQ4plex); K15(iTRAQ4plex)	HCD	1.0000	0.0000	1	1	10825	Unique	Used	0.610	8.911E4	6.626E4	173	6.95	0.00	0	12	35	2	969.50555	1938.00383	-14.05	47.11	5531	5531	MS2	24/28	Glioma_Nuclear_prep_Gradeall_4plex.raw
7706501	NP_057396.1	WBP11	51729	WW domain-binding protein 11 [Homo sapiens]	7.64	1	1	2	1.952	1;1	1.6	15.65	641	70.0	8.38																			
7706501		High	aVSILPLLGHGVPR	Unambiguous	1	1	N-Term(iTRAQ4plex)	HCD	1.0000	0.0000	1	1	11781	Unique	Used	1.975	2.440E2	5.830E2	54	4.29	0.00	0	21	300	3	524.99542	1572.97171	0.65	68.22	6473	6473	MS2	27/52	Glioma_Nuclear_prep_Gradeall_repeat_4plex.raw
7706501	High		aVSILPLLGHGVPR	Unambiguous	1	1	N-Term(iTRAQ4plex)	HCD	1.0000	0.0000	1	1	10681	Unique	Used	1.930	2.129E3	5.013E3	73	3.99	0.00	0	29	39	3	524.98700	1572.94645	-15.41	46.01	5374	5374	MS2	26/52	Glioma_Nuclear_prep_Gradeall_4plex.raw
8923444	NP_060308.1	NHP2	55651	H/ACA ribonucleoprotein complex subunit 2 isoform a [Homo sapiens]	36.60	2	4	6	1.812	3;3	7.9	19.22	153	17.2	8.22																			
8923444		High	tYQELLVNQNPIAQPLASR	Unambiguous	2	1	N-Term(iTRAQ4plex)	HCD	1.0000	0.0000	1	1	11631	Unique	Used	1.808	3.712E2	8.120E2	122	5.82	0.00	0	3	300	2	1150.12976	2299.25224	-0.21	65.98	6285	6285	MS2	26/36	Glioma_Nuclear_prep_Gradeall_repeat_4plex.raw
8923444	High		tYQELLVNQNPIAQPLASR	Unambiguous	2	1	N-Term(iTRAQ4plex)	HCD	1.0000	0.0000	1	1	10861	Unique	Used	1.644	2.155E4	4.323E4	145	5.33	0.00	0	2	17	2	1150.11169	2299.21611	-15.93	47.38	5570	5570	MS2	27/36	Glioma_Nuclear_prep_Gradeall_4plex.raw
8923444	High		aDPDGPEAQAEAcSGER	Unambiguous	2	1	N-Term(iTRAQ4plex); C13(Methylthio)	HCD	1.0000	0.0000	1	1	8818	Unique	Used	1.594	3.442E4	6.694E4	4	4.44	0.00	0	18	34	2	946.88135	1892.75542	-16.79	32.21	3341	3341	MS2	22/32	Glioma_Nuclear_prep_Gradeall_4plex.raw
8923444		High	nLPYVYIPSk	Unambiguous	1	1	N-Term(iTRAQ4plex); K10(iTRAQ4plex)	HCD	1.0000	0.0000	1	1	10987	Unique	Used	1.959	5.660E3	1.341E4	210	4.12	0.00	0	21	300	2	741.43561	1481.86394	2.23	56.98	5517	5517	MS2	16/18	Glioma_Nuclear_prep_Gradeall_repeat_4plex.raw
8923444		High	tDLGAAAGSk	Unambiguous	1	1	N-Term(iTRAQ4plex); K10(iTRAQ4plex)	HCD	1.0000	0.0000	1	1	7257	Unique	Used	1.816	3.368E4	7.400E4	221	3.54	0.00	0	0	53	2	589.82434	1178.64141	-17.45	17.01	1540	1540	MS2	17/18	Glioma_Nuclear_prep_Gradeall_repeat_4plex.raw
8923444	High		nLPYVYIPSk	Unambiguous	1	1	N-Term(iTRAQ4plex); K10(iTRAQ4plex)	HCD	1.0000	0.0000	1	1	10164	Unique	Used	1.887	1.775E5	4.086E5	254	3.46	0.00	0	22	10	2	741.42297	1481.83867	-14.82	42.09	4809	4809	MS2	16/18	Glioma_Nuclear_prep_Gradeall_4plex.raw
8923900	NP_061156.1	CMAS	55907	N-acylneuraminate cytidylyltransferase [Homo sapiens]	12.67	1	4	7	0.647	3;4	19.8	19.63	434	48.3	7.93																			
8923900		High	eEGYDSVFSVVR	Unambiguous	1	1	N-Term(iTRAQ4plex)	HCD	1.0000	0.0000	1	1	11058	Unique	Used	0.773	6.205E3	5.800E3	161	4.16	0.00	0	38	300	2	765.88263	1530.75798	1.49	57.91	5598	5598	MS2	21/22	Glioma_Nuclear_prep_Gradeall_repeat_4plex.raw
8923900		High	vGLSGAPADAcSTAQk	Unambiguous	1	1	N-Term(iTRAQ4plex); C11(Methylthio); K16(iTRAQ4plex)	HCD	1.0000	0.0000	1	1	9709	Unique	Used	0.647	5.222E3	4.089E3	188	5.89	0.00	0	7	300	2	905.46283	1809.91838	5.85	41.63	4139	4139	MS2	23/30	Glioma_Nuclear_prep_Gradeall_repeat_4plex.raw
8923900	High		vGLSGAPADAcSTAQk	Unambiguous	1	1	N-Term(iTRAQ4plex); C11(Methylthio); K16(iTRAQ4plex)	HCD	1.0000	0.0000	1	1	9221	Unique	Used	0.477	1.659E5	9.649E4	97	5.45	0.00	0	6	11	2	905.44586	1809.88445	-12.90	35.16	3781	3781	MS2	26/30	Glioma_Nuclear_prep_Gradeall_4plex.raw
8923900		High	eVTEPLNLNPAk	Unambiguous	1	1	N-Term(iTRAQ4plex); K12(iTRAQ4plex)	HCD	1.0000	0.0000	1	1	9671	Unique	Used	0.669	8.276E3	6.702E3	226	4.95	0.00	0	34	300	2	806.96393	1612.92058	3.52	41.20	4099	4099	MS2	20/22	Glioma_Nuclear_prep_Gradeall_repeat_4plex.raw
8923900	High		eVTEPLNLNPAk	Unambiguous	1	1	N-Term(iTRAQ4plex); K12(iTRAQ4plex)	HCD	1.0000	0.0000	1	1	9120	Unique	Used	0.643	1.315E5	1.032E5	239	4.08	0.00	0	20	20	2	806.94867	1612.89006	-15.40	34.43	3671	3671	MS2	19/22	Glioma_Nuclear_prep_Gradeall_4plex.raw
8923900		High	gVEkPPHLAALILAR	Unambiguous	1	1	N-Term(iTRAQ4plex); K4(iTRAQ4plex)	HCD	1.0000	0.0000	1	1	10834	Unique	Used	0.457	4.342E2	2.403E2	21	4.63	0.00	0	77	300	4	469.04745	1873.16799	2.86	55.12	5351	5351	MS2	28/84	Glioma_Nuclear_prep_Gradeall_repeat_4plex.raw
8923900	High		gVEkPPHLAALILAR	Unambiguous	1	1	N-Term(iTRAQ4plex); K4(iTRAQ4plex)	HCD	1.0000	0.0000	1	1	9767	Unique	Used	0.768	6.679E4	6.262E4	42	3.88	0.00	0	23	25	2	937.07251	1873.13774	-13.29	39.16	4376	4376	MS2	17/28	Glioma_Nuclear_prep_Gradeall_4plex.raw
8923942	NP_061118.1	NOP10	55505	H/ACA ribonucleoprotein complex subunit 3 [Homo sapiens]	20.31	1	1	3	1.819	2;1	11.4	8.73	64	7.7	9.99																			
8923942	High		mFLQYYLNEQGDR	Unambiguous	1	1	N-Term(iTRAQ4plex)	HCD	1.0000	0.0000	1	1	11445	Unique	Used	1.946	3.464E4	8.225E4	137	4.94	0.00	0	16	53	2	910.94080	1820.87432	-0.83	52.15	6208	6208	MS2	19/24	Glioma_Nuclear_prep_Gradeall_4plex.raw
8923942		High	mFLQYYLNEQGDR	Unambiguous	1	1	N-Term(iTRAQ4plex); M1(Oxidation)	HCD	1.0000	0.0000	1	1	11470	Unique	Used	1.819	8.466E2	1.863E3	132	3.80	0.00	0	9	300	2	918.94031	1836.87334	1.41	63.76	6095	6095	MS2	16/24	Glioma_Nuclear_prep_Gradeall_repeat_4plex.raw
8923942	High		mFLQYYLNEQGDR	Unambiguous	1	1	N-Term(iTRAQ4plex); M1(Oxidation)	HCD	1.0000	0.0000	1	1	10673	Unique	Used	1.549	3.556E4	6.723E4	90	3.78	0.00	0	8	42	2	918.92694	1836.84661	-13.14	45.94	5365	5365	MS2	18/24	Glioma_Nuclear_prep_Gradeall_4plex.raw
9506713	NP_061856.1	GAR1	54433	H/ACA ribonucleoprotein complex subunit 1 [Homo sapiens]	13.36	1	3	6	1.616	2;4	25.5	22.06	217	22.3	10.92																			
9506713		High	vDEIFGQLR	Unambiguous	1	1	N-Term(iTRAQ4plex)	HCD	1.0000	0.0000	1	1	11180	Unique	Used	1.364	1.114E4	1.839E4	349	3.25	0.00	0	38	300	2	610.84186	1220.67644	0.68	59.54	5739	5739	MS2	14/16	Glioma_Nuclear_prep_Gradeall_repeat_4plex.raw
9506713		High	cTTDENkVPYFNAPVYLENk	Unambiguous	1	1	N-Term(iTRAQ4plex); C1(Methylthio); K7(iTRAQ4plex); K20(iTRAQ4plex)	HCD	1.0000	0.0000	1	1	11564	Unique	Used	1.292	1.527E2	2.386E2	78	4.76	0.00	1	9	300	3	941.80634	2823.40445	-0.48	65.05	6206	6206	MS2	29/76	Glioma_Nuclear_prep_Gradeall_repeat_4plex.raw
9506713	High		cTTDENkVPYFNAPVYLENk	Unambiguous	1	1	N-Term(iTRAQ4plex); C1(Methylthio); K7(iTRAQ4plex); K20(iTRAQ4plex)	HCD	1.0000	0.0000	1	1	10612	Unique	Used	1.331	6.426E3	1.044E4	53	5.92	0.00	1	22	26	3	941.79138	2823.35959	-16.37	45.47	5298	5298	MS2	27/76	Glioma_Nuclear_prep_Gradeall_4plex.raw
9506713		High	vPYFNAPVYLENk	Unambiguous	1	1	N-Term(iTRAQ4plex); K13(iTRAQ4plex)	HCD	1.0000	0.0000	1	1	11741	Unique	Used	2.367	1.248E3	3.572E3	217	4.56	0.00	0	12	300	2	921.50787	1842.00847	2.41	67.54	6417	6417	MS2	17/24	Glioma_Nuclear_prep_Gradeall_repeat_4plex.raw
9506713		High	vPYFNAPVYLENk	Unambiguous	1	1	N-Term(iTRAQ4plex); K13(iTRAQ4plex)	HCD	1.0000	0.0000	1	1	11738	Unique	Used	1.896	2.840E2	6.514E2	186	5.03	0.00	0	37	300	3	614.67310	1842.00473	0.38	67.50	6414	6414	MS2	21/48	Glioma_Nuclear_prep_Gradeall_repeat_4plex.raw
9506713	High		vPYFNAPVYLENk	Unambiguous	1	1	N-Term(iTRAQ4plex); K13(iTRAQ4plex)	HCD	1.0000	0.0000	1	1	10899	Unique	Used	1.867	3.795E4	8.644E4	245	5.38	0.00	0	39	31	2	921.49365	1841.98003	-13.03	47.67	5611	5611	MS2	17/24	Glioma_Nuclear_prep_Gradeall_4plex.raw
10800138	NP_066407.1	HIST1H2BD	3017	histone H2B type 1-D [Homo sapiens]	42.06	11	1	4	1.553	2;2	19.1	69.78	126	13.9	10.32																			
10800138		High	eSYSVYVYk	Unambiguous	10	1	N-Term(iTRAQ4plex); K9(iTRAQ4plex)	HCD	1.0000	0.0000	1	1	9542	Unique	Used	2.075	8.585E3	2.155E4	183	4.08	0.00	0	27	90	2	713.37048	1425.73369	-11.72	39.77	3961	3961	MS2	16/16	Glioma_Nuclear_prep_Gradeall_repeat_4plex.raw
10800138		High	eSYSVYVYk	Unambiguous	10	1	N-Term(iTRAQ4plex); K9(iTRAQ4plex)	HCD	1.0000	0.0000	1	1	9638	Unique	Used	1.358	4.043E3	6.643E3	222	4.22	0.00	0	29	300	2	713.37665	1425.74602	-3.07	40.83	4064	4064	MS2	15/16	Glioma_Nuclear_prep_Gradeall_repeat_4plex.raw
10800138	High		eSYSVYVYk	Unambiguous	10	1	N-Term(iTRAQ4plex); K9(iTRAQ4plex)	HCD	1.0000	0.0000	1	1	9009	Unique	Used	1.618	5.070E4	1.001E5	182	4.10	0.00	0	20	23	2	713.36597	1425.72466	-18.05	33.58	3548	3548	MS2	16/16	Glioma_Nuclear_prep_Gradeall_4plex.raw
10800138	High		eSYSVYVYk	Unambiguous	10	1	N-Term(iTRAQ4plex); K9(iTRAQ4plex)	HCD	1.0000	0.0000	1	1	9093	Unique	Used	1.489	8.509E4	1.545E5	173	3.72	0.00	0	21	9	2	713.36658	1425.72588	-17.20	34.24	3642	3642	MS2	16/16	Glioma_Nuclear_prep_Gradeall_4plex.raw
10800140	NP_066406.1	HIST1H2BB	3018	histone H2B type 1-B [Homo sapiens]	42.06	6	2	3	1.755	2;1	18.4	69.41	126	13.9	10.32																			
10800140	High		kESYSIYVYk	Unambiguous	5	1	N-Term(iTRAQ4plex); K1(iTRAQ4plex); K10(iTRAQ4plex)	HCD	1.0000	0.0000	1	1	8667	Unique	Used	1.755	4.290E4	9.185E4	170	3.34	0.00	1	26	15	2	856.47137	1711.93547	-16.13	31.14	3178	3178	MS2	18/18	Glioma_Nuclear_prep_Gradeall_4plex.raw
10800140		High	eSYSIYVYk	Unambiguous	5	1	N-Term(iTRAQ4plex); K9(iTRAQ4plex)	HCD	1.0000	0.0000	1	1	10054	Unique	Used	1.906	9.556E3	2.203E4	255	4.37	0.00	0	48	226	2	720.38745	1439.76763	1.10	45.74	4512	4512	MS2	15/16	Glioma_Nuclear_prep_Gradeall_repeat_4plex.raw
10800140	High		eSYSIYVYk	Unambiguous	5	1	N-Term(iTRAQ4plex); K9(iTRAQ4plex)	HCD	1.0000	0.0000	1	1	9411	Unique	Used	1.318	1.247E5	2.004E5	219	4.11	0.00	0	26	7	2	720.37354	1439.73979	-18.23	36.59	3990	3990	MS2	15/16	Glioma_Nuclear_prep_Gradeall_4plex.raw
10800144	NP_066544.1	HIST1H2AJ	8331	histone cluster 1, H2aj [Homo sapiens]	27.34	11	1	5	2.115	4;1	12.1	65.65	128	13.9	10.89																			
10800144		High	vTIAQGGVLPNIQAVLLPk	Unambiguous	10	1	N-Term(iTRAQ4plex); K19(iTRAQ4plex)	HCD	1.0000	0.0000	1	1	12453	Unique	Used	2.282	6.354E1	1.754E2	24	4.44	0.00	0	2	300	2	1110.18982	2219.37236	-0.30	88.19	7944	7944	MS2	19/36	Glioma_Nuclear_prep_Gradeall_repeat_4plex.raw
10800144	High		vTIAQGGVLPNIQAVLLPk	Unambiguous	10	1	N-Term(iTRAQ4plex); K19(iTRAQ4plex)	HCD	1.0000	0.0000	1	1	12404	Unique	Used	2.138	2.046E3	5.338E3	25	7.87	0.00	0	3	249	2	1110.18555	2219.36382	-4.15	61.18	7256	7256	MS2	23/36	Glioma_Nuclear_prep_Gradeall_4plex.raw
10800144	High		vTIAQGGVLPNIQAVLLPk	Unambiguous	10	1	N-Term(iTRAQ4plex); K19(iTRAQ4plex)	HCD	1.0000	0.0000	1	1	12457	Unique	Used	2.115	2.333E4	6.019E4	23	8.00	0.00	0	2	13	2	1110.18958	2219.37187	-0.52	61.81	7316	7316	MS2	27/36	Glioma_Nuclear_prep_Gradeall_4plex.raw
10800144	High		vTIAQGGVLPNIQAVLLPk	Unambiguous	10	1	N-Term(iTRAQ4plex); K19(iTRAQ4plex)	HCD	1.0000	0.0000	1	1	12514	Unique	Used	1.861	2.084E3	4.730E3	21	7.78	0.00	0	3	185	2	1110.18896	2219.37065	-1.07	62.53	7381	7381	MS2	24/36	Glioma_Nuclear_prep_Gradeall_4plex.raw
10800144	High		vTIAQGGVLPNIQAVLLPk	Unambiguous	10	1	N-Term(iTRAQ4plex); K19(iTRAQ4plex)	HCD	1.0000	0.0000	1	1	12498	Unique	Used	1.671	2.104E3	4.291E3	20	5.94	0.00	0	6	14	3	740.46484	2219.37998	3.13	62.33	7363	7363	MS2	28/72	Glioma_Nuclear_prep_Gradeall_4plex.raw
10835240	NP_006344.1	HMGN4	10473	high mobility group nucleosome-binding domain-containing protein 4 [Homo sapiens]	10.00	1	1	2	1.757	1;1	1.9	6.73	90	9.5	10.48																			
10835240		High	dASTLQSQk	Unambiguous	1	1	N-Term(iTRAQ4plex); K9(iTRAQ4plex)	HCD	1.0000	0.0000	1	1	7017	Unique	Used	1.733	9.659E4	2.025E5	289	3.66	0.00	0	8	19	2	633.34735	1265.68743	-5.18	14.97	1286	1286	MS2	16/16	Glioma_Nuclear_prep_Gradeall_repeat_4plex.raw
10835240	High		dASTLQSQk	Unambiguous	1	1	N-Term(iTRAQ4plex); K9(iTRAQ4plex)	HCD	1.0000	0.0000	1	1	7227	Unique	Used	1.781	5.068E4	1.101E5	317	3.99	0.00	0	22	54	2	633.34766	1265.68804	-4.69	20.76	1612	1612	MS2	16/16	Glioma_Nuclear_prep_Gradeall_4plex.raw
10863927	NP_066953.1	PPIA	5478	peptidyl-prolyl cis-trans isomerase A isoform 1 [Homo sapiens]	19.39	3	3	5	1.950	3;2	9.4	10.67	165	18.0	7.81																			
10863927	High		iIPGFMcQGGDFTR	Unambiguous	2	1	N-Term(iTRAQ4plex); C7(Methylthio)	HCD	1.0000	0.0000	1	1	12014	Unique	Used	2.129	2.883E4	7.488E4	108	3.40	0.00	0	57	132	2	866.41138	1731.81548	0.97	57.13	6828	6828	MS2	12/26	Glioma_Nuclear_prep_Gradeall_4plex.raw
10863927		High	vSFELFADk	Unambiguous	1	1	N-Term(iTRAQ4plex); K9(iTRAQ4plex)	HCD	1.0000	0.0000	1	1	11661	Unique	Used	1.965	3.151E3	7.490E3	232	3.97	0.00	0	26	300	2	672.37524	1343.74321	-1.31	66.45	6324	6324	MS2	16/16	Glioma_Nuclear_prep_Gradeall_repeat_4plex.raw
10863927		High	fEDENFILk	Unambiguous	2	1	N-Term(iTRAQ4plex); K9(iTRAQ4plex)	HCD	1.0000	0.0000	1	1	11065	Unique	Used	1.950	4.923E3	1.162E4	223	3.46	0.00	0	36	300	2	721.89392	1442.78057	2.47	58.00	5606	5606	MS2	16/16	Glioma_Nuclear_prep_Gradeall_repeat_4plex.raw
10863927	High		fEDENFILk	Unambiguous	2	1	N-Term(iTRAQ4plex); K9(iTRAQ4plex)	HCD	1.0000	0.0000	1	1	10234	Unique	Used	1.810	1.200E5	2.651E5	163	3.25	0.00	0	33	13	2	721.88068	1442.75408	-15.89	42.61	4885	4885	MS2	16/16	Glioma_Nuclear_prep_Gradeall_4plex.raw
10863927	High		vSFELFADk	Unambiguous	1	1	N-Term(iTRAQ4plex); K9(iTRAQ4plex)	HCD	1.0000	0.0000	1	1	10769	Unique	Used	1.655	5.468E4	1.104E5	246	4.03	0.00	0	52	38	2	672.36462	1343.72197	-17.11	46.68	5470	5470	MS2	16/16	Glioma_Nuclear_prep_Gradeall_4plex.raw
11067747	NP_001244.1	CDC5L	988	cell division cycle 5-like protein [Homo sapiens]	14.34	1	4	6	0.668	4;2	10.2	42.26	802	92.2	8.18																			
11067747	High		eIDDTYIEDAADVDAR	Unambiguous	1	1	N-Term(iTRAQ4plex)	HCD	1.0000	0.0000	1	1	10255	Unique	Used	0.678	8.394E4	6.947E4	45	5.02	0.00	0	7	18	2	977.93860	1954.86992	-15.33	42.75	4907	4907	MS2	26/30	Glioma_Nuclear_prep_Gradeall_4plex.raw
11067747		High	lGLLGLPAPk	Unambiguous	1	1	N-Term(iTRAQ4plex); K10(iTRAQ4plex)	HCD	1.0000	0.0000	1	1	11479	Unique	Used	0.775	5.306E3	4.975E3	37	3.72	0.00	0	61	300	2	633.92383	1266.84038	1.23	63.87	6105	6105	MS2	17/18	Glioma_Nuclear_prep_Gradeall_repeat_4plex.raw
11067747	High		lGLLGLPAPk	Unambiguous	1	1	N-Term(iTRAQ4plex); K10(iTRAQ4plex)	HCD	1.0000	0.0000	1	1	10581	Unique	Used	0.661	1.408E5	1.135E5	54	3.64	0.00	0	36	14	2	633.91388	1266.82048	-14.47	45.25	5265	5265	MS2	17/18	Glioma_Nuclear_prep_Gradeall_4plex.raw
11067747		High	lVLPAPQISDAELQEVVk	Unambiguous	1	1	N-Term(iTRAQ4plex); K18(iTRAQ4plex)	HCD	1.0000	0.0000	1	1	12318	Unique	Used	0.575	1.523E2	1.060E2	69	4.90	0.00	0	20	300	3	746.43872	2237.30161	0.90	79.53	7353	7353	MS2	29/68	Glioma_Nuclear_prep_Gradeall_repeat_4plex.raw
11067747	High		lVLPAPQISDAELQEVVk	Unambiguous	1	1	N-Term(iTRAQ4plex); K18(iTRAQ4plex)	HCD	1.0000	0.0000	1	1	11860	Unique	Used	0.615	1.923E4	1.442E4	93	5.76	0.00	0	13	86	2	1119.14880	2237.29033	-4.14	55.72	6659	6659	MS2	23/34	Glioma_Nuclear_prep_Gradeall_4plex.raw
11067747	High		eSDLPSAILQTSGVSEFTk	Unambiguous	1	1	N-Term(iTRAQ4plex); K19(iTRAQ4plex)	HCD	1.0000	0.0000	1	1	11540	Unique	Used	0.675	8.004E3	6.593E3	122	6.68	0.00	0	7	142	2	1149.10608	2297.20488	-2.91	52.92	6311	6311	MS2	26/36	Glioma_Nuclear_prep_Gradeall_4plex.raw
11225260	NP_003277.1	TOP1	7150	DNA topoisomerase 1 [Homo sapiens]	12.42	3	6	8	1.682	4;4	13.7	25.37	765	90.7	9.31																			
11225260	High		cDFTQMSQYFk	Unambiguous	1	1	N-Term(iTRAQ4plex); C1(Methylthio); K11(iTRAQ4plex)	HCD	1.0000	0.0000	1	1	11940	Unique	Used	1.757	2.399E4	5.144E4	54	5.02	0.00	0	42	115	2	866.39508	1731.78288	2.63	56.40	6746	6746	MS2	19/20	Glioma_Nuclear_prep_Gradeall_4plex.raw
11225260	High		iMPEDIIINcSk	Unambiguous	1	1	N-Term(iTRAQ4plex); C10(Methylthio); K12(iTRAQ4plex)	HCD	1.0000	0.0000	1	1	11954	Unique	Used	1.654	2.155E3	4.349E3	238	3.73	0.00	0	23	66	3	570.63281	1709.88388	-2.35	56.54	6762	6762	MS2	19/44	Glioma_Nuclear_prep_Gradeall_4plex.raw
11225260	High		iMPEDIIINcSk	Unambiguous	1	1	N-Term(iTRAQ4plex); C10(Methylthio); K12(iTRAQ4plex)	HCD	1.0000	0.0000	1	1	11948	Unique	Used	1.384	1.974E4	3.333E4	273	4.81	0.00	0	38	152	2	855.44910	1709.89092	1.76	56.47	6755	6755	MS2	18/22	Glioma_Nuclear_prep_Gradeall_4plex.raw
11225260		High	iTVAWck	Unambiguous	1	1	N-Term(iTRAQ4plex); C6(Methylthio); K7(iTRAQ4plex)	HCD	1.0000	0.0000	1	1	11310	Unique	Used	1.505	4.160E3	7.575E3	240	2.86	0.00	0	59	300	2	577.81921	1154.63115	0.60	61.46	5900	5900	MS2	12/12	Glioma_Nuclear_prep_Gradeall_repeat_4plex.raw
11225260		High	gPVFAPPYEPLPENVk	Unambiguous	1	1	N-Term(iTRAQ4plex); K16(iTRAQ4plex)	HCD	1.0000	0.0000	1	1	11346	Unique	Used	2.113	6.586E2	1.683E3	167	4.27	0.00	0	12	300	2	1021.56476	2042.12224	1.02	62.07	5949	5949	MS2	19/30	Glioma_Nuclear_prep_Gradeall_repeat_4plex.raw
11225260	High		gPVFAPPYEPLPENVk	Unambiguous	1	1	N-Term(iTRAQ4plex); K16(iTRAQ4plex)	HCD	1.0000	0.0000	1	1	10510	Unique	Used	1.709	4.554E4	9.497E4	190	4.58	0.00	0	2	11	2	1021.54779	2042.08830	-15.60	44.73	5188	5188	MS2	21/30	Glioma_Nuclear_prep_Gradeall_4plex.raw
11225260		High	nIITNLSk	Unambiguous	1	1	N-Term(iTRAQ4plex); K8(iTRAQ4plex)	HCD	1.0000	0.0000	1	1	9084	Unique	Used	1.780	1.562E4	3.364E4	173	2.84	0.00	0	58	300	2	595.87122	1190.73516	0.37	34.69	3472	3472	MS2	13/14	Glioma_Nuclear_prep_Gradeall_repeat_4plex.raw
11225260		High	qIALGTSk	Unambiguous	1	1	N-Term(iTRAQ4plex); K8(iTRAQ4plex)	HCD	1.0000	0.0000	1	1	7830	Unique	Used	1.470	2.042E4	3.632E4	331	2.96	0.00	0	44	165	2	553.34253	1105.67778	-3.78	22.15	2147	2147	MS2	13/14	Glioma_Nuclear_prep_Gradeall_repeat_4plex.raw
11386147	NP_002769.1	PSAP	5660	prosaposin isoform a preproprotein [Homo sapiens]	3.82	3	2	3	0.641	2;1	16.7	6.48	524	58.1	5.17																			
11386147		High	gcSFLPDPYQk	Unambiguous	3	1	N-Term(iTRAQ4plex); C2(Methylthio); K11(iTRAQ4plex)	HCD	1.0000	0.0000	1	1	11531	Unique	Used	0.475	2.733E3	1.571E3	101	3.40	0.00	0	46	300	2	794.89221	1588.77715	1.84	64.54	6163	6163	MS2	16/20	Glioma_Nuclear_prep_Gradeall_repeat_4plex.raw
11386147	High		gcSFLPDPYQk	Unambiguous	3	1	N-Term(iTRAQ4plex); C2(Methylthio); K11(iTRAQ4plex)	HCD	1.0000	0.0000	1	1	10720	Unique	Used	0.649	5.155E4	4.079E4	81	3.19	0.00	0	60	41	2	794.87885	1588.75041	-14.99	46.29	5416	5416	MS2	13/20	Glioma_Nuclear_prep_Gradeall_4plex.raw
11386147	High		qEILAALEk	Unambiguous	3	1	N-Term(iTRAQ4plex); K9(iTRAQ4plex)	HCD	1.0000	0.0000	1	1	9939	Unique	Used	0.641	1.132E5	8.850E4	260	3.29	0.00	0	62	28	2	651.88641	1302.76555	-16.59	40.41	4563	4563	MS2	16/16	Glioma_Nuclear_prep_Gradeall_4plex.raw
13236587	NP_077310.1	TMEM43	79188	transmembrane protein 43 [Homo sapiens]	2.50	1	1	2	1.757	1;1	5.3	3.64	400	44.8	8.13																			
13236587		High	fFLSSGLIDk	Unambiguous	1	1	N-Term(iTRAQ4plex); K10(iTRAQ4plex)	HCD	1.0000	0.0000	1	1	11832	Unique	Used	1.823	1.974E3	4.355E3	234	3.62	0.00	0	27	300	2	707.91193	1414.81658	-1.34	69.14	6547	6547	MS2	18/18	Glioma_Nuclear_prep_Gradeall_repeat_4plex.raw
13236587	High		fFLSSGLIDk	Unambiguous	1	1	N-Term(iTRAQ4plex); K10(iTRAQ4plex)	HCD	1.0000	0.0000	1	1	11040	Unique	Used	1.690	4.297E4	8.864E4	252	3.64	0.00	0	37	42	2	707.91046	1414.81365	-3.41	48.75	5764	5764	MS2	18/18	Glioma_Nuclear_prep_Gradeall_4plex.raw
13676857	NP_068814.2	HSPA2	3306	heat shock-related 70 kDa protein 2 [Homo sapiens]	13.77	1	2	2	0.341	1;1	14.6	27.89	639	70.0	5.74																			
13676857	High		cQEVINWLDR	Unambiguous	1	1	N-Term(iTRAQ4plex); C1(Methylthio)	HCD	1.0000	0.0000	1	1	12170	Unique	Used	0.377	2.892E4	1.330E4	132	3.67	0.00	0	24	241	2	733.35657	1465.70586	0.67	58.66	6998	6998	MS2	16/18	Glioma_Nuclear_prep_Gradeall_4plex.raw
13676857		High	vcNPIISk	Unambiguous	1	1	N-Term(iTRAQ4plex); C2(Methylthio); K8(iTRAQ4plex)	HCD	1.0000	0.0000	1	1	9805	Unique	Used	0.306	1.984E4	7.349E3	357	3.28	0.00	0	69	300	2	604.34686	1207.68645	6.88	42.72	4240	4240	MS2	13/14	Glioma_Nuclear_prep_Gradeall_repeat_4plex.raw

14043072	NP_112533.1	HNRNPA2B1	3181	heterogeneous nuclear ribonucleoproteins A2/B1 isoform B1 [Homo sapiens]	46.74	2	11	28	0.649	13;15	11.1	112.17	353	37.4	8.95																			
14043072		High	nMGGPYGGGNYGPGGSGGSGGYGGR	Unambiguous	2	1	N-Term(iTRAQ4plex)	HCD	1.0000	0.0000	1	1	8851	Unique	Used	0.592	2.137E3	1.530E3	22	8.92	0.00	0	14	179	3	778.67645	2334.01480	3.12	32.16	3222	3222	MS2	28/96	Glioma_Nuclear_prep_Gradeall_repeat_4plex.raw
14043072		High	iDTIEIITDR	Unambiguous	2	1	N-Term(iTRAQ4plex)	HCD	1.0000	0.0000	1	1	11150	Unique	Used	0.588	1.558E5	1.108E5	221	4.00	0.00	0	7	30	2	666.88007	1332.75286	2.77	59.14	5704	5704	MS2	17/18	Glioma_Nuclear_prep_Gradeall_repeat_4plex.raw
14043072		High	nMGGPYGGGNYGPGGSGGSGGYGGR	Unambiguous	2	1	N-Term(iTRAQ4plex)	HCD	1.0000	0.0000	1	1	8860	Unique	Used	0.636	1.876E4	1.443E4	17	7.78	0.00	0	0	115	2	1167.50415	2334.00102	-2.78	32.25	3232	3232	MS2	33/48	Glioma_Nuclear_prep_Gradeall_repeat_4plex.raw
14043072	High		lFIGGLSFETTEESLR	Unambiguous	2	1	N-Term(iTRAQ4plex)	HCD	1.0000	0.0000	1	1	12382	Unique	Used	0.588	2.532E4	1.816E4	228	5.43	0.00	0	19	293	2	972.01550	1943.02373	-0.30	60.94	7232	7232	MS2	23/30	Glioma_Nuclear_prep_Gradeall_4plex.raw
14043072	High		iDTIEIITDR	Unambiguous	2	1	N-Term(iTRAQ4plex)	HCD	1.0000	0.0000	1	1	10357	Unique	Used	0.591	9.457E6	6.819E6	198	3.74	0.00	0	7	1	2	666.86932	1332.73137	-13.35	43.52	5019	5019	MS2	17/18	Glioma_Nuclear_prep_Gradeall_4plex.raw
14043072	High		lFIGGLSFETTEESLR	Unambiguous	2	1	N-Term(iTRAQ4plex)	HCD	1.0000	0.0000	1	1	12251	Unique	Used	0.602	6.302E4	4.630E4	208	5.65	0.00	0	15	116	2	972.01471	1943.02214	-1.11	59.51	7088	7088	MS2	22/30	Glioma_Nuclear_prep_Gradeall_4plex.raw
14043072	High		lFIGGLSFETTEESLR	Unambiguous	2	1	N-Term(iTRAQ4plex)	HCD	1.0000	0.0000	1	1	12314	Unique	Used	0.644	1.086E5	8.531E4	203	5.62	0.00	0	31	64	2	972.01392	1943.02056	-1.93	60.20	7158	7158	MS2	22/30	Glioma_Nuclear_prep_Gradeall_4plex.raw
14043072	High		nMGGPYGGGNYGPGGSGGSGGYGGR	Unambiguous	2	1	N-Term(iTRAQ4plex)	HCD	1.0000	0.0000	1	1	8580	Unique	Used	0.685	1.091E5	9.118E4	8	6.76	0.00	0	0	17	2	1167.48657	2333.96587	-17.85	30.46	3081	3081	MS2	35/48	Glioma_Nuclear_prep_Gradeall_4plex.raw
14043072	High		gGGGNFGPGPGSNFR	Unambiguous	2	1	N-Term(iTRAQ4plex)	HCD	1.0000	0.0000	1	1	8224	Unique	Used	0.777	2.390E5	2.265E5	63	5.36	0.00	0	14	15	2	761.35645	1521.70561	-17.07	27.93	2695	2695	MS2	22/28	Glioma_Nuclear_prep_Gradeall_4plex.raw
14043072		High	lTDcVVMR	Unambiguous	2	1	N-Term(iTRAQ4plex); C4(Methylthio)	HCD	1.0000	0.0000	1	1	10778	Unique	Used	0.522	1.356E4	8.562E3	157	2.86	0.00	0	40	300	2	563.78253	1126.55779	3.38	54.39	5288	5288	MS2	12/14	Glioma_Nuclear_prep_Gradeall_repeat_4plex.raw
14043072		High	lTDcVVMR	Unambiguous	2	1	N-Term(iTRAQ4plex); C4(Methylthio)	HCD	1.0000	0.0000	1	1	10860	Unique	Used	0.564	2.349E4	1.602E4	146	3.01	0.00	0	18	288	2	563.78192	1126.55657	2.30	55.43	5379	5379	MS2	13/14	Glioma_Nuclear_prep_Gradeall_repeat_4plex.raw
14043072	High		kLFIGGLSFETTEESLR	Unambiguous	2	1	N-Term(iTRAQ4plex); K1(iTRAQ4plex)	HCD	1.0000	0.0000	1	1	11185	Unique	Used	0.739	1.127E4	1.016E4	105	6.65	0.00	1	28	29	3	739.07678	2215.21579	-2.50	49.91	5923	5923	MS2	27/64	Glioma_Nuclear_prep_Gradeall_4plex.raw
14043072		High	kLFVGGIk	Unambiguous	2	1	N-Term(iTRAQ4plex); K1(iTRAQ4plex); K8(iTRAQ4plex)	HCD	1.0000	0.0000	1	1	8942	Unique	Used	0.653	5.273E4	4.167E4	26	3.03	0.00	1	55	142	2	647.43115	1293.85503	-5.30	33.03	3318	3318	MS2	14/14	Glioma_Nuclear_prep_Gradeall_repeat_4plex.raw
14043072		High	kLFVGGIk	Unambiguous	2	1	N-Term(iTRAQ4plex); K1(iTRAQ4plex); K8(iTRAQ4plex)	HCD	1.0000	0.0000	1	1	8749	Unique	Used	0.665	1.310E4	1.054E4	27	3.46	0.00	1	66	115	3	431.95746	1293.85782	-3.14	31.06	3115	3115	MS2	19/28	Glioma_Nuclear_prep_Gradeall_repeat_4plex.raw
14043072		High	kLFVGGIk	Unambiguous	2	1	N-Term(iTRAQ4plex); K1(iTRAQ4plex); K8(iTRAQ4plex)	HCD	1.0000	0.0000	1	1	9099	Unique	Used	0.684	4.237E3	3.505E3	21	3.61	0.00	1	75	240	3	431.95746	1293.85782	-3.14	34.85	3488	3488	MS2	19/28	Glioma_Nuclear_prep_Gradeall_repeat_4plex.raw
14043072		High	kLFVGGIk	Unambiguous	2	1	N-Term(iTRAQ4plex); K1(iTRAQ4plex); K8(iTRAQ4plex)	HCD	1.0000	0.0000	1	1	8925	Unique	Used	0.728	1.519E4	1.338E4	22	3.38	0.00	1	79	157	3	431.96130	1293.86936	5.77	32.86	3300	3300	MS2	20/28	Glioma_Nuclear_prep_Gradeall_repeat_4plex.raw
14043072	High		kLFVGGIk	Unambiguous	2	1	N-Term(iTRAQ4plex); K1(iTRAQ4plex); K8(iTRAQ4plex)	HCD	1.0000	0.0000	1	1	8346	Unique	Used	0.543	2.189E5	1.449E5	26	3.63	0.00	1	59	3	3	431.95651	1293.85498	-5.34	28.81	2828	2828	MS2	19/28	Glioma_Nuclear_prep_Gradeall_4plex.raw
14043072		High	gFGFVTFDDHDPVDk	Unambiguous	2	1	N-Term(iTRAQ4plex); K15(iTRAQ4plex)	HCD	1.0000	0.0000	1	1	11238	Unique	Used	0.708	1.030E4	8.825E3	104	4.58	0.00	0	8	96	3	661.99634	1983.97446	2.68	60.29	5805	5805	MS2	27/56	Glioma_Nuclear_prep_Gradeall_repeat_4plex.raw
14043072		High	gFGFVTFDDHDPVDk	Unambiguous	2	1	N-Term(iTRAQ4plex); K15(iTRAQ4plex)	HCD	1.0000	0.0000	1	1	11302	Unique	Used	0.714	6.564E3	5.671E3	99	5.13	0.00	0	8	139	3	661.99646	1983.97483	2.87	61.30	5888	5888	MS2	29/56	Glioma_Nuclear_prep_Gradeall_repeat_4plex.raw
14043072	High		gFGFVTFDDHDPVDk	Unambiguous	2	1	N-Term(iTRAQ4plex); K15(iTRAQ4plex)	HCD	1.0000	0.0000	1	1	10462	Unique	Used	0.713	3.079E4	2.678E4	61	4.01	0.00	0	51	15	3	661.98358	1983.93619	-16.61	44.35	5135	5135	MS2	27/56	Glioma_Nuclear_prep_Gradeall_4plex.raw
14043072	High		gFGFVTFDDHDPVDk	Unambiguous	2	1	N-Term(iTRAQ4plex); K15(iTRAQ4plex)	HCD	1.0000	0.0000	1	1	10340	Unique	Used	0.739	8.865E5	7.990E5	71	4.36	0.00	0	8	1	3	661.98633	1983.94443	-12.45	43.38	5000	5000	MS2	29/56	Glioma_Nuclear_prep_Gradeall_4plex.raw
14043072	High		gFGFVTFDDHDPVDk	Unambiguous	2	1	N-Term(iTRAQ4plex); K15(iTRAQ4plex)	HCD	1.0000	0.0000	1	1	10414	Unique	Used	0.740	1.287E5	1.161E5	73	5.45	0.00	0	15	15	2	992.47351	1983.93974	-14.82	43.95	5081	5081	MS2	25/28	Glioma_Nuclear_prep_Gradeall_4plex.raw
14043072	High		eESGkPGAHVTVk	Unambiguous	2	1	N-Term(iTRAQ4plex); K5(iTRAQ4plex); K13(iTRAQ4plex)	HCD	1.0000	0.0000	1	1	7223	Unique	Used	0.751	3.951E4	3.620E4	151	4.24	0.00	0	10	67	2	886.00848	1771.00969	1.26	20.73	1608	1608	MS2	18/24	Glioma_Nuclear_prep_Gradeall_4plex.raw
14043072		High	dYFEEYGk	Unambiguous	2	1	N-Term(iTRAQ4plex); K8(iTRAQ4plex)	HCD	1.0000	0.0000	1	1	9635	Unique	Used	0.631	7.409E4	5.657E4	88	3.61	0.00	0	36	157	2	669.82617	1338.64507	-0.41	40.80	4061	4061	MS2	13/14	Glioma_Nuclear_prep_Gradeall_repeat_4plex.raw
14043072		High	nYYEQWGk	Unambiguous	2	1	N-Term(iTRAQ4plex); K8(iTRAQ4plex)	HCD	1.0000	0.0000	1	1	9311	Unique	Used	0.731	2.090E4	1.848E4	145	3.05	0.00	0	71	300	2	688.34686	1375.68645	-1.47	37.21	3711	3711	MS2	12/14	Glioma_Nuclear_prep_Gradeall_repeat_4plex.raw
14043072	High		dYFEEYGk	Unambiguous	2	1	N-Term(iTRAQ4plex); K8(iTRAQ4plex)	HCD	1.0000	0.0000	1	1	9232	Unique	Used	0.587	3.931E6	2.816E6	50	2.92	0.00	0	9	1	2	669.81757	1338.62786	-13.27	35.24	3793	3793	MS2	14/14	Glioma_Nuclear_prep_Gradeall_4plex.raw
14043072		High	nmGGPYGGGNYGPGGSGGSGGYGGR	Unambiguous	2	1	N-Term(iTRAQ4plex); M2(Oxidation)	HCD	1.0000	0.0000	1	1	8294	Unique	Used	0.560	1.213E3	8.218E2	20	5.39	0.00	0	11	102	3	784.01093	2350.01822	6.72	26.65	2636	2636	MS2	29/96	Glioma_Nuclear_prep_Gradeall_repeat_4plex.raw
14043072		High	nmGGPYGGGNYGPGGSGGSGGYGGR	Unambiguous	2	1	N-Term(iTRAQ4plex); M2(Oxidation)	HCD	1.0000	0.0000	1	1	8283	Unique	Used	0.633	6.744E3	5.168E3	21	5.99	0.00	0	0	288	2	1175.51099	2350.01470	5.22	26.52	2624	2624	MS2	29/48	Glioma_Nuclear_prep_Gradeall_repeat_4plex.raw
14165466	NP_114368.1	PTBP1	5725	polypyrimidine tract-binding protein 1 isoform c [Homo sapiens]	17.51	9	6	8	2.022	4;4	38.9	31.83	531	57.2	9.17																			
14165466	High		iAIPGLAGAGNSVLLVSNLNPER	Unambiguous	3	1	N-Term(iTRAQ4plex)	HCD	1.0000	0.0000	1	1	12565	Unique	Used	1.744	1.489E3	3.167E3	72	9.28	0.00	0	11	67	3	807.13159	2419.38022	0.49	63.21	7440	7440	MS2	32/88	Glioma_Nuclear_prep_Gradeall_4plex.raw
14165466	High		kLPIDVTEGEVISLGLPFGk	Unambiguous	3	1	N-Term(iTRAQ4plex); K1(iTRAQ4plex); K20(iTRAQ4plex)	HCD	1.0000	0.0000	1	1	12528	Unique	Used	1.825	5.589E2	1.244E3	27	9.13	0.00	1	13	300	3	848.83893	2544.50223	0.33	62.73	7398	7398	MS2	27/76	Glioma_Nuclear_prep_Gradeall_4plex.raw
14165466		High	hQNVQLPR	Unambiguous	3	1	N-Term(iTRAQ4plex)	HCD	1.0000	0.0000	1	1	7121	Unique	Used	2.150	4.102E3	1.067E4	310	3.74	0.00	0	23	104	3	379.21384	1135.62696	-16.14	15.87	1397	1397	MS2	17/28	Glioma_Nuclear_prep_Gradeall_repeat_4plex.raw
14165466	High		iAIPGLAGAGNSVLLVSNLNPER	Unambiguous	3	1	N-Term(iTRAQ4plex)	HCD	1.0000	0.0000	1	1	12569	Unique	Used	4.205	2.203E3	1.130E4	61	6.82	0.00	0	0	300	2	1210.18982	2419.37236	-2.76	63.24	7444	7444	MS2	27/44	Glioma_Nuclear_prep_Gradeall_4plex.raw
14165466		High	eGQEDQGLTk	Unambiguous	9	1	N-Term(iTRAQ4plex); K10(iTRAQ4plex)	HCD	1.0000	0.0000	1	1	7170	Unique	Used	1.894	6.247E3	1.431E4	179	2.96	0.00	0	17	140	2	696.86163	1392.71599	-3.55	16.30	1448	1448	MS2	15/18	Glioma_Nuclear_prep_Gradeall_repeat_4plex.raw
14165466		High	gQPIYIQFSNHk	Unambiguous	3	1	N-Term(iTRAQ4plex); K12(iTRAQ4plex)	HCD	1.0000	0.0000	1	1	9572	Unique	Used	2.511	2.785E3	8.461E3	260	4.39	0.00	0	0	300	2	860.46863	1719.92998	-7.04	40.09	3993	3993	MS2	19/22	Glioma_Nuclear_prep_Gradeall_repeat_4plex.raw
14165466		High	gQPIYIQFSNHk	Unambiguous	3	1	N-Term(iTRAQ4plex); K12(iTRAQ4plex)	HCD	1.0000	0.0000	1	1	9543	Unique	Used	2.414	1.747E3	5.102E3	158	6.23	0.00	0	35	170	3	573.97870	1719.92154	-11.94	39.78	3962	3962	MS2	26/44	Glioma_Nuclear_prep_Gradeall_repeat_4plex.raw
14165466	High		nNQFQALLQYADPVSAQHAk	Unambiguous	3	1	N-Term(iTRAQ4plex); K20(iTRAQ4plex)	HCD	1.0000	0.0000	1	1	11255	Unique	Used	1.304	3.103E3	4.938E3	94	6.60	0.00	0	44	64	3	844.44727	2531.32724	1.06	50.47	5999	5999	MS2	28/76	Glioma_Nuclear_prep_Gradeall_4plex.raw
15451775	NP_150249.1	PML	5371	protein PML isoform 7 [Homo sapiens]	2.84	9	1	2	1.723	1;1	17.8	4.02	423	47.5	6.02																			
15451775		High	aPASEEEFQFLR	Unambiguous	9	1	N-Term(iTRAQ4plex)	HCD	1.0000	0.0000	1	1	11136	Unique	Used	1.940	1.639E3	3.846E3	157	4.02	0.00	0	26	300	2	784.39874	1567.79021	1.82	58.96	5688	5688	MS2	19/22	Glioma_Nuclear_prep_Gradeall_repeat_4plex.raw
15451775	High		aPASEEEFQFLR	Unambiguous	9	1	N-Term(iTRAQ4plex)	HCD	1.0000	0.0000	1	1	10286	Unique	Used	1.506	5.206E4	9.566E4	142	3.32	0.00	0	37	26	2	784.38538	1567.76348	-15.23	42.98	4941	4941	MS2	12/22	Glioma_Nuclear_prep_Gradeall_4plex.raw
16507237	NP_005338.1	HSPA5	3309	78 kDa glucose-regulated protein precursor [Homo sapiens]	38.07	1	16	24	0.605	12;12	18.0	82.15	654	72.3	5.16																			
16507237		High	iTPSYVAFTPEGER	Unambiguous	1	1	N-Term(iTRAQ4plex)	HCD	1.0000	0.0000	1	1	10625	Unique	Used	0.543	5.877E3	3.861E3	243	4.43	0.00	0	37	300	2	855.94690	1710.88652	2.66	52.53	5123	5123	MS2	19/26	Glioma_Nuclear_prep_Gradeall_repeat_4plex.raw
16507237		High	iTITNDQNR	Unambiguous	1	1	N-Term(iTRAQ4plex)	HCD	1.0000	0.0000	1	1	7699	Unique	Used	0.609	2.962E4	2.181E4	252	3.29	0.00	0	35	198	2	609.82666	1218.64604	-8.10	20.92	2009	2009	MS2	14/16	Glioma_Nuclear_prep_Gradeall_repeat_4plex.raw
16507237		High	vTHAVVTVPAYFNDAQR	Unambiguous	1	1	N-Term(iTRAQ4plex)	HCD	1.0000	0.0000	1	1	10433	Unique	Used	0.626	1.339E3	1.014E3	260	4.42	0.00	0	8	300	2	1016.54010	2032.07292	-0.19	50.21	4916	4916	MS2	19/32	Glioma_Nuclear_prep_Gradeall_repeat_4plex.raw
16507237		High	sDIDEIVLVGGSTR	Unambiguous	1	1	N-Term(iTRAQ4plex)	HCD	1.0000	0.0000	1	1	10932	Unique	Used	0.745	2.318E3	2.091E3	260	4.53	0.00	0	34	300	2	802.93652	1604.86577	2.81	56.33	5458	5458	MS2	21/26	Glioma_Nuclear_prep_Gradeall_repeat_4plex.raw
16507237	High		iTPSYVAFTPEGER	Unambiguous	1	1	N-Term(iTRAQ4plex)	HCD	1.0000	0.0000	1	1	9942	Unique	Used	0.446	5.099E5	2.772E5	141	4.12	0.00	0	10	5	2	855.93298	1710.85869	-13.61	40.44	4567	4567	MS2	17/26	Glioma_Nuclear_prep_Gradeall_4plex.raw
16507237	High		kSDIDEIVLVGGSTR	Unambiguous	1	1	N-Term(iTRAQ4plex); K1(iTRAQ4plex)	HCD	1.0000	0.0000	1	1	9541	Unique	Used	0.632	6.014E4	4.640E4	172	4.26	0.00	1	0	29	2	939.01959	1877.03191	-14.05	37.54	4131	4131	MS2	21/28	Glioma_Nuclear_prep_Gradeall_4plex.raw
16507237		High	nELESYAYSLk	Unambiguous	1	1	N-Term(iTRAQ4plex); K11(iTRAQ4plex)	HCD	1.0000	0.0000	1	1	11147	Unique	Used	0.537	6.326E3	4.112E3	221	4.94	0.00	0	17	300	2	802.92560	1604.84392	1.80	59.09	5700	5700	MS2	19/20	Glioma_Nuclear_prep_Gradeall_repeat_4plex.raw
16507237	High		nELESYAYSLk	Unambiguous	1	1	N-Term(iTRAQ4plex); K11(iTRAQ4plex)	HCD	1.0000	0.0000	1	1	10296	Unique	Used	0.478	1.599E5	9.322E4	194	4.36	0.00	0	46	20	2	802.91260	1604.81792	-14.40	43.05	4952	4952	MS2	19/20	Glioma_Nuclear_prep_Gradeall_4plex.raw
16507237		High	eLEEIVQPIISk	Unambiguous	1	1	N-Term(iTRAQ4plex); K12(iTRAQ4plex)	HCD	1.0000	0.0000	1	1	11879	Unique	Used	0.601	2.342E3	1.703E3	149	5.04	0.00	0	18	300	2	843.50269	1685.99809	3.13	69.88	6608	6608	MS2	21/22	Glioma_Nuclear_prep_Gradeall_repeat_4plex.raw
16507237	High		eLEEIVQPIISk	Unambiguous	1	1	N-Term(iTRAQ4plex); K12(iTRAQ4plex)	HCD	1.0000	0.0000	1	1	11088	Unique	Used	0.529	6.743E3	4.354E3	136	3.59	0.00	0	48	22	3	562.66681	1685.98587	-4.12	49.15	5817	5817	MS2	13/44	Glioma_Nuclear_prep_Gradeall_4plex.raw
16507237	High		eLEEIVQPIISk	Unambiguous	1	1	N-Term(iTRAQ4plex); K12(iTRAQ4plex)	HCD	1.0000	0.0000	1	1	11087	Unique	Used	0.624	6.900E4	5.256E4	155	5.67	0.00	0	35	33	2	843.49994	1685.99260	-0.13	49.14	5816	5816	MS2	19/22	Glioma_Nuclear_prep_Gradeall_4plex.raw
16507237	High		tFAPEEISAMVLTk	Unambiguous	1	1	N-Term(iTRAQ4plex); K14(iTRAQ4plex)	HCD	1.0000	0.0000	1	1	11766	Unique	Used	0.500	9.449E4	5.762E4	247	5.03	0.00	0	45	50	2	913.00800	1825.00871	3.67	54.92	6557	6557	MS2	24/26	Glioma_Nuclear_prep_Gradeall_4plex.raw
16507237		High	nQLTSNPENTVFDAk	Unambiguous	1	1	N-Term(iTRAQ4plex); K15(iTRAQ4plex)	HCD	1.0000	0.0000	1	1	9893	Unique	Used	0.766	1.298E3	1.203E3	180	4.12	0.00	0	13	300	3	656.01044	1966.01676	2.40	43.69	4333	4333	MS2	18/56	Glioma_Nuclear_prep_Gradeall_repeat_4plex.raw
16507237	High		nQLTSNPENTVFDAk	Unambiguous	1	1	N-Term(iTRAQ4plex); K15(iTRAQ4plex)	HCD	1.0000	0.0000	1	1	9344	Unique	Used	0.368	1.617E5	7.270E4	136	5.34	0.00	0	9	10	2	983.49347	1965.97966	-16.47	36.10	3917	3917	MS2	23/28	Glioma_Nuclear_prep_Gradeall_4plex.raw
16507237	High		nQLTSNPENTVFDAk	Unambiguous	1	1	N-Term(iTRAQ4plex); K15(iTRAQ4plex)	HCD	1.0000	0.0000	1	1	9325	Unique	Used	0.478	9.077E4	5.297E4	162	5.00	0.00	0	34	25	2	983.50903	1966.01079	-0.64	35.96	3896	3896	MS2	19/28	Glioma_Nuclear_prep_Gradeall_4plex.raw
16507237		High	iEWLESHQDADIEDFk	Unambiguous	1	1	N-Term(iTRAQ4plex); K16(iTRAQ4plex)	HCD	1.0000	0.0000	1	1	11766	Unique	Used	0.769	3.688E2	3.433E2	105	4.55	0.00	0	34	300	3	755.04388	2263.11710	2.18	67.99	6454	6454	MS2	24/60	Glioma_Nuclear_prep_Gradeall_repeat_4plex.raw
16507237	High		iEWLESHQDADIEDFk	Unambiguous	1	1	N-Term(iTRAQ4plex); K16(iTRAQ4plex)	HCD	1.0000	0.0000	1	1	10875	Unique	Used	0.686	1.505E4	1.260E4	75	5.38	0.00	0	40	22	3	755.03278	2263.08377	-12.55	47.48	5585	5585	MS2	30/60	Glioma_Nuclear_prep_Gradeall_4plex.raw
16507237	High		sQIFSTASDNQPTVTIk	Unambiguous	1	1	N-Term(iTRAQ4plex); K17(iTRAQ4plex)	HCD	1.0000	0.0000	1	1	9560	Unique	Used	0.556	7.345E4	4.986E4	203	5.63	0.00	0	10	16	2	1063.05640	2125.10552	-15.27	37.68	4152	4152	MS2	26/32	Glioma_Nuclear_prep_Gradeall_4plex.raw
16507237	High		tkPYIQVDIGGGQTk	Unambiguous	1	1	N-Term(iTRAQ4plex); K2(iTRAQ4plex); K15(iTRAQ4plex)	HCD	1.0000	0.0000	1	1	8697	Unique	Used	0.524	5.435E4	3.475E4	107	5.00	0.00	0	0	20	2	1019.07404	2037.14079	-14.58	31.35	3210	3210	MS2	23/28	Glioma_Nuclear_prep_Gradeall_4plex.raw
16507237		High	vLEDSDLk	Unambiguous	1	1	N-Term(iTRAQ4plex); K8(iTRAQ4plex)	HCD	1.0000	0.0000	1	1	8616	Unique	Used	0.654	1.920E4	1.519E4	315	3.04	0.00	0	47	294	2	603.84644	1206.68559	2.96	29.80	2975	2975	MS2	14/14	Glioma_Nuclear_prep_Gradeall_repeat_4plex.raw
16507237		High	eTAEAYLGk	Unambiguous	1	1	N-Term(iTRAQ4plex); K9(iTRAQ4plex)	HCD	1.0000	0.0000	1	1	8671	Unique	Used	0.753	2.550E4	2.324E4	317	3.38	0.00	0	56	170	2	635.35150	1269.69573	2.21	30.37	3033	3033	MS2	15/16	Glioma_Nuclear_prep_Gradeall_repeat_4plex.raw
16507237		High	dAGTIAGLNVmR	Unambiguous	1	1	N-Term(iTRAQ4plex); M11(Oxidation)	HCD	1.0000	0.0000	1	1	9121	Unique	Used	0.704	7.433E3	6.329E3	286	3.64	0.00	0	16	300	2	689.36975	1377.73223	3.26	35.10	3511	3511	MS2	18/22	Glioma_Nuclear_prep_Gradeall_repeat_4plex.raw
16507237	High		dAGTIAGLNVmR	Unambiguous	1	1	N-Term(iTRAQ4plex); M11(Oxidation)	HCD	1.0000	0.0000	1	1	8769	Unique	Used	0.670	9.998E4	8.171E4	223	3.59	0.00	0	9	14	2	689.35663	1377.70598	-15.79	31.86	3288	3288	MS2	17/22	Glioma_Nuclear_prep_Gradeall_4plex.raw
16507237		High	fEELNmDLFR	Unambiguous	1	1	N-Term(iTRAQ4plex); M6(Oxidation)	HCD	1.0000	0.0000	1	1	11669	Unique	Used	0.573	5.569E3	3.862E3	135	2.97	0.00	0	35	300	2	737.36353	1473.71977	2.22	66.56	6333	6333	MS2	15/18	Glioma_Nuclear_prep_Gradeall_repeat_4plex.raw
19923653	NP_150091.2	PSIP1	11168	PC4 and SFRS1-interacting protein isoform 2 [Homo sapiens]	34.34	2	11	30	0.559	14;16	14.1	96.58	530	60.1	9.13																			
19923653		High	qPcPSESDIITEEDk	Unambiguous	2	1	N-Term(iTRAQ4plex); C3(Methylthio); K15(iTRAQ4plex)	HCD	1.0000	0.0000	1	1	10079	Unique	Used	0.554	2.752E3	1.844E3	84	5.16	0.00	0	13	83	3	675.65344	2024.94577	3.07	46.06	4540	4540	MS2	22/56	Glioma_Nuclear_prep_Gradeall_repeat_4plex.raw
19923653		High	qPcPSESDIITEEDk	Unambiguous	2	1	N-Term(iTRAQ4plex); C3(Methylthio); K15(iTRAQ4plex)	HCD	1.0000	0.0000	1	1	10067	Unique	Used	0.642	6.944E3	5.390E3	73	5.42	0.00	0	11	300	2	1012.97614	2024.94499	2.69	45.89	4526	4526	MS2	21/28	Glioma_Nuclear_prep_Gradeall_repeat_4plex.raw
19923653	High		qPcPSESDIITEEDk	Unambiguous	2	1	N-Term(iTRAQ4plex); C3(Methylthio); K15(iTRAQ4plex)	HCD	1.0000	0.0000	1	1	9522	Unique	Used	0.529	3.341E5	2.157E5	41	5.71	0.00	0	3	8	2	1012.95758	2024.90788	-15.64	37.41	4111	4111	MS2	21/28	Glioma_Nuclear_prep_Gradeall_4plex.raw
19923653	High		qPcPSESDIITEEDk	Unambiguous	2	1	N-Term(iTRAQ4plex); C3(Methylthio); K15(iTRAQ4plex)	HCD	1.0000	0.0000	1	1	9531	Unique	Used	0.585	1.424E4	1.016E4	45	3.73	0.00	0	50	18	3	675.64038	2024.90659	-16.28	37.46	4120	4120	MS2	18/56	Glioma_Nuclear_prep_Gradeall_4plex.raw
19923653		High	kGFNEGLWEIDNNPk	Unambiguous	2	1	N-Term(iTRAQ4plex); K1(iTRAQ4plex); K15(iTRAQ4plex)	HCD	1.0000	0.0000	1	1	10427	Unique	Used	0.701	1.020E3	8.648E2	182	6.24	0.00	1	41	300	3	731.72705	2193.16660	0.05	50.15	4910	4910	MS2	23/56	Glioma_Nuclear_prep_Gradeall_repeat_4plex.raw
19923653	High		kGFNEGLWEIDNNPk	Unambiguous	2	1	N-Term(iTRAQ4plex); K1(iTRAQ4plex); K15(iTRAQ4plex)	HCD	1.0000	0.0000	1	1	9600	Unique	Used	0.519	3.151E4	1.996E4	185	5.98	0.00	1	40	10	3	731.71692	2193.13620	-13.81	37.96	4195	4195	MS2	23/56	Glioma_Nuclear_prep_Gradeall_4plex.raw
19923653		High	vDEVPDGAVkPPTNk	Unambiguous	2	1	N-Term(iTRAQ4plex); K10(iTRAQ4plex); K15(iTRAQ4plex)	HCD	1.0000	0.0000	1	1	8485	Unique	Used	0.434	3.014E4	1.584E4	103	3.71	0.00	0	9	16	3	666.71460	1998.12925	3.01	28.47	2837	2837	MS2	22/56	Glioma_Nuclear_prep_Gradeall_repeat_4plex.raw
19923653		High	vDEVPDGAVkPPTNk	Unambiguous	2	1	N-Term(iTRAQ4plex); K10(iTRAQ4plex); K15(iTRAQ4plex)	HCD	1.0000	0.0000	1	1	8500	Unique	Used	0.543	1.295E3	8.507E2	127	3.58	0.00	0	51	132	4	500.28598	1998.12209	-0.58	28.62	2853	2853	MS2	17/84	Glioma_Nuclear_prep_Gradeall_repeat_4plex.raw
19923653		High	vDEVPDGAVkPPTNk	Unambiguous	2	1	N-Term(iTRAQ4plex); K10(iTRAQ4plex); K15(iTRAQ4plex)	HCD	1.0000	0.0000	1	1	8522	Unique	Used	0.722	9.947E2	8.688E2	85	2.98	0.00	0	0	300	2	999.56824	1998.12920	2.98	28.85	2876	2876	MS2	15/28	Glioma_Nuclear_prep_Gradeall_repeat_4plex.raw
19923653	High		vDEVPDGAVkPPTNk	Unambiguous	2	1	N-Term(iTRAQ4plex); K10(iTRAQ4plex); K15(iTRAQ4plex)	HCD	1.0000	0.0000	1	1	8215	Unique	Used	0.512	1.335E5	8.337E4	138	3.77	0.00	0	11	2	3	666.70203	1998.09153	-15.87	27.88	2686	2686	MS2	22/56	Glioma_Nuclear_prep_Gradeall_4plex.raw
19923653	High		vDEVPDGAVkPPTNk	Unambiguous	2	1	N-Term(iTRAQ4plex); K10(iTRAQ4plex); K15(iTRAQ4plex)	HCD	1.0000	0.0000	1	1	8171	Unique	Used	0.539	2.172E5	1.428E5	127	4.72	0.00	0	13	7	2	999.54926	1998.09123	-16.02	27.57	2638	2638	MS2	20/28	Glioma_Nuclear_prep_Gradeall_4plex.raw
19923653		High	qSNASSDVEVEEk	Unambiguous	2	1	N-Term(iTRAQ4plex); K13(iTRAQ4plex)	HCD	1.0000	0.0000	1	1	7421	Unique	Used	0.443	2.451E4	1.312E4	105	5.63	0.00	0	11	174	2	855.41418	1709.82109	-12.96	18.40	1712	1712	MS2	24/24	Glioma_Nuclear_prep_Gradeall_repeat_4plex.raw
19923653	High		qSNASSDVEVEEk	Unambiguous	2	1	N-Term(iTRAQ4plex); K13(iTRAQ4plex)	HCD	1.0000	0.0000	1	1	7450	Unique	Used	0.408	3.407E5	1.695E5	143	5.84	0.00	0	6	10	2	855.42322	1709.83916	-2.39	22.46	1857	1857	MS2	24/24	Glioma_Nuclear_prep_Gradeall_4plex.raw
19923653		High	gFNEGLWEIDNNPk	Unambiguous	2	1	N-Term(iTRAQ4plex); K14(iTRAQ4plex)	HCD	1.0000	0.0000	1	1	11247	Unique	Used	0.598	2.317E3	1.675E3	162	5.84	0.00	0	13	300	2	960.98914	1920.97099	0.80	60.40	5815	5815	MS2	24/26	Glioma_Nuclear_prep_Gradeall_repeat_4plex.raw
19923653	High		gFNEGLWEIDNNPk	Unambiguous	2	1	N-Term(iTRAQ4plex); K14(iTRAQ4plex)	HCD	1.0000	0.0000	1	1	10397	Unique	Used	0.580	6.251E4	4.425E4	130	6.17	0.00	0	0	33	2	960.97516	1920.94304	-13.76	43.82	5062	5062	MS2	24/26	Glioma_Nuclear_prep_Gradeall_4plex.raw
19923653	High		lPIFFFGTHETAFLGPk	Unambiguous	2	1	N-Term(iTRAQ4plex); K17(iTRAQ4plex)	HCD	1.0000	0.0000	1	1	12744	Unique	Used	0.545	1.987E3	1.322E3	109	5.89	0.00	0	45	300	3	737.41449	2210.22892	1.64	65.55	7646	7646	MS2	27/64	Glioma_Nuclear_prep_Gradeall_4plex.raw
19923653	High		lPIFFFGTHETAFLGPk	Unambiguous	2	1	N-Term(iTRAQ4plex); K17(iTRAQ4plex)	HCD	1.0000	0.0000	1	1	12794	Unique	Used	0.564	2.720E3	1.872E3	101	6.70	0.00	0	17	300	3	737.41638	2210.23459	4.21	66.28	7707	7707	MS2	27/64	Glioma_Nuclear_prep_Gradeall_4plex.raw
19923653		High	dFkPGDLIFAk	Unambiguous	2	1	N-Term(iTRAQ4plex); K3(iTRAQ4plex); K11(iTRAQ4plex)	HCD	1.0000	0.0000	1	1	11014	Unique	Used	0.537	5.977E3	3.885E3	143	3.67	0.00	0	58	300	2	841.99731	1682.98735	1.87	57.33	5547	5547	MS2	18/20	Glioma_Nuclear_prep_Gradeall_repeat_4plex.raw
19923653		High	dFkPGDLIFAk	Unambiguous	2	1	N-Term(iTRAQ4plex); K3(iTRAQ4plex); K11(iTRAQ4plex)	HCD	1.0000	0.0000	1	1	11098	Unique	Used	0.575	2.432E3	1.692E3	117	3.20	0.00	0	84	300	2	842.00507	1683.00286	11.08	58.42	5642	5642	MS2	17/20	Glioma_Nuclear_prep_Gradeall_repeat_4plex.raw
19923653		High	dFkPGDLIFAk	Unambiguous	2	1	N-Term(iTRAQ4plex); K3(iTRAQ4plex); K11(iTRAQ4plex)	HCD	1.0000	0.0000	1	1	11010	Unique	Used	0.652	3.784E3	2.984E3	120	4.27	0.00	0	27	266	3	561.66833	1682.99045	3.71	57.28	5543	5543	MS2	23/40	Glioma_Nuclear_prep_Gradeall_repeat_4plex.raw
19923653	High		dFkPGDLIFAk	Unambiguous	2	1	N-Term(iTRAQ4plex); K3(iTRAQ4plex); K11(iTRAQ4plex)	HCD	1.0000	0.0000	1	1	10051	Unique	Used	0.521	1.509E5	9.591E4	159	4.07	0.00	0	25	3	3	561.65826	1682.96024	-14.24	41.23	4685	4685	MS2	23/40	Glioma_Nuclear_prep_Gradeall_4plex.raw
19923653	High		dFkPGDLIFAk	Unambiguous	2	1	N-Term(iTRAQ4plex); K3(iTRAQ4plex); K11(iTRAQ4plex)	HCD	1.0000	0.0000	1	1	10055	Unique	Used	0.546	2.617E5	1.742E5	190	3.72	0.00	0	37	13	2	841.98444	1682.96160	-13.44	41.25	4689	4689	MS2	17/20	Glioma_Nuclear_prep_Gradeall_4plex.raw
19923653		High	aVDITTPk	Unambiguous	2	1	N-Term(iTRAQ4plex); K8(iTRAQ4plex)	HCD	1.0000	0.0000	1	1	8022	Unique	Used	0.726	5.094E4	4.471E4	268	3.27	0.00	0	38	120	2	566.84460	1132.68193	0.27	23.99	2349	2349	MS2	13/14	Glioma_Nuclear_prep_Gradeall_repeat_4plex.raw
19923653	High		aSNEDVTk	Unambiguous	2	1	N-Term(iTRAQ4plex); K8(iTRAQ4plex)	HCD	1.0000	0.0000	1	1	6991	Unique	Used	0.588	6.414E3	4.602E3	226	3.18	0.00	0	82	300	2	576.31494	1151.62261	6.89	18.42	1354	1354	MS2	14/14	Glioma_Nuclear_prep_Gradeall_4plex.raw
19923653		High	fSSQQAATk	Unambiguous	2	1	N-Term(iTRAQ4plex); K9(iTRAQ4plex)	HCD	1.0000	0.0000	1	1	7156	Unique	Used	0.499	2.347E5	1.416E5	166	3.89	0.00	0	8	20	2	628.34619	1255.68511	-2.70	16.21	1434	1434	MS2	16/16	Glioma_Nuclear_prep_Gradeall_repeat_4plex.raw
19923653		High	dIFPYSENk	Unambiguous	2	1	N-Term(iTRAQ4plex); K9(iTRAQ4plex)	HCD	1.0000	0.0000	1	1	9619	Unique	Used	0.564	2.990E4	2.040E4	197	2.90	0.00	0	17	138	2	700.86725	1400.72722	-2.00	40.62	4044	4044	MS2	14/16	Glioma_Nuclear_prep_Gradeall_repeat_4plex.raw
19923653		High	fSSQQAATk	Unambiguous	2	1	N-Term(iTRAQ4plex); K9(iTRAQ4plex)	HCD	1.0000	0.0000	1	1	7126	Unique	Used	0.642	4.529E4	3.517E4	201	3.83	0.00	0	64	214	2	628.33734	1255.66741	-16.80	15.91	1402	1402	MS2	16/16	Glioma_Nuclear_prep_Gradeall_repeat_4plex.raw
19923653		High	dIFPYSENk	Unambiguous	2	1	N-Term(iTRAQ4plex); K9(iTRAQ4plex)	HCD	1.0000	0.0000	1	1	9529	Unique	Used	0.648	3.274E4	2.566E4	164	3.13	0.00	0	37	138	2	700.86395	1400.72063	-6.71	39.60	3946	3946	MS2	13/16	Glioma_Nuclear_prep_Gradeall_repeat_4plex.raw
19923653	High		dIFPYSENk	Unambiguous	2	1	N-Term(iTRAQ4plex); K9(iTRAQ4plex)	HCD	1.0000	0.0000	1	1	9091	Unique	Used	0.458	1.061E6	5.933E5	127	3.13	0.00	0	3	2	2	700.85840	1400.70952	-14.64	34.23	3640	3640	MS2	14/16	Glioma_Nuclear_prep_Gradeall_4plex.raw
19923653	High		dIFPYSENk	Unambiguous	2	1	N-Term(iTRAQ4plex); K9(iTRAQ4plex)	HCD	1.0000	0.0000	1	1	9008	Unique	Used	0.638	1.893E5	1.473E5	133	3.32	0.00	0	52	18	2	700.85547	1400.70366	-18.82	33.57	3547	3547	MS2	13/16	Glioma_Nuclear_prep_Gradeall_4plex.raw
21536286	NP_001814.2	CKB	1152	creatine kinase B-type [Homo sapiens]	9.71	1	3	4	1.610	2;2	19.6	9.63	381	42.6	5.59																			
21536286		High	vLTPELYAELR	Unambiguous	1	1	N-Term(iTRAQ4plex)	HCD	1.0000	0.0000	1	1	11665	Unique	Used	1.520	5.587E3	1.027E4	296	3.86	0.00	0	17	300	2	724.41827	1447.82927	1.05	66.51	6329	6329	MS2	16/20	Glioma_Nuclear_prep_Gradeall_repeat_4plex.raw
21536286		High	dLFDPIIEDR	Unambiguous	1	1	N-Term(iTRAQ4plex)	HCD	1.0000	0.0000	1	1	11553	Unique	Used	1.346	2.631E3	4.285E3	231	3.45	0.00	0	48	300	2	688.86298	1376.71868	0.58	64.87	6191	6191	MS2	17/18	Glioma_Nuclear_prep_Gradeall_repeat_4plex.raw
21536286	High		lAVEALSSLDGDLAGR	Unambiguous	1	1	N-Term(iTRAQ4plex)	HCD	1.0000	0.0000	1	1	12047	Unique	Used	2.110	2.841E4	7.313E4	272	5.59	0.00	0	29	85	2	865.97430	1730.94133	0.43	57.46	6864	6864	MS2	23/30	Glioma_Nuclear_prep_Gradeall_4plex.raw
21536286	High		lAVEALSSLDGDLAGR	Unambiguous	1	1	N-Term(iTRAQ4plex)	HCD	1.0000	0.0000	1	1	12038	Unique	Used	1.699	8.479E2	1.758E3	249	4.04	0.00	0	26	77	3	577.65063	1730.93735	-1.87	57.36	6854	6854	MS2	23/60	Glioma_Nuclear_prep_Gradeall_4plex.raw
22208967	NP_665906.1	HMGA1	3159	high mobility group protein HMG-I/HMG-Y isoform a [Homo sapiens]	30.84	2	3	13	2.201	6;7	22.8	35.48	107	11.7	10.32																			
22208967		High	kQPPVSPGTALVGSQk	Unambiguous	1	1	N-Term(iTRAQ4plex); K1(iTRAQ4plex); K16(iTRAQ4plex)	HCD	1.0000	0.0000	1	1	8383	Unique	Used	1.956	1.970E3	4.663E3	65	8.56	0.00	1	36	212	3	676.07263	2026.20334	0.59	27.49	2729	2729	MS2	28/60	Glioma_Nuclear_prep_Gradeall_repeat_4plex.raw
22208967		High	kQPPVSPGTALVGSQk	Unambiguous	1	1	N-Term(iTRAQ4plex); K1(iTRAQ4plex); K16(iTRAQ4plex)	HCD	1.0000	0.0000	1	1	8421	Unique	Used	3.101	6.722E3	2.522E4	44	4.90	0.00	1	4	161	2	1013.60370	2026.20012	-1.00	27.84	2769	2769	MS2	21/30	Glioma_Nuclear_prep_Gradeall_repeat_4plex.raw
22208967		High	kQPPVSPGTALVGSQk	Unambiguous	1	1	N-Term(iTRAQ4plex); K1(iTRAQ4plex); K16(iTRAQ4plex)	HCD	1.0000	0.0000	1	1	8484	Unique	Used	1.849	1.078E4	2.410E4	59	7.43	0.00	1	15	15	3	676.07324	2026.20517	1.49	28.46	2836	2836	MS2	30/60	Glioma_Nuclear_prep_Gradeall_repeat_4plex.raw
22208967		High	kQPPVSPGTALVGSQk	Unambiguous	1	1	N-Term(iTRAQ4plex); K1(iTRAQ4plex); K16(iTRAQ4plex)	HCD	1.0000	0.0000	1	1	8402	Unique	Used	1.486	1.153E3	2.073E3	93	5.20	0.00	1	45	91	4	507.30341	2026.19179	-5.11	27.67	2749	2749	MS2	24/90	Glioma_Nuclear_prep_Gradeall_repeat_4plex.raw
22208967	High		kQPPVSPGTALVGSQk	Unambiguous	1	1	N-Term(iTRAQ4plex); K1(iTRAQ4plex); K16(iTRAQ4plex)	HCD	1.0000	0.0000	1	1	8097	Unique	Used	3.122	1.156E5	4.403E5	60	4.90	0.00	1	0	7	2	1013.58667	2026.16606	-17.81	27.05	2558	2558	MS2	22/30	Glioma_Nuclear_prep_Gradeall_4plex.raw
22208967	High		kQPPVSPGTALVGSQk	Unambiguous	1	1	N-Term(iTRAQ4plex); K1(iTRAQ4plex); K16(iTRAQ4plex)	HCD	1.0000	0.0000	1	1	8141	Unique	Used	2.216	3.142E4	8.495E4	136	4.70	0.00	1	9	1	4	507.29742	2026.16787	-16.92	27.36	2606	2606	MS2	29/90	Glioma_Nuclear_prep_Gradeall_4plex.raw
22208967	High		kQPPVSPGTALVGSQk	Unambiguous	1	1	N-Term(iTRAQ4plex); K1(iTRAQ4plex); K16(iTRAQ4plex)	HCD	1.0000	0.0000	1	1	8178	Unique	Used	1.849	4.143E4	9.345E4	79	6.01	0.00	1	39	2	3	676.06030	2026.16635	-17.67	27.62	2646	2646	MS2	29/60	Glioma_Nuclear_prep_Gradeall_4plex.raw
22208967	High		kQPPVSPGTALVGSQk	Unambiguous	1	1	N-Term(iTRAQ4plex); K1(iTRAQ4plex); K16(iTRAQ4plex)	HCD	1.0000	0.0000	1	1	8077	Unique	Used	1.806	2.554E4	5.628E4	112	7.09	0.00	1	17	6	3	676.06024	2026.16617	-17.76	26.90	2536	2536	MS2	28/60	Glioma_Nuclear_prep_Gradeall_4plex.raw
22208967		High	sSQPLASk	Unambiguous	2	1	N-Term(iTRAQ4plex); K8(iTRAQ4plex)	HCD	1.0000	0.0000	1	1	6876	Unique	Used	2.285	9.295E4	2.569E5	346	3.06	0.00	0	14	21	2	553.32385	1105.64043	-4.65	13.67	1137	1137	MS2	14/14	Glioma_Nuclear_prep_Gradeall_repeat_4plex.raw
22208967	High		sSQPLASk	Unambiguous	2	1	N-Term(iTRAQ4plex); K8(iTRAQ4plex)	HCD	1.0000	0.0000	1	1	7162	Unique	Used	2.201	6.541E4	1.756E5	306	2.99	0.00	0	16	29	2	553.31842	1105.62956	-14.47	20.26	1542	1542	MS2	14/14	Glioma_Nuclear_prep_Gradeall_4plex.raw
22208967		High	ePSEVPTPk	Unambiguous	2	1	N-Term(iTRAQ4plex); K9(iTRAQ4plex)	HCD	1.0000	0.0000	1	1	7790	Unique	Used	2.938	1.089E5	3.871E5	246	3.08	0.00	0	5	10	2	636.35913	1271.71099	1.89	21.78	2105	2105	MS2	15/16	Glioma_Nuclear_prep_Gradeall_repeat_4plex.raw
22208967		High	ePSEVPTPk	Unambiguous	2	1	N-Term(iTRAQ4plex); K9(iTRAQ4plex)	HCD	1.0000	0.0000	1	1	7681	Unique	Used	2.138	9.845E3	2.546E4	284	3.25	0.00	0	41	254	2	636.35181	1271.69634	-9.63	20.74	1990	1990	MS2	13/16	Glioma_Nuclear_prep_Gradeall_repeat_4plex.raw
22208967	High		ePSEVPTPk	Unambiguous	2	1	N-Term(iTRAQ4plex); K9(iTRAQ4plex)	HCD	1.0000	0.0000	1	1	7615	Unique	Used	2.419	1.571E5	4.635E5	270	2.92	0.00	0	20	7	2	636.35638	1271.70549	-2.43	23.64	2036	2036	MS2	13/16	Glioma_Nuclear_prep_Gradeall_4plex.raw
25121993	NP_733839.1	MSI2	124540	RNA-binding protein Musashi homolog 2 isoform b [Homo sapiens]	5.98	2	1	2	2.525	1;1	0.9	5.23	251	28.4	8.50																			
25121993		High	gFGFVTFADPASVDk	Unambiguous	2	1	N-Term(iTRAQ4plex); K15(iTRAQ4plex)	HCD	1.0000	0.0000	1	1	12060	Unique	Used	2.541	1.024E3	3.147E3	185	4.82	0.00	0	13	300	2	923.48749	1845.96770	2.76	73.14	6868	6868	MS2	25/28	Glioma_Nuclear_prep_Gradeall_repeat_4plex.raw

25121993	High		gFGFVTFADPASVDk	Unambiguous	2	1	N-Term(iTRAQ4plex); K15(iTRAQ4plex)	HCD	1.0000	0.0000	1	1	11354	Unique	Used	2.508	4.599E4	1.407E5	187	5.23	0.00	0	31	36	2	923.48523	1845.96318	0.32	51.36	6108	6108	MS2	21/28	Glioma_Nuclear_prep_Gradeall_4plex.raw
25952118	NP_741960.1	CAMK2A	815	calcium/calmodulin-dependent protein kinase type II subunit alpha isoform 2 [Homo sapiens]	2.51	2	1	2	0.357	1;1	3.6	3.85	478	54.1	7.08																			
25952118		High	iTQYLDAGGIPR	Unambiguous	2	1	N-Term(iTRAQ4plex)	HCD	1.0000	0.0000	1	1	11026	Unique	Used	0.348	9.949E3	4.190E3	321	3.85	0.00	0	42	300	2	724.40668	1447.80608	2.41	57.49	5561	5561	MS2	16/22	Glioma_Nuclear_prep_Gradeall_repeat_4plex.raw
25952118	High		iTQYLDAGGIPR	Unambiguous	2	1	N-Term(iTRAQ4plex)	HCD	1.0000	0.0000	1	1	10237	Unique	Used	0.367	1.314E5	5.875E4	304	3.66	0.00	0	41	23	2	724.39508	1447.78288	-13.61	42.62	4888	4888	MS2	18/22	Glioma_Nuclear_prep_Gradeall_4plex.raw
27436946	NP_733821.1	LMNA	4000	lamin isoform A [Homo sapiens]	46.23	4	1	2	0.436	1;1	8.4	183.70	664	74.1	7.02																			
27436946		High	sVGGSGGGSFGDNLVTR	Unambiguous	3	1	N-Term(iTRAQ4plex)	HCD	1.0000	0.0000	1	1	9267	Unique	Used	0.462	8.668E3	4.844E3	120	5.49	0.00	0	37	300	2	855.93384	1710.86040	4.43	36.71	3665	3665	MS2	23/32	Glioma_Nuclear_prep_Gradeall_repeat_4plex.raw
27436946	High		sVGGSGGGSFGDNLVTR	Unambiguous	3	1	N-Term(iTRAQ4plex)	HCD	1.0000	0.0000	1	1	8860	Unique	Used	0.410	1.370E5	6.863E4	84	4.73	0.00	0	24	15	2	855.91742	1710.82756	-14.77	32.52	3387	3387	MS2	26/32	Glioma_Nuclear_prep_Gradeall_4plex.raw
29788768	NP_821080.1	TUBB2B	347733	tubulin beta-2B chain [Homo sapiens]	32.13	3	2	5	0.537	3;2	37.0	101.57	445	49.9	4.89																			
29788768	High		yLTVAAIFR	Unambiguous	2	1	N-Term(iTRAQ4plex)	HCD	1.0000	0.0000	1	1	11606	Unique	Used	0.537	4.564E4	2.991E4	188	3.46	0.00	0	43	96	2	599.36139	1197.71550	3.55	53.46	6383	6383	MS2	15/16	Glioma_Nuclear_prep_Gradeall_4plex.raw
29788768		High	aLTVPELTQQMFDSk	Unambiguous	2	1	N-Term(iTRAQ4plex); K15(iTRAQ4plex)	HCD	1.0000	0.0000	1	1	12311	Unique	Used	0.214	2.049E2	5.302E1	207	4.29	0.00	0	32	300	3	666.02863	1996.07132	2.46	79.31	7337	7337	MS2	20/56	Glioma_Nuclear_prep_Gradeall_repeat_4plex.raw
29788768	High		aLTVPELTQQMFDSk	Unambiguous	2	1	N-Term(iTRAQ4plex); K15(iTRAQ4plex)	HCD	1.0000	0.0000	1	1	11827	Unique	Used	0.478	3.841E4	2.242E4	184	5.16	0.00	0	20	95	2	998.53510	1996.06291	-1.75	55.43	6623	6623	MS2	18/28	Glioma_Nuclear_prep_Gradeall_4plex.raw
29788768	High		aLTVPELTQQMFDSk	Unambiguous	2	1	N-Term(iTRAQ4plex); K15(iTRAQ4plex)	HCD	1.0000	0.0000	1	1	11855	Unique	Used	0.686	1.323E4	1.108E4	175	6.06	0.00	0	13	15	3	666.02582	1996.06290	-1.76	55.69	6654	6654	MS2	24/56	Glioma_Nuclear_prep_Gradeall_4plex.raw
29788768		High	aLTVPELTQQmFDSk	Unambiguous	2	1	N-Term(iTRAQ4plex); M11(Oxidation); K15(iTRAQ4plex)	HCD	1.0000	0.0000	1	1	11090	Unique	Used	0.675	7.791E2	6.358E2	200	4.98	0.00	0	48	300	3	671.36023	2012.06614	2.39	58.31	5633	5633	MS2	20/56	Glioma_Nuclear_prep_Gradeall_repeat_4plex.raw
31742498	NP_056189.1	SUN2	25777	SUN domain-containing protein 2 isoform b [Homo sapiens]	14.37	2	4	7	1.523	4;3	25.1	33.52	717	80.3	6.73																			
31742498		High	iGLADYALESGGASVISTR	Unambiguous	2	1	N-Term(iTRAQ4plex)	HCD	1.0000	0.0000	1	1	11907	Unique	Used	2.602	1.524E2	4.800E2	157	4.98	0.00	0	23	300	3	675.36572	2024.08261	2.21	70.31	6644	6644	MS2	24/72	Glioma_Nuclear_prep_Gradeall_repeat_4plex.raw
31742498		High	iGLADYALESGGASVISTR	Unambiguous	2	1	N-Term(iTRAQ4plex)	HCD	1.0000	0.0000	1	1	11915	Unique	Used	1.481	5.796E2	1.038E3	162	5.24	0.00	0	13	300	2	1012.54291	2024.07854	0.20	70.45	6655	6655	MS2	24/36	Glioma_Nuclear_prep_Gradeall_repeat_4plex.raw
31742498	High		iGLADYALESGGASVISTR	Unambiguous	2	1	N-Term(iTRAQ4plex)	HCD	1.0000	0.0000	1	1	11176	Unique	Used	1.523	3.132E4	5.819E4	160	6.48	0.00	0	9	41	2	1012.54407	2024.08086	1.35	49.84	5913	5913	MS2	26/36	Glioma_Nuclear_prep_Gradeall_4plex.raw
31742498	High		iGLADYALESGGASVISTR	Unambiguous	2	1	N-Term(iTRAQ4plex)	HCD	1.0000	0.0000	1	1	11194	Unique	Used	1.516	3.837E4	7.096E4	164	6.84	0.00	0	0	24	2	1012.53455	2024.06182	-8.06	49.98	5933	5933	MS2	26/36	Glioma_Nuclear_prep_Gradeall_4plex.raw
31742498	High		iLTHVAEMQGk	Unambiguous	2	1	N-Term(iTRAQ4plex); K11(iTRAQ4plex)	HCD	1.0000	0.0000	1	1	8547	Unique	Used	1.301	5.319E4	8.446E4	229	4.56	0.00	0	36	38	2	757.92279	1514.83830	-14.56	30.22	3045	3045	MS2	19/20	Glioma_Nuclear_prep_Gradeall_4plex.raw
31742498		High	aEHQQDSEDLFk	Unambiguous	2	1	N-Term(iTRAQ4plex); K12(iTRAQ4plex)	HCD	1.0000	0.0000	1	1	8080	Unique	Used	1.710	3.730E3	7.718E3	112	6.01	0.00	0	31	84	3	578.95532	1734.85141	-1.35	24.55	2410	2410	MS2	22/44	Glioma_Nuclear_prep_Gradeall_repeat_4plex.raw
31742498	High		lEDQLAGLQQELAALk	Unambiguous	2	1	N-Term(iTRAQ4plex); K18(iTRAQ4plex)	HCD	1.0000	0.0000	1	1	13114	Unique	Used	1.874	8.454E1	1.933E2	82	5.21	0.00	0	9	300	3	738.09894	2212.28226	1.38	74.05	8282	8282	MS2	28/68	Glioma_Nuclear_prep_Gradeall_4plex.raw
32189394	NP_001677.2	ATP5B	506	ATP synthase subunit beta, mitochondrial precursor [Homo sapiens]	16.07	1	5	7	0.607	4;3	14.7	27.75	529	56.5	5.40																			
32189394		High	aIAELGIYPAVDPLDSTSR	Unambiguous	1	1	N-Term(iTRAQ4plex)	HCD	1.0000	0.0000	1	1	12223	Unique	Used	0.607	4.447E2	3.267E2	113	4.86	0.00	0	0	300	2	1066.57019	2132.13310	-1.20	76.67	7142	7142	MS2	20/36	Glioma_Nuclear_prep_Gradeall_repeat_4plex.raw
32189394		High	iMNVIGEPIDER	Unambiguous	1	1	N-Term(iTRAQ4plex)	HCD	1.0000	0.0000	1	1	10974	Unique	Used	0.690	4.377E3	3.654E3	296	3.94	0.00	0	54	300	2	765.41028	1529.81328	1.18	56.83	5503	5503	MS2	16/22	Glioma_Nuclear_prep_Gradeall_repeat_4plex.raw
32189394	High		aIAELGIYPAVDPLDSTSR	Unambiguous	1	1	N-Term(iTRAQ4plex)	HCD	1.0000	0.0000	1	1	11664	Unique	Used	0.503	1.280E4	7.855E3	134	5.65	0.00	0	7	101	2	1066.56995	2132.13262	-1.43	53.99	6446	6446	MS2	21/36	Glioma_Nuclear_prep_Gradeall_4plex.raw
32189394	High		lVLEVAQHLGESTVR	Unambiguous	1	1	N-Term(iTRAQ4plex)	HCD	1.0000	0.0000	1	1	10956	Unique	Used	0.550	1.562E4	1.049E4	162	4.39	0.00	0	34	24	3	599.00189	1794.99112	-15.80	48.11	5673	5673	MS2	27/56	Glioma_Nuclear_prep_Gradeall_4plex.raw
32189394	High		iMNVIGEPIDER	Unambiguous	1	1	N-Term(iTRAQ4plex)	HCD	1.0000	0.0000	1	1	10213	Unique	Used	0.560	1.045E5	7.133E4	237	4.01	0.00	0	36	22	2	765.39807	1529.78887	-14.78	42.45	4862	4862	MS2	17/22	Glioma_Nuclear_prep_Gradeall_4plex.raw
32189394	High		fTQAGSEVSALLGR	Unambiguous	1	1	N-Term(iTRAQ4plex)	HCD	1.0000	0.0000	1	1	11077	Unique	Used	0.768	3.603E4	3.377E4	282	4.81	0.00	0	37	52	2	790.42651	1579.84575	-6.55	49.06	5805	5805	MS2	23/26	Glioma_Nuclear_prep_Gradeall_4plex.raw
32189394		High	vLDSGAPIk	Unambiguous	1	1	N-Term(iTRAQ4plex); K9(iTRAQ4plex)	HCD	1.0000	0.0000	1	1	8575	Unique	Used	0.612	2.093E4	1.549E4	183	3.35	0.00	0	53	216	2	594.36456	1187.72185	-1.67	29.37	2932	2932	MS2	16/16	Glioma_Nuclear_prep_Gradeall_repeat_4plex.raw
33946291	NP_079106.3	LPCAT1	79888	lysophosphatidylcholine acyltransferase 1 [Homo sapiens]	3.75	1	2	3	2.322	2;1	8.5	7.58	534	59.1	6.02																			
33946291		High	iTFADFHR	Unambiguous	1	1	N-Term(iTRAQ4plex)	HCD	1.0000	0.0000	1	1	9962	Unique	Used	2.397	8.278E2	2.401E3	280	3.40	0.00	0	49	300	3	384.20914	1150.61286	0.23	44.58	4410	4410	MS2	16/28	Glioma_Nuclear_prep_Gradeall_repeat_4plex.raw
33946291	High		lPADTcLLEFAR	Unambiguous	1	1	N-Term(iTRAQ4plex); C6(Methylthio)	HCD	1.0000	0.0000	1	1	12474	Unique	Used	2.322	6.537E3	1.852E4	253	4.07	0.00	0	37	300	2	769.89532	1538.78337	0.37	61.99	7334	7334	MS2	15/22	Glioma_Nuclear_prep_Gradeall_4plex.raw
33946291	High		lPADTcLLEFAR	Unambiguous	1	1	N-Term(iTRAQ4plex); C6(Methylthio)	HCD	1.0000	0.0000	1	1	12526	Unique	Used	2.035	3.616E3	8.978E3	255	3.51	0.00	0	38	300	2	769.89539	1538.78349	0.45	62.69	7395	7395	MS2	16/22	Glioma_Nuclear_prep_Gradeall_4plex.raw
34101286	NP_057191.2	ZFR	51663	zinc finger RNA-binding protein [Homo sapiens]	6.24	1	4	4	0.671	3;1	15.3	11.14	1074	116.9	9.04																			
34101286	High		cLDALAALR	Unambiguous	1	1	N-Term(iTRAQ4plex); C1(Methylthio)	HCD	1.0000	0.0000	1	1	11522	Unique	Used	0.747	1.329E5	1.210E5	279	3.46	0.00	0	21	46	2	568.30682	1135.60637	-1.84	52.78	6292	6292	MS2	15/16	Glioma_Nuclear_prep_Gradeall_4plex.raw
34101286	High		cAVSEAAIILNScVEPk	Unambiguous	1	1	N-Term(iTRAQ4plex); C1(Methylthio); C13(Methylthio); K17(iTRAQ4plex)	HCD	1.0000	0.0000	1	1	12974	Unique	Used	0.773	3.486E2	3.290E2	115	4.20	0.00	0	30	300	3	709.69281	2127.06388	3.64	69.42	7956	7956	MS2	21/64	Glioma_Nuclear_prep_Gradeall_4plex.raw
34101286	High		vSAVPTNMAAk	Unambiguous	1	1	N-Term(iTRAQ4plex); K11(iTRAQ4plex)	HCD	1.0000	0.0000	1	1	8206	Unique	Used	0.571	1.760E5	1.227E5	325	3.48	0.00	0	29	13	2	688.88409	1376.76091	-14.63	27.82	2676	2676	MS2	15/20	Glioma_Nuclear_prep_Gradeall_4plex.raw
34101286		High	sTPVTSAVQIPEVk	Unambiguous	1	1	N-Term(iTRAQ4plex); K14(iTRAQ4plex)	HCD	1.0000	0.0000	1	1	10225	Unique	Used	0.596	4.569E3	3.294E3	173	4.77	0.00	0	43	300	2	872.51343	1744.01958	5.76	47.75	4694	4694	MS2	20/26	Glioma_Nuclear_prep_Gradeall_4plex.raw
34419635	NP_002146.2	HSPA6	3310	heat shock 70 kDa protein 6 [Homo sapiens]	10.26	1	1	2	0.553	1;1	1.8	15.15	643	71.0	6.14																			
34419635		High	vEILANDQGNR	Unambiguous	1	1	N-Term(iTRAQ4plex)	HCD	1.0000	0.0000	1	1	8255	Unique	Used	0.546	2.148E4	1.418E4	223	3.40	0.00	0	7	130	2	686.87018	1372.73308	2.12	26.25	2595	2595	MS2	16/20	Glioma_Nuclear_prep_Gradeall_repeat_4plex.raw
34419635	High		vEILANDQGNR	Unambiguous	1	1	N-Term(iTRAQ4plex)	HCD	1.0000	0.0000	1	1	8048	Unique	Used	0.560	1.865E5	1.273E5	224	3.15	0.00	0	14	11	2	686.85797	1372.70867	-15.66	26.70	2505	2505	MS2	16/20	Glioma_Nuclear_prep_Gradeall_4plex.raw
34740329	NP_919223.1	HNRNPA3	220988	heterogeneous nuclear ribonucleoprotein A3 [Homo sapiens]	31.22	1	10	31	0.613	17;14	13.5	97.89	378	39.6	9.01																			
34740329		High	sSGSPYGGGYGSGGGSGGYGSR	Unambiguous	1	1	N-Term(iTRAQ4plex)	HCD	1.0000	0.0000	1	1	7948	Unique	Used	0.521	1.593E4	1.005E4	31	7.21	0.00	0	0	300	2	1027.94800	2054.88872	-1.65	23.27	2271	2271	MS2	31/42	Glioma_Nuclear_prep_Gradeall_repeat_4plex.raw
34740329		High	eDTEEYNLR	Unambiguous	1	1	N-Term(iTRAQ4plex)	HCD	1.0000	0.0000	1	1	7928	Unique	Used	0.592	7.413E4	5.309E4	107	3.74	0.00	0	10	128	2	656.81165	1312.61601	1.71	23.07	2250	2250	MS2	16/16	Glioma_Nuclear_prep_Gradeall_repeat_4plex.raw
34740329		High	lFIGGLSFETTDDSLR	Unambiguous	1	1	N-Term(iTRAQ4plex)	HCD	1.0000	0.0000	1	1	12394	Unique	Used	0.631	2.010E2	1.535E2	184	4.25	0.00	0	16	300	3	639.00293	1914.99424	0.64	83.05	7599	7599	MS2	23/60	Glioma_Nuclear_prep_Gradeall_repeat_4plex.raw
34740329		High	iETIEVMEDR	Unambiguous	1	1	N-Term(iTRAQ4plex)	HCD	1.0000	0.0000	1	1	10250	Unique	Used	0.688	4.830E4	4.023E4	231	4.21	0.00	0	38	193	2	689.85565	1378.70403	2.55	48.04	4721	4721	MS2	14/18	Glioma_Nuclear_prep_Gradeall_repeat_4plex.raw
34740329		High	sSGSPYGGGYGSGGGSGGYGSR	Unambiguous	1	1	N-Term(iTRAQ4plex)	HCD	1.0000	0.0000	1	1	7988	Unique	Used	0.693	1.556E3	1.305E3	27	5.75	0.00	0	19	106	3	685.63824	2054.90018	3.93	23.68	2313	2313	MS2	25/84	Glioma_Nuclear_prep_Gradeall_repeat_4plex.raw
34740329		High	sSGSPYGGGYGSGGGSGGYGSR	Unambiguous	1	1	N-Term(iTRAQ4plex)	HCD	1.0000	0.0000	1	1	7479	Unique	Used	0.770	1.283E3	1.194E3	29	7.13	0.00	0	26	212	3	685.63562	2054.89231	0.10	18.91	1773	1773	MS2	27/84	Glioma_Nuclear_prep_Gradeall_repeat_4plex.raw
34740329	High		sSGSPYGGGYGSGGGSGGYGSR	Unambiguous	1	1	N-Term(iTRAQ4plex)	HCD	1.0000	0.0000	1	1	7861	Unique	Used	0.523	1.219E5	7.776E4	33	6.75	0.00	0	0	14	2	1027.95142	2054.89556	1.68	25.37	2302	2302	MS2	33/42	Glioma_Nuclear_prep_Gradeall_4plex.raw
34740329	High		iETIEVMEDR	Unambiguous	1	1	N-Term(iTRAQ4plex)	HCD	1.0000	0.0000	1	1	9697	Unique	Used	0.535	1.246E6	8.139E5	242	3.85	0.00	0	54	5	2	689.86011	1378.71294	9.01	38.67	4301	4301	MS2	14/18	Glioma_Nuclear_prep_Gradeall_4plex.raw
34740329	High		lFIGGLSFETTDDSLR	Unambiguous	1	1	N-Term(iTRAQ4plex)	HCD	1.0000	0.0000	1	1	12115	Unique	Used	0.605	1.394E5	1.030E5	235	5.40	0.00	0	6	35	2	958.00354	1914.99980	3.55	58.18	6939	6939	MS2	23/30	Glioma_Nuclear_prep_Gradeall_4plex.raw
34740329	High		eDTEEYNLR	Unambiguous	1	1	N-Term(iTRAQ4plex)	HCD	1.0000	0.0000	1	1	7783	Unique	Used	0.606	8.174E5	6.040E5	109	3.55	0.00	0	7	5	2	656.81104	1312.61479	0.78	24.83	2218	2218	MS2	16/16	Glioma_Nuclear_prep_Gradeall_4plex.raw
34740329	High		lFIGGLSFETTDDSLR	Unambiguous	1	1	N-Term(iTRAQ4plex)	HCD	1.0000	0.0000	1	1	12193	Unique	Used	0.613	2.424E5	1.811E5	239	5.25	0.00	0	13	31	2	958.00537	1915.00347	5.46	58.91	7024	7024	MS2	23/30	Glioma_Nuclear_prep_Gradeall_4plex.raw
34740329	High		iETIEVMEDR	Unambiguous	1	1	N-Term(iTRAQ4plex)	HCD	1.0000	0.0000	1	1	9630	Unique	Used	0.649	4.496E5	3.562E5	173	3.76	0.00	0	34	10	2	689.84448	1378.68169	-13.66	38.18	4228	4228	MS2	18/18	Glioma_Nuclear_prep_Gradeall_4plex.raw
34740329	High		lFIGGLSFETTDDSLR	Unambiguous	1	1	N-Term(iTRAQ4plex)	HCD	1.0000	0.0000	1	1	12309	Unique	Used	0.675	1.187E3	9.781E2	223	4.30	0.00	0	29	300	3	639.00098	1914.98838	-2.42	60.13	7152	7152	MS2	17/60	Glioma_Nuclear_prep_Gradeall_4plex.raw
34740329	High		wGTLTDcVVMR	Unambiguous	1	1	N-Term(iTRAQ4plex); C7(Methylthio)	HCD	1.0000	0.0000	1	1	11830	Unique	Used	0.490	3.722E5	2.227E5	89	4.21	0.00	0	10	23	2	735.85382	1470.70036	-1.41	55.46	6627	6627	MS2	19/20	Glioma_Nuclear_prep_Gradeall_4plex.raw
34740329	High		wGTLTDcVVMR	Unambiguous	1	1	N-Term(iTRAQ4plex); C7(Methylthio)	HCD	1.0000	0.0000	1	1	11758	Unique	Used	0.520	8.888E4	5.639E4	103	3.96	0.00	0	34	98	2	735.85516	1470.70305	0.41	54.85	6548	6548	MS2	19/20	Glioma_Nuclear_prep_Gradeall_4plex.raw
34740329	High		kLFIGGLSFETTDDSLR	Unambiguous	1	1	N-Term(iTRAQ4plex); K1(iTRAQ4plex)	HCD	1.0000	0.0000	1	1	11117	Unique	Used	0.493	6.051E4	3.642E4	154	5.46	0.00	1	5	47	2	1094.09705	2187.18682	-1.47	49.36	5848	5848	MS2	25/32	Glioma_Nuclear_prep_Gradeall_4plex.raw
34740329	High		kLFIGGLSFETTDDSLR	Unambiguous	1	1	N-Term(iTRAQ4plex); K1(iTRAQ4plex)	HCD	1.0000	0.0000	1	1	11062	Unique	Used	0.643	1.834E4	1.438E4	136	6.96	0.00	1	28	18	3	729.73425	2187.18821	-0.84	48.94	5789	5789	MS2	27/64	Glioma_Nuclear_prep_Gradeall_4plex.raw
34740329		High	gFAFVTFDDHDTVDk	Unambiguous	1	1	N-Term(iTRAQ4plex); K15(iTRAQ4plex)	HCD	1.0000	0.0000	1	1	11232	Unique	Used	0.597	6.908E3	4.987E3	108	4.99	0.00	0	11	116	3	667.99921	2001.98307	1.68	60.21	5798	5798	MS2	30/56	Glioma_Nuclear_prep_Gradeall_repeat_4plex.raw
34740329		High	gFAFVTFDDHDTVDk	Unambiguous	1	1	N-Term(iTRAQ4plex); K15(iTRAQ4plex)	HCD	1.0000	0.0000	1	1	11299	Unique	Used	0.613	4.554E3	3.377E3	117	4.90	0.00	0	13	209	3	667.99994	2001.98526	2.78	61.24	5883	5883	MS2	29/56	Glioma_Nuclear_prep_Gradeall_repeat_4plex.raw
34740329		High	gFAFVTFDDHDTVDk	Unambiguous	1	1	N-Term(iTRAQ4plex); K15(iTRAQ4plex)	HCD	1.0000	0.0000	1	1	11239	Unique	Used	0.656	5.979E3	4.748E3	121	6.09	0.00	0	10	300	2	1001.49506	2001.98284	1.57	60.30	5806	5806	MS2	24/28	Glioma_Nuclear_prep_Gradeall_repeat_4plex.raw
34740329		High	gFAFVTFDDHDTVDk	Unambiguous	1	1	N-Term(iTRAQ4plex); K15(iTRAQ4plex)	HCD	1.0000	0.0000	1	1	11364	Unique	Used	0.703	1.279E3	1.087E3	106	4.97	0.00	0	30	300	3	668.00067	2001.98746	3.88	62.31	5970	5970	MS2	27/56	Glioma_Nuclear_prep_Gradeall_repeat_4plex.raw
34740329		High	gFAFVTFDDHDTVDk	Unambiguous	1	1	N-Term(iTRAQ4plex); K15(iTRAQ4plex)	HCD	1.0000	0.0000	1	1	11448	Unique	Used	0.736	8.937E2	7.962E2	105	3.81	0.00	0	54	300	3	667.99841	2001.98069	0.49	63.47	6070	6070	MS2	25/56	Glioma_Nuclear_prep_Gradeall_repeat_4plex.raw
34740329	High		gFAFVTFDDHDTVDk	Unambiguous	1	1	N-Term(iTRAQ4plex); K15(iTRAQ4plex)	HCD	1.0000	0.0000	1	1	10395	Unique	Used	0.599	9.822E4	7.180E4	85	5.74	0.00	0	21	16	2	1001.47864	2001.95000	-14.83	43.80	5060	5060	MS2	24/28	Glioma_Nuclear_prep_Gradeall_4plex.raw
34740329	High		gFAFVTFDDHDTVDk	Unambiguous	1	1	N-Term(iTRAQ4plex); K15(iTRAQ4plex)	HCD	1.0000	0.0000	1	1	10283	Unique	Used	0.678	6.342E4	5.245E4	78	4.61	0.00	0	31	8	3	667.98853	2001.95102	-14.32	42.96	4938	4938	MS2	28/56	Glioma_Nuclear_prep_Gradeall_4plex.raw
34740329	High		gFAFVTFDDHDTVDk	Unambiguous	1	1	N-Term(iTRAQ4plex); K15(iTRAQ4plex)	HCD	1.0000	0.0000	1	1	10293	Unique	Used	0.719	2.238E5	1.963E5	85	5.64	0.00	0	4	9	2	1001.47919	2001.95110	-14.29	43.03	4949	4949	MS2	24/28	Glioma_Nuclear_prep_Gradeall_4plex.raw
34740329	High		gFAFVTFDDHDTVDk	Unambiguous	1	1	N-Term(iTRAQ4plex); K15(iTRAQ4plex)	HCD	1.0000	0.0000	1	1	10443	Unique	Used	0.731	6.117E4	5.455E4	87	4.94	0.00	0	29	7	3	667.98877	2001.95176	-13.96	44.19	5114	5114	MS2	31/56	Glioma_Nuclear_prep_Gradeall_4plex.raw
34740329		High	eDSVkPGAHLTVk	Unambiguous	1	1	N-Term(iTRAQ4plex); K5(iTRAQ4plex); K13(iTRAQ4plex)	HCD	1.0000	0.0000	1	1	7708	Unique	Used	0.625	1.616E4	1.222E4	126	4.91	0.00	0	19	300	2	907.02472	1813.04216	-6.76	21.01	2018	2018	MS2	17/24	Glioma_Nuclear_prep_Gradeall_repeat_4plex.raw
34740329		High	iFVGGIkEDTEEYNLR	Unambiguous	1	1	N-Term(iTRAQ4plex); K7(iTRAQ4plex)	HCD	1.0000	0.0000	1	1	10937	Unique	Used	0.452	4.754E3	2.601E3	134	3.50	0.00	1	14	263	3	724.39355	2171.16611	3.40	56.39	5464	5464	MS2	25/60	Glioma_Nuclear_prep_Gradeall_repeat_4plex.raw
34740329		High	iFVGGIkEDTEEYNLR	Unambiguous	1	1	N-Term(iTRAQ4plex); K7(iTRAQ4plex)	HCD	1.0000	0.0000	1	1	10950	Unique	Used	0.544	4.178E3	2.751E3	121	5.80	0.00	1	5	300	2	1086.08557	2171.16387	2.37	56.54	5477	5477	MS2	25/30	Glioma_Nuclear_prep_Gradeall_repeat_4plex.raw
34740329	High		iFVGGIkEDTEEYNLR	Unambiguous	1	1	N-Term(iTRAQ4plex); K7(iTRAQ4plex)	HCD	1.0000	0.0000	1	1	10057	Unique	Used	0.488	1.893E5	1.126E5	152	5.26	0.00	1	2	12	2	1086.06665	2171.12602	-15.06	41.26	4691	4691	MS2	24/30	Glioma_Nuclear_prep_Gradeall_4plex.raw
34740329	High		gEEGHDPkEPEQLR	Unambiguous	1	1	N-Term(iTRAQ4plex); K8(iTRAQ4plex)	HCD	1.0000	0.0000	1	1	7282	Unique	Used	0.640	2.799E4	2.187E4	98	3.88	0.00	1	4	28	2	954.98309	1908.95891	-3.42	21.22	1674	1674	MS2	18/26	Glioma_Nuclear_prep_Gradeall_4plex.raw
38201710	NP_006377.2	DDX17	10521	probable ATP-dependent RNA helicase DDX17 isoform 1 [Homo sapiens]	32.24	7	14	22	0.567	10;12	18.3	87.65	729	80.2	8.27																			
38201710		High	lMQLVDHR	Unambiguous	2	1	N-Term(iTRAQ4plex)	HCD	1.0000	0.0000	1	1	9388	Unique	Used	0.475	6.504E3	3.734E3	181	3.84	0.00	0	17	239	3	385.88513	1155.64084	-1.46	38.02	3792	3792	MS2	16/28	Glioma_Nuclear_prep_Gradeall_repeat_4plex.raw
38201710		High	lTPYEVDELR	Unambiguous	2	1	N-Term(iTRAQ4plex)	HCD	1.0000	0.0000	1	1	10338	Unique	Used	0.566	1.968E4	1.347E4	289	3.96	0.00	0	56	300	2	689.87238	1378.73747	2.88	49.11	4816	4816	MS2	15/18	Glioma_Nuclear_prep_Gradeall_repeat_4plex.raw
38201710		High	lTPYEVDELR	Unambiguous	2	1	N-Term(iTRAQ4plex)	HCD	1.0000	0.0000	1	1	10430	Unique	Used	0.604	6.631E3	4.845E3	298	3.23	0.00	0	49	300	2	689.87115	1378.73503	1.11	50.18	4913	4913	MS2	13/18	Glioma_Nuclear_prep_Gradeall_repeat_4plex.raw
38201710		High	lTPYEVDELRR	Unambiguous	2	1	N-Term(iTRAQ4plex)	HCD	1.0000	0.0000	1	1	9645	Unique	Used	0.640	3.352E3	2.596E3	237	3.51	0.00	1	31	300	3	512.28143	1534.82975	-3.18	40.91	4071	4071	MS2	15/40	Glioma_Nuclear_prep_Gradeall_repeat_4plex.raw
38201710		High	dWVLNEFR	Unambiguous	2	1	N-Term(iTRAQ4plex)	HCD	1.0000	0.0000	1	1	11588	Unique	Used	0.647	5.811E3	4.547E3	279	3.17	0.00	0	48	300	2	611.82257	1222.63786	3.38	65.42	6236	6236	MS2	13/14	Glioma_Nuclear_prep_Gradeall_repeat_4plex.raw
38201710		High	nFYVEHPEVAR	Unambiguous	2	1	N-Term(iTRAQ4plex)	HCD	1.0000	0.0000	1	1	8590	Unique	Used	0.658	1.139E4	9.062E3	150	4.12	0.00	0	43	123	3	502.25986	1504.76502	-1.01	29.53	2948	2948	MS2	21/40	Glioma_Nuclear_prep_Gradeall_repeat_4plex.raw
38201710		High	sSQSSSQQFSGIGR	Unambiguous	2	1	N-Term(iTRAQ4plex)	HCD	1.0000	0.0000	1	1	7908	Unique	Used	0.672	1.000E4	8.133E3	119	4.84	0.00	0	0	293	2	800.39044	1599.77361	-6.73	22.89	2229	2229	MS2	23/26	Glioma_Nuclear_prep_Gradeall_repeat_4plex.raw
38201710	High		aPLPDLYPFGTMR	Unambiguous	2	1	N-Term(iTRAQ4plex)	HCD	1.0000	0.0000	1	1	11594	Unique	Used	0.325	1.812E5	7.189E4	310	3.18	0.00	0	33	27	2	811.43036	1621.85344	0.31	53.36	6370	6370	MS2	12/24	Glioma_Nuclear_prep_Gradeall_4plex.raw
38201710	High		nFYVEHPEVAR	Unambiguous	2	1	N-Term(iTRAQ4plex)	HCD	1.0000	0.0000	1	1	8209	Unique	Used	0.489	1.608E5	9.604E4	142	2.89	0.00	0	13	13	2	752.87524	1504.74321	-15.50	27.83	2679	2679	MS2	15/20	Glioma_Nuclear_prep_Gradeall_4plex.raw
38201710	High		lTPYEVDELR	Unambiguous	2	1	N-Term(iTRAQ4plex)	HCD	1.0000	0.0000	1	1	9710	Unique	Used	0.522	1.314E6	8.370E5	243	4.00	0.00	0	9	4	2	689.86035	1378.71343	-14.56	38.76	4315	4315	MS2	16/18	Glioma_Nuclear_prep_Gradeall_4plex.raw
38201710		High	cTYLVLDEADR	Unambiguous	2	1	N-Term(iTRAQ4plex); C1(Methylthio)	HCD	1.0000	0.0000	1	1	11402	Unique	Used	0.624	4.999E3	3.772E3	123	4.09	0.00	0	34	300	2	744.35461	1487.70195	1.91	62.83	6015	6015	MS2	17/20	Glioma_Nuclear_prep_Gradeall_repeat_4plex.raw
38201710		High	dMVGIAQTGSGk	Unambiguous	2	1	N-Term(iTRAQ4plex); K12(iTRAQ4plex)	HCD	1.0000	0.0000	1	1	8387	Unique	Used	0.774	1.432E4	1.341E4	253	4.15	0.00	0	66	300	2	726.39417	1451.78105	3.00	27.53	2733	2733	MS2	18/22	Glioma_Nuclear_prep_Gradeall_repeat_4plex.raw
38201710	High		vLEEANQAINPk	Unambiguous	2	1	N-Term(iTRAQ4plex); K12(iTRAQ4plex)	HCD	1.0000	0.0000	1	1	9071	Unique	Used	0.541	1.492E5	9.851E4	234	4.87	0.00	0	11	15	2	807.44653	1613.88579	-15.09	34.06	3617	3617	MS2	21/22	Glioma_Nuclear_prep_Gradeall_4plex.raw
38201710	High		dMVGIAQTGSGk	Unambiguous	2	1	N-Term(iTRAQ4plex); K12(iTRAQ4plex)	HCD	1.0000	0.0000	1	1	8169	Unique	Used	0.569	2.863E5	1.987E5	206	3.94	0.00	0	5	7	2	726.38080	1451.75432	-15.41	27.56	2636	2636	MS2	19/22	Glioma_Nuclear_prep_Gradeall_4plex.raw
38201710	High		gTAYTFFTPGNLk	Unambiguous	2	1	N-Term(iTRAQ4plex); K13(iTRAQ4plex)	HCD	1.0000	0.0000	1	1	10533	Unique	Used	0.729	1.946E5	1.732E5	205	5.06	0.00	0	14	9	2	852.95264	1704.89800	-12.89	44.90	5213	5213	MS2	21/24	Glioma_Nuclear_prep_Gradeall_4plex.raw
38201710		High	eLAQQVQQVADDYGk	Unambiguous	2	1	N-Term(iTRAQ4plex); K15(iTRAQ4plex)	HCD	1.0000	0.0000	1	1	11222	Unique	Used	0.502	2.980E3	1.811E3	193	6.35	0.00	0	20	300	2	990.51953	1980.03179	2.06	60.09	5787	5787	MS2	26/28	Glioma_Nuclear_prep_Gradeall_repeat_4plex.raw
38201710	High		eLAQQVQQVADDYGk	Unambiguous	2	1	N-Term(iTRAQ4plex); K15(iTRAQ4plex)	HCD	1.0000	0.0000	1	1	10415	Unique	Used	0.515	1.088E5	6.832E4	163	6.27	0.00	0	6	20	2	990.50336	1979.99944	-14.27	43.96	5082	5082	MS2	26/28	Glioma_Nuclear_prep_Gradeall_4plex.raw
38201710	High		eLAQQVQQVADDYGk	Unambiguous	2	1	N-Term(iTRAQ4plex); K15(iTRAQ4plex)	HCD	1.0000	0.0000	1	1	10435	Unique	Used	0.746	1.277E4	1.163E4	115	3.94	0.00	0	17	6	3	660.67169	1980.00052	-13.72	44.13	5105	5105	MS2	22/56	Glioma_Nuclear_prep_Gradeall_4plex.raw
38201710		High	wDLSELPk	Unambiguous	2	1	N-Term(iTRAQ4plex); K8(iTRAQ4plex)	HCD	1.0000	0.0000	1	1	11023	Unique	Used	0.682	5.558E3	4.588E3	271	3.01	0.00	0	60	300	2	638.36493	1275.72258	3.01	57.44	5557	5557	MS2	13/14	Glioma_Nuclear_prep_Gradeall_repeat_4plex.raw
38201710		High	lIDFLESGk	Unambiguous	2	1	N-Term(iTRAQ4plex); K9(iTRAQ4plex)	HCD	1.0000	0.0000	1	1	12023	Unique	Used	0.521	1.722E4	1.085E4	281	3.49	0.00	0	16	300	2	655.38495	1309.76262	1.53	72.44	6813	6813	MS2	15/16	Glioma_Nuclear_prep_Gradeall_repeat_4plex.raw
38201710	High		lIDFLESGk	Unambiguous	2	1	N-Term(iTRAQ4plex); K9(iTRAQ4plex)	HCD	1.0000	0.0000	1	1	11339	Unique	Used	0.489	1.553E5	9.274E4	297	3.56	0.00	0	35	30	2	655.38434	1309.76140	0.60	51.22	6092	6092	MS2	15/16	Glioma_Nuclear_prep_Gradeall_4plex.raw
38201710	High		lIDFLESGk	Unambiguous	2	1	N-Term(iTRAQ4plex); K9(iTRAQ4plex)	HCD	1.0000	0.0000	1	1	11261	Unique	Used	0.522	1.603E5	1.021E5	297	3.80	0.00	0	21	39	2	655.38251	1309.75774	-2.20	50.53	6006	6006	MS2	15/16	Glioma_Nuclear_prep_Gradeall_4plex.raw
39725636	NP_059980.2	TMED9	54732	transmembrane emp24 domain-containing protein 9 precursor [Homo sapiens]	11.91	2	1	3	0.662	1;2	7.7	10.26	235	27.3	8.02																			
39725636		High	qLVEQVEQIQk	Unambiguous	1	1	N-Term(iTRAQ4plex); K11(iTRAQ4plex)	HCD	1.0000	0.0000	1	1	9957	Unique	Used	0.610	2.543E3	1.878E3	176	4.94	0.00	0	41	300	3	543.98541	1629.94168	0.15	44.52	4405	4405	MS2	21/40	Glioma_Nuclear_prep_Gradeall_repeat_4plex.raw
39725636		High	qLVEQVEQIQk	Unambiguous	1	1	N-Term(iTRAQ4plex); K11(iTRAQ4plex)	HCD	1.0000	0.0000	1	1	9932	Unique	Used	0.662	4.241E3	3.398E3	181	5.00	0.00	0	6	300	2	815.47552	1629.94377	1.43	44.21	4377	4377	MS2	20/20	Glioma_Nuclear_prep_Gradeall_repeat_4plex.raw
39725636	High		qLVEQVEQIQk	Unambiguous	1	1	N-Term(iTRAQ4plex); K11(iTRAQ4plex)	HCD	1.0000	0.0000	1	1	9368	Unique	Used	0.712	1.368E5	1.188E5	241	4.96	0.00	0	14	16	2	815.46167	1629.91606	-15.57	36.28	3943	3943	MS2	20/20	Glioma_Nuclear_prep_Gradeall_4plex.raw
39753957	NP_056417.2	TOR1AIP1	26092	torsin-1A-interacting protein 1 isoform 2 [Homo sapiens]	19.38	1	1	2	0.475	1;1	31.8	61.97	583	66.2	8.18																			
39753957		High	sIQEAPVSEDLVIR	Unambiguous	1	1	N-Term(iTRAQ4plex)	HCD	1.0000	0.0000	1	1	10550	Unique	Used	0.368	5.780E3	2.575E3	231	4.63	0.00	0	10	300	2	850.47345	1699.93962	2.86	51.64	5042	5042	MS2	21/26	Glioma_Nuclear_prep_Gradeall_repeat_4plex.raw
39753957	High		sIQEAPVSEDLVIR	Unambiguous	1	1	N-Term(iTRAQ4plex)	HCD	1.0000	0.0000	1	1	9876	Unique	Used	0.582	1.142E5	8.112E4	259	3.97	0.00	0	51	25	2	850.45850	1699.90972	-14.73	39.96	4495	4495	MS2	17/26	Glioma_Nuclear_prep_Gradeall_4plex.raw
40556376	NP_115958.2	GLYR1	84656	putative oxidoreductase GLYR1 [Homo sapiens]	8.68	1	2	3	1.507	2;1	16.4	11.88	553	60.5	9.17																			
40556376	High		fQQAVDAVEEFLR	Unambiguous	1	1	N-Term(iTRAQ4plex)	HCD	1.0000	0.0000	1	1	13090	Unique	Used	1.507	1.606E3	2.954E3	282	4.95	0.00	0	21	300	2	848.44629	1695.88530	1.76	72.69	8192	8192	MS2	21/24	Glioma_Nuclear_prep_Gradeall_4plex.raw
40556376		High	dLTIPESSTVk	Unambiguous	1	1	N-Term(iTRAQ4plex); K11(iTRAQ4plex)	HCD	1.0000	0.0000	1	1	9211	Unique	Used	1.302	1.033E4	1.628E4	264	3.58	0.00	0	30	148	2	739.42139	1477.83550	0.18	36.09	3606	3606	MS2	14/20	Glioma_Nuclear_prep_Gradeall_repeat_4plex.raw
40556376	High		dLTIPESSTVk	Unambiguous	1	1	N-Term(iTRAQ4plex); K11(iTRAQ4plex)	HCD	1.0000	0.0000	1	1	8776	Unique	Used	1.803	7.678E4	1.689E5	266	3.32	0.00	0	9	10	2	739.40778	1477.80828	-18.24	31.91	3296	3296	MS2	15/20	Glioma_Nuclear_prep_Gradeall_4plex.raw
41327773	NP_055644.2	DDX46	9879	probable ATP-dependent RNA helicase DDX46 isoform 2 [Homo sapiens]	4.07	2	3	4	0.659	2;2	9.1	11.45	1031	117.3	9.29																			
41327773		High	fDETEQALANER	Unambiguous	2	1	N-Term(iTRAQ4plex)	HCD	1.0000	0.0000	1	1	9487	Unique	Used	0.601	7.379E3	5.366E3	141	4.42	0.00	0	33	300	2	783.88232	1566.75737	3.62	39.11	3898	3898	MS2	18/22	Glioma_Nuclear_prep_Gradeall_repeat_4plex.raw
41327773	High		tIAFLLPMFR	Unambiguous	2	1	N-Term(iTRAQ4plex)	HCD	1.0000	0.0000	1	1	13035	Unique	Used	0.633	5.469E3	4.226E3	288	3.33	0.00	0	53	300	2	676.89948	1352.79167	2.61	70.58	8045	8045	MS2	13/18	Glioma_Nuclear_prep_Gradeall_4plex.raw
41327773		High	aMENIGELk	Unambiguous	2	1	N-Term(iTRAQ4plex); K9(iTRAQ4plex)	HCD	1.0000	0.0000	1	1	9357	Unique	Used	0.738	6.574E3	5.873E3	313	3.18	0.00	0	55	300	2	646.86188	1292.71648	3.23	37.69	3759	3759	MS2	15/16	Glioma_Nuclear_prep_Gradeall_repeat_4plex.raw
41327773	High		tIAFLLPmFR	Unambiguous	2	1	N-Term(iTRAQ4plex); M8(Oxidation)	HCD	1.0000	0.0000	1	1	12426	Unique	Used	0.685	1.204E4	1.007E4	239	3.05	0.00	0	62	300	2	684.89594	1368.78459	1.12	61.43	7280	7280	MS2	14/18	Glioma_Nuclear_prep_Gradeall_4plex.raw
48255898	NP_620614.2	SMARCA2	6595	probable global transcription activator SNF2L2 isoform b [Homo sapiens]	6.62	6	3	4	0.595	2;2	5.2	22.76	1572	179.2	7.27																			
48255898	High		iLLDPNSEEVSEk	Unambiguous	3	1	N-Term(iTRAQ4plex); K13(iTRAQ4plex)	HCD	1.0000	0.0000	1	1	9428	Unique	Used	0.554	8.536E4	5.767E4	261	4.21	0.00	0	44	33	2	880.96527	1760.92327	-16.36	36.71	4008	4008	MS2	17/24	Glioma_Nuclear_prep_Gradeall_4plex.raw
48255898		High	vLFGPEAPk	Unambiguous	3	1	N-Term(iTRAQ4plex); K9(iTRAQ4plex)	HCD	1.0000	0.0000	1	1	9600	Unique	Used	0.585	1.426E4	1.008E4	250	3.44	0.00	0	80	300	2	623.37854	1245.74980	4.20	40.40	4023	4023	MS2	14/16	Glioma_Nuclear_prep_Gradeall_repeat_4plex.raw
48255898	High		vLFGPEAPk	Unambiguous	3	1	N-Term(iTRAQ4plex); K9(iTRAQ4plex)	HCD	1.0000	0.0000	1	1	8970	Unique	Used	0.605	1.184E5	8.744E4	272	3.08	0.00	0	13	19	2	623.36536	1245.72344	-16.97	33.30	3506	3506	MS2	14/16	Glioma_Nuclear_prep_Gradeall_4plex.raw
48255898		High	aIEDGNLEEmEEEVR	Unambiguous	5	1	N-Term(iTRAQ4plex); M10(Oxidation)	HCD	1.0000	0.0000	1	1	9299	Unique	Used	0.627	1.928E3	1.461E3	40	3.96	0.00	0	15	300	2	961.94476	1922.88225	2.69	37.07	3698	3698	MS2	20/28	Glioma_Nuclear_prep_Gradeall_repeat_4plex.raw
49355765	NP_115657.2	ELAVL3	1995	ELAV-like protein 3 isoform 2 [Homo sapiens]	26.39	2	2	4	0.630	3;1	14.0	29.87	360	38.8	9.28																			
49355765		High	sLFGSIGDIESck	Unambiguous	2	1	N-Term(iTRAQ4plex); C12(Methylthio); K13(iTRAQ4plex)	HCD	1.0000	0.0000	1	1	12321	Unique	Used	0.579	3.147E3	2.206E3	149	4.31	0.00	0	30	300	2	845.43024	1689.85320	6.00	79.59	7357	7357	MS2	20/24	Glioma_Nuclear_prep_Gradeall_repeat_4plex.raw
49355765	High		sLFGSIGDIESck	Unambiguous	2	1	N-Term(iTRAQ4plex); C12(Methylthio); K13(iTRAQ4plex)	HCD	1.0000	0.0000	1	1	11822	Unique	Used	0.576	1.094E5	7.689E4	184	5.77	0.00	0	20	74	2	845.42706	1689.84685	2.24	55.39	6618	6618	MS2	22/24	Glioma_Nuclear_prep_Gradeall_4plex.raw
49355765	High		sLFGSIGDIESck	Unambiguous	2	1	N-Term(iTRAQ4plex); C12(Methylthio); K13(iTRAQ4plex)	HCD	1.0000	0.0000	1	1	11844	Unique	Used	0.681	4.944E3	4.109E3	164	4.18	0.00	0	36	57	3	563.95416	1689.84793	2.89	55.58	6642	6642	MS2	17/48	Glioma_Nuclear_prep_Gradeall_4plex.raw
49355765	High		tNLIVNYLPQNMTQDEFk	Unambiguous	2	1	N-Term(iTRAQ4plex); K18(iTRAQ4plex)	HCD	1.0000	0.0000	1	1	11798	Unique	Used	0.766	5.400E3	5.044E3	170	5.36	0.00	0	11	33	3	819.42944	2456.27378	0.14	55.19	6592	6592	MS2	22/68	Glioma_Nuclear_prep_Gradeall_4plex.raw
55956788	NP_005372.2	NCL	4691	nucleolin [Homo sapiens]	28.59	1	17	27	1.489	10;17	26.5	85.90	710	76.6	4.70																			
55956788	High		tLVLSNLSYSATEETLQEVFEk	Unambiguous	1	1	N-Term(iTRAQ4plex); K22(iTRAQ4plex)	HCD	1.0000	0.0000	1	1	13032	Unique	Used	1.469	2.421E2	4.340E2	141	8.76	0.00	0	9	300	3	930.49915	2789.48288	4.63	70.54	8042	8042	MS2	29/84	Glioma_Nuclear_prep_Gradeall_4plex.raw
55956788		High	nDLAVVDVR	Unambiguous	1	1	N-Term(iTRAQ4plex)	HCD	1.0000	0.0000	1	1	9007	Unique	Used	2.052	1.012E4	2.514E4	282	3.21	0.00	0	35	287	2	572.82654	1144.64580	1.31	33.80	3390	3390	MS2	15/16	Glioma_Nuclear_prep_Gradeall_repeat_4plex.raw
55956788		High	eVFEDAAEIR	Unambiguous	1	1	N-Term(iTRAQ4plex)	HCD	1.0000	0.0000	1	1	9891	Unique	Used	1.425	1.180E4	2.034E4	212	4.03	0.00	0	4	300	2	661.84033	1322.67339	1.86	43.67	4331	4331	MS2	17/18	Glioma_Nuclear_prep_Gradeall_repeat_4plex.raw
55956788		High	eALNScNk	Unambiguous	1	1	N-Term(iTRAQ4plex); C6(Methylthio); K8(iTRAQ4plex)	HCD	1.0000	0.0000	1	1	7863	Unique	Used	1.352	3.963E4	6.481E4	92	3.28	0.00	0	30	87	2	606.79822	1212.58916	-5.25	22.49	2182	2182	MS2	14/14	Glioma_Nuclear_prep_Gradeall_repeat_4plex.raw
55956788	High		kFGYVDFESAEDLEk	Unambiguous	1	1	N-Term(iTRAQ4plex); K1(iTRAQ4plex); K15(iTRAQ4plex)	HCD	1.0000	0.0000	1	1	10212	Unique	Used	1.286	2.018E4	3.165E4	159	6.72	0.00	1	19	11	3	737.04047	2209.10685	-14.53	42.44	4861	4861	MS2	26/56	Glioma_Nuclear_prep_Gradeall_4plex.raw
55956788		High	sISLYYTGEk	Unambiguous	1	1	N-Term(iTRAQ4plex); K10(iTRAQ4plex)	HCD	1.0000	0.0000	1	1	9773	Unique	Used	1.489	1.889E4	3.403E4	270	4.03	0.00	0	53	300	2	724.89935	1448.79143	2.69	42.36	4206	4206	MS2	15/18	Glioma_Nuclear_prep_Gradeall_repeat_4plex.raw
55956788		High	eAMEDGEIDGNk	Unambiguous	1	1	N-Term(iTRAQ4plex); K12(iTRAQ4plex)	HCD	1.0000	0.0000	1	1	7924	Unique	Used	1.572	2.518E4	4.789E4	59	5.18	0.00	0	28	72	2	798.37744	1595.74761	0.88	23.05	2246	2246	MS2	21/22	Glioma_Nuclear_prep_Gradeall_repeat_4plex.raw
55956788		High	gLSEDTTEETLk	Unambiguous	1	1	N-Term(iTRAQ4plex); K12(iTRAQ4plex)	HCD	1.0000	0.0000	1	1	8584	Unique	Used	1.525	6.230E3	1.150E4	176	5.05	0.00	0	78	300	2	805.91699	1610.82671	-6.00	29.46	2941	2941	MS2	21/22	Glioma_Nuclear_prep_Gradeall_repeat_4plex.raw
55956788		High	gLSEDTTEETLk	Unambiguous	1	1	N-Term(iTRAQ4plex); K12(iTRAQ4plex)	HCD	1.0000	0.0000	1	1	8605	Unique	Used	1.486	3.321E4	5.970E4	196	5.03	0.00	0	7	44	2	805.92334	1610.83940	1.88	29.70	2964	2964	MS2	22/22	Glioma_Nuclear_prep_Gradeall_repeat_4plex.raw
55956788	High		eAMEDGEIDGNk	Unambiguous	1	1	N-Term(iTRAQ4plex); K12(iTRAQ4plex)	HCD	1.0000	0.0000	1	1	7820	Unique	Used	1.365	2.202E5	3.668E5	53	4.99	0.00	0	5	6	2	798.37726	1595.74724	0.65	25.09	2258	2258	MS2	21/22	Glioma_Nuclear_prep_Gradeall_4plex.raw
55956788		High	gFGFVDFNSEEDAk	Unambiguous	1	1	N-Term(iTRAQ4plex); K14(iTRAQ4plex)	HCD	1.0000	0.0000	1	1	11297	Unique	Used	1.439	4.138E3	7.202E3	92	5.64	0.00	0	20	300	2	925.44861	1849.88994	2.81	61.20	5880	5880	MS2	23/26	Glioma_Nuclear_prep_Gradeall_repeat_4plex.raw
55956788		High	gFGFVDFNSEEDAk	Unambiguous	1	1	N-Term(iTRAQ4plex); K14(iTRAQ4plex)	HCD	1.0000	0.0000	1	1	11365	Unique	Used	1.292	2.220E3	3.471E3	96	4.94	0.00	0	40	300	2	925.44800	1849.88872	2.15	62.32	5971	5971	MS2	25/26	Glioma_Nuclear_prep_Gradeall_repeat_4plex.raw

55956788	High		fGYVDFESAEDLEk	Unambiguous	1	1	N-Term(iTRAQ4plex); K14(iTRAQ4plex)	HCD	1.0000	0.0000	1	1	11023	Unique	Used	2.649	7.563E4	2.444E5	118	5.24	0.00	0	9	19	2	968.97314	1936.93901	-1.49	48.62	5746	5746	MS2	23/26	Glioma_Nuclear_prep_Gradeall_4plex.raw
55956788	High		fGYVDFESAEDLEk	Unambiguous	1	1	N-Term(iTRAQ4plex); K14(iTRAQ4plex)	HCD	1.0000	0.0000	1	1	10998	Unique	Used	2.544	3.923E4	1.218E5	71	5.45	0.00	0	29	59	2	968.95886	1936.91045	-16.24	48.43	5718	5718	MS2	22/26	Glioma_Nuclear_prep_Gradeall_4plex.raw
55956788	High		gFGFVDFNSEEDAk	Unambiguous	1	1	N-Term(iTRAQ4plex); K14(iTRAQ4plex)	HCD	1.0000	0.0000	1	1	10503	Unique	Used	1.311	2.278E5	3.644E5	53	5.10	0.00	0	29	10	2	925.43341	1849.85954	-13.62	44.67	5180	5180	MS2	22/26	Glioma_Nuclear_prep_Gradeall_4plex.raw
55956788	High		vEGTEPTTAFNLFVGNLNFNk	Unambiguous	1	1	N-Term(iTRAQ4plex); K21(iTRAQ4plex)	HCD	1.0000	0.0000	1	1	12804	Unique	Used	2.576	1.953E2	6.137E2	143	6.72	0.00	0	13	200	3	867.45880	2600.36185	0.72	66.42	7719	7719	MS2	27/80	Glioma_Nuclear_prep_Gradeall_4plex.raw
55956788	High		vEGTEPTTAFNLFVGNLNFNk	Unambiguous	1	1	N-Term(iTRAQ4plex); K21(iTRAQ4plex)	HCD	1.0000	0.0000	1	1	12783	Unique	Used	2.298	9.664E2	2.710E3	125	7.06	0.00	0	0	300	2	1300.68652	2600.36577	2.23	66.12	7694	7694	MS2	28/40	Glioma_Nuclear_prep_Gradeall_4plex.raw
55956788	High		vEGTEPTTAFNLFVGNLNFNk	Unambiguous	1	1	N-Term(iTRAQ4plex); K21(iTRAQ4plex)	HCD	1.0000	0.0000	1	1	12755	Unique	Used	1.480	6.545E2	1.182E3	155	7.09	0.00	0	12	175	3	867.46100	2600.36844	3.26	65.69	7659	7659	MS2	26/80	Glioma_Nuclear_prep_Gradeall_4plex.raw
55956788		High	vTQDELk	Unambiguous	1	1	N-Term(iTRAQ4plex); K7(iTRAQ4plex)	HCD	1.0000	0.0000	1	1	7569	Unique	Used	1.317	1.085E5	1.729E5	413	2.91	0.00	0	36	33	2	560.82391	1120.64055	-4.18	19.69	1868	1868	MS2	12/12	Glioma_Nuclear_prep_Gradeall_repeat_4plex.raw
55956788		High	aLELTGLk	Unambiguous	1	1	N-Term(iTRAQ4plex); K8(iTRAQ4plex)	HCD	1.0000	0.0000	1	1	9975	Unique	Used	2.517	9.656E3	2.941E4	192	3.34	0.00	0	50	300	2	566.86230	1132.71733	-0.60	44.76	4425	4425	MS2	14/14	Glioma_Nuclear_prep_Gradeall_repeat_4plex.raw
55956788		High	aLELTGLk	Unambiguous	1	1	N-Term(iTRAQ4plex); K8(iTRAQ4plex)	HCD	1.0000	0.0000	1	1	10056	Unique	Used	1.924	8.493E3	1.977E4	196	3.38	0.00	0	27	300	2	566.86285	1132.71843	0.37	45.76	4514	4514	MS2	14/14	Glioma_Nuclear_prep_Gradeall_repeat_4plex.raw
55956788		High	gIAYIEFk	Unambiguous	1	1	N-Term(iTRAQ4plex); K8(iTRAQ4plex)	HCD	1.0000	0.0000	1	1	10782	Unique	Used	1.604	5.235E3	1.016E4	198	3.40	0.00	0	55	300	2	614.86273	1228.71819	0.15	54.45	5293	5293	MS2	14/14	Glioma_Nuclear_prep_Gradeall_repeat_4plex.raw
55956788		High	vAVATPAk	Unambiguous	1	1	N-Term(iTRAQ4plex); K8(iTRAQ4plex)	HCD	1.0000	0.0000	1	1	7587	Unique	Used	1.602	6.865E4	1.331E5	143	3.12	0.00	0	56	38	2	522.83264	1044.65801	-7.26	19.83	1887	1887	MS2	12/14	Glioma_Nuclear_prep_Gradeall_repeat_4plex.raw
55956788		High	aLVATPGk	Unambiguous	1	1	N-Term(iTRAQ4plex); K8(iTRAQ4plex)	HCD	1.0000	0.0000	1	1	7713	Unique	Used	1.574	3.852E4	7.337E4	171	2.88	0.00	0	42	103	2	522.83295	1044.65862	-6.67	21.07	2024	2024	MS2	11/14	Glioma_Nuclear_prep_Gradeall_repeat_4plex.raw
55956788		High	gIAYIEFk	Unambiguous	1	1	N-Term(iTRAQ4plex); K8(iTRAQ4plex)	HCD	1.0000	0.0000	1	1	10870	Unique	Used	1.448	7.436E3	1.303E4	207	3.59	0.00	0	28	300	2	614.86243	1228.71758	-0.35	55.56	5390	5390	MS2	14/14	Glioma_Nuclear_prep_Gradeall_repeat_4plex.raw
55956788	High		gIAYIEFk	Unambiguous	1	1	N-Term(iTRAQ4plex); K8(iTRAQ4plex)	HCD	1.0000	0.0000	1	1	9990	Unique	Used	1.446	8.338E5	1.471E6	225	3.47	0.00	0	34	2	2	614.85315	1228.69902	-15.45	40.78	4619	4619	MS2	14/14	Glioma_Nuclear_prep_Gradeall_4plex.raw
55956788		High	tGISDVFAk	Unambiguous	1	1	N-Term(iTRAQ4plex); K9(iTRAQ4plex)	HCD	1.0000	0.0000	1	1	9664	Unique	Used	2.321	3.294E4	9.250E4	268	3.71	0.00	0	16	105	2	613.35559	1225.70391	0.66	41.14	4092	4092	MS2	16/16	Glioma_Nuclear_prep_Gradeall_repeat_4plex.raw
56118234	NP_859067.2	CENPV	201161	centromere protein V [Homo sapiens]	23.16	1	3	4	0.597	2;2	14.0	19.48	272	29.7	9.73																			
56118234	High		sGASAAPAASAAAALAPSATR	Unambiguous	1	1	N-Term(iTRAQ4plex)	HCD	1.0000	0.0000	1	1	9064	Unique	Used	0.482	2.927E4	1.722E4	176	6.60	0.00	0	35	31	2	957.49518	1913.98308	-17.32	34.01	3609	3609	MS2	30/40	Glioma_Nuclear_prep_Gradeall_4plex.raw
56118234		High	cGVQSFYTPR	Unambiguous	1	1	N-Term(iTRAQ4plex); C1(Methylthio)	HCD	1.0000	0.0000	1	1	10325	Unique	Used	0.636	1.193E4	9.179E3	70	3.58	0.00	0	46	300	2	674.32123	1347.63518	3.38	48.97	4802	4802	MS2	17/18	Glioma_Nuclear_prep_Gradeall_repeat_4plex.raw
56118234	High		cGVQSFYTPR	Unambiguous	1	1	N-Term(iTRAQ4plex); C1(Methylthio)	HCD	1.0000	0.0000	1	1	9657	Unique	Used	0.664	1.615E5	1.307E5	55	2.91	0.00	0	37	17	2	674.30853	1347.60979	-15.46	38.37	4257	4257	MS2	17/18	Glioma_Nuclear_prep_Gradeall_4plex.raw
56118234		High	sNPGGFGIAPHcLDEGTVR	Unambiguous	1	1	N-Term(iTRAQ4plex); C12(Methylthio)	HCD	1.0000	0.0000	1	1	10907	Unique	Used	0.558	1.235E3	8.338E2	66	5.32	0.00	0	37	300	3	706.34021	2117.00608	1.66	56.02	5431	5431	MS2	30/72	Glioma_Nuclear_prep_Gradeall_repeat_4plex.raw
56118310	NP_073568.2	NUCKS1	64710	nuclear ubiquitous casein and cyclin-dependent kinase substrate 1 [Homo sapiens]	11.93	1	3	3	3.108	1;2	34.6	16.88	243	27.3	5.08																			
56118310		High	vVDYSQFQESDDADEDYGR	Unambiguous	1	1	N-Term(iTRAQ4plex)	HCD	1.0000	0.0000	1	1	10196	Unique	Used	3.492	6.261E2	2.645E3	16	6.29	0.00	0	3	300	2	1191.51172	2382.01616	1.46	47.42	4664	4664	MS2	26/36	Glioma_Nuclear_prep_Gradeall_repeat_4plex.raw
56118310		High	kVVDYSQFQESDDADEDYGR	Unambiguous	1	1	N-Term(iTRAQ4plex); K1(iTRAQ4plex)	HCD	1.0000	0.0000	1	1	9579	Unique	Used	3.108	2.538E2	9.543E2	37	7.25	0.00	1	5	131	3	885.40448	2654.19889	-4.08	40.18	4001	4001	MS2	33/76	Glioma_Nuclear_prep_Gradeall_repeat_4plex.raw
56118310	High		aTVTPSPVk	Unambiguous	1	1	N-Term(iTRAQ4plex); K9(iTRAQ4plex)	HCD	1.0000	0.0000	1	1	7476	Unique	Used	1.679	5.643E4	1.156E5	225	3.34	0.00	0	35	20	2	594.36053	1187.71379	-8.45	22.65	1885	1885	MS2	13/16	Glioma_Nuclear_prep_Gradeall_4plex.raw
56676330	NP_057371.2	HP1BP3	50809	heterochromatin protein 1-binding protein 3 [Homo sapiens]	26.76	1	14	41	2.025	17;24	24.0	111.59	553	61.2	9.67																			
56676330		High	sGASVVAIR	Unambiguous	1	1	N-Term(iTRAQ4plex)	HCD	1.0000	0.0000	1	1	7791	Unique	Used	3.006	1.197E5	4.355E5	218	3.05	0.00	0	14	15	2	502.30493	1003.60259	0.88	21.79	2106	2106	MS2	16/16	Glioma_Nuclear_prep_Gradeall_repeat_4plex.raw
56676330		High	tIPSWATLSASQLAR	Unambiguous	1	1	N-Term(iTRAQ4plex)	HCD	1.0000	0.0000	1	1	11989	Unique	Used	1.686	1.974E2	4.027E2	201	5.10	0.00	0	6	300	3	582.66077	1745.96775	0.59	71.82	6764	6764	MS2	26/56	Glioma_Nuclear_prep_Gradeall_repeat_4plex.raw
56676330		High	lEDVLPLAFTR	Unambiguous	1	1	N-Term(iTRAQ4plex)	HCD	1.0000	0.0000	1	1	12387	Unique	Used	1.601	3.302E4	6.397E4	225	3.52	0.00	0	5	52	2	709.41357	1417.81987	1.89	82.71	7575	7575	MS2	19/20	Glioma_Nuclear_prep_Gradeall_repeat_4plex.raw
56676330		High	tIPSWATLSASQLAR	Unambiguous	1	1	N-Term(iTRAQ4plex)	HCD	1.0000	0.0000	1	1	11988	Unique	Used	1.594	2.784E3	5.369E3	119	4.14	0.00	0	8	300	2	873.48877	1745.97026	2.03	71.80	6763	6763	MS2	21/28	Glioma_Nuclear_prep_Gradeall_repeat_4plex.raw
56676330		High	lEDVLPLAFTR	Unambiguous	1	1	N-Term(iTRAQ4plex)	HCD	1.0000	0.0000	1	1	12370	Unique	Used	1.446	1.124E3	1.966E3	248	3.00	0.00	0	23	300	2	709.41284	1417.81841	0.86	81.70	7506	7506	MS2	15/20	Glioma_Nuclear_prep_Gradeall_repeat_4plex.raw
56676330		High	tIPSWATLSASQLAR	Unambiguous	1	1	N-Term(iTRAQ4plex)	HCD	1.0000	0.0000	1	1	11933	Unique	Used	1.436	1.183E3	2.054E3	126	3.63	0.00	0	32	300	2	873.48883	1745.97038	2.10	70.80	6683	6683	MS2	20/28	Glioma_Nuclear_prep_Gradeall_repeat_4plex.raw
56676330		High	tIPSWATLSASQLAR	Unambiguous	1	1	N-Term(iTRAQ4plex)	HCD	1.0000	0.0000	1	1	11935	Unique	Used	1.408	1.366E2	2.326E2	194	3.76	0.00	0	38	300	3	582.66101	1745.96848	1.01	70.82	6685	6685	MS2	21/56	Glioma_Nuclear_prep_Gradeall_repeat_4plex.raw
56676330	High		lEDVLPLAFTR	Unambiguous	1	1	N-Term(iTRAQ4plex)	HCD	1.0000	0.0000	1	1	12035	Unique	Used	1.828	9.428E5	2.103E6	223	3.61	0.00	0	6	3	2	709.41302	1417.81877	1.11	57.34	6851	6851	MS2	19/20	Glioma_Nuclear_prep_Gradeall_4plex.raw
56676330	High		tIPSWATLSASQLAR	Unambiguous	1	1	N-Term(iTRAQ4plex)	HCD	1.0000	0.0000	1	1	11166	Unique	Used	1.731	2.540E5	5.365E5	143	4.16	0.00	0	27	6	2	873.48871	1745.97014	1.96	49.78	5903	5903	MS2	22/28	Glioma_Nuclear_prep_Gradeall_4plex.raw
56676330	High		tIPSWATLSASQLAR	Unambiguous	1	1	N-Term(iTRAQ4plex)	HCD	1.0000	0.0000	1	1	11190	Unique	Used	1.698	3.604E5	7.468E5	137	4.44	0.00	0	9	4	2	873.48132	1745.95537	-6.50	49.95	5929	5929	MS2	22/28	Glioma_Nuclear_prep_Gradeall_4plex.raw
56676330	High		lEDVLPLAFTR	Unambiguous	1	1	N-Term(iTRAQ4plex)	HCD	1.0000	0.0000	1	1	12105	Unique	Used	1.570	4.163E5	7.974E5	214	3.60	0.00	0	5	8	2	709.41656	1417.82585	6.11	58.07	6928	6928	MS2	18/20	Glioma_Nuclear_prep_Gradeall_4plex.raw
56676330	High		tcSTTALk	Unambiguous	1	1	N-Term(iTRAQ4plex); C2(Methylthio); K8(iTRAQ4plex)	HCD	1.0000	0.0000	1	1	7877	Unique	Used	1.838	3.841E5	8.613E5	138	3.19	0.00	0	29	4	2	579.80774	1158.60820	-1.65	25.49	2320	2320	MS2	14/14	Glioma_Nuclear_prep_Gradeall_4plex.raw
56676330		High	kYVSQYYPk	Unambiguous	1	1	N-Term(iTRAQ4plex); K1(iTRAQ4plex); K9(iTRAQ4plex)	HCD	1.0000	0.0000	1	1	8352	Unique	Used	2.025	1.427E4	3.497E4	246	3.30	0.00	1	34	111	2	804.46576	1607.92424	5.29	27.20	2697	2697	MS2	14/16	Glioma_Nuclear_prep_Gradeall_repeat_4plex.raw
56676330	High		kYVSQYYPk	Unambiguous	1	1	N-Term(iTRAQ4plex); K1(iTRAQ4plex); K9(iTRAQ4plex)	HCD	1.0000	0.0000	1	1	8028	Unique	Used	2.064	1.539E5	3.876E5	226	2.95	0.00	1	11	11	2	804.44836	1607.88945	-16.34	26.55	2483	2483	MS2	14/16	Glioma_Nuclear_prep_Gradeall_4plex.raw
56676330		High	mDAILTEAlk	Unambiguous	1	1	N-Term(iTRAQ4plex); K10(iTRAQ4plex)	HCD	1.0000	0.0000	1	1	11432	Unique	Used	3.212	7.307E3	2.840E4	251	4.30	0.00	0	8	137	2	696.90472	1392.80217	0.76	63.25	6051	6051	MS2	18/18	Glioma_Nuclear_prep_Gradeall_repeat_4plex.raw
56676330		High	mDAILTEAlk	Unambiguous	1	1	N-Term(iTRAQ4plex); K10(iTRAQ4plex)	HCD	1.0000	0.0000	1	1	11357	Unique	Used	2.147	2.004E3	5.205E3	279	3.94	0.00	0	55	300	2	696.90436	1392.80144	0.23	62.22	5962	5962	MS2	18/18	Glioma_Nuclear_prep_Gradeall_repeat_4plex.raw
56676330	High		mDAILTEAlk	Unambiguous	1	1	N-Term(iTRAQ4plex); K10(iTRAQ4plex)	HCD	1.0000	0.0000	1	1	10539	Unique	Used	2.927	6.108E4	2.181E5	298	3.97	0.00	0	38	20	2	696.89337	1392.77947	-15.54	44.94	5219	5219	MS2	18/18	Glioma_Nuclear_prep_Gradeall_4plex.raw
56676330	High		mDAILTEAlk	Unambiguous	1	1	N-Term(iTRAQ4plex); K10(iTRAQ4plex)	HCD	1.0000	0.0000	1	1	10627	Unique	Used	2.395	1.471E5	4.299E5	276	3.77	0.00	0	16	7	2	696.89380	1392.78032	-14.93	45.59	5315	5315	MS2	18/18	Glioma_Nuclear_prep_Gradeall_4plex.raw
56676330		High	gASGSFVVVQk	Unambiguous	1	1	N-Term(iTRAQ4plex); K11(iTRAQ4plex)	HCD	1.0000	0.0000	1	1	8838	Unique	Used	3.431	4.989E3	2.071E4	251	4.00	0.00	0	6	30	3	456.26984	1366.79496	1.20	32.05	3209	3209	MS2	18/40	Glioma_Nuclear_prep_Gradeall_repeat_4plex.raw
56676330		High	gASGSFVVVQk	Unambiguous	1	1	N-Term(iTRAQ4plex); K11(iTRAQ4plex)	HCD	1.0000	0.0000	1	1	8783	Unique	Used	2.691	1.485E4	4.834E4	296	4.32	0.00	0	16	91	2	683.89032	1366.77336	-14.60	31.44	3151	3151	MS2	20/20	Glioma_Nuclear_prep_Gradeall_repeat_4plex.raw
56676330		High	gASGSFVVVQk	Unambiguous	1	1	N-Term(iTRAQ4plex); K11(iTRAQ4plex)	HCD	1.0000	0.0000	1	1	8837	Unique	Used	2.629	7.913E4	2.516E5	254	4.55	0.00	0	8	16	2	683.90131	1366.79534	1.48	32.05	3208	3208	MS2	20/20	Glioma_Nuclear_prep_Gradeall_repeat_4plex.raw
56676330		High	sSAVDPEPQVk	Unambiguous	1	1	N-Term(iTRAQ4plex); K11(iTRAQ4plex)	HCD	1.0000	0.0000	1	1	7607	Unique	Used	2.053	1.102E4	2.738E4	195	4.09	0.00	0	7	139	2	722.89386	1444.78044	-5.66	19.99	1908	1908	MS2	19/20	Glioma_Nuclear_prep_Gradeall_repeat_4plex.raw
56676330	High		gASGSFVVVQk	Unambiguous	1	1	N-Term(iTRAQ4plex); K11(iTRAQ4plex)	HCD	1.0000	0.0000	1	1	8383	Unique	Used	1.927	4.358E4	1.025E5	311	4.41	0.00	0	20	10	2	683.88916	1366.77104	-16.30	29.07	2868	2868	MS2	20/20	Glioma_Nuclear_prep_Gradeall_4plex.raw
56676330	High		sSAVDPEPQVk	Unambiguous	1	1	N-Term(iTRAQ4plex); K11(iTRAQ4plex)	HCD	1.0000	0.0000	1	1	7567	Unique	Used	1.771	1.771E5	3.826E5	191	4.17	0.00	0	12	6	2	722.89648	1444.78569	-2.03	23.30	1984	1984	MS2	19/20	Glioma_Nuclear_prep_Gradeall_4plex.raw
56676330		High	aLPLIVGAQLIHADk	Unambiguous	1	1	N-Term(iTRAQ4plex); K15(iTRAQ4plex)	HCD	1.0000	0.0000	1	1	12209	Unique	Used	2.542	1.646E3	5.062E3	34	7.28	0.00	0	8	259	3	616.38416	1847.13791	1.17	76.37	7119	7119	MS2	39/56	Glioma_Nuclear_prep_Gradeall_repeat_4plex.raw
56676330		High	aLPLIVGAQLIHADk	Unambiguous	1	1	N-Term(iTRAQ4plex); K15(iTRAQ4plex)	HCD	1.0000	0.0000	1	1	12247	Unique	Used	2.338	5.121E2	1.449E3	37	7.57	0.00	0	26	300	3	616.38354	1847.13608	0.18	77.41	7197	7197	MS2	39/56	Glioma_Nuclear_prep_Gradeall_repeat_4plex.raw
56676330		High	aLPLIVGAQLIHADk	Unambiguous	1	1	N-Term(iTRAQ4plex); K15(iTRAQ4plex)	HCD	1.0000	0.0000	1	1	12198	Unique	Used	1.908	1.099E3	2.536E3	34	5.00	0.00	0	28	300	2	924.07251	1847.13774	1.08	76.03	7093	7093	MS2	25/28	Glioma_Nuclear_prep_Gradeall_repeat_4plex.raw
56676330		High	aLPLIVGAQLIHADk	Unambiguous	1	1	N-Term(iTRAQ4plex); K15(iTRAQ4plex)	HCD	1.0000	0.0000	1	1	12165	Unique	Used	1.868	7.325E2	1.656E3	37	6.47	0.00	0	61	300	3	616.38324	1847.13517	-0.31	75.35	7040	7040	MS2	36/56	Glioma_Nuclear_prep_Gradeall_repeat_4plex.raw
56676330	High		aLPLIVGAQLIHADk	Unambiguous	1	1	N-Term(iTRAQ4plex); K15(iTRAQ4plex)	HCD	1.0000	0.0000	1	1	11303	Unique	Used	2.329	6.843E4	1.945E5	45	7.29	0.00	0	16	7	3	616.38104	1847.12857	-3.88	50.90	6052	6052	MS2	37/56	Glioma_Nuclear_prep_Gradeall_4plex.raw
56676330	High		aLPLIVGAQLIHADk	Unambiguous	1	1	N-Term(iTRAQ4plex); K15(iTRAQ4plex)	HCD	1.0000	0.0000	1	1	11386	Unique	Used	2.163	1.494E4	3.944E4	38	7.12	0.00	0	47	25	3	616.38300	1847.13443	-0.71	51.62	6143	6143	MS2	39/56	Glioma_Nuclear_prep_Gradeall_4plex.raw
56676330	High		aLPLIVGAQLIHADk	Unambiguous	1	1	N-Term(iTRAQ4plex); K15(iTRAQ4plex)	HCD	1.0000	0.0000	1	1	11284	Unique	Used	2.116	1.953E4	5.043E4	37	6.37	0.00	0	31	97	2	924.07147	1847.13567	-0.04	50.73	6031	6031	MS2	26/28	Glioma_Nuclear_prep_Gradeall_4plex.raw
56676330	High		yVLENHPGTNSNYQMHLLk	Unambiguous	1	1	N-Term(iTRAQ4plex); K19(iTRAQ4plex)	HCD	1.0000	0.0000	1	1	9334	Unique	Used	2.276	1.513E4	4.200E4	135	6.53	0.00	0	4	8	3	849.42743	2546.26773	-15.20	36.03	3906	3906	MS2	29/72	Glioma_Nuclear_prep_Gradeall_4plex.raw
56676330		High	yVSQYYPk	Unambiguous	1	1	N-Term(iTRAQ4plex); K8(iTRAQ4plex)	HCD	1.0000	0.0000	1	1	9046	Unique	Used	2.030	1.107E4	2.719E4	265	3.40	0.00	0	54	300	2	668.36646	1335.72563	5.19	34.26	3431	3431	MS2	13/14	Glioma_Nuclear_prep_Gradeall_repeat_4plex.raw
56676330		High	gEPENEEkEENk	Unambiguous	1	1	N-Term(iTRAQ4plex); K8(iTRAQ4plex); K12(iTRAQ4plex)	HCD	1.0000	0.0000	1	1	6726	Unique	Used	1.799	1.838E3	3.999E3	129	4.00	0.00	1	5	299	2	932.47113	1863.93498	2.85	12.34	979	979	MS2	21/22	Glioma_Nuclear_prep_Gradeall_repeat_4plex.raw
56676330		High	gASGTFQLk	Unambiguous	1	1	N-Term(iTRAQ4plex); K9(iTRAQ4plex)	HCD	1.0000	0.0000	1	1	8486	Unique	Used	2.115	4.733E4	1.211E5	233	3.76	0.00	0	15	47	2	598.84753	1196.68779	0.01	28.47	2838	2838	MS2	16/16	Glioma_Nuclear_prep_Gradeall_repeat_4plex.raw
56676330		High	gQLEQITGk	Unambiguous	1	1	N-Term(iTRAQ4plex); K9(iTRAQ4plex)	HCD	1.0000	0.0000	1	1	8525	Unique	Used	2.025	1.006E5	2.464E5	249	3.69	0.00	0	8	15	2	631.36591	1261.72453	-8.66	28.90	2880	2880	MS2	16/16	Glioma_Nuclear_prep_Gradeall_repeat_4plex.raw
56676330		High	gQLEQITGk	Unambiguous	1	1	N-Term(iTRAQ4plex); K9(iTRAQ4plex)	HCD	1.0000	0.0000	1	1	8529	Unique	Used	1.306	6.988E3	1.104E4	299	3.57	0.00	0	21	51	3	421.24612	1261.72382	-9.22	28.93	2884	2884	MS2	16/32	Glioma_Nuclear_prep_Gradeall_repeat_4plex.raw
56676330	High		gQLEQITGk	Unambiguous	1	1	N-Term(iTRAQ4plex); K9(iTRAQ4plex)	HCD	1.0000	0.0000	1	1	8214	Unique	Used	2.112	4.041E5	1.042E6	225	3.11	0.00	0	9	3	2	631.36017	1261.71306	-17.75	27.88	2685	2685	MS2	16/16	Glioma_Nuclear_prep_Gradeall_4plex.raw
56676330	High		gASGTFQLk	Unambiguous	1	1	N-Term(iTRAQ4plex); K9(iTRAQ4plex)	HCD	1.0000	0.0000	1	1	8095	Unique	Used	1.573	4.409E5	8.461E5	266	3.67	0.00	0	34	6	2	598.83575	1196.66423	-19.68	27.04	2556	2556	MS2	16/16	Glioma_Nuclear_prep_Gradeall_4plex.raw
56676330		High	mDAILTEAlk	Unambiguous	1	1	N-Term(iTRAQ4plex); M1(Oxidation); K10(iTRAQ4plex)	HCD	1.0000	0.0000	1	1	9909	Unique	Used	2.767	4.733E3	1.585E4	254	4.40	0.00	0	21	300	2	704.90326	1408.79924	2.28	43.90	4351	4351	MS2	18/18	Glioma_Nuclear_prep_Gradeall_repeat_4plex.raw
56676330	High		mDAILTEAlk	Unambiguous	1	1	N-Term(iTRAQ4plex); M1(Oxidation); K10(iTRAQ4plex)	HCD	1.0000	0.0000	1	1	9353	Unique	Used	1.964	1.492E5	3.576E5	253	3.79	0.00	0	17	6	2	704.89014	1408.77300	-16.35	36.17	3927	3927	MS2	16/18	Glioma_Nuclear_prep_Gradeall_4plex.raw
62750354	NP_954659.1	MATR3	9782	matrin-3 isoform a [Homo sapiens]	23.49	3	11	29	0.644	17;12	12.9	126.57	847	94.6	6.25																			
62750354		High	tEEGPTLSYGR	Unambiguous	1	1	N-Term(iTRAQ4plex)	HCD	1.0000	0.0000	1	1	8234	Unique	Used	0.474	4.768E4	2.737E4	153	3.36	0.00	0	12	103	2	677.34473	1353.68218	4.04	26.07	2573	2573	MS2	16/20	Glioma_Nuclear_prep_Gradeall_repeat_4plex.raw
62750354		High	rTEEGPTLSYGR	Unambiguous	1	1	N-Term(iTRAQ4plex)	HCD	1.0000	0.0000	1	1	7705	Unique	Used	0.581	3.286E3	2.311E3	175	3.32	0.00	1	0	300	2	755.38690	1509.76653	-7.49	20.98	2015	2015	MS2	17/22	Glioma_Nuclear_prep_Gradeall_repeat_4plex.raw
62750354		High	gDADQASNILASFGLSAR	Unambiguous	1	1	N-Term(iTRAQ4plex)	HCD	1.0000	0.0000	1	1	12393	Unique	Used	0.610	9.668E2	7.133E2	144	4.95	0.00	0	12	300	2	968.99695	1936.98662	1.06	83.02	7597	7597	MS2	26/34	Glioma_Nuclear_prep_Gradeall_repeat_4plex.raw
62750354		High	gPLPLSSQHR	Unambiguous	1	1	N-Term(iTRAQ4plex)	HCD	1.0000	0.0000	1	1	7537	Unique	Used	0.631	3.377E4	2.576E4	330	3.40	0.00	0	21	146	2	618.34619	1235.68511	-10.21	19.41	1834	1834	MS2	15/18	Glioma_Nuclear_prep_Gradeall_repeat_4plex.raw
62750354		High	gPSLNPVLDYDHGSR	Unambiguous	1	1	N-Term(iTRAQ4plex)	HCD	1.0000	0.0000	1	1	9722	Unique	Used	0.643	4.466E3	3.474E3	101	5.51	0.00	0	33	157	3	590.96924	1770.89316	2.25	41.77	4153	4153	MS2	23/56	Glioma_Nuclear_prep_Gradeall_repeat_4plex.raw
62750354		High	gPSLNPVLDYDHGSR	Unambiguous	1	1	N-Term(iTRAQ4plex)	HCD	1.0000	0.0000	1	1	9729	Unique	Used	0.738	7.678E3	6.852E3	110	4.99	0.00	0	38	300	2	885.95282	1770.89836	5.18	41.85	4160	4160	MS2	20/28	Glioma_Nuclear_prep_Gradeall_repeat_4plex.raw
62750354		High	gDADQASNILASFGLSAR	Unambiguous	1	1	N-Term(iTRAQ4plex)	HCD	1.0000	0.0000	1	1	12392	Unique	Used	0.773	1.626E2	1.521E2	153	4.83	0.00	0	7	300	3	646.33386	1936.98703	1.27	82.92	7590	7590	MS2	25/68	Glioma_Nuclear_prep_Gradeall_repeat_4plex.raw
62750354	High		tEEGPTLSYGR	Unambiguous	1	1	N-Term(iTRAQ4plex)	HCD	1.0000	0.0000	1	1	8049	Unique	Used	0.593	2.585E5	1.870E5	114	3.26	0.00	0	27	12	2	677.33185	1353.65642	-14.99	26.71	2506	2506	MS2	17/20	Glioma_Nuclear_prep_Gradeall_4plex.raw
62750354	High		gDADQASNILASFGLSAR	Unambiguous	1	1	N-Term(iTRAQ4plex)	HCD	1.0000	0.0000	1	1	12207	Unique	Used	0.611	6.112E4	4.557E4	149	6.31	0.00	0	6	35	2	969.00220	1936.99712	6.48	59.07	7040	7040	MS2	27/34	Glioma_Nuclear_prep_Gradeall_4plex.raw
62750354	High		gPSLNPVLDYDHGSR	Unambiguous	1	1	N-Term(iTRAQ4plex)	HCD	1.0000	0.0000	1	1	9104	Unique	Used	0.623	2.877E4	2.187E4	89	4.64	0.00	0	12	7	3	590.95776	1770.85874	-17.19	34.32	3654	3654	MS2	25/56	Glioma_Nuclear_prep_Gradeall_4plex.raw
62750354	High		gDADQASNILASFGLSAR	Unambiguous	1	1	N-Term(iTRAQ4plex)	HCD	1.0000	0.0000	1	1	12139	Unique	Used	0.660	5.401E4	4.349E4	156	6.49	0.00	0	17	40	2	968.99481	1936.98235	-1.15	58.39	6965	6965	MS2	27/34	Glioma_Nuclear_prep_Gradeall_4plex.raw
62750354	High		gPSLNPVLDYDHGSR	Unambiguous	1	1	N-Term(iTRAQ4plex)	HCD	1.0000	0.0000	1	1	9117	Unique	Used	0.745	1.315E5	1.195E5	92	4.47	0.00	0	11	17	2	885.93506	1770.86284	-14.88	34.42	3668	3668	MS2	22/28	Glioma_Nuclear_prep_Gradeall_4plex.raw
62750354	High		dLSAAGIGLLAAATQSLSMPASLGR	Unambiguous	1	1	N-Term(iTRAQ4plex)	HCD	1.0000	0.0000	1	1	13133	Unique	Used	0.769	7.133E1	6.689E1	75	4.22	0.00	0	17	300	3	839.12964	2515.37436	2.86	76.38	8434	8434	MS2	26/96	Glioma_Nuclear_prep_Gradeall_4plex.raw
62750354		High	lcSLFYTNEEVAk	Unambiguous	3	1	N-Term(iTRAQ4plex); C2(Methylthio); K13(iTRAQ4plex)	HCD	1.0000	0.0000	1	1	12360	Unique	Used	0.714	2.324E3	2.008E3	153	4.83	0.00	0	4	300	2	925.97003	1850.93279	3.07	80.88	7451	7451	MS2	22/24	Glioma_Nuclear_prep_Gradeall_repeat_4plex.raw
62750354	High		lcSLFYTNEEVAk	Unambiguous	3	1	N-Term(iTRAQ4plex); C2(Methylthio); K13(iTRAQ4plex)	HCD	1.0000	0.0000	1	1	11962	Unique	Used	0.528	4.576E4	2.947E4	163	5.69	0.00	0	26	106	2	925.96802	1850.92876	0.89	56.63	6771	6771	MS2	21/24	Glioma_Nuclear_prep_Gradeall_4plex.raw
62750354	High		lcSLFYTNEEVAk	Unambiguous	3	1	N-Term(iTRAQ4plex); C2(Methylthio); K13(iTRAQ4plex)	HCD	1.0000	0.0000	1	1	11880	Unique	Used	0.650	8.423E4	6.676E4	207	5.10	0.00	0	9	38	2	925.97003	1850.93279	3.07	55.89	6681	6681	MS2	21/24	Glioma_Nuclear_prep_Gradeall_4plex.raw
62750354	High		lcSLFYTNEEVAk	Unambiguous	3	1	N-Term(iTRAQ4plex); C2(Methylthio); K13(iTRAQ4plex)	HCD	1.0000	0.0000	1	1	11923	Unique	Used	0.704	1.616E4	1.388E4	165	4.62	0.00	0	22	15	3	617.65088	1850.93808	5.93	56.25	6728	6728	MS2	21/48	Glioma_Nuclear_prep_Gradeall_4plex.raw
62750354	High		nTHcSSLPHYQk	Unambiguous	3	1	N-Term(iTRAQ4plex); C4(Methylthio); K12(iTRAQ4plex)	HCD	1.0000	0.0000	1	1	7725	Unique	Used	0.702	5.466E4	4.680E4	66	3.50	0.00	0	43	3	4	437.96671	1748.84499	-0.05	24.42	2155	2155	MS2	25/66	Glioma_Nuclear_prep_Gradeall_4plex.raw
62750354	High		iTPENLPQILLQLk	Unambiguous	1	1	N-Term(iTRAQ4plex); K14(iTRAQ4plex)	HCD	1.0000	0.0000	1	1	13021	Unique	Used	0.479	3.361E3	1.966E3	28	5.65	0.00	0	8	65	3	636.73273	1908.18363	3.33	70.30	8024	8024	MS2	24/52	Glioma_Nuclear_prep_Gradeall_4plex.raw
62750354	High		iTPENLPQILLQLk	Unambiguous	1	1	N-Term(iTRAQ4plex); K14(iTRAQ4plex)	HCD	1.0000	0.0000	1	1	13024	Unique	Used	0.570	6.607E3	4.597E3	28	5.05	0.00	0	18	300	2	954.59485	1908.18242	2.70	70.38	8030	8030	MS2	22/26	Glioma_Nuclear_prep_Gradeall_4plex.raw
62750354	High		iTPENLPQILLQLk	Unambiguous	1	1	N-Term(iTRAQ4plex); K14(iTRAQ4plex)	HCD	1.0000	0.0000	1	1	12989	Unique	Used	0.594	1.076E4	7.793E3	29	5.56	0.00	0	23	300	2	954.59357	1908.17986	1.36	69.71	7978	7978	MS2	21/26	Glioma_Nuclear_prep_Gradeall_4plex.raw
62750354		High	gAPPSSNIEDFHGLLPk	Unambiguous	1	1	N-Term(iTRAQ4plex); K17(iTRAQ4plex)	HCD	1.0000	0.0000	1	1	10918	Unique	Used	0.720	2.030E3	1.769E3	177	5.56	0.00	0	0	300	2	1034.06226	2067.11724	2.83	56.16	5443	5443	MS2	23/32	Glioma_Nuclear_prep_Gradeall_repeat_4plex.raw
62750354		High	gAPPSSNIEDFHGLLPk	Unambiguous	1	1	N-Term(iTRAQ4plex); K17(iTRAQ4plex)	HCD	1.0000	0.0000	1	1	10954	Unique	Used	0.774	2.821E3	2.641E3	136	4.68	0.00	0	6	119	3	689.71002	2067.11551	2.00	56.59	5482	5482	MS2	28/64	Glioma_Nuclear_prep_Gradeall_repeat_4plex.raw
62750354	High		gAPPSSNIEDFHGLLPk	Unambiguous	1	1	N-Term(iTRAQ4plex); K17(iTRAQ4plex)	HCD	1.0000	0.0000	1	1	9982	Unique	Used	0.694	6.898E4	5.845E4	179	4.63	0.00	0	23	20	2	1034.04333	2067.07939	-15.48	40.72	4610	4610	MS2	24/32	Glioma_Nuclear_prep_Gradeall_4plex.raw
62750354	High		gAPPSSNIEDFHGLLPk	Unambiguous	1	1	N-Term(iTRAQ4plex); K17(iTRAQ4plex)	HCD	1.0000	0.0000	1	1	10052	Unique	Used	0.733	6.404E4	5.731E4	114	4.09	0.00	0	7	4	3	689.69934	2067.08347	-13.51	41.23	4686	4686	MS2	26/64	Glioma_Nuclear_prep_Gradeall_4plex.raw
62750354		High	iGPYQPNVPVGIDYVIPk	Unambiguous	3	1	N-Term(iTRAQ4plex); K18(iTRAQ4plex)	HCD	1.0000	0.0000	1	1	12154	Unique	Used	0.576	2.474E2	1.724E2	106	5.07	0.00	0	26	271	3	753.10120	2257.28904	2.44	75.16	7025	7025	MS2	20/68	Glioma_Nuclear_prep_Gradeall_repeat_4plex.raw
62750354		High	iGPYQPNVPVGIDYVIPk	Unambiguous	3	1	N-Term(iTRAQ4plex); K18(iTRAQ4plex)	HCD	1.0000	0.0000	1	1	12161	Unique	Used	0.644	7.368E2	5.743E2	71	4.17	0.00	0	2	300	2	1129.14612	2257.28496	0.63	75.27	7034	7034	MS2	18/34	Glioma_Nuclear_prep_Gradeall_repeat_4plex.raw
62750354	High		iGPYQPNVPVGIDYVIPk	Unambiguous	3	1	N-Term(iTRAQ4plex); K18(iTRAQ4plex)	HCD	1.0000	0.0000	1	1	11498	Unique	Used	0.638	1.026E4	7.990E3	104	4.44	0.00	0	27	13	3	753.09662	2257.27530	-3.64	52.59	6266	6266	MS2	19/68	Glioma_Nuclear_prep_Gradeall_4plex.raw
62750354	High		iGPYQPNVPVGIDYVIPk	Unambiguous	3	1	N-Term(iTRAQ4plex); K18(iTRAQ4plex)	HCD	1.0000	0.0000	1	1	11508	Unique	Used	0.752	6.943E4	6.370E4	91	5.95	0.00	0	3	17	2	1129.14001	2257.27275	-4.77	52.67	6277	6277	MS2	22/34	Glioma_Nuclear_prep_Gradeall_4plex.raw
63025214	NP_001017980.1	VMA21	203547	vacuolar ATPase assembly integral membrane protein VMA21 [Homo sapiens]	11.88	1	1	2	0.558	1;1	11.1	4.67	101	11.3	7.24																			
63025214		High	aALNALQPPEFR	Unambiguous	1	1	N-Term(iTRAQ4plex)	HCD	1.0000	0.0000	1	1	10837	Unique	Used	0.602	5.212E3	3.796E3	261	4.67	0.00	0	38	300	2	735.91443	1470.82158	2.03	55.15	5354	5354	MS2	17/22	Glioma_Nuclear_prep_Gradeall_repeat_4plex.raw
63025214	High		aALNALQPPEFR	Unambiguous	1	1	N-Term(iTRAQ4plex)	HCD	1.0000	0.0000	1	1	10098	Unique	Used	0.514	1.516E5	9.514E4	275	4.34	0.00	0	32	12	2	735.90161	1470.79595	-15.40	41.57	4736	4736	MS2	17/22	Glioma_Nuclear_prep_Gradeall_4plex.raw
66346730	NP_001015001.1	CKMT1A	548596	creatine kinase U-type, mitochondrial precursor [Homo sapiens]	18.71	2	6	7	0.444	3;4	28.7	25.84	417	47.0	8.34																			
66346730		High	lYPPSAEYPDLR	Unambiguous	1	1	N-Term(iTRAQ4plex)	HCD	1.0000	0.0000	1	1	10599	Unique	Used	0.290	1.135E4	3.990E3	259	3.14	0.00	0	28	300	2	782.91235	1564.81743	2.95	52.21	5095	5095	MS2	15/22	Glioma_Nuclear_prep_Gradeall_repeat_4plex.raw
66346730	High		gTGGVDTAATGGVFDISNLDR	Unambiguous	1	1	N-Term(iTRAQ4plex)	HCD	1.0000	0.0000	1	1	10864	Unique	Used	0.317	4.993E4	1.929E4	139	7.51	0.00	0	12	33	2	1084.02380	2167.04033	-15.94	47.40	5573	5573	MS2	31/40	Glioma_Nuclear_prep_Gradeall_4plex.raw
66346730	High		lGYILTcPSNLGTGLR	Unambiguous	2	1	N-Term(iTRAQ4plex); C7(Methylthio)	HCD	1.0000	0.0000	1	1	12355	Unique	Used	0.326	4.118E4	1.638E4	217	4.74	0.00	0	14	137	2	934.49872	1867.99016	0.56	60.65	7203	7203	MS2	21/30	Glioma_Nuclear_prep_Gradeall_4plex.raw
66346730		High	gLSLPPAcTR	Unambiguous	2	1	N-Term(iTRAQ4plex); C8(Methylthio)	HCD	1.0000	0.0000	1	1	10882	Unique	Used	0.593	6.321E3	4.532E3	254	2.87	0.00	0	54	300	2	602.81946	1204.63164	1.43	55.70	5403	5403	MS2	13/18	Glioma_Nuclear_prep_Gradeall_repeat_4plex.raw
66346730		High	vVVDALSGLk	Unambiguous	1	1	N-Term(iTRAQ4plex); K10(iTRAQ4plex)	HCD	1.0000	0.0000	1	1	11107	Unique	Used	0.468	1.065E4	6.031E3	128	4.30	0.00	0	47	300	2	644.90942	1288.81157	2.84	58.53	5652	5652	MS2	18/18	Glioma_Nuclear_prep_Gradeall_repeat_4plex.raw
66346730	High		vVVDALSGLk	Unambiguous	1	1	N-Term(iTRAQ4plex); K10(iTRAQ4plex)	HCD	1.0000	0.0000	1	1	10306	Unique	Used	0.444	5.423E5	2.936E5	193	3.86	0.00	0	50	8	2	644.90204	1288.79680	-8.62	43.13	4963	4963	MS2	16/18	Glioma_Nuclear_prep_Gradeall_4plex.raw
66346730		High	iPTPVIHTk	Unambiguous	1	1	N-Term(iTRAQ4plex); K9(iTRAQ4plex)	HCD	1.0000	0.0000	1	1	8696	Unique	Used	0.571	1.360E4	9.394E3	88	4.00	0.00	0	60	116	3	431.94684	1293.82596	9.78	30.60	3059	3059	MS2	17/32	Glioma_Nuclear_prep_Gradeall_repeat_4plex.raw
66529396	NP_001018171.1	PON2	5445	serum paraoxonase/arylesterase 2 isoform 2 [Homo sapiens]	14.91	2	4	10	4.699	5;5	33.7	26.20	342	38.0	5.72																			
66529396		High	lFVYDPNNPPSSEVLR	Unambiguous	2	1	N-Term(iTRAQ4plex)	HCD	1.0000	0.0000	1	1	11667	Unique	Used	6.693	6.836E2	5.536E3	165	4.42	0.00	0	5	300	2	996.02203	1991.03679	0.64	66.54	6331	6331	MS2	18/30	Glioma_Nuclear_prep_Gradeall_repeat_4plex.raw
66529396		High	lLIGTLYHR	Unambiguous	2	1	N-Term(iTRAQ4plex)	HCD	1.0000	0.0000	1	1	10463	Unique	Used	5.079	3.547E2	2.179E3	139	3.73	0.00	0	34	300	3	410.58746	1229.74784	-0.69	50.58	4948	4948	MS2	17/32	Glioma_Nuclear_prep_Gradeall_repeat_4plex.raw
66529396		High	lFVYDPNNPPSSEVLR	Unambiguous	2	1	N-Term(iTRAQ4plex)	HCD	1.0000	0.0000	1	1	11599	Unique	Used	4.369	9.547E2	5.046E3	198	4.47	0.00	0	11	300	2	996.02185	1991.03642	0.45	65.55	6248	6248	MS2	21/30	Glioma_Nuclear_prep_Gradeall_repeat_4plex.raw
66529396	High		lFVYDPNNPPSSEVLR	Unambiguous	2	1	N-Term(iTRAQ4plex)	HCD	1.0000	0.0000	1	1	10842	Unique	Used	4.841	2.236E4	1.321E5	140	4.53	0.00	0	9	29	2	996.00720	1991.00713	-14.26	47.23	5549	5549	MS2	21/30	Glioma_Nuclear_prep_Gradeall_4plex.raw
66529396	High		lLIGTLYHR	Unambiguous	2	1	N-Term(iTRAQ4plex)	HCD	1.0000	0.0000	1	1	9636	Unique	Used	4.800	7.050E3	4.129E4	201	3.78	0.00	0	8	13	3	410.58063	1229.72733	-17.37	38.22	4234	4234	MS2	20/32	Glioma_Nuclear_prep_Gradeall_4plex.raw
66529396	High		lFVYDPNNPPSSEVLR	Unambiguous	2	1	N-Term(iTRAQ4plex)	HCD	1.0000	0.0000	1	1	10930	Unique	Used	4.598	2.667E4	1.497E5	139	4.53	0.00	0	2	18	2	996.00604	1991.00481	-15.43	47.92	5645	5645	MS2	20/30	Glioma_Nuclear_prep_Gradeall_4plex.raw
66529396		High	eVESVDLPHcHLIk	Unambiguous	2	1	N-Term(iTRAQ4plex); C10(Methylthio); K14(iTRAQ4plex)	HCD	1.0000	0.0000	1	1	10863	Unique	Used	4.893	1.264E2	7.483E2	210	4.82	0.00	0	17	300	4	489.01044	1953.01992	1.15	55.46	5382	5382	MS2	33/78	Glioma_Nuclear_prep_Gradeall_repeat_4plex.raw
66529396	High		eVESVDLPHcHLIk	Unambiguous	2	1	N-Term(iTRAQ4plex); C10(Methylthio); K14(iTRAQ4plex)	HCD	1.0000	0.0000	1	1	9880	Unique	Used	1.549	3.386E4	6.402E4	171	5.24	0.00	0	26	33	2	976.99512	1952.98296	-17.77	39.98	4499	4499	MS2	24/26	Glioma_Nuclear_prep_Gradeall_4plex.raw
66529396		High	fEEAENSLLHLk	Unambiguous	2	1	N-Term(iTRAQ4plex); K12(iTRAQ4plex)	HCD	1.0000	0.0000	1	1	11397	Unique	Used	4.041	2.751E2	1.345E3	195	4.67	0.00	0	37	300	3	573.31671	1717.93558	-0.45	62.74	6008	6008	MS2	27/44	Glioma_Nuclear_prep_Gradeall_repeat_4plex.raw
66529396	High		fEEAENSLLHLk	Unambiguous	2	1	N-Term(iTRAQ4plex); K12(iTRAQ4plex)	HCD	1.0000	0.0000	1	1	10404	Unique	Used	2.279	1.325E4	3.684E4	241	4.43	0.00	0	22	8	3	573.30786	1717.90903	-15.91	43.87	5070	5070	MS2	26/44	Glioma_Nuclear_prep_Gradeall_4plex.raw
68051721	NP_065843.3	NCEH1	57552	neutral cholesterol ester hydrolase 1 isoform b [Homo sapiens]	9.09	3	3	7	0.528	5;2	24.8	26.34	440	49.0	7.93																			
68051721		High	iVQELPQLLDAR	Unambiguous	3	1	N-Term(iTRAQ4plex)	HCD	1.0000	0.0000	1	1	12148	Unique	Used	0.375	4.070E3	1.846E3	196	3.73	0.00	0	21	300	2	769.95703	1538.90679	2.90	75.08	7018	7018	MS2	18/22	Glioma_Nuclear_prep_Gradeall_repeat_4plex.raw
68051721		High	vTDTDFDGVEVR	Unambiguous	2	1	N-Term(iTRAQ4plex)	HCD	1.0000	0.0000	1	1	9845	Unique	Used	0.712	7.866E3	6.775E3	192	4.39	0.00	0	33	300	2	748.87231	1496.73735	1.58	43.15	4282	4282	MS2	18/22	Glioma_Nuclear_prep_Gradeall_repeat_4plex.raw
68051721	High		iVQELPQLLDAR	Unambiguous	3	1	N-Term(iTRAQ4plex)	HCD	1.0000	0.0000	1	1	11561	Unique	Used	0.452	4.267E4	2.351E4	223	3.76	0.00	0	33	82	2	769.95557	1538.90386	1.00	53.10	6334	6334	MS2	17/22	Glioma_Nuclear_prep_Gradeall_4plex.raw
68051721	High		iVQELPQLLDAR	Unambiguous	3	1	N-Term(iTRAQ4plex)	HCD	1.0000	0.0000	1	1	11473	Unique	Used	0.452	4.849E4	2.676E4	226	3.56	0.00	0	47	94	2	769.95526	1538.90325	0.60	52.37	6238	6238	MS2	18/22	Glioma_Nuclear_prep_Gradeall_4plex.raw
68051721	High		sAPLIADQAVLQLLPk	Unambiguous	3	1	N-Term(iTRAQ4plex); K16(iTRAQ4plex)	HCD	1.0000	0.0000	1	1	12439	Unique	Used	0.528	1.079E4	6.954E3	39	6.73	0.00	0	29	300	2	983.10126	1965.19524	-1.79	61.58	7295	7295	MS2	26/30	Glioma_Nuclear_prep_Gradeall_4plex.raw
68051721	High		sAPLIADQAVLQLLPk	Unambiguous	3	1	N-Term(iTRAQ4plex); K16(iTRAQ4plex)	HCD	1.0000	0.0000	1	1	12470	Unique	Used	0.571	2.071E3	1.443E3	28	6.04	0.00	0	25	145	3	655.73907	1965.20267	2.00	61.95	7330	7330	MS2	27/60	Glioma_Nuclear_prep_Gradeall_4plex.raw
68051721	High		sAPLIADQAVLQLLPk	Unambiguous	3	1	N-Term(iTRAQ4plex); K16(iTRAQ4plex)	HCD	1.0000	0.0000	1	1	12413	Unique	Used	0.732	1.879E3	1.679E3	28	6.26	0.00	0	49	183	3	655.73926	1965.20322	2.28	61.28	7266	7266	MS2	25/60	Glioma_Nuclear_prep_Gradeall_4plex.raw
68509932	NP_001020263.1	MBP	4155	myelin basic protein isoform 4 [Homo sapiens]	33.75	6	5	30	0.404	13;17	32.4	78.27	160	17.3	11.14																			
68509932		High	yLATASTMDHAR	Unambiguous	6	1	N-Term(iTRAQ4plex)	HCD	1.0000	0.0000	1	1	8568	Unique	Used	0.295	4.927E4	1.759E4	196	4.34	0.00	0	19	109	2	740.87335	1480.73943	3.99	29.31	2925	2925	MS2	18/22	Glioma_Nuclear_prep_Gradeall_repeat_4plex.raw
68509932		High	dTGILDSIGR	Unambiguous	6	1	N-Term(iTRAQ4plex)	HCD	1.0000	0.0000	1	1	10053	Unique	Used	0.369	1.013E5	4.525E4	255	3.59	0.00	0	5	67	2	595.83032	1190.65337	3.01	45.73	4511	4511	MS2	17/18	Glioma_Nuclear_prep_Gradeall_repeat_4plex.raw
68509932		High	yLATASTMDHAR	Unambiguous	6	1	N-Term(iTRAQ4plex)	HCD	1.0000	0.0000	1	1	8534	Unique	Used	0.417	1.797E4	9.059E3	115	6.36	0.00	0	42	92	3	494.24536	1480.72153	-8.10	28.96	2889	2889	MS2	23/44	Glioma_Nuclear_prep_Gradeall_repeat_4plex.raw
68509932		High	dTGILDSIGR	Unambiguous	6	1	N-Term(iTRAQ4plex)	HCD	1.0000	0.0000	1	1	9972	Unique	Used	0.460	2.672E4	1.487E4	286	3.70	0.00	0	10	244	2	595.83038	1190.65349	3.11	44.72	4422	4422	MS2	17/18	Glioma_Nuclear_prep_Gradeall_repeat_4plex.raw
68509932		High	yLATASTMDHAR	Unambiguous	6	1	N-Term(iTRAQ4plex)	HCD	1.0000	0.0000	1	1	8222	Unique	Used	0.730	5.407E3	4.774E3	143	4.95	0.00	0	37	130	3	494.25058	1480.73719	2.47	25.95	2560	2560	MS2	21/44	Glioma_Nuclear_prep_Gradeall_repeat_4plex.raw
68509932	High		yLATASTMDHAR	Unambiguous	6	1	N-Term(iTRAQ4plex)	HCD	1.0000	0.0000	1	1	8180	Unique	Used	0.320	1.649E5	6.429E4	93	5.92	0.00	0	24	4	3	494.24185	1480.71100	-15.21	27.64	2648	2648	MS2	22/44	Glioma_Nuclear_prep_Gradeall_4plex.raw
68509932	High		dTGILDSIGR	Unambiguous	6	1	N-Term(iTRAQ4plex)	HCD	1.0000	0.0000	1	1	9578	Unique	Used	0.387	1.103E6	5.206E5	185	3.07	0.00	0	10	2	2	595.81958	1190.63188	-15.03	37.82	4172	4172	MS2	17/18	Glioma_Nuclear_prep_Gradeall_4plex.raw
68509932	High		dTGILDSIGR	Unambiguous	6	1	N-Term(iTRAQ4plex)	HCD	1.0000	0.0000	1	1	9489	Unique	Used	0.493	2.808E5	1.691E5	220	3.25	0.00	0	45	10	2	595.82001	1190.63274	-14.32	37.17	4075	4075	MS2	15/18	Glioma_Nuclear_prep_Gradeall_4plex.raw
68509932		High	tAHYGSLPQk	Unambiguous	5	1	N-Term(iTRAQ4plex); K10(iTRAQ4plex)	HCD	1.0000	0.0000	1	1	7617	Unique	Used	0.391	1.129E5	5.342E4	224	3.74	0.00	0	35	39	2	695.39362	1389.77995	5.09	20.10	1919	1919	MS2	17/18	Glioma_Nuclear_prep_Gradeall_repeat_4plex.raw
68509932		High	tAHYGSLPQk	Unambiguous	5	1	N-Term(iTRAQ4plex); K10(iTRAQ4plex)	HCD	1.0000	0.0000	1	1	7673	Unique	Used	0.437	9.796E3	5.182E3	232	3.91	0.00	0	36	86	3	463.92545	1389.76178	-7.99	20.67	1982	1982	MS2	18/36	Glioma_Nuclear_prep_Gradeall_repeat_4plex.raw
68509932		High	gVDAQGTLSk	Unambiguous	5	1	N-Term(iTRAQ4plex); K10(iTRAQ4plex)	HCD	1.0000	0.0000	1	1	7568	Unique	Used	0.513	1.320E5	8.189E4	255	3.65	0.00	0	10	28	2	632.35974	1263.71221	-2.00	19.68	1867	1867	MS2	18/18	Glioma_Nuclear_prep_Gradeall_repeat_4plex.raw
68509932		High	tAHYGSLPQk	Unambiguous	5	1	N-Term(iTRAQ4plex); K10(iTRAQ4plex)	HCD	1.0000	0.0000	1	1	7584	Unique	Used	0.553	1.686E4	1.128E4	256	4.06	0.00	0	44	92	3	463.92877	1389.77176	-0.81	19.79	1883	1883	MS2	18/36	Glioma_Nuclear_prep_Gradeall_repeat_4plex.raw
68509932		High	gVDAQGTLSk	Unambiguous	5	1	N-Term(iTRAQ4plex); K10(iTRAQ4plex)	HCD	1.0000	0.0000	1	1	7694	Unique	Used	0.694	3.445E4	2.892E4	279	3.76	0.00	0	17	118	2	632.35486	1263.70244	-9.72	20.88	2004	2004	MS2	18/18	Glioma_Nuclear_prep_Gradeall_4plex.raw

68509932	High		tAHYGSLPQk	Unambiguous	5	1	N-Term(iTRAQ4plex); K10(iTRAQ4plex)	HCD	1.0000	0.0000	1	1	7516	Unique	Used	0.322	5.515E5	2.168E5	265	4.15	0.00	0	23	12	2	695.38824	1389.76921	-2.64	22.92	1928	1928	MS2	17/18	Glioma_Nuclear_prep_Gradeall_4plex.raw
68509932	High		gVDAQGTLSk	Unambiguous	5	1	N-Term(iTRAQ4plex); K10(iTRAQ4plex)	HCD	1.0000	0.0000	1	1	7535	Unique	Used	0.475	6.797E5	3.939E5	273	4.06	0.00	0	27	7	2	632.36218	1263.71709	1.87	23.07	1949	1949	MS2	18/18	Glioma_Nuclear_prep_Gradeall_4plex.raw
68509932	High		tAHYGSLPQk	Unambiguous	5	1	N-Term(iTRAQ4plex); K10(iTRAQ4plex)	HCD	1.0000	0.0000	1	1	7499	Unique	Used	0.619	5.979E4	4.515E4	296	3.58	0.00	0	49	8	3	463.92868	1389.77149	-1.01	22.81	1910	1910	MS2	18/36	Glioma_Nuclear_prep_Gradeall_4plex.raw
68509932		High	tQDENPVVHFFk	Unambiguous	5	1	N-Term(iTRAQ4plex); K12(iTRAQ4plex)	HCD	1.0000	0.0000	1	1	10574	Unique	Used	0.262	1.023E3	3.240E2	170	3.65	0.00	0	27	300	4	437.98669	1748.92495	2.24	51.92	5068	5068	MS2	20/66	Glioma_Nuclear_prep_Gradeall_repeat_4plex.raw
68509932		High	tQDENPVVHFFk	Unambiguous	5	1	N-Term(iTRAQ4plex); K12(iTRAQ4plex)	HCD	1.0000	0.0000	1	1	10591	Unique	Used	0.281	3.971E4	1.352E4	123	4.12	0.00	0	8	23	3	583.64740	1748.92765	3.78	52.13	5087	5087	MS2	26/44	Glioma_Nuclear_prep_Gradeall_repeat_4plex.raw
68509932		High	tQDENPVVHFFk	Unambiguous	5	1	N-Term(iTRAQ4plex); K12(iTRAQ4plex)	HCD	1.0000	0.0000	1	1	10592	Unique	Used	0.286	3.918E4	1.356E4	204	5.38	0.00	0	8	115	2	874.96832	1748.92937	4.76	52.14	5088	5088	MS2	21/22	Glioma_Nuclear_prep_Gradeall_repeat_4plex.raw
68509932		High	tQDENPVVHFFk	Unambiguous	5	1	N-Term(iTRAQ4plex); K12(iTRAQ4plex)	HCD	1.0000	0.0000	1	1	10515	Unique	Used	0.421	7.042E3	3.584E3	206	5.28	0.00	0	29	300	2	874.96686	1748.92644	3.09	51.22	5004	5004	MS2	21/22	Glioma_Nuclear_prep_Gradeall_repeat_4plex.raw
68509932		High	tQDENPVVHFFk	Unambiguous	5	1	N-Term(iTRAQ4plex); K12(iTRAQ4plex)	HCD	1.0000	0.0000	1	1	10505	Unique	Used	0.447	2.800E3	1.513E3	139	4.13	0.00	0	35	300	3	583.64563	1748.92234	0.74	51.11	4994	4994	MS2	25/44	Glioma_Nuclear_prep_Gradeall_repeat_4plex.raw
68509932		High	tQDENPVVHFFk	Unambiguous	5	1	N-Term(iTRAQ4plex); K12(iTRAQ4plex)	HCD	1.0000	0.0000	1	1	10686	Unique	Used	0.662	3.147E3	2.518E3	137	4.09	0.00	0	22	272	3	583.64502	1748.92051	-0.30	53.27	5189	5189	MS2	25/44	Glioma_Nuclear_prep_Gradeall_repeat_4plex.raw
68509932	High		tQDENPVVHFFk	Unambiguous	5	1	N-Term(iTRAQ4plex); K12(iTRAQ4plex)	HCD	1.0000	0.0000	1	1	9721	Unique	Used	0.294	8.362E5	2.995E5	147	4.66	0.00	0	8	1	3	583.63715	1748.89688	-13.81	38.84	4327	4327	MS2	27/44	Glioma_Nuclear_prep_Gradeall_4plex.raw
68509932	High		tQDENPVVHFFk	Unambiguous	5	1	N-Term(iTRAQ4plex); K12(iTRAQ4plex)	HCD	1.0000	0.0000	1	1	9745	Unique	Used	0.303	5.883E5	2.176E5	176	5.16	0.00	0	6	5	2	874.95319	1748.89910	-12.55	39.01	4353	4353	MS2	21/22	Glioma_Nuclear_prep_Gradeall_4plex.raw
68509932	High		tQDENPVVHFFk	Unambiguous	5	1	N-Term(iTRAQ4plex); K12(iTRAQ4plex)	HCD	1.0000	0.0000	1	1	9632	Unique	Used	0.354	4.076E4	1.761E4	126	4.92	0.00	0	43	9	3	583.63696	1748.89634	-14.12	38.19	4230	4230	MS2	23/44	Glioma_Nuclear_prep_Gradeall_4plex.raw
68509932	High		tQDENPVVHFFk	Unambiguous	5	1	N-Term(iTRAQ4plex); K12(iTRAQ4plex)	HCD	1.0000	0.0000	1	1	9653	Unique	Used	0.366	2.330E5	1.042E5	179	5.33	0.00	0	13	14	2	874.95319	1748.89910	-12.55	38.35	4253	4253	MS2	22/22	Glioma_Nuclear_prep_Gradeall_4plex.raw
68509932	High		tQDENPVVHFFk	Unambiguous	5	1	N-Term(iTRAQ4plex); K12(iTRAQ4plex)	HCD	1.0000	0.0000	1	1	9822	Unique	Used	0.759	3.997E4	3.699E4	111	4.44	0.00	0	42	5	3	583.63727	1748.89725	-13.60	39.56	4436	4436	MS2	24/44	Glioma_Nuclear_prep_Gradeall_4plex.raw
68509932		High	yLATASTmDHAR	Unambiguous	6	1	N-Term(iTRAQ4plex); M8(Oxidation)	HCD	1.0000	0.0000	1	1	7571	Unique	Used	0.464	3.503E4	1.966E4	118	6.36	0.00	0	20	28	3	499.57956	1496.72412	-2.88	19.70	1870	1870	MS2	23/44	Glioma_Nuclear_prep_Gradeall_repeat_4plex.raw
68509932	High		yLATASTmDHAR	Unambiguous	6	1	N-Term(iTRAQ4plex); M8(Oxidation)	HCD	1.0000	0.0000	1	1	7510	Unique	Used	0.289	1.515E5	5.349E4	114	6.01	0.00	0	33	5	3	499.57874	1496.72165	-4.53	22.89	1922	1922	MS2	23/44	Glioma_Nuclear_prep_Gradeall_4plex.raw
68509932	High		yLATASTmDHAR	Unambiguous	6	1	N-Term(iTRAQ4plex); M8(Oxidation)	HCD	1.0000	0.0000	1	1	7520	Unique	Used	0.377	3.547E5	1.629E5	181	2.91	0.00	0	20	9	2	748.86938	1496.73149	2.04	22.96	1933	1933	MS2	13/22	Glioma_Nuclear_prep_Gradeall_4plex.raw
70167113	NP_001020278.1	ADAR	103	double-stranded RNA-specific adenosine deaminase isoform d [Homo sapiens]	17.29	4	5	12	0.560	8;4	12.2	71.76	931	103.6	8.66																			
70167113		High	yLNTNPVGGLLEYAR	Unambiguous	4	1	N-Term(iTRAQ4plex)	HCD	1.0000	0.0000	1	1	12293	Unique	Used	0.499	2.782E3	1.680E3	242	4.22	0.00	0	10	300	2	912.49359	1823.97991	1.45	78.85	7302	7302	MS2	19/28	Glioma_Nuclear_prep_Gradeall_repeat_4plex.raw
70167113		High	dINAVLIDMER	Unambiguous	4	1	N-Term(iTRAQ4plex)	HCD	1.0000	0.0000	1	1	11835	Unique	Used	0.615	3.302E3	2.457E3	289	3.75	0.00	0	34	300	2	716.88452	1432.76177	2.14	69.19	6551	6551	MS2	17/20	Glioma_Nuclear_prep_Gradeall_repeat_4plex.raw
70167113	High		dINAVLIDMER	Unambiguous	4	1	N-Term(iTRAQ4plex)	HCD	1.0000	0.0000	1	1	11121	Unique	Used	0.503	1.557E5	9.558E4	264	4.44	0.00	0	40	22	2	716.88391	1432.76055	1.29	49.40	5853	5853	MS2	16/20	Glioma_Nuclear_prep_Gradeall_4plex.raw
70167113	High		yLNTNPVGGLLEYAR	Unambiguous	4	1	N-Term(iTRAQ4plex)	HCD	1.0000	0.0000	1	1	11796	Unique	Used	0.521	3.810E5	2.422E5	294	4.90	0.00	0	14	11	2	912.49103	1823.97478	-1.36	55.17	6590	6590	MS2	20/28	Glioma_Nuclear_prep_Gradeall_4plex.raw
70167113	High		sPVTTLLEcMHk	Unambiguous	4	1	N-Term(iTRAQ4plex); C9(Methylthio); K12(iTRAQ4plex)	HCD	1.0000	0.0000	1	1	11933	Unique	Used	0.609	5.632E4	4.184E4	162	7.01	0.00	0	10	14	3	564.96387	1692.87705	2.64	56.35	6739	6739	MS2	34/44	Glioma_Nuclear_prep_Gradeall_4plex.raw
70167113	High		sPVTTLLEcMHk	Unambiguous	4	1	N-Term(iTRAQ4plex); C9(Methylthio); K12(iTRAQ4plex)	HCD	1.0000	0.0000	1	1	11858	Unique	Used	0.614	1.636E4	1.226E4	156	6.19	0.00	0	41	55	3	564.96161	1692.87027	-1.36	55.70	6657	6657	MS2	34/44	Glioma_Nuclear_prep_Gradeall_4plex.raw
70167113		High	lVDQSGPPHEPk	Unambiguous	4	1	N-Term(iTRAQ4plex); K12(iTRAQ4plex)	HCD	1.0000	0.0000	1	1	7692	Unique	Used	0.560	1.784E4	1.208E4	188	4.06	0.00	0	16	58	3	531.29028	1591.85630	-7.53	20.87	2002	2002	MS2	24/44	Glioma_Nuclear_prep_Gradeall_repeat_4plex.raw
70167113	High		lVDQSGPPHEPk	Unambiguous	4	1	N-Term(iTRAQ4plex); K12(iTRAQ4plex)	HCD	1.0000	0.0000	1	1	7633	Unique	Used	0.561	1.043E5	7.139E4	239	4.76	0.00	0	6	18	2	796.43866	1591.87004	1.11	23.77	2055	2055	MS2	20/22	Glioma_Nuclear_prep_Gradeall_4plex.raw
70167113	High		lVDQSGPPHEPk	Unambiguous	4	1	N-Term(iTRAQ4plex); K12(iTRAQ4plex)	HCD	1.0000	0.0000	1	1	7608	Unique	Used	0.683	7.913E4	6.595E4	213	4.20	0.00	0	35	8	3	531.29547	1591.87186	2.25	23.59	2028	2028	MS2	22/44	Glioma_Nuclear_prep_Gradeall_4plex.raw
70167113	High		tAESQTPTPSATSFFSGk	Unambiguous	4	1	N-Term(iTRAQ4plex); K18(iTRAQ4plex)	HCD	1.0000	0.0000	1	1	9667	Unique	Used	0.725	7.980E4	7.054E4	128	6.40	0.00	0	0	18	2	1066.52258	2132.03789	-17.43	38.45	4268	4268	MS2	22/34	Glioma_Nuclear_prep_Gradeall_4plex.raw
70167113		High	dINAVLIDmER	Unambiguous	4	1	N-Term(iTRAQ4plex); M9(Oxidation)	HCD	1.0000	0.0000	1	1	10126	Unique	Used	0.548	1.006E4	6.678E3	229	4.01	0.00	0	18	300	2	724.88239	1448.75749	2.67	46.60	4590	4590	MS2	18/20	Glioma_Nuclear_prep_Gradeall_repeat_4plex.raw
70167113	High		dINAVLIDmER	Unambiguous	4	1	N-Term(iTRAQ4plex); M9(Oxidation)	HCD	1.0000	0.0000	1	1	9597	Unique	Used	0.531	1.612E5	1.045E5	183	3.20	0.00	0	23	14	2	724.86945	1448.73161	-15.19	37.94	4192	4192	MS2	17/20	Glioma_Nuclear_prep_Gradeall_4plex.raw
89145417	NP_054752.3	METTL7A	25840	methyltransferase-like protein 7A precursor [Homo sapiens]	11.07	1	1	2	1.764	1;1	14.1	9.23	244	28.3	8.38																			
89145417		High	vTcIDPNPNFEk	Unambiguous	1	1	N-Term(iTRAQ4plex); C3(Methylthio); K12(iTRAQ4plex)	HCD	1.0000	0.0000	1	1	10569	Unique	Used	1.940	2.213E3	5.193E3	128	4.45	0.00	0	48	300	2	855.92700	1710.84673	1.95	51.85	5062	5062	MS2	17/22	Glioma_Nuclear_prep_Gradeall_repeat_4plex.raw
89145417	High		vTcIDPNPNFEk	Unambiguous	1	1	N-Term(iTRAQ4plex); C3(Methylthio); K12(iTRAQ4plex)	HCD	1.0000	0.0000	1	1	9854	Unique	Used	1.588	4.660E4	9.029E4	85	4.61	0.00	0	11	25	2	855.91235	1710.81743	-15.17	39.79	4471	4471	MS2	17/22	Glioma_Nuclear_prep_Gradeall_4plex.raw
93004092	NP_005587.2	NFIB	4781	nuclear factor 1 B-type isoform 3 [Homo sapiens]	10.48	12	3	4	1.823	1;3	9.6	14.26	420	47.4	8.87																			
93004092		High	sGVFNVSELVR	Unambiguous	3	1	N-Term(iTRAQ4plex)	HCD	1.0000	0.0000	1	1	11422	Unique	Used	1.708	3.554E3	7.343E3	369	3.86	0.00	0	39	300	2	675.87885	1350.75041	0.43	63.11	6039	6039	MS2	18/20	Glioma_Nuclear_prep_Gradeall_repeat_4plex.raw
93004092	High		sGVFNVSELVR	Unambiguous	3	1	N-Term(iTRAQ4plex)	HCD	1.0000	0.0000	1	1	10536	Unique	Used	1.678	1.011E5	2.069E5	342	4.01	0.00	0	31	20	2	675.86859	1350.72991	-14.75	44.92	5216	5216	MS2	19/20	Glioma_Nuclear_prep_Gradeall_4plex.raw
93004092		High	eDFVLTVTGk	Unambiguous	7	1	N-Term(iTRAQ4plex); K10(iTRAQ4plex)	HCD	1.0000	0.0000	1	1	10757	Unique	Used	1.939	3.534E3	8.289E3	278	4.02	0.00	0	44	300	2	698.90155	1396.79582	2.27	54.12	5265	5265	MS2	15/18	Glioma_Nuclear_prep_Gradeall_repeat_4plex.raw
93004092		High	nPPGYLEDSFVk	Unambiguous	3	1	N-Term(iTRAQ4plex); K12(iTRAQ4plex)	HCD	1.0000	0.0000	1	1	10487	Unique	Used	2.039	2.013E3	4.966E3	240	3.09	0.00	0	46	300	2	827.43994	1653.87261	-0.05	50.87	4974	4974	MS2	12/22	Glioma_Nuclear_prep_Gradeall_repeat_4plex.raw
93102371	NP_060783.3	PBRM1	55193	protein polybromo-1 [Homo sapiens]	1.20	1	2	3	1.743	2;1	18.8	7.94	1582	181.0	6.87																			
93102371		High	vQYPDYYAIIk	Unambiguous	1	1	N-Term(iTRAQ4plex); K11(iTRAQ4plex)	HCD	1.0000	0.0000	1	1	11472	Unique	Used	2.107	1.411E3	3.596E3	270	4.04	0.00	0	25	300	2	830.96423	1660.92119	1.39	63.78	6097	6097	MS2	18/20	Glioma_Nuclear_prep_Gradeall_repeat_4plex.raw
93102371	High		vQYPDYYAIIk	Unambiguous	1	1	N-Term(iTRAQ4plex); K11(iTRAQ4plex)	HCD	1.0000	0.0000	1	1	10621	Unique	Used	1.743	3.896E4	8.282E4	272	4.64	0.00	0	27	45	2	830.95056	1660.89385	-15.07	45.54	5308	5308	MS2	18/20	Glioma_Nuclear_prep_Gradeall_4plex.raw
93102371	High		lISELFQk	Unambiguous	1	1	N-Term(iTRAQ4plex); K8(iTRAQ4plex)	HCD	1.0000	0.0000	1	1	10993	Unique	Used	1.446	5.406E4	9.539E4	260	3.30	0.00	0	63	42	2	633.38019	1265.75310	-13.97	48.40	5713	5713	MS2	13/14	Glioma_Nuclear_prep_Gradeall_4plex.raw
109240550	NP_001035879.1	PSPC1	55269	paraspeckle component 1 [Homo sapiens]	20.65	1	8	14	0.315	7;7	40.1	38.59	523	58.7	6.67																			
109240550	High		nLSPVVSNELLEQAFSQFGPVEk	Unambiguous	1	1	N-Term(iTRAQ4plex); K23(iTRAQ4plex)	HCD	1.0000	0.0000	1	1	12958	Unique	Used	0.498	1.029E3	6.251E2	109	9.06	0.00	0	13	235	3	940.84186	2820.51102	3.10	68.97	7924	7924	MS2	34/88	Glioma_Nuclear_prep_Gradeall_4plex.raw
109240550		High	fAQPGTFEFEYASR	Unambiguous	1	1	N-Term(iTRAQ4plex)	HCD	1.0000	0.0000	1	1	11306	Unique	Used	0.248	7.395E3	2.221E3	134	4.26	0.00	0	12	300	2	897.43707	1793.86687	2.95	61.38	5894	5894	MS2	20/26	Glioma_Nuclear_prep_Gradeall_repeat_4plex.raw
109240550		High	yGEPSEVFINR	Unambiguous	1	1	N-Term(iTRAQ4plex)	HCD	1.0000	0.0000	1	1	10006	Unique	Used	0.283	2.175E4	7.447E3	220	3.67	0.00	0	41	300	2	727.87573	1454.74419	3.12	45.12	4458	4458	MS2	15/20	Glioma_Nuclear_prep_Gradeall_repeat_4plex.raw
109240550	High		fAQPGTFEFEYASR	Unambiguous	1	1	N-Term(iTRAQ4plex)	HCD	1.0000	0.0000	1	1	10496	Unique	Used	0.281	2.908E5	9.966E4	86	4.34	0.00	0	28	27	2	897.42389	1793.84050	-11.75	44.61	5172	5172	MS2	17/26	Glioma_Nuclear_prep_Gradeall_4plex.raw
109240550		High	fATHGAALTVk	Unambiguous	1	1	N-Term(iTRAQ4plex); K11(iTRAQ4plex)	HCD	1.0000	0.0000	1	1	8429	Unique	Used	0.413	1.898E4	9.490E3	264	3.63	0.00	0	53	300	2	702.41937	1403.83147	4.65	27.91	2777	2777	MS2	18/20	Glioma_Nuclear_prep_Gradeall_repeat_4plex.raw
109240550	High		fATHGAALTVk	Unambiguous	1	1	N-Term(iTRAQ4plex); K11(iTRAQ4plex)	HCD	1.0000	0.0000	1	1	8055	Unique	Used	0.324	2.097E5	8.285E4	235	4.02	0.00	0	10	23	2	702.40753	1403.80779	-12.22	26.74	2512	2512	MS2	20/20	Glioma_Nuclear_prep_Gradeall_4plex.raw
109240550		High	lFVGNLPTDITEEDFkR	Unambiguous	1	1	N-Term(iTRAQ4plex); K16(iTRAQ4plex)	HCD	1.0000	0.0000	1	1	12030	Unique	Used	0.268	1.603E3	5.194E2	162	4.55	0.00	1	26	300	3	761.41620	2282.23404	3.02	72.55	6822	6822	MS2	27/64	Glioma_Nuclear_prep_Gradeall_repeat_4plex.raw
109240550		High	lFVGNLPTDITEEDFk	Unambiguous	1	1	N-Term(iTRAQ4plex); K16(iTRAQ4plex)	HCD	1.0000	0.0000	1	1	12356	Unique	Used	0.575	2.917E2	2.031E2	173	4.54	0.00	0	17	300	3	709.38214	2126.13187	2.74	80.79	7444	7444	MS2	25/60	Glioma_Nuclear_prep_Gradeall_repeat_4plex.raw
109240550	High		lFVGNLPTDITEEDFk	Unambiguous	1	1	N-Term(iTRAQ4plex); K16(iTRAQ4plex)	HCD	1.0000	0.0000	1	1	11992	Unique	Used	0.232	7.421E4	2.102E4	218	6.23	0.00	0	11	67	2	1063.56628	2126.12529	-0.35	56.91	6804	6804	MS2	22/30	Glioma_Nuclear_prep_Gradeall_4plex.raw
109240550	High		lFVGNLPTDITEEDFk	Unambiguous	1	1	N-Term(iTRAQ4plex); K16(iTRAQ4plex)	HCD	1.0000	0.0000	1	1	11981	Unique	Used	0.422	1.169E4	6.024E3	172	5.57	0.00	0	19	21	3	709.38043	2126.12674	0.33	56.81	6792	6792	MS2	23/60	Glioma_Nuclear_prep_Gradeall_4plex.raw
109240550	High		nLSPVVSNELLEQAFSQFGPVEk	Unambiguous	1	1	N-Term(iTRAQ4plex); K23(iTRAQ4plex)	HCD	1.0000	0.0000	1	1	12959	Unique	Used	0.769	7.133E1	6.689E1	101	6.27	0.00	0	26	300	4	705.88501	2820.51821	5.65	69.00	7926	7926	MS2	30/132	Glioma_Nuclear_prep_Gradeall_4plex.raw
109240550		High	gFVEFAAkPPAR	Unambiguous	1	1	N-Term(iTRAQ4plex); K8(iTRAQ4plex)	HCD	1.0000	0.0000	1	1	9360	Unique	Used	0.334	1.371E4	5.531E3	166	3.77	0.00	0	25	104	3	526.64221	1577.91208	4.94	37.74	3763	3763	MS2	25/44	Glioma_Nuclear_prep_Gradeall_repeat_4plex.raw
109240550		High	aELDGTILk	Unambiguous	1	1	N-Term(iTRAQ4plex); K9(iTRAQ4plex)	HCD	1.0000	0.0000	1	1	9383	Unique	Used	0.307	4.272E4	1.586E4	258	3.70	0.00	0	32	207	2	624.37762	1247.74797	2.41	37.97	3787	3787	MS2	16/16	Glioma_Nuclear_prep_Gradeall_repeat_4plex.raw
109240550	High		aELDGTILk	Unambiguous	1	1	N-Term(iTRAQ4plex); K9(iTRAQ4plex)	HCD	1.0000	0.0000	1	1	8908	Unique	Used	0.298	4.397E5	1.599E5	266	3.10	0.00	0	37	10	2	624.36835	1247.72942	-12.46	32.87	3439	3439	MS2	16/16	Glioma_Nuclear_prep_Gradeall_4plex.raw
109689718	NP_001035937.1	ABHD12	26090	monoacylglycerol lipase ABHD12 isoform a [Homo sapiens]	9.55	2	2	3	0.622	1;2	24.8	13.25	398	45.1	8.65																			
109689718		High	eTPPDALILESPFTNIR	Unambiguous	2	1	N-Term(iTRAQ4plex)	HCD	1.0000	0.0000	1	1	12417	Unique	Used	0.425	1.178E2	6.056E1	133	3.90	0.00	0	16	300	3	686.37372	2057.10660	1.45	84.63	7706	7706	MS2	18/64	Glioma_Nuclear_prep_Gradeall_repeat_4plex.raw
109689718		High	vQFVPFHSDLGYR	Unambiguous	2	1	N-Term(iTRAQ4plex)	HCD	1.0000	0.0000	1	1	11073	Unique	Used	0.622	1.496E3	1.126E3	206	3.96	0.00	0	32	300	3	570.30334	1708.89548	1.57	58.09	5614	5614	MS2	21/48	Glioma_Nuclear_prep_Gradeall_repeat_4plex.raw
109689718	High		eTPPDALILESPFTNIR	Unambiguous	2	1	N-Term(iTRAQ4plex)	HCD	1.0000	0.0000	1	1	12214	Unique	Used	0.709	1.594E4	1.379E4	246	5.44	0.00	0	48	300	2	1029.06226	2057.11724	6.62	59.13	7047	7047	MS2	18/32	Glioma_Nuclear_prep_Gradeall_4plex.raw
119395750	NP_006112.3	KRT1	3848	keratin, type II cytoskeletal 1 [Homo sapiens]	8.54	2	4	6	2.249	3;3	23.9	20.68	644	66.0	8.12																			
119395750		High	yEELQITAGR	Unambiguous	2	1	N-Term(iTRAQ4plex)	HCD	1.0000	0.0000	1	1	9492	Unique	Used	1.975	5.742E3	1.372E4	301	3.25	0.00	0	53	300	2	662.35535	1323.70342	0.67	39.16	3903	3903	MS2	15/18	Glioma_Nuclear_prep_Gradeall_repeat_4plex.raw
119395750		High	sLNNQFASFIDk	Unambiguous	1	1	N-Term(iTRAQ4plex); K12(iTRAQ4plex)	HCD	1.0000	0.0000	1	1	11469	Unique	Used	2.112	1.531E3	3.912E3	259	4.91	0.00	0	39	300	2	836.45258	1671.89787	2.03	63.75	6094	6094	MS2	21/22	Glioma_Nuclear_prep_Gradeall_repeat_4plex.raw
119395750	High		sLDLDSIIAEVk	Unambiguous	1	1	N-Term(iTRAQ4plex); K12(iTRAQ4plex)	HCD	1.0000	0.0000	1	1	12291	Unique	Used	3.330	9.011E2	3.661E3	221	4.34	0.00	0	24	135	3	530.97754	1590.91806	-0.79	59.94	7132	7132	MS2	20/44	Glioma_Nuclear_prep_Gradeall_4plex.raw
119395750	High		sLDLDSIIAEVk	Unambiguous	1	1	N-Term(iTRAQ4plex); K12(iTRAQ4plex)	HCD	1.0000	0.0000	1	1	12297	Unique	Used	3.002	1.398E4	5.121E4	212	5.44	0.00	0	30	115	2	795.96283	1590.91838	-0.59	60.01	7139	7139	MS2	22/22	Glioma_Nuclear_prep_Gradeall_4plex.raw
119395750	High		sLNNQFASFIDk	Unambiguous	1	1	N-Term(iTRAQ4plex); K12(iTRAQ4plex)	HCD	1.0000	0.0000	1	1	10582	Unique	Used	2.386	6.582E4	1.916E5	189	5.21	0.00	0	18	18	2	836.43958	1671.87187	-13.53	45.26	5266	5266	MS2	22/22	Glioma_Nuclear_prep_Gradeall_4plex.raw
119395750		High	aQYEDIAQk	Unambiguous	1	1	N-Term(iTRAQ4plex); K9(iTRAQ4plex)	HCD	1.0000	0.0000	1	1	8188	Unique	Used	1.906	1.006E4	2.319E4	231	3.88	0.00	0	22	249	2	677.36493	1353.72258	-1.99	25.60	2524	2524	MS2	16/16	Glioma_Nuclear_prep_Gradeall_repeat_4plex.raw
125656165	NP_001075019.1	THOC2	57187	THO complex subunit 2 [Homo sapiens]	3.58	1	4	5	1.596	3;2	17.2	13.23	1593	182.7	8.44																			
125656165		High	vLNLGQALER	Unambiguous	1	1	N-Term(iTRAQ4plex)	HCD	1.0000	0.0000	1	1	10796	Unique	Used	1.296	3.197E3	5.014E3	239	2.94	0.00	0	58	300	2	628.87592	1256.74455	0.16	54.61	5308	5308	MS2	15/18	Glioma_Nuclear_prep_Gradeall_repeat_4plex.raw
125656165	High		lDPETLESLGLIk	Unambiguous	1	1	N-Term(iTRAQ4plex); K13(iTRAQ4plex)	HCD	1.0000	0.0000	1	1	12091	Unique	Used	1.596	8.896E3	1.733E4	149	5.13	0.00	0	39	300	2	858.50769	1716.00810	2.75	57.92	6912	6912	MS2	22/24	Glioma_Nuclear_prep_Gradeall_4plex.raw
125656165	High		lDPETLESLGLIk	Unambiguous	1	1	N-Term(iTRAQ4plex); K13(iTRAQ4plex)	HCD	1.0000	0.0000	1	1	12086	Unique	Used	1.288	1.422E3	2.234E3	162	4.35	0.00	0	26	132	3	572.67285	1716.00400	0.36	57.87	6907	6907	MS2	21/48	Glioma_Nuclear_prep_Gradeall_4plex.raw
125656165		High	sFDLLILk	Unambiguous	1	1	N-Term(iTRAQ4plex); K8(iTRAQ4plex)	HCD	1.0000	0.0000	1	1	12167	Unique	Used	1.851	2.026E3	4.537E3	129	3.23	0.00	0	45	300	2	618.89301	1236.77873	-1.52	75.40	7044	7044	MS2	14/14	Glioma_Nuclear_prep_Gradeall_repeat_4plex.raw
125656165	High		lGLLEALLk	Unambiguous	1	1	N-Term(iTRAQ4plex); K9(iTRAQ4plex)	HCD	1.0000	0.0000	1	1	12931	Unique	Used	1.804	4.312E3	9.492E3	41	3.75	0.00	0	46	300	2	629.42255	1257.83782	-0.53	68.51	7887	7887	MS2	16/16	Glioma_Nuclear_prep_Gradeall_4plex.raw
148596949	NP_004732.2	NOLC1	9221	nucleolar and coiled-body phosphoprotein 1 isoform 2 [Homo sapiens]	9.16	3	4	5	0.651	2;3	8.4	20.27	699	73.6	9.47																			
148596949	High		vVPSDLYPLVLGFLR	Unambiguous	3	1	N-Term(iTRAQ4plex)	HCD	1.0000	0.0000	1	1	13146	Unique	Used	0.651	2.229E3	1.771E3	53	3.37	0.00	0	15	300	2	916.54718	1832.08708	3.71	78.34	8561	8561	MS2	16/28	Glioma_Nuclear_prep_Gradeall_4plex.raw
148596949		High	dNQLSEVANk	Unambiguous	3	1	N-Term(iTRAQ4plex); K10(iTRAQ4plex)	HCD	1.0000	0.0000	1	1	8116	Unique	Used	0.600	2.329E4	1.690E4	215	4.91	0.00	0	28	165	2	703.37488	1405.74248	-7.18	24.90	2448	2448	MS2	18/18	Glioma_Nuclear_prep_Gradeall_repeat_4plex.raw
148596949	High		dNQLSEVANk	Unambiguous	3	1	N-Term(iTRAQ4plex); K10(iTRAQ4plex)	HCD	1.0000	0.0000	1	1	7938	Unique	Used	0.664	2.625E5	2.127E5	215	4.60	0.00	0	18	8	2	703.38092	1405.75456	1.42	25.93	2386	2386	MS2	17/18	Glioma_Nuclear_prep_Gradeall_4plex.raw
148596949		High	aALSLPAk	Unambiguous	3	1	N-Term(iTRAQ4plex); K8(iTRAQ4plex)	HCD	1.0000	0.0000	1	1	8905	Unique	Used	0.602	7.295E4	5.317E4	153	2.95	0.00	0	61	97	2	529.84137	1058.67546	-5.45	32.69	3279	3279	MS2	13/14	Glioma_Nuclear_prep_Gradeall_repeat_4plex.raw
148596949		High	vADNSFDAk	Unambiguous	3	1	N-Term(iTRAQ4plex); K9(iTRAQ4plex)	HCD	1.0000	0.0000	1	1	7864	Unique	Used	0.733	3.217E4	2.853E4	207	2.87	0.00	0	32	160	2	627.82922	1254.65117	-4.55	22.50	2183	2183	MS2	15/16	Glioma_Nuclear_prep_Gradeall_repeat_4plex.raw
149999611	NP_003125.3	SRP14	6727	signal recognition particle 14 kDa protein [Homo sapiens]	13.24	1	2	3	1.662	1;2	1.0	6.84	136	14.6	10.04																			
149999611		High	fQMAYSNLLR	Unambiguous	1	1	N-Term(iTRAQ4plex)	HCD	1.0000	0.0000	1	1	11589	Unique	Used	1.660	2.535E3	5.091E3	278	3.68	0.00	0	43	300	2	693.87103	1386.73479	1.96	65.43	6237	6237	MS2	17/18	Glioma_Nuclear_prep_Gradeall_repeat_4plex.raw
149999611	High		fQMAYSNLLR	Unambiguous	1	1	N-Term(iTRAQ4plex)	HCD	1.0000	0.0000	1	1	10712	Unique	Used	1.662	9.478E4	1.922E5	224	3.21	0.00	0	30	19	2	693.85974	1386.71221	-14.32	46.24	5408	5408	MS2	17/18	Glioma_Nuclear_prep_Gradeall_4plex.raw
149999611		High	iSTVVSSk	Unambiguous	1	1	N-Term(iTRAQ4plex); K8(iTRAQ4plex)	HCD	1.0000	0.0000	1	1	8081	Unique	Used	1.690	1.544E4	3.157E4	226	3.16	0.00	0	44	123	2	554.84393	1108.68059	-0.93	24.56	2411	2411	MS2	13/14	Glioma_Nuclear_prep_Gradeall_repeat_4plex.raw
151101292	NP_110382.3	TMX1	81542	thioredoxin-related transmembrane protein 1 precursor [Homo sapiens]	7.86	1	2	3	2.209	2;1	38.1	11.00	280	31.8	4.98																			
151101292	High		fIITALPTIYHck	Unambiguous	1	1	N-Term(iTRAQ4plex); C12(Methylthio); K13(iTRAQ4plex)	HCD	1.0000	0.0000	1	1	11820	Unique	Used	3.914	6.536E3	3.121E4	208	7.00	0.00	0	23	31	3	618.67883	1854.02195	-2.20	55.38	6616	6616	MS2	28/48	Glioma_Nuclear_prep_Gradeall_4plex.raw
151101292		High	dFINFISDk	Unambiguous	1	1	N-Term(iTRAQ4plex); K9(iTRAQ4plex)	HCD	1.0000	0.0000	1	1	11690	Unique	Used	2.039	1.767E3	4.361E3	278	3.88	0.00	0	45	300	2	693.87970	1386.75212	0.97	66.84	6357	6357	MS2	16/16	Glioma_Nuclear_prep_Gradeall_repeat_4plex.raw
151101292	High		dFINFISDk	Unambiguous	1	1	N-Term(iTRAQ4plex); K9(iTRAQ4plex)	HCD	1.0000	0.0000	1	1	10895	Unique	Used	2.209	6.505E4	1.753E5	230	4.00	0.00	0	38	20	2	693.86829	1386.72930	-15.49	47.65	5607	5607	MS2	16/16	Glioma_Nuclear_prep_Gradeall_4plex.raw
153070260	NP_002347.5	MARCKS	4082	myristoylated alanine-rich C-kinase substrate [Homo sapiens]	15.06	1	3	5	2.987	1;4	38.7	16.50	332	31.5	4.45																			
153070260		High	aEDGATPSPSNETPk	Unambiguous	1	1	N-Term(iTRAQ4plex); K15(iTRAQ4plex)	HCD	1.0000	0.0000	1	1	7285	Unique	Used	2.987	5.456E2	1.972E3	78	3.96	0.00	0	41	300	2	894.93042	1788.85356	-17.83	17.25	1569	1569	MS2	19/28	Glioma_Nuclear_prep_Gradeall_repeat_4plex.raw
153070260		High	eAGEGGEAEAPAAEGGk	Unambiguous	1	1	N-Term(iTRAQ4plex); K17(iTRAQ4plex)	HCD	1.0000	0.0000	1	1	7209	Unique	Used	3.209	3.143E2	1.220E3	54	4.08	0.00	0	10	300	2	909.42712	1817.84697	-15.78	16.61	1489	1489	MS2	28/32	Glioma_Nuclear_prep_Gradeall_repeat_4plex.raw
153070260		High	gEPAAAAAPEAGASPVEk	Unambiguous	1	1	N-Term(iTRAQ4plex); K18(iTRAQ4plex)	HCD	1.0000	0.0000	1	1	8212	Unique	Used	1.705	1.111E3	2.292E3	166	4.99	0.00	0	0	300	2	956.00629	1911.00530	-0.51	25.84	2549	2549	MS2	19/34	Glioma_Nuclear_prep_Gradeall_repeat_4plex.raw
153070260		High	gEPAAAAAPEAGASPVEk	Unambiguous	1	1	N-Term(iTRAQ4plex); K18(iTRAQ4plex)	HCD	1.0000	0.0000	1	1	8204	Unique	Used	1.441	2.142E3	3.735E3	145	3.47	0.00	0	33	101	3	637.67401	1911.00748	0.63	25.77	2541	2541	MS2	26/68	Glioma_Nuclear_prep_Gradeall_repeat_4plex.raw
153070260	High		gEPAAAAAPEAGASPVEk	Unambiguous	1	1	N-Term(iTRAQ4plex); K18(iTRAQ4plex)	HCD	1.0000	0.0000	1	1	8059	Unique	Used	3.820	1.434E4	6.681E4	169	5.71	0.00	0	11	14	2	955.98950	1910.97173	-18.08	26.78	2517	2517	MS2	26/34	Glioma_Nuclear_prep_Gradeall_4plex.raw
156523968	NP_001609.2	PARP1	142	poly [ADP-ribose] polymerase 1 [Homo sapiens]	21.60	1	18	31	1.788	13;18	18.3	90.89	1014	113.0	8.88																			
156523968		High	aEPVEVVAPR	Unambiguous	1	1	N-Term(iTRAQ4plex)	HCD	1.0000	0.0000	1	1	8392	Unique	Used	2.044	4.176E4	1.033E5	151	3.24	0.00	0	7	51	2	605.84784	1210.68840	-2.36	27.59	2739	2739	MS2	17/18	Glioma_Nuclear_prep_Gradeall_repeat_4plex.raw
156523968		High	qQVPSGESAILDR	Unambiguous	1	1	N-Term(iTRAQ4plex)	HCD	1.0000	0.0000	1	1	8978	Unique	Used	1.913	3.693E3	8.548E3	260	4.07	0.00	0	22	300	2	772.41547	1543.82366	2.55	33.45	3357	3357	MS2	18/24	Glioma_Nuclear_prep_Gradeall_repeat_4plex.raw
156523968		High	tTNFAGILSQGLR	Unambiguous	1	1	N-Term(iTRAQ4plex)	HCD	1.0000	0.0000	1	1	11840	Unique	Used	1.577	1.729E3	3.297E3	247	4.59	0.00	0	32	300	2	761.42950	1521.85173	0.73	69.28	6558	6558	MS2	21/24	Glioma_Nuclear_prep_Gradeall_repeat_4plex.raw
156523968		High	dSEEAEIIR	Unambiguous	1	1	N-Term(iTRAQ4plex)	HCD	1.0000	0.0000	1	1	8157	Unique	Used	1.346	1.621E4	2.640E4	172	3.33	0.00	0	21	170	2	603.30878	1205.61028	-2.31	25.29	2491	2491	MS2	16/16	Glioma_Nuclear_prep_Gradeall_repeat_4plex.raw
156523968		High	hPDVEVDGFSELR	Unambiguous	1	1	N-Term(iTRAQ4plex)	HCD	1.0000	0.0000	1	1	10027	Unique	Used	1.309	1.320E3	2.090E3	164	5.49	0.00	0	40	300	3	548.60938	1643.81357	-0.65	45.37	4481	4481	MS2	23/48	Glioma_Nuclear_prep_Gradeall_repeat_4plex.raw
156523968	High		qQVPSGESAILDR	Unambiguous	1	1	N-Term(iTRAQ4plex)	HCD	1.0000	0.0000	1	1	8621	Unique	Used	1.843	7.909E4	1.779E5	240	3.11	0.00	0	9	15	2	772.40070	1543.79411	-16.58	30.81	3128	3128	MS2	17/24	Glioma_Nuclear_prep_Gradeall_4plex.raw
156523968	High		tTNFAGILSQGLR	Unambiguous	1	1	N-Term(iTRAQ4plex)	HCD	1.0000	0.0000	1	1	11015	Unique	Used	1.807	7.952E4	1.753E5	234	5.01	0.00	0	17	17	2	761.42804	1521.84880	-1.19	48.56	5737	5737	MS2	20/24	Glioma_Nuclear_prep_Gradeall_4plex.raw
156523968		High	wYHPGcFVk	Unambiguous	1	1	N-Term(iTRAQ4plex); C6(Methylthio); K9(iTRAQ4plex)	HCD	1.0000	0.0000	1	1	10709	Unique	Used	1.549	1.277E3	2.393E3	102	3.60	0.00	0	93	300	3	490.91003	1470.71555	-7.43	53.53	5213	5213	MS2	14/32	Glioma_Nuclear_prep_Gradeall_repeat_4plex.raw
156523968		High	kTAEAGGVTGk	Unambiguous	1	1	N-Term(iTRAQ4plex); K1(iTRAQ4plex); K11(iTRAQ4plex)	HCD	1.0000	0.0000	1	1	6763	Unique	Used	1.575	5.281E3	1.006E4	175	4.46	0.00	1	57	100	3	484.29041	1450.85666	-1.61	12.67	1018	1018	MS2	22/40	Glioma_Nuclear_prep_Gradeall_repeat_4plex.raw
156523968		High	kGDEVDGVDEVAk	Unambiguous	1	1	N-Term(iTRAQ4plex); K1(iTRAQ4plex); K13(iTRAQ4plex)	HCD	1.0000	0.0000	1	1	7887	Unique	Used	1.959	2.163E3	5.125E3	201	6.18	0.00	1	29	77	3	598.32269	1792.95353	-6.59	22.70	2207	2207	MS2	28/48	Glioma_Nuclear_prep_Gradeall_repeat_4plex.raw
156523968		High	kGDEVDGVDEVAk	Unambiguous	1	1	N-Term(iTRAQ4plex); K1(iTRAQ4plex); K13(iTRAQ4plex)	HCD	1.0000	0.0000	1	1	7911	Unique	Used	1.635	2.108E3	4.169E3	222	4.74	0.00	1	38	300	2	896.97998	1792.95268	-7.06	22.92	2232	2232	MS2	20/24	Glioma_Nuclear_prep_Gradeall_repeat_4plex.raw
156523968	High		kGDEVDGVDEVAk	Unambiguous	1	1	N-Term(iTRAQ4plex); K1(iTRAQ4plex); K13(iTRAQ4plex)	HCD	1.0000	0.0000	1	1	7761	Unique	Used	2.022	3.940E4	9.719E4	164	4.46	0.00	1	48	3	3	598.32654	1792.96506	-0.16	24.67	2194	2194	MS2	26/48	Glioma_Nuclear_prep_Gradeall_4plex.raw
156523968	High		kGDEVDGVDEVAk	Unambiguous	1	1	N-Term(iTRAQ4plex); K1(iTRAQ4plex); K13(iTRAQ4plex)	HCD	1.0000	0.0000	1	1	7765	Unique	Used	1.630	7.422E4	1.476E5	178	5.02	0.00	1	11	11	2	896.98718	1792.96709	0.98	24.69	2198	2198	MS2	21/24	Glioma_Nuclear_prep_Gradeall_4plex.raw
156523968		High	kPPLLNNADSVQAk	Unambiguous	1	1	N-Term(iTRAQ4plex); K1(iTRAQ4plex); K14(iTRAQ4plex)	HCD	1.0000	0.0000	1	1	8670	Unique	Used	1.508	2.771E3	5.054E3	70	6.99	0.00	0	29	101	3	643.04919	1927.13303	-0.36	30.37	3032	3032	MS2	24/52	Glioma_Nuclear_prep_Gradeall_repeat_4plex.raw
156523968	High		kPPLLNNADSVQAk	Unambiguous	1	1	N-Term(iTRAQ4plex); K1(iTRAQ4plex); K14(iTRAQ4plex)	HCD	1.0000	0.0000	1	1	8255	Unique	Used	2.021	2.848E4	7.023E4	118	4.21	0.00	0	20	6	3	643.03717	1927.09696	-19.08	28.16	2729	2729	MS2	24/52	Glioma_Nuclear_prep_Gradeall_4plex.raw
156523968		High	kFYPLEIDYGQDEEAVk	Unambiguous	1	1	N-Term(iTRAQ4plex); K1(iTRAQ4plex); K17(iTRAQ4plex)	HCD	1.0000	0.0000	1	1	11185	Unique	Used	1.386	6.004E2	1.007E3	175	7.17	0.00	1	15	300	3	826.10455	2476.29911	0.76	59.61	5745	5745	MS2	37/64	Glioma_Nuclear_prep_Gradeall_repeat_4plex.raw
156523968		High	gIYFADMVSk	Unambiguous	1	1	N-Term(iTRAQ4plex); K10(iTRAQ4plex)	HCD	1.0000	0.0000	1	1	11087	Unique	Used	1.939	3.374E3	7.913E3	269	3.98	0.00	0	62	300	2	709.88293	1418.75859	-0.45	58.28	5630	5630	MS2	17/18	Glioma_Nuclear_prep_Gradeall_repeat_4plex.raw
156523968		High	gFSLLATEDk	Unambiguous	1	1	N-Term(iTRAQ4plex); K10(iTRAQ4plex)	HCD	1.0000	0.0000	1	1	10304	Unique	Used	1.871	1.028E4	2.326E4	226	4.35	0.00	0	17	220	2	684.88501	1368.76274	1.02	48.72	4780	4780	MS2	18/18	Glioma_Nuclear_prep_Gradeall_repeat_4plex.raw
156523968	High		gIYFADMVSk	Unambiguous	1	1	N-Term(iTRAQ4plex); K10(iTRAQ4plex)	HCD	1.0000	0.0000	1	1	10262	Unique	Used	1.745	8.083E4	1.721E5	220	3.09	0.00	0	35	15	2	709.87207	1418.73686	-15.77	42.80	4915	4915	MS2	17/18	Glioma_Nuclear_prep_Gradeall_4plex.raw
156523968	High		gFSLLATEDk	Unambiguous	1	1	N-Term(iTRAQ4plex); K10(iTRAQ4plex)	HCD	1.0000	0.0000	1	1	9615	Unique	Used	1.584	3.126E5	6.039E5	232	3.52	0.00	0	20	4	2	684.87335	1368.73943	-16.01	38.08	4212	4212	MS2	18/18	Glioma_Nuclear_prep_Gradeall_4plex.raw
156523968		High	tLGDFAAEYAk	Unambiguous	1	1	N-Term(iTRAQ4plex); K11(iTRAQ4plex)	HCD	1.0000	0.0000	1	1	10762	Unique	Used	1.849	8.247E3	1.845E4	208	4.48	0.00	0	20	300	2	737.39630	1473.78533	1.71	54.19	5271	5271	MS2	20/20	Glioma_Nuclear_prep_Gradeall_repeat_4plex.raw
156523968		High	tLGDFAAEYAk	Unambiguous	1	1	N-Term(iTRAQ4plex); K11(iTRAQ4plex)	HCD	1.0000	0.0000	1	1	10767	Unique	Used	1.584	8.555E2	1.639E3	280	3.49	0.00	0	37	300	3	491.93161	1473.78028	-1.72	54.25	5276	5276	MS2	18/40	Glioma_Nuclear_prep_Gradeall_repeat_4plex.raw
156523968	High		tLGDFAAEYAk	Unambiguous	1	1	N-Term(iTRAQ4plex); K11(iTRAQ4plex)	HCD	1.0000	0.0000	1	1	10028	Unique	Used	1.487	2.123E5	3.850E5	198	4.32	0.00	0	20	11	2	737.38245	1473.75762	-17.09	41.06	4660	4660	MS2	20/20	Glioma_Nuclear_prep_Gradeall_4plex.raw
156523968	High		fYTLIPHDFGMk	Unambiguous	1	1	N-Term(iTRAQ4plex); K12(iTRAQ4plex)	HCD	1.0000	0.0000	1	1	11156	Unique	Used	2.113	9.336E3	2.407E4	158	3.94	0.00	0	77	31	3	586.31543	1756.93174	-1.01	49.67	5891	5891	MS2	24/44	Glioma_Nuclear_prep_Gradeall_4plex.raw
156523968	High		fYTLIPHDFGMk	Unambiguous	1	1	N-Term(iTRAQ4plex); K12(iTRAQ4plex)	HCD	1.0000	0.0000	1	1	11231	Unique	Used	1.512	1.957E4	3.611E4	264	3.64	0.00	0	53	170	2	878.97174	1756.93620	1.53	50.28	5973	5973	MS2	14/22	Glioma_Nuclear_prep_Gradeall_4plex.raw
156523968		High	vVSEDFLQDVSASTk	Unambiguous	1	1	N-Term(iTRAQ4plex); K15(iTRAQ4plex)	HCD	1.0000	0.0000	1	1	11518	Unique	Used	2.479	1.050E3	3.150E3	186	6.33	0.00	0	7	300	2	957.01007	1913.01286	1.15	64.36	6148	6148	MS2	25/28	Glioma_Nuclear_prep_Gradeall_repeat_4plex.raw
156523968	High		vVSEDFLQDVSASTk	Unambiguous	1	1	N-Term(iTRAQ4plex); K15(iTRAQ4plex)	HCD	1.0000	0.0000	1	1	10754	Unique	Used	2.544	8.154E4	2.531E5	166	6.57	0.00	0	10	11	2	956.99445	1912.98161	-15.18	46.57	5454	5454	MS2	24/28	Glioma_Nuclear_prep_Gradeall_4plex.raw
156523968	High		vVSEDFLQDVSASTk	Unambiguous	1	1	N-Term(iTRAQ4plex); K15(iTRAQ4plex)	HCD	1.0000	0.0000	1	1	10742	Unique	Used	1.788	1.401E4	3.057E4	161	4.21	0.00	0	11	5	3	638.33252	1912.98301	-14.45	46.48	5441	5441	MS2	22/56	Glioma_Nuclear_prep_Gradeall_4plex.raw
156523968		High	eLLIFNk	Unambiguous	1	1	N-Term(iTRAQ4plex); K7(iTRAQ4plex)	HCD	1.0000	0.0000	1	1	10911	Unique	Used	2.632	6.297E3	2.005E4	265	3.11	0.00	0	41	300	2	582.86475	1164.72222	-0.76	56.08	5436	5436	MS2	12/12	Glioma_Nuclear_prep_Gradeall_repeat_4plex.raw
156523968		High	lTVNPGTk	Unambiguous	1	1	N-Term(iTRAQ4plex); K8(iTRAQ4plex)	HCD	1.0000	0.0000	1	1	7846	Unique	Used	1.823	2.322E4	5.121E4	313	3.21	0.00	0	51	119	2	559.34167	1117.67607	-5.26	22.32	2164	2164	MS2	11/14	Glioma_Nuclear_prep_Gradeall_repeat_4plex.raw
156523968	High		mIFDVESMk	Unambiguous	1	1	N-Term(iTRAQ4plex); K9(iTRAQ4plex)	HCD	1.0000	0.0000	1	1	10629	Unique	Used	1.707	1.268E5	2.642E5	125	3.51	0.00	0	18	11	2	694.35297	1387.69866	-15.69	45.61	5317	5317	MS2	15/16	Glioma_Nuclear_prep_Gradeall_4plex.raw
157412270	NP_112480.2	HNRNPM	4670	heterogeneous nuclear ribonucleoprotein M isoform b [Homo sapiens]	20.26	3	9	15	0.548	8;7	22.0	41.50	691	73.6	8.82																			
157412270		High	gNFGGSFAGSFGGAGGHAPGVAR	Unambiguous	3	1	N-Term(iTRAQ4plex)	HCD	1.0000	0.0000	1	1	10150	Unique	Used	0.453	1.550E3	8.491E2	146	8.75	0.00	0	28	296	3	727.02515	2179.06089	2.66	46.87	4615	4615	MS2	39/88	Glioma_Nuclear_prep_Gradeall_repeat_4plex.raw
157412270		High	fESPEVAER	Unambiguous	3	1	N-Term(iTRAQ4plex)	HCD	1.0000	0.0000	1	1	8338	Unique	Used	0.530	3.179E4	2.039E4	178	3.22	0.00	0	44	182	2	604.31018	1207.61308	4.55	27.06	2682	2682	MS2	15/16	Glioma_Nuclear_prep_Gradeall_repeat_4plex.raw
157412270		High	mGPAMGPALGAGIER	Unambiguous	3	1	N-Term(iTRAQ4plex)	HCD	1.0000	0.0000	1	1	10189	Unique	Used	0.747	2.872E3	2.596E3	247	3.04	0.00	0	57	300	2	786.41364	1571.81999	2.83	47.33	4656	4656	MS2	16/28	Glioma_Nuclear_prep_Gradeall_repeat_4plex.raw
157412270	High		mGPAMGPALGAGIER	Unambiguous	3	1	N-Term(iTRAQ4plex)	HCD	1.0000	0.0000	1	1	9606	Unique	Used	0.320	2.417E5	9.448E4	154	4.33	0.00	0	31	14	2	786.40015	1571.79302	-14.33	38.01	4202	4202	MS2	23/28	Glioma_Nuclear_prep_Gradeall_4plex.raw

157412270	High		gNFGGSFAGSFGGAGGHAPGVAR	Unambiguous	3	1	N-Term(iTRAQ4plex)	HCD	1.0000	0.0000	1	1	9412	Unique	Used	0.475	1.547E4	8.957E3	95	5.47	0.00	0	25	8	3	727.01166	2179.02042	-15.91	36.60	3991	3991	MS2	39/88	Glioma_Nuclear_prep_Gradeall_4plex.raw
157412270	High		mGPLGLDHMASSIER	Unambiguous	3	1	N-Term(iTRAQ4plex)	HCD	1.0000	0.0000	1	1	9975	Unique	Used	0.503	9.449E4	5.799E4	124	4.76	0.00	0	5	25	2	879.43085	1757.85442	-14.32	40.67	4602	4602	MS2	21/28	Glioma_Nuclear_prep_Gradeall_4plex.raw
157412270	High		mGPLGLDHMASSIER	Unambiguous	3	1	N-Term(iTRAQ4plex)	HCD	1.0000	0.0000	1	1	9957	Unique	Used	0.607	5.280E4	3.910E4	121	3.58	0.00	0	25	7	3	586.62225	1757.85221	-15.57	40.55	4583	4583	MS2	24/56	Glioma_Nuclear_prep_Gradeall_4plex.raw
157412270		High	iNEILSNALk	Unambiguous	3	1	N-Term(iTRAQ4plex); K10(iTRAQ4plex)	HCD	1.0000	0.0000	1	1	11475	Unique	Used	0.448	6.644E3	3.603E3	158	4.06	0.00	0	21	300	2	701.93066	1402.85405	2.30	63.83	6101	6101	MS2	18/18	Glioma_Nuclear_prep_Gradeall_repeat_4plex.raw
157412270		High	iNEILSNALk	Unambiguous	3	1	N-Term(iTRAQ4plex); K10(iTRAQ4plex)	HCD	1.0000	0.0000	1	1	11490	Unique	Used	0.570	4.458E2	3.074E2	137	3.62	0.00	0	21	300	3	468.29068	1402.85749	4.75	64.01	6117	6117	MS2	20/36	Glioma_Nuclear_prep_Gradeall_repeat_4plex.raw
157412270	High		iNEILSNALk	Unambiguous	3	1	N-Term(iTRAQ4plex); K10(iTRAQ4plex)	HCD	1.0000	0.0000	1	1	10597	Unique	Used	0.548	2.310E5	1.545E5	209	4.20	0.00	0	49	20	2	701.91925	1402.83122	-13.97	45.37	5282	5282	MS2	17/18	Glioma_Nuclear_prep_Gradeall_4plex.raw
157412270		High	aFITNIPFDVk	Unambiguous	2	1	N-Term(iTRAQ4plex); K11(iTRAQ4plex)	HCD	1.0000	0.0000	1	1	12031	Unique	Used	0.625	2.738E3	2.069E3	196	4.22	0.00	0	37	300	2	776.95386	1552.90044	1.71	72.57	6824	6824	MS2	17/20	Glioma_Nuclear_prep_Gradeall_repeat_4plex.raw
157412270	High		aFITNIPFDVk	Unambiguous	2	1	N-Term(iTRAQ4plex); K11(iTRAQ4plex)	HCD	1.0000	0.0000	1	1	11249	Unique	Used	0.595	3.509E4	2.548E4	214	4.46	0.00	0	35	49	2	776.95209	1552.89690	-0.57	50.43	5993	5993	MS2	17/20	Glioma_Nuclear_prep_Gradeall_4plex.raw
157412270	High		gIGMGNIGPAGMGMEGIGFGINk	Unambiguous	2	1	N-Term(iTRAQ4plex); K23(iTRAQ4plex)	HCD	1.0000	0.0000	1	1	11609	Unique	Used	0.693	3.013E3	2.547E3	129	5.34	0.00	0	54	85	3	822.76038	2466.26657	4.81	53.48	6386	6386	MS2	19/88	Glioma_Nuclear_prep_Gradeall_4plex.raw
157412270		High	fEPYANPTk	Unambiguous	2	1	N-Term(iTRAQ4plex); K9(iTRAQ4plex)	HCD	1.0000	0.0000	1	1	8693	Unique	Used	0.551	4.249E4	2.834E4	238	3.44	0.00	0	16	147	2	677.87012	1354.73296	6.21	30.58	3056	3056	MS2	15/16	Glioma_Nuclear_prep_Gradeall_repeat_4plex.raw
157412270	High		nLPFDFTWk	Unambiguous	3	1	N-Term(iTRAQ4plex); K9(iTRAQ4plex)	HCD	1.0000	0.0000	1	1	11755	Unique	Used	0.335	7.123E4	2.910E4	277	3.87	0.00	0	44	73	2	728.39569	1455.78411	-2.33	54.83	6545	6545	MS2	13/16	Glioma_Nuclear_prep_Gradeall_4plex.raw
160707950	NP_001104262.1	MECP2	4204	methyl-CpG-binding protein 2 isoform 2 [Homo sapiens]	37.15	2	14	35	0.608	15;20	18.8	113.42	498	53.3	9.88																			
160707950		High	vGDTSLDPNDFDFTVTGR	Unambiguous	2	1	N-Term(iTRAQ4plex)	HCD	1.0000	0.0000	1	1	11790	Unique	Used	0.425	5.034E2	2.590E2	97	5.01	0.00	0	31	292	3	700.67334	2100.00547	2.47	68.41	6488	6488	MS2	21/68	Glioma_Nuclear_prep_Gradeall_repeat_4plex.raw
160707950		High	vGDTSLDPNDFDFTVTGR	Unambiguous	2	1	N-Term(iTRAQ4plex)	HCD	1.0000	0.0000	1	1	11776	Unique	Used	0.594	1.938E3	1.393E3	69	5.17	0.00	0	0	300	2	1050.50513	2100.00298	1.28	68.15	6467	6467	MS2	24/34	Glioma_Nuclear_prep_Gradeall_repeat_4plex.raw
160707950	High		vGDTSLDPNDFDFTVTGR	Unambiguous	2	1	N-Term(iTRAQ4plex)	HCD	1.0000	0.0000	1	1	11212	Unique	Used	0.546	1.967E4	1.311E4	73	5.92	0.00	0	36	94	2	1050.49500	2099.98271	-8.37	50.11	5952	5952	MS2	23/34	Glioma_Nuclear_prep_Gradeall_4plex.raw
160707950	High		vGDTSLDPNDFDFTVTGR	Unambiguous	2	1	N-Term(iTRAQ4plex)	HCD	1.0000	0.0000	1	1	11109	Unique	Used	0.573	1.150E5	8.037E4	66	6.00	0.00	0	6	20	2	1050.49988	2099.99248	-3.72	49.31	5840	5840	MS2	24/34	Glioma_Nuclear_prep_Gradeall_4plex.raw
160707950		High	gGSLESDGcPk	Unambiguous	2	1	N-Term(iTRAQ4plex); C9(Methylthio); K11(iTRAQ4plex)	HCD	1.0000	0.0000	1	1	8376	Unique	Used	0.452	1.846E4	1.010E4	87	4.00	0.00	0	28	209	2	692.33191	1383.65654	5.66	27.42	2722	2722	MS2	18/20	Glioma_Nuclear_prep_Gradeall_repeat_4plex.raw
160707950		High	gGSLESDGcPk	Unambiguous	2	1	N-Term(iTRAQ4plex); C9(Methylthio); K11(iTRAQ4plex)	HCD	1.0000	0.0000	1	1	8269	Unique	Used	0.553	8.687E3	5.814E3	102	3.57	0.00	0	42	300	2	692.33301	1383.65874	7.25	26.38	2609	2609	MS2	16/20	Glioma_Nuclear_prep_Gradeall_repeat_4plex.raw
160707950	High		gGSLESDGcPk	Unambiguous	2	1	N-Term(iTRAQ4plex); C9(Methylthio); K11(iTRAQ4plex)	HCD	1.0000	0.0000	1	1	8082	Unique	Used	0.403	3.124E5	1.536E5	50	4.34	0.00	0	37	11	2	692.31897	1383.63066	-13.04	26.94	2542	2542	MS2	19/20	Glioma_Nuclear_prep_Gradeall_4plex.raw
160707950	High		kAEADPQAIPk	Unambiguous	1	1	N-Term(iTRAQ4plex); K1(iTRAQ4plex); K11(iTRAQ4plex)	HCD	1.0000	0.0000	1	1	7449	Unique	Used	0.671	1.861E5	1.523E5	139	3.60	0.00	1	9	7	2	800.47180	1599.93633	-4.21	22.46	1856	1856	MS2	17/20	Glioma_Nuclear_prep_Gradeall_4plex.raw
160707950		High	kPGSVVAAAAAEAk	Unambiguous	2	1	N-Term(iTRAQ4plex); K1(iTRAQ4plex); K14(iTRAQ4plex)	HCD	1.0000	0.0000	1	1	8824	Unique	Used	0.660	1.092E3	8.715E2	104	4.45	0.00	0	52	243	4	426.26117	1702.02285	0.26	31.89	3194	3194	MS2	22/78	Glioma_Nuclear_prep_Gradeall_repeat_4plex.raw
160707950		High	kPGSVVAAAAAEAk	Unambiguous	2	1	N-Term(iTRAQ4plex); K1(iTRAQ4plex); K14(iTRAQ4plex)	HCD	1.0000	0.0000	1	1	8821	Unique	Used	0.716	1.792E4	1.552E4	90	5.70	0.00	0	25	300	2	851.51697	1702.02666	2.50	31.86	3191	3191	MS2	24/26	Glioma_Nuclear_prep_Gradeall_repeat_4plex.raw
160707950		High	kPGSVVAAAAAEAk	Unambiguous	2	1	N-Term(iTRAQ4plex); K1(iTRAQ4plex); K14(iTRAQ4plex)	HCD	1.0000	0.0000	1	1	8801	Unique	Used	0.775	4.692E3	4.399E3	136	6.03	0.00	0	35	93	3	568.00439	1701.99863	-13.97	31.64	3170	3170	MS2	27/52	Glioma_Nuclear_prep_Gradeall_repeat_4plex.raw
160707950		High	sEDQDLQGLk	Unambiguous	2	1	N-Term(iTRAQ4plex); K10(iTRAQ4plex)	HCD	1.0000	0.0000	1	1	8391	Unique	Used	0.567	8.212E4	5.629E4	180	4.20	0.00	0	4	52	2	710.87982	1420.75237	0.09	27.59	2738	2738	MS2	18/18	Glioma_Nuclear_prep_Gradeall_repeat_4plex.raw
160707950		High	sVQETVLPIk	Unambiguous	2	1	N-Term(iTRAQ4plex); K10(iTRAQ4plex)	HCD	1.0000	0.0000	1	1	9777	Unique	Used	0.653	4.892E4	3.867E4	139	4.10	0.00	0	41	87	2	701.43262	1401.85796	1.70	42.41	4211	4211	MS2	16/18	Glioma_Nuclear_prep_Gradeall_repeat_4plex.raw
160707950		High	aATSEGVQVk	Unambiguous	2	1	N-Term(iTRAQ4plex); K10(iTRAQ4plex)	HCD	1.0000	0.0000	1	1	7214	Unique	Used	0.720	7.557E4	6.579E4	244	3.07	0.00	0	11	44	2	639.35919	1277.71111	-15.08	16.66	1495	1495	MS2	18/18	Glioma_Nuclear_prep_Gradeall_repeat_4plex.raw
160707950		High	aEADPQAIPk	Unambiguous	2	1	N-Term(iTRAQ4plex); K10(iTRAQ4plex)	HCD	1.0000	0.0000	1	1	7783	Unique	Used	0.725	1.381E4	1.212E4	268	3.28	0.00	0	23	155	2	664.37604	1327.74480	-0.93	21.71	2097	2097	MS2	15/18	Glioma_Nuclear_prep_Gradeall_repeat_4plex.raw
160707950	High		sVQETVLPIk	Unambiguous	2	1	N-Term(iTRAQ4plex); K10(iTRAQ4plex)	HCD	1.0000	0.0000	1	1	9182	Unique	Used	0.527	1.023E6	6.576E5	159	3.27	0.00	0	29	3	2	701.42194	1401.83660	-13.54	34.89	3739	3739	MS2	16/18	Glioma_Nuclear_prep_Gradeall_4plex.raw
160707950	High		sEDQDLQGLk	Unambiguous	2	1	N-Term(iTRAQ4plex); K10(iTRAQ4plex)	HCD	1.0000	0.0000	1	1	8155	Unique	Used	0.527	8.328E5	5.358E5	144	4.25	0.00	0	10	4	2	710.86884	1420.73039	-15.37	27.46	2621	2621	MS2	18/18	Glioma_Nuclear_prep_Gradeall_4plex.raw
160707950	High		aEADPQAIPk	Unambiguous	2	1	N-Term(iTRAQ4plex); K10(iTRAQ4plex)	HCD	1.0000	0.0000	1	1	7684	Unique	Used	0.695	1.020E5	8.657E4	263	3.18	0.00	0	23	15	2	664.37653	1327.74578	-0.20	24.12	2110	2110	MS2	16/18	Glioma_Nuclear_prep_Gradeall_4plex.raw
160707950		High	tQPAVATAATAAEk	Unambiguous	2	1	N-Term(iTRAQ4plex); K14(iTRAQ4plex)	HCD	1.0000	0.0000	1	1	8136	Unique	Used	0.358	2.403E4	1.041E4	217	5.33	0.00	0	52	300	2	809.45740	1617.90752	1.51	25.09	2469	2469	MS2	21/26	Glioma_Nuclear_prep_Gradeall_repeat_4plex.raw
160707950		High	tQPAVATAATAAEk	Unambiguous	2	1	N-Term(iTRAQ4plex); K14(iTRAQ4plex)	HCD	1.0000	0.0000	1	1	8152	Unique	Used	0.481	8.912E3	5.183E3	218	5.96	0.00	0	25	51	3	539.97394	1617.90726	1.36	25.26	2486	2486	MS2	23/52	Glioma_Nuclear_prep_Gradeall_repeat_4plex.raw
160707950	High		tQPAVATAATAAEk	Unambiguous	2	1	N-Term(iTRAQ4plex); K14(iTRAQ4plex)	HCD	1.0000	0.0000	1	1	7939	Unique	Used	0.329	4.780E5	1.921E5	230	5.06	0.00	0	6	8	2	809.45905	1617.91081	3.55	25.94	2387	2387	MS2	22/26	Glioma_Nuclear_prep_Gradeall_4plex.raw
160707950		High	aEGGGATTSTQVMVIk	Unambiguous	2	1	N-Term(iTRAQ4plex); K16(iTRAQ4plex)	HCD	1.0000	0.0000	1	1	8944	Unique	Used	0.612	1.074E4	7.958E3	203	7.20	0.00	0	0	196	2	919.50018	1837.99309	-0.09	33.05	3320	3320	MS2	29/30	Glioma_Nuclear_prep_Gradeall_repeat_4plex.raw
160707950		High	aEGGGATTSTQVMVIk	Unambiguous	2	1	N-Term(iTRAQ4plex); K16(iTRAQ4plex)	HCD	1.0000	0.0000	1	1	9043	Unique	Used	0.662	2.536E3	2.032E3	211	6.58	0.00	0	13	300	2	919.50397	1838.00066	4.03	34.23	3428	3428	MS2	30/30	Glioma_Nuclear_prep_Gradeall_repeat_4plex.raw
160707950	High		aEGGGATTSTQVMVIk	Unambiguous	2	1	N-Term(iTRAQ4plex); K16(iTRAQ4plex)	HCD	1.0000	0.0000	1	1	8509	Unique	Used	0.617	1.704E4	1.282E4	182	5.18	0.00	0	8	22	2	919.48737	1837.96745	-14.03	29.96	3004	3004	MS2	29/30	Glioma_Nuclear_prep_Gradeall_4plex.raw
160707950		High	hEPVQPSAHHSAEPAEAGk	Unambiguous	2	1	N-Term(iTRAQ4plex); K19(iTRAQ4plex)	HCD	1.0000	0.0000	1	1	6741	Unique	Used	0.608	4.840E3	3.558E3	150	5.02	0.00	0		30	4	567.53339	2267.11172	-12.82	12.50	995	995	MS2	25/108	Glioma_Nuclear_prep_Gradeall_repeat_4plex.raw
160707950		High	hEPVQPSAHHSAEPAEAGk	Unambiguous	2	1	N-Term(iTRAQ4plex); K19(iTRAQ4plex)	HCD	1.0000	0.0000	1	1	6752	Unique	Used	0.628	4.121E3	3.130E3	89	4.17	0.00	0	14	131	3	756.38641	2267.14469	1.72	12.58	1006	1006	MS2	21/72	Glioma_Nuclear_prep_Gradeall_repeat_4plex.raw
160707950	High		hEPVQPSAHHSAEPAEAGk	Unambiguous	2	1	N-Term(iTRAQ4plex); K19(iTRAQ4plex)	HCD	1.0000	0.0000	1	1	7101	Unique	Used	0.655	6.197E3	4.950E3	161	5.16	0.00	0	15	22	4	567.54199	2267.14614	2.36	19.65	1475	1475	MS2	26/108	Glioma_Nuclear_prep_Gradeall_4plex.raw
160707950	High		hEPVQPSAHHSAEPAEAGk	Unambiguous	2	1	N-Term(iTRAQ4plex); K19(iTRAQ4plex)	HCD	1.0000	0.0000	1	1	7092	Unique	Used	0.665	6.821E3	5.536E3	94	3.71	0.00	0	21	300	3	756.38770	2267.14853	3.42	19.53	1464	1464	MS2	22/72	Glioma_Nuclear_prep_Gradeall_4plex.raw
160707950		High	eVVkPLLVSTLGEk	Unambiguous	2	1	N-Term(iTRAQ4plex); K4(iTRAQ4plex); K14(iTRAQ4plex)	HCD	1.0000	0.0000	1	1	10872	Unique	Used	0.515	2.029E3	1.264E3	10	4.12	0.00	0	42	300	3	648.74677	1944.22574	7.79	55.58	5392	5392	MS2	24/52	Glioma_Nuclear_prep_Gradeall_repeat_4plex.raw
160707950	High		eVVkPLLVSTLGEk	Unambiguous	2	1	N-Term(iTRAQ4plex); K4(iTRAQ4plex); K14(iTRAQ4plex)	HCD	1.0000	0.0000	1	1	9940	Unique	Used	0.439	1.421E5	7.619E4	32	4.75	0.00	0	12	25	2	972.60138	1944.19548	-7.77	40.42	4564	4564	MS2	21/26	Glioma_Nuclear_prep_Gradeall_4plex.raw
160707950		High	eTVSIEVk	Unambiguous	2	1	N-Term(iTRAQ4plex); K8(iTRAQ4plex)	HCD	1.0000	0.0000	1	1	8585	Unique	Used	0.658	9.364E4	7.449E4	221	3.57	0.00	0	15	58	2	596.85474	1192.70220	-0.47	29.48	2943	2943	MS2	14/14	Glioma_Nuclear_prep_Gradeall_repeat_4plex.raw
160707950	High		eTVSIEVk	Unambiguous	2	1	N-Term(iTRAQ4plex); K8(iTRAQ4plex)	HCD	1.0000	0.0000	1	1	8217	Unique	Used	0.717	5.163E5	4.518E5	296	3.38	0.00	0	43	7	2	596.84741	1192.68755	-12.75	27.89	2688	2688	MS2	14/14	Glioma_Nuclear_prep_Gradeall_4plex.raw
160707950		High	vELIAYFEk	Unambiguous	2	1	N-Term(iTRAQ4plex); K9(iTRAQ4plex)	HCD	1.0000	0.0000	1	1	12124	Unique	Used	0.594	4.559E3	3.277E3	205	3.67	0.00	0	55	300	2	700.40961	1399.81194	3.13	74.67	6984	6984	MS2	15/16	Glioma_Nuclear_prep_Gradeall_repeat_4plex.raw
160707950	High		vELIAYFEk	Unambiguous	2	1	N-Term(iTRAQ4plex); K9(iTRAQ4plex)	HCD	1.0000	0.0000	1	1	11464	Unique	Used	0.562	3.273E5	2.246E5	217	3.98	0.00	0	23	15	2	700.40765	1399.80803	0.34	52.31	6229	6229	MS2	16/16	Glioma_Nuclear_prep_Gradeall_4plex.raw
160707950	High		vELIAYFEk	Unambiguous	2	1	N-Term(iTRAQ4plex); K9(iTRAQ4plex)	HCD	1.0000	0.0000	1	1	11387	Unique	Used	0.671	7.945E4	6.507E4	225	4.24	0.00	0	42	51	2	700.40808	1399.80889	0.95	51.63	6144	6144	MS2	15/16	Glioma_Nuclear_prep_Gradeall_4plex.raw
189217895	NP_689901.2	KHDRBS2	202559	KH domain-containing, RNA-binding, signal transduction-associated protein 2 [Homo sapiens]	10.03	5	2	3	0.294	2;1	16.9	13.20	349	38.9	6.29																			
189217895		High	fLVPDYNDEIR	Unambiguous	1	1	N-Term(iTRAQ4plex)	HCD	1.0000	0.0000	1	1	11164	Unique	Used	0.242	1.118E4	3.278E3	280	3.24	0.00	0	38	300	2	762.89685	1524.78642	3.21	59.32	5720	5720	MS2	13/20	Glioma_Nuclear_prep_Gradeall_repeat_4plex.raw
189217895	High		fLVPDYNDEIR	Unambiguous	1	1	N-Term(iTRAQ4plex)	HCD	1.0000	0.0000	1	1	10388	Unique	Used	0.294	1.484E5	5.316E4	194	3.02	0.00	0	49	33	2	762.88330	1524.75932	-14.56	43.75	5052	5052	MS2	12/20	Glioma_Nuclear_prep_Gradeall_4plex.raw
189217895	High		ILAEEIEk	Unambiguous	1	1	N-Term(iTRAQ4plex); K8(iTRAQ4plex)	HCD	1.0000	0.0000	1	1	9223	Unique	Used	0.341	3.521E5	1.467E5	292	3.08	0.00	0	35	12	2	616.86536	1232.72344	-8.62	35.17	3783	3783	MS2	14/14	Glioma_Nuclear_prep_Gradeall_4plex.raw
192449447	NP_001122306.1	PLP1	5354	myelin proteolipid protein isoform 1 [Homo sapiens]	20.22	3	3	4	0.633	3;1	13.6	26.76	277	30.1	8.35																			
192449447	High		vcGSNLLSIck	Unambiguous	3	1	N-Term(iTRAQ4plex); C2(Methylthio); C10(Methylthio); K11(iTRAQ4plex)	HCD	1.0000	0.0000	1	1	12185	Unique	Used	0.629	8.151E4	6.254E4	154	4.77	0.00	0	18	53	2	758.88757	1516.76787	5.29	58.83	7015	7015	MS2	18/20	Glioma_Nuclear_prep_Gradeall_4plex.raw
192449447		High	tSASIGSLcADAR	Unambiguous	3	1	N-Term(iTRAQ4plex); C9(Methylthio)	HCD	1.0000	0.0000	1	1	10729	Unique	Used	0.677	8.208E3	6.719E3	125	4.40	0.00	0	44	300	2	721.34924	1441.69121	1.10	53.78	5235	5235	MS2	19/24	Glioma_Nuclear_prep_Gradeall_repeat_4plex.raw
192449447	High		tSASIGSLcADAR	Unambiguous	3	1	N-Term(iTRAQ4plex); C9(Methylthio)	HCD	1.0000	0.0000	1	1	9956	Unique	Used	0.488	3.838E5	2.283E5	80	4.17	0.00	0	36	7	2	721.33728	1441.66728	-15.50	40.54	4582	4582	MS2	20/24	Glioma_Nuclear_prep_Gradeall_4plex.raw
192449447	High		mYGVLPWNAFPGk	Unambiguous	3	1	N-Term(iTRAQ4plex); K13(iTRAQ4plex)	HCD	1.0000	0.0000	1	1	11868	Unique	Used	0.638	7.217E4	5.619E4	269	4.67	0.00	0	30	43	2	884.47876	1767.95024	0.41	55.79	6668	6668	MS2	18/24	Glioma_Nuclear_prep_Gradeall_4plex.raw
193794826	NP_001123291.1	CYB5R3	1727	NADH-cytochrome b5 reductase 3 isoform 2 [Homo sapiens]	4.68	3	1	3	1.551	1;2	2.4	9.52	278	31.6	7.58																			
193794826		High	sTPAITLESPDIk	Unambiguous	3	1	N-Term(iTRAQ4plex); K13(iTRAQ4plex)	HCD	1.0000	0.0000	1	1	10137	Unique	Used	1.551	4.187E3	7.859E3	215	5.08	0.00	0	40	300	2	830.47577	1659.94426	2.10	46.72	4601	4601	MS2	20/24	Glioma_Nuclear_prep_Gradeall_repeat_4plex.raw
193794826		High	sTPAITLESPDIk	Unambiguous	3	1	N-Term(iTRAQ4plex); K13(iTRAQ4plex)	HCD	1.0000	0.0000	1	1	10141	Unique	Used	1.526	9.521E2	1.758E3	200	4.44	0.00	0	29	168	3	553.98572	1659.94260	1.10	46.78	4606	4606	MS2	17/48	Glioma_Nuclear_prep_Gradeall_repeat_4plex.raw
193794826	High		sTPAITLESPDIk	Unambiguous	3	1	N-Term(iTRAQ4plex); K13(iTRAQ4plex)	HCD	1.0000	0.0000	1	1	9500	Unique	Used	1.600	9.147E4	1.785E5	227	4.44	0.00	0	21	15	2	830.46063	1659.91399	-16.14	37.25	4087	4087	MS2	21/24	Glioma_Nuclear_prep_Gradeall_4plex.raw
194688133	NP_001124160.1	HMGB2	3148	high mobility group protein B2 [Homo sapiens]	26.32	1	5	7	2.729	3;4	40.0	34.59	209	24.0	7.81																			
194688133		High	kHPDSSVNFAEFSk	Unambiguous	1	1	N-Term(iTRAQ4plex); K1(iTRAQ4plex); K14(iTRAQ4plex)	HCD	1.0000	0.0000	1	1	8993	Unique	Used	2.415	8.297E2	2.425E3	145	6.39	0.00	1	30	259	3	675.69824	2025.08017	1.76	33.63	3374	3374	MS2	25/52	Glioma_Nuclear_prep_Gradeall_repeat_4plex.raw
194688133		High	sEHPGLSIGDTAk	Unambiguous	1	1	N-Term(iTRAQ4plex); K13(iTRAQ4plex)	HCD	1.0000	0.0000	1	1	7978	Unique	Used	2.665	1.162E4	3.748E4	169	5.30	0.00	0	15	25	3	533.95837	1599.86057	1.54	23.61	2303	2303	MS2	28/48	Glioma_Nuclear_prep_Gradeall_repeat_4plex.raw
194688133		High	hPDSSVNFAEFSk	Unambiguous	1	1	N-Term(iTRAQ4plex); K13(iTRAQ4plex)	HCD	1.0000	0.0000	1	1	9557	Unique	Used	2.142	7.852E2	2.035E3	140	5.39	0.00	0	13	215	3	584.95868	1752.86148	-10.32	39.93	3977	3977	MS2	24/48	Glioma_Nuclear_prep_Gradeall_repeat_4plex.raw
194688133	High		sEHPGLSIGDTAk	Unambiguous	1	1	N-Term(iTRAQ4plex); K13(iTRAQ4plex)	HCD	1.0000	0.0000	1	1	7762	Unique	Used	4.984	6.605E4	4.016E5	182	4.13	0.00	0	8	6	2	800.43286	1599.85845	0.21	24.68	2195	2195	MS2	22/24	Glioma_Nuclear_prep_Gradeall_4plex.raw
194688133	High		sEHPGLSIGDTAk	Unambiguous	1	1	N-Term(iTRAQ4plex); K13(iTRAQ4plex)	HCD	1.0000	0.0000	1	1	7771	Unique	Used	2.729	9.432E4	3.140E5	175	4.43	0.00	0	11	1	3	533.95703	1599.85654	-0.98	24.74	2205	2205	MS2	28/48	Glioma_Nuclear_prep_Gradeall_4plex.raw
194688133		High	dkQPYEQk	Unambiguous	1	1	N-Term(iTRAQ4plex); K2(iTRAQ4plex); K8(iTRAQ4plex)	HCD	1.0000	0.0000	1	1	6805	Unique	Used	5.503	2.649E3	1.764E4	254	3.13	0.00	1	9	121	2	734.41266	1467.81804	0.87	13.06	1062	1062	MS2	13/14	Glioma_Nuclear_prep_Gradeall_repeat_4plex.raw
194688133	High		skFEDMAk	Unambiguous	1	1	N-Term(iTRAQ4plex); K2(iTRAQ4plex); K8(iTRAQ4plex)	HCD	1.0000	0.0000	1	1	7730	Unique	Used	2.899	8.216E4	2.906E5	245	3.03	0.00	1	14	13	2	694.38489	1387.76250	0.65	24.45	2160	2160	MS2	14/14	Glioma_Nuclear_prep_Gradeall_4plex.raw
197927454	NP_001128181.1	DEK	7913	protein DEK isoform 2 [Homo sapiens]	20.82	2	6	16	1.889	6;10	26.8	48.25	341	38.7	8.15																			
197927454		High	vYENYPTYDLTER	Unambiguous	2	1	N-Term(iTRAQ4plex)	HCD	1.0000	0.0000	1	1	10274	Unique	Used	3.034	2.736E3	1.004E4	139	4.64	0.00	0	8	300	2	903.94019	1806.87309	3.55	48.32	4746	4746	MS2	19/24	Glioma_Nuclear_prep_Gradeall_repeat_4plex.raw
197927454	High		vYENYPTYDLTER	Unambiguous	2	1	N-Term(iTRAQ4plex)	HCD	1.0000	0.0000	1	1	9689	Unique	Used	2.772	5.379E4	1.819E5	84	4.58	0.00	0	10	15	2	903.92401	1806.84075	-14.36	38.61	4292	4292	MS2	19/24	Glioma_Nuclear_prep_Gradeall_4plex.raw
197927454		High	sIcEVLDLER	Unambiguous	2	1	N-Term(iTRAQ4plex); C3(Methylthio)	HCD	1.0000	0.0000	1	1	12416	Unique	Used	2.135	1.219E3	3.149E3	161	3.37	0.00	0	14	300	2	683.84576	1366.68425	1.10	84.60	7704	7704	MS2	17/18	Glioma_Nuclear_prep_Gradeall_repeat_4plex.raw
197927454		High	sIcEVLDLER	Unambiguous	2	1	N-Term(iTRAQ4plex); C3(Methylthio)	HCD	1.0000	0.0000	1	1	12402	Unique	Used	1.757	1.078E3	2.290E3	199	3.12	0.00	0	22	300	2	683.84528	1366.68328	0.39	83.60	7636	7636	MS2	15/18	Glioma_Nuclear_prep_Gradeall_repeat_4plex.raw
197927454	High		sIcEVLDLER	Unambiguous	2	1	N-Term(iTRAQ4plex); C3(Methylthio)	HCD	1.0000	0.0000	1	1	12226	Unique	Used	2.080	1.348E5	3.421E5	162	3.92	0.00	0	11	18	2	683.84637	1366.68547	2.00	59.26	7061	7061	MS2	17/18	Glioma_Nuclear_prep_Gradeall_4plex.raw
197927454	High		sIcEVLDLER	Unambiguous	2	1	N-Term(iTRAQ4plex); C3(Methylthio)	HCD	1.0000	0.0000	1	1	12161	Unique	Used	2.000	1.197E5	2.921E5	160	3.77	0.00	0	18	17	2	683.84369	1366.68010	-1.93	58.59	6989	6989	MS2	16/18	Glioma_Nuclear_prep_Gradeall_4plex.raw
197927454	High		sIcEVLDLER	Unambiguous	2	1	N-Term(iTRAQ4plex); C3(Methylthio)	HCD	1.0000	0.0000	1	1	12153	Unique	Used	1.689	5.325E4	1.097E5	181	3.38	0.00	0	56	117	2	683.84631	1366.68535	1.91	58.51	6980	6980	MS2	15/18	Glioma_Nuclear_prep_Gradeall_4plex.raw
197927454	High		sIcEVLDLER	Unambiguous	2	1	N-Term(iTRAQ4plex); C3(Methylthio)	HCD	1.0000	0.0000	1	1	12300	Unique	Used	1.354	9.940E3	1.642E4	197	4.24	0.00	0	40	300	2	683.84467	1366.68205	-0.51	60.03	7142	7142	MS2	16/18	Glioma_Nuclear_prep_Gradeall_4plex.raw
197927454		High	nVGQFSGFPFEk	Unambiguous	2	1	N-Term(iTRAQ4plex); K12(iTRAQ4plex)	HCD	1.0000	0.0000	1	1	11435	Unique	Used	1.778	5.966E3	1.283E4	207	4.62	0.00	0	9	300	2	822.93604	1644.86479	1.41	63.28	6054	6054	MS2	21/22	Glioma_Nuclear_prep_Gradeall_repeat_4plex.raw
197927454		High	nVGQFSGFPFEk	Unambiguous	2	1	N-Term(iTRAQ4plex); K12(iTRAQ4plex)	HCD	1.0000	0.0000	1	1	11433	Unique	Used	1.447	9.352E2	1.637E3	156	4.24	0.00	0	10	190	3	548.95923	1644.86313	0.40	63.26	6052	6052	MS2	22/44	Glioma_Nuclear_prep_Gradeall_repeat_4plex.raw
197927454		High	ILASANLEEVTMk	Unambiguous	2	1	N-Term(iTRAQ4plex); K13(iTRAQ4plex)	HCD	1.0000	0.0000	1	1	11343	Unique	Used	2.531	1.456E3	4.460E3	221	5.61	0.00	0	25	300	2	853.98492	1706.96257	1.42	62.03	5946	5946	MS2	22/24	Glioma_Nuclear_prep_Gradeall_repeat_4plex.raw
197927454		High	ILYNRPGTVSSLk	Unambiguous	2	1	N-Term(iTRAQ4plex); K13(iTRAQ4plex)	HCD	1.0000	0.0000	1	1	9391	Unique	Used	1.360	5.279E3	8.683E3	145	4.47	0.00	0	40	300	2	868.51660	1736.02593	-2.87	38.05	3795	3795	MS2	18/24	Glioma_Nuclear_prep_Gradeall_repeat_4plex.raw
197927454	High		ILASANLEEVTMk	Unambiguous	2	1	N-Term(iTRAQ4plex); K13(iTRAQ4plex)	HCD	1.0000	0.0000	1	1	10485	Unique	Used	2.678	4.376E4	1.430E5	256	5.37	0.00	0	41	28	2	853.97198	1706.93669	-13.74	44.52	5160	5160	MS2	23/24	Glioma_Nuclear_prep_Gradeall_4plex.raw
197927454		High	sGVNSELVk	Unambiguous	2	1	N-Term(iTRAQ4plex); K9(iTRAQ4plex)	HCD	1.0000	0.0000	1	1	7751	Unique	Used	1.604	6.062E4	1.176E5	291	3.57	0.00	0	12	36	2	610.85645	1220.70561	-2.70	21.44	2064	2064	MS2	16/16	Glioma_Nuclear_prep_Gradeall_repeat_4plex.raw
197927454		High	ILASANLEEVTmk	Unambiguous	2	1	N-Term(iTRAQ4plex); M12(Oxidation); K13(iTRAQ4plex)	HCD	1.0000	0.0000	1	1	10865	Unique	Used	2.391	2.969E3	8.588E3	230	5.33	0.00	0	34	300	2	861.98334	1722.95940	2.52	55.48	5384	5384	MS2	22/24	Glioma_Nuclear_prep_Gradeall_repeat_4plex.raw
197927454		High	ILASANLEEVTmk	Unambiguous	2	1	N-Term(iTRAQ4plex); M12(Oxidation); K13(iTRAQ4plex)	HCD	1.0000	0.0000	1	1	10861	Unique	Used	1.481	6.661E2	1.193E3	176	4.04	0.00	0	30	300	3	574.99023	1722.95615	0.63	55.44	5380	5380	MS2	20/48	Glioma_Nuclear_prep_Gradeall_repeat_4plex.raw
217330646	NP_006704.3	SUB1	10923	activated RNA polymerase II transcriptional coactivator p15 [Homo sapiens]	18.90	1	2	3	1.505	2;1	8.5	8.73	127	14.4	9.60																			
217330646		High	eQISDIDDAVR	Unambiguous	1	1	N-Term(iTRAQ4plex)	HCD	1.0000	0.0000	1	1	9152	Unique	Used	1.377	1.048E4	1.746E4	196	4.45	0.00	0	11	237	2	702.85931	1404.71135	1.84	35.45	3544	3544	MS2	19/20	Glioma_Nuclear_prep_Gradeall_repeat_4plex.raw
217330646	High		eQISDIDDAVR	Unambiguous	1	1	N-Term(iTRAQ4plex)	HCD	1.0000	0.0000	1	1	8813	Unique	Used	1.632	8.036E4	1.600E5	135	3.55	0.00	0	4	16	2	702.84607	1404.68486	-17.02	32.18	3336	3336	MS2	20/20	Glioma_Nuclear_prep_Gradeall_4plex.raw
217330646	High		gISLNPEQWSQLk	Unambiguous	1	1	N-Term(iTRAQ4plex); K13(iTRAQ4plex)	HCD	1.0000	0.0000	1	1	11102	Unique	Used	1.505	2.670E4	4.904E4	203	5.18	0.00	0	20	34	2	894.49408	1787.98088	-4.80	49.25	5832	5832	MS2	22/24	Glioma_Nuclear_prep_Gradeall_4plex.raw
221316767	NP_001138246.1	ELAVL4	1996	ELAV-like protein 4 isoform 2 [Homo sapiens]	24.04	6	1	2	0.450	1;1	35.0	23.79	366	40.4	9.39																			
221316767		High	sSQALLSQLYQSPNR	Unambiguous	6	1	N-Term(iTRAQ4plex)	HCD	1.0000	0.0000	1	1	10892	Unique	Used	0.339	2.419E3	9.908E2	278	4.28	0.00	0	63	300	2	918.49414	1835.98100	4.23	55.83	5414	5414	MS2	20/28	Glioma_Nuclear_prep_Gradeall_repeat_4plex.raw
221316767	High		sSQALLSQLYQSPNR	Unambiguous	6	1	N-Term(iTRAQ4plex)	HCD	1.0000	0.0000	1	1	10150	Unique	Used	0.561	3.118E4	2.135E4	268	3.99	0.00	0	0	43	2	918.48303	1835.95879	-7.87	41.96	4793	4793	MS2	17/28	Glioma_Nuclear_prep_Gradeall_4plex.raw
223555917	NP_848927.2	MTDH	92140	protein LYRIC [Homo sapiens]	7.04	1	2	4	0.646	2;2	6.5	13.12	582	63.8	9.32																			
223555917		High	tVEVAEGEAVR	Unambiguous	1	1	N-Term(iTRAQ4plex)	HCD	1.0000	0.0000	1	1	8093	Unique	Used	0.650	3.422E4	2.691E4	256	3.09	0.00	0	42	143	2	652.35114	1303.69499	-1.90	24.68	2424	2424	MS2	18/20	Glioma_Nuclear_prep_Gradeall_repeat_4plex.raw
223555917	High		tVEVAEGEAVR	Unambiguous	1	1	N-Term(iTRAQ4plex)	HCD	1.0000	0.0000	1	1	7923	Unique	Used	0.571	1.041E5	7.250E4	250	3.13	0.00	0	51	27	2	652.35291	1303.69853	0.81	25.81	2369	2369	MS2	18/20	Glioma_Nuclear_prep_Gradeall_4plex.raw
223555917		High	eEAAAVPAAAPDDLALLk	Unambiguous	1	1	N-Term(iTRAQ4plex); K18(iTRAQ4plex)	HCD	1.0000	0.0000	1	1	11440	Unique	Used	0.662	6.660E2	5.331E2	145	5.27	0.00	0	36	300	3	685.05408	2053.14768	2.74	63.35	6060	6060	MS2	25/68	Glioma_Nuclear_prep_Gradeall_repeat_4plex.raw
223555917	High		eEAAAVPAAAPDDLALLk	Unambiguous	1	1	N-Term(iTRAQ4plex); K18(iTRAQ4plex)	HCD	1.0000	0.0000	1	1	10623	Unique	Used	0.642	1.343E4	1.052E4	215	4.27	0.00	0	26	52	2	1027.05896	2053.11064	-15.30	45.56	5310	5310	MS2	17/34	Glioma_Nuclear_prep_Gradeall_4plex.raw
225903430	NP_001139630.1	MOGS	7841	mannosyl-oligosaccharide glucosidase isoform 2 [Homo sapiens]	9.85	2	4	9	2.091	8;1	23.0	43.95	731	80.7	7.11																			
225903430		High	tLPSGLDDYPR	Unambiguous	2	1	N-Term(iTRAQ4plex)	HCD	1.0000	0.0000	1	1	9781	Unique	Used	1.517	9.852E3	1.808E4	246	3.37	0.00	0	30	258	2	689.36060	1377.71391	0.59	42.45	4215	4215	MS2	16/20	Glioma_Nuclear_prep_Gradeall_repeat_4plex.raw
225903430	High		IAGSLLTQALESHAEGFR	Unambiguous	2	1	N-Term(iTRAQ4plex)	HCD	1.0000	0.0000	1	1	11953	Unique	Used	2.500	5.281E3	1.611E4	158	6.98	0.00	0	38	63	3	682.03583	2044.09293	-0.75	56.53	6761	6761	MS2	30/68	Glioma_Nuclear_prep_Gradeall_4plex.raw
225903430	High		vDPALFPPVPLFTAVPSR	Unambiguous	2	1	N-Term(iTRAQ4plex)	HCD	1.0000	0.0000	1	1	12845	Unique	Used	2.472	2.011E3	6.065E3	92	4.09	0.00	0	21	300	2	1034.09180	2067.17632	0.15	67.07	7772	7772	MS2	19/34	Glioma_Nuclear_prep_Gradeall_4plex.raw
225903430	High		vDPALFPPVPLFTAVPSR	Unambiguous	2	1	N-Term(iTRAQ4plex)	HCD	1.0000	0.0000	1	1	12892	Unique	Used	2.457	1.103E3	3.307E3	96	3.94	0.00	0	4	300	2	1034.09241	2067.17754	0.74	67.84	7834	7834	MS2	21/34	Glioma_Nuclear_prep_Gradeall_4plex.raw
225903430	High		IGPLLDILADSR	Unambiguous	2	1	N-Term(iTRAQ4plex)	HCD	1.0000	0.0000	1	1	12884	Unique	Used	2.252	8.234E3	2.262E4	127	4.67	0.00	0	10	237	2	713.92462	1426.84197	2.31	67.73	7825	7825	MS2	20/22	Glioma_Nuclear_prep_Gradeall_4plex.raw
225903430	High		IGPLLDILADSR	Unambiguous	2	1	N-Term(iTRAQ4plex)	HCD	1.0000	0.0000	1	1	12925	Unique	Used	2.091	3.695E3	9.426E3	200	4.35	0.00	0	29	300	2	713.92456	1426.84184	2.23	68.42	7880	7880	MS2	20/22	Glioma_Nuclear_prep_Gradeall_4plex.raw
225903430	High		IGPLLDILADSR	Unambiguous	2	1	N-Term(iTRAQ4plex)	HCD	1.0000	0.0000	1	1	12842	Unique	Used	1.825	3.548E3	7.898E3	222	4.31	0.00	0	37	300	2	713.92462	1426.84197	2.31	67.02	7768	7768	MS2	20/22	Glioma_Nuclear_prep_Gradeall_4plex.raw
225903430	High		vDPALFPPVPLFTAVPSR	Unambiguous	2	1	N-Term(iTRAQ4plex)	HCD	1.0000	0.0000	1	1	12886	Unique	Used	1.448	4.238E2	7.489E2	109	3.74	0.00	0	25	300	3	689.73181	2067.18088	2.35	67.75	7827	7827	MS2	18/68	Glioma_Nuclear_prep_Gradeall_4plex.raw
225903430	High		IAGSLLTQALESHAEGFR	Unambiguous	2	1	N-Term(iTRAQ4plex)	HCD	1.0000	0.0000	1	1	11872	Unique	Used	1.400	5.891E3	1.006E4	173	6.81	0.00	0	28	55	3	682.03595	2044.09330	-0.57	55.82	6672	6672	MS2	33/68	Glioma_Nuclear_prep_Gradeall_4plex.raw
238776833	NP_005773.3	ALYREF	10189	THO complex subunit 4 [Homo sapiens]	14.77	1	3	9	1.554	5;4	19.9	26.08	264	27.5	11.05																			
238776833		High	sLGTADVHFER	Unambiguous	1	1	N-Term(iTRAQ4plex)	HCD	1.0000	0.0000	1	1	8685	Unique	Used	2.244	2.222E4	6.031E4	169	3.65	0.00	0	8	18	3	459.24280	1375.71384	3.74	30.52	3048	3048	MS2	26/40	Glioma_Nuclear_prep_Gradeall_repeat_4plex.raw
238776833		High	sLGTADVHFER	Unambiguous	1	1	N-Term(iTRAQ4plex)	HCD	1.0000	0.0000	1	1	8690	Unique	Used	2.140	1.913E4	4.952E4	173	4.66	0.00	0	15	94	2	688.36078	1375.71428	4.06	30.56	3053	3053	MS2	19/20	Glioma_Nuclear_prep_Gradeall_repeat_4plex.raw
238776833		High	qQLSAEELDAQLDAYNAR	Unambiguous	1	1	N-Term(iTRAQ4plex)	HCD	1.0000	0.0000	1	1	11182	Unique	Used	1.583	1.267E3	2.425E3	159	6.78	0.00	0	0	300	2	1090.03955	2179.07182	-1.38	59.56	5741	5741	MS2	30/34	Glioma_Nuclear_prep_Gradeall_repeat_4plex.raw
238776833		High	qQLSAEELDAQLDAYNAR	Unambiguous	1	1	N-Term(iTRAQ4plex)	HCD	1.0000	0.0000	1	1	11169	Unique	Used	1.377	2.866E2	4.776E2	185	7.29	0.00	0	23	300	3	727.03070	2179.07755	1.25	59.39	5726	5726	MS2	28/68	Glioma_Nuclear_prep_Gradeall_repeat_4plex.raw
238776833	High		sLGTADVHFER	Unambiguous	1	1	N-Term(iTRAQ4plex)	HCD	1.0000	0.0000	1	1	8261	Unique	Used	1.554	1.126E5	2.135E5	147	3.48	0.00	0	15	3	3	459.23291	1375.68418	-17.82	28.21	2736	2736	MS2	26/40	Glioma_Nuclear_prep_Gradeall_4plex.raw
238776833	High		qQLSAEELDAQLDAYNAR	Unambiguous	1	1	N-Term(iTRAQ4plex)	HCD	1.0000	0.0000	1	1	10436	Unique	Used	1.506	6.429E4	1.181E5	120	6.54	0.00	0	7	13	2	1090.02344	2179.03960	-16.17	44.14	5106	5106	MS2	29/34	Glioma_Nuclear_prep_Gradeall_4plex.raw
238776833	High		sLGTADVHFER	Unambiguous	1	1	N-Term(iTRAQ4plex)	HCD	1.0000	0.0000	1	1	8267	Unique	Used	1.465	1.829E5	3.270E5	161	4.36	0.00	0	22	11	2	688.34644	1375.68559	-16.79	28.24	2742	2742	MS2	19/20	Glioma_Nuclear_prep_Gradeall_4plex.raw
238776833	High		mDMSLDDIIk	Unambiguous	1	1	N-Term(iTRAQ4plex); K10(iTRAQ4plex)	HCD	1.0000	0.0000	1	1	10858	Unique	Used	1.295	3.962E5	6.258E5	277	3.63	0.00	0	61	7	2	734.88599	1468.76470	1.14	47.36	5567	5567	MS2	17/18	Glioma_Nuclear_prep_Gradeall_4plex.raw
238776833	High		mDMSLDDIIk	Unambiguous	1	1	N-Term(iTRAQ4plex); M1(Oxidation); K10(iTRAQ4plex)	HCD	1.0000	0.0000	1	1	10145	Unique	Used	1.760	5.782E4	1.241E5	150	3.03	0.00	0	50	29	2	742.87146	1484.73564	-15.02	41.93	4788	4788	MS2	18/18	Glioma_Nuclear_prep_Gradeall_4plex.raw
291621647	NP_006311.2	PGRMC2	10424	membrane-associated progesterone receptor component 2 [Homo sapiens]	20.24	1	3	5	1.598	2;3	8.6	18.49	247	26.2	5.31																			
291621647		High	dFSLEQLR	Unambiguous	1	1	N-Term(iTRAQ4plex)	HCD	1.0000	0.0000	1	1	10353	Unique	Used	1.639	1.012E4	2.006E4	256	3.17	0.00	0	27	300	2	576.31323	1151.61919	1.25	49.29	4832	4832	MS2	14/14	Glioma_Nuclear_prep_Gradeall_repeat_4plex.raw
291621647		High	fYGPAGPYGIFAGR	Unambiguous	1	1	N-Term(iTRAQ4plex)	HCD	1.0000	0.0000	1	1	11980	Unique	Used	1.598	2.620E3	5.065E3	252	3.87	0.00	0	50	300	2	808.92218	1616.83708	1.76	71.67	6752	6752	MS2	17/26	Glioma_Nuclear_prep_Gradeall_repeat_4plex.raw
291621647		High	gLGAGAGAGEESPATSLPR	Unambiguous	1	1	N-Term(iTRAQ4plex)	HCD	1.0000	0.0000	1	1	9198	Unique	Used	1.370	1.772E3	2.936E3	133	6.65	0.00	0	11	300	2	921.47998	1841.95268	2.83	35.94	3592	3592	MS2	31/36	Glioma_Nuclear_prep_Gradeall_repeat_4plex.raw
291621647	High		gLGAGAGAGEESPATSLPR	Unambiguous	1	1	N-Term(iTRAQ4plex)	HCD	1.0000	0.0000	1	1	8771	Unique	Used	1.721	3.232E4	6.785E4	102	6.21	0.00	0	0	18	2	921.46375	1841.92021	-14.80	31.87	3290	3290	MS2	31/36	Glioma_Nuclear_prep_Gradeall_4plex.raw
291621647	High		fYGPAGPYGIFAGR	Unambiguous	1	1	N-Term(iTRAQ4plex)	HCD	1.0000	0.0000	1	1	11216	Unique	Used	1.505	5.364E4	9.850E4	252	4.78	0.00	0	30	53	2	808.91980	1616.83232	-1.18	50.15	5957	5957	MS2	21/26	Glioma_Nuclear_prep_Gradeall_4plex.raw
302129652	NP_001180473.1	ANXA6	309	annexin A6 isoform 2 [Homo sapiens]	14.35	2	6	10	1.828	7;3	38.0	45.45	641	72.4	5.60																			
302129652		High	aLIEILATR	Unambiguous	2	1	N-Term(iTRAQ4plex)	HCD	1.0000	0.0000	1	1	11871	Unique	Used	3.228	1.168E3	4.563E3	93	3.10	0.00	0	29	300	2	572.36462	1143.72197	0.13	69.77	6599	6599	MS2	15/16	Glioma_Nuclear_prep_Gradeall_repeat_4plex.raw
302129652		High	eAILDIITSR	Unambiguous	2	1	N-Term(iTRAQ4plex)	HCD	1.0000	0.0000	1	1	11938	Unique	Used	1.861	1.128E3	2.539E3	324	3.33	0.00	0	47	300	2	637.87555	1274.74382	0.11	70.87	6689	6689	MS2	16/18	Glioma_Nuclear_prep_Gradeall_repeat_4plex.raw
302129652	High		iLISLATGHR	Unambiguous	2	1	N-Term(iTRAQ4plex)	HCD	1.0000	0.0000	1	1	9420	Unique	Used	3.696	1.722E3	7.768E3	137	4.13	0.00	0	9	39	3	408.91583	1224.73294	-17.61	36.65	3999	3999	MS2	22/36	Glioma_Nuclear_prep_Gradeall_4plex.raw
302129652	High		aLIEILATR	Unambiguous	2	1	N-Term(iTRAQ4plex)	HCD	1.0000	0.0000	1	1	11018	Unique	Used	2.971	2.733E4	9.908E4	137	3.28	0.00	0	20	18	2	572.36298	1143.71868	-2.75	48.58	5740	5740	MS2	15/16	Glioma_Nuclear_prep_Gradeall_4plex.raw
302129652	High		eAILDIITSR	Unambiguous	2	1	N-Term(iTRAQ4plex)	HCD	1.0000	0.0000	1	1	11179	Unique	Used	2.756	3.911E4	1.315E5	224	3.48	0.00	0	31	15	2	637.87250	1274.73772	-4.68	49.87	5917	5917	MS2	16/18	Glioma_Nuclear_prep_Gradeall_4plex.raw
302129652	High		aLIEILATR	Unambiguous	2	1	N-Term(iTRAQ4plex)	HCD	1.0000	0.0000	1	1	10974	Unique	Used	1.795	1.131E4	2.477E4	186	3.36	0.00	0	57	97	2	572.35565	1143.70403	-15.56	48.24	5692	5692	MS2	15/16	Glioma_Nuclear_prep_Gradeall_4plex.raw
302129652	High		gLGTDEDTIIDIITHR	Unambiguous	2	1	N-Term(iTRAQ4plex)	HCD	1.0000	0.0000	1	1	12389	Unique	Used	1.575	1.186E3	2.279E3	148	7.08	0.00	0	9	66	3	638.34082	1913.00791	-0.94	61.03	7240	7240	MS2	29/60	Glioma_Nuclear_prep_Gradeall_4plex.raw
302129652	High		gLGTDEDTIIDIITHR	Unambiguous	2	1	N-Term(iTRAQ4plex)	HCD	1.0000	0.0000	1	1	12331	Unique	Used	1.313	9.266E2	1.485E3	169	6.66	0.00	0	36	226	3	638.34045	1913.00681	-1.52	60.37	7176	7176	MS2	27/60	Glioma_Nuclear_prep_Gradeall_4plex.raw
302129652	High		IIVGLMRPPAYcDAk	Unambiguous	2	1	N-Term(iTRAQ4plex); C12(Methylthio); K15(iTRAQ4plex)	HCD	1.0000	0.0000	1	1	11160	Unique	Used	1.668	6.246E3	1.271E4	161	3.43	0.00	0	28	54	3	661.02423	1981.05814	-4.78	49.72	5896	5896	MS2	16/56	Glioma_Nuclear_prep_Gradeall_4plex.raw

302129652		High	vFQEFIk	Unambiguous	2	1	N-Term(iTRAQ4plex); K7(iTRAQ4plex)	HCD	1.0000	0.0000	1	1	10878	Unique	Used	1.463	3.929E3	6.953E3	253	2.83	0.00	0	69	300	2	599.85590	1198.70452	-2.45	55.64	5398	5398	MS2	12/12	Glioma_Nuclear_prep_GradeII_repeat_4plex.raw
344925834	NP_001230710.1	FAM213A	84293	redox-regulatory protein FAM213A isoform 2 precursor [Homo sapiens]	21.10	3	4	8	2.140	7;1	30.7	33.29	218	24.4	9.01																			
344925834	High		vNLLSVLEAAk	Unambiguous	3	1	N-Term(iTRAQ4plex); K11(iTRAQ4plex)	HCD	1.0000	0.0000	1	1	12480	Unique	Used	3.803	8.709E2	4.041E3	146	3.98	0.00	0	55	103	3	482.29849	1444.88092	-11.67	62.07	7341	7341	MS2	20/40	Glioma_Nuclear_prep_GradeII_4plex.raw
344925834	High		vNLLSVLEAAk	Unambiguous	3	1	N-Term(iTRAQ4plex); K11(iTRAQ4plex)	HCD	1.0000	0.0000	1	1	12424	Unique	Used	2.700	1.022E3	3.367E3	120	4.58	0.00	0	24	300	3	482.30249	1444.89292	-3.37	61.41	7278	7278	MS2	24/40	Glioma_Nuclear_prep_GradeII_4plex.raw
344925834	High		vNLLSVLEAAk	Unambiguous	3	1	N-Term(iTRAQ4plex); K11(iTRAQ4plex)	HCD	1.0000	0.0000	1	1	12428	Unique	Used	2.072	1.395E4	3.526E4	125	4.88	0.00	0	60	255	2	722.95276	1444.89824	0.31	61.46	7283	7283	MS2	19/20	Glioma_Nuclear_prep_GradeII_4plex.raw
344925834	High		vNLLSVLEAAk	Unambiguous	3	1	N-Term(iTRAQ4plex); K11(iTRAQ4plex)	HCD	1.0000	0.0000	1	1	12487	Unique	Used	1.996	7.172E3	1.747E4	157	4.80	0.00	0	55	300	2	722.94482	1444.88237	-10.67	62.16	7349	7349	MS2	18/20	Glioma_Nuclear_prep_GradeII_4plex.raw
344925834	High		aALEYLEDIDLk	Unambiguous	3	1	N-Term(iTRAQ4plex); K12(iTRAQ4plex)	HCD	1.0000	0.0000	1	1	11869	Unique	Used	2.299	3.365E4	9.437E4	248	5.65	0.00	0	28	73	2	840.96942	1680.93157	1.00	55.80	6669	6669	MS2	21/22	Glioma_Nuclear_prep_GradeII_4plex.raw
344925834	High		aALEYLEDIDLk	Unambiguous	3	1	N-Term(iTRAQ4plex); K12(iTRAQ4plex)	HCD	1.0000	0.0000	1	1	11853	Unique	Used	1.439	1.080E3	1.895E3	248	3.63	0.00	0	42	241	3	560.98016	1680.92594	-2.34	55.65	6651	6651	MS2	16/44	Glioma_Nuclear_prep_GradeII_4plex.raw
344925834	High		sMLDQLGVPLYAVVk	Unambiguous	3	1	N-Term(iTRAQ4plex); K15(iTRAQ4plex)	HCD	1.0000	0.0000	1	1	12371	Unique	Used	1.879	1.327E3	3.041E3	128	5.77	0.00	0	38	173	3	641.04022	1921.10611	-0.54	60.82	7220	7220	MS2	21/56	Glioma_Nuclear_prep_GradeII_4plex.raw
344925834		High	gEIFLDEk	Unambiguous	3	1	N-Term(iTRAQ4plex); K8(iTRAQ4plex)	HCD	1.0000	0.0000	1	1	9504	Unique	Used	2.209	4.989E3	1.333E4	293	3.38	0.00	0	0	300	2	619.84381	1238.68035	-5.47	39.30	3917	3917	MS2	14/14	Glioma_Nuclear_prep_GradeII_repeat_4plex.raw
378404908	NP_001243728.1	GAPDH	2597	glyceraldehyde-3-phosphate dehydrogenase isoform 2 [Homo sapiens]	9.90	2	2	4	1.875	2;2	7.4	9.94	293	31.5	7.61																			
378404908		High	vPTANVSVVDLTcR	Unambiguous	2	1	N-Term(iTRAQ4plex); C13(Methylthio)	HCD	1.0000	0.0000	1	1	11641	Unique	Used	1.628	3.330E3	6.560E3	216	4.67	0.00	0	22	300	2	832.43585	1663.86443	0.95	66.12	6297	6297	MS2	17/26	Glioma_Nuclear_prep_GradeII_repeat_4plex.raw
378404908	High		vPTANVSVVDLTcR	Unambiguous	2	1	N-Term(iTRAQ4plex); C13(Methylthio)	HCD	1.0000	0.0000	1	1	10846	Unique	Used	1.918	8.852E4	2.072E5	110	4.43	0.00	0	14	12	2	832.42334	1663.83940	-14.09	47.27	5554	5554	MS2	17/26	Glioma_Nuclear_prep_GradeII_4plex.raw
378404908		High	gALQNIIPASTGAAk	Unambiguous	2	1	N-Term(iTRAQ4plex); K15(iTRAQ4plex)	HCD	1.0000	0.0000	1	1	10127	Unique	Used	1.846	2.585E3	5.771E3	142	5.27	0.00	0	37	300	2	850.50165	1699.99602	0.86	46.61	4591	4591	MS2	19/28	Glioma_Nuclear_prep_GradeII_repeat_4plex.raw
378404908	High		gALQNIIPASTGAAk	Unambiguous	2	1	N-Term(iTRAQ4plex); K15(iTRAQ4plex)	HCD	1.0000	0.0000	1	1	9476	Unique	Used	1.904	5.659E4	1.315E5	195	4.56	0.00	0	60	19	2	850.48730	1699.96733	-16.01	37.08	4061	4061	MS2	19/28	Glioma_Nuclear_prep_GradeII_4plex.raw
379317177	NP_001243839.1	DDX21	9188	nucleolar RNA helicase 2 isoform 2 [Homo sapiens]	11.75	2	5	8	1.788	7;1	14.0	31.54	715	79.6	9.38																			
379317177		High	aPQVLVLAPTR	Unambiguous	2	1	N-Term(iTRAQ4plex)	HCD	1.0000	0.0000	1	1	10112	Unique	Used	1.781	8.087E3	1.743E4	165	4.06	0.00	0	51	286	2	654.90997	1308.81267	0.48	46.43	4575	4575	MS2	16/20	Glioma_Nuclear_prep_GradeII_repeat_4plex.raw
379317177	High		gAVEALAAALAHISGATSVDQR	Unambiguous	2	1	N-Term(iTRAQ4plex)	HCD	1.0000	0.0000	1	1	12975	Unique	Used	2.113	1.700E2	4.383E2	109	5.36	0.00	0	80	300	3	751.40997	2252.21537	1.66	69.44	7957	7957	MS2	38/84	Glioma_Nuclear_prep_GradeII_4plex.raw
379317177	High		aPQVLVLAPTR	Unambiguous	2	1	N-Term(iTRAQ4plex)	HCD	1.0000	0.0000	1	1	9479	Unique	Used	1.422	4.427E4	7.681E4	204	3.70	0.00	0	58	31	2	654.89917	1308.79106	-16.03	37.09	4064	4064	MS2	15/20	Glioma_Nuclear_prep_GradeII_4plex.raw
379317177	High		gVTFLFPIQAk	Unambiguous	2	1	N-Term(iTRAQ4plex); K11(iTRAQ4plex)	HCD	1.0000	0.0000	1	1	11459	Unique	Used	1.974	9.840E3	2.370E4	166	5.08	0.00	0	49	90	2	754.95648	1508.90569	-1.50	52.26	6223	6223	MS2	16/20	Glioma_Nuclear_prep_GradeII_4plex.raw
379317177	High		tFSFAIPLIEk	Unambiguous	2	1	N-Term(iTRAQ4plex); K11(iTRAQ4plex)	HCD	1.0000	0.0000	1	1	12174	Unique	Used	1.943	1.637E4	3.881E4	126	3.85	0.00	0	30	143	2	777.46649	1553.92571	4.84	58.71	7003	7003	MS2	16/20	Glioma_Nuclear_prep_GradeII_4plex.raw
379317177	High		tFSFAIPLIEk	Unambiguous	2	1	N-Term(iTRAQ4plex); K11(iTRAQ4plex)	HCD	1.0000	0.0000	1	1	12244	Unique	Used	1.794	6.587E3	1.442E4	175	4.36	0.00	0	32	262	2	777.46283	1553.91838	0.13	59.43	7080	7080	MS2	16/20	Glioma_Nuclear_prep_GradeII_4plex.raw
379317177	High		gVTFLFPIQAk	Unambiguous	2	1	N-Term(iTRAQ4plex); K11(iTRAQ4plex)	HCD	1.0000	0.0000	1	1	11480	Unique	Used	1.666	3.050E3	6.199E3	169	4.11	0.00	0	24	56	3	503.64020	1508.90604	-1.27	52.43	6246	6246	MS2	19/40	Glioma_Nuclear_prep_GradeII_4plex.raw
379317177	High		lLDSVPPTAISHFk	Unambiguous	2	1	N-Term(iTRAQ4plex); K14(iTRAQ4plex)	HCD	1.0000	0.0000	1	1	10239	Unique	Used	1.448	7.144E3	1.262E4	203	5.06	0.00	0	38	23	3	605.01099	1813.01841	-15.36	42.64	4890	4890	MS2	29/52	Glioma_Nuclear_prep_GradeII_4plex.raw
380837121	NP_004883.3	SEC22B	9554	vesicle-trafficking protein SEC22b precursor [Homo sapiens]	14.42	1	2	3	0.555	2;1	10.7	11.16	215	24.7	8.51																			
380837121	High		iMVANIEEVLQR	Unambiguous	1	1	N-Term(iTRAQ4plex)	HCD	1.0000	0.0000	1	1	11835	Unique	Used	0.489	9.506E4	5.666E4	231	4.27	0.00	0	26	56	2	779.93945	1558.87163	-1.78	55.50	6632	6632	MS2	20/22	Glioma_Nuclear_prep_GradeII_4plex.raw
380837121	High		gEALSALDSk	Unambiguous	1	1	N-Term(iTRAQ4plex); K10(iTRAQ4plex)	HCD	1.0000	0.0000	1	1	8432	Unique	Used	0.555	1.984E5	1.344E5	302	3.67	0.00	0	11	7	2	639.84967	1278.69206	-17.47	29.42	2921	2921	MS2	18/18	Glioma_Nuclear_prep_GradeII_4plex.raw
380837121		High	imVANIEEVLQR	Unambiguous	1	1	N-Term(iTRAQ4plex); M2(Oxidation)	HCD	1.0000	0.0000	1	1	11943	Unique	Used	0.606	2.180E3	1.599E3	297	3.69	0.00	0	41	300	2	787.93982	1574.87236	1.93	71.02	6700	6700	MS2	18/22	Glioma_Nuclear_prep_GradeII_repeat_4plex.raw
386869506	NP_001247436.1	RPS3	6188	40S ribosomal protein S3 isoform 3 [Homo sapiens]	11.11	3	1	2	0.615	1;1	1.2	4.46	117	12.6	9.28																			
386869506		High	dEILPTTPISEQk	Unambiguous	3	1	N-Term(iTRAQ4plex); K13(iTRAQ4plex)	HCD	1.0000	0.0000	1	1	9888	Unique	Used	0.609	2.733E3	2.014E3	231	3.56	0.00	0	21	300	2	879.99219	1758.97710	2.44	43.62	4327	4327	MS2	15/24	Glioma_Nuclear_prep_GradeII_repeat_4plex.raw
386869506	High		dEILPTTPISEQk	Unambiguous	3	1	N-Term(iTRAQ4plex); K13(iTRAQ4plex)	HCD	1.0000	0.0000	1	1	9373	Unique	Used	0.620	5.367E4	4.059E4	225	4.46	0.00	0	20	29	2	879.97888	1758.95049	-12.69	36.31	3948	3948	MS2	16/24	Glioma_Nuclear_prep_GradeII_4plex.raw
395132436	NP_001257419.1	RPL18	6141	60S ribosomal protein L18 isoform 2 [Homo sapiens]	15.09	2	2	2	0.620	1;1	18.9	9.92	159	18.1	11.84																			
395132436	High		gcGTVLLSGPR	Unambiguous	2	1	N-Term(iTRAQ4plex); C2(Methylthio)	HCD	1.0000	0.0000	1	1	9619	Unique	Used	0.703	1.090E5	9.356E4	88	3.94	0.00	0	30	13	2	625.31921	1249.63115	-16.19	38.10	4216	4216	MS2	17/20	Glioma_Nuclear_prep_GradeII_4plex.raw
395132436		High	iLTFDQLALDSPk	Unambiguous	2	1	N-Term(iTRAQ4plex); K13(iTRAQ4plex)	HCD	1.0000	0.0000	1	1	12091	Unique	Used	0.537	3.269E2	2.125E2	162	3.74	0.00	0	37	300	3	583.67261	1749.00327	-0.26	74.07	6937	6937	MS2	17/48	Glioma_Nuclear_prep_GradeII_repeat_4plex.raw
402534522	NP_001257973.1	NFIX	4784	nuclear factor 1 X-type isoform 3 [Homo sapiens]	4.85	8	1	2	1.906	1;1	16.4	7.29	433	47.9	8.53																			
402534522		High	eDFVLTITGk	Unambiguous	3	1	N-Term(iTRAQ4plex); K10(iTRAQ4plex)	HCD	1.0000	0.0000	1	1	11337	Unique	Used	2.127	1.592E3	4.096E3	300	4.00	0.00	0	39	300	2	705.90833	1410.80937	0.76	61.94	5938	5938	MS2	16/18	Glioma_Nuclear_prep_GradeII_repeat_4plex.raw
402534522	High		eDFVLTITGk	Unambiguous	3	1	N-Term(iTRAQ4plex); K10(iTRAQ4plex)	HCD	1.0000	0.0000	1	1	10494	Unique	Used	1.684	7.093E4	1.457E5	281	4.01	0.00	0	33	21	2	705.89703	1410.78679	-15.24	44.60	5170	5170	MS2	15/18	Glioma_Nuclear_prep_GradeII_4plex.raw
404501455	NP_001258218.1	ZNF512	84450	zinc finger protein 512 isoform d [Homo sapiens]	18.61	4	5	7	1.570	2;5	19.4	22.27	462	53.1	9.60																			
404501455		High	qQPGIELPETELSLR	Unambiguous	4	1	N-Term(iTRAQ4plex)	HCD	1.0000	0.0000	1	1	11400	Unique	Used	2.216	1.434E3	3.844E3	185	4.33	0.00	0	11	300	2	927.50989	1854.01250	1.90	62.79	6012	6012	MS2	18/28	Glioma_Nuclear_prep_GradeII_repeat_4plex.raw
404501455		High	aGDEIDEPSER	Unambiguous	4	1	N-Term(iTRAQ4plex)	HCD	1.0000	0.0000	1	1	7399	Unique	Used	2.055	8.177E3	2.033E4	43	3.36	0.00	0	29	103	2	681.30762	1361.60796	-16.32	18.22	1689	1689	MS2	15/20	Glioma_Nuclear_prep_GradeII_repeat_4plex.raw
404501455		High	vLQDLVPDDR	Unambiguous	4	1	N-Term(iTRAQ4plex)	HCD	1.0000	0.0000	1	1	10548	Unique	Used	1.471	5.955E3	1.060E4	311	3.85	0.00	0	48	300	2	657.36371	1313.72014	1.47	51.61	5040	5040	MS2	15/18	Glioma_Nuclear_prep_GradeII_repeat_4plex.raw
404501455	High		aGDEIDEPSER	Unambiguous	4	1	N-Term(iTRAQ4plex)	HCD	1.0000	0.0000	1	1	7431	Unique	Used	1.861	3.694E4	8.386E4	60	3.79	0.00	0	41	31	2	681.31476	1361.62224	-5.83	22.32	1836	1836	MS2	14/20	Glioma_Nuclear_prep_GradeII_4plex.raw
404501455		High	sEHGPISFFPESGQPEcLk	Unambiguous	4	1	N-Term(iTRAQ4plex); C17(Methylthio); K19(iTRAQ4plex)	HCD	1.0000	0.0000	1	1	11713	Unique	Used	1.546	2.509E2	4.694E2	107	7.08	0.00	0	17	300	3	808.39331	2423.16538	1.62	67.15	6384	6384	MS2	29/72	Glioma_Nuclear_prep_GradeII_repeat_4plex.raw
404501455		High	ePEQEPVPAQFQk	Unambiguous	4	1	N-Term(iTRAQ4plex); K13(iTRAQ4plex)	HCD	1.0000	0.0000	1	1	9124	Unique	Used	1.302	1.022E3	1.610E3	169	3.65	0.00	0	46	300	3	605.65686	1814.95603	1.81	35.13	3514	3514	MS2	16/48	Glioma_Nuclear_prep_GradeII_repeat_4plex.raw
404501455	High		ePEQEPVPAQFQk	Unambiguous	4	1	N-Term(iTRAQ4plex); K13(iTRAQ4plex)	HCD	1.0000	0.0000	1	1	8701	Unique	Used	1.570	3.272E4	6.267E4	175	4.09	0.00	0	17	31	2	907.96735	1814.92742	-13.96	31.37	3214	3214	MS2	17/24	Glioma_Nuclear_prep_GradeII_4plex.raw
557440899	NP_001273490.1	NUMA1	4926	nuclear mitotic apparatus protein 1 isoform 2 [Homo sapiens]	21.85	3	24	32	0.642	19;13	15.5	126.70	2101	236.4	5.80																			
557440899		High	lEILQQQLQVANEAR	Unambiguous	2	1	N-Term(iTRAQ4plex)	HCD	1.0000	0.0000	1	1	11709	Unique	Used	0.483	1.756E3	1.025E3	183	5.59	0.00	0	18	300	2	949.03503	1897.06279	0.20	67.09	6379	6379	MS2	24/28	Glioma_Nuclear_prep_GradeII_repeat_4plex.raw
557440899		High	eLGELIPLR	Unambiguous	2	1	N-Term(iTRAQ4plex)	HCD	1.0000	0.0000	1	1	11503	Unique	Used	0.487	6.917E3	4.077E3	116	3.16	0.00	0	19	300	2	592.36176	1183.71623	-0.43	64.18	6132	6132	MS2	15/16	Glioma_Nuclear_prep_GradeII_repeat_4plex.raw
557440899		High	lQQLGEAHQAETEVLR	Unambiguous	2	1	N-Term(iTRAQ4plex)	HCD	1.0000	0.0000	1	1	9960	Unique	Used	0.523	1.763E3	1.116E3	207	5.05	0.00	0	15	300	3	656.02167	1966.05045	1.50	44.56	4408	4408	MS2	28/60	Glioma_Nuclear_prep_GradeII_repeat_4plex.raw
557440899		High	aADALEEQQR	Unambiguous	2	1	N-Term(iTRAQ4plex)	HCD	1.0000	0.0000	1	1	7418	Unique	Used	0.608	1.703E4	1.252E4	95	3.94	0.00	0	5	132	2	637.81580	1274.62432	-16.83	18.38	1709	1709	MS2	18/18	Glioma_Nuclear_prep_GradeII_repeat_4plex.raw
557440899		High	lLQAETASNSAR	Unambiguous	2	1	N-Term(iTRAQ4plex)	HCD	1.0000	0.0000	1	1	8209	Unique	Used	0.647	9.835E3	7.699E3	294	3.80	0.00	0	33	213	2	702.88330	1404.75932	2.10	25.82	2546	2546	MS2	18/22	Glioma_Nuclear_prep_GradeII_repeat_4plex.raw
557440899		High	qFLEVELDQAR	Unambiguous	2	1	N-Term(iTRAQ4plex)	HCD	1.0000	0.0000	1	1	11024	Unique	Used	0.682	2.589E3	2.137E3	258	4.24	0.00	0	14	300	2	746.40082	1491.79436	1.29	57.46	5559	5559	MS2	18/20	Glioma_Nuclear_prep_GradeII_repeat_4plex.raw
557440899		High	vESLESLYFTPIPAR	Unambiguous	2	1	N-Term(iTRAQ4plex)	HCD	1.0000	0.0000	1	1	12277	Unique	Used	0.701	1.133E3	9.610E2	212	5.62	0.00	0	6	300	2	933.51208	1866.01689	2.09	78.47	7273	7273	MS2	22/28	Glioma_Nuclear_prep_GradeII_repeat_4plex.raw
557440899	High		sQAPLESSLDSLGDVFLDSGR	Unambiguous	2	1	N-Term(iTRAQ4plex)	HCD	1.0000	0.0000	1	1	12871	Unique	Used	0.354	2.633E3	1.136E3	126	5.81	0.00	0	7	300	2	1169.08862	2337.16997	0.35	67.52	7808	7808	MS2	30/40	Glioma_Nuclear_prep_GradeII_4plex.raw
557440899	High		nSLISSLEEEVSILNR	Unambiguous	2	1	N-Term(iTRAQ4plex)	HCD	1.0000	0.0000	1	1	13073	Unique	Used	0.507	1.726E2	1.067E2	184	4.47	0.00	0	30	300	3	649.68872	1947.05161	0.02	71.78	8131	8131	MS2	23/60	Glioma_Nuclear_prep_GradeII_4plex.raw
557440899	High		lEILQQQLQVANEAR	Unambiguous	2	1	N-Term(iTRAQ4plex)	HCD	1.0000	0.0000	1	1	10931	Unique	Used	0.553	6.504E4	4.392E4	244	5.69	0.00	0	16	25	2	949.02124	1897.03520	-14.34	47.92	5646	5646	MS2	24/28	Glioma_Nuclear_prep_GradeII_4plex.raw
557440899	High		vESLESLYFTPIPAR	Unambiguous	2	1	N-Term(iTRAQ4plex)	HCD	1.0000	0.0000	1	1	11771	Unique	Used	0.563	4.099E4	2.818E4	222	6.19	0.00	0	19	72	2	933.51093	1866.01457	0.85	54.95	6562	6562	MS2	22/28	Glioma_Nuclear_prep_GradeII_4plex.raw
557440899	High		nSLISSLEEEVSILNR	Unambiguous	2	1	N-Term(iTRAQ4plex)	HCD	1.0000	0.0000	1	1	13075	Unique	Used	0.573	9.954E2	6.953E2	193	4.97	0.00	0	14	300	2	974.02832	1947.04936	-1.14	71.82	8134	8134	MS2	24/30	Glioma_Nuclear_prep_GradeII_4plex.raw
557440899	High		aQELGHSQSALASAQR	Unambiguous	2	1	N-Term(iTRAQ4plex)	HCD	1.0000	0.0000	1	1	7622	Unique	Used	0.582	3.822E4	2.713E4	123	5.57	0.00	0	7	18	2	899.46930	1797.93132	-0.63	23.69	2043	2043	MS2	26/30	Glioma_Nuclear_prep_GradeII_4plex.raw
557440899	High		qFLEVELDQAR	Unambiguous	2	1	N-Term(iTRAQ4plex)	HCD	1.0000	0.0000	1	1	10266	Unique	Used	0.702	7.406E4	6.346E4	207	4.08	0.00	0	32	20	2	746.38855	1491.76982	-15.16	42.83	4919	4919	MS2	18/20	Glioma_Nuclear_prep_GradeII_4plex.raw
557440899	High		aQELGHSQSALASAQR	Unambiguous	2	1	N-Term(iTRAQ4plex)	HCD	1.0000	0.0000	1	1	7616	Unique	Used	0.722	2.893E4	2.549E4	146	4.46	0.00	0	40	5	3	599.98163	1797.93033	-1.18	23.65	2037	2037	MS2	24/60	Glioma_Nuclear_prep_GradeII_4plex.raw
557440899	High		eLAEAMAAQHTAESEcEQLVk	Unambiguous	2	1	N-Term(iTRAQ4plex); C16(Methylthio); K21(iTRAQ4plex)	HCD	1.0000	0.0000	1	1	12157	Unique	Used	0.739	6.243E2	5.629E2	113	4.76	0.00	0	34	135	4	656.31763	2622.24868	1.29	58.54	6984	6984	MS2	19/120	Glioma_Nuclear_prep_GradeII_4plex.raw
557440899		High	lEQQcQk	Unambiguous	3	1	N-Term(iTRAQ4plex); C5(Methylthio); K7(iTRAQ4plex)	HCD	1.0000	0.0000	1	1	7932	Unique	Used	0.642	1.864E4	1.448E4	210	2.90	0.00	0	58	300	2	605.81036	1210.61345	-2.32	23.11	2254	2254	MS2	12/12	Glioma_Nuclear_prep_GradeII_repeat_4plex.raw
557440899	High		kQQNQELQEQLR	Unambiguous	2	1	N-Term(iTRAQ4plex); K1(iTRAQ4plex)	HCD	1.0000	0.0000	1	1	7505	Unique	Used	0.652	1.551E4	1.233E4	140	3.93	0.00	1	26	13	3	610.67145	1829.99979	-4.06	22.85	1916	1916	MS2	28/44	Glioma_Nuclear_prep_GradeII_4plex.raw
557440899		High	kVEELQAcVETAR	Unambiguous	2	1	N-Term(iTRAQ4plex); K1(iTRAQ4plex); C8(Methylthio)	HCD	1.0000	0.0000	1	1	10607	Unique	Used	0.723	1.475E3	1.290E3	165	2.85	0.00	1	38	300	2	905.47668	1809.94609	1.06	52.30	5103	5103	MS2	16/24	Glioma_Nuclear_prep_GradeII_repeat_4plex.raw
557440899		High	lTAQVEELSk	Unambiguous	1	1	N-Term(iTRAQ4plex); K10(iTRAQ4plex)	HCD	1.0000	0.0000	1	1	9678	Unique	Used	0.605	8.496E3	6.220E3	281	4.63	0.00	0	26	300	2	703.41138	1405.81548	0.97	41.28	4106	4106	MS2	18/18	Glioma_Nuclear_prep_GradeII_repeat_4plex.raw
557440899	High		lTAQVEELSk	Unambiguous	1	1	N-Term(iTRAQ4plex); K10(iTRAQ4plex)	HCD	1.0000	0.0000	1	1	9149	Unique	Used	0.642	1.445E5	1.133E5	286	4.25	0.00	0	29	11	2	703.39795	1405.78862	-18.13	34.65	3703	3703	MS2	18/18	Glioma_Nuclear_prep_GradeII_4plex.raw
557440899		High	hQVEQLSSSLk	Unambiguous	2	1	N-Term(iTRAQ4plex); K11(iTRAQ4plex)	HCD	1.0000	0.0000	1	1	8179	Unique	Used	0.716	5.961E3	5.161E3	231	3.45	0.00	0	11	300	2	772.43463	1543.86199	-4.06	25.50	2514	2514	MS2	17/20	Glioma_Nuclear_prep_GradeII_repeat_4plex.raw
557440899	High		lADDLSTLQEk	Unambiguous	2	1	N-Term(iTRAQ4plex); K11(iTRAQ4plex)	HCD	1.0000	0.0000	1	1	9718	Unique	Used	0.607	1.335E5	9.884E4	227	4.14	0.00	0	63	17	2	760.91083	1520.81438	-17.54	38.81	4323	4323	MS2	20/20	Glioma_Nuclear_prep_GradeII_4plex.raw
557440899	High		hQVEQLSSSLk	Unambiguous	2	1	N-Term(iTRAQ4plex); K11(iTRAQ4plex)	HCD	1.0000	0.0000	1	1	7904	Unique	Used	0.652	1.407E5	1.119E5	204	3.26	0.00	0	40	16	2	772.43787	1543.86846	0.13	25.69	2349	2349	MS2	16/20	Glioma_Nuclear_prep_GradeII_4plex.raw
557440899	High		aSYAEQLSMLk	Unambiguous	2	1	N-Term(iTRAQ4plex); K11(iTRAQ4plex)	HCD	1.0000	0.0000	1	1	10041	Unique	Used	0.767	9.251E4	8.660E4	224	3.82	0.00	0	54	23	2	764.90588	1528.80449	-15.62	41.15	4674	4674	MS2	18/20	Glioma_Nuclear_prep_GradeII_4plex.raw
557440899	High		gEVLGDVLQLETLk	Unambiguous	2	1	N-Term(iTRAQ4plex); K14(iTRAQ4plex)	HCD	1.0000	0.0000	1	1	12026	Unique	Used	0.766	2.827E4	2.642E4	148	5.71	0.00	0	18	94	2	901.52893	1802.05058	-0.46	57.25	6841	6841	MS2	23/26	Glioma_Nuclear_prep_GradeII_4plex.raw
557440899	High		vEFATLQEALAHALTEk	Unambiguous	2	1	N-Term(iTRAQ4plex); K17(iTRAQ4plex)	HCD	1.0000	0.0000	1	1	12875	Unique	Used	0.565	2.722E3	1.876E3	106	7.64	0.00	0	10	115	3	720.40424	2159.19815	1.41	67.60	7814	7814	MS2	38/64	Glioma_Nuclear_prep_GradeII_4plex.raw
557440899	High		vEFATLQEALAHALTEk	Unambiguous	2	1	N-Term(iTRAQ4plex); K17(iTRAQ4plex)	HCD	1.0000	0.0000	1	1	12834	Unique	Used	0.624	1.893E3	1.442E3	101	7.10	0.00	0	15	223	3	720.40369	2159.19651	0.64	66.90	7758	7758	MS2	37/64	Glioma_Nuclear_prep_GradeII_4plex.raw
557440899		High	sLEQLQk	Unambiguous	2	1	N-Term(iTRAQ4plex); K7(iTRAQ4plex)	HCD	1.0000	0.0000	1	1	8177	Unique	Used	0.747	2.026E4	1.831E4	408	3.21	0.00	0	79	300	2	567.33771	1133.66814	-7.70	25.48	2512	2512	MS2	12/12	Glioma_Nuclear_prep_GradeII_repeat_4plex.raw
557440899		High	gLEAAQIk	Unambiguous	2	1	N-Term(iTRAQ4plex); K8(iTRAQ4plex)	HCD	1.0000	0.0000	1	1	8155	Unique	Used	0.761	2.640E4	2.430E4	319	3.29	0.00	0	63	172	2	559.34167	1117.67607	-5.27	25.27	2489	2489	MS2	14/14	Glioma_Nuclear_prep_GradeII_repeat_4plex.raw
557440899	High		fQVATDALk	Unambiguous	2	1	N-Term(iTRAQ4plex); K9(iTRAQ4plex)	HCD	1.0000	0.0000	1	1	9147	Unique	Used	0.594	6.752E5	4.896E5	278	3.29	0.00	0	40	5	2	640.86487	1280.72246	-17.83	34.64	3701	3701	MS2	16/16	Glioma_Nuclear_prep_GradeII_4plex.raw
557440899	High		aEELGQELk	Unambiguous	2	1	N-Term(iTRAQ4plex); K9(iTRAQ4plex)	HCD	1.0000	0.0000	1	1	8349	Unique	Used	0.734	1.423E5	1.274E5	227	3.24	0.00	0	52	12	2	652.85791	1304.70854	-16.48	28.83	2831	2831	MS2	16/16	Glioma_Nuclear_prep_GradeII_4plex.raw
584277111	NP_001276868.1	SLC1A3	6507	excitatory amino acid transporter 1 isoform 4 [Homo sapiens]	5.04	6	2	6	2.238	5;1	27.2	22.01	496	54.2	7.44																			
584277111		High	vTAADAFLDLIR	Unambiguous	4	1	N-Term(iTRAQ4plex)	HCD	1.0000	0.0000	1	1	12473	Unique	Used	2.884	3.394E2	1.184E3	232	4.15	0.00	0	13	300	2	724.91437	1448.82146	-1.07	92.52	8228	8228	MS2	19/22	Glioma_Nuclear_prep_GradeII_repeat_4plex.raw
584277111	High		vTAADAFLDLIR	Unambiguous	4	1	N-Term(iTRAQ4plex)	HCD	1.0000	0.0000	1	1	12729	Unique	Used	3.209	1.416E4	5.542E4	229	4.91	0.00	0	9	82	2	724.91608	1448.82488	1.29	65.37	7630	7630	MS2	21/22	Glioma_Nuclear_prep_GradeII_4plex.raw
584277111	High		vTAADAFLDLIR	Unambiguous	4	1	N-Term(iTRAQ4plex)	HCD	1.0000	0.0000	1	1	12777	Unique	Used	2.433	4.526E3	1.343E4	239	5.11	0.00	0	31	300	2	724.91602	1448.82475	1.20	66.03	7687	7687	MS2	21/22	Glioma_Nuclear_prep_GradeII_4plex.raw
584277111	High		vTAADAFLDLIR	Unambiguous	4	1	N-Term(iTRAQ4plex)	HCD	1.0000	0.0000	1	1	12674	Unique	Used	1.768	3.554E3	7.666E3	332	4.55	0.00	0	67	300	2	724.91498	1448.82268	-0.23	64.67	7568	7568	MS2	20/22	Glioma_Nuclear_prep_GradeII_4plex.raw
584277111	High		vTAADAFLDLIR	Unambiguous	4	1	N-Term(iTRAQ4plex)	HCD	1.0000	0.0000	1	1	12767	Unique	Used	1.614	1.716E2	3.380E2	230	3.64	0.00	0	50	300	3	483.61221	1448.82209	-0.64	65.87	7674	7674	MS2	20/44	Glioma_Nuclear_prep_GradeII_4plex.raw
584277111	High		nMFPPNLVEAcFk	Unambiguous	5	1	N-Term(iTRAQ4plex); C11(Methylthio); K13(iTRAQ4plex)	HCD	1.0000	0.0000	1	1	12920	Unique	Used	2.044	1.912E3	4.769E3	170	3.79	0.00	0	59	300	2	922.46204	1843.91680	1.08	68.32	7872	7872	MS2	14/24	Glioma_Nuclear_prep_GradeII_4plex.raw
631790805	NP_001278830.1	SYT1	6857	synaptotagmin-1 isoform 2 [Homo sapiens]	7.40	4	2	2	0.548	1;1	14.2	7.43	419	47.2	7.94																			
631790805		High	vFVGYNSTGAELR	Unambiguous	2	1	N-Term(iTRAQ4plex)	HCD	1.0000	0.0000	1	1	10100	Unique	Used	0.603	7.777E3	5.676E3	267	4.29	0.00	0	29	300	2	778.91479	1556.82231	2.14	46.30	4562	4562	MS2	17/24	Glioma_Nuclear_prep_GradeII_repeat_4plex.raw
631790805	High		lGDIcFSLR	Unambiguous	4	1	N-Term(iTRAQ4plex); C5(Methylthio)	HCD	1.0000	0.0000	1	1	11994	Unique	Used	0.493	3.915E4	2.356E4	142	3.14	0.00	0	64	160	2	607.31238	1213.61748	-1.27	56.93	6806	6806	MS2	14/16	Glioma_Nuclear_prep_GradeII_4plex.raw
664806003	NP_001287744.1	CIRBP	1153	cold-inducible RNA-binding protein isoform 2 [Homo sapiens]	14.88	3	2	3	0.434	2;1	10.7	14.01	168	18.1	9.64																			
664806003	High		yGQISEVVVVk	Unambiguous	3	1	N-Term(iTRAQ4plex); K11(iTRAQ4plex)	HCD	1.0000	0.0000	1	1	9702	Unique	Used	0.434	1.640E5	8.685E4	240	3.96	0.00	0	52	22	2	754.94128	1508.87529	-11.53	38.70	4306	4306	MS2	19/20	Glioma_Nuclear_prep_GradeII_4plex.raw
664806003		High	gFGFVTFENIDDAk	Unambiguous	3	1	N-Term(iTRAQ4plex); K14(iTRAQ4plex)	HCD	1.0000	0.0000	1	1	12168	Unique	Used	0.457	2.554E3	1.413E3	184	5.21	0.00	0	35	300	2	924.47766	1847.94805	3.35	75.42	7045	7045	MS2	23/26	Glioma_Nuclear_prep_GradeII_repeat_4plex.raw
664806003	High		gFGFVTFENIDDAk	Unambiguous	3	1	N-Term(iTRAQ4plex); K14(iTRAQ4plex)	HCD	1.0000	0.0000	1	1	11512	Unique	Used	0.370	1.036E5	4.679E4	166	6.40	0.00	0	38	63	2	924.47278	1847.93828	-1.94	52.70	6281	6281	MS2	25/26	Glioma_Nuclear_prep_GradeII_4plex.raw
665821291	NP_001287919.1	PTBP2	58155	polypyrimidine tract-binding protein 2 isoform 7 [Homo sapiens]	1.67	7	1	2	3.041	1;1	2.3	3.35	480	52.3	8.97																			
665821291		High	vTNILMLk	Unambiguous	7	1	N-Term(iTRAQ4plex); K8(iTRAQ4plex)	HCD	1.0000	0.0000	1	1	11335	Unique	Used	2.992	3.795E3	1.374E4	160	3.35	0.00	0	49	300	2	610.38867	1219.77007	1.14	61.91	5936	5936	MS2	14/14	Glioma_Nuclear_prep_GradeII_repeat_4plex.raw
665821291	High		vTNILMLk	Unambiguous	7	1	N-Term(iTRAQ4plex); K8(iTRAQ4plex)	HCD	1.0000	0.0000	1	1	10434	Unique	Used	3.089	3.029E5	1.142E6	241	3.16	0.00	0	33	4	2	610.37848	1219.74968	-15.57	44.13	5104	5104	MS2	14/14	Glioma_Nuclear_prep_GradeII_4plex.raw
667743700	NP_001288139.1	MYEF2	50804	myelin expression factor 2 isoform b [Homo sapiens]	15.10	2	6	9	0.589	4;5	14.9	31.17	576	61.9	8.75																			
667743700		High	mGPGIGAILER	Unambiguous	2	1	N-Term(iTRAQ4plex)	HCD	1.0000	0.0000	1	1	11068	Unique	Used	0.453	1.640E4	8.996E3	262	3.24	0.00	0	28	300	2	629.36108	1257.71489	3.38	58.04	5609	5609	MS2	19/20	Glioma_Nuclear_prep_GradeII_repeat_4plex.raw
667743700		High	aEVPGATGGDSPHLQPAEPPGEPR	Unambiguous	2	1	N-Term(iTRAQ4plex)	HCD	1.0000	0.0000	1	1	9006	Unique	Used	0.589	2.173E3	1.549E3	69	5.23	0.00	0	10	161	3	837.42126	2510.24924	3.95	33.79	3389	3389	MS2	26/92	Glioma_Nuclear_prep_GradeII_repeat_4plex.raw
667743700	High		mGPGIGAILER	Unambiguous	2	1	N-Term(iTRAQ4plex)	HCD	1.0000	0.0000	1	1	10201	Unique	Used	0.455	2.237E5	1.241E5	231	3.27	0.00	0	28	19	2	629.35065	1257.69402	-13.21	42.36	4849	4849	MS2	19/20	Glioma_Nuclear_prep_GradeII_4plex.raw
667743700	High		aEVPGATGGDSPHLQPAEPPGEPR	Unambiguous	2	1	N-Term(iTRAQ4plex)	HCD	1.0000	0.0000	1	1	8613	Unique	Used	0.492	1.440E4	8.640E3	56	3.98	0.00	0	0	18	3	837.40521	2510.20108	-15.23	30.74	3119	3119	MS2	26/92	Glioma_Nuclear_prep_GradeII_4plex.raw
667743700	High		gFLSGPMGSGMR	Unambiguous	2	1	N-Term(iTRAQ4plex)	HCD	1.0000	0.0000	1	1	9599	Unique	Used	0.673	3.068E5	2.520E5	118	3.99	0.00	0	31	14	2	670.82251	1340.63774	-14.53	37.96	4194	4194	MS2	15/22	Glioma_Nuclear_prep_GradeII_4plex.raw
667743700		High	gcGVVEFk	Unambiguous	2	1	N-Term(iTRAQ4plex); C2(Methylthio); K8(iTRAQ4plex)	HCD	1.0000	0.0000	1	1	10083	Unique	Used	0.657	1.235E4	9.813E3	143	2.96	0.00	0	63	300	2	586.80756	1172.60784	2.72	46.10	4544	4544	MS2	14/14	Glioma_Nuclear_prep_GradeII_repeat_4plex.raw
667743700		High	rEPHPAEAEk	Unambiguous	2	1	N-Term(iTRAQ4plex); K10(iTRAQ4plex)	HCD	1.0000	0.0000	1	1	6509	Unique	Used	0.638	3.502E3	2.704E3	262	4.07	0.00	1	55	162	4	363.70331	1451.79140	4.72	10.34	750	750	MS2	17/54	Glioma_Nuclear_prep_GradeII_repeat_4plex.raw
667743700		High	vGEVTYVELFk	Unambiguous	2	1	N-Term(iTRAQ4plex); K11(iTRAQ4plex)	HCD	1.0000	0.0000	1	1	11894	Unique	Used	0.593	4.970E3	3.568E3	211	4.57	0.00	0	27	300	2	786.45331	1571.89934	4.44	70.12	6628	6628	MS2	18/20	Glioma_Nuclear_prep_GradeII_repeat_4plex.raw
667743700	High		vGEVTYVELFk	Unambiguous	2	1	N-Term(iTRAQ4plex); K11(iTRAQ4plex)	HCD	1.0000	0.0000	1	1	11084	Unique	Used	0.552	3.818E5	2.570E5	207	5.31	0.00	0	28	13	2	786.44916	1571.89104	-0.84	49.12	5813	5813	MS2	19/20	Glioma_Nuclear_prep_GradeII_4plex.raw
751368126	NP_001291434.1	HMG20A	10363	high mobility group protein 20A isoform b [Homo sapiens]	11.11	2	2	2	0.546	1;1	24.6	6.92	234	27.7	6.92																			
751368126		High	lEVDVIQER	Unambiguous	2	1	N-Term(iTRAQ4plex)	HCD	1.0000	0.0000	1	1	9869	Unique	Used	0.641	9.984E3	7.740E3	302	3.34	0.00	0	61	300	2	622.85181	1244.69634	-0.32	43.40	4307	4307	MS2	13/16	Glioma_Nuclear_prep_GradeII_repeat_4plex.raw
751368126	High		sVFDIPIFTEEFLNHSk	Unambiguous	2	1	N-Term(iTRAQ4plex); K17(iTRAQ4plex)	HCD	1.0000	0.0000	1	1	13023	Unique	Used	0.451	6.054E2	3.329E2	139	6.92	0.00	0	34	300	3	771.08203	2311.23154	4.42	70.34	8027	8027	MS2	34/64	Glioma_Nuclear_prep_GradeII_4plex.raw

Supplementary Table S5: Differentially expressed proteins in Grade IV astrocytoma (GBM). A total of 147 nuclear proteins (≥1.5 fold change) were identified after applying quality control filters as detailed in Methods section. The Table describes the total number of proteins and peptides identified in replicate mass spectrometry runs along with their quantity values.

Protein Accession	RefSeq id	Gene Symbol	Gene id	Protein name	Σ Coverage	Σ# Proteins	Σ# Unique Peptides	Σ# PSMs	117/114	117/114 Count	117/114 Variability [%]	Maximum Score	# AAs	MW [kDa]	calc. pI																			
Protein Group Accessions	A2	B2	Sequence	PSM Ambiguity	# Proteins	# Protein Groups	Modifications	Activation Type	ΔScore	ΔCn	Rank	Search Engine Rank	QuanResultID	Quan Info	Quan Usage	117/114	114	117	Peptides Matched	XCorr	Probability	# Missed Cleavages	Isolation Interference [%]	Ion Inject Time [ms]	Charge	m/z [Da]	MH+ [Da]	ΔM [ppm]	RT [min]	First Scan	Last Scan	MS Order	Ions Matched	Spectrum File
4502027	NP_000468.1	ALB	213	serum albumin preproprotein [Homo sapiens]	23.32	1	11	18	2.684	7;11	39.8	46.84	609	69.3	6.28																			
4502027		High	fQNALLVR	Unambiguous	1	1	N-Term(iTRAQ4plex)	HCD	1.0000	0.0000	1	1	10037	Unique	Used	2.504	4.349E3	1.475E4	360	3.27	0.00	0	37	300	2	552.83624	1104.66521	0.52	45.50	4492	4492	MS2	14/14	Glioma_Nuclear_prep_Gradeall_repeat_4plex.raw
4502027		High	tcVADESAENcDk	Unambiguous	1	1	N-Term(iTRAQ4plex);	HCD	1.0000	0.0000	1	1	9004	Unique	Used	4.583	1.109E3	6.883E3	16	4.91	0.00	0	18	300	2	882.86334	1764.71941	2.41	33.75	3386	3386	MS2	23/24	Glioma_Nuclear_prep_Gradeall_repeat_4plex.raw
4502027	High		yIcENQDSISSk	Unambiguous	1	1	N-Term(iTRAQ4plex);	HCD	1.0000	0.0000	1	1	9141	Unique	Used	6.073	5.508E4	4.207E5	35	4.24	0.00	0	3	10	2	860.89545	1720.78362	-16.76	34.59	3694	3694	MS2	20/22	Glioma_Nuclear_prep_Gradeall_4plex.raw
4502027		High	yIcENQDSISSk	Unambiguous	1	1	N-Term(iTRAQ4plex);	HCD	1.0000	0.0000	1	1	9609	Unique	Used	4.720	2.815E3	1.800E4	62	5.08	0.00	0	19	300	2	860.90955	1720.81182	-0.37	40.50	4033	4033	MS2	19/22	Glioma_Nuclear_prep_Gradeall_repeat_4plex.raw
4502027		High	nEcFLQHk	Unambiguous	1	1	N-Term(iTRAQ4plex);	HCD	1.0000	0.0000	1	1	8637	Unique	Used	2.639	1.902E3	6.799E3	149	3.53	0.00	0	39	210	3	451.56073	1352.66764	-1.27	30.01	2997	2997	MS2	19/28	Glioma_Nuclear_prep_Gradeall_repeat_4plex.raw
4502027	High		eTYGEMADccAk	Unambiguous	1	1	N-Term(iTRAQ4plex);	HCD	1.0000	0.0000	1	1	10220	Unique	Used	3.288	2.593E4	1.072E5	6	4.36	0.00	0	43	47	2	850.82495	1700.64263	-16.17	42.49	4869	4869	MS2	19/22	Glioma_Nuclear_prep_Gradeall_4plex.raw
4502027	High		lVNEVTEFAk	Unambiguous	1	1	N-Term(iTRAQ4plex);	HCD	1.0000	0.0000	1	1	10132	Unique	Used	3.992	1.621E5	8.139E5	237	4.01	0.00	0	33	4	2	719.40125	1437.79521	-16.68	41.84	4774	4774	MS2	17/18	Glioma_Nuclear_prep_Gradeall_4plex.raw
4502027		High	lVNEVTEFAk	Unambiguous	1	1	N-Term(iTRAQ4plex);	HCD	1.0000	0.0000	1	1	10924	Unique	Used	1.925	3.621E2	9.441E2	298	3.39	0.00	0	53	300	3	479.94406	1437.81763	-1.09	56.22	5449	5449	MS2	16/36	Glioma_Nuclear_prep_Gradeall_repeat_4plex.raw
4502027		High	lVNEVTEFAk	Unambiguous	1	1	N-Term(iTRAQ4plex);	HCD	1.0000	0.0000	1	1	10914	Unique	Used	3.280	3.308E3	1.470E4	278	4.44	0.00	0	24	300	2	719.41309	1437.81890	-0.21	56.11	5439	5439	MS2	18/18	Glioma_Nuclear_prep_Gradeall_repeat_4plex.raw
4502027	High		aVMDDFAAFVEk	Unambiguous	1	1	N-Term(iTRAQ4plex);	HCD	1.0000	0.0000	1	1	11832	Unique	Used	2.729	3.708E3	1.272E4	200	4.20	0.00	0	32	31	3	544.28186	1630.83103	-4.87	55.48	6629	6629	MS2	19/44	Glioma_Nuclear_prep_Gradeall_4plex.raw
4502027	High		vFDEFkPLVEEPQN	Unambiguous	1	1	N-Term(iTRAQ4plex);	HCD	1.0000	0.0000	1	1	11833	Unique	Used	3.619	3.837E3	1.747E4	72	6.38	0.00	0	29	29	3	826.46887	2477.39206	-3.87	55.48	6630	6630	MS2	28/64	Glioma_Nuclear_prep_Gradeall_4plex.raw
4502027		High	aEFAEVSk	Unambiguous	1	1	N-Term(iTRAQ4plex);	HCD	1.0000	0.0000	1	1	8298	Unique	Used	1.572	2.163E4	4.605E4	284	3.19	0.00	0	60	196	2	584.82880	1168.65032	4.34	26.68	2640	2640	MS2	14/14	Glioma_Nuclear_prep_Gradeall_repeat_4plex.raw
4502027	High		sLHTLFGDk	Unambiguous	1	1	N-Term(iTRAQ4plex);	HCD	1.0000	0.0000	1	1	8652	Unique	Used	1.819	6.819E4	1.560E5	279	3.39	0.00	0	35	21	2	653.36340	1305.71953	-16.09	31.02	3161	3161	MS2	15/16	Glioma_Nuclear_prep_Gradeall_4plex.raw
4502027	High		qTALVELVk	Unambiguous	1	1	N-Term(iTRAQ4plex);	HCD	1.0000	0.0000	1	1	9521	Unique	Used	2.036	1.484E5	3.800E5	196	3.89	0.00	0	36	7	2	644.89648	1288.78569	-17.23	37.40	4110	4110	MS2	15/16	Glioma_Nuclear_prep_Gradeall_4plex.raw
4502027		High	sLHTLFGDk	Unambiguous	1	1	N-Term(iTRAQ4plex);	HCD	1.0000	0.0000	1	1	9348	Unique	Used	1.930	5.666E3	1.481E4	354	3.69	0.00	0	27	270	2	653.37341	1305.73955	-0.76	37.59	3750	3750	MS2	16/16	Glioma_Nuclear_prep_Gradeall_repeat_4plex.raw
4502027		High	qTALVELVk	Unambiguous	1	1	N-Term(iTRAQ4plex);	HCD	1.0000	0.0000	1	1	10214	Unique	Used	2.240	8.136E3	2.469E4	177	3.86	0.00	0	39	286	2	644.90710	1288.80693	-0.75	47.63	4683	4683	MS2	15/16	Glioma_Nuclear_prep_Gradeall_repeat_4plex.raw
4502027		High	sLHTLFGDk	Unambiguous	1	1	N-Term(iTRAQ4plex);	HCD	1.0000	0.0000	1	1	9326	Unique	Used	2.830	1.514E3	5.802E3	323	3.86	0.00	0	22	147	3	435.91788	1305.73908	-1.12	37.37	3727	3727	MS2	16/32	Glioma_Nuclear_prep_Gradeall_repeat_4plex.raw
4502027		High	aVmDDFAAFVEk	Unambiguous	1	1	N-Term(iTRAQ4plex);	HCD	1.0000	0.0000	1	1	11406	Unique	Used	2.461	1.763E3	5.878E3	172	4.54	0.00	0	49	300	2	823.92010	1646.83293	-0.58	62.89	6020	6020	MS2	19/22	Glioma_Nuclear_prep_Gradeall_repeat_4plex.raw
4502101	NP_000691.1	ANXA1	301	annexin A1 [Homo sapiens]	19.36	1	4	5	3.894	3;2	36.1	27.74	346	38.7	7.02																			
4502101	High		gLGTDEDTLIEILASR	Unambiguous	1	1	N-Term(iTRAQ4plex)	HCD	1.0000	0.0000	1	1	12803	Unique	Used	3.894	8.657E2	4.240E3	170	5.67	0.00	0	13	300	2	924.00195	1846.99663	4.71	66.39	7717	7717	MS2	27/30	Glioma_Nuclear_prep_Gradeall_4plex.raw
4502101		High	aLYEAGER	Unambiguous	1	1	N-Term(iTRAQ4plex)	HCD	1.0000	0.0000	1	1	7682	Unique	Used	2.094	1.194E4	3.388E4	182	2.94	0.00	0	31	280	2	526.77203	1052.53679	-11.91	20.75	1991	1991	MS2	11/14	Glioma_Nuclear_prep_Gradeall_repeat_4plex.raw
4502101		High	sEDFGVNEDLADSDAR	Unambiguous	1	1	N-Term(iTRAQ4plex)	HCD	1.0000	0.0000	1	1	9697	Unique	Used	6.008	1.245E3	1.014E4	35	5.55	0.00	0	8	300	2	942.42340	1883.83953	1.00	41.49	4126	4126	MS2	28/30	Glioma_Nuclear_prep_Gradeall_repeat_4plex.raw
4502101	High		gVDEATIIDILTk	Unambiguous	1	1	N-Term(iTRAQ4plex); K13(iTRAQ4plex)	HCD	1.0000	0.0000	1	1	12469	Unique	Used	3.676	7.097E2	3.281E3	205	4.64	0.00	0	11	139	3	559.32861	1675.97129	-0.48	61.94	7329	7329	MS2	21/48	Glioma_Nuclear_prep_Gradeall_4plex.raw
4502101	High		gVDEATIIDILTk	Unambiguous	1	1	N-Term(iTRAQ4plex); K13(iTRAQ4plex)	HCD	1.0000	0.0000	1	1	12471	Unique	Used	5.201	5.285E3	3.457E4	212	5.64	0.00	0	24	275	2	838.49103	1675.97478	1.61	61.96	7331	7331	MS2	22/24	Glioma_Nuclear_prep_Gradeall_4plex.raw
4502107	NP_001145.1	ANXA5	308	annexin A5 [Homo sapiens]	22.19	1	6	8	2.963	5;3	38.4	30.13	320	35.9	5.05																			
4502107	High		sEIDLFNIR	Unambiguous	1	1	N-Term(iTRAQ4plex)	HCD	1.0000	0.0000	1	1	10747	Unique	Used	1.927	5.126E4	1.242E5	310	3.45	0.00	0	52	28	2	625.83740	1250.66753	-14.90	46.51	5446	5446	MS2	15/16	Glioma_Nuclear_prep_Gradeall_4plex.raw
4502107	High		gLGTDEESILTLLTSR	Unambiguous	1	1	N-Term(iTRAQ4plex)	HCD	1.0000	0.0000	1	1	12926	Unique	Used	3.592	1.040E3	4.699E3	153	6.32	0.00	0	22	300	2	925.00751	1849.00774	2.26	68.44	7881	7881	MS2	27/30	Glioma_Nuclear_prep_Gradeall_4plex.raw
4502107	High		gLGTDEESILTLLTSR	Unambiguous	1	1	N-Term(iTRAQ4plex)	HCD	1.0000	0.0000	1	1	12885	Unique	Used	5.253	1.605E3	1.060E4	136	6.42	0.00	0	6	300	2	925.00800	1849.00871	2.79	67.74	7826	7826	MS2	26/30	Glioma_Nuclear_prep_Gradeall_4plex.raw
4502107		High	gTVTDFPGFDER	Unambiguous	1	1	N-Term(iTRAQ4plex)	HCD	1.0000	0.0000	1	1	10351	Unique	Used	3.917	3.187E3	1.691E4	135	4.34	0.00	0	25	241	2	742.86212	1484.71697	2.10	49.27	4830	4830	MS2	19/22	Glioma_Nuclear_prep_Gradeall_repeat_4plex.raw
4502107		High	dLLDDLk	Unambiguous	1	1	N-Term(iTRAQ4plex); K7(iTRAQ4plex)	HCD	1.0000	0.0000	1	1	10781	Unique	Used	1.933	2.903E3	7.599E3	330	3.08	0.00	0	39	300	2	560.32770	1119.64812	-1.68	54.43	5292	5292	MS2	12/12	Glioma_Nuclear_prep_Gradeall_repeat_4plex.raw
4502107	High		lYDAYELk	Unambiguous	1	1	N-Term(iTRAQ4plex); K8(iTRAQ4plex)	HCD	1.0000	0.0000	1	1	9546	Unique	Used	2.131	1.249E5	3.348E5	214	2.93	0.00	0	47	13	2	651.85236	1302.69744	-16.08	37.58	4137	4137	MS2	14/14	Glioma_Nuclear_prep_Gradeall_4plex.raw
4502107	High		qEISAAFk	Unambiguous	1	1	N-Term(iTRAQ4plex); K8(iTRAQ4plex)	HCD	1.0000	0.0000	1	1	8223	Unique	Used	2.432	5.580E4	1.707E5	345	3.05	0.00	0	52	15	2	591.33167	1181.65605	-17.62	27.93	2694	2694	MS2	14/14	Glioma_Nuclear_prep_Gradeall_4plex.raw
4502107		High	lYDAYELk	Unambiguous	1	1	N-Term(iTRAQ4plex); K8(iTRAQ4plex)	HCD	1.0000	0.0000	1	1	10177	Unique	Used	3.493	4.678E3	2.213E4	292	3.34	0.00	0	32	300	2	651.86285	1302.71843	0.03	47.20	4644	4644	MS2	14/14	Glioma_Nuclear_prep_Gradeall_repeat_4plex.raw
4503149	NP_001902.1	CTSG	1511	cathepsin G preproprotein [Homo sapiens]	21.18	1	3	4	3.675	2;2	27.9	17.01	255	28.8	11.19																			
4503149	High		tIQNDIMLLQLSR	Unambiguous	1	1	N-Term(iTRAQ4plex)	HCD	1.0000	0.0000	1	1	11666	Unique	Used	3.653	5.085E3	2.337E4	250	4.73	0.00	0	24	150	2	844.97949	1688.95171	1.83	54.01	6448	6448	MS2	20/24	Glioma_Nuclear_prep_Gradeall_4plex.raw
4503149		High	sSGVPPEVFTR	Unambiguous	1	1	N-Term(iTRAQ4plex)	HCD	1.0000	0.0000	1	1	9141	Unique	Used	2.036	5.530E3	1.525E4	261	2.99	0.00	0	32	300	2	660.35638	1319.70549	-1.62	35.32	3532	3532	MS2	14/20	Glioma_Nuclear_prep_Gradeall_repeat_4plex.raw
4503149		High	sSGVPPEVFTR	Unambiguous	1	1	N-Term(iTRAQ4plex)	HCD	1.0000	0.0000	1	1	8986	Unique	Used	4.227	4.899E3	2.805E4	261	3.21	0.00	0	46	300	2	660.35803	1319.70879	0.87	33.55	3366	3366	MS2	14/20	Glioma_Nuclear_prep_Gradeall_repeat_4plex.raw
4503149	High		gDSGGPLLcNNVAHGIVSYGk	Unambiguous	1	1	N-Term(iTRAQ4plex); C9(Methylthio); K21(iTRAQ4plex)	HCD	1.0000	0.0000	1	1	10750	Unique	Used	3.696	5.284E3	2.456E4	61	5.76	0.00	0	21	25	3	798.06018	2392.16599	-13.90	46.53	5449	5449	MS2	33/80	Glioma_Nuclear_prep_Gradeall_4plex.raw
4503183	NP_001905.1	CYB5A	1528	cytochrome b5 isoform 2 [Homo sapiens]	43.88	3	2	3	1.871	1;2	33.0	15.63	98	11.3	5.14																			
4503183		High	fLEEHPGGEEVLR	Unambiguous	3	1	N-Term(iTRAQ4plex)	HCD	1.0000	0.0000	1	1	9640	Unique	Used	2.696	1.087E3	3.972E3	174	3.34	0.00	0	63	300	3	552.62006	1655.84562	-3.27	40.85	4066	4066	MS2	14/48	Glioma_Nuclear_prep_Gradeall_repeat_4plex.raw
4503183	High		yYTLEEIQk	Unambiguous	3	1	N-Term(iTRAQ4plex); K9(iTRAQ4plex)	HCD	1.0000	0.0000	1	1	9609	Unique	Used	1.399	1.294E5	2.277E5	242	3.83	0.00	0	47	20	2	737.89368	1474.78008	-15.66	38.03	4205	4205	MS2	15/16	Glioma_Nuclear_prep_Gradeall_4plex.raw
4503183		High	yYTLEEIQk	Unambiguous	3	1	N-Term(iTRAQ4plex); K9(iTRAQ4plex)	HCD	1.0000	0.0000	1	1	10273	Unique	Used	1.871	4.587E3	1.163E4	286	3.89	0.00	0	45	300	2	737.90649	1474.80571	1.72	48.31	4745	4745	MS2	16/16	Glioma_Nuclear_prep_Gradeall_repeat_4plex.raw
4503979	NP_002046.1	GFAP	2670	glial fibrillary acidic protein isoform 1 [Homo sapiens]	38.66	3	14	21	1.940	9;12	24.2	54.61	432	49.8	5.52																			
4503979	High		lADVYQAELR	Unambiguous	3	1	N-Term(iTRAQ4plex)	HCD	1.0000	0.0000	1	1	9628	Unique	Used	1.418	1.128E5	2.012E5	268	3.40	0.00	0	22	10	2	661.35413	1321.70098	-16.87	38.17	4226	4226	MS2	17/18	Glioma_Nuclear_prep_Gradeall_4plex.raw
4503979	High		aLAAELNQLR	Unambiguous	3	1	N-Term(iTRAQ4plex)	HCD	1.0000	0.0000	1	1	9896	Unique	Used	1.453	1.054E5	1.925E5	249	3.35	0.00	0	33	11	2	621.85791	1242.70854	-16.22	40.11	4517	4517	MS2	17/18	Glioma_Nuclear_prep_Gradeall_4plex.raw
4503979	High		lEAENNLAAYR	Unambiguous	3	1	N-Term(iTRAQ4plex)	HCD	1.0000	0.0000	1	1	8902	Unique	Used	1.559	1.493E5	2.928E5	196	3.41	0.00	0	39	12	2	704.35925	1407.71123	-16.82	32.81	3432	3432	MS2	17/20	Glioma_Nuclear_prep_Gradeall_4plex.raw
4503979	High		eAASYQEALAR	Unambiguous	3	1	N-Term(iTRAQ4plex)	HCD	1.0000	0.0000	1	1	8189	Unique	Used	2.066	2.555E5	6.639E5	174	3.91	0.00	0	32	4	2	676.83807	1352.66887	-17.62	27.70	2658	2658	MS2	17/20	Glioma_Nuclear_prep_Gradeall_4plex.raw
4503979	High		dNLAQDLATVR	Unambiguous	3	1	N-Term(iTRAQ4plex)	HCD	1.0000	0.0000	1	1	9713	Unique	Used	2.387	1.272E5	3.820E5	276	3.91	0.00	0	22	6	2	680.36011	1359.71294	-16.16	38.78	4318	4318	MS2	18/20	Glioma_Nuclear_prep_Gradeall_4plex.raw
4503979		High	lLEGEENR	Unambiguous	3	1	N-Term(iTRAQ4plex)	HCD	1.0000	0.0000	1	1	7815	Unique	Used	1.576	1.811E4	3.867E4	230	2.90	0.00	0	43	180	2	552.29462	1103.58196	0.54	22.00	2131	2131	MS2	12/14	Glioma_Nuclear_prep_Gradeall_repeat_4plex.raw
4503979		High	qEADEATLAR	Unambiguous	3	1	N-Term(iTRAQ4plex)	HCD	1.0000	0.0000	1	1	7280	Unique	Used	1.671	8.225E3	1.862E4	130	3.47	0.00	0	35	284	2	624.31091	1247.61455	-16.28	17.20	1564	1564	MS2	17/18	Glioma_Nuclear_prep_Gradeall_repeat_4plex.raw
4503979		High	fADLTDAAAR	Unambiguous	3	1	N-Term(iTRAQ4plex)	HCD	1.0000	0.0000	1	1	9402	Unique	Used	1.723	4.014E4	9.369E4	273	3.53	0.00	0	16	48	2	597.81573	1194.62419	0.52	38.17	3807	3807	MS2	17/18	Glioma_Nuclear_prep_Gradeall_repeat_4plex.raw
4503979		High	dNLAQDLATVR	Unambiguous	3	1	N-Term(iTRAQ4plex)	HCD	1.0000	0.0000	1	1	10376	Unique	Used	1.763	4.927E3	1.177E4	329	4.13	0.00	0	23	300	2	680.37109	1359.73491	0.00	49.55	4856	4856	MS2	19/20	Glioma_Nuclear_prep_Gradeall_repeat_4plex.raw
4503979		High	lADVYQAELR	Unambiguous	3	1	N-Term(iTRAQ4plex)	HCD	1.0000	0.0000	1	1	10231	Unique	Used	1.765	5.526E3	1.321E4	271	3.61	0.00	0	16	300	2	661.36517	1321.72307	-0.15	47.82	4701	4701	MS2	17/18	Glioma_Nuclear_prep_Gradeall_repeat_4plex.raw
4503979		High	aLAAELNQLR	Unambiguous	3	1	N-Term(iTRAQ4plex)	HCD	1.0000	0.0000	1	1	10676	Unique	Used	1.851	5.326E3	1.335E4	241	3.67	0.00	0	25	300	2	621.86816	1242.72905	0.28	53.15	5178	5178	MS2	17/18	Glioma_Nuclear_prep_Gradeall_repeat_4plex.raw
4503979		High	lEAENNLAAYR	Unambiguous	3	1	N-Term(iTRAQ4plex)	HCD	1.0000	0.0000	1	1	9361	Unique	Used	2.031	8.523E3	2.345E4	203	4.18	0.00	0	13	146	2	704.37256	1407.73784	2.09	37.74	3764	3764	MS2	19/20	Glioma_Nuclear_prep_Gradeall_repeat_4plex.raw
4503979	High		qLQSLTcDLESLR	Unambiguous	3	1	N-Term(iTRAQ4plex); C7(Methylthio)	HCD	1.0000	0.0000	1	1	11894	Unique	Used	3.047	2.868E4	1.099E5	204	4.74	0.00	0	6	50	2	848.43439	1695.86150	5.21	56.00	6696	6696	MS2	20/24	Glioma_Nuclear_prep_Gradeall_4plex.raw
4503979		High	qLQSLTcDLESLR	Unambiguous	3	1	N-Term(iTRAQ4plex); C7(Methylthio)	HCD	1.0000	0.0000	1	1	12342	Unique	Used	2.201	6.196E2	1.847E3	169	3.56	0.00	0	18	300	2	848.43103	1695.85478	1.25	80.48	7420	7420	MS2	16/24	Glioma_Nuclear_prep_Gradeall_repeat_4plex.raw
4503979	High		kIESLEEEIR	Unambiguous	3	1	N-Term(iTRAQ4plex); K1(iTRAQ4plex)	HCD	1.0000	0.0000	1	1	9124	Unique	Used	1.902	8.576E4	2.052E5	230	3.36	0.00	1	9	6	2	767.42664	1533.84599	-17.41	34.47	3676	3676	MS2	18/18	Glioma_Nuclear_prep_Gradeall_4plex.raw
4503979	High		hLQEYQDLLNVk	Unambiguous	3	1	N-Term(iTRAQ4plex); K12(iTRAQ4plex)	HCD	1.0000	0.0000	1	1	9750	Unique	Used	2.720	6.902E4	2.361E5	192	5.26	0.00	0	13	15	2	894.48425	1787.96123	-15.78	39.04	4358	4358	MS2	22/22	Glioma_Nuclear_prep_Gradeall_4plex.raw
4503979		High	hLQEYQDLLNVk	Unambiguous	3	1	N-Term(iTRAQ4plex); K12(iTRAQ4plex)	HCD	1.0000	0.0000	1	1	10576	Unique	Used	1.940	2.188E3	5.749E3	197	5.37	0.00	0	38	300	2	894.49872	1787.99016	0.40	51.94	5070	5070	MS2	22/22	Glioma_Nuclear_prep_Gradeall_repeat_4plex.raw
4503979		High	fASYIEk	Unambiguous	3	1	N-Term(iTRAQ4plex); K7(iTRAQ4plex)	HCD	1.0000	0.0000	1	1	9591	Unique	Used	3.005	2.108E4	8.579E4	158	2.97	0.00	0	19	66	2	573.32288	1145.63848	-5.26	40.31	4014	4014	MS2	12/12	Glioma_Nuclear_prep_Gradeall_repeat_4plex.raw
4503979		High	sVSEGHLk	Unambiguous	1	1	N-Term(iTRAQ4plex); K8(iTRAQ4plex)	HCD	1.0000	0.0000	1	1	6864	Unique	Used	2.377	1.054E4	3.395E4	249	2.97	0.00	0	21	93	2	572.82861	1144.64995	-5.69	13.56	1124	1124	MS2	14/14	Glioma_Nuclear_prep_Gradeall_repeat_4plex.raw
4503979	High		lEEEGQSLkDEMAR	Unambiguous	3	1	N-Term(iTRAQ4plex); K9(iTRAQ4plex)	HCD	1.0000	0.0000	1	1	9427	Unique	Used	2.782	1.981E4	6.932E4	119	4.96	0.00	1	20	28	2	961.97290	1922.93852	-18.06	36.70	4007	4007	MS2	23/26	Glioma_Nuclear_prep_Gradeall_4plex.raw
4503979		High	lEEEGQSLkDEMAR	Unambiguous	3	1	N-Term(iTRAQ4plex); K9(iTRAQ4plex)	HCD	1.0000	0.0000	1	1	10102	Unique	Used	2.583	8.163E2	2.855E3	175	5.00	0.00	1	4	300	2	961.99164	1922.97600	1.43	46.32	4564	4564	MS2	24/26	Glioma_Nuclear_prep_Gradeall_repeat_4plex.raw
4504255	NP_002097.1	H2AFZ	3015	histone H2A.Z [Homo sapiens]	25.78	6	2	6	2.701	3;3	38.7	24.17	128	13.5	10.58																			
4504255	High		gDEELDSLIk	Unambiguous	5	1	N-Term(iTRAQ4plex); K10(iTRAQ4plex)	HCD	1.0000	0.0000	1	1	9666	Unique	Used	1.537	1.493E5	2.886E5	236	3.83	0.00	0	34	15	2	703.87286	1406.73845	-16.56	38.44	4267	4267	MS2	18/18	Glioma_Nuclear_prep_Gradeall_4plex.raw
4504255		High	gDEELDSLIk	Unambiguous	5	1	N-Term(iTRAQ4plex); K10(iTRAQ4plex)	HCD	1.0000	0.0000	1	1	10297	Unique	Used	2.072	5.113E3	1.435E4	260	4.50	0.00	0	19	300	2	703.88446	1406.76164	-0.07	48.63	4772	4772	MS2	18/18	Glioma_Nuclear_prep_Gradeall_repeat_4plex.raw
4504255	High		aTIAGGGVIPHIHk	Unambiguous	4	1	N-Term(iTRAQ4plex); K14(iTRAQ4plex)	HCD	1.0000	0.0000	1	1	8448	Unique	Used	3.662	4.939E4	2.275E5	165	5.62	0.00	0	4	8	2	829.98712	1658.96697	-16.59	29.53	2938	2938	MS2	25/26	Glioma_Nuclear_prep_Gradeall_4plex.raw
4504255	High		aTIAGGGVIPHIHk	Unambiguous	4	1	N-Term(iTRAQ4plex); K14(iTRAQ4plex)	HCD	1.0000	0.0000	1	1	8440	Unique	Used	4.402	2.192E4	1.213E5	177	4.27	0.00	0	8	1	4	415.49701	1658.96621	-17.05	29.48	2930	2930	MS2	31/78	Glioma_Nuclear_prep_Gradeall_4plex.raw
4504255		High	aTIAGGGVIPHIHk	Unambiguous	4	1	N-Term(iTRAQ4plex); K14(iTRAQ4plex)	HCD	1.0000	0.0000	1	1	9057	Unique	Used	2.145	2.108E3	6.125E3	108	5.89	0.00	0	35	300	2	830.00323	1658.99919	2.84	34.39	3443	3443	MS2	25/26	Glioma_Nuclear_prep_Gradeall_repeat_4plex.raw
4504255		High	aTIAGGGVIPHIHk	Unambiguous	4	1	N-Term(iTRAQ4plex); K14(iTRAQ4plex)	HCD	1.0000	0.0000	1	1	9038	Unique	Used	3.257	4.776E2	2.107E3	127	5.07	0.00	0	54	300	4	415.50314	1658.99074	-2.26	34.17	3423	3423	MS2	29/78	Glioma_Nuclear_prep_Gradeall_repeat_4plex.raw
4504313	NP_003535.1	HIST1H4B	8366	histone H4 [Homo sapiens]	51.46	1	6	27	3.327	12;15	39.9	68.81	103	11.4	11.36																			
4504313	High		iSGLIYEETR	Unambiguous	1	1	N-Term(iTRAQ4plex)	HCD	1.0000	0.0000	1	1	9793	Unique	Used	2.375	2.742E5	8.191E5	286	3.66	0.00	0	23	4	2	662.85443	1324.70159	-16.12	39.35	4405	4405	MS2	17/18	Glioma_Nuclear_prep_Gradeall_4plex.raw
4504313	High		vFLENVIR	Unambiguous	1	1	N-Term(iTRAQ4plex)	HCD	1.0000	0.0000	1	1	10257	Unique	Used	4.454	1.636E5	9.163E5	322	3.25	0.00	0	11	4	2	567.33478	1133.66228	-15.59	42.77	4910	4910	MS2	13/14	Glioma_Nuclear_prep_Gradeall_4plex.raw
4504313		High	iSGLIYEETR	Unambiguous	1	1	N-Term(iTRAQ4plex)	HCD	1.0000	0.0000	1	1	10478	Unique	Used	2.217	4.199E3	1.261E4	366	3.71	0.00	0	41	300	2	662.86560	1324.72392	0.75	50.76	4964	4964	MS2	17/18	Glioma_Nuclear_prep_Gradeall_repeat_4plex.raw
4504313		High	iSGLIYEETR	Unambiguous	1	1	N-Term(iTRAQ4plex)	HCD	1.0000	0.0000	1	1	10555	Unique	Used	3.234	1.120E4	4.907E4	274	3.66	0.00	0	8	64	2	662.86560	1324.72392	0.75	51.70	5048	5048	MS2	17/18	Glioma_Nuclear_prep_Gradeall_repeat_4plex.raw
4504313		High	vFLENVIR	Unambiguous	1	1	N-Term(iTRAQ4plex)	HCD	1.0000	0.0000	1	1	11126	Unique	Used	3.248	2.166E3	9.528E3	340	2.84	0.00	0	29	300	2	567.34338	1133.67949	-0.41	58.82	5676	5676	MS2	13/14	Glioma_Nuclear_prep_Gradeall_repeat_4plex.raw
4504313		High	vFLENVIR	Unambiguous	1	1	N-Term(iTRAQ4plex)	HCD	1.0000	0.0000	1	1	11209	Unique	Used	4.633	1.534E4	9.625E4	271	3.03	0.00	0	6	71	2	567.34393	1133.68059	0.56	59.91	5772	5772	MS2	14/14	Glioma_Nuclear_prep_Gradeall_repeat_4plex.raw
4504313	High		kTVTAMDVVYALk	Unambiguous	1	1	N-Term(iTRAQ4plex); K1(iTRAQ4plex); K13(iTRAQ4plex)	HCD	1.0000	0.0000	1	1	10700	Unique	Used	3.428	4.159E4	1.793E5	147	5.63	0.00	1	28	22	2	936.04224	1871.07720	-14.15	46.16	5395	5395	MS2	23/24	Glioma_Nuclear_prep_Gradeall_4plex.raw
4504313	High		dAVTYTEHAk	Unambiguous	1	1	N-Term(iTRAQ4plex); K10(iTRAQ4plex)	HCD	1.0000	0.0000	1	1	7400	Unique	Used	3.640	4.602E4	2.107E5	229	4.71	0.00	0	11	11	2	711.87225	1422.73723	-6.68	22.11	1803	1803	MS2	18/18	Glioma_Nuclear_prep_Gradeall_4plex.raw
4504313	High		dAVTYTEHAk	Unambiguous	1	1	N-Term(iTRAQ4plex); K10(iTRAQ4plex)	HCD	1.0000	0.0000	1	1	7389	Unique	Used	5.237	2.899E4	1.909E5	210	4.12	0.00	0	16	3	3	474.91730	1422.73734	-6.61	22.02	1791	1791	MS2	24/36	Glioma_Nuclear_prep_Gradeall_4plex.raw
4504313		High	dAVTYTEHAk	Unambiguous	1	1	N-Term(iTRAQ4plex); K10(iTRAQ4plex)	HCD	1.0000	0.0000	1	1	7327	Unique	Used	3.736	2.058E4	1.041E5	211	4.29	0.00	0	9	5	3	474.91681	1422.73587	-7.64	17.65	1614	1614	MS2	24/36	Glioma_Nuclear_prep_Gradeall_repeat_4plex.raw
4504313		High	dAVTYTEHAk	Unambiguous	1	1	N-Term(iTRAQ4plex); K10(iTRAQ4plex)	HCD	1.0000	0.0000	1	1	7329	Unique	Used	3.885	1.667E4	8.771E4	223	4.68	0.00	0	8	31	2	711.87164	1422.73601	-7.54	17.66	1616	1616	MS2	18/18	Glioma_Nuclear_prep_Gradeall_repeat_4plex.raw
4504313	High		tVTAMDVVYALk	Unambiguous	1	1	N-Term(iTRAQ4plex); K12(iTRAQ4plex)	HCD	1.0000	0.0000	1	1	11685	Unique	Used	2.974	3.890E4	1.455E5	234	6.00	0.00	0	22	53	2	799.95740	1598.90752	0.55	54.19	6469	6469	MS2	21/22	Glioma_Nuclear_prep_Gradeall_4plex.raw
4504313	High		tVTAMDVVYALk	Unambiguous	1	1	N-Term(iTRAQ4plex); K12(iTRAQ4plex)	HCD	1.0000	0.0000	1	1	11694	Unique	Used	3.206	6.707E3	2.704E4	251	4.10	0.00	0	30	9	3	533.63824	1598.90018	-4.04	54.27	6479	6479	MS2	19/44	Glioma_Nuclear_prep_Gradeall_4plex.raw
4504313	High		tVTAMDVVYALk	Unambiguous	1	1	N-Term(iTRAQ4plex); K12(iTRAQ4plex)	HCD	1.0000	0.0000	1	1	11762	Unique	Used	5.066	8.596E4	5.477E5	230	5.78	0.00	0	8	6	2	799.95691	1598.90654	-0.06	54.89	6553	6553	MS2	21/22	Glioma_Nuclear_prep_Gradeall_4plex.raw
4504313	High		tVTAMDVVYALk	Unambiguous	1	1	N-Term(iTRAQ4plex); K12(iTRAQ4plex)	HCD	1.0000	0.0000	1	1	11775	Unique	Used	6.889	3.969E3	3.439E4	239	4.23	0.00	0	9	7	3	533.63898	1598.90238	-2.66	55.00	6567	6567	MS2	23/44	Glioma_Nuclear_prep_Gradeall_4plex.raw
4504313		High	tVTAMDVVYALk	Unambiguous	1	1	N-Term(iTRAQ4plex); K12(iTRAQ4plex)	HCD	1.0000	0.0000	1	1	12259	Unique	Used	1.321	3.019E2	5.400E2	233	3.67	0.00	0	37	300	3	533.63983	1598.90494	-1.06	77.78	7224	7224	MS2	18/44	Glioma_Nuclear_prep_Gradeall_repeat_4plex.raw
4504313		High	tVTAMDVVYALk	Unambiguous	1	1	N-Term(iTRAQ4plex); K12(iTRAQ4plex)	HCD	1.0000	0.0000	1	1	12257	Unique	Used	1.472	1.625E3	3.240E3	232	5.16	0.00	0	40	300	2	799.95746	1598.90764	0.63	77.75	7221	7221	MS2	21/22	Glioma_Nuclear_prep_Gradeall_repeat_4plex.raw
4504313		High	tVTAMDVVYALk	Unambiguous	1	1	N-Term(iTRAQ4plex); K12(iTRAQ4plex)	HCD	1.0000	0.0000	1	1	12289	Unique	Used	4.719	1.634E3	1.045E4	233	5.90	0.00	0	6	300	2	799.95709	1598.90691	0.17	78.76	7295	7295	MS2	21/22	Glioma_Nuclear_prep_Gradeall_repeat_4plex.raw
4504313		High	tVTAMDVVYALk	Unambiguous	1	1	N-Term(iTRAQ4plex); K12(iTRAQ4plex)	HCD	1.0000	0.0000	1	1	12291	Unique	Used	7.067	1.728E2	1.654E3	230	4.16	0.00	0	9	179	3	533.64001	1598.90549	-0.72	78.81	7299	7299	MS2	22/44	Glioma_Nuclear_prep_Gradeall_repeat_4plex.raw
4504313	High		dNIQGITkPAIR	Unambiguous	1	1	N-Term(iTRAQ4plex); K8(iTRAQ4plex)	HCD	1.0000	0.0000	1	1	8312	Unique	Used	3.376	8.531E4	3.622E5	205	3.85	0.00	0	21	7	2	807.46869	1613.93010	-17.14	28.57	2791	2791	MS2	17/22	Glioma_Nuclear_prep_Gradeall_4plex.raw
4504313		High	dNIQGITkPAIR	Unambiguous	1	1	N-Term(iTRAQ4plex); K8(iTRAQ4plex)	HCD	1.0000	0.0000	1	1	8757	Unique	Used	3.075	3.192E3	1.330E4	119	3.90	0.00	0	25	296	2	807.47198	1613.93669	-13.06	31.13	3123	3123	MS2	16/22	Glioma_Nuclear_prep_Gradeall_repeat_4plex.raw
4504313	High		tVTAmDVVYALk	Unambiguous	1	1	N-Term(iTRAQ4plex); M5(Oxidation); K12(iTRAQ4plex)	HCD	1.0000	0.0000	1	1	10515	Unique	Used	2.480	4.122E4	1.286E5	223	4.93	0.00	0	32	32	2	807.94171	1614.87615	-15.73	44.76	5193	5193	MS2	20/22	Glioma_Nuclear_prep_Gradeall_4plex.raw
4504313	High		tVTAmDVVYALk	Unambiguous	1	1	N-Term(iTRAQ4plex); M5(Oxidation); K12(iTRAQ4plex)	HCD	1.0000	0.0000	1	1	10606	Unique	Used	3.327	6.613E4	2.767E5	226	5.01	0.00	0	15	9	2	807.94202	1614.87676	-15.35	45.44	5292	5292	MS2	20/22	Glioma_Nuclear_prep_Gradeall_4plex.raw
4504313		High	tVTAmDVVYALk	Unambiguous	1	1	N-Term(iTRAQ4plex); M5(Oxidation); K12(iTRAQ4plex)	HCD	1.0000	0.0000	1	1	11439	Unique	Used	1.866	7.399E2	1.870E3	220	4.84	0.00	0	11	217	3	538.97186	1614.90104	-0.32	63.34	6059	6059	MS2	21/44	Glioma_Nuclear_prep_Gradeall_repeat_4plex.raw
4504313		High	tVTAmDVVYALk	Unambiguous	1	1	N-Term(iTRAQ4plex); M5(Oxidation); K12(iTRAQ4plex)	HCD	1.0000	0.0000	1	1	11361	Unique	Used	2.366	2.945E2	9.439E2	216	4.25	0.00	0	22	300	3	538.97205	1614.90158	0.02	62.27	5967	5967	MS2	20/44	Glioma_Nuclear_prep_Gradeall_repeat_4plex.raw
4504313		High	tVTAmDVVYALk	Unambiguous	1	1	N-Term(iTRAQ4plex); M5(Oxidation); K12(iTRAQ4plex)	HCD	1.0000	0.0000	1	1	11356	Unique	Used	2.698	1.371E3	5.010E3	210	4.84	0.00	0	18	300	2	807.95477	1614.90227	0.44	62.20	5961	5961	MS2	22/22	Glioma_Nuclear_prep_Gradeall_repeat_4plex.raw
4504313		High	tVTAmDVVYALk	Unambiguous	1	1	N-Term(iTRAQ4plex); M5(Oxidation); K12(iTRAQ4plex)	HCD	1.0000	0.0000	1	1	11434	Unique	Used	3.443	2.367E3	1.104E4	210	5.11	0.00	0	23	300	2	807.95465	1614.90203	0.29	63.27	6053	6053	MS2	20/22	Glioma_Nuclear_prep_Gradeall_repeat_4plex.raw
4504425	NP_002119.1	HMGB1	3146	high mobility group protein B1 [Homo sapiens]	27.44	1	5	16	3.346	5;11	36.2	64.64	215	24.9	5.74																			
4504425	High		kHPDASVNFSEFSk	Unambiguous	1	1	N-Term(iTRAQ4plex); K1(iTRAQ4plex); K14(iTRAQ4plex)	HCD	1.0000	0.0000	1	1	8459	Unique	Used	4.467	2.296E4	1.290E5	140	4.04	0.00	1	7	12	2	1013.02338	2025.03948	-18.33	29.60	2950	2950	MS2	20/26	Glioma_Nuclear_prep_Gradeall_4plex.raw
4504425		High	kHPDASVNFSEFSk	Unambiguous	1	1	N-Term(iTRAQ4plex); K1(iTRAQ4plex); K14(iTRAQ4plex)	HCD	1.0000	0.0000	1	1	9095	Unique	Used	3.517	5.103E2	2.431E3	178	4.06	0.00	1	24	68	4	507.02374	2025.07314	-1.71	34.82	3484	3484	MS2	15/78	Glioma_Nuclear_prep_Gradeall_repeat_4plex.raw
4504425		High	kHPDASVNFSEFSk	Unambiguous	1	1	N-Term(iTRAQ4plex); K1(iTRAQ4plex); K14(iTRAQ4plex)	HCD	1.0000	0.0000	1	1	9078	Unique	Used	4.637	1.265E3	7.945E3	119	5.57	0.00	1	14	70	3	675.69849	2025.08091	2.13	34.63	3466	3466	MS2	24/52	Glioma_Nuclear_prep_Gradeall_repeat_4plex.raw
4504425		High	kHPDASVNFSEFSk	Unambiguous	1	1	N-Term(iTRAQ4plex); K1(iTRAQ4plex); K14(iTRAQ4plex)	HCD	1.0000	0.0000	1	1	9087	Unique	Used	4.968	1.344E3	9.042E3	97	4.85	0.00	1	0	300	2	1013.04340	2025.07952	1.44	34.72	3475	3475	MS2	22/26	Glioma_Nuclear_prep_Gradeall_repeat_4plex.raw
4504425	High		hPDASVNFSEFSk	Unambiguous	1	1	N-Term(iTRAQ4plex); K13(iTRAQ4plex)	HCD	1.0000	0.0000	1	1	9015	Unique	Used	1.803	1.521E4	3.450E4	125	5.25	0.00	0	19	9	3	584.95447	1752.84885	-17.52	33.63	3555	3555	MS2	24/48	Glioma_Nuclear_prep_Gradeall_4plex.raw
4504425	High		gEHPGLSIGDVAk	Unambiguous	1	1	N-Term(iTRAQ4plex); K13(iTRAQ4plex)	HCD	1.0000	0.0000	1	1	8284	Unique	Used	2.740	3.025E4	1.042E5	254	3.76	0.00	0	56	21	2	784.42365	1567.84001	-18.03	28.36	2760	2760	MS2	18/24	Glioma_Nuclear_prep_Gradeall_4plex.raw
4504425	High		hPDASVNFSEFSk	Unambiguous	1	1	N-Term(iTRAQ4plex); K13(iTRAQ4plex)	HCD	1.0000	0.0000	1	1	9006	Unique	Used	4.754	2.967E4	1.774E5	120	4.44	0.00	0	0	14	2	876.92950	1752.85173	-15.88	33.56	3545	3545	MS2	17/24	Glioma_Nuclear_prep_Gradeall_4plex.raw
4504425		High	gEHPGLSIGDVAk	Unambiguous	1	1	N-Term(iTRAQ4plex); K13(iTRAQ4plex)	HCD	1.0000	0.0000	1	1	8832	Unique	Used	1.454	3.742E3	7.371E3	282	3.30	0.00	0	35	300	2	784.43988	1567.87248	2.68	31.98	3202	3202	MS2	13/24	Glioma_Nuclear_prep_Gradeall_repeat_4plex.raw
4504425		High	gEHPGLSIGDVAk	Unambiguous	1	1	N-Term(iTRAQ4plex); K13(iTRAQ4plex)	HCD	1.0000	0.0000	1	1	8723	Unique	Used	1.884	1.720E3	4.387E3	230	6.04	0.00	0	39	242	3	523.29498	1567.87040	1.34	30.84	3087	3087	MS2	21/48	Glioma_Nuclear_prep_Gradeall_repeat_4plex.raw
4504425		High	gEHPGLSIGDVAk	Unambiguous	1	1	N-Term(iTRAQ4plex); K13(iTRAQ4plex)	HCD	1.0000	0.0000	1	1	8745	Unique	Used	2.344	2.713E3	8.612E3	242	5.47	0.00	0	10	53	3	523.28613	1567.84385	-15.59	31.02	3111	3111	MS2	24/48	Glioma_Nuclear_prep_Gradeall_repeat_4plex.raw
4504425		High	hPDASVNFSEFSk	Unambiguous	1	1	N-Term(iTRAQ4plex); K13(iTRAQ4plex)	HCD	1.0000	0.0000	1	1	9650	Unique	Used	2.534	1.394E3	4.786E3	165	6.29	0.00	0	9	138	3	584.96356	1752.87613	-1.96	40.97	4077	4077	MS2	24/48	Glioma_Nuclear_prep_Gradeall_repeat_4plex.raw
4504425		High	gEHPGLSIGDVAk	Unambiguous	1	1	N-Term(iTRAQ4plex); K13(iTRAQ4plex)	HCD	1.0000	0.0000	1	1	8755	Unique	Used	2.986	1.566E3	6.334E3	204	3.13	0.00	0	6	257	2	784.42700	1567.84673	-13.75	31.11	3121	3121	MS2	20/24	Glioma_Nuclear_prep_Gradeall_repeat_4plex.raw
4504425		High	hPDASVNFSEFSk	Unambiguous	1	1	N-Term(iTRAQ4plex); K13(iTRAQ4plex)	HCD	1.0000	0.0000	1	1	9659	Unique	Used	4.210	1.863E3	1.062E4	147	4.49	0.00	0	10	300	2	876.94238	1752.87749	-1.19	41.07	4086	4086	MS2	19/24	Glioma_Nuclear_prep_Gradeall_repeat_4plex.raw
4504425		High	ikGEHPGLSIGDVAk	Unambiguous	1	1	N-Term(iTRAQ4plex); K2(iTRAQ4plex); K15(iTRAQ4plex)	HCD	1.0000	0.0000	1	1	8902	Unique	Used	3.909	1.443E3	7.640E3	105	3.50	0.00	1	14	45	3	651.71893	1953.14225	-3.66	32.67	3276	3276	MS2	25/56	Glioma_Nuclear_prep_Gradeall_repeat_4plex.raw
4504425	High		tYIPPkGETk	Unambiguous	1	1	N-Term(iTRAQ4plex); K6(iTRAQ4plex); K10(iTRAQ4plex)	HCD	1.0000	0.0000	1	1	7511	Unique	Used	3.176	6.612E4	2.641E5	143	3.29	0.00	1	18	8	2	783.46265	1565.91802	-5.31	22.90	1923	1923	MS2	14/18	Glioma_Nuclear_prep_Gradeall_4plex.raw
4504425		High	tYIPPkGETk	Unambiguous	1	1	N-Term(iTRAQ4plex); K6(iTRAQ4plex); K10(iTRAQ4plex)	HCD	1.0000	0.0000	1	1	7564	Unique	Used	5.405	1.325E3	9.703E3	156	2.96	0.00	1	10	167	2	783.46857	1565.92986	2.25	19.64	1862	1862	MS2	15/18	Glioma_Nuclear_prep_Gradeall_repeat_4plex.raw
4504445	NP_002127.1	HNRNPA1	3178	heterogeneous nuclear ribonucleoprotein A1 isoform a [Homo sapiens]	36.56	3	7	16	0.165	8;8	38.7	49.91	320	34.2	9.23																			
4504445	High		iEVIEIMTDR	Unambiguous	3	1	N-Term(iTRAQ4plex)	HCD	1.0000	0.0000	1	1	11291	Unique	Used	0.151	2.192E5	4.158E4	329	3.99	0.00	0	22	27	2	681.87372	1362.74016	-1.34	50.80	6039	6039	MS2	15/18	Glioma_Nuclear_prep_Gradeall_4plex.raw
4504445	High		lFIGGLSFETTDESLR	Unambiguous	3	1	N-Term(iTRAQ4plex)	HCD	1.0000	0.0000	1	1	12107	Unique	Used	0.161	4.547E4	9.185E3	231	5.11	0.00	0	21	184	2	965.01422	1929.02117	6.49	58.09	6930	6930	MS2	23/30	Glioma_Nuclear_prep_Gradeall_4plex.raw
4504445	High		iEVIEIMTDR	Unambiguous	3	1	N-Term(iTRAQ4plex)	HCD	1.0000	0.0000	1	1	11205	Unique	Used	0.169	2.436E5	5.183E4	304	4.05	0.00	0	33	21	2	681.87054	1362.73381	-6.00	50.06	5945	5945	MS2	15/18	Glioma_Nuclear_prep_Gradeall_4plex.raw
4504445		High	nQGGYGGSSSSSSYGSGR	Unambiguous	2	1	N-Term(iTRAQ4plex)	HCD	1.0000	0.0000	1	1	7004	Unique	Used	0.049	2.648E4	1.746E3	13	6.00	0.00	0	4	192	2	919.90302	1838.79875	-1.88	14.83	1272	1272	MS2	25/34	Glioma_Nuclear_prep_Gradeall_repeat_4plex.raw
4504445		High	iEVIEIMTDR	Unambiguous	3	1	N-Term(iTRAQ4plex)	HCD	1.0000	0.0000	1	1	11990	Unique	Used	0.141	1.852E4	3.529E3	337	3.73	0.00	0	34	300	2	681.87610	1362.74492	2.15	71.84	6766	6766	MS2	14/18	Glioma_Nuclear_prep_Gradeall_repeat_4plex.raw
4504445	High		eDSQRPGAHLTVk	Unambiguous	3	1	N-Term(iTRAQ4plex); K13(iTRAQ4plex)	HCD	1.0000	0.0000	1	1	7328	Unique	Used	0.205	6.510E4	1.677E4	219	4.05	0.00	0	12	22	2	863.47632	1725.94536	-1.91	21.57	1724	1724	MS2	18/24	Glioma_Nuclear_prep_Gradeall_4plex.raw
4504445		High	eDSQRPGAHLTVk	Unambiguous	3	1	N-Term(iTRAQ4plex); K13(iTRAQ4plex)	HCD	1.0000	0.0000	1	1	7203	Unique	Used	0.194	4.880E3	1.279E3	200	3.25	0.00	0	9	168	2	863.46692	1725.92656	-12.80	16.55	1483	1483	MS2	16/24	Glioma_Nuclear_prep_Gradeall_repeat_4plex.raw
4504445	High		sSGPYGGGGQYFAkPR	Unambiguous	2	1	N-Term(iTRAQ4plex); K14(iTRAQ4plex)	HCD	1.0000	0.0000	1	1	8202	Unique	Used	0.207	1.545E5	4.025E4	88	4.95	0.00	0	32	3	3	639.65662	1916.95530	-15.90	27.79	2672	2672	MS2	30/60	Glioma_Nuclear_prep_Gradeall_4plex.raw
4504445		High	sSGPYGGGGQYFAkPR	Unambiguous	2	1	N-Term(iTRAQ4plex); K14(iTRAQ4plex)	HCD	1.0000	0.0000	1	1	8618	Unique	Used	0.106	7.967E3	1.143E3	93	5.65	0.00	0	13	300	2	958.99927	1916.99126	2.86	29.82	2977	2977	MS2	28/30	Glioma_Nuclear_prep_Gradeall_repeat_4plex.raw
4504445		High	sSGPYGGGGQYFAkPR	Unambiguous	2	1	N-Term(iTRAQ4plex); K14(iTRAQ4plex)	HCD	1.0000	0.0000	1	1	8587	Unique	Used	0.245	7.323E3	2.426E3	130	5.02	0.00	0	21	119	3	639.66583	1916.98294	-1.47	29.50	2945	2945	MS2	32/60	Glioma_Nuclear_prep_Gradeall_repeat_4plex.raw
4504445	High		gFAFVTFDDHDSVDk	Unambiguous	3	1	N-Term(iTRAQ4plex); K15(iTRAQ4plex)	HCD	1.0000	0.0000	1	1	10260	Unique	Used	0.155	1.672E5	3.263E4	92	5.16	0.00	0	5	12	2	994.47174	1987.93620	-14.01	42.79	4913	4913	MS2	25/28	Glioma_Nuclear_prep_Gradeall_4plex.raw
4504445	High		gFAFVTFDDHDSVDk	Unambiguous	3	1	N-Term(iTRAQ4plex); K15(iTRAQ4plex)	HCD	1.0000	0.0000	1	1	10245	Unique	Used	0.355	7.780E4	3.470E4	92	4.09	0.00	0	15	6	3	663.31683	1987.93595	-14.14	42.69	4897	4897	MS2	27/56	Glioma_Nuclear_prep_Gradeall_4plex.raw
4504445		High	gFAFVTFDDHDSVDk	Unambiguous	3	1	N-Term(iTRAQ4plex); K15(iTRAQ4plex)	HCD	1.0000	0.0000	1	1	11213	Unique	Used	0.134	4.258E3	7.703E2	124	5.89	0.00	0	3	300	2	994.48755	1987.96782	1.90	59.98	5777	5777	MS2	23/28	Glioma_Nuclear_prep_Gradeall_repeat_4plex.raw
4504445		High	gFAFVTFDDHDSVDk	Unambiguous	3	1	N-Term(iTRAQ4plex); K15(iTRAQ4plex)	HCD	1.0000	0.0000	1	1	11172	Unique	Used	0.260	1.557E3	5.488E2	110	5.40	0.00	0	14	300	3	663.32794	1987.96927	2.63	59.44	5730	5730	MS2	29/56	Glioma_Nuclear_prep_Gradeall_repeat_4plex.raw
4504445	High		dYFEQYGk	Unambiguous	3	1	N-Term(iTRAQ4plex); K8(iTRAQ4plex)	HCD	1.0000	0.0000	1	1	9044	Unique	Used	0.147	7.061E5	1.306E5	87	3.15	0.00	0	31	7	2	669.32495	1337.64263	-14.18	33.85	3587	3587	MS2	13/14	Glioma_Nuclear_prep_Gradeall_4plex.raw
4504445		High	dYFEQYGk	Unambiguous	3	1	N-Term(iTRAQ4plex); K8(iTRAQ4plex)	HCD	1.0000	0.0000	1	1	9472	Unique	Used	0.191	7.780E4	2.017E4	103	3.18	0.00	0	50	140	2	669.33630	1337.66533	2.79	38.93	3882	3882	MS2	13/14	Glioma_Nuclear_prep_Gradeall_repeat_4plex.raw
4505881	NP_000292.1	PLG	5340	plasminogen isoform 1 precursor [Homo sapiens]	4.32	1	3	3	4.338	2;1	27.3	8.13	810	90.5	7.24																			
4505881	High		fSPATHPSEGLEENYcR	Unambiguous	1	1	N-Term(iTRAQ4plex); C16(Methylthio)	HCD	1.0000	0.0000	1	1	9462	Unique	Used	5.728	1.297E4	9.341E4	25	5.11	0.00	0	23	6	3	709.64056	2126.90714	-15.10	36.98	4046	4046	MS2	27/64	Glioma_Nuclear_prep_Gradeall_4plex.raw
4505881	High		wELcDIPR	Unambiguous	1	1	N-Term(iTRAQ4plex); C4(Methylthio)	HCD	1.0000	0.0000	1	1	11389	Unique	Used	4.338	2.041E4	1.113E5	105	3.02	0.00	0	40	67	2	611.29657	1221.58586	-1.52	51.64	6146	6146	MS2	13/14	Glioma_Nuclear_prep_Gradeall_4plex.raw

4505881		High	ISSPAVITDk	Unambiguous	1	1	N-Term(iTRAQ4plex); K10(iTRAQ4plex)	HCD	1.0000	0.0000	1	1	8963	Unique	Used	3.306	6.561E3	2.938E4	299	3.70	0.00	0	45	300	2	659.89063	1318.77397	-6.15	33.26	3340	3340	MS2	16/18	Glioma_Nuclear_prep_Gradeall_repeat_4plex.raw
4506671	NP_000995.1	RPLP2	6181	60S acidic ribosomal protein P2 [Homo sapiens]	26.96	1	1	2	0.575	1;1	5.1	15.37	115	11.7	4.54																			
4506671	High		nIEDVIAQGIGk	Unambiguous	1	1	N-Term(iTRAQ4plex); K12(iTRAQ4plex)	HCD	1.0000	0.0000	1	1	10887	Unique	Used	0.554	3.787E4	2.637E4	219	4.35	0.00	0	58	65	2	772.93610	1544.86492	-15.39	47.58	5598	5598	MS2	20/22	Glioma_Nuclear_prep_Gradeall_4plex.raw
4506671		High	nIEDVIAQGIGk	Unambiguous	1	1	N-Term(iTRAQ4plex); K12(iTRAQ4plex)	HCD	1.0000	0.0000	1	1	11701	Unique	Used	0.596	3.187E3	2.571E3	221	4.36	0.00	0	48	300	2	772.94995	1544.89263	2.55	66.99	6370	6370	MS2	21/22	Glioma_Nuclear_prep_Gradeall_repeat_4plex.raw
4506903	NP_003760.1	SRSF9	8683	serine/arginine-rich splicing factor 9 [Homo sapiens]	39.82	1	9	13	1.719	5;8	19.1	37.17	221	25.5	8.65																			
4506903	High		dGVGMVEYLR	Unambiguous	1	1	N-Term(iTRAQ4plex)	HCD	1.0000	0.0000	1	1	9842	Unique	Used	1.345	1.259E5	2.130E5	154	3.20	0.00	0	31	10	2	641.82220	1282.63713	-16.46	39.71	4458	4458	MS2	18/18	Glioma_Nuclear_prep_Gradeall_4plex.raw
4506903	High		iYVGNLPTDVR	Unambiguous	1	1	N-Term(iTRAQ4plex)	HCD	1.0000	0.0000	1	1	9772	Unique	Used	1.603	1.087E5	2.191E5	305	3.77	0.00	0	34	20	2	695.88336	1390.75945	-15.59	39.20	4382	4382	MS2	15/20	Glioma_Nuclear_prep_Gradeall_4plex.raw
4506903		High	sHEGETSYIR	Unambiguous	1	1	N-Term(iTRAQ4plex)	HCD	1.0000	0.0000	1	1	6969	Unique	Used	1.581	1.337E4	2.863E4	156	2.92	0.00	0	37	178	2	661.82452	1322.64177	-3.00	14.49	1235	1235	MS2	13/18	Glioma_Nuclear_prep_Gradeall_repeat_4plex.raw
4506903		High	dAEDAIYGR	Unambiguous	1	1	N-Term(iTRAQ4plex)	HCD	1.0000	0.0000	1	1	8119	Unique	Used	1.602	2.318E4	5.028E4	111	3.51	0.00	0	31	184	2	577.27875	1153.55022	-9.02	24.92	2451	2451	MS2	15/16	Glioma_Nuclear_prep_Gradeall_repeat_4plex.raw
4506903		High	iYVGNLPTDVR	Unambiguous	1	1	N-Term(iTRAQ4plex)	HCD	1.0000	0.0000	1	1	10457	Unique	Used	1.719	8.164E3	1.901E4	319	3.98	0.00	0	32	300	2	695.89502	1390.78276	1.18	50.51	4942	4942	MS2	15/20	Glioma_Nuclear_prep_Gradeall_repeat_4plex.raw
4506903		High	dAEDAIYGR	Unambiguous	1	1	N-Term(iTRAQ4plex)	HCD	1.0000	0.0000	1	1	8128	Unique	Used	1.728	2.978E4	6.971E4	125	3.34	0.00	0	16	80	2	577.28339	1153.55950	-0.98	25.02	2461	2461	MS2	15/16	Glioma_Nuclear_prep_Gradeall_repeat_4plex.raw
4506903		High	hGLVPFAFVR	Unambiguous	1	1	N-Term(iTRAQ4plex)	HCD	1.0000	0.0000	1	1	11199	Unique	Used	2.705	6.577E2	2.410E3	172	3.58	0.00	0	28	300	3	429.58792	1286.74921	0.13	59.79	5761	5761	MS2	16/36	Glioma_Nuclear_prep_Gradeall_repeat_4plex.raw
4506903	High		eAGDVcYADVQk	Unambiguous	1	1	N-Term(iTRAQ4plex); C6(Methylthio); K12(iTRAQ4plex)	HCD	1.0000	0.0000	1	1	9037	Unique	Used	1.844	6.447E4	1.495E5	40	4.77	0.00	0	8	21	2	816.37311	1631.73894	-15.85	33.79	3579	3579	MS2	21/22	Glioma_Nuclear_prep_Gradeall_4plex.raw
4506903		High	eAGDVcYADVQk	Unambiguous	1	1	N-Term(iTRAQ4plex); C6(Methylthio); K12(iTRAQ4plex)	HCD	1.0000	0.0000	1	1	9468	Unique	Used	1.799	3.057E3	7.449E3	75	4.98	0.00	0	0	300	2	816.38586	1631.76445	-0.21	38.87	3877	3877	MS2	22/22	Glioma_Nuclear_prep_Gradeall_repeat_4plex.raw
4506903		High	vLVSGLPPSGSWQDLk	Unambiguous	1	1	N-Term(iTRAQ4plex); K16(iTRAQ4plex)	HCD	1.0000	0.0000	1	1	11851	Unique	Used	1.410	2.466E2	4.709E2	131	3.59	0.00	0	27	300	3	657.70984	1971.11496	-0.23	69.46	6573	6573	MS2	19/60	Glioma_Nuclear_prep_Gradeall_repeat_4plex.raw
4506903	High		ekDLEDLFYk	Unambiguous	1	1	N-Term(iTRAQ4plex); K2(iTRAQ4plex); K10(iTRAQ4plex)	HCD	1.0000	0.0000	1	1	10068	Unique	Used	1.722	1.129E5	2.446E5	213	3.83	0.00	1	18	12	2	866.46814	1731.92900	-13.83	41.34	4703	4703	MS2	17/18	Glioma_Nuclear_prep_Gradeall_4plex.raw
4506903	High		dLEDLFYk	Unambiguous	1	1	N-Term(iTRAQ4plex); K8(iTRAQ4plex)	HCD	1.0000	0.0000	1	1	10865	Unique	Used	1.713	5.476E4	1.180E5	162	3.49	0.00	0	67	38	2	665.84961	1330.69194	-16.06	47.41	5574	5574	MS2	14/14	Glioma_Nuclear_prep_Gradeall_4plex.raw
4506903		High	dLEDLFYk	Unambiguous	1	1	N-Term(iTRAQ4plex); K8(iTRAQ4plex)	HCD	1.0000	0.0000	1	1	11657	Unique	Used	1.992	5.769E3	1.556E4	275	3.63	0.00	0	43	300	2	665.86078	1330.71428	0.72	66.39	6319	6319	MS2	14/14	Glioma_Nuclear_prep_Gradeall_repeat_4plex.raw
4507127	NP_003084.1	SNRPC	6631	U1 small nuclear ribonucleoprotein C [Homo sapiens]	24.53	1	2	2	0.618	1;1	16.7	12.59	159	17.4	9.67																			
4507127	High		wMEEQAQSLIDk	Unambiguous	1	1	N-Term(iTRAQ4plex); K12(iTRAQ4plex)	HCD	1.0000	0.0000	1	1	10641	Unique	Used	0.691	8.240E4	7.158E4	164	5.66	0.00	0	24	36	2	883.44366	1765.88005	-13.20	45.70	5330	5330	MS2	22/22	Glioma_Nuclear_prep_Gradeall_4plex.raw
4507127		High	tTAAFQQGk	Unambiguous	1	1	N-Term(iTRAQ4plex); K9(iTRAQ4plex)	HCD	1.0000	0.0000	1	1	7315	Unique	Used	0.545	6.970E4	5.142E4	237	3.76	0.00	0	11	58	2	620.34906	1239.69084	-2.21	17.55	1601	1601	MS2	16/16	Glioma_Nuclear_prep_Gradeall_repeat_4plex.raw
4557321	NP_000030.1	APOA1	335	apolipoprotein A-I preproprotein [Homo sapiens]	24.72	1	6	11	2.036	7;4	17.1	31.76	267	30.8	5.76																			
4557321		High	ISPLGEEMR	Unambiguous	1	1	N-Term(iTRAQ4plex)	HCD	1.0000	0.0000	1	1	9384	Unique	Used	2.058	1.429E4	3.983E4	251	3.48	0.00	0	23	227	2	588.31262	1175.61797	-2.69	37.98	3788	3788	MS2	13/16	Glioma_Nuclear_prep_Gradeall_repeat_4plex.raw
4557321	High		vQPYLDDFQk	Unambiguous	1	1	N-Term(iTRAQ4plex); K10(iTRAQ4plex)	HCD	1.0000	0.0000	1	1	9704	Unique	Used	2.502	6.022E4	1.895E5	207	3.58	0.00	0	28	26	2	770.90442	1540.80156	-15.21	38.71	4308	4308	MS2	15/18	Glioma_Nuclear_prep_Gradeall_4plex.raw
4557321		High	vQPYLDDFQk	Unambiguous	1	1	N-Term(iTRAQ4plex); K10(iTRAQ4plex)	HCD	1.0000	0.0000	1	1	10395	Unique	Used	2.634	6.077E3	2.168E4	253	4.13	0.00	0	31	300	2	770.91699	1540.82671	1.11	49.77	4876	4876	MS2	15/18	Glioma_Nuclear_prep_Gradeall_repeat_4plex.raw
4557321	High		aTEHLSTLSEk	Unambiguous	1	1	N-Term(iTRAQ4plex); K11(iTRAQ4plex)	HCD	1.0000	0.0000	1	1	7647	Unique	Used	1.624	1.141E5	2.330E5	207	4.39	0.00	0	17	14	2	752.41486	1503.82244	-2.19	23.86	2070	2070	MS2	18/20	Glioma_Nuclear_prep_Gradeall_4plex.raw
4557321	High		qGLLPVLESFk	Unambiguous	1	1	N-Term(iTRAQ4plex); K11(iTRAQ4plex)	HCD	1.0000	0.0000	1	1	12051	Unique	Used	1.828	3.230E3	7.425E3	128	4.65	0.00	0	21	84	3	506.97488	1518.91010	-2.20	57.49	6868	6868	MS2	21/40	Glioma_Nuclear_prep_Gradeall_4plex.raw
4557321	High		qGLLPVLESFk	Unambiguous	1	1	N-Term(iTRAQ4plex); K11(iTRAQ4plex)	HCD	1.0000	0.0000	1	1	12061	Unique	Used	2.036	2.444E4	6.257E4	127	4.11	0.00	0	6	54	2	759.95996	1518.91265	-0.52	57.59	6879	6879	MS2	18/20	Glioma_Nuclear_prep_Gradeall_4plex.raw
4557321		High	qGLLPVLESFk	Unambiguous	1	1	N-Term(iTRAQ4plex); K11(iTRAQ4plex)	HCD	1.0000	0.0000	1	1	12399	Unique	Used	2.269	4.076E2	1.252E3	123	2.89	0.00	0	3	300	2	759.96045	1518.91362	0.12	83.45	7626	7626	MS2	14/20	Glioma_Nuclear_prep_Gradeall_repeat_4plex.raw
4557321	High		vSFLSALEEYTk	Unambiguous	1	1	N-Term(iTRAQ4plex); K12(iTRAQ4plex)	HCD	1.0000	0.0000	1	1	12533	Unique	Used	1.475	5.378E2	9.975E2	214	4.29	0.00	0	29	204	3	558.97760	1674.91825	-0.63	62.80	7404	7404	MS2	16/44	Glioma_Nuclear_prep_Gradeall_4plex.raw
4557321	High		vSFLSALEEYTk	Unambiguous	1	1	N-Term(iTRAQ4plex); K12(iTRAQ4plex)	HCD	1.0000	0.0000	1	1	12537	Unique	Used	1.963	8.540E3	2.109E4	221	5.74	0.00	0	19	237	2	837.96204	1674.91680	-1.49	62.86	7409	7409	MS2	20/22	Glioma_Nuclear_prep_Gradeall_4plex.raw
4557321	High		dYVSQFEGSALGk	Unambiguous	1	1	N-Term(iTRAQ4plex); K13(iTRAQ4plex)	HCD	1.0000	0.0000	1	1	10261	Unique	Used	2.139	7.440E4	2.001E5	170	5.00	0.00	0	16	14	2	844.92902	1688.85076	-13.42	42.80	4914	4914	MS2	22/24	Glioma_Nuclear_prep_Gradeall_4plex.raw
4557321		High	dYVSQFEGSALGk	Unambiguous	1	1	N-Term(iTRAQ4plex); K13(iTRAQ4plex)	HCD	1.0000	0.0000	1	1	10947	Unique	Used	1.840	1.954E3	4.872E3	181	5.05	0.00	0	37	300	2	844.94214	1688.87700	2.12	56.51	5474	5474	MS2	22/24	Glioma_Nuclear_prep_Gradeall_repeat_4plex.raw
4557759	NP_000241.1	MPO	4353	myeloperoxidase precursor [Homo sapiens]	9.26	1	5	6	4.077	5;1	30.5	22.16	745	83.8	8.97																			
4557759	High		vVLEGGIDPILR	Unambiguous	1	1	N-Term(iTRAQ4plex)	HCD	1.0000	0.0000	1	1	10715	Unique	Used	3.263	3.121E4	1.281E5	251	3.32	0.00	0	30	28	2	712.92328	1424.83928	-14.12	46.26	5411	5411	MS2	17/22	Glioma_Nuclear_prep_Gradeall_4plex.raw
4557759	High		iANVFTNAFR	Unambiguous	1	1	N-Term(iTRAQ4plex)	HCD	1.0000	0.0000	1	1	10946	Unique	Used	3.550	3.599E4	1.607E5	319	2.91	0.00	0	48	58	2	648.85291	1296.69853	-15.12	48.03	5662	5662	MS2	17/18	Glioma_Nuclear_prep_Gradeall_4plex.raw
4557759	High		iGLDLPALNMQR	Unambiguous	1	1	N-Term(iTRAQ4plex)	HCD	1.0000	0.0000	1	1	11317	Unique	Used	4.604	9.463E3	5.479E4	257	4.00	0.00	0	41	116	2	742.92310	1484.83891	0.87	51.01	6067	6067	MS2	18/22	Glioma_Nuclear_prep_Gradeall_4plex.raw
4557759	High		iIcDNTGITTVSk	Unambiguous	1	1	N-Term(iTRAQ4plex); C3(Methylthio); K13(iTRAQ4plex)	HCD	1.0000	0.0000	1	1	9938	Unique	Used	4.892	3.035E4	1.867E5	201	4.58	0.00	0	39	29	2	849.94196	1698.87663	-14.28	40.40	4562	4562	MS2	21/24	Glioma_Nuclear_prep_Gradeall_4plex.raw
4557759	High		nQADcIPFFR	Unambiguous	1	1	N-Term(iTRAQ4plex); C5(Methylthio)	HCD	1.0000	0.0000	1	1	11230	Unique	Used	1.987	1.274E4	3.185E4	84	3.64	0.00	0	68	180	2	700.83209	1400.65691	-0.21	50.27	5972	5972	MS2	14/18	Glioma_Nuclear_prep_Gradeall_4plex.raw
4557759		High	nQADcIPFFR	Unambiguous	1	1	N-Term(iTRAQ4plex); C5(Methylthio)	HCD	1.0000	0.0000	1	1	12022	Unique	Used	5.076	1.342E3	9.227E3	84	3.57	0.00	0	28	300	2	700.83264	1400.65801	0.58	72.41	6811	6811	MS2	13/18	Glioma_Nuclear_prep_Gradeall_repeat_4plex.raw
4758138	NP_004387.1	DDX5	1655	probable ATP-dependent RNA helicase DDX5 [Homo sapiens]	25.08	6	8	13	0.552	6;7	20.2	57.08	614	69.1	8.92																			
4758138	High		ILQLVEDR	Unambiguous	1	1	N-Term(iTRAQ4plex)	HCD	1.0000	0.0000	1	1	10349	Unique	Used	0.448	7.002E5	3.945E5	196	3.14	0.00	0	7	4	2	565.33069	1129.65410	-13.88	43.45	5010	5010	MS2	14/14	Glioma_Nuclear_prep_Gradeall_4plex.raw
4758138	High		tTYLVLDEADR	Unambiguous	1	1	N-Term(iTRAQ4plex)	HCD	1.0000	0.0000	1	1	9593	Unique	Used	0.583	3.629E5	2.662E5	169	2.98	0.00	0	23	7	2	720.36810	1439.72893	-14.55	37.92	4188	4188	MS2	16/20	Glioma_Nuclear_prep_Gradeall_4plex.raw
4758138		High	ILQLVEDR	Unambiguous	1	1	N-Term(iTRAQ4plex)	HCD	1.0000	0.0000	1	1	11231	Unique	Used	0.413	3.162E4	1.768E4	268	3.30	0.00	0	5	152	2	565.33978	1129.67229	2.22	60.20	5797	5797	MS2	14/14	Glioma_Nuclear_prep_Gradeall_repeat_4plex.raw
4758138		High	nFYQEHPDLAR	Unambiguous	1	1	N-Term(iTRAQ4plex)	HCD	1.0000	0.0000	1	1	8607	Unique	Used	0.524	1.197E4	8.502E3	175	4.21	0.00	0	37	108	3	511.92468	1533.75949	1.83	29.72	2966	2966	MS2	21/40	Glioma_Nuclear_prep_Gradeall_repeat_4plex.raw
4758138		High	tTYLVLDEADR	Unambiguous	1	1	N-Term(iTRAQ4plex)	HCD	1.0000	0.0000	1	1	10144	Unique	Used	0.552	1.267E4	9.473E3	260	4.32	0.00	0	27	300	2	720.38013	1439.75298	2.15	46.81	4609	4609	MS2	18/20	Glioma_Nuclear_prep_Gradeall_repeat_4plex.raw
4758138		High	fVINYDYPNSSEDYIHR	Unambiguous	1	1	N-Term(iTRAQ4plex)	HCD	1.0000	0.0000	1	1	11084	Unique	Used	0.687	4.716E2	4.390E2	105	4.40	0.00	0	55	300	3	759.36365	2276.07639	1.02	58.23	5626	5626	MS2	19/64	Glioma_Nuclear_prep_Gradeall_repeat_4plex.raw
4758138		High	qVSDLISVLR	Unambiguous	1	1	N-Term(iTRAQ4plex)	HCD	1.0000	0.0000	1	1	11777	Unique	Used	0.690	8.649E3	8.085E3	196	2.90	0.00	0	40	300	2	637.38483	1273.76238	2.13	68.17	6469	6469	MS2	14/18	Glioma_Nuclear_prep_Gradeall_repeat_4plex.raw
4758138	High		eLAQQVQQVAAEYcR	Unambiguous	1	1	N-Term(iTRAQ4plex); C14(Methylthio)	HCD	1.0000	0.0000	1	1	12581	Unique	Used	0.401	2.013E4	1.015E4	162	6.33	0.00	0	34	150	2	963.46948	1925.93169	-0.70	63.42	7459	7459	MS2	24/28	Glioma_Nuclear_prep_Gradeall_4plex.raw
4758138	High		eLAQQVQQVAAEYcR	Unambiguous	1	1	N-Term(iTRAQ4plex); C14(Methylthio)	HCD	1.0000	0.0000	1	1	12530	Unique	Used	0.485	1.088E4	6.640E3	172	6.13	0.00	0	13	300	2	963.47015	1925.93303	-0.01	62.75	7400	7400	MS2	24/28	Glioma_Nuclear_prep_Gradeall_4plex.raw
4758138	High		eLAQQVQQVAAEYcR	Unambiguous	1	1	N-Term(iTRAQ4plex); C14(Methylthio)	HCD	1.0000	0.0000	1	1	12570	Unique	Used	0.683	1.020E3	8.765E2	156	6.15	0.00	0	45	293	3	642.64911	1925.93277	-0.14	63.26	7445	7445	MS2	22/56	Glioma_Nuclear_prep_Gradeall_4plex.raw
4758138	High		IIDFLEcGk	Unambiguous	1	1	N-Term(iTRAQ4plex); C7(Methylthio); K9(iTRAQ4plex)	HCD	1.0000	0.0000	1	1	12296	Unique	Used	0.496	1.923E5	1.200E5	202	3.75	0.00	0	27	32	2	686.36743	1371.72759	1.52	60.00	7138	7138	MS2	14/16	Glioma_Nuclear_prep_Gradeall_4plex.raw
4758138		High	IIDFLEcGk	Unambiguous	1	1	N-Term(iTRAQ4plex); C7(Methylthio); K9(iTRAQ4plex)	HCD	1.0000	0.0000	1	1	12428	Unique	Used	0.559	3.346E3	2.532E3	197	3.36	0.00	0	42	300	2	686.36798	1371.72869	2.32	85.65	7774	7774	MS2	14/16	Glioma_Nuclear_prep_Gradeall_repeat_4plex.raw
4758138		High	tIVFVETk	Unambiguous	1	1	N-Term(iTRAQ4plex); K8(iTRAQ4plex)	HCD	1.0000	0.0000	1	1	9624	Unique	Used	0.743	1.566E4	1.576E4	205	3.53	0.00	0	62	300	2	612.87482	1224.74236	-1.53	40.67	4049	4049	MS2	13/14	Glioma_Nuclear_prep_Gradeall_repeat_4plex.raw
4758876	NP_004634.1	PABPN1	8106	polyadenylate-binding protein 2 [Homo sapiens]	9.15	4	3	4	0.567	1;3	11.7	11.38	306	32.7	5.06																			
4758876		High	tSLALDESLFR	Unambiguous	2	1	N-Term(iTRAQ4plex)	HCD	1.0000	0.0000	1	1	11514	Unique	Used	0.533	7.059E3	5.095E3	344	4.21	0.00	0	14	300	2	698.38568	1395.76409	2.88	64.32	6144	6144	MS2	17/20	Glioma_Nuclear_prep_Gradeall_repeat_4plex.raw
4758876		High	vTILcDk	Unambiguous	4	1	N-Term(iTRAQ4plex); C5(Methylthio); K7(iTRAQ4plex)	HCD	1.0000	0.0000	1	1	10299	Unique	Used	0.601	1.551E4	1.262E4	284	2.96	0.00	0	53	300	2	563.31512	1125.62297	-1.84	48.65	4774	4774	MS2	12/12	Glioma_Nuclear_prep_Gradeall_repeat_4plex.raw
4758876	High		gFAYIEFSDk	Unambiguous	2	1	N-Term(iTRAQ4plex); K10(iTRAQ4plex)	HCD	1.0000	0.0000	1	1	10297	Unique	Used	0.516	1.643E5	1.067E5	159	3.75	0.00	0	41	20	2	732.87317	1464.73906	-15.21	43.06	4953	4953	MS2	17/18	Glioma_Nuclear_prep_Gradeall_4plex.raw
4758876		High	gFAYIEFSDk	Unambiguous	2	1	N-Term(iTRAQ4plex); K10(iTRAQ4plex)	HCD	1.0000	0.0000	1	1	11155	Unique	Used	0.664	3.317E3	2.983E3	220	4.21	0.00	0	53	300	2	732.88672	1464.76616	3.29	59.19	5709	5709	MS2	18/18	Glioma_Nuclear_prep_Gradeall_repeat_4plex.raw
4758950	NP_000933.1	PPIB	5479	peptidyl-prolyl cis-trans isomerase B precursor [Homo sapiens]	14.81	1	3	5	2.399	3;2	19.5	12.70	216	23.7	9.41																			
4758950	High		tVDNFVALATGEk	Unambiguous	1	1	N-Term(iTRAQ4plex); K13(iTRAQ4plex)	HCD	1.0000	0.0000	1	1	10866	Unique	Used	1.719	3.351E4	7.244E4	247	4.81	0.00	0	33	44	2	826.94623	1652.88518	-14.91	47.41	5575	5575	MS2	22/24	Glioma_Nuclear_prep_Gradeall_4plex.raw
4758950	High		iEVEkPFAIAk	Unambiguous	1	1	N-Term(iTRAQ4plex); K5(iTRAQ4plex); K11(iTRAQ4plex)	HCD	1.0000	0.0000	1	1	9516	Unique	Used	2.929	6.192E4	2.281E5	133	4.79	0.00	0	12	15	2	839.00519	1677.00310	-16.73	37.36	4104	4104	MS2	19/20	Glioma_Nuclear_prep_Gradeall_4plex.raw
4758950		High	iEVEkPFAIAk	Unambiguous	1	1	N-Term(iTRAQ4plex); K5(iTRAQ4plex); K11(iTRAQ4plex)	HCD	1.0000	0.0000	1	1	10284	Unique	Used	2.297	1.960E3	6.099E3	57	4.33	0.00	0	50	300	2	839.02039	1677.03349	1.39	48.45	4757	4757	MS2	19/20	Glioma_Nuclear_prep_Gradeall_repeat_4plex.raw
4758950	High		vIFGLFGk	Unambiguous	1	1	N-Term(iTRAQ4plex); K8(iTRAQ4plex)	HCD	1.0000	0.0000	1	1	11570	Unique	Used	2.399	4.998E4	1.508E5	132	3.09	0.00	0	40	33	2	584.86908	1168.73088	-2.05	53.17	6344	6344	MS2	13/14	Glioma_Nuclear_prep_Gradeall_4plex.raw
4758950		High	vIFGLFGk	Unambiguous	1	1	N-Term(iTRAQ4plex); K8(iTRAQ4plex)	HCD	1.0000	0.0000	1	1	12214	Unique	Used	2.772	1.785E3	6.700E3	143	3.10	0.00	0	59	300	2	584.86835	1168.72942	-3.30	76.51	7129	7129	MS2	13/14	Glioma_Nuclear_prep_Gradeall_repeat_4plex.raw
4759302	NP_004729.1	VAPB	9217	vesicle-associated membrane protein-associated protein B/C isoform 1 [Homo sapiens]	15.64	2	1	2	0.505	1;1	6.9	13.73	243	27.2	7.30																			
4759302	High		sLSSSLDDTEVk	Unambiguous	1	1	N-Term(iTRAQ4plex); K12(iTRAQ4plex)	HCD	1.0000	0.0000	1	1	8685	Unique	Used	0.530	1.566E5	1.043E5	212	4.97	0.00	0	39	19	2	784.90491	1568.80254	-14.83	31.26	3197	3197	MS2	22/22	Glioma_Nuclear_prep_Gradeall_4plex.raw
4759302		High	sLSSSLDDTEVk	Unambiguous	1	1	N-Term(iTRAQ4plex); K12(iTRAQ4plex)	HCD	1.0000	0.0000	1	1	9072	Unique	Used	0.481	1.130E4	7.356E3	257	5.03	0.00	0	20	275	2	784.91711	1568.82695	0.74	34.55	3459	3459	MS2	22/22	Glioma_Nuclear_prep_Gradeall_repeat_4plex.raw
4826972	NP_005096.1	RBM8A	9939	RNA-binding protein 8A [Homo sapiens]	21.84	1	2	4	2.238	2;2	8.9	14.31	174	19.9	5.72																			
4826972	High		gYTLVEYETYk	Unambiguous	1	1	N-Term(iTRAQ4plex); K11(iTRAQ4plex)	HCD	1.0000	0.0000	1	1	9798	Unique	Used	2.460	1.126E5	3.484E5	166	4.95	0.00	0	39	12	2	827.42297	1653.83867	-13.75	39.39	4410	4410	MS2	19/20	Glioma_Nuclear_prep_Gradeall_4plex.raw
4826972		High	gYTLVEYETYk	Unambiguous	1	1	N-Term(iTRAQ4plex); K11(iTRAQ4plex)	HCD	1.0000	0.0000	1	1	10525	Unique	Used	2.090	7.794E3	2.206E4	214	4.90	0.00	0	21	202	2	827.43604	1653.86479	2.04	51.35	5016	5016	MS2	19/20	Glioma_Nuclear_prep_Gradeall_repeat_4plex.raw
4826972	High		fAEYGEIk	Unambiguous	1	1	N-Term(iTRAQ4plex); K8(iTRAQ4plex)	HCD	1.0000	0.0000	1	1	8828	Unique	Used	2.068	1.154E5	3.001E5	161	3.03	0.00	0	21	24	2	622.83051	1244.65373	-18.32	32.29	3352	3352	MS2	14/14	Glioma_Nuclear_prep_Gradeall_4plex.raw
4826972		High	fAEYGEIk	Unambiguous	1	1	N-Term(iTRAQ4plex); K8(iTRAQ4plex)	HCD	1.0000	0.0000	1	1	9330	Unique	Used	2.386	1.868E4	6.036E4	209	3.28	0.00	0	12	295	2	622.84210	1244.67693	0.31	37.41	3731	3731	MS2	14/14	Glioma_Nuclear_prep_Gradeall_repeat_4plex.raw
4826998	NP_005057.1	SFPQ	6421	splicing factor, proline- and glutamine-rich [Homo sapiens]	27.72	1	13	28	0.638	15;13	19.7	134.65	707	76.1	9.44																			
4826998	High		fGQGGAGPVGGQGPR	Unambiguous	1	1	N-Term(iTRAQ4plex)	HCD	1.0000	0.0000	1	1	7871	Unique	Used	0.437	4.284E5	2.354E5	199	4.82	0.00	0	8	8	2	743.38818	1485.76909	0.75	25.45	2313	2313	MS2	24/28	Glioma_Nuclear_prep_Gradeall_4plex.raw
4826998	High		fAQHGTFEYEYSQR	Unambiguous	1	1	N-Term(iTRAQ4plex)	HCD	1.0000	0.0000	1	1	8719	Unique	Used	0.543	1.067E5	7.288E4	49	4.89	0.00	0	23	16	2	953.93140	1906.85552	-14.98	31.51	3234	3234	MS2	20/26	Glioma_Nuclear_prep_Gradeall_4plex.raw
4826998	High		fATHAAALSVR	Unambiguous	1	1	N-Term(iTRAQ4plex)	HCD	1.0000	0.0000	1	1	8079	Unique	Used	0.644	2.339E5	1.894E5	283	3.72	0.00	0	19	13	2	644.35724	1287.70720	-16.96	26.91	2538	2538	MS2	18/20	Glioma_Nuclear_prep_Gradeall_4plex.raw
4826998	High		qHHPPYHQQHHQGPPPGGPGGR	Unambiguous	1	1	N-Term(iTRAQ4plex)	HCD	1.0000	0.0000	1	1	6874	Unique	Used	0.697	3.727E2	3.269E2	78	5.12	0.00	0	8	300	3	849.75104	2547.23856	0.54	17.08	1226	1226	MS2	23/84	Glioma_Nuclear_prep_Gradeall_4plex.raw
4826998	High		nLSPYVSNELLEEAFSQFGPIER	Unambiguous	1	1	N-Term(iTRAQ4plex)	HCD	1.0000	0.0000	1	1	13108	Unique	Used	0.720	1.036E3	9.382E2	107	5.80	0.00	0	0	300	2	1392.20593	2783.40459	1.31	73.67	8257	8257	MS2	27/44	Glioma_Nuclear_prep_Gradeall_4plex.raw
4826998	High		nLSPYVSNELLEEAFSQFGPIER	Unambiguous	1	1	N-Term(iTRAQ4plex)	HCD	1.0000	0.0000	1	1	13102	Unique	Used	0.778	7.133E1	6.976E1	124	6.77	0.00	0	13	300	3	928.47748	2783.41788	6.08	73.45	8242	8242	MS2	35/88	Glioma_Nuclear_prep_Gradeall_4plex.raw
4826998		High	fGQGGAGPVGGQGPR	Unambiguous	1	1	N-Term(iTRAQ4plex)	HCD	1.0000	0.0000	1	1	8034	Unique	Used	0.478	4.335E4	2.808E4	167	4.90	0.00	0	9	66	2	743.38483	1485.76238	-3.77	24.12	2362	2362	MS2	25/28	Glioma_Nuclear_prep_Gradeall_repeat_4plex.raw
4826998		High	fAQHGTFEYEYSQR	Unambiguous	1	1	N-Term(iTRAQ4plex)	HCD	1.0000	0.0000	1	1	9290	Unique	Used	0.633	4.027E3	3.452E3	83	4.89	0.00	0	13	300	2	953.94720	1906.88713	1.60	36.97	3689	3689	MS2	20/26	Glioma_Nuclear_prep_Gradeall_repeat_4plex.raw
4826998		High	fATHAAALSVR	Unambiguous	1	1	N-Term(iTRAQ4plex)	HCD	1.0000	0.0000	1	1	8434	Unique	Used	0.669	4.043E4	3.661E4	271	3.69	0.00	0	11	132	2	644.37061	1287.73393	3.81	27.97	2783	2783	MS2	18/20	Glioma_Nuclear_prep_Gradeall_repeat_4plex.raw
4826998		High	fAQHGTFEYEYSQR	Unambiguous	1	1	N-Term(iTRAQ4plex)	HCD	1.0000	0.0000	1	1	9282	Unique	Used	0.724	3.606E3	3.536E3	51	5.60	0.00	0	22	146	3	636.30115	1906.88889	2.52	36.89	3681	3681	MS2	22/52	Glioma_Nuclear_prep_Gradeall_repeat_4plex.raw
4826998	High		yGEPGEVFINk	Unambiguous	1	1	N-Term(iTRAQ4plex); K11(iTRAQ4plex)	HCD	1.0000	0.0000	1	1	9473	Unique	Used	0.730	4.329E5	3.974E5	189	3.45	0.00	0	15	6	2	770.90540	1540.80351	-13.95	37.06	4058	4058	MS2	20/20	Glioma_Nuclear_prep_Gradeall_4plex.raw
4826998	High		mEELHNQEMQk	Unambiguous	1	1	N-Term(iTRAQ4plex); K11(iTRAQ4plex)	HCD	1.0000	0.0000	1	1	7766	Unique	Used	0.772	1.186E5	1.151E5	89	5.15	0.00	0	33	13	2	852.91797	1704.82866	-0.09	24.70	2199	2199	MS2	19/20	Glioma_Nuclear_prep_Gradeall_4plex.raw
4826998		High	mEELHNQEMQk	Unambiguous	1	1	N-Term(iTRAQ4plex); K11(iTRAQ4plex)	HCD	1.0000	0.0000	1	1	7970	Unique	Used	0.668	2.671E3	2.417E3	98	3.32	0.00	0	0	300	2	852.91644	1704.82561	-1.88	23.52	2294	2294	MS2	20/20	Glioma_Nuclear_prep_Gradeall_repeat_4plex.raw
4826998		High	mEELHNQEMQk	Unambiguous	1	1	N-Term(iTRAQ4plex); K11(iTRAQ4plex)	HCD	1.0000	0.0000	1	1	7957	Unique	Used	0.728	5.737E3	5.659E3	83	4.27	0.00	0	23	115	3	568.94672	1704.82560	-1.88	23.39	2281	2281	MS2	21/40	Glioma_Nuclear_prep_Gradeall_repeat_4plex.raw
4826998		High	yGEPGEVFINk	Unambiguous	1	1	N-Term(iTRAQ4plex); K11(iTRAQ4plex)	HCD	1.0000	0.0000	1	1	10107	Unique	Used	0.764	4.503E3	4.663E3	234	3.36	0.00	0	18	80	3	514.28033	1540.82645	0.94	46.39	4570	4570	MS2	16/40	Glioma_Nuclear_prep_Gradeall_repeat_4plex.raw
4826998	High		rMEELHNQEMQk	Unambiguous	1	1	N-Term(iTRAQ4plex); K12(iTRAQ4plex)	HCD	1.0000	0.0000	1	1	7440	Unique	Used	0.656	7.512E4	6.197E4	147	4.90	0.00	1	7	16	2	930.96503	1860.92278	-3.84	22.39	1846	1846	MS2	19/22	Glioma_Nuclear_prep_Gradeall_4plex.raw
4826998	High		IFVGNLPADITEDEFk	Unambiguous	1	1	N-Term(iTRAQ4plex); K16(iTRAQ4plex)	HCD	1.0000	0.0000	1	1	12126	Unique	Used	0.462	1.333E4	7.749E3	194	5.23	0.00	0	6	22	3	699.38196	2096.13132	7.56	58.28	6951	6951	MS2	26/60	Glioma_Nuclear_prep_Gradeall_4plex.raw
4826998	High		IFVGNLPADITEDEFk	Unambiguous	1	1	N-Term(iTRAQ4plex); K16(iTRAQ4plex)	HCD	1.0000	0.0000	1	1	12127	Unique	Used	0.529	5.699E4	3.793E4	192	5.69	0.00	0	4	50	2	1048.56824	2096.12920	6.55	58.28	6952	6952	MS2	22/30	Glioma_Nuclear_prep_Gradeall_4plex.raw
4826998	High		IFVGNLPADITEDEFk	Unambiguous	1	1	N-Term(iTRAQ4plex); K16(iTRAQ4plex)	HCD	1.0000	0.0000	1	1	12060	Unique	Used	0.563	1.242E5	8.787E4	204	6.23	0.00	0	5	30	2	1048.56287	2096.11846	1.42	57.59	6878	6878	MS2	22/30	Glioma_Nuclear_prep_Gradeall_4plex.raw
4826998	High		IFVGNLPADITEDEFk	Unambiguous	1	1	N-Term(iTRAQ4plex); K16(iTRAQ4plex)	HCD	1.0000	0.0000	1	1	12059	Unique	Used	0.740	1.835E4	1.708E4	196	5.83	0.00	0	6	14	3	699.37988	2096.12510	4.59	57.58	6877	6877	MS2	27/60	Glioma_Nuclear_prep_Gradeall_4plex.raw
4826998		High	IFVGNLPADITEDEFk	Unambiguous	1	1	N-Term(iTRAQ4plex); K16(iTRAQ4plex)	HCD	1.0000	0.0000	1	1	12395	Unique	Used	0.507	9.180E1	6.309E1	177	4.45	0.00	0	11	300	3	699.37811	2096.11979	2.06	83.20	7609	7609	MS2	23/60	Glioma_Nuclear_prep_Gradeall_repeat_4plex.raw
4826998		High	IFVGNLPADITEDEFk	Unambiguous	1	1	N-Term(iTRAQ4plex); K16(iTRAQ4plex)	HCD	1.0000	0.0000	1	1	12384	Unique	Used	0.529	1.535E3	1.101E3	194	5.50	0.00	0	2	300	2	1048.56165	2096.11601	0.26	82.30	7548	7548	MS2	22/30	Glioma_Nuclear_prep_Gradeall_repeat_4plex.raw
4826998		High	IFVGNLPADITEDEFk	Unambiguous	1	1	N-Term(iTRAQ4plex); K16(iTRAQ4plex)	HCD	1.0000	0.0000	1	1	12379	Unique	Used	0.567	2.349E2	1.803E2	192	4.97	0.00	0	12	300	3	699.37775	2096.11869	1.53	82.18	7539	7539	MS2	25/60	Glioma_Nuclear_prep_Gradeall_repeat_4plex.raw
4826998	High		mGGGGAMNMGDPYGSGGQk	Unambiguous	1	1	N-Term(iTRAQ4plex); K19(iTRAQ4plex)	HCD	1.0000	0.0000	1	1	8718	Unique	Used	0.356	1.180E5	5.282E4	19	6.86	0.00	0	0	14	2	1030.44849	2059.88970	-16.60	31.50	3233	3233	MS2	24/36	Glioma_Nuclear_prep_Gradeall_4plex.raw
4826998		High	mGGGGAMNMGDPYGSGGQk	Unambiguous	1	1	N-Term(iTRAQ4plex); K19(iTRAQ4plex)	HCD	1.0000	0.0000	1	1	9048	Unique	Used	0.473	1.930E3	1.237E3	43	4.69	0.00	0	10	300	2	1030.46448	2059.92168	-1.08	34.28	3433	3433	MS2	24/36	Glioma_Nuclear_prep_Gradeall_repeat_4plex.raw
4826998	High		dkLESEMEDAYHEHQANLLR	Unambiguous	1	1	N-Term(iTRAQ4plex); K2(iTRAQ4plex)	HCD	1.0000	0.0000	1	1	9924	Unique	Used	0.630	9.345E3	7.404E3	53	3.63	0.00	1	5	9	3	906.10065	2716.28739	-13.47	40.30	4547	4547	MS2	29/76	Glioma_Nuclear_prep_Gradeall_4plex.raw
4826998		High	eEYEGPNk	Unambiguous	1	1	N-Term(iTRAQ4plex); K8(iTRAQ4plex)	HCD	1.0000	0.0000	1	1	7194	Unique	Used	0.437	6.112E4	3.620E4	108	3.00	0.00	0	14	51	2	627.30585	1253.60442	-16.60	16.49	1474	1474	MS2	13/14	Glioma_Nuclear_prep_Gradeall_repeat_4plex.raw
4826998		High	iSDSEGFk	Unambiguous	1	1	N-Term(iTRAQ4plex); K8(iTRAQ4plex)	HCD	1.0000	0.0000	1	1	8169	Unique	Used	0.707	9.243E4	8.857E4	181	3.06	0.00	0	17	58	2	585.81378	1170.62029	-3.61	25.41	2504	2504	MS2	14/14	Glioma_Nuclear_prep_Gradeall_repeat_4plex.raw
4885373	NP_005316.1	HIST1H1A	3024	histone H1.1 [Homo sapiens]	32.09	2	3	3	2.124	2;1	28.3	69.48	215	21.8	10.99																			
4885373	High		kkPAGPSVSELIVQAASSSk	Unambiguous	1	1	N-Term(iTRAQ4plex); K1(iTRAQ4plex); K2(iTRAQ4plex); K20(iTRAQ4plex)	HCD	1.0000	0.0000	1	1	9711	Unique	Used	1.298	1.501E4	2.451E4	35	5.83	0.00	1	11	3	3	854.16431	2560.47837	-14.57	38.77	4316	4316	MS2	24/76	Glioma_Nuclear_prep_Gradeall_4plex.raw
4885373	High		aSSVETkPGASk	Unambiguous	1	1	N-Term(iTRAQ4plex); K7(iTRAQ4plex); K12(iTRAQ4plex)	HCD	1.0000	0.0000	1	1	7156	Unique	Used	2.124	2.178E3	5.821E3	190	4.30	0.00	0	12	168	2	797.46027	1593.91326	-2.50	20.20	1535	1535	MS2	18/22	Glioma_Nuclear_prep_Gradeall_4plex.raw
4885373		High	gGVSLAALk	Unambiguous	1	1	N-Term(iTRAQ4plex); K9(iTRAQ4plex)	HCD	1.0000	0.0000	1	1	9507	Unique	Used	2.317	5.445E3	1.709E4	238	3.42	0.00	0	29	126	2	552.35126	1103.69524	-6.77	39.34	3921	3921	MS2	15/16	Glioma_Nuclear_prep_Gradeall_repeat_4plex.raw
4885375	NP_005310.1	HIST1H1C	3006	histone H1.2 [Homo sapiens]	36.62	2	3	10	3.483	5;5	29.1	188.38	213	21.4	10.93																			
4885375	High		kAAGGATPk	Unambiguous	1	1	N-Term(iTRAQ4plex); K1(iTRAQ4plex); K9(iTRAQ4plex)	HCD	1.0000	0.0000	1	1	6736	Unique	Used	2.744	7.379E2	2.547E3	149	3.53	0.00	1	54	300	3	411.59396	1232.76734	-1.12	15.20	1061	1061	MS2	26/32	Glioma_Nuclear_prep_Gradeall_4plex.raw
4885375	High		kPAAATVTk	Unambiguous	1	1	N-Term(iTRAQ4plex); K1(iTRAQ4plex); K9(iTRAQ4plex)	HCD	1.0000	0.0000	1	1	7200	Unique	Used	3.465	9.019E4	3.931E5	111	2.95	0.00	0	10	7	2	659.92352	1318.83977	-1.60	20.56	1583	1583	MS2	15/16	Glioma_Nuclear_prep_Gradeall_4plex.raw
4885375	High		kPAAATVTk	Unambiguous	1	1	N-Term(iTRAQ4plex); K1(iTRAQ4plex); K9(iTRAQ4plex)	HCD	1.0000	0.0000	1	1	7198	Unique	Used	3.501	5.368E4	2.364E5	117	4.61	0.00	0	11	3	3	440.28479	1318.83982	-1.56	20.55	1581	1581	MS2	23/32	Glioma_Nuclear_prep_Gradeall_4plex.raw
4885375	High		kPAAATVTk	Unambiguous	1	1	N-Term(iTRAQ4plex); K1(iTRAQ4plex); K9(iTRAQ4plex)	HCD	1.0000	0.0000	1	1	7163	Unique	Used	4.364	4.943E4	2.713E5	157	3.11	0.00	0	15	31	2	659.91516	1318.82305	-14.28	20.27	1543	1543	MS2	14/16	Glioma_Nuclear_prep_Gradeall_4plex.raw
4885375	High		kPAAATVTk	Unambiguous	1	1	N-Term(iTRAQ4plex); K1(iTRAQ4plex); K9(iTRAQ4plex)	HCD	1.0000	0.0000	1	1	7161	Unique	Used	4.988	1.717E4	1.077E5	170	4.43	0.00	0	14	10	3	440.27896	1318.82233	-14.82	20.25	1541	1541	MS2	23/32	Glioma_Nuclear_prep_Gradeall_4plex.raw
4885375		High	kPAAATVTk	Unambiguous	1	1	N-Term(iTRAQ4plex); K1(iTRAQ4plex); K9(iTRAQ4plex)	HCD	1.0000	0.0000	1	1	7032	Unique	Used	1.344	6.980E3	1.270E4	124	4.50	0.00	0	56	112	3	440.28391	1318.83716	-3.58	15.08	1301	1301	MS2	22/32	Glioma_Nuclear_prep_Gradeall_repeat_4plex.raw
4885375		High	kPAAATVTk	Unambiguous	1	1	N-Term(iTRAQ4plex); K1(iTRAQ4plex); K9(iTRAQ4plex)	HCD	1.0000	0.0000	1	1	6860	Unique	Used	3.395	1.191E4	5.476E4	124	4.62	0.00	0	39	39	3	440.28333	1318.83542	-4.90	13.53	1120	1120	MS2	23/32	Glioma_Nuclear_prep_Gradeall_repeat_4plex.raw
4885375		High	kPAAATVTk	Unambiguous	1	1	N-Term(iTRAQ4plex); K1(iTRAQ4plex); K9(iTRAQ4plex)	HCD	1.0000	0.0000	1	1	6868	Unique	Used	4.298	3.128E4	1.821E5	115	2.94	0.00	0	34	157	2	659.92169	1318.83611	-4.38	13.59	1128	1128	MS2	14/16	Glioma_Nuclear_prep_Gradeall_repeat_4plex.raw
4885375		High	kAAGGATPk	Unambiguous	1	1	N-Term(iTRAQ4plex); K1(iTRAQ4plex); K9(iTRAQ4plex)	HCD	1.0000	0.0000	1	1	6415	Unique	Used	4.516	3.698E3	2.262E4	188	4.45	0.00	1	28	58	3	411.59473	1232.76963	0.74	9.38	651	651	MS2	27/32	Glioma_Nuclear_prep_Gradeall_repeat_4plex.raw
4885375		High	aAGGATPk	Unambiguous	1	1	N-Term(iTRAQ4plex); K8(iTRAQ4plex)	HCD	1.0000	0.0000	1	1	6453	Unique	Used	3.288	1.625E4	7.238E4	228	2.93	0.00	0	7	91	2	480.78970	960.57213	0.46	9.77	691	691	MS2	13/14	Glioma_Nuclear_prep_Gradeall_repeat_4plex.raw
4885377	NP_005311.1	HIST1H1D	3007	histone H1.3 [Homo sapiens]	30.77	2	2	4	7.321	1;3	23.1	176.89	221	22.3	11.02																			
4885377	High		kVAGAATPk	Unambiguous	1	1	N-Term(iTRAQ4plex); K1(iTRAQ4plex); K9(iTRAQ4plex)	HCD	1.0000	0.0000	1	1	7019	Unique	Used	7.633	3.655E3	3.509E4	109	4.53	0.00	1	23	41	3	425.61038	1274.81659	0.72	18.75	1385	1385	MS2	23/32	Glioma_Nuclear_prep_Gradeall_4plex.raw
4885377		High	kPATAAGTk	Unambiguous	1	1	N-Term(iTRAQ4plex); K1(iTRAQ4plex); K9(iTRAQ4plex)	HCD	1.0000	0.0000	1	1	6539	Unique	Used	4.843	1.041E4	6.828E4	186	3.49	0.00	0	10	14	3	426.27023	1276.79615	0.95	10.61	782	782	MS2	19/32	Glioma_Nuclear_prep_Gradeall_repeat_4plex.raw
4885377		High	kVAGAATPk	Unambiguous	1	1	N-Term(iTRAQ4plex); K1(iTRAQ4plex); K9(iTRAQ4plex)	HCD	1.0000	0.0000	1	1	6656	Unique	Used	7.008	4.418E3	4.194E4	103	4.72	0.00	1	26	42	3	425.60880	1274.81183	-3.01	11.72	906	906	MS2	22/32	Glioma_Nuclear_prep_Gradeall_repeat_4plex.raw
4885377		High	kVAGAATPk	Unambiguous	1	1	N-Term(iTRAQ4plex); K1(iTRAQ4plex); K9(iTRAQ4plex)	HCD	1.0000	0.0000	1	1	6679	Unique	Used	8.705	1.524E4	1.798E5	104	2.89	0.00	1	5	26	2	637.90979	1274.81230	-2.64	11.93	930	930	MS2	15/16	Glioma_Nuclear_prep_Gradeall_repeat_4plex.raw
4885379	NP_005312.1	HIST1H1E	3008	histone H1.4 [Homo sapiens]	35.62	2	2	8	1.811	4;4	17.4	184.31	219	21.9	11.03																			

4885379	High		kATGAATPk	Unambiguous	1	1	N-Term(iTRAQ4plex); K1(iTRAQ4plex); K9(iTRAQ4plex)	HCD	1.0000	0.0000	1	1	6798	Unique	Used	1.312	9.606E2	1.585E3	133	4.36	0.00	1	48	300	3	426.26968	1276.79450	-0.34	16.12	1140	1140	MS2	22/32	Glioma_Nuclear_prep_Gradeall_4plex.raw
4885379	High		kPAAAAGAk	Unambiguous	1	1	N-Term(iTRAQ4plex); K1(iTRAQ4plex); K9(iTRAQ4plex)	HCD	1.0000	0.0000	1	1	6966	Unique	Used	1.772	5.317E3	1.185E4	155	4.10	0.00	0	24	91	3	406.26263	1216.77335	-0.38	18.14	1327	1327	MS2	23/32	Glioma_Nuclear_prep_Gradeall_4plex.raw
4885379	High		kPAAAAGAk	Unambiguous	1	1	N-Term(iTRAQ4plex); K1(iTRAQ4plex); K9(iTRAQ4plex)	HCD	1.0000	0.0000	1	1	6967	Unique	Used	1.851	1.002E4	2.333E4	141	2.90	0.00	0	20	154	2	608.89026	1216.77324	-0.47	18.14	1328	1328	MS2	15/16	Glioma_Nuclear_prep_Gradeall_4plex.raw
4885379	High		kPAAAAGAk	Unambiguous	1	1	N-Term(iTRAQ4plex); K1(iTRAQ4plex); K9(iTRAQ4plex)	HCD	1.0000	0.0000	1	1	6900	Unique	Used	2.365	3.815E3	1.135E4	156	3.96	0.00	0	44	157	3	406.26218	1216.77198	-1.51	17.38	1255	1255	MS2	21/32	Glioma_Nuclear_prep_Gradeall_4plex.raw
4885379		High	kPAAAAGAk	Unambiguous	1	1	N-Term(iTRAQ4plex); K1(iTRAQ4plex); K9(iTRAQ4plex)	HCD	1.0000	0.0000	1	1	6604	Unique	Used	1.676	5.772E4	1.310E5	150	4.11	0.00	0	8	11	3	406.26361	1216.77628	2.03	11.23	851	851	MS2	24/32	Glioma_Nuclear_prep_Gradeall_repeat_4plex.raw
4885379		High	kATGAATPk	Unambiguous	1	1	N-Term(iTRAQ4plex); K1(iTRAQ4plex); K9(iTRAQ4plex)	HCD	1.0000	0.0000	1	1	6471	Unique	Used	1.703	7.034E3	1.622E4	169	4.18	0.00	1	63	164	3	426.26987	1276.79505	0.09	9.95	710	710	MS2	20/32	Glioma_Nuclear_prep_Gradeall_repeat_4plex.raw
4885379		High	kPAAAAGAk	Unambiguous	1	1	N-Term(iTRAQ4plex); K1(iTRAQ4plex); K9(iTRAQ4plex)	HCD	1.0000	0.0000	1	1	6556	Unique	Used	2.030	8.903E4	2.448E5	173	4.13	0.00	0	11	7	3	406.25598	1216.75339	-16.78	10.75	800	800	MS2	22/32	Glioma_Nuclear_prep_Gradeall_repeat_4plex.raw
4885379		High	kPAAAAGAk	Unambiguous	1	1	N-Term(iTRAQ4plex); K1(iTRAQ4plex); K9(iTRAQ4plex)	HCD	1.0000	0.0000	1	1	6558	Unique	Used	2.150	1.387E5	4.040E5	157	2.95	0.00	0	4	18	2	608.88013	1216.75298	-17.12	10.76	802	802	MS2	15/16	Glioma_Nuclear_prep_Gradeall_repeat_4plex.raw
4885381	NP_005313.1	HIST1H1B	3009	histone H1.5 [Homo sapiens]	37.61	1	7	18	4.726	9;9	39.3	153.21	226	22.6	10.92																			
4885381	High		kALAAGGYDVEk	Unambiguous	1	1	N-Term(iTRAQ4plex); K1(iTRAQ4plex); K12(iTRAQ4plex)	HCD	1.0000	0.0000	1	1	7925	Unique	Used	3.150	1.075E5	4.261E5	224	5.45	0.00	1	33	2	3	551.98889	1653.95212	-0.91	25.84	2372	2372	MS2	26/44	Glioma_Nuclear_prep_Gradeall_4plex.raw
4885381		High	kALAAGGYDVEk	Unambiguous	1	1	N-Term(iTRAQ4plex); K1(iTRAQ4plex); K12(iTRAQ4plex)	HCD	1.0000	0.0000	1	1	8183	Unique	Used	5.211	6.946E3	4.903E4	214	5.96	0.00	1	12	19	3	551.98767	1653.94846	-3.13	25.56	2519	2519	MS2	28/44	Glioma_Nuclear_prep_Gradeall_repeat_4plex.raw
4885381	High		kATGPPVSELITk	Unambiguous	1	1	N-Term(iTRAQ4plex); K1(iTRAQ4plex); K13(iTRAQ4plex)	HCD	1.0000	0.0000	1	1	8681	Unique	Used	7.787	2.414E4	2.364E5	95	4.56	0.00	1	4	8	2	887.03210	1773.05693	-15.63	31.24	3193	3193	MS2	21/24	Glioma_Nuclear_prep_Gradeall_4plex.raw
4885381	High		kATGPPVSELITk	Unambiguous	1	1	N-Term(iTRAQ4plex); K1(iTRAQ4plex); K13(iTRAQ4plex)	HCD	1.0000	0.0000	1	1	8513	Unique	Used	8.229	1.712E4	1.772E5	117	5.41	0.00	1	7	2	3	591.68878	1773.05179	-18.53	30.00	3009	3009	MS2	22/48	Glioma_Nuclear_prep_Gradeall_4plex.raw
4885381		High	kATGPPVSELITk	Unambiguous	1	1	N-Term(iTRAQ4plex); K1(iTRAQ4plex); K13(iTRAQ4plex)	HCD	1.0000	0.0000	1	1	8610	Unique	Used	3.218	1.495E3	6.517E3	49	4.61	0.00	1	43	140	3	591.70020	1773.08603	0.78	29.74	2969	2969	MS2	18/48	Glioma_Nuclear_prep_Gradeall_repeat_4plex.raw
4885381		High	kATGPPVSELITk	Unambiguous	1	1	N-Term(iTRAQ4plex); K1(iTRAQ4plex); K13(iTRAQ4plex)	HCD	1.0000	0.0000	1	1	9147	Unique	Used	3.574	1.221E3	5.914E3	76	4.67	0.00	1	79	300	2	887.04419	1773.08110	-2.00	35.39	3538	3538	MS2	20/24	Glioma_Nuclear_prep_Gradeall_repeat_4plex.raw
4885381		High	kATGPPVSELITk	Unambiguous	1	1	N-Term(iTRAQ4plex); K1(iTRAQ4plex); K13(iTRAQ4plex)	HCD	1.0000	0.0000	1	1	9230	Unique	Used	5.648	3.850E2	2.945E3	52	5.17	0.00	1	6	89	4	444.02740	1773.08779	1.77	36.29	3626	3626	MS2	23/72	Glioma_Nuclear_prep_Gradeall_repeat_4plex.raw
4885381	High		kPAAAGVk	Unambiguous	1	1	N-Term(iTRAQ4plex); K1(iTRAQ4plex); K8(iTRAQ4plex)	HCD	1.0000	0.0000	1	1	7109	Unique	Used	4.843	2.360E3	1.437E4	83	3.43	0.00	0	43	108	3	391.92831	1173.77039	2.04	19.74	1484	1484	MS2	20/28	Glioma_Nuclear_prep_Gradeall_4plex.raw
4885381	High		kAAGAGAAk	Unambiguous	1	1	N-Term(iTRAQ4plex); K1(iTRAQ4plex); K9(iTRAQ4plex)	HCD	1.0000	0.0000	1	1	6725	Unique	Used	10.530	1.563E2	2.070E3	139	3.45	0.00	1	41	300	3	392.91870	1176.74155	-0.82	14.68	1023	1023	MS2	22/32	Glioma_Nuclear_prep_Gradeall_4plex.raw
4885381		High	aLAAGGYDVEk	Unambiguous	1	1	N-Term(iTRAQ4plex); K11(iTRAQ4plex)	HCD	1.0000	0.0000	1	1	8842	Unique	Used	5.205	6.042E3	4.259E4	236	4.34	0.00	0	20	120	2	691.38281	1381.75835	1.27	32.08	3213	3213	MS2	20/20	Glioma_Nuclear_prep_Gradeall_repeat_4plex.raw
4885381	High		aTGPPVSELITk	Unambiguous	1	1	N-Term(iTRAQ4plex); K12(iTRAQ4plex)	HCD	1.0000	0.0000	1	1	8539	Unique	Used	3.995	2.127E4	1.069E5	193	2.92	0.00	0	9	10	2	750.93536	1500.86345	-16.10	30.18	3037	3037	MS2	17/22	Glioma_Nuclear_prep_Gradeall_4plex.raw
4885381	High		aTGPPVSELITk	Unambiguous	1	1	N-Term(iTRAQ4plex); K12(iTRAQ4plex)	HCD	1.0000	0.0000	1	1	9290	Unique	Used	6.814	3.807E3	3.262E4	190	3.09	0.00	0	15	13	2	750.93335	1500.85942	-18.79	35.68	3857	3857	MS2	17/22	Glioma_Nuclear_prep_Gradeall_4plex.raw
4885381		High	aTGPPVSELITk	Unambiguous	1	1	N-Term(iTRAQ4plex); K12(iTRAQ4plex)	HCD	1.0000	0.0000	1	1	10193	Unique	Used	3.126	3.088E3	1.308E4	155	4.77	0.00	0	26	300	2	750.94843	1500.88957	1.30	47.39	4661	4661	MS2	21/22	Glioma_Nuclear_prep_Gradeall_repeat_4plex.raw
4885381		High	aTGPPVSELITk	Unambiguous	1	1	N-Term(iTRAQ4plex); K12(iTRAQ4plex)	HCD	1.0000	0.0000	1	1	9925	Unique	Used	4.535	2.579E3	1.584E4	164	4.28	0.00	0	39	300	2	750.94489	1500.88249	-3.41	44.11	4369	4369	MS2	20/22	Glioma_Nuclear_prep_Gradeall_repeat_4plex.raw
4885381	High		nGLSLAALk	Unambiguous	1	1	N-Term(iTRAQ4plex); K9(iTRAQ4plex)	HCD	1.0000	0.0000	1	1	9579	Unique	Used	3.843	1.246E5	6.021E5	256	3.47	0.00	0	18	4	2	587.86420	1174.72112	-15.92	37.83	4173	4173	MS2	16/16	Glioma_Nuclear_prep_Gradeall_4plex.raw
4885381	High		nGLSLAALk	Unambiguous	1	1	N-Term(iTRAQ4plex); K9(iTRAQ4plex)	HCD	1.0000	0.0000	1	1	9484	Unique	Used	4.609	1.279E5	7.412E5	254	3.79	0.00	0	24	5	2	587.86414	1174.72099	-16.02	37.14	4070	4070	MS2	16/16	Glioma_Nuclear_prep_Gradeall_4plex.raw
4885381		High	nGLSLAALk	Unambiguous	1	1	N-Term(iTRAQ4plex); K9(iTRAQ4plex)	HCD	1.0000	0.0000	1	1	10308	Unique	Used	3.274	4.280E3	1.898E4	183	4.14	0.00	0	33	300	2	587.87335	1174.73943	-0.33	48.76	4784	4784	MS2	16/16	Glioma_Nuclear_prep_Gradeall_repeat_4plex.raw
4885381		High	nGLSLAALk	Unambiguous	1	1	N-Term(iTRAQ4plex); K9(iTRAQ4plex)	HCD	1.0000	0.0000	1	1	10215	Unique	Used	5.577	5.890E2	4.449E3	155	3.59	0.00	0	12	206	3	392.25165	1174.74039	0.49	47.64	4684	4684	MS2	18/32	Glioma_Nuclear_prep_Gradeall_repeat_4plex.raw
5031755	NP_005817.1	HNRNPR	10236	heterogeneous nuclear ribonucleoprotein R isoform 2 [Homo sapiens]	36.33	7	16	41	0.216	23;18	39.0	179.51	633	70.9	8.13																			
5031755	High		sTAYEDYYYHPPPR	Unambiguous	7	1	N-Term(iTRAQ4plex)	HCD	1.0000	0.0000	1	1	8614	Unique	Used	0.124	1.003E5	1.567E4	33	5.67	0.00	0	0	21	2	951.92761	1902.84795	-15.74	30.75	3120	3120	MS2	23/26	Glioma_Nuclear_prep_Gradeall_4plex.raw
5031755	High		lMMDPLSGQNR	Unambiguous	6	1	N-Term(iTRAQ4plex)	HCD	1.0000	0.0000	1	1	9757	Unique	Used	0.223	8.422E5	2.362E5	121	2.97	0.00	0	15	4	2	703.34570	1405.68413	-14.77	39.10	4366	4366	MS2	15/20	Glioma_Nuclear_prep_Gradeall_4plex.raw
5031755	High		sTAYEDYYYHPPPR	Unambiguous	7	1	N-Term(iTRAQ4plex)	HCD	1.0000	0.0000	1	1	8609	Unique	Used	0.227	4.182E4	1.193E4	35	3.70	0.00	0	26	6	3	634.95398	1902.84739	-16.04	30.72	3115	3115	MS2	27/52	Glioma_Nuclear_prep_Gradeall_4plex.raw
5031755		High	sTAYEDYYYHPPPR	Unambiguous	7	1	N-Term(iTRAQ4plex)	HCD	1.0000	0.0000	1	1	9089	Unique	Used	0.137	6.727E3	1.249E3	51	6.05	0.00	0	0	300	2	951.94531	1902.88335	2.86	34.74	3477	3477	MS2	23/26	Glioma_Nuclear_prep_Gradeall_repeat_4plex.raw
5031755		High	sTAYEDYYYHPPPR	Unambiguous	7	1	N-Term(iTRAQ4plex)	HCD	1.0000	0.0000	1	1	9079	Unique	Used	0.216	5.850E3	1.711E3	55	4.86	0.00	0	14	133	3	634.96637	1902.88456	3.50	34.64	3467	3467	MS2	26/52	Glioma_Nuclear_prep_Gradeall_repeat_4plex.raw
5031755		High	lMMDPLSGQNR	Unambiguous	6	1	N-Term(iTRAQ4plex)	HCD	1.0000	0.0000	1	1	10386	Unique	Used	0.254	2.170E4	7.461E3	188	3.74	0.00	0	16	258	2	703.35767	1405.70806	2.25	49.68	4867	4867	MS2	15/20	Glioma_Nuclear_prep_Gradeall_repeat_4plex.raw
5031755		High	sTAYEDYYYHPPPR	Unambiguous	7	1	N-Term(iTRAQ4plex)	HCD	1.0000	0.0000	1	1	9186	Unique	Used	0.268	1.932E3	7.001E2	49	5.56	0.00	0	10	300	2	951.93958	1902.87187	-3.17	35.81	3579	3579	MS2	23/26	Glioma_Nuclear_prep_Gradeall_repeat_4plex.raw
5031755		High	sTAYEDYYYHPPPR	Unambiguous	7	1	N-Term(iTRAQ4plex)	HCD	1.0000	0.0000	1	1	9174	Unique	Used	0.337	3.866E3	1.766E3	44	4.63	0.00	0	12	115	3	634.96265	1902.87339	-2.37	35.68	3567	3567	MS2	27/52	Glioma_Nuclear_prep_Gradeall_repeat_4plex.raw
5031755		High	dYAFVHFEDR	Unambiguous	7	1	N-Term(iTRAQ4plex)	HCD	1.0000	0.0000	1	1	10110	Unique	Used	0.405	3.822E3	2.096E3	99	4.08	0.00	0	29	182	3	481.56610	1442.68375	1.12	46.41	4573	4573	MS2	21/36	Glioma_Nuclear_prep_Gradeall_repeat_4plex.raw
5031755	High		lcDSYEIRPGk	Unambiguous	6	1	N-Term(iTRAQ4plex); C2(Methylthio); K11(iTRAQ4plex)	HCD	1.0000	0.0000	1	1	9251	Unique	Used	0.324	8.473E4	3.453E4	88	3.41	0.00	0	21	31	2	807.90234	1614.79741	-15.38	35.37	3813	3813	MS2	16/20	Glioma_Nuclear_prep_Gradeall_4plex.raw
5031755	High		gYAFITFcGk	Unambiguous	6	1	N-Term(iTRAQ4plex); C8(Methylthio); K10(iTRAQ4plex)	HCD	1.0000	0.0000	1	1	11751	Unique	Used	0.173	1.620E5	3.525E4	181	4.02	0.00	0	53	34	2	720.86652	1440.72576	-0.04	54.80	6541	6541	MS2	15/18	Glioma_Nuclear_prep_Gradeall_4plex.raw
5031755	High		gYAFITFcGk	Unambiguous	6	1	N-Term(iTRAQ4plex); C8(Methylthio); K10(iTRAQ4plex)	HCD	1.0000	0.0000	1	1	11831	Unique	Used	0.252	9.465E4	2.996E4	186	4.23	0.00	0	39	41	2	720.86713	1440.72698	0.80	55.47	6628	6628	MS2	15/18	Glioma_Nuclear_prep_Gradeall_4plex.raw
5031755		High	gYAFITFcGk	Unambiguous	6	1	N-Term(iTRAQ4plex); C8(Methylthio); K10(iTRAQ4plex)	HCD	1.0000	0.0000	1	1	12320	Unique	Used	0.246	3.241E3	1.080E3	180	3.65	0.00	0	28	300	2	720.86768	1440.72807	1.57	79.57	7356	7356	MS2	16/18	Glioma_Nuclear_prep_Gradeall_repeat_4plex.raw
5031755		High	kYGGPPPDSVYSGVQPGIGTEVFVGk	Unambiguous	4	1	N-Term(iTRAQ4plex); K1(iTRAQ4plex); K26(iTRAQ4plex)	HCD	1.0000	0.0000	1	1	11224	Unique	Used	0.384	1.267E2	6.596E1	71	5.39	0.00	1	33	300	4	767.66901	3067.65420	2.49	60.11	5789	5789	MS2	21/150	Glioma_Nuclear_prep_Gradeall_repeat_4plex.raw
5031755		High	eSDLSHVQNk	Unambiguous	3	1	N-Term(iTRAQ4plex); K10(iTRAQ4plex)	HCD	1.0000	0.0000	1	1	7141	Unique	Used	0.446	7.755E3	4.683E3	193	4.80	0.00	0	39	214	2	722.87561	1444.74394	-13.51	16.06	1418	1418	MS2	17/18	Glioma_Nuclear_prep_Gradeall_repeat_4plex.raw
5031755		High	eSDLSHVQNk	Unambiguous	3	1	N-Term(iTRAQ4plex); K10(iTRAQ4plex)	HCD	1.0000	0.0000	1	1	7137	Unique	Used	0.456	9.583E3	5.916E3	159	3.49	0.00	0	29	84	3	482.25342	1444.74570	-12.29	16.03	1414	1414	MS2	18/36	Glioma_Nuclear_prep_Gradeall_repeat_4plex.raw
5031755	High		nLATTVTEEILEk	Unambiguous	7	1	N-Term(iTRAQ4plex); K13(iTRAQ4plex)	HCD	1.0000	0.0000	1	1	11946	Unique	Used	0.110	2.132E5	2.955E4	221	5.72	0.00	0	10	19	2	875.00085	1748.99443	3.42	56.46	6753	6753	MS2	23/24	Glioma_Nuclear_prep_Gradeall_4plex.raw
5031755	High		dLYEDELVPLFEk	Unambiguous	6	1	N-Term(iTRAQ4plex); K13(iTRAQ4plex)	HCD	1.0000	0.0000	1	1	12114	Unique	Used	0.130	1.345E5	2.194E4	176	5.44	0.00	0	6	17	2	949.50867	1898.01006	3.31	58.17	6938	6938	MS2	20/24	Glioma_Nuclear_prep_Gradeall_4plex.raw
5031755	High		nLATTVTEEILEk	Unambiguous	7	1	N-Term(iTRAQ4plex); K13(iTRAQ4plex)	HCD	1.0000	0.0000	1	1	11945	Unique	Used	0.193	2.406E4	5.851E3	219	5.21	0.00	0	8	10	3	583.66882	1748.99192	1.98	56.45	6752	6752	MS2	26/48	Glioma_Nuclear_prep_Gradeall_4plex.raw
5031755	High		nLATTVTEEILEk	Unambiguous	7	1	N-Term(iTRAQ4plex); K13(iTRAQ4plex)	HCD	1.0000	0.0000	1	1	11873	Unique	Used	0.195	8.651E4	2.125E4	220	5.81	0.00	0	20	85	2	874.99957	1748.99187	1.95	55.82	6673	6673	MS2	22/24	Glioma_Nuclear_prep_Gradeall_4plex.raw
5031755	High		dLYEDELVPLFEk	Unambiguous	6	1	N-Term(iTRAQ4plex); K13(iTRAQ4plex)	HCD	1.0000	0.0000	1	1	12264	Unique	Used	0.351	1.111E4	4.908E3	222	5.31	0.00	0	53	300	2	949.50476	1898.00224	-0.81	59.64	7102	7102	MS2	19/24	Glioma_Nuclear_prep_Gradeall_4plex.raw
5031755		High	dLYEDELVPLFEk	Unambiguous	6	1	N-Term(iTRAQ4plex); K13(iTRAQ4plex)	HCD	1.0000	0.0000	1	1	12390	Unique	Used	0.097	1.683E3	2.221E2	179	4.97	0.00	0	5	300	2	949.50635	1898.00542	0.86	82.86	7586	7586	MS2	20/24	Glioma_Nuclear_prep_Gradeall_repeat_4plex.raw
5031755		High	dLYEDELVPLFEk	Unambiguous	6	1	N-Term(iTRAQ4plex); K13(iTRAQ4plex)	HCD	1.0000	0.0000	1	1	12407	Unique	Used	0.184	1.007E3	2.507E2	174	4.80	0.00	0	11	300	2	949.50726	1898.00725	1.83	83.87	7655	7655	MS2	19/24	Glioma_Nuclear_prep_Gradeall_repeat_4plex.raw
5031755		High	nLATTVTEEILEk	Unambiguous	7	1	N-Term(iTRAQ4plex); K13(iTRAQ4plex)	HCD	1.0000	0.0000	1	1	12341	Unique	Used	0.189	2.261E3	5.791E2	220	4.93	0.00	0	13	300	2	874.99988	1748.99248	2.30	80.47	7419	7419	MS2	23/24	Glioma_Nuclear_prep_Gradeall_repeat_4plex.raw
5031755		High	nLATTVTEEILEk	Unambiguous	7	1	N-Term(iTRAQ4plex); K13(iTRAQ4plex)	HCD	1.0000	0.0000	1	1	12338	Unique	Used	0.222	1.990E2	5.974E1	174	4.06	0.00	0	22	300	3	583.66815	1748.98990	0.83	80.35	7411	7411	MS2	24/48	Glioma_Nuclear_prep_Gradeall_repeat_4plex.raw
5031755	High		eIEGEEIEIVLAkPPDk	Unambiguous	7	1	N-Term(iTRAQ4plex); K13(iTRAQ4plex); K17(iTRAQ4plex)	HCD	1.0000	0.0000	1	1	10389	Unique	Used	0.215	1.848E4	4.992E3	97	5.65	0.00	0	0	42	2	1171.14905	2341.29082	-13.64	43.75	5053	5053	MS2	24/32	Glioma_Nuclear_prep_Gradeall_4plex.raw
5031755	High		eFNEEGALSVLQQFk	Unambiguous	3	1	N-Term(iTRAQ4plex); K15(iTRAQ4plex)	HCD	1.0000	0.0000	1	1	12337	Unique	Used	0.409	3.018E3	1.551E3	201	6.16	0.00	0	22	93	3	676.36096	2027.06833	-0.25	60.44	7183	7183	MS2	25/56	Glioma_Nuclear_prep_Gradeall_4plex.raw
5031755	High		vTEGLVDVILYHQPDDk	Unambiguous	5	1	N-Term(iTRAQ4plex); K17(iTRAQ4plex)	HCD	1.0000	0.0000	1	1	11486	Unique	Used	0.126	1.000E5	1.590E4	151	6.85	0.00	0	7	4	3	743.73688	2229.19608	-2.02	52.49	6253	6253	MS2	34/64	Glioma_Nuclear_prep_Gradeall_4plex.raw
5031755	High		vTEGLVDVILYHQPDDk	Unambiguous	5	1	N-Term(iTRAQ4plex); K17(iTRAQ4plex)	HCD	1.0000	0.0000	1	1	11470	Unique	Used	0.179	3.424E4	7.716E3	181	7.93	0.00	0	0	39	2	1115.10193	2229.19658	-1.80	52.35	6235	6235	MS2	25/32	Glioma_Nuclear_prep_Gradeall_4plex.raw
5031755	High		vTEGLVDVILYHQPDDk	Unambiguous	5	1	N-Term(iTRAQ4plex); K17(iTRAQ4plex)	HCD	1.0000	0.0000	1	1	11404	Unique	Used	0.210	2.986E4	7.877E3	160	7.49	0.00	0	17	19	3	743.73749	2229.19791	-1.20	51.79	6163	6163	MS2	34/64	Glioma_Nuclear_prep_Gradeall_4plex.raw
5031755	High		vTEGLVDVILYHQPDDk	Unambiguous	5	1	N-Term(iTRAQ4plex); K17(iTRAQ4plex)	HCD	1.0000	0.0000	1	1	11539	Unique	Used	0.260	8.711E2	2.843E2	141	3.44	0.00	0	39	99	4	558.05420	2229.19497	-2.52	52.91	6310	6310	MS2	20/96	Glioma_Nuclear_prep_Gradeall_4plex.raw
5031755		High	vTEGLVDVILYHQPDDk	Unambiguous	5	1	N-Term(iTRAQ4plex); K17(iTRAQ4plex)	HCD	1.0000	0.0000	1	1	12178	Unique	Used	0.264	8.220E2	2.936E2	143	6.75	0.00	0	28	300	3	743.73944	2229.20377	1.43	75.68	7065	7065	MS2	32/64	Glioma_Nuclear_prep_Gradeall_repeat_4plex.raw
5031755	High		lkDYAFVHFEDR	Unambiguous	7	1	N-Term(iTRAQ4plex); K2(iTRAQ4plex)	HCD	1.0000	0.0000	1	1	9635	Unique	Used	0.163	1.626E4	3.334E3	201	3.62	0.00	1	33	9	4	457.73895	1827.93398	-16.01	38.21	4233	4233	MS2	24/66	Glioma_Nuclear_prep_Gradeall_4plex.raw
5031755	High		tkENILEEFSk	Unambiguous	7	1	N-Term(iTRAQ4plex); K2(iTRAQ4plex); K11(iTRAQ4plex)	HCD	1.0000	0.0000	1	1	9624	Unique	Used	0.196	1.937E5	4.769E4	188	3.72	0.00	1	34	22	2	885.49170	1769.97612	-14.04	38.13	4221	4221	MS2	17/20	Glioma_Nuclear_prep_Gradeall_4plex.raw
5031755		High	tkENILEEFSk	Unambiguous	7	1	N-Term(iTRAQ4plex); K2(iTRAQ4plex); K11(iTRAQ4plex)	HCD	1.0000	0.0000	1	1	10508	Unique	Used	0.216	6.980E3	2.045E3	172	4.76	0.00	1	29	300	2	885.50598	1770.00469	2.10	51.14	4997	4997	MS2	19/20	Glioma_Nuclear_prep_Gradeall_repeat_4plex.raw
5031755	High		yGGPPPDSVYSGVQPGIGTEVFVGk	Unambiguous	4	1	N-Term(iTRAQ4plex); K25(iTRAQ4plex)	HCD	1.0000	0.0000	1	1	10967	Unique	Used	0.110	5.614E4	7.780E3	104	5.70	0.00	0	0	17	2	1398.20752	2795.40776	-14.94	48.20	5685	5685	MS2	25/48	Glioma_Nuclear_prep_Gradeall_4plex.raw
5031755	High		yGGPPPDSVYSGVQPGIGTEVFVGk	Unambiguous	4	1	N-Term(iTRAQ4plex); K25(iTRAQ4plex)	HCD	1.0000	0.0000	1	1	11069	Unique	Used	0.207	5.830E3	1.516E3	93	5.70	0.00	0	31	89	2	1398.23169	2795.45610	2.36	48.99	5796	5796	MS2	27/48	Glioma_Nuclear_prep_Gradeall_4plex.raw
5031755	High		yGGPPPDSVYSGVQPGIGTEVFVGk	Unambiguous	4	1	N-Term(iTRAQ4plex); K25(iTRAQ4plex)	HCD	1.0000	0.0000	1	1	11003	Unique	Used	0.223	1.683E4	4.727E3	101	6.91	0.00	0	6	5	3	932.48889	2795.45212	0.93	48.48	5724	5724	MS2	27/96	Glioma_Nuclear_prep_Gradeall_4plex.raw
5031755		High	yGGPPPDSVYSGVQPGIGTEVFVGk	Unambiguous	4	1	N-Term(iTRAQ4plex); K25(iTRAQ4plex)	HCD	1.0000	0.0000	1	1	11737	Unique	Used	0.228	4.245E2	1.308E2	90	6.68	0.00	0	11	300	3	932.49182	2795.46091	4.08	67.49	6413	6413	MS2	28/96	Glioma_Nuclear_prep_Gradeall_repeat_4plex.raw
5031755	High		eNILEEFSk	Unambiguous	7	1	N-Term(iTRAQ4plex); K9(iTRAQ4plex)	HCD	1.0000	0.0000	1	1	10358	Unique	Used	0.180	3.454E5	7.832E4	216	3.54	0.00	0	21	7	2	698.87219	1396.73711	-13.71	43.53	5020	5020	MS2	15/16	Glioma_Nuclear_prep_Gradeall_4plex.raw
5031755		High	eNILEEFSk	Unambiguous	7	1	N-Term(iTRAQ4plex); K9(iTRAQ4plex)	HCD	1.0000	0.0000	1	1	11241	Unique	Used	0.240	9.874E3	3.216E3	250	3.58	0.00	0	16	300	2	698.88177	1396.75627	0.01	60.32	5808	5808	MS2	15/16	Glioma_Nuclear_prep_Gradeall_repeat_4plex.raw
5031877	NP_005564.1	LMNB1	4001	lamin-B1 isoform 1 [Homo sapiens]	47.10	2	24	50	3.019	25;25	39.8	150.40	586	66.4	5.16																			
5031877	High		lAQALHEMR	Unambiguous	2	1	N-Term(iTRAQ4plex)	HCD	1.0000	0.0000	1	1	8426	Unique	Used	1.296	1.285E5	2.095E5	223	3.18	0.00	0	33	17	2	606.82623	1212.64519	-15.51	29.37	2914	2914	MS2	15/16	Glioma_Nuclear_prep_Gradeall_4plex.raw
5031877	High		aGGPTTPLSPTR	Unambiguous	1	1	N-Term(iTRAQ4plex)	HCD	1.0000	0.0000	1	1	7676	Unique	Used	1.312	3.980E5	6.566E5	244	3.99	0.00	0	21	4	2	649.86218	1298.71709	-1.11	24.08	2102	2102	MS2	16/22	Glioma_Nuclear_prep_Gradeall_4plex.raw
5031877	High		aLYETELADAR	Unambiguous	1	1	N-Term(iTRAQ4plex)	HCD	1.0000	0.0000	1	1	9261	Unique	Used	1.724	4.630E4	1.004E5	183	3.34	0.00	0	42	12	2	698.35425	1395.70122	-16.09	35.46	3825	3825	MS2	18/20	Glioma_Nuclear_prep_Gradeall_4plex.raw
5031877	High		lALDMEISAYR	Unambiguous	2	1	N-Term(iTRAQ4plex)	HCD	1.0000	0.0000	1	1	10802	Unique	Used	2.070	3.083E4	8.028E4	232	3.61	0.00	0	53	52	2	713.37000	1425.73271	-14.14	46.93	5506	5506	MS2	18/20	Glioma_Nuclear_prep_Gradeall_4plex.raw
5031877	High		tTIPEEEEEEEAAGVVVEEELFHQQGTPR	Unambiguous	2	1	N-Term(iTRAQ4plex)	HCD	1.0000	0.0000	1	1	12492	Unique	Used	3.959	1.143E3	5.689E3	46	10.53	0.00	0	0	69	3	1185.89844	3555.68076	2.11	62.23	7355	7355	MS2	36/116	Glioma_Nuclear_prep_Gradeall_4plex.raw
5031877	High		lSSEMNTSTVNSAR	Unambiguous	2	1	N-Term(iTRAQ4plex)	HCD	1.0000	0.0000	1	1	8149	Unique	Used	4.027	3.550E4	1.798E5	105	4.16	0.00	0	9	18	2	820.89117	1640.77507	-17.07	27.41	2614	2614	MS2	20/26	Glioma_Nuclear_prep_Gradeall_4plex.raw
5031877	High		sLETENSALQLQVTER	Unambiguous	1	1	N-Term(iTRAQ4plex)	HCD	1.0000	0.0000	1	1	9637	Unique	Used	4.733	3.481E4	2.072E5	202	6.26	0.00	0	3	14	2	981.50037	1961.99346	-16.63	38.22	4235	4235	MS2	26/30	Glioma_Nuclear_prep_Gradeall_4plex.raw
5031877	High		lAQALHEMR	Unambiguous	2	1	N-Term(iTRAQ4plex)	HCD	1.0000	0.0000	1	1	8433	Unique	Used	4.928	2.723E4	1.687E5	169	3.59	0.00	0	25	6	3	404.88565	1212.64240	-17.81	29.43	2922	2922	MS2	20/32	Glioma_Nuclear_prep_Gradeall_4plex.raw
5031877		High	sLETENSALQLQVTER	Unambiguous	1	1	N-Term(iTRAQ4plex)	HCD	1.0000	0.0000	1	1	10256	Unique	Used	1.571	7.656E2	1.629E3	190	5.60	0.00	0	33	300	3	654.68036	1962.02652	0.22	48.11	4727	4727	MS2	24/60	Glioma_Nuclear_prep_Gradeall_repeat_4plex.raw
5031877		High	aLYETELADAR	Unambiguous	1	1	N-Term(iTRAQ4plex)	HCD	1.0000	0.0000	1	1	9841	Unique	Used	2.027	7.880E3	2.163E4	270	4.16	0.00	0	33	219	2	698.36694	1395.72661	2.10	43.10	4278	4278	MS2	19/20	Glioma_Nuclear_prep_Gradeall_repeat_4plex.raw
5031877		High	aGGPTTPLSPTR	Unambiguous	1	1	N-Term(iTRAQ4plex)	HCD	1.0000	0.0000	1	1	7770	Unique	Used	2.030	5.931E4	1.631E5	156	4.30	0.00	0	9	19	2	649.86041	1298.71355	-3.84	21.62	2084	2084	MS2	16/22	Glioma_Nuclear_prep_Gradeall_repeat_4plex.raw
5031877		High	sMYEEEINETR	Unambiguous	1	1	N-Term(iTRAQ4plex)	HCD	1.0000	0.0000	1	1	8861	Unique	Used	2.722	6.725E3	2.479E4	72	4.49	0.00	0	23	207	2	772.85272	1544.69817	-2.45	32.26	3233	3233	MS2	18/20	Glioma_Nuclear_prep_Gradeall_repeat_4plex.raw
5031877		High	lAQALHEMR	Unambiguous	2	1	N-Term(iTRAQ4plex)	HCD	1.0000	0.0000	1	1	8958	Unique	Used	2.958	2.881E3	1.154E4	210	3.80	0.00	0	43	142	3	404.89056	1212.65714	-5.65	33.21	3335	3335	MS2	20/32	Glioma_Nuclear_prep_Gradeall_repeat_4plex.raw
5031877		High	aLYETELADAR	Unambiguous	1	1	N-Term(iTRAQ4plex)	HCD	1.0000	0.0000	1	1	9776	Unique	Used	3.231	2.368E4	1.036E5	267	3.99	0.00	0	7	53	2	698.36560	1395.72392	0.18	42.40	4210	4210	MS2	18/20	Glioma_Nuclear_prep_Gradeall_repeat_4plex.raw
5031877		High	lSSEMNTSTVNSAR	Unambiguous	2	1	N-Term(iTRAQ4plex)	HCD	1.0000	0.0000	1	1	8362	Unique	Used	4.067	2.185E3	1.203E4	159	4.10	0.00	0	7	225	2	820.91040	1640.81352	6.37	27.28	2707	2707	MS2	20/26	Glioma_Nuclear_prep_Gradeall_repeat_4plex.raw
5031877		High	sLETENSALQLQVTER	Unambiguous	1	1	N-Term(iTRAQ4plex)	HCD	1.0000	0.0000	1	1	10245	Unique	Used	4.328	1.092E3	6.403E3	222	6.48	0.00	0	0	300	2	981.51550	1962.02373	-1.20	47.98	4715	4715	MS2	27/30	Glioma_Nuclear_prep_Gradeall_repeat_4plex.raw
5031877	High		cQSLTEDLEFR	Unambiguous	1	1	N-Term(iTRAQ4plex); C1(Methylthio)	HCD	1.0000	0.0000	1	1	11007	Unique	Used	1.533	1.441E5	2.779E5	78	3.92	0.00	0	31	15	2	765.85590	1530.70452	-0.27	48.50	5728	5728	MS2	18/20	Glioma_Nuclear_prep_Gradeall_4plex.raw
5031877	High		cQSLTEDLEFR	Unambiguous	1	1	N-Term(iTRAQ4plex); C1(Methylthio)	HCD	1.0000	0.0000	1	1	10932	Unique	Used	2.799	4.986E4	1.755E5	50	4.51	0.00	0	18	27	2	765.84552	1530.68376	-13.83	47.93	5647	5647	MS2	19/20	Glioma_Nuclear_prep_Gradeall_4plex.raw
5031877		High	cQSLTEDLEFR	Unambiguous	1	1	N-Term(iTRAQ4plex); C1(Methylthio)	HCD	1.0000	0.0000	1	1	11746	Unique	Used	3.452	1.245E3	5.824E3	77	4.05	0.00	0	22	300	2	765.85699	1530.70671	1.16	67.66	6427	6427	MS2	20/20	Glioma_Nuclear_prep_Gradeall_repeat_4plex.raw
5031877	High		kIGDTSVSYk	Unambiguous	2	1	N-Term(iTRAQ4plex); K1(iTRAQ4plex); K10(iTRAQ4plex)	HCD	1.0000	0.0000	1	1	7649	Unique	Used	1.437	2.163E4	3.909E4	143	3.39	0.00	1	56	15	3	510.63409	1529.88773	-1.45	23.87	2072	2072	MS2	14/36	Glioma_Nuclear_prep_Gradeall_4plex.raw
5031877	High		kQLADETLLk	Unambiguous	1	1	N-Term(iTRAQ4plex); K1(iTRAQ4plex); K10(iTRAQ4plex)	HCD	1.0000	0.0000	1	1	8290	Unique	Used	3.745	3.310E4	1.559E5	127	3.89	0.00	1	34	21	2	795.98059	1590.95391	-15.84	28.41	2767	2767	MS2	18/18	Glioma_Nuclear_prep_Gradeall_4plex.raw
5031877		High	kQLADETLLk	Unambiguous	1	1	N-Term(iTRAQ4plex); K1(iTRAQ4plex); K10(iTRAQ4plex)	HCD	1.0000	0.0000	1	1	8767	Unique	Used	1.650	2.628E3	5.872E3	149	3.65	0.00	1	36	180	3	530.98889	1590.95212	-16.96	31.25	3134	3134	MS2	18/36	Glioma_Nuclear_prep_Gradeall_repeat_4plex.raw
5031877		High	kQLADETLLk	Unambiguous	1	1	N-Term(iTRAQ4plex); K1(iTRAQ4plex); K10(iTRAQ4plex)	HCD	1.0000	0.0000	1	1	8779	Unique	Used	3.246	1.615E3	7.100E3	120	2.99	0.00	1	41	300	2	795.98175	1590.95622	-14.39	31.38	3146	3146	MS2	18/18	Glioma_Nuclear_prep_Gradeall_repeat_4plex.raw
5031877	High		eELEQTYHAk	Unambiguous	2	1	N-Term(iTRAQ4plex); K10(iTRAQ4plex)	HCD	1.0000	0.0000	1	1	7549	Unique	Used	1.612	1.577E4	3.197E4	163	3.59	0.00	0	50	12	3	512.60211	1535.79178	-1.72	23.16	1964	1964	MS2	21/36	Glioma_Nuclear_prep_Gradeall_4plex.raw
5031877	High		iQELEDLLAk	Unambiguous	2	1	N-Term(iTRAQ4plex); K10(iTRAQ4plex)	HCD	1.0000	0.0000	1	1	11890	Unique	Used	3.081	1.367E5	5.298E5	214	4.19	0.00	0	18	14	2	730.43774	1459.86821	4.89	55.97	6692	6692	MS2	18/18	Glioma_Nuclear_prep_Gradeall_4plex.raw
5031877	High		iQELEDLLAk	Unambiguous	2	1	N-Term(iTRAQ4plex); K10(iTRAQ4plex)	HCD	1.0000	0.0000	1	1	11807	Unique	Used	4.058	1.154E5	5.889E5	236	4.39	0.00	0	17	15	2	730.43365	1459.86003	-0.71	55.28	6602	6602	MS2	18/18	Glioma_Nuclear_prep_Gradeall_4plex.raw
5031877		High	iQELEDLLAk	Unambiguous	2	1	N-Term(iTRAQ4plex); K10(iTRAQ4plex)	HCD	1.0000	0.0000	1	1	12309	Unique	Used	2.241	1.842E3	5.591E3	268	3.90	0.00	0	41	300	2	730.43475	1459.86223	0.80	79.29	7335	7335	MS2	17/18	Glioma_Nuclear_prep_Gradeall_repeat_4plex.raw
5031877		High	iQELEDLLAk	Unambiguous	2	1	N-Term(iTRAQ4plex); K10(iTRAQ4plex)	HCD	1.0000	0.0000	1	1	12337	Unique	Used	4.480	2.613E3	1.586E4	231	4.55	0.00	0	6	300	2	730.43488	1459.86247	0.96	80.31	7408	7408	MS2	18/18	Glioma_Nuclear_prep_Gradeall_repeat_4plex.raw
5031877	High		dAALATALGDk	Unambiguous	1	1	N-Term(iTRAQ4plex); K11(iTRAQ4plex)	HCD	1.0000	0.0000	1	1	9082	Unique	Used	2.306	1.726E5	5.007E5	299	4.23	0.00	0	15	5	2	667.37073	1333.73418	-16.82	34.16	3630	3630	MS2	19/20	Glioma_Nuclear_prep_Gradeall_4plex.raw
5031877		High	dAALATALGDk	Unambiguous	1	1	N-Term(iTRAQ4plex); K11(iTRAQ4plex)	HCD	1.0000	0.0000	1	1	9595	Unique	Used	1.791	9.541E3	2.314E4	310	4.45	0.00	0	28	231	2	667.37866	1333.75005	-4.92	40.34	4018	4018	MS2	19/20	Glioma_Nuclear_prep_Gradeall_repeat_4plex.raw
5031877	High		dAALATALGDkk	Unambiguous	1	1	N-Term(iTRAQ4plex); K11(iTRAQ4plex); K12(iTRAQ4plex)	HCD	1.0000	0.0000	1	1	8476	Unique	Used	3.319	7.091E4	2.960E5	150	3.51	0.00	1	7	2	3	535.98016	1605.92594	-17.25	29.73	2969	2969	MS2	27/44	Glioma_Nuclear_prep_Gradeall_4plex.raw
5031877	High		dAALATALGDkk	Unambiguous	1	1	N-Term(iTRAQ4plex); K11(iTRAQ4plex); K12(iTRAQ4plex)	HCD	1.0000	0.0000	1	1	8481	Unique	Used	4.761	3.144E4	1.882E5	210	4.33	0.00	1	7	13	2	803.46765	1605.92803	-15.95	29.76	2974	2974	MS2	20/22	Glioma_Nuclear_prep_Gradeall_4plex.raw
5031877	High		aEHDQLLLNYAk	Unambiguous	1	1	N-Term(iTRAQ4plex); K12(iTRAQ4plex)	HCD	1.0000	0.0000	1	1	8846	Unique	Used	3.276	8.294E4	3.417E5	163	5.04	0.00	0	10	8	2	851.95844	1702.90959	-15.90	32.43	3372	3372	MS2	22/22	Glioma_Nuclear_prep_Gradeall_4plex.raw
5031877		High	aEHDQLLLNYAk	Unambiguous	1	1	N-Term(iTRAQ4plex); K12(iTRAQ4plex)	HCD	1.0000	0.0000	1	1	9489	Unique	Used	3.126	1.862E3	7.883E3	193	5.39	0.00	0	15	300	2	851.97540	1702.94353	4.02	39.13	3900	3900	MS2	22/22	Glioma_Nuclear_prep_Gradeall_repeat_4plex.raw
5031877	High		iESLSSQLSNLQk	Unambiguous	2	1	N-Term(iTRAQ4plex); K13(iTRAQ4plex)	HCD	1.0000	0.0000	1	1	10191	Unique	Used	3.687	6.032E4	2.797E5	254	5.44	0.00	0	19	20	2	867.98315	1734.95903	-14.41	42.28	4838	4838	MS2	24/24	Glioma_Nuclear_prep_Gradeall_4plex.raw
5031877		High	iESLSSQLSNLQk	Unambiguous	2	1	N-Term(iTRAQ4plex); K13(iTRAQ4plex)	HCD	1.0000	0.0000	1	1	11003	Unique	Used	3.335	2.566E3	1.159E4	264	5.91	0.00	0	14	300	2	867.99768	1734.98808	2.34	57.18	5535	5535	MS2	24/24	Glioma_Nuclear_prep_Gradeall_repeat_4plex.raw
5031877	High		dQIAQLEASLAAAk	Unambiguous	1	1	N-Term(iTRAQ4plex); K14(iTRAQ4plex)	HCD	1.0000	0.0000	1	1	11440	Unique	Used	1.831	3.167E3	7.294E3	180	3.64	0.00	0	38	81	3	572.99371	1716.96659	-4.02	52.09	6202	6202	MS2	23/52	Glioma_Nuclear_prep_Gradeall_4plex.raw
5031877	High		dQMQQQLNDYEQLLDVk	Unambiguous	2	1	N-Term(iTRAQ4plex); K17(iTRAQ4plex)	HCD	1.0000	0.0000	1	1	11974	Unique	Used	2.253	2.322E3	6.579E3	158	7.74	0.00	0	18	68	3	799.40289	2396.19413	-2.73	56.73	6784	6784	MS2	28/64	Glioma_Nuclear_prep_Gradeall_4plex.raw
5031877	High		lYkEELEQTYHAk	Unambiguous	2	1	N-Term(iTRAQ4plex); K3(iTRAQ4plex); K13(iTRAQ4plex)	HCD	1.0000	0.0000	1	1	8735	Unique	Used	5.462	2.175E4	1.494E5	161	5.40	0.00	1	0	13	2	1042.55481	2084.10234	-17.51	31.62	3251	3251	MS2	22/24	Glioma_Nuclear_prep_Gradeall_4plex.raw
5031877		High	lYkEELEQTYHAk	Unambiguous	2	1	N-Term(iTRAQ4plex); K3(iTRAQ4plex); K13(iTRAQ4plex)	HCD	1.0000	0.0000	1	1	9412	Unique	Used	3.595	6.182E2	3.010E3	139	5.05	0.00	1	20	170	3	695.38544	2084.14176	1.40	38.25	3817	3817	MS2	27/48	Glioma_Nuclear_prep_Gradeall_repeat_4plex.raw
5031877		High	qIEYEYk	Unambiguous	2	1	N-Term(iTRAQ4plex); K7(iTRAQ4plex)	HCD	1.0000	0.0000	1	1	8436	Unique	Used	1.366	2.053E4	3.800E4	223	2.88	0.00	0	43	196	2	630.83978	1260.67229	0.68	27.98	2785	2785	MS2	11/12	Glioma_Nuclear_prep_Gradeall_repeat_4plex.raw
5031877		High	lAVYIDk	Unambiguous	1	1	N-Term(iTRAQ4plex); K7(iTRAQ4plex)	HCD	1.0000	0.0000	1	1	9757	Unique	Used	4.696	1.724E4	1.097E5	213	2.84	0.00	0	12	112	2	555.34491	1109.68254	1.49	42.18	4190	4190	MS2	12/12	Glioma_Nuclear_prep_Gradeall_repeat_4plex.raw
5031877	High		qLADETLLk	Unambiguous	1	1	N-Term(iTRAQ4plex); K9(iTRAQ4plex)	HCD	1.0000	0.0000	1	1	8941	Unique	Used	1.971	9.957E4	2.468E5	277	3.45	0.00	0	48	13	2	659.88373	1318.76018	-16.60	33.10	3475	3475	MS2	16/16	Glioma_Nuclear_prep_Gradeall_4plex.raw
5031877		High	qLADETLLk	Unambiguous	1	1	N-Term(iTRAQ4plex); K9(iTRAQ4plex)	HCD	1.0000	0.0000	1	1	9505	Unique	Used	2.483	1.486E4	4.998E4	253	3.69	0.00	0	40	80	2	659.89038	1318.77349	-6.52	39.32	3919	3919	MS2	16/16	Glioma_Nuclear_prep_Gradeall_repeat_4plex.raw
5031877		High	eYEAALNSk	Unambiguous	1	1	N-Term(iTRAQ4plex); K9(iTRAQ4plex)	HCD	1.0000	0.0000	1	1	8290	Unique	Used	2.848	3.564E4	1.375E5	165	3.44	0.00	0	8	39	2	656.85645	1312.70561	5.25	26.61	2632	2632	MS2	16/16	Glioma_Nuclear_prep_Gradeall_repeat_4plex.raw
5031877		High	smYEEEINETR	Unambiguous	1	1	N-Term(iTRAQ4plex); M2(Oxidation)	HCD	1.0000	0.0000	1	1	8279	Unique	Used	3.720	5.123E3	2.582E4	52	3.20	0.00	0	29	258	2	780.85693	1560.70659	6.23	26.48	2620	2620	MS2	16/20	Glioma_Nuclear_prep_Gradeall_repeat_4plex.raw
5031877	High		dQmQQQLNDYEQLLDVk	Unambiguous	2	1	N-Term(iTRAQ4plex); M3(Oxidation); K17(iTRAQ4plex)	HCD	1.0000	0.0000	1	1	11082	Unique	Used	4.919	5.479E3	3.389E4	111	5.92	0.00	0	3	41	2	1206.59436	2412.18144	-5.86	49.09	5810	5810	MS2	25/32	Glioma_Nuclear_prep_Gradeall_4plex.raw
5031877		High	dQmQQQLNDYEQLLDVk	Unambiguous	2	1	N-Term(iTRAQ4plex); M3(Oxidation); K17(iTRAQ4plex)	HCD	1.0000	0.0000	1	1	11794	Unique	Used	1.495	1.335E2	2.702E2	132	6.36	0.00	0	19	300	3	804.73755	2412.19809	1.04	68.47	6493	6493	MS2	27/64	Glioma_Nuclear_prep_Gradeall_repeat_4plex.raw
5031877		High	lSSEmNTSTVNSAR	Unambiguous	2	1	N-Term(iTRAQ4plex); M5(Oxidation)	HCD	1.0000	0.0000	1	1	7323	Unique	Used	4.472	5.104E3	3.092E4	115	4.88	0.00	0	25	146	2	828.90106	1656.79485	-1.90	17.60	1609	1609	MS2	20/26	Glioma_Nuclear_prep_Gradeall_repeat_4plex.raw

5031877		High	lAQALHEmR	Unambiguous	2	1	N-Term(iTRAQ4plex); M8(Oxidation)	HCD	1.0000	0.0000	1	1	7740	Unique	Used	1.645	4.500E3	1.003E4	213	3.80	0.00	0	24	112	3	410.22388	1228.65708	-1.49	21.32	2052	2052	MS2	18/32	Glioma_Nuclear_prep_Gradeall_repeat_4plex.raw
5032007	NP_005850.1	PURA	5813	transcriptional activator protein Pur-alpha [Homo sapiens]	9.94	1	2	2	0.147	1;1	38.6	11.23	322	34.9	6.44																			
5032007	High		gPGLGSTQGQTIA LPAQGLIEFR	Unambiguous	1	1	N-Term(iTRAQ4plex)	HCD	1.0000	0.0000	1	1	11841	Unique	Used	0.107	2.891E4	3.878E3	89	7.50	0.00	0	0	94	2	1228.17200	2455.33672	-2.42	55.54	6638	6638	MS2	30/44	Glioma_Nuclear_prep_Gradeall_4plex.raw
5032007		High	fFFDVGSNk	Unambiguous	1	1	N-Term(iTRAQ4plex); K9(iTRAQ4plex)	HCD	1.0000	0.0000	1	1	11289	Unique	Used	0.187	1.124E4	2.845E3	310	3.38	0.00	0	60	300	2	674.86194	1348.71660	1.93	61.05	5868	5868	MS2	15/16	Glioma_Nuclear_prep_Gradeall_repeat_4plex.raw
5174449	NP_006017.1	H1FX	8971	histone H1x [Homo sapiens]	23.00	1	4	16	1.889	7;9	20.4	54.20	213	22.5	10.76																			
5174449	High		ySQLVVETIR	Unambiguous	1	1	N-Term(iTRAQ4plex)	HCD	1.0000	0.0000	1	1	9962	Unique	Used	1.846	1.144E5	2.656E5	259	3.27	0.00	0	22	14	2	676.37793	1351.74858	-16.00	40.58	4588	4588	MS2	17/18	Glioma_Nuclear_prep_Gradeall_4plex.raw
5174449	High		ySQLVVETIR	Unambiguous	1	1	N-Term(iTRAQ4plex)	HCD	1.0000	0.0000	1	1	10050	Unique	Used	2.356	1.970E5	5.836E5	283	3.30	0.00	0	7	4	2	676.37854	1351.74980	-15.10	41.22	4684	4684	MS2	17/18	Glioma_Nuclear_prep_Gradeall_4plex.raw
5174449		High	ySQLVVETIR	Unambiguous	1	1	N-Term(iTRAQ4plex)	HCD	1.0000	0.0000	1	1	10740	Unique	Used	2.212	5.680E3	1.701E4	323	3.88	0.00	0	54	300	2	676.38989	1351.77251	1.70	53.92	5247	5247	MS2	16/18	Glioma_Nuclear_prep_Gradeall_repeat_4plex.raw
5174449		High	ySQLVVETIR	Unambiguous	1	1	N-Term(iTRAQ4plex)	HCD	1.0000	0.0000	1	1	10814	Unique	Used	2.232	2.466E4	7.455E4	204	3.68	0.00	0	5	47	2	676.38959	1351.77190	1.25	54.86	5329	5329	MS2	17/18	Glioma_Nuclear_prep_Gradeall_repeat_4plex.raw
5174449	High		aGGSAALSPSk	Unambiguous	1	1	N-Term(iTRAQ4plex); K11(iTRAQ4plex)	HCD	1.0000	0.0000	1	1	7309	Unique	Used	1.808	6.617E4	1.504E5	314	4.01	0.00	0	21	17	2	617.35223	1233.69719	-5.65	21.44	1704	1704	MS2	17/20	Glioma_Nuclear_prep_Gradeall_4plex.raw
5174449		High	aGGSAALSPSk	Unambiguous	1	1	N-Term(iTRAQ4plex); K11(iTRAQ4plex)	HCD	1.0000	0.0000	1	1	7119	Unique	Used	1.461	2.112E4	4.180E4	266	4.09	0.00	0	11	79	2	617.34595	1233.68462	-15.85	15.86	1395	1395	MS2	19/20	Glioma_Nuclear_prep_Gradeall_repeat_4plex.raw
5174449		High	aGGSAALSPSk	Unambiguous	1	1	N-Term(iTRAQ4plex); K11(iTRAQ4plex)	HCD	1.0000	0.0000	1	1	7153	Unique	Used	2.089	6.225E4	1.762E5	296	4.29	0.00	0	5	16	2	617.35303	1233.69878	-4.37	16.19	1431	1431	MS2	19/20	Glioma_Nuclear_prep_Gradeall_repeat_4plex.raw
5174449	High		aLVQNDTLLQVk	Unambiguous	1	1	N-Term(iTRAQ4plex); K12(iTRAQ4plex)	HCD	1.0000	0.0000	1	1	9786	Unique	Used	1.972	6.782E4	1.682E5	161	4.65	0.00	0	27	11	2	815.48041	1629.95354	-14.90	39.30	4397	4397	MS2	22/22	Glioma_Nuclear_prep_Gradeall_4plex.raw
5174449	High		aLVQNDTLLQVk	Unambiguous	1	1	N-Term(iTRAQ4plex); K12(iTRAQ4plex)	HCD	1.0000	0.0000	1	1	9686	Unique	Used	2.892	1.088E5	3.958E5	154	4.85	0.00	0	8	5	2	815.47968	1629.95207	-15.80	38.59	4289	4289	MS2	22/22	Glioma_Nuclear_prep_Gradeall_4plex.raw
5174449		High	aLVQNDTLLQVk	Unambiguous	1	1	N-Term(iTRAQ4plex); K12(iTRAQ4plex)	HCD	1.0000	0.0000	1	1	10398	Unique	Used	1.523	1.227E3	2.531E3	128	5.34	0.00	0	16	300	3	543.99811	1629.97977	1.19	49.80	4879	4879	MS2	22/44	Glioma_Nuclear_prep_Gradeall_repeat_4plex.raw
5174449		High	aLVQNDTLLQVk	Unambiguous	1	1	N-Term(iTRAQ4plex); K12(iTRAQ4plex)	HCD	1.0000	0.0000	1	1	10472	Unique	Used	1.892	3.706E3	9.498E3	129	5.67	0.00	0	13	300	2	815.49384	1629.98039	1.57	50.69	4958	4958	MS2	22/22	Glioma_Nuclear_prep_Gradeall_repeat_4plex.raw
5174449		High	aLVQNDTLLQVk	Unambiguous	1	1	N-Term(iTRAQ4plex); K12(iTRAQ4plex)	HCD	1.0000	0.0000	1	1	10400	Unique	Used	2.091	5.268E3	1.492E4	128	5.95	0.00	0	38	300	2	815.49310	1629.97893	0.68	49.83	4881	4881	MS2	22/22	Glioma_Nuclear_prep_Gradeall_repeat_4plex.raw
5174449	High		gAPAAATAPAPTAH k	Unambiguous	1	1	N-Term(iTRAQ4plex); K15(iTRAQ4plex)	HCD	1.0000	0.0000	1	1	7279	Unique	Used	1.487	2.407E4	4.501E4	162	5.99	0.00	0	16	5	3	540.63879	1619.90183	-5.56	21.21	1671	1671	MS2	27/56	Glioma_Nuclear_prep_Gradeall_4plex.raw
5174449	High		gAPAAATAPAPTAH k	Unambiguous	1	1	N-Term(iTRAQ4plex); K15(iTRAQ4plex)	HCD	1.0000	0.0000	1	1	7287	Unique	Used	1.886	2.202E4	5.223E4	243	6.22	0.00	0	0	44	2	810.45483	1619.90239	-5.21	21.25	1679	1679	MS2	24/28	Glioma_Nuclear_prep_Gradeall_4plex.raw
5174449		High	gAPAAATAPAPTAH k	Unambiguous	1	1	N-Term(iTRAQ4plex); K15(iTRAQ4plex)	HCD	1.0000	0.0000	1	1	7082	Unique	Used	1.404	4.256E3	8.093E3	174	5.95	0.00	0	24	83	3	540.63165	1619.88041	-18.78	15.48	1354	1354	MS2	23/56	Glioma_Nuclear_prep_Gradeall_repeat_4plex.raw
5174449		High	gAPAAATAPAPTAH k	Unambiguous	1	1	N-Term(iTRAQ4plex); K15(iTRAQ4plex)	HCD	1.0000	0.0000	1	1	7090	Unique	Used	1.609	5.384E3	1.173E4	239	5.27	0.00	0	10	86	2	810.44672	1619.88616	-15.23	15.56	1363	1363	MS2	24/28	Glioma_Nuclear_prep_Gradeall_repeat_4plex.raw
5453740	NP_006462.1	MYL12A	10627	myosin regulatory light chain 12A isoform 1 [Homo sapiens]	6.43	3	1	2	1.663	1;1	22.2	4.00	171	19.8	4.81																			
5453740	High		fTDEEVDELYR	Unambiguous	3	1	N-Term(iTRAQ4plex)	HCD	1.0000	0.0000	1	1	10042	Unique	Used	1.925	4.009E4	9.705E4	66	3.30	0.00	0	43	33	2	780.35919	1559.71111	-15.08	41.16	4675	4675	MS2	17/20	Glioma_Nuclear_prep_Gradeall_4plex.raw
5453740		High	fTDEEVDELYR	Unambiguous	3	1	N-Term(iTRAQ4plex)	HCD	1.0000	0.0000	1	1	10696	Unique	Used	1.402	4.479E3	8.503E3	104	4.00	0.00	0	49	300	2	780.37225	1559.73723	1.66	53.38	5199	5199	MS2	19/20	Glioma_Nuclear_prep_Gradeall_repeat_4plex.raw
5730027	NP_006550.1	KHDRBS1	10657	KH domain-containing, RNA-binding, signal transduction-associated protein 1 isoform 1 [Homo sapiens]	16.70	6	4	12	0.277	7;5	38.9	41.67	443	48.2	8.66																			
5730027	High		sGSMDPSGAHPSV R	Unambiguous	2	1	N-Term(iTRAQ4plex)	HCD	1.0000	0.0000	1	1	7298	Unique	Used	0.572	2.630E4	1.893E4	109	3.12	0.00	0	12	76	2	764.86591	1528.72453	-3.26	21.34	1691	1691	MS2	15/26	Glioma_Nuclear_prep_Gradeall_4plex.raw
5730027		High	sGSMDPSGAHPSV R	Unambiguous	2	1	N-Term(iTRAQ4plex)	HCD	1.0000	0.0000	1	1	7122	Unique	Used	0.253	1.219E4	4.169E3	70	4.04	0.00	0	13	213	2	764.85870	1528.71013	-12.69	15.88	1398	1398	MS2	18/26	Glioma_Nuclear_prep_Gradeall_repeat_4plex.raw
5730027	High		kDDEENYLDLFSHk	Unambiguous	2	1	N-Term(iTRAQ4plex); K1(iTRAQ4plex); K14(iTRAQ4plex)	HCD	1.0000	0.0000	1	1	9796	Unique	Used	0.331	7.273E4	3.026E4	125	6.32	0.00	1	26	5	3	729.03448	2185.08890	-11.38	39.37	4408	4408	MS2	28/52	Glioma_Nuclear_prep_Gradeall_4plex.raw
5730027		High	kDDEENYLDLFSHk	Unambiguous	2	1	N-Term(iTRAQ4plex); K1(iTRAQ4plex); K14(iTRAQ4plex)	HCD	1.0000	0.0000	1	1	10766	Unique	Used	0.293	1.142E3	4.539E2	146	6.55	0.00	1	30	300	3	729.04706	2185.12662	5.88	54.24	5275	5275	MS2	33/52	Glioma_Nuclear_prep_Gradeall_repeat_4plex.raw
5730027	High		iLGPQGNTIk	Unambiguous	1	1	N-Term(iTRAQ4plex); K10(iTRAQ4plex)	HCD	1.0000	0.0000	1	1	8322	Unique	Used	0.167	1.482E6	3.107E5	147	3.64	0.00	0	13	3	2	664.90179	1328.79631	-13.35	28.64	2802	2802	MS2	17/18	Glioma_Nuclear_prep_Gradeall_4plex.raw
5730027		High	iLGPQGNTIk	Unambiguous	1	1	N-Term(iTRAQ4plex); K10(iTRAQ4plex)	HCD	1.0000	0.0000	1	1	8708	Unique	Used	0.235	1.250E5	3.976E4	86	3.14	0.00	0	28	62	2	664.91547	1328.82366	7.23	30.73	3072	3072	MS2	17/18	Glioma_Nuclear_prep_Gradeall_repeat_4plex.raw
5730027		High	iLGPQGNTIk	Unambiguous	1	1	N-Term(iTRAQ4plex); K10(iTRAQ4plex)	HCD	1.0000	0.0000	1	1	8739	Unique	Used	0.258	9.978E4	3.487E4	153	3.94	0.00	0	16	80	2	664.90356	1328.79985	-10.68	30.97	3104	3104	MS2	17/18	Glioma_Nuclear_prep_Gradeall_repeat_4plex.raw
5730027	High		dSLDPSFTHAMQL LTAEIEk	Unambiguous	2	1	N-Term(iTRAQ4plex); K20(iTRAQ4plex)	HCD	1.0000	0.0000	1	1	12863	Unique	Used	0.214	1.083E3	2.916E2	150	6.33	0.00	0	13	300	3	845.44232	2534.31241	2.86	67.38	7797	7797	MS2	28/76	Glioma_Nuclear_prep_Gradeall_4plex.raw
5730027	High		dSLDPSFTHAMQL LTAEIEk	Unambiguous	2	1	N-Term(iTRAQ4plex); K20(iTRAQ4plex)	HCD	1.0000	0.0000	1	1	12908	Unique	Used	0.260	9.833E2	3.221E2	131	6.33	0.00	0	16	300	3	845.44263	2534.31333	3.22	68.12	7856	7856	MS2	27/76	Glioma_Nuclear_prep_Gradeall_4plex.raw
5730027	High		dSLDPSFTHAMQL LTAEIEk	Unambiguous	2	1	N-Term(iTRAQ4plex); K20(iTRAQ4plex)	HCD	1.0000	0.0000	1	1	12853	Unique	Used	0.449	1.196E2	6.749E1	152	3.68	0.00	0	37	300	4	634.33398	2534.31411	3.53	67.22	7784	7784	MS2	20/114	Glioma_Nuclear_prep_Gradeall_4plex.raw
5730027	High		dSLDPSFTHAMQL LTAEIEk	Unambiguous	2	1	N-Term(iTRAQ4plex); K20(iTRAQ4plex)	HCD	1.0000	0.0000	1	1	12951	Unique	Used	0.490	3.128E2	1.926E2	144	5.63	0.00	0	18	300	3	845.44250	2534.31296	3.08	68.81	7912	7912	MS2	27/76	Glioma_Nuclear_prep_Gradeall_4plex.raw
5730027		High	sGSmDPSGAHPSV R	Unambiguous	2	1	N-Term(iTRAQ4plex); M4(Oxidation)	HCD	1.0000	0.0000	1	1	6668	Unique	Used	0.470	4.580E3	2.918E3	78	5.02	0.00	0	47	188	3	515.57904	1544.72257	-1.21	11.81	918	918	MS2	21/52	Glioma_Nuclear_prep_Gradeall_repeat_4plex.raw
5730073	NP_006549.1	KHDRBS3	10656	KH domain-containing, RNA-binding, signal transduction-associated protein 3 [Homo sapiens]	20.81	5	5	8	0.200	5;3	27.7	32.90	346	38.8	7.61																			
5730073	High		fLIPDYNDEIR	Unambiguous	1	1	N-Term(iTRAQ4plex)	HCD	1.0000	0.0000	1	1	10790	Unique	Used	0.123	6.674E5	1.036E5	239	3.19	0.00	0	17	10	2	769.89221	1538.77715	-13.01	46.85	5493	5493	MS2	15/20	Glioma_Nuclear_prep_Gradeall_4plex.raw
5730073	High		dSLDPSFTHALR	Unambiguous	1	1	N-Term(iTRAQ4plex)	HCD	1.0000	0.0000	1	1	9043	Unique	Used	0.308	7.496E4	2.901E4	84	4.28	0.00	0	13	5	3	501.58771	1502.74857	-15.61	33.84	3586	3586	MS2	27/44	Glioma_Nuclear_prep_Gradeall_4plex.raw
5730073		High	fLIPDYNDEIR	Unambiguous	1	1	N-Term(iTRAQ4plex)	HCD	1.0000	0.0000	1	1	11565	Unique	Used	0.181	5.741E3	1.409E3	276	3.23	0.00	0	15	300	2	769.90417	1538.80107	2.53	65.08	6208	6208	MS2	14/20	Glioma_Nuclear_prep_Gradeall_repeat_4plex.raw
5730073		High	fLIPDYNDEIR	Unambiguous	1	1	N-Term(iTRAQ4plex)	HCD	1.0000	0.0000	1	1	11640	Unique	Used	0.186	1.473E4	3.705E3	274	3.53	0.00	0	29	300	2	769.90393	1538.80058	2.22	66.11	6296	6296	MS2	13/20	Glioma_Nuclear_prep_Gradeall_repeat_4plex.raw
5730073	High		lQEETLTk	Unambiguous	1	1	N-Term(iTRAQ4plex); K8(iTRAQ4plex)	HCD	1.0000	0.0000	1	1	8026	Unique	Used	0.214	5.245E5	1.409E5	291	3.18	0.00	0	28	10	2	625.35840	1249.70952	-11.76	26.54	2481	2481	MS2	14/14	Glioma_Nuclear_prep_Gradeall_4plex.raw
5730073	High		yIDVVINk	Unambiguous	1	1	N-Term(iTRAQ4plex); K8(iTRAQ4plex)	HCD	1.0000	0.0000	1	1	9725	Unique	Used	0.256	5.592E5	1.803E5	259	3.07	0.00	0	52	8	2	626.37323	1251.73918	-12.73	38.87	4331	4331	MS2	13/14	Glioma_Nuclear_prep_Gradeall_4plex.raw
5730073		High	yIDVVINk	Unambiguous	1	1	N-Term(iTRAQ4plex); K8(iTRAQ4plex)	HCD	1.0000	0.0000	1	1	10407	Unique	Used	0.170	3.301E4	7.589E3	179	3.54	0.00	0	28	300	2	626.38336	1251.75945	3.46	49.92	4889	4889	MS2	13/14	Glioma_Nuclear_prep_Gradeall_repeat_4plex.raw
5730073	High		mGHALEEIk	Unambiguous	1	1	N-Term(iTRAQ4plex); K9(iTRAQ4plex)	HCD	1.0000	0.0000	1	1	8232	Unique	Used	0.251	5.378E5	1.697E5	182	3.57	0.00	0	52	10	2	658.35742	1315.70757	-15.74	28.00	2704	2704	MS2	14/16	Glioma_Nuclear_prep_Gradeall_4plex.raw
5803036	NP_006796.1	HNRNPA0	10949	heterogeneous nuclear ribonucleoprotein A0 [Homo sapiens]	18.36	1	4	7	0.209	3;4	39.6	18.26	305	30.8	9.29																			
5803036		High	fHPIQGHR	Unambiguous	1	1	N-Term(iTRAQ4plex)	HCD	1.0000	0.0000	1	1	6846	Unique	Used	0.149	4.656E4	9.412E3	275	3.70	0.00	0	37	65	3	379.21201	1135.62146	-2.37	13.42	1105	1105	MS2	18/28	Glioma_Nuclear_prep_Gradeall_repeat_4plex.raw
5803036		High	eDIYSGGGGGGSR	Unambiguous	1	1	N-Term(iTRAQ4plex)	HCD	1.0000	0.0000	1	1	7258	Unique	Used	0.189	4.434E4	1.133E4	41	3.37	0.00	0	5	72	2	678.30884	1355.61040	-15.08	17.01	1541	1541	MS2	17/24	Glioma_Nuclear_prep_Gradeall_repeat_4plex.raw
5803036	High		gFGFVYFQNHDAA Dk	Unambiguous	1	1	N-Term(iTRAQ4plex); K15(iTRAQ4plex)	HCD	1.0000	0.0000	1	1	10008	Unique	Used	0.330	7.088E4	2.940E4	102	5.95	0.00	0	11	25	2	1002.48206	2003.95683	-14.27	40.91	4638	4638	MS2	25/28	Glioma_Nuclear_prep_Gradeall_4plex.raw
5803036		High	gFGFVYFQNHDAA Dk	Unambiguous	1	1	N-Term(iTRAQ4plex); K15(iTRAQ4plex)	HCD	1.0000	0.0000	1	1	10964	Unique	Used	0.209	2.134E3	6.029E2	137	5.50	0.00	0	18	240	3	668.66827	2003.99027	2.41	56.71	5493	5493	MS2	30/56	Glioma_Nuclear_prep_Gradeall_repeat_4plex.raw
5803036	High		gDVAEGDLIEHFSQ FGTVEk	Unambiguous	1	1	N-Term(iTRAQ4plex); K20(iTRAQ4plex)	HCD	1.0000	0.0000	1	1	11259	Unique	Used	0.169	3.102E4	6.598E3	138	6.47	0.00	0	24	14	3	822.74890	2466.23215	-2.85	50.52	6004	6004	MS2	30/76	Glioma_Nuclear_prep_Gradeall_4plex.raw
5803036	High		gDVAEGDLIEHFSQ FGTVEk	Unambiguous	1	1	N-Term(iTRAQ4plex); K20(iTRAQ4plex)	HCD	1.0000	0.0000	1	1	11382	Unique	Used	0.409	6.256E3	3.219E3	139	5.76	0.00	0	25	62	3	822.75409	2466.24771	3.46	51.58	6138	6138	MS2	29/76	Glioma_Nuclear_prep_Gradeall_4plex.raw
5803036		High	gDVAEGDLIEHFSQ FGTVEk	Unambiguous	1	1	N-Term(iTRAQ4plex); K20(iTRAQ4plex)	HCD	1.0000	0.0000	1	1	12029	Unique	Used	0.343	6.084E2	2.825E2	132	5.70	0.00	0	24	300	3	822.75385	2466.24698	3.16	72.53	6821	6821	MS2	30/76	Glioma_Nuclear_prep_Gradeall_repeat_4plex.raw
6005721	NP_009106.1	ERLIN2	11160	erlin-2 isoform 1 [Homo sapiens]	11.50	2	3	6	0.552	3;3	14.8	16.03	339	37.8	5.62																			
6005721	High		iSEIEDAAFLAR	Unambiguous	2	1	N-Term(iTRAQ4plex)	HCD	1.0000	0.0000	1	1	10917	Unique	Used	0.466	2.278E5	1.335E5	211	4.13	0.00	0	21	15	2	739.89276	1478.77825	-12.81	47.82	5631	5631	MS2	20/22	Glioma_Nuclear_prep_Gradeall_4plex.raw
6005721		High	iSEIEDAAFLAR	Unambiguous	2	1	N-Term(iTRAQ4plex)	HCD	1.0000	0.0000	1	1	11715	Unique	Used	0.493	5.555E3	3.708E3	243	4.17	0.00	0	33	300	2	739.90289	1478.79851	0.89	67.17	6386	6386	MS2	20/22	Glioma_Nuclear_prep_Gradeall_repeat_4plex.raw
6005721	High		vAQVAEITYGQk	Unambiguous	1	1	N-Term(iTRAQ4plex); K12(iTRAQ4plex)	HCD	1.0000	0.0000	1	1	9030	Unique	Used	0.614	6.093E4	4.707E4	243	4.94	0.00	0	9	48	2	797.94379	1594.88030	-15.06	33.73	3571	3571	MS2	18/22	Glioma_Nuclear_prep_Gradeall_4plex.raw
6005721		High	vAQVAEITYGQk	Unambiguous	1	1	N-Term(iTRAQ4plex); K12(iTRAQ4plex)	HCD	1.0000	0.0000	1	1	9545	Unique	Used	0.465	7.362E3	4.639E3	231	4.54	0.00	0	19	300	2	797.94678	1594.88628	-11.31	39.79	3964	3964	MS2	18/22	Glioma_Nuclear_prep_Gradeall_repeat_4plex.raw
6005721		High	vAQVAEITYGQk	Unambiguous	1	1	N-Term(iTRAQ4plex); K12(iTRAQ4plex)	HCD	1.0000	0.0000	1	1	9521	Unique	Used	0.612	2.818E3	2.335E3	236	4.16	0.00	0	53	271	3	532.30322	1594.89511	-5.77	39.51	3937	3937	MS2	16/44	Glioma_Nuclear_prep_Gradeall_repeat_4plex.raw
6005721	High		lSFGLEDEPLETATk	Unambiguous	1	1	N-Term(iTRAQ4plex); K15(iTRAQ4plex)	HCD	1.0000	0.0000	1	1	10825	Unique	Used	0.635	8.911E4	7.122E4	173	6.95	0.00	0	12	35	2	969.50555	1938.00383	-14.05	47.11	5531	5531	MS2	24/28	Glioma_Nuclear_prep_Gradeall_4plex.raw
6912292	NP_036249.1	CBX5	23468	chromobox protein homolog 5 [Homo sapiens]	16.75	1	2	4	1.510	3;1	5.1	18.47	191	22.2	5.86																			
6912292	High		nLDcPELISEFMk	Unambiguous	1	1	N-Term(iTRAQ4plex); C4(Methylthio); K13(iTRAQ4plex)	HCD	1.0000	0.0000	1	1	13068	Unique	Used	1.574	2.754E3	5.452E3	149	5.02	0.00	0	32	300	2	936.96429	1872.92131	3.45	71.58	8117	8117	MS2	20/24	Glioma_Nuclear_prep_Gradeall_4plex.raw
6912292	High		nLDcPELISEFmk	Unambiguous	1	1	N-Term(iTRAQ4plex); C4(Methylthio); M12(Oxidation); K13(iTRAQ4plex)	HCD	1.0000	0.0000	1	1	12376	Unique	Used	1.450	7.901E3	1.441E4	125	4.66	0.00	0	41	300	2	944.96069	1888.91411	2.30	60.87	7225	7225	MS2	18/24	Glioma_Nuclear_prep_Gradeall_4plex.raw
6912292	High		gQVEYLLk	Unambiguous	1	1	N-Term(iTRAQ4plex); K8(iTRAQ4plex)	HCD	1.0000	0.0000	1	1	9000	Unique	Used	1.431	2.145E5	3.860E5	273	3.32	0.00	0	28	10	2	619.36328	1237.71929	-16.31	33.53	3539	3539	MS2	14/14	Glioma_Nuclear_prep_Gradeall_4plex.raw
6912292		High	gQVEYLLk	Unambiguous	1	1	N-Term(iTRAQ4plex); K8(iTRAQ4plex)	HCD	1.0000	0.0000	1	1	9668	Unique	Used	1.570	2.041E4	4.340E4	223	3.65	0.00	0	24	223	2	619.37402	1237.74077	1.05	41.17	4096	4096	MS2	14/14	Glioma_Nuclear_prep_Gradeall_repeat_4plex.raw
6912676	NP_036377.1	SNW1	22938	SNW domain-containing protein 1 [Homo sapiens]	9.70	1	1	2	1.619	1;1	15.0	13.98	536	61.5	9.52																			
6912676	High		lAEALYIADR	Unambiguous	1	1	N-Term(iTRAQ4plex)	HCD	1.0000	0.0000	1	1	10223	Unique	Used	1.447	1.596E5	2.904E5	317	3.25	0.00	0	33	15	2	639.85272	1278.69817	-15.09	42.52	4873	4873	MS2	16/18	Glioma_Nuclear_prep_Gradeall_4plex.raw
6912676		High	lAEALYIADR	Unambiguous	1	1	N-Term(iTRAQ4plex)	HCD	1.0000	0.0000	1	1	11049	Unique	Used	1.791	6.346E3	1.539E4	347	3.47	0.00	0	37	300	2	639.86261	1278.71794	0.37	57.79	5588	5588	MS2	17/18	Glioma_Nuclear_prep_Gradeall_repeat_4plex.raw
8922328	NP_060517.1	RBM22	55696	pre-mRNA-splicing factor RBM22 [Homo sapiens]	2.62	1	1	2	1.493	1;1	4.1	4.08	420	46.9	8.54																			
8922328	High		yYGINDPVADk	Unambiguous	1	1	N-Term(iTRAQ4plex); K11(iTRAQ4plex)	HCD	1.0000	0.0000	1	1	9205	Unique	Used	1.450	2.534E4	4.620E4	167	3.13	0.00	0	20	20	2	771.89270	1542.77812	-16.94	35.04	3763	3763	MS2	17/20	Glioma_Nuclear_prep_Gradeall_4plex.raw
8922328		High	yYGINDPVADk	Unambiguous	1	1	N-Term(iTRAQ4plex); K11(iTRAQ4plex)	HCD	1.0000	0.0000	1	1	9680	Unique	Used	1.537	5.948E3	1.238E4	224	4.08	0.00	0	33	300	2	771.90765	1542.80803	2.45	41.30	4108	4108	MS2	20/20	Glioma_Nuclear_prep_Gradeall_repeat_4plex.raw
8923444	NP_060308.1	NHP2	55651	H/ACA ribonucleoprotein complex subunit 2 isoform a [Homo sapiens]	36.60	2	3	5	1.510	2;3	9.0	19.22	153	17.2	8.22																			
8923444	High		tYQELLVNQNPIAQ PLASR	Unambiguous	2	1	N-Term(iTRAQ4plex)	HCD	1.0000	0.0000	1	1	10861	Unique	Used	1.451	2.155E4	3.933E4	145	5.33	0.00	0	2	17	2	1150.11169	2299.21611	-15.93	47.38	5570	5570	MS2	27/36	Glioma_Nuclear_prep_Gradeall_4plex.raw
8923444		High	tYQELLVNQNPIAQ PLASR	Unambiguous	2	1	N-Term(iTRAQ4plex)	HCD	1.0000	0.0000	1	1	11631	Unique	Used	1.418	3.712E2	7.127E2	122	5.82	0.00	0	3	300	2	1150.12976	2299.25224	-0.21	65.98	6285	6285	MS2	26/36	Glioma_Nuclear_prep_Gradeall_repeat_4plex.raw
8923444	High		nLPYVYIPSk	Unambiguous	1	1	N-Term(iTRAQ4plex); K10(iTRAQ4plex)	HCD	1.0000	0.0000	1	1	10164	Unique	Used	1.735	1.775E5	3.873E5	254	3.46	0.00	0	22	10	2	741.42297	1481.83867	-14.82	42.09	4809	4809	MS2	16/18	Glioma_Nuclear_prep_Gradeall_4plex.raw
8923444		High	tDLGAAAGSk	Unambiguous	1	1	N-Term(iTRAQ4plex); K10(iTRAQ4plex)	HCD	1.0000	0.0000	1	1	7257	Unique	Used	1.510	3.368E4	6.887E4	221	3.54	0.00	0	0	53	2	589.82434	1178.64141	-17.45	17.01	1540	1540	MS2	17/18	Glioma_Nuclear_prep_Gradeall_repeat_4plex.raw
8923444		High	nLPYVYIPSk	Unambiguous	1	1	N-Term(iTRAQ4plex); K10(iTRAQ4plex)	HCD	1.0000	0.0000	1	1	10987	Unique	Used	1.676	5.660E3	1.285E4	210	4.12	0.00	0	21	300	2	741.43561	1481.86394	2.23	56.98	5517	5517	MS2	16/18	Glioma_Nuclear_prep_Gradeall_repeat_4plex.raw
8923900	NP_061156.1	CMAS	55907	N-acylneuraminate cytidylyltransferase [Homo sapiens]	12.67	1	4	7	0.535	3;4	20.8	19.63	434	48.3	7.93																			
8923900		High	eEGYDSVFSVVR	Unambiguous	1	1	N-Term(iTRAQ4plex)	HCD	1.0000	0.0000	1	1	11058	Unique	Used	0.439	6.205E3	3.687E3	161	4.16	0.00	0	38	300	2	765.88263	1530.75798	1.49	57.91	5598	5598	MS2	21/22	Glioma_Nuclear_prep_Gradeall_repeat_4plex.raw
8923900	High		vGLSGAPADAcSTA Qk	Unambiguous	1	1	N-Term(iTRAQ4plex); C11(Methylthio); K16(iTRAQ4plex)	HCD	1.0000	0.0000	1	1	9221	Unique	Used	0.453	1.659E5	9.449E4	97	5.45	0.00	0	6	11	2	905.44586	1809.88445	-12.90	35.16	3781	3781	MS2	26/30	Glioma_Nuclear_prep_Gradeall_4plex.raw
8923900		High	vGLSGAPADAcSTA Qk	Unambiguous	1	1	N-Term(iTRAQ4plex); C11(Methylthio); K16(iTRAQ4plex)	HCD	1.0000	0.0000	1	1	9709	Unique	Used	0.605	5.222E3	4.276E3	188	5.89	0.00	0	7	300	2	905.46283	1809.91838	5.85	41.63	4139	4139	MS2	23/30	Glioma_Nuclear_prep_Gradeall_repeat_4plex.raw
8923900	High		eVTEPLNLNPAk	Unambiguous	1	1	N-Term(iTRAQ4plex); K12(iTRAQ4plex)	HCD	1.0000	0.0000	1	1	9120	Unique	Used	0.551	1.315E5	9.112E4	239	4.08	0.00	0	20	20	2	806.94867	1612.89006	-15.40	34.43	3671	3671	MS2	19/22	Glioma_Nuclear_prep_Gradeall_4plex.raw
8923900		High	eVTEPLNLNPAk	Unambiguous	1	1	N-Term(iTRAQ4plex); K12(iTRAQ4plex)	HCD	1.0000	0.0000	1	1	9671	Unique	Used	0.535	8.276E3	5.999E3	226	4.95	0.00	0	34	300	2	806.96393	1612.92058	3.52	41.20	4099	4099	MS2	20/22	Glioma_Nuclear_prep_Gradeall_repeat_4plex.raw
8923900	High		gVEkPPHLAALILA R	Unambiguous	1	1	N-Term(iTRAQ4plex); K4(iTRAQ4plex)	HCD	1.0000	0.0000	1	1	9767	Unique	Used	0.743	6.679E4	6.244E4	42	3.88	0.00	0	23	25	2	937.07251	1873.13774	-13.29	39.16	4376	4376	MS2	17/28	Glioma_Nuclear_prep_Gradeall_4plex.raw
8923900		High	gVEkPPHLAALILA R	Unambiguous	1	1	N-Term(iTRAQ4plex); K4(iTRAQ4plex)	HCD	1.0000	0.0000	1	1	10834	Unique	Used	0.432	4.342E2	2.542E2	21	4.63	0.00	0	77	300	4	469.04745	1873.16799	2.86	55.12	5351	5351	MS2	28/84	Glioma_Nuclear_prep_Gradeall_repeat_4plex.raw
8923942	NP_061118.1	NOP10	55505	H/ACA ribonucleoprotein complex subunit 3 [Homo sapiens]	20.31	1	1	3	1.774	2;1	5.8	8.73	64	7.7	9.99																			
8923942	High		mFLQYYLNEQGD R	Unambiguous	1	1	N-Term(iTRAQ4plex)	HCD	1.0000	0.0000	1	1	11445	Unique	Used	1.868	3.464E4	8.137E4	137	4.94	0.00	0	16	53	2	910.94080	1820.87432	-0.83	52.15	6208	6208	MS2	19/24	Glioma_Nuclear_prep_Gradeall_4plex.raw
8923942	High		mFLQYYLNEQGD R	Unambiguous	1	1	N-Term(iTRAQ4plex); M1(Oxidation)	HCD	1.0000	0.0000	1	1	10673	Unique	Used	1.774	3.556E4	7.935E4	90	3.78	0.00	0	8	42	2	918.92694	1836.84661	-13.14	45.94	5365	5365	MS2	18/24	Glioma_Nuclear_prep_Gradeall_4plex.raw
8923942		High	mFLQYYLNEQGD R	Unambiguous	1	1	N-Term(iTRAQ4plex); M1(Oxidation)	HCD	1.0000	0.0000	1	1	11470	Unique	Used	1.661	8.466E2	1.905E3	132	3.80	0.00	0	9	300	2	918.94031	1836.87334	1.41	63.76	6095	6095	MS2	16/24	Glioma_Nuclear_prep_Gradeall_repeat_4plex.raw
10567816	NP_066268.1	GNAO1	2775	guanine nucleotide-binding protein G(o) subunit alpha isoform a [Homo sapiens]	13.56	15	4	5	0.626	2;3	24.6	11.97	354	40.0	5.53																			
10567816	High		iGAADYQPTEQDIL R	Unambiguous	2	1	N-Term(iTRAQ4plex)	HCD	1.0000	0.0000	1	1	9898	Unique	Used	0.389	9.391E4	4.596E4	151	4.41	0.00	0	3	13	2	917.46368	1833.92009	-14.33	40.12	4519	4519	MS2	21/28	Glioma_Nuclear_prep_Gradeall_4plex.raw
10567816	High		lLLLGAGESGk	Unambiguous	15	1	N-Term(iTRAQ4plex); K11(iTRAQ4plex)	HCD	1.0000	0.0000	1	1	9904	Unique	Used	0.699	1.203E5	1.058E5	260	3.65	0.00	0	43	16	2	673.40656	1345.80583	-17.49	40.16	4525	4525	MS2	17/20	Glioma_Nuclear_prep_Gradeall_4plex.raw
10567816		High	lLLLGAGESGk	Unambiguous	15	1	N-Term(iTRAQ4plex); K11(iTRAQ4plex)	HCD	1.0000	0.0000	1	1	10694	Unique	Used	0.626	4.606E3	3.905E3	196	3.69	0.00	0	48	300	2	673.41809	1345.82891	-0.35	53.36	5197	5197	MS2	16/20	Glioma_Nuclear_prep_Gradeall_repeat_4plex.raw
10567816		High	iIHEDGFSGEDVk	Unambiguous	2	1	N-Term(iTRAQ4plex); K13(iTRAQ4plex)	HCD	1.0000	0.0000	1	1	9029	Unique	Used	0.721	1.644E3	1.607E3	189	4.55	0.00	0	10	300	2	867.45264	1733.89800	1.79	34.06	3413	3413	MS2	21/24	Glioma_Nuclear_prep_Gradeall_repeat_4plex.raw
10567816		High	eYQLNDSAk	Unambiguous	2	1	N-Term(iTRAQ4plex); K9(iTRAQ4plex)	HCD	1.0000	0.0000	1	1	7816	Unique	Used	0.483	1.712E4	1.119E4	194	3.74	0.00	0	26	192	2	678.35736	1355.70745	2.14	22.00	2132	2132	MS2	16/16	Glioma_Nuclear_prep_Gradeall_repeat_4plex.raw
10800138	NP_066407.1	HIST1H2BD	3017	histone H2B type 1-D [Homo sapiens]	42.06	11	2	5	2.499	2;3	32.6	69.78	126	13.9	10.32																			
10800138		High	kESYSVYVYk	Unambiguous	10	1	N-Term(iTRAQ4plex); K1(iTRAQ4plex); K10(iTRAQ4plex)	HCD	1.0000	0.0000	1	1	8878	Unique	Used	2.338	6.454E3	2.044E4	250	3.57	0.00	1	8	133	2	849.47577	1697.94426	-1.87	32.44	3251	3251	MS2	18/18	Glioma_Nuclear_prep_Gradeall_repeat_4plex.raw
10800138	High		eSYSVYVYk	Unambiguous	10	1	N-Term(iTRAQ4plex); K9(iTRAQ4plex)	HCD	1.0000	0.0000	1	1	9093	Unique	Used	2.491	8.509E4	2.666E5	173	3.72	0.00	0	21	9	2	713.36658	1425.72588	-17.20	34.24	3642	3642	MS2	16/16	Glioma_Nuclear_prep_Gradeall_4plex.raw
10800138	High		eSYSVYVYk	Unambiguous	10	1	N-Term(iTRAQ4plex); K9(iTRAQ4plex)	HCD	1.0000	0.0000	1	1	9009	Unique	Used	3.852	5.070E4	2.456E5	182	4.10	0.00	0	20	23	2	713.36597	1425.72466	-18.05	33.58	3548	3548	MS2	16/16	Glioma_Nuclear_prep_Gradeall_4plex.raw
10800138		High	eSYSVYVYk	Unambiguous	10	1	N-Term(iTRAQ4plex); K9(iTRAQ4plex)	HCD	1.0000	0.0000	1	1	9638	Unique	Used	2.499	4.043E3	1.369E4	222	4.22	0.00	0	29	300	2	713.37665	1425.74602	-3.07	40.83	4064	4064	MS2	15/16	Glioma_Nuclear_prep_Gradeall_repeat_4plex.raw
10800138		High	eSYSVYVYk	Unambiguous	10	1	N-Term(iTRAQ4plex); K9(iTRAQ4plex)	HCD	1.0000	0.0000	1	1	9542	Unique	Used	4.653	8.585E3	5.410E4	183	4.08	0.00	0	27	90	2	713.37048	1425.73369	-11.72	39.77	3961	3961	MS2	16/16	Glioma_Nuclear_prep_Gradeall_repeat_4plex.raw
10800140	NP_066406.1	HIST1H2BB	3018	histone H2B type 1-B [Homo sapiens]	42.06	6	2	3	2.666	2;1	23.0	69.41	126	13.9	10.32																			
10800140	High		kESYSIYVYk	Unambiguous	5	1	N-Term(iTRAQ4plex); K1(iTRAQ4plex); K10(iTRAQ4plex)	HCD	1.0000	0.0000	1	1	8667	Unique	Used	2.666	4.290E4	1.438E5	170	3.34	0.00	1	26	15	2	856.47137	1711.93547	-16.13	31.14	3178	3178	MS2	18/18	Glioma_Nuclear_prep_Gradeall_4plex.raw
10800140	High		eSYSIYVYk	Unambiguous	5	1	N-Term(iTRAQ4plex); K9(iTRAQ4plex)	HCD	1.0000	0.0000	1	1	9411	Unique	Used	1.737	1.247E5	2.724E5	219	4.11	0.00	0	26	7	2	720.37354	1439.73979	-18.23	36.59	3990	3990	MS2	15/16	Glioma_Nuclear_prep_Gradeall_4plex.raw
10800140		High	eSYSIYVYk	Unambiguous	5	1	N-Term(iTRAQ4plex); K9(iTRAQ4plex)	HCD	1.0000	0.0000	1	1	10054	Unique	Used	2.696	9.556E3	3.490E4	255	4.37	0.00	0	48	226	2	720.38745	1439.76763	1.10	45.74	4512	4512	MS2	15/16	Glioma_Nuclear_prep_Gradeall_repeat_4plex.raw
10800144	NP_066544.1	HIST1H2AJ	8331	histone cluster 1, H2aj [Homo sapiens]	27.34	11	1	7	4.403	6;1	35.2	65.65	128	13.9	10.89																			
10800144	High		vTIAQGGVLPNIQA VLLPk	Unambiguous	10	1	N-Term(iTRAQ4plex); K19(iTRAQ4plex)	HCD	1.0000	0.0000	1	1	12555	Unique	Used	2.114	6.047E2	1.608E3	23	6.34	0.00	0	32	147	3	740.46149	2219.36991	-1.41	63.09	7429	7429	MS2	25/72	Glioma_Nuclear_prep_Gradeall_4plex.raw
10800144	High		vTIAQGGVLPNIQA VLLPk	Unambiguous	10	1	N-Term(iTRAQ4plex); K19(iTRAQ4plex)	HCD	1.0000	0.0000	1	1	12514	Unique	Used	3.286	2.084E3	8.610E3	21	7.78	0.00	0	3	185	2	1110.18896	2219.37065	-1.07	62.53	7381	7381	MS2	24/36	Glioma_Nuclear_prep_Gradeall_4plex.raw
10800144	High		vTIAQGGVLPNIQA VLLPk	Unambiguous	10	1	N-Term(iTRAQ4plex); K19(iTRAQ4plex)	HCD	1.0000	0.0000	1	1	12457	Unique	Used	4.403	2.333E4	1.292E5	23	8.00	0.00	0	2	13	2	1110.18958	2219.37187	-0.52	61.81	7316	7316	MS2	27/36	Glioma_Nuclear_prep_Gradeall_4plex.raw
10800144	High		vTIAQGGVLPNIQA VLLPk	Unambiguous	10	1	N-Term(iTRAQ4plex); K19(iTRAQ4plex)	HCD	1.0000	0.0000	1	1	12404	Unique	Used	4.570	2.046E3	1.176E4	25	7.87	0.00	0	3	249	2	1110.18555	2219.36382	-4.15	61.18	7256	7256	MS2	23/36	Glioma_Nuclear_prep_Gradeall_4plex.raw
10800144	High		vTIAQGGVLPNIQA VLLPk	Unambiguous	10	1	N-Term(iTRAQ4plex); K19(iTRAQ4plex)	HCD	1.0000	0.0000	1	1	12498	Unique	Used	4.924	2.104E3	1.303E4	20	5.94	0.00	0	6	14	3	740.46484	2219.37998	3.13	62.33	7363	7363	MS2	28/72	Glioma_Nuclear_prep_Gradeall_4plex.raw
10800144	High		vTIAQGGVLPNIQA VLLPk	Unambiguous	10	1	N-Term(iTRAQ4plex); K19(iTRAQ4plex)	HCD	1.0000	0.0000	1	1	12479	Unique	Used	6.658	6.726E1	5.632E2	24	5.70	0.00	0	5	52	4	555.59857	2219.37246	-0.26	62.06	7340	7340	MS2	29/108	Glioma_Nuclear_prep_Gradeall_4plex.raw
10800144		High	vTIAQGGVLPNIQA VLLPk	Unambiguous	10	1	N-Term(iTRAQ4plex); K19(iTRAQ4plex)	HCD	1.0000	0.0000	1	1	12459	Unique	Used	3.203	6.621E1	2.872E2	25	5.33	0.00	0	6	129	3	740.46295	2219.37430	0.57	88.80	7985	7985	MS2	26/72	Glioma_Nuclear_prep_Gradeall_repeat_4plex.raw
10835071	NP_004346.1	CD74	972	HLA class II histocompatibility antigen gamma chain isoform b [Homo sapiens]	12.93	3	2	2	1.843	1;1	7.2	9.34	232	26.4	9.14																			
10835071	High		lTVTSQNLQLENLR	Unambiguous	3	1	N-Term(iTRAQ4plex)	HCD	1.0000	0.0000	1	1	10206	Unique	Used	1.300	4.823E4	7.883E4	286	4.83	0.00	0	38	43	2	886.99127	1772.97527	-13.24	42.39	4854	4854	MS2	21/26	Glioma_Nuclear_prep_Gradeall_4plex.raw
10835071		High	eSLELEDPSSGLG VTk	Unambiguous	1	1	N-Term(iTRAQ4plex); K16(iTRAQ4plex)	HCD	1.0000	0.0000	1	1	10451	Unique	Used	2.386	7.302E2	2.360E3	240	5.55	0.00	0	32	300	2	975.01996	1949.03264	0.43	50.43	4935	4935	MS2	24/30	Glioma_Nuclear_prep_Gradeall_repeat_4plex.raw
11225260	NP_003277.1	TOP1	7150	DNA topoisomerase 1 [Homo sapiens]	12.42	3	7	9	1.934	5;4	12.9	25.37	765	90.7	9.31																			
11225260	High		cDFTQMSQYFk	Unambiguous	1	1	N-Term(iTRAQ4plex); C1(Methylthio); K11(iTRAQ4plex)	HCD	1.0000	0.0000	1	1	11940	Unique	Used	1.845	2.399E4	5.569E4	54	5.02	0.00	0	42	115	2	866.39508	1731.78288	2.63	56.40	6746	6746	MS2	19/20	Glioma_Nuclear_prep_Gradeall_4plex.raw
11225260	High		iMPEDIINcSk	Unambiguous	1	1	N-Term(iTRAQ4plex); C10(Methylthio); K12(iTRAQ4plex)	HCD	1.0000	0.0000	1	1	11954	Unique	Used	1.934	2.155E3	5.240E3	238	3.73	0.00	0	23	66	3	570.63281	1709.88388	-2.35	56.54	6762	6762	MS2	19/44	Glioma_Nuclear_prep_Gradeall_4plex.raw
11225260	High		iMPEDIINcSk	Unambiguous	1	1	N-Term(iTRAQ4plex); C10(Methylthio); K12(iTRAQ4plex)	HCD	1.0000	0.0000	1	1	11948	Unique	Used	2.280	1.974E4	5.663E4	273	4.81	0.00	0	38	152	2	855.44910	1709.89092	1.76	56.47	6755	6755	MS2	18/22	Glioma_Nuclear_prep_Gradeall_4plex.raw
11225260		High	iTVAWck	Unambiguous	1	1	N-Term(iTRAQ4plex); C6(Methylthio); K7(iTRAQ4plex)	HCD	1.0000	0.0000	1	1	11310	Unique	Used	1.920	4.160E3	1.082E4	240	2.86	0.00	0	59	300	2	577.81921	1154.63115	0.60	61.46	5900	5900	MS2	12/12	Glioma_Nuclear_prep_Gradeall_repeat_4plex.raw
11225260	High		lEVQATDREENk	Unambiguous	1	1	N-Term(iTRAQ4plex); K12(iTRAQ4plex)	HCD	1.0000	0.0000	1	1	7564	Unique	Used	2.045	1.577E4	4.057E4	266	4.45	0.00	1	29	38	2	860.45923	1719.91118	-0.25	23.27	1980	1980	MS2	19/22	Glioma_Nuclear_prep_Gradeall_4plex.raw
11225260	High		gPVFAPPYEPLPE NVk	Unambiguous	1	1	N-Term(iTRAQ4plex); K16(iTRAQ4plex)	HCD	1.0000	0.0000	1	1	10510	Unique	Used	2.198	4.554E4	1.259E5	190	4.58	0.00	0	2	11	2	1021.54779	2042.08830	-15.60	44.73	5188	5188	MS2	21/30	Glioma_Nuclear_prep_Gradeall_4plex.raw

11225260		High	gPVFAPPYEPLPENVk	Unambiguous	1	1	N-Term(iTRAQ4plex); K16(iTRAQ4plex)	HCD	1.0000	0.0000	1	1	11346	Unique	Used	2.315	6.586E2	2.065E3	167	4.27	0.00	0	12	300	2	1021.56476	2042.12224	1.02	62.07	5949	5949	MS2	19/30	Glioma_Nuclear_prep_Gradeall_repeat_4plex.raw
11225260		High	nIITNLSk	Unambiguous	1	1	N-Term(iTRAQ4plex); K8(iTRAQ4plex)	HCD	1.0000	0.0000	1	1	9084	Unique	Used	1.501	1.562E4	3.175E4	173	2.84	0.00	0	58	300	2	595.87122	1190.73516	0.37	34.69	3472	3472	MS2	13/14	Glioma_Nuclear_prep_Gradeall_repeat_4plex.raw
11225260		High	qIALGTSk	Unambiguous	1	1	N-Term(iTRAQ4plex); K8(iTRAQ4plex)	HCD	1.0000	0.0000	1	1	7830	Unique	Used	1.837	2.042E4	5.080E4	331	2.96	0.00	0	44	165	2	553.34253	1105.67778	-3.78	22.15	2147	2147	MS2	13/14	Glioma_Nuclear_prep_Gradeall_repeat_4plex.raw
12056465	NP_001427.2	FBL	2091	rRNA 2'-O-methyltransferase fibrillarin [Homo sapiens]	31.15	1	6	11	1.662	3;8	13.4	38.70	321	33.8	10.18																			
12056465		High	hEGVFIcR	Unambiguous	1	1	N-Term(iTRAQ4plex); C7(Methylthio)	HCD	1.0000	0.0000	1	1	9155	Unique	Used	1.624	4.972E3	1.094E4	116	3.64	0.00	0	18	211	3	384.19284	1150.56397	1.86	35.48	3547	3547	MS2	16/28	Glioma_Nuclear_prep_Gradeall_repeat_4plex.raw
12056465	High		nLVPGESVYGEk	Unambiguous	1	1	N-Term(iTRAQ4plex); K12(iTRAQ4plex)	HCD	1.0000	0.0000	1	1	8809	Unique	Used	1.564	1.518E5	2.986E5	208	3.00	0.00	0	8	9	2	790.41895	1579.83061	-16.73	32.16	3332	3332	MS2	15/22	Glioma_Nuclear_prep_Gradeall_4plex.raw
12056465		High	nLVPGESVYGEk	Unambiguous	1	1	N-Term(iTRAQ4plex); K12(iTRAQ4plex)	HCD	1.0000	0.0000	1	1	9264	Unique	Used	1.394	2.627E3	4.963E3	221	3.84	0.00	0	14	132	3	527.29169	1579.86051	2.20	36.68	3662	3662	MS2	16/44	Glioma_Nuclear_prep_Gradeall_repeat_4plex.raw
12056465		High	nLVPGESVYGEk	Unambiguous	1	1	N-Term(iTRAQ4plex); K12(iTRAQ4plex)	HCD	1.0000	0.0000	1	1	9248	Unique	Used	1.653	1.021E4	2.286E4	269	3.51	0.00	0	23	198	2	790.43115	1579.85503	-1.27	36.50	3645	3645	MS2	14/22	Glioma_Nuclear_prep_Gradeall_repeat_4plex.raw
12056465	High		dHAVVVGVYRPPPk	Unambiguous	1	1	N-Term(iTRAQ4plex); K14(iTRAQ4plex)	HCD	1.0000	0.0000	1	1	7928	Unique	Used	1.900	6.724E4	1.607E5	124	3.74	0.00	0	18	2	3	608.02338	1822.05558	-1.23	25.86	2375	2375	MS2	25/52	Glioma_Nuclear_prep_Gradeall_4plex.raw
12056465		High	dHAVVVGVYRPPPk	Unambiguous	1	1	N-Term(iTRAQ4plex); K14(iTRAQ4plex)	HCD	1.0000	0.0000	1	1	8259	Unique	Used	1.662	3.230E3	7.270E3	103	3.51	0.00	0	27	110	3	608.02539	1822.06162	2.09	26.28	2599	2599	MS2	23/52	Glioma_Nuclear_prep_Gradeall_repeat_4plex.raw
12056465		High	dHAVVVGVYRPPPk	Unambiguous	1	1	N-Term(iTRAQ4plex); K14(iTRAQ4plex)	HCD	1.0000	0.0000	1	1	8268	Unique	Used	2.024	2.683E3	7.355E3	136	5.33	0.00	0	14	300	2	911.53418	1822.06108	1.79	26.37	2608	2608	MS2	21/26	Glioma_Nuclear_prep_Gradeall_repeat_4plex.raw
12056465	High		IAAAILGGVDQIHIkPGAk	Unambiguous	1	1	N-Term(iTRAQ4plex); K15(iTRAQ4plex); K19(iTRAQ4plex)	HCD	1.0000	0.0000	1	1	10893	Unique	Used	1.886	5.211E3	1.236E4	21	4.98	0.00	0	29	7	4	576.85016	2304.37880	-14.77	47.64	5605	5605	MS2	35/108	Glioma_Nuclear_prep_Gradeall_4plex.raw
12056465		High	gkEDALVTk	Unambiguous	1	1	N-Term(iTRAQ4plex); K2(iTRAQ4plex); K9(iTRAQ4plex)	HCD	1.0000	0.0000	1	1	7353	Unique	Used	1.736	1.376E4	3.236E4	218	2.90	0.00	1	12	113	2	696.92523	1392.84319	0.65	17.87	1641	1641	MS2	15/16	Glioma_Nuclear_prep_Gradeall_repeat_4plex.raw
12056465		High	gkEDALVTk	Unambiguous	1	1	N-Term(iTRAQ4plex); K2(iTRAQ4plex); K9(iTRAQ4plex)	HCD	1.0000	0.0000	1	1	7374	Unique	Used	2.038	1.733E4	4.782E4	285	2.89	0.00	1	12	41	2	696.91254	1392.81780	-17.58	18.04	1663	1663	MS2	15/16	Glioma_Nuclear_prep_Gradeall_repeat_4plex.raw
12056465		High	dLINLAk	Unambiguous	1	1	N-Term(iTRAQ4plex); K7(iTRAQ4plex)	HCD	1.0000	0.0000	1	1	9685	Unique	Used	1.362	4.911E4	9.058E4	265	3.26	0.00	0	30	94	2	537.84229	1074.67729	1.07	41.36	4114	4114	MS2	12/12	Glioma_Nuclear_prep_Gradeall_repeat_4plex.raw
13676857	NP_068814.2	HSPA2	3306	heat shock-related 70 kDa protein 2 [Homo sapiens]	13.77	1	2	2	0.449	1;1	2.9	27.89	639	70.0	5.74																			
13676857	High		cQEVINWLDR	Unambiguous	1	1	N-Term(iTRAQ4plex); C1(Methylthio)	HCD	1.0000	0.0000	1	1	12170	Unique	Used	0.458	2.892E4	1.665E4	132	3.67	0.00	0	24	241	2	733.35657	1465.70586	0.67	58.66	6998	6998	MS2	16/18	Glioma_Nuclear_prep_Gradeall_4plex.raw
13676857		High	vcNPIISk	Unambiguous	1	1	N-Term(iTRAQ4plex); C2(Methylthio); K8(iTRAQ4plex)	HCD	1.0000	0.0000	1	1	9805	Unique	Used	0.440	1.984E4	1.181E4	357	3.28	0.00	0	69	300	2	604.34686	1207.68645	6.88	42.72	4240	4240	MS2	13/14	Glioma_Nuclear_prep_Gradeall_repeat_4plex.raw
14043072	NP_112533.1	HNRNPA2B1	3181	heterogeneous nuclear ribonucleoproteins A2/B1 isoform B1 [Homo sapiens]	46.74	2	12	29	0.173	10;19	39.4	112.17	353	37.4	8.95																			
14043072		High	nMGGPYGGGNYGPGGSGGSGGYGGR	Unambiguous	2	1	N-Term(iTRAQ4plex)	HCD	1.0000	0.0000	1	1	8851	Unique	Used	0.250	2.137E3	7.242E2	22	8.92	0.00	0	14	179	3	778.67645	2334.01480	3.12	32.16	3222	3222	MS2	28/96	Glioma_Nuclear_prep_Gradeall_repeat_4plex.raw
14043072	High		IFIGGLSFETTEESLR	Unambiguous	2	1	N-Term(iTRAQ4plex)	HCD	1.0000	0.0000	1	1	12314	Unique	Used	0.104	1.086E5	1.413E4	203	5.62	0.00	0	31	64	2	972.01392	1943.02056	-1.93	60.20	7158	7158	MS2	22/30	Glioma_Nuclear_prep_Gradeall_4plex.raw
14043072	High		IFIGGLSFETTEESLR	Unambiguous	2	1	N-Term(iTRAQ4plex)	HCD	1.0000	0.0000	1	1	12251	Unique	Used	0.113	6.302E4	8.917E3	208	5.65	0.00	0	15	116	2	972.01471	1943.02214	-1.11	59.51	7088	7088	MS2	22/30	Glioma_Nuclear_prep_Gradeall_4plex.raw
14043072	High		IFIGGLSFETTEESLR	Unambiguous	2	1	N-Term(iTRAQ4plex)	HCD	1.0000	0.0000	1	1	12382	Unique	Used	0.163	2.532E4	5.194E3	228	5.43	0.00	0	19	293	2	972.01550	1943.02373	-0.30	60.94	7232	7232	MS2	23/30	Glioma_Nuclear_prep_Gradeall_4plex.raw
14043072	High		gGGGNFGPGPGSNFR	Unambiguous	2	1	N-Term(iTRAQ4plex)	HCD	1.0000	0.0000	1	1	8224	Unique	Used	0.172	2.390E5	5.165E4	63	5.36	0.00	0	14	15	2	761.35645	1521.70561	-17.07	27.93	2695	2695	MS2	22/28	Glioma_Nuclear_prep_Gradeall_4plex.raw
14043072		High	iDTIEIITDR	Unambiguous	2	1	N-Term(iTRAQ4plex)	HCD	1.0000	0.0000	1	1	11150	Unique	Used	0.077	1.558E5	1.627E4	221	4.00	0.00	0	7	30	2	666.88007	1332.75286	2.77	59.14	5704	5704	MS2	17/18	Glioma_Nuclear_prep_Gradeall_repeat_4plex.raw
14043072		High	tLETVPLER	Unambiguous	1	1	N-Term(iTRAQ4plex)	HCD	1.0000	0.0000	1	1	9110	Unique	Used	0.189	1.234E5	3.154E4	205	3.04	0.00	0	17	100	2	601.35022	1201.69316	1.87	34.98	3500	3500	MS2	12/16	Glioma_Nuclear_prep_Gradeall_repeat_4plex.raw
14043072		High	gGGGNFGPGPGSNFR	Unambiguous	2	1	N-Term(iTRAQ4plex)	HCD	1.0000	0.0000	1	1	8567	Unique	Used	0.197	1.939E4	5.183E3	110	5.29	0.00	0	25	123	2	761.37262	1521.73796	4.19	29.30	2924	2924	MS2	25/28	Glioma_Nuclear_prep_Gradeall_repeat_4plex.raw
14043072		High	gGNFGFGDSR	Unambiguous	2	1	N-Term(iTRAQ4plex)	HCD	1.0000	0.0000	1	1	8426	Unique	Used	0.207	4.806E4	1.345E4	92	3.51	0.00	0	42	231	2	579.27667	1157.54607	0.36	27.88	2774	2774	MS2	17/18	Glioma_Nuclear_prep_Gradeall_repeat_4plex.raw
14043072		High	iDTIEIITDR	Unambiguous	2	1	N-Term(iTRAQ4plex)	HCD	1.0000	0.0000	1	1	11075	Unique	Used	0.250	1.142E4	3.870E3	307	3.88	0.00	0	33	300	2	666.87964	1332.75200	2.13	58.13	5617	5617	MS2	16/18	Glioma_Nuclear_prep_Gradeall_repeat_4plex.raw
14043072		High	gGNFGFGDSR	Unambiguous	2	1	N-Term(iTRAQ4plex)	HCD	1.0000	0.0000	1	1	8537	Unique	Used	0.307	1.854E4	7.719E3	66	3.47	0.00	0	27	181	2	579.27179	1157.53630	-8.08	28.99	2892	2892	MS2	17/18	Glioma_Nuclear_prep_Gradeall_repeat_4plex.raw
14043072		High	lTDcVVMR	Unambiguous	2	1	N-Term(iTRAQ4plex); C4(Methylthio)	HCD	1.0000	0.0000	1	1	10860	Unique	Used	0.142	2.349E4	4.514E3	146	3.01	0.00	0	18	288	2	563.78192	1126.55657	2.30	55.43	5379	5379	MS2	13/14	Glioma_Nuclear_prep_Gradeall_repeat_4plex.raw
14043072		High	lTDcVVMR	Unambiguous	2	1	N-Term(iTRAQ4plex); C4(Methylthio)	HCD	1.0000	0.0000	1	1	10778	Unique	Used	0.209	1.356E4	3.831E3	157	2.86	0.00	0	40	300	2	563.78253	1126.55779	3.38	54.39	5288	5288	MS2	12/14	Glioma_Nuclear_prep_Gradeall_repeat_4plex.raw
14043072	High		kLFVGGIk	Unambiguous	2	1	N-Term(iTRAQ4plex); K1(iTRAQ4plex); K8(iTRAQ4plex)	HCD	1.0000	0.0000	1	1	8346	Unique	Used	0.278	2.189E5	7.667E4	26	3.63	0.00	1	59	3	3	431.95651	1293.85498	-5.34	28.81	2828	2828	MS2	19/28	Glioma_Nuclear_prep_Gradeall_4plex.raw
14043072		High	kLFVGGIk	Unambiguous	2	1	N-Term(iTRAQ4plex); K1(iTRAQ4plex); K8(iTRAQ4plex)	HCD	1.0000	0.0000	1	1	8942	Unique	Used	0.222	5.273E4	1.588E4	26	3.03	0.00	1	55	142	2	647.43115	1293.85503	-5.30	33.03	3318	3318	MS2	14/14	Glioma_Nuclear_prep_Gradeall_repeat_4plex.raw
14043072		High	kLFVGGIk	Unambiguous	2	1	N-Term(iTRAQ4plex); K1(iTRAQ4plex); K8(iTRAQ4plex)	HCD	1.0000	0.0000	1	1	9099	Unique	Used	0.238	4.237E3	1.367E3	21	3.61	0.00	1	75	240	3	431.95746	1293.85782	-3.14	34.85	3488	3488	MS2	19/28	Glioma_Nuclear_prep_Gradeall_repeat_4plex.raw
14043072		High	kLFVGGIk	Unambiguous	2	1	N-Term(iTRAQ4plex); K1(iTRAQ4plex); K8(iTRAQ4plex)	HCD	1.0000	0.0000	1	1	8925	Unique	Used	0.272	1.519E4	5.603E3	22	3.38	0.00	1	79	157	3	431.96130	1293.86936	5.77	32.86	3300	3300	MS2	20/28	Glioma_Nuclear_prep_Gradeall_repeat_4plex.raw
14043072		High	kLFVGGIk	Unambiguous	2	1	N-Term(iTRAQ4plex); K1(iTRAQ4plex); K8(iTRAQ4plex)	HCD	1.0000	0.0000	1	1	8749	Unique	Used	0.284	1.310E4	5.043E3	27	3.46	0.00	1	66	115	3	431.95746	1293.85782	-3.14	31.06	3115	3115	MS2	19/28	Glioma_Nuclear_prep_Gradeall_repeat_4plex.raw
14043072	High		gFGFVTFDDHDPVDk	Unambiguous	2	1	N-Term(iTRAQ4plex); K15(iTRAQ4plex)	HCD	1.0000	0.0000	1	1	10340	Unique	Used	0.078	8.865E5	8.692E4	71	4.36	0.00	0	8	1	3	661.98633	1983.94443	-12.45	43.38	5000	5000	MS2	29/56	Glioma_Nuclear_prep_Gradeall_4plex.raw
14043072	High		gFGFVTFDDHDPVDk	Unambiguous	2	1	N-Term(iTRAQ4plex); K15(iTRAQ4plex)	HCD	1.0000	0.0000	1	1	10320	Unique	Used	0.129	1.858E5	3.016E4	71	5.53	0.00	0	21	17	2	992.47443	1983.94158	-13.89	43.23	4978	4978	MS2	25/28	Glioma_Nuclear_prep_Gradeall_4plex.raw
14043072	High		gFGFVTFDDHDPVDk	Unambiguous	2	1	N-Term(iTRAQ4plex); K15(iTRAQ4plex)	HCD	1.0000	0.0000	1	1	10414	Unique	Used	0.229	1.287E5	3.715E4	73	5.45	0.00	0	15	15	2	992.47351	1983.93974	-14.82	43.95	5081	5081	MS2	25/28	Glioma_Nuclear_prep_Gradeall_4plex.raw
14043072		High	gFGFVTFDDHDPVDk	Unambiguous	2	1	N-Term(iTRAQ4plex); K15(iTRAQ4plex)	HCD	1.0000	0.0000	1	1	11238	Unique	Used	0.080	1.030E4	1.115E3	104	4.58	0.00	0	8	96	3	661.99634	1983.97446	2.68	60.29	5805	5805	MS2	27/56	Glioma_Nuclear_prep_Gradeall_repeat_4plex.raw
14043072		High	gFGFVTFDDHDPVDk	Unambiguous	2	1	N-Term(iTRAQ4plex); K15(iTRAQ4plex)	HCD	1.0000	0.0000	1	1	11302	Unique	Used	0.104	6.564E3	9.266E2	99	5.13	0.00	0	8	139	3	661.99646	1983.97483	2.87	61.30	5888	5888	MS2	29/56	Glioma_Nuclear_prep_Gradeall_repeat_4plex.raw
14043072		High	gFGFVTFDDHDPVDk	Unambiguous	2	1	N-Term(iTRAQ4plex); K15(iTRAQ4plex)	HCD	1.0000	0.0000	1	1	11303	Unique	Used	0.129	4.639E3	8.115E2	106	5.29	0.00	0	4	300	2	992.48987	1983.97246	1.67	61.32	5889	5889	MS2	25/28	Glioma_Nuclear_prep_Gradeall_repeat_4plex.raw
14043072		High	gFGFVTFDDHDPVDk	Unambiguous	2	1	N-Term(iTRAQ4plex); K15(iTRAQ4plex)	HCD	1.0000	0.0000	1	1	11240	Unique	Used	0.156	5.874E3	1.238E3	102	5.04	0.00	0	3	300	2	992.48981	1983.97234	1.61	60.31	5807	5807	MS2	25/28	Glioma_Nuclear_prep_Gradeall_repeat_4plex.raw
14043072	High		eESGkPGAHVTVk	Unambiguous	2	1	N-Term(iTRAQ4plex); K5(iTRAQ4plex); K13(iTRAQ4plex)	HCD	1.0000	0.0000	1	1	7223	Unique	Used	0.271	3.951E4	1.348E4	151	4.24	0.00	0	10	67	2	886.00848	1771.00969	1.26	20.73	1608	1608	MS2	18/24	Glioma_Nuclear_prep_Gradeall_4plex.raw
14043072		High	IFVGGIk	Unambiguous	2	1	N-Term(iTRAQ4plex); K7(iTRAQ4plex)	HCD	1.0000	0.0000	1	1	10128	Unique	Used	0.173	2.042E4	4.794E3	204	2.86	0.00	0	67	300	2	511.33368	1021.66008	-4.67	46.62	4592	4592	MS2	12/12	Glioma_Nuclear_prep_Gradeall_repeat_4plex.raw
14043072	High		dYFEEYGk	Unambiguous	2	1	N-Term(iTRAQ4plex); K8(iTRAQ4plex)	HCD	1.0000	0.0000	1	1	9232	Unique	Used	0.075	3.931E6	3.717E5	50	2.92	0.00	0	9	1	2	669.81757	1338.62786	-13.27	35.24	3793	3793	MS2	14/14	Glioma_Nuclear_prep_Gradeall_4plex.raw
14043072		High	dYFEEYGk	Unambiguous	2	1	N-Term(iTRAQ4plex); K8(iTRAQ4plex)	HCD	1.0000	0.0000	1	1	9635	Unique	Used	0.130	7.409E4	1.302E4	88	3.61	0.00	0	36	157	2	669.82617	1338.64507	-0.41	40.80	4061	4061	MS2	13/14	Glioma_Nuclear_prep_Gradeall_repeat_4plex.raw
14141159	NP_067676.2	HNRNPH3	3189	heterogeneous nuclear ribonucleoprotein H3 isoform b [Homo sapiens]	17.82	2	4	7	0.267	6;1	39.2	42.66	331	35.2	6.87																			
14141159	High		aTENDIANFFSPLNPIR	Unambiguous	2	1	N-Term(iTRAQ4plex)	HCD	1.0000	0.0000	1	1	12595	Unique	Used	0.172	4.382E4	9.463E3	175	6.96	0.00	0	2	54	2	1032.03723	2063.06719	-0.35	63.66	7479	7479	MS2	25/32	Glioma_Nuclear_prep_Gradeall_4plex.raw
14141159	High		aTENDIANFFSPLNPIR	Unambiguous	2	1	N-Term(iTRAQ4plex)	HCD	1.0000	0.0000	1	1	12546	Unique	Used	0.267	1.154E4	3.882E3	179	6.80	0.00	0	23	300	2	1032.03882	2063.07036	1.19	62.98	7419	7419	MS2	25/32	Glioma_Nuclear_prep_Gradeall_4plex.raw
14141159	High		aTENDIANFFSPLNPIR	Unambiguous	2	1	N-Term(iTRAQ4plex)	HCD	1.0000	0.0000	1	1	12553	Unique	Used	0.550	1.696E3	1.173E3	166	6.76	0.00	0	12	124	3	688.36096	2063.06833	0.21	63.07	7427	7427	MS2	28/64	Glioma_Nuclear_prep_Gradeall_4plex.raw
14141159	High		sTGEAFVQFASk	Unambiguous	2	1	N-Term(iTRAQ4plex); K12(iTRAQ4plex)	HCD	1.0000	0.0000	1	1	9584	Unique	Used	0.252	1.957E5	6.191E4	152	4.44	0.00	0	18	14	2	780.40826	1559.80925	-13.83	37.85	4178	4178	MS2	21/22	Glioma_Nuclear_prep_Gradeall_4plex.raw
14141159		High	sTGEAFVQFASk	Unambiguous	2	1	N-Term(iTRAQ4plex); K12(iTRAQ4plex)	HCD	1.0000	0.0000	1	1	10260	Unique	Used	0.259	8.339E3	2.926E3	166	4.37	0.00	0	17	300	2	780.42078	1559.83428	2.21	48.15	4731	4731	MS2	21/22	Glioma_Nuclear_prep_Gradeall_repeat_4plex.raw
14141159	High		aTGEADVEFVTHEDAVAAMSk	Unambiguous	2	1	N-Term(iTRAQ4plex); K21(iTRAQ4plex)	HCD	1.0000	0.0000	1	1	10133	Unique	Used	0.412	1.961E4	1.016E4	60	7.47	0.00	0	20	9	3	822.72858	2466.17118	-14.20	41.85	4775	4775	MS2	33/80	Glioma_Nuclear_prep_Gradeall_4plex.raw
14141159	High		eIAENALGk	Unambiguous	2	1	N-Term(iTRAQ4plex); K9(iTRAQ4plex)	HCD	1.0000	0.0000	1	1	8101	Unique	Used	0.406	2.074E5	1.060E5	288	3.80	0.00	0	58	13	2	616.84814	1232.68901	-16.15	27.07	2562	2562	MS2	16/16	Glioma_Nuclear_prep_Gradeall_4plex.raw
14141161	NP_004492.2	HNRNPU	3192	heterogeneous nuclear ribonucleoprotein U isoform b [Homo sapiens]	12.66	2	8	17	0.209	7;10	38.9	68.48	806	88.9	5.78																			
14141161	High		ekPYFPIPEEYTFIQNVPLEDR	Unambiguous	2	1	N-Term(iTRAQ4plex); K2(iTRAQ4plex)	HCD	1.0000	0.0000	1	1	12150	Unique	Used	0.190	6.575E3	1.568E3	108	8.72	0.00	0	6	19	3	1004.86108	3012.56870	2.96	58.49	6977	6977	MS2	27/84	Glioma_Nuclear_prep_Gradeall_4plex.raw
14141161	High		nFILDQTNVSAAAQR	Unambiguous	2	1	N-Term(iTRAQ4plex)	HCD	1.0000	0.0000	1	1	9707	Unique	Used	0.298	1.063E5	3.991E4	262	4.77	0.00	0	33	33	2	896.46423	1791.92119	-14.43	38.73	4311	4311	MS2	21/28	Glioma_Nuclear_prep_Gradeall_4plex.raw
14141161		High	nFILDQTNVSAAAQR	Unambiguous	2	1	N-Term(iTRAQ4plex)	HCD	1.0000	0.0000	1	1	10328	Unique	Used	0.179	5.771E3	1.396E3	239	4.88	0.00	0	4	300	2	896.47925	1791.95122	2.33	49.00	4805	4805	MS2	22/28	Glioma_Nuclear_prep_Gradeall_repeat_4plex.raw
14141161		High	sSGPTSLFAVTVAPPGAR	Unambiguous	2	1	N-Term(iTRAQ4plex)	HCD	1.0000	0.0000	1	1	11410	Unique	Used	0.202	3.250E3	8.886E2	151	5.63	0.00	0	17	300	2	930.01428	1859.02129	3.70	62.95	6025	6025	MS2	26/34	Glioma_Nuclear_prep_Gradeall_repeat_4plex.raw
14141161		High	fIEIAAR	Unambiguous	2	1	N-Term(iTRAQ4plex)	HCD	1.0000	0.0000	1	1	9627	Unique	Used	0.265	2.488E4	8.930E3	333	2.95	0.00	0	28	300	2	482.29086	963.57445	0.03	40.70	4052	4052	MS2	11/12	Glioma_Nuclear_prep_Gradeall_repeat_4plex.raw
14141161	High		gYFEYIEENk	Unambiguous	2	1	N-Term(iTRAQ4plex); K10(iTRAQ4plex)	HCD	1.0000	0.0000	1	1	10317	Unique	Used	0.221	4.219E5	1.174E5	103	4.52	0.00	0	18	7	2	790.38715	1579.76702	-13.45	43.21	4975	4975	MS2	18/18	Glioma_Nuclear_prep_Gradeall_4plex.raw
14141161		High	gYFEYIEENk	Unambiguous	2	1	N-Term(iTRAQ4plex); K10(iTRAQ4plex)	HCD	1.0000	0.0000	1	1	11212	Unique	Used	0.164	7.422E3	1.644E3	148	4.66	0.00	0	14	300	2	790.39941	1579.79155	2.08	59.96	5776	5776	MS2	18/18	Glioma_Nuclear_prep_Gradeall_repeat_4plex.raw
14141161		High	gYFEYIEENk	Unambiguous	2	1	N-Term(iTRAQ4plex); K10(iTRAQ4plex)	HCD	1.0000	0.0000	1	1	11134	Unique	Used	0.348	6.084E3	2.866E3	168	4.56	0.00	0	44	300	2	790.39966	1579.79204	2.39	58.93	5686	5686	MS2	17/18	Glioma_Nuclear_prep_Gradeall_repeat_4plex.raw
14141161	High		yNILGTNTIMDk	Unambiguous	2	1	N-Term(iTRAQ4plex); K12(iTRAQ4plex)	HCD	1.0000	0.0000	1	1	10258	Unique	Used	0.159	6.393E5	1.279E5	211	4.39	0.00	0	16	5	2	835.94427	1670.88127	-12.77	42.78	4911	4911	MS2	20/22	Glioma_Nuclear_prep_Gradeall_4plex.raw
14141161		High	yNILGTNTIMDk	Unambiguous	2	1	N-Term(iTRAQ4plex); K12(iTRAQ4plex)	HCD	1.0000	0.0000	1	1	11149	Unique	Used	0.359	3.341E3	1.623E3	265	3.97	0.00	0	48	300	2	835.95837	1670.90947	4.11	59.12	5702	5702	MS2	18/22	Glioma_Nuclear_prep_Gradeall_repeat_4plex.raw
14141161		High	yNILGTNTIMDk	Unambiguous	2	1	N-Term(iTRAQ4plex); K12(iTRAQ4plex)	HCD	1.0000	0.0000	1	1	11064	Unique	Used	0.476	3.862E3	2.491E3	258	4.10	0.00	0	83	300	2	835.96014	1670.91301	6.23	57.98	5604	5604	MS2	20/22	Glioma_Nuclear_prep_Gradeall_repeat_4plex.raw
14141161	High		vSELkEELk	Unambiguous	2	1	N-Term(iTRAQ4plex); K5(iTRAQ4plex); K9(iTRAQ4plex)	HCD	1.0000	0.0000	1	1	8560	Unique	Used	0.107	3.665E5	4.946E4	225	3.58	0.00	1	9	7	2	753.94598	1506.88469	-17.03	30.33	3060	3060	MS2	16/16	Glioma_Nuclear_prep_Gradeall_4plex.raw
14141161		High	vSELkEELk	Unambiguous	2	1	N-Term(iTRAQ4plex); K5(iTRAQ4plex); K9(iTRAQ4plex)	HCD	1.0000	0.0000	1	1	9145	Unique	Used	0.209	1.333E4	3.783E3	189	3.76	0.00	1	31	300	2	753.95679	1506.90630	-2.70	35.36	3536	3536	MS2	16/16	Glioma_Nuclear_prep_Gradeall_repeat_4plex.raw
14141161	High		dLPEHAVLk	Unambiguous	2	1	N-Term(iTRAQ4plex); K9(iTRAQ4plex)	HCD	1.0000	0.0000	1	1	7934	Unique	Used	0.174	4.843E5	1.063E5	242	3.94	0.00	0	18	8	2	655.38977	1309.77226	0.32	25.89	2381	2381	MS2	16/16	Glioma_Nuclear_prep_Gradeall_4plex.raw
14141161		High	dLPEHAVLk	Unambiguous	2	1	N-Term(iTRAQ4plex); K9(iTRAQ4plex)	HCD	1.0000	0.0000	1	1	8227	Unique	Used	0.340	2.185E4	1.007E4	195	3.75	0.00	0	31	298	2	655.39233	1309.77739	4.23	25.99	2565	2565	MS2	15/16	Glioma_Nuclear_prep_Gradeall_repeat_4plex.raw
14141161	High		yNILGTNTImDk	Unambiguous	2	1	N-Term(iTRAQ4plex); M10(Oxidation); K12(iTRAQ4plex)	HCD	1.0000	0.0000	1	1	9557	Unique	Used	0.180	2.004E5	4.539E4	195	4.09	0.00	0	18	13	2	843.94183	1686.87639	-12.53	37.66	4149	4149	MS2	21/22	Glioma_Nuclear_prep_Gradeall_4plex.raw
14141161		High	yNILGTNTImDk	Unambiguous	2	1	N-Term(iTRAQ4plex); M10(Oxidation); K12(iTRAQ4plex)	HCD	1.0000	0.0000	1	1	10183	Unique	Used	0.255	5.736E3	1.983E3	223	4.31	0.00	0	10	300	2	843.95459	1686.90190	2.60	47.27	4650	4650	MS2	20/22	Glioma_Nuclear_prep_Gradeall_repeat_4plex.raw
14165435	NP_112552.1	HNRNPK	3190	heterogeneous nuclear ribonucleoprotein K isoform b [Homo sapiens]	41.90	2	14	28	0.225	13;15	38.6	115.96	463	50.9	5.54																			
14165435	High		tDYNASVSVPDSSGPER	Unambiguous	2	1	N-Term(iTRAQ4plex)	HCD	1.0000	0.0000	1	1	8503	Unique	Used	0.083	1.836E5	1.924E4	59	4.94	0.00	0	0	8	2	962.93762	1924.86797	-16.92	29.92	2998	2998	MS2	24/32	Glioma_Nuclear_prep_Gradeall_4plex.raw
14165435	High		nLPLPPPPPPR	Unambiguous	2	1	N-Term(iTRAQ4plex)	HCD	1.0000	0.0000	1	1	8955	Unique	Used	0.144	7.780E5	1.413E5	243	2.99	0.00	0	9	4	2	669.89429	1338.78130	-15.08	33.20	3490	3490	MS2	14/20	Glioma_Nuclear_prep_Gradeall_4plex.raw
14165435	High		iDEPLEGSEDR	Unambiguous	2	1	N-Term(iTRAQ4plex)	HCD	1.0000	0.0000	1	1	8263	Unique	Used	0.214	5.240E5	1.407E5	80	3.78	0.00	0	24	7	2	702.33099	1403.65471	-15.98	28.22	2738	2738	MS2	16/20	Glioma_Nuclear_prep_Gradeall_4plex.raw
14165435	High		nLPLPPPPPPR	Unambiguous	2	1	N-Term(iTRAQ4plex)	HCD	1.0000	0.0000	1	1	8857	Unique	Used	0.223	5.933E5	1.667E5	233	3.05	0.00	0	13	6	2	669.89417	1338.78105	-15.26	32.51	3384	3384	MS2	14/20	Glioma_Nuclear_prep_Gradeall_4plex.raw
14165435		High	tDYNASVSVPDSSGPER	Unambiguous	2	1	N-Term(iTRAQ4plex)	HCD	1.0000	0.0000	1	1	8677	Unique	Used	0.093	2.077E4	2.616E3	91	5.35	0.00	0	8	198	2	962.95660	1924.90593	2.80	30.43	3039	3039	MS2	23/32	Glioma_Nuclear_prep_Gradeall_repeat_4plex.raw
14165435		High	iDEPLEGSEDR	Unambiguous	2	1	N-Term(iTRAQ4plex)	HCD	1.0000	0.0000	1	1	8457	Unique	Used	0.155	5.372E4	1.129E4	109	3.75	0.00	0	25	142	2	702.34180	1403.67632	-0.58	28.18	2807	2807	MS2	18/20	Glioma_Nuclear_prep_Gradeall_repeat_4plex.raw
14165435		High	nLPLPPPPPPR	Unambiguous	2	1	N-Term(iTRAQ4plex)	HCD	1.0000	0.0000	1	1	9451	Unique	Used	0.170	5.110E4	1.177E4	172	3.32	0.00	0	30	160	2	669.90662	1338.80596	3.34	38.68	3859	3859	MS2	14/20	Glioma_Nuclear_prep_Gradeall_repeat_4plex.raw
14165435		High	nLPLPPPPPPR	Unambiguous	2	1	N-Term(iTRAQ4plex)	HCD	1.0000	0.0000	1	1	9625	Unique	Used	0.206	3.070E4	8.553E3	191	3.03	0.00	0	44	300	2	669.90399	1338.80071	-0.58	40.68	4050	4050	MS2	13/20	Glioma_Nuclear_prep_Gradeall_repeat_4plex.raw
14165435		High	nLPLPPPPPPR	Unambiguous	2	1	N-Term(iTRAQ4plex)	HCD	1.0000	0.0000	1	1	9354	Unique	Used	0.236	2.149E4	6.884E3	194	3.35	0.00	0	52	300	2	669.90564	1338.80400	1.88	37.66	3756	3756	MS2	14/20	Glioma_Nuclear_prep_Gradeall_repeat_4plex.raw
14165435		High	gGDLMAYDR	Unambiguous	2	1	N-Term(iTRAQ4plex)	HCD	1.0000	0.0000	1	1	8570	Unique	Used	0.247	3.691E4	1.237E4	99	3.31	0.00	0	26	133	2	571.27716	1141.54704	3.65	29.32	2927	2927	MS2	15/16	Glioma_Nuclear_prep_Gradeall_repeat_4plex.raw
14165435		High	nLPLPPPPPPR	Unambiguous	2	1	N-Term(iTRAQ4plex)	HCD	1.0000	0.0000	1	1	9485	Unique	Used	0.363	2.959E3	1.454E3	142	3.85	0.00	0	46	300	3	446.94080	1338.80783	4.74	39.09	3896	3896	MS2	14/40	Glioma_Nuclear_prep_Gradeall_repeat_4plex.raw
14165435		High	IFQEccPHSTDR	Unambiguous	2	1	N-Term(iTRAQ4plex); C5(Methylthio); C6(Methylthio)	HCD	1.0000	0.0000	1	1	11383	Unique	Used	0.246	1.695E3	5.644E2	18	4.10	0.00	0	59	300	3	557.90173	1671.69065	2.27	62.55	5991	5991	MS2	22/44	Glioma_Nuclear_prep_Gradeall_repeat_4plex.raw
14165435		High	gSDFDcELR	Unambiguous	2	1	N-Term(iTRAQ4plex); C6(Methylthio)	HCD	1.0000	0.0000	1	1	10007	Unique	Used	0.322	2.043E4	8.916E3	13	3.42	0.00	0	34	300	2	616.26532	1231.52336	2.39	45.13	4459	4459	MS2	16/16	Glioma_Nuclear_prep_Gradeall_repeat_4plex.raw
14165435	High		iILDLISESPIk	Unambiguous	2	1	N-Term(iTRAQ4plex); K12(iTRAQ4plex)	HCD	1.0000	0.0000	1	1	12772	Unique	Used	0.124	1.296E4	2.019E3	66	5.23	0.00	0	17	300	2	815.01111	1629.01494	4.42	65.94	7680	7680	MS2	20/22	Glioma_Nuclear_prep_Gradeall_4plex.raw
14165435	High		iILDLISESPIk	Unambiguous	2	1	N-Term(iTRAQ4plex); K12(iTRAQ4plex)	HCD	1.0000	0.0000	1	1	12664	Unique	Used	0.236	1.014E4	3.010E3	70	5.26	0.00	0	29	300	2	815.00928	1629.01128	2.17	64.56	7557	7557	MS2	20/22	Glioma_Nuclear_prep_Gradeall_4plex.raw
14165435	High		iILDLISESPIk	Unambiguous	2	1	N-Term(iTRAQ4plex); K12(iTRAQ4plex)	HCD	1.0000	0.0000	1	1	12672	Unique	Used	0.288	1.521E3	5.518E2	71	4.61	0.00	0	38	176	3	543.67419	1629.00803	0.18	64.65	7566	7566	MS2	18/44	Glioma_Nuclear_prep_Gradeall_4plex.raw
14165435	High		rPAEDMEEEQAFk	Unambiguous	2	1	N-Term(iTRAQ4plex); K13(iTRAQ4plex)	HCD	1.0000	0.0000	1	1	8133	Unique	Used	0.375	2.044E5	9.642E4	47	4.44	0.00	0	35	10	2	934.44543	1867.88359	-14.09	27.30	2597	2597	MS2	16/24	Glioma_Nuclear_prep_Gradeall_4plex.raw
14165435		High	rPAEDMEEEQAFk	Unambiguous	2	1	N-Term(iTRAQ4plex); K13(iTRAQ4plex)	HCD	1.0000	0.0000	1	1	8459	Unique	Used	0.349	9.290E3	4.395E3	83	3.68	0.00	0	49	231	2	934.46021	1867.91313	1.72	28.19	2809	2809	MS2	17/24	Glioma_Nuclear_prep_Gradeall_repeat_4plex.raw
14165435	High		ILIHQSLAGGIIGVk	Unambiguous	2	1	N-Term(iTRAQ4plex); K15(iTRAQ4plex)	HCD	1.0000	0.0000	1	1	10294	Unique	Used	0.352	2.049E5	9.077E4	38	5.11	0.00	0	51	16	2	904.06311	1807.11894	-12.11	43.04	4950	4950	MS2	22/28	Glioma_Nuclear_prep_Gradeall_4plex.raw
14165435	High		ILIHQSLAGGIIGVk	Unambiguous	2	1	N-Term(iTRAQ4plex); K15(iTRAQ4plex)	HCD	1.0000	0.0000	1	1	10282	Unique	Used	0.364	3.115E4	1.426E4	38	4.77	0.00	0	43	7	3	603.04309	1807.11472	-14.45	42.95	4937	4937	MS2	25/56	Glioma_Nuclear_prep_Gradeall_4plex.raw
14165435	High		iLSISADIETIGEILk	Unambiguous	2	1	N-Term(iTRAQ4plex); K16(iTRAQ4plex)	HCD	1.0000	0.0000	1	1	13145	Unique	Used	0.214	3.072E2	8.286E1	55	3.45	0.00	0	35	300	3	668.40332	2003.19541	3.74	78.31	8559	8559	MS2	20/60	Glioma_Nuclear_prep_Gradeall_4plex.raw
14165435		High	gSYGDLGGPIITTQVTIPk	Unambiguous	2	1	N-Term(iTRAQ4plex); K19(iTRAQ4plex)	HCD	1.0000	0.0000	1	1	11600	Unique	Used	0.098	2.141E3	2.849E2	91	6.58	0.00	0	4	300	2	1103.12415	2205.24101	1.83	65.57	6249	6249	MS2	29/36	Glioma_Nuclear_prep_Gradeall_repeat_4plex.raw
14165435	High		vVLIGGkPDR	Unambiguous	2	1	N-Term(iTRAQ4plex); K7(iTRAQ4plex)	HCD	1.0000	0.0000	1	1	7996	Unique	Used	0.111	5.256E5	7.313E4	103	3.28	0.00	0	31	6	2	671.41730	1341.82732	-13.70	26.33	2448	2448	MS2	15/18	Glioma_Nuclear_prep_Gradeall_4plex.raw
14165435		High	eNTQTTIk	Unambiguous	2	1	N-Term(iTRAQ4plex); K8(iTRAQ4plex)	HCD	1.0000	0.0000	1	1	6878	Unique	Used	0.161	7.185E4	1.563E4	307	3.13	0.00	0	22	58	2	611.84772	1222.68816	0.00	13.68	1139	1139	MS2	14/14	Glioma_Nuclear_prep_Gradeall_repeat_4plex.raw
14165435	High		dLAGSIIGk	Unambiguous	2	1	N-Term(iTRAQ4plex); K9(iTRAQ4plex)	HCD	1.0000	0.0000	1	1	9116	Unique	Used	0.270	3.074E5	1.045E5	347	3.47	0.00	0	44	12	2	581.34711	1161.68694	-18.29	34.41	3667	3667	MS2	15/16	Glioma_Nuclear_prep_Gradeall_4plex.raw
14165435		High	dLAGSIIGk	Unambiguous	2	1	N-Term(iTRAQ4plex); K9(iTRAQ4plex)	HCD	1.0000	0.0000	1	1	9578	Unique	Used	0.227	3.618E4	1.111E4	299	3.51	0.00	0	49	177	2	581.35773	1161.70818	-0.01	40.17	4000	4000	MS2	15/16	Glioma_Nuclear_prep_Gradeall_repeat_4plex.raw
14165435		High	rPAEDmEEEQAFk	Unambiguous	2	1	N-Term(iTRAQ4plex); M6(Oxidation); K13(iTRAQ4plex)	HCD	1.0000	0.0000	1	1	7766	Unique	Used	0.214	2.370E3	6.861E2	83	3.16	0.00	0	8	300	2	942.45447	1883.90166	-1.68	21.56	2079	2079	MS2	15/24	Glioma_Nuclear_prep_Gradeall_repeat_4plex.raw
14165435		High	rPAEDmEEEQAFk	Unambiguous	2	1	N-Term(iTRAQ4plex); M6(Oxidation); K13(iTRAQ4plex)	HCD	1.0000	0.0000	1	1	7736	Unique	Used	0.241	3.938E3	1.288E3	104	5.84	0.00	0	16	77	3	628.64093	1883.90824	1.81	21.29	2048	2048	MS2	20/48	Glioma_Nuclear_prep_Gradeall_repeat_4plex.raw
14670392	NP_115784.1	BAZ1B	9031	tyrosine-protein kinase BAZ1B [Homo sapiens]	6.88	1	8	11	1.819	7;4	19.2	35.09	1483	170.8	8.48																			
14670392	High		ISEIPLTLHSVSELVR	Unambiguous	1	1	N-Term(iTRAQ4plex)	HCD	1.0000	0.0000	1	1	12119	Unique	Used	1.740	3.553E3	7.777E3	103	5.24	0.00	0	41	59	3	646.37878	1937.12180	1.52	58.20	6943	6943	MS2	25/60	Glioma_Nuclear_prep_Gradeall_4plex.raw
14670392	High		iISNVPADSLIR	Unambiguous	1	1	N-Term(iTRAQ4plex)	HCD	1.0000	0.0000	1	1	10067	Unique	Used	2.296	9.863E4	2.848E5	293	3.25	0.00	0	36	10	2	721.41736	1441.82744	-15.34	41.34	4702	4702	MS2	16/22	Glioma_Nuclear_prep_Gradeall_4plex.raw
14670392		High	yQDIIHSIHLAR	Unambiguous	1	1	N-Term(iTRAQ4plex)	HCD	1.0000	0.0000	1	1	10785	Unique	Used	1.353	1.426E2	2.613E2	215	4.67	0.00	0	55	300	4	403.22842	1609.89187	-0.78	54.48	5296	5296	MS2	29/66	Glioma_Nuclear_prep_Gradeall_repeat_4plex.raw
14670392		High	iISNVPADSLIR	Unambiguous	1	1	N-Term(iTRAQ4plex)	HCD	1.0000	0.0000	1	1	10846	Unique	Used	1.884	6.660E3	1.699E4	232	4.17	0.00	0	33	300	2	721.42938	1441.85149	1.34	55.26	5364	5364	MS2	15/22	Glioma_Nuclear_prep_Gradeall_repeat_4plex.raw
14670392	High		IQILTALcHR	Unambiguous	1	1	N-Term(iTRAQ4plex); C8(Methylthio)	HCD	1.0000	0.0000	1	1	11888	Unique	Used	1.336	4.131E3	6.941E3	172	4.60	0.00	0	20	74	3	453.25656	1357.75513	-1.01	55.94	6689	6689	MS2	20/36	Glioma_Nuclear_prep_Gradeall_4plex.raw
14670392	High		fSDFLLDPYk	Unambiguous	1	1	N-Term(iTRAQ4plex); K10(iTRAQ4plex)	HCD	1.0000	0.0000	1	1	11608	Unique	Used	1.656	9.932E3	2.069E4	246	3.51	0.00	0	44	101	2	766.91705	1532.82683	1.89	53.47	6385	6385	MS2	17/18	Glioma_Nuclear_prep_Gradeall_4plex.raw
14670392		High	fSDFLLDPYk	Unambiguous	1	1	N-Term(iTRAQ4plex); K10(iTRAQ4plex)	HCD	1.0000	0.0000	1	1	12221	Unique	Used	1.890	2.156E3	5.519E3	219	3.48	0.00	0	22	300	2	766.91644	1532.82561	1.09	76.64	7140	7140	MS2	17/18	Glioma_Nuclear_prep_Gradeall_repeat_4plex.raw
14670392	High		aPPVDDAEVDELVLQTk	Unambiguous	1	1	N-Term(iTRAQ4plex); K17(iTRAQ4plex)	HCD	1.0000	0.0000	1	1	10916	Unique	Used	1.726	7.833E3	1.700E4	200	6.11	0.00	0	9	8	3	709.70966	2127.11441	-13.17	47.81	5630	5630	MS2	30/64	Glioma_Nuclear_prep_Gradeall_4plex.raw
14670392	High		fEPQVDTEAEDMISAVk	Unambiguous	1	1	N-Term(iTRAQ4plex); K17(iTRAQ4plex)	HCD	1.0000	0.0000	1	1	11589	Unique	Used	2.034	1.164E4	2.978E4	146	6.26	0.00	0	36	109	2	1099.04504	2197.08281	-4.99	53.32	6364	6364	MS2	24/32	Glioma_Nuclear_prep_Gradeall_4plex.raw
14670392	High		aPPVDDAEVDELVLQTk	Unambiguous	1	1	N-Term(iTRAQ4plex); K17(iTRAQ4plex)	HCD	1.0000	0.0000	1	1	10937	Unique	Used	2.507	2.553E4	8.050E4	147	6.12	0.00	0	10	30	2	1064.05981	2127.11235	-14.14	47.96	5652	5652	MS2	27/32	Glioma_Nuclear_prep_Gradeall_4plex.raw
14670392		High	sLSGSPLk	Unambiguous	1	1	N-Term(iTRAQ4plex); K8(iTRAQ4plex)	HCD	1.0000	0.0000	1	1	7964	Unique	Used	1.819	2.005E4	4.938E4	426	3.02	0.00	0	54	277	2	538.82867	1076.65007	-4.96	23.45	2288	2288	MS2	13/14	Glioma_Nuclear_prep_Gradeall_repeat_4plex.raw
15451775	NP_150249.1	PML	5371	protein PML isoform 7 [Homo sapiens]	2.84	9	1	2	2.423	1;1	37.0	4.02	423	47.5	6.02																			
15451775	High		aPASEEEFQFLR	Unambiguous	9	1	N-Term(iTRAQ4plex)	HCD	1.0000	0.0000	1	1	10286	Unique	Used	1.789	5.206E4	1.172E5	142	3.32	0.00	0	37	26	2	784.38538	1567.76348	-15.23	42.98	4941	4941	MS2	12/22	Glioma_Nuclear_prep_Gradeall_4plex.raw
15451775		High	aPASEEEFQFLR	Unambiguous	9	1	N-Term(iTRAQ4plex)	HCD	1.0000	0.0000	1	1	11136	Unique	Used	3.058	1.639E3	6.787E3	157	4.02	0.00	0	26	300	2	784.39874	1567.79021	1.82	58.96	5688	5688	MS2	19/22	Glioma_Nuclear_prep_Gradeall_repeat_4plex.raw
19923653	NP_150091.2	PSIP1	11168	PC4 and SFRS1-interacting protein isoform 2 [Homo sapiens]	34.34	2	11	29	0.594	14;15	15.3	96.58	530	60.1	9.13																			
19923653	High		qPcPSESDIITEEDk	Unambiguous	2	1	N-Term(iTRAQ4plex); C3(Methylthio); K15(iTRAQ4plex)	HCD	1.0000	0.0000	1	1	9531	Unique	Used	0.508	1.424E4	9.105E3	45	3.73	0.00	0	50	18	3	675.64038	2024.90659	-16.28	37.46	4120	4120	MS2	18/56	Glioma_Nuclear_prep_Gradeall_4plex.raw
19923653	High		qPcPSESDIITEEDk	Unambiguous	2	1	N-Term(iTRAQ4plex); C3(Methylthio); K15(iTRAQ4plex)	HCD	1.0000	0.0000	1	1	9522	Unique	Used	0.565	3.341E5	2.373E5	41	5.71	0.00	0	3	8	2	1012.95758	2024.90788	-15.64	37.41	4111	4111	MS2	21/28	Glioma_Nuclear_prep_Gradeall_4plex.raw
19923653		High	qPcPSESDIITEEDk	Unambiguous	2	1	N-Term(iTRAQ4plex); C3(Methylthio); K15(iTRAQ4plex)	HCD	1.0000	0.0000	1	1	10079	Unique	Used	0.494	2.752E3	1.842E3	84	5.16	0.00	0	13	83	3	675.65344	2024.94577	3.07	46.06	4540	4540	MS2	22/56	Glioma_Nuclear_prep_Gradeall_repeat_4plex.raw
19923653		High	qPcPSESDIITEEDk	Unambiguous	2	1	N-Term(iTRAQ4plex); C3(Methylthio); K15(iTRAQ4plex)	HCD	1.0000	0.0000	1	1	10067	Unique	Used	0.600	6.944E3	5.643E3	73	5.42	0.00	0	11	300	2	1012.97614	2024.94499	2.69	45.89	4526	4526	MS2	21/28	Glioma_Nuclear_prep_Gradeall_repeat_4plex.raw
19923653	High		kGFNEGLWEIDNNPk	Unambiguous	2	1	N-Term(iTRAQ4plex); K1(iTRAQ4plex); K15(iTRAQ4plex)	HCD	1.0000	0.0000	1	1	9600	Unique	Used	0.691	3.151E4	2.738E4	185	5.98	0.00	1	40	10	3	731.71692	2193.13620	-13.81	37.96	4195	4195	MS2	23/56	Glioma_Nuclear_prep_Gradeall_4plex.raw

19923653	High		vDEVPDGAVkPPTNk	Unambiguous	2	1	N-Term(iTRAQ4plex); K10(iTRAQ4plex); K15(iTRAQ4plex)	HCD	1.0000	0.0000	1	1	8215	Unique	Used	0.496	1.335E5	8.330E4	138	3.77	0.00	0	11	2	3	666.70203	1998.09153	-15.87	27.88	2686	2686	MS2	22/56	Glioma_Nuclear_prep_Gradeall_4plex.raw
19923653	High		vDEVPDGAVkPPTNk	Unambiguous	2	1	N-Term(iTRAQ4plex); K10(iTRAQ4plex); K15(iTRAQ4plex)	HCD	1.0000	0.0000	1	1	8171	Unique	Used	0.630	2.172E5	1.722E5	127	4.72	0.00	0	13	7	2	999.54926	1998.09123	-16.02	27.57	2638	2638	MS2	20/28	Glioma_Nuclear_prep_Gradeall_4plex.raw
19923653		High	vDEVPDGAVkPPTNk	Unambiguous	2	1	N-Term(iTRAQ4plex); K10(iTRAQ4plex); K15(iTRAQ4plex)	HCD	1.0000	0.0000	1	1	8485	Unique	Used	0.462	3.014E4	1.886E4	103	3.71	0.00	0	9	16	3	666.71460	1998.12925	3.01	28.47	2837	2837	MS2	22/56	Glioma_Nuclear_prep_Gradeall_repeat_4plex.raw
19923653		High	vDEVPDGAVkPPTNk	Unambiguous	2	1	N-Term(iTRAQ4plex); K10(iTRAQ4plex); K15(iTRAQ4plex)	HCD	1.0000	0.0000	1	1	8500	Unique	Used	0.577	1.295E3	1.013E3	127	3.58	0.00	0	51	132	4	500.28598	1998.12209	-0.58	28.62	2853	2853	MS2	17/84	Glioma_Nuclear_prep_Gradeall_repeat_4plex.raw
19923653		High	vDEVPDGAVkPPTNk	Unambiguous	2	1	N-Term(iTRAQ4plex); K10(iTRAQ4plex); K15(iTRAQ4plex)	HCD	1.0000	0.0000	1	1	8522	Unique	Used	0.720	9.947E2	9.699E2	85	2.98	0.00	0	0	300	2	999.56824	1998.12920	2.98	28.85	2876	2876	MS2	15/28	Glioma_Nuclear_prep_Gradeall_repeat_4plex.raw
19923653	High		qSNASSDVEVEEk	Unambiguous	2	1	N-Term(iTRAQ4plex); K13(iTRAQ4plex)	HCD	1.0000	0.0000	1	1	7450	Unique	Used	0.476	3.407E5	2.041E5	143	5.84	0.00	0	6	10	2	855.42322	1709.83916	-2.39	22.46	1857	1857	MS2	24/24	Glioma_Nuclear_prep_Gradeall_4plex.raw
19923653		High	qSNASSDVEVEEk	Unambiguous	2	1	N-Term(iTRAQ4plex); K13(iTRAQ4plex)	HCD	1.0000	0.0000	1	1	7421	Unique	Used	0.518	2.451E4	1.721E4	105	5.63	0.00	0	11	174	2	855.41418	1709.82109	-12.96	18.40	1712	1712	MS2	24/24	Glioma_Nuclear_prep_Gradeall_repeat_4plex.raw
19923653	High		gFNEGLWEIDNNPk	Unambiguous	2	1	N-Term(iTRAQ4plex); K14(iTRAQ4plex)	HCD	1.0000	0.0000	1	1	10397	Unique	Used	0.625	6.251E4	4.911E4	130	6.17	0.00	0	0	33	2	960.97516	1920.94304	-13.76	43.82	5062	5062	MS2	24/26	Glioma_Nuclear_prep_Gradeall_4plex.raw
19923653		High	gFNEGLWEIDNNPk	Unambiguous	2	1	N-Term(iTRAQ4plex); K14(iTRAQ4plex)	HCD	1.0000	0.0000	1	1	11247	Unique	Used	0.716	2.317E3	2.246E3	162	5.84	0.00	0	13	300	2	960.98914	1920.97099	0.80	60.40	5815	5815	MS2	24/26	Glioma_Nuclear_prep_Gradeall_repeat_4plex.raw
19923653	High		lPIFFFGTHETAFLGPk	Unambiguous	2	1	N-Term(iTRAQ4plex); K17(iTRAQ4plex)	HCD	1.0000	0.0000	1	1	12744	Unique	Used	0.505	1.987E3	1.262E3	109	5.89	0.00	0	45	300	3	737.41449	2210.22892	1.64	65.55	7646	7646	MS2	27/64	Glioma_Nuclear_prep_Gradeall_4plex.raw
19923653	High		lPIFFFGTHETAFLGPk	Unambiguous	2	1	N-Term(iTRAQ4plex); K17(iTRAQ4plex)	HCD	1.0000	0.0000	1	1	12794	Unique	Used	0.594	2.720E3	2.033E3	101	6.70	0.00	0	17	300	3	737.41638	2210.23459	4.21	66.28	7707	7707	MS2	27/64	Glioma_Nuclear_prep_Gradeall_4plex.raw
19923653	High		dFkPGDLIFAk	Unambiguous	2	1	N-Term(iTRAQ4plex); K3(iTRAQ4plex); K11(iTRAQ4plex)	HCD	1.0000	0.0000	1	1	10051	Unique	Used	0.475	1.509E5	9.025E4	159	4.07	0.00	0	25	3	3	561.65826	1682.96024	-14.24	41.23	4685	4685	MS2	23/40	Glioma_Nuclear_prep_Gradeall_4plex.raw
19923653	High		dFkPGDLIFAk	Unambiguous	2	1	N-Term(iTRAQ4plex); K3(iTRAQ4plex); K11(iTRAQ4plex)	HCD	1.0000	0.0000	1	1	10055	Unique	Used	0.650	2.617E5	2.138E5	190	3.72	0.00	0	37	13	2	841.98444	1682.96160	-13.44	41.25	4689	4689	MS2	17/20	Glioma_Nuclear_prep_Gradeall_4plex.raw
19923653		High	dFkPGDLIFAk	Unambiguous	2	1	N-Term(iTRAQ4plex); K3(iTRAQ4plex); K11(iTRAQ4plex)	HCD	1.0000	0.0000	1	1	11014	Unique	Used	0.629	5.977E3	5.089E3	143	3.67	0.00	0	58	300	2	841.99731	1682.98735	1.87	57.33	5547	5547	MS2	18/20	Glioma_Nuclear_prep_Gradeall_repeat_4plex.raw
19923653		High	dFkPGDLIFAk	Unambiguous	2	1	N-Term(iTRAQ4plex); K3(iTRAQ4plex); K11(iTRAQ4plex)	HCD	1.0000	0.0000	1	1	11098	Unique	Used	0.664	2.432E3	2.187E3	117	3.20	0.00	0	84	300	2	842.00507	1683.00286	11.08	58.42	5642	5642	MS2	17/20	Glioma_Nuclear_prep_Gradeall_repeat_4plex.raw
19923653		High	dFkPGDLIFAk	Unambiguous	2	1	N-Term(iTRAQ4plex); K3(iTRAQ4plex); K11(iTRAQ4plex)	HCD	1.0000	0.0000	1	1	11010	Unique	Used	0.717	3.784E3	3.673E3	120	4.27	0.00	0	27	266	3	561.66833	1682.99045	3.71	57.28	5543	5543	MS2	23/40	Glioma_Nuclear_prep_Gradeall_repeat_4plex.raw
19923653	High		aSNEDVTk	Unambiguous	2	1	N-Term(iTRAQ4plex); K8(iTRAQ4plex)	HCD	1.0000	0.0000	1	1	6991	Unique	Used	0.681	6.414E3	5.492E3	226	3.18	0.00	0	82	300	2	576.31494	1151.62261	6.89	18.42	1354	1354	MS2	14/14	Glioma_Nuclear_prep_Gradeall_4plex.raw
19923653		High	aVDITTPk	Unambiguous	2	1	N-Term(iTRAQ4plex); K8(iTRAQ4plex)	HCD	1.0000	0.0000	1	1	8022	Unique	Used	0.718	5.094E4	4.956E4	268	3.27	0.00	0	38	120	2	566.84460	1132.68193	0.27	23.99	2349	2349	MS2	13/14	Glioma_Nuclear_prep_Gradeall_repeat_4plex.raw
19923653	High		dIFPYSENk	Unambiguous	2	1	N-Term(iTRAQ4plex); K9(iTRAQ4plex)	HCD	1.0000	0.0000	1	1	9091	Unique	Used	0.508	1.061E6	6.783E5	127	3.13	0.00	0	3	2	2	700.85840	1400.70952	-14.64	34.23	3640	3640	MS2	14/16	Glioma_Nuclear_prep_Gradeall_4plex.raw
19923653	High		dIFPYSENk	Unambiguous	2	1	N-Term(iTRAQ4plex); K9(iTRAQ4plex)	HCD	1.0000	0.0000	1	1	9008	Unique	Used	0.683	1.893E5	1.626E5	133	3.32	0.00	0	52	18	2	700.85547	1400.70366	-18.82	33.57	3547	3547	MS2	13/16	Glioma_Nuclear_prep_Gradeall_4plex.raw
19923653		High	fSSQQAATk	Unambiguous	2	1	N-Term(iTRAQ4plex); K9(iTRAQ4plex)	HCD	1.0000	0.0000	1	1	7156	Unique	Used	0.463	2.347E5	1.472E5	166	3.89	0.00	0	8	20	2	628.34619	1255.68511	-2.70	16.21	1434	1434	MS2	16/16	Glioma_Nuclear_prep_Gradeall_repeat_4plex.raw
19923653		High	dIFPYSENk	Unambiguous	2	1	N-Term(iTRAQ4plex); K9(iTRAQ4plex)	HCD	1.0000	0.0000	1	1	9619	Unique	Used	0.491	2.990E4	1.988E4	197	2.90	0.00	0	17	138	2	700.86725	1400.72722	-2.00	40.62	4044	4044	MS2	14/16	Glioma_Nuclear_prep_Gradeall_repeat_4plex.raw
19923653		High	dIFPYSENk	Unambiguous	2	1	N-Term(iTRAQ4plex); K9(iTRAQ4plex)	HCD	1.0000	0.0000	1	1	9529	Unique	Used	0.572	3.274E4	2.538E4	164	3.13	0.00	0	37	138	2	700.86395	1400.72063	-6.71	39.60	3946	3946	MS2	13/16	Glioma_Nuclear_prep_Gradeall_repeat_4plex.raw
19923653		High	fSSQQAATk	Unambiguous	2	1	N-Term(iTRAQ4plex); K9(iTRAQ4plex)	HCD	1.0000	0.0000	1	1	7126	Unique	Used	0.692	4.529E4	4.246E4	201	3.83	0.00	0	64	214	2	628.33734	1255.66741	-16.80	15.91	1402	1402	MS2	16/16	Glioma_Nuclear_prep_Gradeall_repeat_4plex.raw
20336746	NP_613075.1	H2AFY	9555	core histone macro-H2A.1 isoform 1 [Homo sapiens]	33.33	4	7	11	3.786	7;4	31.9	42.73	369	39.2	9.83																			
20336746	High		sIAFPSIGSGR	Unambiguous	3	1	N-Term(iTRAQ4plex)	HCD	1.0000	0.0000	1	1	9598	Unique	Used	1.994	1.307E5	3.277E5	317	2.91	0.00	0	31	14	2	618.33710	1235.66692	-15.85	37.95	4193	4193	MS2	14/20	Glioma_Nuclear_prep_Gradeall_4plex.raw
20336746	High		eFVEAVLELR	Unambiguous	3	1	N-Term(iTRAQ4plex)	HCD	1.0000	0.0000	1	1	12082	Unique	Used	3.786	1.712E4	8.151E4	338	3.37	0.00	0	54	76	2	674.88245	1348.75762	-1.28	57.82	6902	6902	MS2	17/18	Glioma_Nuclear_prep_Gradeall_4plex.raw
20336746	High		eFVEAVLELR	Unambiguous	3	1	N-Term(iTRAQ4plex)	HCD	1.0000	0.0000	1	1	12013	Unique	Used	4.495	8.800E3	4.975E4	315	3.81	0.00	0	28	97	2	674.88300	1348.75871	-0.47	57.12	6827	6827	MS2	17/18	Glioma_Nuclear_prep_Gradeall_4plex.raw
20336746		High	sIAFPSIGSGR	Unambiguous	3	1	N-Term(iTRAQ4plex)	HCD	1.0000	0.0000	1	1	10255	Unique	Used	4.748	4.176E3	2.686E4	320	3.45	0.00	0	5	300	2	618.34668	1235.68608	-0.34	48.10	4726	4726	MS2	15/20	Glioma_Nuclear_prep_Gradeall_repeat_4plex.raw
20336746	High		cEELLEk	Unambiguous	3	1	N-Term(iTRAQ4plex); C1(Methylthio); K7(iTRAQ4plex)	HCD	1.0000	0.0000	1	1	9601	Unique	Used	3.579	7.850E4	3.533E5	130	2.93	0.00	0	19	15	2	599.29889	1197.59050	-16.10	37.97	4196	4196	MS2	12/12	Glioma_Nuclear_prep_Gradeall_4plex.raw
20336746		High	cEELLEk	Unambiguous	3	1	N-Term(iTRAQ4plex); C1(Methylthio); K7(iTRAQ4plex)	HCD	1.0000	0.0000	1	1	10279	Unique	Used	6.326	6.081E3	5.210E4	179	2.91	0.00	0	14	300	2	599.30841	1197.60954	-0.20	48.39	4752	4752	MS2	12/12	Glioma_Nuclear_prep_Gradeall_repeat_4plex.raw
20336746	High		ncLALADDk	Unambiguous	3	1	N-Term(iTRAQ4plex); C2(Methylthio); K9(iTRAQ4plex)	HCD	1.0000	0.0000	1	1	9497	Unique	Used	3.436	1.032E5	4.457E5	133	3.07	0.00	0	30	12	2	648.81952	1296.63176	-16.42	37.23	4084	4084	MS2	16/16	Glioma_Nuclear_prep_Gradeall_4plex.raw
20336746	High		gVTIASGGVLPNIHPELLAk	Unambiguous	3	1	N-Term(iTRAQ4plex); K20(iTRAQ4plex)	HCD	1.0000	0.0000	1	1	10896	Unique	Used	2.931	5.466E3	2.015E4	69	5.44	0.00	0	19	16	3	758.77484	2274.30997	-14.29	47.66	5608	5608	MS2	27/76	Glioma_Nuclear_prep_Gradeall_4plex.raw
20336746	High		nGPLEVAGAAVSAGHGLPAk	Unambiguous	3	1	N-Term(iTRAQ4plex); K20(iTRAQ4plex)	HCD	1.0000	0.0000	1	1	9853	Unique	Used	4.383	1.434E4	7.903E4	109	5.25	0.00	0	17	7	3	702.05225	2104.14219	-15.79	39.78	4470	4470	MS2	42/76	Glioma_Nuclear_prep_Gradeall_4plex.raw
20336746		High	nGPLEVAGAAVSAGHGLPAk	Unambiguous	3	1	N-Term(iTRAQ4plex); K20(iTRAQ4plex)	HCD	1.0000	0.0000	1	1	10680	Unique	Used	2.316	5.272E2	1.654E3	108	5.81	0.00	0	38	300	3	702.06409	2104.17771	1.09	53.19	5182	5182	MS2	33/76	Glioma_Nuclear_prep_Gradeall_repeat_4plex.raw
20336746		High	sLFLGQk	Unambiguous	3	1	N-Term(iTRAQ4plex); K7(iTRAQ4plex)	HCD	1.0000	0.0000	1	1	9191	Unique	Used	4.786	1.818E4	1.179E5	168	3.09	0.00	0	17	185	2	540.83698	1080.66667	1.01	35.87	3585	3585	MS2	12/12	Glioma_Nuclear_prep_Gradeall_repeat_4plex.raw
21361809	NP_062556.2	RBMXL1	494115	RNA binding motif protein, X-linked-like-1 [Homo sapiens]	22.05	2	3	6	0.346	4;2	30.5	64.29	390	42.1	9.89																			
21361809	High		vEQATkPSFER	Unambiguous	1	1	N-Term(iTRAQ4plex); K6(iTRAQ4plex)	HCD	1.0000	0.0000	1	1	7721	Unique	Used	0.316	1.161E5	4.622E4	255	3.76	0.00	0	5	16	2	790.43921	1579.87114	1.81	24.38	2150	2150	MS2	16/20	Glioma_Nuclear_prep_Gradeall_4plex.raw
21361809	High		iVEVLLk	Unambiguous	1	1	N-Term(iTRAQ4plex); K8(iTRAQ4plex)	HCD	1.0000	0.0000	1	1	11004	Unique	Used	0.282	4.983E5	1.769E5	22	3.51	0.00	0	27	8	2	607.92010	1214.83293	0.23	48.48	5725	5725	MS2	14/14	Glioma_Nuclear_prep_Gradeall_4plex.raw
21361809	High		aLETVFGk	Unambiguous	1	1	N-Term(iTRAQ4plex); K8(iTRAQ4plex)	HCD	1.0000	0.0000	1	1	9444	Unique	Used	0.375	3.841E5	1.810E5	336	3.13	0.00	0	48	6	2	576.83807	1152.66887	-15.48	36.84	4026	4026	MS2	13/14	Glioma_Nuclear_prep_Gradeall_4plex.raw
21361809	High		iVEVLLk	Unambiguous	1	1	N-Term(iTRAQ4plex); K8(iTRAQ4plex)	HCD	1.0000	0.0000	1	1	10978	Unique	Used	0.514	3.305E5	2.136E5	43	3.34	0.00	0	54	13	2	607.91217	1214.81706	-12.84	48.29	5697	5697	MS2	13/14	Glioma_Nuclear_prep_Gradeall_4plex.raw
21361809		High	iVEVLLk	Unambiguous	1	1	N-Term(iTRAQ4plex); K8(iTRAQ4plex)	HCD	1.0000	0.0000	1	1	11880	Unique	Used	0.228	6.957E3	2.145E3	23	3.34	0.00	0	51	300	2	607.92145	1214.83562	2.44	69.89	6609	6609	MS2	14/14	Glioma_Nuclear_prep_Gradeall_repeat_4plex.raw
21361809		High	aLETVFGk	Unambiguous	1	1	N-Term(iTRAQ4plex); K8(iTRAQ4plex)	HCD	1.0000	0.0000	1	1	10036	Unique	Used	0.473	1.388E4	8.887E3	290	3.27	0.00	0	49	300	2	576.84735	1152.68743	0.61	45.48	4491	4491	MS2	13/14	Glioma_Nuclear_prep_Gradeall_repeat_4plex.raw
21536286	NP_001814.2	CKB	1152	creatine kinase B-type [Homo sapiens]	9.71	1	3	3	0.665	1;2	23.6	9.63	381	42.6	5.59																			
21536286	High		lAVEALSSLDGDLAGR	Unambiguous	1	1	N-Term(iTRAQ4plex)	HCD	1.0000	0.0000	1	1	12047	Unique	Used	0.683	2.841E4	2.441E4	272	5.59	0.00	0	29	85	2	865.97430	1730.94133	0.43	57.46	6864	6864	MS2	23/30	Glioma_Nuclear_prep_Gradeall_4plex.raw
21536286		High	vLTPELYAELR	Unambiguous	1	1	N-Term(iTRAQ4plex)	HCD	1.0000	0.0000	1	1	11665	Unique	Used	0.432	5.587E3	3.269E3	296	3.86	0.00	0	17	300	2	724.41827	1447.82927	1.05	66.51	6329	6329	MS2	16/20	Glioma_Nuclear_prep_Gradeall_repeat_4plex.raw
21536286		High	dLFDPIIEDR	Unambiguous	1	1	N-Term(iTRAQ4plex)	HCD	1.0000	0.0000	1	1	11553	Unique	Used	0.665	2.631E3	2.371E3	231	3.45	0.00	0	48	300	2	688.86298	1376.71868	0.58	64.87	6191	6191	MS2	17/18	Glioma_Nuclear_prep_Gradeall_repeat_4plex.raw
24431933	NP_722550.1	RTN4	57142	reticulon-4 isoform B [Homo sapiens]	3.49	3	1	2	2.053	1;1	1.5	4.28	373	40.3	4.78																			
24431933	High		gPLPAAPPVAPER	Unambiguous	3	1	N-Term(iTRAQ4plex)	HCD	1.0000	0.0000	1	1	8708	Unique	Used	2.074	9.415E4	2.456E5	296	3.84	0.00	0	8	13	2	708.39825	1415.78923	-16.64	31.43	3222	3222	MS2	15/24	Glioma_Nuclear_prep_Gradeall_4plex.raw
24431933		High	gPLPAAPPVAPER	Unambiguous	3	1	N-Term(iTRAQ4plex)	HCD	1.0000	0.0000	1	1	9132	Unique	Used	2.031	5.291E3	1.456E4	240	4.28	0.00	0	13	250	2	708.40796	1415.80864	-2.93	35.23	3523	3523	MS2	18/24	Glioma_Nuclear_prep_Gradeall_repeat_4plex.raw
24762236	NP_116253.2	PRPF38A	84950	pre-mRNA-splicing factor 38A [Homo sapiens]	6.41	1	2	3	1.813	1;2	17.6	7.02	312	37.5	9.96																			
24762236	High		yVLEEAEQLEPR	Unambiguous	1	1	N-Term(iTRAQ4plex)	HCD	1.0000	0.0000	1	1	10703	Unique	Used	1.495	7.844E4	1.475E5	179	4.50	0.00	0	54	34	2	810.41223	1619.81719	-13.94	46.17	5398	5398	MS2	19/22	Glioma_Nuclear_prep_Gradeall_4plex.raw
24762236		High	yVLEEAEQLEPR	Unambiguous	1	1	N-Term(iTRAQ4plex)	HCD	1.0000	0.0000	1	1	11492	Unique	Used	1.813	1.753E3	4.305E3	254	4.15	0.00	0	33	300	2	810.42603	1619.84477	3.09	64.03	6119	6119	MS2	20/22	Glioma_Nuclear_prep_Gradeall_repeat_4plex.raw
24762236		High	vcDIILPR	Unambiguous	1	1	N-Term(iTRAQ4plex); C2(Methylthio)	HCD	1.0000	0.0000	1	1	11881	Unique	Used	2.135	1.961E3	5.669E3	285	2.87	0.00	0	36	300	2	559.81348	1118.61968	1.24	69.90	6610	6610	MS2	13/14	Glioma_Nuclear_prep_Gradeall_repeat_4plex.raw
25121993	NP_733839.1	MSI2	124540	RNA-binding protein Musashi homolog 2 isoform b [Homo sapiens]	5.98	2	1	2	0.318	1;1	23.4	5.23	251	28.4	8.50																			
25121993	High		gFGFVTFADPASVDk	Unambiguous	2	1	N-Term(iTRAQ4plex); K15(iTRAQ4plex)	HCD	1.0000	0.0000	1	1	11354	Unique	Used	0.266	4.599E4	1.538E4	187	5.23	0.00	0	31	36	2	923.48523	1845.96318	0.32	51.36	6108	6108	MS2	21/28	Glioma_Nuclear_prep_Gradeall_4plex.raw
25121993		High	gFGFVTFADPASVDk	Unambiguous	2	1	N-Term(iTRAQ4plex); K15(iTRAQ4plex)	HCD	1.0000	0.0000	1	1	12060	Unique	Used	0.371	1.024E3	5.144E2	185	4.82	0.00	0	13	300	2	923.48749	1845.96770	2.76	73.14	6868	6868	MS2	25/28	Glioma_Nuclear_prep_Gradeall_repeat_4plex.raw
25952118	NP_741960.1	CAMK2A	815	calcium/calmodulin-dependent protein kinase type II subunit alpha isoform 2 [Homo sapiens]	2.51	2	1	2	0.294	1;1	40.0	3.85	478	54.1	7.08																			
25952118	High		iTQYLDAGGIPR	Unambiguous	2	1	N-Term(iTRAQ4plex)	HCD	1.0000	0.0000	1	1	10237	Unique	Used	0.380	1.314E5	6.275E4	304	3.66	0.00	0	41	23	2	724.39508	1447.78288	-13.61	42.62	4888	4888	MS2	18/22	Glioma_Nuclear_prep_Gradeall_4plex.raw
25952118		High	iTQYLDAGGIPR	Unambiguous	2	1	N-Term(iTRAQ4plex)	HCD	1.0000	0.0000	1	1	11026	Unique	Used	0.209	9.949E3	2.817E3	321	3.85	0.00	0	42	300	2	724.40668	1447.80608	2.41	57.49	5561	5561	MS2	16/22	Glioma_Nuclear_prep_Gradeall_repeat_4plex.raw
29568103	NP_003080.2	SNRNP70	6625	U1 small nuclear ribonucleoprotein 70 kDa isoform 1 [Homo sapiens]	13.73	2	6	9	0.638	5;4	8.4	29.88	437	51.5	9.94																			
29568103	High		gYAFIEYEHER	Unambiguous	2	1	N-Term(iTRAQ4plex)	HCD	1.0000	0.0000	1	1	8886	Unique	Used	0.688	1.154E5	9.988E4	93	3.68	0.00	0	0	15	2	779.36499	1557.72270	-14.60	32.71	3415	3415	MS2	19/20	Glioma_Nuclear_prep_Gradeall_4plex.raw
29568103		High	lGGGLGGTR	Unambiguous	2	1	N-Term(iTRAQ4plex)	HCD	1.0000	0.0000	1	1	7691	Unique	Used	0.597	8.028E4	6.496E4	248	3.13	0.00	0	18	70	2	466.27176	931.53624	-8.54	20.86	2001	2001	MS2	13/16	Glioma_Nuclear_prep_Gradeall_repeat_4plex.raw
29568103		High	gYAFIEYEHER	Unambiguous	2	1	N-Term(iTRAQ4plex)	HCD	1.0000	0.0000	1	1	9528	Unique	Used	0.641	4.169E3	3.618E3	86	4.61	0.00	0	26	91	3	519.91632	1557.73441	-7.09	39.59	3945	3945	MS2	22/40	Glioma_Nuclear_prep_Gradeall_repeat_4plex.raw
29568103	High		rQQEVETELk	Unambiguous	2	1	N-Term(iTRAQ4plex); K10(iTRAQ4plex)	HCD	1.0000	0.0000	1	1	7462	Unique	Used	0.711	1.033E5	9.246E4	258	3.25	0.00	1	10	12	2	774.43152	1547.85576	-4.80	22.55	1870	1870	MS2	16/18	Glioma_Nuclear_prep_Gradeall_4plex.raw
29568103	High		dPIPYLPPLEk	Unambiguous	2	1	N-Term(iTRAQ4plex); K11(iTRAQ4plex)	HCD	1.0000	0.0000	1	1	10834	Unique	Used	0.593	5.415E5	4.038E5	214	3.51	0.00	0	17	5	2	785.45032	1569.89336	-12.57	47.18	5541	5541	MS2	16/20	Glioma_Nuclear_prep_Gradeall_4plex.raw
29568103		High	dPIPYLPPLEk	Unambiguous	2	1	N-Term(iTRAQ4plex); K11(iTRAQ4plex)	HCD	1.0000	0.0000	1	1	11721	Unique	Used	0.587	8.963E3	7.121E3	172	3.54	0.00	0	10	300	2	785.46234	1569.91741	2.74	67.25	6393	6393	MS2	16/20	Glioma_Nuclear_prep_Gradeall_repeat_4plex.raw
29568103	High		eFEVYGPIk	Unambiguous	2	1	N-Term(iTRAQ4plex); K9(iTRAQ4plex)	HCD	1.0000	0.0000	1	1	9581	Unique	Used	0.614	4.351E5	3.358E5	231	3.53	0.00	0	30	7	2	685.37366	1369.74004	-15.02	37.84	4175	4175	MS2	14/16	Glioma_Nuclear_prep_Gradeall_4plex.raw
29568103	High		qQEVETELk	Unambiguous	2	1	N-Term(iTRAQ4plex); K9(iTRAQ4plex)	HCD	1.0000	0.0000	1	1	7800	Unique	Used	0.731	3.472E5	3.193E5	247	4.13	0.00	0	13	11	2	696.38458	1391.76189	-0.13	24.94	2236	2236	MS2	16/16	Glioma_Nuclear_prep_Gradeall_4plex.raw
29568103		High	eFEVYGPIk	Unambiguous	2	1	N-Term(iTRAQ4plex); K9(iTRAQ4plex)	HCD	1.0000	0.0000	1	1	10252	Unique	Used	0.638	1.472E4	1.272E4	252	4.18	0.00	0	19	300	2	685.38513	1369.76299	1.74	48.06	4723	4723	MS2	15/16	Glioma_Nuclear_prep_Gradeall_repeat_4plex.raw
29788768	NP_821080.1	TUBB2B	347733	tubulin beta-2B chain [Homo sapiens]	32.13	3	2	4	0.404	3;1	26.6	101.57	445	49.9	4.89																			
29788768	High		yLTVAAIFR	Unambiguous	2	1	N-Term(iTRAQ4plex)	HCD	1.0000	0.0000	1	1	11606	Unique	Used	0.293	4.564E4	1.685E4	188	3.46	0.00	0	43	96	2	599.36139	1197.71550	3.55	53.46	6383	6383	MS2	15/16	Glioma_Nuclear_prep_Gradeall_4plex.raw
29788768	High		aLTVPELTQQMFDSk	Unambiguous	2	1	N-Term(iTRAQ4plex); K15(iTRAQ4plex)	HCD	1.0000	0.0000	1	1	11827	Unique	Used	0.379	3.841E4	1.830E4	184	5.16	0.00	0	20	95	2	998.53510	1996.06291	-1.75	55.43	6623	6623	MS2	18/28	Glioma_Nuclear_prep_Gradeall_4plex.raw
29788768	High		aLTVPELTQQMFDSk	Unambiguous	2	1	N-Term(iTRAQ4plex); K15(iTRAQ4plex)	HCD	1.0000	0.0000	1	1	11855	Unique	Used	0.430	1.323E4	7.149E3	175	6.06	0.00	0	13	15	3	666.02582	1996.06290	-1.76	55.69	6654	6654	MS2	24/56	Glioma_Nuclear_prep_Gradeall_4plex.raw
29788768		High	aLTVPELTQQMFDSk	Unambiguous	2	1	N-Term(iTRAQ4plex); K15(iTRAQ4plex)	HCD	1.0000	0.0000	1	1	12311	Unique	Used	0.557	2.049E2	1.547E2	207	4.29	0.00	0	32	300	3	666.02863	1996.07132	2.46	79.31	7337	7337	MS2	20/56	Glioma_Nuclear_prep_Gradeall_repeat_4plex.raw
30795231	NP_006308.3	BASP1	10409	brain acid soluble protein 1 [Homo sapiens]	22.91	1	4	5	0.564	3;2	14.7	15.71	227	22.7	4.63																			
30795231	High		eSEPQAAAEPAEAk	Unambiguous	1	1	N-Term(iTRAQ4plex); K14(iTRAQ4plex)	HCD	1.0000	0.0000	1	1	7475	Unique	Used	0.446	7.547E4	4.229E4	146	4.81	0.00	0	0	15	2	858.43524	1715.86321	-3.43	22.64	1884	1884	MS2	22/26	Glioma_Nuclear_prep_Gradeall_4plex.raw
30795231	High		eTPAATEAPSSTPk	Unambiguous	1	1	N-Term(iTRAQ4plex); K14(iTRAQ4plex)	HCD	1.0000	0.0000	1	1	7429	Unique	Used	0.545	6.699E4	4.588E4	181	4.88	0.00	0	6	26	2	837.93958	1674.87187	-4.20	22.31	1834	1834	MS2	21/26	Glioma_Nuclear_prep_Gradeall_4plex.raw
30795231		High	eTPAATEAPSSTPk	Unambiguous	1	1	N-Term(iTRAQ4plex); K14(iTRAQ4plex)	HCD	1.0000	0.0000	1	1	7360	Unique	Used	0.564	4.637E3	3.542E3	157	3.23	0.00	0	0	199	2	837.94452	1674.88176	1.70	17.92	1648	1648	MS2	21/26	Glioma_Nuclear_prep_Gradeall_repeat_4plex.raw
30795231		High	aPEQEQAAPGPAAGGEAPk	Unambiguous	1	1	N-Term(iTRAQ4plex); K19(iTRAQ4plex)	HCD	1.0000	0.0000	1	1	7661	Unique	Used	0.665	7.381E2	6.648E2	170	5.34	0.00	0	11	177	3	688.68732	2064.04740	-6.16	20.54	1968	1968	MS2	26/72	Glioma_Nuclear_prep_Gradeall_repeat_4plex.raw
30795231	High		aEPPkAPEQEQAAPGPAAGGEAPk	Unambiguous	1	1	N-Term(iTRAQ4plex); K5(iTRAQ4plex); K24(iTRAQ4plex)	HCD	1.0000	0.0000	1	1	7840	Unique	Used	0.623	6.151E3	4.820E3	72	6.01	0.00	1	75	24	3	910.82172	2730.45060	3.00	25.22	2279	2279	MS2	32/92	Glioma_Nuclear_prep_Gradeall_4plex.raw
31542947	NP_002147.2	HSPD1	3329	60 kDa heat shock protein, mitochondrial [Homo sapiens]	8.20	1	2	3	1.491	2;1	4.5	15.79	573	61.0	5.87																			
31542947	High		tLNDELEIIEGMk	Unambiguous	1	1	N-Term(iTRAQ4plex); K13(iTRAQ4plex)	HCD	1.0000	0.0000	1	1	12147	Unique	Used	1.524	1.211E4	2.322E4	223	5.66	0.00	0	34	145	2	896.98364	1792.96001	-0.30	58.45	6973	6973	MS2	22/24	Glioma_Nuclear_prep_Gradeall_4plex.raw
31542947	High		lSDGVAVLk	Unambiguous	1	1	N-Term(iTRAQ4plex); K9(iTRAQ4plex)	HCD	1.0000	0.0000	1	1	9027	Unique	Used	1.397	7.304E4	1.283E5	333	2.92	0.00	0	40	21	2	595.36261	1189.71794	-18.11	33.71	3568	3568	MS2	16/16	Glioma_Nuclear_prep_Gradeall_4plex.raw
31542947		High	lSDGVAVLk	Unambiguous	1	1	N-Term(iTRAQ4plex); K9(iTRAQ4plex)	HCD	1.0000	0.0000	1	1	9569	Unique	Used	1.491	7.937E3	1.603E4	271	3.41	0.00	0	17	273	2	595.36792	1189.72856	-9.18	40.06	3990	3990	MS2	16/16	Glioma_Nuclear_prep_Gradeall_repeat_4plex.raw
32171175	NP_003901.2	BUD31	8896	protein BUD31 homolog [Homo sapiens]	11.11	1	2	2	2.025	1;1	13.9	3.82	144	17.0	8.82																			
32171175		High	eLYEYclk	Unambiguous	1	1	N-Term(iTRAQ4plex); C6(Methylthio); K8(iTRAQ4plex)	HCD	1.0000	0.0000	1	1	11596	Unique	Used	1.826	4.312E3	1.067E4	160	3.34	0.00	0	64	300	2	697.85168	1394.69609	1.62	65.52	6245	6245	MS2	13/14	Glioma_Nuclear_prep_Gradeall_repeat_4plex.raw
32171175	High		yIFDLFYk	Unambiguous	1	1	N-Term(iTRAQ4plex); K8(iTRAQ4plex)	HCD	1.0000	0.0000	1	1	12583	Unique	Used	2.224	9.944E3	2.781E4	218	3.82	0.00	0	33	300	2	698.89063	1396.77397	-1.10	63.47	7463	7463	MS2	13/14	Glioma_Nuclear_prep_Gradeall_4plex.raw
33946297	NP_892021.1	ZNF326	284695	DBIRD complex subunit ZNF326 isoform 1 [Homo sapiens]	4.47	1	2	3	0.438	1;2	26.3	11.92	582	65.6	5.15																			
33946297		High	fGPYESYDSR	Unambiguous	1	1	N-Term(iTRAQ4plex)	HCD	1.0000	0.0000	1	1	9179	Unique	Used	0.277	1.672E4	6.282E3	67	3.08	0.00	0	8	300	2	682.81464	1364.62200	-1.42	35.73	3572	3572	MS2	12/18	Glioma_Nuclear_prep_Gradeall_repeat_4plex.raw
33946297	High		eSVLTATSILNNPIVk	Unambiguous	1	1	N-Term(iTRAQ4plex); K16(iTRAQ4plex)	HCD	1.0000	0.0000	1	1	11311	Unique	Used	0.438	2.289E4	1.260E4	99	7.53	0.00	0	13	108	2	994.08472	1987.16216	-2.85	50.95	6060	6060	MS2	24/30	Glioma_Nuclear_prep_Gradeall_4plex.raw
33946297		High	eSVLTATSILNNPIVk	Unambiguous	1	1	N-Term(iTRAQ4plex); K16(iTRAQ4plex)	HCD	1.0000	0.0000	1	1	12038	Unique	Used	0.473	2.860E2	1.831E2	94	5.01	0.00	0	32	300	3	663.06213	1987.17185	2.02	72.71	6835	6835	MS2	24/60	Glioma_Nuclear_prep_Gradeall_repeat_4plex.raw
34101286	NP_057191.2	ZFR	51663	zinc finger RNA-binding protein [Homo sapiens]	6.24	1	4	4	0.592	2;2	39.0	11.14	1074	116.9	9.04																			
34101286	High		cAVSEAAILNScVEPk	Unambiguous	1	1	N-Term(iTRAQ4plex); C1(Methylthio); C13(Methylthio); K17(iTRAQ4plex)	HCD	1.0000	0.0000	1	1	12974	Unique	Used	0.748	3.486E2	3.280E2	115	4.20	0.00	0	30	300	3	709.69281	2127.06388	3.64	69.42	7956	7956	MS2	21/64	Glioma_Nuclear_prep_Gradeall_4plex.raw
34101286	High		vSAVPTNMAAk	Unambiguous	1	1	N-Term(iTRAQ4plex); K11(iTRAQ4plex)	HCD	1.0000	0.0000	1	1	8206	Unique	Used	0.454	1.760E5	1.004E5	325	3.48	0.00	0	29	13	2	688.88409	1376.76091	-14.63	27.82	2676	2676	MS2	15/20	Glioma_Nuclear_prep_Gradeall_4plex.raw
34101286		High	sTPVTSAVQIPEVk	Unambiguous	1	1	N-Term(iTRAQ4plex); K14(iTRAQ4plex)	HCD	1.0000	0.0000	1	1	10225	Unique	Used	0.301	4.569E3	1.866E3	173	4.77	0.00	0	43	300	2	872.51343	1744.01958	5.76	47.75	4694	4694	MS2	20/26	Glioma_Nuclear_prep_Gradeall_repeat_4plex.raw
34101286		High	hATIYPTEEELQAVQk	Unambiguous	1	1	N-Term(iTRAQ4plex); K16(iTRAQ4plex)	HCD	1.0000	0.0000	1	1	9918	Unique	Used	0.730	1.132E3	1.119E3	163	6.17	0.00	0	52	300	3	715.72443	2145.15873	7.30	44.02	4361	4361	MS2	25/60	Glioma_Nuclear_prep_Gradeall_repeat_4plex.raw
34740329	NP_919223.1	HNRNPA3	220988	heterogeneous nuclear ribonucleoprotein A3 [Homo sapiens]	31.22	1	10	26	0.148	16;10	38.8	97.89	378	39.6	9.01																			
34740329	High		sSGSPYGGGYGSGGGSGGYGSR	Unambiguous	1	1	N-Term(iTRAQ4plex)	HCD	1.0000	0.0000	1	1	7861	Unique	Used	0.065	1.219E5	9.920E3	33	6.75	0.00	0	0	14	2	1027.95142	2054.89556	1.68	25.37	2302	2302	MS2	33/42	Glioma_Nuclear_prep_Gradeall_4plex.raw
34740329	High		lFIGGLSFETTDDSLR	Unambiguous	1	1	N-Term(iTRAQ4plex)	HCD	1.0000	0.0000	1	1	12193	Unique	Used	0.076	2.424E5	2.305E4	239	5.25	0.00	0	13	31	2	958.00537	1915.00347	5.46	58.91	7024	7024	MS2	23/30	Glioma_Nuclear_prep_Gradeall_4plex.raw
34740329	High		lFIGGLSFETTDDSLR	Unambiguous	1	1	N-Term(iTRAQ4plex)	HCD	1.0000	0.0000	1	1	12115	Unique	Used	0.096	1.394E5	1.691E4	235	5.40	0.00	0	6	35	2	958.00354	1914.99980	3.55	58.18	6939	6939	MS2	23/30	Glioma_Nuclear_prep_Gradeall_4plex.raw
34740329	High		iETIEVMEDR	Unambiguous	1	1	N-Term(iTRAQ4plex)	HCD	1.0000	0.0000	1	1	9630	Unique	Used	0.173	4.496E5	9.801E4	173	3.76	0.00	0	34	10	2	689.84448	1378.68169	-13.66	38.18	4228	4228	MS2	18/18	Glioma_Nuclear_prep_Gradeall_4plex.raw
34740329	High		eDTEEYNLR	Unambiguous	1	1	N-Term(iTRAQ4plex)	HCD	1.0000	0.0000	1	1	7783	Unique	Used	0.219	8.174E5	2.249E5	109	3.55	0.00	0	7	5	2	656.81104	1312.61479	0.78	24.83	2218	2218	MS2	16/16	Glioma_Nuclear_prep_Gradeall_4plex.raw
34740329	High		iETIEVMEDR	Unambiguous	1	1	N-Term(iTRAQ4plex)	HCD	1.0000	0.0000	1	1	9697	Unique	Used	0.272	1.246E6	4.268E5	242	3.85	0.00	0	54	5	2	689.86011	1378.71294	9.01	38.67	4301	4301	MS2	14/18	Glioma_Nuclear_prep_Gradeall_4plex.raw
34740329		High	eDTEEYNLR	Unambiguous	1	1	N-Term(iTRAQ4plex)	HCD	1.0000	0.0000	1	1	7928	Unique	Used	0.129	7.413E4	1.296E4	107	3.74	0.00	0	10	128	2	656.81165	1312.61601	1.71	23.07	2250	2250	MS2	16/16	Glioma_Nuclear_prep_Gradeall_repeat_4plex.raw
34740329		High	iETIEVMEDR	Unambiguous	1	1	N-Term(iTRAQ4plex)	HCD	1.0000	0.0000	1	1	10250	Unique	Used	0.149	4.830E4	9.764E3	231	4.21	0.00	0	38	193	2	689.85565	1378.70403	2.55	48.04	4721	4721	MS2	14/18	Glioma_Nuclear_prep_Gradeall_repeat_4plex.raw
34740329		High	lFIGGLSFETTDDSLR	Unambiguous	1	1	N-Term(iTRAQ4plex)	HCD	1.0000	0.0000	1	1	12394	Unique	Used	0.233	2.010E2	6.336E1	184	4.25	0.00	0	16	300	3	639.00293	1914.99424	0.64	83.05	7599	7599	MS2	23/60	Glioma_Nuclear_prep_Gradeall_repeat_4plex.raw
34740329	High		wGTLTDcVVMR	Unambiguous	1	1	N-Term(iTRAQ4plex); C7(Methylthio)	HCD	1.0000	0.0000	1	1	11830	Unique	Used	0.096	3.722E5	4.481E4	89	4.21	0.00	0	10	23	2	735.85382	1470.70036	-1.41	55.46	6627	6627	MS2	19/20	Glioma_Nuclear_prep_Gradeall_4plex.raw
34740329	High		wGTLTDcVVMR	Unambiguous	1	1	N-Term(iTRAQ4plex); C7(Methylthio)	HCD	1.0000	0.0000	1	1	11758	Unique	Used	0.127	8.888E4	1.422E4	103	3.96	0.00	0	34	98	2	735.85516	1470.70305	0.41	54.85	6548	6548	MS2	19/20	Glioma_Nuclear_prep_Gradeall_4plex.raw
34740329	High		kLFIGGLSFETTDDSLR	Unambiguous	1	1	N-Term(iTRAQ4plex); K1(iTRAQ4plex)	HCD	1.0000	0.0000	1	1	11117	Unique	Used	0.146	6.051E4	1.109E4	154	5.46	0.00	1	5	47	2	1094.09705	2187.18682	-1.47	49.36	5848	5848	MS2	25/32	Glioma_Nuclear_prep_Gradeall_4plex.raw
34740329	High		kLFIGGLSFETTDDSLR	Unambiguous	1	1	N-Term(iTRAQ4plex); K1(iTRAQ4plex)	HCD	1.0000	0.0000	1	1	11062	Unique	Used	0.331	1.834E4	7.634E3	136	6.96	0.00	1	28	18	3	729.73425	2187.18821	-0.84	48.94	5789	5789	MS2	27/64	Glioma_Nuclear_prep_Gradeall_4plex.raw
34740329	High		gFAFVTFDDHDTVDk	Unambiguous	1	1	N-Term(iTRAQ4plex); K15(iTRAQ4plex)	HCD	1.0000	0.0000	1	1	10293	Unique	Used	0.152	2.238E5	4.289E4	85	5.64	0.00	0	4	9	2	1001.47919	2001.95110	-14.29	43.03	4949	4949	MS2	24/28	Glioma_Nuclear_prep_Gradeall_4plex.raw
34740329	High		gFAFVTFDDHDTVDk	Unambiguous	1	1	N-Term(iTRAQ4plex); K15(iTRAQ4plex)	HCD	1.0000	0.0000	1	1	10395	Unique	Used	0.192	9.822E4	2.372E4	85	5.74	0.00	0	21	16	2	1001.47864	2001.95000	-14.83	43.80	5060	5060	MS2	24/28	Glioma_Nuclear_prep_Gradeall_4plex.raw
34740329	High		gFAFVTFDDHDTVDk	Unambiguous	1	1	N-Term(iTRAQ4plex); K15(iTRAQ4plex)	HCD	1.0000	0.0000	1	1	10283	Unique	Used	0.203	6.342E4	1.622E4	78	4.61	0.00	0	31	8	3	667.98853	2001.95102	-14.32	42.96	4938	4938	MS2	28/56	Glioma_Nuclear_prep_Gradeall_4plex.raw
34740329	High		gFAFVTFDDHDTVDk	Unambiguous	1	1	N-Term(iTRAQ4plex); K15(iTRAQ4plex)	HCD	1.0000	0.0000	1	1	10443	Unique	Used	0.229	6.117E4	1.759E4	87	4.94	0.00	0	29	7	3	667.98877	2001.95176	-13.96	44.19	5114	5114	MS2	31/56	Glioma_Nuclear_prep_Gradeall_4plex.raw
34740329		High	gFAFVTFDDHDTVDk	Unambiguous	1	1	N-Term(iTRAQ4plex); K15(iTRAQ4plex)	HCD	1.0000	0.0000	1	1	11239	Unique	Used	0.113	5.979E3	9.133E2	121	6.09	0.00	0	10	300	2	1001.49506	2001.98284	1.57	60.30	5806	5806	MS2	24/28	Glioma_Nuclear_prep_Gradeall_repeat_4plex.raw
34740329		High	gFAFVTFDDHDTVDk	Unambiguous	1	1	N-Term(iTRAQ4plex); K15(iTRAQ4plex)	HCD	1.0000	0.0000	1	1	11299	Unique	Used	0.129	4.554E3	7.967E2	117	4.90	0.00	0	13	209	3	667.99994	2001.98526	2.78	61.24	5883	5883	MS2	29/56	Glioma_Nuclear_prep_Gradeall_repeat_4plex.raw
34740329		High	gFAFVTFDDHDTVDk	Unambiguous	1	1	N-Term(iTRAQ4plex); K15(iTRAQ4plex)	HCD	1.0000	0.0000	1	1	11232	Unique	Used	0.158	6.908E3	1.476E3	108	4.99	0.00	0	11	116	3	667.99921	2001.98307	1.68	60.21	5798	5798	MS2	30/56	Glioma_Nuclear_prep_Gradeall_repeat_4plex.raw
34740329		High	gFAFVTFDDHDTVDk	Unambiguous	1	1	N-Term(iTRAQ4plex); K15(iTRAQ4plex)	HCD	1.0000	0.0000	1	1	11364	Unique	Used	0.167	1.279E3	2.886E2	106	4.97	0.00	0	30	300	3	668.00067	2001.98746	3.88	62.31	5970	5970	MS2	27/56	Glioma_Nuclear_prep_Gradeall_repeat_4plex.raw
34740329		High	eDSVkPGAHLTVk	Unambiguous	1	1	N-Term(iTRAQ4plex); K5(iTRAQ4plex); K13(iTRAQ4plex)	HCD	1.0000	0.0000	1	1	7708	Unique	Used	0.101	1.616E4	2.206E3	126	4.91	0.00	0	19	300	2	907.02472	1813.04216	-6.76	21.01	2018	2018	MS2	17/24	Glioma_Nuclear_prep_Gradeall_repeat_4plex.raw
34740329	High		iFVGGIkEDTEEYNLR	Unambiguous	1	1	N-Term(iTRAQ4plex); K7(iTRAQ4plex)	HCD	1.0000	0.0000	1	1	10057	Unique	Used	0.124	1.893E5	2.952E4	152	5.26	0.00	1	2	12	2	1086.06665	2171.12602	-15.06	41.26	4691	4691	MS2	24/30	Glioma_Nuclear_prep_Gradeall_4plex.raw
34740329		High	iFVGGIkEDTEEYNLR	Unambiguous	1	1	N-Term(iTRAQ4plex); K7(iTRAQ4plex)	HCD	1.0000	0.0000	1	1	10950	Unique	Used	0.128	4.178E3	7.250E2	121	5.80	0.00	1	5	300	2	1086.08557	2171.16387	2.37	56.54	5477	5477	MS2	25/30	Glioma_Nuclear_prep_Gradeall_repeat_4plex.raw
34740329		High	iFVGGIkEDTEEYNLR	Unambiguous	1	1	N-Term(iTRAQ4plex); K7(iTRAQ4plex)	HCD	1.0000	0.0000	1	1	10937	Unique	Used	0.147	4.754E3	9.493E2	134	3.50	0.00	1	14	263	3	724.39355	2171.16611	3.40	56.39	5464	5464	MS2	25/60	Glioma_Nuclear_prep_Gradeall_repeat_4plex.raw
34740329	High		gEEGHDPkEPEQLR	Unambiguous	1	1	N-Term(iTRAQ4plex); K8(iTRAQ4plex)	HCD	1.0000	0.0000	1	1	7282	Unique	Used	0.163	2.799E4	5.750E3	98	3.88	0.00	1	4	28	2	954.98309	1908.95891	-3.42	21.22	1674	1674	MS2	18/26	Glioma_Nuclear_prep_Gradeall_4plex.raw
38201710	NP_006377.2	DDX17	10521	probable ATP-dependent RNA helicase DDX17 isoform 1 [Homo sapiens]	32.24	7	15	25	0.535	11;14	23.5	87.65	729	80.2	8.27																			
38201710	High		aPLPDLYPFGTMR	Unambiguous	2	1	N-Term(iTRAQ4plex)	HCD	1.0000	0.0000	1	1	11594	Unique	Used	0.254	1.812E5	5.789E4	310	3.18	0.00	0	33	27	2	811.43036	1621.85344	0.31	53.36	6370	6370	MS2	12/24	Glioma_Nuclear_prep_Gradeall_4plex.raw
38201710	High		lTPYEVDELR	Unambiguous	2	1	N-Term(iTRAQ4plex)	HCD	1.0000	0.0000	1	1	9710	Unique	Used	0.376	1.314E6	6.210E5	243	4.00	0.00	0	9	4	2	689.86035	1378.71343	-14.56	38.76	4315	4315	MS2	16/18	Glioma_Nuclear_prep_Gradeall_4plex.raw
38201710	High		nFYVEHPEVAR	Unambiguous	2	1	N-Term(iTRAQ4plex)	HCD	1.0000	0.0000	1	1	8209	Unique	Used	0.617	1.608E5	1.247E5	142	2.89	0.00	0	13	13	2	752.87524	1504.74321	-15.50	27.83	2679	2679	MS2	15/20	Glioma_Nuclear_prep_Gradeall_4plex.raw

38201710	High		fVINYDYPNSSEDYVHR	Unambiguous	2	1	N-Term(iTRAQ4plex)	HCD	1.0000	0.0000	1	1	9998	Unique	Used	0.678	1.427E4	1.216E4	58	5.46	0.00	0	36	16	3	754.68011	2262.02579	-14.42	40.83	4627	4627	MS2	26/64	Glioma_Nuclear_prep_Gradeall_4plex.raw
38201710		High	sSQSSSQQFSGIGR	Unambiguous	2	1	N-Term(iTRAQ4plex)	HCD	1.0000	0.0000	1	1	7908	Unique	Used	0.340	1.000E4	4.600E3	119	4.84	0.00	0	0	293	2	800.39044	1599.77361	-6.73	22.89	2229	2229	MS2	23/26	Glioma_Nuclear_prep_Gradeall_repeat_4plex.raw
38201710		High	lTPYEVDELR	Unambiguous	2	1	N-Term(iTRAQ4plex)	HCD	1.0000	0.0000	1	1	10338	Unique	Used	0.349	1.968E4	9.299E3	289	3.96	0.00	0	56	300	2	689.87238	1378.73747	2.88	49.11	4816	4816	MS2	15/18	Glioma_Nuclear_prep_Gradeall_repeat_4plex.raw
38201710		High	lMQLVDHR	Unambiguous	2	1	N-Term(iTRAQ4plex)	HCD	1.0000	0.0000	1	1	9388	Unique	Used	0.447	6.504E3	3.939E3	181	3.84	0.00	0	17	239	3	385.88513	1155.64084	-1.46	38.02	3792	3792	MS2	16/28	Glioma_Nuclear_prep_Gradeall_repeat_4plex.raw
38201710		High	lTPYEVDELR	Unambiguous	2	1	N-Term(iTRAQ4plex)	HCD	1.0000	0.0000	1	1	10430	Unique	Used	0.450	6.631E3	4.040E3	298	3.23	0.00	0	49	300	2	689.87115	1378.73503	1.11	50.18	4913	4913	MS2	13/18	Glioma_Nuclear_prep_Gradeall_repeat_4plex.raw
38201710		High	dWVLNEFR	Unambiguous	2	1	N-Term(iTRAQ4plex)	HCD	1.0000	0.0000	1	1	11588	Unique	Used	0.577	5.811E3	4.543E3	279	3.17	0.00	0	48	300	2	611.82257	1222.63786	3.38	65.42	6236	6236	MS2	13/14	Glioma_Nuclear_prep_Gradeall_repeat_4plex.raw
38201710		High	nFYVEHPEVAR	Unambiguous	2	1	N-Term(iTRAQ4plex)	HCD	1.0000	0.0000	1	1	8598	Unique	Used	0.591	1.321E4	1.057E4	160	3.48	0.00	0	32	300	2	752.88635	1504.76543	-0.73	29.61	2956	2956	MS2	14/20	Glioma_Nuclear_prep_Gradeall_repeat_4plex.raw
38201710		High	nFYVEHPEVAR	Unambiguous	2	1	N-Term(iTRAQ4plex)	HCD	1.0000	0.0000	1	1	8590	Unique	Used	0.671	1.139E4	1.036E4	150	4.12	0.00	0	43	123	3	502.25986	1504.76502	-1.01	29.53	2948	2948	MS2	21/40	Glioma_Nuclear_prep_Gradeall_repeat_4plex.raw
38201710		High	cTYLVLDEADR	Unambiguous	2	1	N-Term(iTRAQ4plex); C1(Methylthio)	HCD	1.0000	0.0000	1	1	11402	Unique	Used	0.542	4.999E3	3.667E3	123	4.09	0.00	0	34	300	2	744.35461	1487.70195	1.91	62.83	6015	6015	MS2	17/20	Glioma_Nuclear_prep_Gradeall_repeat_4plex.raw
38201710	High		dMVGIAQTGSGk	Unambiguous	2	1	N-Term(iTRAQ4plex); K12(iTRAQ4plex)	HCD	1.0000	0.0000	1	1	8169	Unique	Used	0.534	2.863E5	1.923E5	206	3.94	0.00	0	5	7	2	726.38080	1451.75432	-15.41	27.56	2636	2636	MS2	19/22	Glioma_Nuclear_prep_Gradeall_4plex.raw
38201710	High		vLEEANQAINPk	Unambiguous	2	1	N-Term(iTRAQ4plex); K12(iTRAQ4plex)	HCD	1.0000	0.0000	1	1	9071	Unique	Used	0.535	1.492E5	1.003E5	234	4.87	0.00	0	11	15	2	807.44653	1613.88579	-15.09	34.06	3617	3617	MS2	21/22	Glioma_Nuclear_prep_Gradeall_4plex.raw
38201710		High	dMVGIAQTGSGk	Unambiguous	2	1	N-Term(iTRAQ4plex); K12(iTRAQ4plex)	HCD	1.0000	0.0000	1	1	8387	Unique	Used	0.759	1.432E4	1.472E4	253	4.15	0.00	0	66	300	2	726.39417	1451.78105	3.00	27.53	2733	2733	MS2	18/22	Glioma_Nuclear_prep_Gradeall_repeat_4plex.raw
38201710	High		gTAYTFFTPGNLk	Unambiguous	2	1	N-Term(iTRAQ4plex); K13(iTRAQ4plex)	HCD	1.0000	0.0000	1	1	10533	Unique	Used	0.556	1.946E5	1.362E5	205	5.06	0.00	0	14	9	2	852.95264	1704.89800	-12.89	44.90	5213	5213	MS2	21/24	Glioma_Nuclear_prep_Gradeall_4plex.raw
38201710		High	gTAYTFFTPGNLk	Unambiguous	2	1	N-Term(iTRAQ4plex); K13(iTRAQ4plex)	HCD	1.0000	0.0000	1	1	11411	Unique	Used	0.560	2.965E3	2.249E3	235	4.82	0.00	0	13	300	2	852.96509	1704.92290	1.72	62.96	6026	6026	MS2	20/24	Glioma_Nuclear_prep_Gradeall_repeat_4plex.raw
38201710	High		eLAQQVQQVADDYGk	Unambiguous	2	1	N-Term(iTRAQ4plex); K15(iTRAQ4plex)	HCD	1.0000	0.0000	1	1	10415	Unique	Used	0.549	1.088E5	7.516E4	163	6.27	0.00	0	6	20	2	990.50336	1979.99944	-14.27	43.96	5082	5082	MS2	26/28	Glioma_Nuclear_prep_Gradeall_4plex.raw
38201710		High	eLAQQVQQVADDYGk	Unambiguous	2	1	N-Term(iTRAQ4plex); K15(iTRAQ4plex)	HCD	1.0000	0.0000	1	1	11222	Unique	Used	0.440	2.980E3	1.776E3	193	6.35	0.00	0	20	300	2	990.51953	1980.03179	2.06	60.09	5787	5787	MS2	26/28	Glioma_Nuclear_prep_Gradeall_repeat_4plex.raw
38201710	High		tIIFVETk	Unambiguous	2	1	N-Term(iTRAQ4plex); K8(iTRAQ4plex)	HCD	1.0000	0.0000	1	1	9404	Unique	Used	0.673	7.834E4	6.633E4	235	3.45	0.00	0	37	15	2	619.87274	1238.73821	-17.49	36.54	3982	3982	MS2	13/14	Glioma_Nuclear_prep_Gradeall_4plex.raw
38201710		High	tIIFVETk	Unambiguous	2	1	N-Term(iTRAQ4plex); K8(iTRAQ4plex)	HCD	1.0000	0.0000	1	1	10028	Unique	Used	0.482	1.548E4	1.011E4	176	3.54	0.00	0	59	300	2	619.88477	1238.76225	1.92	45.38	4482	4482	MS2	13/14	Glioma_Nuclear_prep_Gradeall_repeat_4plex.raw
38201710		High	wDLSELPk	Unambiguous	2	1	N-Term(iTRAQ4plex); K8(iTRAQ4plex)	HCD	1.0000	0.0000	1	1	11023	Unique	Used	0.734	5.558E3	5.526E3	271	3.01	0.00	0	60	300	2	638.36493	1275.72258	3.01	57.44	5557	5557	MS2	13/14	Glioma_Nuclear_prep_Gradeall_repeat_4plex.raw
38201710	High		lIDFLESGk	Unambiguous	2	1	N-Term(iTRAQ4plex); K9(iTRAQ4plex)	HCD	1.0000	0.0000	1	1	11339	Unique	Used	0.501	1.553E5	9.783E4	297	3.56	0.00	0	35	30	2	655.38434	1309.76140	0.60	51.22	6092	6092	MS2	15/16	Glioma_Nuclear_prep_Gradeall_4plex.raw
38201710	High		lIDFLESGk	Unambiguous	2	1	N-Term(iTRAQ4plex); K9(iTRAQ4plex)	HCD	1.0000	0.0000	1	1	11261	Unique	Used	0.535	1.603E5	1.079E5	297	3.80	0.00	0	21	39	2	655.38251	1309.75774	-2.20	50.53	6006	6006	MS2	15/16	Glioma_Nuclear_prep_Gradeall_4plex.raw
38201710		High	lIDFLESGk	Unambiguous	2	1	N-Term(iTRAQ4plex); K9(iTRAQ4plex)	HCD	1.0000	0.0000	1	1	12023	Unique	Used	0.459	1.722E4	1.072E4	281	3.49	0.00	0	16	300	2	655.38495	1309.76262	1.53	72.44	6813	6813	MS2	15/16	Glioma_Nuclear_prep_Gradeall_repeat_4plex.raw
38201714	NP_001410.2	ELAVL1	1994	ELAV-like protein 1 [Homo sapiens]	31.29	1	7	11	0.174	4;7	39.7	43.19	326	36.1	9.17																			
38201714	High		nVALLSQLYHSPAR	Unambiguous	1	1	N-Term(iTRAQ4plex)	HCD	1.0000	0.0000	1	1	9882	Unique	Used	0.287	4.772E4	1.724E4	136	5.61	0.00	0	14	4	3	571.64819	1712.93003	-15.43	40.01	4502	4502	MS2	32/52	Glioma_Nuclear_prep_Gradeall_4plex.raw
38201714	High		dANLYISGLPR	Unambiguous	1	1	N-Term(iTRAQ4plex)	HCD	1.0000	0.0000	1	1	9821	Unique	Used	0.338	3.605E5	1.532E5	277	3.91	0.00	0	26	8	2	681.86853	1362.72978	-14.71	39.55	4435	4435	MS2	17/20	Glioma_Nuclear_prep_Gradeall_4plex.raw
38201714		High	vLVDQTTGLSR	Unambiguous	1	1	N-Term(iTRAQ4plex)	HCD	1.0000	0.0000	1	1	8901	Unique	Used	0.167	8.129E4	1.837E4	253	3.62	0.00	0	20	69	2	666.88263	1332.75798	-1.81	32.67	3275	3275	MS2	17/20	Glioma_Nuclear_prep_Gradeall_repeat_4plex.raw
38201714		High	nVALLSQLYHSPAR	Unambiguous	1	1	N-Term(iTRAQ4plex)	HCD	1.0000	0.0000	1	1	10843	Unique	Used	0.173	4.569E3	1.069E3	178	5.09	0.00	0	20	300	2	856.98328	1712.95928	1.64	55.21	5360	5360	MS2	22/26	Glioma_Nuclear_prep_Gradeall_repeat_4plex.raw
38201714	High		sLFSSIGEVESAk	Unambiguous	1	1	N-Term(iTRAQ4plex); K13(iTRAQ4plex)	HCD	1.0000	0.0000	1	1	10617	Unique	Used	0.100	3.200E5	4.036E4	217	5.19	0.00	0	8	8	2	821.43945	1641.87163	-13.52	45.52	5304	5304	MS2	23/24	Glioma_Nuclear_prep_Gradeall_4plex.raw
38201714		High	sLFSSIGEVESAk	Unambiguous	1	1	N-Term(iTRAQ4plex); K13(iTRAQ4plex)	HCD	1.0000	0.0000	1	1	11486	Unique	Used	0.105	8.261E3	1.177E3	217	5.59	0.00	0	12	300	2	821.45276	1641.89824	2.69	63.96	6113	6113	MS2	22/24	Glioma_Nuclear_prep_Gradeall_repeat_4plex.raw
38201714		High	sLFSSIGEVESAk	Unambiguous	1	1	N-Term(iTRAQ4plex); K13(iTRAQ4plex)	HCD	1.0000	0.0000	1	1	11485	Unique	Used	0.179	1.372E3	3.331E2	225	5.16	0.00	0	9	281	3	547.97009	1641.89573	1.16	63.95	6112	6112	MS2	22/48	Glioma_Nuclear_prep_Gradeall_repeat_4plex.raw
38201714		High	iLQVSFk	Unambiguous	1	1	N-Term(iTRAQ4plex); K7(iTRAQ4plex)	HCD	1.0000	0.0000	1	1	10482	Unique	Used	0.213	2.330E4	6.720E3	148	2.97	0.00	0	52	299	2	561.86072	1122.71416	1.45	50.82	4969	4969	MS2	11/12	Glioma_Nuclear_prep_Gradeall_repeat_4plex.raw
38201714		High	fAANPNQNk	Unambiguous	1	1	N-Term(iTRAQ4plex); K9(iTRAQ4plex)	HCD	1.0000	0.0000	1	1	7239	Unique	Used	0.200	6.692E4	1.816E4	198	3.33	0.00	0	19	71	2	646.34338	1291.67949	-15.67	16.86	1521	1521	MS2	16/16	Glioma_Nuclear_prep_Gradeall_repeat_4plex.raw
38201714	High		tNLIVNYLPQNmTQDELR	Unambiguous	1	1	N-Term(iTRAQ4plex); M12(Oxidation)	HCD	1.0000	0.0000	1	1	10837	Unique	Used	0.174	3.644E4	7.990E3	115	4.38	0.00	0	17	23	2	1161.58154	2322.15581	-13.90	47.20	5544	5544	MS2	19/34	Glioma_Nuclear_prep_Gradeall_4plex.raw
38201714		High	tNLIVNYLPQNmTQDELR	Unambiguous	1	1	N-Term(iTRAQ4plex); M12(Oxidation)	HCD	1.0000	0.0000	1	1	11625	Unique	Used	0.110	6.121E2	9.130E1	130	3.80	0.00	0	4	300	2	1161.59644	2322.18559	-1.07	65.90	6278	6278	MS2	21/34	Glioma_Nuclear_prep_Gradeall_repeat_4plex.raw
48255933	NP_004956.5	HMGN1	3150	non-histone chromosomal protein HMG-14 [Homo sapiens]	33.00	1	4	9	1.940	4;5	18.2	24.98	100	10.7	9.60																			
48255933	High		kVSSAEGAAk	Unambiguous	1	1	N-Term(iTRAQ4plex); K1(iTRAQ4plex); K10(iTRAQ4plex)	HCD	1.0000	0.0000	1	1	7026	Unique	Used	1.766	3.690E3	8.199E3	178	3.72	0.00	1	24	300	2	690.41461	1379.82195	0.05	18.81	1392	1392	MS2	18/18	Glioma_Nuclear_prep_Gradeall_4plex.raw
48255933	High		kVSSAEGAAk	Unambiguous	1	1	N-Term(iTRAQ4plex); K1(iTRAQ4plex); K10(iTRAQ4plex)	HCD	1.0000	0.0000	1	1	7007	Unique	Used	1.940	3.284E3	8.014E3	192	5.03	0.00	1	43	105	3	460.61206	1379.82163	-0.18	18.61	1372	1372	MS2	24/36	Glioma_Nuclear_prep_Gradeall_4plex.raw
48255933		High	kVSSAEGAAk	Unambiguous	1	1	N-Term(iTRAQ4plex); K1(iTRAQ4plex); K10(iTRAQ4plex)	HCD	1.0000	0.0000	1	1	6643	Unique	Used	1.807	6.239E3	1.528E4	273	5.24	0.00	1	42	88	3	460.60678	1379.80579	-11.66	11.59	892	892	MS2	25/36	Glioma_Nuclear_prep_Gradeall_repeat_4plex.raw
48255933	High		qAEVANQETk	Unambiguous	1	1	N-Term(iTRAQ4plex); K10(iTRAQ4plex)	HCD	1.0000	0.0000	1	1	7091	Unique	Used	2.252	5.295E3	1.500E4	202	4.17	0.00	0	39	300	2	703.38110	1405.75493	1.68	19.52	1463	1463	MS2	18/18	Glioma_Nuclear_prep_Gradeall_4plex.raw
48255933		High	qAEVANQETk	Unambiguous	1	1	N-Term(iTRAQ4plex); K10(iTRAQ4plex)	HCD	1.0000	0.0000	1	1	6787	Unique	Used	1.375	4.293E3	7.996E3	250	3.78	0.00	0	77	181	3	469.25674	1405.75568	2.21	12.89	1043	1043	MS2	14/36	Glioma_Nuclear_prep_Gradeall_repeat_4plex.raw
48255933		High	qAEVANQETk	Unambiguous	1	1	N-Term(iTRAQ4plex); K10(iTRAQ4plex)	HCD	1.0000	0.0000	1	1	6783	Unique	Used	2.344	1.133E4	3.598E4	196	4.09	0.00	0	16	185	2	703.38104	1405.75481	1.59	12.86	1039	1039	MS2	18/18	Glioma_Nuclear_prep_Gradeall_repeat_4plex.raw
48255933	High		tEESPASDEAGEk	Unambiguous	1	1	N-Term(iTRAQ4plex); K13(iTRAQ4plex)	HCD	1.0000	0.0000	1	1	7219	Unique	Used	1.900	9.934E3	2.374E4	79	4.22	0.00	0	26	88	2	819.38757	1637.76787	-4.05	20.69	1603	1603	MS2	21/24	Glioma_Nuclear_prep_Gradeall_4plex.raw
48255933		High	tEESPASDEAGEk	Unambiguous	1	1	N-Term(iTRAQ4plex); K13(iTRAQ4plex)	HCD	1.0000	0.0000	1	1	6980	Unique	Used	2.593	1.433E4	5.034E4	83	4.91	0.00	0	5	66	2	819.38947	1637.77165	-1.74	14.61	1247	1247	MS2	24/24	Glioma_Nuclear_prep_Gradeall_repeat_4plex.raw
48255933		High	vSSAEGAAk	Unambiguous	1	1	N-Term(iTRAQ4plex); K9(iTRAQ4plex)	HCD	1.0000	0.0000	1	1	6797	Unique	Used	2.254	4.950E4	1.511E5	295	3.37	0.00	0	13	37	2	554.31647	1107.62566	0.73	13.01	1054	1054	MS2	16/16	Glioma_Nuclear_prep_Gradeall_repeat_4plex.raw
49355765	NP_115657.2	ELAVL3	1995	ELAV-like protein 3 isoform 2 [Homo sapiens]	26.39	2	1	2	0.127	1;1	5.9	29.87	360	38.8	9.28																			
49355765	High		sLFGSIGDIESck	Unambiguous	2	1	N-Term(iTRAQ4plex); C12(Methylthio); K13(iTRAQ4plex)	HCD	1.0000	0.0000	1	1	11822	Unique	Used	0.132	1.094E5	1.816E4	184	5.77	0.00	0	20	74	2	845.42706	1689.84685	2.24	55.39	6618	6618	MS2	22/24	Glioma_Nuclear_prep_Gradeall_4plex.raw
49355765		High	sLFGSIGDIESck	Unambiguous	2	1	N-Term(iTRAQ4plex); C12(Methylthio); K13(iTRAQ4plex)	HCD	1.0000	0.0000	1	1	12321	Unique	Used	0.121	3.147E3	5.176E2	149	4.31	0.00	0	30	300	2	845.43024	1689.85320	6.00	79.59	7357	7357	MS2	20/24	Glioma_Nuclear_prep_Gradeall_repeat_4plex.raw
50428938	NP_004308.2	ASNA1	439	ATPase ASNA1 [Homo sapiens]	5.17	1	1	2	0.667	1;1	23.2	5.74	348	38.8	4.91																			
50428938	High		vNTFSALLLEPYkPPSAQ	Unambiguous	1	1	N-Term(iTRAQ4plex); K13(iTRAQ4plex)	HCD	1.0000	0.0000	1	1	11956	Unique	Used	0.777	2.888E3	2.821E3	135	5.74	0.00	0	27	152	3	755.09137	2263.25956	0.82	56.56	6764	6764	MS2	21/68	Glioma_Nuclear_prep_Gradeall_4plex.raw
50428938		High	vNTFSALLLEPYkPPSAQ	Unambiguous	1	1	N-Term(iTRAQ4plex); K13(iTRAQ4plex)	HCD	1.0000	0.0000	1	1	12365	Unique	Used	0.558	8.724E1	6.596E1	122	3.93	0.00	0	22	300	3	755.09229	2263.26230	2.03	81.27	7477	7477	MS2	22/68	Glioma_Nuclear_prep_Gradeall_repeat_4plex.raw
50726968	NP_005917.2	MFAP1	4236	microfibrillar-associated protein 1 [Homo sapiens]	5.92	1	2	4	1.521	2;2	19.3	10.43	439	51.9	4.98																			
50726968	High		gAFFMDEDEEVYk	Unambiguous	1	1	N-Term(iTRAQ4plex); K13(iTRAQ4plex)	HCD	1.0000	0.0000	1	1	10356	Unique	Used	1.485	2.812E4	5.251E4	38	5.94	0.00	0	8	54	2	934.42297	1867.83867	-14.80	43.50	5017	5017	MS2	23/24	Glioma_Nuclear_prep_Gradeall_4plex.raw
50726968	High		dFSAPTLEDHFNk	Unambiguous	1	1	N-Term(iTRAQ4plex); K13(iTRAQ4plex)	HCD	1.0000	0.0000	1	1	9381	Unique	Used	2.059	2.012E4	5.212E4	100	4.49	0.00	0	49	15	3	603.62903	1808.87253	-18.38	36.37	3957	3957	MS2	24/48	Glioma_Nuclear_prep_Gradeall_4plex.raw
50726968		High	dFSAPTLEDHFNk	Unambiguous	1	1	N-Term(iTRAQ4plex); K13(iTRAQ4plex)	HCD	1.0000	0.0000	1	1	10059	Unique	Used	1.557	1.298E3	2.738E3	137	5.57	0.00	0	79	300	3	603.64063	1808.90732	0.85	45.79	4517	4517	MS2	24/48	Glioma_Nuclear_prep_Gradeall_repeat_4plex.raw
50726968		High	gAFFmDEDEEVYk	Unambiguous	1	1	N-Term(iTRAQ4plex); M5(Oxidation); K13(iTRAQ4plex)	HCD	1.0000	0.0000	1	1	10404	Unique	Used	1.348	1.591E3	2.904E3	55	4.77	0.00	0	11	300	2	942.43439	1883.86150	0.15	49.87	4885	4885	MS2	20/24	Glioma_Nuclear_prep_Gradeall_repeat_4plex.raw
50845386	NP_001002857.1	ANXA2	302	annexin A2 isoform 2 [Homo sapiens]	2.36	2	1	2	3.360	1;1	2.3	3.10	339	38.6	7.75																			
50845386	High		dLYDAGVk	Unambiguous	2	1	N-Term(iTRAQ4plex); K8(iTRAQ4plex)	HCD	1.0000	0.0000	1	1	8173	Unique	Used	3.304	7.127E4	2.962E5	313	3.10	0.00	0	58	14	2	584.81573	1168.62419	-18.01	27.58	2640	2640	MS2	14/14	Glioma_Nuclear_prep_Gradeall_4plex.raw
50845386		High	dLYDAGVk	Unambiguous	2	1	N-Term(iTRAQ4plex); K8(iTRAQ4plex)	HCD	1.0000	0.0000	1	1	8417	Unique	Used	3.416	1.589E4	7.351E4	318	2.94	0.00	0	52	135	2	584.82587	1168.64446	-0.67	27.81	2765	2765	MS2	14/14	Glioma_Nuclear_prep_Gradeall_repeat_4plex.raw
51477708	NP_001003810.1	HNRNPD	3184	heterogeneous nuclear ribonucleoprotein D0 isoform d [Homo sapiens]	20.56	4	4	10	0.132	8;2	26.1	66.61	287	30.7	8.41																			
51477708	High		gFcFITFk	Unambiguous	4	1	N-Term(iTRAQ4plex); C3(Methylthio); K8(iTRAQ4plex)	HCD	1.0000	0.0000	1	1	12229	Unique	Used	0.155	4.933E4	9.594E3	254	3.12	0.00	0	44	178	2	648.84271	1296.67815	4.49	59.28	7064	7064	MS2	13/14	Glioma_Nuclear_prep_Gradeall_4plex.raw
51477708	High		fGEVVDcTLk	Unambiguous	4	1	N-Term(iTRAQ4plex); C7(Methylthio); K10(iTRAQ4plex)	HCD	1.0000	0.0000	1	1	10663	Unique	Used	0.098	5.436E5	6.702E4	134	3.66	0.00	0	43	9	2	722.86627	1444.72527	-11.50	45.88	5355	5355	MS2	18/18	Glioma_Nuclear_prep_Gradeall_4plex.raw
51477708	High		fGEVVDcTLk	Unambiguous	4	1	N-Term(iTRAQ4plex); C7(Methylthio); K10(iTRAQ4plex)	HCD	1.0000	0.0000	1	1	10571	Unique	Used	0.140	4.928E5	8.708E4	128	3.79	0.00	0	32	11	2	722.86511	1444.72295	-13.10	45.18	5254	5254	MS2	18/18	Glioma_Nuclear_prep_Gradeall_4plex.raw
51477708		High	fGEVVDcTLk	Unambiguous	4	1	N-Term(iTRAQ4plex); C7(Methylthio); K10(iTRAQ4plex)	HCD	1.0000	0.0000	1	1	11453	Unique	Used	0.110	1.508E4	2.241E3	165	3.81	0.00	0	49	300	2	722.87622	1444.74516	2.27	63.54	6076	6076	MS2	18/18	Glioma_Nuclear_prep_Gradeall_repeat_4plex.raw
51477708		High	fGEVVDcTLk	Unambiguous	4	1	N-Term(iTRAQ4plex); C7(Methylthio); K10(iTRAQ4plex)	HCD	1.0000	0.0000	1	1	11621	Unique	Used	0.124	1.641E4	2.746E3	188	3.85	0.00	0	38	300	2	722.87689	1444.74651	3.20	65.85	6274	6274	MS2	18/18	Glioma_Nuclear_prep_Gradeall_repeat_4plex.raw
51477708	High		iFVGGLSPDTPEEk	Unambiguous	4	1	N-Term(iTRAQ4plex); K14(iTRAQ4plex)	HCD	1.0000	0.0000	1	1	9910	Unique	Used	0.168	2.086E5	4.395E4	244	4.79	0.00	0	11	11	2	888.97406	1776.94084	-12.05	40.21	4532	4532	MS2	22/26	Glioma_Nuclear_prep_Gradeall_4plex.raw
51477708	High		iFVGGLSPDTPEEk	Unambiguous	4	1	N-Term(iTRAQ4plex); K14(iTRAQ4plex)	HCD	1.0000	0.0000	1	1	9811	Unique	Used	0.184	2.280E5	5.276E4	233	5.37	0.00	0	6	21	2	888.97565	1776.94402	-10.26	39.48	4424	4424	MS2	21/26	Glioma_Nuclear_prep_Gradeall_4plex.raw
51477708	High		eYFGGFGEVESIELPMDNk	Unambiguous	4	1	N-Term(iTRAQ4plex); K19(iTRAQ4plex)	HCD	1.0000	0.0000	1	1	12210	Unique	Used	0.072	3.193E4	2.910E3	113	7.91	0.00	0	2	89	2	1225.10352	2449.19975	6.58	59.09	7043	7043	MS2	27/36	Glioma_Nuclear_prep_Gradeall_4plex.raw
51477708	High		eYFGGFGEVESIELPMDNk	Unambiguous	4	1	N-Term(iTRAQ4plex); K19(iTRAQ4plex)	HCD	1.0000	0.0000	1	1	12275	Unique	Used	0.110	9.045E3	1.248E3	91	7.50	0.00	0	17	190	2	1225.09485	2449.18242	-0.50	59.76	7114	7114	MS2	27/36	Glioma_Nuclear_prep_Gradeall_4plex.raw
51477708	High		eYFGGFGEVESIELPMDNk	Unambiguous	4	1	N-Term(iTRAQ4plex); K19(iTRAQ4plex)	HCD	1.0000	0.0000	1	1	12217	Unique	Used	0.145	1.126E4	2.049E3	95	6.58	0.00	0	6	13	3	817.06836	2449.19052	2.81	59.18	7051	7051	MS2	27/72	Glioma_Nuclear_prep_Gradeall_4plex.raw
52632383	NP_001524.2	HNRNPL	3191	heterogeneous nuclear ribonucleoprotein L isoform a [Homo sapiens]	23.43	2	9	17	0.195	8;9	38.4	71.74	589	64.1	8.22																			
52632383		High	yYGGGSEGGR	Unambiguous	1	1	N-Term(iTRAQ4plex)	HCD	1.0000	0.0000	1	1	7031	Unique	Used	0.132	7.232E4	1.290E4	49	2.85	0.00	0	33	165	2	573.76556	1146.52385	-5.05	15.07	1300	1300	MS2	17/18	Glioma_Nuclear_prep_Gradeall_repeat_4plex.raw
52632383		High	tPASPVVHIR	Unambiguous	1	1	N-Term(iTRAQ4plex)	HCD	1.0000	0.0000	1	1	7922	Unique	Used	0.276	2.526E4	9.435E3	236	5.17	0.00	0	25	41	3	407.57953	1220.72403	0.67	23.03	2244	2244	MS2	22/36	Glioma_Nuclear_prep_Gradeall_repeat_4plex.raw
52632383		High	lcFSTAQHAS	Unambiguous	2	1	N-Term(iTRAQ4plex); C2(Methylthio)	HCD	1.0000	0.0000	1	1	9527	Unique	Used	0.345	2.332E4	1.091E4	47	2.94	0.00	0	18	105	2	627.78662	1254.56597	-5.43	39.58	3944	3944	MS2	11/18	Glioma_Nuclear_prep_Gradeall_repeat_4plex.raw
52632383	High		nPNGPYPYTLk	Unambiguous	2	1	N-Term(iTRAQ4plex); K11(iTRAQ4plex)	HCD	1.0000	0.0000	1	1	8747	Unique	Used	0.099	3.541E5	4.387E4	247	3.82	0.00	0	13	10	2	776.41351	1551.81975	-13.67	31.70	3264	3264	MS2	16/20	Glioma_Nuclear_prep_Gradeall_4plex.raw
52632383	High		sSSGLLEWESk	Unambiguous	2	1	N-Term(iTRAQ4plex); K11(iTRAQ4plex)	HCD	1.0000	0.0000	1	1	9558	Unique	Used	0.252	2.406E5	7.616E4	176	4.49	0.00	0	37	15	2	755.89264	1510.77800	-14.02	37.67	4150	4150	MS2	20/20	Glioma_Nuclear_prep_Gradeall_4plex.raw
52632383		High	sSSGLLEWESk	Unambiguous	2	1	N-Term(iTRAQ4plex); K11(iTRAQ4plex)	HCD	1.0000	0.0000	1	1	10232	Unique	Used	0.195	1.507E4	3.979E3	218	4.74	0.00	0	31	300	2	755.90564	1510.80400	3.19	47.83	4702	4702	MS2	19/20	Glioma_Nuclear_prep_Gradeall_repeat_4plex.raw
52632383	High		aITHLNNNFMFGQk	Unambiguous	2	1	N-Term(iTRAQ4plex); K14(iTRAQ4plex)	HCD	1.0000	0.0000	1	1	9576	Unique	Used	0.152	1.414E5	2.704E4	193	6.04	0.00	0	0	17	2	961.99634	1922.98540	-15.38	37.79	4169	4169	MS2	22/26	Glioma_Nuclear_prep_Gradeall_4plex.raw
52632383	High		aITHLNNNFMFGQk	Unambiguous	2	1	N-Term(iTRAQ4plex); K14(iTRAQ4plex)	HCD	1.0000	0.0000	1	1	9580	Unique	Used	0.194	1.433E5	3.496E4	165	5.71	0.00	0	16	3	3	641.66718	1922.98697	-14.56	37.83	4174	4174	MS2	25/52	Glioma_Nuclear_prep_Gradeall_4plex.raw
52632383		High	aITHLNNNFMFGQk	Unambiguous	2	1	N-Term(iTRAQ4plex); K14(iTRAQ4plex)	HCD	1.0000	0.0000	1	1	10394	Unique	Used	0.197	2.340E3	6.237E2	185	6.43	0.00	0	27	300	3	641.67810	1923.01975	2.49	49.76	4875	4875	MS2	23/52	Glioma_Nuclear_prep_Gradeall_repeat_4plex.raw
52632383	High		sDALETLGFLNHYQMk	Unambiguous	2	1	N-Term(iTRAQ4plex); K16(iTRAQ4plex)	HCD	1.0000	0.0000	1	1	11181	Unique	Used	0.143	4.449E4	8.001E3	114	6.40	0.00	0	23	16	3	719.04059	2155.10721	-1.13	49.89	5919	5919	MS2	34/60	Glioma_Nuclear_prep_Gradeall_4plex.raw
52632383	High		sDALETLGFLNHYQMk	Unambiguous	2	1	N-Term(iTRAQ4plex); K16(iTRAQ4plex)	HCD	1.0000	0.0000	1	1	11282	Unique	Used	0.209	2.215E4	5.810E3	110	6.96	0.00	0	13	26	3	719.04144	2155.10978	0.05	50.71	6029	6029	MS2	34/60	Glioma_Nuclear_prep_Gradeall_4plex.raw
52632383	High		skPGAAMVEMADGYAVDR	Unambiguous	2	1	N-Term(iTRAQ4plex); K2(iTRAQ4plex)	HCD	1.0000	0.0000	1	1	9688	Unique	Used	0.302	3.432E4	1.304E4	99	4.99	0.00	0	14	6	3	719.35187	2156.04105	-14.33	38.60	4291	4291	MS2	29/68	Glioma_Nuclear_prep_Gradeall_4plex.raw
52632383		High	skPGAAmVEmADGYAVDR	Unambiguous	2	1	N-Term(iTRAQ4plex); K2(iTRAQ4plex); M7(Oxidation); M10(Oxidation)	HCD	1.0000	0.0000	1	1	8458	Unique	Used	0.253	1.785E3	6.122E2	112	5.17	0.00	0	11	139	3	730.02649	2188.06491	1.44	28.19	2808	2808	MS2	28/68	Glioma_Nuclear_prep_Gradeall_repeat_4plex.raw
52632383	High		fSTPEQAAk	Unambiguous	2	1	N-Term(iTRAQ4plex); K9(iTRAQ4plex)	HCD	1.0000	0.0000	1	1	7711	Unique	Used	0.134	2.800E6	4.712E5	292	3.13	0.00	0	13	2	2	633.85144	1266.69560	1.85	24.32	2140	2140	MS2	15/16	Glioma_Nuclear_prep_Gradeall_4plex.raw
52632383		High	fSTPEQAAk	Unambiguous	2	1	N-Term(iTRAQ4plex); K9(iTRAQ4plex)	HCD	1.0000	0.0000	1	1	7828	Unique	Used	0.187	8.876E4	2.242E4	305	3.08	0.00	0	29	83	2	633.84991	1266.69255	-0.56	22.14	2145	2145	MS2	14/16	Glioma_Nuclear_prep_Gradeall_repeat_4plex.raw
52632383		High	aITHLNNNFmFGQk	Unambiguous	2	1	N-Term(iTRAQ4plex); M10(Oxidation); K14(iTRAQ4plex)	HCD	1.0000	0.0000	1	1	9826	Unique	Used	0.342	2.284E3	1.058E3	191	5.68	0.00	0	16	268	3	647.00995	1939.01529	2.79	42.94	4262	4262	MS2	24/52	Glioma_Nuclear_prep_Gradeall_repeat_4plex.raw
52632383		High	sDALETLGFLNHYQmk	Unambiguous	2	1	N-Term(iTRAQ4plex); M15(Oxidation); K16(iTRAQ4plex)	HCD	1.0000	0.0000	1	1	11702	Unique	Used	0.089	2.139E3	2.586E2	129	5.56	0.00	0	16	274	3	724.37488	2171.11008	2.54	67.01	6372	6372	MS2	29/60	Glioma_Nuclear_prep_Gradeall_repeat_4plex.raw
54114974	NP_444292.1	GNG2	54331	guanine nucleotide-binding protein G(I)/G(S)/G(O) subunit gamma-2 precursor [Homo sapiens]	42.25	1	2	3	0.425	2;1	11.1	9.90	71	7.8	7.99																			
54114974	High		eDPLLTPVPASENPFR	Unambiguous	1	1	N-Term(iTRAQ4plex)	HCD	1.0000	0.0000	1	1	10942	Unique	Used	0.418	6.697E4	3.517E4	187	3.95	0.00	0	8	29	2	963.49377	1925.98027	-14.91	48.00	5658	5658	MS2	19/30	Glioma_Nuclear_prep_Gradeall_4plex.raw
54114974		High	eDPLLTPVPASENPFR	Unambiguous	1	1	N-Term(iTRAQ4plex)	HCD	1.0000	0.0000	1	1	11726	Unique	Used	0.425	1.544E3	8.896E2	181	3.92	0.00	0	21	300	2	963.50879	1926.01030	0.68	67.32	6399	6399	MS2	20/30	Glioma_Nuclear_prep_Gradeall_repeat_4plex.raw
54114974	High		aAADLMAYcEAHAk	Unambiguous	1	1	N-Term(iTRAQ4plex); C9(Methylthio); K14(iTRAQ4plex)	HCD	1.0000	0.0000	1	1	11330	Unique	Used	0.508	1.017E4	6.500E3	79	5.96	0.00	0	27	47	3	600.28876	1798.85172	-0.67	51.12	6081	6081	MS2	36/52	Glioma_Nuclear_prep_Gradeall_4plex.raw
55956788	NP_005372.2	NCL	4691	nucleolin [Homo sapiens]	28.59	1	14	29	0.614	11;18	15.8	85.90	710	76.6	4.70																			
55956788	High		eVFEDAAEIR	Unambiguous	1	1	N-Term(iTRAQ4plex)	HCD	1.0000	0.0000	1	1	9356	Unique	Used	0.472	2.009E5	1.192E5	176	3.49	0.00	0	22	12	2	661.82831	1322.64934	-16.32	36.19	3930	3930	MS2	16/18	Glioma_Nuclear_prep_Gradeall_4plex.raw
55956788		High	eVFEDAAEIR	Unambiguous	1	1	N-Term(iTRAQ4plex)	HCD	1.0000	0.0000	1	1	9973	Unique	Used	0.493	1.312E4	8.766E3	212	3.95	0.00	0	12	276	2	661.84021	1322.67314	1.68	44.73	4423	4423	MS2	17/18	Glioma_Nuclear_prep_Gradeall_repeat_4plex.raw
55956788		High	eVFEDAAEIR	Unambiguous	1	1	N-Term(iTRAQ4plex)	HCD	1.0000	0.0000	1	1	9891	Unique	Used	0.600	1.180E4	9.601E3	212	4.03	0.00	0	4	300	2	661.84033	1322.67339	1.86	43.67	4331	4331	MS2	17/18	Glioma_Nuclear_prep_Gradeall_repeat_4plex.raw
55956788		High	eALNScNk	Unambiguous	1	1	N-Term(iTRAQ4plex); C6(Methylthio); K8(iTRAQ4plex)	HCD	1.0000	0.0000	1	1	7863	Unique	Used	0.583	3.963E4	3.128E4	92	3.28	0.00	0	30	87	2	606.79822	1212.58916	-5.25	22.49	2182	2182	MS2	14/14	Glioma_Nuclear_prep_Gradeall_repeat_4plex.raw
55956788	High		kFGYVDFESAEDLEk	Unambiguous	1	1	N-Term(iTRAQ4plex); K1(iTRAQ4plex); K15(iTRAQ4plex)	HCD	1.0000	0.0000	1	1	10212	Unique	Used	0.667	2.018E4	1.692E4	159	6.72	0.00	1	19	11	3	737.04047	2209.10685	-14.53	42.44	4861	4861	MS2	26/56	Glioma_Nuclear_prep_Gradeall_4plex.raw
55956788		High	kVVVSPTk	Unambiguous	1	1	N-Term(iTRAQ4plex); K1(iTRAQ4plex); K8(iTRAQ4plex)	HCD	1.0000	0.0000	1	1	7165	Unique	Used	0.770	1.179E4	1.230E4	73	3.59	0.00	1	60	57	3	430.62009	1289.84571	-4.65	16.26	1443	1443	MS2	17/28	Glioma_Nuclear_prep_Gradeall_repeat_4plex.raw
55956788	High		sISLYYTGEk	Unambiguous	1	1	N-Term(iTRAQ4plex); K10(iTRAQ4plex)	HCD	1.0000	0.0000	1	1	9216	Unique	Used	0.616	1.534E5	1.189E5	185	3.30	0.00	0	28	9	2	724.88580	1448.76433	-16.01	35.12	3775	3775	MS2	15/18	Glioma_Nuclear_prep_Gradeall_4plex.raw
55956788		High	sISLYYTGEk	Unambiguous	1	1	N-Term(iTRAQ4plex); K10(iTRAQ4plex)	HCD	1.0000	0.0000	1	1	9817	Unique	Used	0.661	4.711E4	4.215E4	268	4.08	0.00	0	57	82	2	724.88806	1448.76885	-12.89	42.86	4253	4253	MS2	15/18	Glioma_Nuclear_prep_Gradeall_repeat_4plex.raw
55956788		High	sISLYYTGEk	Unambiguous	1	1	N-Term(iTRAQ4plex); K10(iTRAQ4plex)	HCD	1.0000	0.0000	1	1	9773	Unique	Used	0.768	1.889E4	1.965E4	270	4.03	0.00	0	53	300	2	724.89935	1448.79143	2.69	42.36	4206	4206	MS2	15/18	Glioma_Nuclear_prep_Gradeall_repeat_4plex.raw
55956788	High		eAMEDGEIDGNk	Unambiguous	1	1	N-Term(iTRAQ4plex); K12(iTRAQ4plex)	HCD	1.0000	0.0000	1	1	7820	Unique	Used	0.578	2.202E5	1.600E5	53	4.99	0.00	0	5	6	2	798.37726	1595.74724	0.65	25.09	2258	2258	MS2	21/22	Glioma_Nuclear_prep_Gradeall_4plex.raw
55956788		High	eAMEDGEIDGNk	Unambiguous	1	1	N-Term(iTRAQ4plex); K12(iTRAQ4plex)	HCD	1.0000	0.0000	1	1	7924	Unique	Used	0.514	2.518E4	1.754E4	59	5.18	0.00	0	28	72	2	798.37744	1595.74761	0.88	23.05	2246	2246	MS2	21/22	Glioma_Nuclear_prep_Gradeall_repeat_4plex.raw
55956788		High	gLSEDTTEETLk	Unambiguous	1	1	N-Term(iTRAQ4plex); K12(iTRAQ4plex)	HCD	1.0000	0.0000	1	1	8605	Unique	Used	0.553	3.321E4	2.487E4	196	5.03	0.00	0	7	44	2	805.92334	1610.83940	1.88	29.70	2964	2964	MS2	22/22	Glioma_Nuclear_prep_Gradeall_repeat_4plex.raw
55956788	High		gFGFVDFNSEEDAk	Unambiguous	1	1	N-Term(iTRAQ4plex); K14(iTRAQ4plex)	HCD	1.0000	0.0000	1	1	10503	Unique	Used	0.614	2.278E5	1.759E5	53	5.10	0.00	0	29	10	2	925.43341	1849.85954	-13.62	44.67	5180	5180	MS2	22/26	Glioma_Nuclear_prep_Gradeall_4plex.raw
55956788	High		fGYVDFESAEDLEk	Unambiguous	1	1	N-Term(iTRAQ4plex); K14(iTRAQ4plex)	HCD	1.0000	0.0000	1	1	11013	Unique	Used	0.632	8.109E3	6.445E3	86	3.54	0.00	0	26	11	3	646.31702	1936.93650	-2.79	48.55	5735	5735	MS2	19/52	Glioma_Nuclear_prep_Gradeall_4plex.raw
55956788	High		fGYVDFESAEDLEk	Unambiguous	1	1	N-Term(iTRAQ4plex); K14(iTRAQ4plex)	HCD	1.0000	0.0000	1	1	10998	Unique	Used	0.754	3.923E4	3.718E4	71	5.45	0.00	0	29	59	2	968.95886	1936.91045	-16.24	48.43	5718	5718	MS2	22/26	Glioma_Nuclear_prep_Gradeall_4plex.raw
55956788		High	gFGFVDFNSEEDAk	Unambiguous	1	1	N-Term(iTRAQ4plex); K14(iTRAQ4plex)	HCD	1.0000	0.0000	1	1	11365	Unique	Used	0.454	2.220E3	1.366E3	96	4.94	0.00	0	40	300	2	925.44800	1849.88872	2.15	62.32	5971	5971	MS2	25/26	Glioma_Nuclear_prep_Gradeall_repeat_4plex.raw
55956788		High	gFGFVDFNSEEDAk	Unambiguous	1	1	N-Term(iTRAQ4plex); K14(iTRAQ4plex)	HCD	1.0000	0.0000	1	1	11297	Unique	Used	0.641	4.138E3	3.592E3	92	5.64	0.00	0	20	300	2	925.44861	1849.88994	2.81	61.20	5880	5880	MS2	23/26	Glioma_Nuclear_prep_Gradeall_repeat_4plex.raw
55956788		High	gFGFVDFNSEEDAk	Unambiguous	1	1	N-Term(iTRAQ4plex); K14(iTRAQ4plex)	HCD	1.0000	0.0000	1	1	11298	Unique	Used	0.716	7.097E2	6.879E2	96	4.03	0.00	0	58	300	3	617.30078	1849.88779	1.65	61.22	5881	5881	MS2	14/52	Glioma_Nuclear_prep_Gradeall_repeat_4plex.raw
55956788	High		vEGTEPTTAFNLFVGNLNFNk	Unambiguous	1	1	N-Term(iTRAQ4plex); K21(iTRAQ4plex)	HCD	1.0000	0.0000	1	1	12783	Unique	Used	0.443	9.664E2	5.381E2	125	7.06	0.00	0	0	300	2	1300.68652	2600.36577	2.23	66.12	7694	7694	MS2	28/40	Glioma_Nuclear_prep_Gradeall_4plex.raw
55956788	High		vEGTEPTTAFNLFVGNLNFNk	Unambiguous	1	1	N-Term(iTRAQ4plex); K21(iTRAQ4plex)	HCD	1.0000	0.0000	1	1	12755	Unique	Used	0.565	6.545E2	4.652E2	155	7.09	0.00	0	12	175	3	867.46100	2600.36844	3.26	65.69	7659	7659	MS2	26/80	Glioma_Nuclear_prep_Gradeall_4plex.raw
55956788		High	vTQDELk	Unambiguous	1	1	N-Term(iTRAQ4plex); K7(iTRAQ4plex)	HCD	1.0000	0.0000	1	1	7569	Unique	Used	0.638	1.085E5	9.372E4	413	2.91	0.00	0	36	33	2	560.82391	1120.64055	-4.18	19.69	1868	1868	MS2	12/12	Glioma_Nuclear_prep_Gradeall_repeat_4plex.raw
55956788	High		gIAYIEFk	Unambiguous	1	1	N-Term(iTRAQ4plex); K8(iTRAQ4plex)	HCD	1.0000	0.0000	1	1	9990	Unique	Used	0.564	8.338E5	5.911E5	225	3.47	0.00	0	34	2	2	614.85315	1228.69902	-15.45	40.78	4619	4619	MS2	14/14	Glioma_Nuclear_prep_Gradeall_4plex.raw
55956788		High	gIAYIEFk	Unambiguous	1	1	N-Term(iTRAQ4plex); K8(iTRAQ4plex)	HCD	1.0000	0.0000	1	1	10870	Unique	Used	0.586	7.436E3	5.905E3	207	3.59	0.00	0	28	300	2	614.86243	1228.71758	-0.35	55.56	5390	5390	MS2	14/14	Glioma_Nuclear_prep_Gradeall_repeat_4plex.raw
55956788		High	aLELTGLk	Unambiguous	1	1	N-Term(iTRAQ4plex); K8(iTRAQ4plex)	HCD	1.0000	0.0000	1	1	10056	Unique	Used	0.619	8.493E3	7.117E3	196	3.38	0.00	0	27	300	2	566.86285	1132.71843	0.37	45.76	4514	4514	MS2	14/14	Glioma_Nuclear_prep_Gradeall_repeat_4plex.raw
55956788		High	gIAYIEFk	Unambiguous	1	1	N-Term(iTRAQ4plex); K8(iTRAQ4plex)	HCD	1.0000	0.0000	1	1	10782	Unique	Used	0.739	5.235E3	5.238E3	198	3.40	0.00	0	55	300	2	614.86273	1228.71819	0.15	54.45	5293	5293	MS2	14/14	Glioma_Nuclear_prep_Gradeall_repeat_4plex.raw
55956788		High	aLELTGLk	Unambiguous	1	1	N-Term(iTRAQ4plex); K8(iTRAQ4plex)	HCD	1.0000	0.0000	1	1	9975	Unique	Used	0.775	9.656E3	1.013E4	192	3.34	0.00	0	50	300	2	566.86230	1132.71733	-0.60	44.76	4425	4425	MS2	14/14	Glioma_Nuclear_prep_Gradeall_repeat_4plex.raw
55956788		High	tGISDVFAk	Unambiguous	1	1	N-Term(iTRAQ4plex); K9(iTRAQ4plex)	HCD	1.0000	0.0000	1	1	9664	Unique	Used	0.525	3.294E4	2.343E4	268	3.71	0.00	0	16	105	2	613.35559	1225.70391	0.66	41.14	4092	4092	MS2	16/16	Glioma_Nuclear_prep_Gradeall_repeat_4plex.raw
55956788	High		eAmEDGEIDGNk	Unambiguous	1	1	N-Term(iTRAQ4plex); M3(Oxidation); K12(iTRAQ4plex)	HCD	1.0000	0.0000	1	1	7339	Unique	Used	0.717	4.171E4	3.759E4	33	3.96	0.00	0	0	35	2	806.37054	1611.73381	-4.53	21.65	1736	1736	MS2	19/22	Glioma_Nuclear_prep_Gradeall_4plex.raw
55956788		High	eAmEDGEIDGNk	Unambiguous	1	1	N-Term(iTRAQ4plex); M3(Oxidation); K12(iTRAQ4plex)	HCD	1.0000	0.0000	1	1	7229	Unique	Used	0.532	5.885E3	4.238E3	17	3.51	0.00	0	0	267	2	806.36133	1611.71538	-15.97	16.77	1510	1510	MS2	17/22	Glioma_Nuclear_prep_Gradeall_repeat_4plex.raw
55956921	NP_004490.2	HNRNPAB	3182	heterogeneous nuclear ribonucleoprotein A/B isoform b [Homo sapiens]	13.68	2	3	9	0.146	5;4	22.8	23.07	285	30.6	7.91																			

55956921	High		iFVGGLNPEATEEk	Unambiguous	2	1	N-Term(iTRAQ4plex); K14(iTRAQ4plex)	HCD	1.0000	0.0000	1	1	9873	Unique	Used	0.167	3.947E5	8.284E4	229	5.23	0.00	0	13	9	2	896.47772	1791.94817	-13.95	39.94	4492	4492	MS2	21/26	Glioma_Nuclear_prep_Gradeall_4plex.raw
55956921		High	iFVGGLNPEATEEk	Unambiguous	2	1	N-Term(iTRAQ4plex); K14(iTRAQ4plex)	HCD	1.0000	0.0000	1	1	10631	Unique	Used	0.090	2.652E4	3.240E3	220	5.94	0.00	0	6	166	2	896.49432	1791.98137	4.58	52.61	5130	5130	MS2	22/26	Glioma_Nuclear_prep_Gradeall_repeat_4plex.raw
55956921	High		gFGFILFk	Unambiguous	2	1	N-Term(iTRAQ4plex); K8(iTRAQ4plex)	HCD	1.0000	0.0000	1	1	12095	Unique	Used	0.134	9.805E4	1.647E4	163	3.48	0.00	0	26	48	2	608.87067	1216.73406	0.64	57.97	6917	6917	MS2	14/14	Glioma_Nuclear_prep_Gradeall_4plex.raw
55956921	High		gFVFITFk	Unambiguous	2	1	N-Term(iTRAQ4plex); K8(iTRAQ4plex)	HCD	1.0000	0.0000	1	1	11532	Unique	Used	0.143	3.146E5	5.676E4	241	3.26	0.00	0	21	15	2	623.87445	1246.74162	-1.77	52.86	6303	6303	MS2	13/14	Glioma_Nuclear_prep_Gradeall_4plex.raw
55956921	High		gFGFILFk	Unambiguous	2	1	N-Term(iTRAQ4plex); K8(iTRAQ4plex)	HCD	1.0000	0.0000	1	1	12045	Unique	Used	0.146	6.527E3	1.200E3	140	3.39	0.00	0	34	240	3	406.24963	1216.73435	0.88	57.43	6861	6861	MS2	13/28	Glioma_Nuclear_prep_Gradeall_4plex.raw
55956921	High		gFGFILFk	Unambiguous	2	1	N-Term(iTRAQ4plex); K8(iTRAQ4plex)	HCD	1.0000	0.0000	1	1	12023	Unique	Used	0.203	8.669E4	2.209E4	159	3.39	0.00	0	28	46	2	608.87036	1216.73345	0.14	57.22	6838	6838	MS2	14/14	Glioma_Nuclear_prep_Gradeall_4plex.raw
55956921		High	gFGFILFk	Unambiguous	2	1	N-Term(iTRAQ4plex); K8(iTRAQ4plex)	HCD	1.0000	0.0000	1	1	12414	Unique	Used	0.108	3.643E3	5.328E2	210	3.42	0.00	0	23	300	2	608.87067	1216.73406	0.64	84.38	7689	7689	MS2	14/14	Glioma_Nuclear_prep_Gradeall_repeat_4plex.raw
55956921		High	gFVFITFk	Unambiguous	2	1	N-Term(iTRAQ4plex); K8(iTRAQ4plex)	HCD	1.0000	0.0000	1	1	12237	Unique	Used	0.160	6.477E3	1.402E3	293	3.18	0.00	0	25	300	2	623.87701	1246.74675	2.34	77.14	7176	7176	MS2	13/14	Glioma_Nuclear_prep_Gradeall_repeat_4plex.raw
55956921		High	gFGFILFk	Unambiguous	2	1	N-Term(iTRAQ4plex); K8(iTRAQ4plex)	HCD	1.0000	0.0000	1	1	12397	Unique	Used	0.164	3.325E3	7.392E2	200	3.21	0.00	0	39	300	2	608.87091	1216.73454	1.04	83.35	7619	7619	MS2	14/14	Glioma_Nuclear_prep_Gradeall_repeat_4plex.raw
56118234	NP_859067.2	CENPV	201161	centromere protein V [Homo sapiens]	23.16	1	2	4	0.582	3;1	9.0	19.48	272	29.7	9.73																			
56118234	High		cGVQSFYTPR	Unambiguous	1	1	N-Term(iTRAQ4plex); C1(Methylthio)	HCD	1.0000	0.0000	1	1	9657	Unique	Used	0.609	1.615E5	1.238E5	55	2.91	0.00	0	37	17	2	674.30853	1347.60979	-15.46	38.37	4257	4257	MS2	17/18	Glioma_Nuclear_prep_Gradeall_4plex.raw
56118234		High	cGVQSFYTPR	Unambiguous	1	1	N-Term(iTRAQ4plex); C1(Methylthio)	HCD	1.0000	0.0000	1	1	10325	Unique	Used	0.573	1.193E4	9.249E3	70	3.58	0.00	0	46	300	2	674.32123	1347.63518	3.38	48.97	4802	4802	MS2	17/18	Glioma_Nuclear_prep_Gradeall_repeat_4plex.raw
56118234	High		ILLDTFEYQGLVk	Unambiguous	1	1	N-Term(iTRAQ4plex); K13(iTRAQ4plex)	HCD	1.0000	0.0000	1	1	12165	Unique	Used	0.494	4.415E3	2.741E3	113	4.26	0.00	0	49	115	3	609.68671	1827.04557	-2.79	58.62	6993	6993	MS2	21/48	Glioma_Nuclear_prep_Gradeall_4plex.raw
56118234	High		ILLDTFEYQGLVk	Unambiguous	1	1	N-Term(iTRAQ4plex); K13(iTRAQ4plex)	HCD	1.0000	0.0000	1	1	12177	Unique	Used	0.592	1.568E4	1.168E4	132	5.71	0.00	0	65	300	2	914.03717	1827.06706	8.98	58.74	7006	7006	MS2	23/24	Glioma_Nuclear_prep_Gradeall_4plex.raw
56699409	NP_002130.2	RBMX	27316	RNA-binding motif protein, X chromosome isoform 1 [Homo sapiens]	22.51	4	3	13	0.341	5;8	22.4	86.27	391	42.3	10.05																			
56699409	High		vEQATkPSFESGR	Unambiguous	1	1	N-Term(iTRAQ4plex); K6(iTRAQ4plex)	HCD	1.0000	0.0000	1	1	7714	Unique	Used	0.237	3.331E5	9.931E4	149	4.58	0.00	0	8	5	2	862.46582	1723.92436	1.50	24.34	2143	2143	MS2	19/24	Glioma_Nuclear_prep_Gradeall_4plex.raw
56699409		High	vEQATkPSFESGR	Unambiguous	1	1	N-Term(iTRAQ4plex); K6(iTRAQ4plex)	HCD	1.0000	0.0000	1	1	7861	Unique	Used	0.381	4.195E4	2.165E4	172	4.13	0.00	0	21	48	2	862.46301	1723.91875	-1.76	22.48	2180	2180	MS2	19/24	Glioma_Nuclear_prep_Gradeall_repeat_4plex.raw
56699409	High		iVEVLLMk	Unambiguous	3	1	N-Term(iTRAQ4plex); K8(iTRAQ4plex)	HCD	1.0000	0.0000	1	1	11178	Unique	Used	0.334	4.873E5	2.048E5	94	3.60	0.00	0	22	10	2	616.89667	1232.78606	-2.46	49.86	5916	5916	MS2	13/14	Glioma_Nuclear_prep_Gradeall_4plex.raw
56699409	High		aLEAVFGk	Unambiguous	2	1	N-Term(iTRAQ4plex); K8(iTRAQ4plex)	HCD	1.0000	0.0000	1	1	9651	Unique	Used	0.388	3.596E5	1.756E5	267	3.12	0.00	0	60	6	2	561.83270	1122.65813	-16.06	38.34	4251	4251	MS2	13/14	Glioma_Nuclear_prep_Gradeall_4plex.raw
56699409		High	iVEVLLMk	Unambiguous	3	1	N-Term(iTRAQ4plex); K8(iTRAQ4plex)	HCD	1.0000	0.0000	1	1	12079	Unique	Used	0.273	1.210E4	4.478E3	92	3.60	0.00	0	30	300	2	616.89844	1232.78960	0.41	73.88	6921	6921	MS2	13/14	Glioma_Nuclear_prep_Gradeall_repeat_4plex.raw
56699409		High	iVEVLLMk	Unambiguous	3	1	N-Term(iTRAQ4plex); K8(iTRAQ4plex)	HCD	1.0000	0.0000	1	1	12048	Unique	Used	0.299	9.314E3	3.769E3	93	3.63	0.00	0	54	300	2	616.89868	1232.79009	0.81	72.88	6848	6848	MS2	13/14	Glioma_Nuclear_prep_Gradeall_repeat_4plex.raw
56699409		High	iVEVLLMk	Unambiguous	3	1	N-Term(iTRAQ4plex); K8(iTRAQ4plex)	HCD	1.0000	0.0000	1	1	12144	Unique	Used	0.304	4.155E3	1.709E3	102	3.27	0.00	0	63	300	2	616.89777	1232.78826	-0.67	75.02	7013	7013	MS2	13/14	Glioma_Nuclear_prep_Gradeall_repeat_4plex.raw
56699409		High	aLEAVFGk	Unambiguous	2	1	N-Term(iTRAQ4plex); K8(iTRAQ4plex)	HCD	1.0000	0.0000	1	1	10349	Unique	Used	0.341	2.616E4	1.209E4	212	3.63	0.00	0	18	191	2	561.84314	1122.67900	2.53	49.25	4828	4828	MS2	14/14	Glioma_Nuclear_prep_Gradeall_repeat_4plex.raw
56699409	High		iVEVLLmk	Unambiguous	3	1	N-Term(iTRAQ4plex); M7(Oxidation); K8(iTRAQ4plex)	HCD	1.0000	0.0000	1	1	10598	Unique	Used	0.476	1.304E5	7.813E4	265	2.99	0.00	0	45	21	2	624.88641	1248.76555	-14.78	45.37	5283	5283	MS2	13/14	Glioma_Nuclear_prep_Gradeall_4plex.raw
56699409	High		iVEVLLmk	Unambiguous	3	1	N-Term(iTRAQ4plex); M7(Oxidation); K8(iTRAQ4plex)	HCD	1.0000	0.0000	1	1	10685	Unique	Used	0.488	8.019E5	4.926E5	232	3.01	0.00	0	43	4	2	624.88745	1248.76763	-13.11	46.05	5379	5379	MS2	13/14	Glioma_Nuclear_prep_Gradeall_4plex.raw
56699409		High	iVEVLLmk	Unambiguous	3	1	N-Term(iTRAQ4plex); M7(Oxidation); K8(iTRAQ4plex)	HCD	1.0000	0.0000	1	1	11572	Unique	Used	0.297	2.819E4	1.134E4	191	3.46	0.00	0	12	142	2	624.89746	1248.78765	2.92	65.18	6217	6217	MS2	13/14	Glioma_Nuclear_prep_Gradeall_repeat_4plex.raw
56699409		High	iVEVLLmk	Unambiguous	3	1	N-Term(iTRAQ4plex); M7(Oxidation); K8(iTRAQ4plex)	HCD	1.0000	0.0000	1	1	11504	Unique	Used	0.431	7.695E3	4.489E3	197	3.54	0.00	0	50	300	2	624.89648	1248.78569	1.35	64.19	6133	6133	MS2	13/14	Glioma_Nuclear_prep_Gradeall_repeat_4plex.raw
56699409		High	iVEVLLmk	Unambiguous	3	1	N-Term(iTRAQ4plex); M7(Oxidation); K8(iTRAQ4plex)	HCD	1.0000	0.0000	1	1	11700	Unique	Used	0.446	4.190E3	2.534E3	191	3.33	0.00	0	47	300	2	624.89703	1248.78679	2.23	66.98	6369	6369	MS2	13/14	Glioma_Nuclear_prep_Gradeall_repeat_4plex.raw
60279268	NP_001012496.1	U2AF2	11338	splicing factor U2AF 65 kDa subunit isoform b [Homo sapiens]	11.04	2	3	6	1.662	3;3	14.5	21.99	471	53.1	9.09																			
60279268	High		sIEIPRPVDGVEVPGcGk	Unambiguous	2	1	N-Term(iTRAQ4plex); C16(Methylthio); K18(iTRAQ4plex)	HCD	1.0000	0.0000	1	1	10534	Unique	Used	1.901	2.537E4	6.067E4	108	3.58	0.00	0	25	9	3	729.37988	2186.12510	-13.80	44.91	5214	5214	MS2	26/68	Glioma_Nuclear_prep_Gradeall_4plex.raw
60279268		High	sIEIPRPVDGVEVPGcGk	Unambiguous	2	1	N-Term(iTRAQ4plex); C16(Methylthio); K18(iTRAQ4plex)	HCD	1.0000	0.0000	1	1	11420	Unique	Used	1.645	6.015E2	1.341E3	101	3.62	0.00	0	17	300	3	729.39093	2186.15824	1.36	63.08	6036	6036	MS2	27/68	Glioma_Nuclear_prep_Gradeall_repeat_4plex.raw
60279268		High	eLLTSFGPLk	Unambiguous	2	1	N-Term(iTRAQ4plex); K10(iTRAQ4plex)	HCD	1.0000	0.0000	1	1	11544	Unique	Used	1.312	4.117E3	7.316E3	269	3.94	0.00	0	49	300	2	696.92059	1392.83391	-0.15	64.74	6180	6180	MS2	17/18	Glioma_Nuclear_prep_Gradeall_repeat_4plex.raw
60279268	High		IFIGGLPNYLNDDQVk	Unambiguous	2	1	N-Term(iTRAQ4plex); K16(iTRAQ4plex)	HCD	1.0000	0.0000	1	1	11871	Unique	Used	1.571	5.624E3	1.111E4	245	5.82	0.00	0	38	51	3	698.72040	2094.14664	-0.38	55.81	6671	6671	MS2	20/60	Glioma_Nuclear_prep_Gradeall_4plex.raw
60279268	High		IFIGGLPNYLNDDQVk	Unambiguous	2	1	N-Term(iTRAQ4plex); K16(iTRAQ4plex)	HCD	1.0000	0.0000	1	1	11895	Unique	Used	1.680	2.822E4	5.960E4	208	5.96	0.00	0	9	63	2	1047.58215	2094.15703	4.59	56.00	6697	6697	MS2	25/30	Glioma_Nuclear_prep_Gradeall_4plex.raw
60279268		High	IFIGGLPNYLNDDQVk	Unambiguous	2	1	N-Term(iTRAQ4plex); K16(iTRAQ4plex)	HCD	1.0000	0.0000	1	1	12330	Unique	Used	2.001	1.630E2	4.416E2	228	3.81	0.00	0	25	300	3	698.72137	2094.14957	1.02	79.90	7380	7380	MS2	22/60	Glioma_Nuclear_prep_Gradeall_repeat_4plex.raw
62414289	NP_003371.2	VIM	7431	vimentin [Homo sapiens]	29.40	2	9	14	3.373	10;4	39.1	44.10	466	53.6	5.12																			
62414289	High		iSLPLPNFSSLNLR	Unambiguous	1	1	N-Term(iTRAQ4plex)	HCD	1.0000	0.0000	1	1	12623	Unique	Used	1.352	3.376E3	5.740E3	169	4.19	0.00	0	39	300	2	858.00201	1714.99675	-0.31	64.03	7511	7511	MS2	17/26	Glioma_Nuclear_prep_Gradeall_4plex.raw
62414289	High		eEAENTLQSFR	Unambiguous	1	1	N-Term(iTRAQ4plex)	HCD	1.0000	0.0000	1	1	8800	Unique	Used	3.272	3.024E4	1.245E5	112	3.57	0.00	0	28	33	2	734.35138	1467.69548	-16.47	32.09	3322	3322	MS2	18/20	Glioma_Nuclear_prep_Gradeall_4plex.raw
62414289	High		fADLSEAANR	Unambiguous	1	1	N-Term(iTRAQ4plex)	HCD	1.0000	0.0000	1	1	8713	Unique	Used	3.506	7.018E4	3.094E5	158	3.06	0.00	0	35	23	2	619.30750	1237.60771	-17.51	31.46	3227	3227	MS2	17/18	Glioma_Nuclear_prep_Gradeall_4plex.raw
62414289		High	fADLSEAANR	Unambiguous	1	1	N-Term(iTRAQ4plex)	HCD	1.0000	0.0000	1	1	9085	Unique	Used	2.982	4.985E3	2.013E4	246	3.36	0.00	0	43	300	2	619.31927	1237.63127	1.53	34.70	3473	3473	MS2	16/18	Glioma_Nuclear_prep_Gradeall_repeat_4plex.raw
62414289		High	vELQELNDR	Unambiguous	2	1	N-Term(iTRAQ4plex)	HCD	1.0000	0.0000	1	1	9214	Unique	Used	3.777	5.458E3	2.792E4	250	3.56	0.00	0	17	232	2	630.33990	1259.67253	1.02	36.11	3609	3609	MS2	15/16	Glioma_Nuclear_prep_Gradeall_repeat_4plex.raw
62414289		High	eEAENTLQSFR	Unambiguous	1	1	N-Term(iTRAQ4plex)	HCD	1.0000	0.0000	1	1	9221	Unique	Used	4.012	2.889E3	1.570E4	150	4.36	0.00	0	0	300	2	734.36353	1467.71977	0.08	36.19	3616	3616	MS2	19/20	Glioma_Nuclear_prep_Gradeall_repeat_4plex.raw
62414289	High		qVQSLTcEVDALk	Unambiguous	1	1	N-Term(iTRAQ4plex); C7(Methylthio); K13(iTRAQ4plex)	HCD	1.0000	0.0000	1	1	10588	Unique	Used	3.473	1.914E4	8.360E4	207	5.54	0.00	0	23	60	2	884.45343	1767.89958	-12.89	45.29	5272	5272	MS2	23/24	Glioma_Nuclear_prep_Gradeall_4plex.raw
62414289	High		kVESLQEEIAFLk	Unambiguous	1	1	N-Term(iTRAQ4plex); K1(iTRAQ4plex); K13(iTRAQ4plex)	HCD	1.0000	0.0000	1	1	10677	Unique	Used	2.280	4.392E3	1.260E4	111	5.38	0.00	1	31	24	3	656.04803	1966.12955	-14.75	45.98	5370	5370	MS2	24/48	Glioma_Nuclear_prep_Gradeall_4plex.raw
62414289	High		kVESLQEEIAFLk	Unambiguous	1	1	N-Term(iTRAQ4plex); K1(iTRAQ4plex); K13(iTRAQ4plex)	HCD	1.0000	0.0000	1	1	10707	Unique	Used	4.976	1.878E4	1.175E5	123	5.65	0.00	1	23	38	2	983.56842	1966.12956	-14.74	46.20	5402	5402	MS2	23/24	Glioma_Nuclear_prep_Gradeall_4plex.raw
62414289	High		iLLAELEQLk	Unambiguous	1	1	N-Term(iTRAQ4plex); K10(iTRAQ4plex)	HCD	1.0000	0.0000	1	1	12402	Unique	Used	3.514	1.546E4	6.834E4	74	4.12	0.00	0	49	270	2	729.46106	1457.91484	-2.29	61.16	7254	7254	MS2	17/18	Glioma_Nuclear_prep_Gradeall_4plex.raw
62414289	High		iLLAELEQLk	Unambiguous	1	1	N-Term(iTRAQ4plex); K10(iTRAQ4plex)	HCD	1.0000	0.0000	1	1	12401	Unique	Used	6.289	1.100E3	8.698E3	64	4.13	0.00	0	21	149	3	486.64212	1457.91181	-4.38	61.15	7253	7253	MS2	21/36	Glioma_Nuclear_prep_Gradeall_4plex.raw
62414289	High		eTNLDSLPLVDTHSk	Unambiguous	1	1	N-Term(iTRAQ4plex); K15(iTRAQ4plex)	HCD	1.0000	0.0000	1	1	9400	Unique	Used	2.108	1.542E4	4.088E4	145	5.51	0.00	0	18	7	3	653.01001	1957.01548	-16.67	36.51	3978	3978	MS2	27/56	Glioma_Nuclear_prep_Gradeall_4plex.raw
62414289		High	eTNLDSLPLVDTHSk	Unambiguous	1	1	N-Term(iTRAQ4plex); K15(iTRAQ4plex)	HCD	1.0000	0.0000	1	1	10060	Unique	Used	2.235	5.572E2	1.686E3	151	5.40	0.00	0	26	300	3	653.02161	1957.05027	1.11	45.80	4518	4518	MS2	26/56	Glioma_Nuclear_prep_Gradeall_repeat_4plex.raw
62414289	High		fANYIDk	Unambiguous	1	1	N-Term(iTRAQ4plex); K7(iTRAQ4plex)	HCD	1.0000	0.0000	1	1	8924	Unique	Used	2.149	1.108E5	2.994E5	193	2.97	0.00	0	48	14	2	579.81281	1158.61833	-18.48	32.97	3456	3456	MS2	12/12	Glioma_Nuclear_prep_Gradeall_4plex.raw
62750354	NP_954659.1	MATR3	9782	matrin-3 isoform a [Homo sapiens]	23.49	3	14	38	0.375	22;16	36.0	126.57	847	94.6	6.25																			
62750354	High		gPSLNPVLDYDHGSR	Unambiguous	1	1	N-Term(iTRAQ4plex)	HCD	1.0000	0.0000	1	1	9117	Unique	Used	0.261	1.315E5	4.317E4	92	4.47	0.00	0	11	17	2	885.93506	1770.86284	-14.88	34.42	3668	3668	MS2	22/28	Glioma_Nuclear_prep_Gradeall_4plex.raw
62750354	High		gDADQASNILASFGLSAR	Unambiguous	1	1	N-Term(iTRAQ4plex)	HCD	1.0000	0.0000	1	1	12207	Unique	Used	0.262	6.112E4	2.013E4	149	6.31	0.00	0	6	35	2	969.00220	1936.99712	6.48	59.07	7040	7040	MS2	27/34	Glioma_Nuclear_prep_Gradeall_4plex.raw
62750354	High		gDADQASNILASFGLSAR	Unambiguous	1	1	N-Term(iTRAQ4plex)	HCD	1.0000	0.0000	1	1	12139	Unique	Used	0.329	5.401E4	2.237E4	156	6.49	0.00	0	17	40	2	968.99481	1936.98235	-1.15	58.39	6965	6965	MS2	27/34	Glioma_Nuclear_prep_Gradeall_4plex.raw
62750354	High		tEEGPTLSYGR	Unambiguous	1	1	N-Term(iTRAQ4plex)	HCD	1.0000	0.0000	1	1	8049	Unique	Used	0.334	2.585E5	1.085E5	114	3.26	0.00	0	27	12	2	677.33185	1353.65642	-14.99	26.71	2506	2506	MS2	17/20	Glioma_Nuclear_prep_Gradeall_4plex.raw
62750354	High		gPSLNPVLDYDHGSR	Unambiguous	1	1	N-Term(iTRAQ4plex)	HCD	1.0000	0.0000	1	1	9104	Unique	Used	0.587	2.877E4	2.125E4	89	4.64	0.00	0	12	7	3	590.95776	1770.85874	-17.19	34.32	3654	3654	MS2	25/56	Glioma_Nuclear_prep_Gradeall_4plex.raw
62750354	High		dLSAAGIGLLAAATQSLSMPASLGR	Unambiguous	1	1	N-Term(iTRAQ4plex)	HCD	1.0000	0.0000	1	1	13133	Unique	Used	0.778	7.133E1	6.976E1	75	4.22	0.00	0	17	300	3	839.12964	2515.37436	2.86	76.38	8434	8434	MS2	26/96	Glioma_Nuclear_prep_Gradeall_4plex.raw
62750354		High	gPSLNPVLDYDHGSR	Unambiguous	1	1	N-Term(iTRAQ4plex)	HCD	1.0000	0.0000	1	1	9729	Unique	Used	0.186	7.678E3	1.932E3	110	4.99	0.00	0	38	300	2	885.95282	1770.89836	5.18	41.85	4160	4160	MS2	20/28	Glioma_Nuclear_prep_Gradeall_repeat_4plex.raw
62750354		High	tEEGPTLSYGR	Unambiguous	1	1	N-Term(iTRAQ4plex)	HCD	1.0000	0.0000	1	1	8234	Unique	Used	0.208	4.768E4	1.346E4	153	3.36	0.00	0	12	103	2	677.34473	1353.68218	4.04	26.07	2573	2573	MS2	16/20	Glioma_Nuclear_prep_Gradeall_repeat_4plex.raw
62750354		High	gDADQASNILASFGLSAR	Unambiguous	1	1	N-Term(iTRAQ4plex)	HCD	1.0000	0.0000	1	1	12393	Unique	Used	0.232	9.668E2	3.035E2	144	4.95	0.00	0	12	300	2	968.99695	1936.98662	1.06	83.02	7597	7597	MS2	26/34	Glioma_Nuclear_prep_Gradeall_repeat_4plex.raw
62750354		High	gDADQASNILASFGLSAR	Unambiguous	1	1	N-Term(iTRAQ4plex)	HCD	1.0000	0.0000	1	1	12392	Unique	Used	0.288	1.626E2	6.339E1	153	4.83	0.00	0	7	300	3	646.33386	1936.98703	1.27	82.92	7590	7590	MS2	25/68	Glioma_Nuclear_prep_Gradeall_repeat_4plex.raw
62750354		High	gPLPLSSQHR	Unambiguous	1	1	N-Term(iTRAQ4plex)	HCD	1.0000	0.0000	1	1	7537	Unique	Used	0.314	3.377E4	1.435E4	330	3.40	0.00	0	21	146	2	618.34619	1235.68511	-10.21	19.41	1834	1834	MS2	15/18	Glioma_Nuclear_prep_Gradeall_repeat_4plex.raw
62750354		High	gPSLNPVLDYDHGSR	Unambiguous	1	1	N-Term(iTRAQ4plex)	HCD	1.0000	0.0000	1	1	9722	Unique	Used	0.366	4.466E3	2.217E3	101	5.51	0.00	0	33	157	3	590.96924	1770.89316	2.25	41.77	4153	4153	MS2	23/56	Glioma_Nuclear_prep_Gradeall_repeat_4plex.raw
62750354		High	vVHIMDFQR	Unambiguous	3	1	N-Term(iTRAQ4plex)	HCD	1.0000	0.0000	1	1	9705	Unique	Used	0.403	4.724E3	2.577E3	158	3.93	0.00	0	20	269	3	430.23706	1288.69663	1.04	41.59	4135	4135	MS2	19/32	Glioma_Nuclear_prep_Gradeall_repeat_4plex.raw
62750354		High	rTEEGPTLSYGR	Unambiguous	1	1	N-Term(iTRAQ4plex)	HCD	1.0000	0.0000	1	1	7705	Unique	Used	0.495	3.286E3	2.204E3	175	3.32	0.00	1	0	300	2	755.38690	1509.76653	-7.49	20.98	2015	2015	MS2	17/22	Glioma_Nuclear_prep_Gradeall_repeat_4plex.raw
62750354		High	gPLPLSSQHR	Unambiguous	1	1	N-Term(iTRAQ4plex)	HCD	1.0000	0.0000	1	1	7557	Unique	Used	0.671	1.169E4	1.062E4	212	4.36	0.00	0	29	67	3	412.57187	1235.70105	2.69	19.58	1855	1855	MS2	19/36	Glioma_Nuclear_prep_Gradeall_repeat_4plex.raw
62750354	High		IcSLFYTNEEVAk	Unambiguous	3	1	N-Term(iTRAQ4plex); C2(Methylthio); K13(iTRAQ4plex)	HCD	1.0000	0.0000	1	1	11962	Unique	Used	0.341	4.576E4	1.961E4	163	5.69	0.00	0	26	106	2	925.96802	1850.92876	0.89	56.63	6771	6771	MS2	21/24	Glioma_Nuclear_prep_Gradeall_4plex.raw
62750354	High		IcSLFYTNEEVAk	Unambiguous	3	1	N-Term(iTRAQ4plex); C2(Methylthio); K13(iTRAQ4plex)	HCD	1.0000	0.0000	1	1	11880	Unique	Used	0.347	8.423E4	3.680E4	207	5.10	0.00	0	9	38	2	925.97003	1850.93279	3.07	55.89	6681	6681	MS2	21/24	Glioma_Nuclear_prep_Gradeall_4plex.raw
62750354	High		IcSLFYTNEEVAk	Unambiguous	3	1	N-Term(iTRAQ4plex); C2(Methylthio); K13(iTRAQ4plex)	HCD	1.0000	0.0000	1	1	11923	Unique	Used	0.541	1.616E4	1.099E4	165	4.62	0.00	0	22	15	3	617.65088	1850.93808	5.93	56.25	6728	6728	MS2	21/48	Glioma_Nuclear_prep_Gradeall_4plex.raw
62750354		High	IcSLFYTNEEVAk	Unambiguous	3	1	N-Term(iTRAQ4plex); C2(Methylthio); K13(iTRAQ4plex)	HCD	1.0000	0.0000	1	1	12360	Unique	Used	0.387	2.324E3	1.219E3	153	4.83	0.00	0	4	300	2	925.97003	1850.93279	3.07	80.88	7451	7451	MS2	22/24	Glioma_Nuclear_prep_Gradeall_repeat_4plex.raw
62750354		High	IcSLFYTNEEVAk	Unambiguous	3	1	N-Term(iTRAQ4plex); C2(Methylthio); K13(iTRAQ4plex)	HCD	1.0000	0.0000	1	1	12355	Unique	Used	0.602	2.720E2	2.219E2	136	3.54	0.00	0	17	300	3	617.64844	1850.93076	1.97	80.78	7443	7443	MS2	19/48	Glioma_Nuclear_prep_Gradeall_repeat_4plex.raw
62750354	High		nTHcSSLPHYQk	Unambiguous	3	1	N-Term(iTRAQ4plex); C4(Methylthio); K12(iTRAQ4plex)	HCD	1.0000	0.0000	1	1	7725	Unique	Used	0.505	5.466E4	3.473E4	66	3.50	0.00	0	43	3	4	437.96671	1748.84499	-0.05	24.42	2155	2155	MS2	25/66	Glioma_Nuclear_prep_Gradeall_4plex.raw
62750354	High		gYPHLcSIcDLPVHSNk	Unambiguous	1	1	N-Term(iTRAQ4plex); C6(Methylthio); C9(Methylthio); K17(iTRAQ4plex)	HCD	1.0000	0.0000	1	1	11152	Unique	Used	0.452	3.220E3	1.831E3	52	4.93	0.00	0	48	21	4	566.52124	2263.06313	-4.57	49.65	5887	5887	MS2	31/96	Glioma_Nuclear_prep_Gradeall_4plex.raw
62750354	High		iTPENLPQILLQLk	Unambiguous	1	1	N-Term(iTRAQ4plex); K14(iTRAQ4plex)	HCD	1.0000	0.0000	1	1	13021	Unique	Used	0.322	3.361E3	1.363E3	28	5.65	0.00	0	8	65	3	636.73273	1908.18363	3.33	70.30	8024	8024	MS2	24/52	Glioma_Nuclear_prep_Gradeall_4plex.raw
62750354	High		iTPENLPQILLQLk	Unambiguous	1	1	N-Term(iTRAQ4plex); K14(iTRAQ4plex)	HCD	1.0000	0.0000	1	1	12989	Unique	Used	0.339	1.076E4	4.591E3	29	5.56	0.00	0	23	300	2	954.59357	1908.17986	1.36	69.71	7978	7978	MS2	21/26	Glioma_Nuclear_prep_Gradeall_4plex.raw
62750354	High		iTPENLPQILLQLk	Unambiguous	1	1	N-Term(iTRAQ4plex); K14(iTRAQ4plex)	HCD	1.0000	0.0000	1	1	13024	Unique	Used	0.349	6.607E3	2.896E3	28	5.05	0.00	0	18	300	2	954.59485	1908.18242	2.70	70.38	8030	8030	MS2	22/26	Glioma_Nuclear_prep_Gradeall_4plex.raw
62750354	High		iTPENLPQILLQLk	Unambiguous	1	1	N-Term(iTRAQ4plex); K14(iTRAQ4plex)	HCD	1.0000	0.0000	1	1	12983	Unique	Used	0.510	9.530E2	6.117E2	27	5.62	0.00	0	48	300	3	636.73187	1908.18106	1.99	69.63	7971	7971	MS2	24/52	Glioma_Nuclear_prep_Gradeall_4plex.raw
62750354	High		gAPPSSNIEDFHGLLPk	Unambiguous	1	1	N-Term(iTRAQ4plex); K17(iTRAQ4plex)	HCD	1.0000	0.0000	1	1	9982	Unique	Used	0.446	6.898E4	3.873E4	179	4.63	0.00	0	23	20	2	1034.04333	2067.07939	-15.48	40.72	4610	4610	MS2	24/32	Glioma_Nuclear_prep_Gradeall_4plex.raw
62750354	High		gAPPSSNIEDFHGLLPk	Unambiguous	1	1	N-Term(iTRAQ4plex); K17(iTRAQ4plex)	HCD	1.0000	0.0000	1	1	10052	Unique	Used	0.496	6.404E4	3.992E4	114	4.09	0.00	0	7	4	3	689.69934	2067.08347	-13.51	41.23	4686	4686	MS2	26/64	Glioma_Nuclear_prep_Gradeall_4plex.raw
62750354	High		gAPPSSNIEDFHGLLPk	Unambiguous	1	1	N-Term(iTRAQ4plex); K17(iTRAQ4plex)	HCD	1.0000	0.0000	1	1	9954	Unique	Used	0.572	1.561E5	1.124E5	124	4.19	0.00	0	15	2	3	689.69824	2067.08017	-15.10	40.53	4580	4580	MS2	27/64	Glioma_Nuclear_prep_Gradeall_4plex.raw
62750354		High	gAPPSSNIEDFHGLLPk	Unambiguous	1	1	N-Term(iTRAQ4plex); K17(iTRAQ4plex)	HCD	1.0000	0.0000	1	1	10918	Unique	Used	0.307	2.030E3	8.443E2	177	5.56	0.00	0	0	300	2	1034.06226	2067.11724	2.83	56.16	5443	5443	MS2	23/32	Glioma_Nuclear_prep_Gradeall_repeat_4plex.raw
62750354		High	gAPPSSNIEDFHGLLPk	Unambiguous	1	1	N-Term(iTRAQ4plex); K17(iTRAQ4plex)	HCD	1.0000	0.0000	1	1	10954	Unique	Used	0.318	2.821E3	1.215E3	136	4.68	0.00	0	6	119	3	689.71002	2067.11551	2.00	56.59	5482	5482	MS2	28/64	Glioma_Nuclear_prep_Gradeall_repeat_4plex.raw
62750354		High	gAPPSSNIEDFHGLLPk	Unambiguous	1	1	N-Term(iTRAQ4plex); K17(iTRAQ4plex)	HCD	1.0000	0.0000	1	1	10852	Unique	Used	0.547	1.148E3	8.495E2	133	4.50	0.00	0	43	300	3	689.70953	2067.11405	1.29	55.33	5370	5370	MS2	23/64	Glioma_Nuclear_prep_Gradeall_repeat_4plex.raw
62750354	High		iGPYQPNVPVGIDYVIPk	Unambiguous	3	1	N-Term(iTRAQ4plex); K18(iTRAQ4plex)	HCD	1.0000	0.0000	1	1	11508	Unique	Used	0.324	6.943E4	2.830E4	91	5.95	0.00	0	3	17	2	1129.14001	2257.27275	-4.77	52.67	6277	6277	MS2	22/34	Glioma_Nuclear_prep_Gradeall_4plex.raw
62750354	High		iGPYQPNVPVGIDYVIPk	Unambiguous	3	1	N-Term(iTRAQ4plex); K18(iTRAQ4plex)	HCD	1.0000	0.0000	1	1	11498	Unique	Used	0.476	1.026E4	6.139E3	104	4.44	0.00	0	27	13	3	753.09662	2257.27530	-3.64	52.59	6266	6266	MS2	19/68	Glioma_Nuclear_prep_Gradeall_4plex.raw
62750354		High	iGPYQPNVPVGIDYVIPk	Unambiguous	3	1	N-Term(iTRAQ4plex); K18(iTRAQ4plex)	HCD	1.0000	0.0000	1	1	12161	Unique	Used	0.369	7.368E2	3.687E2	71	4.17	0.00	0	2	300	2	1129.14612	2257.28496	0.63	75.27	7034	7034	MS2	18/34	Glioma_Nuclear_prep_Gradeall_repeat_4plex.raw
62750354		High	iGPYQPNVPVGIDYVIPk	Unambiguous	3	1	N-Term(iTRAQ4plex); K18(iTRAQ4plex)	HCD	1.0000	0.0000	1	1	12154	Unique	Used	0.750	2.474E2	2.512E2	106	5.07	0.00	0	26	271	3	753.10120	2257.28904	2.44	75.16	7025	7025	MS2	20/68	Glioma_Nuclear_prep_Gradeall_repeat_4plex.raw
62750354	High		vIHLSNLPHSGYSDSAVLk	Unambiguous	3	1	N-Term(iTRAQ4plex); K19(iTRAQ4plex)	HCD	1.0000	0.0000	1	1	9461	Unique	Used	0.381	3.877E4	1.856E4	143	6.44	0.00	0	13	4	3	775.75354	2325.24607	-14.83	36.97	4045	4045	MS2	36/72	Glioma_Nuclear_prep_Gradeall_4plex.raw
62750354	High		dLSAAGIGLLAAATQSLSmPASLGR	Unambiguous	1	1	N-Term(iTRAQ4plex); M19(Oxidation)	HCD	1.0000	0.0000	1	1	13081	Unique	Used	0.764	7.120E1	6.844E1	135	6.67	0.00	0	18	300	3	844.46161	2531.37027	3.23	72.20	8159	8159	MS2	31/96	Glioma_Nuclear_prep_Gradeall_4plex.raw
63025214	NP_001017980.1	VMA21	203547	vacuolar ATPase assembly integral membrane protein VMA21 [Homo sapiens]	11.88	1	1	2	0.549	1;1	9.6	4.67	101	11.3	7.24																			
63025214	High		aALNALQPPEFR	Unambiguous	1	1	N-Term(iTRAQ4plex)	HCD	1.0000	0.0000	1	1	10098	Unique	Used	0.586	1.516E5	1.118E5	275	4.34	0.00	0	32	12	2	735.90161	1470.79595	-15.40	41.57	4736	4736	MS2	17/22	Glioma_Nuclear_prep_Gradeall_4plex.raw
63025214		High	aALNALQPPEFR	Unambiguous	1	1	N-Term(iTRAQ4plex)	HCD	1.0000	0.0000	1	1	10837	Unique	Used	0.512	5.212E3	3.612E3	261	4.67	0.00	0	38	300	2	735.91443	1470.82158	2.03	55.15	5354	5354	MS2	17/22	Glioma_Nuclear_prep_Gradeall_repeat_4plex.raw
66346730	NP_001015001.1	CKMT1A	548596	creatine kinase U-type, mitochondrial precursor [Homo sapiens]	18.71	2	5	7	0.458	3;4	35.1	25.84	417	47.0	8.34																			
66346730	High		gTGGVDTAATGGVFDISNLDR	Unambiguous	1	1	N-Term(iTRAQ4plex)	HCD	1.0000	0.0000	1	1	10864	Unique	Used	0.215	4.993E4	1.352E4	139	7.51	0.00	0	12	33	2	1084.02380	2167.04033	-15.94	47.40	5573	5573	MS2	31/40	Glioma_Nuclear_prep_Gradeall_4plex.raw
66346730	High		gTGGVDTAATGGVFDISNLDR	Unambiguous	1	1	N-Term(iTRAQ4plex)	HCD	1.0000	0.0000	1	1	10872	Unique	Used	0.461	2.446E4	1.417E4	116	5.41	0.00	0	38	18	3	723.02075	2167.04770	-12.53	47.47	5582	5582	MS2	25/80	Glioma_Nuclear_prep_Gradeall_4plex.raw
66346730		High	lYPPSAEYPDLR	Unambiguous	1	1	N-Term(iTRAQ4plex)	HCD	1.0000	0.0000	1	1	10599	Unique	Used	0.285	1.135E4	4.388E3	259	3.14	0.00	0	28	300	2	782.91235	1564.81743	2.95	52.21	5095	5095	MS2	15/22	Glioma_Nuclear_prep_Gradeall_repeat_4plex.raw
66346730		High	gLSLPPAcTR	Unambiguous	2	1	N-Term(iTRAQ4plex); C8(Methylthio)	HCD	1.0000	0.0000	1	1	10882	Unique	Used	0.458	6.321E3	3.923E3	254	2.87	0.00	0	54	300	2	602.81946	1204.63164	1.43	55.70	5403	5403	MS2	13/18	Glioma_Nuclear_prep_Gradeall_repeat_4plex.raw
66346730	High		vVVDALSGLk	Unambiguous	1	1	N-Term(iTRAQ4plex); K10(iTRAQ4plex)	HCD	1.0000	0.0000	1	1	10306	Unique	Used	0.477	5.423E5	3.253E5	193	3.86	0.00	0	50	8	2	644.90204	1288.79680	-8.62	43.13	4963	4963	MS2	16/18	Glioma_Nuclear_prep_Gradeall_4plex.raw
66346730		High	vVVDALSGLk	Unambiguous	1	1	N-Term(iTRAQ4plex); K10(iTRAQ4plex)	HCD	1.0000	0.0000	1	1	11107	Unique	Used	0.267	1.065E4	3.854E3	128	4.30	0.00	0	47	300	2	644.90942	1288.81157	2.84	58.53	5652	5652	MS2	18/18	Glioma_Nuclear_prep_Gradeall_repeat_4plex.raw
66346730		High	iPTPVIHTk	Unambiguous	1	1	N-Term(iTRAQ4plex); K9(iTRAQ4plex)	HCD	1.0000	0.0000	1	1	8696	Unique	Used	0.592	1.360E4	1.090E4	88	4.00	0.00	0	60	116	3	431.94684	1293.82596	9.78	30.60	3059	3059	MS2	17/32	Glioma_Nuclear_prep_Gradeall_repeat_4plex.raw
66529396	NP_001018171.1	PON2	5445	serum paraoxonase/arylesterase 2 isoform 2 [Homo sapiens]	14.91	2	4	11	2.579	5;6	29.6	26.20	342	38.0	5.72																			
66529396	High		lLIGTLYHR	Unambiguous	2	1	N-Term(iTRAQ4plex)	HCD	1.0000	0.0000	1	1	9636	Unique	Used	2.579	7.050E3	2.287E4	201	3.78	0.00	0	8	13	3	410.58063	1229.72733	-17.37	38.22	4234	4234	MS2	20/32	Glioma_Nuclear_prep_Gradeall_4plex.raw
66529396	High		IFVYDPNNPPSSEVLR	Unambiguous	2	1	N-Term(iTRAQ4plex)	HCD	1.0000	0.0000	1	1	10930	Unique	Used	2.686	2.667E4	9.009E4	139	4.53	0.00	0	2	18	2	996.00604	1991.00481	-15.43	47.92	5645	5645	MS2	20/30	Glioma_Nuclear_prep_Gradeall_4plex.raw
66529396	High		IFVYDPNNPPSSEVLR	Unambiguous	2	1	N-Term(iTRAQ4plex)	HCD	1.0000	0.0000	1	1	10842	Unique	Used	3.111	2.236E4	8.750E4	140	4.53	0.00	0	9	29	2	996.00720	1991.00713	-14.26	47.23	5549	5549	MS2	21/30	Glioma_Nuclear_prep_Gradeall_4plex.raw
66529396		High	IFVYDPNNPPSSEVLR	Unambiguous	2	1	N-Term(iTRAQ4plex)	HCD	1.0000	0.0000	1	1	11606	Unique	Used	1.315	3.480E2	6.200E2	204	4.08	0.00	0	31	300	3	664.35138	1991.03958	2.04	65.65	6256	6256	MS2	19/60	Glioma_Nuclear_prep_Gradeall_repeat_4plex.raw
66529396		High	lLIGTLYHR	Unambiguous	2	1	N-Term(iTRAQ4plex)	HCD	1.0000	0.0000	1	1	10463	Unique	Used	2.408	3.547E2	1.157E3	139	3.73	0.00	0	34	300	3	410.58746	1229.74784	-0.69	50.58	4948	4948	MS2	17/32	Glioma_Nuclear_prep_Gradeall_repeat_4plex.raw
66529396		High	IFVYDPNNPPSSEVLR	Unambiguous	2	1	N-Term(iTRAQ4plex)	HCD	1.0000	0.0000	1	1	11599	Unique	Used	2.629	9.547E2	3.400E3	198	4.47	0.00	0	11	300	2	996.02185	1991.03642	0.45	65.55	6248	6248	MS2	21/30	Glioma_Nuclear_prep_Gradeall_repeat_4plex.raw
66529396		High	IFVYDPNNPPSSEVLR	Unambiguous	2	1	N-Term(iTRAQ4plex)	HCD	1.0000	0.0000	1	1	11667	Unique	Used	3.378	6.836E2	3.128E3	165	4.42	0.00	0	5	300	2	996.02203	1991.03679	0.64	66.54	6331	6331	MS2	18/30	Glioma_Nuclear_prep_Gradeall_repeat_4plex.raw
66529396	High		eVESVDLPHcHLIk	Unambiguous	2	1	N-Term(iTRAQ4plex); C10(Methylthio); K14(iTRAQ4plex)	HCD	1.0000	0.0000	1	1	9880	Unique	Used	1.285	3.386E4	5.472E4	171	5.24	0.00	0	26	33	2	976.99512	1952.98296	-17.77	39.98	4499	4499	MS2	24/26	Glioma_Nuclear_prep_Gradeall_4plex.raw
66529396		High	eVESVDLPHcHLIk	Unambiguous	2	1	N-Term(iTRAQ4plex); C10(Methylthio); K14(iTRAQ4plex)	HCD	1.0000	0.0000	1	1	10863	Unique	Used	3.304	1.264E2	5.658E2	210	4.82	0.00	0	17	300	4	489.01044	1953.01992	1.15	55.46	5382	5382	MS2	33/78	Glioma_Nuclear_prep_Gradeall_repeat_4plex.raw
66529396	High		fEEAENSLLHLk	Unambiguous	2	1	N-Term(iTRAQ4plex); K12(iTRAQ4plex)	HCD	1.0000	0.0000	1	1	10404	Unique	Used	1.762	1.325E4	2.936E4	241	4.43	0.00	0	22	8	3	573.30786	1717.90903	-15.91	43.87	5070	5070	MS2	26/44	Glioma_Nuclear_prep_Gradeall_4plex.raw
66529396		High	fEEAENSLLHLk	Unambiguous	2	1	N-Term(iTRAQ4plex); K12(iTRAQ4plex)	HCD	1.0000	0.0000	1	1	11397	Unique	Used	2.437	2.751E2	9.082E2	195	4.67	0.00	0	37	300	3	573.31671	1717.93558	-0.45	62.74	6008	6008	MS2	27/44	Glioma_Nuclear_prep_Gradeall_repeat_4plex.raw
68051721	NP_065843.3	NCEH1	57552	neutral cholesterol ester hydrolase 1 isoform b [Homo sapiens]	9.09	3	3	7	0.590	5;2	17.7	26.34	440	49.0	7.93																			
68051721	High		iVQELPQLLDAR	Unambiguous	3	1	N-Term(iTRAQ4plex)	HCD	1.0000	0.0000	1	1	11473	Unique	Used	0.523	4.849E4	3.192E4	226	3.56	0.00	0	47	94	2	769.95526	1538.90325	0.60	52.37	6238	6238	MS2	18/22	Glioma_Nuclear_prep_Gradeall_4plex.raw
68051721	High		iVQELPQLLDAR	Unambiguous	3	1	N-Term(iTRAQ4plex)	HCD	1.0000	0.0000	1	1	11561	Unique	Used	0.590	4.267E4	3.166E4	223	3.76	0.00	0	33	82	2	769.95557	1538.90386	1.00	53.10	6334	6334	MS2	17/22	Glioma_Nuclear_prep_Gradeall_4plex.raw
68051721		High	iVQELPQLLDAR	Unambiguous	3	1	N-Term(iTRAQ4plex)	HCD	1.0000	0.0000	1	1	12148	Unique	Used	0.418	4.070E3	2.307E3	196	3.73	0.00	0	21	300	2	769.95703	1538.90679	2.90	75.08	7018	7018	MS2	18/22	Glioma_Nuclear_prep_Gradeall_repeat_4plex.raw
68051721		High	vTDTDFDGVEVR	Unambiguous	2	1	N-Term(iTRAQ4plex)	HCD	1.0000	0.0000	1	1	9845	Unique	Used	0.600	7.866E3	6.388E3	192	4.39	0.00	0	33	300	2	748.87231	1496.73735	1.58	43.15	4282	4282	MS2	18/22	Glioma_Nuclear_prep_Gradeall_repeat_4plex.raw
68051721	High		sAPLIADQAVLQLLPk	Unambiguous	3	1	N-Term(iTRAQ4plex); K16(iTRAQ4plex)	HCD	1.0000	0.0000	1	1	12439	Unique	Used	0.589	1.079E4	7.992E3	39	6.73	0.00	0	29	300	2	983.10126	1965.19524	-1.79	61.58	7295	7295	MS2	26/30	Glioma_Nuclear_prep_Gradeall_4plex.raw
68051721	High		sAPLIADQAVLQLLPk	Unambiguous	3	1	N-Term(iTRAQ4plex); K16(iTRAQ4plex)	HCD	1.0000	0.0000	1	1	12470	Unique	Used	0.682	2.071E3	1.776E3	28	6.04	0.00	0	25	145	3	655.73907	1965.20267	2.00	61.95	7330	7330	MS2	27/60	Glioma_Nuclear_prep_Gradeall_4plex.raw
68051721	High		sAPLIADQAVLQLLPk	Unambiguous	3	1	N-Term(iTRAQ4plex); K16(iTRAQ4plex)	HCD	1.0000	0.0000	1	1	12413	Unique	Used	0.744	1.879E3	1.758E3	28	6.26	0.00	0	49	183	3	655.73926	1965.20322	2.28	61.28	7266	7266	MS2	25/60	Glioma_Nuclear_prep_Gradeall_4plex.raw
68509932	NP_001020263.1	MBP	4155	myelin basic protein isoform 4 [Homo sapiens]	33.75	6	5	32	0.400	14;18	35.8	78.27	160	17.3	11.14																			
68509932	High		yLATASTMDHAR	Unambiguous	6	1	N-Term(iTRAQ4plex)	HCD	1.0000	0.0000	1	1	8180	Unique	Used	0.260	1.649E5	5.397E4	93	5.92	0.00	0	24	4	3	494.24185	1480.71100	-15.21	27.64	2648	2648	MS2	22/44	Glioma_Nuclear_prep_Gradeall_4plex.raw
68509932	High		dTGILDSIGR	Unambiguous	6	1	N-Term(iTRAQ4plex)	HCD	1.0000	0.0000	1	1	9578	Unique	Used	0.368	1.103E6	5.110E5	185	3.07	0.00	0	10	2	2	595.81958	1190.63188	-15.03	37.82	4172	4172	MS2	17/18	Glioma_Nuclear_prep_Gradeall_4plex.raw
68509932	High		dTGILDSIGR	Unambiguous	6	1	N-Term(iTRAQ4plex)	HCD	1.0000	0.0000	1	1	9489	Unique	Used	0.514	2.808E5	1.814E5	220	3.25	0.00	0	45	10	2	595.82001	1190.63274	-14.32	37.17	4075	4075	MS2	15/18	Glioma_Nuclear_prep_Gradeall_4plex.raw
68509932		High	yLATASTMDHAR	Unambiguous	6	1	N-Term(iTRAQ4plex)	HCD	1.0000	0.0000	1	1	8568	Unique	Used	0.228	4.927E4	1.521E4	196	4.34	0.00	0	19	109	2	740.87335	1480.73943	3.99	29.31	2925	2925	MS2	18/22	Glioma_Nuclear_prep_Gradeall_repeat_4plex.raw
68509932		High	yLATASTMDHAR	Unambiguous	6	1	N-Term(iTRAQ4plex)	HCD	1.0000	0.0000	1	1	8534	Unique	Used	0.276	1.797E4	6.707E3	115	6.36	0.00	0	42	92	3	494.24536	1480.72153	-8.10	28.96	2889	2889	MS2	23/44	Glioma_Nuclear_prep_Gradeall_repeat_4plex.raw
68509932		High	dTGILDSIGR	Unambiguous	6	1	N-Term(iTRAQ4plex)	HCD	1.0000	0.0000	1	1	10053	Unique	Used	0.318	1.013E5	4.366E4	255	3.59	0.00	0	5	67	2	595.83032	1190.65337	3.01	45.73	4511	4511	MS2	17/18	Glioma_Nuclear_prep_Gradeall_repeat_4plex.raw
68509932		High	dTGILDSIGR	Unambiguous	6	1	N-Term(iTRAQ4plex)	HCD	1.0000	0.0000	1	1	9972	Unique	Used	0.398	2.672E4	1.441E4	286	3.70	0.00	0	10	244	2	595.83038	1190.65349	3.11	44.72	4422	4422	MS2	17/18	Glioma_Nuclear_prep_Gradeall_repeat_4plex.raw
68509932		High	yLATASTMDHAR	Unambiguous	6	1	N-Term(iTRAQ4plex)	HCD	1.0000	0.0000	1	1	8222	Unique	Used	0.668	5.407E3	4.895E3	143	4.95	0.00	0	37	130	3	494.25058	1480.73719	2.47	25.95	2560	2560	MS2	21/44	Glioma_Nuclear_prep_Gradeall_repeat_4plex.raw
68509932		High	dTGILDSIGR	Unambiguous	6	1	N-Term(iTRAQ4plex)	HCD	1.0000	0.0000	1	1	10152	Unique	Used	0.748	7.329E3	7.426E3	283	3.19	0.00	0	56	300	2	595.83002	1190.65276	2.50	46.90	4617	4617	MS2	15/18	Glioma_Nuclear_prep_Gradeall_repeat_4plex.raw
68509932	High		tAHYGSLPQk	Unambiguous	5	1	N-Term(iTRAQ4plex); K10(iTRAQ4plex)	HCD	1.0000	0.0000	1	1	7516	Unique	Used	0.249	5.515E5	1.726E5	265	4.15	0.00	0	23	12	2	695.38824	1389.76921	-2.64	22.92	1928	1928	MS2	17/18	Glioma_Nuclear_prep_Gradeall_4plex.raw
68509932	High		gVDAQGTLSk	Unambiguous	5	1	N-Term(iTRAQ4plex); K10(iTRAQ4plex)	HCD	1.0000	0.0000	1	1	7535	Unique	Used	0.441	6.797E5	3.771E5	273	4.06	0.00	0	27	7	2	632.36218	1263.71709	1.87	23.07	1949	1949	MS2	18/18	Glioma_Nuclear_prep_Gradeall_4plex.raw

68509932	High		gVDAQGTLSk	Unambiguous	5	1	N-Term(iTRAQ4plex); K10(iTRAQ4plex)	HCD	1.0000	0.0000	1	1	7667	Unique	Used	0.592	2.235E5	1.663E5	279	3.70	0.00	0	20	10	2	632.36041	1263.71355	-0.93	24.01	2092	2092	MS2	18/18	Glioma_Nuclear_prep_Gradeall_4plex.raw
68509932	High		tAHYGSLPQk	Unambiguous	5	1	N-Term(iTRAQ4plex); K10(iTRAQ4plex)	HCD	1.0000	0.0000	1	1	7499	Unique	Used	0.758	5.979E4	5.698E4	296	3.58	0.00	0	49	8	3	463.92868	1389.77149	-1.01	22.81	1910	1910	MS2	18/36	Glioma_Nuclear_prep_Gradeall_4plex.raw
68509932		High	tAHYGSLPQk	Unambiguous	5	1	N-Term(iTRAQ4plex); K10(iTRAQ4plex)	HCD	1.0000	0.0000	1	1	7617	Unique	Used	0.266	1.129E5	4.067E4	224	3.74	0.00	0	35	39	2	695.39362	1389.77995	5.09	20.10	1919	1919	MS2	17/18	Glioma_Nuclear_prep_Gradeall_repeat_4plex.raw
68509932		High	tAHYGSLPQk	Unambiguous	5	1	N-Term(iTRAQ4plex); K10(iTRAQ4plex)	HCD	1.0000	0.0000	1	1	7584	Unique	Used	0.423	1.686E4	9.659E3	256	4.06	0.00	0	44	92	3	463.92877	1389.77176	-0.81	19.79	1883	1883	MS2	18/36	Glioma_Nuclear_prep_Gradeall_repeat_4plex.raw
68509932		High	tAHYGSLPQk	Unambiguous	5	1	N-Term(iTRAQ4plex); K10(iTRAQ4plex)	HCD	1.0000	0.0000	1	1	7673	Unique	Used	0.439	9.796E3	5.824E3	232	3.91	0.00	0	36	86	3	463.92545	1389.76178	-7.99	20.67	1982	1982	MS2	18/36	Glioma_Nuclear_prep_Gradeall_repeat_4plex.raw
68509932		High	gVDAQGTLSk	Unambiguous	5	1	N-Term(iTRAQ4plex); K10(iTRAQ4plex)	HCD	1.0000	0.0000	1	1	7568	Unique	Used	0.449	1.320E5	8.026E4	255	3.65	0.00	0	10	28	2	632.35974	1263.71221	-2.00	19.68	1867	1867	MS2	18/18	Glioma_Nuclear_prep_Gradeall_repeat_4plex.raw
68509932		High	gVDAQGTLSk	Unambiguous	5	1	N-Term(iTRAQ4plex); K10(iTRAQ4plex)	HCD	1.0000	0.0000	1	1	7694	Unique	Used	0.528	3.445E4	2.463E4	279	3.76	0.00	0	17	118	2	632.35486	1263.70244	-9.72	20.88	2004	2004	MS2	18/18	Glioma_Nuclear_prep_Gradeall_repeat_4plex.raw
68509932	High		tQDENPVVHFFk	Unambiguous	5	1	N-Term(iTRAQ4plex); K12(iTRAQ4plex)	HCD	1.0000	0.0000	1	1	9745	Unique	Used	0.287	5.883E5	2.121E5	176	5.16	0.00	0	6	5	2	874.95319	1748.89910	-12.55	39.01	4353	4353	MS2	21/22	Glioma_Nuclear_prep_Gradeall_4plex.raw
68509932	High		tQDENPVVHFFk	Unambiguous	5	1	N-Term(iTRAQ4plex); K12(iTRAQ4plex)	HCD	1.0000	0.0000	1	1	9721	Unique	Used	0.297	8.362E5	3.122E5	147	4.66	0.00	0	8	1	3	583.63715	1748.89688	-13.81	38.84	4327	4327	MS2	27/44	Glioma_Nuclear_prep_Gradeall_4plex.raw
68509932	High		tQDENPVVHFFk	Unambiguous	5	1	N-Term(iTRAQ4plex); K12(iTRAQ4plex)	HCD	1.0000	0.0000	1	1	9822	Unique	Used	0.405	3.997E4	2.035E4	111	4.44	0.00	0	42	5	3	583.63727	1748.89725	-13.60	39.56	4436	4436	MS2	24/44	Glioma_Nuclear_prep_Gradeall_4plex.raw
68509932	High		tQDENPVVHFFk	Unambiguous	5	1	N-Term(iTRAQ4plex); K12(iTRAQ4plex)	HCD	1.0000	0.0000	1	1	9653	Unique	Used	0.409	2.330E5	1.199E5	179	5.33	0.00	0	13	14	2	874.95319	1748.89910	-12.55	38.35	4253	4253	MS2	22/22	Glioma_Nuclear_prep_Gradeall_4plex.raw
68509932	High		tQDENPVVHFFk	Unambiguous	5	1	N-Term(iTRAQ4plex); K12(iTRAQ4plex)	HCD	1.0000	0.0000	1	1	9632	Unique	Used	0.434	4.076E4	2.225E4	126	4.92	0.00	0	43	9	3	583.63696	1748.89634	-14.12	38.19	4230	4230	MS2	23/44	Glioma_Nuclear_prep_Gradeall_4plex.raw
68509932		High	tQDENPVVHFFk	Unambiguous	5	1	N-Term(iTRAQ4plex); K12(iTRAQ4plex)	HCD	1.0000	0.0000	1	1	10574	Unique	Used	0.210	1.023E3	2.910E2	170	3.65	0.00	0	27	300	4	437.98669	1748.92495	2.24	51.92	5068	5068	MS2	20/66	Glioma_Nuclear_prep_Gradeall_repeat_4plex.raw
68509932		High	tQDENPVVHFFk	Unambiguous	5	1	N-Term(iTRAQ4plex); K12(iTRAQ4plex)	HCD	1.0000	0.0000	1	1	10591	Unique	Used	0.255	3.971E4	1.371E4	123	4.12	0.00	0	8	23	3	583.64740	1748.92765	3.78	52.13	5087	5087	MS2	26/44	Glioma_Nuclear_prep_Gradeall_repeat_4plex.raw
68509932		High	tQDENPVVHFFk	Unambiguous	5	1	N-Term(iTRAQ4plex); K12(iTRAQ4plex)	HCD	1.0000	0.0000	1	1	10592	Unique	Used	0.255	3.918E4	1.356E4	204	5.38	0.00	0	8	115	2	874.96832	1748.92937	4.76	52.14	5088	5088	MS2	21/22	Glioma_Nuclear_prep_Gradeall_repeat_4plex.raw
68509932		High	tQDENPVVHFFk	Unambiguous	5	1	N-Term(iTRAQ4plex); K12(iTRAQ4plex)	HCD	1.0000	0.0000	1	1	10515	Unique	Used	0.366	7.042E3	3.494E3	206	5.28	0.00	0	29	300	2	874.96686	1748.92644	3.09	51.22	5004	5004	MS2	21/22	Glioma_Nuclear_prep_Gradeall_repeat_4plex.raw
68509932		High	tQDENPVVHFFk	Unambiguous	5	1	N-Term(iTRAQ4plex); K12(iTRAQ4plex)	HCD	1.0000	0.0000	1	1	10505	Unique	Used	0.408	2.800E3	1.548E3	139	4.13	0.00	0	35	300	3	583.64563	1748.92234	0.74	51.11	4994	4994	MS2	25/44	Glioma_Nuclear_prep_Gradeall_repeat_4plex.raw
68509932		High	tQDENPVVHFFk	Unambiguous	5	1	N-Term(iTRAQ4plex); K12(iTRAQ4plex)	HCD	1.0000	0.0000	1	1	10686	Unique	Used	0.415	3.147E3	1.769E3	137	4.09	0.00	0	22	272	3	583.64502	1748.92051	-0.30	53.27	5189	5189	MS2	25/44	Glioma_Nuclear_prep_Gradeall_repeat_4plex.raw
68509932	High		yLATASTmDHAR	Unambiguous	6	1	N-Term(iTRAQ4plex); M8(Oxidation)	HCD	1.0000	0.0000	1	1	7510	Unique	Used	0.287	1.515E5	5.465E4	114	6.01	0.00	0	33	5	3	499.57874	1496.72165	-4.53	22.89	1922	1922	MS2	23/44	Glioma_Nuclear_prep_Gradeall_4plex.raw
68509932	High		yLATASTmDHAR	Unambiguous	6	1	N-Term(iTRAQ4plex); M8(Oxidation)	HCD	1.0000	0.0000	1	1	7520	Unique	Used	0.354	3.547E5	1.579E5	181	2.91	0.00	0	20	9	2	748.86938	1496.73149	2.04	22.96	1933	1933	MS2	13/22	Glioma_Nuclear_prep_Gradeall_4plex.raw
68509932		High	yLATASTmDHAR	Unambiguous	6	1	N-Term(iTRAQ4plex); M8(Oxidation)	HCD	1.0000	0.0000	1	1	7571	Unique	Used	0.402	3.503E4	1.908E4	118	6.36	0.00	0	20	28	3	499.57956	1496.72412	-2.88	19.70	1870	1870	MS2	23/44	Glioma_Nuclear_prep_Gradeall_repeat_4plex.raw
70167113	NP_001020278.1	ADAR	103	double-stranded RNA-specific adenosine deaminase isoform d [Homo sapiens]	17.29	4	11	23	0.509	14;9	25.3	71.76	931	103.6	8.66																			
70167113	High		tLPLTGSTFHDQIAMLSHR	Unambiguous	2	1	N-Term(iTRAQ4plex)	HCD	1.0000	0.0000	1	1	10545	Unique	Used	0.428	9.994E3	5.379E3	141	4.67	0.00	0	30	6	4	568.04382	2269.15346	-15.24	44.99	5226	5226	MS2	31/108	Glioma_Nuclear_prep_Gradeall_4plex.raw
70167113	High		tLPLTGSTFHDQIAMLSHR	Unambiguous	2	1	N-Term(iTRAQ4plex)	HCD	1.0000	0.0000	1	1	10538	Unique	Used	0.468	1.710E4	1.006E4	149	4.49	0.00	0	28	21	3	757.05652	2269.15500	-14.56	44.93	5218	5218	MS2	26/72	Glioma_Nuclear_prep_Gradeall_4plex.raw
70167113	High		yLNTNPVGGLLEYAR	Unambiguous	4	1	N-Term(iTRAQ4plex)	HCD	1.0000	0.0000	1	1	11796	Unique	Used	0.596	3.810E5	2.855E5	294	4.90	0.00	0	14	11	2	912.49103	1823.97478	-1.36	55.17	6590	6590	MS2	20/28	Glioma_Nuclear_prep_Gradeall_4plex.raw
70167113	High		dINAVLIDMER	Unambiguous	4	1	N-Term(iTRAQ4plex)	HCD	1.0000	0.0000	1	1	11121	Unique	Used	0.628	1.557E5	1.229E5	264	4.44	0.00	0	40	22	2	716.88391	1432.76055	1.29	49.40	5853	5853	MS2	16/20	Glioma_Nuclear_prep_Gradeall_4plex.raw
70167113		High	yLNTNPVGGLLEYAR	Unambiguous	4	1	N-Term(iTRAQ4plex)	HCD	1.0000	0.0000	1	1	12293	Unique	Used	0.357	2.782E3	1.344E3	242	4.22	0.00	0	10	300	2	912.49359	1823.97991	1.45	78.85	7302	7302	MS2	19/28	Glioma_Nuclear_prep_Gradeall_repeat_4plex.raw
70167113		High	dINAVLIDMER	Unambiguous	4	1	N-Term(iTRAQ4plex)	HCD	1.0000	0.0000	1	1	11835	Unique	Used	0.591	3.302E3	2.645E3	289	3.75	0.00	0	34	300	2	716.88452	1432.76177	2.14	69.19	6551	6551	MS2	17/20	Glioma_Nuclear_prep_Gradeall_repeat_4plex.raw
70167113		High	dGSAFEDGLR	Unambiguous	4	1	N-Term(iTRAQ4plex)	HCD	1.0000	0.0000	1	1	8719	Unique	Used	0.681	1.164E4	1.073E4	116	3.05	0.00	0	33	188	2	605.79614	1210.58501	2.40	30.81	3083	3083	MS2	15/18	Glioma_Nuclear_prep_Gradeall_repeat_4plex.raw
70167113		High	qEAADAALR	Unambiguous	4	1	N-Term(iTRAQ4plex)	HCD	1.0000	0.0000	1	1	7197	Unique	Used	0.696	4.466E4	4.209E4	166	3.40	0.00	0	23	78	2	544.78552	1088.56377	-16.48	16.51	1477	1477	MS2	13/16	Glioma_Nuclear_prep_Gradeall_repeat_4plex.raw
70167113	High		icDYLFNVSDSSALNLAk	Unambiguous	4	1	N-Term(iTRAQ4plex); C2(Methylthio); K18(iTRAQ4plex)	HCD	1.0000	0.0000	1	1	13015	Unique	Used	0.559	2.696E2	1.897E2	132	4.67	0.00	0	26	300	3	769.72699	2307.16642	2.62	70.19	8015	8015	MS2	22/68	Glioma_Nuclear_prep_Gradeall_4plex.raw
70167113	High		sPVTTLLEcMHk	Unambiguous	4	1	N-Term(iTRAQ4plex); C9(Methylthio); K12(iTRAQ4plex)	HCD	1.0000	0.0000	1	1	11858	Unique	Used	0.324	1.636E4	6.668E3	156	6.19	0.00	0	41	55	3	564.96161	1692.87027	-1.36	55.70	6657	6657	MS2	34/44	Glioma_Nuclear_prep_Gradeall_4plex.raw
70167113	High		sPVTTLLEcMHk	Unambiguous	4	1	N-Term(iTRAQ4plex); C9(Methylthio); K12(iTRAQ4plex)	HCD	1.0000	0.0000	1	1	11933	Unique	Used	0.356	5.632E4	2.522E4	162	7.01	0.00	0	10	14	3	564.96387	1692.87705	2.64	56.35	6739	6739	MS2	34/44	Glioma_Nuclear_prep_Gradeall_4plex.raw
70167113	High		aMTILLEEAk	Unambiguous	4	1	N-Term(iTRAQ4plex); K10(iTRAQ4plex)	HCD	1.0000	0.0000	1	1	10521	Unique	Used	0.467	2.526E5	1.484E5	269	4.15	0.00	0	28	8	2	703.90125	1406.79521	-15.32	44.81	5200	5200	MS2	18/18	Glioma_Nuclear_prep_Gradeall_4plex.raw
70167113		High	aMTILLEEAk	Unambiguous	4	1	N-Term(iTRAQ4plex); K10(iTRAQ4plex)	HCD	1.0000	0.0000	1	1	11392	Unique	Used	0.519	3.642E3	2.562E3	234	4.34	0.00	0	37	300	2	703.91516	1406.82305	4.46	62.68	6002	6002	MS2	18/18	Glioma_Nuclear_prep_Gradeall_repeat_4plex.raw
70167113	High		lVDQSGPPHEPk	Unambiguous	4	1	N-Term(iTRAQ4plex); K12(iTRAQ4plex)	HCD	1.0000	0.0000	1	1	7608	Unique	Used	0.417	7.913E4	4.151E4	213	4.20	0.00	0	35	8	3	531.29547	1591.87186	2.25	23.59	2028	2028	MS2	22/44	Glioma_Nuclear_prep_Gradeall_4plex.raw
70167113	High		lVDQSGPPHEPk	Unambiguous	4	1	N-Term(iTRAQ4plex); K12(iTRAQ4plex)	HCD	1.0000	0.0000	1	1	7633	Unique	Used	0.514	1.043E5	6.750E4	239	4.76	0.00	0	6	18	2	796.43866	1591.87004	1.11	23.77	2055	2055	MS2	20/22	Glioma_Nuclear_prep_Gradeall_4plex.raw
70167113		High	lVDQSGPPHEPk	Unambiguous	4	1	N-Term(iTRAQ4plex); K12(iTRAQ4plex)	HCD	1.0000	0.0000	1	1	7692	Unique	Used	0.452	1.784E4	1.092E4	188	4.06	0.00	0	16	58	3	531.29028	1591.85630	-7.53	20.87	2002	2002	MS2	24/44	Glioma_Nuclear_prep_Gradeall_repeat_4plex.raw
70167113	High		tAESQTPTPSATSFFSGk	Unambiguous	4	1	N-Term(iTRAQ4plex); K18(iTRAQ4plex)	HCD	1.0000	0.0000	1	1	9667	Unique	Used	0.194	7.980E4	1.946E4	128	6.40	0.00	0	0	18	2	1066.52258	2132.03789	-17.43	38.45	4268	4268	MS2	22/34	Glioma_Nuclear_prep_Gradeall_4plex.raw
70167113		High	tAESQTPTPSATSFFSGk	Unambiguous	4	1	N-Term(iTRAQ4plex); K18(iTRAQ4plex)	HCD	1.0000	0.0000	1	1	10336	Unique	Used	0.652	9.643E2	8.510E2	137	6.73	0.00	0	16	269	3	711.36450	2132.07895	1.83	49.09	4814	4814	MS2	22/68	Glioma_Nuclear_prep_Gradeall_repeat_4plex.raw
70167113	High		nIFLLFk	Unambiguous	4	1	N-Term(iTRAQ4plex); K7(iTRAQ4plex)	HCD	1.0000	0.0000	1	1	12321	Unique	Used	0.487	1.661E4	1.018E4	129	3.23	0.00	0	50	177	2	591.87830	1182.74932	0.34	60.25	7165	7165	MS2	11/12	Glioma_Nuclear_prep_Gradeall_4plex.raw
70167113	High		nIFLLFk	Unambiguous	4	1	N-Term(iTRAQ4plex); K7(iTRAQ4plex)	HCD	1.0000	0.0000	1	1	12260	Unique	Used	0.572	1.933E4	1.391E4	163	3.24	0.00	0	66	300	2	591.87665	1182.74602	-2.45	59.59	7097	7097	MS2	11/12	Glioma_Nuclear_prep_Gradeall_4plex.raw
70167113		High	nIFLLFk	Unambiguous	4	1	N-Term(iTRAQ4plex); K7(iTRAQ4plex)	HCD	1.0000	0.0000	1	1	12446	Unique	Used	0.405	1.581E3	8.680E2	152	3.04	0.00	0	39	300	2	591.87726	1182.74724	-1.42	86.94	7862	7862	MS2	11/12	Glioma_Nuclear_prep_Gradeall_repeat_4plex.raw
70167113	High		dINAVLIDmER	Unambiguous	4	1	N-Term(iTRAQ4plex); M9(Oxidation)	HCD	1.0000	0.0000	1	1	9597	Unique	Used	0.658	1.612E5	1.333E5	183	3.20	0.00	0	23	14	2	724.86945	1448.73161	-15.19	37.94	4192	4192	MS2	17/20	Glioma_Nuclear_prep_Gradeall_4plex.raw
70167113		High	dINAVLIDmER	Unambiguous	4	1	N-Term(iTRAQ4plex); M9(Oxidation)	HCD	1.0000	0.0000	1	1	10126	Unique	Used	0.509	1.006E4	6.941E3	229	4.01	0.00	0	18	300	2	724.88239	1448.75749	2.67	46.60	4590	4590	MS2	18/20	Glioma_Nuclear_prep_Gradeall_repeat_4plex.raw
73760415	NP_001027392.1	STXBP1	6812	syntaxin-binding protein 1 isoform b [Homo sapiens]	5.56	2	3	5	0.453	3;2	31.1	13.57	594	67.5	6.96																			
73760415		High	sQLLILDR	Unambiguous	2	1	N-Term(iTRAQ4plex)	HCD	1.0000	0.0000	1	1	10236	Unique	Used	0.453	1.035E4	6.353E3	195	3.23	0.00	0	34	300	2	551.34100	1101.67473	-0.12	47.88	4706	4706	MS2	14/14	Glioma_Nuclear_prep_Gradeall_repeat_4plex.raw
73760415	High		lAEQIATLcATLk	Unambiguous	2	1	N-Term(iTRAQ4plex); C9(Methylthio); K13(iTRAQ4plex)	HCD	1.0000	0.0000	1	1	13123	Unique	Used	0.280	1.187E3	4.179E2	217	4.35	0.00	0	25	300	2	854.98480	1708.96233	2.52	75.14	8353	8353	MS2	21/24	Glioma_Nuclear_prep_Gradeall_4plex.raw
73760415	High		lAEQIATLcATLk	Unambiguous	2	1	N-Term(iTRAQ4plex); C9(Methylthio); K13(iTRAQ4plex)	HCD	1.0000	0.0000	1	1	13122	Unique	Used	0.434	8.250E2	4.500E2	205	4.60	0.00	0	0	300	2	854.98358	1708.95989	1.09	75.06	8348	8348	MS2	21/24	Glioma_Nuclear_prep_Gradeall_4plex.raw
73760415	High		dNALLAQLIQDk	Unambiguous	2	1	N-Term(iTRAQ4plex); K12(iTRAQ4plex)	HCD	1.0000	0.0000	1	1	11529	Unique	Used	0.497	5.010E3	3.134E3	209	4.62	0.00	0	15	52	3	543.98163	1629.93033	-6.82	52.83	6299	6299	MS2	21/44	Glioma_Nuclear_prep_Gradeall_4plex.raw
73760415		High	dNALLAQLIQDk	Unambiguous	2	1	N-Term(iTRAQ4plex); K12(iTRAQ4plex)	HCD	1.0000	0.0000	1	1	12175	Unique	Used	0.687	1.945E2	1.811E2	206	3.93	0.00	0	37	300	3	543.98492	1629.94022	-0.75	75.60	7059	7059	MS2	20/44	Glioma_Nuclear_prep_Gradeall_repeat_4plex.raw
93102371	NP_060783.3	PBRM1	55193	protein polybromo-1 [Homo sapiens]	1.20	1	2	3	1.938	2;1	12.9	7.94	1582	181.0	6.87																			
93102371	High		vQYPDYYAIIk	Unambiguous	1	1	N-Term(iTRAQ4plex); K11(iTRAQ4plex)	HCD	1.0000	0.0000	1	1	10621	Unique	Used	1.938	3.896E4	9.494E4	272	4.64	0.00	0	27	45	2	830.95056	1660.89385	-15.07	45.54	5308	5308	MS2	18/20	Glioma_Nuclear_prep_Gradeall_4plex.raw
93102371		High	vQYPDYYAIIk	Unambiguous	1	1	N-Term(iTRAQ4plex); K11(iTRAQ4plex)	HCD	1.0000	0.0000	1	1	11472	Unique	Used	2.177	1.411E3	4.159E3	270	4.04	0.00	0	25	300	2	830.96423	1660.92119	1.39	63.78	6097	6097	MS2	18/20	Glioma_Nuclear_prep_Gradeall_repeat_4plex.raw
93102371	High		lISELFQk	Unambiguous	1	1	N-Term(iTRAQ4plex); K8(iTRAQ4plex)	HCD	1.0000	0.0000	1	1	10993	Unique	Used	1.677	5.406E4	1.140E5	260	3.30	0.00	0	63	42	2	633.38019	1265.75310	-13.97	48.40	5713	5713	MS2	13/14	Glioma_Nuclear_prep_Gradeall_4plex.raw
94721261	NP_149124.3	CNP	1267	2',3'-cyclic-nucleotide 3'-phosphodiesterase [Homo sapiens]	5.23	1	1	3	0.641	1;2	9.5	13.51	421	47.5	9.07																			
94721261	High		mDLVTYFGk	Unambiguous	1	1	N-Term(iTRAQ4plex); K9(iTRAQ4plex)	HCD	1.0000	0.0000	1	1	10892	Unique	Used	0.641	3.994E5	3.222E5	260	3.76	0.00	0	32	9	2	681.36230	1361.71733	-15.01	47.63	5604	5604	MS2	15/16	Glioma_Nuclear_prep_Gradeall_4plex.raw
94721261		High	mDLVTYFGk	Unambiguous	1	1	N-Term(iTRAQ4plex); K9(iTRAQ4plex)	HCD	1.0000	0.0000	1	1	11706	Unique	Used	0.632	5.670E3	4.853E3	318	3.65	0.00	0	58	300	2	681.37317	1361.73906	0.95	67.06	6376	6376	MS2	14/16	Glioma_Nuclear_prep_Gradeall_repeat_4plex.raw
94721261		High	mDLVTYFGk	Unambiguous	1	1	N-Term(iTRAQ4plex); M1(Oxidation); K9(iTRAQ4plex)	HCD	1.0000	0.0000	1	1	10627	Unique	Used	0.747	5.496E3	5.559E3	296	3.37	0.00	0	75	300	2	689.36896	1377.73064	-1.48	52.55	5125	5125	MS2	14/16	Glioma_Nuclear_prep_Gradeall_repeat_4plex.raw
100913206	NP_001348.2	DHX9	1660	ATP-dependent RNA helicase A [Homo sapiens]	20.71	1	11	23	0.612	12;11	12.1	136.22	1270	140.9	6.84																			
100913206	High		aIEPPPLDAVIEAEHTLR	Unambiguous	1	1	N-Term(iTRAQ4plex)	HCD	1.0000	0.0000	1	1	11774	Unique	Used	0.534	3.602E4	2.419E4	141	7.86	0.00	0	5	6	3	705.72333	2115.15543	-0.62	54.99	6566	6566	MS2	36/68	Glioma_Nuclear_prep_Gradeall_4plex.raw
100913206	High		hLEMNPHFGSHR	Unambiguous	1	1	N-Term(iTRAQ4plex)	HCD	1.0000	0.0000	1	1	7496	Unique	Used	0.571	7.299E4	5.245E4	81	4.58	0.00	0	26	3	4	402.19879	1605.77334	-5.73	22.80	1907	1907	MS2	27/66	Glioma_Nuclear_prep_Gradeall_4plex.raw
100913206	High		tTQVPQFILDDFIQNDR	Unambiguous	1	1	N-Term(iTRAQ4plex)	HCD	1.0000	0.0000	1	1	12677	Unique	Used	0.621	4.691E3	3.661E3	120	5.90	0.00	0	8	245	2	1097.56482	2194.12236	-1.72	64.72	7572	7572	MS2	21/32	Glioma_Nuclear_prep_Gradeall_4plex.raw
100913206	High		tTQVPQFILDDFIQNDR	Unambiguous	1	1	N-Term(iTRAQ4plex)	HCD	1.0000	0.0000	1	1	12622	Unique	Used	0.641	7.463E3	6.014E3	115	6.03	0.00	0	0	194	2	1097.56506	2194.12285	-1.50	64.02	7510	7510	MS2	20/32	Glioma_Nuclear_prep_Gradeall_4plex.raw
100913206	High		aIEPPPLDAVIEAEHTLR	Unambiguous	1	1	N-Term(iTRAQ4plex)	HCD	1.0000	0.0000	1	1	11707	Unique	Used	0.659	2.896E4	2.399E4	124	4.78	0.00	0	5	51	2	1058.07983	2115.15239	-2.05	54.39	6493	6493	MS2	19/34	Glioma_Nuclear_prep_Gradeall_4plex.raw
100913206	High		qPAIISQLDPVNER	Unambiguous	1	1	N-Term(iTRAQ4plex)	HCD	1.0000	0.0000	1	1	10166	Unique	Used	0.662	7.591E4	6.324E4	252	3.98	0.00	0	41	43	2	862.46442	1723.92156	-14.17	42.10	4811	4811	MS2	14/26	Glioma_Nuclear_prep_Gradeall_4plex.raw
100913206	High		gISHVIVDEIHER	Unambiguous	1	1	N-Term(iTRAQ4plex)	HCD	1.0000	0.0000	1	1	8863	Unique	Used	0.714	6.612E4	5.938E4	190	4.35	0.00	0	36	18	2	824.43811	1647.86894	-14.93	32.54	3390	3390	MS2	22/24	Glioma_Nuclear_prep_Gradeall_4plex.raw
100913206	High		eLDALDANDELTPLGR	Unambiguous	1	1	N-Term(iTRAQ4plex)	HCD	1.0000	0.0000	1	1	10688	Unique	Used	0.723	1.364E5	1.239E5	133	4.93	0.00	0	13	13	2	943.47089	1885.93450	-14.82	46.07	5382	5382	MS2	26/30	Glioma_Nuclear_prep_Gradeall_4plex.raw
100913206		High	qPAIISQLDPVNER	Unambiguous	1	1	N-Term(iTRAQ4plex)	HCD	1.0000	0.0000	1	1	10944	Unique	Used	0.492	3.306E3	2.202E3	267	4.18	0.00	0	25	300	2	862.47968	1723.95207	3.53	56.47	5471	5471	MS2	19/26	Glioma_Nuclear_prep_Gradeall_repeat_4plex.raw
100913206		High	aIEPPPLDAVIEAEHTLR	Unambiguous	1	1	N-Term(iTRAQ4plex)	HCD	1.0000	0.0000	1	1	12317	Unique	Used	0.510	7.394E2	5.106E2	120	3.92	0.00	0	3	300	2	1058.08264	2115.15801	0.60	79.50	7351	7351	MS2	17/34	Glioma_Nuclear_prep_Gradeall_repeat_4plex.raw
100913206		High	tTQVPQFILDDFIQNDR	Unambiguous	1	1	N-Term(iTRAQ4plex)	HCD	1.0000	0.0000	1	1	12468	Unique	Used	0.567	8.595E1	6.596E1	160	4.72	0.00	0	6	300	3	732.04736	2194.12754	0.63	91.20	8142	8142	MS2	21/64	Glioma_Nuclear_prep_Gradeall_repeat_4plex.raw
100913206		High	eLDALDANDELTPLGR	Unambiguous	1	1	N-Term(iTRAQ4plex)	HCD	1.0000	0.0000	1	1	11370	Unique	Used	0.609	2.209E3	1.823E3	171	5.63	0.00	0	6	300	2	943.48566	1885.96404	0.84	62.38	5977	5977	MS2	26/30	Glioma_Nuclear_prep_Gradeall_repeat_4plex.raw
100913206		High	aIEPPPLDAVIEAEHTLR	Unambiguous	1	1	N-Term(iTRAQ4plex)	HCD	1.0000	0.0000	1	1	12295	Unique	Used	0.616	2.983E2	2.491E2	141	6.85	0.00	0	10	300	3	705.72485	2115.16001	1.55	78.90	7306	7306	MS2	36/68	Glioma_Nuclear_prep_Gradeall_repeat_4plex.raw
100913206	High		lGGIGQFLAk	Unambiguous	1	1	N-Term(iTRAQ4plex); K10(iTRAQ4plex)	HCD	1.0000	0.0000	1	1	10608	Unique	Used	0.567	1.756E5	1.252E5	228	3.50	0.00	0	22	13	2	646.39301	1291.77873	-14.66	45.45	5294	5294	MS2	18/18	Glioma_Nuclear_prep_Gradeall_4plex.raw
100913206		High	lGGIGQFLAk	Unambiguous	1	1	N-Term(iTRAQ4plex); K10(iTRAQ4plex)	HCD	1.0000	0.0000	1	1	11523	Unique	Used	0.598	6.499E3	5.262E3	175	3.91	0.00	0	35	300	2	646.40186	1291.79643	-0.96	64.43	6154	6154	MS2	18/18	Glioma_Nuclear_prep_Gradeall_repeat_4plex.raw
100913206	High		yPSPFFVFGEk	Unambiguous	1	1	N-Term(iTRAQ4plex); K11(iTRAQ4plex)	HCD	1.0000	0.0000	1	1	11912	Unique	Used	0.666	1.420E5	1.189E5	246	3.38	0.00	0	22	23	2	803.43628	1605.86528	6.04	56.16	6716	6716	MS2	15/20	Glioma_Nuclear_prep_Gradeall_4plex.raw
100913206		High	tPLHEIALSIk	Unambiguous	1	1	N-Term(iTRAQ4plex); K11(iTRAQ4plex)	HCD	1.0000	0.0000	1	1	10411	Unique	Used	0.470	8.532E3	5.432E3	93	3.95	0.00	0	66	300	2	755.46832	1509.92937	3.34	49.96	4893	4893	MS2	16/20	Glioma_Nuclear_prep_Gradeall_repeat_4plex.raw
100913206		High	tPLHEIALSIk	Unambiguous	1	1	N-Term(iTRAQ4plex); K11(iTRAQ4plex)	HCD	1.0000	0.0000	1	1	10409	Unique	Used	0.521	1.819E3	1.284E3	101	4.61	0.00	0	40	300	3	503.97952	1509.92401	-0.21	49.94	4891	4891	MS2	22/40	Glioma_Nuclear_prep_Gradeall_repeat_4plex.raw
100913206		High	yPSPFFVFGEk	Unambiguous	1	1	N-Term(iTRAQ4plex); K11(iTRAQ4plex)	HCD	1.0000	0.0000	1	1	12344	Unique	Used	0.612	2.775E3	2.300E3	219	3.76	0.00	0	20	300	2	803.43262	1605.85796	1.48	80.51	7423	7423	MS2	16/20	Glioma_Nuclear_prep_Gradeall_repeat_4plex.raw
100913206	High		qLYHLGVVEAYSGLTk	Unambiguous	1	1	N-Term(iTRAQ4plex); K16(iTRAQ4plex)	HCD	1.0000	0.0000	1	1	10373	Unique	Used	0.682	3.217E4	2.759E4	214	6.43	0.00	0	9	49	2	1033.56201	2066.11675	-17.30	43.64	5036	5036	MS2	25/30	Glioma_Nuclear_prep_Gradeall_4plex.raw
100913206		High	qLYHLGVVEAYSGLTk	Unambiguous	1	1	N-Term(iTRAQ4plex); K16(iTRAQ4plex)	HCD	1.0000	0.0000	1	1	11379	Unique	Used	0.717	6.467E2	6.280E2	136	6.07	0.00	0	30	300	3	689.38947	2066.15384	0.66	62.50	5987	5987	MS2	28/60	Glioma_Nuclear_prep_Gradeall_repeat_4plex.raw
100913206	High		eGETVEPYk	Unambiguous	1	1	N-Term(iTRAQ4plex); K9(iTRAQ4plex)	HCD	1.0000	0.0000	1	1	7628	Unique	Used	0.639	3.109E5	2.497E5	202	3.37	0.00	0	7	7	2	670.35175	1339.69621	-1.63	23.74	2050	2050	MS2	16/16	Glioma_Nuclear_prep_Gradeall_4plex.raw
100913206		High	eGETVEPYk	Unambiguous	1	1	N-Term(iTRAQ4plex); K9(iTRAQ4plex)	HCD	1.0000	0.0000	1	1	7710	Unique	Used	0.539	4.592E4	3.352E4	180	3.29	0.00	0	15	75	2	670.35010	1339.69292	-4.09	21.05	2021	2021	MS2	16/16	Glioma_Nuclear_prep_Gradeall_repeat_4plex.raw
109240550	NP_001035879.1	PSPC1	55269	paraspeckle component 1 [Homo sapiens]	20.65	1	8	14	0.423	7;7	35.6	38.59	523	58.7	6.67																			
109240550	High		nLSPVVSNELLEQAFSQFGPVEk	Unambiguous	1	1	N-Term(iTRAQ4plex); K23(iTRAQ4plex)	HCD	1.0000	0.0000	1	1	12958	Unique	Used	0.519	1.029E3	6.717E2	109	9.06	0.00	0	13	235	3	940.84186	2820.51102	3.10	68.97	7924	7924	MS2	34/88	Glioma_Nuclear_prep_Gradeall_4plex.raw
109240550	High		fAQPGTFEFEYASR	Unambiguous	1	1	N-Term(iTRAQ4plex)	HCD	1.0000	0.0000	1	1	10496	Unique	Used	0.285	2.908E5	1.041E5	86	4.34	0.00	0	28	27	2	897.42389	1793.84050	-11.75	44.61	5172	5172	MS2	17/26	Glioma_Nuclear_prep_Gradeall_4plex.raw
109240550		High	fAQPGTFEFEYASR	Unambiguous	1	1	N-Term(iTRAQ4plex)	HCD	1.0000	0.0000	1	1	11306	Unique	Used	0.242	7.395E3	2.421E3	134	4.26	0.00	0	12	300	2	897.43707	1793.86687	2.95	61.38	5894	5894	MS2	20/26	Glioma_Nuclear_prep_Gradeall_repeat_4plex.raw
109240550		High	yGEPSEVFINR	Unambiguous	1	1	N-Term(iTRAQ4plex)	HCD	1.0000	0.0000	1	1	10006	Unique	Used	0.318	2.175E4	9.361E3	220	3.67	0.00	0	41	300	2	727.87573	1454.74419	3.12	45.12	4458	4458	MS2	15/20	Glioma_Nuclear_prep_Gradeall_repeat_4plex.raw
109240550	High		fATHGAALTVk	Unambiguous	1	1	N-Term(iTRAQ4plex); K11(iTRAQ4plex)	HCD	1.0000	0.0000	1	1	8055	Unique	Used	0.380	2.097E5	1.003E5	235	4.02	0.00	0	10	23	2	702.40753	1403.80779	-12.22	26.74	2512	2512	MS2	20/20	Glioma_Nuclear_prep_Gradeall_4plex.raw
109240550		High	fATHGAALTVk	Unambiguous	1	1	N-Term(iTRAQ4plex); K11(iTRAQ4plex)	HCD	1.0000	0.0000	1	1	8429	Unique	Used	0.524	1.898E4	1.347E4	264	3.63	0.00	0	53	300	2	702.41937	1403.83147	4.65	27.91	2777	2777	MS2	18/20	Glioma_Nuclear_prep_Gradeall_repeat_4plex.raw
109240550	High		lFVGNLPTDITEEDFk	Unambiguous	1	1	N-Term(iTRAQ4plex); K16(iTRAQ4plex)	HCD	1.0000	0.0000	1	1	11992	Unique	Used	0.191	7.421E4	1.786E4	218	6.23	0.00	0	11	67	2	1063.56628	2126.12529	-0.35	56.91	6804	6804	MS2	22/30	Glioma_Nuclear_prep_Gradeall_4plex.raw
109240550	High		lFVGNLPTDITEEDFk	Unambiguous	1	1	N-Term(iTRAQ4plex); K16(iTRAQ4plex)	HCD	1.0000	0.0000	1	1	11981	Unique	Used	0.408	1.169E4	5.996E3	172	5.57	0.00	0	19	21	3	709.38043	2126.12674	0.33	56.81	6792	6792	MS2	23/60	Glioma_Nuclear_prep_Gradeall_4plex.raw
109240550		High	lFVGNLPTDITEEDFkR	Unambiguous	1	1	N-Term(iTRAQ4plex); K16(iTRAQ4plex)	HCD	1.0000	0.0000	1	1	12030	Unique	Used	0.331	1.603E3	7.198E2	162	4.55	0.00	1	26	300	3	761.41620	2282.23404	3.02	72.55	6822	6822	MS2	27/64	Glioma_Nuclear_prep_Gradeall_repeat_4plex.raw
109240550		High	lFVGNLPTDITEEDFk	Unambiguous	1	1	N-Term(iTRAQ4plex); K16(iTRAQ4plex)	HCD	1.0000	0.0000	1	1	12356	Unique	Used	0.531	2.917E2	2.099E2	173	4.54	0.00	0	17	300	3	709.38214	2126.13187	2.74	80.79	7444	7444	MS2	25/60	Glioma_Nuclear_prep_Gradeall_repeat_4plex.raw
109240550	High		nLSPVVSNELLEQAFSQFGPVEk	Unambiguous	1	1	N-Term(iTRAQ4plex); K23(iTRAQ4plex)	HCD	1.0000	0.0000	1	1	12959	Unique	Used	0.778	7.133E1	6.976E1	101	6.27	0.00	0	26	300	4	705.88501	2820.51821	5.65	69.00	7926	7926	MS2	30/132	Glioma_Nuclear_prep_Gradeall_4plex.raw
109240550		High	gFVEFAAkPPAR	Unambiguous	1	1	N-Term(iTRAQ4plex); K8(iTRAQ4plex)	HCD	1.0000	0.0000	1	1	9360	Unique	Used	0.517	1.371E4	9.605E3	166	3.77	0.00	0	25	104	3	526.64221	1577.91208	4.94	37.74	3763	3763	MS2	25/44	Glioma_Nuclear_prep_Gradeall_repeat_4plex.raw
109240550	High		aELDGTILk	Unambiguous	1	1	N-Term(iTRAQ4plex); K9(iTRAQ4plex)	HCD	1.0000	0.0000	1	1	8908	Unique	Used	0.444	4.397E5	2.455E5	266	3.10	0.00	0	37	10	2	624.36835	1247.72942	-12.46	32.87	3439	3439	MS2	16/16	Glioma_Nuclear_prep_Gradeall_4plex.raw
109240550		High	aELDGTILk	Unambiguous	1	1	N-Term(iTRAQ4plex); K9(iTRAQ4plex)	HCD	1.0000	0.0000	1	1	9383	Unique	Used	0.439	4.272E4	2.538E4	258	3.70	0.00	0	32	207	2	624.37762	1247.74797	2.41	37.97	3787	3787	MS2	16/16	Glioma_Nuclear_prep_Gradeall_repeat_4plex.raw
109689718	NP_001035937.1	ABHD12	26090	monoacylglycerol lipase ABHD12 isoform a [Homo sapiens]	9.55	2	2	3	0.654	1;2	8.5	13.25	398	45.1	8.65																			
109689718	High		eTPPDALILESPFTNIR	Unambiguous	2	1	N-Term(iTRAQ4plex)	HCD	1.0000	0.0000	1	1	12214	Unique	Used	0.654	1.594E4	1.311E4	246	5.44	0.00	0	48	300	2	1029.06226	2057.11724	6.62	59.13	7047	7047	MS2	18/32	Glioma_Nuclear_prep_Gradeall_4plex.raw
109689718		High	eTPPDALILESPFTNIR	Unambiguous	2	1	N-Term(iTRAQ4plex)	HCD	1.0000	0.0000	1	1	12417	Unique	Used	0.611	1.178E2	9.755E1	133	3.90	0.00	0	16	300	3	686.37372	2057.10660	1.45	84.63	7706	7706	MS2	18/64	Glioma_Nuclear_prep_Gradeall_repeat_4plex.raw
109689718		High	vQFVPFHSDLGYR	Unambiguous	2	1	N-Term(iTRAQ4plex)	HCD	1.0000	0.0000	1	1	11073	Unique	Used	0.723	1.496E3	1.466E3	206	3.96	0.00	0	32	300	3	570.30334	1708.89548	1.57	58.09	5614	5614	MS2	21/48	Glioma_Nuclear_prep_Gradeall_repeat_4plex.raw
116812577	NP_057103.2	LUC7L2	51631	putative RNA-binding protein Luc7-like 2 isoform 1 [Homo sapiens]	15.56	6	4	4	1.724	2;2	37.1	15.43	392	46.5	10.01																			
116812577	High		lHLGFIEIR	Unambiguous	4	1	N-Term(iTRAQ4plex)	HCD	1.0000	0.0000	1	1	10638	Unique	Used	2.752	1.079E4	3.733E4	155	3.62	0.00	0	13	33	3	414.58090	1241.72815	-16.55	45.66	5326	5326	MS2	21/32	Glioma_Nuclear_prep_Gradeall_4plex.raw
116812577	High		mDLGEcLk	Unambiguous	4	1	N-Term(iTRAQ4plex); C6(Methylthio); K8(iTRAQ4plex)	HCD	1.0000	0.0000	1	1	10464	Unique	Used	2.117	6.930E4	1.845E5	80	3.04	0.00	0	62	25	2	621.80084	1242.59441	-15.37	44.36	5137	5137	MS2	14/14	Glioma_Nuclear_prep_Gradeall_4plex.raw
116812577		High	vHELNEEIGk	Unambiguous	6	1	N-Term(iTRAQ4plex); K10(iTRAQ4plex)	HCD	1.0000	0.0000	1	1	8164	Unique	Used	1.330	3.594E3	6.473E3	231	4.19	0.00	0	51	229	3	485.93860	1455.80124	-2.31	25.36	2498	2498	MS2	19/36	Glioma_Nuclear_prep_Gradeall_repeat_4plex.raw
116812577		High	aDYEIASk	Unambiguous	6	1	N-Term(iTRAQ4plex); K8(iTRAQ4plex)	HCD	1.0000	0.0000	1	1	7960	Unique	Used	1.301	2.363E4	4.163E4	189	3.39	0.00	0	31	225	2	592.82135	1184.63542	-3.99	23.41	2284	2284	MS2	14/14	Glioma_Nuclear_prep_Gradeall_repeat_4plex.raw
117190174	NP_004491.2	HNRNPC	3183	heterogeneous nuclear ribonucleoproteins C1/C2 isoform b [Homo sapiens]	41.64	5	13	40	0.383	22;18	38.0	113.18	293	32.3	5.08																			
117190174	High		sAAEMYGSSFDLDYDFQR	Unambiguous	1	1	N-Term(iTRAQ4plex)	HCD	1.0000	0.0000	1	1	11390	Unique	Used	0.287	2.470E4	8.931E3	36	6.58	0.00	0	14	64	2	1123.49646	2245.98564	1.22	51.65	6147	6147	MS2	27/34	Glioma_Nuclear_prep_Gradeall_4plex.raw
117190174	High		gFAFVQYVNER	Unambiguous	2	1	N-Term(iTRAQ4plex)	HCD	1.0000	0.0000	1	1	10522	Unique	Used	0.617	4.202E5	3.258E5	178	4.35	0.00	0	37	13	2	737.37433	1473.74138	-13.13	44.82	5201	5201	MS2	17/20	Glioma_Nuclear_prep_Gradeall_4plex.raw
117190174		High	gFAFVQYVNER	Unambiguous	2	1	N-Term(iTRAQ4plex)	HCD	1.0000	0.0000	1	1	11385	Unique	Used	0.586	1.285E4	1.020E4	249	4.27	0.00	0	26	300	2	737.38544	1473.76360	1.95	62.58	5994	5994	MS2	16/20	Glioma_Nuclear_prep_Gradeall_repeat_4plex.raw
117190174		High	aAVAGEDGR	Unambiguous	5	1	N-Term(iTRAQ4plex)	HCD	1.0000	0.0000	1	1	6592	Unique	Used	0.590	2.529E4	2.023E4	107	3.22	0.00	0	42	223	2	495.25595	989.50463	-8.76	11.09	838	838	MS2	15/16	Glioma_Nuclear_prep_Gradeall_repeat_4plex.raw
117190174	High		kSDVEAIFSk	Unambiguous	5	1	N-Term(iTRAQ4plex); K1(iTRAQ4plex); K10(iTRAQ4plex)	HCD	1.0000	0.0000	1	1	8991	Unique	Used	0.613	4.812E4	3.713E4	210	3.44	0.00	1	54	5	3	519.30243	1555.89273	-8.28	33.46	3529	3529	MS2	17/36	Glioma_Nuclear_prep_Gradeall_4plex.raw
117190174	High		kSDVEAIFSk	Unambiguous	5	1	N-Term(iTRAQ4plex); K1(iTRAQ4plex); K10(iTRAQ4plex)	HCD	1.0000	0.0000	1	1	8995	Unique	Used	0.622	3.177E5	2.484E5	226	4.15	0.00	1	54	9	2	778.44763	1555.88799	-11.33	33.48	3533	3533	MS2	15/18	Glioma_Nuclear_prep_Gradeall_4plex.raw
117190174		High	kSDVEAIFSk	Unambiguous	5	1	N-Term(iTRAQ4plex); K1(iTRAQ4plex); K10(iTRAQ4plex)	HCD	1.0000	0.0000	1	1	9622	Unique	Used	0.667	3.319E3	2.997E3	190	4.11	0.00	1	81	253	3	519.30322	1555.89511	-6.75	40.65	4047	4047	MS2	22/36	Glioma_Nuclear_prep_Gradeall_repeat_4plex.raw
117190174	High		vDSLLENLEk	Unambiguous	5	1	N-Term(iTRAQ4plex); K10(iTRAQ4plex)	HCD	1.0000	0.0000	1	1	10709	Unique	Used	0.256	9.726E5	3.128E5	259	4.32	0.00	0	43	4	2	724.40656	1447.80583	-13.02	46.22	5405	5405	MS2	18/18	Glioma_Nuclear_prep_Gradeall_4plex.raw
117190174	High		vDSLLENLEk	Unambiguous	5	1	N-Term(iTRAQ4plex); K10(iTRAQ4plex)	HCD	1.0000	0.0000	1	1	10798	Unique	Used	0.292	6.780E5	2.486E5	335	3.55	0.00	0	21	5	2	724.40710	1447.80693	-12.26	46.91	5502	5502	MS2	16/18	Glioma_Nuclear_prep_Gradeall_4plex.raw
117190174		High	vDSLLENLEk	Unambiguous	5	1	N-Term(iTRAQ4plex); K10(iTRAQ4plex)	HCD	1.0000	0.0000	1	1	11521	Unique	Used	0.194	1.355E4	3.562E3	270	4.79	0.00	0	19	286	2	724.41724	1447.82720	1.74	64.41	6152	6152	MS2	18/18	Glioma_Nuclear_prep_Gradeall_repeat_4plex.raw
117190174		High	vDSLLENLEk	Unambiguous	5	1	N-Term(iTRAQ4plex); K10(iTRAQ4plex)	HCD	1.0000	0.0000	1	1	11580	Unique	Used	0.237	9.491E2	3.041E2	304	3.72	0.00	0	34	300	3	483.28049	1447.82691	1.54	65.31	6227	6227	MS2	16/36	Glioma_Nuclear_prep_Gradeall_repeat_4plex.raw
117190174		High	vDSLLENLEk	Unambiguous	5	1	N-Term(iTRAQ4plex); K10(iTRAQ4plex)	HCD	1.0000	0.0000	1	1	11591	Unique	Used	0.417	1.434E4	8.094E3	288	4.16	0.00	0	52	300	2	724.41766	1447.82805	2.33	65.46	6240	6240	MS2	17/18	Glioma_Nuclear_prep_Gradeall_repeat_4plex.raw
117190174		High	sEEEQSSSSVk	Unambiguous	2	1	N-Term(iTRAQ4plex); K11(iTRAQ4plex)	HCD	1.0000	0.0000	1	1	6651	Unique	Used	0.269	6.120E3	2.226E3	92	3.90	0.00	0	8	300	2	742.86237	1484.71745	-9.72	11.65	900	900	MS2	20/20	Glioma_Nuclear_prep_Gradeall_repeat_4plex.raw
117190174	High		vFIGNLNTLVVk	Unambiguous	5	1	N-Term(iTRAQ4plex); K12(iTRAQ4plex)	HCD	1.0000	0.0000	1	1	11149	Unique	Used	0.547	1.430E5	9.838E4	93	5.62	0.00	0	43	33	2	802.99988	1604.99248	-3.34	49.63	5884	5884	MS2	22/22	Glioma_Nuclear_prep_Gradeall_4plex.raw
117190174		High	vFIGNLNTLVVk	Unambiguous	5	1	N-Term(iTRAQ4plex); K12(iTRAQ4plex)	HCD	1.0000	0.0000	1	1	11991	Unique	Used	0.442	1.636E4	9.794E3	79	5.66	0.00	0	18	300	2	803.00543	1605.00359	3.58	71.86	6767	6767	MS2	22/22	Glioma_Nuclear_prep_Gradeall_repeat_4plex.raw
117190174		High	vFIGNLNTLVVk	Unambiguous	5	1	N-Term(iTRAQ4plex); K12(iTRAQ4plex)	HCD	1.0000	0.0000	1	1	11992	Unique	Used	0.596	6.608E2	5.335E2	88	4.02	0.00	0	19	300	3	535.67163	1605.00034	1.56	71.87	6768	6768	MS2	17/44	Glioma_Nuclear_prep_Gradeall_repeat_4plex.raw
117190174	High		mIAGQVLDINLAAEPk	Unambiguous	2	1	N-Term(iTRAQ4plex); K16(iTRAQ4plex)	HCD	1.0000	0.0000	1	1	11260	Unique	Used	0.363	6.350E4	2.903E4	107	6.98	0.00	0	14	32	2	986.05878	1971.11028	-4.32	50.52	6005	6005	MS2	29/30	Glioma_Nuclear_prep_Gradeall_4plex.raw
117190174	High		mIAGQVLDINLAAEPk	Unambiguous	2	1	N-Term(iTRAQ4plex); K16(iTRAQ4plex)	HCD	1.0000	0.0000	1	1	11246	Unique	Used	0.377	1.893E4	8.971E3	127	4.90	0.00	0	9	9	3	657.71191	1971.12119	1.21	50.41	5990	5990	MS2	26/60	Glioma_Nuclear_prep_Gradeall_4plex.raw
117190174	High		mIAGQVLDINLAAEPk	Unambiguous	2	1	N-Term(iTRAQ4plex); K16(iTRAQ4plex)	HCD	1.0000	0.0000	1	1	11168	Unique	Used	0.458	1.293E5	7.458E4	102	6.63	0.00	0	30	21	2	986.06342	1971.11955	0.38	49.79	5905	5905	MS2	29/30	Glioma_Nuclear_prep_Gradeall_4plex.raw
117190174	High		mIAGQVLDINLAAEPk	Unambiguous	2	1	N-Term(iTRAQ4plex); K16(iTRAQ4plex)	HCD	1.0000	0.0000	1	1	11370	Unique	Used	0.648	2.133E4	1.739E4	136	6.24	0.00	0	44	109	2	986.05975	1971.11223	-3.33	51.48	6125	6125	MS2	28/30	Glioma_Nuclear_prep_Gradeall_4plex.raw

117190174	High		mIAGQVLDINLAAEPk	Unambiguous	2	1	N-Term(iTRAQ4plex); K16(iTRAQ4plex)	HCD	1.0000	0.0000	1	1	11157	Unique	Used	0.722	1.018E4	9.236E3	138	4.28	0.00	0	38	24	3	657.70831	1971.11039	-4.27	49.69	5893	5893	MS2	20/60	Glioma_Nuclear_prep_Gradeall_4plex.raw
117190174		High	mIAGQVLDINLAAEPk	Unambiguous	2	1	N-Term(iTRAQ4plex); K16(iTRAQ4plex)	HCD	1.0000	0.0000	1	1	11953	Unique	Used	0.765	3.213E2	3.327E2	134	3.46	0.00	0	39	300	3	657.71063	1971.11734	-0.74	71.18	6713	6713	MS2	23/60	Glioma_Nuclear_prep_Gradeall_repeat_4plex.raw
117190174	High		lkGDDLQAIk	Unambiguous	5	1	N-Term(iTRAQ4plex); K2(iTRAQ4plex); K10(iTRAQ4plex)	HCD	1.0000	0.0000	1	1	8273	Unique	Used	0.255	4.969E5	1.592E5	182	3.87	0.00	1	13	1	3	511.64252	1532.91300	-15.82	28.29	2749	2749	MS2	20/36	Glioma_Nuclear_prep_Gradeall_4plex.raw
117190174	High		lkGDDLQAIk	Unambiguous	5	1	N-Term(iTRAQ4plex); K2(iTRAQ4plex); K10(iTRAQ4plex)	HCD	1.0000	0.0000	1	1	8264	Unique	Used	0.331	3.822E5	1.589E5	193	3.27	0.00	1	29	8	2	766.95898	1532.91069	-17.33	28.23	2739	2739	MS2	17/18	Glioma_Nuclear_prep_Gradeall_4plex.raw
117190174		High	lkGDDLQAIk	Unambiguous	5	1	N-Term(iTRAQ4plex); K2(iTRAQ4plex); K10(iTRAQ4plex)	HCD	1.0000	0.0000	1	1	8727	Unique	Used	0.179	3.807E4	9.252E3	163	3.59	0.00	1	11	13	3	511.64398	1532.91739	-12.95	30.89	3092	3092	MS2	18/36	Glioma_Nuclear_prep_Gradeall_repeat_4plex.raw
117190174		High	lkGDDLQAIk	Unambiguous	5	1	N-Term(iTRAQ4plex); K2(iTRAQ4plex); K10(iTRAQ4plex)	HCD	1.0000	0.0000	1	1	8734	Unique	Used	0.234	4.442E4	1.407E4	178	3.31	0.00	1	13	51	2	766.96307	1532.91887	-11.99	30.93	3099	3099	MS2	17/18	Glioma_Nuclear_prep_Gradeall_repeat_4plex.raw
117190174	High		qkVDSLLENLEk	Unambiguous	5	1	N-Term(iTRAQ4plex); K2(iTRAQ4plex); K12(iTRAQ4plex)	HCD	1.0000	0.0000	1	1	9946	Unique	Used	0.286	2.833E5	1.020E5	157	4.74	0.00	1	26	11	2	924.53107	1848.05486	-13.76	40.47	4571	4571	MS2	22/22	Glioma_Nuclear_prep_Gradeall_4plex.raw
117190174	High		qkVDSLLENLEk	Unambiguous	5	1	N-Term(iTRAQ4plex); K2(iTRAQ4plex); K12(iTRAQ4plex)	HCD	1.0000	0.0000	1	1	9943	Unique	Used	0.359	7.636E4	3.445E4	155	4.45	0.00	1	37	4	3	616.68970	1848.05454	-13.93	40.45	4568	4568	MS2	18/44	Glioma_Nuclear_prep_Gradeall_4plex.raw
117190174		High	qkVDSLLENLEk	Unambiguous	5	1	N-Term(iTRAQ4plex); K2(iTRAQ4plex); K12(iTRAQ4plex)	HCD	1.0000	0.0000	1	1	10897	Unique	Used	0.281	4.747E3	1.806E3	100	5.09	0.00	1	29	300	2	924.54694	1848.08660	3.41	55.90	5420	5420	MS2	22/22	Glioma_Nuclear_prep_Gradeall_repeat_4plex.raw
117190174		High	qkVDSLLENLEk	Unambiguous	5	1	N-Term(iTRAQ4plex); K2(iTRAQ4plex); K12(iTRAQ4plex)	HCD	1.0000	0.0000	1	1	10879	Unique	Used	0.390	2.118E3	1.118E3	98	5.02	0.00	1	50	300	3	616.70001	1848.08548	2.81	55.67	5400	5400	MS2	17/44	Glioma_Nuclear_prep_Gradeall_repeat_4plex.raw
117190174	High		gDDLQAIk	Unambiguous	5	1	N-Term(iTRAQ4plex); K8(iTRAQ4plex)	HCD	1.0000	0.0000	1	1	7901	Unique	Used	0.604	1.507E5	1.145E5	308	3.15	0.00	0	15	7	2	574.33026	1147.65325	-2.53	25.67	2346	2346	MS2	14/14	Glioma_Nuclear_prep_Gradeall_4plex.raw
117190174		High	gDDLQAIk	Unambiguous	5	1	N-Term(iTRAQ4plex); K8(iTRAQ4plex)	HCD	1.0000	0.0000	1	1	8147	Unique	Used	0.300	6.861E4	2.784E4	278	2.97	0.00	0	13	45	2	574.33118	1147.65508	-0.93	25.22	2481	2481	MS2	14/14	Glioma_Nuclear_prep_Gradeall_repeat_4plex.raw
117190174	High		sDVEAIFSk	Unambiguous	5	1	N-Term(iTRAQ4plex); K9(iTRAQ4plex)	HCD	1.0000	0.0000	1	1	9533	Unique	Used	0.611	4.734E5	3.635E5	231	3.67	0.00	0	43	8	2	642.35071	1283.69414	-11.25	37.49	4123	4123	MS2	15/16	Glioma_Nuclear_prep_Gradeall_4plex.raw
117190174		High	sDVEAIFSk	Unambiguous	5	1	N-Term(iTRAQ4plex); K9(iTRAQ4plex)	HCD	1.0000	0.0000	1	1	10143	Unique	Used	0.589	2.030E4	1.620E4	226	3.16	0.00	0	43	300	2	642.36163	1283.71599	5.77	46.80	4608	4608	MS2	15/16	Glioma_Nuclear_prep_Gradeall_repeat_4plex.raw
117190174	High		gDDQLELIkDDEk	Unambiguous	2	1	N-Term(iTRAQ4plex); K9(iTRAQ4plex); K13(iTRAQ4plex)	HCD	1.0000	0.0000	1	1	8874	Unique	Used	0.355	6.100E4	2.727E4	159	4.57	0.00	1	0	14	2	975.51056	1950.01384	-13.02	32.62	3402	3402	MS2	21/24	Glioma_Nuclear_prep_Gradeall_4plex.raw
117190174	High		mIAGQVLDINLAAEPk	Unambiguous	2	1	N-Term(iTRAQ4plex); M1(Oxidation); K16(iTRAQ4plex)	HCD	1.0000	0.0000	1	1	10573	Unique	Used	0.375	1.322E5	6.237E4	192	5.83	0.00	0	7	13	2	994.04657	1987.08586	-14.02	45.19	5256	5256	MS2	25/30	Glioma_Nuclear_prep_Gradeall_4plex.raw
117190174	High		mIAGQVLDINLAAEPk	Unambiguous	2	1	N-Term(iTRAQ4plex); M1(Oxidation); K16(iTRAQ4plex)	HCD	1.0000	0.0000	1	1	10594	Unique	Used	0.523	3.993E4	2.624E4	143	4.48	0.00	0	15	5	3	663.03400	1987.08744	-13.22	45.35	5279	5279	MS2	25/60	Glioma_Nuclear_prep_Gradeall_4plex.raw
117190174		High	mIAGQVLDINLAAEPk	Unambiguous	2	1	N-Term(iTRAQ4plex); M1(Oxidation); K16(iTRAQ4plex)	HCD	1.0000	0.0000	1	1	11376	Unique	Used	0.233	9.809E2	3.092E2	142	4.64	0.00	0	45	300	3	663.04395	1987.11728	1.80	62.47	5984	5984	MS2	23/60	Glioma_Nuclear_prep_Gradeall_repeat_4plex.raw
117190174		High	mIAGQVLDINLAAEPk	Unambiguous	2	1	N-Term(iTRAQ4plex); M1(Oxidation); K16(iTRAQ4plex)	HCD	1.0000	0.0000	1	1	11389	Unique	Used	0.359	2.519E3	1.224E3	149	6.14	0.00	0	20	300	2	994.06183	1987.11638	1.34	62.64	5999	5999	MS2	25/30	Glioma_Nuclear_prep_Gradeall_repeat_4plex.raw
117190174	High		sAAEmYGSSFDLDYDFQR	Unambiguous	1	1	N-Term(iTRAQ4plex); M5(Oxidation)	HCD	1.0000	0.0000	1	1	10879	Unique	Used	0.474	4.129E4	2.460E4	16	5.78	0.00	0	33	49	2	1131.47522	2261.94316	-15.32	47.51	5589	5589	MS2	22/34	Glioma_Nuclear_prep_Gradeall_4plex.raw
118498359	NP_056474.2	RSL1D1	26156	ribosomal L1 domain-containing protein 1 [Homo sapiens]	12.24	1	3	6	1.876	2;4	15.1	23.07	490	54.9	10.13																			
118498359	High		sDSEDIcLFTk	Unambiguous	1	1	N-Term(iTRAQ4plex); C7(Methylthio); K11(iTRAQ4plex)	HCD	1.0000	0.0000	1	1	10550	Unique	Used	1.781	6.397E4	1.433E5	56	4.17	0.00	0	37	20	2	796.37201	1591.73674	-13.76	45.02	5231	5231	MS2	19/20	Glioma_Nuclear_prep_Gradeall_4plex.raw
118498359		High	sDSEDIcLFTk	Unambiguous	1	1	N-Term(iTRAQ4plex); C7(Methylthio); K11(iTRAQ4plex)	HCD	1.0000	0.0000	1	1	11363	Unique	Used	1.736	2.893E3	6.801E3	73	4.62	0.00	0	29	300	2	796.38434	1591.76140	1.73	62.30	5969	5969	MS2	19/20	Glioma_Nuclear_prep_Gradeall_repeat_4plex.raw
118498359	High		dDVAPESGDTTVk	Unambiguous	1	1	N-Term(iTRAQ4plex); K13(iTRAQ4plex)	HCD	1.0000	0.0000	1	1	7598	Unique	Used	2.319	4.258E4	1.242E5	173	4.09	0.00	0	0	14	2	811.41138	1621.81548	-0.31	23.51	2017	2017	MS2	20/24	Glioma_Nuclear_prep_Gradeall_4plex.raw
118498359		High	dDVAPESGDTTVk	Unambiguous	1	1	N-Term(iTRAQ4plex); K13(iTRAQ4plex)	HCD	1.0000	0.0000	1	1	7604	Unique	Used	1.978	4.675E3	1.253E4	167	4.48	0.00	0	5	123	2	811.40790	1621.80852	-4.60	19.97	1905	1905	MS2	22/24	Glioma_Nuclear_prep_Gradeall_repeat_4plex.raw
118498359		High	aLPASETPk	Unambiguous	1	1	N-Term(iTRAQ4plex); K9(iTRAQ4plex)	HCD	1.0000	0.0000	1	1	7495	Unique	Used	1.481	2.876E4	5.769E4	223	3.38	0.00	0	37	129	2	601.35614	1201.70500	1.59	19.07	1790	1790	MS2	14/16	Glioma_Nuclear_prep_Gradeall_repeat_4plex.raw
118498359		High	aLPASETPk	Unambiguous	1	1	N-Term(iTRAQ4plex); K9(iTRAQ4plex)	HCD	1.0000	0.0000	1	1	7515	Unique	Used	1.971	3.232E4	8.628E4	215	3.35	0.00	0	24	62	2	601.34827	1201.68926	-11.52	19.24	1811	1811	MS2	14/16	Glioma_Nuclear_prep_Gradeall_repeat_4plex.raw
118572613	NP_057417.3	SRRM2	23524	serine/arginine repetitive matrix protein 2 [Homo sapiens]	4.72	1	7	8	0.550	4;4	24.7	27.07	2752	299.4	12.06																			
118572613	High		tAVAPSAVNLADPR	Unambiguous	1	1	N-Term(iTRAQ4plex)	HCD	1.0000	0.0000	1	1	8825	Unique	Used	0.400	8.508E4	4.285E4	285	4.03	0.00	0	4	20	2	763.41479	1525.82231	-15.23	32.27	3349	3349	MS2	19/26	Glioma_Nuclear_prep_Gradeall_4plex.raw
118572613	High		mSQVPAPVPLMSLR	Unambiguous	1	1	N-Term(iTRAQ4plex)	HCD	1.0000	0.0000	1	1	10950	Unique	Used	0.641	3.102E4	2.501E4	315	3.39	0.00	0	54	108	2	835.45612	1669.90495	-12.05	48.06	5666	5666	MS2	12/26	Glioma_Nuclear_prep_Gradeall_4plex.raw
118572613	High		sPGMLEPLGSSR	Unambiguous	1	1	N-Term(iTRAQ4plex)	HCD	1.0000	0.0000	1	1	8888	Unique	Used	0.666	2.032E5	1.703E5	195	2.93	0.00	0	45	17	2	687.85083	1374.69438	-16.33	32.72	3417	3417	MS2	14/22	Glioma_Nuclear_prep_Gradeall_4plex.raw
118572613		High	tPAIPTAVNLADSR	Unambiguous	1	1	N-Term(iTRAQ4plex)	HCD	1.0000	0.0000	1	1	10344	Unique	Used	0.427	3.697E3	2.138E3	305	3.24	0.00	0	39	300	2	785.44006	1569.87285	0.70	49.18	4822	4822	MS2	14/26	Glioma_Nuclear_prep_Gradeall_repeat_4plex.raw
118572613		High	aAFGISDSYVDGSFDPQR	Unambiguous	1	1	N-Term(iTRAQ4plex)	HCD	1.0000	0.0000	1	1	11399	Unique	Used	0.447	1.257E3	7.609E2	67	5.75	0.00	0	7	300	2	1082.00952	2163.01177	0.27	62.78	6011	6011	MS2	28/36	Glioma_Nuclear_prep_Gradeall_repeat_4plex.raw
118572613		High	tAVAPSAVNLADPR	Unambiguous	1	1	N-Term(iTRAQ4plex)	HCD	1.0000	0.0000	1	1	9236	Unique	Used	0.459	5.281E3	3.283E3	275	3.84	0.00	0	43	300	2	763.42896	1525.85063	3.34	36.36	3632	3632	MS2	17/26	Glioma_Nuclear_prep_Gradeall_repeat_4plex.raw
118572613		High	sSTGPEPPAPTPLLAER	Unambiguous	1	1	N-Term(iTRAQ4plex)	HCD	1.0000	0.0000	1	1	9809	Unique	Used	0.759	3.345E3	3.439E3	173	4.73	0.00	0	9	300	2	932.50446	1864.00163	4.45	42.76	4244	4244	MS2	25/32	Glioma_Nuclear_prep_Gradeall_repeat_4plex.raw
118572613	High		sEEPAGQILSHLSSELk	Unambiguous	1	1	N-Term(iTRAQ4plex); K17(iTRAQ4plex)	HCD	1.0000	0.0000	1	1	11302	Unique	Used	0.655	5.101E3	4.203E3	132	5.27	0.00	0	39	115	3	705.04993	2113.13523	-1.31	50.88	6050	6050	MS2	22/64	Glioma_Nuclear_prep_Gradeall_4plex.raw
118601081	NP_001073027.1	HNRNPUL2	221092	heterogeneous nuclear ribonucleoprotein U-like protein 2 [Homo sapiens]	23.96	1	14	23	0.244	13;10	34.7	76.08	747	85.1	4.91																			
118601081	High		lQEALDAEMLEDEAGGGGAGPGGAck	Unambiguous	1	1	N-Term(iTRAQ4plex); C25(Methylthio); K26(iTRAQ4plex)	HCD	1.0000	0.0000	1	1	11353	Unique	Used	0.242	8.650E3	2.630E3	64	9.04	0.00	0	15	29	3	927.43158	2780.28019	0.75	51.35	6107	6107	MS2	39/100	Glioma_Nuclear_prep_Gradeall_4plex.raw
118601081	High		ePFFPPPEEFVFIHAVPVEER	Unambiguous	1	1	N-Term(iTRAQ4plex)	HCD	1.0000	0.0000	1	1	12782	Unique	Used	0.128	1.261E3	2.032E2	125	7.71	0.00	0	9	300	3	929.14111	2785.40879	3.33	66.11	7693	7693	MS2	30/84	Glioma_Nuclear_prep_Gradeall_4plex.raw
118601081	High		ePFFPPPEEFVFIHAVPVEER	Unambiguous	1	1	N-Term(iTRAQ4plex)	HCD	1.0000	0.0000	1	1	12730	Unique	Used	0.133	2.730E3	4.567E2	125	7.95	0.00	0	5	258	3	929.14105	2785.40860	3.26	65.38	7631	7631	MS2	28/84	Glioma_Nuclear_prep_Gradeall_4plex.raw
118601081	High		yNVLGAETVLNQMR	Unambiguous	1	1	N-Term(iTRAQ4plex)	HCD	1.0000	0.0000	1	1	11296	Unique	Used	0.166	9.903E4	2.064E4	239	5.17	0.00	0	24	51	2	876.46490	1751.92253	-0.34	50.83	6044	6044	MS2	22/26	Glioma_Nuclear_prep_Gradeall_4plex.raw
118601081		High	nYYGYQGYR	Unambiguous	1	1	N-Term(iTRAQ4plex)	HCD	1.0000	0.0000	1	1	8674	Unique	Used	0.244	3.760E4	1.243E4	89	2.94	0.00	0	23	211	2	664.31488	1327.62248	2.81	30.40	3036	3036	MS2	13/16	Glioma_Nuclear_prep_Gradeall_repeat_4plex.raw
118601081	High		cDYMDEVTYGELEk	Unambiguous	1	1	N-Term(iTRAQ4plex); C1(Methylthio); K14(iTRAQ4plex)	HCD	1.0000	0.0000	1	1	11032	Unique	Used	0.200	4.841E4	1.220E4	30	6.28	0.00	0	23	61	2	1014.94659	2028.88591	0.78	48.68	5755	5755	MS2	22/26	Glioma_Nuclear_prep_Gradeall_4plex.raw
118601081	High		cDYMDEVTYGELEkEEAQPIVTk	Unambiguous	1	1	N-Term(iTRAQ4plex); C1(Methylthio); K14(iTRAQ4plex); K23(iTRAQ4plex)	HCD	1.0000	0.0000	1	1	11240	Unique	Used	0.390	7.133E1	3.497E1	84	6.62	0.00	1	21	20	4	792.88647	3168.52407	2.80	50.36	5983	5983	MS2	30/132	Glioma_Nuclear_prep_Gradeall_4plex.raw
118601081	High		dLLVQQASQcLSk	Unambiguous	1	1	N-Term(iTRAQ4plex); C10(Methylthio); K13(iTRAQ4plex)	HCD	1.0000	0.0000	1	1	11239	Unique	Used	0.224	1.114E5	3.143E4	242	5.68	0.00	0	20	33	2	883.97406	1766.94084	1.41	50.36	5982	5982	MS2	20/24	Glioma_Nuclear_prep_Gradeall_4plex.raw
118601081	High		dLLVQQASQcLSk	Unambiguous	1	1	N-Term(iTRAQ4plex); C10(Methylthio); K13(iTRAQ4plex)	HCD	1.0000	0.0000	1	1	11236	Unique	Used	0.328	1.750E4	7.220E3	196	6.28	0.00	0	30	15	3	589.65155	1766.94010	0.99	50.34	5979	5979	MS2	22/48	Glioma_Nuclear_prep_Gradeall_4plex.raw
118601081		High	dLLVQQASQcLSk	Unambiguous	1	1	N-Term(iTRAQ4plex); C10(Methylthio); K13(iTRAQ4plex)	HCD	1.0000	0.0000	1	1	11979	Unique	Used	0.255	6.196E2	2.141E2	241	4.92	0.00	0	19	300	3	589.65210	1766.94175	1.92	71.65	6751	6751	MS2	23/48	Glioma_Nuclear_prep_Gradeall_repeat_4plex.raw
118601081		High	dLLVQQASQcLSk	Unambiguous	1	1	N-Term(iTRAQ4plex); C10(Methylthio); K13(iTRAQ4plex)	HCD	1.0000	0.0000	1	1	11982	Unique	Used	0.416	2.553E3	1.437E3	238	4.46	0.00	0	18	300	2	883.97510	1766.94292	2.59	71.70	6755	6755	MS2	19/24	Glioma_Nuclear_prep_Gradeall_repeat_4plex.raw
118601081		High	eGcTEVSLLR	Unambiguous	1	1	N-Term(iTRAQ4plex); C3(Methylthio)	HCD	1.0000	0.0000	1	1	10444	Unique	Used	0.281	1.101E4	4.190E3	147	3.64	0.00	0	36	300	2	648.82544	1296.64360	2.10	50.35	4928	4928	MS2	17/18	Glioma_Nuclear_prep_Gradeall_repeat_4plex.raw
118601081		High	aLLPHVLck	Unambiguous	1	1	N-Term(iTRAQ4plex); C8(Methylthio); K9(iTRAQ4plex)	HCD	1.0000	0.0000	1	1	10773	Unique	Used	0.321	9.844E3	4.285E3	218	3.05	0.00	0	33	300	2	664.39722	1327.78716	2.92	54.33	5283	5283	MS2	15/16	Glioma_Nuclear_prep_Gradeall_repeat_4plex.raw
118601081	High		yGGQPLFSEk	Unambiguous	1	1	N-Term(iTRAQ4plex); K10(iTRAQ4plex)	HCD	1.0000	0.0000	1	1	8931	Unique	Used	0.200	4.446E5	1.117E5	196	2.93	0.00	0	25	6	2	707.37408	1413.74089	-14.70	33.03	3464	3464	MS2	17/18	Glioma_Nuclear_prep_Gradeall_4plex.raw
118601081	High		gLEEPEMDPk	Unambiguous	1	1	N-Term(iTRAQ4plex); K10(iTRAQ4plex)	HCD	1.0000	0.0000	1	1	8325	Unique	Used	0.276	2.645E5	9.192E4	106	3.42	0.00	0	13	7	2	716.85431	1432.70134	-15.30	28.66	2805	2805	MS2	17/18	Glioma_Nuclear_prep_Gradeall_4plex.raw
118601081		High	yGGQPLFSEk	Unambiguous	1	1	N-Term(iTRAQ4plex); K10(iTRAQ4plex)	HCD	1.0000	0.0000	1	1	9483	Unique	Used	0.184	3.905E4	9.713E3	237	3.57	0.00	0	22	147	2	707.38641	1413.76555	2.75	39.07	3894	3894	MS2	17/18	Glioma_Nuclear_prep_Gradeall_repeat_4plex.raw
118601081		High	gLEEPEMDPk	Unambiguous	1	1	N-Term(iTRAQ4plex); K10(iTRAQ4plex)	HCD	1.0000	0.0000	1	1	8632	Unique	Used	0.307	1.517E4	6.319E3	166	3.48	0.00	0	32	245	2	716.86780	1432.72832	3.53	29.96	2992	2992	MS2	17/18	Glioma_Nuclear_prep_Gradeall_repeat_4plex.raw
118601081	High		sGDETPGSEVPGDk	Unambiguous	1	1	N-Term(iTRAQ4plex); K14(iTRAQ4plex)	HCD	1.0000	0.0000	1	1	7500	Unique	Used	0.370	1.575E5	7.337E4	103	4.29	0.00	0	0	10	2	831.90533	1662.80339	-1.66	22.82	1911	1911	MS2	21/26	Glioma_Nuclear_prep_Gradeall_4plex.raw
118601081	High		aNFSLPEk	Unambiguous	1	1	N-Term(iTRAQ4plex); K8(iTRAQ4plex)	HCD	1.0000	0.0000	1	1	8797	Unique	Used	0.341	2.792E5	1.198E5	329	2.89	0.00	0	43	15	2	597.33081	1193.65434	-18.88	32.07	3319	3319	MS2	13/14	Glioma_Nuclear_prep_Gradeall_4plex.raw
118601081		High	aNFSLPEk	Unambiguous	1	1	N-Term(iTRAQ4plex); K8(iTRAQ4plex)	HCD	1.0000	0.0000	1	1	9336	Unique	Used	0.267	3.260E4	1.179E4	335	3.23	0.00	0	65	300	2	597.34332	1193.67937	2.09	37.47	3737	3737	MS2	13/14	Glioma_Nuclear_prep_Gradeall_repeat_4plex.raw
118601081		High	eEAQPIVTk	Unambiguous	1	1	N-Term(iTRAQ4plex); K9(iTRAQ4plex)	HCD	1.0000	0.0000	1	1	7637	Unique	Used	0.215	2.652E4	7.712E3	223	4.09	0.00	0	35	131	2	651.87506	1302.74285	-6.09	20.28	1941	1941	MS2	16/16	Glioma_Nuclear_prep_Gradeall_repeat_4plex.raw
118601081	High		yNVLGAETVLNQmR	Unambiguous	1	1	N-Term(iTRAQ4plex); M13(Oxidation)	HCD	1.0000	0.0000	1	1	10189	Unique	Used	0.151	2.023E5	3.837E4	189	3.66	0.00	0	10	16	2	884.45190	1767.89653	-12.17	42.27	4836	4836	MS2	19/26	Glioma_Nuclear_prep_Gradeall_4plex.raw
118601081		High	yNVLGAETVLNQmR	Unambiguous	1	1	N-Term(iTRAQ4plex); M13(Oxidation)	HCD	1.0000	0.0000	1	1	10985	Unique	Used	0.102	8.292E3	1.144E3	222	3.89	0.00	0	8	300	2	884.46552	1767.92375	3.23	56.96	5515	5515	MS2	20/26	Glioma_Nuclear_prep_Gradeall_repeat_4plex.raw
119395750	NP_006112.3	KRT1	3848	keratin, type II cytoskeletal 1 [Homo sapiens]	8.54	2	4	6	1.936	3;3	25.5	20.68	644	66.0	8.12																			
119395750		High	yEELQITAGR	Unambiguous	2	1	N-Term(iTRAQ4plex)	HCD	1.0000	0.0000	1	1	9492	Unique	Used	1.741	5.742E3	1.354E4	301	3.25	0.00	0	53	300	2	662.35535	1323.70342	0.67	39.16	3903	3903	MS2	15/18	Glioma_Nuclear_prep_Gradeall_repeat_4plex.raw
119395750	High		sLNNQFASFIDk	Unambiguous	1	1	N-Term(iTRAQ4plex); K12(iTRAQ4plex)	HCD	1.0000	0.0000	1	1	10582	Unique	Used	2.132	6.582E4	1.764E5	189	5.21	0.00	0	18	18	2	836.43958	1671.87187	-13.53	45.26	5266	5266	MS2	22/22	Glioma_Nuclear_prep_Gradeall_4plex.raw
119395750	High		sLDLDSIIAEVk	Unambiguous	1	1	N-Term(iTRAQ4plex); K12(iTRAQ4plex)	HCD	1.0000	0.0000	1	1	12297	Unique	Used	2.698	1.398E4	4.744E4	212	5.44	0.00	0	30	115	2	795.96283	1590.91838	-0.59	60.01	7139	7139	MS2	22/22	Glioma_Nuclear_prep_Gradeall_4plex.raw
119395750	High		sLDLDSIIAEVk	Unambiguous	1	1	N-Term(iTRAQ4plex); K12(iTRAQ4plex)	HCD	1.0000	0.0000	1	1	12291	Unique	Used	2.973	9.011E2	3.370E3	221	4.34	0.00	0	24	135	3	530.97754	1590.91806	-0.79	59.94	7132	7132	MS2	20/44	Glioma_Nuclear_prep_Gradeall_4plex.raw
119395750		High	sLNNQFASFIDk	Unambiguous	1	1	N-Term(iTRAQ4plex); K12(iTRAQ4plex)	HCD	1.0000	0.0000	1	1	11469	Unique	Used	1.720	1.531E3	3.567E3	259	4.91	0.00	0	39	300	2	836.45258	1671.89787	2.03	63.75	6094	6094	MS2	21/22	Glioma_Nuclear_prep_Gradeall_repeat_4plex.raw
119395750		High	aQYEDIAQk	Unambiguous	1	1	N-Term(iTRAQ4plex); K9(iTRAQ4plex)	HCD	1.0000	0.0000	1	1	8188	Unique	Used	1.716	1.006E4	2.338E4	231	3.88	0.00	0	22	249	2	677.36493	1353.72258	-1.99	25.60	2524	2524	MS2	16/16	Glioma_Nuclear_prep_Gradeall_repeat_4plex.raw
126032350	NP_001075109.1	PRKDC	5591	DNA-dependent protein kinase catalytic subunit isoform 2 [Homo sapiens]	4.44	2	12	12	0.642	7;5	17.1	61.98	4097	465.2	7.17																			
126032350	High		lAGANPAVITcDELLGHEk	Unambiguous	2	1	N-Term(iTRAQ4plex); C11(Methylthio); K20(iTRAQ4plex)	HCD	1.0000	0.0000	1	1	12736	Unique	Used	0.639	8.508E2	6.843E2	120	8.50	0.00	0	44	300	3	800.09808	2398.27970	3.48	65.44	7637	7637	MS2	33/76	Glioma_Nuclear_prep_Gradeall_4plex.raw
126032350	High		dPTVHDDVLELEMDELNR	Unambiguous	2	1	N-Term(iTRAQ4plex)	HCD	1.0000	0.0000	1	1	11103	Unique	Used	0.724	7.150E3	6.508E3	78	6.91	0.00	0	19	27	3	762.03204	2284.08158	-3.02	49.25	5833	5833	MS2	27/68	Glioma_Nuclear_prep_Gradeall_4plex.raw
126032350		High	cGAALAGHQLIR	Unambiguous	2	1	N-Term(iTRAQ4plex); C1(Methylthio)	HCD	1.0000	0.0000	1	1	9663	Unique	Used	0.669	1.752E3	1.587E3	203	3.70	0.00	0	72	300	3	467.25314	1399.74488	2.10	41.11	4090	4090	MS2	23/44	Glioma_Nuclear_prep_Gradeall_repeat_4plex.raw
126032350	High		gLSSLLcNFTk	Unambiguous	2	1	N-Term(iTRAQ4plex); C7(Methylthio); K11(iTRAQ4plex)	HCD	1.0000	0.0000	1	1	12323	Unique	Used	0.578	1.575E4	1.145E4	265	4.91	0.00	0	46	284	2	758.90747	1516.80766	-1.95	60.27	7167	7167	MS2	18/20	Glioma_Nuclear_prep_Gradeall_4plex.raw
126032350		High	lPVLAGcLk	Unambiguous	2	1	N-Term(iTRAQ4plex); C7(Methylthio); K9(iTRAQ4plex)	HCD	1.0000	0.0000	1	1	12220	Unique	Used	0.357	4.573E3	2.209E3	311	2.92	0.00	0	40	300	2	624.37701	1247.74675	0.73	76.62	7138	7138	MS2	13/16	Glioma_Nuclear_prep_Gradeall_repeat_4plex.raw
126032350		High	lAAVVSAck	Unambiguous	2	1	N-Term(iTRAQ4plex); C8(Methylthio); K9(iTRAQ4plex)	HCD	1.0000	0.0000	1	1	11043	Unique	Used	0.608	4.618E3	3.801E3	287	3.08	0.00	0	52	300	2	598.34210	1195.67693	-1.02	57.72	5581	5581	MS2	14/16	Glioma_Nuclear_prep_Gradeall_repeat_4plex.raw
126032350	High		yPEETLSLMTk	Unambiguous	2	1	N-Term(iTRAQ4plex); K11(iTRAQ4plex)	HCD	1.0000	0.0000	1	1	10277	Unique	Used	0.644	7.800E4	6.322E4	225	3.18	0.00	0	28	30	2	800.41931	1599.83135	-14.32	42.91	4931	4931	MS2	16/20	Glioma_Nuclear_prep_Gradeall_4plex.raw
126032350	High		qLFSSLFSGILk	Unambiguous	2	1	N-Term(iTRAQ4plex); K12(iTRAQ4plex)	HCD	1.0000	0.0000	1	1	12954	Unique	Used	0.771	2.447E3	2.373E3	141	4.69	0.00	0	35	300	2	814.48914	1627.97099	2.95	68.88	7917	7917	MS2	21/22	Glioma_Nuclear_prep_Gradeall_4plex.raw
126032350	High		lSDFNDITNMLLLk	Unambiguous	2	1	N-Term(iTRAQ4plex); K14(iTRAQ4plex)	HCD	1.0000	0.0000	1	1	12961	Unique	Used	0.632	2.162E3	1.719E3	214	5.53	0.00	0	34	300	2	963.03888	1925.07048	2.50	69.06	7930	7930	MS2	24/26	Glioma_Nuclear_prep_Gradeall_4plex.raw
126032350		High	eIFNFVLk	Unambiguous	2	1	N-Term(iTRAQ4plex); K8(iTRAQ4plex)	HCD	1.0000	0.0000	1	1	12143	Unique	Used	0.671	2.630E3	2.390E3	239	3.63	0.00	0	41	300	2	649.39178	1297.77629	0.33	75.01	7012	7012	MS2	14/14	Glioma_Nuclear_prep_Gradeall_repeat_4plex.raw
126032350		High	sVGPDFGk	Unambiguous	2	1	N-Term(iTRAQ4plex); K8(iTRAQ4plex)	HCD	1.0000	0.0000	1	1	8160	Unique	Used	0.759	2.706E4	2.783E4	291	3.24	0.00	0	55	233	2	547.80621	1094.60515	-3.03	25.32	2494	2494	MS2	11/14	Glioma_Nuclear_prep_Gradeall_repeat_4plex.raw
126032350	High		iPALDLLIk	Unambiguous	2	1	N-Term(iTRAQ4plex); K9(iTRAQ4plex)	HCD	1.0000	0.0000	1	1	12068	Unique	Used	0.576	6.681E3	4.836E3	15	3.40	0.00	0	79	300	2	642.43701	1283.86675	9.83	57.65	6886	6886	MS2	13/16	Glioma_Nuclear_prep_Gradeall_4plex.raw
148596949	NP_004732.2	NOLC1	9221	nucleolar and coiled-body phosphoprotein 1 isoform 2 [Homo sapiens]	9.16	3	6	7	0.583	2;5	12.9	20.27	699	73.6	9.47																			
148596949	High		vVPSDLYPLVLGFLR	Unambiguous	3	1	N-Term(iTRAQ4plex)	HCD	1.0000	0.0000	1	1	13146	Unique	Used	0.545	2.229E3	1.527E3	53	3.37	0.00	0	15	300	2	916.54718	1832.08708	3.71	78.34	8561	8561	MS2	16/28	Glioma_Nuclear_prep_Gradeall_4plex.raw
148596949		High	vREEEIEVDSR	Unambiguous	3	1	N-Term(iTRAQ4plex)	HCD	1.0000	0.0000	1	1	7906	Unique	Used	0.764	3.424E3	3.545E3	115	3.57	0.00	1	15	190	2	752.88391	1504.76055	-7.90	22.87	2227	2227	MS2	18/20	Glioma_Nuclear_prep_Gradeall_repeat_4plex.raw
148596949	High		dNQLSEVANk	Unambiguous	3	1	N-Term(iTRAQ4plex); K10(iTRAQ4plex)	HCD	1.0000	0.0000	1	1	7938	Unique	Used	0.567	2.625E5	1.872E5	215	4.60	0.00	0	18	8	2	703.38092	1405.75456	1.42	25.93	2386	2386	MS2	17/18	Glioma_Nuclear_prep_Gradeall_4plex.raw
148596949		High	dNQLSEVANk	Unambiguous	3	1	N-Term(iTRAQ4plex); K10(iTRAQ4plex)	HCD	1.0000	0.0000	1	1	8116	Unique	Used	0.583	2.329E4	1.840E4	215	4.91	0.00	0	28	165	2	703.37488	1405.74248	-7.18	24.90	2448	2448	MS2	18/18	Glioma_Nuclear_prep_Gradeall_repeat_4plex.raw
148596949		High	sPAVkPAAAPk	Unambiguous	3	1	N-Term(iTRAQ4plex); K5(iTRAQ4plex); K11(iTRAQ4plex)	HCD	1.0000	0.0000	1	1	7036	Unique	Used	0.620	5.938E3	4.990E3	73	2.92	0.00	0	49	257	2	734.95959	1468.91191	-6.33	15.11	1305	1305	MS2	13/20	Glioma_Nuclear_prep_Gradeall_repeat_4plex.raw
148596949		High	aALSLPAk	Unambiguous	3	1	N-Term(iTRAQ4plex); K8(iTRAQ4plex)	HCD	1.0000	0.0000	1	1	8905	Unique	Used	0.544	7.295E4	5.380E4	153	2.95	0.00	0	61	97	2	529.84137	1058.67546	-5.45	32.69	3279	3279	MS2	13/14	Glioma_Nuclear_prep_Gradeall_repeat_4plex.raw
148596949		High	vADNSFDAk	Unambiguous	3	1	N-Term(iTRAQ4plex); K9(iTRAQ4plex)	HCD	1.0000	0.0000	1	1	7864	Unique	Used	0.661	3.217E4	2.879E4	207	2.87	0.00	0	32	160	2	627.82922	1254.65117	-4.55	22.50	2183	2183	MS2	15/16	Glioma_Nuclear_prep_Gradeall_repeat_4plex.raw
149999611	NP_003125.3	SRP14	6727	signal recognition particle 14 kDa protein [Homo sapiens]	13.24	1	2	3	1.745	1;2	3.0	6.84	136	14.6	10.04																			
149999611	High		fQMAYSNLLR	Unambiguous	1	1	N-Term(iTRAQ4plex)	HCD	1.0000	0.0000	1	1	10712	Unique	Used	1.719	9.478E4	2.049E5	224	3.21	0.00	0	30	19	2	693.85974	1386.71221	-14.32	46.24	5408	5408	MS2	17/18	Glioma_Nuclear_prep_Gradeall_4plex.raw
149999611		High	fQMAYSNLLR	Unambiguous	1	1	N-Term(iTRAQ4plex)	HCD	1.0000	0.0000	1	1	11589	Unique	Used	1.820	2.535E3	6.248E3	278	3.68	0.00	0	43	300	2	693.87103	1386.73479	1.96	65.43	6237	6237	MS2	17/18	Glioma_Nuclear_prep_Gradeall_repeat_4plex.raw
149999611		High	iSTVVSSk	Unambiguous	1	1	N-Term(iTRAQ4plex); K8(iTRAQ4plex)	HCD	1.0000	0.0000	1	1	8081	Unique	Used	1.745	1.544E4	3.649E4	226	3.16	0.00	0	44	123	2	554.84393	1108.68059	-0.93	24.56	2411	2411	MS2	13/14	Glioma_Nuclear_prep_Gradeall_repeat_4plex.raw
151101292	NP_110382.3	TMX1	81542	thioredoxin-related transmembrane protein 1 precursor [Homo sapiens]	7.86	1	2	3	1.511	1;2	17.4	11.00	280	31.8	4.98																			
151101292		High	fIITALPTIYHck	Unambiguous	1	1	N-Term(iTRAQ4plex); C12(Methylthio); K13(iTRAQ4plex)	HCD	1.0000	0.0000	1	1	12373	Unique	Used	2.004	2.367E2	6.425E2	172	4.35	0.00	0	33	300	3	618.68005	1854.02561	-0.23	81.83	7515	7515	MS2	25/48	Glioma_Nuclear_prep_Gradeall_repeat_4plex.raw
151101292	High		dFINFISDk	Unambiguous	1	1	N-Term(iTRAQ4plex); K9(iTRAQ4plex)	HCD	1.0000	0.0000	1	1	10895	Unique	Used	1.493	6.505E4	1.221E5	230	4.00	0.00	0	38	20	2	693.86829	1386.72930	-15.49	47.65	5607	5607	MS2	16/16	Glioma_Nuclear_prep_Gradeall_4plex.raw
151101292		High	dFINFISDk	Unambiguous	1	1	N-Term(iTRAQ4plex); K9(iTRAQ4plex)	HCD	1.0000	0.0000	1	1	11690	Unique	Used	1.511	1.767E3	3.617E3	278	3.88	0.00	0	45	300	2	693.87970	1386.75212	0.97	66.84	6357	6357	MS2	16/16	Glioma_Nuclear_prep_Gradeall_4plex.raw
157412270	NP_112480.2	HNRNPM	4670	heterogeneous nuclear ribonucleoprotein M isoform b [Homo sapiens]	20.26	3	6	9	0.674	5;4	17.0	41.50	691	73.6	8.82																			
157412270		High	gNFGGSFAGSFGGAGGHAPGVAR	Unambiguous	3	1	N-Term(iTRAQ4plex)	HCD	1.0000	0.0000	1	1	10150	Unique	Used	0.629	1.550E3	1.320E3	146	8.75	0.00	0	28	296	3	727.02515	2179.06089	2.66	46.87	4615	4615	MS2	39/88	Glioma_Nuclear_prep_Gradeall_repeat_4plex.raw
157412270	High		mGPAMGPALGAGIER	Unambiguous	3	1	N-Term(iTRAQ4plex)	HCD	1.0000	0.0000	1	1	9606	Unique	Used	0.443	2.417E5	1.345E5	154	4.33	0.00	0	31	14	2	786.40015	1571.79302	-14.33	38.01	4202	4202	MS2	23/28	Glioma_Nuclear_prep_Gradeall_4plex.raw
157412270	High		gNFGGSFAGSFGGAGGHAPGVAR	Unambiguous	3	1	N-Term(iTRAQ4plex)	HCD	1.0000	0.0000	1	1	9412	Unique	Used	0.527	1.547E4	1.026E4	95	5.47	0.00	0	25	8	3	727.01166	2179.02042	-15.91	36.60	3991	3991	MS2	39/88	Glioma_Nuclear_prep_Gradeall_4plex.raw
157412270	High		mGPLGLDHMASSIER	Unambiguous	3	1	N-Term(iTRAQ4plex)	HCD	1.0000	0.0000	1	1	9957	Unique	Used	0.613	5.280E4	4.073E4	121	3.58	0.00	0	25	7	3	586.62225	1757.85221	-15.57	40.55	4583	4583	MS2	24/56	Glioma_Nuclear_prep_Gradeall_4plex.raw
157412270	High		mGPLGLDHMASSIER	Unambiguous	3	1	N-Term(iTRAQ4plex)	HCD	1.0000	0.0000	1	1	9975	Unique	Used	0.697	9.449E4	8.278E4	124	4.76	0.00	0	5	25	2	879.43085	1757.85442	-14.32	40.67	4602	4602	MS2	21/28	Glioma_Nuclear_prep_Gradeall_4plex.raw
157412270		High	mGPAMGPALGAGIER	Unambiguous	3	1	N-Term(iTRAQ4plex)	HCD	1.0000	0.0000	1	1	10189	Unique	Used	0.674	2.872E3	2.623E3	247	3.04	0.00	0	57	300	2	786.41364	1571.81999	2.83	47.33	4656	4656	MS2	16/28	Glioma_Nuclear_prep_Gradeall_repeat_4plex.raw
157412270		High	fESPEVAER	Unambiguous	3	1	N-Term(iTRAQ4plex)	HCD	1.0000	0.0000	1	1	8338	Unique	Used	0.760	3.179E4	3.270E4	178	3.22	0.00	0	44	182	2	604.31018	1207.61308	4.55	27.06	2682	2682	MS2	15/16	Glioma_Nuclear_prep_Gradeall_repeat_4plex.raw
157412270		High	iNEILSNALk	Unambiguous	3	1	N-Term(iTRAQ4plex); K10(iTRAQ4plex)	HCD	1.0000	0.0000	1	1	11475	Unique	Used	0.755	6.644E3	6.791E3	158	4.06	0.00	0	21	300	2	701.93066	1402.85405	2.30	63.83	6101	6101	MS2	18/18	Glioma_Nuclear_prep_Gradeall_repeat_4plex.raw
157412270	High		aFITNIPFDVk	Unambiguous	2	1	N-Term(iTRAQ4plex); K11(iTRAQ4plex)	HCD	1.0000	0.0000	1	1	11249	Unique	Used	0.763	3.509E4	3.367E4	214	4.46	0.00	0	35	49	2	776.95209	1552.89690	-0.57	50.43	5993	5993	MS2	17/20	Glioma_Nuclear_prep_Gradeall_4plex.raw
160707950	NP_001104262.1	MECP2	4204	methyl-CpG-binding protein 2 isoform 2 [Homo sapiens]	37.15	2	16	39	0.456	16;23	34.4	113.42	498	53.3	9.88																			
160707950	High		vGDTSLDPNDFDFTVTGR	Unambiguous	2	1	N-Term(iTRAQ4plex)	HCD	1.0000	0.0000	1	1	11109	Unique	Used	0.471	1.150E5	6.808E4	66	6.00	0.00	0	6	20	2	1050.49988	2099.99248	-3.72	49.31	5840	5840	MS2	24/34	Glioma_Nuclear_prep_Gradeall_4plex.raw
160707950	High		vGDTSLDPNDFDFTVTGR	Unambiguous	2	1	N-Term(iTRAQ4plex)	HCD	1.0000	0.0000	1	1	11212	Unique	Used	0.685	1.967E4	1.695E4	73	5.92	0.00	0	36	94	2	1050.49500	2099.98271	-8.37	50.11	5952	5952	MS2	23/34	Glioma_Nuclear_prep_Gradeall_4plex.raw
160707950		High	vGDTSLDPNDFDFTVTGR	Unambiguous	2	1	N-Term(iTRAQ4plex)	HCD	1.0000	0.0000	1	1	11776	Unique	Used	0.543	1.938E3	1.424E3	69	5.17	0.00	0	0	300	2	1050.50513	2100.00298	1.28	68.15	6467	6467	MS2	24/34	Glioma_Nuclear_prep_Gradeall_repeat_4plex.raw
160707950		High	vGDTSLDPNDFDFTVTGR	Unambiguous	2	1	N-Term(iTRAQ4plex)	HCD	1.0000	0.0000	1	1	11790	Unique	Used	0.552	5.034E2	3.764E2	97	5.01	0.00	0	31	292	3	700.67334	2100.00547	2.47	68.41	6488	6488	MS2	21/68	Glioma_Nuclear_prep_Gradeall_repeat_4plex.raw
160707950	High		gGSLESDGcPk	Unambiguous	2	1	N-Term(iTRAQ4plex); C9(Methylthio); K11(iTRAQ4plex)	HCD	1.0000	0.0000	1	1	8082	Unique	Used	0.491	3.124E5	1.929E5	50	4.34	0.00	0	37	11	2	692.31897	1383.63066	-13.04	26.94	2542	2542	MS2	19/20	Glioma_Nuclear_prep_Gradeall_4plex.raw

160707950		High	gGSLESDGcPk	Unambiguous	2	1	N-Term(iTRAQ4plex); C9(Methylthio); K11(iTRAQ4plex)	HCD	1.0000	0.0000	1	1	8376	Unique	Used	0.466	1.846E4	1.166E4	87	4.00	0.00	0	28	209	2	692.33191	1383.65654	5.66	27.42	2722	2722	MS2	18/20	Glioma_Nuclear_prep_Gradeall_repeat_4plex.raw
160707950		High	gGSLESDGcPk	Unambiguous	2	1	N-Term(iTRAQ4plex); C9(Methylthio); K11(iTRAQ4plex)	HCD	1.0000	0.0000	1	1	8269	Unique	Used	0.551	8.687E3	6.483E3	102	3.57	0.00	0	42	300	2	692.33301	1383.65874	7.25	26.38	2609	2609	MS2	16/20	Glioma_Nuclear_prep_Gradeall_repeat_4plex.raw
160707950	High		kAEADPQAIPk	Unambiguous	1	1	N-Term(iTRAQ4plex); K1(iTRAQ4plex); K11(iTRAQ4plex)	HCD	1.0000	0.0000	1	1	7449	Unique	Used	0.492	1.861E5	1.151E5	139	3.60	0.00	1	9	7	2	800.47180	1599.93633	-4.21	22.46	1856	1856	MS2	17/20	Glioma_Nuclear_prep_Gradeall_4plex.raw
160707950	High		kAEADPQAIPk	Unambiguous	1	1	N-Term(iTRAQ4plex); K1(iTRAQ4plex); K11(iTRAQ4plex)	HCD	1.0000	0.0000	1	1	7425	Unique	Used	0.493	3.530E4	2.190E4	128	4.69	0.00	1	49	8	3	533.98468	1599.93949	-2.24	22.28	1830	1830	MS2	18/40	Glioma_Nuclear_prep_Gradeall_4plex.raw
160707950		High	kPGSVVAAAAAEAk	Unambiguous	2	1	N-Term(iTRAQ4plex); K1(iTRAQ4plex); K14(iTRAQ4plex)	HCD	1.0000	0.0000	1	1	8821	Unique	Used	0.471	1.792E4	1.143E4	90	5.70	0.00	0	25	300	2	851.51697	1702.02666	2.50	31.86	3191	3191	MS2	24/26	Glioma_Nuclear_prep_Gradeall_repeat_4plex.raw
160707950		High	kPGSVVAAAAAEAk	Unambiguous	2	1	N-Term(iTRAQ4plex); K1(iTRAQ4plex); K14(iTRAQ4plex)	HCD	1.0000	0.0000	1	1	8801	Unique	Used	0.521	4.692E3	3.309E3	136	6.03	0.00	0	35	93	3	568.00439	1701.99863	-13.97	31.64	3170	3170	MS2	27/52	Glioma_Nuclear_prep_Gradeall_repeat_4plex.raw
160707950		High	kPGSVVAAAAAEAk	Unambiguous	2	1	N-Term(iTRAQ4plex); K1(iTRAQ4plex); K14(iTRAQ4plex)	HCD	1.0000	0.0000	1	1	8824	Unique	Used	0.707	1.092E3	1.046E3	104	4.45	0.00	0	52	243	4	426.26117	1702.02285	0.26	31.89	3194	3194	MS2	22/78	Glioma_Nuclear_prep_Gradeall_repeat_4plex.raw
160707950	High		sVQETVLPIk	Unambiguous	2	1	N-Term(iTRAQ4plex); K10(iTRAQ4plex)	HCD	1.0000	0.0000	1	1	9182	Unique	Used	0.341	1.023E6	4.382E5	159	3.27	0.00	0	29	3	2	701.42194	1401.83660	-13.54	34.89	3739	3739	MS2	16/18	Glioma_Nuclear_prep_Gradeall_4plex.raw
160707950	High		sEDQDLQGLk	Unambiguous	2	1	N-Term(iTRAQ4plex); K10(iTRAQ4plex)	HCD	1.0000	0.0000	1	1	8155	Unique	Used	0.432	8.328E5	4.526E5	144	4.25	0.00	0	10	4	2	710.86884	1420.73039	-15.37	27.46	2621	2621	MS2	18/18	Glioma_Nuclear_prep_Gradeall_4plex.raw
160707950	High		mPFQTSPGGk	Unambiguous	2	1	N-Term(iTRAQ4plex); K10(iTRAQ4plex)	HCD	1.0000	0.0000	1	1	8372	Unique	Used	0.510	4.182E5	2.684E5	208	2.95	0.00	0	39	7	2	669.35095	1337.69463	-13.46	28.99	2856	2856	MS2	15/18	Glioma_Nuclear_prep_Gradeall_4plex.raw
160707950		High	aATSEGVQVk	Unambiguous	2	1	N-Term(iTRAQ4plex); K10(iTRAQ4plex)	HCD	1.0000	0.0000	1	1	7214	Unique	Used	0.401	7.557E4	4.102E4	244	3.07	0.00	0	11	44	2	639.35919	1277.71111	-15.08	16.66	1495	1495	MS2	18/18	Glioma_Nuclear_prep_Gradeall_repeat_4plex.raw
160707950		High	sVQETVLPIk	Unambiguous	2	1	N-Term(iTRAQ4plex); K10(iTRAQ4plex)	HCD	1.0000	0.0000	1	1	9777	Unique	Used	0.412	4.892E4	2.732E4	139	4.10	0.00	0	41	87	2	701.43262	1401.85796	1.70	42.41	4211	4211	MS2	16/18	Glioma_Nuclear_prep_Gradeall_repeat_4plex.raw
160707950		High	sEDQDLQGLk	Unambiguous	2	1	N-Term(iTRAQ4plex); K10(iTRAQ4plex)	HCD	1.0000	0.0000	1	1	8391	Unique	Used	0.414	8.212E4	4.609E4	180	4.20	0.00	0	4	52	2	710.87982	1420.75237	0.09	27.59	2738	2738	MS2	18/18	Glioma_Nuclear_prep_Gradeall_repeat_4plex.raw
160707950		High	aEADPQAIPk	Unambiguous	2	1	N-Term(iTRAQ4plex); K10(iTRAQ4plex)	HCD	1.0000	0.0000	1	1	7783	Unique	Used	0.634	1.381E4	1.186E4	268	3.28	0.00	0	23	155	2	664.37604	1327.74480	-0.93	21.71	2097	2097	MS2	15/18	Glioma_Nuclear_prep_Gradeall_repeat_4plex.raw
160707950		High	aATSEGVQVk	Unambiguous	2	1	N-Term(iTRAQ4plex); K10(iTRAQ4plex)	HCD	1.0000	0.0000	1	1	7225	Unique	Used	0.758	9.618E3	9.875E3	265	3.59	0.00	0	56	129	3	426.57532	1277.71140	-14.85	16.74	1506	1506	MS2	16/36	Glioma_Nuclear_prep_Gradeall_repeat_4plex.raw
160707950		High	yDVYLINPQGk	Unambiguous	2	1	N-Term(iTRAQ4plex); K11(iTRAQ4plex)	HCD	1.0000	0.0000	1	1	10685	Unique	Used	0.576	3.599E3	2.808E3	185	3.99	0.00	0	23	166	3	533.30054	1597.88706	2.64	53.26	5188	5188	MS2	16/40	Glioma_Nuclear_prep_Gradeall_repeat_4plex.raw
160707950	High		tQPAVATAATAAEk	Unambiguous	2	1	N-Term(iTRAQ4plex); K14(iTRAQ4plex)	HCD	1.0000	0.0000	1	1	7939	Unique	Used	0.224	4.780E5	1.346E5	230	5.06	0.00	0	6	8	2	809.45905	1617.91081	3.55	25.94	2387	2387	MS2	22/26	Glioma_Nuclear_prep_Gradeall_4plex.raw
160707950		High	tQPAVATAATAAEk	Unambiguous	2	1	N-Term(iTRAQ4plex); K14(iTRAQ4plex)	HCD	1.0000	0.0000	1	1	8136	Unique	Used	0.293	2.403E4	9.543E3	217	5.33	0.00	0	52	300	2	809.45740	1617.90752	1.51	25.09	2469	2469	MS2	21/26	Glioma_Nuclear_prep_Gradeall_repeat_4plex.raw
160707950		High	tQPAVATAATAAEk	Unambiguous	2	1	N-Term(iTRAQ4plex); K14(iTRAQ4plex)	HCD	1.0000	0.0000	1	1	8152	Unique	Used	0.617	8.912E3	7.452E3	218	5.96	0.00	0	25	51	3	539.97394	1617.90726	1.36	25.26	2486	2486	MS2	23/52	Glioma_Nuclear_prep_Gradeall_repeat_4plex.raw
160707950	High		aEGGGATTSTQVMVIk	Unambiguous	2	1	N-Term(iTRAQ4plex); K16(iTRAQ4plex)	HCD	1.0000	0.0000	1	1	8509	Unique	Used	0.312	1.704E4	6.695E3	182	5.18	0.00	0	8	22	2	919.48737	1837.96745	-14.03	29.96	3004	3004	MS2	29/30	Glioma_Nuclear_prep_Gradeall_4plex.raw
160707950		High	aEGGGATTSTQVMVIk	Unambiguous	2	1	N-Term(iTRAQ4plex); K16(iTRAQ4plex)	HCD	1.0000	0.0000	1	1	8944	Unique	Used	0.335	1.074E4	4.876E3	203	7.20	0.00	0	0	196	2	919.50018	1837.99309	-0.09	33.05	3320	3320	MS2	29/30	Glioma_Nuclear_prep_Gradeall_repeat_4plex.raw
160707950		High	aEGGGATTSTQVMVIk	Unambiguous	2	1	N-Term(iTRAQ4plex); K16(iTRAQ4plex)	HCD	1.0000	0.0000	1	1	9043	Unique	Used	0.389	2.536E3	1.336E3	211	6.58	0.00	0	13	300	2	919.50397	1838.00066	4.03	34.23	3428	3428	MS2	30/30	Glioma_Nuclear_prep_Gradeall_repeat_4plex.raw
160707950		High	aEGGGATTSTQVMVIk	Unambiguous	2	1	N-Term(iTRAQ4plex); K16(iTRAQ4plex)	HCD	1.0000	0.0000	1	1	8923	Unique	Used	0.653	3.153E3	2.786E3	204	6.33	0.00	0	37	117	3	613.33319	1837.98502	-4.48	32.85	3298	3298	MS2	23/60	Glioma_Nuclear_prep_Gradeall_repeat_4plex.raw
160707950	High		hEPVQPSAHHSAEPAEAGk	Unambiguous	2	1	N-Term(iTRAQ4plex); K19(iTRAQ4plex)	HCD	1.0000	0.0000	1	1	7101	Unique	Used	0.210	6.197E3	1.639E3	161	5.16	0.00	0	15	22	4	567.54199	2267.14614	2.36	19.65	1475	1475	MS2	26/108	Glioma_Nuclear_prep_Gradeall_4plex.raw
160707950	High		hEPVQPSAHHSAEPAEAGk	Unambiguous	2	1	N-Term(iTRAQ4plex); K19(iTRAQ4plex)	HCD	1.0000	0.0000	1	1	7092	Unique	Used	0.335	6.821E3	2.875E3	94	3.71	0.00	0	21	300	3	756.38770	2267.14853	3.42	19.53	1464	1464	MS2	22/72	Glioma_Nuclear_prep_Gradeall_4plex.raw
160707950		High	hEPVQPSAHHSAEPAEAGk	Unambiguous	2	1	N-Term(iTRAQ4plex); K19(iTRAQ4plex)	HCD	1.0000	0.0000	1	1	6741	Unique	Used	0.186	4.840E3	1.221E3	150	5.02	0.00	0		30	4	567.53339	2267.11172	-12.82	12.50	995	995	MS2	25/108	Glioma_Nuclear_prep_Gradeall_repeat_4plex.raw
160707950		High	hEPVQPSAHHSAEPAEAGk	Unambiguous	2	1	N-Term(iTRAQ4plex); K19(iTRAQ4plex)	HCD	1.0000	0.0000	1	1	6752	Unique	Used	0.307	4.121E3	1.714E3	89	4.17	0.00	0	14	131	3	756.38641	2267.14469	1.72	12.58	1006	1006	MS2	21/72	Glioma_Nuclear_prep_Gradeall_repeat_4plex.raw
160707950	High		eVVkPLLVSTLGEk	Unambiguous	2	1	N-Term(iTRAQ4plex); K4(iTRAQ4plex); K14(iTRAQ4plex)	HCD	1.0000	0.0000	1	1	9940	Unique	Used	0.133	1.421E5	2.382E4	32	4.75	0.00	0	12	25	2	972.60138	1944.19548	-7.77	40.42	4564	4564	MS2	21/26	Glioma_Nuclear_prep_Gradeall_4plex.raw
160707950		High	eVVkPLLVSTLGEk	Unambiguous	2	1	N-Term(iTRAQ4plex); K4(iTRAQ4plex); K14(iTRAQ4plex)	HCD	1.0000	0.0000	1	1	10872	Unique	Used	0.165	2.029E3	4.534E2	10	4.12	0.00	0	42	300	3	648.74677	1944.22574	7.79	55.58	5392	5392	MS2	24/52	Glioma_Nuclear_prep_Gradeall_repeat_4plex.raw
160707950	High		eTVSIEVk	Unambiguous	2	1	N-Term(iTRAQ4plex); K8(iTRAQ4plex)	HCD	1.0000	0.0000	1	1	8217	Unique	Used	0.449	5.163E5	2.913E5	296	3.38	0.00	0	43	7	2	596.84741	1192.68755	-12.75	27.89	2688	2688	MS2	14/14	Glioma_Nuclear_prep_Gradeall_4plex.raw
160707950		High	eTVSIEVk	Unambiguous	2	1	N-Term(iTRAQ4plex); K8(iTRAQ4plex)	HCD	1.0000	0.0000	1	1	8585	Unique	Used	0.311	9.364E4	3.945E4	221	3.57	0.00	0	15	58	2	596.85474	1192.70220	-0.47	29.48	2943	2943	MS2	14/14	Glioma_Nuclear_prep_Gradeall_repeat_4plex.raw
160707950	High		vELIAYFEk	Unambiguous	2	1	N-Term(iTRAQ4plex); K9(iTRAQ4plex)	HCD	1.0000	0.0000	1	1	11464	Unique	Used	0.390	3.273E5	1.606E5	217	3.98	0.00	0	23	15	2	700.40765	1399.80803	0.34	52.31	6229	6229	MS2	16/16	Glioma_Nuclear_prep_Gradeall_4plex.raw
160707950	High		vELIAYFEk	Unambiguous	2	1	N-Term(iTRAQ4plex); K9(iTRAQ4plex)	HCD	1.0000	0.0000	1	1	11387	Unique	Used	0.456	7.945E4	4.558E4	225	4.24	0.00	0	42	51	2	700.40808	1399.80889	0.95	51.63	6144	6144	MS2	15/16	Glioma_Nuclear_prep_Gradeall_4plex.raw
160707950		High	vELIAYFEk	Unambiguous	2	1	N-Term(iTRAQ4plex); K9(iTRAQ4plex)	HCD	1.0000	0.0000	1	1	12124	Unique	Used	0.461	4.559E3	2.845E3	205	3.67	0.00	0	55	300	2	700.40961	1399.81194	3.13	74.67	6984	6984	MS2	15/16	Glioma_Nuclear_prep_Gradeall_repeat_4plex.raw
167234419	NP_005110.2	THRAP3	9967	thyroid hormone receptor-associated protein 3 [Homo sapiens]	6.07	1	4	9	0.582	4;5	7.3	27.54	955	108.6	10.15																			
167234419	High		kTEELEEESFPER	Unambiguous	1	1	N-Term(iTRAQ4plex); K1(iTRAQ4plex)	HCD	1.0000	0.0000	1	1	8408	Unique	Used	0.560	1.749E4	1.232E4	79	4.33	0.00	1	42	15	3	637.64600	1910.92344	-18.42	29.25	2895	2895	MS2	19/48	Glioma_Nuclear_prep_Gradeall_4plex.raw
167234419	High		kTEELEEESFPER	Unambiguous	1	1	N-Term(iTRAQ4plex); K1(iTRAQ4plex)	HCD	1.0000	0.0000	1	1	8420	Unique	Used	0.627	8.962E4	7.065E4	131	3.45	0.00	1	10	12	2	955.96600	1910.92473	-17.74	29.33	2908	2908	MS2	21/24	Glioma_Nuclear_prep_Gradeall_4plex.raw
167234419		High	kTEELEEESFPER	Unambiguous	1	1	N-Term(iTRAQ4plex); K1(iTRAQ4plex)	HCD	1.0000	0.0000	1	1	8819	Unique	Used	0.520	3.170E3	2.233E3	141	5.14	0.00	1	70	95	3	637.65924	1910.96317	2.38	31.85	3189	3189	MS2	20/48	Glioma_Nuclear_prep_Gradeall_repeat_4plex.raw
167234419		High	kTEELEEESFPER	Unambiguous	1	1	N-Term(iTRAQ4plex); K1(iTRAQ4plex)	HCD	1.0000	0.0000	1	1	8811	Unique	Used	0.616	4.060E3	3.385E3	116	3.34	0.00	1	9	300	2	955.96899	1910.93071	-14.61	31.74	3180	3180	MS2	20/24	Glioma_Nuclear_prep_Gradeall_repeat_4plex.raw
167234419	High		wEGLVYAPPGk	Unambiguous	1	1	N-Term(iTRAQ4plex); K11(iTRAQ4plex)	HCD	1.0000	0.0000	1	1	10165	Unique	Used	0.662	8.043E4	6.699E4	282	3.88	0.00	0	30	27	2	752.91309	1504.81890	-14.20	42.09	4810	4810	MS2	16/20	Glioma_Nuclear_prep_Gradeall_4plex.raw
167234419		High	wEGLVYAPPGk	Unambiguous	1	1	N-Term(iTRAQ4plex); K11(iTRAQ4plex)	HCD	1.0000	0.0000	1	1	10967	Unique	Used	0.592	3.573E3	2.865E3	268	3.81	0.00	0	27	300	2	752.92468	1504.84209	1.21	56.75	5496	5496	MS2	16/20	Glioma_Nuclear_prep_Gradeall_repeat_4plex.raw
167234419	High		gSFSDTGLGDGk	Unambiguous	1	1	N-Term(iTRAQ4plex); K12(iTRAQ4plex)	HCD	1.0000	0.0000	1	1	8188	Unique	Used	0.564	1.566E5	1.110E5	98	4.82	0.00	0	19	17	2	714.85144	1428.69560	-17.74	27.68	2656	2656	MS2	22/22	Glioma_Nuclear_prep_Gradeall_4plex.raw
167234419		High	gSFSDTGLGDGk	Unambiguous	1	1	N-Term(iTRAQ4plex); K12(iTRAQ4plex)	HCD	1.0000	0.0000	1	1	8435	Unique	Used	0.558	1.564E4	1.183E4	169	4.07	0.00	0	15	177	2	714.86694	1428.72661	3.96	27.98	2784	2784	MS2	22/22	Glioma_Nuclear_prep_Gradeall_repeat_4plex.raw
167234419		High	eSEFDDEPk	Unambiguous	1	1	N-Term(iTRAQ4plex); K9(iTRAQ4plex)	HCD	1.0000	0.0000	1	1	7802	Unique	Used	0.582	1.867E4	1.470E4	104	3.12	0.00	0	28	180	2	692.33124	1383.65520	2.42	21.87	2117	2117	MS2	16/16	Glioma_Nuclear_prep_Gradeall_repeat_4plex.raw
189217895	NP_689901.2	KHDRBS2	202559	KH domain-containing, RNA-binding, signal transduction-associated protein 2 [Homo sapiens]	10.03	5	2	3	0.185	2;1	34.5	13.20	349	38.9	6.29																			
189217895	High		fLVPDYNDEIR	Unambiguous	1	1	N-Term(iTRAQ4plex)	HCD	1.0000	0.0000	1	1	10388	Unique	Used	0.185	1.484E5	3.461E4	194	3.02	0.00	0	49	33	2	762.88330	1524.75932	-14.56	43.75	5052	5052	MS2	12/20	Glioma_Nuclear_prep_Gradeall_4plex.raw
189217895		High	fLVPDYNDEIR	Unambiguous	1	1	N-Term(iTRAQ4plex)	HCD	1.0000	0.0000	1	1	11164	Unique	Used	0.127	1.118E4	1.916E3	280	3.24	0.00	0	38	300	2	762.89685	1524.78642	3.21	59.32	5720	5720	MS2	13/20	Glioma_Nuclear_prep_Gradeall_repeat_4plex.raw
189217895	High		ILAEEIEk	Unambiguous	1	1	N-Term(iTRAQ4plex); K8(iTRAQ4plex)	HCD	1.0000	0.0000	1	1	9223	Unique	Used	0.257	3.521E5	1.139E5	292	3.08	0.00	0	35	12	2	616.86536	1232.72344	-8.62	35.17	3783	3783	MS2	14/14	Glioma_Nuclear_prep_Gradeall_4plex.raw
194688133	NP_001124160.1	HMGB2	3148	high mobility group protein B2 [Homo sapiens]	26.32	1	5	7	3.890	3;4	38.0	34.59	209	24.0	7.81																			
194688133		High	kHPDSSVNFAEFSk	Unambiguous	1	1	N-Term(iTRAQ4plex); K1(iTRAQ4plex); K14(iTRAQ4plex)	HCD	1.0000	0.0000	1	1	8993	Unique	Used	2.474	8.297E2	2.780E3	145	6.39	0.00	1	30	259	3	675.69824	2025.08017	1.76	33.63	3374	3374	MS2	25/52	Glioma_Nuclear_prep_Gradeall_repeat_4plex.raw
194688133	High		sEHPGLSIGDTAk	Unambiguous	1	1	N-Term(iTRAQ4plex); K13(iTRAQ4plex)	HCD	1.0000	0.0000	1	1	7771	Unique	Used	3.733	9.432E4	4.428E5	175	4.43	0.00	0	11	1	3	533.95703	1599.85654	-0.98	24.74	2205	2205	MS2	28/48	Glioma_Nuclear_prep_Gradeall_4plex.raw
194688133	High		sEHPGLSIGDTAk	Unambiguous	1	1	N-Term(iTRAQ4plex); K13(iTRAQ4plex)	HCD	1.0000	0.0000	1	1	7762	Unique	Used	5.632	6.605E4	4.678E5	182	4.13	0.00	0	8	6	2	800.43286	1599.85845	0.21	24.68	2195	2195	MS2	22/24	Glioma_Nuclear_prep_Gradeall_4plex.raw
194688133		High	hPDSSVNFAEFSk	Unambiguous	1	1	N-Term(iTRAQ4plex); K13(iTRAQ4plex)	HCD	1.0000	0.0000	1	1	9557	Unique	Used	2.609	7.852E2	2.775E3	140	5.39	0.00	0	13	215	3	584.95868	1752.86148	-10.32	39.93	3977	3977	MS2	24/48	Glioma_Nuclear_prep_Gradeall_repeat_4plex.raw
194688133		High	sEHPGLSIGDTAk	Unambiguous	1	1	N-Term(iTRAQ4plex); K13(iTRAQ4plex)	HCD	1.0000	0.0000	1	1	7985	Unique	Used	6.486	3.496E3	3.072E4	175	4.06	0.00	0	5	122	2	800.43475	1599.86223	2.58	23.66	2310	2310	MS2	20/24	Glioma_Nuclear_prep_Gradeall_repeat_4plex.raw
194688133	High		skFEDMAk	Unambiguous	1	1	N-Term(iTRAQ4plex); K2(iTRAQ4plex); K8(iTRAQ4plex)	HCD	1.0000	0.0000	1	1	7730	Unique	Used	3.890	8.216E4	4.020E5	245	3.03	0.00	1	14	13	2	694.38489	1387.76250	0.65	24.45	2160	2160	MS2	14/14	Glioma_Nuclear_prep_Gradeall_4plex.raw
194688133		High	dkQPYEQk	Unambiguous	1	1	N-Term(iTRAQ4plex); K2(iTRAQ4plex); K8(iTRAQ4plex)	HCD	1.0000	0.0000	1	1	6805	Unique	Used	6.328	2.649E3	2.271E4	254	3.13	0.00	1	9	121	2	734.41266	1467.81804	0.87	13.06	1062	1062	MS2	13/14	Glioma_Nuclear_prep_Gradeall_repeat_4plex.raw
212549553	NP_060090.2	ILF3	3609	interleukin enhancer-binding factor 3 isoform d [Homo sapiens]	33.18	5	21	60	0.377	26;34	36.7	170.51	898	95.7	8.81																			
212549553	High		hSSVYPTQEELEAVQNMVSHTER	Unambiguous	5	1	N-Term(iTRAQ4plex)	HCD	1.0000	0.0000	1	1	10965	Unique	Used	0.252	4.501E4	1.426E4	52	9.60	0.00	0	6	9	3	939.10748	2815.30790	-12.76	48.19	5683	5683	MS2	34/88	Glioma_Nuclear_prep_Gradeall_4plex.raw
212549553	High		vLAGETLSVNDPPDVLDR	Unambiguous	5	1	N-Term(iTRAQ4plex)	HCD	1.0000	0.0000	1	1	10644	Unique	Used	0.228	1.965E5	5.627E4	137	6.68	0.00	0	1	9	2	1027.53357	2054.05986	-14.05	45.73	5334	5334	MS2	28/34	Glioma_Nuclear_prep_Gradeall_4plex.raw
212549553	High		eDITQSAQHALR	Unambiguous	5	1	N-Term(iTRAQ4plex)	HCD	1.0000	0.0000	1	1	8011	Unique	Used	0.273	2.610E5	8.964E4	191	3.78	0.00	0	6	6	2	756.88623	1512.76518	-15.57	26.44	2465	2465	MS2	21/22	Glioma_Nuclear_prep_Gradeall_4plex.raw
212549553	High		eATDAIGHLDR	Unambiguous	5	1	N-Term(iTRAQ4plex)	HCD	1.0000	0.0000	1	1	7930	Unique	Used	0.318	4.207E5	1.683E5	211	3.93	0.00	0	9	6	2	671.34766	1341.68804	0.05	25.87	2377	2377	MS2	19/20	Glioma_Nuclear_prep_Gradeall_4plex.raw
212549553	High		vLQDMGLPTGAEGR	Unambiguous	5	1	N-Term(iTRAQ4plex)	HCD	1.0000	0.0000	1	1	9971	Unique	Used	0.382	1.956E5	9.401E4	125	4.15	0.00	0	7	11	2	794.40601	1587.80474	-14.77	40.64	4598	4598	MS2	19/26	Glioma_Nuclear_prep_Gradeall_4plex.raw
212549553	High		eATDAIGHLDR	Unambiguous	5	1	N-Term(iTRAQ4plex)	HCD	1.0000	0.0000	1	1	7918	Unique	Used	0.541	3.376E4	2.295E4	188	3.93	0.00	0	42	9	3	447.90045	1341.68680	-0.87	25.78	2364	2364	MS2	22/40	Glioma_Nuclear_prep_Gradeall_4plex.raw
212549553	High		ePPLSLTIHLTSPVVR	Unambiguous	5	1	N-Term(iTRAQ4plex)	HCD	1.0000	0.0000	1	1	10676	Unique	Used	0.592	1.700E4	1.267E4	118	3.74	0.00	0	40	16	3	635.03387	1903.08707	-13.83	45.98	5369	5369	MS2	25/60	Glioma_Nuclear_prep_Gradeall_4plex.raw
212549553	High		eDITQSAQHALR	Unambiguous	5	1	N-Term(iTRAQ4plex)	HCD	1.0000	0.0000	1	1	8001	Unique	Used	0.673	6.333E4	5.358E4	153	4.74	0.00	0	26	4	3	504.92676	1512.76572	-15.21	26.37	2454	2454	MS2	25/44	Glioma_Nuclear_prep_Gradeall_4plex.raw
212549553	High		hSSVYPTQEELEAVQNMVSHTER	Unambiguous	5	1	N-Term(iTRAQ4plex)	HCD	1.0000	0.0000	1	1	11010	Unique	Used	0.675	4.395E3	3.731E3	91	5.99	0.00	0	13	9	4	704.59137	2815.34365	-0.06	48.52	5731	5731	MS2	32/132	Glioma_Nuclear_prep_Gradeall_4plex.raw
212549553		High	fNYSGSGGR	Unambiguous	2	1	N-Term(iTRAQ4plex)	HCD	1.0000	0.0000	1	1	7572	Unique	Used	0.192	1.397E5	3.631E4	71	3.39	0.00	0	23	56	2	544.76404	1088.52080	-3.09	19.71	1871	1871	MS2	16/16	Glioma_Nuclear_prep_Gradeall_repeat_4plex.raw
212549553		High	vLAGETLSVNDPPDVLDR	Unambiguous	5	1	N-Term(iTRAQ4plex)	HCD	1.0000	0.0000	1	1	11358	Unique	Used	0.235	1.528E3	4.862E2	139	5.89	0.00	0	4	300	2	1027.54944	2054.09160	1.41	62.23	5963	5963	MS2	28/34	Glioma_Nuclear_prep_Gradeall_repeat_4plex.raw
212549553		High	eATDAIGHLDR	Unambiguous	5	1	N-Term(iTRAQ4plex)	HCD	1.0000	0.0000	1	1	8168	Unique	Used	0.270	5.406E4	1.977E4	205	4.34	0.00	0	9	59	2	671.34570	1341.68413	-2.86	25.41	2503	2503	MS2	19/20	Glioma_Nuclear_prep_Gradeall_repeat_4plex.raw
212549553		High	eDITQSAQHALR	Unambiguous	5	1	N-Term(iTRAQ4plex)	HCD	1.0000	0.0000	1	1	8262	Unique	Used	0.318	1.549E4	6.677E3	205	4.40	0.00	0	2	159	2	756.90045	1512.79363	3.23	26.31	2602	2602	MS2	22/22	Glioma_Nuclear_prep_Gradeall_repeat_4plex.raw
212549553		High	vLQDMGLPTGAEGR	Unambiguous	5	1	N-Term(iTRAQ4plex)	HCD	1.0000	0.0000	1	1	10609	Unique	Used	0.391	5.850E3	3.099E3	226	4.45	0.00	0	17	300	2	794.41907	1587.83086	1.68	52.34	5106	5106	MS2	18/26	Glioma_Nuclear_prep_Gradeall_repeat_4plex.raw
212549553		High	sIGTANRPMGAGEALR	Unambiguous	5	1	N-Term(iTRAQ4plex)	HCD	1.0000	0.0000	1	1	8246	Unique	Used	0.427	3.537E3	2.046E3	169	2.86	0.00	0	20	210	2	872.96930	1744.93132	3.87	26.17	2585	2585	MS2	14/30	Glioma_Nuclear_prep_Gradeall_repeat_4plex.raw
212549553		High	eATDAIGHLDR	Unambiguous	5	1	N-Term(iTRAQ4plex)	HCD	1.0000	0.0000	1	1	8153	Unique	Used	0.505	1.657E4	1.133E4	183	4.94	0.00	0	19	49	3	447.90054	1341.68708	-0.66	25.26	2487	2487	MS2	26/40	Glioma_Nuclear_prep_Gradeall_repeat_4plex.raw
212549553		High	aEPPQAMNALMR	Unambiguous	5	1	N-Term(iTRAQ4plex)	HCD	1.0000	0.0000	1	1	9830	Unique	Used	0.595	8.078E3	6.510E3	216	3.76	0.00	0	20	300	2	736.88013	1472.75298	3.99	42.98	4266	4266	MS2	16/22	Glioma_Nuclear_prep_Gradeall_repeat_4plex.raw
212549553		High	eDITQSAQHALR	Unambiguous	5	1	N-Term(iTRAQ4plex)	HCD	1.0000	0.0000	1	1	8253	Unique	Used	0.623	1.311E4	1.106E4	175	5.88	0.00	0	18	44	3	504.93570	1512.79255	2.52	26.24	2593	2593	MS2	28/44	Glioma_Nuclear_prep_Gradeall_repeat_4plex.raw
212549553		High	aEPPQAMNALMR	Unambiguous	5	1	N-Term(iTRAQ4plex)	HCD	1.0000	0.0000	1	1	9711	Unique	Used	0.634	7.182E3	6.166E3	215	3.65	0.00	0	31	300	2	736.87958	1472.75188	3.24	41.65	4141	4141	MS2	16/22	Glioma_Nuclear_prep_Gradeall_repeat_4plex.raw
212549553	High		cLAALASLR	Unambiguous	5	1	N-Term(iTRAQ4plex); C1(Methylthio)	HCD	1.0000	0.0000	1	1	11426	Unique	Used	0.155	7.701E5	1.504E5	255	3.12	0.00	0	4	5	2	554.30896	1107.61064	-2.61	51.98	6187	6187	MS2	15/16	Glioma_Nuclear_prep_Gradeall_4plex.raw
212549553	High		cLAALASLR	Unambiguous	5	1	N-Term(iTRAQ4plex); C1(Methylthio)	HCD	1.0000	0.0000	1	1	11343	Unique	Used	0.342	4.179E4	1.799E4	305	3.28	0.00	0	40	84	2	554.31012	1107.61296	-0.52	51.25	6096	6096	MS2	15/16	Glioma_Nuclear_prep_Gradeall_4plex.raw
212549553		High	cLAALASLR	Unambiguous	5	1	N-Term(iTRAQ4plex); C1(Methylthio)	HCD	1.0000	0.0000	1	1	12184	Unique	Used	0.195	6.202E3	1.639E3	328	2.85	0.00	0	19	300	2	554.31134	1107.61540	1.69	75.78	7073	7073	MS2	15/16	Glioma_Nuclear_prep_Gradeall_repeat_4plex.raw
212549553		High	cLAALASLR	Unambiguous	5	1	N-Term(iTRAQ4plex); C1(Methylthio)	HCD	1.0000	0.0000	1	1	12127	Unique	Used	0.254	1.168E4	4.017E3	234	3.28	0.00	0	16	300	2	554.31134	1107.61540	1.69	74.77	6992	6992	MS2	15/16	Glioma_Nuclear_prep_Gradeall_repeat_4plex.raw
212549553	High		vLGMDPLPSk	Unambiguous	5	1	N-Term(iTRAQ4plex); K10(iTRAQ4plex)	HCD	1.0000	0.0000	1	1	9699	Unique	Used	0.416	4.141E5	2.168E5	236	3.67	0.00	0	42	7	2	672.88361	1344.75994	-14.91	38.68	4303	4303	MS2	16/18	Glioma_Nuclear_prep_Gradeall_4plex.raw
212549553	High		aYAALAALEk	Unambiguous	5	1	N-Term(iTRAQ4plex); K10(iTRAQ4plex)	HCD	1.0000	0.0000	1	1	9977	Unique	Used	0.427	2.345E5	1.261E5	270	4.55	0.00	0	76	20	2	654.88074	1308.75420	-17.11	40.68	4604	4604	MS2	18/18	Glioma_Nuclear_prep_Gradeall_4plex.raw
212549553	High		aYAALAALEk	Unambiguous	5	1	N-Term(iTRAQ4plex); K10(iTRAQ4plex)	HCD	1.0000	0.0000	1	1	10065	Unique	Used	0.448	2.393E5	1.347E5	311	4.09	0.00	0	33	9	2	654.88257	1308.75786	-14.32	41.33	4700	4700	MS2	17/18	Glioma_Nuclear_prep_Gradeall_4plex.raw
212549553		High	vLGMDPLPSk	Unambiguous	5	1	N-Term(iTRAQ4plex); K10(iTRAQ4plex)	HCD	1.0000	0.0000	1	1	10372	Unique	Used	0.287	1.554E4	6.033E3	221	3.93	0.00	0	28	300	2	672.89520	1344.78313	2.34	49.51	4852	4852	MS2	16/18	Glioma_Nuclear_prep_Gradeall_repeat_4plex.raw
212549553		High	aYAALAALEk	Unambiguous	5	1	N-Term(iTRAQ4plex); K10(iTRAQ4plex)	HCD	1.0000	0.0000	1	1	10894	Unique	Used	0.295	1.747E4	6.991E3	219	4.46	0.00	0	27	262	2	654.89368	1308.78008	2.66	55.86	5417	5417	MS2	18/18	Glioma_Nuclear_prep_Gradeall_repeat_4plex.raw
212549553		High	aYAALAALEk	Unambiguous	5	1	N-Term(iTRAQ4plex); K10(iTRAQ4plex)	HCD	1.0000	0.0000	1	1	10812	Unique	Used	0.373	7.179E2	3.623E2	233	3.44	0.00	0	46	300	3	436.93073	1308.77762	0.78	54.83	5326	5326	MS2	18/36	Glioma_Nuclear_prep_Gradeall_repeat_4plex.raw
212549553		High	aYAALAALEk	Unambiguous	5	1	N-Term(iTRAQ4plex); K10(iTRAQ4plex)	HCD	1.0000	0.0000	1	1	10895	Unique	Used	0.422	9.878E2	5.645E2	232	3.45	0.00	0	11	300	3	436.93115	1308.77890	1.76	55.88	5418	5418	MS2	18/36	Glioma_Nuclear_prep_Gradeall_repeat_4plex.raw
212549553		High	aYAALAALEk	Unambiguous	5	1	N-Term(iTRAQ4plex); K10(iTRAQ4plex)	HCD	1.0000	0.0000	1	1	10808	Unique	Used	0.563	7.640E3	5.828E3	255	4.15	0.00	0	52	300	2	654.89301	1308.77873	1.63	54.78	5322	5322	MS2	17/18	Glioma_Nuclear_prep_Gradeall_repeat_4plex.raw
212549553	High		aVSDWIDEQEk	Unambiguous	5	1	N-Term(iTRAQ4plex); K11(iTRAQ4plex)	HCD	1.0000	0.0000	1	1	9656	Unique	Used	0.440	9.726E4	5.383E4	150	3.94	0.00	0	36	16	2	804.39960	1607.79192	-14.72	38.37	4256	4256	MS2	20/20	Glioma_Nuclear_prep_Gradeall_4plex.raw
212549553		High	aVSDWIDEQEk	Unambiguous	5	1	N-Term(iTRAQ4plex); K11(iTRAQ4plex)	HCD	1.0000	0.0000	1	1	10174	Unique	Used	0.365	9.491E3	4.692E3	183	4.38	0.00	0	12	259	2	804.41382	1607.82036	2.97	47.17	4641	4641	MS2	20/20	Glioma_Nuclear_prep_Gradeall_repeat_4plex.raw
212549553	High		IFPDTPLALDANk	Unambiguous	5	1	N-Term(iTRAQ4plex); K13(iTRAQ4plex)	HCD	1.0000	0.0000	1	1	10904	Unique	Used	0.198	4.859E4	1.211E4	178	3.50	0.00	0	12	4	3	568.31799	1702.93943	-13.17	47.72	5617	5617	MS2	20/48	Glioma_Nuclear_prep_Gradeall_4plex.raw
212549553	High		IFPDTPLALDANk	Unambiguous	5	1	N-Term(iTRAQ4plex); K13(iTRAQ4plex)	HCD	1.0000	0.0000	1	1	10894	Unique	Used	0.282	3.705E5	1.313E5	212	4.92	0.00	0	26	14	2	851.97418	1702.94109	-12.20	47.64	5606	5606	MS2	20/24	Glioma_Nuclear_prep_Gradeall_4plex.raw
212549553		High	IFPDTPLALDANk	Unambiguous	5	1	N-Term(iTRAQ4plex); K13(iTRAQ4plex)	HCD	1.0000	0.0000	1	1	11770	Unique	Used	0.198	9.853E3	2.649E3	199	4.95	0.00	0	10	300	2	851.98755	1702.96782	3.50	68.06	6459	6459	MS2	20/24	Glioma_Nuclear_prep_Gradeall_repeat_4plex.raw
212549553		High	IFPDTPLALDANk	Unambiguous	5	1	N-Term(iTRAQ4plex); K13(iTRAQ4plex)	HCD	1.0000	0.0000	1	1	11703	Unique	Used	0.265	8.630E3	3.094E3	201	4.70	0.00	0	31	300	2	851.98718	1702.96709	3.07	67.03	6373	6373	MS2	20/24	Glioma_Nuclear_prep_Gradeall_repeat_4plex.raw
212549553		High	IFPDTPLALDANk	Unambiguous	5	1	N-Term(iTRAQ4plex); K13(iTRAQ4plex)	HCD	1.0000	0.0000	1	1	11763	Unique	Used	0.296	2.128E3	8.544E2	182	5.02	0.00	0	12	170	3	568.32697	1702.96634	2.63	67.95	6450	6450	MS2	19/48	Glioma_Nuclear_prep_Gradeall_repeat_4plex.raw
212549553		High	IFPDTPLALDANk	Unambiguous	5	1	N-Term(iTRAQ4plex); K13(iTRAQ4plex)	HCD	1.0000	0.0000	1	1	11699	Unique	Used	0.492	7.514E2	5.011E2	203	4.87	0.00	0	36	300	3	568.32587	1702.96305	0.70	66.97	6368	6368	MS2	20/48	Glioma_Nuclear_prep_Gradeall_repeat_4plex.raw
212549553	High		IFPDTPLALDANkk	Unambiguous	5	1	N-Term(iTRAQ4plex); K13(iTRAQ4plex); K14(iTRAQ4plex)	HCD	1.0000	0.0000	1	1	10092	Unique	Used	0.575	5.148E4	3.723E4	124	3.56	0.00	1	54	14	3	659.04742	1975.12772	-15.78	41.51	4729	4729	MS2	28/52	Glioma_Nuclear_prep_Gradeall_4plex.raw
212549553		High	IFPDTPLALDANkk	Unambiguous	5	1	N-Term(iTRAQ4plex); K13(iTRAQ4plex); K14(iTRAQ4plex)	HCD	1.0000	0.0000	1	1	11019	Unique	Used	0.469	2.182E3	1.386E3	95	4.68	0.00	1	44	300	3	659.05853	1975.16104	1.09	57.40	5553	5553	MS2	26/52	Glioma_Nuclear_prep_Gradeall_repeat_4plex.raw
212549553	High		ekPTTALLDk	Unambiguous	5	1	N-Term(iTRAQ4plex); K2(iTRAQ4plex); K10(iTRAQ4plex)	HCD	1.0000	0.0000	1	1	8124	Unique	Used	0.342	2.235E5	9.611E4	191	3.35	0.00	0	29	8	2	774.45801	1547.90874	-18.20	27.24	2587	2587	MS2	17/18	Glioma_Nuclear_prep_Gradeall_4plex.raw
212549553	High		ekPTTALLDk	Unambiguous	5	1	N-Term(iTRAQ4plex); K2(iTRAQ4plex); K10(iTRAQ4plex)	HCD	1.0000	0.0000	1	1	8129	Unique	Used	0.645	8.797E4	7.135E4	175	3.41	0.00	0	45	3	3	516.64087	1547.90805	-18.64	27.28	2593	2593	MS2	17/36	Glioma_Nuclear_prep_Gradeall_4plex.raw
212549553		High	ekPTTALLDk	Unambiguous	5	1	N-Term(iTRAQ4plex); K2(iTRAQ4plex); K10(iTRAQ4plex)	HCD	1.0000	0.0000	1	1	8501	Unique	Used	0.308	8.018E3	3.341E3	132	3.09	0.00	0	27	271	2	774.47339	1547.93950	1.67	28.62	2854	2854	MS2	16/18	Glioma_Nuclear_prep_Gradeall_repeat_4plex.raw
212549553		High	ekPTTALLDk	Unambiguous	5	1	N-Term(iTRAQ4plex); K2(iTRAQ4plex); K10(iTRAQ4plex)	HCD	1.0000	0.0000	1	1	8489	Unique	Used	0.590	5.100E3	4.077E3	142	3.85	0.00	0	57	116	3	516.64893	1547.93222	-3.03	28.49	2841	2841	MS2	20/36	Glioma_Nuclear_prep_Gradeall_repeat_4plex.raw
212549553	High		nENPVDYTVQIPPSTTYAITPMk	Unambiguous	5	1	N-Term(iTRAQ4plex); K23(iTRAQ4plex)	HCD	1.0000	0.0000	1	1	11083	Unique	Used	0.266	7.472E3	2.497E3	105	5.68	0.00	0	0	45	2	1434.23572	2867.46416	-3.43	49.10	5811	5811	MS2	25/44	Glioma_Nuclear_prep_Gradeall_4plex.raw
212549553	High		INQLkPGLQYk	Unambiguous	5	1	N-Term(iTRAQ4plex); K5(iTRAQ4plex); K11(iTRAQ4plex)	HCD	1.0000	0.0000	1	1	8705	Unique	Used	0.337	3.212E5	1.361E5	118	4.35	0.00	0	26	9	2	867.52228	1734.03728	-15.31	31.41	3219	3219	MS2	17/20	Glioma_Nuclear_prep_Gradeall_4plex.raw
212549553		High	qQGPILTk	Unambiguous	2	1	N-Term(iTRAQ4plex); K8(iTRAQ4plex)	HCD	1.0000	0.0000	1	1	7755	Unique	Used	0.455	6.015E4	3.704E4	170	2.84	0.00	0	31	67	2	586.86450	1172.72173	-2.07	21.47	2068	2068	MS2	13/14	Glioma_Nuclear_prep_Gradeall_repeat_4plex.raw
212549553		High	fQGAGSNk	Unambiguous	5	1	N-Term(iTRAQ4plex); K8(iTRAQ4plex)	HCD	1.0000	0.0000	1	1	6896	Unique	Used	0.542	9.085E4	6.668E4	207	2.85	0.00	0	41	69	2	548.80084	1096.59441	-4.15	13.84	1158	1158	MS2	14/14	Glioma_Nuclear_prep_Gradeall_repeat_4plex.raw
212549553	High		IAAFGQLHk	Unambiguous	5	1	N-Term(iTRAQ4plex); K9(iTRAQ4plex)	HCD	1.0000	0.0000	1	1	8715	Unique	Used	0.186	5.752E5	1.346E5	195	4.13	0.00	0	10	1	3	424.92010	1272.74576	-16.45	31.48	3230	3230	MS2	23/32	Glioma_Nuclear_prep_Gradeall_4plex.raw
212549553	High		IAAFGQLHk	Unambiguous	5	1	N-Term(iTRAQ4plex); K9(iTRAQ4plex)	HCD	1.0000	0.0000	1	1	8726	Unique	Used	0.316	1.181E6	4.686E5	200	3.47	0.00	0	42	3	2	636.87769	1272.74809	-14.62	31.56	3242	3242	MS2	16/16	Glioma_Nuclear_prep_Gradeall_4plex.raw
212549553		High	IAAFGQLHk	Unambiguous	5	1	N-Term(iTRAQ4plex); K9(iTRAQ4plex)	HCD	1.0000	0.0000	1	1	9407	Unique	Used	0.252	1.191E4	4.058E3	161	4.53	0.00	0	20	101	3	424.92758	1272.76819	1.17	38.21	3812	3812	MS2	22/32	Glioma_Nuclear_prep_Gradeall_repeat_4plex.raw
212549553		High	IAAFGQLHk	Unambiguous	5	1	N-Term(iTRAQ4plex); K9(iTRAQ4plex)	HCD	1.0000	0.0000	1	1	9497	Unique	Used	0.292	1.099E4	4.349E3	172	4.25	0.00	0	25	61	3	424.92358	1272.75620	-8.25	39.22	3909	3909	MS2	21/32	Glioma_Nuclear_prep_Gradeall_repeat_4plex.raw
212549553		High	IAAFGQLHk	Unambiguous	5	1	N-Term(iTRAQ4plex); K9(iTRAQ4plex)	HCD	1.0000	0.0000	1	1	9450	Unique	Used	0.477	3.786E4	2.444E4	185	3.40	0.00	0	38	140	2	636.88879	1272.77031	2.84	38.67	3858	3858	MS2	16/16	Glioma_Nuclear_prep_Gradeall_repeat_4plex.raw
212549553		High	hSSVYPTQEELEAVQNmVSHTER	Unambiguous	5	1	N-Term(iTRAQ4plex); M17(Oxidation)	HCD	1.0000	0.0000	1	1	10866	Unique	Used	0.303	4.835E2	1.985E2	80	5.23	0.00	0	11	300	3	944.45221	2831.34208	1.18	55.49	5385	5385	MS2	22/88	Glioma_Nuclear_prep_Gradeall_repeat_4plex.raw
212549553		High	nENPVDYTVQIPPSTTYAITPmk	Unambiguous	5	1	N-Term(iTRAQ4plex); M22(Oxidation); K23(iTRAQ4plex)	HCD	1.0000	0.0000	1	1	11373	Unique	Used	0.682	6.745E1	6.230E1	106	3.60	0.00	0	8	300	3	961.82959	2883.47422	1.84	62.42	5980	5980	MS2	18/88	Glioma_Nuclear_prep_Gradeall_repeat_4plex.raw
212549553	High		vLGmDPLPSk	Unambiguous	5	1	N-Term(iTRAQ4plex); M4(Oxidation); K10(iTRAQ4plex)	HCD	1.0000	0.0000	1	1	8876	Unique	Used	0.407	2.482E5	1.271E5	262	3.85	0.00	0	36	18	2	680.87933	1360.75139	-17.28	32.63	3404	3404	MS2	15/18	Glioma_Nuclear_prep_Gradeall_4plex.raw
212549553		High	vLGmDPLPSk	Unambiguous	5	1	N-Term(iTRAQ4plex); M4(Oxidation); K10(iTRAQ4plex)	HCD	1.0000	0.0000	1	1	9342	Unique	Used	0.473	3.350E4	2.146E4	265	3.72	0.00	0	20	101	2	680.89307	1360.77886	2.91	37.54	3744	3744	MS2	15/18	Glioma_Nuclear_prep_Gradeall_repeat_4plex.raw
212549553	High		vLQDmGLPTGAEGR	Unambiguous	5	1	N-Term(iTRAQ4plex); M5(Oxidation)	HCD	1.0000	0.0000	1	1	8795	Unique	Used	0.515	4.299E4	2.784E4	170	3.59	0.00	0	34	43	2	802.40308	1603.79888	-15.11	32.04	3316	3316	MS2	19/26	Glioma_Nuclear_prep_Gradeall_4plex.raw
212549553		High	vLQDmGLPTGAEGR	Unambiguous	5	1	N-Term(iTRAQ4plex); M5(Oxidation)	HCD	1.0000	0.0000	1	1	9108	Unique	Used	0.392	6.699E3	3.558E3	261	4.07	0.00	0	14	300	2	802.41620	1603.82512	1.25	34.94	3497	3497	MS2	19/26	Glioma_Nuclear_prep_Gradeall_repeat_4plex.raw
213511012	NP_001132913.1	BCAP31	10134	B-cell receptor-associated protein 31 isoform b [Homo sapiens]	14.63	2	2	4	1.532	2;2	6.6	13.61	246	28.0	8.44																			
213511012	High		IkDELASTk	Unambiguous	2	1	N-Term(iTRAQ4plex); K2(iTRAQ4plex); K9(iTRAQ4plex)	HCD	1.0000	0.0000	1	1	7942	Unique	Used	1.541	1.218E5	2.361E5	168	3.10	0.00	1	26	12	2	718.93658	1436.86589	-1.81	25.95	2390	2390	MS2	16/16	Glioma_Nuclear_prep_Gradeall_4plex.raw
213511012		High	IkDELASTk	Unambiguous	2	1	N-Term(iTRAQ4plex); K2(iTRAQ4plex); K9(iTRAQ4plex)	HCD	1.0000	0.0000	1	1	8218	Unique	Used	1.384	9.616E3	1.803E4	160	2.89	0.00	1	12	132	2	718.93933	1436.87139	2.01	25.92	2556	2556	MS2	16/16	Glioma_Nuclear_prep_Gradeall_repeat_4plex.raw
213511012	High		IDVGNAEVk	Unambiguous	2	1	N-Term(iTRAQ4plex); K9(iTRAQ4plex)	HCD	1.0000	0.0000	1	1	8191	Unique	Used	1.523	2.698E5	5.167E5	282	3.70	0.00	0	41	6	2	616.84735	1232.68743	-17.43	27.71	2660	2660	MS2	16/16	Glioma_Nuclear_prep_Gradeall_4plex.raw
213511012		High	IDVGNAEVk	Unambiguous	2	1	N-Term(iTRAQ4plex); K9(iTRAQ4plex)	HCD	1.0000	0.0000	1	1	8469	Unique	Used	1.626	2.050E4	4.515E4	284	3.71	0.00	0	42	142	2	616.85895	1232.71062	1.38	28.30	2820	2820	MS2	16/16	Glioma_Nuclear_prep_Gradeall_repeat_4plex.raw

217330646	NP_006704.3	SUB1	10923	activated RNA polymerase II transcriptional coactivator p15 [Homo sapiens]	18.90	1	2	4	1.642	2;2	16.9	8.73	127	14.4	9.60																			
217330646	High		eQISDIDDAVR	Unambiguous	1	1	N-Term(iTRAQ4plex)	HCD	1.0000	0.0000	1	1	8813	Unique	Used	2.052	8.036E4	2.074E5	135	3.55	0.00	0	4	16	2	702.84607	1404.68486	-17.02	32.18	3336	3336	MS2	20/20	Glioma_Nuclear_prep_Gradeall_4plex.raw
217330646		High	eQISDIDDAVR	Unambiguous	1	1	N-Term(iTRAQ4plex)	HCD	1.0000	0.0000	1	1	9152	Unique	Used	1.640	1.048E4	2.329E4	196	4.45	0.00	0	11	237	2	702.85931	1404.71135	1.84	35.45	3544	3544	MS2	19/20	Glioma_Nuclear_prep_Gradeall_repeat_4plex.raw
217330646	High		gISLNPEQWSQLk	Unambiguous	1	1	N-Term(iTRAQ4plex); K13(iTRAQ4plex)	HCD	1.0000	0.0000	1	1	11102	Unique	Used	1.643	2.670E4	5.518E4	203	5.18	0.00	0	20	34	2	894.49408	1787.98088	-4.80	49.25	5832	5832	MS2	22/24	Glioma_Nuclear_prep_Gradeall_4plex.raw
217330646		High	gISLNPEQWSQLk	Unambiguous	1	1	N-Term(iTRAQ4plex); K13(iTRAQ4plex)	HCD	1.0000	0.0000	1	1	11892	Unique	Used	1.367	2.211E2	4.096E2	201	4.02	0.00	0	25	300	3	596.66736	1787.98752	-1.08	70.08	6625	6625	MS2	21/48	Glioma_Nuclear_prep_Gradeall_repeat_4plex.raw
221316767	NP_001138246.1	ELAVL4	1996	ELAV-like protein 4 isoform 2 [Homo sapiens]	24.04	6	1	2	0.418	1;1	35.6	23.79	366	40.4	9.39																			
221316767	High		sSQALLSQLYQSPNR	Unambiguous	6	1	N-Term(iTRAQ4plex)	HCD	1.0000	0.0000	1	1	10150	Unique	Used	0.523	3.118E4	2.051E4	268	3.99	0.00	0	0	43	2	918.48303	1835.95879	-7.87	41.96	4793	4793	MS2	17/28	Glioma_Nuclear_prep_Gradeall_4plex.raw
221316767		High	sSQALLSQLYQSPNR	Unambiguous	6	1	N-Term(iTRAQ4plex)	HCD	1.0000	0.0000	1	1	10892	Unique	Used	0.313	2.419E3	1.025E3	278	4.28	0.00	0	63	300	2	918.49414	1835.98100	4.23	55.83	5414	5414	MS2	20/28	Glioma_Nuclear_prep_Gradeall_repeat_4plex.raw
222352151	NP_006187.2	PCBP1	5093	poly(rC)-binding protein 1 [Homo sapiens]	15.45	1	2	3	1.643	2;1	22.7	18.52	356	37.5	7.09																			
222352151	High		IVVPATQcGSLIGk	Unambiguous	1	1	N-Term(iTRAQ4plex); C8(Methylthio); K14(iTRAQ4plex)	HCD	1.0000	0.0000	1	1	11244	Unique	Used	2.199	4.609E4	1.274E5	178	4.80	0.00	0	20	38	2	860.49133	1719.97539	0.80	50.39	5987	5987	MS2	17/26	Glioma_Nuclear_prep_Gradeall_4plex.raw
222352151	High		iITLTGPTNAIFk	Unambiguous	1	1	N-Term(iTRAQ4plex); K13(iTRAQ4plex)	HCD	1.0000	0.0000	1	1	11127	Unique	Used	1.426	3.303E4	5.926E4	107	4.49	0.00	0	44	52	2	839.01410	1677.02092	1.17	49.43	5859	5859	MS2	19/24	Glioma_Nuclear_prep_Gradeall_4plex.raw
222352151		High	iITLTGPTNAIFk	Unambiguous	1	1	N-Term(iTRAQ4plex); K13(iTRAQ4plex)	HCD	1.0000	0.0000	1	1	11947	Unique	Used	1.643	1.250E3	2.782E3	92	4.46	0.00	0	25	300	2	839.01489	1677.02251	2.11	71.08	6705	6705	MS2	21/24	Glioma_Nuclear_prep_Gradeall_repeat_4plex.raw
223555917	NP_848927.2	MTDH	92140	protein LYRIC [Homo sapiens]	7.04	1	3	6	0.645	3;3	12.4	13.12	582	63.8	9.32																			
223555917	High		tVEVAEGEAVR	Unambiguous	1	1	N-Term(iTRAQ4plex)	HCD	1.0000	0.0000	1	1	7923	Unique	Used	0.686	1.041E5	8.978E4	250	3.13	0.00	0	51	27	2	652.35291	1303.69853	0.81	25.81	2369	2369	MS2	18/20	Glioma_Nuclear_prep_Gradeall_4plex.raw
223555917		High	tVEVAEGEAVR	Unambiguous	1	1	N-Term(iTRAQ4plex)	HCD	1.0000	0.0000	1	1	8093	Unique	Used	0.762	3.422E4	3.530E4	256	3.09	0.00	0	42	143	2	652.35114	1303.69499	-1.90	24.68	2424	2424	MS2	18/20	Glioma_Nuclear_prep_Gradeall_repeat_4plex.raw
223555917	High		tELGLDLGLEPk	Unambiguous	1	1	N-Term(iTRAQ4plex); K12(iTRAQ4plex)	HCD	1.0000	0.0000	1	1	10495	Unique	Used	0.603	8.597E4	6.521E4	224	5.08	0.00	0	30	24	2	786.94739	1572.88750	-13.52	44.61	5171	5171	MS2	21/22	Glioma_Nuclear_prep_Gradeall_4plex.raw
223555917		High	tELGLDLGLEPk	Unambiguous	1	1	N-Term(iTRAQ4plex); K12(iTRAQ4plex)	HCD	1.0000	0.0000	1	1	11316	Unique	Used	0.557	3.087E3	2.329E3	183	4.76	0.00	0	38	300	2	786.96039	1572.91350	3.02	61.57	5909	5909	MS2	21/22	Glioma_Nuclear_prep_Gradeall_repeat_4plex.raw
223555917	High		eEAAAVPAAAPDDLALLk	Unambiguous	1	1	N-Term(iTRAQ4plex); K18(iTRAQ4plex)	HCD	1.0000	0.0000	1	1	10623	Unique	Used	0.594	1.343E4	1.003E4	215	4.27	0.00	0	26	52	2	1027.05896	2053.11064	-15.30	45.56	5310	5310	MS2	17/34	Glioma_Nuclear_prep_Gradeall_4plex.raw
223555917		High	eEAAAVPAAAPDDLALLk	Unambiguous	1	1	N-Term(iTRAQ4plex); K18(iTRAQ4plex)	HCD	1.0000	0.0000	1	1	11440	Unique	Used	0.723	6.660E2	6.525E2	145	5.27	0.00	0	36	300	3	685.05408	2053.14768	2.74	63.35	6060	6060	MS2	25/68	Glioma_Nuclear_prep_Gradeall_repeat_4plex.raw
224282117	NP_055699.2	SUGP2	10147	SURP and G-patch domain-containing protein 2 [Homo sapiens]	2.87	1	3	3	0.638	1;2	13.3	6.77	1082	120.1	7.28																			
224282117		High	aPSSLSDAVPQR	Unambiguous	1	1	N-Term(iTRAQ4plex)	HCD	1.0000	0.0000	1	1	8562	Unique	Used	0.721	1.001E4	9.770E3	266	3.81	0.00	0	18	300	2	686.37158	1371.73589	0.71	29.24	2918	2918	MS2	17/22	Glioma_Nuclear_prep_Gradeall_repeat_4plex.raw
224282117	High		vVGTIDQLVk	Unambiguous	1	1	N-Term(iTRAQ4plex); K10(iTRAQ4plex)	HCD	1.0000	0.0000	1	1	10029	Unique	Used	0.638	2.004E5	1.608E5	239	3.71	0.00	0	51	13	2	680.41498	1359.82268	-16.43	41.07	4661	4661	MS2	18/18	Glioma_Nuclear_prep_Gradeall_4plex.raw
224282117		High	vIEGSLSPk	Unambiguous	1	1	N-Term(iTRAQ4plex); K9(iTRAQ4plex)	HCD	1.0000	0.0000	1	1	8518	Unique	Used	0.551	1.896E4	1.415E4	242	2.96	0.00	0	71	300	2	609.37079	1217.73430	-0.08	28.81	2872	2872	MS2	15/16	Glioma_Nuclear_prep_Gradeall_repeat_4plex.raw
224593278	NP_060365.7	DDX27	55661	probable ATP-dependent RNA helicase DDX27 [Homo sapiens]	4.15	1	3	3	1.728	2;1	13.9	8.84	796	89.8	9.28																			
224593278	High		aLQEFDLALR	Unambiguous	1	1	N-Term(iTRAQ4plex)	HCD	1.0000	0.0000	1	1	10855	Unique	Used	1.600	3.710E4	7.464E4	310	3.57	0.00	0	38	53	2	660.36578	1319.72429	-14.95	47.33	5563	5563	MS2	17/18	Glioma_Nuclear_prep_Gradeall_4plex.raw
224593278	High		iFVNSNTDVAPFLR	Unambiguous	1	1	N-Term(iTRAQ4plex)	HCD	1.0000	0.0000	1	1	11287	Unique	Used	2.083	1.814E4	4.751E4	311	5.27	0.00	0	31	127	2	868.97626	1736.94524	-0.01	50.75	6034	6034	MS2	21/26	Glioma_Nuclear_prep_Gradeall_4plex.raw
224593278		High	iLPQDVILk	Unambiguous	1	1	N-Term(iTRAQ4plex); K9(iTRAQ4plex)	HCD	1.0000	0.0000	1	1	11091	Unique	Used	1.728	3.047E3	7.132E3	57	3.16	0.00	0	62	300	2	663.93384	1326.86040	0.35	58.32	5634	5634	MS2	13/16	Glioma_Nuclear_prep_Gradeall_repeat_4plex.raw
228008295	NP_001153147.1	SYNCRIP	10492	heterogeneous nuclear ribonucleoprotein Q isoform 4 [Homo sapiens]	30.78	7	9	15	0.261	12;3	38.1	106.20	588	65.6	8.60																			
228008295	High		IDEIYVAGLVAHSDLDER	Unambiguous	5	1	N-Term(iTRAQ4plex)	HCD	1.0000	0.0000	1	1	11593	Unique	Used	0.121	1.831E4	2.795E3	166	7.84	0.00	0	15	13	3	720.37469	2159.10953	-0.29	53.36	6369	6369	MS2	40/68	Glioma_Nuclear_prep_Gradeall_4plex.raw
228008295	High		lMMDPLTGLNR	Unambiguous	7	1	N-Term(iTRAQ4plex)	HCD	1.0000	0.0000	1	1	10909	Unique	Used	0.261	1.941E5	6.361E4	199	3.24	0.00	0	34	23	2	702.86682	1404.72637	-14.00	47.75	5622	5622	MS2	16/20	Glioma_Nuclear_prep_Gradeall_4plex.raw
228008295	High		gYAFVTFcTk	Unambiguous	7	1	N-Term(iTRAQ4plex); C8(Methylthio); K10(iTRAQ4plex)	HCD	1.0000	0.0000	1	1	11560	Unique	Used	0.264	5.427E4	1.802E4	177	4.03	0.00	0	69	77	2	735.87152	1470.73577	-0.42	53.09	6333	6333	MS2	15/18	Glioma_Nuclear_prep_Gradeall_4plex.raw
228008295	High		dLFEDELVPLFEk	Unambiguous	7	1	N-Term(iTRAQ4plex); K13(iTRAQ4plex)	HCD	1.0000	0.0000	1	1	12851	Unique	Used	0.115	7.689E4	1.110E4	203	5.71	0.00	0	8	61	2	941.51093	1882.01457	3.03	67.20	7782	7782	MS2	19/24	Glioma_Nuclear_prep_Gradeall_4plex.raw
228008295	High		nLANTVTEEILEk	Unambiguous	7	1	N-Term(iTRAQ4plex); K13(iTRAQ4plex)	HCD	1.0000	0.0000	1	1	11247	Unique	Used	0.157	9.206E4	1.813E4	210	5.95	0.00	0	20	44	2	881.49792	1761.98857	2.76	50.42	5991	5991	MS2	24/24	Glioma_Nuclear_prep_Gradeall_4plex.raw
228008295	High		dLFEDELVPLFEk	Unambiguous	7	1	N-Term(iTRAQ4plex); K13(iTRAQ4plex)	HCD	1.0000	0.0000	1	1	12852	Unique	Used	0.168	3.935E3	8.302E2	196	3.46	0.00	0	7	49	3	628.01019	1882.01603	3.80	67.21	7783	7783	MS2	19/48	Glioma_Nuclear_prep_Gradeall_4plex.raw
228008295	High		dLFEDELVPLFEk	Unambiguous	7	1	N-Term(iTRAQ4plex); K13(iTRAQ4plex)	HCD	1.0000	0.0000	1	1	12816	Unique	Used	0.175	6.014E3	1.324E3	209	5.82	0.00	0	8	300	2	941.50970	1882.01213	1.73	66.56	7732	7732	MS2	20/24	Glioma_Nuclear_prep_Gradeall_4plex.raw
228008295		High	nLANTVTEEILEk	Unambiguous	7	1	N-Term(iTRAQ4plex); K13(iTRAQ4plex)	HCD	1.0000	0.0000	1	1	12013	Unique	Used	0.145	2.906E3	5.707E2	211	5.69	0.00	0	34	300	2	881.49847	1761.98967	3.38	72.25	6799	6799	MS2	24/24	Glioma_Nuclear_prep_Gradeall_repeat_4plex.raw
228008295	High		eFNEDGALAVLQQFk	Unambiguous	5	1	N-Term(iTRAQ4plex); K15(iTRAQ4plex)	HCD	1.0000	0.0000	1	1	12405	Unique	Used	0.203	1.690E4	4.307E3	199	7.53	0.00	0	40	300	2	999.03064	1997.05400	-2.15	61.19	7257	7257	MS2	24/28	Glioma_Nuclear_prep_Gradeall_4plex.raw
228008295	High		eFNEDGALAVLQQFk	Unambiguous	5	1	N-Term(iTRAQ4plex); K15(iTRAQ4plex)	HCD	1.0000	0.0000	1	1	12450	Unique	Used	0.382	2.502E3	1.202E3	194	6.45	0.00	0	18	123	3	666.35815	1997.05991	0.81	61.72	7308	7308	MS2	26/56	Glioma_Nuclear_prep_Gradeall_4plex.raw
228008295	High		vTEGLTDVILYHQPDDk	Unambiguous	7	1	N-Term(iTRAQ4plex); K17(iTRAQ4plex)	HCD	1.0000	0.0000	1	1	10341	Unique	Used	0.263	5.304E4	1.754E4	148	6.68	0.00	0	13	6	3	744.38763	2231.14835	-14.12	43.39	5001	5001	MS2	34/64	Glioma_Nuclear_prep_Gradeall_4plex.raw
228008295		High	vTEGLTDVILYHQPDDk	Unambiguous	7	1	N-Term(iTRAQ4plex); K17(iTRAQ4plex)	HCD	1.0000	0.0000	1	1	11260	Unique	Used	0.270	9.184E2	3.356E2	170	6.83	0.00	0	21	300	3	744.39984	2231.18497	2.30	60.58	5830	5830	MS2	34/64	Glioma_Nuclear_prep_Gradeall_repeat_4plex.raw
228008295	High		yGGPPPDSVYSGQQPSVGTEIFVGk	Unambiguous	6	1	N-Term(iTRAQ4plex); K25(iTRAQ4plex)	HCD	1.0000	0.0000	1	1	10671	Unique	Used	0.298	7.319E3	2.741E3	94	5.60	0.00	0	4	24	3	952.14148	2854.40989	-14.14	45.93	5363	5363	MS2	29/96	Glioma_Nuclear_prep_Gradeall_4plex.raw
228008295	High		eQILEEFSk	Unambiguous	7	1	N-Term(iTRAQ4plex); K9(iTRAQ4plex)	HCD	1.0000	0.0000	1	1	9923	Unique	Used	0.269	2.914E5	9.842E4	269	3.78	0.00	0	28	11	2	705.87970	1410.75212	-14.02	40.30	4546	4546	MS2	16/16	Glioma_Nuclear_prep_Gradeall_4plex.raw
228008295		High	eQILEEFSk	Unambiguous	7	1	N-Term(iTRAQ4plex); K9(iTRAQ4plex)	HCD	1.0000	0.0000	1	1	10673	Unique	Used	0.407	1.419E4	7.827E3	298	4.03	0.00	0	24	300	2	705.89209	1410.77690	3.54	53.11	5175	5175	MS2	16/16	Glioma_Nuclear_prep_Gradeall_repeat_4plex.raw
264681410	NP_001161086.1	LEMD3	23592	inner nuclear membrane protein Man1 isoform 2 [Homo sapiens]	3.85	2	3	4	0.655	2;2	6.1	14.00	910	99.8	7.78																			
264681410	High		iGGADFLVWR	Unambiguous	2	1	N-Term(iTRAQ4plex)	HCD	1.0000	0.0000	1	1	11505	Unique	Used	0.663	3.771E4	3.146E4	270	3.44	0.00	0	43	77	2	639.35510	1277.70293	-7.36	52.64	6273	6273	MS2	16/18	Glioma_Nuclear_prep_Gradeall_4plex.raw
264681410	High		tLSVQEAAAYLk	Unambiguous	2	1	N-Term(iTRAQ4plex); K12(iTRAQ4plex)	HCD	1.0000	0.0000	1	1	10562	Unique	Used	0.673	1.053E5	8.917E4	256	4.81	0.00	0	55	26	2	791.44751	1581.88774	-13.48	45.10	5244	5244	MS2	21/22	Glioma_Nuclear_prep_Gradeall_4plex.raw
264681410		High	tLSVQEAAAYLk	Unambiguous	2	1	N-Term(iTRAQ4plex); K12(iTRAQ4plex)	HCD	1.0000	0.0000	1	1	11387	Unique	Used	0.646	4.582E3	4.011E3	225	5.41	0.00	0	36	300	2	791.46039	1581.91350	2.80	62.61	5996	5996	MS2	21/22	Glioma_Nuclear_prep_Gradeall_repeat_4plex.raw
264681410		High	lSEPEEELLQQFk	Unambiguous	2	1	N-Term(iTRAQ4plex); K13(iTRAQ4plex)	HCD	1.0000	0.0000	1	1	12240	Unique	Used	0.586	3.276E2	2.599E2	202	4.52	0.00	0	19	300	3	626.67609	1878.01371	2.01	77.20	7181	7181	MS2	24/48	Glioma_Nuclear_prep_Gradeall_repeat_4plex.raw
283135173	NP_001164408.1	FUS	2521	RNA-binding protein FUS isoform 3 [Homo sapiens]	6.70	5	3	5	1.486	2;3	10.8	11.77	522	53.2	9.36																			
283135173	High		gEATVSFDDPPSAk	Unambiguous	5	1	N-Term(iTRAQ4plex); K14(iTRAQ4plex)	HCD	1.0000	0.0000	1	1	8541	Unique	Used	1.654	5.654E4	1.176E5	114	4.29	0.00	0	0	12	2	854.92249	1708.83769	-14.97	30.19	3039	3039	MS2	19/26	Glioma_Nuclear_prep_Gradeall_4plex.raw
283135173		High	gEATVSFDDPPSAk	Unambiguous	5	1	N-Term(iTRAQ4plex); K14(iTRAQ4plex)	HCD	1.0000	0.0000	1	1	8881	Unique	Used	1.486	4.570E3	9.200E3	149	4.82	0.00	0	12	224	2	854.93512	1708.86296	-0.18	32.47	3254	3254	MS2	20/26	Glioma_Nuclear_prep_Gradeall_repeat_4plex.raw
283135173	High		aAIDWFDGk	Unambiguous	5	1	N-Term(iTRAQ4plex); K9(iTRAQ4plex)	HCD	1.0000	0.0000	1	1	10328	Unique	Used	1.358	3.433E4	5.864E4	254	3.25	0.00	0	44	31	2	655.84265	1310.67803	-15.51	43.27	4986	4986	MS2	16/16	Glioma_Nuclear_prep_Gradeall_4plex.raw
283135173		High	aAIDWFDGk	Unambiguous	5	1	N-Term(iTRAQ4plex); K9(iTRAQ4plex)	HCD	1.0000	0.0000	1	1	11153	Unique	Used	1.755	4.259E3	1.013E4	283	3.77	0.00	0	34	300	2	655.85443	1310.70159	2.46	59.17	5707	5707	MS2	16/16	Glioma_Nuclear_prep_Gradeall_repeat_4plex.raw
283135173		High	tGQPmINLYTDR	Unambiguous	3	1	N-Term(iTRAQ4plex); M5(Oxidation)	HCD	1.0000	0.0000	1	1	9277	Unique	Used	1.416	3.510E3	6.734E3	230	3.18	0.00	0	34	300	2	784.89948	1568.79167	3.64	36.82	3675	3675	MS2	17/22	Glioma_Nuclear_prep_Gradeall_repeat_4plex.raw
283806604	NP_001164554.1	SMPD4	55627	sphingomyelin phosphodiesterase 4 isoform 3 [Homo sapiens]	6.81	3	2	3	1.766	2;1	12.6	11.83	764	86.1	8.65																			
283806604	High		gEQLFLEPELVIPHR	Unambiguous	3	1	N-Term(iTRAQ4plex)	HCD	1.0000	0.0000	1	1	11277	Unique	Used	1.766	3.319E3	7.372E3	198	4.52	0.00	0	52	54	3	641.02673	1921.06565	-0.42	50.66	6023	6023	MS2	21/56	Glioma_Nuclear_prep_Gradeall_4plex.raw
283806604		High	gEQLFLEPELVIPHR	Unambiguous	3	1	N-Term(iTRAQ4plex)	HCD	1.0000	0.0000	1	1	12084	Unique	Used	2.163	1.224E2	3.584E2	201	4.05	0.00	0	36	300	3	641.02783	1921.06894	1.30	73.97	6928	6928	MS2	20/56	Glioma_Nuclear_prep_Gradeall_repeat_4plex.raw
283806604	High		qLPDcIVGEDGLILTPLGR	Unambiguous	3	1	N-Term(iTRAQ4plex); C5(Methylthio)	HCD	1.0000	0.0000	1	1	12815	Unique	Used	1.734	2.623E2	5.720E2	166	4.00	0.00	0	12	300	3	733.72546	2199.16184	-0.75	66.55	7731	7731	MS2	20/72	Glioma_Nuclear_prep_Gradeall_4plex.raw
302129652	NP_001180473.1	ANXA6	309	annexin A6 isoform 2 [Homo sapiens]	14.35	2	7	10	2.405	7;3	37.1	45.45	641	72.4	5.60																			
302129652	High		sEIDLLNIR	Unambiguous	2	1	N-Term(iTRAQ4plex)	HCD	1.0000	0.0000	1	1	10337	Unique	Used	1.373	3.066E4	5.294E4	274	3.00	0.00	0	63	56	2	608.84528	1216.68328	-15.24	43.35	4996	4996	MS2	15/16	Glioma_Nuclear_prep_Gradeall_4plex.raw
302129652	High		gLGTDEDTIIDIITHR	Unambiguous	2	1	N-Term(iTRAQ4plex)	HCD	1.0000	0.0000	1	1	12389	Unique	Used	2.199	1.186E3	3.279E3	148	7.08	0.00	0	9	66	3	638.34082	1913.00791	-0.94	61.03	7240	7240	MS2	29/60	Glioma_Nuclear_prep_Gradeall_4plex.raw
302129652	High		iLISLATGHR	Unambiguous	2	1	N-Term(iTRAQ4plex)	HCD	1.0000	0.0000	1	1	9420	Unique	Used	2.382	1.722E3	5.160E3	137	4.13	0.00	0	9	39	3	408.91583	1224.73294	-17.61	36.65	3999	3999	MS2	22/36	Glioma_Nuclear_prep_Gradeall_4plex.raw
302129652	High		aLIEILATR	Unambiguous	2	1	N-Term(iTRAQ4plex)	HCD	1.0000	0.0000	1	1	10974	Unique	Used	2.455	1.131E4	3.491E4	186	3.36	0.00	0	57	97	2	572.35565	1143.70403	-15.56	48.24	5692	5692	MS2	15/16	Glioma_Nuclear_prep_Gradeall_4plex.raw
302129652	High		eAILDIITSR	Unambiguous	2	1	N-Term(iTRAQ4plex)	HCD	1.0000	0.0000	1	1	11179	Unique	Used	3.802	3.911E4	1.870E5	224	3.48	0.00	0	31	15	2	637.87250	1274.73772	-4.68	49.87	5917	5917	MS2	16/18	Glioma_Nuclear_prep_Gradeall_4plex.raw
302129652	High		aLIEILATR	Unambiguous	2	1	N-Term(iTRAQ4plex)	HCD	1.0000	0.0000	1	1	11018	Unique	Used	3.948	2.733E4	1.357E5	137	3.28	0.00	0	20	18	2	572.36298	1143.71868	-2.75	48.58	5740	5740	MS2	15/16	Glioma_Nuclear_prep_Gradeall_4plex.raw
302129652		High	eAILDIITSR	Unambiguous	2	1	N-Term(iTRAQ4plex)	HCD	1.0000	0.0000	1	1	11938	Unique	Used	2.429	1.128E3	3.711E3	324	3.33	0.00	0	47	300	2	637.87555	1274.74382	0.11	70.87	6689	6689	MS2	16/18	Glioma_Nuclear_prep_Gradeall_repeat_4plex.raw
302129652		High	aLIEILATR	Unambiguous	2	1	N-Term(iTRAQ4plex)	HCD	1.0000	0.0000	1	1	11871	Unique	Used	3.912	1.168E3	6.192E3	93	3.10	0.00	0	29	300	2	572.36462	1143.72197	0.13	69.77	6599	6599	MS2	15/16	Glioma_Nuclear_prep_Gradeall_repeat_4plex.raw
302129652	High		lIVGLMRPPAYcDAk	Unambiguous	2	1	N-Term(iTRAQ4plex); C12(Methylthio); K15(iTRAQ4plex)	HCD	1.0000	0.0000	1	1	11160	Unique	Used	1.821	6.246E3	1.431E4	161	3.43	0.00	0	28	54	3	661.02423	1981.05814	-4.78	49.72	5896	5896	MS2	16/56	Glioma_Nuclear_prep_Gradeall_4plex.raw
302129652		High	vFQEFIk	Unambiguous	2	1	N-Term(iTRAQ4plex); K7(iTRAQ4plex)	HCD	1.0000	0.0000	1	1	10878	Unique	Used	1.615	3.929E3	8.593E3	253	2.83	0.00	0	69	300	2	599.85590	1198.70452	-2.45	55.64	5398	5398	MS2	12/12	Glioma_Nuclear_prep_Gradeall_repeat_4plex.raw
312433998	NP_001186078.1	LTF	4057	lactotransferrin isoform 2 [Homo sapiens]	18.32	2	7	11	4.622	6;5	37.9	41.49	666	73.1	7.85																			
312433998	High		cGLVPVLAENYk	Unambiguous	2	1	N-Term(iTRAQ4plex); C1(Methylthio); K12(iTRAQ4plex)	HCD	1.0000	0.0000	1	1	11169	Unique	Used	5.224	2.257E4	1.483E5	237	4.38	0.00	0	38	29	2	820.44464	1639.88201	1.81	49.80	5906	5906	MS2	16/22	Glioma_Nuclear_prep_Gradeall_4plex.raw
312433998		High	cGLVPVLAENYk	Unambiguous	2	1	N-Term(iTRAQ4plex); C1(Methylthio); K12(iTRAQ4plex)	HCD	1.0000	0.0000	1	1	11981	Unique	Used	2.683	2.287E2	8.310E2	224	3.38	0.00	0	39	300	3	547.29791	1639.87918	0.09	71.68	6753	6753	MS2	18/44	Glioma_Nuclear_prep_Gradeall_repeat_4plex.raw
312433998		High	cGLVPVLAENYk	Unambiguous	2	1	N-Term(iTRAQ4plex); C1(Methylthio); K12(iTRAQ4plex)	HCD	1.0000	0.0000	1	1	11993	Unique	Used	3.509	1.170E3	5.561E3	222	3.36	0.00	0	29	300	2	820.44452	1639.88176	1.66	71.88	6769	6769	MS2	15/22	Glioma_Nuclear_prep_Gradeall_repeat_4plex.raw
312433998	High		cLAENAGDVAFVk	Unambiguous	2	1	N-Term(iTRAQ4plex); C1(Methylthio); K13(iTRAQ4plex)	HCD	1.0000	0.0000	1	1	10168	Unique	Used	7.178	2.320E4	2.094E5	123	5.60	0.00	0	28	40	2	835.91589	1670.82451	-14.35	42.11	4813	4813	MS2	23/24	Glioma_Nuclear_prep_Gradeall_4plex.raw
312433998		High	cLAENAGDVAFVk	Unambiguous	2	1	N-Term(iTRAQ4plex); C1(Methylthio); K13(iTRAQ4plex)	HCD	1.0000	0.0000	1	1	10969	Unique	Used	3.797	1.966E3	1.011E4	187	5.42	0.00	0	23	300	2	835.93066	1670.85405	3.33	56.77	5498	5498	MS2	23/24	Glioma_Nuclear_prep_Gradeall_repeat_4plex.raw
312433998	High		nLLFNDNTEcLAR	Unambiguous	2	1	N-Term(iTRAQ4plex); C10(Methylthio)	HCD	1.0000	0.0000	1	1	10815	Unique	Used	8.070	3.006E4	3.051E5	94	4.32	0.00	0	37	32	2	856.90283	1712.79839	-13.61	47.03	5520	5520	MS2	17/24	Glioma_Nuclear_prep_Gradeall_4plex.raw
312433998	High		fcLFQSETk	Unambiguous	2	1	N-Term(iTRAQ4plex); C2(Methylthio); K9(iTRAQ4plex)	HCD	1.0000	0.0000	1	1	10585	Unique	Used	2.671	3.929E4	1.320E5	156	3.23	0.00	0	61	52	2	718.85364	1436.70000	-10.89	45.28	5269	5269	MS2	14/16	Glioma_Nuclear_prep_Gradeall_4plex.raw
312433998	High		dSPIQcIQAIAENR	Unambiguous	2	1	N-Term(iTRAQ4plex); C6(Methylthio)	HCD	1.0000	0.0000	1	1	12573	Unique	Used	6.161	2.079E3	1.611E4	156	5.59	0.00	0	34	300	2	874.43414	1747.86101	1.25	63.29	7448	7448	MS2	23/26	Glioma_Nuclear_prep_Gradeall_4plex.raw
312433998		High	fQLFGSPSGQk	Unambiguous	2	1	N-Term(iTRAQ4plex); K11(iTRAQ4plex)	HCD	1.0000	0.0000	1	1	10447	Unique	Used	6.706	1.541E3	1.400E4	240	3.62	0.00	0	53	300	2	742.41290	1483.81853	2.53	50.39	4931	4931	MS2	18/20	Glioma_Nuclear_prep_Gradeall_repeat_4plex.raw
312433998	High		yLGPQYVAGITNLk	Unambiguous	2	1	N-Term(iTRAQ4plex); K14(iTRAQ4plex)	HCD	1.0000	0.0000	1	1	10903	Unique	Used	4.622	1.303E4	7.573E4	226	5.20	0.00	0	50	47	2	913.01501	1825.02275	-12.86	47.70	5615	5615	MS2	22/26	Glioma_Nuclear_prep_Gradeall_4plex.raw
312433998		High	yLGPQYVAGITNLk	Unambiguous	2	1	N-Term(iTRAQ4plex); K14(iTRAQ4plex)	HCD	1.0000	0.0000	1	1	11752	Unique	Used	3.633	2.235E2	1.100E3	167	4.80	0.00	0	24	300	3	609.02032	1825.04642	0.11	67.76	6435	6435	MS2	24/52	Glioma_Nuclear_prep_Gradeall_repeat_4plex.raw
316659409	NP_001186883.1	ACTG1	71	actin, cytoplasmic 2 [Homo sapiens]	36.27	8	2	5	1.867	3;2	14.6	59.70	375	41.8	5.48																			
316659409	High		tTGIVMDSGDGVTHTVPIYEGYALPHAILR	Unambiguous	2	1	N-Term(iTRAQ4plex)	HCD	1.0000	0.0000	1	1	11280	Unique	Used	2.253	8.101E2	2.295E3	77	8.19	0.00	0	0	75	3	1109.90894	3327.71225	-1.28	50.68	6026	6026	MS2	31/116	Glioma_Nuclear_prep_Gradeall_4plex.raw
316659409	High		vAPEEHPVLLTEAPLNPk	Unambiguous	2	1	N-Term(iTRAQ4plex); K18(iTRAQ4plex)	HCD	1.0000	0.0000	1	1	9604	Unique	Used	1.867	4.212E4	9.887E4	96	5.42	0.00	0	0	8	2	1121.61926	2242.23125	-16.67	38.00	4200	4200	MS2	22/34	Glioma_Nuclear_prep_Gradeall_4plex.raw
316659409	High		vAPEEHPVLLTEAPLNPk	Unambiguous	2	1	N-Term(iTRAQ4plex); K18(iTRAQ4plex)	HCD	1.0000	0.0000	1	1	9591	Unique	Used	2.068	2.845E4	7.399E4	115	5.98	0.00	0	6	3	3	748.08301	2242.23447	-15.23	37.91	4186	4186	MS2	30/68	Glioma_Nuclear_prep_Gradeall_4plex.raw
316659409		High	vAPEEHPVLLTEAPLNPk	Unambiguous	2	1	N-Term(iTRAQ4plex); K18(iTRAQ4plex)	HCD	1.0000	0.0000	1	1	10387	Unique	Used	1.597	9.502E2	2.056E3	113	7.07	0.00	0	12	200	3	748.09558	2242.27219	1.59	49.69	4868	4868	MS2	31/68	Glioma_Nuclear_prep_Gradeall_repeat_4plex.raw
316659409		High	vAPEEHPVLLTEAPLNPk	Unambiguous	2	1	N-Term(iTRAQ4plex); K18(iTRAQ4plex)	HCD	1.0000	0.0000	1	1	10474	Unique	Used	1.653	5.160E2	1.155E3	86	6.98	0.00	0	11	300	3	748.09558	2242.27219	1.59	50.71	4960	4960	MS2	30/68	Glioma_Nuclear_prep_Gradeall_repeat_4plex.raw
325651836	NP_003592.3	SMARCA5	8467	SWI/SNF-related matrix-associated actin-dependent regulator of chromatin subfamily A member 5 [Homo sapiens]	8.94	1	3	4	1.955	2;2	9.0	22.94	1052	121.8	8.09																			
325651836	High		eSEITDEDIDGILER	Unambiguous	1	1	N-Term(iTRAQ4plex)	HCD	1.0000	0.0000	1	1	10717	Unique	Used	2.100	3.353E4	8.857E4	84	4.50	0.00	0	8	30	2	939.44623	1877.88518	-13.08	46.27	5413	5413	MS2	25/28	Glioma_Nuclear_prep_Gradeall_4plex.raw
325651836		High	eSEITDEDIDGILER	Unambiguous	1	1	N-Term(iTRAQ4plex)	HCD	1.0000	0.0000	1	1	11419	Unique	Used	2.078	9.247E2	2.602E3	121	5.29	0.00	0	5	300	2	939.46045	1877.91362	2.07	63.06	6035	6035	MS2	26/28	Glioma_Nuclear_prep_Gradeall_repeat_4plex.raw
325651836		High	eIQEPDPTYEEk	Unambiguous	1	1	N-Term(iTRAQ4plex); K12(iTRAQ4plex)	HCD	1.0000	0.0000	1	1	8599	Unique	Used	1.832	3.060E3	7.593E3	139	4.42	0.00	0	32	300	2	883.43933	1765.87139	-1.18	29.62	2957	2957	MS2	16/22	Glioma_Nuclear_prep_Gradeall_repeat_4plex.raw
325651836	High		qTELFAHFIQPAAQk	Unambiguous	1	1	N-Term(iTRAQ4plex); K15(iTRAQ4plex)	HCD	1.0000	0.0000	1	1	10134	Unique	Used	1.753	1.837E4	4.051E4	191	4.99	0.00	0	33	17	3	673.03149	2017.07993	-15.39	41.85	4776	4776	MS2	22/56	Glioma_Nuclear_prep_Gradeall_4plex.raw
332801090	NP_001193929.1	HNRNPDL	9987	heterogeneous nuclear ribonucleoprotein D-like isoform b [Homo sapiens]	28.10	2	8	17	0.196	8;9	36.5	88.24	363	40.0	9.96																			
332801090	High		qLAPLLPSLAPSSAR	Unambiguous	2	1	N-Term(iTRAQ4plex)	HCD	1.0000	0.0000	1	1	10721	Unique	Used	0.282	1.045E5	3.710E4	212	3.47	0.00	0	28	27	2	832.98395	1664.96062	-12.63	46.31	5418	5418	MS2	17/28	Glioma_Nuclear_prep_Gradeall_4plex.raw
332801090		High	qLAPLLPSLAPSSAR	Unambiguous	2	1	N-Term(iTRAQ4plex)	HCD	1.0000	0.0000	1	1	11542	Unique	Used	0.251	3.116E3	1.058E3	154	3.49	0.00	0	31	300	2	832.99683	1664.98638	2.84	64.70	6177	6177	MS2	15/28	Glioma_Nuclear_prep_Gradeall_repeat_4plex.raw
332801090		High	dLTEYLSR	Unambiguous	2	1	N-Term(iTRAQ4plex)	HCD	1.0000	0.0000	1	1	10178	Unique	Used	0.297	1.041E4	4.183E3	264	2.84	0.00	0	46	300	2	570.80573	1140.60417	2.13	47.21	4645	4645	MS2	13/14	Glioma_Nuclear_prep_Gradeall_repeat_4plex.raw
332801090	High		gFcFITYTDEEPVk	Unambiguous	2	1	N-Term(iTRAQ4plex); C3(Methylthio); K14(iTRAQ4plex)	HCD	1.0000	0.0000	1	1	11811	Unique	Used	0.111	6.233E4	8.664E3	105	5.49	0.00	0	15	48	2	991.97717	1982.94707	-0.59	55.30	6606	6606	MS2	23/26	Glioma_Nuclear_prep_Gradeall_4plex.raw
332801090	High		gFcFITYTDEEPVk	Unambiguous	2	1	N-Term(iTRAQ4plex); C3(Methylthio); K14(iTRAQ4plex)	HCD	1.0000	0.0000	1	1	11735	Unique	Used	0.292	2.610E4	9.594E3	105	5.50	0.00	0	44	204	2	991.97693	1982.94658	-0.84	54.64	6523	6523	MS2	20/26	Glioma_Nuclear_prep_Gradeall_4plex.raw
332801090		High	gFcFITYTDEEPVk	Unambiguous	2	1	N-Term(iTRAQ4plex); C3(Methylthio); K14(iTRAQ4plex)	HCD	1.0000	0.0000	1	1	12298	Unique	Used	0.105	1.708E3	2.436E2	109	4.55	0.00	0	3	300	2	991.97919	1982.95110	1.44	78.97	7311	7311	MS2	22/26	Glioma_Nuclear_prep_Gradeall_repeat_4plex.raw
332801090	High		gFcFITYTDEEPVkk	Unambiguous	2	1	N-Term(iTRAQ4plex); C3(Methylthio); K14(iTRAQ4plex); K15(iTRAQ4plex)	HCD	1.0000	0.0000	1	1	10731	Unique	Used	0.071	9.524E4	8.542E3	88	4.89	0.00	1	12	4	3	752.37750	2255.11795	-12.12	46.39	5429	5429	MS2	30/56	Glioma_Nuclear_prep_Gradeall_4plex.raw
332801090		High	gFcFITYTDEEPVkk	Unambiguous	2	1	N-Term(iTRAQ4plex); C3(Methylthio); K14(iTRAQ4plex); K15(iTRAQ4plex)	HCD	1.0000	0.0000	1	1	11783	Unique	Used	0.225	1.061E3	3.236E2	114	5.19	0.00	1	10	300	3	752.38953	2255.15403	3.88	68.26	6476	6476	MS2	31/56	Glioma_Nuclear_prep_Gradeall_repeat_4plex.raw
332801090		High	kDLTEYLSR	Unambiguous	2	1	N-Term(iTRAQ4plex); K1(iTRAQ4plex)	HCD	1.0000	0.0000	1	1	9333	Unique	Used	0.226	1.740E4	5.328E3	203	3.11	0.00	1	35	300	2	706.90540	1412.80351	3.35	37.44	3734	3734	MS2	15/16	Glioma_Nuclear_prep_Gradeall_repeat_4plex.raw
332801090		High	kDLTEYLSR	Unambiguous	2	1	N-Term(iTRAQ4plex); K1(iTRAQ4plex)	HCD	1.0000	0.0000	1	1	9324	Unique	Used	0.331	4.108E3	1.840E3	184	3.65	0.00	1	34	127	3	471.60529	1412.80130	1.79	37.36	3725	3725	MS2	18/32	Glioma_Nuclear_prep_Gradeall_repeat_4plex.raw
332801090	High		mFIGGLSWDTSk	Unambiguous	2	1	N-Term(iTRAQ4plex); K12(iTRAQ4plex)	HCD	1.0000	0.0000	1	1	11143	Unique	Used	0.171	1.042E5	2.245E4	209	4.66	0.00	0	34	40	2	815.43390	1629.86052	3.42	49.57	5877	5877	MS2	20/22	Glioma_Nuclear_prep_Gradeall_4plex.raw
332801090		High	mFIGGLSWDTSk	Unambiguous	2	1	N-Term(iTRAQ4plex); K12(iTRAQ4plex)	HCD	1.0000	0.0000	1	1	11944	Unique	Used	0.126	3.861E3	6.592E2	202	3.93	0.00	0	34	300	2	815.43347	1629.85967	2.90	71.03	6701	6701	MS2	19/22	Glioma_Nuclear_prep_Gradeall_repeat_4plex.raw
332801090	High		vFVGGLSPDTSEEQIk	Unambiguous	2	1	N-Term(iTRAQ4plex); K16(iTRAQ4plex)	HCD	1.0000	0.0000	1	1	9918	Unique	Used	0.195	5.119E4	1.256E4	190	5.45	0.00	0	9	2	3	665.35211	1994.04178	-13.41	40.27	4541	4541	MS2	24/60	Glioma_Nuclear_prep_Gradeall_4plex.raw
332801090		High	vFVGGLSPDTSEEQIk	Unambiguous	2	1	N-Term(iTRAQ4plex); K16(iTRAQ4plex)	HCD	1.0000	0.0000	1	1	10583	Unique	Used	0.283	1.283E3	4.914E2	222	6.87	0.00	0	16	300	3	665.36316	1994.07492	3.21	52.03	5078	5078	MS2	24/60	Glioma_Nuclear_prep_Gradeall_repeat_4plex.raw
332801090	High		yHQIGSGk	Unambiguous	2	1	N-Term(iTRAQ4plex); K8(iTRAQ4plex)	HCD	1.0000	0.0000	1	1	7151	Unique	Used	0.185	5.367E4	1.248E4	361	3.61	0.00	0	41	50	3	393.22375	1177.65671	-0.06	20.16	1530	1530	MS2	15/28	Glioma_Nuclear_prep_Gradeall_4plex.raw
332801090	High		mFIGGLSWDTSk	Unambiguous	2	1	N-Term(iTRAQ4plex); M1(Oxidation); K12(iTRAQ4plex)	HCD	1.0000	0.0000	1	1	10370	Unique	Used	0.181	1.272E5	2.888E4	150	3.98	0.00	0	51	29	2	823.41852	1645.82976	-12.21	43.62	5033	5033	MS2	19/22	Glioma_Nuclear_prep_Gradeall_4plex.raw
332801090		High	mFIGGLSWDTSk	Unambiguous	2	1	N-Term(iTRAQ4plex); M1(Oxidation); K12(iTRAQ4plex)	HCD	1.0000	0.0000	1	1	11174	Unique	Used	0.196	4.852E3	1.285E3	197	3.97	0.00	0	25	300	2	823.43127	1645.85527	3.29	59.46	5732	5732	MS2	20/22	Glioma_Nuclear_prep_Gradeall_repeat_4plex.raw
366039949	NP_001242992.1	CNTN1	1272	contactin-1 isoform 3 precursor [Homo sapiens]	1.59	3	1	2	0.432	1;1	7.2	3.14	627	70.6	6.11																			
366039949	High		fIPLIPIPER	Unambiguous	3	1	N-Term(iTRAQ4plex)	HCD	1.0000	0.0000	1	1	11863	Unique	Used	0.454	2.101E4	1.201E4	189	3.14	0.00	0	43	110	2	669.91425	1338.82121	-4.05	55.74	6662	6662	MS2	11/18	Glioma_Nuclear_prep_Gradeall_4plex.raw
366039949		High	fIPLIPIPER	Unambiguous	3	1	N-Term(iTRAQ4plex)	HCD	1.0000	0.0000	1	1	12339	Unique	Used	0.410	2.119E3	1.178E3	141	3.11	0.00	0	27	300	2	669.91693	1338.82659	-0.04	80.42	7416	7416	MS2	12/18	Glioma_Nuclear_prep_Gradeall_repeat_4plex.raw
385198063	NP_001245239.1	NOP2	4839	probable 28S rRNA (cytosine(4447)-C(5))-methyltransferase isoform 4 [Homo sapiens]	5.25	4	3	3	2.666	2;1	31.9	8.63	628	69.6	6.58																			
385198063	High		iQDIVGILR	Unambiguous	4	1	N-Term(iTRAQ4plex)	HCD	1.0000	0.0000	1	1	11325	Unique	Used	3.541	3.484E4	1.551E5	74	3.20	0.00	0	42	34	2	585.86975	1170.73223	-0.43	51.09	6076	6076	MS2	14/16	Glioma_Nuclear_prep_Gradeall_4plex.raw
385198063		High	dLAQALINR	Unambiguous	4	1	N-Term(iTRAQ4plex)	HCD	1.0000	0.0000	1	1	10202	Unique	Used	1.830	4.765E3	1.181E4	216	3.18	0.00	0	65	300	2	579.34186	1157.67644	0.44	47.49	4670	4670	MS2	14/16	Glioma_Nuclear_prep_Gradeall_repeat_4plex.raw
385198063	High		vLLDAPcSGTGVISk	Unambiguous	4	1	N-Term(iTRAQ4plex); C7(Methylthio); K15(iTRAQ4plex)	HCD	1.0000	0.0000	1	1	10784	Unique	Used	2.666	2.962E4	9.931E4	274	5.43	0.00	0	24	48	2	897.47778	1793.94829	-14.56	46.79	5486	5486	MS2	21/28	Glioma_Nuclear_prep_Gradeall_4plex.raw
387598076	NP_001248376.1	GPM6A	2823	neuronal membrane glycoprotein M6-a isoform 2 [Homo sapiens]	14.40	4	2	2	0.484	1;1	36.3	7.37	243	26.8	7.36																			
387598076		High	icTVSENFLR	Unambiguous	4	1	N-Term(iTRAQ4plex); C2(Methylthio)	HCD	1.0000	0.0000	1	1	11891	Unique	Used	0.360	3.248E3	1.583E3	140	3.94	0.00	0	38	300	2	686.34784	1371.68840	0.18	70.07	6624	6624	MS2	17/18	Glioma_Nuclear_prep_Gradeall_repeat_4plex.raw

387598076	High		qFGIVTIGEEk	Unambiguous	4	1	N-Term(iTRAQ4plex); K11(iTRAQ4plex)	HCD	1.0000	0.0000	1	1	9814	Unique	Used	0.608	4.748E4	3.633E4	299	3.57	0.00	0	28	29	2	754.91913	1508.83098	-16.79	39.49	4427	4427	MS2	20/20	Glioma_Nuclear_prep_Gradeall_4plex.raw
390125204	NP_001254527.1	MGST1	4257	microsomal glutathione S-transferase 1 isoform c [Homo sapiens]	16.09	3	2	3	1.965	2;1	28.7	10.87	87	10.0	9.14																			
390125204	High		vFANPEDcVAFGk	Unambiguous	3	1	N-Term(iTRAQ4plex); C8(Methylthio); K13(iTRAQ4plex)	HCD	1.0000	0.0000	1	1	11118	Unique	Used	3.103	1.803E4	7.034E4	157	5.34	0.00	0	39	74	2	865.92578	1730.84429	-2.43	49.36	5849	5849	MS2	19/24	Glioma_Nuclear_prep_Gradeall_4plex.raw
390125204		High	vFANPEDcVAFGk	Unambiguous	3	1	N-Term(iTRAQ4plex); C8(Methylthio); K13(iTRAQ4plex)	HCD	1.0000	0.0000	1	1	11904	Unique	Used	1.965	1.928E2	5.130E2	92	3.83	0.00	0	24	300	3	577.62115	1730.84891	0.24	70.26	6640	6640	MS2	20/48	Glioma_Nuclear_prep_Gradeall_repeat_4plex.raw
390125204	High		kVFANPEDcVAFGk	Unambiguous	3	1	N-Term(iTRAQ4plex); K1(iTRAQ4plex); C9(Methylthio); K14(iTRAQ4plex)	HCD	1.0000	0.0000	1	1	10186	Unique	Used	1.925	2.243E4	5.430E4	133	5.53	0.00	1	18	8	3	668.34369	2003.01651	-14.48	42.25	4833	4833	MS2	23/52	Glioma_Nuclear_prep_Gradeall_4plex.raw
392513662	NP_001254738.1	ILF2	3608	interleukin enhancer-binding factor 2 isoform 2 [Homo sapiens]	26.14	2	7	15	0.326	8;7	35.1	49.89	352	38.9	4.94																			
392513662	High		vLQSALAAIR	Unambiguous	2	1	N-Term(iTRAQ4plex)	HCD	1.0000	0.0000	1	1	10687	Unique	Used	0.377	5.090E5	2.414E5	155	3.30	0.00	0	36	8	2	593.36700	1185.72673	-14.25	46.06	5381	5381	MS2	17/18	Glioma_Nuclear_prep_Gradeall_4plex.raw
392513662		High	vLQSALAAIR	Unambiguous	2	1	N-Term(iTRAQ4plex)	HCD	1.0000	0.0000	1	1	11601	Unique	Used	0.181	1.796E4	4.414E3	133	3.29	0.00	0	10	239	2	593.37604	1185.74480	0.99	65.59	6251	6251	MS2	17/18	Glioma_Nuclear_prep_Gradeall_repeat_4plex.raw
392513662		High	iLITTVPPNLR	Unambiguous	2	1	N-Term(iTRAQ4plex)	HCD	1.0000	0.0000	1	1	11106	Unique	Used	0.413	8.953E3	5.011E3	99	3.21	0.00	0	50	300	2	690.93884	1380.87041	0.62	58.52	5651	5651	MS2	12/20	Glioma_Nuclear_prep_Gradeall_repeat_4plex.raw
392513662	High		kLDPELHLDIk	Unambiguous	2	1	N-Term(iTRAQ4plex); K1(iTRAQ4plex); K11(iTRAQ4plex)	HCD	1.0000	0.0000	1	1	9098	Unique	Used	0.348	8.099E4	3.541E4	119	3.32	0.00	1	40	25	2	877.02209	1753.03691	-12.27	34.27	3647	3647	MS2	18/20	Glioma_Nuclear_prep_Gradeall_4plex.raw
392513662		High	lDPELHLDIk	Unambiguous	2	1	N-Term(iTRAQ4plex); K10(iTRAQ4plex)	HCD	1.0000	0.0000	1	1	10658	Unique	Used	0.441	1.220E3	7.280E2	221	3.65	0.00	0	25	300	3	494.29166	1480.86042	-0.66	52.92	5158	5158	MS2	17/36	Glioma_Nuclear_prep_Gradeall_repeat_4plex.raw
392513662	High		iLPTLEAVAALGNk	Unambiguous	2	1	N-Term(iTRAQ4plex); K14(iTRAQ4plex)	HCD	1.0000	0.0000	1	1	12396	Unique	Used	0.326	1.612E4	6.615E3	54	5.85	0.00	0	23	257	2	849.52515	1698.04302	1.51	61.08	7247	7247	MS2	21/26	Glioma_Nuclear_prep_Gradeall_4plex.raw
392513662	High		iLPTLEAVAALGNk	Unambiguous	2	1	N-Term(iTRAQ4plex); K14(iTRAQ4plex)	HCD	1.0000	0.0000	1	1	12378	Unique	Used	0.350	3.702E3	1.629E3	44	4.94	0.00	0	10	49	3	566.68488	1698.04007	-0.22	60.90	7228	7228	MS2	27/52	Glioma_Nuclear_prep_Gradeall_4plex.raw
392513662	High		iLPTLEAVAALGNk	Unambiguous	2	1	N-Term(iTRAQ4plex); K14(iTRAQ4plex)	HCD	1.0000	0.0000	1	1	12326	Unique	Used	0.467	2.801E4	1.645E4	54	5.03	0.00	0	59	184	2	849.52472	1698.04216	1.01	60.32	7171	7171	MS2	21/26	Glioma_Nuclear_prep_Gradeall_4plex.raw
392513662	High		vkPAPDETSFSEALLk	Unambiguous	2	1	N-Term(iTRAQ4plex); K2(iTRAQ4plex); K16(iTRAQ4plex)	HCD	1.0000	0.0000	1	1	9785	Unique	Used	0.307	2.672E4	1.031E4	153	7.04	0.00	0	15	6	3	722.06934	2164.19345	-13.48	39.29	4396	4396	MS2	29/60	Glioma_Nuclear_prep_Gradeall_4plex.raw
392513662		High	vkPAPDETSFSEALLk	Unambiguous	2	1	N-Term(iTRAQ4plex); K2(iTRAQ4plex); K16(iTRAQ4plex)	HCD	1.0000	0.0000	1	1	10557	Unique	Used	0.164	1.237E4	2.751E3	98	4.98	0.00	0	3	161	2	1082.61719	2164.22710	2.07	51.72	5050	5050	MS2	22/30	Glioma_Nuclear_prep_Gradeall_repeat_4plex.raw
392513662		High	vkPAPDETSFSEALLk	Unambiguous	2	1	N-Term(iTRAQ4plex); K2(iTRAQ4plex); K16(iTRAQ4plex)	HCD	1.0000	0.0000	1	1	10595	Unique	Used	0.245	3.811E2	1.262E2	109	5.26	0.00	0	21	245	4	541.81506	2164.23842	7.30	52.17	5091	5091	MS2	23/90	Glioma_Nuclear_prep_Gradeall_repeat_4plex.raw
392513662		High	vkPAPDETSFSEALLk	Unambiguous	2	1	N-Term(iTRAQ4plex); K2(iTRAQ4plex); K16(iTRAQ4plex)	HCD	1.0000	0.0000	1	1	10554	Unique	Used	0.269	1.096E4	3.997E3	103	7.53	0.00	0	6	18	3	722.08154	2164.23008	3.44	51.70	5047	5047	MS2	28/60	Glioma_Nuclear_prep_Gradeall_repeat_4plex.raw
392513662	High		nQDLAPNSAEQASILSLVTk	Unambiguous	2	1	N-Term(iTRAQ4plex); K20(iTRAQ4plex)	HCD	1.0000	0.0000	1	1	11550	Unique	Used	0.187	1.935E4	4.546E3	108	6.34	0.00	0	12	60	2	1194.15479	2387.30229	0.08	53.01	6322	6322	MS2	31/38	Glioma_Nuclear_prep_Gradeall_4plex.raw
392513662	High		nQDLAPNSAEQASILSLVTk	Unambiguous	2	1	N-Term(iTRAQ4plex); K20(iTRAQ4plex)	HCD	1.0000	0.0000	1	1	11479	Unique	Used	0.542	4.558E3	3.108E3	165	6.45	0.00	0	18	53	3	796.43842	2387.30069	-0.59	52.43	6245	6245	MS2	29/76	Glioma_Nuclear_prep_Gradeall_4plex.raw
392513662		High	nQDLAPNSAEQASILSLVTk	Unambiguous	2	1	N-Term(iTRAQ4plex); K20(iTRAQ4plex)	HCD	1.0000	0.0000	1	1	12185	Unique	Used	0.218	2.613E2	7.714E1	137	6.18	0.00	0	7	300	3	796.44135	2387.30948	3.09	75.79	7074	7074	MS2	30/76	Glioma_Nuclear_prep_Gradeall_repeat_4plex.raw
402534522	NP_001257973.1	NFIX	4784	nuclear factor 1 X-type isoform 3 [Homo sapiens]	4.85	8	1	2	1.590	1;1	4.5	7.29	433	47.9	8.53																			
402534522	High		eDFVLTITGk	Unambiguous	3	1	N-Term(iTRAQ4plex); K10(iTRAQ4plex)	HCD	1.0000	0.0000	1	1	10494	Unique	Used	1.641	7.093E4	1.464E5	281	4.01	0.00	0	33	21	2	705.89703	1410.78679	-15.24	44.60	5170	5170	MS2	15/18	Glioma_Nuclear_prep_Gradeall_4plex.raw
402534522		High	eDFVLTITGk	Unambiguous	3	1	N-Term(iTRAQ4plex); K10(iTRAQ4plex)	HCD	1.0000	0.0000	1	1	11337	Unique	Used	1.540	1.592E3	3.320E3	300	4.00	0.00	0	39	300	2	705.90833	1410.80937	0.76	61.94	5938	5938	MS2	16/18	Glioma_Nuclear_prep_Gradeall_repeat_4plex.raw
503774453	NP_055204.3	PELP1	27043	proline-, glutamic acid- and leucine-rich protein 1 isoform 1 [Homo sapiens]	2.97	2	2	2	1.652	1;1	0.5	12.02	1180	124.9	4.41																			
503774453	High		lLLLESVSGLLQPR	Unambiguous	1	1	N-Term(iTRAQ4plex)	HCD	1.0000	0.0000	1	1	13096	Unique	Used	1.658	1.049E3	2.188E3	59	4.18	0.00	0	17	300	2	841.52246	1682.03765	2.56	73.04	8215	8215	MS2	20/26	Glioma_Nuclear_prep_Gradeall_4plex.raw
503774453		High	lPSLGAGFSQGLk	Unambiguous	2	1	N-Term(iTRAQ4plex); K13(iTRAQ4plex)	HCD	1.0000	0.0000	1	1	11393	Unique	Used	1.647	2.497E2	5.569E2	167	3.86	0.00	0	53	300	3	521.64307	1562.91465	0.09	62.69	6003	6003	MS2	23/48	Glioma_Nuclear_prep_Gradeall_repeat_4plex.raw
538919685	NP_001269332.1	MDH2	4191	malate dehydrogenase, mitochondrial isoform 2 precursor [Homo sapiens]	18.24	2	2	2	0.528	1;1	18.4	10.00	296	30.9	8.43																			
538919685		High	gYLGPEQLPDcLk	Unambiguous	2	1	N-Term(iTRAQ4plex); C11(Methylthio); K13(iTRAQ4plex)	HCD	1.0000	0.0000	1	1	11978	Unique	Used	0.460	2.134E3	1.328E3	243	3.81	0.00	0	18	300	2	883.95807	1766.90886	1.63	71.63	6749	6749	MS2	17/24	Glioma_Nuclear_prep_Gradeall_repeat_4plex.raw
538919685	High		iTLYDIAHTPGVAADLSHIETk	Unambiguous	2	1	N-Term(iTRAQ4plex); K22(iTRAQ4plex)	HCD	1.0000	0.0000	1	1	11454	Unique	Used	0.597	2.443E3	1.835E3	73	4.34	0.00	0	20	21	4	664.11511	2653.43862	-2.03	52.23	6218	6218	MS2	30/126	Glioma_Nuclear_prep_Gradeall_4plex.raw
609876591	NP_001278340.1	SON	6651	protein SON isoform E [Homo sapiens]	3.65	4	4	5	1.559	3;2	15.7	12.68	2108	228.1	5.40																			
609876591	High		iEEVLSGVLDTELR	Unambiguous	4	1	N-Term(iTRAQ4plex)	HCD	1.0000	0.0000	1	1	12813	Unique	Used	1.375	4.987E3	8.621E3	216	5.19	0.00	0	24	300	2	858.97919	1716.95110	0.60	66.53	7729	7729	MS2	21/26	Glioma_Nuclear_prep_Gradeall_4plex.raw
609876591		High	eSDQTLAALLSPk	Unambiguous	3	1	N-Term(iTRAQ4plex); K13(iTRAQ4plex)	HCD	1.0000	0.0000	1	1	11404	Unique	Used	1.936	1.202E3	3.152E3	247	4.26	0.00	0	37	300	2	830.97327	1660.93926	1.94	62.85	6017	6017	MS2	18/24	Glioma_Nuclear_prep_Gradeall_repeat_4plex.raw
609876591	High		lPEQPVDVPSEIADSSMTRPQELPELPk	Unambiguous	3	1	N-Term(iTRAQ4plex); K28(iTRAQ4plex)	HCD	1.0000	0.0000	1	1	11419	Unique	Used	1.586	2.523E3	5.033E3	73	4.44	0.00	0	10	63	3	1130.92993	3390.77524	1.35	51.91	6179	6179	MS2	19/108	Glioma_Nuclear_prep_Gradeall_4plex.raw
609876591	High		aQLLEIAk	Unambiguous	4	1	N-Term(iTRAQ4plex); K8(iTRAQ4plex)	HCD	1.0000	0.0000	1	1	9219	Unique	Used	1.313	1.308E5	2.160E5	176	2.96	0.00	0	33	8	2	587.36584	1173.72441	-17.17	35.15	3779	3779	MS2	14/14	Glioma_Nuclear_prep_Gradeall_4plex.raw
609876591		High	aQLLEIAk	Unambiguous	4	1	N-Term(iTRAQ4plex); K8(iTRAQ4plex)	HCD	1.0000	0.0000	1	1	9876	Unique	Used	1.559	1.243E4	2.624E4	166	3.55	0.00	0	44	300	2	587.37671	1173.74614	1.34	43.49	4315	4315	MS2	14/14	Glioma_Nuclear_prep_Gradeall_repeat_4plex.raw
631790805	NP_001278830.1	SYT1	6857	synaptotagmin-1 isoform 2 [Homo sapiens]	7.40	4	3	4	0.514	2;2	17.2	7.43	419	47.2	7.94																			
631790805		High	vFVGYNSTGAELR	Unambiguous	2	1	N-Term(iTRAQ4plex)	HCD	1.0000	0.0000	1	1	10100	Unique	Used	0.493	7.777E3	5.194E3	267	4.29	0.00	0	29	300	2	778.91479	1556.82231	2.14	46.30	4562	4562	MS2	17/24	Glioma_Nuclear_prep_Gradeall_repeat_4plex.raw
631790805	High		lGDIcFSLR	Unambiguous	4	1	N-Term(iTRAQ4plex); C5(Methylthio)	HCD	1.0000	0.0000	1	1	11994	Unique	Used	0.360	3.915E4	1.772E4	142	3.14	0.00	0	64	160	2	607.31238	1213.61748	-1.27	56.93	6806	6806	MS2	14/16	Glioma_Nuclear_prep_Gradeall_4plex.raw
631790805	High		lTVVILEAk	Unambiguous	2	1	N-Term(iTRAQ4plex); K9(iTRAQ4plex)	HCD	1.0000	0.0000	1	1	10355	Unique	Used	0.535	6.150E4	4.138E4	85	3.20	0.00	0	87	53	2	637.41681	1273.82634	-5.53	43.49	5016	5016	MS2	15/16	Glioma_Nuclear_prep_Gradeall_4plex.raw
631790805		High	lTVVILEAk	Unambiguous	2	1	N-Term(iTRAQ4plex); K9(iTRAQ4plex)	HCD	1.0000	0.0000	1	1	11245	Unique	Used	0.534	5.118E3	3.704E3	64	3.13	0.00	0	75	300	2	637.42401	1273.84075	5.78	60.36	5812	5812	MS2	15/16	Glioma_Nuclear_prep_Gradeall_repeat_4plex.raw
664806003	NP_001287744.1	CIRBP	1153	cold-inducible RNA-binding protein isoform 2 [Homo sapiens]	14.88	3	1	2	0.157	1;1	36.1	14.01	168	18.1	9.64																			
664806003	High		gFGFVTFENIDDAk	Unambiguous	3	1	N-Term(iTRAQ4plex); K14(iTRAQ4plex)	HCD	1.0000	0.0000	1	1	11512	Unique	Used	0.197	1.036E5	2.569E4	166	6.40	0.00	0	38	63	2	924.47278	1847.93828	-1.94	52.70	6281	6281	MS2	25/26	Glioma_Nuclear_prep_Gradeall_4plex.raw
664806003		High	gFGFVTFENIDDAk	Unambiguous	3	1	N-Term(iTRAQ4plex); K14(iTRAQ4plex)	HCD	1.0000	0.0000	1	1	12168	Unique	Used	0.117	2.554E3	4.051E2	184	5.21	0.00	0	35	300	2	924.47766	1847.94805	3.35	75.42	7045	7045	MS2	23/26	Glioma_Nuclear_prep_Gradeall_repeat_4plex.raw
667743700	NP_001288139.1	MYEF2	50804	myelin expression factor 2 isoform b [Homo sapiens]	15.10	2	7	13	0.303	5;8	37.3	31.17	576	61.9	8.75																			
667743700	High		aEVPGATGGDSPHLQPAEPPGEPR	Unambiguous	2	1	N-Term(iTRAQ4plex)	HCD	1.0000	0.0000	1	1	8613	Unique	Used	0.272	1.440E4	4.935E3	56	3.98	0.00	0	0	18	3	837.40521	2510.20108	-15.23	30.74	3119	3119	MS2	26/92	Glioma_Nuclear_prep_Gradeall_4plex.raw
667743700	High		mGPGIGAILER	Unambiguous	2	1	N-Term(iTRAQ4plex)	HCD	1.0000	0.0000	1	1	10201	Unique	Used	0.273	2.237E5	7.676E4	231	3.27	0.00	0	28	19	2	629.35065	1257.69402	-13.21	42.36	4849	4849	MS2	19/20	Glioma_Nuclear_prep_Gradeall_4plex.raw
667743700	High		gFLSGPMGSGMR	Unambiguous	2	1	N-Term(iTRAQ4plex)	HCD	1.0000	0.0000	1	1	9599	Unique	Used	0.295	3.068E5	1.137E5	118	3.99	0.00	0	31	14	2	670.82251	1340.63774	-14.53	37.96	4194	4194	MS2	15/22	Glioma_Nuclear_prep_Gradeall_4plex.raw
667743700		High	mGPGIGAILER	Unambiguous	2	1	N-Term(iTRAQ4plex)	HCD	1.0000	0.0000	1	1	11068	Unique	Used	0.176	1.640E4	3.920E3	262	3.24	0.00	0	28	300	2	629.36108	1257.71489	3.38	58.04	5609	5609	MS2	19/20	Glioma_Nuclear_prep_Gradeall_repeat_4plex.raw
667743700		High	aEVPGATGGDSPHLQPAEPPGEPR	Unambiguous	2	1	N-Term(iTRAQ4plex)	HCD	1.0000	0.0000	1	1	9006	Unique	Used	0.374	2.173E3	1.100E3	69	5.23	0.00	0	10	161	3	837.42126	2510.24924	3.95	33.79	3389	3389	MS2	26/92	Glioma_Nuclear_prep_Gradeall_repeat_4plex.raw
667743700		High	gcGVVEFk	Unambiguous	2	1	N-Term(iTRAQ4plex); C2(Methylthio); K8(iTRAQ4plex)	HCD	1.0000	0.0000	1	1	10083	Unique	Used	0.345	1.235E4	5.777E3	143	2.96	0.00	0	63	300	2	586.80756	1172.60784	2.72	46.10	4544	4544	MS2	14/14	Glioma_Nuclear_prep_Gradeall_repeat_4plex.raw
667743700		High	rEPHPAEAEk	Unambiguous	2	1	N-Term(iTRAQ4plex); K10(iTRAQ4plex)	HCD	1.0000	0.0000	1	1	6509	Unique	Used	0.578	3.502E3	2.743E3	262	4.07	0.00	1	55	162	4	363.70331	1451.79140	4.72	10.34	750	750	MS2	17/54	Glioma_Nuclear_prep_Gradeall_repeat_4plex.raw
667743700	High		vGEVTYVELFk	Unambiguous	2	1	N-Term(iTRAQ4plex); K11(iTRAQ4plex)	HCD	1.0000	0.0000	1	1	11084	Unique	Used	0.154	3.818E5	7.381E4	207	5.31	0.00	0	28	13	2	786.44916	1571.89104	-0.84	49.12	5813	5813	MS2	19/20	Glioma_Nuclear_prep_Gradeall_4plex.raw
667743700	High		vFISNIPYDMk	Unambiguous	2	1	N-Term(iTRAQ4plex); K11(iTRAQ4plex)	HCD	1.0000	0.0000	1	1	10698	Unique	Used	0.334	3.627E5	1.524E5	257	4.34	0.00	0	53	12	2	807.93475	1614.86223	-11.26	46.14	5393	5393	MS2	16/20	Glioma_Nuclear_prep_Gradeall_4plex.raw
667743700		High	vGEVTYVELFk	Unambiguous	2	1	N-Term(iTRAQ4plex); K11(iTRAQ4plex)	HCD	1.0000	0.0000	1	1	11894	Unique	Used	0.163	4.970E3	1.096E3	211	4.57	0.00	0	27	300	2	786.45331	1571.89934	4.44	70.12	6628	6628	MS2	18/20	Glioma_Nuclear_prep_Gradeall_repeat_4plex.raw
667743700		High	vFISNIPYDMk	Unambiguous	2	1	N-Term(iTRAQ4plex); K11(iTRAQ4plex)	HCD	1.0000	0.0000	1	1	11513	Unique	Used	0.303	9.226E3	3.781E3	271	4.49	0.00	0	50	300	2	807.94672	1614.88616	3.55	64.31	6143	6143	MS2	17/20	Glioma_Nuclear_prep_Gradeall_repeat_4plex.raw
667743700		High	vGEVTYVELFk	Unambiguous	2	1	N-Term(iTRAQ4plex); K11(iTRAQ4plex)	HCD	1.0000	0.0000	1	1	11899	Unique	Used	0.406	2.662E2	1.465E2	212	3.47	0.00	0	18	300	3	524.63702	1571.89652	2.65	70.21	6635	6635	MS2	18/40	Glioma_Nuclear_prep_Gradeall_repeat_4plex.raw
667743700		High	vFISNIPYDmk	Unambiguous	2	1	N-Term(iTRAQ4plex); M10(Oxidation); K11(iTRAQ4plex)	HCD	1.0000	0.0000	1	1	10726	Unique	Used	0.417	9.737E3	5.502E3	186	3.14	0.00	0	58	300	2	815.94543	1630.88359	5.06	53.75	5232	5232	MS2	13/20	Glioma_Nuclear_prep_Gradeall_repeat_4plex.raw
733606247	NP_001290046.1	TUBA1C	84790	tubulin alpha-1C chain isoform b [Homo sapiens]	36.47	11	4	7	0.556	2;5	33.2	79.20	414	46.0	5.12																			
733606247	High		aVFVDLEPTVIDEVR	Unambiguous	6	1	N-Term(iTRAQ4plex)	HCD	1.0000	0.0000	1	1	11909	Unique	Used	0.297	2.839E5	1.061E5	180	5.28	0.00	0	9	9	2	923.51398	1846.02068	6.90	56.14	6713	6713	MS2	21/28	Glioma_Nuclear_prep_Gradeall_4plex.raw
733606247	High		aVFVDLEPTVIDEVR	Unambiguous	6	1	N-Term(iTRAQ4plex)	HCD	1.0000	0.0000	1	1	11838	Unique	Used	0.429	5.495E4	2.967E4	192	5.56	0.00	0	19	93	2	923.50635	1846.00542	-1.36	55.52	6635	6635	MS2	20/28	Glioma_Nuclear_prep_Gradeall_4plex.raw
733606247		High	aVFVDLEPTVIDEVR	Unambiguous	6	1	N-Term(iTRAQ4plex)	HCD	1.0000	0.0000	1	1	12316	Unique	Used	0.404	1.592E3	8.705E2	204	4.67	0.00	0	15	300	2	923.50970	1846.01213	2.27	79.48	7349	7349	MS2	20/28	Glioma_Nuclear_prep_Gradeall_repeat_4plex.raw
733606247		High	aVFVDLEPTVIDEVR	Unambiguous	6	1	N-Term(iTRAQ4plex)	HCD	1.0000	0.0000	1	1	12319	Unique	Used	0.556	2.250E2	1.695E2	209	3.71	0.00	0	31	300	3	616.00818	1846.00998	1.11	79.55	7354	7354	MS2	18/56	Glioma_Nuclear_prep_Gradeall_repeat_4plex.raw
733606247		High	eIIDLVLDR	Unambiguous	6	1	N-Term(iTRAQ4plex)	HCD	1.0000	0.0000	1	1	12283	Unique	Used	0.658	1.542E3	1.375E3	269	3.34	0.00	0	32	300	2	615.36426	1229.72124	-0.80	78.65	7286	7286	MS2	15/16	Glioma_Nuclear_prep_Gradeall_repeat_4plex.raw
733606247		High	dVNAAIATIk	Unambiguous	8	1	N-Term(iTRAQ4plex); K10(iTRAQ4plex)	HCD	1.0000	0.0000	1	1	9753	Unique	Used	0.766	1.202E4	1.247E4	223	4.23	0.00	0	67	300	2	652.39801	1303.78874	4.85	42.13	4185	4185	MS2	17/18	Glioma_Nuclear_prep_Gradeall_repeat_4plex.raw
733606247		High	tIGGGDDSFNTFFSETGAGk	Unambiguous	8	1	N-Term(iTRAQ4plex); K20(iTRAQ4plex)	HCD	1.0000	0.0000	1	1	11786	Unique	Used	0.759	1.423E3	1.463E3	88	6.39	0.00	0	3	300	2	1148.55457	2296.10185	2.00	68.31	6480	6480	MS2	28/38	Glioma_Nuclear_prep_Gradeall_repeat_4plex.raw
747165394	NP_001290556.1	MLEC	9761	malectin isoform 2 [Homo sapiens]	6.22	3	1	2	1.722	1;1	2.9	3.60	209	23.9	5.14																			
747165394	High		sNPEDQILYQTER	Unambiguous	3	1	N-Term(iTRAQ4plex)	HCD	1.0000	0.0000	1	1	9291	Unique	Used	1.686	8.023E4	1.702E5	133	3.60	0.00	0	62	17	2	868.91913	1736.83098	-15.10	35.69	3858	3858	MS2	19/24	Glioma_Nuclear_prep_Gradeall_4plex.raw
747165394		High	sNPEDQILYQTER	Unambiguous	3	1	N-Term(iTRAQ4plex)	HCD	1.0000	0.0000	1	1	9744	Unique	Used	1.757	6.279E3	1.494E4	178	3.36	0.00	0	49	300	2	868.93561	1736.86394	3.88	42.03	4176	4176	MS2	18/24	Glioma_Nuclear_prep_Gradeall_repeat_4plex.raw

Supplementary Table S6: Ingenuity Pathway Analysis of the non-redundant list of differentially expressed nuclear proteins (n=244) identified in different grades of astrocytomas. The proteins associated with major canonical pathways (A), molecular and cellular functions (B) and networks and processes (C), are listed with their p-value. The Table shows only top 5 canonical pathways, molecular and cellular functions, and networks. In (C), proteins present in the dataset mapping to respective networks are shown in bold.

A. Canonical pathways

Pathway	Molecules	-log (P-value)	Ratio
Granzyme A Signaling	**HIST1H1A, HIST1H1B, HIST1H1C, HIST1H1E, H1FX, HIST1H1D, HMGB2, H1F0**	1.48E-11	4.71E-01
Granzyme B Signaling	**PRKDC, NUMA1, LMNB2, LMNB1, PARP1**	1.32E-06	3.12E-01
EIF2 Signaling	**RPS19, RPL22, RPLP2, RPS25, RPS3, PPP1CA, RPL7A, RPL18, RPL31, RPS14**	8.07E-05	5.81E-02
DNA Double-Strand Break Repair by Non-Homologous End Joining	**PRKDC, XRCC5, PARP1**	6.92E-04	2.14E-01
Telomere Extension by Telomerase	**HNRNPA1, HNRNPA2B1, XRCC5**	8.56E-04	2E-01

B. Molecular and cellular functions

Category	p-value	Molecules
RNA Post-Transcriptional Modification	6.57E-35-1.26E-03	**HNRNPL, DDX21, SRSF9, PRPF8, HNRNPA2B1, SFPQ, ELAVL4, DDX17, HNRNPK, ELAVL1, TRA2A, SRSF1, DDX5, THRAP3, U2AF2, RBMX, NOLC1, HNRNPC, CDC5L, FBL, HNRNPA0, RPS19, SNRPA, SON, HNRNPU, KHDRBS1, RBM5, PPIG, DKC1, SYNCRIP, HNRNPD, SNW1, ADAR, HBB, HNRNPM, PTBP1, ALB, ZNF326, FUS, SNRPC, HNRNPA1, CIRBP, SRSF7, PABPN1, RBM8A, SNRNP70, SRSF5, HNRNPH3**
Cell Death and Survival	4.42E-12-1.29E-02	**CRYAB, THOC2, MBP, TRAP1, CTSG, SFPQ, RSL1D1, HBA1/HBA2, HSPA5, CBX5, LMNB1, ELAVL1, HMGB1, SRSF1, TOP1, LTF, SMPD4, SUB1, VAPB, PPP1CA, PGRMC1, MGST1, PELP1, SON, ATP5A1, LMNA, SNW1, ANXA2, HMGA1, SSRP1, MPO, CD9, SMARCA2, HNRNPA1, H2AFX, TMX1, HNRNPUL2, RTN4, GAPDH, GPM6A, VDAC1, PML,**

		**MTDH, XRCC5, RBBP4, HIST1H1C, APOE, DEK, NFIX, PRDX5, PRPF8, SNRPE, PPIA, ILF2, TMEM214, DDX17, CD74, HNRNPK, TOP2B, ANXA1, ANXA5, VCP, HNRNPC, NCL, PCBP2, RPS3, BCAP31, DHX9, PRKDC, RPS19, HNRNPU, KHDRBS1, RBM5, TRIM28, GLUD1, HSPD1, MSI2, PSIP1, HBB, PARP1, PLG, ENO1, ALB, APOA1, NFIB, TP53BP1, CYB5A**
Cellular Growth and Proliferation	9.35E-12-1.29E-02	**HP1BP3, ILF3, PLP1, MBP, CTSG, TRAP1, SFPQ, RSL1D1, HSPA5, LMNB1, ELAVL1, HMGB1, SRSF1, TOP1, DDX5, THRAP3, LTF, PPP1CA, HNRNPA0, SON, PELP1, NUMA1, ATP5A1, DKC1, LMNA, ANXA2, SNW1, HMGA1, ADAR, PTBP1, HNRNPAB, ATP5B, CD9, FUS, SMARCA2, HNRNPA1, RTN4, HIST1H1D, GAPDH, RPS25, VDAC1, PML, ACTG1, XRCC5, RBBP4, PBRM1, DDX21, APOE, PRPF8, TPR, HNRNPA2B1, ILF2, PPIA, HMGB2, CD74, DDX17, H2AFZ, HNRNPK, TOP2B, TRA2A, HNRNPR, ANXA1, HNRNPC, BCAP31, NCL, PURA, RPS19, HNRNPU, KHDRBS1, RBM5, TRIM28, HNRNPD, HSPD1, HNRNPM, PARP1, HIST1H1B, PLG, ENO1, ANXA6, APOA1, H2AFY, CIRBP, NOP2, PSAP, TP53BP1, NFIB, UBC, RPS14**
Gene Expression	5.23E-10-1.29E-02	**HP1BP3, ILF3, SFPQ, CBX5, ELAVL1, HMGB1, DDX5, TOP1, LTF, THRAP3, SUB1, NOLC1, ALYREF, PELP1, GNG2, SMARCA5, SYNCRIP, LMNA, SNW1, HMGA1, ADAR, SSRP1, MPO, TFAM, BASP1, CD9, SMARCA2, HNRNPA1, BUD31, PABPN1, GAPDH, PML, MTDH, XRCC5, DEK, DDX21, APOE, NFIX, PPIB, PRPF8, TPR, HNRNPA2B1, ILF2, HMGB2, DDX17, RPL7A, H2AFZ, HNRNPK, RBMX, RPS3, PURA, HNRNPC, DHX9, PRKDC, RBM5, MECP2, TRIM28, PSIP1, HBB, PARP1, PLG, HIST1H1B, ENO1, ALB, SNRPC, CIRBP, H2AFY, TP53BP1, NFIB, RPS14**
Cellular Development	1.08E-08-1.29E-02	**ILF3, MBP, CTSG, SFPQ, RSL1D1, HSPA5, LMNB1, ELAVL1, HMGB1, TOP1, DDX5, LTF, HIST1H4B, ALYREF, FBL, SON, PELP1, NUMA1, SMARCA5, SYNCRIP, ANXA2, SNW1, HMGA1, PTBP1, ATP5B, FUS, CD9, SMARCA2, HNRNPA1, HIST1H1D, GAPDH, FAM213A, GPM6A, RPS25, PML, XRCC5, DEK, DDX21, APOE, PRPF8, HNRNPA2B1, HMGB2, DDX17, CD74, H2AFZ, HNRNPK, TRA2A, RBMX, ANXA1, HSPE1, HNRNPC, NCL, PURA, DHX9, RPS19, HNRNPU, RBM5, TRIM28, HSPD1, PARP1, PLG, ENO1, ALB, ANXA6, APOA1, H2AFY, CIRBP, NFIB, RPS14**

C. Networks and processes

Top Functions	Molecules in Network	Score	Focus Molecules
Gene Expression, Cell Death and Survival, Embryonic	**ANXA1, CD74, CDC5L, ELAVL1,** GSK3, **H2AFX, HIST1H1C, HMGA1, HNRNPD,** IFN Beta, **ILF2, ILF3, LTF, MECP2,** MEK, **MPO, MSI2, MTDH, NCL, NFIX,** NFkB (complex), PI3K (family), **PRKDC,** Rnr, RPA, **RPS14, SNW1, SSRP1, TFAM, THRAP3, TOP1,**	43	28

Development	**TOP2B, TP53BP1, U2AF2, XRCC5**		
RNA Post-Transcriptional Modification, Infectious Diseases, Cellular Development	**ANXA2, ANXA5, ANXA6,** Cyclin A, Cyclin E, **DDX21, DHX9,** ERK1/2, **FBL, HBB, HNRNPAB, HNRNPC, HNRNPL, HNRNPR, HNRNPU, HSPA5,** JINK1/2, **KHDRBS1, KHDRBS2, KHDRBS3,** MAP2K1/2,p85 (pik3r), **PELP1, PLG, PPIA, PPIB,** Ras, **RSL1D1, SFPQ, SNRNP70, SRSF1, SRSF5, SYNCRIP, TUBA1C, VDAC1**	43	28
RNA Post-Transcriptional Modification, Cell Death and Survival, Neurological Disease	Actin, **ALYREF, BAZ1B, DDX5, DDX17, ENO1, FUS, GAPDH,** Histone h3, Histone h4, **HMGB1, HMGB2, HNRNPA1, HNRNPDL, HNRNPH3, HNRNPK,** Hsp90, **HSPD1, MATR3, MYEF2, MYL12A, NUMA1,** P38 MAPK, **PCBP1, RBBP4, RBMX,** RNA polymerase II, **RPS3, SMARCA5, SNRPD3, SRSF9, THOC2, TRAP1,** VEGF, **ZNF326**	43	28
RNA Post-Transcriptional Modification, Molecular Transport, RNA Trafficking	APP, ATXN2, CASC3, CSNK2A1, DDX6, Dynamin, Eif4g, G3BP1, GRB2, **HNRNPA2B1,** MAGOH, **MBP,** NME1, NOP58, NTRK3, PABPC1, PABPC4, **PABPN1,** PAPOLG, **PPIA, PPIG, RBM8A, RPL18,** RPL36, **RPL7A, RPS19, RTN4, SNRPA,** STRAP, **STXBP1, SUB1, SYNCRIP, SYT1,** TPI1, UPF3B	17	15
Dermatological Diseases and Conditions, Developmental Disorder, Hereditary Disorder	ADD2, AGTR1, ARID2, CARD16, CARD17, CASP1, CHD4, **CNTN1, DKC1,** FBP1, **GAR1, GPM6A,** GSTT2/GSTT2B, Histone h3, HMBOX1, **HMGN1,HSPA6,** MAFF, **NCEH1, NFIB, NHP2, NOP10, PBRM1,** PSG5, **PTBP2,** RGS10, **RPL22,** SBDS, SMARCA4, **SRRM2,** TERC, **UBC,** UBE2H, USP24, WDR1	17	15

Supplementary Table S7: Top 5 significant Networks and processes identified in nuclear protein analysis of different grades of astrocytoma when analyzed separately using Ingenuity Pathway Analysis.

Astrocytoma Grade	Networks and processes
**Diffuse astrocytoma (Grade II)**	1. RNA Post-Transcriptional Modification, DNA Replication, Recombination, and Repair, Gene Expression<br>2. Cell Death and Survival, Cellular Growth and Proliferation, DNA Replication, Recombination, and Repair<br>3. Gene Expression, Cell Death and Survival, Embryonic

	Development<br>4. RNA Post-Transcriptional Modification, Cellular Movement, Hematological Disease<br>5. Lipid Metabolism, Molecular Transport, Small Molecule Biochemistry
**Anaplastic astrocytoma (Grade III)**	1. Cell Death and Survival, Cell Morphology, Cellular Assembly and Organization<br>2. Dermatological Diseases and Conditions, Developmental Disorder, Hereditary Disorder<br>3. DNA Replication, Recombination, and Repair, Cell Death and Survival, Nervous System Development and Function<br>4. Cell Cycle, Cellular Development, Connective Tissue Development and Function<br>5. Embryonic Development, Organ Development, Organ Morphology
**Glioblastoma multiforme (Grade IV)**	1. RNA Post-Transcriptional Modification, Cell Death and Survival, Cell-To-Cell Signaling and Interaction<br>2. RNA Post-Transcriptional Modification, Cell Cycle, DNA Replication, Recombination, and Repair<br>3. Organismal Survival, Nervous System Development and Function, Cellular Development<br>4. RNA Post-Transcriptional Modification, DNA Replication, Recombination, and Repair, Gene Expression<br>5. Cell Cycle, Cell Morphology, Cellular Function and Maintenance

Supplementary Table S8: Proteins identified to be differentially expressed in all the grades studied - Grade II, III and IV. A total of 53 proteins identified were found common to all the grades. This protein subset has been derived from **Supplementary Table S2.**

Accession	NP accession	Gene Symbol	Gene id	Protein name	Grade II protein fold change [115/114]	Grade III protein fold change [116/114]	Grade IV protein fold change [117/114]	Molecular class
14043072	NP_112533.1	HNRNPA2B1	3181	heterogeneous nuclear ribonucleoproteins A2/B1 isoform B1	0.226	0.649	0.173	RNA binding protein,Ribonucleoprotein
109240550	NP_001035879.1	PSPC1	55269	paraspeckle component 1	0.261	0.315	0.423	RNA binding protein
34740329	NP_919223.1	HNRNPA3	220988	heterogeneous nuclear ribonucleoprotein A3	0.278	0.613	0.148	Ribonucleoprotein
5730027	NP_006550.1	KHDRBS1	10657	KH domain-containing, RNA-binding, signal transduction-associated protein 1 isoform 1	0.282	0.468	0.277	RNA binding protein
189217895	NP_689901.2	KHDRBS2	202559	KH domain-containing, RNA-binding, signal transduction-associated protein 2	0.282	0.294	0.185	RNA binding protein
5730073	NP_006549.1	KHDRBS3	10656	KH domain-containing, RNA-binding, signal transduction-associated protein 3	0.316	0.657	0.200	RNA binding protein
13676857	NP_068814.2	HSPA2	3306	heat shock-related 70 kDa protein 2	0.322	0.341	0.449	Heat shock protein
5032007	NP_005850.1	PURA	5813	transcriptional activator protein Pur-alpha	0.331	0.594	0.147	Transcription factor
667743700	NP_001288139.1	MYEF2	50804	myelin expression factor 2 isoform b	0.345	0.589	0.303	Transcription regulatory protein
160707950	NP_001104262.1	MECP2	4204	methyl-CpG-binding protein 2 isoform 2	0.359	0.608	0.456	DNA binding protein
68509932	NP_001020263.1	MBP	4155	myelin basic protein isoform 4	0.373	0.404	0.400	Structural protein
664806003	NP_001287744.1	CIRBP	1153	cold-inducible RNA-binding protein isoform 2	0.381	0.434	0.157	RNA binding protein
56118234	NP_859067.2	CENPV	201161	centromere protein V	0.397	0.597	0.582	DNA binding protein
62750354	NP_954659.1	MATR3	9782	matrin-3 isoform a	0.400	0.644	0.375	RNA binding protein
34101286	NP_057191.2	ZFR	51663	zinc finger RNA-binding protein	0.401	0.671	0.592	Transcription regulatory protein
38201710	NP_006377.2	DDX17	10521	probable ATP-dependent RNA helicase DDX17 isoform 1	0.417	0.567	0.535	ATPase,RNA helicase
221316767	NP_001138246.1	ELAVL4	1996	ELAV-like protein 4 isoform 2	0.427	0.450	0.418	RNA binding protein
70167113	NP_001020278.1	ADAR	103	double-stranded RNA-specific adenosine deaminase isoform d	0.448	0.560	0.509	Enzyme: Deaminase
4758138	NP_004387.1	DDX5	1655	probable ATP-dependent RNA helicase DDX5	0.450	0.597	0.552	RNA binding protein
25952118	NP_741960.1	CAMK2A	815	calcium/calmodulin-dependent protein kinase type II subunit alpha isoform 2	0.459	0.357	0.294	Serine/threonine kinase
8923900	NP_061156.1	CMAS	55907	N-acylneuraminate cytidylyltransferase	0.481	0.647	0.535	Enzyme: Ligase
109689718	NP_001035937.1	ABHD12	26090	monoacylglycerol lipase ABHD12 isoform a	0.486	0.622	0.654	Integral membrane protein
4758876	NP_004634.1	PABPN1	8106	polyadenylate-binding protein 2	0.488	0.617	0.567	RNA binding protein
68051721	NP_065843.3	NCEH1	57552	neutral cholesterol ester hydrolase 1 isoform b	0.500	0.528	0.590	Unclassified
66346730	NP_001015001.1	CKMT1A	548596	creatine kinase U-type, mitochondrial precursor	0.503	0.444	0.458	Enzyme: Phosphotransferase
157412270	NP_112480.2	HNRNPM	4670	heterogeneous nuclear ribonucleoprotein M isoform b	0.504	0.548	0.674	Ribonucleoprotein
19923653	NP_150091.2	PSIP1	11168	PC4 and SFRS1-interacting protein isoform 2	0.524	0.559	0.594	Transcription regulatory protein
49355765	NP_115657.2	ELAVL3	1995	ELAV-like protein 3 isoform 2	0.581	0.630	0.127	RNA binding protein
4506671	NP_000995.1	RPLP2	6181	60S acidic ribosomal protein P2	0.633	0.673	0.575	Ribosomal subunit
223555917	NP_848927.2	MTDH	92140	protein LYRIC	0.651	0.646	0.645	Unclassified
4759302	NP_004729.1	VAPB	9217	vesicle-associated membrane protein-associated protein B/C isoform 1	0.657	0.442	0.505	Membrane transport protein
5174449	NP_006017.1	H1FX	8971	histone H1x	1.653	2.438	1.889	DNA binding protein
5031877	NP_005564.1	LMNB1	4001	lamin-B1 isoform 1	1.846	1.943	3.019	Structural protein
21536286	NP_001814.2	CKB	1152	creatine kinase B-type	1.867	1.610	0.665	Enzyme: Phosphotransferase
4503183	NP_001905.1	CYB5A	1528	cytochrome b5 isoform 2	1.899	1.887	1.871	Enzyme: Oxidoreductase
194688133	NP_001124160.1	HMGB2	3148	high mobility group protein B2	1.959	2.729	3.890	Transcription regulatory protein
4504255	NP_002097.1	H2AFZ	3015	histone H2A.Z	1.998	1.561	2.701	DNA binding protein
4885377	NP_005311.1	HIST1H1D	3007	histone H1.3	2.183	4.208	7.321	DNA binding protein
217330646	NP_006704.3	SUB1	10923	activated RNA polymerase II transcriptional coactivator p15	2.200	1.505	1.642	Transcription factor
302129652	NP_001180473.1	ANXA6	309	annexin A6 isoform 2	2.613	1.828	2.405	Calcium binding protein
10800138	NP_066407.1	HIST1H2BD	3017	histone H2B type 1-D	2.654	1.553	2.499	DNA binding protein
402534522	NP_001257973.1	NFIX	4784	nuclear factor 1 X-type isoform 3	2.655	1.906	1.590	Transcription factor
4502107	NP_001145.1	ANXA5	308	annexin A5	2.695	2.263	2.963	Calcium binding protein
4885381	NP_005313.1	HIST1H1B	3009	histone H1.5	2.815	2.788	4.726	DNA binding protein
151101292	NP_110382.3	TMX1	81542	thioredoxin-related transmembrane protein 1 precursor	2.917	2.209	1.511	Enzyme: Oxidoreductase
4503979	NP_002046.1	GFAP	2670	glial fibrillary acidic protein isoform 1	3.054	2.076	1.940	Structural protein
4502027	NP_000468.1	ALB	213	serum albumin preproprotein	3.103	1.887	2.684	Transport/cargo protein
66529396	NP_001018171.1	PON2	5445	serum paraoxonase/arylesterase 2 isoform 2	3.150	4.699	2.579	Enzyme: Esterase
10800140	NP_066406.1	HIST1H2BB	3018	histone H2B type 1-B	3.463	1.755	2.666	DNA binding protein
4885375	NP_005310.1	HIST1H1C	3006	histone H1.2	3.478	4.044	3.483	DNA binding protein
4504425	NP_002119.1	HMGB1	3146	high mobility group protein B1	3.679	2.634	3.346	DNA binding protein
4504313	NP_003535.1	HIST1H4B	8366	histone H4	3.717	2.142	3.327	DNA binding protein
10800144	NP_066544.1	HIST1H2AJ	8331	histone cluster 1, H2aj	3.829	2.115	4.403	DNA binding protein

Supplementary Table S9: Immunohistochemistry on tissue microarray for six differentially expressed proteins, NUCKS1, SMARCA5, PARP1, PTBP1, HMGB2, and NFIB, identified by quantitative mass spectrometric analysis. Immunohistochemistry was performed using commercially available tissue microarrays (US BioMax) consisting of 4 control subjects, 13 diffuse astrocytomas (Grade II), 11 anaplastic astrocytomas (Grade III), and 27 glioblastoma (Grade IV) patients samples. Scoring criteria used were based on both staining intensities and distributions. Scores 0, +1, +2 and +3 indicate negative, low, medium and strong staining, respectively. The distribution of staining of cancer cells was scored as 0 (less than 5% of cells staining), 1+ (5–30% of cell staining), 2+ (31–60% of cells staining) and 3+ (greater than 60% of cells staining). TMA slides were evaluated independently by two neuropathologists.

Sl. No	Proteins	Mass spectrometry analysis	Samples (n) used for IHC	IHC scores (Staining intensity)			
				0	1+	2+	3+
1	NUCKS1	Grade II (Up)	Grade II (13)	0	0	0	13
		Grade III (Up)	Grade III (11)	0	0	0	11
		Grade IV (Up)	Grade IV (27)	0	1	0	26
		-	Control (4)	1	2	1	0
2	SMARCA5	Grade II No change	Grade II (13)	0	1	0	12
		Grade III No change	Grade III (11)	0	0	0	11
		Grade IV (Up)	Grade IV (27)	0	1	0	26
		-	Control (4)	2	1	1	0
3	PARP1	Grade II (Up)	Grade II (13)	0	1	0	12
		Grade III (Up)	Grade III (11)	0	2	0	9
		Grade IV No change	Grade IV (27)	1	6	1	19
		-	Control (4)	0	2	1	1
4	PTBP1	Grade II (Up)	Grade II (13)	1	3	0	9
		Grade III (Up)	Grade III (11)	0	0	0	11
		Grade IV No change	Grade IV (27)	2	6	5	14
		-	Control (4)	3	1	0	0
5	HMGB2	Grade II (Up)	Grade II (13)	0	1	1	11
		Grade III (Up)	Grade III (11)	0	2	3	6
		Grade IV (Up)	Grade IV (27)	0	9	4	14
		-	Control (4)	2	1	1	0
6	NFIB	Grade II (Up)	Grade II (13)	4	4	1	4
		Grade III (Up)	Grade III (11)	5	2	3	1
		Grade IV (Up)	Grade IV (27)	11	5	2	9
		-	Control (4)	4	0	0	0

Supplementary Table S10: List of 61 concordant mRNA and protein target entities present in the transcription interaction network and their microRNA regulators. The miRNA interaction were identified, as per pipeline shown in **Figure 5A** and described in the Methods, for mRNA and protein entities present in the transcription network as shown in **Figure 4**. Each of the miRNA-target interaction was supported by at least one PubMed ID.

Gene Symbol	Protein Fold change in our study	Transcript Fold change in GBM TCGA data	MicroRNA interactor	MIMATid	MicroRNA Fold change in GBM data from TCGA
ADAR	0.51	0.77	hsa-miR-106a-5p	MIMAT0000103	2.12
			hsa-miR-106b-5p	MIMAT0000680	2.58
			hsa-miR-130b-3p	MIMAT0000691	2.29
			hsa-miR-17-5p	MIMAT0000070	1.99
			hsa-miR-20a-5p	MIMAT0000075	2.23
			hsa-miR-20b-5p	MIMAT0001413	1.59
			hsa-miR-27a-3p	MIMAT0000084	3.40
			hsa-miR-93-5p	MIMAT0000093	2.25
BAZ1B	1.82	2.27	hsa-let-7e-5p	MIMAT0000066	0.59
BUD31	2.02	1.57	hsa-miR-218-5p	MIMAT0000275	0.12
BZW1	1.55	1.36	hsa-let-7d-5p	MIMAT0000065	0.72
			hsa-let-7e-5p	MIMAT0000066	0.59
			hsa-let-7g-5p	MIMAT0000414	0.58
			hsa-miR-101-3p	MIMAT0000099	0.45
			hsa-miR-136-5p	MIMAT0000448	0.21
			hsa-miR-144-3p	MIMAT0000436	0.78
			hsa-miR-323a-3p	MIMAT0000755	0.42
			hsa-miR-376c-3p	MIMAT0000720	0.79
			hsa-miR-582-5p	MIMAT0003247	0.63
			hsa-miR-98-5p	MIMAT0000096	0.75
CBX5	1.51	1.78	hsa-let-7d-5p	MIMAT0000065	0.72
			hsa-let-7e-5p	MIMAT0000066	0.59
			hsa-let-7g-5p	MIMAT0000414	0.58
			hsa-miR-1-3p	MIMAT0000416	0.79
			hsa-miR-98-5p	MIMAT0000096	0.75
CENPV	0.58	0.56	hsa-let-7b-5p	MIMAT0000063	1.16
			hsa-miR-28-5p	MIMAT0000085	1.19
DDX17	0.53	0.69	hsa-let-7b-5p	MIMAT0000063	1.16
			hsa-miR-106b-5p	MIMAT0000680	2.58
			hsa-miR-142-3p	MIMAT0000434	1.54
			hsa-miR-155-5p	MIMAT0000646	2.52
			hsa-miR-92a-3p	MIMAT0000092	2.69
DDX27	1.73	1.35	hsa-miR-23b-3p	MIMAT0000418	0.62
			hsa-let-7g-5p	MIMAT0000414	0.58

EEF1A1	1.95	1.40	hsa-miR-125a-5p	MIMAT0000443	0.85
			hsa-miR-185-5p	MIMAT0000455	0.79
			hsa-miR-221-3p	MIMAT0000278	0.58
			hsa-miR-23b-3p	MIMAT0000418	0.62
			hsa-miR-30e-5p	MIMAT0000692	0.69
			hsa-miR-340-3p	MIMAT0000750	0.61
ELAVL2	0.44	0.37	hsa-miR-106a-5p	MIMAT0000103	2.12
			hsa-miR-106b-5p	MIMAT0000680	2.58
			hsa-miR-17-5p	MIMAT0000070	1.99
			hsa-miR-20a-5p	MIMAT0000075	2.23
			hsa-miR-20b-5p	MIMAT0001413	1.59
			hsa-miR-26b-5p	MIMAT0000083	1.35
			hsa-miR-373-5p	MIMAT0000725	1.46
			hsa-miR-500a-3p	MIMAT0002871	1.21
			hsa-miR-93-5p	MIMAT0000093	2.25
ELAVL3	0.13	0.28	hsa-miR-26b-5p	MIMAT0000083	1.35
			hsa-miR-296-5p	MIMAT0000690	1.99
ELAVL4	0.42	0.15	hsa-miR-21-5p	MIMAT0000076	15.50
			hsa-miR-373-5p	MIMAT0000725	1.46
			hsa-miR-526b-5p	MIMAT0002835	1.30
FBL	1.66	1.78	hsa-miR-324-5p	MIMAT0000761	0.58
FUS	1.49	1.60	hsa-miR-221-3p	MIMAT0000278	0.58
			hsa-miR-331-3p	MIMAT0000760	0.54
H1FX	1.89	1.55	hsa-miR-1-3p	MIMAT0000416	0.79
H2AFY	3.79	1.49	hsa-miR-148b-3p	MIMAT0000759	0.82
H2AFZ	2.70	1.84	hsa-miR-148b-3p	MIMAT0000759	0.82
HIST1H1B	4.73	13.30	hsa-miR-1-3p	MIMAT0000416	0.79
HIST1H1C	3.48	2.65	hsa-miR-27b-3p	MIMAT0000419	0.56
			hsa-miR-98-5p	MIMAT0000096	0.75
HIST1H1E	1.81	1.89	hsa-miR-331-3p	MIMAT0000760	0.54
HIST1H2BD	2.50	1.39	hsa-let-7d-5p	MIMAT0000065	0.72
			hsa-let-7e-5p	MIMAT0000066	0.59
			hsa-let-7g-5p	MIMAT0000414	0.58
			hsa-miR-490-3p	MIMAT0002806	0.72
			hsa-miR-98-5p	MIMAT0000096	0.75
HIST1H3F	7.32	4.79	hsa-miR-299-5p	MIMAT0002890	0.57
HMGB1	3.35	1.56	hsa-let-7e-5p	MIMAT0000066	0.59
			hsa-let-7g-5p	MIMAT0000414	0.58
			hsa-miR-129-5p	MIMAT0000242	0.14
			hsa-miR-148b-3p	MIMAT0000759	0.82
			hsa-miR-218-5p	MIMAT0000275	0.12
			hsa-miR-22-3p	MIMAT0000077	0.70
HMGB2	3.89	3.31	hsa-miR-139-5p	MIMAT0000250	0.08
			hsa-miR-218-5p	MIMAT0000275	0.12
			hsa-miR-23b-3p	MIMAT0000418	0.62
			hsa-miR-329-3p	MIMAT0001629	0.55
			hsa-miR-33a-5p	MIMAT0000091	0.34
			hsa-miR-381-3p	MIMAT0000736	0.50
			hsa-miR-582-5p	MIMAT0003247	0.63
HMGN1	1.94	1.48	hsa-miR-27b-3p	MIMAT0000419	0.56
			hsa-miR-495-3p	MIMAT0002817	0.73
			hsa-miR-101-3p	MIMAT0000099	0.45
			hsa-miR-144-3p	MIMAT0000436	0.78

HNRNPAB	2.03	2.03	hsa-miR-149-5p	MIMAT0000450	0.31
			hsa-miR-504-5p	MIMAT0002875	0.68
			hsa-miR-625-5p	MIMAT0003294	0.81
HNRNPC	1.46	2.98	hsa-let-7e-5p	MIMAT0000066	0.59
			hsa-miR-342-3p	MIMAT0000753	0.45
			hsa-miR-485-3p	MIMAT0002176	0.67
HNRNPD	1.49	1.59	hsa-miR-1-3p	MIMAT0000416	0.79
			hsa-miR-221-3p	MIMAT0000278	0.58
			hsa-miR-222-3p	MIMAT0000279	0.43
HNRNPM	1.49	1.72	hsa-miR-191-5p	MIMAT0000440	0.82
			hsa-miR-29a-3p	MIMAT0000086	0.57
			hsa-miR-331-3p	MIMAT0000760	0.54
HNRNPR	1.65	1.61	hsa-miR-203a-3p	MIMAT0000264	0.80
			hsa-miR-409-3p	MIMAT0001639	0.73
KHDRBS3	0.20	0.67	hsa-miR-192-5p	MIMAT0000222	1.15
KHSRP	2.15	1.36	hsa-miR-149-5p	MIMAT0000450	0.31
			hsa-miR-185-5p	MIMAT0000455	0.79
			hsa-miR-221-3p	MIMAT0000278	0.58
			hsa-miR-27b-3p	MIMAT0000419	0.56
			hsa-miR-410-3p	MIMAT0002171	0.22
LMNB1	3.02	3.58	hsa-miR-1-3p	MIMAT0000416	0.79
			hsa-miR-101-3p	MIMAT0000099	0.45
			hsa-miR-124-3p	MIMAT0000422	0.03
			hsa-miR-128-3p	MIMAT0000424	0.22
			hsa-miR-154-5p	MIMAT0000452	0.69
			hsa-miR-218-5p	MIMAT0000275	0.12
			hsa-miR-23b-3p	MIMAT0000418	0.62
MATR3	0.38	0.73	hsa-miR-125b-5p	MIMAT0000423	1.34
			hsa-miR-155-5p	MIMAT0000646	2.52
			hsa-miR-18a-5p	MIMAT0000072	1.28
			hsa-miR-24-3p	MIMAT0000080	1.50
			hsa-miR-26b-5p	MIMAT0000083	1.35
			hsa-miR-615-3p	MIMAT0003283	1.26
MECP2	0.46	0.77	hsa-miR-106a-5p	MIMAT0000103	2.12
			hsa-miR-106b-5p	MIMAT0000680	2.58
			hsa-miR-155-5p	MIMAT0000646	2.52
			hsa-miR-17-5p	MIMAT0000070	1.99
			hsa-miR-195-5p	MIMAT0000461	1.50
			hsa-miR-199a-3p	MIMAT0000232	1.27
			hsa-miR-19a-3p	MIMAT0000073	1.61
			hsa-miR-19b-3p	MIMAT0000074	2.02
			hsa-miR-20a-5p	MIMAT0000075	2.23
			hsa-miR-20b-5p	MIMAT0001413	1.59
			hsa-miR-527	MIMAT0002862	1.43
			hsa-miR-92a-3p	MIMAT0000092	2.69
			hsa-miR-93-5p	MIMAT0000093	2.25
NFIB	1.41	1.42	hsa-miR-124-3p	MIMAT0000422	0.03
			hsa-miR-301a-3p	MIMAT0000688	0.83
			hsa-miR-331-3p	MIMAT0000760	0.54

			hsa-miR-379-5p	MIMAT0000733	0.29
			hsa-miR-491-5p	MIMAT0002807	0.30
NFIX	1.59	1.56	hsa-miR-124-3p	MIMAT0000422	0.03
			hsa-miR-149-5p	MIMAT0000450	0.31
			hsa-miR-324-5p	MIMAT0000761	0.58
NHP2	1.51	1.30	hsa-miR-218-5p	MIMAT0000275	0.12
NOLC1	0.58	0.71	hsa-let-7b-5p	MIMAT0000063	1.16
			hsa-miR-155-5p	MIMAT0000646	2.52
			hsa-miR-195-5p	MIMAT0000461	1.50
			hsa-miR-27a-3p	MIMAT0000084	3.40
			hsa-miR-324-3p	MIMAT0000762	1.79
			hsa-miR-513a-5p	MIMAT0002877	1.65
			hsa-miR-615-3p	MIMAT0003283	1.26
NT5E	1.79	1.54	hsa-miR-1-3p	MIMAT0000416	0.79
			hsa-miR-124-3p	MIMAT0000422	0.03
PARP1	1.93	1.54	hsa-miR-128-3p	MIMAT0000424	0.22
			hsa-miR-149-5p	MIMAT0000450	0.31
			hsa-miR-301a-3p	MIMAT0000688	0.83
			hsa-miR-31-5p	MIMAT0000089	0.81
			hsa-miR-379-5p	MIMAT0000733	0.29
			hsa-miR-7-5p	MIMAT0000252	0.11
PBRM1	1.94	1.59	hsa-miR-181c-5p	MIMAT0000258	0.73
			hsa-miR-30e-5p	MIMAT0000692	0.69
PRKDC	1.95	1.34	hsa-miR-218-5p	MIMAT0000275	0.12
PSIP1	0.59	0.57	hsa-miR-155-5p	MIMAT0000646	2.52
			hsa-miR-193b-3p	MIMAT0002819	1.67
PURA	0.15	0.62	hsa-miR-106b-5p	MIMAT0000680	2.58
			hsa-miR-15a-5p	MIMAT0000068	1.54
			hsa-miR-15b-5p	MIMAT0000417	3.33
			hsa-miR-16-5p	MIMAT0000069	2.29
			hsa-miR-192-5p	MIMAT0000222	1.15
			hsa-miR-195-5p	MIMAT0000461	1.50
			hsa-miR-20a-5p	MIMAT0000075	2.23
			hsa-miR-21-5p	MIMAT0000076	15.50
			hsa-miR-34b-5p	MIMAT0000685	1.30
			hsa-miR-93-5p	MIMAT0000093	2.25
RBBP4	1.57	2.01	hsa-let-7e-5p	MIMAT0000066	0.59
			hsa-miR-128-3p	MIMAT0000424	0.22
			hsa-miR-129-5p	MIMAT0000242	0.14
			hsa-miR-324-5p	MIMAT0000761	0.58
			hsa-miR-342-3p	MIMAT0000753	0.45
RBM8A	2.24	1.81	hsa-let-7e-5p	MIMAT0000066	0.59
			hsa-miR-136-5p	MIMAT0000448	0.21
			hsa-miR-144-3p	MIMAT0000436	0.78
			hsa-miR-148b-3p	MIMAT0000759	0.82
RPS15	1.49	1.27	hsa-miR-124-3p	MIMAT0000422	0.03
RPS27	1.77	1.32	hsa-let-7e-5p	MIMAT0000066	0.59
			hsa-miR-143-3p	MIMAT0000435	0.58
			hsa-miR-331-3p	MIMAT0000760	0.54

SFPQ	1.52	1.39	hsa-miR-218-5p	MIMAT0000275	0.12
			hsa-miR-26a-5p	MIMAT0000082	0.76
			hsa-miR-29b-3p	MIMAT0000100	0.26
			hsa-miR-331-3p	MIMAT0000760	0.54
			hsa-miR-383-5p	MIMAT0000738	0.49
SMARCA5	1.95	2.54	hsa-miR-101-3p	MIMAT0000099	0.45
			hsa-miR-103a-3p	MIMAT0000101	0.64
			hsa-miR-107	MIMAT0000104	0.50
			hsa-miR-144-3p	MIMAT0000436	0.78
SNRPD2	2.01	1.37	hsa-miR-149-5p	MIMAT0000450	0.31
SNRPD3	1.75	1.58	hsa-miR-124-3p	MIMAT0000422	0.03
			hsa-miR-218-5p	MIMAT0000275	0.12
			hsa-miR-330-3p	MIMAT0000751	0.35
			hsa-miR-410-3p	MIMAT0002171	0.22
			hsa-miR-490-3p	MIMAT0002806	0.72
SNRPE	1.77	1.90	hsa-miR-137	MIMAT0000429	0.09
SON	1.56	1.34	hsa-miR-103a-3p	MIMAT0000101	0.64
			hsa-miR-203a-3p	MIMAT0000264	0.80
			hsa-miR-222-3p	MIMAT0000279	0.43
SRSF1	1.87	1.46	hsa-miR-103a-3p	MIMAT0000101	0.64
			hsa-miR-107	MIMAT0000104	0.50
			hsa-miR-128-3p	MIMAT0000424	0.22
			hsa-miR-149-5p	MIMAT0000450	0.31
			hsa-miR-27b-3p	MIMAT0000419	0.56
			hsa-miR-377-3p	MIMAT0000730	0.29
			hsa-miR-7-5p	MIMAT0000252	0.11
SRSF9	1.72	1.40	hsa-miR-1-3p	MIMAT0000416	0.79
			hsa-miR-218-5p	MIMAT0000275	0.12
SSRP1	2.39	1.62	hsa-miR-23b-3p	MIMAT0000418	0.62
SUB1	1.64	1.55	hsa-let-7e-5p	MIMAT0000066	0.59
			hsa-miR-101-3p	MIMAT0000099	0.45
XRCC5	1.68	2.40	hsa-miR-331-3p	MIMAT0000760	0.54
			hsa-miR-7-5p	MIMAT0000252	0.11
YBX1	6.20	2.02	hsa-miR-124-3p	MIMAT0000422	0.03
			hsa-miR-137	MIMAT0000429	0.09
			hsa-miR-149-5p	MIMAT0000450	0.31
			hsa-miR-29b-3p	MIMAT0000100	0.26
			hsa-miR-379-5p	MIMAT0000733	0.29

Supplementary Table S11: Immunohistochemistry on tissue microarrays of GBM for YBX1 and its interacting proteins, EGFR, MAPK1, CD44, TNC, MMP13 and SOX2 identified by protein-protein network analysis. Immunohistochemistry was performed using in-house prepared tissue microarrays consisting of 4 control subjects and 18 glioblastoma (Grade IV) patient samples. Scoring criteria used were based on both staining intensities and distributions. TMA slides were evaluated independently by two neuropathologists. The distribution of staining of cancer cells was scored as 0 (less than 5% of cells staining), 1+ (5–30% of cell staining), 2+ (31–60% of cells staining) and 3+ (greater than 60% of cells staining). Scores 0, +1, +2 and +3 indicate negative, low, medium and strong staining respectively.

		**Pathologist I**				**Pathologist II**		**FINAL SCORING**	
**Gene Symbol**	**Subjects**	**IHC Scoring**				**IHC Scoring**			
		**0**	**1+**	**2+**	**3+**	**Negative**	**Positive (2+ or 3+)**	**Negative**	**Positive**
YBX1	GBM	0	0	1	17	0	18	0	18
	Controls	4	0	0	0	4	0	4	0
EGFR	GBM	1	0	8	9	1	17	1	17
	Controls	4	0	0	0	4	0	4	0
MAPK1	GBM	1	0	9	8	1	17	1	17
	Controls	1	3	0	0	4	0	4	0
CD44	GBM	1	1	6	10	2	16	2	16
	Controls	4	0	0	0	4	0	4	0
TNC	GBM	6	0	6	6	6	12	6	12
	Controls	4	0	0	0	4	0	4	0
MMP13	GBM	2	0	11	5	2	16	2	16
	Controls	4	0	0	0	4	0	4	0
SOX2	GBM	0	0	1	17	0	18	0	18
	Controls	4	0	0	0	4	0	4	0

Supplementary Table S12: List of selected differentially expressed transcription regulatory proteins identified in the study and their role in tumorigenesis.

Protein	Expression status Up / Down (Gr II, III, IV)*	Molecular function	Molecular mechanism	Role in cancer	Ref.
HMGA1	Up in Gr III	DNA binding protein with chromatin remodelling activity	HMGA1 expression correlates with Ki-67, MMP-9, and VEGF-A; The downstream targets include COX2 and STAT3, involved in tumor progression	regulator of cell invasion and metastasis, potential therapeutic target	[1-5]
HMGB1	Up in Gr II, III, IV	DNA binding protein with chromatin remodelling activity	HMGB1 promotes autophagy with low p53 expression and leads to tumor cell survival	promotes autophagy and tumor cell survival, correlated to shorter overall survival and linked to chemoresistance in GBM cells	[6-10]
HMGB2	Up in Gr II, III, IV	DNA binding protein with chromatin remodelling activity	HMGB2 affects cell invasion by altering expression of p53and MMP2/ TIMP2	involved in cell invasion activity, prognostic factor and therapeutic target for GBM	[7]
NUCKS1	Up in Gr II, III; **novel to gliomas**	DNA binding protein involved in DNA damage repair	NUCKS1 repairs DNA double-strand breaks by homologous recombination	having a role in tumor progression, prognostic factor	[11, 12]
SMARCA5	Up in Gr IV	Helicase and ATPase activity and regulates transcription by	Knockdown of SMARCA5-arrested cell cycle. Analysis of	overexpression of SMARCA5 correlates with increased cell	[13, 14]

		altering chromatin structure	cell cycle and invasion-related molecules showed that SMARCA5 downregulated cyclin A, MMP2 expression and upregulated p21 expression	proliferation, invasion and chemo resistance in glioma, potential therapeutic target	
TFAM	Up in Gr II, III	Transcription factor for mitochondrial DNA replication, transcription	TFAM is involved in the activity of PI3K/Akt signaling pathway for cell proliferation. In addition, cell migration and invasion activity-related MMP2 and MMP9, is increased with overexpression of TFAM	involved in increased malignancy of glioma,promising target for therapeutics	[15, 16]
NF1B	Up in Gr II, III, IV	Transcription factor, promotes differentiation of astrocytes	NF1B is involved in STAT3 signaling in classical and mesenchymal GBM cells	GBM sub-type specific tumor suppressor, associated with improved survival in GBM	[17]
NF1X	Up in Gr II, III, IV	Transcription factor, important for activation of GFAP transcription in astrocytes	NF1X regulates the expression of GFAP, an astrocyte marker protein	implicated in glioma	[18]
PURA	Down in Gr II, III, IV	Transcription factor, implicated in progression of cell cycle	PURA binds to hypophosphorylated form of the retinoblastoma protein, pRb, a regulator of S-phase initiation	ectopic overexpression of PURA suppress tumor cell proliferation in GBM cells,potential use of this protein and its derivative(s) in blocking proliferation of tumor cells	[19]

SON	Up in Gr IV; **novel to gliomas**	Transcription factor, involved in mRNA splicing and having a role inmitotic spindle pole separation, chromosome alignment and microtubule dynamics	SON is regulates RNA splicing for effective cell cycle progression and genome stability; Knockdown of SON leads to splicing defects in transcripts containing weak splice sites, and many genes necessary for cell-cycle progression and epigenetic modification	regulates genes involved in cell cycle progression and DNA repair, Potential therapeutic target	[20]
SUB1	Up in Gr II, III, IV	Transcription factor, ssDNA-binding protein involved in DNA replication, repair and heterochromatinization	SUB1 binds to promoter regions of several oncogenes such as PLK1 (Polo-like kinase 1), C-MYC, serine-threonine kinase BUB1B and regulates their expression	Elevated PC4 expression is strongly correlated with the progression of astrocytoma; Role in cell proliferation, invasion and migration; Potential prognostic marker and therapeutic target	[21-23]
SSRP1	Up in Gr II	Transcription factor, involved in chromatin remodeling	SSRP1 is a subunit of the FACT (Facilitates Chromatin Transcription) complex involved in chromatin remodeling	Involved in maintenance of pluripotent cell state; Role in cell proliferation, invasion and migration in GBM cells; Predictive marker for disease progression and therapeutic target	[24-26]
PTBP1	Up in Gr II, III	RNA-binding protein and a major repressive regulator of alternative splicing, causing exon skipping in numerous alternatively spliced pre-mRNAs	PTBP1 is involved in tumerigenesis through an aberrant alternate splicing of several genes involved in cell proliferation (FGFR1, FGFR2), invasion	Role in cell proliferation and migration in glioma; Potential therapeutic target	[27-29]

			(CSRC), motility (ACTN, FBN), apoptosis (FAS, CASP2), and multi-drug resistance (ABCC1)		

*if fold change for a grade is not shown in column 2, it implies differential expression was not observed

References

1. Shah SN and Resar LM. High mobility group A1 and cancer: potential biomarker and therapeutic target. Histology and histopathology. 2012; 27(5):567-579.
2. Pang B, Fan H, Zhang IY, Liu B, Feng B, Meng L, Zhang R, Sadeghi S, Guo H and Pang Q. HMGA1 expression in human gliomas and its correlation with tumor proliferation, invasion and angiogenesis. Journal of neuro-oncology. 2012; 106(3):543-549.
3. Donato G, Martinez Hoyos J, Amorosi A, Maltese L, Lavano A, Volpentesta G, Signorelli F, Pentimalli F, Pallante P, Ferraro G, Tucci L, Signorelli CD, Viglietto G and Fusco A. High mobility group A1 expression correlates with the histological grade of human glial tumors. Oncology reports. 2004; 11(6):1209-1213.
4. Huso TH and Resar LM. The high mobility group A1 molecular switch: turning on cancer - can we turn it off? Expert opinion on therapeutic targets. 2014; 18(5):541-553.
5. Hillion J, Smail SS, Di Cello F, Belton A, Shah SN, Huso T, Schuldenfrei A, Nelson DM, Cope L, Campbell N, Karikari C, Aderinto A, Maitra A, Huso DL and Resar LM. The HMGA1-COX-2 axis: a key molecular pathway and potential target in pancreatic adenocarcinoma. Pancreatology. 2012; 12(4):372-379.
6. Wang XJ, Zhou SL, Fu XD, Zhang YY, Liang B, Shou JX, Wang JY and Ma J. Clinical and prognostic significance of high-mobility group box-1 in human gliomas. Experimental and therapeutic medicine. 2015; 9(2):513-518.
7. Wu ZB, Cai L, Lin SJ, Xiong ZK, Lu JL, Mao Y, Yao Y and Zhou LF. High-mobility group box 2 is associated with prognosis of glioblastoma by promoting cell viability, invasion, and chemotherapeutic resistance. Neuro-oncology. 2013; 15(9):1264-1275.
8. Livesey KM, Kang R, Vernon P, Buchser W, Loughran P, Watkins SC, Zhang L, Manfredi JJ, Zeh HJ, 3rd, Li L, Lotze MT and Tang D. p53/HMGB1 complexes regulate autophagy and apoptosis. Cancer research. 2012; 72(8):1996-2005.
9. Tang D, Kang R, Zeh HJ, 3rd and Lotze MT. High-mobility group box 1 and cancer. Biochimica et biophysica acta. 2010; 1799(1-2):131-140.

10. Stros M, Ozaki T, Bacikova A, Kageyama H and Nakagawara A. HMGB1 and HMGB2 cell-specifically down-regulate the p53- and p73-dependent sequence-specific transactivation from the human Bax gene promoter. The Journal of biological chemistry. 2002; 277(9):7157-7164.
11. Schaner ME, Ross DT, Ciaravino G, Sorlie T, Troyanskaya O, Diehn M, Wang YC, Duran GE, Sikic TL, Caldeira S, Skomedal H, Tu IP, Hernandez-Boussard T, Johnson SW, O'Dwyer PJ, Fero MJ, et al. Gene expression patterns in ovarian carcinomas. Molecular biology of the cell. 2003; 14(11):4376-4386.
12. Ziolkowski P, Gamian E, Osiecka B, Zougman A and Wisniewski JR. Immunohistochemical and proteomic evaluation of nuclear ubiquitous casein and cyclin-dependent kinases substrate in invasive ductal carcinoma of the breast. Journal of biomedicine & biotechnology. 2009; 2009:919645.
13. Zhao XC, An P, Wu XY, Zhang LM, Long B, Tian Y, Chi XY and Tong DY. Overexpression of hSNF2H in glioma promotes cell proliferation, invasion, and chemoresistance through its interaction with Rsf-1. Tumour biology : the journal of the International Society for Oncodevelopmental Biology and Medicine. 2016; 37(6):7203-7212.
14. Jin Q, Mao X, Li B, Guan S, Yao F and Jin F. Overexpression of SMARCA5 correlates with cell proliferation and migration in breast cancer. Tumour biology : the journal of the International Society for Oncodevelopmental Biology and Medicine. 2015; 36(3):1895-1902.
15. Lee H, Park J, Tran Q, Kim D, Hong Y, Cho H, Kwon SH, Brazil D, Kim SH and Park J. Mitochondrial transcription factor A (TFAM) is upregulated in glioma. Molecular medicine reports. 2017; 15(6):3781-3786.
16. Jiang J, Yang J, Wang Z, Wu G and Liu F. TFAM is directly regulated by miR-23b in glioma. Oncology reports. 2013; 30(5):2105-2110.
17. Stringer BW, Bunt J, Day BW, Barry G, Jamieson PR, Ensbey KS, Bruce ZC, Goasdoue K, Vidal H, Charmsaz S, Smith FM, Cooper LT, Piper M, Boyd AW and Richards LJ. Nuclear factor one B (NFIB) encodes a subtype-specific tumour suppressor in glioblastoma. Oncotarget. 2016; 7(20):29306-29320.
18. Gopalan SM, Wilczynska KM, Konik BS, Bryan L and Kordula T. Nuclear factor-1-X regulates astrocyte-specific expression of the alpha1-antichymotrypsin and glial fibrillary acidic protein genes. The Journal of biological chemistry. 2006; 281(19):13126-13133.
19. Darbinian N, Gallia GL, King J, Del Valle L, Johnson EM and Khalili K. Growth inhibition of glioblastoma cells by human Pur(alpha). Journal of cellular physiology. 2001; 189(3):334-340.
20. Ahn EY, DeKelver RC, Lo MC, Nguyen TA, Matsuura S, Boyapati A, Pandit S, Fu XD and Zhang DE. SON controls cell-cycle progression by coordinated regulation of RNA splicing. Molecular cell. 2011; 42(2):185-198.

21. Chakravarthi BV, Goswami MT, Pathi SS, Robinson AD, Cieslik M, Chandrashekar DS, Agarwal S, Siddiqui J, Daignault S, Carskadon SL, Jing X, Chinnaiyan AM, Kunju LP, Palanisamy N and Varambally S. MicroRNA-101 regulated transcriptional modulator SUB1 plays a role in prostate cancer. Oncogene. 2016; 35(49):6330-6340.
22. Conesa C and Acker J. Sub1/PC4 a chromatin associated protein with multiple functions in transcription. RNA biology. 2010; 7(3):287-290.
23. Chen L, Du C, Wang L, Yang C, Zhang JR, Li N, Li Y, Xie XD and Gao GD. Human positive coactivator 4 (PC4) is involved in the progression and prognosis of astrocytoma. Journal of the neurological sciences. 2014; 346(1-2):293-298.
24. Dermawan JK, Hitomi M, Silver DJ, Wu Q, Sandlesh P, Sloan AE, Purmal AA, Gurova KV, Rich JN, Lathia JD, Stark GR and Venere M. Pharmacological Targeting of the Histone Chaperone Complex FACT Preferentially Eliminates Glioblastoma Stem Cells and Prolongs Survival in Preclinical Models. Cancer research. 2016; 76(8):2432-2442.
25. Liao J, Tao X, Ding Q, Liu J, Yang X, Yuan FE, Yang JA, Liu B, Xiang GA and Chen Q. SSRP1 silencing inhibits the proliferation and malignancy of human glioma cells via the MAPK signaling pathway. Oncology reports. 2017; 38(5):2667-2676.
26. Fleyshman D, Prendergast L, Safina A, Paszkiewicz G, Commane M, Morgan K, Attwood K and Gurova K. Level of FACT defines the transcriptional landscape and aggressive phenotype of breast cancer cells. Oncotarget. 2017; 8(13):20525-20542.
27. Cheung HC, Hai T, Zhu W, Baggerly KA, Tsavachidis S, Krahe R and Cote GJ. Splicing factors PTBP1 and PTBP2 promote proliferation and migration of glioma cell lines. Brain : a journal of neurology. 2009; 132(Pt 8):2277-2288.
28. Wang ZN, Liu D, Yin B, Ju WY, Qiu HZ, Xiao Y, Chen YJ, Peng XZ and Lu CM. High expression of PTBP1 promote invasion of colorectal cancer by alternative splicing of cortactin. Oncotarget. 2017; 8(22):36185-36202.
29. Takahashi H, Nishimura J, Kagawa Y, Kano Y, Takahashi Y, Wu X, Hiraki M, Hamabe A, Konno M, Haraguchi N, Takemasa I, Mizushima T, Ishii M, Mimori K, Ishii H, Doki Y, et al. Significance of Polypyrimidine Tract-Binding Protein 1 Expression in Colorectal Cancer. Molecular cancer therapeutics. 2015; 14(7):1705-1716.
